# The repertoire of G-protein-coupled receptors in *Xenopus tropicalis*

**DOI:** 10.1186/1471-2164-10-263

**Published:** 2009-06-09

**Authors:** Yanping Ji, Zhen Zhang, Yinghe Hu

**Affiliations:** 1 Shanghai Institute of Brain Functional Genomics, East China Normal University, 3663 Zhongshan Road N., Shanghai, 200062, PR China

## Abstract

**Background:**

The G-protein-coupled receptor (GPCR) superfamily represents the largest protein family in the human genome. These proteins have a variety of physiological functions that give them well recognized roles in clinical medicine. In *Xenopus tropicalis*, a widely used animal model for physiology research, the repertoire of GPCRs may help link the GPCR evolutionary history in vertebrates from teleost fish to mammals.

**Results:**

We have identified 1452 GPCRs in the *X. tropicalis *genome. Phylogenetic analyses classified these receptors into the following seven families: *Glutamate*, *Rhodopsin*, *Adhesion*, *Frizzled*, *Secretin*, *Taste 2 *and *Vomeronasal 1*. Nearly 70% of *X. tropicalis *GPCRs are represented by the following three types of receptors thought to receive chemosensory information from the outside world: olfactory, vomeronasal 1 and vomeronasal 2 receptors.

**Conclusion:**

*X. tropicalis *shares a more similar repertoire of GPCRs with mammals than it does with fish. An examination of the three major groups of receptors related to olfactory/pheromone detection shows that in *X. tropicalis*, these groups have undergone lineage specific expansion. A comparison of GPCRs in *X. tropicalis*, teleost fish and mammals reveals the GPCR evolutionary history in vertebrates.

## Background

The G-protein-coupled receptor (GPCR) superfamily represents the largest protein family in the human genome [1,2]. These proteins have ancient origins and their sequences are very diverse in a number of species. Nonetheless, all GPCRs share a common molecular architecture consisting of seven transmembrane (TM) domains. GPCRs transduce outside signals to intracellular effectors and play a central role in almost all physiological functions. The disruption of their normal activities in humans results in a wide variety of diseases and disorders, including retinitis pigmentosa (RP), hypo- and hyperthyroidism, nephrogenic diabetes insipidus, several fertility disorders and carcinomas [3]. GPCRs are the richest source of targets for the pharmaceutical industry [4].

The evolutionary data for GPCRs can be applied to further functional analyses and are particularly useful for structural evaluation of disease-causing mutations. In this respect, because of their common physiological traits with all vertebrates, amphibians can be ideal non-mammalian models for GPCR-related functional and medical research [5]. Although the overall repertoires of GPCRs have been documented in teleost fish [6] and mammals [7,8], no such report yet exists for amphibians. However, recent developments relating to genomic sequence data have allowed us to conduct extensive analyses of the amphibian GPCR family, which may help us to understand the connections in the GPCR evolution from teleost fish to mammals.

For this purpose, we chose the western clawed frog *Xenopus (Silurana) tropicalis *for our investigation. *X. tropicalis *is a preferred model for genetic studies because it has a smaller diploid genome than that of its relatives. Recently, its genome has been sequenced, and the sequences have been released in the form of scaffolds. In this study, we present a strategy for detecting and identifying GPCR genes from a genomic assembly. Using this strategy, we have provided the first overall map of the GPCRs in *X. tropicalis*. By comparing *X. tropicalis *receptor groups with those of mammals and/or fish, we performed detailed phylogenetic analyses. These comparative data provide information about the evolutionary history of GPCRs, and the available receptor sequences may help to identify conserved sequence motifs that are responsible for certain aspects of GPCR functionality, especially those which may cause disease through mutation.

## Results and Discussion

### The overall GPCR repertoire in *Xenopus tropicalis*

The genome of *X. tropicalis *is estimated to be approximately 1.7 billion base pairs contained in 10 pairs of chromosomes. The protein sequences used in our research were obtained from the Joint Genome Institute's *X. tropicalis *genome project [9]. The current database, JGI *Xenopus tropicalis v4.1*, contains 27,917 protein sequences. As the protein sequences represent approximately 95% of the full-length cDNAs of *X. tropicalis*, we expect that the GPCR genes identified here comprise the majority of the GPCR repertoire in *X. tropicalis*. The gene sequences of *X. tropicalis *have been released in the form of scaffolds, so highly polymorphic genes might be overestimated. In order to avoid overestimation, CD-HIT [10] 90% sequence identity was applied to remove polymorphisms, splice variants, pseudogenes and duplicates from the database.

**Figure 1:**
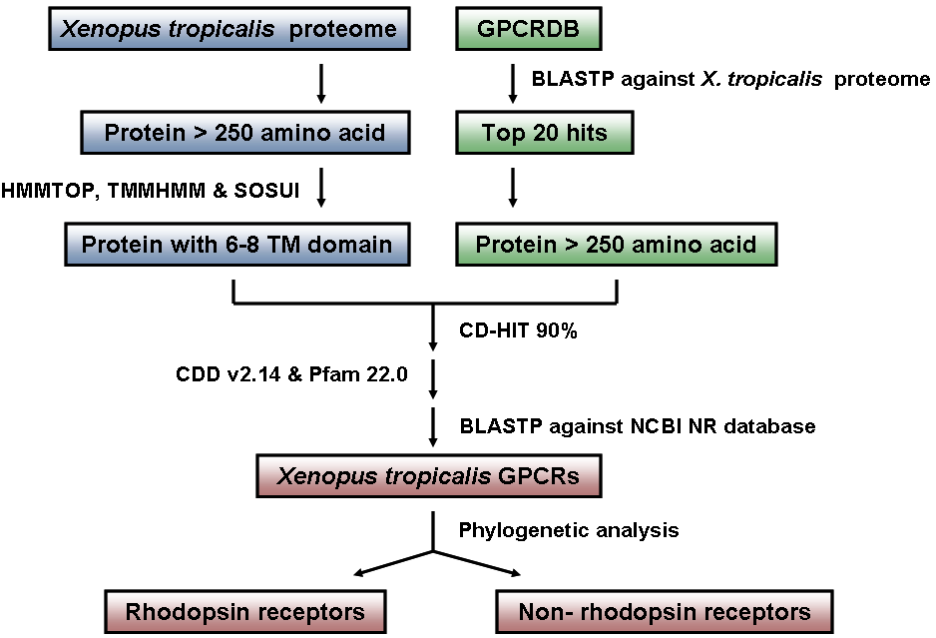
**Sequence analysis strategy for the identification of *X. tropicalis *GPCRs**. Two parallel methods were used for searching the crude sequence database. One was retrieval of proteins with six to eight TM domains from the *X. tropicalis *proteome database, and the other was extraction of the BLASTP top 20 hits by comparing the GPCR sequences from GPCRDB against the *X. tropicalis *proteome database. The crude database, which eliminated polymorphism, splice variants, pseudogenes and duplicates by CD-HIT 90% sequence identity, was searched for GPCR conserved domains using CDD v2.14 (E-value = 10^-4^) and Plam 22.0 (E-value = 0.01). The sequences with conserved seven TM domain were searched using BLASTP against the NCBI non-redundant database. Phylogenetic analyses were carried out to separate the GPCR sequences into rhodopsin like receptors and non-rhodopsin like receptors.

Using the strategy shown in Figure 1, 1,452 GPCRs were identified. GPCRs represent about 5.2% of the total number of genes predicted from the *X. tropicalis *genome. Homologues of the chemosensory GPCRs from nematodes, plant GPCRs, yeast pheromone receptors, or insect gustatory and olfactory receptors were not identified. The *X. tropicalis *receptors were named after their closest known GPCRs by BLASTP against the NCBI non-redundant database. The names in each family are listed in Additional file 1 and the sequences of each receptor are listed in Additional file 2.

**Figure 2:**
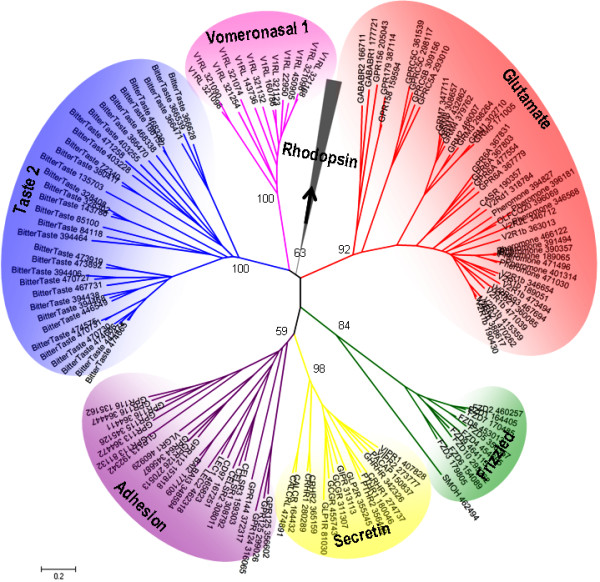
**Phylogenetic tree of the non-rhodopsin receptors**. The tree was calculated using the neighbour-joining method with 1000 bootstrap replicas. The 339 CaSR like receptors in *Glutamate *receptors were represented by 25 members in this group using CD-HIT 40% sequence identity. The position of the *Rhodopsin *family was established by including twelve random receptors from the *Rhodopsin *family.

**Figure 3:**
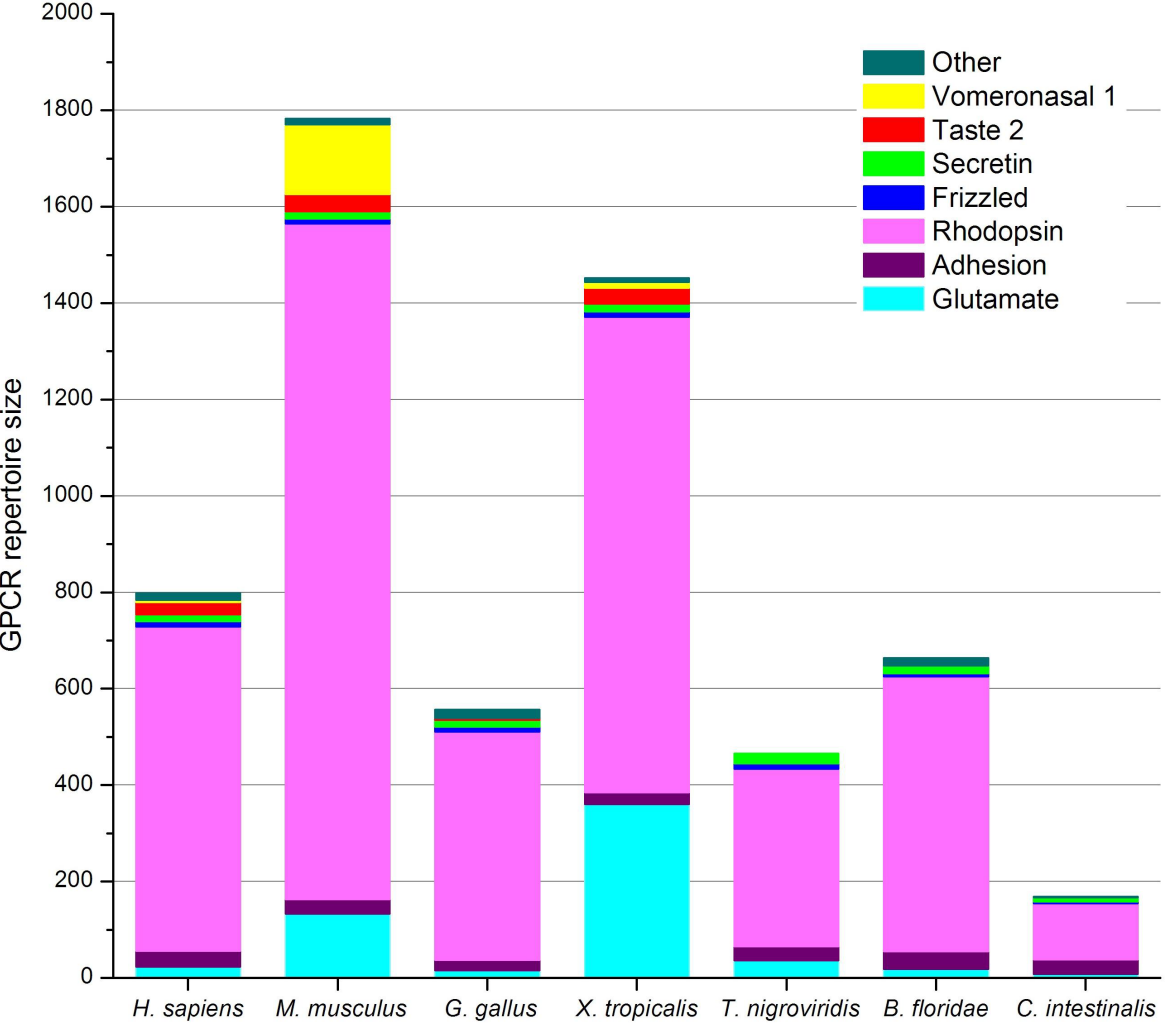
**Distribution of the number of different GPCR families in 7 different species**. The species displayed, from left to right, were *Homo sapiens *[7], *Mus musculus *[7], *Gallus gallus *[11], *Xenopus tropicalis*, *Tetraodon nigroviridis *[6], *Branchiostoma floridae *[12] and *Ciona intestinalis *[13].

Phylogenetic analysis was performed for strict family classification. With regard to the large number of genes for olfactory receptors and CaSR like receptors (consisting of extracellular calcium-sensing receptors and pheromone receptors) would hamper the preliminary phylogenetic analysis, the positions of the two groups were established by diverse receptors of the two groups using CD-HIT 40% sequence identity. The phylogenetic analysis first separated the GPCR sequences into rhodopsin like receptors and non-rhodopsin like receptors, then separated the rhodopsin like receptors into six families. In addition to the five main GPCR families (GRAFS: *Glutamate *(G), *Rhodopsin *(R), *Adhesion *(A), *Frizzled *(F) and *Secretin *(S) [8]), *Taste 2 *receptors (T2Rs) and *Vomeronasal 1 *receptors (V1Rs) were considered as two independent receptor families (Figure 2). Besides the above seven families, nine other *X. tropicalis *GPCRs that could not be classified into any of these families were referred to as "Other GPCRs". Figure 3 compares the distribution of a number of different GPCR families in *X. tropicalis *with those from six different species [6,7,11-13]. The main difference in the GPCR repertoire between *X. tropicalis *and other species genomes is the number of vomeronasal 2 receptors (V2Rs) in *Glutamate *receptor family.

### The *Adhesion *(24 members), *Frizzled *(11 members) and *Secretin *(16 members) families

**Figure 4:**
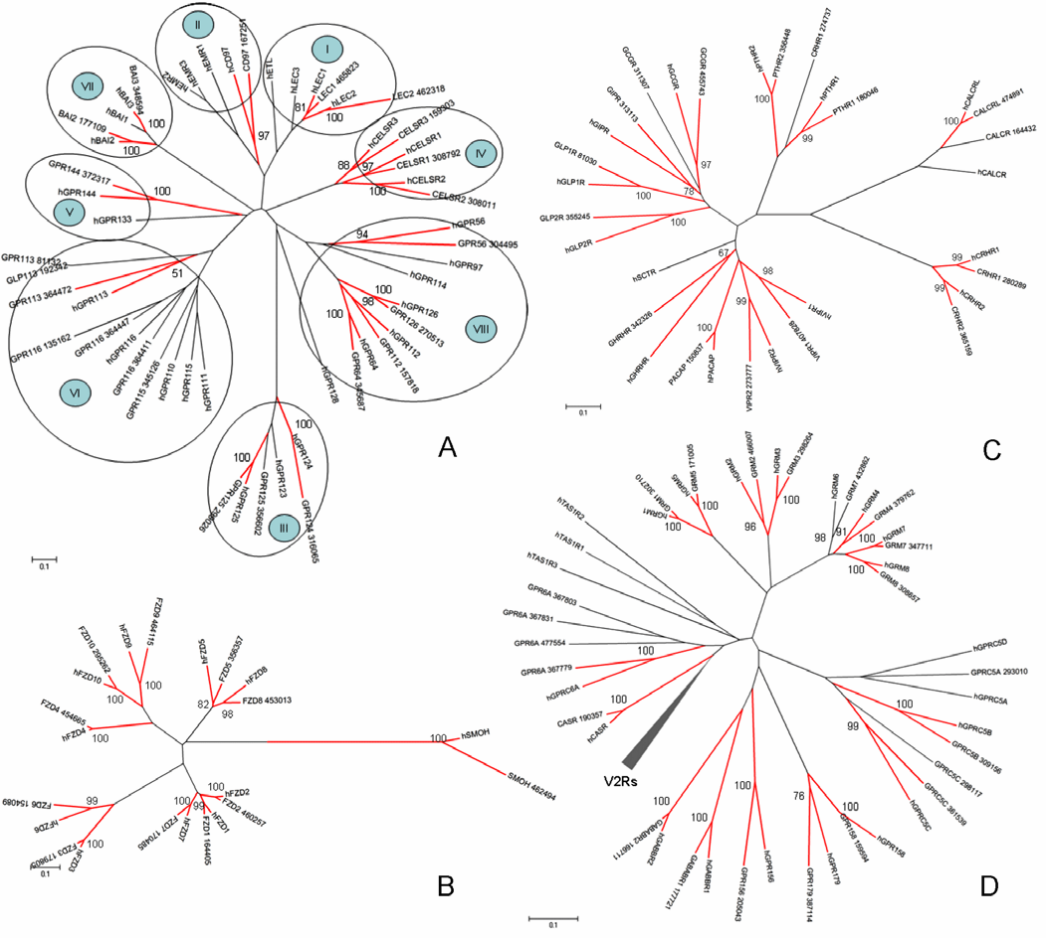
**Phylogenetic tree of *Adhesion*, *Frizzled*, *Secretin *and *Glutamate *families**. The trees were calculated using the neighbour-joining method with 1000 bootstrap replicas. The one-to-one orthologous pairs are represented in red. (A) The *Adhesion *receptor family. I-VIII represent the different groups of the *Adhesion *family. (B) The *Frizzled *receptor family. (C) The *Secretin *receptor family. (D) The *Glutamate *receptor family.

In the phylogenetic tree (Figure 4A), 24 *Adhesion *receptors of *X. tropicalis *were compared with the related receptors in humans. The I-VIII groups of the *Adhesion *family based on phylogeny [14] are shown in the tree. There are 17 cases of one-to-one orthologous relationships between human and *X. tropicalis Adhesion *receptors, while the ortholog to human EGF-TM7-latrophilin-related protein (ETL) receptor and EGF-like modules containing mucin-like receptor proteins (EMRs) seem to be missing in *X. tropicalis*. The location of brain-specific angiogenesis-inhibitory receptor 1 (BAI1) in *X. tropicalis *could not be calculated in this tree, but its one-to-one human ortholog was determined (Additional file 3).

One smoothened receptor and 10 frizzled receptors were identified in *X. tropicalis*, which was in agreement with the findings in both humans [8] and chickens [11]. Figure 4B describes the relationship between human and *X. tropicalis Frizzled *receptors. Indeed, it can be seen that this family displays 100% ortholog conservation between humans and *X. tropicalis*. This high conservation could be related to their crucial role in early embryonic development [15].

The *X. tropicalis *genome contains 16 *Secretin *receptors, while the human genome contains 15. There is a high degree of 13 one-to-one orthologs between humans and *X. tropicalis *(Figure 4C). Only the calcitonin receptor (CALCR) lacks a human ortholog. One of the two *X. tropicalis *corticotropin-releasing hormone receptor 1 (CRHR1, named after their best BLASTP hits) copies does not cluster with the CRHR group, indicating that it may not function as a CRHR. No protein sequence could be found for the secretin receptor (SCTR) in *X. tropicalis*.

### The *Glutamate *family (360 members)

*X. tropicalis Glutamate *receptors represent all of the human *Glutamate *receptors with the exception of Taste 1 receptors (T1Rs) and have an almost 100% ortholog conservation with human *Glutamate *receptors (Figure 4D). T1Rs are responsible for detecting sweet and umami tastes in humans [16], so the lack of these receptors in *X. tropicalis *suggests that it may have other modes for detecting sweet and umami tastes.

Although two copies of metabotropic glutamate receptor (GRM) 7 were identified in *X. tropicalis*, one of them clustered with human GRM6. Both GRM6 and GRM7 belong to GRM group III and share the same agonist [17]. Human GPRC5A (GPCR family C, group 5, member A) and GPRC5D (GPCR family C, group 5, member D) may represent a duplication of the ancestral GPRC5A. Indeed, this is in accord with the findings in chickens [11].

The main difference between the *Glutamate *receptor family in humans and *X. tropicalis *can be attributed to the gene expansions of V2Rs. In mice, V2Rs bind peptides and are responsible for pheromone-induced male-male aggression [18]. The V2R was not found in humans [8], although we identified more than 330 V2Rs in *X. tropicalis*. This is nearly six times greater the number of intact V2Rs found in the mice [19]. V2Rs are related to the CaSR like subfamily of the *Glutamate *receptor family by means of the phylogenetic relationship [20]. Previous studies have revealed two phylogenetic V2R groups (family A + B and family C). Only one gene in fish and three in mice were identified as family C V2Rs, while a large number were determined to be family A + B V2Rs [19,21].

**Figure 5:**
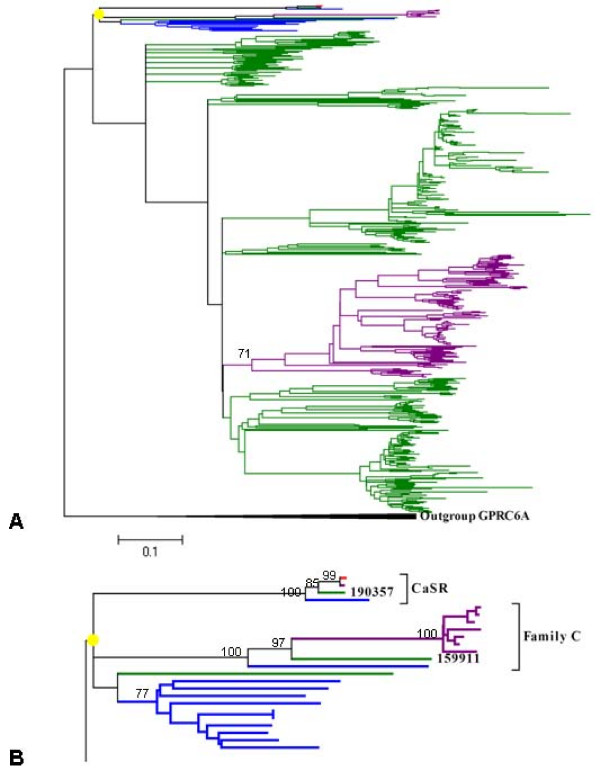
**Phylogenetic tree of vomeronasal 2 receptors**. (A) The tree was calculated using the neighbour-joining method with 1000 bootstrap replicas and contained CaSRs and V2Rs from *X. tropicalis *(green branch), pufferfish (blue branch), human (red branch) and mice (purple branch). The GPRC6A genes were used as an outgroup. (B) Magnification of the marked clade (yellow solid circle) in A. Only one CaSR and one family C V2R were found in *X. tropicalis *according to the phylogenetic tree.

Figure 5 shows a neighbour-joining tree for *X. tropicalis *V2Rs, CaSRs and the related receptor family members in pufferfish, humans and mice (the pufferfish V2R sequences were obtained from [21] and the human and mouse sequences were downloaded from the NCBI Reference Sequence database). According to this tree, one CaSR and one family C V2R were identified in *X. tropicalis *(supported by 100% bootstrap percentage). The fish family A + B V2Rs have a monophyletic relationship. Except one gene, all *X. tropicalis *family A + B V2Rs separate from the fish genes. No particular subgroups of *X. tropicalis *family A + B V2Rs can be distinguished by a high bootstrap percentage. All mouse family A + B V2Rs form a monophyletic clade within the main cluster of *X. tropicalis *family A + B V2Rs. This indicates that family A + B V2Rs were created through multiple local duplications from perhaps a single ancestral gene. To summarize, the divergence of family A + B and family C predated the separation of amphibians and teleost fish. Furthermore, the divergence of family A + B was the results of multiple local duplications.

### The *Taste *2 family (33 members)

Thirty-three T2Rs were identified in *X. tropicalis*. *Taste 2 *receptors are involved in the bitter taste sensing and help to avoid the ingestion of potentially poisonous and harmful substances [22]. In humans, this family clustered together with the *Frizzled *receptors [8], but in our phylogenetic tree, *Taste 2 *receptors are displayed as one independent receptor family.

Previous study has found no orthologous relationship between mammal and amphibian T2Rs, and it was deduced that the origins of mammal and amphibian T2Rs were independent [23]. To better understand the evolutionary position of *X. tropicalis *T2Rs, the phylogenetic tree of 33 *X. tropicalis*, 36 human, 4 zebrafish and 3 chicken T2Rs was calculated (see Additional file 4; human, fish and chicken gene sequences were obtained from [23]). Gene expansions appear to have occurred independently in *X. tropicalis *and humans. However, the origin of mammalian T2Rs still requires further investigation as three human T2Rs are grouped with *X. tropicalis *T2Rs (low bootstrap percentage).

### The *Vomeronasal *1 family (12 members)

In mice, V1Rs bind small volatile molecules and are involved in gender discrimination [18]. Fish are known to possess only a single V1R [24], whereas mice and rats contain 102 and 187 receptors respectively [25], suggesting the investigation of *X. tropicalis *V1Rs might help to better understand this discrepancy. Twelve V1Rs were identified in *X. tropicalis*. The phylogenetic relationship among the 12 *X. tropicalis *V1Rs, 2 fish and 166 mouse V1Rs (fish and mouse gene sequences were downloaded from GPCRDB [26]) was calculated by the neighbour-joining method (Additional file 5). The V1R subfamilies (V1RA to V1RL [27]) in mice are clearly distinct. Fish and *X. tropicalis *V1Rs have a monophyletic relationship that is distant from the mammalian families. The divergence of mammalian V1Rs was posterior to their separation from amphibians, meaning that the V1R gene expansions in mammals and amphibians were independent.

### The *Rhodopsin *family (987 members)

The *Rhodopsin *receptor family is the largest family of GPCRs in vertebrates [12] and perhaps in all animals (Figure 3). In *X. tropicalis*, the family includes 987 receptors that constitute about 68% of the entire GPCR repertoire. If the V2Rs are excluded, this percentage can reach to 89%. The *Rhodopsin *receptor family has been divided into four main groups that in turn are subdivided into 13 major subfamilies in humans [8]. Unlike humans, no distinct clustering into four major groups was observed in our phylogenetic analysis of *X. tropicalis*. Although 8 of the 13 subfamily clusters (MAS-related, MCH, opsins, prostaglandin, glycoprotein, melatonin, olfactory and purin) could be distinguished from each other in the neighbour-joining tree, the rest of the subfamilies (chemokine, SOG, peptide, amine and MECA) were not observed to form distinct clusters. For the above reason, we did not further subdivide the *Rhodopsin *family by phylogenetic clustering.

The main difference between the *Rhodopsin *receptor family in humans and *X. tropicalis *is the number of olfactory receptors (ORs). There are 665 ORs in *X. tropicalis *(approximately 62% more than previously predicted functional ORs in *X. tropicalis *[28]), while 388 ORs exist in humans [7,28]. The number of ORs varies greatly among vertebrates, ranging from approximately 22 genes in *Tetraodon nigroviridis *[6] to 1234 genes in the rats [7].

Previous studies have classified vertebrate ORs as class I or II genes [29], and this distinction was first proposed in *Xenopus laevis *[30]. Class I genes are referred to as "fish-like" genes because all fish ORs are believed to belong to this class. Class II genes are referred to as "mammal-like" genes because the majority of mammalian ORs belong to this class. A phylogenetic tree of *X. tropicalis *ORs with those of humans and fish (zebrafish) was constructed (human and zebrafish OR sequences were downloaded from GPCRDB [26] and the NCBI Reference Sequence database, respectively). The tree (Additional file 6) suggests that *X. tropicalis *has 66 genes in Class I and 599 genes in Class II. The 66 Class I ORs may be further classified into at least 3 subgroups, while no clear subgroups of Class II ORs can be distinguished by the high bootstrap percentage. Significant gene expansion is observed in Class II ORs, and most *X. tropicalis *ORs are mammal-like. There is more diversity in *X. tropicalis *ORs than there is in mammals or fish ORs.

Class I genes are exclusively expressed in the water-filled lateral diverticulum of the nasal cavities and are specialized for detecting water-soluble odorants, whereas class II genes are expressed in the air-filled medial diverticulum and are specialized for detecting airborne odorants [31]. The number of odorants in water is much more limited when compared with the number of volatile airborne odorants. This suggests that more ORs would have been required in terrestrial environments as opposed to aquatic environments. Therefore, the diversity and expansion of *X. tropicalis *ORs might be an adaptation to life in both aquatic and terrestrial environments.

The MAS-related receptor subfamily is related to the MAS1 oncogene receptor (MAS) and the MAS-related receptors (MRGs) [8]. MAS is expressed predominantly in the testis and forebrain and expressed less strongly, but at detectable levels, in the kidney and heart [32,33]. It mediates the vasodilator effect of Ang-(1–7) playing an important role in cardiac function [34]. MRGs are selectively expressed in the small diameter sensory neurons of trigeminal and dorsal root ganglia (DRG) and are involved in the perception of pain [35]. These receptors seem to be missing in fish [36], while the *X. tropicalis *genome contains five MAS-related receptor subfamily members. Hence, it is suggested that the common ancestral genes for modern day vertebrates might exist in the ancestral amphibians. A neighbour-joining tree of five *X. tropicalis*, six chicken and nine human MAS-related receptor subfamily genes was constructed (the chicken genes were obtained from [11] and the human genes were downloaded from the NCBI Reference Sequence database). One *X. tropicalis *MAS has weak orthologous relationship with human and chicken MASs (a bootstrap percentage of 47%), while the other four *X. tropicalis *genes have a monophyletic relationship (Additional file 7). We investigated the gene expression pattern for functional information. However, no EST hit was obtained and it remains unknown whether these receptors work as MASs or MRGs.

**Figure 6:**
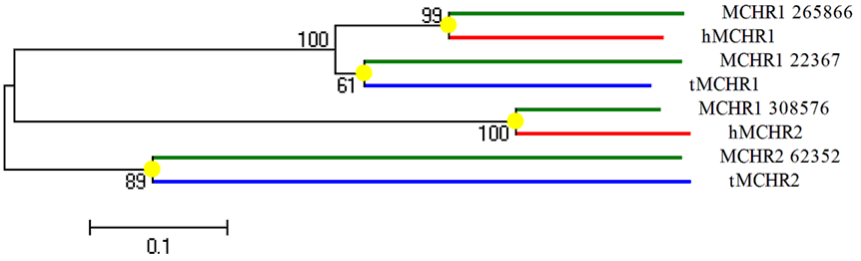
**Phylogenetic tree of *X. tropicalis*, fish (*Takifugu rubripes*) and human MCHRs**. The tree was calculated using the neighbour-joining method with 1000 bootstrap replicas and contained four *X. tropicalis *MCHRs (green branch), two fish MCHRs (blue branch) and two human MCHRs (red branch). Each *X. tropicalis *MCHR formed a monophyletic clade with that of humans or fish MCHR.

*X. tropicalis *has four melanin-concentrating hormone receptors (MCHRs), consisting of two MCHR1s and two MCHR2s. The split into MCHR1 and MCHR2 occurred before the divergence of the vertebrate lineage [37]. The MCHR exists in all vertebrates and its function may be an evolutionary novelty. In fish, the MCHR acts as a colour-regulating hormone receptor in skin, whereas in mammals, it acts in the brain as a neurotransmitter or neuromodulator receptor that regulates food intake, energy homeostasis, stress and behaviour [38]. Figure 6 shows a neighbour-joining tree for four *X. tropicalis *MCHRs, two human MCHRs and two fish MCHRs. Each *X. tropicalis *MCHR formed a monophyletic clade with one human or fish MCHR. This suggests that MCHR1 and MCHR2 gene duplications occurred before the mammal lineage split from the amphibian lineage. Subsequent to gene duplication, a new function evolved. In modern-day humans, only one MCHR1 and one MCHR2 are retained. We investigated the tissue distribution of the four *X. tropicalis *MCHRs in order to support our hypothesis. Because of the lack of EST data in the database, the four MCHR sequences had a common best EST hit identified as the MCHR1 specifically expressed in the brain.

### The extensive expansion of olfactory/pheromone receptors

Vertebrate olfactory/pheromone receptors comprise at least three types of GPCRs: the ORs, V1Rs and V2Rs. In mammals, these receptors are expressed in two distinct organs, the main olfactory epithelium (MOE) and the vomeronasal organ (VNO) [39]. It seems that the VNO, which is absent in fish, first evolved in amphibians [40]. In the MOE, ORs bind volatile odorants and are responsible for the conscious perception of odours. In the VNO, two evolutionarily distinct vomeronasal receptor families (V1Rs and V2Rs) bind pheromones and are responsible for various behavioural and neuroendocrine responses between individuals of the same specie. The ability to detect these particular molecules from outside is important in that odorant detection is necessary for the survival of the individual and pheromone detection is necessary for the survival of the species [41].

As many as 70% of *X. tropicalis *GPCRs belong to olfactory/pheromone receptors. In our phylogenetic analysis, most *X. tropicalis *olfactory/pheromone receptors were found to have no one-to-one orthologs in other species. They were further found to match another gene within the same genome better than one in the genome of the other species. The orthologous relationships among OR genes are often one copy to many copies or many copies to many copies rather than one to one. This indicates that the repertoire of these receptor groups in *X. tropicalis *have been shaped by extensive local expansions: new gene family members have frequently arisen by gene duplication followed by divergence.

Gene expansion is the major mechanism for functional innovation in the evolution of life [42]. The findings among vertebrate species suggest that the ability of chemosensation may be largely determined by the number of related genes [43]. Amphibians occupy a transitional state in the evolution of air breathing and terrestriality, so the extensive expansion of olfactory/pheromone receptors may be an evolutionary adaptation to their special living environment. Multiple specific receptors help amphibians extract information from both aquatic and terrestrial environments, which helps them to find food, identify mates and offspring, recognize territories and avoid danger.

There may be an alternative explanation for these expansions [41]: the lack of specific evolutionary pressure on these receptors. Because of lower selective constraints on these receptors, new gene family members arose frequently by gene duplication. This hypothesis is supported by two facts: (i) in our phylogenetic trees, many genes in the same subfamily clusters are so similar and they are supposed to play the same physiological function; furthermore, (ii) previous study has shown that large proportion of OR genes consist of pseudogenes (over 53%) in *X. tropicalis *[28].

## Conclusion

This is the first study of the overall GPCR repertoire in *X. tropicalis*. We have identified a total of 1452 GPCR sequences from the *X. tropicalis *genome and classified them into seven receptor families through careful phylogenetic analyses. *X. tropicalis *shares a more similar repertoire of GPCRs with mammals than it does with fish. A large percentage (70%) of *X. tropicalis *GPCRs are related to the detection of odorants and pheromones in the external environment. The repertoires of these receptors are shaped by lineage specific expansions which are demanded by functional innovation or are simply the results of lower selective constraints. However, the current analyses are based only on predictions from a preliminary genome assembly and hence may need to be revised.

*X. tropicalis *is a suitable model for physiology and biomedical research. It has adapted to both aquatic and terrestrial environments and occupies a useful transitional state from an evolutionary perspective. The availability of the repertoire of *X. tropicalis *GPCRs can yield insights into the physiological functions of GPCRs and, more importantly, into the evolution of GPCRs in vertebrates.

## Methods

### Identification of GPCRs

The complete proteome of *X. tropicalis *was obtained from the JGI *Xenopus tropicalis *v4.1 database [9]. Protein transcripts shorter than 250 amino acids were removed as they were too short to hold seven transmembrane regions. Transmembrane regions were predicted using the HMMTOP [44], TMMHMM [45] and SOSUI [46] programs with default settings. For each program, a range of six to eight predicted TM domains were retrieved into Temporary file 1.

In this temporary file, some GPCR sequences were still likely to be missed because a few GPCRs might have under predicted or over predicted the number of six to eight TM domains (when we tested on a dataset of 1426 human GPCRs obtained from GPCRDB, 99% of the receptors had between six to eight predicted TM domains). To detect the potential missing GPCRs, additional BLASTP searches were conducted. GPCR amino acid sequences were downloaded from GPCRDB and included the A-F GPCR classes, the putative families, the non-classified and the non-GPCR families. We performed a BLASTP search with an E-value below 10^-20 ^against the complete proteome of *X. tropicalis *by using the sequences from GPCRDB as queries. For each sequence, the top 20 hits longer than 250 amino acids were extracted into Temporary file 2. These two temporary files were merged together into one database.

CD-HIT was performed with 90% sequence identity to remove polymorphisms, splice variants, pseudogenes and duplicates from the database. CDD v2.14 [47] (E-value = 10^-4^) and Pfam 22.0 [48] (E-value = 0.01) were used for a GPCR conserved domain search. The remaining sequences in the database used BLASTP against the NCBI non-redundant database. The GPCRs were named according to the BLASTP best hit if at least four of the five best hits were in the same family. Throughout this article the GPCR families are written in italics.

### Phylogenetic analysis

CDD v2.14 (E-value = 10^-4^) and Pfam 22.0 (E-value = 0.01) were used to identify the borders of the transmembrane region and remove the N- and C-termini. The GPCR sequences were aligned using ClustalX 2.0 [49] with default alignment parameters. Neighbour-joining trees were constructed with MEGA4 [50] using the Poisson correction model with 1000 bootstrap replicates. Gap sites in the alignment were not used in the phylogenetic reconstruction (the complete-deletion option). In the phylogenetic tree, we opted to use a suffix of sequence number (or only the sequence number) for each *X. tropicalis *receptor gene. When referring to other species, a one-letter symbol was used as a prefix (e.g., hGLP1R, zV1R1, etc.).

### Tissue expression distribution

The tissue expression distribution of *X. tropicalis *GPCRs was investigated based on expressed sequence tag (EST) data. The *X. tropicalis *GPCR sequences were queried using TBLASTN against Xenbase 2.3 database [51] with an E-value of 1e^-15^.

## Authors' contributions

YJ has carried out the study, participated in the design and written the first draft of the manuscript. ZZ participated in the sequence alignment. YH was involved in useful discussions and drafting of the final manuscript. All authors read and approved the final manuscript.

## Supplementary Material

Additional file 1 ***X. tropicalis *GPCRs**. Click here for file

Additional file 2 **Protein sequences of *X. tropicalis *GPCRs**. Click here for file

Additional file 3 **One-to-one orthologous relationships between human and *X. tropicalis *brain-specific angiogenesis-inhibitory receptors**. Click here for file

Additional file 4 **Phylogenetic tree of *Taste 2 *receptor family**. Click here for file

Additional file 5 **Phylogenetic tree of *Vomeronasal 1 *receptors family**. Click here for file

Additional file 6 **Phylogenetic tree of olfactory receptor subfamily**. Click here for file

Additional file 7 **Phylogenetic tree of MAS-related receptor subfamily**. Click here for file
