# Supplementary material for: The repertoire of G-protein-coupled receptors in Xenopus tropicalis
Source: BMC Genomics. 2009 Jun 9;10:263. doi: 10.1186/1471-2164-10-263 (PMC2709155; doi:10.1186/1471-2164-10-263)
Supplement: Additional file 2 — Protein sequences of X. tropicalis GPCRs. [file 1471-2164-10-263-S2.pdf]

>jgi|Xentr4|261|gw1.1042.1.1
YRHLVEFLFLVKELNDNPSRLPNVTLGYHIHDSCGASQRALMNVFKILSGTREPVPNYSCV
AMRNIVGFIGDLESQPTISMALILGVLGYSQISYGATDPSLSDRVTFPYFYRTVQSDEEEYIA
LCKLLKYFGWNWVGIIQFDDYSGYKDHRLLMEHLSREGICVAFTISVREQLPIKALNKNIS
KFYLASVFIICGDVGISNLQSSSAEMYYLRRKTFIFLSKWLNYVDVLDYSVLELFHGSLLF
VQKRRNDQFVPRFKEFSDSYKPSNYPEDNLLEDIWIRKFSCLSKDQSKNAHHRYLFHFLN
NCSGKEKLTDLPAYLNPYHSASLMNAVEIMFVALTHMQNSLPKQTSQYRHIVVQTHLKLH
KYLRKVSYTSDGSPALSFTEKGDFIYEYEIVNPQLGSNKTWTWNTVGSYVPWAQRLTLTQ
EKILWKTTNNKLPISWCSESCVPGYRKVTRPGIYMCCYDCVWCPEGEISNTTDSENCIQCP
KMEWPNRKKTKCIPKTVDFLSYTHILSAFFSATSVLFFLASLLILGVFIAHQDTPIVRANNR
SLSFLLLVSIKLSFLSVFLFLGRPVDITCMMRIITFGITFSIAVSSLLAKTIMVCVAFKATKPGS
SWRKWLGVKLSNSVVLFCSSIQIIICMTWLAISPPFQELDIHTSPGTIIIQCNEGSAIGFYSVI
GYMGLLAAVSFVLAFLARSLPDSFNEAKYITFSMLLFCSVWITMIPAYLSTKGKNTVCVEIF
AILNSSAGLLACIFLPKCYIIQFRAE
>jgi|Xentr4|335|gw1.827.2.1
QNNSLSWLACGALALLANGWSILSLASRGGSGQRPRPLELLLCLLAGTHLLMAAIPLSLFS
VVQLRHKHSGYEWNEGLCKVFVSTYYTLALSTCLTVACLSYHRMWMVRWPVSYRLGG
ARRQALQGALGIWAASFVLSTLPSIGWHDNARRFYTRGCHFLASRIGLGFGLCFQLLLLG
GVGTGLGCLGVTIYHACCCPASGQRGGAGEGGVEGKIEVPAIVVQDAQGKRRSSLDGSES
PRTAAQVTGLVGGIVLLYDALTGLPILVVSFVSLRYDSTPPWMVLCVLWCSMAQTLLLPSF
IWSCARYRADLRTVWEHCASMLSDSGADEDSGRDDFADGRLCEVRFDSNGAAIKRPPHY
LGGDLRDGKYLPVRHVLLPTDRGQYLQAPLSRRLSHDEMDLFPPRSSPLLLRWSSSDDIR
GGTGSVRSSSPSSPRRSHNLPPWNRHQPSHANGEESLSTLHQFLSGGMGGSSGFGGIQSSF
FRDEITAFIDDTPCPSPMGTPHRVRASPRP
>jgi|Xentr4|2123|gw1.3411.1.1
LGLALGTISALTVVLNILVLYAVRTERKLHTVGNLYIVSLSIADLIVGAAVMPLNIVYLLNRE
WILGRPACLFWLSMDYVASTASIFSLFILCIDRYRSIQQPLRYLKYRTKTRASLMISGAWLL
SFTWVVPILGWHVFANGGVRSVPESTCETEFHKVTWFKVLTATLNFYIPSLLMLWFYAKIY
KAVREHYQHREVVNGSFQLFYDSKLVQSVRVLKGQQSCTKTQCKDGNLTVPEMNFPAQY
TPISKEFRQSRSTQEKDPVEDYGKQKGFSHCDNNVFKLHCFPLAIIQGSPQECRNYVTVTR
GALEEHNCLRPRQREEISEEHTFAEAASCMVDLSQVGKSNMEAPGRTEARLSGNFRYLKH
TWERFRTHSKQHIQGLHMNRERKAAKQLGFIMAAFMLCWIPYFVLFMVIAFCHNCCNHN
FHMFTIWLGYINSTLNPLIYPLCNENFKKTFKKIFHV
>jgi|Xentr4|3116|gw1.1762.1.1
xGNFLLIILVVTVPALRSPMYVLLSQLSLSDILLTTNITPNLLRLLLNGGGEMLVTDCLTQFF
FYCVSTSIECLLLTAMSYDRYLAICHPLRYYSIMDFKLCLSMSFWSWGLSFLVALVLDLLIG
HLQFCGPFILDHYFCDFSPLLELSCSDIKDAEVTDVILAVPFVVFPFCFIIFTYISIALAISRISS
TNGRHKAFSTCSSHLIVVCTYYGSLIIVYMVPSKGHTFNMNKILSLLYTVGTPFLNPVIYSL
RNNGIKVTLQKCIT
>jgi|Xentr4|3152|gw1.964.9.1
FVLLGIEEMERFKYLYCSLLLLIYFFILLFSCTIISVVLLDESLHEPMYTLIASLLLNGIFGSSC
VFPKLIADLLISSKEISRVGCFTQKFFAALFVYSEISTFTIMAHDTYLAVGHPLRYPTLMTNS
MALKLILGSLIFNIILMLPASVLSARLPLCGTHISNVFCDNPSILYLSCVNDSVSKLYGTINFV
GYLILMALLIAHSYLRILLICLKVSKDACRKAIHTLVTHLLNFSIFLIGVLFLFARYRLGATN

LPLTYHIILSITGFVFPPFLTPLLYGIRMQTLQTKIIQ
>jgi|Xentr4|8679|gw1.16971.1.1
MTSGTKENVSIFILQGFADTPELQISLFVLFLGIYLIILLGNLIIFLVISCNPHLHTPMYIFLLNL
SLIDISSTSNILPNLLHILLTQQNNISFLGCMTQMYVFVSLAASEYFLLTAMAYDRYVAICDP
LHYIARMSRKHCAGLITAAFTVGFVQPVGLVVLISNLSYCASHLINHFFCDVTPLLNISCSS
TFSVELLIYVEGSMLTFNAFLLTLTSYIFIISAILKIQSSEGRQKAFSTCASHLACVITLYGTAF
FLYMRPTRS
>jgi|Xentr4|11552|gw1.190.47.1
LAFIYAIEEINSNKNILPNVSLGYIIYDACSNEVIALDRIFRILSEINEALPNYSCQKRHKPVAV
IGHSLSSTTYTIAQITQLYGYSQLSYGAMDPVFNDQTLFPSVFRTVPNEYLQFEVIVQLLIHF
KWKWVGIVSSDDISNHKASLELIKEMKNRGICVEYHIVIPASANVNMSHILKLIQQSTATVI
ILYCTLLQIINLTKLWHTDESQITGKVFLTSVALNAIDDYIYMESFVTLNGSLLISVHKGEIPG
FMSYLFHRDWERMPDKNFVNNFKMFLKCFTVPSNDSQSRCLLFHNWKNTNYVTSVIYN
TVHILAQALHQMRLDRALFDTESKDLAAESKYKLNRYLKKVHLKSLSDEIFFNEQGSVAG
KFDILNLIIESKNQIRKIPVGSFVPSASPKLIINESAIVWNPCFKKTPVSLCNDICPTGYRKAIE
KGKFPCCYDCIRCSEGEIASSPDTENCLQCPEDHWSNPSRDFCLRKAVDFLSYGNCLGMV
LATAALLFSVCTGAVLWLFIKNRSSQIVKANNRNLSYILLVSLFLSFCCCFLFIGRPVPITCIL
RQTAFLFLYTVAISSLLGKTLTVIIAFHATKPGTRLRKFVGSRVSISLVLLCSLGKLAICSTWL
IWATPFVALDTKTTQQTMTLWCNEGSIATFCVAVSYTAVLALLSFIVAFMARTLPDRYNEAQ
HITFSMLVFFSVWASFIPTYLSTKGKYMVAVEIFAILASTAGLLFCVFVPKCYIILLKGNLNT
>jgi|Xentr4|11772|gw1.378.2.1
VRYYRHVLVIIFAIEEINRNPLLLPNVTLGYEIYDSCDYVSKAVEATLKLFSGRQDHVPGYR
CTTKGPLAAFIGLPYSMAELTQIYRYPHISYGTQDPILDDKTLFPSFFRIVPNDRAMYNGYV
QLLHYFGWTWVGLVASDEEHNMRTSQELRAVLYESGICIAYFAVVSSHYAISYVHVVNVIK
ESKANVVIPFCNHIVFLHLIYSAAQNAQHGRVWIIPASLSFFTDKLLLSILSVLSGSLQFSTH
REEIPGFQDFLYSINPTTFPNFVAAGFWNDIFLCLPPNKAAYRALDDPQNNCTGNESLKSFD
NIFEIKTFRVTYAIYRAVYSFAIALHKMLSSMQLSGYQLNLRNSFQPTMLTQYLKNLHLQT
KSKENVYTTNEGLIGQYDIMNWVTSKGTVTLTEIGSFFPFAPQGQQLIINNSDIKWNHKFK
QVPISTCSTSCLSGYRKATQKGQPVCCFDCILCSEDEFSNESDSTSCTKCPEDMWANEMHD
GCRDKTLEFLSFEEALGGTLAAFSVVGSLFPLSILFIFLRNADTPVVKANNRNLSYILLVSLF
FCYFCALTFIGRPITITCILRQFIFGISFVMCISCVLGKTMMVVIAFNLTQPRSSRRMWLNSR
VTNTLVLVCTAIQVIICAGWLAHSPPFQYTDNKSKAGTIIVECNKGSPIAYSCTMGYMGFL
ASLCFVVAYLARKLPGSFNEAKLITFSMLIFGAVWISFIPAYLSTTGKYMVAVEVFAILSSSS
GLLACIFLPKVYIIILTPEINKIK
>jgi|Xentr4|13191|gw1.251.7.1
IILSTVLGNILVVLAIFTSRALRAPQNLFLVSLASADILVGALIIPFSLAREVMGYWHFGSVW
CSMYLALDILFCTSSIVHLCAISIDRYWSVTKAVKYNLKRTPKRIKRSIAIIWVVSAIISFPPL
LKSKHKEWECLLNDDTWYVLFSCTVSFFVPCLIMILLYCRIYRVAKHRVSSLRNGVSDCNS
AAAGGTCETHHTIQNNHEAEELDLEESTSSVHKFPKKHHHKTKDKPSSAKAKRLSWSSN
RGQQHRDQSICISQMRLTQLREKRLTFVLAVVIGGFVICWFPFFFTYSLESVCRKRCGISDA
LFNFFFWIGYCNSSLNPIIYTVFNRDFRKAFRRILTRSSRR
>jgi|Xentr4|14062|gw1.556.11.1
CQLDESYKHIFLPICYLFTFLLSLGLNSVVLTRCLRHPRNPSLVYMFNLALSDLMYSLSLPF
LITSYISRDRWLFGDPMCRLVRFLFYFNLYCSIFFLTCISYHRYRGICHPMRTMRIETLRWVR

ATCVLVWTLVFALTSPILFFARTGPLDGPVDGVTCWDDALDEDLPKYVPYGVFLHISGFFL
PFSLTAWCYSRVVRTLCRTLKGGVVPGPVPGVAQRRKSIRTIVTITLLFALCFLPFHVTRTIF
LALRAGGAALGGCRALGVVAVCYKVTRPLASANAFLNALLYFLTK
>jgi|Xentr4|14635|gw1.150.34.1
VSDFLIQGFSDDPELQLLPFIVFLGIYLLILLGNLTVVLVISLHSHLHTPMYLLLLTLSMNDIV
STTNILPTLLHLLLTRQKNMTYSGCMAQMYVYISLLLVEYSLLTIMAYDRYVAICDPLRYIA
RMTPKLCLTLIVASLVVSFLDPIGHVTVISKLSYCGSHVIEHFFCDVTPLLKLSCSDTFMVQ
LLNYIEGALVVFSFFLPILTSYIFIISAILKIQSSEGRQKAFSTCASHLSCVIIFYGTLVFLYVRP
TTSYYSKRDKFFALLHIVLIPMLNPLIYTLKNKEFQSAFIKLRDR
>jgi|Xentr4|14699|gw1.406.16.1
MEISNETSSNQFFLLSLADTPFLKALSVLIFLIMYILALSVNSLLIIAVRINLRLHTPMYFFLS
NLSIIDIGLSSSIVPKLIIITITQDTSISRHDCASQMFFHSALIASECIILAIMAYDRYIAICKPLH
YNTITNKKFCISMAAVCWALGCINSSFHVPYTLQLPFCRSHVNHFFCEIPVILHLSCQDTWL
HEVSMYISACTLGLSAFILTLFSYAYIISTILNIRSIKGRHKAFSTCASHLTVVSLYYGPVMFM
YLRPHSRYSL
>jgi|Xentr4|15325|gw1.186.8.1
NSTKCSSVHVPEVVYLTVSAIGLLENLLVLLAVIKNKNLHLPMYFFICSLAVSDMLFSLYKI
LETIIIILANIGFLDRNGPFEKKMDDVMDWIFVLSLLGSIFSISAIAADRYITVFHALHYHNI
MTVKRASVILAVIWTFCGGSGIAIIMLFHDTAMIICLTVMFLLLLVLIVCLYIHMFLLARSHA
KKIASLSGQWNSVQQRANINGAITLTILLGLFICCWSPFVLHLLLYVLCRYNPYCACYLSM
LNVNGTLILFSSVIDPLIYAFRSPELRNTFKKMLCC
>jgi|Xentr4|22339|gw1.38.30.1
SFIGYVLTSITGLIGNAVVIFVLAFIMKEQKCKVWILNLAVADFLFLLILPLYATAVLKQNWI
YGSLLCKLYHFMSSCNMYSSIFIITALNIDRVLSVAKPIWHLKVFSRRVSHFTCAVIWATALL
LSVPVIFLTNEYKSGKADSVTQWHQMKPLIEGLIIPLILIGYFIPLCTIILSNIMIAHQVNKSH
TLKSSKLYRIVMAVILIFFLTWTPLIIAEVTYIVAVNTLNFSLMHNIYTIIPLLTSIAYSNCCLN
PIIYVLVGR
>jgi|Xentr4|22352|gw1.38.32.1
EPVRYASFVLSVVTCALGLVGNALVIWVTGFHMKKYTCKIWFLNLAVADFAFALCLPLYC
VALFTDNWPFGRYSCKVYNYVSTCNMYASIFIITTLTIDRGLATAKPIWHHKFASRRICYST
CAVIWVITALSSLPVFLLINSSDEFMTMGSLDTIPPPDITSETSFLTKCILIPFLVVGYFIPLFVI
LFSNIIIAHKAGKSQSVKSPRLYRIIIMVILFFFLTWTPVVIAPDYFAGLSEQSQLYTHVSNI
>jgi|Xentr4|22367|gw1.27.47.1
SGRQDHSSVVLPVIFGIICLVGIIGNCIVIYTINKKPKGNHNIADIFIVSLSVTDLLFLLGMPFL
IHQLLGNGVWHFGATMCTIITALDANSQFASTYILTAMSVDRYLATVHPIRSSYMRTTCAA
ALVILLLCLCSLITIIPVFMYTQLIEHPDGNAGCGIILPNFSIDIYWYTLYQFFLAFVIPLLIICV
VYIKIQKHISCVVVPLPQRNFRARSKKITRTSVTICSAFFICWAPYYILQLVHLKVTNPTVVF
FYAYKVAISLGYANSCINPFIYIVLSDTFKRHLIKAVCPA
>jgi|Xentr4|22950|gw1.61.25.1
IKAIGFILLAVFGIPGNVYIVLKFVLIRIIEKRLLANNIILTVLALVNLLVVLSRVIPQSMQVM
GVENLLDDTRCKFVIFTYRMSRAMSICITSLLSCHQCILIAPTTKFWMYLKQKVTKNVSFII
LLILLINIVVHPASILYSYSKKNSTSSPYTLHLVYCDTDFITYVSYIVNGSFYACRDFIFVGM
MALASIYIVYMLLQHAKSAAFIKISVSSQRKSAEQKASKAVILLVVCYVLLFGLDNSMWIY
TLTLSNVTADMNDARIVLSCSYATLSPIVIIATN

>jgi|Xentr4|40471|gw1.793.47.1
KDGDIIIGGVFTVHNNIEYNSTGPVRYTSLCTLPLFEFYRQIHSFRFAIDEINRNPDLLPNITL
GYRVYDSCGDPRLAIGSALQILSGPGNVVPNYSCRGKGEIAGFIGDQSSLTSLPIAQLDTHR
YISYGATDPTLNDRKLFPYFFSTGLSDDIQHIAVAELMEQLGWTWVIIVAPDNDSGEKQSR
NLQNEINKRGACVEMIILLTDDIYMNMRKFEQIRITTAEVIVLCGAPSDLMLLSLSFLQKM
APEKTLVITLTWGLIGYEFSLLYNCSLTFLYPQKRIKALNDQFNDYLLSVEEDMLLKDILEK
YPLCHSPEPFNLTDFSNCTKTAALKDLDTYFIINDEVYRSVYTMVHALRTELSVSFICKLYK
CVDVGKLHRYMRNLHFKDPWGEQSYYEEFREILRIYWIINSFYMSDMGISSSLVGKVMFS
QSKPRIEIDIQQIMWKKDTKNQTLKSQCSVNCPPGFRKMPGKSLAPCCYACVPCSHGEISN
RTDMENCLKCEDNKWPNQEKTLCIEKQIEFLSYSDDPLTLIFIIISMMLFIIAAVILGIFISFRD
TPVVRANNHTLSFILLVSIKLSFLSVFLFLGRPVNITCMLRQTSFGITFSIAVSCVLAKTLMV
CIAFKATKPGSRWQKWVGVKLADWLVFICSLIQFLISVIWLVIAPPYVEHNTHSEPGKIIIQC
NEGSAIGFYIVLSYMGLLASVSFIVAFLARSLPDSFNEAKYITFSMLLFCSVWITMIPAYLST
KGKYMVAVEIFAIISSSCGLLFCIFLPKCYIILFKPQMNSKQYLLGK
>jgi|Xentr4|41688|gw1.325.42.1
NILSCVVFLMTFLFGLVVNNLYLWIIGFKMYKSINSTWFFHLISTNSVFTLTMPFVAVYLLM
SPNWIMGYFLCKLINSMVSLCVYANVLFLTVISLDRHTFVFYPIWYRRHMTHRRASAICFA
VWGLSMVSSSPYFAFRQIRLLDDNETTICYNDYSLTGKWDNEQKAIVQMKWLLFSFRLVL
CFLLPFSVITVCYLKIALKMKKGNLVKSSKPYKIIFIAVTSFFLSFIPYHLWYGMSIEEGIFRE
TTLNGLKLLATCLACFHYCFTPVLYLFIAENFKKTLRKSILSLIESVFSE
>jgi|Xentr4|42615|gw1.390.21.1
LQAEFQFGFFTVTYSLVFILGLPGNAIALYYLSQRKQRARSTNVYFLNLSVVDTVFICLLPF
RIYYHNTGNNWVFGDIACRITGGLFYGNIYLSIGFFTCISLDRYLAVVHPLTYRRLRFSHYP
LVLTILIWMICGAIVLPLIFGGPLNNILEANRTSCFEEFSPRSWHNRLVPYNVCALIFGFLVPF
TVIAIVFPLMARKIGKIRKSIHRTVALRIIGFILTVSLICFLPYNITHLFHFLMRLQFIQKCSSSII
IYKLRRITLALISLNSCLNPLLYFIPSLSRS
>jgi|Xentr4|62352|gw1.30.94.1
VYGVICGCGVLCNGLVLAVLLSCRHTLVSDLYVVNLALADLLSLLGMPLLIHQLLHDRGW
VFGDLLCRAVTALDLNNQITGVGIITALCVDRYVAVVHSATMGQRRSVRCTWLVTGCVW
VCSLLLSTPALLYSGVRWGEGVALCVLDLPGAPPSLYWYTLVHSLLTFLLPLSVIVVLYSLT
LHHLSRVMRRVQRAPSQRSRRVTRMALAIVAAFLLCWAPFHVVQLLNLISTGPPSNSAFYL
NQAAICLGYAHSCVSPLLVICCTEGFRERL
>jgi|Xentr4|70564|gw1.8029.3.1
RISFRHYRHLLVFIYTIGEINKDPEILPNVTLGYRIYDSCASGIMSIASALSILSGTEQPIPNYS
CWNNRKVVGFIGDLSLESSLSIARLAGIYRYPQISYGSADPIFNNRLEFPSFYRMIPNELSEI
DAIMSLIRHFGWKWVGLIISDDDTGHRANKRLQEAMSKDGVCLAFLIILKDWSQCNRAY
VMEISKTFYRSSAKVIILFLSSQSVSYISVLFDDDKIPHKIWIATSSLSLISELKYLPALVTFNG
TLVISLQQGEIPGFKQFFYSLNPYKYQRDTFFPYIWGLLLDCTFTETDTSRKKCTGNETFDD
TVLEFYETFNYRIAYGLLKSMLTQESHIFLESTHPPESKMGTHVFLLQSIKPQSFYNSNFDDI
SKNPLKASTLQHLISHIVTHPPESKMGTHVFLLQSIKPQSFYNSNFDDISKNPLKASTLQHLI
SHIVCMKCSMYEKLNSIRNGCILRNIDYLSYEDQLGSTLSSISVTFSITCAVILGIFIKYRETPI
VRANNRYLSCLLLISLMLCFLCTLLFIGRPTQICCLLRQVTFGIVFTISVSSVLAKTLTVIIAF
NATKPGSKLKKYVGTQLAIVLVIVCCLGEIIISIVWLASNPPFPEADTLSDPNYIILLCNEGS
GFFFFCIIGYIGTLALLSFIAAFLAKDFPDLFNEAKNITFSMLGFCSVWGAFVPAYLSSKGSR

MVAVEIFAILSSSAGLLGCIFIPKCYILFLRPE
>jgi|Xentr4|72076|gw1.23.112.1
ILFLVVSALGLIANALILYVVLRYRQMRTTPNIYVFSLALGDLLYMLCLLLFALEIAYTRWP
MGEHLCKVFWVVSTMVAFSSIYFLTIMSVGICVQVHFPIFYSKRLGPKVAIGISICVWLFCL
LLGIPIYIFSGLNDTDSCKINWPNPFWSVTFTAYQIVLAFGVPLIIIGICLILTACTTKCYAHEG
VDKTNIIFLALLTLVFVIIWLPIYVLEMMAVTTNLVVFSEEVYYIISLLPYCKCCIYPIMYGTL
SSSFREIFRSIFCCR
>jgi|Xentr4|72340|gw1.1831.5.1
IIMSQTLIGSLANGFMFLINLMDFASHGSLGSGDALLLCLGLSRFVFQWLLFTIYLLSFWFT
DLAVLYIPKIIFSFLFFSSTSLWFATLLCSFYCVTLSKLNNCFFSIHKKDFDRWLPKYLLFSVA
MSVIFSLPPVYVTFHNMGNWSISGTSSAFLFVRTNSVAFVSVSFLGSVLPFLVFCRAVIVLV
VFLWKHVLKMKRQERTDYKEPSMQAYYRAAKALGSFFLFYSFYIVAFNLYISGIATLNNLI
GCFCTMLIGSYPSVHSVFLILQNTKLQQALASFQQKIRC
>jgi|Xentr4|72564|gw1.1343.22.1
MENQTTVYTLVLAGLSDLPSLQLPLFLVFLLIYLITLTGNLLILLLIFTDSHLHTPMYFFLGT
LACLDMGYSSVTVPRMLFDLLRELRMISVPACITQVFFLLFFAVSETSVLAVMSYDRYIAIC
QPLHYMQIMSWNVCVQFVSGVLVIVTIYSLVHTLFLTKLTFCLPNILQSFFCDLPQLLEASC
SDTFINRLLISVLGILFGVIILGLTFYPYIPIITTVLKMTSKHSRAKAFSTCSSHLTVVSIFYTTS
FFDFFRSNANDHLVEDKVASVFYAILTPSLNPVIYSLRNQELK
>jgi|Xentr4|81030|gw1.1039.34.1
SICVPHRHLHCTRNYIHLNLFTSFILRAISVFIKDSVLKWMYNLAMNDNQWEGLVSYQESL
SCRLVFAMMQYCVAANYYWLLVEGIYLHTLLVLSVFSEQRLFRLYLCIGWGVPVLFVVPW
AIVKYLYEDNGCWTRNYNMNFWLIIRLPILMAIGVRFRINARVNFPFICKDLNIQKSCSSLH
DCRLAKSTLTLIPLLGTHEIIFAFITDEHAKGALRYIKLFFELSFSSFQGLMVAILYCFINNEV
QTEFQRRWERWRLEHFYSHRDSGLKPIKFPANSLSSGVTVGSS
>jgi|Xentr4|81132|gw1.831.28.1
GSQVQLLFNSTKEVTNVRWFIKNKYWILMTELYNGAKISIETSAFNSTLTIRDSTRMWGGQ
YICNFIYKNLYWQVNYTVDFPLRSQDITNIPARASLMSNGFLGITLECCVWDDGQNYTVL
WDPGAMSSVVLRGTWNLCYTLSITTVPAADTNFTCRFQGNVEQVAEAQISIVVIQAGDLY
CPQDVFNPSWKETKAGYDAEILCPEGKSGTITRSCNLYGWWAFPYFKCQDIKVLSLLEQA
KWLKGGLGMPATEIPLMIGTLKYYTMGRGLYVSTSWDVTAAIQALYTISLAAIENNIVISV
AIMSDFIAVCSQILEYEMPNLWYNIHYEDPAIGSKFTQSLENMLKVLEPQTKDLQIIAPNIEL
NVLVLESSTLASYSTLNVNPRVQMFFKSTALQELTQTGNLTMASLAINKLSKLLANNYGD
SLKGSDHAIKDLILMNVFNINQDGIDVIFERASDSNKTKVDLSRCVLWDYNLFDGNGGWS
AEECVTYNKDNLTVCSCSHQMSLSLVTFIPTAQEECRWKSLSLFSRSTSIASLTLSLIIYLVE
WKFIVKTLISFCHQLSAVNIALCWLIADLCYLGSSCIMSTTPTPDLCIAAAFFKHLFYTAASF
WMLFEGLFLFQQLVLGFLKLQKKALVVIMLAIGYLCPLIIALVTLSLYHPSGTYINADTCFL
NVDNGVINTFSAPVLLISFMNIFVVIVGIWKLMKPSQSERSEDYDREDLIAIFKPLLILTTIFA
LTWVLQLAMSVMCGVQGFLDFISTLLNSFQGFFISVFSCLMDKRV
>jgi|Xentr4|81355|gw1.631.89.1
YLEIQALILTVDEINRNPDLLPNITLGYRVYDSCGDPRLAIGSALQILSGPGNVVPNYSCRG
KGEIAGFIADRSSLTSLPIAQLLGTYGYSQISYGATDPALNDRVQYPYYFSTGPNDHIQHIAI
AELVERLGWTWVIILAASGDYEDRGSKNLRNEITKHGVYVDFIGTLTEDRKTDRKTLERIQ
MSTAEVVILYGEQFQTKALSVHVEEMIKDKTLLVPVTWFTSYLVYLFNGSLRFEEKIVEFG

YTTKFTTHVMAIEDDVLLKDLMATASYCLTQDEEKDELFQKVNKFIYFNCSKLQLIPRIFY
PSHRVHRAVMGLAHAAHDLLSTHRNKATHINSHRKEVTMTFLQEKSQEKTFSNFHATIQK
SQNTALKTQISKVSRQNKNLQGREAIDFYMNSASLTWQILIFNHSDSIYMVLAAYSSVKPT
VTSLKSLSPSCRCQRQPSYSPSISYLDEVYFLADAENCLKCGEYEWPNQEKTHCIQKQNEF
LSYRDDSLTVAFILLSLVFLIIAAVILGIFISFRDTPVVRANNHTLSFILLVSIKLSFLSVFLFLG
RPVDITCMLRQTSFGITFSIAVSCVLAKTLMVSITFKATKPGSPWRKWVGVKLENGLVFICS
LIQFLISVIWLVIAPPYVEHNTHSEPGKIIIQCNEGSAIGFYIVLSYMGLLASVSFIVAFLARSL
PDSFNEAKYITFSMLLFCSVWITMIPAYLSTKGKYMVAVEIFAIISSSCGLLFCIFLPKCYIILL
KPEMNT
>jgi|Xentr4|83743|gw1.325.92.1
YGAMRIFSIICHSITCLVGVIGNGLVIWIAGFKMKSISAKWYLNLAITDFICSVSTAVRIAEWI
LVDDNILCEFSFSLLILNMLTSVYFLTAISIDRCITIMWPFWAKSHRTKRSATTAIVVIWVVS
VLFSVSNGFINWGFHVLLMCAPAESNYYRVFYKSVVKKPGILMQSIHITLFLVMFLLPFTII
LLCYGLIICKVASVRRRNKSQRSLKIIIAIVTCFFGCWFPYNLWQFIALRSRNDHIVADLIISS
VSVCLVYTSSCLNPILYVFLGREFKSSLTRSIPAILKNVFSD
>jgi|Xentr4|83822|gw1.325.97.1
MSITCFSLTCIIGTVGNGLVIWITGFKMRKTMTTTWFLNLGISDFSFCLFLPLYITEVAMWG
NWPFGQIMCKVRFFIISLNAIASVLFLMAISIDRCVCVLCPIWSRNHRTSSSAAIISVIIWFFS
VALSSSYLLMTDINIYYISVCLVTNRAWENITTTDETFNQTFQAIVIFDCVFTLLIPFSIILVCY
GLIVFRVKKSRRIHGSAQTLKIIVTTVICFFFCWVLYSVLPVIDVVGHYIPLEHRFLIYTLAD
CLAFFNSCLNPIIYVFMGRKFKQVLRKSIPFLLESTFRE
>jgi|Xentr4|84118|gw1.672.51.1
ILAVTAGAFTNAYITFVILLDYFKTKMMSSSNKILLALSLSNGYFSFLLFVCSIISFVWPHIAT
NNYIKGCILALLIFGISSTAWITTCLCVFYFVKIINFSSGLFAWFKLKIDIIVPWLILVSEVVSL
GCSFLTLLPSVNIQEPSSNSSMFYSLNSTSGATGISADFIKVTFIAVCVPLLIMIVTTFPTIRTL
YLHSRRMKNTGTSSSLAPHQSAVFMMAWLLFLYTVFFVVLFTGFIQSFTPPSFAYWMTYN
LIYVATLVQSVVQILGNPKLKEAITICC
>jgi|Xentr4|84303|gw1.532.131.1
LVLAGLSDLPSLQLPLFLVFLLIYLMTLTGNLLILLLIFTDSHLHTPMYFFLGTLACLDMSYS
SVTAPRMLFDLLRERRIISVPACITQFYFFLFFDISEMSVLAVMSYDRYIAICRPLHYMQIMN
WKVCVQFVSGVLLFSAVDSLVHTLSLTKLTYCHSNVLQSFFCDLPQLLQASCSDTFINLLLI
FLCGILFGGVILGVTLYPYITIIRTVLKIPSKQMRSKAFSTCFAHLNVVFIFYTTTFFNYFRTN
ANDQHMEDKIASVFYAILTPFLNPLIYSLRNQELKISLKKTLQK
>jgi|Xentr4|84330|gw1.657.94.1
MNTTSSCNQSKITEFLLVGFSAPRPLRVLLFSICLVIYIMALGANLMIIALYLGSHHLRSPMY
FFLSNLSATDISLSTNVGPNLLCTLFKDGNPMSVSACVTQFFAAGFFTGFECLLLTVMAYD
RYLAICKPLHYVTIMTNKHCLHLVILSWVEILLLSLSGTILTSVPGFCGCNTLDYVYCDFAP
LLKVSCSDAFVMASMTTIFIPAGVVLLFFSPLFIIITTYVSIFLSIFRISSKTGRQKAFSTCGSH
LTVVCTYYGILISKYAAPSKGQSLNVNKLTSLLYTLVTPLFNPIVYCFRNKEIQKTLSTLIS
>jgi|Xentr4|84485|gw1.327.52.1
YKNLTTVREFLLLGFENLYNFNILVFLLFLIIYILTLVGNLLIIVLVSISKKVNSPMYFFLSHLS
LSDILLTTNAVPNMLRVLLKGRSPISITGCLTQFYLLCASTIGECFLLMVMSYDRYLAICHPL
RYTSIMDEKLCLCLCLVLLSWMLGLMLPLIAVIPVFQLHFCGPNVIDHFYCDFAPLMELSCSD
TSLIEREVFLCSVPTIVLPFIFIVVTYGYIWHTIIKIPSSNARKKSFSTCSSHLAVVSAYYGTLI

TKYMVPFRGHSVTLNKCVSLLYTVFTPLFNPIIYSLRNKDIKEALK
>jgi|Xentr4|84501|gw1.1189.14.1
MENQTTVYTLVLAGLSDLPSLQLPLFLVFLLIYLITLTGNLLILLLIFTDSHLHTPMYFFLGT
LACLDMSCSSVTVPRMLFDLLRERRIISVPACITQFYFFFFFVLSEASVLAVMSYDRYIAICC
PLHYMQIMSWNVCVQFVSGVLVISAIYSLVHTLFLSKLTFCRQKVLQSFFCDLPQLLEASC
SDTSINRLLIFLLGILFGGGVLGGTIYPYIPIITTVLKMTSKHTRAKAFSTCSSHLTVVSMFYS
TVFFNYFRSNANDHLVEDKVASVVFAILTPSLNPVIYSLRNQELKLSLRRALG
>jgi|Xentr4|84696|gw1.1115.48.1
LTGLSDLPSLQLPLFLVFLLIYLITLTGNLLILLLIFTDSHLHTPMYFFLGTLACLDMGYSSTT
VPRMLFDLLTDRRVISVPACITQIYFFLCFCSFETSVLAVMSYDRYIAICRPLHYMQIMKWK
VCVQLVSVILVFGAVYSLLHAVFLTKLTFCRPDALQSFFCDLPQLLQISCSDTFINVLLIFVF
GILFGVSLLGVTFYPYIPIISTVLKIPSKHTRSKAFSTCSSHLTVVFLFYTTTFFNYLHSKTND
HHTEDKVASVFYAILTPFLNPLIYSLRNQELKASVKRTLQR
>jgi|Xentr4|84842|gw1.964.42.1
TNFILLGLVEMESLKYLYSVLSLVLYMFTLLSNLMVVLVVLGNESLHQPMYILIANLVVNG
LFGSSAFFPKLIVDLFFSLLMISRAGCFIQAFCLLVATYCEISTFTIMAYDMYVAVCNPLRYG
TLMTSTVALNLIVGSLFLNLTLALSTVLLATRLPLCGSKLNSIFCDNMSIINLSCVDTSLNKV
YGSILFISYLLSAILIIAVSYIQIFLVCLKVSKDASRKAIHTVVTHLLNFSIFLIGVLFIFIRYRM
ENTGIPLYIPAVTALVFPPMLTPLIYGIRIKALK
>jgi|Xentr4|85100|gw1.672.60.1
IVISVVGIVINMFILSVNFHSWIKGQSLNPSDLLIVTLAFSNLVFSVTAGVWIIYFGFITYGDF
KEYLSYSIMVYVLFCNSWLSTCLCFYYFVKVSNFKPGYLARLKSKINTLVPRLILGAQVFS
ILNSLFYMLTFFKVNNDNSTLLFLTNKASSTTNNRIDVFYNAFFLLVNCLIPFFIIVVTTSLII
ASLYKHTRRMQRNVGEFGGPSLHIHHRAARTMISFLIIYLSFYVLSLGNSIFLNKHLLNWV
NYMLGCAFSPTQSIVLIMGNSRLRQTCRNIL
>jgi|Xentr4|93752|gw1.68.122.1
SPLLHGVSVACQALLLLAIFLLSCLGNCAVILVIAKHRQLRTVTNAFILSLSVSDLLAALLCL
PFSFLLLLRGGAWPFGDRLCLASGFLNCCFGIVSSLTMTLISMDRYYAIVRQPQGKIGRAR
ALQLLLAAWLLALAFSLPWFLLAREQWQVQRKGHYHCLYVFHSAGSRLGAAYSISLIVLC
YLLPFALMCFCHYNICKAVRLSEIRVRPVTTYAHLLRFYSEMRTATTVLIMIVFIICCWGPY
CIMGLVAAAGDYPFTPLMDTVAIWMAWANGAINPLIYAIRNPNISMLLGRNREEGYRTRNI
AAYLCTQGQNRDPIRDRYGNRHGQGSRVSSCSPANGGDVAMWACKNPTVLFCRDGQPDT
VTDNSVGIKPETVDTSL
>jgi|Xentr4|94320|gw1.94.88.1
PRSFPGEIISKNNRTYLCISPSSIDHLKSPITVLVIPSIYTLVFLVGLPANVVALWVLATRVKK
MTSTVFLINLAVADLLLIVTLPFKISYYFLGNDWIFGETLCRVVTSFFYANIYCSVLLLMSIS
VDRYMAVVHPFFSRTFRSKNFAIFMCTISWMIAALSILPLAAMRQSYPLSTTDLTLCHDGLP
RNEQVTYLFYYFLCLVVLGFLLPLSIIIFCYVSVIRVLMRNEGKYGYALKLIALVLVIVIVLL
TPSNVVLLIHYSERFLHTYGDLYSVYMVCLAISSLNSCVDPFVYYYVSDEFREKVRQQFR
KRSKLSITS
>jgi|Xentr4|94372|gw1.178.214.1
ELLITGTFGTVLSLMYVSGVAGNVYTLAVMCQSVKCGASMYISIINLALADLLYLSSIPFIV
CTYFARDWYFGDLGCRILLSLDLLTMHASIFTLTGMCTERYMAVTKPLDTVRRSKGYRKG
MAGAVWSLSLLLTLPMMIMVTLTEGKGKGVKRMCAPTWSLDAYRIYLTVLFSTSIMAPG

MVIGYLYSRLARTYLESQRNPINKKENKRSPKQKVLIMIFSIVLLFWACFLPFWVWQLLRL
YDMSPQLSSQTQKCINYLVTCLTYSNSCINPFLYTLLTKNYREYLKNRHK
>jgi|Xentr4|94799|gw1.325.117.1
PAAMVLASVFLFVALLVGLVVNSLYLWVLRFRMSRSINTTWFFHLILSFFLSTFTIPFTAVYV
LMFPHWPFGDLLCKLTDYLLNVCTYTCVFLFTVISLDRYCLVFHPVWYRGHMNNRYASAI
CICIWGVAILFNFPYLAFSQTHLLEDNKTSICIDYILSMSIWPKSATQLKWVMFSFHVILSFP
LPFAVMTVCYVRIALWMKKGNLARSTKSYKVMFITVASFFVSWLPYPLSYAMIGRIDESTS
NILLALVICVSCMSYCFTPLLYLFIAENFKKSIQKSVLLLIESVFNE
>jgi|Xentr4|95013|gw1.57.116.1
CDKNDIYEFATLFNTILNSVLFSFSIIGNCLVLWILIKYESLVSLTNVFIFNLSIADLILSSWLPL
FIVYHRQGWVFGEVACKILNAFFSIGFYSGIIFLTFMTFHRYLSVVDPLSALKAKNPLFGVA
ASLLSWLISICASIPVIIYKAQVDRNGFIICEYRDNLPHLVSNYQQNIVFLIAFVVIIVCYFSII
KTLRRSRSQRNHKPVKLIFIIVVVYFVSWAPYNIVMLLQSFEKQQLFKSFRDCDFSKNLDY
AKSVSEKLAISHCCLNPILYAFVGIKFRQHLKRLL
>jgi|Xentr4|95053|gw1.24.375.1
NQTSGISFLLLGFQNSQIINEFLFVPFLWIYILTLFGNLLIIILVITVSALRSPMYALLSQLSLSD
VLLSTSITPNFLWLLLNGGGTISATGCITQFYFYGVSAGSEVFLLTAMAYDRYLAICSPLHY
VSIMGFRLCLYMSLCSWGLALILGLFFNRLTFNLQFCGPFVIDHYYCDFTPLIKLSCTDYKA
VELTHIILLLPFVLLPFCFINYTYVSIGLAILRISSTEGRHRAFSTCSSHLIVVCMYYGTLIIVY
MAPSKGHNFNINKIMSLLYTVGTPCFNPIVYSLRNSDIKVALYKWIS
>jgi|Xentr4|95220|gw1.24.444.1
GNTTQLTHFFLLGFDHLQSITTYLFILFLIIYILTLFGNALIVGLVSVSQRLKSPMFLFLRNLS
LCEMIFTTNIVPKMLQVILEGGCKISLMGCALQLYAFGATGIAECLLLTSMSYDRYLAICKP
LHYTKIMNFRCYLWLVVLSWAAAIILPTISISMILQLNFCGSSVVDHFFCDLAPVLELSCSD
TSPVEFEVFAQTIPVFVFTFIYIMATYISIFIAILKIKSTMGRQKAFSTCSSHLTVVCTYYGTM
ISLYVTPRGRQSVKVNKTLSLLYSVVTPLLNPIIYSFRSAEIRKALASIIFRKK
>jgi|Xentr4|100987|gw1.616.169.1
LIIYIIVLFLVPCTSTVQTINPACELKIIPTYEDYEYIQEGDIMIGGALTVNSYTIPFRYAQDGY
LRMACVDVFPEYYRQLVDLLIAIKQINQSPNVLLNLTLGYHIYDSCGDPRKAVRSVLQILS
GTREPVPNYSCVGKRNIAGFIGDLSSENTVAIAQILNLYGYSQISYGATDNMLSDRISFPYFF
RTTQSDRGHYFVLSKLLKYFGWTWVGIIRLDDYGGEKEHQLLKSILSNDGICIEFTIKITGY
ISRTEIETNIPNYMVYKRVIEESTASVIVFCGTVPTSAVGELIHLSDLFIKKTIIVSYNLASSM
HFMDYAIEIFNGSLGITQMLLGSSYSPENTQVLKEFHPSKYPKDKLLEDIWMQYHSCLSKD
PAKNKAYEKLYSGFLYNCSGRELITNVRHFDNKIYSPRVLLAVDIMARALGIMISLANRTTE
KAKKEQNYRNQMHHYLKKVQYNFSDPTMNRTISFEEDGEFITQYKIRYVTFQSYKQITMQ
HFGVFTPWAPPDKYLKFISKIGWKTKNNEVPKSQCTDNCLPGFRKVPKPGAHSCCYNCVP
CSNGEIANTSEWAHRDIKSQSPERERGKDSCTSDQPLQLLFGHYFLQTVELLLWDFGTVFL
LFYFLEGFVAVLSFLLLVSIKLSFLSVFLFLGRPVDITCMLRIITFGITFSIAVSSLLAKTIMVC
VAFKATKPGSSWRKWLGVKLSNSVVLFCSSIQIIICMTWLAISPPFQELDIHTSPGTIIIQCNE
GSAIGFYSVIGYMGLLAAVSFVLAFLARSLPDSFNEAKYITFSMLLFCSVWITMIPAYLSTK
GKNTVCVEIFAILTSSAGLLACIFLPKCYVILIKSEMNTKTHLLGNKM
>jgi|Xentr4|101163|gw1.779.104.1
VLIYLILVYVGPCGCRNQPLNSACNLQIFKAVKEYEYMQEGDIIIGGVMASHFYMFNFTFP
WDNSTGFACYTPNQQAYRYLVDFRFAIEQINKDPARLPNMTLGYHISDSCGDPRKALRSVF

QILSGTREPVPNYSCVGKRNIAGFIGDLISKTTVPIAQILTLYGYSQISYGATDPSLRDRAAFP
YFFRTIQSDEANYFAISKLLSHFGWTWVGIITSDDMSGEEESHSLAKYLNREGVCIEFTAK
MKISKANDFSLNQYKSTIERSSTSVIIFSGTASIMFVNALTSAFRQKTLILSPNWGNNDIVLG
YNIIIFNNSLVFVPRYHYDLGTPEMSRFLEGLHPSKFPDDELIEDIFLMFHLCLSKVRIKNNL
YEYTSPHTLRNCTGQERITEIDCFNGETNSPLVHVAVDIMSQALHEMNMLFIRKSNQHPYK
YQGLHYFLKNHQYSPQTGPTSSFDEHGEYMSGLWIYNYIISPERDLNRKLIGEFSPRAPPD
QQLNVTSSLIQWKTNYNEIPRAQCSDNCLPGFRRAQRPRAQSCCYDCVPCSEGEISNRTDL
FSAGKLCLLLPTPIAGTENREPDLLTSAGILIGGFFCILMLPLALESWEMLHIMVLIINQICLR
ANNRSLSFFLLVSIKLSFLSVFLFLGRPVDITCMLRIITFGITFSIAVSSLLAKTIMVCVAFKAT
KPGSSWRKWLGVKLSNSVVLFCSSIQIIICMTWLAISPPFQELDIHTSPGTIIIQCNEGSAIGF
YSVIGYMGLLAAVSFVLAFLARSLPDSFNEAKYITFSMLLFCSVWITMIPAYLSTKGKNTV
CVEIFAILTSSAGLLACIFLPKCYIILFRPEINTKSHLL
>jgi|Xentr4|101173|gw1.745.118.1
VKPGDWVIAAFLQLNDIFLTSDPGFTSRPDSVLCTRATFRYYRHYLAFIFAVEEINRSPWILP
NITLGYQIFDSCAAGHKSLSGALDAISGKKLNVPNFSCWGNSKMVGFVGDLSTSSTYSIA
QLVGVLSYPQACISYGATDPVFNDRTQFPSFYRTIPNEEAEIDGIVQILKHFGWKWVGLIIS
DDDTGYRARERISKELANSGGCLAFTSVIKQNRYIDEEHENRIVGEIEKTTANVIVLFISTKF
TYGFAHLFSRHKIPPKLWITSSFIPSILTFEEASMETTLNGSMSLMIQEGKIPGFEQFFYTFSP
NNYPNDALIVSTWQKLFNCYFIDTPSFIREIPGEPVLRTCTGNETMSEADVSVYGNHNYRV
TYRVYTAVYALACALHNLYSAQPPANHWGSKLNQFVRNVTFTIFPNDTHSFNKYGDPPAR
FDIIKWLFSPGHHIVTRKVGSFNASDHATQFYINHSADLWGPLFNMIPQSLCNEPCAPGYR
KSKREGAPSCCYDCVSCVDGEMSNTSDSPKCFKCPEYQMSNKERTACVSKFINYLSYEDT
LGASLASVALMLFLTTSTVQGIFVKYWETPIVRANNQNLSCLLLISLMLCFLCTLLFIGRPT
QICCLLRQVTFGIVFTISVSSVLAKTLTVIIAFNATKPGSKLKKYVGTQLAIVLVIICSLGKIGI
STMWMASCPPFLEADMFSEMDTIILKCNEGSVTFFFCIIGYIGTLALLSFIAAFLAKDFPDR
FNEAKNITFSMLGFCSVWVAFVPAYLSSKGSRMVAVEIFAILSSSAGLLGCIFAPKCYIIFIRP
ELNTR
>jgi|Xentr4|101511|gw1.290.214.1
YQHYLAAIFAIEEINQNANILPNLTLGYRIYDACTSESRAAANTLSILTGRAASVPNYSCND
KGTLAAFVGHLLPTLTHVVSDMTKIYRFPQISYGAQDPALDDRQQYPTFYRTIINEKYHFV
TISQMLKLFGWTWVGIIYSDDESHQRAANKISSEITISGGCVEFVAVYTTENILTLSITIEKIR
HSSANVIIISSNSHMLLHIVILIEMTTTLKKHLLFPSSLENMSSFAYSSLNGSLIVSGNQGAIP
GFKEFFYTAHPTKFPNDEITALLWLVLFSCLKYGDNSSGIYIPACTMNDTLSFFGMSFYAVN
NLHITYNMYVAVYALAHALHKAYKGNYFLETTVNTFMFLFQLNNHLKKVHFQTVSGDEI
FFNEHGEVQGNLEVENCISYPNETTAIRKVGTFNSSAPPGEQLLINKPDIIWGTPLNDIPQSV
CIGTCQPGYRKAGIKGRPACCYECVQCSDGEIANVSDMENCIRCQEDEWSNENRIKCIKK
TIDFLSYAEPLGLSLAILAILLSITASLVLGIFIRNRHSQIVKANNRELSYSLLLTLILSFLCTLF
FIGRPLKVTCMLRQVTFAVIFTISISSVLGKTVTVLIAFHAITPGSMLKIWVGKRVPRCLVLL
LSLGEVIICITWFLHCPPFPDYDTRSQPSVMTLQCNEGSAVAFYIAVGYIGMLASVSFITAYL
ARGLPDIFNEASHITFSMLVFCCVWIAFIPTYLSSKGKYMVAVEIFAILSSSSGLLGCIFIPKC
YIILLKPTKSVK
>jgi|Xentr4|101799|gw1.3102.5.1
QDFRRLIDLRFAVEQTNKDPARLPNLTLGYHISDSCGDPRKAVRSVLQILSGTREPVPNYSC
VGKRNIAGFIGDLSSETTVPIAQILTLYGYSQVRKTDIISHRGVPWPYNCIYSAFYTGHGTPS

HFGWTWVGIITSDDISGDKEHQILAKYLFTEGVCIEFTIRIKMNALSNIHSDVSIIKYSSTRII
LLCGTVSLYFLTILLFFMKSVLLEKTLILSTNWGTNDMSVRYAHDIFNCSLSFVPGFHYHLD
TPEMRSFLETLHPSKYPEDKLLENIWMIFHFCLSEEPIKNHLYEFIYINTLHNCTGQERITDL
PYFTADYGSPQVHLAVDMMSQALHEMSIQLREKAVKTREMYHYVHQVIINTKVPNVHSN
TKQTVNKEAIILHRIIIFIIPYLYITLYTVFYITLTHVYIKHFISSILKIQVKIPTTHTIIPRAQCSD
NCLPGFRKAPRPGAQSCCYDCVPCSEGEISTITDSIRCSKCPDMEWPNKRKNQCIPRREDF
LSYASDKISVCLSVIAILFFLITLWISAVFVTHRETVIVRANNRSLSFLLLVSIKLSFLSVFLFL
GRPVDITCMLRIITFGITFSIAVSSLLAKTIMVCVAFKATKPGSSWRKWLGVKLSNSVVLFC
SSIQIIICMTWLAISPPFQELDIHTSPGTIIIQCNEGSAIGFYSVIGYMGL
>jgi|Xentr4|105402|gw1.74.176.1
PATFLFVVLVGLSLNLSVVWILFSRIKRWTRSTVFLCNLALADITWILCLPCLIYYHFNGLH
WSFGNALCKVTRTLYHSCFYCSIYFVSCMSIDRYLAIVHPLKSLRMLRKSQALALCLTIWAI
TFISSVPVTFIAGTELCENNKTICSLYVFSKNTDVSLPLSLFSTVAGCLLPFASICYCYCSSLG
QLRRIELQRLKKRDMLIKLMFSAWVIFALLYLPYHASRNSCIILRVIQPKLDKAIETADAVFF
IEMAVCSLNTCINPLFCFLAGADFREQVCR
>jgi|Xentr4|105604|gw1.55.257.1
VIYIPTFIFGLVFNLLALWMVFCRIKKWVEATTYVTALIVFDSLLLFTLPFKMTAYREGNEW
SLGSSFCSFLEGLYFLNMYGSILTSVCICVDRYIAIQYPFYAIILRSPKKATFLCLFLFTLVWGI
TIWYLCKPQKNKRGPQCFYGFSVVIWMNVALIITLELVFFISSILMTFCTAHITFTLKRKTQL
DPSISSARKSVRILLGNLFIFLFAFGPFHLSLLLYYFVRNGIISADLKETMRVFIQVSLCLANT
NCCLDGLFYY
>jgi|Xentr4|105619|gw1.366.113.1
HTFIPVFLSFIFSVGFLLNCLSLWVFWFRIKPWNPSIILQFNLAISDAVISPAAPMLLIYSFSDH
WSFGTFACQYSVFSLSTNMYGGVYFLTLIAIHRYCTIVHSGKWNVLAKNSFITKLCLGLWA
FLFLQGLPFFFVLRTSVIDGATKCLSIHQTEQVLLFFIWNWAITILGLLVPFLLTVLCYSLLSQ
HILRANPMNPQSQTMISRSLVTIGVSLTIFIICYIPVHITRTAGVTIKLFSPESCSLLVKTEAVY
YFSWMLSVANCCIDPVLYCFASNRFRK
>jgi|Xentr4|111277|gw1.1227.20.1
KMIQFIFSLAALWVTPCSTQLSGSDSQCCIPLTKPKYEYKYIQDGDIIIGGVFSVNYGVKYIL
DSNGKYIPICINPVQDRYIEIQTLLFTINEINKNPDLLPNVTLGYHVYDSCADPRLAMKSVL
QILSGPGIIVPNYSCGDQREIAVFIGDRSTVTALPIAQLLGTYGHTQISYSAIDPVLNDRALYP
YYFSTGPNDYVEHVAIAELVEYLGWTWVIILAAGDERGERESKNLMNELNKHGACVDLI
GTLTEDINTDTRTLERIQKSTAEVVVLCGEKFLTKTYFVQKMIKEKTLVVPATWIPGYTFPL
FNGSLVFKEEFVIFDTSEFEDYVMKITEDVLLKDLLTVASLCLTHDKEKDTLLQTIYRVRYR
NCSGSKLLKFFGRSYRVYTAVYGFAHAEHDMRCLSGKYCSKSIHKNINRKQLHHFLKKTH
FKDPWGQNVSFTELGFLDLSYSISSFYSFIKYVMHRNYVGTYTWSEPKSFLQIDIQQIIWK
KNTSNQTLKSQCSSKCLPGYRKVPQKGAPPCCYDCTSCSEGEISNLTDMENCVTCLDYEW
PDNGKTRCIEKPTDFLSYDRDSLTLVFNVITLIFFVIALSILGTFILYRDTPVVKANNRSLSFIL
LVSIKLSFLSVFLFLGRPVDITCMLRQTSFGITFSIAISCVLAKTIMVSVAFKATKPGSSWRKL
VGARLANTIVLVSSIIQVVISVIWLAISPPFAEHNIHSEPGKIIIQCNEGSVVAFYIVLSYMGLL
ASVSFIVAFLARSLPDSFNEAKYITFSMLLFCSVWITMIPAYLSTKGKYTVAVEIFAIISSSCG
LLFCIFFPKCYIILLKPEMNTKLNLLGNK
>jgi|Xentr4|121376|gw1.913.67.1
MTSESKENVSGFFIQGFSDTPELHISLFVLFLGIYLIILLGNLIIFLVISCNPHLHTPMYIFLLN

LSLIDISFSSNILPNLLHILLTQQNNISFLGCMAQIFVFISMIGSEYFLLTAMAYDRYYLVILRS
GHLPCWFPVLCFKTCRKKWFSCLARLAHCCLLDLNMTSESKENVSGFIIQGFSDTPELHIS
LFVLFLGIYLIILLGNLIIFSVISCNPHLHTPMYIFLLNLSLIDISFPLTVLPNLLHILLTQQNNIS
FLGCMTQMFVFVALASSEYFLLTAMAYDRYVAICDPLHYIARMSRKHCAGLI
>jgi|Xentr4|121896|gw1.1169.29.1
ILVTVISCLILVTVLGNTLVILAFIEDRRLRNRSNFFLLNLAICDFFIGAFCIPFYVPYVFTGK
WLLGKFLCKLWLIVDNLMCTASAFNVVLISYDRFLSVTMAVLHRSIEKEHGRTVLNMALV
WILASLLYSPAIIFWEYVDGDKSIPTDLCLPGYFYAWYFLLGASTFDFLLPLLSISFFNLSLY
CNIRSRSRKKQHIVTSPIDSNLDGNPARPYIISGNDFHNGAVKDLNTPQYKKGKRSLCCFFS
NVLPLHRDQQTVRYNVHVIKLSRDKKIAKSLSVLVCVFGMCWAPYSLLMTIRAACHDNCI
DSYWYDITFWLLWINSSVNPFLYPLCHESFRKAFAKV
>jgi|Xentr4|131535|gw1.285.95.1
NPEDCNMTMEELLFKYLGPRRSTYFTPICIIYLFIFAVGAVGNTLTCIVIIKHKIMRTPTNYYL
LSLAISDLLVLLLGMPLELYELWSNYPFVFGQSGCSFKTLLFETVCFASILNVTALSVERYIA
VVHPLRAKYVVTRNHAKRVIFSVWVISILCSIPNTSLNGILHLRVLGIGIVPGSATCTVVRPR
WIYNLIIQITTIFFFFLPMGTISVLYLLIGLQLKKEKMLQVLETKSGKEGDGYQNVRLQQEK
IRRRQVTKMLFILVVVFGICWAPFHTERLMWSFINDWTEVMHQIFSFVHVISGVLFYLSSV
VNPILYNLLSTRFREMFKEVMCHGRTKRLGSRRSSPSVTRVTTRSTVCE
>jgi|Xentr4|133575|gw1.173.159.1
TERDYLGGHTVWQVFLIASFSGIIALVTVIGNILVILAFKVNKQLKTVNNYFLLSLACADLII
GVISMNLFTTYIIMDHWIMGSLACDLWLMIDYVASNASVMNLLVISFDRYFSITRPLTYRT
KRTTKRAGIMIGLAWIISFILWAPAILFWQYFVGKRTVPSDECFIQFFTEPIITFGTAIAAFYLP
VSIMTILYWRIYKETEKRTKELAGLQASGGKLEVPGLSHRTGNAKGNSNYKIQRNIKFSSR
TRYSWCTFRFKPKMCKSDKKQIEQDQSSSESWNHNDANASLDNSGTSDEEDAPENPRAIY
SIVLKLPGHNSVLSKTKSQSSNQGNTSKGMQKSGPQPKELRFKNLNSQRNLQNKDESISV
SLSPQTDHSMNTSSKTTAPLPISFKEIALAKRFAAKTRNQITKRKRMSLIKEKKAAQTLSAI
LLAFIITWAPYNIMVLVNTFCESCIPRIYWNLGYWLCYINSTINPICYALCNKTFRTTFKILLL
CQCDKIKRRKKQYQ
>jgi|Xentr4|134002|gw1.167.235.1
LCSFFPITTLIPVTIVLLIVMVVGVIGNTITILIIRRYKEMRTTTNFYLSSMAMSDLIILLSLPF
DLYRLWKSMPWIFGGFLCRFLHFISEGCTYSTILHITALSIERYLAICFPLKSKVLITKTRVKC
VIVFLWVFALLSAGPFYFVVGIAQMYNITNDSDTGWECRYTFYAVHSGLLNVMMWVTTT
YFFIPMFCLIILYGFIGKKLWKSQNSIRGRNTAQRDKSHRQTMRILAVVVLAFIICWLPFHIG
RIVFANTEGYEMMKFSQYFNVVAIQLFYLSASINPILYNLISKKYRSAAYKLLRPSTAKKKT
YNTIKNDTGDKTESS
>jgi|Xentr4|135265|gw1.325.171.1
VTLLVGLVVNSLYLWVLRFRMRRSINTTWFFHFILANVIFTFTMPFLATYMAMHPVWIFGS
FLCKLLNTLYSVCMYASVFSLTVISLDRYCLVFHPVWYRGHRNNRYASAICICIWGSAILFS
SPYFAFRQTRLLEDNRTTICYNDYTLSGSIWPKSATQLRWVMFSFHVILGFLLPFAVMTVC
YVRIALRMKKGNLARSTKPYKIMFISVASFFVSWFPYHLWYGMKIEEGRFHKSTLEILMVL
TIGLTCINYCFTPLLYLFIAENFKKSLRKSVLLLIESVFNETFNSQLNRSFEDKSDAPP
>jgi|Xentr4|135297|gw1.325.177.1
VALLVGLVVNSLYLWVLRFRMRRSINTTWFLHLILANVFFTFIMPFIAVHLLMFPHWAFGT
LLCKLISSLISVCMYTSVFSLTVISLDRYCLVFHPVWYRGHRNNRYASAICICIWGSAILFCT

PYFAFRQTRLLGDNKTTICYNDYTLYGSVLQKSATQLRWVMFSFRLILGFLLPFAVMTVCY
VRIALRMKKGNLARSTKPYKIIFISVASFFVSWFPYHLWYGMKIEEGRFHKSTLDILMVLTI
GLTCINYCFTPLLYLFIAENFKKSLRKSVLLLIESVFNETFNSLNRS
>jgi|Xentr4|135513|gw1.262.119.1
SSVFYAVLHLILSILIPVANIVVIVILSKLIKKKCCKSYVFILNLAAADLLVGLMCILEALDDL
LDGDFDSNLFFCLLRLCFTVTPCIASMLTLLLISLDRYLAVKLPLYYANIMSTKSICALITLI
WVVSFFVGYMPLISPSLQQNDYKGICGLFYAAKNEYLYILCFIVFLPALLTMICLHTAVGRI
AYLHHVRIQRSQVVGGLPSNPAAHSSHFKAARTVLIVIICFIISWGPYYITGIIQATCQSCKL
VDLMKDILFALGELNSLLNPIIYTFYCTEIRTYLY
>jgi|Xentr4|135612|gw1.417.89.1
GLIGNAIVIWVLTFKMKRNKYTVYILNLAIADFIYLFFDAVVMLLLVDKMLNARNPSSKTL
QALEIIYDFGYTAGMLFLTAISIERCLSVLFPIWHKCYRPKHLSTWACGFLWLFGALLSLLD
NFVCPANDFNKNTYQCTAMQIFASVLTFAIIVPLMVMSSFTLIHVVRTTSKKCRPPKIYVAII
ITVLVFLISVIPIKVLWILLYFKLFPNNFHSVALFFASTYCTMFNSSANPFIYFFVGRQKMKRF
SSSVNEALSRVFKED
>jgi|Xentr4|135703|gw1.498.104.1
DRTIMMWESTTKLVAQSVFLLFGLAGNMFILVMSFTDWRKTHSWNPYAVITISIGISNILLQ
TTTFLNEFFVLLLTNVSVQETMIKYFIATQISLFINSLLFSLCLCFYYCVKILQMNQPFFRKV
KQEIARITPWFLSVSMLVSLGIGIPSYWDLHWTLTGATNSSASWIQVNVKLSRRYQWVFIL
QMLISSGALIVFFCLAVTIIFSLCRHMRRMKLNSTGFNKSSLDVHVSAAKTLTLILLLHIYFF
AAVCILFNSPLTWGTWFFNLSYIMVGSFPSLDSFILITGN
>jgi|Xentr4|143185|gw1.58.129.1
NRQLRLITMSVIFAVALVGNVVVLYKICSGKDKKRKINFLITQLALADLYVSVMSLLSQIV
WELLEDEWLVGDLGCRIFKVFQVSGLMASSNIIAIVALERHHVIMNPLSTPLPTRSMAAFG
WMFAFLLSVPQAFVFKAAYTGQGNRCLNIFGQLPKWHLQVYIIYCSLIVFILPFCILCVAYT
RILWVIWKRGKACKTSRHHNGMCKVQENHTEFKRRSLRLTATNSCIPRAKLKTLKMTLVII
ILFIVCGLPYFIVELKVAFATITGLDEKVMAVLGIFVVTNSAVNPYVYLFFKTNNMFLRRLE
KKVCFSCC
>jgi|Xentr4|143526|gw1.153.241.1
STSKLILYLLTSGLIMTTFLGNILVISSIVYFKQLRSPTNSFVLSLAVADFLVGVMVMPYSMV
RSIEGCWYFGSGFCRLHSSLDVMLCSASILHLSCIAFDRYYAVCNPLLYGYKMSTRRVSILI
CTCWFIPVLISFAPIMLGLHLLGMEHLWQEGACLFVVNQIYSVCASLIAFYCPMIIMLVAYC
RIYRAARNQALRIHAMERNVSSGNASDGPMKKRKYSLKRERKAAKTLGVIMGLFLLFWT
PFFTANIVDPLIGYKMGTVEWEVCLWLGYVNSALNPFLYGLFHKSYRRAFFMIVGC
>jgi|Xentr4|143546|gw1.48.339.1
VVLVGSILICAIGLIGNALVIYVTGFVMKSHKCKIWFVNLAVADLIYLLCMPLQIADDFTGN
WIFGLALCKMHNFLSTCNNYASVFIITALNIERVLSVAKPIWHLRFFSRRICFWICSIIWSVT
VIFSFPIIFFSSISTDDDGNRICDCDCCLDAEMIAMWSNMIFLAKTVMIPMLLFGCIIPLCIIV
LSNVTIAIKVSKSQTLKPPRLYKIVITVVLVYFLTWIPNVIGWNLAINAIFTMDYSLLAKISL
YLPLLGMISDISSCLNPLIYVLVGQ
>jgi|Xentr4|143685|gw1.31.354.1
MSNFTPDACNVDSGLDSVLPPSLYALVFTLGLPANLLALWAAWLQVRKGRELGVYLLNLS
LSDLLLICALPPWTDYYLRRDVWGYGPGACRLFGFVFYTNLYVGAAFLSCVSADRYLAV
AHPLRFPGARPIRSAAAVSALIWMLELAANAPPLLGEAIHRDRYNHTFCYESYPLSGRGAA

LANVGRVLAGFLLPWGVMMLCYAGLLRALRGSASCEQRERRRVRRLALGLPCVALLCYG
PYHALLLLRSLVFLVGGGSVDAGGGCALEERLFPAYHASLALATLNCLADPALYCLACPG
ARGEVAKVV
>jgi|Xentr4|143736|gw1.61.208.1
IGIPANLIILIIFMQIRFSEKKLLPSNSILAVLALVNLMVIFSKGVPQALHSVGVRNILNDTEC
KLISFTYRICRGMTICVTCLLSCNQWIILAPPSKTWLYLKQGVAKSLPWIMLLLWFINIAIYP
SCLIHVRAIANYTTSIYTLHLEFCNHDFMTFESYVVNGVAIAFRDFFFVGTMTLTSCCIVFL
LYRHGKRVQGIRSSDKNLSKTMEYKAARSVVLLVTIYVALFGLDSSIWIYTLTISRVSPVISD
TRVFFGTLYAALSPIIIIKTNKKIKTVLTCRIKTNLL
>jgi|Xentr4|143743|gw1.55.343.1
PSSSGLWLFLIPSAICSVFTLLANPLVLVAILKKEKLRKETRYLLLANIMVSDLIFLLFNTVIA
TCNAIRWYLHKIICFTMIAFSFAAYCSCVLTFTVMVIDTYIAICFPLRYYSLLSLPRTRKVLL
AIWIFSILYPLCIFLATEAFDMHPLEKQNICLMLYYGPNVKRNNLVTVVCAFALFFLLICTV
MITYLYIRLYKMTKESGIWERRFSRARVTLLTHSILLGLYIIPAFLLAAEILVFRDNEFGMTA
RLWLSAANNAVIMMMPRALAPVLYGLRYREI
>jgi|Xentr4|143786|gw1.91.354.1
PVFVLASVVILGATTIMGIVTNSLIVVVNVADKIKGKSFNPSDLILVTLGMSNITFQFTMTV
NDFSIILWSDLYFSDAVYGTFKALLYSTIFASFWFTVCLSVYYCLQIVIFTHPFLVRLKLGMS
RLVPFFLGASVFTSLVISIPALWSIYKDPQNGNFSSNQSLKIELPKLSAVYLFSSNIIGCSLPL
MLVGISNSLILKSLISKSTMLEKNKSDVYSPRTEARERAARTVGCLLLLYMAFYIFQILMFI
NFFPPSSPGFCTCLMAIYVYSPLQSIILIFGSPKLKK
>jgi|Xentr4|148829|fgenesh1_kg.C_scaff...
MAGGIMATLHPSLLTTLPLPKNLTNNTEDSCVFNEGFKFLLLPISYSGVFMVGLPLNITAIW
IFIAKMRPWNPTTVYMFNLALSDTLYVLSLPTLVYYYADQNNWPFGVALCKIVRFLFYAN
LYSSILFLTCISVHRYRGVCHPITTLRCMNAKHAHVICALVWLSVMLCLVPNLMFVTVSPK
VNGTICHDTTLPEEFDKYVEYSTGIMCLLFGIPCLIIACCYGLMARELMKPLVNGNHQTLP
SYKKRSIKTIVIVMIAFAICFMPFHITRTLYYYARLLGVNCYALNVINFTYKITRPLASANSCI
DPILYFLANDRYRRRLIRTVRRSSVHHRRCMHTNHPGPHPEPEPHMTTGPLPVVSAEETQS
NGRMVRDENEEGAREHRVGWTASKDSKQRKSVYHQSTIKRNSMDKNNMKEHRHGENY
LPHVEVVEKEDYVTEGENRKTTGQGDIAESRKTNDEQDEIQTQISSSLKKGKWRLSSKKG
ATQEYEEGHVESSWEAEGTSTWNLLTPKMYSKKDRLAKNVEGESYWKERELQNIPKI*
>jgi|Xentr4|149432|fgenesh1_kg.C_scaff...
MGIPASLVVYIVFEAVIALISVLGNILVIWAVIVNQALRDTTFFFIVSLAVADIAVGALVIPLAII
ISIGLETEFYSCLMVVCIVLILTQSSILALLAIAVDRYLRVRIPTSYRSVVTARRAGIAITGCW
VLSFLVGLVPMFGWNNVNNLKNEQNSTESGLIITCQFETVISMEYMVYFNFFVWVLPPLF
LMLIIYLEVFYLIQKQLNKKVCANSKDPHKYYGKELKIAKSLALILLLFALSWLPLHTLNCI
TLFCKTCKTPMIITYVAIFLTHGNSAMNPIVYAFRIEKFQSTFLYIWKKYFCCKPSRFINGRH
NNIGESSKNVL*
>jgi|Xentr4|150837|fgenesh1_kg.C_scaff...
MAITLRLFWLLGFLASQVGAMHPYCIIKKEEEACLEKIQRYEIEMWNDTQPGCPGMWDNI
TCWMPAEVGKVVSIRCPALFSMLGSEDEMDFVDRSLGWAPENLEEQQSAGIITRNCTENG
WTDHFPHYSEACDFDINETGPDQDTYYLSVKALYTVGYSTSLVALTTAMVILCRFRKLHC
TRNFIHMNLFVSFILRAISVFIKDEVLYAEQDSNHCHLSTVECKVVMVFFHYCVMSNYFW
LFIEGLYLFTLLVETFFPERRYFYWYTIIGWGTPLICVTIWAVLRLHFDDVGCWDMNDNFA

LWWVIKGPVIGSIMINFVLFVGIIIILVQKLQSPDIGGNESSIYLRLARSTLLLIPLFGIHYTVF
AFSPENVSKRERLVFELGLGSFQGFVVAILYCFLNGEVQSEIKRKWRSWKVNRYFAVDFKH
RHPSLASSGVNGGTQLSILSKSSSQIRMSSINAENLGT*
>jgi|Xentr4|152568|fgenesh1_kg.C_scaff...
MEINSHNKMSIDLTAALTVTRFQSWSPTIISEVFNNSNGSSLPPKQCVRDTRISQVVFPVLYA
LVFLLGLLLNGFSIWIFCKVPSHTVFIVYLKNTLAADFLMICMLPFKILTDSGIGSLQMKAF
VCRFSSVVFYVSMYINIILLGLIGLNRVLKIARPFGKKWVDNVFIARALSIAAWLLMFGISIP
NMVLSNEKATELTVKKCASLKSKLGIKWHEAVNHFCQFIFWTTFTLMVIFYTIISKKVYES
YTNSRSRDSSTTKKTKAKVFIIVAVFFLCFAPFHFARVPYTISQTGGIKDCNVQNKLYIAKET
TLWLAATNVCMDPLIYILLCKPFRQLLTKGSRSATTSMEAQTCQDSRM*
>jgi|Xentr4|152569|fgenesh1_kg.C_scaff...
MFNLTNSSRTNRTCSLDIHANPVIFSVLYTILFIVGVILNGLAVQVFFQIPSNSNFVIFLKNSVI
SDCLMIMTFPFKILSDSRLGLWGLKGFVCQVTSVIFYFTMYTSIIFLGLITIDRYLKTVRPFH
NSATNLRPVKIMSTLIWIVMISISLPNMILTNKTQTPQNVKKCYLLKSDFGLVWHEIVNFIC
QFIFWVVFVIIVVCYLLITRKLLQSYKKTRSERPKSRRKVNVKVFIIIAVFFVCFVPFHFARIP
YTLSQTRDVFKCSAKITLFYVKESTLWLSSLNACLDPLIYFFLCQSFRNSLVAMWRKRQAC
VSSYTRPRNEKRLKLRREETNI*
>jgi|Xentr4|154060|fgenesh1_pg.C_scaff...
MFADLNQTNCSSCTCSAGNRIFRVSFMIALILAILLGNSVILAVFLGTKQLRTPQGYLKASL
AVADLALGILVVPFSVYGEMTLLFTNSSSPHGATQVLAVGSWHPCYLVGPVFAGCTLVSIST
IFLLTLERSIAILKPLHKESVITRKRTLLLILLSWVSSFFLAMSPIIFSKGGIVVEYNHCSRMC
NYALAPASPSHVWQILLLFPAFDFSLLGGTLIINALSLTTIHQYTRRRKLLSGADQENLSFSD
IKAAKTIGTLTIAFTASFTPIAVFVVGDVLGYKWCTFSFFAFWILASNSCCNVLIYSVCDQRF
REGARQLYVTLRSWCCCRSSR*
>jgi|Xentr4|154089|fgenesh1_pg.C_scaff...
MAARLGKVLLVTLLLPLVQSHSLFTCEPITVPRCTGMNYNMTFFPNLLEHYDQDIAALRM
EPFLPLLNLHCSPEVHTFLCRAFVPECTEPKHITMPCRSLCERVYSDCKNLIETFGITWPAEL
ECDRMRDCNNSQSKGGGGPDDPHDALRHPEQVHRDFKFWCPQHLKTSQGSGFKFLGVD
RCAPPCPNMYFKKVDMDFAKYFIGIISIICLCVTLFTVLTFLIDVQRFRYPERPIIFYSVCYSM
VSLMYVIGFLMGNSIVCNEREENFASGDTVVLGSQHKACTIIFMLLYFCTMAGTIWWVML
TITWFLAAGRKWSCEAIEQKSLWFHGVAWGIPGILTIILLAMNKVEGDNISGVCFVGLYDQ
EVSIYFVLLPLCLCVFIGLSLLLAGIISLNNVRQVIQHDERNQQKLKKFMIRIGVFSGTYLVP
LVILLGCYVVYEQVYRSSWEMTWVSDNCQDYHIPCPFQLKNPARPDLGLFITKYIMTLIVGI
SAIFWVGSKKTCSEWVNFFNRSRKQDPISESRRVLQESCEFFLKHNAKVKHKKKKHSKSG
SHKLKVISKSMGTSTSGKINHGMTAVTNTSHDFLGQEASGDVRSTLDFSRTEDPEGASCIH
KSRDDITGEVTTQTLLDGTEAPVEHTDCNNKPGSKRGNGLPGSIHTILHESLHEVAENRVN
VESASLDPDQTEDLSKVPGHTSPHDNDTNMNTTSA*
>jgi|Xentr4|156805|fgenesh1_pg.C_scaff...
MSSFIIPLIYLLVCVIGLSGNTLVIYVVLRYAKMKTVTNIYILNLAVADVLFMLGLPFLATQN
AISYWPFGTFLCRLVMTVDGVNQFTSIFCLTVMSIDRYLAVVHPIKSTKWRRPRVAKLISAT
VWTLSFLVTLPVIIFSEVQPDYHTCNISWPDPVSVWAAAFIIYTSVLGFFGPLSVICLCYLLI
VIKVKSSGLRVGSTRRRRSERKVTRMVVIIVAVFVFCWLPFYILNIVNLSFFVPEEPAFAGV
YFFVVVLSYANSCANPILYGFLSDNFKQSFQKVLCLRKSNGIKDADLTENRQEKSSRLQET
MLPSRNSEFNGHMQTSKV*

>jgi|Xentr4|156919|fgenesh1_pg.C_scaff...
MQVNRKWEIPRSNHSTIQEFLLVGLKDLHHFQIPVFLLVIIVYVATILGNMLIIVLVLTNHHF
KSPMYFFLSHLSLCDILISTNVTPNTLQVILNKNSPISVNCCLTQLYFFGASAVIECCLLTVM
SYDRYLAICNPLLYSSIMSHNLPYYLVLWPWLVGFLLALITNVLVLELYFCGPNIINHFFCD
LAPILELSCSDTSAVEIQVSIVAAVIGVSQMLFVVATYICIFNSILSISSNIGKEKAFSTCSSHLV
VVSTYYGTLIALYLAPSRGYSLTLNKILSILNTVVTPLFNPIIYSLRNKEIRTETVKFVSKIKK
*
>jgi|Xentr4|157818|fgenesh1_pg.C_scaff...
MLGGKKRCQDVIPLHSTSRSTRNFKVADRNLPFGHPCSKVYQVTVSNKGQNKGQFLTFM
ALTGEYLPKVTFYLVLMNISVSDGSINEDLALELTKNLIYHILARSDFAIVQIMVNEQVCCS
NEPQKEQRAKRKVTISHCNVNFTKGKYRFESKLAIQATSSDTPDTLTKNLTNLFENFHYTD
TQLQKNLSVISVRVQHLGQCPQEITNSSEKGTYTWMEKDPTETATVQCQKNPEEFATRYC
NIDITTERPKWESPYLERCLPVQTFPDTIKELQNVVVVTLENAPILAKHVLNLIKDFDILTKD
ETEIILTKITEIVGVGGVDLPRAQTILGILNNIIIKGEELWPFTNRILNLTEEIGYKMDYSGMA
ANVSEENMALSVSNVEFHTFEEIYFTVKTYEEGKYPEITLQKVPADKAVAYIYLPQEIKNH
VNVATSKVQFNFFGKTSLFKDDSFSSVSSPVLNTYVVSASIKNTKIQGLNEPVLFTLKHIKE
NKDMSLSSVHCVFWDFNANDRMGGWNSSGCIAKYTNINYTTCYCTHLTHFGILLDLARK
DINPWDERIMSLLTYVGCGIASLFLGISLVTYLAFKSLRKDYPSKILMNLSASLLMLNLIFLI
NNWLSSFRNHGLCITCAALLHYFLLASFTWMGLEAVHMYFAFVKVFNLYIRNYILKFCLV
GWGVPAIVVAIVLSIKRDFYGNGTKHKQYAVMKHDEDLFCWIQDDVVFYVTVVAYFCFIF
LINIAMFIVVLVQINSLKSKKMKDWKAAFVHDLKSTISLAFLLGLTWGFAFFAWGPVRIAF
LYLFSIFNTLQDWSRLSNGELKRNSRISKLQSDSSQSTLSTATASTSNASSLSGFKDGNSFRN
GGIFINDPNLDSRNQATITYDARRILSFLDLNTQ*
>jgi|Xentr4|157859|fgenesh1_pg.C_scaff...
MNSTSSPHVGGISGPSVTITNIPYFVTELLTAVLSVTGNILICLAVIRDRRLRTVTNYFLLSLA
TADILVGAVAVPCAILLDMGVARCSLYCCLFMLCNLMTFSLASIFGLLAVAVERYICIMRPFH
YRAFVTPRTSFLVILGTWFLAAITGLMPLMGWRKSFPPNSECLFNSLISESYMVYLIFFGCV
IPPLAAMLVLYARIFLEIRKQIRQIAEWEVEMSRRRRRRRIVVKELRMATSLFIVVFCFVICW
LPIHLLNVVRLFFPSCVVPEDIILSAVILSHVNSALNPIIYVFRMSSFRRAVEAILSCSCATKAV
AIPVKGFYSA*
>jgi|Xentr4|158120|fgenesh1_pg.C_scaff...
MHETYQNNTLPTYFSLLSFPSFAEAEILLFIGVLLMYLLAVLGNLVIIALICLVPHLHTPMYF
FLCTLAFQDIVHISAIQPKLMAVTITGDHSISFPGCISQIFLFVFCLDVESFILTTMAYDRYVAI
CVPLRYSVIMNKSVCTLLTIVVWILASLNALMYSLLISNLSFCKSHELNHFFCELKAMLYLS
CSDITTNLIILFVEDLIIGVFLFILILTSYIFILSVVLKIPSSAGRIKAFSSCSSHLIVVTLLYGICL
GFYMKPESEHSQEQDKVLSMVYVLMIPLLNPLVYSLRNKEVWKAFKKLTGIK*
>jgi|Xentr4|158124|fgenesh1_pg.C_scaff...
MTINTKNGTLPSTFYILAFSVSSEKRLLFFIGFLALYLIAITGNLIIIYLVYLKPQLHTSMYFFL
CNLSILDILYISSTLPKLLYIIYTGDHSVSYNACLTQLYFFDLFADTEPFLLTSMAIDRYVAVC
KPLHYPLIMSKKTCALLVVTAWFMAAVNALILNWLVLGSAFSYFNKINNFFCDLKALISIS
GHDKTYLRRWFVLVDGLCIGVVPFGLTIATYVSIISTILKIQGSEGRVKAFSNCSSHLCLVIL
YYGSALLLYMRNSEQGDMLFSLMFVILVPFFNPLIYSLRNKDILRSFKRVFYCNKTTS*
>jgi|Xentr4|159303|fgenesh1_pg.C_scaff...
MAALLRGASLPCHLLPLLLILLIPLFTSSRGKHAGTWDTAGPPPAHPLGTDRHSLAHNRSSI

QGLVWEIYWATMAQCEAKRNGSQEGLLGGPWNLTLCLQGNSTGPNATGTLDGFRGCSFA
KCLLRTAGRKQPSGGWRISLHCPGKPGDAPSVCLCLPNSMFVPPSCIFRTKGKTEDLNGQN
PNARGADLRKETRDSLQGLQSSKAMQPDPFHPPLRTHPQGVKHRSSGRDRERASLPLIRS
RRAANRHPHFPQYNYQVQVAENQPPGTPVITMSAQDPDTGEAGRLRYTMDALMNSKSLE
LFSIDSVTGLISTIEILDRESMDLHYFRVSAIDHGTPRLSATTMVAITVSDRNDHSPLFEQSE
YRESIRENVEEGYPILQLRATDMDSQANANIKYRFVNEQAAHSVFEIDARSGLITTSGQVD
REKREKYSLIVEASDQGKDPGPRSSTVKVDITILDENDNVPQFSEKRYIVQVREDIKTHTEI
VRVTATDIDKDSNALVHYNIISGNSRGQFSIDSVTGEVQIVTPLDFETEREYTLRVRAQDAG
RPPLSNNTGTISIQVVDVNDHAPIFVSTPFQVSVLENAPLGHSIIHIQAIDADYGENSRVEYK
LTGMKPESPFVINSATGWITVSGPLDREMVEQYVFGVEARDHGNPSLSASAGVTITIMDV
NDNRPEFTQREYFIRLNEDATVGTSVLSVTAIDRDVNSAITYQITGGNTRNRYAISTQGGSG
LITLSLPLDYKQERRYVLTVTASDRILHDNCYVHINITDANTHRPVFQSAHYPVDINEDRPI
GSTVVIISASDEDVGENARITYSLEDNIPQFRIDPDTGAITLRAMLDYEDQVTYTLAVIAKD
NGIPQKSDTTYVEIMVNDVNDNAPQFANTQYHGIVSEDAPPFTSVVQISATDRDSHSNGRV
QYTFQNGDDGDGDFTIEPTSGIIRTVRKLDRESVPFYELTAYAVDRGMPPLRSPVRIQVAVQ
DVNDNAPVFPADEFEVYVKENSIVGSVVGKIKATDPDEGPNAQIMYQIVEGNIPEIFQMDF
FSGELTALIDLDYEAKPEYVIVVQATSAPLVSRATVRIKLIDQNDNSPVLKNFQILFNNYISN
SSNTFPSGVIGKIPAYDPDVSDRLLYTFERGNELSLLIVNHSSGELRLSRKLDNNRPLVASML
VTVTDGIHSVTAQCVLRVLIITEDMLSNSITVRLENMSQERFLSPLLTSFLEGVSTVLATPKE
DIFIFNIQNDTDVAGSVLNVSFSALAPHGDRSQFLSSEDLQEQLYMKRMTLTSASMLEVLP
FDDNVCLREPCENYMKCISVLKFDSSAPFIASESILFRPIHPITGLRCRCPQGFTGDYCEIEIN
LCYSNPCQNGGVCARREGGYTCMCRERFTGDNCEVDSGSGRCVPGVCRNGGTCVNLAE
GGFTCQCPSGGFEKPFCELSTRSFPPKSFVMFRGLRQRFHMSLSLSFATKERNGLLFYNGR
LNEKHDFIAVEINDGQVQLKYSTGESSTLVTPYVAGGVSDGRWHTVLLRYYNKPKTGSM
GVAQGPSREKIAVLAVDDCDISVALKFGSEIGNYSCAAEGVQSSSKKWDPYALCSSIGCHG
KHTFCDSNPCKNGGTCSVTWGSFNCNCPLGFGGKDCRIALQYAYQFNGSSLLSWDFKND
VKISVPWFLGLAFRTRRADGVLLQAHAGQYTTILCQLVSGLISFSVSRGSSRTSALLLDQM
LVNDGKWHDLQMELRDVRSGRETRYVISINLDYGFYQDTVTVGSELHGLRVKNLYIGGV
SGPREVQNGFEGCIQGVRLGETPSGITLPKPSSALNVKPGCGVPNMCDSNPCPGNSSCVNE
WMSYSCVCHPGYYGENCIDVCQLNPCENRGVCRHQPSAPMGYTCECSKNHIGRYCEHRL
DQQCPKGWWGNATCGPCHCDVNKGFDPDCNKTNGQCNCKEFHYRPKGSETCLPCDCYP
IGSSSRACDQETGQCYCRPGVIGRECNNCDSPFAQVSPSGCEVIYDGCPKTLTAGVWWPRT
KFGLPAAVPCPKGSLGAAIRHCDDVKGWLEPDLFNCTSPAFVELSPMLDGLERNETELNTI
EAKKLALRLRSVTDQMDQYFGNDVHITYRLLSYLMQYENKQEGFGLTATQDAQFTENLL
KTGSSILTLGNREHWEALLHNEHGSAGLMNLLWEYSSTLAKNMKLTYLNPVGLVTPNIM
LNIERVENQNSARRQFPRYHSHLFRGQTFWDPHTHVVLPESVLVPPKPKAQPTTVPTPTTA
DMNVTAKVPVPKRIAQEPAPTIIILIIYQTLGELLPARYNTEKRSLRLPKNPVMNSPIVSVSV
LRNQSLILGALDTPVVLEFRLLETVNRSKPVCVQWNQSEQVDPLGSWTVKDCELVYRNT
THVRCHCPQFGTYGVLMDGSHREQLEGDLETLAIVSYTSLSISLAALVATFSILTFLKGLKS
NTRGIHSNIAVALFLSELVFVLGINRTESEFLCTVIAILLHYFFLSTFAWLFVEGLHIYRMQTE
VRNVNFGPMRFYFAIGWGVPAIITGLAVGLDPEGFGNPDFCWISSHDKLVWSFAGPIAIVIV
LNGVMFLLVAKMLCSPGQKETKKNSVLMTIRSSFILLLFISITWLFGLLAVNNSVLAFHYLY
VILCCLQGLAVLVLFCMLNEEVQEAWKVVCLGKKGPGEEISRQAQGPNAYNNTALFEESG
LIRITLGASTVSSVSSVRSARTHSSHRPYLRDNKAPRHGSAAAQSVRSQHSGPTDLDVAMF

HRDAGGGQDSDSDSDLSLDEERSLSIPSSESEENFRPRGRFQRQFKKAAHSERLLTHPTNH
HPKDVDGNDLMSYWPALGDSEVQPNSLQKWGSERKLGLDLNKDAANNNQPDLTSGDEN
SLTHSHRQRKGILKNRLQYPPGLASVGRMTNELSWYKTSTLGHRAVPAASYGRMYSGTG
SLSQPASRYSSREQLDMLMRKQMSREQLSRNNSRELLEAVPSRHGSREQLDTIPSRHGSRQ
HLDIISSRHGSREHLESIPSRHGSREHLDQEDIRNDSRDHVNSLPKRHCSREHLETLHSRYG
SKEQIDPGPHYAVSARQSKSHMMRLAESAGWIKKPNGPDVAHGP*
>jgi|Xentr4|159727|fgenesh1_pg.C_scaff...
MENQTLSIGMQSINATGMNETVGSMPQIALEVQVVTISLVLLICGVGIAGNIMVVLVVLRT
KHMMTPTNCYLVSLAIADLIVLLAAGLPNISEVVASWVYGYVGCLCITYLQYLGINISACS
ITAFTVERYIAICHSIKAQFICTVSRAKKIIAFVWFFTSMYCVMWFFLVDITEVKFADGVQV
NCGYRVSRNLYTPIYFLDFTIFYVIPLVLATVLYGLIARILFMNPLPSNPQDLSRMSSKHYGK
PYNSIKLSGKGNKNTASSRKQVTKMLAVVVILFALLWMPYRTLVVVNSFMDPPYLNVWF
VLFCRLCIYLNSAINPIIYNLMSQKFRAAFKNLCKCEQKRTEKAAKYNVPVYYSVMKDSS
HESPDHDVTVQEDLNGFPAKKVNFTQKCVDTTTTYSVA*
>jgi|Xentr4|159911|fgenesh1_pg.C_scaff...
MIFAINKINKQRDILPNITLGYQIFDTCFTISKAVEGTLSFLTGQNETHPNFRCSAGAPLAAV
VGAGGSALSIATARILGLYYFPQVGYASSCSVLSDKFQFPSFLRTIASDTTQSKGIAALVTYF
GWTWVGTIAADDDYGKYGIKLFKEEVEKNGVCISFSETLPRIYNKDSIDKIVATIRKSTANI
IVIFSSDIDLSSLMEALAETNITGKTYISSEAWTTSALIAKPKHFHFLGGTIGFAVQRASIPGF
EEFLHDIHPEHGNDDLVYEFWEEAFNCTWPTNRALFYTNISLEAAMAGRKRDVSPIPCTG
RENLNTSLNTYSDVSELRITYNVYKAVYTIAHALHDLDVCVPKHGPFPNNECANVLDFEP
WQLMYYLKKAKFTSLNNETISFDDSGDIKGHYDVLNWQWKDNQTMSFVKIGYYKDLTS
GYEFKIDNSSILWNTPNTMPPRSVCSESCIPGTRKGIRQGEPVCCFDCIACADGEITNETDA
RECISCPSDFWSNSHRNECVLKEVEFLATDEALGITLIFLSVFGASLVIAFTVVVYIMYRETAL
VKANSRSLSFLIQVSLVFTFMTSVLFVGKPEQWSCMARQITLALGFSLCLSSILGKTIVLML
RKKSAKSKLAERGHDALKPVYQIIVAVIGLTIQAGICTAYLLIYPPWVYKNMESQNVKIILE
CNEGSIEFLCSMFGFDVFLAILSFITAFIARKLPDNFNEAKFITFGMLVFFIVWISFVPAYLST
RGKFKVAVEIFAILASSFGLLGCIFVPKSYIILLKPERNTEEIVGGRAATNDKSAPATSASITSE
INSTTVSTVVLDD*
>jgi|Xentr4|160786|fgenesh1_pg.C_scaff...
MDPYLLLRAIGFLLLVIIGIPGNIFIMMRFTYLRIIEKKLLPTNIILMALALVNLLVILSRIIPQS
INALGVEELLDDTECKFVIFFYRVNRAMSICLTSFLSCYQCILIAPNTKLWNYFKHNVTQNI
VAIIFLFWIINIAIYPYSILNARTRRNQTTSPYTLHLVYCDADFLNYMAYIVNGAIYSCRDFIF
VGLMVLASSYIVYKLLSHEKSVKGIRSSDRTQQRSVEYRASRAVILLVALYVLLYGLDNCM
WIYTLTLSNVTPSMNEIRIFLASSYSSLSPIVIIITNPKLQQKLVSCNRERNRCSEINGQVYSIK
TF*
>jgi|Xentr4|161842|fgenesh1_pg.C_scaff...
MNDEQRLHNNAIAWLICSGISLLANTWGILSISAKQKKWKPLEFLICTLAGTHILNVAIPIT
MYSVVQLRRQHSDYEWNEGLCKVFVSTFYTLSLVTCFSVTSLSYHRMWMVRWPVNYRL
SNTKKQAVHTVMGIWMVSFILSTLPAVGWHDTTERFYARDCRFIVTEIGLGFGVCFLLLIS
GSVVMGVVCIGISLFQTFSIQTGQNIDKNKFNVPTIVVEDAQGKRRSSIDGSEPIKTSLQITY
LISGIVFIYDFLMGFPILVVSFASLKLDKSYEWMVLCVLWCSVVQAMLLPMFLWACDRYR
ADVKAVWDKCIAIMSNDDGDEEHSMDGGIHSDLIYERPYDYNYGGDIMAMDRISKYDLS
ALERGIPQIYPMKELQDDKMHYLQVPPTRRYSHDETDIWTTRQIPAFPHGWGSAEDVAAIS

FFTLPRRERRSSLVSYHEDPSMLYRKCRKSSESLISLKQFTLEELYKGDYKCFSREEVVSFID
ETPLPSPRKSPVRSSSISIIPDALQHQAMTHFALTDFEREPQALRRFSTHEKSCTLNVPGHRR
CHDDHHKRFYTEGHRSHRNSQKRTGLGKEWDGQEHLTKMESKGSANSFLSSPSASSGYIT
FHSDSIGSTS*
>jgi|Xentr4|162181|fgenesh1_pg.C_scaff...
MDGFSGGIDINIFDSNSTENGSGDFEDFSEPCFMHDNSDFNRIFLPTIYSFIFLLGIIGNGLVV
VVMGYQKKSRTMTDKYRLHLSVADLLFVFTLPFWSVDAAIGWYFKEFLCKAVHVIYTVN
LYSSVLILAFISLDRYLAIVHATNSQGSRKMLADKVVYAGVWLPALLLTVPDLVFARVSDE
NGQFVCDRIYPIDNRETWTVGFRFLHITVGLILPGLIILICYCVIISKLSHSKGHQKRKALKT
TVILILAFFACWLPYYVCLTTDTFMLLGLLKADCIWENTLHKAISITEALAFFHCCLNPILY
AFLGAKFKTSAQNAFTSVSRGSSLKILSKKRAGLSSVSTESESSSFHSS*
>jgi|Xentr4|163397|fgenesh1_pg.C_scaff...
MNSSESNILVANHTDVTFFILLGFPGGQEMQVVYFSLFLVIYILTVVSNMLIITMVWMDSRL
HIPMYFFLSNFAILDTWCSTAVVPKTMHYLITQDKVISHAECLAQLCFVHSVGPTEFFLLTV
MAYDRYLAICQPLRYATMMSNKNCLHLAIGSWMSGFFTGWTLTIPTAMLKFCGPNLINHF
FCDYIPLVKLSCSNTSTSETVFFSFAWVVVLCSLGFTTVSYSYIIKTVLRIPSVLGRHKAFST
CAAHLTIVLIFYGTVIFMYVRPRASYFSDKDKVISLFYSVITPLLNPLIYSLRNQQFRQALK
KMQGKIYNSFSRFYTE*
>jgi|Xentr4|163399|fgenesh1_pg.C_scaff...
MEDGNETTVTEFFLRGFPTRLEIQILLFIVFLIAYVITVSENIMIITVIQLHSKLHKPMFFFLSN
MSFLEICYISVTLPNLLVSTLSKNKSISFSGCMAQLYFFISLMCTECVLLAVMAFDRYVAVC
HPLHYVTIISNKLCIQLAAASWIAGFTVSVIKVYFISRLSFCGPNIINHFFCDISPVLNLACED
MSLAEFVDFVLALVILLTPLFVTVASYLCIIFTILKIPTNTGRQKAFSTCASHLTVVTIFFSTT
VFMYARPKKAKSLDYFKLLSLLYAVFTPMMNPFIYCFRNKEIWGTLKKYFCYKITPP*
>jgi|Xentr4|163455|fgenesh1_pg.C_scaff...
MVHCENSTPVKEFVLLGIPLSYQYQIPLFLLLSLCYLITILGNATIIVVSLVNAQLHTPMYFF
LSNLALLDISFTSAIVPKVLFNLLSGSQTISFNGCLVQSYIYFLLGTTEFLLLAVMSFDRYIAI
CHPLRYRNIMSPKLSFQLIICSWVGGFLDTIVQTIMTFRLPFYGSNVLNHYFCDVAPLLKLA
CANTHLIDMLDFILASSLVLGSLFLSLVSYGCIISAIVKISSAADRKKTFSTCASHLTVVFIVY
GSCIFMCIRPYKNSKVDSTKIVSLLNCILTPLLNPFIYSFRNNAFKDALRRTIRQRGIFTKV*
>jgi|Xentr4|163482|fgenesh1_pg.C_scaff...
MNSTYQEYLNPRKVWEHYVYVKENLTREDSSRYAISIIFIIICCIIILENLLVLTSVLRNKKFH
SAMFFFIGNLAFSDFLTGCAYIANILLSGNMTFTLTPMEWFIREGTAFTTLCASVLSLLAIAI
ERKVAIMQVEVYSSDRNCRMVLLIAACWVVSIVIGGLPILGWNCIFNMEQCSTVLPLYSK
KYILFVVTIFTIILLTIVILYVQIYYIVKSSHGEVAAPPTLALLKTVTIVLGVFIICWLPAFIILLL
DVSCKVKSCKILYKADYFFGVATLNSALNPIIYTLRSKDMRKEFLRVLCCFNYFQKNRTPD
KCMLKLRSSSSLERCTQKHDLPTSPIMKDCTTFV*
>jgi|Xentr4|163535|fgenesh1_pg.C_scaff...
MRAGNMSAYEAPGPYDGPQWPHLAPRSTFLTVAAVMCMVVILAFFVNGLVIVVTLKYKK
LRSPLNYILVNLAIANLLVTIFGSSVSFSNNVVGYFFMGKTMCEFEGFMVSLTGIVGLWSL
AILAFERYLVICKPMGDFRFQQKHAILGCSFTWVWSFIWTSPPLFGWCSYVPEGLRTSCGP
NWYTGGTNNNSYIMALFLTCFIMPLSTIIFSYSNLLMALRAVAAQQKDSETTQRAEKEVTR
MVIAMVLAFLICWLPYASFAVVVAVNKDVVIEPTVASLPSYFSKTATVVYNPIIYVFMNKQFR
NCLMTLLCCGRSFGDDETSSASGRTDVTSVSEAGGNKVTPA*

>jgi|Xentr4|164405|fgenesh1_pg.C_scaff...
MAAFPQRCSEQLCLRGSALIPWLLLLSASLAVQGQYNGEKGISIPDHGYCQPISIPLCTDIA
YNQTIMPNLLGHTNQEDAGLEVHQFYPLVKVQCSAELKFFLCSIYAPVCTVLEQALPPCRS
LCDRARQGCEALMNKFGFQWPESLRCEKFPINGAGELCVGQNTTESGTPTPAVPDTWTSN
SRTYYRDKFMCPRALKVPAYVNYHFLGEKECGAPCEAGKVHGLLYFAPEELHFARIWIGI
WSVLCCASTLFTVLTYLVDMKRFSYPERPIIFLSGCYTMVAIAYIAGFLLEDKVVCNDRFAE
DGYKTVAQGTKKEGCTFLFMMLYFFSMASSIWWVILSLTWFLAAGMKWGHEAIEANSQ
YFHLAAWAVPAIKTITILAVGQVDGDILSGVCFVGINNVDALRGFVLAPLFVYLFIGTSFLL
AGFVSLFRIRTIMKHDGTKTEKLEKLMVRIGIFSVLYTVPATIVIACYFYEQAFREQWEKSW
ISQSCKTYAIPCPSTSHPPMSPDFTVFMIKYLMTLIVGITSGFWIWSGKTLNSWRKFYTRLT
NSKQGETTV*
>jgi|Xentr4|164432|fgenesh1_pg.C_scaff...
MPTLYKPCFATEKEARLCEECHCLLLRDSFPETRGPSPSLRQPQTIISLTSNSTVGCVSDSPE
SSCTDIYTTLADTMTIEDVKFVTRIDRSCPVDYINQMPSWGMKMVSSDVRDKTVRKHVE
HCCFLVIVVIRMVPGFASTVDPTLMPIVNEEYISRQRIINSQFKCYEKMKKDSPYSKSGLYC
NRTWDGWLCWDDTPAGINVTQNCPDYFPDFDPTERASKYCDENGNWFQHPESNRTWSN
YTLCNSFTSEKLKMAYILYYMAIVGHALSIVSLMISLGIFFYFKSLSCQRITLHKNLFTSYVL
NSVFTIVHLTAVVPDTDLVRSDPVSCKVLQFFSQYMLGCNYFWMLCEGIYLHTLIVVAVFA
EEQHLHWYYLLGWGFPLVPASIHAFARTKYFNDNCWMSVETHLLYIVHGPIMAALLVNLF
FLLNIVLVLVTKLRDTHRAESNMYMKAVRATLILVPLLGIQFVIFPWRPDTRLAGEIYDYIM
NILMHYQGLLVATIFCFFNGEVQGALKRQWMQYKTQWGQRRREHCSMRSTSYTATSITE
VPIYLYHHDSNSEQLNGKYGDESEITALNSGDTYA*
>jgi|Xentr4|165196|fgenesh1_pg.C_scaff...
MKGAKLDHLLYRNWSEQDVNGTQEPFLNPNADYDDEFLRYLWREYLHPKQYEWVLIVG
YIIVFIIALIGNILVCVAVWKNHHMRTVTNYFIVNLSLADVLVTIICLPATLLVDITETWFFGK
TLCKVIPYLQTVSVSVSVLTLSCIALDRWYAICHPLMFKSTAKRAQQSIVIIWIVSCAIMIPQ
AIVMECRSVFPELANKTILFTVCDERWEGQIYSKVYHICFFCITYMVPLCLMILAYLQIFRK
LWCRQIPGTSSVVQKKWKPLQCSIQSKGQQSTKSRNNAVAAEIKQIHARRKTARMLMVVL
LVFALCYLPISILNILKRVFGMFTHTNDRETVYAWFTFSHWLVYANSAANPIIYNFLSGKFR
EEFKAAFSCCCRGIHNNQDDRLIRGRASTESRKSLTTQISNCDNVSRLSEHVVLTNINTLNA
NGSGAVHNW*
>jgi|Xentr4|166886|fgenesh1_pg.C_scaff...
MDRRLNITPETLSQLLKEHNMTRLQFIAAYGLKPLVYIPELPLSAKAIFLVLYIIIFILALFGN
SLVVYIIIRKKAMRTVTNIFICSMACSDLLVTFFCIPFTLLQNISSEWLGGRFVCKMVPFIQTT
AVVASTLTMTCIAVERYQGIVHPLKMKRQYTNIRAYKMLGCVWSVAIVVGSPMLHVQTLE
VKYDLLYNLYHVCCLESWSDVELRRAYAIFILVALFLVPLAAMLLLYTRIGYELWIKKRVG
DCSVLNTLSRNEMAKITRKKKRAVMMMVIVVLFFTACWAPFHVVHILFEYNNLEDEYDD
VTVKMIVAIVQAVGFFNSFNNPVVYTFMNENFKKSLVAILFCHFRAQEPVEPADKRDSQQS
ERPKGGTTMFFARRDGRSGQSHLSAENLELRLCEQFPAARLESVAYPLVNAHKDLLPNGQ
SA*
>jgi|Xentr4|167251|fgenesh1_pg.C_scaff...
MDPTFYCALFLLMFGNSLVREENCISVPGFYFPGCDDINECQENANICGANMQCNNTMG
GYKCRCDSGYQPKGNKTEFCHNKNYTECEDIDECGTNTHKCGVNAKCINKKGSYQCQC
ESGYHRKGNTNKFTPTDNKEQNKCEDIDECDINTHKCGANAKCKNTPGSYQCQCESGYR

RKDNTTEFTDEFTPTDNKEQNKCDVVCKEGDGDKKSCDSNDSFQCALDDFISSLTPLCAQ
LVNDHSKKIQQLLDQLNGIFSRMSRQSLEERLKSTGKVLKQAERLARNLSRLNHENITLTS
NNRQIELRTQKGSRRSRMISVKSKRSRLEVDPNTAAADSTDDFPLVGFLEYNSLAPLLEGA
PIVGGSNSSPVILISPVISTFLSREDTSSLNQSIRFRLEHTAINSTIDPNRTTCAFWSQDDTAWS
TAGCITLESSEQETNCSCVHMTSFAILMAVNNVEEIVKSWPLTLITLIGLSVSIICLSLCIITFIF
CRSLRGTRNTIHTHLCLSLFLGHCTFLLGINAHNNPVACSVVAGLLHTFYLASFCWMSLEG
VELYLMLVQVFNTHLKKRYLLLIGYGVPLAIVAISASVYPAGYGTQLHCWLSLERSFIWSF
MGPVCVILLVNSGIFVLTVWKLAEKMSQINPEMEKYRRIRSLTVTAVAQLSILGCCWVFGF
LQFSSSSLFFAYAFSILNTLQGLQIFILHCLMSKKVRADYSRWLSAIARCKAPTYSEFTSTSQ
SQTQTR
>jgi|Xentr4|167332|fgenesh1_pg.C_scaff...
MASENVSGFIIQGFSDTPELQISLFVLFLVIYLIILLGNLIVSLVISCNPHLHTPMYIFLLNLSLI
DISFSSNILPNLLHILLTQQNNISFLGCMTQLYVFGSLADIEYFLLTAMAYDRYVAICDPLHYI
ARMSRKHCAGLITAAFTVGFLDTVSIVTLIPKLSYCASRIINNFFCDVAPLLKLSCSSTFSVE
LVIYIEGTLLLFNSFLLTLTSYIFIISAILKIQSSEGRQKAFSTCASHMTCVITLYGTVICLYMR
PTTSYSLERDKYFSLLYIVLGPVLNPLIYTLKNREFLSSLNKVKQKCLHYKS*
>jgi|Xentr4|168918|fgenesh1_pg.C_scaff...
MEQDYSELSNTTELWFTSLSQLDIFGRVTPTNASISNSTTYYDMTSNAILTFIYFVVCIVGLC
GNTLVIYVILRYAKMKTITNIYILNLAIADELFMLGLPFLAMQVALVHWPFGKAICRIVMTV
DGINQFTSIFCLTVMSIDRYLAVVHPIKSAKWRRPRTAKMVNAAVWTVSLLVIMPIMTYAG
VQSYHGRGSCTIIWPGNSSAWYTGFIIYAFILGFLVPLSIICLCYLFIIIKVKSSGIRVGSSKRK
RSEKKVTRMVSIVVAVFIFCWLPFYIFNVSSVSLLIVPTPGLKAMWDFVVVLSYANSCANPI
LYAFLSDNFKKSFQNVLCLSKVSGMDEVDRSDSKQDKSRLNETTETQRTLLNGDLQTSI*
>jgi|Xentr4|839|gw1.3026.1.1
YIEIQTLLFTINEINKNPDLLPNVTLGYHVYDSCGDPSLAMKSVLQILSGPGIIVPNYSCGD
QREIAVFIGDRSTVTALPIAQLLGTYGHTQISYSAIDPVLNDRALYPYYFSTGPNDYIEHVAI
AELVEYLGWTWVIILAAGDERGERESKNLMNELNKHGACVDLIGTLTGDINTDTRTLERI
QKSTAEVVVLCGEKFRTKTYFVEKMIKEKTLVVPATWIPGYTFPLFNGSLAFKEGFVIFDN
SEFEDYVMKIKEDVLLKDLLTVASSCLTHDKEKDTLLQKVYRVSYRNCSGSKLLQFFGRS
YRVYTAVYGFAHAEHDMRSSSGKYCNKCIHKNINRKQLHHFLRKTHFKDPRGQNVSFTE
LGFLDLCYFISSIYSFIRYVMQRNYVGTYTWSEPKGFLEIDIQKIIWKKNTSNQVGQTYLLC
SIHYGAHLIIHECSERLLDRSDRIFHNIFVPCVTFSTLAQLFRIFCAQKIETQTPQLYQCRTAV
HYILTTLKNFHFWSYYLFNWNRLKGALELQTTSFGITFSIAISCVLAKTIMVSVAFKATKPD
SSWRKLAGAKLANTIVLVSSIIQVVISVIWLAISPPFAEQNIHSEPGKIIIQCNEGSVVAFYIVL
SYMGLLASVSFIVAFLARSLPDSFNEAKYITFSMLLFCSVWITMIPAYLSTKGKYTVAVEIFA
IISSSCGLLFCIFLPKCYIILLKPEMNT
>jgi|Xentr4|3178|gw1.1343.11.1
TLTGNLLILLLIFTDSHLHTPMYFFLGTLACLDVSYSSTTAPRMLFDLLRERRIIIVPDCITQV
FFFLFFGASGLFVLAVMSYDRYIAICRPLHYMQIMSWNFCVQLVSGILVINTVYSLVHTLCL
TKLTFCRPNVLQSFFCEIPQLLQASCSDTIINVLLIFLLGTLFGIGVLGGTFYPYIPIITTVLKM
TSKHSRSKAFSTCSSHLTVVFIFYSTVLFNYFRSNANNHLVEDKVASVFYAILPPSLNPVIYS
LRNQELKLSLRRSL
>jgi|Xentr4|105215|gw1.24.642.1
MQMNNHSGVTEIFFLGFQHLNNFKILIFVLILLIHILTVYENALVIALVTVSRGLQSPMFFFL

QQLSFSDLLETMVILWNFLSPPPDFLCQAFQLPFPFCGSQAALQLPVALSLLFRLLLGSSSQ
HKDTVSVLPPTGNSSRRGVKLTLPISWLLALSFIPVAVIPAATQEFCNQNTINHFFCDYFPLL
ELSCSDTSLARILAITVSTPVVLFPFMFIIGSYICIAHEILKIVSSIGRQKAFSTCSSHLAVVSIF
YGTLIGIYVVPTRNQSQTISKLLSLLYTVVTPFINPMIYSLKSTDMKNAI
>jgi|Xentr4|156969|fgenesh1_pg.C_scaff...
MHKVNQTSGISFLLLGFQNSQIINEFLFVLFLWIYIVTLFGNLLIIILVITVSALRSPMYALLS
QLSLVDVLLSTSITPNFLWLLLNGGGTISATGCITQFYFYGVSTGSEFYLLTAMAYDRYLAI
CSPLHYASIMGFRLCLFMSLCSWGLALILALIANLLTFNLQFCGPFVIDHYFCDFAPLIKLSC
TDYKAVELTVIIFDIPFMLLPFCFIIYTYVAIGLAILRISSTEGRHKAFSTCSSHLIVVCTDLPIS
LCVIHGPGDSYRMAVMFLSKENNIGINFSLKLGETENNIGNNFSLKLGEIENNIGINFSLKL
CETEYNIGINFSLKLGEIENNIGNNFSLKLGEIENTIGINFSLKLGEIENNIGINFSLKLGEIEN
NIGINFSLKLGEIENNIGINFSLKLGEIENNIENNIGFKFSLNLGEIENIIGINFSLKLGETENNE
GINFSLKLGEKENNVGINFSLKLGEIENNVGINFSLNLGEIENNIGINFSLKLGEIENNVGINF
SLKLENNIGNNFSLKLDEIENNIGINFSLKFDEIENNIGINFSLKLDKIENNIGINFSLKLDEIK
NNIGVSFSLKLGEIENNIGIKFSLKLGEIENNIGINFSFKLGEIENNIGVNFSLKLDEIENNIGN
NFSLKLGEIENNIGISFSLKLGEIENNS*
>jgi|Xentr4|168472|fgenesh1_pg.C_scaff...
MALLIEAFADDYLVIVDKANIITFIIFWRRDCGVSPSCRCYMMAISVADTMVIIQIVIIEMILQ
YHTLEPFWSRIPWCMIRDVLAYGAYNSSVWLVVCFTIERFVAIKSCHLKPKFCTRNCTLCII
ALVFICSYLFSVPYFWANESQKLNNTERYTCVYNTHPPTPIYAEGLVWFQTTFVYIVPYIIIF
TLNGLILRQICQSNKVQCGMQPNHESSPSLRKLCKQKMRSVVLLVTVSMTFAYLCTTRFV
TQIIIKTLHYGQERRDYTKAINVAADIGTMLDLSNTAINMYLYACTQARFRKELSEAGKALI
NLCRVSQQQKQMSVVFHISLSKCVQK*
>jgi|Xentr4|11017|gw1.592.14.1
VSLHNRISFRHYRHLLVLIYTIGEINKDPEILPNVTLGYQIYDSCASGIISFASALSILSGTEQT
IPNYSCWNNRKVVGFIGDLSSESSLSIARLAGIYRYPQISYGSADPIFNNRLEFPSFYRMIPN
ELSEIDAIMSLIRHFGWKWVGLIVSDDFTGHRASERLQNQMNKDGGCLAFLIRLKLWSDI
NRSEVNITDIYRPIAQTIYKTTANVIILCISSQYINSFNIFFAFNKMPKKIWIASSSFSRVIELQY
SQTPVTFNGTLVLSFQQGEIPGFKQFFYSVNPYTYRDDTLFSKIWEMLFSCRFSDLVRRKT
AKISLPICTGNETFDDTVLESYGTFNYRIAYGVYTAVYTMAHTLHELYGTMTRSPKSAESL
HMYFKQWQLNALMRHVAFRTSSGDQIYFRDNGDPPANYDILKWYFFGEGNIQRIKVGSF
DTSRSDGDQLFINNSANLWGPYFSEFAHSRCSEPCKPGFRKAKVEGAPSCCYTCVLCADG
EMSNITGICTKCSKYEKSNSRRISCILKEMNYLSYDEHLGSTFSSISVILSITCAVILGIFIKYR
ETPIVRANNRYLSCLLLISLMLCFLCTLLFIGRPTQICCLLRQVTFGIVFTISVSSVLAKTLTV
IIAFNATKPGSKLKKYVGTQLAIILVNVCSLGEIIISIVWLASSPPFPEDEILSDADTIILLCNE
GSGCFFFCIIGYIGTLALLKFIAAFLAKDFP
>jgi|Xentr4|11312|gw1.190.35.1
FAIEEINQDQNILPNVTLGFHIHDSWANERKAISSTFSMLTGSSDYVPNYKCTQNRIPSAFIG
HLLSSVSNVMYQITSIYGFAQVSYGAVDPAFSDRIRFPLFYRTVPSETAQYQVIIQLIKAFN
WNWVGMISSDDETHQKASEEMKDEIIKNGICVAFLVQIGERDGTSLRFALQAIRQANASV
VIVYSRTVIFLFCLIYLYNLSTNVVWLMLSPLASASGELYNNEFNGSLVIFFRQGNIPGLKDF
LYKADPSRFPKDPFTAAVWLEVFDCHSVQAFPKSPSRSYRICNANDTLKRHVKYGYDVNC
FRLTYSMYIAVYAVAHALHMDKASGGTIMYSDKTTNGLMPTQVLNLNHHLKKIHFRTDT
GDEIFFNEKGVVLGHFDIINWNIFHNGTVTTRHVGTFNSSQSPGLAIDKETILWASCFNGIPI

SRCCASCLPGYRKTFVKGKQVCCYECVQCSEGEISTLPDMENCKRCPEDQWSNESRDKCI
MRTIDFLSYKDLFGMALTTAALVLSCFTSAVFCIFLTYKKSPIVKANNQELSYILLLSLMLSF
LCSLLFIGRPTKATCLLRQTSFGIIFAICISSILGKTITVIIAFTATRPGSRLRNYVGTRVPKYIL
LLCTLPEVFICALWLIISPPFPDYDTHSATGKIILQCNEGSPSAFYIMVGYIALLAFVSFFVAY
LARKLPDIFNEAQYITFSMLLFCSVWISFILAYVSTTGKYLAAVEIFAILVSSAGLLGLIFIPK
CYFILIKLPVNT
>jgi|Xentr4|169656|fgenesh1_pg.C_scaff...
MRTKLVKSSKIQENNVKSEKRTHINWWDHKKGVECRKKNQNRGVKLKMENITKTTVTE
FTLVAFSDFHEFQMIIFIIVLLMYITCILGNITIITLVRTEPSLQTPMYFFISVFAVLEILFVSVTV
PKLLDNLIAHNKNISFAGCFAQLYAFSSLGETECSLLAIMVFDRYLAIHNPLRYSAIMTHRF
CTGLAALPWIIGFVISFIPTVSTAVLDFCGPNELNHYYCDLAPLQNLACSNPLISNALTSLTA
ALTVASPFFVIVGFYIHIIYTIVSKMNSRESKYKAFSTCSSHLIVSSMFYGSGITVYINPKGSK
YDRFLALTFTVITPLLNPFIYTLRNKNVKEAFRKSFGQLLKQLPIKFHNQTSQSHPSHMLSS
LHF*
>jgi|Xentr4|169661|fgenesh1_pg.C_scaff...
MNQTNETTVKDFIFLAFSSFHQFQIFLFIVIQLAYIGCLIGNISVIILVRAKPSLHTPMYFFIST
LSALEICFASAVIPKLLASLIAADNTISFAGCFAQLLVSDSLGATECFLLAVMAFDRDFAINN
PLHYKVIMTQNTCIGLAALPWALGFITVLIPTIYMARSKFCGSNEINHFYCYLAPIKNMICS
NQFTTILLTNSAAIFTTILPFIIILGFYTHIIVAISKIKGTKSKQKVFSTCSSHLIVVSLIYFAGIF
GYLAPKDGPYGSFFGLLYTVVTPILNPFIYTFRNKEVKAALWKSSALRM*
>jgi|Xentr4|169663|fgenesh1_pg.C_scaff...
MIHEDVLLLICPCIERVYMENWTSLYAEDFPYQSAELKKQLSSEISSVTPCVAEELDKIVVN
EFILLAFPSFHQTLLFVIIQLAYTVSVTGNLFIIILVGVEPLLQTPMYFFISILAVLEIIYMSAIH
PRFLASLISKNNRISFIGCFVQMFVADTLGAAESYLLAVMAFDRDLAINRPLHYPAVMTKR
FCIGLAVLPWLIGTVIMLIGTMLTANLEFCGPNEIDHFFCDFPPLQILACSDSLVSNAVTSVL
AVIGCGIPFFIILVVYIRIIITVSKIKTSKGKQRAFSTCSSHLIVASLYYVTLGIVYFKPNGNPY
KKYFTLMYALISPVLNPFIYTLRNKDVKAALSKSKLLNIVPCLFHYSQSR*
>jgi|Xentr4|169683|fgenesh1_pg.C_scaff...
MVYAILEVNNSSDILPNITLGFQLYDSCYNEVRSLMGTSWILSGKENFVPNFNCHRNIMPS
AIVGDMPSKASMPIARILGLYRYPQVSYASAHPLLSDKTQFPSFLRTISNDNYEVFAIARLV
QYFNWTWVGIITSDNDLGRSGTQLLTKEIESNGGCIAFQEILPLYNSMESVYRIVDVLKKT
KATVIIVYSTMENLISLMEVASFNNITDKVWVGSGSWSITSDFPRREILTTLNGSLGLAPQS
GKIPGFKEFLYSIHPTKYPDDIYIKTFWELAFGCVWPANKSTPLKEGSVWCTGTERLDTIDT
SVYDVYNFRYTYKIHNAVFAIAHALHQMNTCVPGHGPFKNGSCADIHNHQPWQLLHYIK
HGKFNNTAGEPVFFDENGDVPIHVDILNWQLFPNGSNQYVSIGSFDAGSEKGHELKIEQK
NILWNQGQTQVPTSVCSDTCPRGHRRATRQGQQICCFDCVPCSEGEILNPFDDSECIRCPE
DKWPNSKKEICLPKVIQFLSYEEMLGTSLACISVLLCLLTFSVFCLFIAKHKTPIVKANNRE
LSYLLLISLMLCFLCSLVFIGRPNMITCMIRQVVFGVIFSVCLSVILAKTMTVILVFSATNPDS
KIRRLVGLLCTMVQIILCIIWLTTEAPFAEFNMAAELGKIVIECNEGSKVLFSCVLGYMGVL
AAISLLVAFLARKLPDTFNETKFITFSMLVFASVWVTFIPAYLSTKGKQTVAVEIFAILSSSAG
LLVCIFFPKCYIILLHPEMNSKQYITGRNVRYRGT*
>jgi|Xentr4|169748|fgenesh1_pg.C_scaff...
MALRSGGGLGCWASGLVLYGLLFLLFPVSRARFHHLMLKDDVRQKVLLNTFGFFKDGSM
NVTINSFSVKDNNDFSNLDSSTFGFSLDRTRNDGFSTYLDQELRACILKSPPTDVPIMLLK

MDFAKKLVFVRSAKHDMPIVSVSEKVEQPPKNISKAEEPAPLPAKMSRRDTADQKKDDAA
DKKEGTAEAASKDDKDATQEANQKKVEKKEKEQKGPIEYNGTMHVDKGVASFQFSFNIS
SEELEGLYSLYFHSCVGNDNSGKQHFFSLDINITEKNTESYLSADEIPLPKLYISMALFFFLA
GLAWVHILRKRRNDVFKIHWLMAALPFTKSLSLVFHAIDYHYISSQGFPAEVWAVVYYIT
HLLKGALLFITIALIGTGWAFVKHILSDKDKKIFMIVIPLQVLANVAYIIIESTEEGTTEYGLW
KDILFLVDFLCCGAILFPVKWSIRHLHEASATDGKAAINLAKLKLFRHYYVMVSSQETSPP
FVHTSALIFPYSYRSQLGPFNGYFQLLTNSQNKPLPVTTTGATEGVKKVKKVVNGSSEPRS
DWESTA*
>jgi|Xentr4|169805|fgenesh1_pg.C_scaff...
MNNHSGVTEIFLLGFQHLNNFKILIFSLILLIHILTVYENALVIALVTVSRGLQSPMFFFLQQL
SFSDLLDSMFIVPTLLSTCFLLTEMSYDRYLAICNPLRYSSIMSHRVCVKLIALSWLLALSV
TSVVVIAAATQEFCNQNTINHFFCDFFPLLELSCSDTSLARILIMSITVPAVLFPFMLIIGSYIC
IAHEILKIVSSIGRQKAFSTCSSHLAVVSIFYGTLIGIYVVPTRNQSQTIGKLLSLLYTVVTPFI
NPMIYSLKSADMKNAIKIIIQ*
>jgi|Xentr4|170485|fgenesh1_pg.C_scaff...
MFATVSLLFCLLLQPSPSAQQYHGEKGISVPDHGFCQPISIPLCTDIAYNQTIMPNLLGHTN
QEDAGLEVHQFYPLVKVQCSPELRFFLCSMYAPVCTVLEQAIPPCRSLCERARQGCEALM
NKFGFQWPERLRCENFPVHGAGEICVGQNTSDNSPSGPTARPTPYLPDSITFHPHPNRDFT
CPRQLKVPPYLGYRFLGEKDCGAPCEPGKANGLMYFKEEEVRFARLWVGIWAILCGISTL
FTVLTYLVDMRRFSYPERPIIFLSGCYFMVAVAYTAGFLLEERGVCVERFSEDSYRTVAQGT
KKEGCTILFMILYFFGMASSIWWVILSLTWFLAAGMKWGHEAIEANSQYFHLAAWAVPAV
KTITILAMGQVDGDILSGVCYVGINSVDSLRGFVLAPLFVYLFIGTSFLLAGFVSLFRIRTIM
KHDGTKTEKLEKLMVRIGVFSVMYTVPATIVLACYFYEQAFRDTWEKTWLVQTCKGFAV
PCPNYNFAPMSPDFTVFMIKYLMTMIVGITSSFWIWSGKTLQSWRRFYHRLSNGGKGETA
V*
>jgi|Xentr4|171005|fgenesh1_pg.C_scaff...
MVLHFVLLLLLLRDDGNGNVWIMASAQTNERRVVSYMAGDIIIGALFSVHHQPTVDKVH
ERKCGEIREQYGIQRVEAMLHALDRINADQTLLPNISLGCEIRDSCWHTAVALEQSIEFIRD
SLISSEEEEGLVRCVDGSSAPSRSKKPIVGVIGPGSSSVAIQVQNLLQLFNIPQIAYSATSMDL
SDKTLYKYFMRVVPSDAQQARAMVDIVKRYNWTYVSAVHTEGNYGERGMEAFKDMSA
KDGICIAHSYKIYSNAGEQSFDKLLKKLRSHLPKARVVVCFCEGMTVRGLLMAMKRQNII
GEFLLLGRCTPLPILSQQNKSKVSPGSSAVKQPFTQSQIIVLRGLTYSILEVDLCSVVRSNLG
NYGERGMEAFKDMSAKDGICIAHSYKIYSNAGEQSFDKLLKKLRSHLPKARVVVCFCEG
MTVRGLLMAMKRQNIIGEFLLLGSDGWADRYDVTDGYQREAAGGITIKLQSPDVKWFD
DYYLQLRPENNLRNPWFQEFWQHRFQCKLKGYSQENQKYNRTCNSRMSLRSQYVQDSK
MGFVINAIYAMAYGLHNMQTVLCPGYAGLCDAMKPIDGQKLLDSIMKANFTGVSGDMIT
FDENGDSPGRYEIMNFKKMGKDYYDYINVGSWDNGELKMDDDEIWSNKNLVIRSVCSDP
CAKGQIKVIRKGEVSCCWTCTPCKDNEIVFDEYTCKACDLGSWPTDDLTGCDPIPVEFLR
WSDPEPIAAVVFSCLGLLATMFVMAIFILYRDTPVVKSSSRELCYIILAGICLGYLCTFSLIA
KPQQIYCYLQRIGIGLSPAMSYSALVTKTNRIARILAGSKKKICTKKPRFMSACAQLVIAFV
LICIQLGIIVALFIMEPPDVIFDYPSIREVYLICNTNNLGVVTPLGYNGLLILSCTFYAFKTRN
VPANFNEAKYIAFTMYTTCIIWLAFVPIYFGSNYKIITMCFSVSLSATVALGCMFVPKVYIIL
AKPERNVRSAFTTSTVVRMHVGDGKSSSAASRSSSLVNLWKRRGSSGETLSRPMSFIRPRV
PDVSKALSYAKEYGSVSLVHLGNVQMCFFVYFSSNGKSVTWAQNEKSSKSQHLWQRLSV

HVKKKDSTNQTAVIKPFSKSSSENRYISTSFPDSSAKMLYDVSEAEEHYPVQYRPQTPSPIS
TVSQRAISTTRTEDDIPSFHSDTAQRSGSSQGSLMDQISSVVTRFTANISELNSMMLSANTP
GTMVPTPLCSSYLIPREIQLPTTMTTFAEIQPAPNIDFNGGTQSVRKSTDSVKDSTSEAAAV
KQDVEELVALTPPSPFRDSIDSESTSPSSPVSESALCIPSSPKYDNMIMRDYSQSSSSL*
>jgi|Xentr4|171846|fgenesh1_pg.C_scaff...
MTRKCARAPRREPAQGADGAAGVPADGSAAGVPAVPPQCANMTRHVWFSLPYASKPFRR
TAASNSSGIFSTLYGFPNISSLFNGDLNIDDIGDFENDTKYEAESQSSTVKALLIVAYSVIIVIS
LFGNILVRFVSSTWVFGKLMCHVSRFVQYCSVHVSVLTLMAIALDRHQVIMHPLKPRMST
VKGIICIIVIWFMASGFSLPHAVYQKLEQFFIGNTVRMVCLPSFPYPADLFWKYLDLATFVL
LYVLPLIVISITYTTVAKKLWLRNAIGDITMEQYYAHRRKKKMTLKMLMLVVVVFAVCWF
PLNCYLVLMSSKVISSNNAIYFAFHWFAMSSTCYNPFIYCWLNESFRSELKALLCVCRRRS
PAQSHPLQSVSPQFRHAWTENCHFKQGSSSQAAGSATNVNSGRTDISSVEPIVTVS*
>jgi|Xentr4|172056|fgenesh1_pg.C_scaff...
MVTLSNPRANGFLLLGFIMQRDMNVTLFVVFLMIYMTSLFGNIIIISLSRTEASLRTPMYFF
LSNFSFLEICYTSVTVPKMLFDLLSGNMAVSFNCCLTQMYFFFLFGTTEFYILALMGLDRY
VAICHPLHYSVIMNRQFCYQLILGSWLSGCIVPVLPTTLLTQKPFCGSKIINHFFCDVGPLIK
LSSPDTFYLDAIVFTVSGIVVLCSLTLVTVSYLFIISAILRISSSAGRHKAFSTCASHFSVVTLF
FGTVIFMYLRPTSGENYELDKVVSVFYSIVTPALNPLIYSLRNKDMKRALVKMLQKRDNS
CRKLENINSISLNTFYRKH*
>jgi|Xentr4|173175|fgenesh1_pg.C_scaff...
MDHNFSSDNGSQTHFTFFSDFKTNNKVAVTVLETLVMSLVFIVSIFTNISAIILMVKKKRLV
TANCFVLNLFCADLLFISMIPFILVIRWTEVWVLGDFICHMHFYIICLSGCVTLISLSAVSLER
MISIMKITQATTCNVKVVVCGLLGIWVFSAFTALPMCLFFNVVEQKVNGTVIHICTLVWPN
VGEEIAWDVSFIILNFFIPGLIIVVSYTKIFKITKSVRNRLISCTTYPENNQMKVSHRDYKLFR
TLFILMISFFIMWTPVAIIVLLLLLQNLHKHVSIPPTVFFWITTLTFSNSVLNPILYNINLFRQK
WVHIILCHSVEEIADTETTTKRNENANISHGTF*
>jgi|Xentr4|173333|fgenesh1_pg.C_scaff...
MASHWNEAVFAARRRNDDDDTTRSSVFTYTNSNNTRGPFEGPNYHIAPRWVYNISSLWM
IFVVLASVFTNGLVLVATLKFKKLRHPLNWILVNMAIADLGETVIASTISVCNQIFGYFVLG
HPMCILEGYTVSVCGIAALWSLTVIAWERWFVVCKPFGNIKFDGKLAATGIIFSWVWAAG
WCAPPIFGWSRYWPHGLKTSCGPDVFSGSSDPGVQSYMLVLMITCCIIPLAIIVLCYMHVW
LTIRQVAQQQKESESTQKAEREVSRMVVVMIIAYIFCWGPYTFFACFAAFNPGYNFHPLAA
AMPAYFAKSATIYNPIIYVFMNRQFRNCIYQLFGKKVDDGSEVSSTSRTEVSSVSNSSVSPA
*
>jgi|Xentr4|173335|fgenesh1_pg.C_scaff...
MSKGRADVRMEMPDDFYIPIPLETTNISSLSPFLVPQTHLGTPGIFMSISAFMLFTIIFGFPLN
LLTIICTAKYKKLRSHLNYILVNLAVANLIVICFGSTTAFYSFSQMYFSMGTLACKIEGFTAT
LGGIIGLWSLAVVAFERFLVICKPMGSFTFRESHAVLGCILTWVMGLLAATPPLLGWSRYIP
EGLQCSCGPDWYTVNNKWNNESYVIFLFCFCFGFPLAIIVFSYGRLLLTLHAVAKQQEQSA
TTQKAEREVTRMVIVMVAGFLVCWLPYASFALWSVTHRGELFDLRMASIPSVFSKASTVY
NPFIYIFMNRQFRSCMMKMIFCGKNPLGDDEETSVSGSTQVSSVSSSQVAPS*
>jgi|Xentr4|173423|fgenesh1_pg.C_scaff...
MDNECAYTGVEENNTLEAIVGDRNGTYRCLFNEEFKYILLPVSYGIVFCLGFVLNTLALY
VFLFRVRPWKVIHLFMFNLALSDLLYVLSLPLLVYYYSKENDWPFSEPMCKIVRFLFYTSL

YCSIFFLLCISIYRFLAVCYPIQFLRWGHTRCARAACVSIWVVVTGAQSPVLYFVSTSNMEG
NIICHDTSSISLFDQFVLFSTVTLVLLYCVPFVVILLCYSLMVFTLMKPTANASPNVESKRKS
IRMILLVLIVFVVCFLPFHVTRTLYYYERKTDLDCGTLNTVNVLYKVTRPLASANSFMDPIL
YFGVWRFRINR*
>jgi|Xentr4|173955|fgenesh1_pg.C_scaff...
MGRNNQTFFQQIVLSGFQMPQSFKIPFFLLLFISYSVALSSNVTILTLVSSSPSLHHPMFFFLS
HLSLSDIILTTSIVPTMLHGILRGELSMPIPACITQLQFLGIALTSECFILAIMSYDRYLAICNPF
HYISVMDSKFQIWLVVCCWVLSFTISFLTVIFVSKLEFCGFLVINHFFCDVAPLVQLSCSDTS
VMELENLVVAIPTTSFPVLFITATYICIFITILRIPSITGRQKAFSTCSSHLSVVAIFFGTLVALY
VVPSNGHSLTANKVLSLLYAVITPLFNPMIYCLRNKEIKAALKKLVRCVCK*
>jgi|Xentr4|173968|fgenesh1_pg.C_scaff...
MCENNSMVTQFLMLGFQGSYSFKIMIFTIFLLIYAVILTGNLLIILLTSINKNLDIPMYSFLKH
LALVDILFTSNILPKLLHVTFTDGDLIAKSACIFQYYIHSISIYTQSLIITVMSFDRYLAICHPL
RYSSIMNQKLCFYLIFCSWATGFFLIPSEFIMISQLKFCDSYIIDHFFCDIDPILALSSSNTAIVK
WEDFALSILLLFLPCLFVLLSYIYIFLTISKISSFAGKKKAFSTCSSHLATVCTYYGSIITVYIFT
DGKRSLQENKLRSLIYTVLTPFFNPIMYSLRNREIQHALKELITKKKRQRNGTSRG*
>jgi|Xentr4|174015|fgenesh1_pg.C_scaff...
MNKSTSLKFLLEGISEDPDLQIPLFLVFCLTYLLTLAGNIVIITLICQTPALHKPMYFFLCNLS
FIDMSYPTVIQPKLLSMLLTGDRSIAFSACMFQMYFFIILTCAEIIVLTVMAYDRYVAICRPLT
YPLLMNKKTCNFMAAATWALSSLDPVPHTVSISRLTFCPPLLINHFYCDPFFVLSSSCTDTF
LIDIFTYVFGTTVTCCAFICITISYSFIISTIVKIKSVGGRQKGFSTCGSHLTAVILFYGTILIMY
TRPSSQYSSSNGKPFSVIYTVLIPMANPFIYTLRNKEVKTAFWKVIKNKM*
>jgi|Xentr4|175043|fgenesh1_pg.C_scaff...
MNHTQFNGFLLEGFLNLNDLRILLFFCFLSTFLLTLAGNMLVILLVQCHTRLHTPMYLFITN
LSFLEIWYTMTTAPKLLAILLTKDNRISYEWCFAQLYMFHGLGMTECALLAVMAFDRCMA
ICNPLRYTSIMNERMCRALAILSWTFGFFASLIPTTLTHKVPLCGPHYIDHYFCDLAPLLAL
ACTDTSFTSAMNSSVIGFATMFNLVFIIVMYISIIWSIMKLRSNTGRMKAFSTCSSHLIVVV
MFYSTAFTVYATPKGSWPTSYDKLIAVVYTIVTPMLNPIIYTLRNNEVRIALKETIQSKRVT
LKLC*
>jgi|Xentr4|175051|fgenesh1_pg.C_scaff...
MEDFNNTAVKEFILLAFSRFPELQVLIFIVVLVMCMISVIGNLSIIVLVKTEHSLHTPMYFFIS
VFAGLEILFMSVTIPKLLANLVADYKNIAFIECFAQLYAFNAFGVTECCLLAVMAFDRNLAI
NNPLRYGAIMNPRLCLTLAAIPWVGGFVIGIIPIIFTADLDFCASKEVNHFFCDLAALQNLSC
SDPIISNVATSIITVFAGLAPFMLIIGLYIHIIITISRIRNVEGKKKAFSTCSSHLIVASLFYGTVII
MYIRPSFSDLDKFIALIYTVLIPFLNPFIYTLRNKDVKEVFKKFTFPKHNFKETSN*
>jgi|Xentr4|175055|fgenesh1_pg.C_scaff...
MDKANMTAVEEFILLAFSDLYQLQILLFFVTLLVYIMCVFGNFLIIFIVIAEPSLHTPMYLFIS
KLAALEIMYVTCIVPNLLANLIAGKKSISFSGCFIQLLFSSTMGTAECYLLAVMAFDRDLAI
SKPLHYSAIMTQGLCVRLAVLPWIVGIITVFISTVCTASLEFCGPNEIDHFFCDFYAVQSLAC
SSIFITQVVTNSGAFFAAVLPFTVTAGLYTHIIIIIYKIKSAESKKKAFSTLSSHLTVAGLYYITA
IIVNAVPKDSHYNKFLSLIYTVITPLLNPFIYAFRNKDIKKTLIKSRRIKLCQKLSQY*
>jgi|Xentr4|175058|fgenesh1_pg.C_scaff...
MDKANKTTVEEFILLAFSDLYQLQIPVFFVTLLVYIMCVFGNCLIVIIVRAEPSLHTPMYFFI
STLSTLEIIFVSAIIPNLLANLIADKKKISFNGCFIQLFVGATLGTTERYLLAVMAFDRVLAIN

KPLQYVSIMTQWVCVELALLPLIVGIIIALIPTVHTAGLEFCGPNEVHHFMCDFGPLQSLAC
SDSFSSQLVTISGASFAIVLPFITTIGFYMYIIVIISKINSTESKQKAFSTCSSHLTVVGLFYITTI
IVYATPQGTQYDRFFALIYTVITPLLNPFIYTFRNKDVKRALIKSKKLQLCQGLFL*
>jgi|Xentr4|176917|fgenesh1_pg.C_scaff...
MWSCEDLNYTNSSEEQYLCNEFHLILFIFSVLYLIICFPVGLCYNVQLVLVNLYNKATMTM
PDVYFVNMAIAGLIINAVAPVYLLGPAYTKWSLWSFGNEVYITLLILFNVSSLVIMYSTTLL
SLDYYIECALPRTYMSSVYNTKHVCGFIWGGAVLTSFSSLLFYICNHISTKIIECSKMQNRE
AADAIMVLIGYVVPIIAVIYALVLILQIRKDTTPLDQESGRLDPSVHRLLIATVCTQFILWTPY
YVTLLVNTFMDARIKSSDTFYIRTFQFIEGLSNFLAFSSSFVLPLIHRHINKNFSGKLQRLLK
KLHCGSQGCTHEHSVVQQVMT*
>jgi|Xentr4|177109|fgenesh1_pg.C_scaff...
MAGIIKKLGQGSNKFFDVTAVEGQWLEWGAWEKCSVTCFNGTQQRSRRCANTNQAGRD
CRGQETETRECSNPDCPASHEVCKDEYVLSMSWKKASAGEIVYNKCPPNATDRHTGQLK
EAVLQFLSLPAWLTGLTAVVLMHARDSNASMMCFGQNCLALIIRFTVKMCWLYRKSHSK
VTRVMARGTSPGYKNGGPDVHVYAQDTQLDGENDGRNLREHLAKGQRVLAGQGMSQV
VRSMLGLVSSKNYYSGDLLFCVDILRNVTDTYKRATYIPSFEDIQKFFHAVSHMLDAENRE
KWEDAQQVAPGSILLMKVLEDYIHLIGDALKPFQSSIIVTENLVISIQREPVSAVSSDINFPM
KGRRGMNDWARNSEDKLFIPKEVLSLPPAEAEDSAYFVIGAVLYRTLGLLLPPPGNSLAISS
KVLTVTVRPLTKPAEQLVVVELSHIINGTSSPQCAVWDYSKSDASTGNWDTESCQTLPTQP
SHTKCLCDQLSTFAILAQLPKEMPPLVLGLAISYSFAARSYHPRIQGLPLKFGIQTDIDIQVM
DVPGSPSVPLMIGCAVSCMALLILLAVYAAFWRFIRSERSIILLNFCLSILASNILILVGQSQA
LSKAVCALIAAFLHFFFLSSFCWVLTEAWQSYLAVIGQMRTRLVRKRFLCLGWGMNKIIFC
SFFVTDCLASYNASLCEKFWKRRSTSPGCCSFRWIYKNKGIWNTSLSSGGFPQTVHNTLS
MMNGFAFAPLSCWLSLEGGLLYAFVGPAAVIVLVNMLIGIIVFNKLMSRDGISDKSKKQRA
GASLWSSCVVLPLLALTWMSAVLAMTDRRSILFQVLFAVFNSVQGCVIITVHCFLRREVQE
VVKCQIGGCRSDENEDSPNSCKNGQLQIMTDFEKDVDLACQSEEKASWYTAHGETVLFK
ELNTCHPSSVTGALSRISLDDEDDLKLHKGSDGLSFSHLPGNISSPGGILLQVPKITHNPVN
ELNDPLPKKEINLERGPLYISPDGNRWPMDFGDGQLEPSLESEYMVLPRRTLSLKPFGREH
GKLNVKLADLSKGRQQGDSEGYPSFLSVDHINVNLTPPFVPSQHQYGLPLKQPPSVRQILA
SEMNERSRTMPRTVPGPSLSAGSLEKPHQPVPVFLPQRKRLRYSDLDFERDKRWSISSGGG
VERTSASEKTSPAGPHQKVWDSYRARSSQIHDRLGLHCVDWNQPPRISMDVSDGDYQTE
V*
>jgi|Xentr4|177256|fgenesh1_pg.C_scaff...
MQKKGKWVQVSVEGMQKKGKWVEVSVEGMRKKGKWVEVSVEGMQKKGKKLMSNSS
VYSAEQYKKKMLEVNFKISESYRGETSEPLQIRSETEDLLANMANSGHGIYDDLDVETSSI
FNYDYSTSSESNDVAPCDLQTTIIFDRSFLPAFYSILFLLGILGNVLVMVVLLQNRKRLQST
DIFLLHLALADILLVVTLPFWATQAVSGWLFGNVLCKTVASIFKINFYACTFLLVCISCDRYL
AIVYAVQVYKKHRTNLVHWSCLFVWCLCVGLSVPDMVHFQVAYEPRTNVTECQPVFGSS
NFKTWRVSMAFLYHIVGFLLPLCFMLYCYTHIIHTLFQTHGFKKQRALRVIITVVVAFFLC
WTPYNIVALLDTLNLLHVLADNCTIDSNIDIALSVTSGLCYFHSCLNPLLYAFVGAKFKKK
LVELLSKLSCICPQIVKNYIKHSPSAKSSTWSESGETSISRM*
>jgi|Xentr4|177721|fgenesh1_pg.C_scaff...
MSGGWPGGQACQPAAQMALEDVNNRRDILPEYELRLIHHDSQCDPGQATRYLYELLYND
PIKIMLMPGCSSVSTLVAEAARMWHLIVWSLQSKFPIAFTQHSTWVFNCGTLNSSLSFSQT

LDDLEERVKEAGIEITFRQSFFSDPAIPVKNLKRQDARVIVGLFYETEARKVFCEVYKERLF
GKKYVWFLIGWYADNWFKIPDPAINCTVEEMTQAVEGHVTTEIVMLNPENTRGISNMTSQ
EFMEKLKKRLGENPEMTGGLQEAPLAYDAIWALALALNKTAYELSKKGRRLEDFNYNNK
MISEEIYRAMNSSSFDGVSGHVVFDASGSRMAWTLIEQLQGGVYKKIGYYDSTKDNLSW
YNNDKWIGGSPPADHTKVIITFRYVSQKLFISVSVLAGLGILLGFVCLSFNIYNSHVRYIQN
SQPHLNDVTAAGCILSLAAVFPLGLDGRHIETWHFPLVCQARLWLLGLGFSLAYGSMFTKI
WWIHTAFTKKDDKKERRKQSQTLEPWKLYTTVSLLLGLDVITLGIWQIVDPLQRSIEEFTK
EEPRGDLDVLILPQLEHCSSLKMNTWLGIVYGSKGLLLLLGIFLAYETKSVSTEKINDHRA
VGMAIYNVAVLCLITAPVTMILSSQQDASFAFSSLAIVFSAYITLVVLFVPKMRRLITRGEW
QSEQQDTLKTGSSTNNNEEEKSRLLERENQELGRIIAEKEERVMELRQQLRSRRRPSVPAR
DNCSDNHFPNSSGAVSSLYQPGLPLASIINSSSIFIS*
>jgi|Xentr4|178178|fgenesh1_pg.C_scaff...
MQEENTTFILEFHLLGFQNLHSLKIVFFIFLLVTHIATLSVNIFLLITVRFHRHLHSPMYFFLT
QLASSEMLFTTTIIPKLLHMTLREGITVSVSSCIIQLYLFSVSTNAESIFLTVMSYDRYLAICY
PLRYSSLMDFSCTYVTTMQKENQTVISEFLLLGFSSLRRFKYSLFTTFFILYVASIAGNSLVI
AIVSISQSLQSPMYFFLRQLSIAEILFTTNIVPTLLSVVFKDGDTISVPGCIVQFIMFSGLTNTE
CSILIMMSYDRYLAICKPLHYTMIMNSTLQLKLIIGSWVGGFITTLSVVLQICVLQFCSPNV
FDHYYCDVAPLLKHSCSDVSFLEMETFVFSTPLTIFPFVFIIVTYIRILFAIFQIPSNIGRQKVF
STCSSHMAVVCMYYGSLVTIYVVPSIGNTWNVKKFLSLLYTLATPFLNPIIYSIRNQEIRMA
MQKHFCRKKAMYM*
>jgi|Xentr4|178485|fgenesh1_pg.C_scaff...
MSQTTFSLERNMEDIEIPNIHNRMFSSLVNLSHIYFKKFQYCSYAPHVRSCKPNSDGISSFE
NLLANILLRVFVWVMACVTCFGNLFVICMRSFIVTENCQHTMSIKSLCCADCLMGFYLFC
LGAFDIKFQGEYNKHAQAWMESWECKLVGSLAMLSSEVSVMMLTYMTLEKYLCIVFPFS
HFRAGRGQTLCTLTAIWGLGFFITVIPFFSYDTFGNFYGRNGVCFPLQSDSTEKYGARGYS
VSIFLELRNDHGKGILGKWLGLNLLAFVTIVFSYSSMFYSIHKTGAKTAERSVLSREVTIAK
RFFFIVFTDALCWIPIFLLKAVSLTEAEIPGTITSWIVIFILPINSALNPILYTLTTSSFQEKLKQ
CLQSKRQESSKSFTLVSIQMNTV*
>jgi|Xentr4|179038|fgenesh1_pg.C_scaff...
MENQTNSTCLFYLAFSNHGEKQPLLSIVFFLIYVIGVLGNLIIIIVIYLDSHLHTPMYFFLFSL
AFVDICYPTVTLPKLMDILLSGNNSITFVQCFTQMYFFLALAAVEATLLSSMAYDRYVAIC
KPLRYHHIMNRRVCVLVIVGTWVSGFANSAFLTFLASKLLICGSNKIKQFFCDIKAVADISC
DRTAFYNAIYAVGGISCDRTAFYAAIYVEVFFFGLMTISLNLMSYINIIKNILNIKSKHGRQK
AFSTCTSHFTVLIIFYGSGLWTYLRPPSESVKLDSVFTVLLVGVTPMLNPLIYSLRNKEVKN
ALKRIVRKKSACP*
>jgi|Xentr4|179046|fgenesh1_pg.C_scaff...
MSGNTSEFHLLAFLIFKEFHMGLSIGVLLMYLLSVIGNMTIIIIVFKDSQLHTPMYFFLCNL
AFQDIVYVSAILPKLLVITVSGNGNICFQCCITQMFLFAFCVGIEFFLLTSMAYDRYVAICVP
LHYTIIMNKRLCALLAFSSWFFAALNSLMHSVLISNLSFCNVQQINHFFCDVKTLLWLSCA
DTRNIQLLISVEGIVLGFIPFLLILISYMYILSTILKISDASGRLKAFSSCSSHLIVVVLFCGTFL
SLNLKPEAHHSEEQDKLLSLIYIAVVPALNPLVYSLRNKEVLRALKKQFLKKIKINTF*
>jgi|Xentr4|179385|fgenesh1_pg.C_scaff...
MEGNFTHVEIFILLGFSHVPQVRLFLIPTFTLMYACTILGNSLIILLIREDHRLHTPMYFFLA
NLSMMDLLFTTVTVPKMIGDLISREGMISFRSCIAQLFLIIAVGASESPLLSVMAVDRYVAIC

DPLHYKTIMSNKLCFRLASGSWLLGSFYSLIHTLLTNSIDFCGPNQLSHFFCDVVPLFQLAC
SDILLNVALVYVSAFFNGICNCSVILGSYVSIITAIMRIRSQEGRGKAFSTCASHLLVVIVYY
TTLTANYLQPMSSNSGSKDRTGAVIYTVVTPVLNPIIYSMRNNDVKKAVKFGIPCPETRYPE
SSELRKGHFP*
>jgi|Xentr4|179805|fgenesh1_pg.C_scaff...
MAAYVISCLWLSVVLAQKSMGHKKPFHPMVNLECSRDFRPFLCALYAPVCMEYGRVTLP
CRKLCQRAYNECFKLMEMFGVPWPEEMECSRFPDCDEPYPRMVDISLSGDVTEETPLAV
QRDYGFWCPRELKIDPDLGYSFLGVRDCSPPCPHMYFRREELSFARYFIGVISIVCLSATLF
TFLTFLIDVTRFRYPERPIIFYAVCYMMVSLIFFIGFLLEDKVACNGANPSQYKAATVTQGSH
NKACTMLFMVLYFFTMAGSVWWVILTITWFLAAVPKWGSEAIEKKALLFHASAWGIPGT
LTIILLAMNKIEGDNISGVCFVGLYDVQALRYFVLAPLCLDVVVGVSLLLAGIISLNRVRIEI
PLEKENQDKLVKFMIRIGVFSILYLVPLLVVIGCYFYEQAYRGVWETTWVQERCKEYHIPC
PYKVTQTSRPDLILFLMKYLMALVVGIPSVFWVGSKKTCFEWASFFHGRKKKAGVNESR
QVLQEPDFAQSLLRDPNTPIVRKSRGTSTQGTSTHASSTQLAMLDDQRSKAGSVHSKVSS
YHGSLHRSRDGRYTPCSYRGMEERLPHGSLSHLTDHSRHSSTHRLNEQSRQGSIRDLSNPL
AHISHGTSMNRVIEEDGASA*
>jgi|Xentr4|180046|fgenesh1_pg.C_scaff...
MGCTWTSFGLTHLGTPLLGTKRSVEIAIAISMALMVTFLEIMQKQLGSKDGLCLPEWDGV
VCWPEGLPGNTVSISCPEYIQDFNHKGHAYRRCDMNGSWELVPGNNRTWANYTECAKYL
FNETKEREVFDRLYLIYTVGYSISLGSLTVAVLILGYFRRLHCTRNYIHMHLFISFMLRAGSI
FVKDAVLYSGSALEETELISEEDLKFITEIPPADKMQFVGCKVVVTFFLYFLAPNYYWILVE
GLYLHSLIFMAFISDKKYLWGFTLFGWGVPAIFVLIWARERATVADTGCWDLSAGYLKWI
VQVPILAAIMVNFGLFINIVRVLATKLRETNAGRCDTRQQYRKLLKSTLVLMPLFGVHYIV
FMAFPYTEVSGILWQVQMHYELLFNSFQGFFVAIIYCFCNGEVQAEIKKSWSRWTLALDF
KRKARSGSTTYSYGPMVSHTSITNVTTRGTLALHLNTRLVSTALNGHRNLPGYVKNGSLS
ENSIPSSGPDQYNKDEEYINGSGLYDGERPTPAVVEEERETVM*
>jgi|Xentr4|181160|fgenesh1_pg.C_scaff...
MAEEFNVHQDEHSYEDNYDYIHNWTDLEYFLNHTFSTCDVDLNENVKQVILFILYLVIFV
MGLAGNLLVLWVNWQSQRSKSSINLYIFNMAVADLGVVFTLPFWMLETMLEYTWLWGG
FLCKFTHYFYFANMYSSIFFLMALSVDRYFTLVSSSVFWRQNQQRIRRALCIGIWVLAGIFP
LPEVVHMKLMDAVEPVCVFMAPLESYDKWSIAVSLLTVIIGFLIPFSIILVFNLMTAYYIRCS
GRPESRKHCCLIYAYILTFLFSWLPYHVTLFALTLHGSYIFLHCQLVHILYFFYEVIDCISLLH
CVVNPILYNFFSKDFKGKFINAVVKYIPKEKITGDGKEEHSTSTTDHSIVITKEGTQQTNHM
HPLPIP*
>jgi|Xentr4|181406|fgenesh1_pg.C_scaff...
MTHWFHRVPVGLARTLLLTTHPLSLTSLLPACSGLSPTAGALCIPLYVPYVLTGRWGFGRS
VCKLWLVLDYLLCTSSVFNIVLISYDRFISVTRAVSYRAQQSNSRHAVLKMTMVWILAFLL
YGPAIITWEYIAGGSIIPDGECFAEFFYNWYFLMTASTLEFFTPFISVTYFNVSIYMNIQKRT
KARLDLLQEAQSHTNTDNIKAPPEKARSSSLKCWKLHQKMARFGGSDHQKTIKLTESEM
MERVRGMKRNSLNGSTVTAMGKSRSARRSFNESSSTPSLEKKMRVVSQGIAQRFRLSRDK
KVAKSLAVIVCAFGLCWAPYTLLMIIRAACRGHCVPNYWYEASFWLLWVNSAVNPILYPL
CHNSFKRAFRKILCPQKLKLKANGKFHYCWK*
>jgi|Xentr4|181423|fgenesh1_pg.C_scaff...
MENQTIVYVLVLAGLSDLPFLRLPLFFVFLFIYLMTFFGNLLILLLIFTDSHLHTPMFFFLGT

LACLDMGYSSVTSPRMLFDLLTSRRIISIPACITQNFFFLFCAESELSVLAVMSYDRYVAICR
PLHYMQIMNWKVCAQLVSFMLVLCLVISSTHTFFLTKLIFCSSRDLQSFFCDLPQLLQVSCS
DTFINILLIYLTGIYSVSFISVVTFYPYITIIGTILKIPSKQMRSKAFSTCSSHLTVVFIFYTTSFV
NYLRSNTNDQHAQDKIATVVYAILIPFLNPLIYSLRNQELKSSLRKALQRM*
>jgi|Xentr4|181425|fgenesh1_pg.C_scaff...
MYTYLQEEIQREDGLDGCLNHDMEKFGVTMIISDVLKIFPCQKVCYATKTFLALMPSMSS
FGSLPQDSMGLDRSNLAKTQFRSKMENQTIVDMLVLTGLSDVPSLQLPLFLVFLLIYLMTL
SINLLIFILIFTDSHLHSPMYFFLGTLACLDMSYSSVTVPRMLFDLLTQNRVISVPACITQIYF
FLSFAVSEMAVLAVMSYDRYIAICRPLHYMQIMSWNVCVRLILIILVFGAVHSLLHTLFLTQ
LTFCSSNTLQSFFCDLPQLLQASCSDTFINVLLIFLFSVLYGVGILVVTFYPYIPIITTVLKISA
KHTRSKAFSTCSSHLTVVSIFYSTAFFNYFRPNANDHFNEDKVSVFYAILTPFLNPLIYSLRN
QELKTSLRKTLHRHKILHG*
>jgi|Xentr4|181687|fgenesh1_pg.C_scaff...
MDITNQTDIPEFVLRVFRGSSKYHLILFSILLLFLLITLTGNLFVIFIICVNRHLHSPMYLFIGS
LSSIEICIISSVIFNLLAILLTNDTHISKAGCFLQSYIYYFFCISDFLILGIMSFDRYVAVCNPLK
YNSIMQNIVCVKLVIGCFVTSFLCLLYPTLMITNLPFCGHMLDHFFCESAALMNLICGDITL
IKLTSLITAVFILIGSLTLTITSYIIIVSAILRLSSDTGRKKTFSTCLSHLTMVGIVFGSAIFILIRPP
RQYSTETDQVVALVATVMGPLLNPFIYTLRNQKVKDSIRAAIKYIKVHIL*
>jgi|Xentr4|181816|fgenesh1_pg.C_scaff...
MNVSVQISSQDSCHPESVLIPVLYSLIFLVGTVGNSLVLAVLLRNGKVNNTTNLFILNLGVA
DLCFIIFCVPFQATIYTLDSWVFGPFMCKAVHFFIYLTMYASSFTLTTVSLDRYLAIRYPLRS
RELRTPKNALLAITLIWSLSLVFSGPYLSYYQEFQLSNLTVCHPVWQDSYRRAMDLCTFAF
SYVIPVLILSLTYARTIRYLWTAVDPIEDMSESKRAKRRVTRMIIIVAVLFCLCWLPHHLVILC
VWFGYFPLNNFTYALRILSHLVSYANSCVNPVVYALVSKHFRKGFRKIFRCLLLRQRAANK
HLCCPQFKTVPSPFLMAPCQHPDSETSL*
>jgi|Xentr4|182743|fgenesh1_pg.C_scaff...
MASLSEFVSEPIGMMSQTSAASESQCYYNETIAFFYNRSGKYLATEWNAVSKLVMGLGIT
VCIFIMLANLLVMVAIYVNRRFHFPIYYLMANLAAADFFAGLAYFYLMFNTGPNTRRLTVS
TWLLRQGLIDTSLTASVANLLAIAIERHITVFRMQLHTRMSNRRVVVVIVVIWTVAIVMGAI
PSVGWNCICDLEHCSNMAPLYSDSYLIFWTIFNLVTFVVMVVLYAHIFVYVRQRTMRMSR
HSSGPRRNRDTMMSLLKTVVIVLGAFIVCWTPGLVLLLLDVCCPQCNILAYEKFFLLLAEF
NSAMNPIIYSYRDKEMSATFKQILCCQRTENVNGPTEGSDRSASSLNHTILAGVHSNDHSV
V*
>jgi|Xentr4|183264|fgenesh1_pg.C_scaff...
MPVINILLYLTVMCVGHCRSETPTYNPACHLEIIETTEEYEYIQEGDIMIGGVLTVSMFRQA
NYFTGLTPSAQNYKYLLDFLYVIEQVNKNPDAFPNTTFGYHIYDSCGDLRKAVKSVLQILS
GTREPVPNYSCTGMRQMAGVIGDLTSETTIPIAQILNVFGYTQISYGATDPVLSDRFSFPYF
FRTTGSDDRYYFLISKIAKYFQWNWVGIITFDDDRGERDHQLLKYYLSSENICIEFTLKITHI
IHGNMRYREIIQKSSTNVVIFCGAVNMQIVLEFNYLYDMLSEKTFILTSNWLDYYHVLDFS
HKVFQGSLILMQNKEEYPSNSPYAQFSKQFHPSRYPDDKLLENIWLLYNFCLPKNHKKDE
FYKKQFIWHHRHNCSEQESISKIPIYERGFHTFNMICAVGMFFRALMHYPPGMHHYLRRA
RYVGTDHVYSFNENGEFDTRYLIINLYFKLGKQSAWKTCGTYTPWAPEHLKLNITPGKIQ
WKTKDNKVPRSQCSESCPTGFRKTLRLRAQPCCYDCVPCSEGEISNRTDSENCFTCPYNE
WPNEKRNQCIARIEEFLSYSDNVIPVFFSVISALLLLINVMISGVFISYRDTPIVRANNRSLSF

LLLVSIKLSFLSVFLFLGRPVDITCMLRIITFGITFSIAVSSLLAKTIMVCVAFKATKPGSSWR
KWLGVKLSNSVVLFCSSIQIIICMTWLAISPPFQELDIHTSPGTIIIQCNEGSAIGFYSVIGYM
GLLAACNEGSAIGFYSVIGYMGLLAAVSFVLAFLARSLPDSFNEAKYITFSMLLFCSVWIT
MIPAYLSTKGKNTVCVEIFAILTSSAGLLASIFLPKCYTVLFRADINIKSHLLVMKT*
>jgi|Xentr4|184056|fgenesh1_pg.C_scaff...
MKQGCSRILLSSCFLHITLSSINSCNQTKITQFLLLGFHGPQILRNCLFYLCLLIYMVVLGGN
LLIIALYLGSHHLRSPMYLFLTYLSITDILLSTSVVPNLLSTLLSDGKLMSVSACVTQFFASG
FFTGGECLLLTVMAYDRYLAICKPLHYVTIMTNKHCLHLVIWSWVEIAVISVSVATATSHSG
FCGCNTLDHIYCDYLALLEVSCADVSFIESFIPIVNLPLVVLPILFILITYVCIFHSIFRISSNTG
RQKAFSTCSSHLTVVCTYYGILFAKYAVPSKGQSLNVNKIISLLYVMVTPLFNPIIYSFRNQE
IWRALSKWMSIGCP*
>jgi|Xentr4|184731|fgenesh1_pg.C_scaff...
MANGSETEELSGSLSQSTVISTHLKLVLLGFIICASLAGNLLVSLLVLKDRSLHKAPYYFLL
DLCLADAVRSSACFPFVLLSIRNGSAWAYSLLSCKVVAFMAVLFCFHASFMLFCISVTRYM
AIAHHRFYSKRMTLWTCIAVICMVWTLSVAMAFPPVFDVGTYKFIREEDQCIFEHRYFKA
NDTLGFMLMLAVLIVATHIVYAKLLLFEYRHRKMKPVQMVPAISQNWTFHGPGATGQAA
ANWIAGFGRGPMPPTLLGIRQNTHTANRRLLGMDEFKSEKRLGRMFYGITLSFLVLWSPYI
VACYWRVFVKTCSIPHRYLSTAVWMTFTQAGVNPIMCFLLNKDLKSCLQLHIPCWRTKAL
LPREPYCVM*
>jgi|Xentr4|186152|fgenesh1_pg.C_scaff...
MASPLEIFLLTLIWIVTAVGILLNAFIVAMPVIWWARYNKVEMIEFLLASVGMSRVVLLILW
EVVNPLVPSSSILFGVVSMFLSFWSLWVATILCVFYSVKISSCHHPFFMFLKLNISKMLLGLF
LVSLASSLLFSLPFKWLVYSTSINNATNSTNTTNSTGQGGTILEVNVINQYFLILIGSSLPLFI
FCVAVAILIRSLWRHTRNMAEGNVDFGNPQIQAHLSAVKSMVSFLVLFTIYFVIVIVMSLPP
LLDQNVLQLVFNIICSAYPLLHSLILVMYSRKLREALYWCLHCTCRVPSTERGSA*
>jgi|Xentr4|186393|fgenesh1_pg.C_scaff...
MRYVYLALGVWGYLTTVLSNVSVITVILIHKALQEPMYIFICALCFNGLYGSLAFYPGLFIN
LLQKVQTISYTSCILQVFAIHTYGGCEMTLLAVMALDRYVCICDPLRYNSIMSLAVVYRLIV
AASFYSFIFITIHIILTVRLTLCDSAILKIYCDNWSVVRLSCIDTTVNNTFGMVVVLALMVA
MPSLIFISYMAILRVCVKSSQDFRAKALKTCTPHLITITNFIVDVLFEIFLYRFTPTAIPYALRV
FMSVHFLVAPPLLNPLIYGLKIREIRVKIIQQVRGTFQRSC*
>jgi|Xentr4|186396|fgenesh1_pg.C_scaff...
MANQSAISEFVLIGFPGLQQSFHIPVSIAMFLIYSVSLVANSLVLILIILKRTLHHPMYIIIGNL
AFSDALFDTMTLPKIIAKYWFDAGSISYYGCVFQLFCVHYLGTIDSFIIMLMAIDRYLAVCH
PLRYHSIISNKLVVILCYLLWIVTIFIVCPVAITTAQLPYCGPNAIRNCFCSNVSLTPLACANN
LFAVQLGYIIAMVVLLLPLAIIILSYILIIRAVHLLASEENWQKTFYTCTTHLFVIALFYIPRVF
VYTSAQLRLILNPDINVLLLCLYTFIPHAANPIIYCLRTQEIKDILRHTFHITFRIKIQQKSPGL
Q*
>jgi|Xentr4|186398|fgenesh1_pg.C_scaff...
MTISMPNQTAVTEFILLGFPGLQPNFFLPVSLTLFLAYIVSLIANSTVIILIILREQLHQPMYIII
ANLALSDLLYDTITLPKIIAKYWFGAGSISFNWCFFQIYCVHHLGCLDSLIIMLMAIDRYVAI
CHPLRYHSIISNKLATLLCYFLWPFAALNGLAMTLISIQVPYCGPNRVKNCFCASQFLIVLA
CVDVALEKKKRFIIIMCVHLSPFIVIILSYILIIRVVHLSTNNENWQKAFYTCTTHLLVIGLYFI
PRLFLHVINQYPLILDADANVLIVCLYTFIPHLASPIIFCLRTKEIRNNLRQMFNSICPRAEHR

PRTPGKRHCIKFYCE*
>jgi|Xentr4|186863|fgenesh1_pg.C_scaff...
MPQGTKGKRPERPGYSSAAKQQVSFRHYRHLLVLIYTIGEINKDPEILPNVTLGYRIYDSC
ASGIMSFANALTILSGTEQPIPNYSCWNNRKVVGFIGDLSSESSLSIARLAGIYRYPQISYGS
ADPIFNNRLEFPSFYRMIPNVLSEIDAIMSLIRHFGWKWVGLIVADDDTGHRANKRLQEA
MSKDGICLAFLIILRDWSQCYWAYVMEIRKTFYRSSAKVIILFLSSQSVSYISVLFDNDNIPH
KIWIATSSLSLISELKYLPVLVTFNGTLVISLQQGEIPGFKQFFYSLNPYTYQRDDLFPYIWG
LLFDCTFSDIDTSRKNCTGNETFDDTVLSFYGTFNYRIAYGVYTAVYTMAHTLHELYGTM
TRSPKSAESLHMYFKQWQLNALMPHVTFRTSSGDKIYFKDNGNPQARYDILKWYFLEIG
NKKSIKVGSFDGSESDGKLFVNESANLWGPYFSECVHSRCSEPCKPGFRKAKVEGAPSCC
YTCVLCADGEMSNITDAQICMICSKYEKSNSVRNRCIPRNIDYLSYGDHLGSTLSSISVILS
VTCAVILGIFIKYRETPIVRANNRYLSCLLLISLMLCFLCTLLFIGRPTQICCLLRQVTFGIVF
TISVSSVLAKTLTVIIAFNATKPGSKLKKYVGTQLAIILVIACSLVEIVMSAVWMASNPPFPE
ADTLSDPDYIILLCNEGSVTFFFCIIGYMGTLALLSFIAAFLAKDFPDRFNEAKNITFSMLGF
CSVWGAFVPAYLSSKGSRMVAVEIFAILSSSAGLLACIFIPKCYIIFLKPELNTKETIIRKQ*
>jgi|Xentr4|187269|fgenesh1_pg.C_scaff...
MELKEIFNKNYSFILVGFSDLPELHYPLLVSFYLVYVLTLIGNTVIVAVVLMNNPLHSPMYY
FLCNLAFLDICYTTTTLPKLIQLHSEQDGTICFASCLLQQYFYISFAVSEYFLLAAMAYDRY
VAICKPLHYSVLMSRKVCVLLAKGCWVIGFVSSSVPISFSSGFQINHFFCDLTALLTLACND
TSAIEFMIFAGGVLIVLNCFLLTVTSYGFIGFAIIRISSSKGRFKAFSTCASHLTVVILFYTMIT
CLYMKPSSSYSLNDGKVLSVFNVQVIPMLNPVIYTLRNKDIKEAVKIISTMLNGCRYNLGQ
YCLL*
>jgi|Xentr4|187329|fgenesh1_pg.C_scaff...
MNTTSSCNHSKITEFLLVGFSAPRPLRELLFSICLVIYIMALGANLMIIALYLGSHHLRSPMY
FFLSNLSATDILLSTTVGPNLLCSFLKDGNPMSVSACVTQFFAYGLFIGGECLLLTVMAYDR
YLAICKPLHYVTIMTNKYCLHLVIWCWVEILILSLSGTIFTSVFGFCGCNTLDHVYCDFAPL
LKVSCSDSFVMGSLTTIFIPTGVVLVFLSPLLFIITTYVSISISIFRISSKTGRQKAFSTCGSHLT
VVCTYYGILISKYAVPSKGQSGNLNKLTSLLYTLVTPLFNPIVYSLRNQDIQKTMGLIY*
>jgi|Xentr4|187330|fgenesh1_pg.C_scaff...
MNTTSSCNHSKITEFLLVGFSAPRPLRVLLFSICLVIYIMALGANLMIIALYLGSHRLRSPMY
FFLSNLSATDISLSTSVGPNLLSTFFTYENPMSVSACVAQYFTAGFFTGFECLLLTVMAYDR
YLAICKPMHYVTIMTNKHCLHLVIWCWVEMLLLSSCSTLFTSVFGFCGCNTLDHVYCDFA
PLLKASCSDAFVMASMTAILIPTGVLILFISPLFIIITTYVFISLSIFRISSKTGRQKAFSTCGSH
LTVVGTYYGILISKYAVPSKGQSRNLNKLTSLLYTLVTPLFNPIVYSLRNQDIQKTMTALISR
LIFMFTKNSLLPLATSHN*
>jgi|Xentr4|187545|fgenesh1_pg.C_scaff...
MASESKENISGFIIQGFSDTPELHISLFVLFLGIYLIILLGNLIIFLVISCNPHLHTPMYIFLLNL
SLIDISFPSNVFPNLLHILLTQQNNISFLGCMTQTYVFVSLAASEYFLLTAMAYDRYVAICDP
LHYIARMSRKHCAGLITAAFTVGFGDPVGLVVLISKLSYCASHHINHFFCDVTALLKLSCS
STFSVELLIYIETILLGFNAFLLTLTSYIFIISAILKIQSSEGRQKAFSTCASHLACVITLYGTAF
CLYMRPTTSYSLKRDKYFSLLYIALGPVLNPLIYTLKNGEFQSSFNKLSVDCFLLSCGEKK
NQSLNRLTKQ*
>jgi|Xentr4|187549|fgenesh1_pg.C_scaff...
MWGKNQNQHEGRLEQKVSESKENVSGFIIQGFSDTPELQISLFVLFLGIYLIILLGNLIIFLVI

SCNPHLHTPMYIFLLNLSLIDISFTTNVLPNLLHILLTQQNNISFLGCMTQMYVFVALASSE
YFLLTAMAYDRYVAICDPLHYIARMSRKHCAGLITAAFTGGFGETVGFIVIIPKLSYCASHLI
NHFFCDITPLLKLSCSSTSNAELFIYIEGALLTCNSFLPTLTSYIFIISAILKIQSSEGRQKAFST
CASHLACVITLYGTAFCLYVRPTSSYSLERDKYFSLLYIALGPVLNPLIYTLKNKAFQSSFN
KVKQRVKQELFYFR*
>jgi|Xentr4|187558|fgenesh1_pg.C_scaff...
MVSENNVSGFIIQGFSDTPELHISLFVLFLVIYLIILLGNLIIFLVISCNPHLHTPMYIFLLNLSL
IDISFSSTVLPNLLHILLTQQNNISFLGCMTQMYAFVALASSEYFLLTAMAYDRYVAICDPLH
YIARMSRKHCAGLITAALTVGFGESVSPIVLISKLSYCASHLINHFFCDSIPLLKLSCSSTFSV
ELVIYIEGTLLVFNAFLLTLTSYIFIISAILKIQTSEGRQKAFSTCASHLACVITLYGTALSLYV
RPTTSYSLKRDKYFSLLYIALGPVLNPLIYTLKNREILSSFNKARQRLLAFLF*
>jgi|Xentr4|187560|fgenesh1_pg.C_scaff...
MVSESKENVSGFIIQGFSDTPELHISLFVLFLGIYLIILLGNLIVFLVISCNPHLHTPMYIFLLN
LSLIDISSTSNVLPNLLHILLTQQNNISFLGCMTQMYVFVTLAASEFFLLTAMAYDRYVAIC
DPLHYIARMSRKHCAGLITAAFTVGFVEPVGHLVLVPKLSYCASRLINHFFCDVTPLLKLS
CSSTFSVELLIYIEGTLVVFSSFLPTLISYIFIISAILKIQSSEGRQKAFSTCASHLACVITLYGT
VICLYLRPTTSYNIKRDKYFSLLYIALGPLLNPLIYTLKNREFKSSFKKITFIYFR*
>jgi|Xentr4|187703|fgenesh1_pg.C_scaff...
MASETKGNVSFFIIQGFSDTPELHISLFVLFLGIYLIILLGNLIIFLVISCNPHLHTPMYIFLLNL
SLIDISFSSNIFPNLLHILLTQQNNISFFGCMAQMYIFGSMVGSEYFLLTAMAYDRYVAICDP
LHYIARMSRKHCAGLITAAFTVGFINTVGVVVLISKLSYCASRLINHFFCDITPLLKLSCSST
FSVELFIYIDGTLLPFNAFLLTLTSYIFIISAILKIQSSEGRQKAFSTCASHLACVITLYGTILCM
YMRPSTSYSPKRDKYFSLLYIVLAPALNPLIYTLKNREFQSSLKQHF*
>jgi|Xentr4|187709|fgenesh1_pg.C_scaff...
MGTGIQENVSGFIIQGFSDTPELQISLFMLFLGIYLIILLGNLIIFLVISCNPHLHTPMYIFLLN
LSLIDISSTSNILPNLLHILLTQQNNISFLGCMTQLYVFVFLAASEFFLLTAMAYDRYVAICDP
LHYIARMSRKHCAGLITAAFTVGFVDPVGHVTFISKLSYCASHLINHFFCDISPLLQLSCSS
TFSVELLFYIEGALLFFNAFLLTLISYIFIISAILKIQSSEGRQKAFSTCASHLACVITLYGTVF
CLYLKPQKSYSLQRDKFFSLLYIVLGPVLNPLIYTLKNREFQSSLNKCQKIRKPLKF*
>jgi|Xentr4|187711|fgenesh1_pg.C_scaff...
MTSQSQENVSGFIIQGFSDTPELQTSLFVLFLGIYLIILLGNLIIFLVISCNPHLHTPMYIFLLN
LSLIDISSTSNILPNLLHILLTQQNNISFLGCMTQMYVFASMACSELLLLTAMAYDRYVAICD
PLHYIARMSRKHCAGLIIASLTVGIVDPVGHVVLLPKLSYCASHLINHFFCDITPLLKLSCSS
TFSVELLIYMEGTLLFLSSFLLTLASYIFIISAILKIQSSEGRQKAFSTCASHLACVITLYGTLF
CLYMRPTTSYSLERDKYFSLLYIALGPVLNPLIYTLKNREIQSSLNKMKQKCFIFLFLC*
>jgi|Xentr4|187713|fgenesh1_pg.C_scaff...
MTSESKENVSGFIIQGFSDTPELHISLFVLFLGIYLIILLGNLIIFSVISCNPHLHTPMYIFLLNL
SLIDISFPLTVLPNLLHILLTQQNNISFLGCMTQMFVFVALASSEYFLLTAMAYDRYVAICDP
LHYIARMSRKHCAGLITAAFTVGLTDPLGIVVCVSKLSYCASHLINHFFCDVTALLKLSCSS
TFSAELSIYIVGTLFVFSSFLLTLTSYIFIISAILKIQSSEGRQKAFSTCASHLASVLTLYGTSLS
LYMRPKTSYSLKRDKYFSLLYIALGPLLNPLIYTLKNREFQSSLNTLKQRCFILCKTIKLHSE
QDSFQYTKIMKEKKSLKLS*
>jgi|Xentr4|187719|fgenesh1_pg.C_scaff...
MTSESKENVSGFIIQGFSDTPELQISLFVLFLGIYLIILLGNLIIFLVISCNPHLHTPMYIFLQNL

SLIDISFPSNILPNLLHILLTQQKNISFLGCMTQMYLFVALASSEYFLLTAMAYDRYVAICDP
LHYIARMSRKHCAGLITAAFTVGLTNPVGIVVLISKLSYCASHLINHFFCDISPLLQLSCSST
FSAELFIYIDGTLLPFNSFLLTLTSYIFIISAILKIQSSEGRQKAFSTCASHLACVITLYGTALSL
YMRPPKSYSLERDKYFSLLYIVLGPVLNPLIYTLKNREFQSSLNKMKHRCFSFFIFV*
>jgi|Xentr4|187720|fgenesh1_pg.C_scaff...
MTSESKENISGFIIQGFSDTPELHISLFVLFLGIYLIILLGNLIIFLVISCNPHLHTPMYIFLQNL
SLIDISFPSNILPNLLHILLTQQNNISFLGCMTQMYVFASLAASEYFLLTAMAYDRYVAICDP
LHYIARMSRKHCAGLITATFTGGFLDPVGLIVLIPKLSYCASHLINHFFCDVAPLLKLSCSST
FSVELLIYIEGTFLLFNSFLLTLTSYIFIISAILKIQSSEGRQKAFSTCASHLACVITLYVPVICL
YVRPTTSYSLERDKYFSLLYIALGPVLNPLIYTLKNREFQSSLNKLKQRCFSFFIFH*
>jgi|Xentr4|187866|fgenesh1_pg.C_scaff...
MASESKENVSGFIIQGFSDTPELQISLFVLFLVIYLIILLGNLIIFLVISCNPHLHTPMYIFLQNL
SLIDISSTSNILPNLLHILLTQQNNISFLGCMTQMYLFASLAASEFFLLTAMAYDRYVAICDP
LHYIARMSRKHCAGLITAAFTGAFVGSVGHLVLIAKLSYCASHLINHFFCDVAALLKLSCR
STFIAELFIYITGTLLIFNAFLLTLASYIFIISAILKIQSSEGRQKAFSTCASHLTNVITLYVPVIC
LYMRPTTSYSLERDKYFSLLYIVLVPVLNPFIYTLKNREFKYSFNRIKNVIYFR*
>jgi|Xentr4|187878|fgenesh1_pg.C_scaff...
MASENVSGFIIQGFSDTPELHISLFVLFLGIYLILLLGNLIIFLVISCNPHLHTPMYIFLLNLSLI
DISIVTNILPTLLHILLTQQNKISFLGCMTQMYFFVSFGASEYFLLTAMAYDRYVAICDPLHY
IARMSRKHCAGLITAAFTGGFAVPVSIVVCVSKLSYCASHLINHYFCDVTPLLKLSCSSTFS
AELFIYIEGTLLVFSSFLLTLASYIFIISAILKIQSSEGRQKASSTCASHLTCVIILYGTVLCLYM
RPTTSYSLKRDKYFSLLYFLLSPVVNPIIYTLKNRDFQSFFNKARQRYLGFLF*
>jgi|Xentr4|187884|fgenesh1_pg.C_scaff...
MASESKENVSGFIIQGFSDTPELQMSLFVLFLGIYLIILLGNLIIFLVISCNPHLHTPMYIFLLN
LSFIDISFTSNILPNLLHILLTQQNNISFLGCMAQMYVFGLLASSEYFLLTAMAYDRYVAICD
PLHYIARMSRKHCAGLITAAFTGGFVESISLIVPLSKLSYCASRLINHFFCDVTPLLKLSCSS
TFSVELFIYVGGTLFTFSSFLLTLTSYIFIISAILKIQSSEGRQKAFSTCASHLACVITLYGTGIC
LYMRPTTSYSPKRDKYFSLLYIVLGPVLNPLIYTLKNREFQLSFNKVRQRGFEHLYFR*
>jgi|Xentr4|188037|fgenesh1_pg.C_scaff...
MKNQTWVSEIVLLGFQNLHNFKVPVFSLFLLIYLLTVWENVLIIVLVAFSRNLHSPMYFFL
QQLSACDLLESAIILPILLQTVIYEKVTLPFIGCMMQFSFFAVPEAFECLLLAVMAYDRYVAI
CIPLRYTSIMSHRVCVTFILISWLLSFMFALSLASTVASLQFCNQNTINHFFCDFFPLLELSCS
DTFLVYINIILESIPLVFFPFILISVSYMCIAHAILKIVSHTGRQKAFSTCSSHLAVVSMFYGTII
ALYMAPPRKESQTTSKVLSLLYTVVTPLVNPLIYSLRSKDIRKIMQRVLNGTK*
>jgi|Xentr4|188157|fgenesh1_pg.C_scaff...
MDPVFHDRTQFPSFYRTIPNEEAEMDGIVQILKHFGWKWVGLIVSDDDTGYRARERISTEL
ARMGGCLAFSFEFEREGYLSSPKEKAIYQTIKSTLVNVIIFYISIRYMDVLTYSFISFCRIPQRI
CITSSLYTNYITSPYNVEQFVIFNGTLSLLIQAGEIPGFHHFLNSFYLHKYENELTGNLYDFLI
NCLNPNSSWTGDYRVTYRVYTAVYALARALHNLYSAQAPTNHWDKLEYMRGNLKPWQI
NTFVRNVTFVTPSGDTIFFNDKGDPPAQFDVMTFLLLPNRTFARQKVGSFPVLSNGTKLLH
INSSADLWGPYYKEMPQSLCNEPCAPGYRKAKIEGKPSCCYDCAKCADGEMSNTTDALS
CFKCFDYEKSNKQRTGCVPKEINYLSYTDTLGASLTSIALVLFIAASVVLGIFVRYWETPIV
RANNQNLSFLLLISLMLCFLCTLLFIGRPTQICCLLRQVTFGVVFTISVSSVLAKTLTVIIAFN
ATKPGSKLKKYVGTQLATILVIVCCLGEMMISAVWMASNPPFLDADTLTDINTVFLMCNE

GSVLFFFSVIGYMTALALFSFIAAFLAKDFPDRFNEAKNITFSMLGFCSVWGAFVPAYLSSK
GSRMVAAEIFAILSSSAGLLACIFVPKCYIIFFKPEQNKRKM*
>jgi|Xentr4|188249|fgenesh1_pg.C_scaff...
MENVFNTSTSFILLGIEEMEHNKYLYCAFLLITYLLILLFSCLIISVVLLDESLHCPMYTLIAN
LMLNGIFGASCFLPKLILDLFFSSKVISRAACFIQSFCVTLFAYCEISTFTIMAYDTYLAVCHP
LRYAAIMTNRTALRLIAGSLIFSLVIVALLLFLSARLPLCGSHINNFFCDYMSIFILSCVSNPLT
KLYGTIVFTAYLTGTMMTIAYSYLRILLVCLKVSRNAGKKAIHTLVTHVLNFSIFLIGTLFIFI
RYRLENINLPLVFHILLSIAGLVFPPLLNPLIYGIRTKALRVRVISHLQKTIVKILIG*
>jgi|Xentr4|188258|fgenesh1_pg.C_scaff...
MEDLRYLYCILXLFLYIFILLLSLGIVLVVLTEESLHEPMYIFICNLTFNGMLGSSSFFPKLIVD
LLASSHQISHVGCFLQVLGMVTYAFYELSSFTLMAYDRYLAVCDPLRYATKMTNTKAVRLI
LSFFGYSFISVLVGVILSARVSYCGTQIKNIFCDNLSLIVLSCGDSSVNNLYGALVTAILLVFT
LLIIAYSYVKIFIVCLKISKEACEKAIHTVVTHLLGFSLFLVGGLFLFIRFRLGNNNLPLFAHI
LLSITFIIFPPLLNPLIYGIRTKALSAKIFHHLHKMRIVSN*
>jgi|Xentr4|188362|fgenesh1_pg.C_scaff...
MATIDTNNSFSHTEFILLGFPGISQSRHWLFIPFFFIYLEILMGNFMIMYRIWVEKSLHFPMY
SLICLLFAVNISCTTAIVPNMLMGLAFGLDHISLGSCLFQMFFIYTAVILESTVLMIMALDRY
LAICRPLRYHNIMNNRLVGQLFLIGLVQSSLFSSSIIIAVSQVQFCRSNIIWNFACENMVLLN
LGCGDISKIQVVGLMVRVLVTAMGISLLLVSYLYIFHSAWRITRGKALHKTLHTCSTHLIVV
VLNYSCGLSSSILYRMPVSVDVQNLFSAIYYLFPATIHPIIYGYRMKEIRMCLVKSLKINR*
>jgi|Xentr4|188437|fgenesh1_pg.C_scaff...
MLNTTSLYGNTTLASWSPKTLEIVKMTLFLGTLLCFCSFLYFVSVILKVFFSTPHVREIARY
VFFVHMLFNDSLHLFVSLFLLMAFLYLIYMPAPFCYFILTLATSTFRVTPYNLAAMALERYV
AICFPLRHVELCTVQKTNVAIALIWVVGLIPNVADLIVMNLSLKTNFFTLYVICNREALTFN
KLQFTIVTVVYIASLALVGLIIIYTYVRVMLVAWKVGLGKSSAFKAAKTILLHGVQLLLYM
VSLVSSFTLTSSSDYSFLTSIVSFLLFMCLPRFLSPFIYGIRDEVFKKRIRNLCGTGAVGQLP*
>jgi|Xentr4|188441|fgenesh1_pg.C_scaff...
MANGTDDLSPIFQLSSSNNKSAVIVKTFFFALLLLFFIMFLYLIAGILNIYFSTPHLRETLRCV
FFAHMLVSDTLYFLVALFLLLSSTYLFQIPMPFCLALQTLATVTYRVTPYNLAVMSIEQYIAI
CFPLRHGEFSTNHNSNVAIAIMWVVGLIPNGAEFLAMSPVVTKDFFLSSALCNQATTVLNP
VQDTIRTFSYIITFILVALVIVFTYIKVTLVARKVTSKQMAASKAGKTVMLHALQLFLCTSG
FLASLTEQYLKDYIPFLFLINFLLFMCLPRFLSPLIYGLRDEAFRKGIRQLFSIRCSR*
>jgi|Xentr4|188443|fgenesh1_pg.C_scaff...
MERSASLNVTTSLLPFVSTPTTVIVLLLLIIFILLCFCIFLYIMAAILKVFFTTPHVRENVRYV
LFIHMLITDSLYLFLSIFVFISAVYMVAMPLPICFAIVILVTTAFVVTPYNLAVLSLERYIAICH
PLRHKELCTERRCNAAMVGMWVVGLIPVIADFITMSYTAGKNSVFSNIMCIPTQMFRVSS
TMQDTMKTIQFLLSLGTVGLVILFTYIKVMLVAFKLGSRSSSAFSAGKTVLLHAFQLLLCM
FSFTYTVTETYLSHYFVFLPVINFFFFMCLPRFISPMIYGMRDEVFRKGIRKCC*
>jgi|Xentr4|188444|fgenesh1_pg.C_scaff...
MANSTDLLAQFSITNSKTGEIVRMTALLVMLVTFCIFLYFALLLLSVIFATPHVREQSRYVLF
AHMIINDTVSLFISVFQFVAAMYSIPIPTVICWVMIIFGKVSFLVTPGNLAVMSLERYVAICH
PLRHAEFCTCQRTNAAILVLWGIGLVPVALEFIIMGYLIGKSVLSVNAICYWLAPEVNRVQT
IIRLITDSVSFTSVGLVIFYTYIRVLLVARRMTSGKSTASKAGKTVLLHGFQLLLCMSSFTSTI
TETYLIAYFAFIRITNFLVLFFLPKIISPLVYGMREEVFSKYVKKRLCPPRGRQLFKKRKVFS

MNGISRS*
>jgi|Xentr4|188445|fgenesh1_pg.C_scaff...
MEVGATKDYYELLAVSVEHDSLEPKSNPTQQDGALRPNAMWNVTSAPGNTTQVSPTNSQ
LGVIIRTTFFVSVLVCLFFFAYLVTIILTVFFTKPSLRENSRYVLFAHMLINDVVYLVMTLYL
AIITSYLPMRLPAHVCYAIVMLSSASYKVTAYNLGVMALERYVAICFPLRHSEFCTRQRSAI
ALVGIWAIGLIPNIADCIILSTSVPSSFFSLHVICARSAFMNTSVQTLIRNLTHILSFTLVGLIIIF
TYIKIMMVALRVDSGKESAIKASRTVMLHAIQLFLCLMAFSNNLLEIYLMEYMYLLPLGSF
FLFMCLPRFISPLIYGIRDEVFRNYIKRFMLCRQLRVHKIKIVVFK*
>jgi|Xentr4|188446|fgenesh1_pg.C_scaff...
MDTNVTVTLIWPQTNRGSIAIVILTLVCFCCFLYSIIVILHIILATPHLRDNTRYLLFAHMLVN
DTIYLVTGLLLFLGAWYLIYLPVPFCYFTYTVATSTYRVTPYNLAVMSLERYVAISFPLRHA
EFCTVRNATVAIVVIWTVALIPNLADFITISSAFGKLRPMVCSQGALSVTPEQNTIRTLTFTIS
FSLVALIILFTYVKVLLVAKSIGSGKSSAIKASKTVMLHAFQLLLCMTSLLSSITERYPFNYIS
LLTVSTFLLFTCLPRFLSPLIYGLRDEMIRKHFIRIHSVRK*
>jgi|Xentr4|188665|fgenesh1_pg.C_scaff...
MVSRSQGNDSEFIIQGFSDTPELQSSLFLLFLGIYLIILLGNLIIFLVISCNPHLHTPMYIFLQN
LSVIDISSTSNIFPNLLHILRTQQNNISFLGCMTQMYVFVALACSEYFLLTAMAYDRYVAICD
PLHYIARMSRKHCAGLITAAFTVGFLDAVGLIVLTSKLSYCASHLINHFFCDVSPLLKLSCS
STFSVELLIFINGILFFFSSFLLTLASYIFIISAILKIQSSEGRQKAFSTCASHLACVITLYGTVL
CLYMRPTTSYSLKRDKYFSLLYIVLGPVLNPLIYTLKNRDFQSSLNKLRQKGLFLLF*
>jgi|Xentr4|188668|fgenesh1_pg.C_scaff...
MASGSKANVSGFIIQGFSDTPELQASLFVLFLGIYLIILLGNLIIFLVISCNPHLHTPMYIFLQ
NLSLIDISFTTNVLPNLLHILLTQQNNISFLGCMTQMYVFVSFGASEYFLLTAMAYDRYVAI
CDPLHYIARMSRKHCAGLITAAFTVGFVDTIGHVVLISKLSYCASHLINHFFCDITPLLKLS
CSSTFNMELFLYIVGALLFFNSFLLILISYIFIISAIQKIQSSEGRQKAFSTCASHLTCVITLYVT
VVCLYLKPTKSYSLDRDKYFSLLYIILGPVLNPLIYTLKNREFKSSFNKMRERIATRKLGKK
II*
>jgi|Xentr4|188671|fgenesh1_pg.C_scaff...
MISESKENVSGFIIQGFSDTPELHISLFVLFLGIYLIILLGNLIVFLVISCNPHLHTPMYIFLLNL
SLIDISFPSNILPNLLHILLTQQHNISFLGCMTQMYVFVALACSEYFLLTAMAYDRYVAICDP
LHYIARMSRKHCAGLITAAFTVGFVEPFGIVLLISKLSYCASHLINHFFCDISPLLKLSCSSTF
SVELLIYIEATLLTFNSFLLTLTSYIFIISAILKIQSSEGRQKAFSTCASHLACVITLYGTAFCLY
MRPTTSYSLERDKYFSLLYIALGPVLNPFIYTLKNREFQSSLNKMRQKCLDNLDKC*
>jgi|Xentr4|188849|fgenesh1_pg.C_scaff...
MDYSDFQTVCEKVEVRNFRSAFLPAMYAIVGLVGLAGNGLVMVRYLYFNRLKTGTDYY
MLNLAIADIVFLLTLPFWAVSVAKTWIFGNEMCKIIYCLYKMSFFSGMFLLMCVSMERYFA
IVQAPSAHRHRSKTVLISKLSSLGIWVFAFLLSIPELLYSGVKENAKVDMCIIFSDSIQSLTAK
LKISQMFFGFFLPLLIMVSCYCMIIRKLLQARNFEKYKAIKVIIAIVIVFVAFQLPYNSVMLI
RAFSNSTECDTSKNLDIADDVTYSLACFRCCLNPFLYAIIGIKFRNDLYKLFKDIGCLSQEK
CSEWSSAKPSKRTSLAMDTETTTTFSP*
>jgi|Xentr4|189051|fgenesh1_pg.C_scaff...
MYAIEEINNSTELLPNITLGYRIYDACTSEAIALMSTFRLLSEEETPALNYICQQDQKLVAFV
GHLLSSITYITAEITQLYGYPQISKIHYFTNLQNLFQISYGALDPVFNDRVRFPSVYRTVPNE
YSQFRVIIKLLKHFGWTWVGIIASDDESNRQASEELRKEMGRNGICVDFLKVILNSEMENK

RVWINSVAPDIVAEYKFKDFLHAFNGSLLISLPKGDIPGFSSFLSQVIWTDTLKNTFVNYLW
GINVGCADVFLYSVKNFTCPNTYKIKEYLLQDETINHRIKKLNYYLKNIHMKMASGEEIFF
TKDGNIPGKFDILNWVIYGNGTIKKIHVGKFFPTTDYLVIHEKAITWGHYFQKMLKIPTSFC
TQKCSSGQRRAHQNGRPPCCFDCVLGKFQILQVFKNHIKLEICVKCPDDHWSNPTRNRCI
KRITDFLSYEDLLGYILSGCASLFIVNTATVLLVFIKHRRTPIVRANNQNISYILLMALLMSF
LCTFMFIGRPTGVTCLLRQALFVCVFSVAISSILGKTMTVLVAFKSAKMANRFRKWGKINI
SVVIVFVCSFVELVICVIWLTMSPPYVESDIKTTPGKIILQCNEGSTISFYLAVSYIGVLSLISF
AVSFIARKLPDRFNEAQHITFSMLVFCSVWASFIPTYLSTKGKHMVAVEIFAIQASAAGLLM
CIFTPKCYIILLKPELNVKANLTFKINPKIMNK*
>jgi|Xentr4|189065|fgenesh1_pg.C_scaff...
MLGAVAFLIRIYLTALRAISCDSQIQTKSLACQLEIIKTYEDYEYIKEGDIIIAGVLTVNTLMV
SLSYPIEFYDIMICVGVLAKNYRQFVDFRFAVEQINNNSAKLPNLTLGYHIYDSCGDPRKA
VRSVLQILSGTREPVPNYSCVGKRNIAGFIGDAASETTVPIAQILNVYGYAQISYGATDPSL
SDRISFPYFFRTLQSDHSHYLALSKLLRYFEWTWIGIITFDDDSGEKEFQLLTNYLSMEGIC
VEFTIKIGNYVDNIAMYEIYSEIIQKSSTSVIILCGTASVNFAETFRNMTDMFLTKTLIVSSTV
AANHNSMDYYLEIFHGSLGFVQHSEYSLDTPEMRSFVESFHPLRYPDDKLLEDIWMQCHL
CLSEDPSKNDLHEKLYASKLHNCTGQDNITNLLGIESKLHFPHVHFAIPRAQCSDNCPPGFR
KATKPGAQSCCYDCVPCPAGDISTTIDSEICTRCPDKEWPNENKNQCITKTEEFLTFDNNM
SLIFLSISVLIFLVTLLILGVFITYQDTPIVRANNRSLSFLLLVSIKLSFLSVFLFLGRPVDITCM
LRIITFGITFSIAVSSLLAKTIMVCVAFKATKPGSSWRKWLGVKLSNSVVLFCSSIQIIICMTW
LAISPPFQELDIHTSPGTIIIQCNEGSAIGFYSVIGYMGLLAAVSFVLAFLARSLPDSFNEAKY
ITFSMLLFCSVWITMIPAYLSTKGKNTVCVEIFAIITSSAGLLACIFLPKCYIILFRPEMNTKS
HLLGNRIEYAV*
>jgi|Xentr4|188049|fgenesh1_pg.C_scaff...
MNEKNQTWVSEIVLLGFQNLHNFKVPLFSLFLLIYILTVWENVLIIVLVAFSRNLHSPMYFF
LQQLSLSDLLESSNIVPTLLRSVINREAILSFVGCVTQYSLFCAPEGFECLLLAVMAYDRYV
AICIPLRYTSIMSHRVCVKITLMLWAVGFGLAVITVNLIGTLQFCDQNTINHFFCDLLPLLEL
SCSDTFLVQLELMIVTIPVIICPFILISVSYMCIAHAILKIVSHTGRQKAFSTCSSHLAVVSMF
YGTLIAIYVVPPRNQSQTTSKVLSLLLTIVIPLFNPIIYSLRNKDMNGALIGDMNPCFAQCSH
R*
>jgi|Xentr4|189154|fgenesh1_pg.C_scaff...
MSNLSGANPISSPFILVGIPGMEEMHPWISVPLCCMYIVTISANISVLLIIRADRRLHQPMYL
LLSMLLLTDLVQSNAALPKMLLIFWFKLREITFEECLVQMFFIHSFSIMGSSVLLTMAFDRY
VAICHPLRYSTILTNSMIAKMGLFVTIRGTLLAIPYPLLVRRLPFCGNHIIQQTYCEHMVVAK
LSCGDIRINFVYGLLVASLVVGLDIICLSGSYTMIIMAVLRLPNREARYKVGNTCSAHVGVI
LVLYIPALFSFISQRFGASSAPSVQILVSSLYLIIPPMCNPIIYGIKTKEIRKKVSKLFSLAEMVL
*
>jgi|Xentr4|189209|fgenesh1_pg.C_scaff...
MDSKNQTVPKDFILLGLSNNPRVKRFLLIFFCFVYSTTLTSNCSLIILTSVIRELHSPMYMFIC
CLSLLDITFTSVTVPKMLVGLLSERCHISYNGCFTQLYFYHFLGCAECLLLSIMGYDRYVAI
CHPLRYPQIMNKNACIRLASSCWITGIIYSSVHTILTASLSFCRSREVSHFFCDMPPLLKISCL
DTTLNEIVILSLGGVVAGASFLLTVISYIYIISAILKIRSARGRKKAFSTCAAHLTVVSIFYGTI
ILMYLRPQSSHSLQHDKMLSVFHNVITPMLNPLIYSLRNKQRNKDVKDSIRKLFRKKILGL
RK*

>jgi|Xentr4|189456|fgenesh1_pg.C_scaff...
MENQTIVYVLVLAGLSDLPSLQLPLFLVFLLIYLITLTGNLLILLLIFTDSHLHTPMYFFLGTL
ACMDLCSSSVTVPRMLFDLLRDTRFISLPACITQFYFFIFFALSEVSVLAVMSYDRYIAICRP
LHYMQIMSWKVCVDLVLIVFVFSAVCSSVHTLFLAKLTFCRSEALQSFFCDLPQLLQISCS
DTFINVLLIFLLGIMFGLGILTVTFLPYIAILTTILKISSKHTRSKAFSTCSSHLTVVFIFYTTILF
NYFHSSVNGHFTEDKVSSVFYTILVPFLNPLIYSLRNQELKISLRKTLQRLQIKKLQ*
>jgi|Xentr4|190057|fgenesh1_pg.C_scaff...
MTSGNQGNVSGFIIQGFSDTPELQISLFVLFLGIYLIILLGNLIIFLVISCNPHLHTPMYIFLLN
LSLIDISSTSTVLPNLLHILLTQQNNISFLGCMTQMYVFASLACSELLLLTAMAYDRYVAICD
PLHYIARMSRKHCAGLITAAFTVGFGEPIGLIVLVPKLSYCASHLINHFFCDVTPLLKLSCSS
TFSVELSIYIEGILLSFNAFLLTLASYIFIISAILKIQSSEGRQKAFSTCASHLACVITLYGTVIC
LYVRPTTSYSLKRDKPTTSYSIKRDKYFSLLYIVLGPVLNPLIYTLKNRQFQSSLNKMRKR*
>jgi|Xentr4|190336|fgenesh1_pg.C_scaff...
MDGENQTTIQSILLLGFQMPPHFKIPIFILIFLLYSLTLTGNLLIISLIYGSPTLQHPMFFFLTHL
SLSDICLTTDIVPNMLYVIKSGPISITMAACMTQFQFFGTVVTFECLLLAVMSYDRQLAICN
PLRYASIMGNQLPLWLVIVCWSVRTFAISRLNFCGPNIIDHFFCDFFPILQLSCSDTSAVELEQ
ILIAVPTVVMPVVFIVGTYICIFIAVLKIPSTSGRQKAFSTFSSHLSVVCIFFGSLICLYVAPPSG
NFLSTNKVVSVLYTVVTPLSNSIIYSLRNSELRSAFKKHVNIYNICKMSKLS*
>jgi|Xentr4|190357|fgenesh1_pg.C_scaff...
MNPLTGLVPPLLFLLCLVPHGSAYGPDKRAQKKGDIILGGLFPIHFGVASKDEDLESRPESL
ECIQFPRLPLVTGNDLCHRGNQQLPYAAPQHHSGATLSFVAQNKIDSLNLDEFCNCSEHMP
STIAVVGATGSGVSTAVANLLGLCTWVSYASSSRLLSNKNQYKSFLRTMPNDEHQATGMA
DIIEYFQWNWVGTIAADDDYGRPGIEKFREEAEEREICFDFSELISQYMEEEEIDRVASVIQ
NSTARVIVVFSSGPDLEPLIKEIVKRNITGRIWLASEAWASSSLIALPNFFDVMGGTIGFALR
AGQIPGFREYLRNVNVKMSNVNGFLKEFWEETFNCHLPSKSLSSPSSFMGSPEDPNRWGN
SSSFRLPCSGKENISSVETPYLDFTHLRISYNVYLAVYSIAYALQDIYSCTPGKGLFANNSCA
DIKKVEAWQLLKHLRHLNFTNNMGEQVDFNDSGDLVGNYSIINWHLSQEDDSIVFEEVG
YYNVYAKVGERLFINESKILWNGFSREVDASACEKCAKDYWSNDNHTSCIAKQIEFLSWT
EPFGIALTMFAILGICLTSFVLGVFIKFRNTPIVKATNRELSYLLLFSLICCFSSSLIFLGEPQD
WTCRLRQPAFGISFVLCISCILVKTNRILLVFEAKIPTSFQRKWWGLNLQFLLVFLCTFVQIV
TCIIWLYTAPPWSYRNQDLEDEIIFITCNEGSLMALGFLIGYTCLLAAICFFFAFKARKLPEN
FNEAKFITFSMLIFFIVWISFIPAYASTYGKFVSAVEVIAILASSFGLLACIFFNKVYIILFKPSR
NTIEEVRCSTVAHAFKVAARATLRRSNVSHKRSNSLGGSTGSTPSSSISSKSNHDEHFSIGD
KNRACKQKVSFGSGTVTLSLSFDETQRNAVSNRTTRAKNSLEGKNSDDSLMRHRALLPLQ
NSDAGSESSFRTASNIDEAGSLESVKPESADLPQMGNSKSVTDGTITENAVNS*
>jgi|Xentr4|190430|fgenesh1_pg.C_scaff...
MAHQYEAGLWTLWPGLWRWRCWFAIEDINRSSWILPNVTLGYQIFDSCAAPPKALSGAL
DIFSGRNYRIPNFACLKNMKLIGLLGDLSSASSYPIAQLAGLFRLVSYGDTDPVYNDRIQFP
SFYRTVPNELSEMDGIVQILKHFGWKWVGLIVSSSESGERALDRIRKGIESYGGCVAFSISL
TDTFYIFFFGIESFIAKINKKGVDVIVLFLNSANLKAFTKLFSSRQITRKIWLSSSFFPAFIPLI
HGNKKTTLNGTLSLSAQGGEIPGFETFLYRMTPINYPNDDTVTEIWETLHGCSFTGFLKTN
TSVPGQKCSGNESLDDEALSRFGNFDFRVGYQVYTAVYALAHSLHNLFSARAPAHPQAPA
GSLKHRFKPWQVSYVFPAPSNYSTDIDLFKKMLDITLDVNAVTCVKCPEDQKSNRQKTDC
VPKALNYLSYMDTLGASLASTAIILFITASVVLGIFVKYWETPIVRANNQNLSCLLLISLML

CFLCTLLFIGRPTQICCLLRQVTFGIVFTISVSSVLAKTLTVIIAFNATKPGSKLKKYVGTQL
AIILVIVCSLVEIVISAVWLASNPPFPEADTLSDPDYIILLCNEGSELFFFCTIGYIGTLALLSFI
AAFLAKDFPDRFNEAKNITFSMLGFCSMWGAFVPAYLSSKGSRMVAVEIFAILSSSAGLLG
CIFAPKCYIIFLRPELNIREIVGRKA*
>jgi|Xentr4|190801|fgenesh1_pg.C_scaff...
MFHRATSSPLASYGSPSYFFSPCLLCSTELLLLPLPPTYLPLNGLFAFFSPIFVLSRYTKMKTA
TNIYIFNLALADALVLVTLPFQGTDIFMGIWPFGNVLCKIAIAIDYYNMFTSTFTLTMMSVD
RYIAICHPVKALDIRTPHKAKVVNVCIWILASFIGIPVMVMGSAEMENDEIECLVQIPPPEH
YWDPVFGICVFLFSFVIPVLIITICYSLMIRRLKKVRVLSGSKEKDRNLRRITRLVLVVVAVF
VICWTPIQIFVLVQSLGAKPDSDIKVAVLHFCIALGYVNSSLNPVLYAFLDENFKACFKKFC
FPSAFRPELQMSNRMCSIAKDVAYACKNSDGPNNPA*
>jgi|Xentr4|190836|fgenesh1_pg.C_scaff...
MENQTIVAEFTLMGISHLPKLEHQLFFMFLLTYLMTMSGNLLILVLILTDNHLHTPMYLFL
GHLAGVDICYSQVTCPRMLLDFYSEKKTISYRSCLTQAFFFLCFVSCECFLLAVMSYDRYV
AVCQPLQYIQIMSWKRCTQLLTLIWTLGSSYTLVEILFTLRLTFCGPSIIHNFFCDLPQLLTLS
CTDPSLNMLLLFVLGGILSFIVFFITFLPYVYIFYTVHRIRSKNPTLKAFSTCMSHLTVVCIFY
GTLCFAYLRPRSRYLDADVVVSVVYAVISPLLNPIIYSLRNGEIKRALGRVKYLLITIYME*
>jgi|Xentr4|191112|fgenesh1_pg.C_scaff...
MPSENEGNNQGNVSGFLIQGFSDNPEVQLSLFLFLYLLILLLNLTVILVISLHSHLHTPMYLF
LLNLSWNDICSTTNIFPNFLHTLFTQKKNVSFLGCMAQLYFFMFSVVIEYTTLTIMAYDRYV
AICDPLHYITQMTLKHCAQLVTASLAVSFLVPTGHAVLISRLSYCGSHVIEHFFCDVIPLLKL
SCSDTFNVELLTYIEGTLVAFSCFLFTLISYICIISSILKIQSLEGRKKAFSTCASHLTSVIIFHGT
LISVYYVRPTTSYVPNRDTFFAFLYIVLIPLLNPLIYTLKNREFQSAFIKLYSDGRMGLHDVEG
*
>jgi|Xentr4|191415|fgenesh1_pg.C_scaff...
MYEKNQTWVSEIVLLGFQNLKNFKVPLFSLFLLIYIMTVCENVLIIVLVSSSRNLQSPMNSF
LQQLALTDLLGSSNIVPTLLQTIIYDGVTVSLHSCIIVFNLFACSESMQCFLLAVMAYDRYV
AICNPLRYTSIMNHRVCVRLILMSWALSIVITINTWSMIQTLRFCNETTIDHFFCDLTPLLEL
SCSNTFFVKLEITLQSVVSLFPPLTLIIGSYICIAREILKIVSNTGRQKAFSTCSSHLAVVSIFY
GTLIGVYVVPPIKQSLTTSKVLSLLYTVVIPLVNPIIYSLRNKDMKDALKNELVNGPLAEIFC
S*
>jgi|Xentr4|191686|fgenesh1_pg.C_scaff...
MASGSQENVSGFIIQGFSDTPELHISLFVLFLGIYLTILLGNLIIFLVISCNPHLHTPMYIFLLN
LSLIDISSTSNILPNLLHILLTQQNNISFLGCMAQVFVFVFLAGSEYFLLTAMAYDRYVAICD
PLHYIARMSRKHCAGLITAAFTVGFGATINFIVLISKLSYCASRLINHFYCDITPLLKLSCSST
FSVELLMYIEGTLLTFNSFLLTLTSYIFIISAILKIQSSEGRQKAFSTCASHLACVITLYGTVFC
LYMRPTTSYSTKRDKYFSLLYIALGPVLNPFIYTLKNREFQSSLNKMQQIGIPFLTLVI*
>jgi|Xentr4|191904|fgenesh1_pg.C_scaff...
MASENKENISGFIIQGFSDTPELHISLFVLFLGIYLMIPLLGNLIIFLVISCNPHLHTPMYIFLL
NLSLIDISFPSTVLPNLLLILLTQQNNISFLGCMTQMYVFASLAASEYFLLTAMAYDRYVAIC
DPLHYIARMSRKHCAGLITAAFTVGFGESFGLIVLISKLSYCASRLINHFFCDITPLLKLSCS
STFSVELLIYITGTLLIVNSFPLTLISYIFIISAILKIQSSEGRQKAFSTCASHLACVITLYGTALS
LYMRPTTSYSPKRDKYFSLLYIALGPVLNPLIYTLKNREFQSSLNKMRQRLLAFSFCKSVV
KKKLKKTPFIKI*

>jgi|Xentr4|192334|fgenesh1_pg.C_scaff...
MTSESKENVSGFIIQGFSDTPELQISLFVLFLGIYLIILLGNLIIFLVISCNPHLHTPMYIFLLNL
SLIDISFSSNVLPNLLHILLTQQNNISFLGCMTQVYLFASLAASEYFLLTAMAYDRYVAICDP
LHYIARMSRKHCAGLITAAFTVGFGAAVSFVFLMSKLSYCASHLINHFFCDATPLLKLSCS
STFSAELFIYIEGTLLLFNCFLPTLISYIFIISAILKIQSSEGRQKAFSTCASHLACVITLYGTVI
CLYLRPTTSYSIKRDKYFSLLYIALGPVLNPLIYTLKNREFLSSLNKMKQRCSLPMLTHQAH
TGRM*
>jgi|Xentr4|192342|fgenesh1_pg.C_scaff...
LETCIALKMCLILVGKRLKLGMMPKSSAQKDFIAVCSQILEYEMPNLWYNIHYEDPAIGSK
FTQSLENMLKVLEPQTKDLQIIAPNIELNVLVLESSTLASYSTLNVNPRVQMFFKSTALQEL
TQTGNLTMASLAINKLSKLLANNYGDSLKGSDHAIKDLILMNVFNINQDGIDVIFERASDS
NKTKVDLSRCVLWDYNLFDGNGGWSAEECVTYNKDNLTVCSCSHQMSLSLVTFIPTAQE
ECRWKSLSLFSRSTSIASLTLSLIIYLVEWKFIVKTLISFCHQLSAVNIALCWLIADLCYLGSS
CIMSTTPTPDLCIAAAFFKHLFYTAASFWMLFEGLFLFQQLVLGFLKLQKKALVVIMLAIG
YLCPLIIALVTLSLYHPSGTYINADTCFLNVDNGAINTFSAPVLLISFMNIFVVIVGIWKLMK
PSQSEKSEEGGEGSAKERLEESAEGSTIESAEESAKQRSEERAKERLEERSEESAEEQAKER
MEERSEETIEGGDDKEDLIAIFKPLLILTTIFALTWVLQLAMSVMCGVQGFLDFISTLLLNSFQ
LPYGQKGLGGHIAATKMASRLNPTTSIPELGVAERKVELLQSGSEQQMEILGKMENLKESP
CNLLLNPMYHVILFVHYYNLPGNKLNGCTEPVLEVQVPTPLVSYRSLGVQETWNELLQS*
>jgi|Xentr4|192833|fgenesh1_pg.C_scaff...
MNTMRTCNQSKITEFLLVGFSAPRPLRELLFSICLVIYIMALGANLMIIALYLGSHHLRSPM
YFFLNGNPMSVSACVTQFFAYTFFTGVECLLLTVMAYDRYLAICKPLHYVTIMTNKHCLH
LVIWCWFEMLLLSFPGTILTSILGFCGCNTLDHVYCDFAPLLKASCSDAFVMGSMTSIVIPA
GMVLVFLSPLLFIITTYVSISLSIFRISSKTGRQKAFSTYGSHLTVVCTYYGILISKYAVPSKG
QSGNLNKLTSLLYTLVTPLFNPIVYSLRNKDIQKTMTTLISVRP*
>jgi|Xentr4|195873|fgenesh1_pg.C_scaff...
MDKANKTTVEEFILLAFSDLFQLQIILFIVTLLVYIMCVFGNCLIVIIVRAEPSLHTPMYFFIS
TLSTLEIMLVSTILPNLLANLIAAKKSISFNGCFIQLSVSGTLGTAECYLLAVMAFDRYLAIN
KPLQYVSIMTQRLCVQLALLPFIVGSVCTLIPVVFTVILEFCGPNEINHFFCDVAPLQALACS
SLYTIHMVTNFVAIFASGLPFAITVGLYIYIIVIISKINSRESKRKAFSTLSSHLTVAGLFYITAII
VYMVLKGTQYDRFFALINTVITPLLNPFIYTFRNKDVKRALIKSRSASLHLLREWE*
>jgi|Xentr4|195938|fgenesh1_pg.C_scaff...
MESMGDRLSVISVPYLRQGLEKSVSKKPLSPTFCSHLGNAFVVSLALADLVVALYPYPLVL
VAIFKNEWAMGEMHCKVSGFVMGLSVIGSIFNITGIAINRYCYICHSFVYDKLFSLWNTILY
VCLIWTLTVVATVPNFFVGSLEYDPRIYSCTFVQTVSSSYTITVVVIHFIVPITVVTFCYLRI
WILVIQVRRKVKSEIKPRMKQSDFRNFLTMFVVFVIFAFCWAPLNVIGLAVSISPTEVAPKIP
EWLFVLSYFMAYFNSCLNAIIYGLLNQNFRKEYKRILMSLWMPRLFFQDISRGGTECPKSK
PSPALNNNDQVKTDTV*
>jgi|Xentr4|196832|fgenesh1_pm.C_scaff...
MNVCHYDKRMDFFYNRSNSQTEDDWSGTKLIIVLCFGACCCFFIFVSNLLVIAAVVINKRF
HYPFYYLLANLAAADFFAGIAYVFLMFHTGPVSKTLTVNRWFLRQGLLDTSMSASLANLL
VIAVERHISIVRMRIHSNLTKRRVTFLIFLIWAIAIFMGAVPTLGWNCICNITTCSSLAPIYSRS
YLTFWTVSNLAVFVIMLVVYLRIYMYVQRKTNVLSPHTTGSINRRRTPIKLMKTVMAVLG
AFVICWTPGMVVLLLDGLNCTKCGVQHVKRWFLLLALMNSIMNPIIYSYKDEEMWRTFK

KMICCLLYKRRGHRPSRMSSTVHSTRSNDTTSHYLEEANNHSPTLKNI*
>jgi|Xentr4|196935|fgenesh1_pm.C_scaff...
MYSLDEDFYARNFTYPFNYSDYEFDDDDVTKTRTFYAAKIVIGVALVCIMLICGIGNFIFIA
ALARYKKLRNLTNLLIANLAISDFIVAIVCCPFEMDYYVVKQLSWEHGHVLCASVNYLRT
VSLYVSTNALLAIAVDRYLAIVHPLKPRMNYQTASFLIGSVWIVSLLIAIPSAYFATESVLFM
VKNQEKIFCGQIWPVDQQIYYKSYFLFIFAIEFVGPVLTMTLCYARISRELWFKTVPGFQTE
QIRKRLRCRRKTVLVLMCILTAYVLCWAPFYGFAIVRDFFPTIFVKEKHYLSAFYIVECIAM
SNSMINTMCFVTVKNNTMKYFKKIMLLRWRSTYSGSKGSAELDLKTSGVPVTEEVDCIRL
K*
>jgi|Xentr4|197268|fgenesh1_pm.C_scaff...
MEFANKTIVTEFILLGFSGIENLKIFLFITFLLIYIFTVMGNMVILAIVYFDSHLHTPMYFFLT
NLSLLEILYTSNIIPRMLLNIITENQAISFTWCIVQLYFFGSLGSTECILLGVMAYDRYIAICYP
LHYSLLMNTKASLRMSFSSWVVGFTATFGAIILVSQLWFCGPNEIKHFYCDLRPVLQLSCK
NPFIMETVSVILASTILLCTCLFTLASYIHIILTILKIPSSEGRQRAFSTCGAHLTVVIIFYGAMI
FMYVTPATVNSFNFNKVLALLYTVMTPLMNPFIYTLRNKEGLRSCLKQKKFLAEAMV*
>jgi|Xentr4|197476|fgenesh1_pm.C_scaff...
MQNRSMVTEIFLLGFQHLNNFKTLTFALILFVHILTVYENALVIALVTVSRGLQSPMFFFLQ
QLSFSDLLLSVLIVPTLLRTVMNEGINISLIGCIVQLYFFSITEALQCFLLTVMSYDRYLAICN
PLRYSSIMNHTFSFRLIAMSWLLCLSCTLVTVISAATQEFCNQNTINHFFCDYFPLLELSCSD
TSLAQILAMATAIPVILIPFMLIIGSYICIAHEILKIVSSIGRQKAFSTCSSHLAVVSIFYGTLIV
TYVVPTRNQSQTISKLLSLLYTVVTPFINPMIYSLKSADMKNALENIIQ*
>jgi|Xentr4|197482|fgenesh1_pm.C_scaff...
MCTDNETEVTEIILLGFHGLNNSKTPLFILFLLIYSVILYGNSIIIFLVTFNEHLQIPMFFFLKH
LGLADVLLTTNIVPMMLHVIVNDELVITLIGCICQLYLFGISGFVQCFLLAVMSYDRYLAVC
YPLHYISIMSPNVCLQLVVGCWLLVFIFITTGLILVCQLHFCGFNQIDHFFCDFGPVVALSTS
DTTVVTMYDFISSIPYISVPLIFIIGTYVHIFITILKMNSKTGRQKTFSTCSSHLTVVCTYYGT
QMIVYLGPTGDNYANMKKFLSLLYVVIIPFMNPIIYSLSNKEIRETLKKYIRLM*
>jgi|Xentr4|197512|fgenesh1_pm.C_scaff...
MHKVNQTSGISFLLLGFQNSQIINEFLFVLFLWIYIVTLFGNLLIIILVITVSALRSPMYALLS
QLSLSDVLLSTSITPNILWLLLNGGGTISPTGCITQFYFYCVSGASEFYLLTAMAYDRYLAIC
SPLHYASIMGFRLCLYMSLCSWGLAFILSLVMNLLTFNLQFCGPFVIDHYFCDFTPLIKLSC
TDYKAVELTDLILTIPFMLLPFYFIIYTYVAIGLAILRISSTEGRHKAFSTCSSHLIVVCMYYG
SMIIVYMVPSKGHNFNINKMLSLLYTVGTPCFNPMVYSLRNNDIKVALWKHIASLFRLHLI
NRKGD*
>jgi|Xentr4|197759|fgenesh1_pm.C_scaff...
MPEGYLNLNETLSSVTRNTAHLRFSNGSDALYQEESADLSPRLEVLATISITYAVIISVGLLG
NTILIKVFFKVKSMQTVPNIFITSLAFGDLLLLLTCVPVDASRYMVDTWLFGRMGCKILSFI
QLTSVGVSVFTLTVLSADRYRAIVKPLELQTSDAVLKTCGKAACVWIISMLLAAPEAVFSD
LYEFSSPDKNTSFKACAPYPVSEKLMQETHSLMCFLVFYIVPLSIISAYYILIAKTLYKSTFN
MPAEEHTHARKQIESRKRVAKTVLVLVALFAFCWLPNHILYLYRSFTYHSAVNSSMLHLSA
TIFARILAFSNSCVNPFALYWLSRSFRQHFKKQVYCCPEQPLQSQPSPTHSSTTTGIAATKG
NMQMSEISFTLLSGYEIKKESDSISLNDSR*
>jgi|Xentr4|197894|fgenesh1_pm.C_scaff...
MNILVNNTVSDFYILAFRVSGSFQILLFISILVMYLLAILGNMMIIVLVCLVSHLQTPMYFFL

CNLAAQDIVFVSSTLPKLMAITATGDTSISFGGCFTQMSSFTFCVITEILLLTFMAYDRYVAI
CIPLRYFIIMSKRACTLFAALSWLLGGFNSVFYFCIMYNLPFCYTQEINHFYCDLKAMIKIS
CGDITHLHTLMSFESVFLGLLPFALILVSYTYIIYNILNIRTSAGRAKTFSRCSSHLTIVIIFCTT
LISTYLKPQSETSEELEKLMSLLYTAVVPMLNPLIYSLRNKDVLKAMKNILSLLIYPPSIYSR
RPPTKKKEQCDPISCISKG*
>jgi|Xentr4|197965|fgenesh1_pm.C_scaff...
MNVTDSNILDTTNDYIYSESTETFENSRTFTFHFLPVLYCLAFTFGLVGNILVIFVLVYCKKL
RTTTDVYLFNMAVSDLLFVVSLPFIAYTIINEWIFGNIMCKILSTIYFVGFFSSIFFITVMSVD
RYFAIVHVVFALRVRNVRWGLIVSIVVWVLALSISTPNFKFHEIVMTGNYTECVLSYPEINR
QNWKIFCSLLINIFGLVIPLFILLFSYLHIIKTLQNSKCKQKRCAIRLILIVGIVFFIFWTPYNIV
VFLNILKTSGAVDLETDQLQTAADVTHTLSLVHCCLNPIIYTFAGEKFRGYLRLIINRPLNFL
AYHTLSASSPV*
>jgi|Xentr4|198170|fgenesh1_pm.C_scaff...
MHKVNQTSGISFLLLGFQNSQIINEFLFVLFLWIYIVTLFGNLLIIILVITVSALRSPMYALLS
QLSLADVLLSTSITPNFLRLLLNGGGTISATGCITQFYFFCVAVGSEFYLLTAMAYDRYLAIC
SPLHYASIMGFRLCLYMSLCSWGLALMLGLFINLLTLNFQFCGPFVIDHYFCDFTSLIKLSC
SDYKAVELTVIILAIPFMLLPFCFIIYTYVAIGLAILRISSTEGRHKAFSTCSSHLIVVCMYYG
TLIIVYMVPSKQYMFNINQMLSLLYTVGTPCFNPIVYSLRNNDIRVALRKYMSNQVSFYTT
FKQQKRRSQLLC*
>jgi|Xentr4|198269|fgenesh1_pm.C_scaff...
MQSEVNLTVRDDISTNMYQTLPYTLSFQISLTCFLMLEIVLGLGSNLTVLVLYCMKSNLINS
VSNIITMNLHVLDVIICVGCIPLTIVILLLPMESNNALICCFHEACVSFSSVATAVNVFAITLD
RYDISVKPASRILTMGRAVILMTTTWIVSFLSFLIPFIEVNFFSVQSESKWENKTLLCVSRNE
YHTELGMYYHLLVQIPIFFFTIIVMLITYSKILQALNIRIGTRFSTVQKKKARKKKTISLATQ
HETTDVSQSSGGRNVVLGVRTSVSVIIALRRAVKRHRERRERQKRVFRMSLLIISTFLFCW
TPISILNTTILCLGPSDLLVKLRLCFLVMAYGTTIFHPLLYAFTRQKFQKVLKSKMKKRVVSI
VEADPMPNNAVIHNSWIEPKRNKKITFEDKEARQKCLATQAATD*
>jgi|Xentr4|198739|fgenesh1_pm.C_scaff...
MAGWNNTHFQEFILLGLTDNPKLQIILFVLFFLFYIVTLLGNIGIIVAVRLDSRLHKPMYFFL
NNLSFLDLCFSTTITPKTLVTFLSETKTISYYECALQMYFFAASATIECFLLGVMAYDRYVAI
CKPLLYSVIMSKRLCVQLVLASYILGYLNATLHTICTFRLPFCKTNKIDHFYCDVPPLLKLS
CTKTTMNEILMFIFGGFAETSSLTTIMVSYSYIISTILRIRSSDGRKKAFSTCASHLVAVTIFYS
TVLFMYLRPASAYTMSQDRIASVFYSVLIPMLNPLIYSLRNNEVAQALRKIKTNYCCKYNG
SVK*
>jgi|Xentr4|197487|fgenesh1_pm.C_scaff...
MIQEFILVGLKDLHHFQTPVFVLVIIVYVATILGNMLIIVLVLTNHHFKSPMYFFLSHLSLCDI
LISTNVTPNTLQVILRGESAISVNECLTQLYFFGSSAIFECCLLSIMSYDRYLAICNPLLYSSI
MNHNFPCYLVLWPWLAGFLLALITNVLVFELYFCGPNIINHFFCDLAPILELSCSDTSAVEIH
VSIAVVVTVISQMLFIIATYICIFNSILNISSKIGRQKAFSTCSSHLVVVSTYYGTLIALYLAPS
RGYSLTLNKILSILNTVVTPLFNPIIYSLRNKEIRTETVKFVSKINNLIQY*
>jgi|Xentr4|199320|fgenesh1_pm.C_scaff...
MPANYSRIDEFILLGFSNVSQRFQLFLFLLFLFIYIFTLLGNITIVSVVTLDSVLHTPMYYFLR
NLSFLEICYVTVTLPKLLQTFLAKRKAISFSGCCAQMYCFFTLGVTECFLLAVMAYDRYVA
ICNPLRYFTVMSKATCLKLATISWVSGNLISLGQTVSIFSLPYCGPNIINHFFCDIPPVLKLAC

TDVSANEIAVAVTGFLVLPLPFVLVLCSYGRIIAAIIRISTSKGRKKVFSTCASHFVSVTLFFG
TGAFTYVRMKTRDTADNDKILSLLYSVITPLLNPLIYTLRNKEVKGGLKKLLPCKTNGKET
QE*
>jgi|Xentr4|199335|fgenesh1_pm.C_scaff...
MPGKNQKENITLGDGFILLGLVTEPFLQKFLFVLFGTTYIITIAGNVVIIIVTRLDIRLKSPMY
FFLVNLALIEMLYTTSIIPNTLKNLIDEEKYISYAGCFIQMFTYIAMGGSECTLLSTMAYDRY
VAICHPLLYTTIMSQSLCFYMTLACWTIGFLNSLIHTVLTSMLPFCHQRLLNHYFCEVPPLL
KISCKDTYINELVVFFIGGFVIVGSLALTLISYIFVIAEVLKIPGLSGKWKTFSTCSSHLTVVSI
FFGTVIFTYLRPTTHSSIDHDHVISLVYGVVTPLINPIIYTFRNKEFQRAFRMILQTRHCLFD
HIKPNSKTEI*
>jgi|Xentr4|199336|fgenesh1_pm.C_scaff...
MVINNSRNVDGFMLLGLGARPALQNLIFVLFFVAYIATLFGNFMVIVIIRQDFRLQTPMYFF
LVNLSVLEILYTTTVIPFTLRMLLDKNKRISFAGCFTQMFIFISLGGSECILLGTMAYDRYVAI
CHPLRYTTIMKPSACYCLAMICWSTGFLNSLVHTVLTVMLPFCHDRKIKHYFCEIPSILKLS
CKDTKVNEMLIFFMGGSMTVGSLTLTLVSYACIIAAVLKIPSIIGRHKAFSTCASHLVVVSIF
FGTVIFIYILPNSQSLSDQNQVISVVYSVLTPLFNPIIYSLRNHEFQRAVVKVLQM*
>jgi|Xentr4|199410|fgenesh1_pm.C_scaff...
MNFTHHHEPHHLHYRNHSRTVGAGANDTKEKGHHSGGCYEQLFVSPEVFVILGIVSLME
NILVIAAISRNKNLHSPMYFFICSLAVADMLVSVSNGFETVVITLFNTTDKNTQHIIVNVDNI
LDSVICSSLLASICSLLSIAVDRYFTIFYALQYHNIITVRRAVVIISCIWTACSISGVLFIIYYDS
AVVIICLISIIFTMLALMASLYVHMFMLARLHIKRIAVLPGTNSVRQVTNMKGAITLTILLGV
FVACWSPFFLHLIFYVSCPRNPYCVCFMSHFNIYLILIMCNSVIDPLIYALRSQELRKTFKEM
MCCYCMGGIWDFSSSY*
>jgi|Xentr4|199755|fgenesh1_pm.C_scaff...
MDLEIEDVNNRTVTKDENSTSETRDSSFPEWEDYNSSVDDIRYFLIGLYTFVSLLGFMGNL
LILLAIFKKRKQKTIINYLIGNLAFSDILVVLFCSPFTLTCVLLDQWIFGEVMCHVVPFLQCV
SVLISTLMLMSIALVRYHMIKHPLSTNLTANHGYFLIATVWTLGFTICSPLPVFHKIINLREA
FNLDSLINSYLCIESWPSDSYRIAFTISLLLVQYILPLSCLTISHTSVCRSIGSRLPSRDTKVEE
NEMINLTLQQPKNHRSELEISSSRRWSYSCVRKHRRRYSKKSASVVPAILRRNQESNTGDIP
ETSMTERSHTLPSSVYLPGVPICFEMKPEENSEMHNMISVSKSITRIRKRSRRVFCRLTILIIA
FAVSWMPLHLFHLVTDFNANLISNRHFKLVYCICHLLGMVSCCLNPILYGFLNDGIKADLM
SLIKCFQIS*
>jgi|Xentr4|200142|fgenesh1_pm.C_scaff...
MTNTTCCNFEVRFLSYLLPPVLILVFVLGSLFNGLSLWIFCFHNKSWKASTVWLFNLALAD
FLLMVCLPFRTDYYLRHKHWQYGDIPCRLVLFVLSMSRSGSIFFLTLVATDRYFRVVHPHH
RINSVSTMCAAGIACVVWLITIAGSVFILTKRREFSGGREGFLACESFAICLDYSEKQHDYI
YLVQFFVPLATVAFCSCRVILRLRQRNLDRHAKIKRAVQCMVLVGMAFCFCFLPSVSTRIE
MLRLLDSPQWQDCNIYRGADTAFYISICLTYMNSMCNPLLYYFSSPSFQAFYLKIAKCCLT
GGPHQARGPPPVWLL*
>jgi|Xentr4|200395|fgenesh1_pm.C_scaff...
MLPKGIVGNQSEVIEFIVQCFSNAPELQVPLFVLFFLIYFIILTGNITICTTVTVDPHLHTPMY
WFLSNFSILEILHTSAILPKLLTMLITQHKTISTTGCKMQLFFFMAFTCIEFFLLTVMAYDRYI
AVCLPLRYHSLMNPQLGAKYIAAVWTTGLLDPVPHNAVISQFSFCSSNQIDHFYCDITPVL
KLTCSDTSTVELLIFLNGTFLLLSTFTFTCISYILIIQTILGIKSSEGQKKAFSTCASHLTCVSIF

YGTIYCLYMRPTTAYDPEKDKFLSLLNIVLVPMLNPFIYTLKNKEFKIAFAKQKKRIFSWGQ
S*
>jgi|Xentr4|200400|fgenesh1_pm.C_scaff...
MTSGNVSGFIVQGFSDTPELQISLFVLFLGIYLIILLGNLIIFLVISCNPHLHTPMYIFLLNLSLI
DISSTSNILPNLLHILLTQQNNISFLGCMAQMYVFASLVGSEYFLLTAMAYDRYVAICDPLH
YIARMSRKHCAGLITAAFTVGFGATVGFFVLISKLSYCASRLINHFFCDMAPLLKLSCSSTF
SVELLIYIEGTLLTFNSFLLTLTSYIFIISAILKIQSSEGRQKAFSTCASHLACVITLYGTAFCLY
MRPTTSYSLKRDKYFSLLYISLGPVLNPFIYTLKNREFLCSLNKMTQRCHQMMAVNKTSV
*
>jgi|Xentr4|200776|fgenesh1_pm.C_scaff...
MQIQYCYESINRSCEKNGWSNNIRIPMYVFMVSTILATVVGNLAVIISIAHFKQLHTPTNYL
ILSMSTVDFLLGSCVMPYSMVRSVENCWYFGDLFCKVHTSTDIMLSTSSIFHLSFISVDRYF
AVCDPLRYKTKMNVFSVSLMIVTSWVVPTIFAFTMVFLELNIKGTESYYYNQVSCFGGCA
VFFSQTSGLVASMVSFYIPGFVMLCVYGKIYVIARRQARSIKDSANQMPFQITLGVNQHVS
RSRDRKAAKTLGIIMGVFLICWTPFFFCTATDGFINYVIPPIVIDAFVWIGYLNSTFNPMVYA
FFYLWFQRALRMILFGKVFHPDSSRTILYSE*
>jgi|Xentr4|200777|fgenesh1_pm.C_scaff...
MDFCSDFVNISCLGTIRSPASYSVMIIVMLGAIILTTIGNLMVIISVSHFKQLHSPTNFLILSLA
VTDFLLGLVIMPYSMVRSLTSCWYFGDLFCKLHGCIDMALSTSSIFHLFFISVDRYYAVCQP
LHYYKCITNNVIQVFLFISWSVSCLYSFGLVFSNIHTEGIQHYIASFTCTGSCSLAFNKIWGA
ISALISFFIPGTLMIGIYIHIFSVANKQAKHMHNHHNIQPDRPSIQIKVSVRAESKAAKTLSIV
MGVFLFCWLPFFILTIVDPYINFSTSEHVYSTVLWLGYFNSALNPIIYALFYPWFQKTFQFII
TGNIFKAGSSFSQVLSNS*
>jgi|Xentr4|200778|fgenesh1_pm.C_scaff...
MDLCFDFVNNSCLRTNRAPANYSVMFIAMFGAIILTTVGNLLVIISISHFRQLHYPTNFLILS
LATADFLIGLVVMPYSMVRSLTSCWYFGDLFCKVHSCLDMTLCTGSIYHLFFISVDRYYAV
CHPLHYYKNLTTNVIELFLLTTWSVSCVFSFGLVFSNVHTEGIQDYITSYYCIGSCSLTLNKL
WGTISSLICFFIPGALMIGIYMHIFSVARNQAKLVHNHHSFQPDKSKSKISVRAERKAAKTL
SIVMGVFLFCWLPFFILTVIDPYINFSVPEDLYNVFLWLGYFNSALNPIIYALFYPWFQKAFA
CIISGNILKAGSSFSQLLTGV*
>jgi|Xentr4|200937|fgenesh1_pm.C_scaff...
MAGNSSYREESGYIPHYERDSDPFASKLSREADIFAGVYLMAIGILSTLGNGYVIYMACSR
KKKLRPAEIMTINLAVCDLGISVTGKPFAIVSCFSHRWVFGWNACRWYGWAGFFFGCGSLI
TLTVVSLDRYLKICHLRYGTWLKRRHAFIALAVIWAYATLWATLPLVGVGNYAPEPFGTTC
TLDWWLAQASVKGQIFVLSMLFFCLLFPTMVIVFSYAKIIAKVKSSAKEVAHFDTRNQNN
HTLEIKLTKVAMLICAGFLIAWFPYAVVSVWSAFGQPDSIPIELSVVPTMMAKSASMYNPII
YQVIDCKPACCKKDNTDAYVEIKPHTSSR*
>jgi|Xentr4|200991|fgenesh1_pm.C_scaff...
MNLSLQQSTLELNISSLLGNNTVPTGKTKSSACEQVVIAAEVFLTLGIVSLLENILVIFAIIKN
KNLHSPMYFFVCSLAVADMLVSVSNAWETITINLINNRHLIMEETFVRHIDNVFDSMICISV
VASMCSLLAIAVDRYVTIFYALRYHNIMTMRRAGIIIACIWTFCTGCGIIFILYYESTYVIICLI
TMFFTMLFLMVSLYIHMFLLARTHVKRIAALPGYNSVHQRTSMKGAITLTILLGIFIVCWAP
FFLHLILMISCPQNLYCVCFMSHFNMYLILIMCNSVIDPMIYAFRSQEMRKTFKEIICCYSLR
MFCDLPSKY*

>jgi|Xentr4|201043|fgenesh1_pm.C_scaff...
MESNQSVNEFIIVGFTQITQHQQLLSLLLLLAYFIIITGNMVVALVILHDHRLQTPMYYLICT
LSLMEVSIVSSVFPTFFPIVLLGKTYISFRCCLLQMYVFHSLVIAENYLLNVMAFDRYLAIV
HALRYHSIMTSRLCKILICGCWLFGFLTPLVLLILVSGMPFCGPNEIRHLFCDSSPLLTLSCA
ENSVNIFLDLAITSFPVSITAFLIIMTYSKIFLVILKMRTNEERKKALSVCASHIITAFFFYGSG
AFLYFELQNRYSSEYDLATAIHHSVLTPLFSPLAYGLPNTEIKKSVKKFSKRLFNRNLRIGS
MQFK*
>jgi|Xentr4|201239|fgenesh1_pm.C_scaff...
MDIASEDYDNYSSQYDYYDFEETPHDSAYPHALHIVSIVIYSLAFILGVPGNAMVIWFTGF
KWEKTVSTLWFLNLAIADLIFVLFLPLHITYVATDFHWPFGKYFCKINSFIAVLNMFASVFF
LTVIGLDRYLFLAHTRFVQRHRTLCKSMILSGIVWISAGIIAAPALYYRDTTEIRKSVTICFN
NFHDYDHSVIKRTHTFLTLIRFFIGYLFPLLTMICSYSLLAIRIKDKNALGSSKFFWTVFTIVV
AFFVCWTPYHIFSLLELNVHHSSEFREWIRIGMPISTSVAFINSCLNPIIYVLISKAFMVHLG
ASLTEIFKNTLRDISQTGVKSEQFVDSKSNPTVCTLKMKN*
>jgi|Xentr4|201852|fgenesh1_pm.C_scaff...
MDKGNGTTVTEFILVGLSEIPEIQQYLFCIFLGIYIITVLGNASIILIYKFSPSLHTPMYYFLSN
FSFLEICYVSASSPKMLQNMLVEKKTISFYCCFTQIYSIVHLAGTEFYVLAAMAYDRYNAI
CHPLLYRIIMNNRVCAQLIAGSWVIGAANSLVHTLLTHTLSFCSNKVNHIFCDVPPLLKLAC
TDTWINQIVMYMVGGSVTGGAFILIIISYTHILSTILTIHSASGRKKAFSTCTSHLTVVTIFYG
SIFFIYLRPKSSYAMHHDRLVAIMYTIVAPLLNPFIYSLRNNDVKAAVRKLLNRRILTWKI*
>jgi|Xentr4|202244|fgenesh1_pm.C_scaff...
MIINGENQTMNYDFCLLGFQLLNSLRIPFFLLILTLYIFIIIGNAMISALVLFYHNLNHPMFFF
LGHLSFCDITLTTTIVPIMLYGILGGGVNISVSACLAQFQIFCAVVASECFLLAVMSFDRYLAI
CNPLRYIYIMRKELCFRLVILCWTLGFSITFNETVLISKLEFCGPNIIDHFFCDFLPILQLSCSD
ISVVKLGQSFVATTIAVFSVMSIIVTYVYILTTVFKIQSASGRQKALSTCSSHMAVLGTFLGS
LIGLYLLPSSGKTLNANKVLSLLYTVVTPLFNPIIYSLRNLEIRAAVKKIIRKMKL*
>jgi|Xentr4|202245|fgenesh1_pm.C_scaff...
MDPVLSDKNQFPSFYRTVPNEIAQYDALVKLIKHFSWTWVGVLVSDNESGLKMSQMLQK
EFALNGICFAFLEFIPYRDALDDTKKLQIVRSLSNSAINVIIAYGDRDYMLTLHLILYMFPISE
KVWIISSQWDVASGFDLLFLRFDPFNGSLALTLHASSIPGFQEFLLNKTPNDFPNDIFIEAAW
LELYLCLWKTNATDLKCTGTEQLQSQYEHFYRDISIYSYSIYNAVYTLSDALHYLQLQKRE
EIRGAHKIQAWEIHKYIKQVSITDGDGNELHFDENGDMPSDFDILNWIVYPNQTLDGIKVG
TYAQRSASQELKINESLIRWSPSFNETPRTPCSETCLIGFRKKPKEGYPECCNDCAPCPEGEI
SNQTDRETCFKCPINQWPNLNRTICTDKVIIHLSFHQPLGASLALLSVLFFIMTCLVLMIFTK
YRNTAVVKANNRDLSYILLVSLKICFLCNLMFIGHPRHVTCILRQTVFGVIFSIVISSVLAKT
VTVVIAFNATRPGSKLRNYLGPRVPNSIVIFCSLIQVIICTSGLINSPPFPYYNTDDIVGVIVAE
CHEHSSFGFYCILGFMGLLAALCFIIAFFARTLPDIFNEAKFITFSMLLFCSVWVTFIPAYLST
KGKYVVAVEIFAILSSSSGILGCIFIPKCYIILFRIDKNKTDFKKKNSD*
>jgi|Xentr4|202252|fgenesh1_pm.C_scaff...
MDRQNETIVTEFFLLGFHNVHGINILLSSVFLVIYMATVAGNFTIFFLISSSQSLKSPMYFFL
RHLSLVDICLATNIVPNMLYIIIKEGGTVSFVGCITQLYLFGVFAVTECLLLTVMSYDRYLAI
GYALQYASVMNPRLCAHLTCCSWAVGFSLTMITVGMVWELRFCGPNVIDHFFCDLVPLLA
LSCSDISSVTVSNFIIATPVTLFPFAFILMSYVRIFLTVLNISSSTGRQKAFSTCSSHLIVVCVY
FGTLIAQYVAPSKSHSVDLKKILSLLYTVFTPLSNPVIYSMRNQEIMKSLIQNTPWLLR*

>jgi|Xentr4|202264|fgenesh1_pm.C_scaff...
MQNESQGQYSDFILLGHSTLPELQFPLFFIFLLIYLLTITGNSLIITCICLDAQIQIPMYFFLQN
LSFLDLSCSCNIQPKMLLGIITGDGAISQIGCFIQLYFFASLTVTEFVLLTAMGYDRYLAICKP
LHYPLLMNKKTCFLLVIICWVVGFLDPLAHTIAISQLPFCRTHLINHFYCDYSALLKLSCVK
TTLIESMAILFSSLVGFSTFSFTLTSYISIISTIIKIHSATGRHKAFSTCTSHLTVILLFYGTIMIM
YIRPISQYSSSLGKSFSLLYTVLIPMFNPLIYTLRNNDVKKSLQKQLFNNVICKGIKMIHSDT
I*
>jgi|Xentr4|202268|fgenesh1_pm.C_scaff...
MPAENQSSSSGFIFQGFSDYPDIQIPLFCLFLLIYLLMLQGNVLILTVIYQTSLLHIPMYFFLC
NLAFIDMFSSSVSQPKLLSMLLGGDNTISFGGCMTQLYCFMSLTCTEFVSLTVMAYDRYVA
ICNPLRYLIVMNRRVCVILFIACWMVGFALPVSHTVLISHLPFCRSQTIDHFFCDPSILLTLSC
ASTFSIELLTYVLCSLVALPAFALVVASYTYIISTIIKIHSATGRKKAFSTCTSHLTVVSLFYGTI
LITYMSPTSQYSSTLSKPFSFLYTALIPLINPFIYTLRNKDIKTHISNIIEFKRSRLQLAGSVL*
>jgi|Xentr4|202272|fgenesh1_pm.C_scaff...
MNQSSSSGFILQGFSDYPDIQIPLFCLFLLIYLLTLPANVLILIVIYQTSLFYIPMYFFLCNLAFI
DLCASSVSQPKLLSILLRRDNTISFGGCITQLYCFIALTCAEFVSLTVMAYDRYVAICNPLRY
LIVMNRRVCVMLVIACWMFSFAEPMSHTVLISHLPFCRSRTIDHFFCDPSILLTLSCASSFPI
DMMTYVLGSLVALPAFALTVASYTYIISTIIKIHSATGRKKAFSTCTSHLTVVILFYGTTIITY
VRPMSQYSSTLSKPFSFVYIALIPLINPFIYTLRNKDIKQHISNKTEIKRGHL*
>jgi|Xentr4|202550|fgenesh1_pm.C_scaff...
MNQTMVTEFILLGITNQPQLQLFLFTVFLVFYLLSFVGNLSIVFVVIFDYHLHNPMYFFLVN
LSFLDFFYSSTTVPKMLAGLVVEEKRISFQGCITQLYIFHFLGSTEAMLLATMSYDRYVAIC
NPLRYHVLMAKATCIQLSAICWTTGFVYSLSHTILTSRLPFCMMNKLTHFYCDIKPLLKLA
CTDTHLNESLVSIVTGFVALFTFILITISYAFISRHLLNIRSTHSRHKAISTCTSHMTVVLLYFG
TAFCTYLRPVTNDSLEQDRITAVLFTVITPALNPLIYALRNKDVKRALTKMFLGNLGTAGN
RSRFIIPLGTFS*
>jgi|Xentr4|202553|fgenesh1_pm.C_scaff...
MENANTTEVTEFVLLAFSNFHHLQGLFFFIILMMFIVSVTGNLAIILLVQSEPSLHTPMYFFI
SVFAALEISFVSATIPKLLANLVGASRTISFVGCFVQLYAFNALGVTECYLLAVMAVDRDLA
INNPLRYEAIMSHTFCIVLSVFPWIISFVIAFIPTIITAKLDFCGPNEVDHFFCDLAPIQNLACS
NPFISNISTSIAAIVLPFIITLLFYIHIIAAIIQIRSENGKKKAFSTCSSHLIVSCLFYSSAIIVYVT
PEGSNSDKYLALIYTVVTPLLNPFIYTFRNKDVKIAFTKLNKTILQHIFV*
>jgi|Xentr4|202662|fgenesh1_pm.C_scaff...
MIKENYTEDNDFFFLGFHNFRKHKALLFIVFFLLYALILAGNLLIIVLVSSHYLKIPMYFFLI
HLSLSDIFLTTDTVPSMLHFLLKEGGSISIASCITQFYFLGCSGITECLLLTVMSYDRYLAICF
PLHYMSIMDLRCCCYLVACCWTVAIGLMMITTLLVSKLQFCSPMTIDYFFCDFAPLIELSCS
DTATVKTANFLLAIPVTIFPFGFIVTTYICIVLAVLRISSNTERKKAFSTCSSHLTIVCMYYGT
LVAIYANPFPGNSLNMKKYLSLLYTLVIPFFNPIIYSLKNQEIRTVLNMYIRKISNRKDL*
>jgi|Xentr4|203232|fgenesh1_pm.C_scaff...
MMYITNTSFPHFSLLGFSDVPYLRTYLIMVFFTMYLLTILGNLAILLCIVYDQHLHLPMYYF
LSNLSLVDICLTSSTISTLLSSLFFNHLLVSFPSCIAQMYCFVSFGNLENNLLAVMAYDRYVA
ICSPLQYYMKMKHRLCIALVFLSWVAACLHSLLHTLMVATLEYTGVKQVNHFFCEISQVL
AISDSDTSVNFLLIFTEGAVSVGAPFICIIMSYFCIVRTIVQMHSADGRSKAFSTCSSHLTAVC
LFYGTIAAVYFLPSTTASLLNGRLATIGHTLLTPLLNPFIYGLRNKAIKTVFRKLFLGKLRH

MFIRINDYN*
>jgi|Xentr4|203311|fgenesh1_pm.C_scaff...
MDSNQTSSNRFILLGLTNIPYLQAIYVLLFFIIYITTLLGNSLLIIVVRINEQLQTPMYFLLCN
LSITDICLSSTIVPRIMINTLSQDRSISLSDCALQMFFSLAVGGTECLILAVMAYDRYAAICQP
LHYNTVMNKTFCICMATGSWIFSFITAVLHVYFTFQLPYCRSHNVNHYFCEMPSFFRLSCR
DTWPNELANYITTIIITMCSFLLILISYFHIIFTILKIRSNEGRKKSFSTCTSHLTVVSMFYGTIL
VIYMSPDSANSSITKTVSIINSSVIPMLNPIIYSMRNKDVKGTIRKQMKNKSFF*
>jgi|Xentr4|203567|fgenesh1_pm.C_scaff...
MENQTIAKEFHLYAFANNGVNQSLLSIVFLLMYLSGVVGNLVILTSIYNDVQLHTPMYFFL
CNLAFVDISFPTTILPKLIDILLSGNNSITFIQCFTQMYFFAVFAVIEVFVLSSMAYDRYVAICK
PLRYHLIMNKRVCVLLIGGTWISGFVNASLFADLACNMPFCQTNKINQLFCDAKALAEISC
DATKFYILIYVENVLFGLIPFSLNLISYINIIHNILQIKSRHGRKKTFSTCTSHLTVLMLFYGAV
AGMYMSPPSKSDIVEPVLSLLYTVVTPMLNPFIYSLRNKEVKVAVSRLLQRKI*
>jgi|Xentr4|204013|fgenesh1_pm.C_scaff...
MPLPLFSSVPPFAVQDQNQTSVLVFHFLGFGNLPGLQMPLFCGFLLVYVAIIAGNLLIVFLV
SFQQSLHSPMYLFLSHLSLCDVWCTTNIIPNMLNIVLFGGGTISATGCIAQFYLFGSLTTTES
FLLTVMSYDRYLAICYPLHYFSIMDLRLLLCLTTWSWVLGFVLTLIPSVLVGSLQFCGPSAI
NHFFCDLSPLLALSCSPRTSEIQTEIIFSSIPVILFPFIFIIGTYLKIFLTILNISSKRGRQKTFSTC
SSHLIIVCTYYGTLIIVYMIPAKQNFSNVNKILSLLYIVVTPLFNPIIYSLRNREIKTALWKIMS
FTAKGEV*
>jgi|Xentr4|204081|fgenesh1_pm.C_scaff...
MNNSSGLTAYVENGNSSNSTNFMNQKISVFFSIIFMTVGIISNSLAIAILLKAYQRFKKKSKA
SFLLFASGLVITDLFGHLINGTIAVFVYASKRDWLRFDYSNILCSVFGMCMVFFGLCPLLLG
SVMAVERCIGVTQPIFHSTKMTSKHVKIILSLVWLFAFLVALMPILTERGYHIQATRTWCFIK
TEHIEEWRDQFNLLLFSFLGILSLAVSFMCNAITGITLLRSKIRNHQYRQGRSHHIEMIVQLL
AIMCVSCICWTPFLVTMANIVFSGDEPLQTRATILFALRMATWNQILDPWVYILLRKTVLK
KLFRIARRCCGIEVISLHTWEFSSIKNSLKGATLSDSPSCTPANAFKSSHKTDTERKCELSET
K*
>jgi|Xentr4|204898|fgenesh1_pm.C_scaff...
MANRSETFEFVLLCFPGLHQKFQILVSITMFIVYISTLSANGIVVGLIILKEHLHQPMYMFIG
NLALSDLLFDTITLPKIIASYWSGDGNISFFGCFSQLFFVHYLASVDIFIITLMAADRYIAICE
PFRYATIVSQKVTTIFCCAFWVLCTLPSAYATLMALNASYCGRNNIYNCFCYSNVVYELAC
SDVFSVRRNLLIISMSILFVPLAFIILSYIIIIKIICTSTSSDNWSKAFYTCTTHLFVIALYFIPRVI
IYTSNWVKLNLNVDVRILILCLYSYTPHLVNPVIYCLRTKEIKSTLEKMLRMKFKLNN*
>jgi|Xentr4|204991|fgenesh1_pm.C_scaff...
MGGFADTTFRTITTDFLNMGPNDVQYDDSKRGISSKSDYFPPKLSSLRGDPLRKIPLDTLD
QINATDFYNKSIFKDNEECGKNFMDMECFMILTPSQQLVIAALSIILGTFTVLENMLVLVVI
LQSRSLRCRPSYHFIGSLAVADLLGSVIFVYSFVDFHVFHRKDSPNVFLFKLGGVTASFTAS
VGSLFLTAIDRYISIHRPLSYKRIVTRTKAVIAFCMMWTIAIVIAVLPLFGWNCKKLRSVCSD
IFPLIDETYLMFWIGVTSVLLLFIVYAYMYILWKAHTHAVRMLQRGTQKSIIVHTSEDGKV
HITRPDQTRMDIRLAKTLVLILVVLIICWGPLMAIMVYDVFGKINKTIKTVFAFCSVLCLLN
STVNPIIYALRSKDLRSAFCSMFPSCEGTAQPLDNSMESDCQNRHVNNSNVHRAAESCIKS
TVKIAKVTMSVSTDTSAEAV*
>jgi|Xentr4|205020|fgenesh1_pm.C_scaff...

MNEKNRTWVDNFIIKGITDIPELQTPIFVLVLLIYLTIIVGNSVILSLICVNPRLHNPMYFFLS
NLSVLDICYTTVTMHVVLLSYVSGKKSISFSGSMSYDRFIAICNPLRYLIIMSHKICVILIFAC
WLLGFLFVIPVIFVIFDILCFQSNEINHFFCDLLALMKLFCSNASSMEHLIYIESVFIGFVPFSL
TVLSYVRIIRAILKVQSTGGRLKAFYTCSSHLTVVLLLYVTIFCLYMRPTPTFTLDSDKLISL
LYTTLTPMLNPIIYSLKNKDVKAAFRQLVKNKSATVKFRKSINCVAR*
>jgi|Xentr4|205043|fgenesh1_pm.C_scaff...
MESPLNCSALFNSHIDTGLEKEEAAKVLQELCKVSWAPQRKQVSAAVLGISGALLSAGLL
LTLFFFIFTLRFRSNRIVKMSSPNLNLVTLIGSALTYISAFLFVVQEPNISMETIFQVRISLLYL
GVTLVFGPLLGKSWRLHRVFTHRVPDKRVIIKDVTLLSLVAGLLFADTLLLLMWVLSDPVV
CARSASASIKAAARGTLYSVTTQDFCASKYSDLWTGLLVGFKAALLIYGGYLAGLTNNISS
PPVNQSLAIMIGNGLVMAGTGAVFLVTHFFHDWSNLVYATTSGSILVCTTAINCLIFIPQLLQ
SRQFEEEQVQSTQMSKYFSSPSKSFRSMYSEEQIYHLLGENTSMRKLLSEKNAIIESLQEQV
SNAKEKLMKLIKSECSFETIEVTSLSVLSASTLAIQHHVAPEHMKTIEEPLDSVENADHEAV
IKDTQEVDIKHDTKSDAFQSPKDQNIQESEEKPQEGSQCTNKPSGCSVSKQEDMSTSDSRS
VSFADIVSKSHHQHPGEASERTHDVLEQLSRKINYVSSEKLQEILQELSIETTCGHHSPKRQ
RRASHSVNRDPGLVPSEGFRKMCVSLSPYMMRRRRGPVYSQRNQHPSHHFPNALPPQTW
CLLNKEARNRCNGVKTTRDERPSPTGEERTAEGNIGHGHHNPSLLTKAMELSVPGREENR
SRTDSSSSEGTICYCHRPYCDLCFANEYDSSDSSETDSGDQLHGWATQKTPPQLVVNFNED
LTPTFV*
>jgi|Xentr4|205499|fgenesh1_pm.C_scaff...
MENQTTVNTFILTGLCDLPSLQLPLFLVFLLIYLITLTGNLLILLLIFTDSHLHTPMYFFLGTL
ACLDMSCSSVTVPRMLFDLLRERKIISMRVCIAQFYFLVFFMTSEMSVLAVMSYDRYIAIC
RPLHYMQIMNWNICVQFVSGVLLFSAIGSLVHTLSLTKLTFCRPKVLQSFFCDLPQLLQVS
CSDTFINVLLIFLFGILGGIGILGATFYPYIPIITTVLKMTSKHTRSKAFSTCSSHLTVVFTCYT
TIFFNYFRSNGNDNLVEDKVASVFFAILSPSLNPVIYSLRNQELKLSLRKTLQRLN*
>jgi|Xentr4|205575|fgenesh1_pm.C_scaff...
MCLSVIIFVIGIMGNMAVMCIVCHNYYMRSISNSLLANLAFWDFLIIFFCLPLVIFHELTKK
WLLEDFSCKIIPYIEIASLGVTTFTLCALCIDRFRAATNVQMYYEMIENCTSTTAKLAVIWV
GALVLALPEVVLRQLSVEDTGVNGRFLRERCVVKISTELPDTIYVLALTYDGARLWWYFG
CYFCLPTLFTITCSLVTARKIKRAEKACTRGNKRQIQLEGQMNCTVVALTILYGFCVIPENIC
NIVAAYMSSGVSRQTLDLLHLISQFLLFFKSCVTPVLLFCLCKPFSRAFMECCCCCCDECIQ
KSSTATSDDNDNEYTTELELSPFSTIRREISTFASVGTHC*
>jgi|Xentr4|205653|fgenesh1_pm.C_scaff...
MENITKVSEFALTALSHQPHVKYFLFLVFLLIYLTTSLINLLILFLSLTDAKLHTPMYFFLGN
LAFLDMSYSSVTVPRMLSDFISKSTTISYPACITQTFFFIYIASAELFLLAAMSYDRYVAVCH
PLRYIQIMSWKVCAQMISVVWILGLLYSLVHILLSLRLTFCSSNIIHNFFCDLPHLFQLSCTD
TFINFVVIFIAGGILGLLAFAITFLPYVRIISAVLKISSTEGKRKAFSTCTSHITVVSIFYASIIFI
YFVPTTSYLFLLNRIVSVIYSVINPLLNPLIYSLRNKDLMSACRRALPGTFAL*
>jgi|Xentr4|205673|fgenesh1_pm.C_scaff...
MAAFLCLIILLTTVGNSLLILLIFTQRSLRNTSNYFLVSLFMSDLMVGLVVMPPAMLNELYG
KWILDVDFCCIWYSFDVMCCSASILNLCVISLDRYLLIISPLKYKLRMTSCRALCLILATWT
LAALASFLPIELGWHEQDFEIQHMNMTSGTEEKQCRLIVSLPYALVASCLTFFLPSVAISFTY
CRILLAARKQAVQVASLTTNVPSASEDHTQVLALPAVDSRKFVTKHSKKALKASLTLGILL
GMFFVAWLPFFVANVVQAVCDCVPPGLFDLLTWLGYCNSTMNPIIYPLFMRDFKRAMGK

YLPCCRRSRVISLSIRNSNSAPRLGLSLRNVLTLRGETDSMYSATLGNEHLLPNSKDHSTDP
LPVTAADSVNLFDLEQEMQVHQLNTPMD*
>jgi|Xentr4|205728|fgenesh1_pm.C_scaff...
MVLEMKENVSGFIIQGFSDTPELHISLFVLFLGIYLIILLGNLIIFLVISCNPHLHTPMYIFLLN
LSLIDISSTSNILPNLLHILLTQQNNISFLGCMTQMYVFGFLAGSEYFLLTAMAYDRYVAICD
PLHYIARMSRKHCAGLITSSYTVGFLDPVSIVTLISKLSYCASHLIDHFFCDMTPLLKLSCSS
TFSAELFIYIEGTLLVFSSFIPTLASYIFIISAILKIQSSEGRQKAFSTCASHLTCVITLYGTAIFL
YLKPTTSYSLKKDKYFSLVYIALGPLLNPFIYTLKNREFQASFTKI*
>jgi|Xentr4|205765|fgenesh1_pm.C_scaff...
MTSESKEFLNVSGFIIQGFSDTPELQTSLFVLFLGIYLIILLGNLIIFLVISCNPHLHTPMYIFLL
NLSLIDISFTTTVLPNLLHILLTQQNNISFLGCMTQMYVFVALASSEYFLLTAMAYDRYVAI
CDPLHYIARMSRKHCAGLITASFTVGFGDPVGIVVLTSKLSYCASHLINHFFCDVTPLLNLS
CSSTFTAELSIYFVGTLLGFSSFILTLTSYIFIISAILKIQSSEGRQKAFSTCASHLTCVITLYGT
GFCLYMRPTTKFSLKRDKYFSLLYIALGPVLNPLIYTLKNREFKSSIHKMRQKCLA*
>jgi|Xentr4|205800|fgenesh1_pm.C_scaff...
MASEIKENVSAFHLQGFSDTPELHISLFVLFLGIYLIILLGNLIIFLVISCNPHLHTPMYIFLLN
LSLIDISFTTNIFPNLLHILLTQQNNISFLGCMTQMYVFVSLASSEYFLLTAMAYDRYVAICD
PLHYIARMSRKHCAGLITAVFTVGFVDPVGHFVLVSKLSYCASHLINHFFCDISPLLQLSCS
STFSVELLNYIEGTLLTFGSFFLTLTSYIFIISAILKIQSSEGRQKAFSTCASHLACVITLYGTAL
SLYMRPTTSYSPKRDKYFSLLYIVLGPVLNPLIYTLKNREFQSSFNKMRQII*
>jgi|Xentr4|205805|fgenesh1_pm.C_scaff...
MASENVSGFIIQGFSDTPELQISLFVLFLGIYLIILLGNLIIFLVISCNPHLHTPMYIFLLNLSLI
DISSTSNIFPNLLHILLTQQNNISFLGCMTQMYVYGFMAASEYLLLTAMAYDRYVAICDPL
HYIARMSRKHCAGLITAVFTVGFGESVSLVILISKLSYCASHLINHFFCDVTPLLQLSCSSTF
SVELVIYIEGTLLAFNCFLLTLTSYVFIISAILKIQSSEGRQKAFSTCASHLACVITLYGTVLSL
YMRPTTNYSLKRDKYVSLLYIVLGPMLNPFIYTLKNREIHSSFNKVRERFLAF*
>jgi|Xentr4|205850|fgenesh1_pm.C_scaff...
MNEKNQTWVNEMTLLGFQNLHNFKVPLFSLFLLIYILTVWENVLIIVLVAFSRNLHSPMYF
FLQQLSLTDLLESSNIVPTLLLSVINNGHKISLVGCITQLYFWGVSEIFECILLAVMSYDRYV
AICIPLRYTSIMSTNVCFQFILVAWALGFGITVISVVLMGRLKFCDRNTINHFFCDLSPLLELS
CSDTFLVQIEGMIVSLILAVTPLILISVSYMCIAHAILKIVSHTGRQKAFSTCSSHLAVVSLYY
GAIIAIYVVPSTEQSQILSKVLSLIFTLVIPMINPVIYSLRSADIKNILSKTKE*
>jgi|Xentr4|205851|fgenesh1_pm.C_scaff...
MSMKNQTWVSEIVLLGFQNLHNFKVPLFSLFLLIYILTVWENVLIIVLVAFSRNLHSPMYFF
LQQLALSDLLESSNIAPTLLLTVMQDGITLPFAGCMTQFYFFSATEGFECLLLAVMAYDRY
VAICIPLRYTSIMSHRVCVTFIVLSWVLGLMSALPLANIMATLQFCDQITIDHFFCDFFPLLQ
FSCSDTFLVHIIMILESIPVVSLPFILISVSYMCIAHAILKIVSHTGRQKAFSTCSSHLAVVSMF
YGTIISIYMVSPSKESQTTNKCLSILYTIATPLMNPLIYSLRSSDIRQGLGLLTAKS*
>jgi|Xentr4|205855|fgenesh1_pm.C_scaff...
MNEKNQTWVSEIVLLGFQNLHNFKVPLFSLFLLIYILTVWENVLIIVLVSFSRNLHSPMYFF
LQQLSLTDLLVSSVIIPILLQTVIGGTATLSLVGCINQLYFFGGSEIYDFLLLAVMSYDRYVAI
CIPLRYTSIMSHRVCVTLVLISWLLGFGITVITAFLVGTLQFCDQNTINHFFCDLFPLVELSCS
DTFKVQLEAFLASVLVTVIPFLVIIASYMCIAHAILKIVSHTGRQKAFSTCSSHLAVVSMFY
GTIISIYVVPPRKESQTISKVLSLFYTVGTPLVNPLIYSLRNKDIKEAINVKFNRIRKYINIYK*

>jgi|Xentr4|205995|fgenesh1_pm.C_scaff...
MVSGSQENVSGFIIQGFSDTPELHISLFVLFLGIYLIILLGNLIIFLVISCNPHLHTPMYIFLLN
LSLIDISFASNILPNLLHILLTQENNISFLGCMTQMYVFVSVAGSEYFLLTAMAYDRYVAICD
PLHYIARMSRKHCAGLITAAFTVGLSESVGLIVLIPKLSYCASHLINHFFCDISPLLKLSCSS
TFSVELFIFIEGTLLISSSFLLTLTSYIFIISAILKIQSSEGRQKAFSTCASHLACVITLYGTVICL
YMRPTTSYSTKRDKYFSLLYIVLGPVLNPLIYTLKNRQFQSSLNKVRQRCLYFYFR*
>jgi|Xentr4|205996|fgenesh1_pm.C_scaff...
MASESQKNVSGFIIQGFSDTPELQISLFVLFLGIYLIILLGNLIIFLVISCNPHLHTPMYIFLLN
LSLIDISFSSNILPNLLHILLTQQNNISFLGCMTQIYVLVSLAASEYFLLTAMAYDRYVAICDP
LHYIARMSRKHCAGLITAAFTVGFGESFTLIVFISKLSYCASHLINHFFCDITPLLKLSCSSTF
SVELSIYIEGSLLTFSSFLLTLISYIFIVSAILKIQSSEGRQKAFSTCASHLACVITLYGTVFCLY
MRPTTSYSLERDKYFSLLYIALGPVLNPLIYTLKNREFQSSLNKQIHISSSLY*
>jgi|Xentr4|206024|fgenesh1_pm.C_scaff...
MASESQANVSGFIIQGFSDIPELHIYLFVLFLGIYLIILLGNVIIFLVISCNPHLHTPMYIFLLN
LSLIDISFTSNILPNLLHILLTQQNNISFLGCMTQMYLLVSFGASEYFLLTAMAYDRYVAICD
PLHYIARMSRKHCAGLITAAFTVGFVDTVGHVTLISKLSYCASHLINHFFCDVIPLLKLSCS
STFSMELFIYFVGTLLFFNSFLLILISYIFIISAIRKIQSSEGRQKAFSTCASHLACVITLYVSVV
CLYLRPTKSYSLERDKYFSLLYIILGPVLNPLIYTLKNREFKSSFNKMRERLVFWINIF*
>jgi|Xentr4|206148|fgenesh1_pm.C_scaff...
MSIPNQNKTMEFLLEGISCLPQFQIPCFLAFLSLYSLTLTGNVLIISIICLDPVLQTPMYFLLC
NLALLDISYSSVTQPKFLSMLLTNDSVISFNQCILQLYVFLSLASTEFLSLTAMAYDRYVAIC
NPLHYFMLMSKKMCVLLTIMCWLVGFLDPMAHTVCISQLNFCRSNIINHFYCDVSVLLKL
SCDDTYYIEFMSYIIGSVFGLPAFILTLSSYVCIVSTIMKIHSAQGRWKTFSTCVSHLTVVIIF
YGTLLIVYMSPITYYSSAKAKSLSLLYTVLIPLCNPLIYTLRNKDVKLSLWKLFNVK*
>jgi|Xentr4|206154|fgenesh1_pm.C_scaff...
METGFAYELAATLDFKDFLNQSKVTQVIILGITDIHVLQTPLFIVFLIIYLLTLAANLLIITVV
SLNPLLHNPMYFFIGNLSFLDIFYSSAIQPKLLSTLLAGPSVVSFPGCIAQLYVFMSLACTEF
VSLTAMAYDRYVAICKPLHYAVLMNTRTCMLLATVCWIVGFLDPLAHTMVISQLPFCKPPI
INHFFCDLSVLLNLSCVDKVFIEILTYVVGSMVALPAFVLTLTSYIFIISSILKIHSSDGRQKAF
STCASHLTVVILFYGTVLVMHMRPSSHYLLAQDKPLSVLYTAIIPILNPLIYSLRNKNVKKA
LGEIPRFYLHR*
>jgi|Xentr4|206201|fgenesh1_pm.C_scaff...
MENQTTVYMLVLTGLSDLPSLQLPLFLVFLLIYLITLTGNLLILLLIFTDSHLHTPMYFFLGT
LACLDMSCSSVTVPRMLYDLLRERRIISVPACITQFYFFLFFAISEMSVLAVMSYDRYIAICR
PLHYMQIMSWNICVKLVSIILVFGAGYSLLHVVFLTKLTFCNPNDLQSFFCDLPQLLQVSCS
DTFINVLLILLLGILLAVCLLGVTFYPYITIITAVLKIPSKNMRSKAFSTCSSHLTVVFMFYTT
ASFNYMHSNANDHHTEDKVASVFYAILTPF*
>jgi|Xentr4|206203|fgenesh1_pm.C_scaff...
MYELVLSALSDLPSLQLPLFLLFLLIYILTFIGNLLILFLIFTDSHLHTPMYFFLGTLACLDMS
FSSVTVPRMLFDLLTNRRIISVRACIAQIFFFVYFAASEIFVLAVMSYDRYIAICRPLHYMQI
MTWKACAQLVLAVAAYSSVHTLFLAKLTFCRSEALQNFFCDLPQLLQISCSDIFINVLLIFL
LGIMFGLAILTVTFFPYIVILRTVLKISSKTTRSKAFSTCSSHLTVVFIFYTTIFFNYFRSSAND
QFTEDKVSSVFYTILTPFLNPLIYSLRNQELKKSLRKTLQRLQIKNYSD*
>jgi|Xentr4|206495|fgenesh1_pm.C_scaff...

MRTFYAYKEQREIQRGDIMLGGVLTIHTTCYLYQIIHFLRLFRQNYRQFVDFAFAVDEINKK
PSQLPNLTLGYQIYDSCGDPRKALWNVFQILSGTKRPVPNYSCVRNRTIAGFIGDLDSDTT
LPIAQILSLFGYSQISYGASDPALSDRDAFPYFFRVLQSSRGQYLALSKLLKYFGWTWVGII
ALTDTGDKDYQLLRESLAKEGICMEFLQKLNYHLEWSLDEDAVRSVKGTIRRASTSVIVLI
GKPSLGTIGAISNIIELSQRKTLVVCSSVAINKFIMNFGLKMFHGSLIFEQYSRPRPDAPSVY
HFMLRNGFSMFHSPEDNDGNSSIFTEEFSYSNVYFAVHVMSLALQHLHRPLGQRDPPGEG
HKYRHKLHQYLKRLHYQTTTKDKISFEENGEMVVDYEIYNCRVTARDGPVMKLIGSFQP
RAQNKLRISPDQIIWNSGKNKIPRARCSSPCPPGYSKAPAVGIQSCCYTCVICSAGEISNVTD
SKNCSKCPDMEWPNRKRNECVAGWEDFLSYSDVISVVLVSVAVFLYLMALLILGVFISYR
DTPIVRANNRSLSFLLLVSIKLSFLSVFLFLGRPVDITCMLRIITFGITFSIAVSSLLAKTIMVC
VAFKATKPGSSWRKWLGVKLSNSVVLFCSSIQIIICMTWLAISPPFQELDIHTSPGTIIIQCNE
GSAIGFYSVIGYMGLLAAVSFVLAFLARSLPDTFNEAKYITFSMLLFCSVWITMIPAYLSTK
GKNTVCVEIFAILTSSAGLLASIFLPKCYIILYRPEMNTKSQLLGNKSL*
>jgi|Xentr4|206753|fgenesh1_pm.C_scaff...
MVLLEPVQTFRTISGHLNNFKILIFSLILLIHILTVYENALVIALVTVSRGLQSPMFFFLQQLS
FSDLLESMVVVPTILKTISFLLSVMSYDRYLAICNPLRYSSLMSHRVCVKLIVMSWLLALS
VTPVVVISAATQEFCNQNTINHFFCDFFPLLELSCSDTSLARILIYIASVPAVLFPFVLIIGSYI
CIAHEILKIVSSIGRQKAFSTCSSHLAVVSIFYGSLIVTFVVPTRIQSENTRKLLSLFYTVVTP
FINPMIYSLKSADMKNALKNIKQ*
>jgi|Xentr4|206841|fgenesh1_pm.C_scaff...
MENVSSVSRHFVLLGLVEMEDLRYLYCILSLFLYVFILLLSLGIVLVVLTEESLHEPMYIFIC
NLTFNGMLGSSSFFPKLIIDLLASSHQISHIGCFLQVLFMMIYVLFEISSFTLMAYDRYLAVC
EPLRYFTLMTNKKAIQLILGFFGLCFISVSIAVILSARLSYCGTGIKNVFCDNLSLIVLSCGDS
SVNNLYGLVGTIVFLVLTLLAIAYSYLRIFMVCLKISKEACEKAIHTVVTHLLGFSLFLVGTF
FVFIRFRLGNNNLPLFAHILLSVTLVVVPPLLNPLIYGIRTKALISGLFPSFLHKDSPRDT*
>jgi|Xentr4|209443|fgenesh_pm_kg.C_sca...
MNISMQNSSICNSTGGNETCIVNPFLQPAWQIALWSIAYSIIVIVSVVGNIIVMWIILAHKRM
RTVTNYFLVNLAFAEASMSAFNTVINFTYAIHNHWYYGLIYCKFHNFFPISAVFSSIYSMTA
IALDRYMAIIHPLKPRLSATATRIVIAVIWGFAFALAFPQGYFATVEPTPDRVVCYMEWPESE
NRKFEKVYQVLLFGLIYFLPLLVIGCSYTFIGMTLWASEIPGDSSDRYHEQVVAKRKVVKM
MIVVVCTFAICWLPYHIYFLLQLFLPDESQKFYQQLYLGIMWLGMSSTMYNPIIYCCLNDR
FRIGFKHVFRWCPFIHAGEYEGLEMKSTRYLQTQSSMYKISRIETTVSSVLSANDEDAEET
TKSSKRLSLDLTSNGSSRSVCKTMSDSSSFYSNNLS*
>jgi|Xentr4|212189|fgenesh_pm_kg.C_sca...
MAAASFQPLKCLLLWVFFVITPPVKAVPEPGIWTVPIGSGAGSLFFRKTLYNSTNIKVKLIS
PNCPTPVKLTVKWYLRSHRCYNQFTNLEEVLERHHTNLDTTDNFCKNVSKPDFCDKNED
KNIDCNKDMHALPTLKMSKLVPKISVNQSAGSKNPLNQMDFDIVARTYQDGPYLFVLQV
KGDENVKWNLSVTVSMKGPHGFISASDWPLMIFYMVMCIMYILLALLWFIWSACYWKD
LLRIQFWIAAVIFLGMLEKAVYYAEYQNTDNTGVSSHGLLIFAELISSIKRTLARLLVTIVSL
GYGIIKPRLGAVMHRVVGMGVLYFVFAAVEGVMRIIGAKEYDLVLLAGIPLALLDSGLCC
LAQTMKTLKLRKNTVKYSLYRHFTNTLIFAILASIIFMIWRTKKFQLVDCQADWMELWVD
DAYWRFLFFIILLVIMFLWRPSANNQRYAFTPLIDDSDDEVEEFLVTDHLAEGMKLRGTKP
ECNGAPKPPATNIDEDLKWVEENIPSSFADAALPVLMDSDEEIMMTKYEMSKIE*
>jgi|Xentr4|213043|fgenesh_pm_kg.C_sca...

MLVLTGLSDLLNLQLLLFLLFLVVYFMTLIINLLILLLIFTDSHLHTPMYFFLGTLACLDMS
YSSVTAPRMLFDLFTGRRIISVPACITQVYFFIFFGVSEVFLLAVMSYDRYIAICRPLHYMQI
MSWNVCVQFVSGVLVSTSVYSLLHTLFLSKLTFCSSNVLQSFFCDLPQLLQVSCSDTFINV
LLIFLFGILLGIGILGVTFYPYITIIITVLKIPSKNMRAKAFSTCSSHLTVVSIFYSTAFFNYFRP
NAKYHFTEDKVASVFYAILTPFLNPLIYSLRNQELKTSLKRTLHSL*
>jgi|Xentr4|265822|estExt_Genewise1.C_...
MCDGQTEIKDTFRYITSVISCLIFIVGITGNSALLRIIYKNTCMRNGPYILIASLALGDLLHIII
DIPITTYKLLAQDWPFGVEICKLVPFLQKATVGITMLSLCALSIDRYRAVASWAHVKTTGVS
KWIAVEIAFIWLVSSALAIPEVIGYDLIVTNYRGELLQTCMLHPIQTSAFMRFYKTYKDMW
LFSFYFCLPLVLTAVFYTLMTFELFRKKNGMALAITDTLRQRREIAKMVFYLVLVYAVCWL
PLHLSRILKFTFYDEKDAKRCDFLSFLLLLDYIGINMANLNSCINPIALYLVSTKFRNCFKSC
LFCCCQSKEMLSLEEKHSCMKFKGHDHGYDNFHSISKDSSS*
>jgi|Xentr4|265866|estExt_Genewise1.C_...
MDFQADLEEDINIQDNNSQIFHNFSMSDPSSNVTYASVIMPTVFGVICLLGIIGNSVVIYTVF
KKSKFRCSSSVPDIFIINLSVVDLLFLLGMPFLIHQLLGNGVWHFGETMCTLITALDTNSQF
TSTYILTAMSIDRYLATVYPFTSAKYRKPPIAIMVICILWVLSLLSITPVWMYARLIALPGGV
LGCGITLPNPESDIYWYTLYQFFLAFAIPFAVISVAYRRILLKMASSEALTAHRSSRIRTKKVT
RTAIAICLVFFICWAPFYVLQIIQLVMDQPTLAFHYAYCVAISMGYANSCINPFIYIILCETFRR
RFIVSVQPAEDHPESRIRMKFRTDPPSGSGQPLLQLVPVSTGS*
>jgi|Xentr4|267641|estExt_Genewise1.C_...
MAPCQALNSTGTFPEVDSNGTTWLSLTQRWMLGAVLSFMIMVIVFGNMLVIIAIAKTPRL
QTMTNVFITSLACADLIMGLLVVPPGATILVTGDWLFETTVCELWTSVDVLCVTASIETLC
VIAVDRYIAITSPLKYEMLVTKVRARLTVCLVWAISALISFLPIMNRWWRDTHNEEAKSCY
DNPKCCDFVTNMPYAIVSSTISFYVPLVIMIFVYVRVFIVATRQVRLIEKDKVRFNGTPVPT
NKSRRASRRRPSGLLAIKEHKALKTLGIIMGIFTLCWLPFFVANIIKVFCR
>jgi|Xentr4|268894|estExt_Genewise1.C_...
MINIDVNCSINHDIDQYLFPVTYITVIVISIPTNCISLYVSCLQIKKKNELGIYLFNLSFSDLLY
TLILPLWVYYSLSHNNWMLSEHVCSLVAFLLHTNLYSSAGFLTCISLDRYLAVVHPLKFSH
LRTIRTAILVSFVVWLIQHLSNAIILTQHELFNSSGDLTCYDIFPMEPWKSSFNIIHICIGHLLP
LFIMVLCYQRIFAAVKTNQATADRDKQKIKQLLLTIIVSFVISFTPYHVVLFIRSIREPGDCDF
AKDIFTPYKFTLALTSINCIADPFLYCFVSEAGRADVRTILHCCGKQTEPLEKSGIMMSAITP
STNQENV*
>jgi|Xentr4|269438|estExt_Genewise1.C_...
MLRHYFLPILYSVTCITGLVGNLLIIIIYAFYEKMKTLTDTFMVNLAMADILFLCTLPFLAYQ
AAEGWIFGNLMCKIIRGGYRINLYSSMLILTCITFDRFISITQAKKLKISHSKKHRWGKLVC
VIVWTVSLILAVPQFMFSKSNDKMECFETYLEGHLHLIVNSFQMTVGFFVPLAAMIFCYTF
IIKTLIFSSNFQKHKSLKIIFLVVIAFIVTQLPYNIAILCHVLYKTINAKVLVITEAIAYLHACIN
PILYFFVGIKFRKNFCKILVDLHLAKPNLELSDNLKTTDRDSRSISAFNNTETITMHQL*
>jgi|Xentr4|270513|estExt_Genewise1.C_...
MVLQRTSQHIDQQNSRILTFITYIGCGISAICTAATLLTYIAFEKIRRDYPSKILMNLSTALLFL
NLFFLLDGWIASFGINELCITVAVLLHFFLLATFTWMGLEAVHMYIALVKVFNTYIRRYML
KFCIIGWGLPAIIVSVVLASTHSNIAYGSSSYGQDSRGNGGDEFCWIKSDTVFYVTCAAYFA
IMFLMNVAMFIVVMVQICGRNGKRTNRSIREEVLRNLRSVVSLTFLLGMTWGFAFFAWGP
VNLAFMYLFTIFNSLQGLFIFVFHCALKENVQKQWRRHLCCGKLRLTDNSDWSKTATNNT

KKVSSDNLGKSLSSSSIGSNSTYLTSKSKSSTKPFFKRNSSTDNGF*
>jgi|Xentr4|270690|estExt_Genewise1.C_...
MNCTNCTIKSKPVTEKMLISLTLAIITILTTVLNSAVILAICTTKKLHQPANYLICSLAVTDFL
VAILVMPLSITYIVMDTWTLGYVICEIWLSVDMTCCTCSILHLCVIALDRYWAITDAIEYAR
KRTVKRAGIMILTVWTISVFISIPPLFWRNHHNINIPSKCIIQHDHLIYTIYSTCGAFYIPLTLIL
ILYYRIYHAAKNLYQKRGSSRHLSNRSNDSQNSFAHCKLTQTFCVSEFSTSDPTTEFEKMN
ASVRTPPYDNDIDIFGDRQQQISSSRERKAARILGLILGAFILSWLPFFIKELVVGIQLCTVSP
EVADFLTWLGYVNSLVNPLLYTSFNDDFKLAFKKLIRCKEHS*
>jgi|Xentr4|271421|estExt_Genewise1.C_...
MEEYEGDSNSGELFEFPGSREEEILVTSTLSAVLAAMFLLGMAGNIYVLFINMLSAKPAGC
MCVHVISLALADLLYLSTIPFVVSTYLARDWYFGDVGCRVLLSMDLFTMHASIYHLTAMS
VERYQAVVHPLKSRVSQSHHKFTSVAIWLTSLLLTLPMMCMMQLQDSPYGSGKRICFPTW
TPNGFKCYLTVIFCTSILGPGLILIYLYLHLARVYWMSGIEVQQSRHKLNQCLGFRIFTIILA
YWACFVPFWAWQLAKLYQCDSMRLTAAAQIYLNFGVTCLTYANSCVNPLLYTLLTRNYW
EYLAGRGR
>jgi|Xentr4|273777|estExt_Genewise1.C_...
MKLALKQQEMKCMAKLKAQATEISSEKGCIGIWDNITCWDTARHGEKVTQNCPVALRHI
LRKQGNITRTCKRTGWTDIYPDIVEACGYNDSEPMKLNFYIIVQTIYTFGHSVSLIALTIGST
ILCLFRKLHCTRNYIHLNMFISFILKAISVLIKDGFLFSNPESCPESLIGCKVILVLMQYCVM
ANFYWLLVEGLYLQTLLVVIFTSHKLFIVYLLIGWGIPTIFIIIWIVSRVYLDDTECWDTNDH
SVPWWIIRIPIIISITLNFCLFINIIRILLQKLLSPDVGGNDQSQFKRLAKSTLLLIPLFGVHYM
VFVGFPMPSFSDCQIWFELCVGSFQGLVVAILYCFLNTEVQGELKRKWRSLDYIRYKKHD
KRMHSLTISRNGSEGVPQFHRDSRAQSIMQTETTMI*
>jgi|Xentr4|274737|estExt_Genewise1.C_...
MTEEAWRLLIYVTMLVTCPIKAAVDADDVITREEQIYLLSRAKRLCQTSMTTQATGAIRNT
SKGLSCPPEWDGIICWPRGSPNELVSVSCPEHIYDFNHQGFAYRKCGQSGTWIQVPGLNRT
WANYSECLTWVGSGHRAKEKELFERLRLIYTIGYSISLAALLLALCILCYFKRLHCTRNYI
HIHLFASFICRAGSIFLKDAILYSGENEGAALDEKIEFGLSKRTGLQWAGCKVTVTLFLYFL
ATNHYWILVEGLYLHSLIFMAFLSDRNYFWALIIIGWGLPAVFVSVWASVRVSLADTQYVQ
HLVGRLYILLQYTSPASSIVNFILFLNIVRVLASKLWETNNRKQDPCQQYGKLLKSTLVLMP
LFGVHYVLFMAIPYTEVTGLLWQIQMHYEMFFNSSQGFFVAIIYCFCNGEVQAEARKFWF
RRNLAVDLKQKSRMTSTGGSCHYGGVLTHTTNSVCLSMVARGKQMASLQSSAHLPGYIS
STVTSNSTTQQLGNVI*
>jgi|Xentr4|275038|estExt_Genewise1.C_...
MNETDSNFSCYDPSIVEYRYFGVTWGIVVSLVGTIGNVLTVLAYALDKKLQTRFNLLIVNL
SLADILYCTFLQPFSVDSYLHLYWRSGTTFCRVFGMLLFVSNSVSILNLCLIAVSRYILIANN
KLFDKIFCRLGVSLILLGTWVIGFASFAPLWYVFVLVPKVCTCSFHRIKGRPYTTILMAFYF
VIGLSCVGVFYFLIHRKVKSAAQALDQYKLKTKNTTKENVSGINSTDVGKYQDMDSGVD
TAVSSEVISEHLPSNKSTSQSAVTSHANDNEKLERRTLSQPKDSGSDFKKVTRMCFVVFIFF
VVSYIPFLLLNIFDAKNSAPQMLHMIAANLTWLNSCINPILYAAMNRQFRDAYKRVISLAL
GKIRRD*
>jgi|Xentr4|276243|estExt_Genewise1.C_...
MNGTEGPNFYIPMSNKTGVVRSPFDYPQYYLAEPWKYSALAAYMFLLILLGFPINFMTLY
VTIQHKKLRTPLNYILLNLVFANHFMVLCGFTVTMYTSMHGYFIFGQTGCYIEGFFATLGG

EMALWSLVVLAIERYVVVCKPMANFRFGENHAIMGVVFTWIMALSCAAPPLFGWSRYIP
EGMQCSCGVDYYTLKPEVNNESFVVYMFIVHFTIPLCVIFFCYGRLLCTVKEAAAQQQES
ATTQKAEKEVTRMVVMMVIFFLICWVPYAYVAFYIFTHQGSDFGPVFMTVPAFFAKSSAIY
NPVIYIVLNKQFRNCLITTLCCGKNPFGDEEGSSAASSKTEASSVSSSQVSPA*
>jgi|Xentr4|276281|estExt_Genewise1.C_...
MNSSEAIAFSGGLQSSEVGAEITLVLPLILSIICLVGMTGNLLVIAILFHDFRKGKCSVVNCL
VISLSATDLLLILFCLPVRIVTYAKQSWVFGSFICKSTEWFLHCCLIAKSFTLAAIGQARYKH
VITPPKFLSFSSRHVVLVMFFTWSLSLLLPLPHLIFTQLQVRQEGLFCLFDIPEYASNFMNVF
SKLYPLLAYVLPMVFSFCCYIKALRRRERRNRVPSPRVLSRRITSMLMSVNLAFDAMWLPE
WIVWMWARHSSFGTLHPPTALMVLAQVILFLNCTLNPGVFLAVSDEFREGLKNIWMVVK
CKTHNEEGLSRAGENGSDVVTSTIQSLQDLRTNSGLRSSQESKLKEEKVLPDVEHFWQDR
RNTTAGDDNDPMPWEHQEKP*
>jgi|Xentr4|277792|estExt_Genewise1.C_...
MRGNSSDLCESQLHLNPGVSPTHSAMMFSLGVLGNLIALVLLEHRRRGIRGKISLFHILAT
GLVITDLMGTCMLSPVVLSAYSSNLTLKALGGSDNFIVCHYFAFAMTFFSLATMLVLFAMA
LERAMAIGHPYFYEKFISKRCGLVMFPVIYSFCIFFCLFPAMGAGEYIQYCPGTWCFINMR
GQHIDGSTSNVYSTLYATLLLIVIIAVLTCNFIVIISLVRMHKRQKSRLGSLMVTKKERMSMS
EEIDHLILLSLMTIIFFICSVPFT
>jgi|Xentr4|277859|estExt_Genewise1.C_...
MQNGSRGGDEKDPEESEGSIERRRRDIQEIIHTATLVTCTFLLIIIFCLGSYGNLVVFLSFFDP
AFRKFRTNFDFMILNLSFCDLFICCISAPMFAFVLFLDSGKNVPDAFCFTFHLTSSGFIIMSL
KTVAVIALHRLRMVLGQQPNRTASFPCTLMLTVLLWTTSFTLATLATLRTRKSRICLPMYSL
INGEGKIILYLYVIDFTFCVAIVSVSYIMIAQTLRKNAQVRKCPIITVDTSRPQPFIGPAVEGV
HCAVPALYRNQNYNKLQHVQTHAYTKKLSQMPVPASRLQLVSAVNLSTAKDSKAVVTCV
VIVLSVLICCLPLGISLVQDVLTSNSSFILYQFELCGFTLIFLKSGLNPFIYSRNSAGLRRRVL
WCLQYVALGFLCCKKKTRLRAMGKGSLEVNRNKSSHHETNSAYMLSPKPQKKFVDQAC
GPSHSKDSVLSPKGSAGHQHYAQSSSTPINTRIEPYYSIYNSSPSQELSTPNSLQPVNSTFGF
AKSYIAMHYHTANDLMQDCESTSAKQIPVPSV*
>jgi|Xentr4|278169|estExt_Genewise1.C_...
MPTVNSTNCSHSSEFQYSLYSTTFIIIFVLGLLTNSIALWVLCSYINKKNKAIIFMINLAIADL
AHVLSLPLRIHYYINHTWPFGRFMCLLCFYLKYLNMYASIVFLMAISVQRYVFTINPFKAK
DWKRRYDVAISAVIWIVVGGACLPFPLLRNSGSSNKNDTCFADLGLRKTNKESMMAMLAI
AEMAGFIVPMAVIIYCTLKTKTQLKSEGLQCHNIIEKRKALRMVTTCAVVFFICFAPYHINF
FFYIMIQSKVIEHCGVYKFVMTLHPYTLCLASINCCLDPILYFFTAKEFQNEVAKHGSTVM
RGRLMSRESSSFVKE*
>jgi|Xentr4|278174|estExt_Genewise1.C_...
MATRFFLFVAWMACLIVGVCCQDTQTQQNFPDISNPSEELNQEQAHRIVQLDSIQNGGEL
NMSASNVLNLSPPPPSPCVSRAKIRHAFKYVTTILSCVIFLVGIVGNSTLLRIIYKNKCMRN
GPNVLIASLALGDLFYILIAIPIHIYKLLAEHWPFGVHICKLVPFIQKASIGVTVLSLCALSID
RYRAVASWNRIQGIGIPVWKAIELTLIWAVAIILAVPEAIAFNLVELDFRGQTILVCMLPLEQS
SDFMRFYQEAKVWWLFGFYFCLPLACTGVFYTLMSCEMLSIKNGMRIALNDHMKQRRE
VAKTVFCLVVIFALCWLPLHVSSILKKTVYDGRDPNRCELLSFFMVMNYIGINMASLNSCI
NPVALYFVSRKFKNCFQSCLCCWCHRPTLTITPMDEKCSAVKWKTNGHDLVLDRSSSRLT
NKYSSS*

>jgi|Xentr4|278183|estExt_Genewise1.C_...
MAVSLEKKLQNATNFFLMSLAVADMLVGILVMPVSLVTILYDYAWPLPRKLCPIWISLDVL
FSTASIMHLCAISLDRYVAIRNPIEHSRFNSRTKAMMKIAAVWTISIGISMPVPVIGLQDDSR
VFVNSSCVLNDENFVLLGSFVAFFIPLIIMVFTYCLTIQVLQKQATVFMYEEVPKQRRSSVN
CLKKENNTENISMIQNHEAASHLNSPINKEGALFRKGTMQSINNERRASKVLGIVFFLFLI
MWCPFFITNIMSVLCKEACDNELMVELLNVFVWVGYVSSGINPLVYTLFNRTYRRAFASY
IRCQYYNSKKTMLQQNQCQHNATSTAMYGKDLNLNSYRNGNELTSVDMDETEEAIEMQ
PGISELSINNSNVVSERVSCV*
>jgi|Xentr4|280289|estExt_Genewise1.C_...
MLLAKTPSLLLVQVITAGISFALTSLQDQCETLQHNSNVTGLACNASIDMIGTCWPRTAAG
QMVARPCPEYFQGVQYNTTGNVYRECHLNGSWAGRGDYTQCQEILKQEKKTKVHYHIAI
VINFLGHSISLCALLVAFILFLRLRSIRCLRNIIHWNLITAFILRNVTWFVMQLTLSHEAHDSN
VVWCRLVTIAHNYFYVTNFFWMFGEGCYLHTAIVLTYSTDKLRKWMFICIGWCIPFPIIVA
WAIGKLYYDNEKCWFGKKAGVYTDFIYQGPVILVLLINFIFLFNIVRILMTKLRASTTSETI
QYRKAVKATLVLLPLLGITYMLFFVTPGEDEISRVVFIYFNSFLQSFQGFFVSVFYCFLNSEV
RSAVRKRWHRWQDKHSIRARVARAMSIPTSPTRISFHSIKQSSAI*
>jgi|Xentr4|283242|estExt_Genewise1.C_...
MSSETTTNYLSKTDPCNVDSTFRYSLFTVTYSIIFVFGFIANCYVLWIFGKVYPAKNLSEIKI
FMINLTVADLLFLVTLPLWIVYYHYGGDWFMPSFLCNVAGCFFFLNTYCSVAFLGVISYNR
FQAVTRPVETAQSTARIRGICISAAVWILLFASSLYFLIFPGTNEVNINGQNYTRCFEGYSIDN
REPVAVIHFILVGAFFVVFLIILTCNLVIARTLLTQPVKSRKSSDMKHRALWMVVTVLAVFVI
CFVPHHIVDGPWTLTVLRMWHERDCEFRLTLNNAHQITLCLMSTNCMLDPIIYCFLTKKF
RKHLSERIQNIKSSRKLSRNTTDTNLETTLPFKEKLDNITSV*
>jgi|Xentr4|285135|estExt_Genewise1.C_...
MTSVWCLAAIVGFLIIFTIVGNVFVVIAVLTSRALKAPQNLFLVSLASADILVAALVMPFSLA
NELMGYWYFGNVWCDIYLALDVLFCTSSIVHLCAISLDRYWSVTQAVEYNLKRTPRRIKG
IIVTVWLISAVISFPPLISMDRDTEDDVYPQCKLNDDTWYILSSCIGSFFAPCIIMILVYIRIYQ
VAKLRTRTLSEKKPTRDGSSHTENGFSKGTTVKMPGDKENGHCPPRPSPPKATDVEDLELE
ESSMSESKRRKSSSKEDNTDSSKERKCSKGNSFSKQSSRLSRTSNKSMELFSSRKKKKRSS
VSRRKLSQAREKRFTFVLAVVMGVFVVCWFPFFFSYSLYGICREACEVPETLFKFFFWIGY
CNSSLNPVIYTIFNQDFRRSFKKIICIGRRKTFTH*
>jgi|Xentr4|286925|estExt_Genewise1.C_...
MADVQPRSGNESLQCKPDETYKYHVYTAIYSVVFIFGLIFNIAALYVFCFVSKKKGNVTIC
LLNLAVADLTFICFLPLRISYYGNNATWIFGDALCRITTYSFYFSMYASVLFLACLSCLRYFS
VVFPDSISVKTTIKLCACVWLFSGASTSPFFLSGTQIRENVTRCFEPSGQLSWTRVMYMNY
FALICGFVIPFLATVIFNGLLIRHIRQIPMEKKNIRKVIIMIVLVLSVFSLCFLPYHIQRTIHLHY
LVHHPNICSLHAVLQRTVVATLCLAVLNSCLDPLLYVFVGHGFK
>jgi|Xentr4|287329|estExt_Genewise1.C_...
MNFETTPSSNNSLYDSEDSDFDDFLNKGKPTSLSSFQTTALFILSVVLVLGVPGNALVVWIT
TCDMKRSVNTVWFLNLAIADLLCCLSVSFTIMEIVMGQWPLGLATCKLIPTLLLINMYASV
LLLTVISIDRWLLVAKPVWCQNKRTVIKAYLACATVWLLALILTSPSFVFRQIVVNIVEKHV
CVMHYNISKDHIEKVKQFIAIFRFLIGFIIPFVVITVCYGVLVRKVSERYNKSGKTMKVVTV
VIIGFFVCWFPYHIAGLIIATHSSNSDLYQNTLKIDPILVSFAIINSCINPIIYVLAGQDLKSKFR
KSIRSVVKNVLAEEESQSFDSKKTKSSSETKNTDTCV*

>jgi|Xentr4|289618|estExt_Genewise1.C_...
MNNSSLVNVSSTNETMMGSPYKTVEVVFIVIVAGSLSLVTIIGNILVMVSIKVNRHLQTVN
NYFLFSLACADLIIGVFSMNLYTLYTVIGYWPIGPVVCDLWLALDYVVSNASVMNLLIISF
DRYFCVTKPLTYPVRRTTKMAGMMIAAAWVLSFILWAPAILFWQFIVGGRTVPEGECYIQF
FSNAAVTFGTAIAAFYLPVIIMTVLYWQISRASKSRIKKGKKEAAPNQDPISPSNVQGKIVK
PNNNNVSANEDGLDHGKIQNGKVTSNSGIENCVQAEGEEKEISNDSTSVSVVNTKDEGA
AKDVPQASGSQANPKTENSKLTCIRIVTKSPKGDCSNATGTTVEIVPGANGRNGEDKQNIV
ARKIVKMTKQPPKKKLPPSREKKVTRTILAILLAFIITWTPYNVMVLINTFCAVCVPNTVW
TIGYWLCYINSTINPACYALCNTTFKKTFKHLLMCQYKNIGSAR*
>jgi|Xentr4|291771|estExt_fgenesh1_kg....
MVTVSNLKNPEDLVYIVLEVAIAVLSILGNVLVCWAVCINSNLQNATNYFVVSLAAADIAV
GVLAIPFAIAISTGFCATFHACLFIACFVLVLTQSSIFSLLAIAADRYIAIRIPLRYNSLVTSRRA
NAIIAVCWLLSFVIGLTPMLGWHKEVPPSGNTTCRSPMIECLFENVVTMDYMVYYNFFAC
VLIPLLLMLGIYLRIFMAARHQLKQMKMKVTCGERSRSTLQREVHAAKSLAIIVGLFALC
WLPLHIINCFTLFCQACNRPPLWIMYTAILLSHANSVVNPLIYAYRIREFRHTFRKILHQHLF
GQGPHLKTRTASANSLTYNGVDEETGITQIRSSSCNLGTHTNGSVCKLKEESRQNGCPSQH
SEKRTGSYRYSGHKKEMMEIS*
>jgi|Xentr4|292008|estExt_fgenesh1_kg....
MEDYLRLLYLNLSGRLGPGWEGEDPCTDSVGNVTFLVVAYSALIAVGLIGNSCLVFVIARQ
REMRNVTNIFIANLSCSDILMALVCLPVTVIYTLMNRWILGEALCKVTNFVQCISVTVSVL
SLVLIALERHQLIIHPTGWKPLPWHAYLAVAVTWAVSCFISLPFLSFTILTRHPFQNLSLPFDP
FIDHFVCTDSWPSENHRLAYTTCLLLFQYCLPLLLILLCYLRIFLRLRKRRDVVERAGGGD
AGGRKGGHRRVNVMLLSIVVAFGLCWLPLTVFNALFDWDHEQISACYHNLIFSLCHLAA
MASTCVNPVMYGFLNSNFQKEVKTILLRCRCAGGRDRYESFPLSTVSTEVSKASLQSVIT
NGNNV*
>jgi|Xentr4|293010|estExt_fgenesh1_kg....
MSTTTTSTCVARPFSYLCDTNAAWGIVLETLAAAGIVFSIVLMLALLIMVPRIGDYAKRAV
VPVQFIFLVATFGIFGLTFAFIVELTDQTCPTRFFLFGVLFAICFSCLLAHASKLVKLVRGGLG
ICWWVMLIMVLFLSLVQVVIAILYIVLSLVRGATPCSVFNTNTNYQQINQDFVLVLIYVYLL
MAITLIVSVISLCGPCKYWKRHGAHIYVTMFLSVGIWVAWICMLLRGNVELNQGNHNWD
DPVLAIALVANGWVFLMMYIVPELCLMTRCQPDTQKDCVQTQPRLLRQTIGVDNRVFTH
ENINQGQDSGRCSPVSSQNDATIAMRDLEQIKDFSIPRPQQRQNPYMQYRSHDLTSM*
>jgi|Xentr4|294710|estExt_fgenesh1_kg....
MCNQSVSCPFPDNYEAVLFPIIYSSVFIVGLPSNLIAIGVIVQLIRKKNILGIYLANLCASDLM
YIMTLPVWIVYTAKEDWLFSTLTCKIVGFFFNANLYSTISFLSCIATDRFVAMVFPLRSRIIRS
MKTAVVICVIVWLIILGSHLYFLTRDDVFTSTQNVELCYERYPMETWMAHLNYFRIFVVFL
IPLILLVFSYCSIIRVIYRTATLETDQKRKITGLLMSMTAIFVVCYLPYHVILFIRSYVSDLGY
CSCTLEKNVRPAYRITFALTSLSSALDPFLNIFVSEGVKRDLMVEIRALWFYLVVRRKEKSK
MRCLDVCDGNVQRNSGLLCTHQTKMQSRL*
>jgi|Xentr4|295110|estExt_fgenesh1_kg....
MTSTYLNISSNPSDKMSCLNMTLTDHQPELMPVLYSIIFTFGIIGNCLVIIVLCLQGDLKSVA
KIYIVNLAVADLVFLATLPFWATYYALGYNWLFGKVMCKISSSLLCLNLFASIFFISCMSVD
RYVAVVYPFSSQRRTKYQACLVSISVWILAILSSVPTFYFRNAFYINRLGAHVCAMDFPKE
KYSSWCVGMSLMKIFLGFCVPVSVIATCYLMIGIHLKRSKGPVINIQNRDRVQKIVTAIVM

AFLICWFPFHALTFLDALVRMDIISHCKIVTFIEKAMPFCICMGFSNSCINPLLYCFVGNQFR
EHFRHLFLSRISVNINSQLNSSRKGSDCKEQEPSGQSNNVVV*
>jgi|Xentr4|295262|estExt_fgenesh1_kg....
MDLSGTGLLRRTALLLVLAAALCSGISSINPDRSGDGRCQPIEIPMCKDIGYNMTRMPNLM
GHENQKEAAIQLHEFAPLVEYGCHSHLKFFLCSLYAPMCTEQVSTPIPACRVMCEQARLKC
SPIMEQFNFKWPDSLDCSKLPNKNDPNYLCMEAPNNGSEEAPRGSSMLPPIFRPQRPNSGH
EIYPKDPTGRTTCENSGKFHHVEKSASCAPLCSSAVDVYWSKDDKKFAFIWIAIWSILCFFS
SAFTVLTFLVDPLRFKYPERPIIFLSMCYCVYSVGYIIRLFAGADSIACDRDSGQLYVIQEGL
ESTGCTIVFLILYYFGMASSLWWVILTLTWFLAAGKKWGHEAIEANSSYFHLAAWAIPAVK
TIMILVMRRVAGDELTGVCYVGSMDVNALTGFVLIPLACYLIIGTSFILSGFVALFHIRRVM
KTGGENTDKLEKLMVRIGVFSVLYTVPATCVIACYFYERLNMDFWKILATQDKCKMDSQ
TKTLDCTMTSSIPAVEIFMVKIFMLLVVGITSGMWIWTSKTVQSWQNVFSKRLKKRNRSKP
ASVITSAGIYKKPQHPPKIHHGKYESALQSPTCV*
>jgi|Xentr4|295495|estExt_fgenesh1_kg....
MGDWRWGLLILGALLCFDAVTGKGIRAACNMPQLSLYCXXXXXXXXXKYVYTVTPFVS
KVLSDALTTAFLPAIYIIVFIVGLPSNAIALWVFFFRTKKKHPAMIYMANLALADLMFVIWL
PLKIAYHLNGNNWIYGEALCKVLIGFFYGNMYCSILFMTCLSVQRYWVIVNPISHTRKNT
KLALIVSITIWVVIMLGTIPLYLINQTLYLSDLRITTCHDVLPLDSATFDMFNYFLALAIGVF
FIPAILTAVVYTLMIKTLTASITDESIGKKRKRAIRLIIIVLVMYLVCFLPSNLMLVIHYGSLKN
SYSANLYAFYITALCLSALNSCIDPFVYYFVSKDFRDHVKNTFLCRSVRTVERMRISFSSMK
YSRKTNSYTTKSTNTESSSC*
>jgi|Xentr4|296993|estExt_fgenesh1_pm....
MANDSHAADNILQNVSPLTAFLKLTSLGFIIGVSVVGNLLISILLVKDKTLHRAPYYFLLDL
CCSDILRSAICFPFVFTSVKNGSTWTYGALTCKVIAFLGVLSCFHTAFMLFCISVTRYLAIA
HHRFYTKRLTFWTCLAVICMVWTLSVAMAFPPVLDVGTYSFIREEDQCTFQHRSFRANDS
LGFMLLLALILLATQLVYLKLIFFVHDRRKMKPVQFVAAVSQNWTFHGPGASGQAAANW
LAGFGRGPTPPTLLGIRQNANTTGRRRLLVLDEFKMEKRISRMFYIMTFLFLTLWGPYLVA
CYWRVFARGPVVPGGFLTAAVWMSFAQAGINPFVCIFSNRELRRCFSTTLLYCRKSRLPRE
PYCVI*
>jgi|Xentr4|297662|estExt_fgenesh1_pm....
MTPTSATQRRNEYYDHEIIIEHYNYTGKYKGNLSTDIKPTSIIFIIICCFIVLENILVLLTIWRT
KKFHRPMYYFIGNLALSDLLAGTAYTANILLSGPHTYKLTPVEWLIREGSMFVALSASVFSL
VAIAIERYITMLKMKLHNGSKSSRSFLLISGCWILSLFLGGLPIMGWNCIKQISACSTVLPLY
HKHYILFCTTIFCALLMAIVILYARIYFLVRTRSRSLTFKRNLARPSRSSEKSMALLKTVIIVL
SVFILCWSPLFIFLLLDFGCKVKTCPVLFKAEYFLSLAVLNSATNPIIYTLTNREMRRAFLK
MACCSHCSIFGSSSKVKRPIITGMEFSRSKSDNSSHPQKDEGEYPVTLMSSGNVTSSS*
>jgi|Xentr4|298117|estExt_fgenesh1_pm....
MGVIQTQKKMASKHFLLILYMLGPLGAASLNLTTPSVELQNLSAVPMGCSQDVQPIFYAL
CDLNAAWGIILEALAGLGIVCALVLAVIFLALAPSVIRDERKGSLAINFMFLFGVFGLFSLVF
AFIIAPNAAVCEVRRFLFGVLFALCFACLVAHSVRLNYLVLHNRGPGGGLIFLLAIGLFLVEA
VINAEWLLITNVRHNLSSTVSVGHPCQIDNQDFVTALVYVMFLILASLIVPCPVLCGHYLP
WKRHGRYIVVTALLSLSIWVTWIVMYVYGNEKLGHQNTWDDPVLAIALVSNGWVFIVC
YFIPEVVEMTRTGYGYETDTLNMIKRFEECPSSIIVENRAFSMENLEMTDQRETIKIQDKPV
SPYSNYCGLYPTLPLYPTEVETVNHVPLPRISTEPWRYHL*

>jgi|Xentr4|298264|estExt_fgenesh1_pm....
MKTSLQVLLLALFSKAVLLSSSDHNFVRKEIKFEGDLVLGGLFPINEKGGGMDECGRINED
RGIQRLEAMLFAIDQINRDSSLLPGVKLGVHILDTCSRDTYALEQSLEFVRASLTKVDEAE
YMCPDGSYAIQENSPLPIAGVIGGSYSSVSIQVANLLRLFHIPQISYASTSAKLSDKSRYDYF
ARTVPPDFYQAKAMAEILRFFNWTYVSTVASEGDYGETGIEAFEHEARLRNICIATSEKVG
RSNLKKSYDNAIKELLQKPNAKVVVLFMRSDDTRELLAAANRFNASFTWIASDGWGAQE
SIVKGNEHIAYGAITLELASHPVKEFDKYFQSLTPYNNHRNPWFKDFWQQKFQCSLQNKN
QQVKFCDKRLNINSSNYEQESKIMFVINAVYAMAYALHQMQRTVCPNTNILCDAMKVLD
GKKLYKDYLLKVNFTAPFIPPSNTDSIVKFDAYGDGIGLYNVFNFQYHNGKYSYLKIGQW
AETLSLDVDIIQWSKSLVPTSQCSDPCAPNEMKNMQPGDVCCWICIPCEKYEYLADEFTCI
DCGEGRWPTADLTSCFDLPEDYIKWEDAWAIGPVTIACLGFVCTCLVGGVFIKNNSTPLVK
ASGRELCYILLIGVFMCYCMTFFFIAKPSPVICTLRRLGLGTSFVVCYSALLTKTNRIARIFS
GVKDGAQRPKFISPKSQVVICLSLISVQIVVVSVWLMLEFPGTRRYTLPEKRETVILKCNV
KDSSMLISLTYDVVLVILCTVYAFKTRKCPENFNEAKFIGFTMYTTCIIWLAFLPIFYVTSSD
YRVQTTTMCISVSLSGFVVLGCLFAPKVHIILFQPQKNVVTHRLHLNRFSVSGTATTYSQSS
ASAHYTPTICNGGEVLDSTTSSL*
>jgi|Xentr4|299026|estExt_fgenesh1_pm....
MASRESVLLLLLLAGILLAAEWEGGGGGSGLVVSLPPGCKQDARPRGAARSAAAEAGKVL
CIGLGAGRSLPLGPLPNRTSTLILNNNKINELNNGSFEGLSFLERLDLRNNLISSIEPGADLA
NNRIGCLNADIFKGLTNLAKLNLSGNMFSSLAQGTFVNLVSLKSLEFQTDFLLCDCNLMW
MLRWIREKNITVRDTRCSYPKSLQGQPVTSLKQEQLTCESPLELPSFYMTPSHRQVVFEGD
SLPFQCMASYIDQDMQVLWYQDGKIVETDESQGIYVEKNTIHNCSLIASALTISNIQAGST
GNWGCQVQSRRGNNTRTVDIVVLESSAQYCPLERVVNNKGDFRWPRTLAGITAYLLCTR
YSAGSGIYPGNQQDDRRAWRRCDRGGFWAEEDYSRCQYANDVTRVLFMFNQMPLNLTN
AVATARQLLAYTVEAANFSDKMDVIFVAEMIEKFGRFAEKYKELSDVMVDIASNIMLADE
RVLWMAQKDAKACSRIVQCLQRIATYRLANAVQVYSTYSPNIALEAYVIKSALFTGMTCT
VFQKVAAADRTVHADFGRRDPDGSLDKQLSFKCNVSNTFSSLALKNTVVEASIQLPSALV
PKEKRERRAMDESFYKLQLIAFRNGKLFPTAGNSSHLGDDGKRRTVVTPVILAKLDGPNR
NIPSVPINITLRRFANGKDAVAAQWDFDLLNGQGAWKSEGCHIIASDENITTIQCSSLSNYA
VLMDLTGTEVYSHPVTLLHPVVYASAVVLLLCLLLLIISYLYFHSLVRVSLKSWHMLVNLS
LHVFLTCAVYVGGINQTRYASVCQAVGILLHYSTLATVLWVGVTARNIYKQVTKKAKRCQ
DPDDLPPPPRPMLRFYLIGGGIPIIVCGITAAANIKNYGSQPNAPYCWMAWEPSLGAFYGP
ASFIVFINCMYFLSIFIQLKRHPDRVFELKEQTEEQQRLAASESGEGNQQDSLSVSLVSTSA
LENEQTFQAQLLGASLTLFLFVSLWIFGALAVSLYYPMDLVFSCFFGVSCLSLGAFLVVHH
CMNREDLRRALMNTFCPGRSTYSVQVNVPPSNSNGTNGDAPKCTNSSAESSCTNKSASSL
KNSSQGCKLTNLQAAAAQCNTNPLPETAAPQLDNSLTEHSVDNDIKMHVAPVEVQYRPN
GHPSRHHKNRSKGHRSSRLTVLREYAHDVPTSVEGSVQSIVPKARQSYAEGHSRSRRAYL
AYRERHFNQCHQDSSDAGSTLPRYSRSIEKVASNNNKKDILKEPIAVELESQQKSYGLNLA
VQNGPAKDDIAEDPPTNADSNGNVRTGLWKHETTV*
>jgi|Xentr4|299983|estExt_fgenesh1_pm....
MPTVSVAKLASLGIIACVSLAGNLLFAFIALKDKNLHRAPYYLMLDLCVGDLIRSLLCFPL
VLKSISSGSQWTYSKRSCRIMAFGAVLLCFHAAFMLLSISLTRYMAIAHHRFYCKRMTGW
ACAIVISMAWILSTAMALPPVFEVGTYKFIREEDQCTFEHRYVKANDSLGFLLMLAFIVGA
THFIYMKLLFFIYDHRKMKPAQLTPAISQNWSFHGPGAAGQAAANWIAGFGRGPTPPTLV

GMRQSTQGHIKRLVVLEEFKQEKRLCKMFYVITFLFLVFWAPYLLACYLRVFLKASSLPQ
GYLTTAVWLTFAQAGVNPIVCLLLNKEVRMSLRAYIPCKSTQTQRDPYMDT*
>jgi|Xentr4|302710|e_gw1.2.56.1
SDGWADRDEVIEGYEAEANGGITMKLQSAEVMSFDTYYLGLRLDTNSRNPWFTEFWQH
RFQCRIPGHPQENRSFKKICKGNETLEENYVQDSKMGFVINAIYAMAHGLHDMHSALCPG
HVGLCDAMKPIDGSKLLEFLIKASFLGISGEDVWFDEKGDAPGRYDIMNLQQIEPNRYDY
VQIGAWHEGILSMDDYRIQANKSGIVRSVCSDPCSKGEIKVIRKGEVSCCWICTPCKENEF
VQDEFTCKACDQGAWPNPGLTGSCEPIPVKYLQWSDIESIIAVAFSCLGILITMFVTLIFILYR
DTPVVKSSSRELCYIILAGIFLGYICPFTLIAKPTTTSCYLQRLLVGLSSAMCYSALVTKTNR
IARILAGSKKKICTRKPRFMSACAQVVIASILISLQLSLVVSLIIMEPPMPILSYPSIKEVYLIC
NTSNLGVVAPLGYNGLLIMSCTYYAFKTRNVPANFNEAKYIAFTMYTTCIIWLAFVPIYFG
SNYKIITTSFAVSLSVTVALGCMFTPKMYIIIAKPERNVRSAFTTSDVVRMHVGDGKTPCRS
NTFLSIFRRKKPSTGNTK*
>jgi|Xentr4|302832|e_gw1.3.29.1
MDYYYSSNNYSWPEDPVFLFPVPVLTGITVTCILLFIIGISGNVMTMLVVSKYKDMRTTTN
LYLSSMAFSDLLIFLCMPLDLYRLWQYRPWNFGSSLCKLFQFVSECCTYSTILNITALSVER
YFAICFPLKAKVVITKGRVKLVISVLWAVSFVSAGPIFVLVGVEHENGTNPLDTNECKATEY
AIKSGLLTIMVWTSSIFFFLPVFCLTVLYTLIGRKLWRRKRETIGPHTSIRDKHNKQTVKML
AVVVFAFILCWLPFHVARYLFSKSFEAGSLEIALISQYCNLVSFVLFYLSAAINPILYNIMSK
KYRVAACRLFRLKQVSRKATYTTNDESSPAWTESNMST*
>jgi|Xentr4|304460|e_gw1.6.36.1
MPAHLAGFNRSAGEVVQLGNACAAGLAEEQLQHYGQFSSSVSIILAVLMALMVLATVLG
NALVILAFVVEKGLRTQGNFFFLNLAIADFLVGGFCIPLYIPYVLTGQWKFGKGLCKLWLV
MDYLLCTASVFNIVLISYDRFISVTKAVSYRAQKGMTRNAVLKMLIVWVAAFLLYGPAIIT
WEYIARTTILPEGECYVEFYYNWYFLMIASTIEFFTPFISVTYFNLSIYINIKRRTMMRNEEM
AQGQEHCELTFQGKRKEHLIFFVKPADRPYGEAKKQGGTLSPAMPSAVGNGAQELESPML
DLNINQDLPPLQVEVSAKKSQDCFYKSAENACSNMRPDMASSIANRFRLSRDKRVAKSLA
IIVCVFGLCWAPYTLLMIIRAACHGRCVQDYLYEISFWLLWLNSAINPVLYPLCHMSFRKA
FMKLLCPGKVKIHPHIFM*
>jgi|Xentr4|304495|e_gw1.6.391.1
FQDAASSTVLGDKVIGVSVENTKVSNLCEDIMFTFEHEPSPKTTWLSLRFPLTCWEQPEL*
STDGWSTYGCKTFPKENHTQCNCSHLTYFAVLMQYSSQTISEEHLVSLSVLTFAGCTISFLA
ALFTICWNCCTRYLQKTRITLINTHSNPTLNIHMNLLGAVLLLDLFFMSSAVLGAFDLPSVC
KGSGMLLHFTQLCMFTWMGIEGFNLYRLVVKVFDSTSFATKKLAVIGWGFPALLVLVIFLI
DFDNYGLYSINVDRPSSCNSRASICWLTEPLIHKVVNLGYFGIVLLFNCGMLTVMVVRVLH
LMPHNRGENTQYCVTLLGLIVMLGLPWGIVFFSFGAVYLPVQYLFSILNSLQGLFIFLWYW
ALSHPHVKDPSRSLDSSSATPASPRSDQSTLMSDHKKLLT*
>jgi|Xentr4|305630|e_gw1.10.119.1
MFHHQHDPWDTNSSTVTTSDLSPDESVNPWDIILCVTGTVMACENAIVIAILFYTPSLRAP
MFILIGSLALADLLAGLGLILNFIVIYVFNSELATLSSTGLLLAAFSASVCSLLAITLDRYLSL
YNALTYHTERTLTFTYTMLILLWALCICIGLLPIMGWNCVREPSSCSVLRPVTKNNAAVLA
VSFLLLFALMMQLYLQICRIAFRHAQQIAVQHQFMATSQASSTRKGVSTLSLILGTFALCWI
PFAVYSLIADSSYPMTYTYCLVLPAACNSVINPIIYAFRNPDIQKSLWLACCGCIPPRFLSGPR
TSSDV*

>jgi|Xentr4|307123|e_gw1.15.303.1
IWLNLQAKTTGYETHLYIFNLAIADLCVLLTLPVWVVSLVQHNQWPMGEMTCKITHLVFS
INLYSSIFFLTCMSVDRYLCVSLNGTAGQRRRKIIRRLVCVLVWLVAFVVSLPDTYYLKTVS
SPVTNETYCRSMYPEETFKEWLLGMEIVSIMLGFVIPFPIIAIFYCLLAWTLSSSSSSSGDQE
RRISGRLIVSYVVVFMVCWLPYHAMVILDVMSFLQLLPFSCFLDNFLYAALHITQCYSLLH
CCINPILYSFIHRNYRYEIMKAFIFRYSSKTGLTKLIESSKVSEAEYSAVDQIPK*
>jgi|Xentr4|307557|e_gw1.17.30.1
MNHSSVTEFILLGLSVTEKTRIGLFIFFLLIYILTILANVLIIILVYTNHKLHSPMYILLSSFSFL
EICYTAVTVPKMLSDLIHRNQSSISVAGSIYFISCLAQFYLCASLGVTECLLLAVMSFDRYM
AICDPLHYQLVMSSQLCLLLIIFSWAGGFMIVLIALIMICQLDFCGPRVVDHFFCDFAPLLEL
SCSNSSAVKTYISLCVVAAIIFPLAFVMGTYVCIIHVIFRIPSSAGRKKAFSTCSSHLSVVYTY
YGTLIILYVIPHHAVSSTSNKILSLMYTVVTPLFNPIIYSLRNQEIKEVLKHLLPGISKSKK*
>jgi|Xentr4|308011|e_gw1.18.273.1
MLIWDIVLSQSDIHTGSGLGAARYKRNVNLSPQFHSPTYQVSVPENKPAGTFVVQLRASD
PDEGESGKLEYSMDALFDSRSASYFSLDSNTGVVTTSGELDRETKSTHVFRVTVRDGGIPS
RTAMATLTVTVSDTNDHDPNFEQQEYRETVRENMEVGYEVMTVRATDGDSLGNANIAYR
LISGGREVFEIDPRSGVIRILGPVDREEIASYQLVVEANDQGHDPGPRSSTATVHITIQDEND
NAPQFSEKRYVARVSEDSSIESQLLVVTATDCDQGANAAVHYSILSGNSRGLFSIDPLTGAIF
LAGTLDFETGREYTLRVRAQDGGRPPLSNVTGLVIIQVLDVNDNAPNFVSGPFQASVLEN
APIGYSVLQVQALDADSGDNARLDYCLSGTTGPFSINNSTGWIMIQTELDREEIEFYNFIVE
VWDRGQPSLSSSASVSVQVLDVNDNAPEFTQSEYHARLNEDAAVGSSVLTVWAADRDAY
ATITYQITAGNTRHRFSISSLSDSQGLLTLALPLDYKLERQFVLTITASDGMRSDTARAIVNV
TDANTHRPVFQSSHYTVSVKEDQPAGTTVVVISATDEDTGENARIAYLAEEGLPQFAIDPE
TGAVTTTMELDYEDQVSYTLAVTAHDHGIPQKSDTTYLEILVIDVNDNPPVFQRASYKGS
VPEDSPPYTSVLQVSATDRDSGLNGRIFYTFAGGDDGDGDFTVEYTSGIVRTLRQLDRENV
VEYRLTVYAVDKGHPLPHRTPAEVLVTVLDVNDNAPVFAREELEVKVFENSPLGPPLARIT
ATDPDEGPNAQIMYQIVEGNIPEVFQLDIFSGELTALVELDYEERSEYTIVVQATSAPLVSRA
IVRVCLVDVNDNAPTLSDFRVVFNHYVGGGGSFPDGVIGRVPARDPDVSDVLTFNFLHGN
ELALLRLNTSSGELQLSHDLDNNRPFEATMNIEVTDGKHRVTAQCTLQVSIITDELLSHSIT
LRLGGISQHQFLSPLLPLFTQAVAQVLSISPQSVVVFSIKDDAEEPTTVQSDRQILNVSLLVQ
GAEEFLPSEKLRELLYLNRTLLGALAQQRVLPFDDNVCLREPCPNYMLCVSALRFDSSAPF
LASDTLLFRPILPVGGLRCRCPIGFAGDYCETEIDLCYTRPCGEHGICQSHEGGYTCQCEEA
YTGTHCEISLRSARCSTGLCKNGGMCVNLLVGGFYCECPPGGYDAPYCAVSTRSFHGGSF
LTFRGLRQRFHFTISLSFATRERNGLLLYNGRFNGKHDFIVLEITNEQIQLTFSAGEFTTVVS
PFVPGGVSDGQWHTVHLHYFNKPVVGQSGELLGPSDQKVAVVAIDDCDTEMSLQFGDML
GNYSCAAKGMQSGTKKSLDLTGPFLLGGIPTLPEGYPVTHRHFVGCMKDLVINNKAIDLE
DYIANNGTTPGCLAKKNQCDGNSCQNGGTCVNRWDGYSCECSLGYGGKNCEQEMLFPL
RFLGNGILSWEGLVLPLSIPWNLSLMFRTRQTDALLLKAHDKRNCTVTLQLSKGGLLLSL
HGSAGSLFSLSLHHVKLNDGIWHHITVEAKNDPAMPGTQLVILDADYGQEQVKSYFPNDL
LETTVSSLNIGGVISDGSGVQLGFRGCIQGVRVGGSLLSLLNAESLNAEKGCSFPDPCDSSP
CPKHSYCRDDWDSYSCACRHGFYGDNCTDACELNPCQHQSTCVKRTSYPHGYMCDCAS
GYYGTYCENRLDQPCARGWWGHPVCGPCNCDVNSGFDTDCNKTTGECRCKDNHYRPV
GASFCLLCDCYAIGSISRTCHPVTGQCPCKPGVIGQHCDLCDNPFAEVTMSGCEVNYDSCP
RAVESEIWWPRTRFGLPAAVSCPRGSVGTAVRHCDEHRGWLPADLSNCTSSAFITLKGLLD

TVNKNLSLLGSAHAQKGAQDLLSAMQAVKTLLDSDVRISYHLLSAILQQQSQESGFSLAA
TQDVHFTENVVKAGSMLLNLGTKKQWDNIQVSEAGTALLLRNFETYASTVAQNMKQTY
LSPFIINTPNIVVSVTQLDKLNFAGTVLPRYETLRGIKPSDQETTVILPRANFLPHTTYAPFAS
KDHEDKNNSRKKRDRQHWEKVIPGDNQAVVTVIIYHSLGLLIPQRFDPDKRSLRVPKRPVI
NSPVVSINIHEAEGGAFDQPITVQFRLLDTQDRSKPICVHWNHSLPIQLPGGGWSARGCEL
VFRNETHISCQCHHMTSFAVLMDMSHRENGEVLPLRAITYPCLGLTLGFLLLSLLFLFILSS
LHCNKHSIHRNLILALLLSQLSFMLGVNQADMQFACTFIAILLHFLSLCCFSWVFLEVLHL
YRRLTEVRDVNSGPMRFYYALGWGVPAFITGLAVGLDPEGYGNPDFCWLSVSDTLIWSFA
GPVAFVVSAGLFLCVLLGRVSCTAQREGFHKKGTVSGLHCSVTLLLIVSISWLLALLSVNS
DLLLLHYLYAGVICAQGPLVFLISVALNRDVHRALKMSLSKKRDSSVTTKSTFTSSHPGTN
CLDNQLYHMPFGSSHGSLHNSGRSHSYLPFVLRDEAGQTNCQSLAELHDLDGVFLGPKE
QSEDSDSESDISMDDDQSCSFASTNSSDSEEEFEGPELPCWEVLTSLNNTKMKTSPKGIIYF
CAEC*
>jgi|Xentr4|308576|e_gw1.19.291.1
MNEVPIALSNGTCDLFNKSWRIIDDYHITDTAETVILPSFIGIICSTGLVGNILVLITIIRSRRK
TIPDIYICNLAVADLVHIIGMPFLIHQWARGGEWVFGSPLCTIITSLDTCNQFACTAIMTAMS
LDRYMALVQPFRLTSLRTISKTIRVNVLLWAASFIVVFPVWVYSKVIQFKDGLESCAFDLTT
PNDVLWYTLYLTITTFFFPLPLILACYILILCYTWEMYHQNKSAGRYNNSIPRQRVLRLTKM
VLVLVGVYIVSSAPYHVIQIVNLQISQPSLTFYVSYYCSICLSYASSGINPFLYVLLSGNFRKR
LSDCTKTKIRMSERELNYIDTLKSSF*
>jgi|Xentr4|308657|e_gw1.20.227.1
MLQSLPFTFLLGSAGTPVTFNENGDAPGRYDIYQYQMTNKTAEYKVIGQWTNQLHLNVE
DMQWTSREHTQPTSVCSLPCKPGERKKTVKGVPCCWHCERCEGYHYQVDELTCELCPLD
QKPNANRTGCQLIPIIKLEWHSPWAVVPVFIAILGIVATTFVIVTFVRYNDTPIVRASGRELS
YVLLTGIFLCYAITFLMIGTPDMFVCSLRRIFLGLGMCFSYAALLTKTNRIHRIFEQGKKSVT
APKFISPASQLVITFSLISVQLIGVFIWFVVDPPHIIIDYGEQRTPDPINARGVLKCDISELSLIC
SLGYSILLMVTCTVYAIKTRGVPETFNEAKPIGFTMYTTCIIWLAFIPIFFGTAQSAERMYIQ
TTTLTISMSLSASVSLGMLYMPKVYIIILHPEQNVQKRKRSFKAVVTAATMQSKLIQKGND
RPNGEVKTELCESLETNSRLFDCLFKVLF*
>jgi|Xentr4|308792|e_gw1.20.157.1
MCRGSPGQVLVRVRRKLRGRHKRIKRSTNSSPQFQLHNYQASVPENEPAGTRVTSLRAVD
PDTGEAGRLEYFMEALFDSRSNDYFEIDSETGTVSTSQALDRETKDTHVLKVTATDFGSPR
RSATTYLTVTVSDTNDHMPVFEQSEYRENIRENLEVGYEVMTIRATDGDALSNANMLYRL
LGGSEGASAVFEIDPRSGIVRTRAPVDREAVADYQLIVEANDQGKDPGPKSATATVYIYVE
DENDNYPQFTEKRYVVQVPENVSPNSHILQVQATDRDKGNNAIVYYSIVSGNIKGQFYIHS
FTGNIDVIHPLDYEAIREYSLRIKAQDGGRPPLSNTTGMVTVQVVDVNDNAPIFVSTPFQA
TVLENVPIGYSVVHIQANDADSGDNARIQYRLTDTPPNFPFVINNNTGWITVSTELDRETV
ELYTFGVEARDQGEPAMTSSASVTITVLDVNDNNPTFTEKAYHLRLNEDAAVGSSVLTMT
AVDRDVNSVVTYQITSGNTRNRFAITSQSGGGLITLALPLDYKQERQYVLTVTASDGTRSD
TVLVFINVTDANTHRPVFQSSHYTVSVSEDKPIGTSIVTISATDEDTGENARISYTMEDNIPQ
FRIDPDTGTITTLIELDYEDQASYTIAVTAKDNGIPQKSDTTYVEILILDANDNMPQFMRDR
YVGSVFEDVPLSTSVLQISATDRDSGLNGRVTYTFQGGNDGDGDFKIEPTSGIIRTNAKLD
RENVPVYNLKVYAVDRGNPSLKSPVDILVTVLDINDNAPVFKSNELDIYVEENSPVGSVVA
RITASDPDEGTNAQIMYQIVEGNNPEVFQLDLFSGDLTALIDLDYEVKTEYVIVVQATSAPL

VSRATVHVKLLDKNDNDPVLQDFEIIFNNYVTNKSNSFPTGVIGKIPAYDPDVSDTLHYSFI
QGNELNLLILDPSTGELKLSRDLDNNRPLEALMRVSVTDGVHSVTAHCTLRVTIITDDMLT
NSITVRLENMSQEKFLSPLLSLFVEGVATVLSTTKDGIFVFNIQNDTDVSSNILNVTFSALLP
GGVRNKFFHSEDLQEQIYLNRTLLTMISTQRVLPFDDNICLREPCENYMKCVSVLKFDSSS
PFISSNTVLFRPIHPINGLRCRCPPGFTGDYCETEIDLCYSNPCGKNGLCRSREGGYSCECH
EEYTGEHCEVSSRSGRCVPGVCKNGGTCINLLIGGFKCECPPGEFERPYCEMTTRSFPPQSF
ITFKGLRQRFHFTISLIFFQEDSIVLLVHKVHIPDLHSFGLGDRIDGLVMFSCSA*ETTTKVTP
YVEGGVSDGQWHSVQVQYYNKPNIGRLGIPHGPSGEKVAVVTVDECDTAVAIRFGNLIKN
YTCAAQGTQTGSKKSLDLTGPLLLGGVPNLPEDFPVQNRQFVGCMRNLTIDNKPIDMASFI
ANNGTLAGCAAKKNFCDTSFCQNGGTCVNKWNTYYCECPLHYGGKNCEQAMPFPQHF
QGEGIVFWTELDITVTVPWFIGLMFRTRQSSGMLMQANAGPSSRINIQISNSNIQFEVYSGY
SRIASMKITQTRVSDGEWHHLLIELKSSKDGKDIKYVAIMYLDYGMYQSSVEIGNELPGLK
LKSIFVGGLSSNDGGVDQGFNGCMKGVRMGETSTNIATLNMNQATVIKVDSGCTMHNPC
DSNPCPYNSECSDEWGSYTCHCEPGYFGRDCLDVCSLNPCEHVSTCTRKPSSSHGYTCEC
GANYYGQYCENKVDLPCPRGWWGNPICGPCNCEVSKGFDPDCNKTNGECRCKENYYRP
KESDTCYPCDCFPSGAYARGCDMETGQCPCKPGVIGRQCNRCDNPFAEVTNQGLIYNGCP
KAFEAGIWWPQTKFGQPAAVPCPKGSVGNAIRHCSEEKGWLPPELFNCTTLSFLELKTLN
EKLHRNETTLDGEKSIQIAKVLQNATNHTKSFYGNDIRTGYQMLVKVLKYESQQHGFDLA
ATRDVEFNENVIYAGSAILDHKNKHHWDQIQRTEAGTAHLLKHYEDYANNVARNMKKTY
MKPFVIVAPNIIIAVDIFNKANFTGAKVPEFNEIRDYPKDLESSVLFPDTLFKPPEINVLPTFK
PSNQKISSKDDEFDPNLSKRKKRQPDPSSQYAVAMVIVYRSLGQLLPESYDPDRRSLRVPN
RPIINTPIVSTTVHNEDDPLPTQLEKSIILEYTMLETEERTKPVCVFWNHSIIVGGSGGWSSK
GCELISRNKSHVTCQCNHMTSFAVLMDISRRENGEVLPLKIITYTSVAVSLVALLLTFILLVII
RTLRSNIHNIHKNLVAALFFSELVFLIGINQTENQFVCTVIAILLHYFYMSTFAWMFVEGLHI
YRMLTEVRNINSGHMRFYYVVGYGIPAIITGLAVGLDPQGYGNPDFCWLSVQDTLIWSFA
GPIAIVVTINTIIFILAVKASCGRRQRAFEKSGILSLLRTAFLLLLLISATWFLGLMAVNSDTM
TFHYLFAIFSCLQGLFIFFFHCIFNKEVRKHLKNTFTGKKPLPDDTATTRATLLTRTLNCNNT
YMDDSNMYRTAIGESTASLESTVRDEAAQKLSMSSGQTRAGVTEVDSSIYHRNPRIYHYFI
CIGHDSDTDSEDSSDEHSSLYASSHSSDSEEDGIDTEKKWKQTSPKNNERGPVHSTPKVDS
FPNHVKPYWPADCMTASDSEDQGGKSKLKVETKVNVELHNENKANHCGEAQQDKENE
ANNKESKLSSNHNNHQPELRKGILKNKVTYPPPLTDKNLKNRLREKLSDYNQSTISSRTSS
LCSNDGVRPLTDCGVTIKNPRRDHSREQVNGMAMSVQTGPLNGETSDSEYVLHGYLHE*
>jgi|Xentr4|309088|e_gw1.21.5.1
MEHNHIYNTSLLSHKYGCGLGYVPVIYYSILLCLGLPANILTVIILSQLVARRQKSSYNYLL
ALAAADIMVLFFIVFVDFLLEDFILNKQMPQMLDKIIEVLEFSSIHTSIWITVPLTIDRYIAVC
HPLTYHTVSFPARTRKVIVSVYITCFLTSIPYYWWPNIWIEDYTSTSVHHILIWIHCFTVYLV
PCSIFFVLNSIIVYKLQRKSNFRLRGYSTGKTTAILFSITSIFAILWAPRIIMILYHLYVSPIHNS
WLVHIVTDIANMLALLNTAINFFLYCFISKRFRTMAAGTLKAFFKCQKQPVQFYTNYNFSI
TSSPWISPANSHCIKMFVYQYDKNGKPLKISP*
>jgi|Xentr4|309156|e_gw1.21.10.1
MKTHPAVILLLFFVIGYGSSENSTTSRGCGLDIFPRYVYLCDLDAIWGIVVEAVAGAGVLTT
LLLMLILLVRLPFITEKEKKSPLGIHFLFLLGTLGLFGLAFAFIIQEDEAICCTRRFLWGVLFA
LCFSCLLAQGWRIRRLVRHGKSPSGWHMVGMVICLMLVQVIIAVEWIILTVVRDRKLACS
YEPMDFVMVLIYVMVLMVATLGLSFFTLCGKFQKWKKNGICLIITTLFSVLIWVAWMTM

YLYGNNELKRSDKWNDPTLAIALISSGWVFLIFHAIIEVHCTILPAPQENTTNYFDTSQPRM
RETPFEEDIQLPRSYMENKAFSMDEHNAGKTSTGGFRNGSLGSRPSAPFRSNVYQPTEMA
VVLNGGTIPTAPPSYTGRHLW*
>jgi|Xentr4|309173|e_gw1.21.312.1
MVSRSQGNVSGFIIQGFSDTPELHISLFVLFLVIYLIILLGNLIVFLVISCNPHLHTPMYIFLLN
LSLIDISFSSNVLPNLLHILLTQQNNISFLGCMTQMFLFAFLAASEYLLLTAMAYDRYVAICD
PLHYIARMSRKHCAGLITAAFTGGFVDAVGLIVLMSKLSYCASHLISHFFCDVAPLLKLSCS
STFSVELLIYIVGTLLFFSSFLLTLTSYIFIISAILKIQSSEGRQKAFSTCASHLACVITLYGTLF
CLYMRPTTSYSVKRDKYFSLLYIALGPVLNPLIYTLKNRDFQYYLNKLRQKSLFLLF*
>jgi|Xentr4|309501|e_gw1.22.66.1
MNITKDTDSITCNNVLWLSSYCTMDRNTSSAAEAQNPFQLPTFSPAAKARVIITFVIFALSA
FCNLAVVWASTNTSRKKRSHVRILILNLTTADLLVTFIVMPLDAIWNITVQWQAGDLACRI
LMFLKLLSMYSCAFVTVVISIDRQSAILNPLGISEAKKRNKIMLSVAWLMSILLSLPQLFLF
HTVTITEPQNFTQCTTRGSFQEHWQETVYNMVSFVCLFLLPLLIMISCYSRILIEISRRMSK
GALSSKEVYLRRSKNNIPKARMRTLKMSIVIVSSFIICWTPYYLLGLWYWFYPEAMEERVS
QSLTHILFIFGLVNACLDPITYGLFTIHFRKGLHRYCRGGRASDLDTSSSVTGSFRCSMSSFR
AKKIVLNQELQVMQGYNGSSNNSEFRTNGLNSSCL*
>jgi|Xentr4|309825|e_gw1.24.685.1
MHKVNQTSGISFLLLGFQNSQIINEFLFVLFLWIYIVTLFGNLLIIILVITVSALRSPMYVLLS
QLSLADVLLSTSITPNVLRLLLNGGGTISATGCITQFYFYGVAGSLEIYSLTAMAYDRYLAIC
SPLHYVSIMGFRLCLYMRLSSWGLALIVSLVMNLLIFNLQFCGPFVIDYYFCDFTPIIELSCT
DYKAVELTQIILAIPFVLLPFCFIIFSYVSIGLAILRISSTEGRHKAFSTCSSHLIVVCMYYGTII
IVYMVPSKQRNFNIKKILSLLYTAGTPCFNPIVYSLRNTEIKVALWKCMSNQISSFILNLSNR
KGD*
>jgi|Xentr4|309832|e_gw1.24.570.1
MKNHSTVSEFFLLGFQHLNNFKILIFSLILLIHILTVCENALVIALVTVSRGLQSPMFFFLQQL
SFSDLLESMVIVPTLLSTVINEGAKIPLIGCIVQLYLFGVSEALQSFLLTVMSYDRYLAICNP
LRYSSVMSHRVCVKLIAISWLLALSFIPVTVIAAATQEFCNRNTINHFFCDYFPLLELSCSDT
SLARILAIKVSGPVVVFLFILIIGSYICIAHEILKIVSSIGRQKAFSTCSSHLAVVSIFYGTLIGIY
AIPTRNQSQTINKLLSLLYTVVTPCINPMIYSLKSADLKKAIKIIMQ*
>jgi|Xentr4|309856|e_gw1.24.651.1
MKANWTTVSHFTILGFYELVNFKGILLPGLLLIYLWTVAGNLTIIMLVTFCQSLYSPMYFFL
GHLSCCDLLLTTVIVPLMLDSIVREVTITFVGCLSQFYLFGSLAATECFILSVMSYDRYLAIC
QPLHYSSAMNLKRCFHLAFWCWVLAFMLMLLTAVQVGRLKFCGPNVIDHFFCDLAPLLQ
LSCSDTAFVEAENLIGGLPASILPLAFITATYIIILRAILRIPSSTGKQKAFYTCSSHLTVVCVY
YGSLIVAYLFLSKGHSLSLNKVLSLLYTVLTPLLNPVIYSLRSKEIKRALCKLCKH*
>jgi|Xentr4|309886|e_gw1.24.107.1
XLWIYIVTLAGNLLLIMLVVRFPALRSPMYVLLSQLSLADILLTSNITPNLLWLLLSGDGRIC
FTDCITQFYFNGVSTAIECLLLTVMSYDRYLAICNPLRYGSIMNFRLCLFMSFSSWGSSFMV
ALVLERLVSNLKFCGPFTLDHYFCDFSPLLELSCSSIKAVQIIDIILTIPFLLFPFCFIIYTYVAIG
LAILRISSTEGRHKAFSTCSSHLIVVCTYFGTLMTVYMVPSKGHGFNMSKALSLLYTSGTP
LFNPIIYSLRNNDIKITLFKCVSNVMTLFTHNETL*
>jgi|Xentr4|309897|e_gw1.24.39.1
NQTMAMELVLLGFGDLYQMKGLLFILFFLIYFVTITGNVLIIGLIVTSSTLHSPMYFFLCNLS

LCEIIFTSNIVPNMLYVIWRKVGTMSMYGCFFQFYLYCSSGSVECLLLTVMAFDRYQAICN
PLRYSSVMHSRACNCLVLGAWLFGFTIMSIITLTLLSNQSCSSKVIDHIFCDLGPILDLSVIDI
HIIQNEALFIVITLGLFPFGLIIGSYGAIFCTILRIPTKTGRRKAFSTCSAHLVSVCTYFGTLFTI
YLLPSAERSVQLNKVLSLLYIVVTPLFNPIIYSLRNQEMRASFGSYYYKVGGCITI*
>jgi|Xentr4|309904|e_gw1.24.686.1
MHKVNQTSGISFLLLGFQNSQIINEFLFVLFLWIYIVTLFGNLLIIILVITISKLRSPMYILLSQ
LSLSDVLLSTSITPNFLWLLLNGGGTISATGCITQFYFYAVSLVSEFYLLTFMAYDRYLAICSP
LHYASIMGFRLCLYMSLCSWGVALILALIANLLTFNLQFCGPFVIDHYFCDFTPLIKLSCTD
YKALELTDIILAIPFMLLPFCFIIYTYVAIGLAILRISSTEGRHKAFSTCSSHLIVVCMYYGTL
LIVYMVPSKQYNFNINKILSLLYTVGTPCFNPIVYSLRNNEINVALRKFMSNFFFILNLRNR
KRRLKLLW*
>jgi|Xentr4|309911|e_gw1.24.571.1
MRMQNRSMVPEIFLLGFQHVDNFKILIFSLILLIHILTVSENFLVIALVQISQNLQSPMFLFLQ
QLSFSDLLQSEVIVPTLLSTVMNEGAKISLIGCIVQLYLFGITEALQCFLLTVMSYDRYLAIC
NPLRYSSLMSHRVCVKLIAISWLLSLSFTLVAQIPAATQEFCNQNTINHFFCDYFPLLELSCS
DTSLARILTITVSGPVVVFPIILIIGSYICIAHEILKIVSSIGRQKAFSTCSSHLAVVSIFYGSLIGI
YVVPTRNQSQTISKLLSLLYTVVTPFINPMIYSLKSADMKNAIKNIIQ*
>jgi|Xentr4|309915|e_gw1.24.539.1
MLERNQTGVTEILLLGFQNVGNAKIPLFLLIILLYTMTVCESSLVIALVWSTKNLQSPMYFF
LQQLALSDILQTSTVVPAMLRVIIYEGATMALTDCITQFYFFAASEAFQCINLAVMSYDRYV
AICNPLRYSSIMTLKVCVQCILWSWLLAFSVILITANAAATQHFCDKDIIDHFFCDFFPLLEL
SCSDTFFVRIEVTLLSVPVVFTPFILIIGSYICIAHEILKIVSSIGRQKAFSTCSSHLAVVSIFYG
TLIGIYVVPSKQRLMTISKLLSLLYTLIIPFVNPMIYSLRNKDIIERLKAILVSGKQKGINI*
>jgi|Xentr4|309919|e_gw1.24.432.1
MHKVNQTSGISFLLLGFQNSQIINEFLFVLFLWIYIVTLFGNLLIIILVITVSALRSPMYVLLS
QLSLADVLLSTSITPNFLWLLLNGGGTISATGCITQFYFYCAPASSELLLLTAMAYDRYLAIC
SPLHYASIMGFRLCLYMSLCSWGVALTLCFFINLRTFNLQFCGPFVIDHYFCDFSPLIKLSCT
DYKAVELTDIILAIPFVLLPFCFIIYTYVAIGLAILRISSTEGRHKAFSTCSSHLIVVCMYYGT
MIIVYMVPSKGHNFNINKILSLLYTVGTPFSNPIVYSLRNNEIKIALWNICQIENLCALFKQQ
NGY*
>jgi|Xentr4|309921|e_gw1.24.424.1
MHKVNQTSGISFLLLGFQNSQIINEFLFVLFLWIYILTLFGNHLMIVLVITVSALRSPMYVLL
SQLSLADVLLSTSITPNFLWLLLNGGGTISATGCITQFFFYNASTASELYLLTAMAYDRYLAI
CSPLHYASIMGFRLCLYMSLSSWGLGFLLSVFISILTFNLQFCGPFVIDHYFCDFTPIIKLSCT
DYKAVELTDIILAIPFVLLPFCFIIYTYVAIGLAILRISSTEGRHKAFSTCSSHLIVVCMYYGTL
IIVYMVPSKGYNFNTKKMLSLLYTVGTPCFNPIVYSLRNKEIKAAFQKHMSN*
>jgi|Xentr4|309936|e_gw1.24.660.1
MHKMNQTSGISFLLLGFQNSQIINEFLFVPFLWIYILTLFGNLLIIILVITVSALRSPMYVLLS
QLSLADVLLSTSITPNFLWLLLNGGGTISATGCITQFYIFGVSASSEFYLLTVMAYDRYLAIC
SPLHYVSIMGFRLCLYMSLCSWGVALLLSLFFNLLTFNLQFCGPFVIDHYFCDFTPLIKLSC
TDSKAVELTDIIVGIPFMLLPFCFIIYTYVSIGLAILRISSTEGRHKAFSTCSSHLIVVCTYYGT
LIIVYMVPSKAHNFNINKMLSLLYTVGTPCFNPTVYSLRNNEIKAALRKFI*
>jgi|Xentr4|309937|e_gw1.24.652.1
MNNHSLVNEIFLLGFQHLNNFKLLTFSLILLIHILTVVYENALVIALVTASRGLQSPMFFFLRQI

SFSDLLLSVLIVPTLLSTVMNEGAKIPLIGCIVQLYLFAVSESLQCFLLTVMSYDRYLAICNP
LRYSSLMNHKLCVKLILISWLFSLSATSVVVISAATQEFCNQNTINHFFCDYFPLLELSCSDT
SLARILIMTVSVPSVLSPFILIIGSYICIAHEILKIVSSIGRQKAFSTCSSHLAVVSMFYGTLIVT
YVVPSRNQSQKIGKLLSLLYTVVTPFINPMIYSLKSADMKNALKNIKPNGISKII*
>jgi|Xentr4|309946|e_gw1.24.513.1
MHKVNQTSGISFLLLGFQNSQIINEFLFVPFLWIYILTLFGNLLIIILVITVSALRSPMYALLSQ
LSLSDVLLSTSITPNFLRLLLDGGGTISSSGCITQFYFYCVSAGSEFYLLTVMAYDRYLAICF
PLHYVSIMDSRLCLYTSLCSWGLGFILGLVMNRLTFNLQFCGPFVIDHYFCDFTPLIKLSCT
DYKAVELTDSILIIPFLLLPFFFIIFSYVSIGLAILRISSTEGRHKAFSTCSSHLIVVCTYYGTM
AIVYMVPSKGHNININKILSLLYTVGTPFFNPTVYSLRNKKIKAVLRKYI*
>jgi|Xentr4|309947|e_gw1.24.505.1
DVLLSTSITPNVLWLLLNGGGTISATGCITQFYFYGAAGSTEFYLLTAMAYDRYLAICSPLH
YVSIMGFRLCLYMSLCSWGLALILSLFFNLLTFNLQFCGPFVIDHYFCDFTAIIKLSCTDYKP
VELTQIILAIPFMLLPFCFIIFSYVSIGLAILRISSTEGRHKAFSTCSSHLIVVCMYYGNMAIVY
MVPSKGHNFNINKILSLLYTVGTPCFNPIVYSLRNNDIKVALYKYMSNQISFFTLNLSRRKL
Y*
>jgi|Xentr4|309954|e_gw1.24.598.1
MRMQNHSMVFEIFLLGFQHLNNFKILMFSLILLIHILTVYENALVIALVTVSRGLQSPMFFF
LQQLSFSDLLQSEVIVPVMLKTVMNEGTKIPLIGCIVQLYFFGATEALQSFLLTAMSYDRYL
AICNPLRYSSLMSHRVCVKLIAISWLLALSVTSVVVIAAATQEFCNQNTINHFFCDLFPLLE
LSCSDTSLARILIMTITVPAVLLPFMFIIGSYICIAHEILKIVSSIGRQKAFSTCSSHLAVVSIFY
GTLIGIYVVPTRNQSQTISKLLSLLYTVVTPFINPMIYSLKSADMKNAIKNIIQGGLPWS*
>jgi|Xentr4|309976|e_gw1.24.48.1
MHKVNQTSGISFLLLGFQNSQIINDFLFVLFLWIYIVTLFGNLLIIILVITVSALRSPMYALLS
QLSLADVLLSTNITPNFLWLLLNGGGTISAIGCITQFYFYCVSASSEFYLLTAMAYDRYLAI
CSPLHYASIMGFRLCLYMSLCSWGLATMLSVLISLLTFNLQFCGPFVIDHYFCEFAPLLKLS
CTDYKAVELTDIIIGIPFMLLPFCFIIYTYVSIGLAILRISSTEGRHKAFSTCSSHLIVVCMYCG
TLIIVYMVPSKAHNFNINKILSLLYTFGTPCFNPIVYSLRNNDIRVALRKYISNQVSFYTTFK
QQKRRSQLLF*
>jgi|Xentr4|309978|e_gw1.24.352.1
MNQTVPSFLLSGFQNIQTVNTLLFVVILCIYILTLVGNVLLIILVVKVPALRSPMYVLLSQLS
LADILMTTNITPNLLWLLLRGGGKVLVTDCLAQFFLFGVSTSIECFLLTAMSYDRYLAICNP
LRYSSIMDFRVCLNMSLWCWGLSFMVALVLDLLMSHLEFCGPFLLDHYFCDFSPVLKLSC
SDIKVLELTDIIFTIPVILLPFCFIIFTYVAIGLAILRISSTEGRHKAFSTCSSHLIVVCTFYGTLI
TVYLVPSKDHTFNINKTLSLLYTVATPFLNPVIYSLRNKEIKVALHKCMSSLFMRLFHSLHE
CTNNSSRLRKTLLRFS*
>jgi|Xentr4|309985|e_gw1.24.679.1
MHKVNQTSGISFLLLGFQNSQIINEFLFVLFLWIYIVTLFGNLLIIILVITVSALRSPMYALLS
QLSLADMSLSTSITPNLLKLLLNGGGTISATGCITQYYFYCASAGSEFYLLTAMAYDRYLAI
CSPLHYASIMGFRLCLYMSLCSWGLALILSMTMTLLIVNPQFCEPFLIDHYFCDLSPLLEHL
CTGYGVVKRTQIILIIPFMLLPFCFIIYTYVAIGLAILRISSTEGRHKAFSTCSSHLIVVCMFYG
TLIIVYMVPSKGYNFNINKILSLLYTVGTPFFNPLVYSLRNNDIKVVLFKHMKKLNLFRRPL
YTQKCVR*
>jgi|Xentr4|309989|e_gw1.24.580.1

MQNHSMVTEIFLLGFQHLHNFKNLIISLILLIYLPIIFENAFVITLIRINQDLQSPMFFFFQQLS
FSDLLLSVVIVPTLLRTVMDEGTKIPLIGCIVQLYFFCATETLQSLLLTVMSYDRYLAICNPL
RYSSLMSHRVCVKLIAISWLLALSVTSVVVFSVATQEFCNQNTINHFFCDLFPLLELSCSDT
SLAWILAITLSFLLTVFPFIFIIGSYICIAHEILKIVSNTGRQKAFSTCSSHLAVVSIFYGTLIVT
YVVPTRNQSQNISKHLSLSYTVVTPFINPLIYSLKSTDIKNALKNIMQ*
>jgi|Xentr4|309997|e_gw1.24.30.1
MNEENQTTVADFLLLGFNDLYSFRDILFICLFLIYLATVSGNLMIIVLVTFSPRLDSPMYFFL
GHLSCCDLLLTTVIVPLMLDSILTEVTISFVGCFAQFYLFGSLATTECYILSVMSYDRYLAIC
DPLHYSSAMNLKRCFHLAFWCWVLAFLLMLLTAVQVGRLKFCGPNVIDHFFCDLAPLLQ
LSCSDTAFVEAENLIVGLPASILPLAFITATYIIILRAILRIPSSTGKQKAFYTCSSHLTVVCVY
YGSLIVAYLFLSKGHSLSLNKVLSLLYTVLTPLLNPVIYSLRSKEIKRALCKLCKH*
>jgi|Xentr4|309998|e_gw1.24.433.1
MHKVNQTSGISFLLLGFQNSQIINEFLFVLFLWIYILTSFSNLIIITLVITASSLKCPMYILLSQL
PLSDILLSTSITPYLLWLLLNGGGTISATGCITQFYFYCVSASLQIYLLTSMSYDRYLAICSPL
HYASIMGFRLCLYMSLCSWGVALLLGLFVNILTFNLQFCGPFVLDHYFCDFTSLIKLSCTG
YKSLQLTDIILAIPFMLFPFSFIIFSYVSIVIAILRISSTEGRHKAFSTCSSHLIVVCMYYGTLII
VYMVPSKGHNFNINKILSLLYTVGTPFFNPIVYSLRNNEIKSALLKYL*
>jgi|Xentr4|310026|e_gw1.24.514.1
MQNHSLVTEIFLVGFQHLHNFKILIISLILLIHILTISENVLVIALVTVSRGLQSPMFFFLRQLSI
SDLLESVVIVPTLLRTVMNEGAKIPLIGCIVQLYLFCATETLQSFLLTVMSYDRYLAICNPLH
YSSIMNPKLSLKLIVMSWLLALSLTLVAQIPAATQEFCNQNTINHFFCDFFPLLELSCSDTSL
AQILAVSLSFLVTVLAFLLIVGSYICIAHEILKIVSSIGRQKAFSTCSSHLAVVSIFYGTLIVTY
VVPTRNQSQTISKHLSLLYTVGTPFINPMIYSLKSADMKNALKNII*
>jgi|Xentr4|310042|e_gw1.24.117.1
MHKVNQTSGISFLLLGFQNSQIINEFLFVLFLWIYIVTLFGNLLIIILVITVSALRSPMYALLS
QLSLADVLLSTSITPNFLRLLLNGGGTISATGCITQFYFYCVSTVSEFYLLTAMAYDRYLAIC
SPLHYASIMGFRLCLYMSLCSWGLALLICMIMKLLIHNLQFCGPFYIDHYFCDSSPLLELSC
TDHKEVVKLTEIILMIPFTILPFCFIIYTYVAIGLAILRISSTEGRHKAFSTCSSHLIVVCTYYG
TLIIVYMVPSKGHNFNINKMLSLLYTVGTPCFNPVVYSLRNNDIKVVLLKQLQNGISLGDL
YTM*
>jgi|Xentr4|310043|e_gw1.24.492.1
MHKVNQTSGISFFLLGFQNSQIINEFLFVPFLWIYIVTLFGNLLIIILVITVSALRSPMYALLSQ
LSLADIFLSTNITPNLLRLLLDGGGTISATGCITQFYFYGVSGSSEFYLLTAMAYDRYLAICS
PLHYASIMGFRLCLYMSLCSWGLALILSLVMNLLTFNLQFCGPFVIDDYFCDFTSLLKLSCT
GYKTLEITDIILLIPFVLFPFCFIIYTYVAIGLAILRISSTEGRHKAFSTCSSHLIVVCMYCGTLL
TVNMVSFKGHKFNIKKMLSLLYTVGTPFFNPIVYSLRNKDIKVAVLKYM*
>jgi|Xentr4|310051|e_gw1.24.468.1
MKNHSMVTEIFLLGFQHLNNFRILTFSLILLIHILTTTENALVIALVTVSRGLQSPMFFFLRQL
SLSDLLQSMVIVPIMLRTVMNEGIKIPLISCFVQLYLFGITETLQCFLLTVMSYDRYLAICNP
LRYSSLMSHRVCVKLIAMSWLLALSFTPVAVISAATQEFCNQNTINHFFCDYFPLLELSCSD
TSLARILTIALCFPSLLFPFMLIVGSYICIAHEILKIVSSIGRQKAFSTCSSHLAVVSIFYGTLIV
TYVVPTRNQSQTISKLLSLLYTVVTPFINPMIYSLKSADMKNALENIIQ*
>jgi|Xentr4|310054|e_gw1.24.603.1
MCTDNKTAVTDIILGFHDFNKFKIPLFILFLLLYSLILCGNVLIIFLVSFNEHLQMSMFFFLKH

VGLADVLLTTNTVPMLLHIILNDEIIITLVGCICQLYIFGLSTVQCLLLAVMSYDRFIAICYPL
HYISIMSPNVCLLLVVGCWLSVFVFTTAESILMYQLDFCGFNHIDHFFCDFGPLVALSTSDT
SVLTLVDFAYSVFMCVPFTFIIGTYVCIFITIFRMNSKTAKKKTFSTCSSHLTVVCT*YGTLSI
VYIDPANENSATIKKFISLFYIVFAPFFNPIIYSLKNREIRETLRKYIMIQKTN*
>jgi|Xentr4|310122|e_gw1.24.493.1
MEVNNHSTVTEIFLLGFQHLSNFKILVFIVILLIHILTLYENALVITLVTVSRGLQSPMFFFLR
QLSFSDLLLSVVIVPTLLRTVINEGAKMSLIGCFKQFYLFGATEALQCLLLTVMSYDRYLAI
CNPLRYSSIMNHRVCVNLFVMSWLLALSLTPCNQNTINHFFCDYFPLLELSCSDTSLAQIL
ALTVAVPVILFPFLLIVGSYICIAHEILKIVSSIGKQKAFSTCSSHLAVVSIFYGTLIVTYVVPT
RNQSQTISKLLSLLYTVTTPFINPTIYSLKSADMKNALQNIIKNILPNER*
>jgi|Xentr4|310130|e_gw1.24.469.1
MNNHSTVTEIFLLGFQHLNSFRILVFFVILLIHILTLYENALVIALVTVSRGLQSPMFFFLQQL
SFSDLLQSEVIVPTLLRTVMNEGAKIPLIGCIVQLYLFAISEALQCLLLSVMSYDRYLAICNP
LRYSSIMNHKLCVKLFVMSWLLALSFPLVALIAAATQEFCNQNTINHFFCDYFPLLELSCSD
TFVAQILAITVSTPVVLFPFILIIISYICIAHEILKIVSSIGRQKAFSTCSSHLAVVSILYGTLIGV
YVVPTKNRSQTVKKLLSLLYTVVTPFINPMIYSLKSADMKNALKNIIQQKK*
>jgi|Xentr4|310135|e_gw1.24.362.1
MKNHSTVSEIFLLGFLHLNNFKILIFSLILLIHILTVYENALVIALVTVSRGLQSPMFFFLQQL
SFSDLLLSVVIVPTLLRTVMNEGAKISLIGCIVQLYLFGVTEALQCFLLTVMSYDRYLAICN
PLRYSSIMNHKFCVKLIAMSWLLALSFIPVTVISAATQEFCNQNTINHFFCDYFPLLELSCS
DTSLARILTITVSVPAILSPFMLIIGSYICIAHEILKIVSSIGRQKAFSTCSSHLAVVSIFYGTLIG
IYVIPTRNQSQTISKLLSLLYTLVTPFINPMIYSLKSTDLKKAIKIIMQ*
>jgi|Xentr4|310139|e_gw1.24.697.1
MHKVNQTSGISFLLLGFQNSQIINEFLFVLFLWIYIVTLFGNLLIIILVIKFSALRSPMYALLS
QLSLADVLLSTNITPNFLRLLLNGGGTISATGCITQLYVYGAAGSTEFFLLTAMAYDRYLAI
CSPLHYASIMGFRLCLYMSLCSWGIGFILSLFISLLAFNLQFCGPFVIDHYFCDFTPLIKLSCT
DYKAVELTDIILFVPCMLLPFCFIIYTYVAIGLAILRISSTEGRHKAFSTCSSHLIVVCMYYGT
LFIVYMVPSKGHNFNINKMLSLLYTVGTPCFNPIVYSLRNNEIKVGLRKYMSNQISFLYLI*
>jgi|Xentr4|310141|e_gw1.24.689.1
MHKVNQTSGISFLLLGFQNSQIINEFLFVPFLWIYILTLFGNLLIIILVITVSALRSPMYALLSQ
LSLADVLLSTSITPNFLWLLLNGGGTISATGCITQLYFYAGSTSSEFYLLTAMAYDRYLAICS
PLHYVSIMGFRLCLYMSLCSWGLALILCLIMKLLTFNLQFCGPFVIDHYFCDFTPLIKLSCT
DYKAVELTDIILIIPCMLLPFCFIIYTYVSIGLAILRISSTEGRHKAFSTCSSHLIVVCMYYGTL
IIVYMVPSKGHNVNINKILSLLYTVGTPFFNPIIYSLRNKEIKSVLLKYMRNQLYL*
>jgi|Xentr4|310156|e_gw1.24.435.1
MEVSNETRIREFIFTGLANSRIQGFILFVLFFFVYVITVVGNSGMVALVCNTSQLQTPMYCF
LGCLSMVDLCYSSVIAPKMLADLVSRVKSISFIGCALQFFFFAALAATESLLLSCMSYDRYV
AICRPLHYSLIITEKKSIGLILTATSIGFSQSSVQTVCIFSLRYCGWNQIDHFYCEVPPLLKLSC
SETFSCDLVTIFFFCSFGMASMMIIVVSYLLIVSSILGINSTTGRRKAFSTCSSHLTCVCIFYG
TTLFIYLRPPSRSFDKKDKVASVFYTMVIPMLNPLIYSLRNQEVMKALKTSIRCSSSL*
>jgi|Xentr4|310172|e_gw1.24.671.1
MHKVNQTSGISFLLLGFQNSQIINEVYFVLFLWIYIFTFF*NLLIIILVITVSALRSPMYALLSQ
LSLSDVLLSTSITPNFLRLLLNGGGTISATGCITQFYFYGVSGASEFYLLTAMAYDRYLAICS
PLHYASIMGFRLCLYMSLCSWGVAFILCLFINLLMFNLQFCGPFVIDHYFCDFAPLLQLSCS

DYTSLELTDMILALPFGMLPFCFIIYTYVAIGLAILRISSTEGRHKAFSTCSSHLIVVCMCYG
TLIAIYMVPSKGHNFNINKMLSLLYTVGTPCFNPIVYSLRNNEIKTAFWKYVPNRISLHVLF
KHQKED*
>jgi|Xentr4|310181|e_gw1.24.524.1
MNQTVTSFLLLGFQNNQSVNTLLFGIILWIYLLTLAGNFLLIILVVKVPALRSPMYVLLSQL
SLSDILLTTSITPNLLWLLSSGEARIPVTDCLAQFFFYAVSTSVECLLLTAMSYDRYLAICNPL
RYGSIMNIRLCLSMTLRCWGSSLMVAFVLDLLISTLQFCGPFIVDHYFCDFSPLLELSCSDS
DMVKLTDLILAIPFVVLPFCYIIFTYISIGFAIARISSANRRHKAFSTCSSHLIVVCTYYGTIITL
YMVPSKGHTFNMNKTLSLLYTVGTPFFNPIIYSLRNKEIQVSLQKSIKC*
>jgi|Xentr4|310198|e_gw1.24.127.1
MAARHNQSVITELWLLGFQNLHNSRLILFFVFLGMSMASTAGNLLIISLVSSTQHLRSSPM
YVFLSHLAVTDITITVTVSWTLLPAIWNKTTLVSIAGCLCQFFVFASSTSIECFLLTVMSYDR
YVAICRPLHYATIMNFTLCHQLAICSWVLGFTVTLIIVIMVNNLILCGPNIIDHIFCDLNPLLQ
LSCSDTTIVQMTLISLGVPETVMEPAFIITTYVCIFLTIFRIPSLNGREKAFSTCSSHLTVVCTY
YGTLIAIYITPSGGHTLNISKFLSLLCTVLSPLFNPIIYCLKNNEIQTALRKCFQKLKCKYSE*
>jgi|Xentr4|310206|e_gw1.24.75.1
MHKVNQTSGISFLLLGFQNSPIINEFLFVPFFWIYILTLFWNLLIIILVITVSALRSPMYVLLSQ
LSLSDVLLSTSITPNFLHLLLNGGGTISATGCITQFLFYSASASAELLLLTAMSYDRYLAICY
PLHYVSIMDFRLCLYTSLCSWGLGFILGLSINLLTFNLEFCGPFVIDHYFCDFAPLLKLSCSG
YKAVELTDIILAIPFMLFPFCFVIYTYVAIGLAILRISSTEGRHKAFSTCSSHLIVVCMYYGTM
ITVYMVPSKGHNFNMNKILSLLYTVGTPFSNPIVYSLRNNEIKAALLKYLSI*
>jgi|Xentr4|310258|e_gw1.24.525.1
LCINNQTLVVELVLLGFGNLQQLKHLVFVLFLIAYITTLTGNVIVIGVIISSPRLHTPMYIFLC
NLSLCEILFTTNIVPNMLSAVWGGVGTMTVYGCFAQFYIYTATGSVECLLLTTMAFDRYLA
ICNPLRYSSMIDLRICKYLALCAWLSGFLIMSVITVTASHLELCGSNIIDHIFCDLAPILKMSS
TDTFLVETEVFVVAIALSLLPFTFIVGTYTSIFRTIVRISSASGRQKAFYTCSTHLASVGCYFG
TLFTIYLFPSEGHSATVNKILSLLYTVVTPLLNPMIYSLRNQEMKFFFALYMSSKPFAKRQE
MA*
>jgi|Xentr4|310279|e_gw1.24.495.1
MCTANETEVTDILLLGFNDLNQSKIPLFIFFLLLYCVILCGNILIICLVSFHEHLQMPMFFFLK
HLGLADILLTSNIVPMMLHIILNEEMIVALVGCICQLYIYGVSTVQCLLLAVMSFDRYLAVC
YPLHYISIMSPNVCLLIVVGCWLLVFVLITSEIILVCQLDFCGFDQIDHFFCDIGPLVALSTSD
TSVLSLVDLVYSVLIISVPFIFIIGTYVCIFITILKMNSKTGRQKMFSTCSSHLTVVCTYYGTQ
IIVYMGPTGEYSTNMKKFLSLLYVVIAPFMNPIIYSLRSKEIRETLRRYIKMA*
>jgi|Xentr4|310291|e_gw1.24.372.1
MHKVNQTSGISFLLLGFQNSQIINEFLFVLFLWIYILTIVGNFVIIILVVKVSALRSPMYVLLS
QLSLADVLLSTSITPNLLWLLLNGGGTISATGCITQLYFCGVLATSEYFLLTAMAYDRYLAI
CSPLHYASIMDFRLCLYMSLCSWGLANIWSLVINLLIYNLQFCGPFVVDHYFCDFTPLLELS
CSNYKPVELTDIIFGIPFTLLPFCFIIYTYVSIGLAILRISSTEGRHKAFSTCSSHLIVVCTYYG
TLITVYMVPSRGNKFNINKILSLLYTVGTPFFNPVVYSLRNSDIKVVLLKFMSKGISL*
>jgi|Xentr4|310067|e_gw1.24.581.1
MNNDSIVSEFFLLGFQHLNHFKIIIFCVILLIHILTVVYENALVIALVTVSRGLQSPMFFFLQQL
SFSDLLQSVTIVPTLLQTVINNGAKIPLIGCIVQLYLFAVSESLQCFLLTVMSYDRYLAICNPL
RYSSLMSHRFCFKLIAMSWLLCLSFTPVTVISAATQEFCNQNTINHFFCDYFPLLELSCSDT

SLARMLTITLSASAVLFPFMLVIISYICIAHEILKIVSSIGRQKAFSTCSSHLAVVSIFYGTLIGI
YVVPTRNQSQTVKKLLSLLYTVVTPFINPMIYSLKSNNMKNALKNIVL*
>jgi|Xentr4|310293|e_gw1.24.356.1
MRMQNRSMVSEIFLLGFQHLDNFKILIFSLILLIHILTVCENFLVIVLVKISQYLQFPMFFFLQ
QLSVSDLLLSVVIVPTLLSTVMNEGAKISLIGCFVQLYLFGITEGLQCLLLSVMSYDRYLAI
CNPLRYSSIMSHRVCVKLIAMSWLLALSVTPVTVISAATQEFCNQNTINHFFCDYFPLLELS
CSDTSLARILTIIFSVPVVLFPFSLIIGSYICIAHEILKIVSSIGRQKAFSTCSSHLAVVSIFYGSL
IGVYGVPIKNESQTIIKVISLLYTVVTPFINPMIYSLKNTDMKNALKNVLQ*
>jgi|Xentr4|310315|e_gw1.24.437.1
NQTSGISFLLLGFQNSQKINEFLFVLFLWIYIVTLFGNHLIIILVITVSALRSPMYALLSQLSL
ADVLLSTSITPNFLWLLLNGGGTISATGCITQFYFYCVSAGSEFYLLTAMAYDRYLAICSPL
HYASIMGFRLCLYMGLCSWGLGFILSLFINLLTFHLQFCGPFVIDHYFCDFTSLIKLSCTDY
KAVELTDIILIIPCMLLPFCFIIFSYVSIGLAILRISSTEGRHKAFSTCSSHLIVVCMYYGTPFTV
YMVPSKQYNFNIKKMLSLLYTVGTPCFNPIVYSLRNNDIRVALYKCISNQISFLYSI*
>jgi|Xentr4|310368|e_gw1.24.373.1
MNQTTVTSFLLLGFQNNQSVNTLLFVVILWIYILTLVGNVLLIILVLTVPALRSPMYVLLSQ
LSLSDILITTNITPKLLTLLLSGGGKIRVSGCITQFFFYVLFTALECILLTAMSYDRYLAICNPL
RYGSIMNVRLCLSMSLLSWGLAFMVALVLDLLISKLQFCGPFILDHYFCDLAPLLELSCSD
VKVVELTDLIFTIPFILFPFCFVIFTYISIGLAILRISSTNGRHKAFSTCSSHLIVVCTYFGSLITL
YMVPSKGQGFNMNKILSILYTVGTPFFNPVIYSLRNNDIKAAVIKFLSNFPVKLFLPSV*
>jgi|Xentr4|310369|e_gw1.24.365.1
MHKVNQTSGISFLLLGFQNSQIINEFLFVPFLWIYIFTFFWNILIIILVITVSALRSPMYALLSQ
LSLADVLLSTSITPNFLRLLLNGGGTISATGCITQFYFYCVSAISELFLLTAMAYDRYLAICSP
LHYVSIMGFRLCLYMSLSSWGLGFLLSVFISLLTFNLQFCGPFVIDHYFCEFALLLKLSCTD
YKAVELTDIILAIPLTLLPFCFIIYTYVAIGLAILRISSTEGRHKAFSTCSSHLIVVCMYYGTVII
VYMVPSKGHNFNINKILSLLYTVGTPCFNPIVYSLRNNEIKNALWKYIVT*
>jgi|Xentr4|310377|e_gw1.24.593.1
MQVNNHTMVSEIFLLGSQHLSHFKILIFSFILLLHILTLYENTLVVALVTISQGLQSPMFFFLH
QLSFSDLLESVVIVPTLLSTVMNEGAKIPLIGCFVQLYLFGITEALQCILLTVMSYDRYLAIC
NPLRYSSLMSHRVCVKLIAMSWLLALSLTLVTVISAATQKFCNQNTINHFFCDYFPLLELSC
SDTSLARILTITVSVPVILFPFLLIIGSYICIPHEILKIVSSIGRQKAFSTCSSHLAVVSIFYGTLI
VTYVIPRRNQSQTISKLLSLLYTVVTPFINPMIYSLKSTDMKNALQNIIKNILPNET*
>jgi|Xentr4|310380|e_gw1.24.577.1
MKNHSMVSEIFLLGFQHLSNCKILTFSLILCIHVLTTTENILVIALVTVSRGLQSPMFFFLQQ
LSFSDLLETMVIVPTLLRALMNEGAKIPLIGCIVQLYLFAVTDIFQCFLLSVMSYDRYLAICN
PLRYSSLMSHRVCVKLIVMSWLLALSLTLVTLKTVATQEFCNQNTINHFFCDYFPLLELSCS
DTSLARILTNTFSLLVTIFPFILIIGSYICIAHEILKIVSSIGRQKAFSTCSSHLAVVSIFYGTLIGI
YMVPTGNQSQTISKLLSLLYTVVTPFINPMIYSLKSADMKMPLKTLYNNNTYYLLYIYLLFI
KY*
>jgi|Xentr4|310393|e_gw1.24.35.1
MPRNNQTEITEILLLGFQNLQNLRILLFISILIIYIISLAGNIVIITLISTLRRLHTPMYTFLGLLS
SSEIISTTNIVPNMLVVIAADGSVMPFTACLSQFYIFSALTNTECFLLTMMSYDRYLAICHPL
RYNSIMNSKLCLQLIIWSWVLGFTITLIILVNLLSTGNFCGPNTINHFFCDLAPILKLSCSDM
SLIEIQALLFSVPVIIIPFIFIVGSYIFIAFTIMKITSTTGRKKTFSTCSSHLATVCTYYGTLISVY

LVPSTENSPNVKVVSFLYVIVTPLLNPIVYSLRNQDIRSAITKSI
>jgi|Xentr4|310395|e_gw1.24.27.1
MLRMNQTTVSSFLLLGFQNTEIVNTLLFVIILWIYVMSLAGNFFLIILVVTVPTLRSPMYVL
LSQLSLADILLTTSVTPTLLWLLLNGGGKMLVSACITQFYFCCSLTSIECLLLTVMSYDRYL
AICNPLHYGSVMNIKLCLSMSLWCWGSSFLVAFVLDLLISHLQFCGPFILDHYFCDFSPLLE
LSCSGIKVVEVTDLILATPIVVFAFCFIIFTYISIGLAILRISSTNGRHKAFSTCSSHLFVVCTY
YGSLIIVYMVPSKGHTFNMNKTLSLLYTVGTPFLNPVIYSLRNKEIKVALQKCMINLCMT*
>jgi|Xentr4|310404|e_gw1.24.690.1
MHKVNQTSGISFLLLGFQNSQIINEFLFVPFLWIYILTLFGNLLIIILVITVSALRSPMYALLSQ
LSLADVLLSTSITPNFLRLLLNGGGTISATGCITQLYVYAVSGASEFYLLTAMAYDRYLAICS
PLHYASIMGFRLCLYMSLCSWGLAIILSMTITLLISNFQFCGPFVIHHYFCDFSPLFELLCTG
NEGMKLTQIILLIPFTIFPFCFIIYTYVAIGLAILRISSTEGRHKAFSTCSSHLIVVCTYYGTLII
VYMVPSKGHHFNINKILSLLYTVGTPFFNPVVYSLRNKDIKVVLLKHM*
>jgi|Xentr4|310420|e_gw1.24.420.1
MHKVNQTSGISFLLLGFQNSQIINEFLFVMFLWIYIVTLFGNLLIIILVITVSALRSPMYALLS
QLSLADVLLSTSITPNFLRLLLNGGGTISATGCITQFYFYCVSASSEFYLLTAMAYDRYLAIC
SPLHYVSIMGFRLCLNMSLCSWGLALILSLFINLLTFNLQFCGPFVIDHYFCDFAPLIKLSCS
DYKAVELTHIILLIPFLLLPFCFIIYTYVAIGLAILRISSTEGRHKAFSTCSSHLIVLCTYYGTAI
IVYMVPSKGHNFNIKKMLSLLYTVGTPCFNPIVYSLRNNDIKASFWKYVPNRSSLHALFK
QQKVD*
>jgi|Xentr4|310458|e_gw1.24.120.1
MHKVNQTSGISFLLLGFQNSQIINEFLFVLFLWVYIVTLFGNLLIIILVITVSALRSPMYALLS
QLSLADVLLSTSITPNFLWLLLNGGGTISATGCITQFYFYGLSAGIECFTLTIMSYDRYLAIC
SPLHYASIMGFRLCLYMSLCSWGLGFIVSSVLNLLIYRLQFCGPFVIDHYFCDLAPLLELSC
SDYKAVELTDMILGIPVVLFPFCLIIYTYVAIGLAILRISSTEGRHKAFSTCSSHLIVVCMYYG
TLIAVYMAPSKGQRFNMNKILSLLYTVGTPFLNPIIYSLRNKEIKVNLVKCMLNRVIL*
>jgi|Xentr4|310467|e_gw1.24.52.1
MHKVNQTSGISFLLLGFQNSQIINEFLFVPFLWIYILTLAGNFLIITLVLNISALRSPMYVLLS
QLSLADVLLSTSITPNFLWLLLNGGGTISATGCFTQFYFYIASVSLECFLLTFMAYDRYLAIC
SPLHYASIMGFRLCLYMSLCSWGLALILSLLLNLLVCKLKFCGPFFIDHYFCDFSPLLKLSC
SDYNMLELMDIILAVPFGLLPFCFIIYTYISIGLAILRISSTEGRHKAFSTCSSHLIVVCMYYG
TLIAVYMVPSKGHGFNIKKSLSLLCTAGTPFFNPIIYTLRNNEIKVALQKYISSLFVRHFCSL
FKHNNHCNLFG*
>jgi|Xentr4|310481|e_gw1.24.691.1
MCSFISTRQNDTNETKITDFILLGINGPRTLKMVLFSLFLVVHITTVCVNLVIIALYLQSQQL
KSPMYFFLSHLSSSDILLSTCIVPNLLDALLRNGKNMSLGGCLAQLLATGTSTGAECYLLT
AMSYDRYLAICNPLHYMKMMSPKLCVHLVAWSWFLSFLICLVIVLLLAQLDFCRCNVLD
YIYCDFSPLLAISCKNSLALEITPMVLSIPIILLPLGFIISTYIYIGLAILRISSSVGRKKTFSTCS
SHLTVVCTYCGILITKYTVPIGGQSLTMNKAISLLYTVGTPLLNPIIYSFRNQEIRAALKKW
MTRRVGY*
>jgi|Xentr4|310488|e_gw1.24.659.1
MHMANQSSVISFLLLGFQNIHVINVLLFDLFLWIYILTLVGNFLIIILVITVSALRSPMYALLS
QLSLADVLLSTSITPNFLRLLLNGGGTISATGCITQFYFYSVAGSSEFYLLTAMAYDRYLAIC
SPLHYVSIMGFRLCLYMSLCSWGLALIMSLVMTLLTFSLQFCGPFVIDHYFCDIVPLLELSC

TDYKDVELTKFILIIPFAFFPFCFIIYTYVSIGLAIFRISSTEARHKAFSTCSSHLIVVCTYYGTL
LTIYMVPSKGHNFNMNKMLSLLSTVGTPFFNPIVYSLKTKEIKAALRKYKSLCKKNAANC
NM*
>jgi|Xentr4|310498|e_gw1.24.421.1
MHKVNQTSGISFLLLGFQNSQIINEFLFVPFLWIYILTLFGNLLIIILVITVSALRSPMYALLSQ
LSLADVLLSTSITPNFLWLLLNGGGTISATGCITQFYFYGVSAGSEFYLLTAMAYDRYLAIC
SPLHYVSIMGFRLCLYMSLCSWGLALILSMTLNLLIFNSQICGPFLIDHYFCDFSPLLEVLCT
GYGVVKLTQIILAIPFTLLPFCYIIFSYVSIGLAILGISSTEGRHKAFSTCSSHLIVVCMYYGT
LIIVYMVPSKGHNFNINKILSLLYTVGTPFFNPVVYSLRNNDIKAVLLKHMKLNLFRRPLY
MQKCVF*
>jgi|Xentr4|310499|e_gw1.24.413.1
MHKVNQTSGISFLLLGFQNSQIINEFLFVLFLWINIFTFFWNLLIIILVITVSALRSPMYVLLS
QLSLADVLLSTSITPNFLWLLLNGGGTISATGCITQFYIYCASAASEIYLLTAMAYDRYLAIC
SPLHYASIMGFRLCLYMSLCSWGVALILCLFTNILTFNLQFCGPFVIDHYFCDFAPLLQLSCS
DYTSLELTHIILIIPFGLLPFCFIIFSYVSIGLAILRISSTEGRHKAFSTCSSHLIVVCMYYGTLII
VYMVPSKQHNFNITKILSLLYTVGTPFFNPIVYSLRNNEIKAAFWKYVLNRISLHALFKQQ
KGY*
>jgi|Xentr4|310513|e_gw1.24.498.1
MNTTSNNGIFLRGFGNLHDFKIPFFFLFFAIYMLTLTSNLLIIILVSTHYHLQSPMFFFLAHLS
FSDLLVTTNILPNMLLITLTDGGMVFLSGCLTQFFFYGLSATTECLLLSAMSYDRYLAICKP
LHYHTVMDSKLCSSLVISSWSLSFILTIIPIYFLQRLRFCGINVIDHFFCDLGPLLELSCSDTSF
VKLEVFAMSTLLTMVPFVFICVTYVYIILSILRISTNTGRQKAFSTCSSHLAVVCAFYGALFA
MYVAPSKGDSAINKGVSLLYTVVTPLFNPIIYSLRNQDIRTTIRKYLRTQKPHI*
>jgi|Xentr4|310518|e_gw1.24.79.1
MHKVNQTSGISFLLLGFQNSQIINEFLFVPFLWIYIVTLFGNHLIIILVITVSALRSPMYALLS
QLSLADVLLSTSITPNILRLLLNGGGTISATGCITQLYFYCVSGTSEFYLLTAMAYDRYLAIC
SPLHYASIMGFRLCLYMSLCSWGLALILSLVMNLLTFNLQFCGPFVIDHYFCDFTPIIKLSCT
DYKAVELTDMILAIPFMLLPFCLIIFSYVAIGLAILRISSTEGRHKAFSTCSSHLIVVCMYYGT
LITVYMVPSKGHNFNIKKMLSLLYTVGTPCFNPIVYSLRNKEIKVALWKHISNRISFHTPFN
QQKYIKHYSHCSTIFI*
>jgi|Xentr4|310524|e_gw1.24.367.1
MEVQNVTKVTEFLFTGLEANHTHVPFLFILFLMVYLITIIGNSGMVALVCYTPHLQTPMYC
FLGCLSMVDLCYSSVIAPKMLADLGSRDKSISFIGCALQFFFFAALAVTEALLLSCMSYDR
YVAICRPLHYSSIMTKNKCLWLILMSFSIGFSQSSVQTKCIFSLEFCRRNQIDHFYCDVPPLL
KISCSETFHCNMVTVFFICSFGVGSMVNILVSYTLIVSSVLQIKSSSGRGRAFSTCSSHLTCV
CIFYWTVFFIYLRPPSSSFDRRDKVASVFYTMVIPMLNPLIYSLRNQEVKKALYRLTTRCFN
TIL*
>jgi|Xentr4|310526|e_gw1.24.359.1
MCTANETEATVILLLGFRDLNKSKIPLFILFLFIYSVILCGNVLIIFLVSFNEHLQIPMFYFLKH
LGLADVLFTTSIVPMMLHIILKEEMIVALVGCICQLYVYGLSSVQCFLLAVMSYDRYLAICY
PLHYISLMAPNVCLLLVIGSWLSVFALVTTELIFVSQLHFCGFNQIDHFFCDFGPLVALSTSD
TSVLMLLDFIISIPMISVPFILVIGTYVGIFITIFKMNSKTGRKKTFSTCSSHLTVVCTYYGTLII
VYMGPTGENAEDLKKFLSLLYIVIAPFMNPIIYSLRNKDIRETLRKYIKFT*
>jgi|Xentr4|310527|e_gw1.24.502.1

MNQTTVPSFLLLGFQNNQIVKSLLFVVILWIYTLTLAGNFLLIILVVKVPALRSPMYVLLSQ
LSLSDILLTTNITPNLLSLLLSGGGTILLTDCLTQFFFYCVSTAIECLLLTAMSYDRYLAICHP
LRYGAIMDFKLRVYMSLWSWSLSFMVALVLDLLISHLQFCGPLIIDHYFCDFSPLLELSCSD
IKVVQLTDVTFGIVFAVFPIFFIIFTYISIGLAVLRISSTNGRHKAFSTCSSHLIVVCTYYGTLIT
VYMVPSKGHTFNVNKVLSLLYTVGTPFFNPIVYSLRNNEIKVALQKCMSNFLVILFRSVQS
HIQSLGPNFNIDET*
>jgi|Xentr4|310541|e_gw1.24.714.1
MHKVNQTSGISLLFLGFQNSQIINEFLFVPFLWIYILTLFGNLLIITLVIRVSNLRSPMYALLS
QLSLCDILFSTSITPNFLRLLLNGGGTISATGCITQFYIYCVSGTSEFYLLTAMAYDRYLAICY
PLHYVSIMDFRLCLYSSLCSWGLAFLLCFFVSFLIFNLQFCGPFVIDHYFCDFAPLIKLSCTD
YKAVELTDIILGIPFMLLPFCFIIFSYVSIGLAILRISSTEGRHKAFSTCSSHLIVVCMYYGSLI
TVYMVPSKGHNFNINKILSLLYTVGAPFSNPIVYSLRNTEIKVALHKFMSN*
>jgi|Xentr4|310560|e_gw1.24.692.1
MHKVNQTSGISFLLLGFQNSQIINEFLFVLFLWIYIVTLFGNLLIIILVITVSALRSPMYALLS
QLSLADVLLSTSITPNFLWLLLNGGGTISATGCITQFFFYGVSVGSEFFLLTAMAYDRYLAIC
SPLHYASIMGFRLCLYMSLGSWCFSFILILFISLLTFNLQFCGPFVIDHYFCDFTPILKLSCTD
YKAVELTDIILAIPFVLLPFCFIIYTYVAIGLAILRISSTEGRHKAFSTCSSHLIVVCMYYGTLII
VYMVPSKSHNFNIKKMMSLLYTVGTPFFNPIVYSLRNNEIKVAFWKYVPN*
>jgi|Xentr4|310572|e_gw1.24.537.1
MRVNNHTMVSEFFLLGFQHLSNFKILVFILILLLDILTVSENVLVIALVTVSRGLQSPMFFFL
RQLSFSDLLQSVVIVPTLLSTVMNEGAKISLIGCFAQFFLFGVSETLQCLFLTVMSYDRYLA
ICNPLRYSSLMNHRLCSQLIAISWLLALSFTPVTVISTATQEFCNQNTINHYFCDYFPLLELS
CSDTSLALILTTALSFPSLLFPFSLIIGSYICIAHEILKIVSSIGRQKAFSTCSSHLAVVSIFYATLI
VTYVVPKRNQSQTIGKLLSLLYTVVTPFINPMIYSLKSADMKNALNNIRKQRNIC*
>jgi|Xentr4|310593|e_gw1.24.499.1
MDKKNLTTVSELFLLGFQNLNCFRIPLFCLILFIYLLTVSGNIVIIVLVSISRNLQSPMYFFVQ
QLSLCDLLQTTCTVPVLLWTVLNAGAPISVGGCITQFYFVDASESVECLLLTVMSFDRYLA
ICNPLHYRSLMNPKLCVELTLIPWLLGFSIGFITANAIGNLQFCNQNIINHFFCDYFPLLELSC
MDTFIAQTEAILQAVLVVFIPVILIIISYVFIIHTVLKIVSTTGRQKAFSTCSSHLIVVLLFYGT
LIGVYVVPPRKQSLTISKVLSLSYTLVTPLMNPVIYSLRSKDMKKAFKKMLSF*
>jgi|Xentr4|311307|e_gw1.27.150.1
VSHGLLSQRCGKDGLWVKGNRSQAWRNVSQCEDDTEVTAEETRDLLLSFRALYTVGYSV
SLLTLISALFVVSLFTFRKLRCTRNSIHANLFASFALRAVSVIVKDVLLAKRWGMQITEVSD
WEVLISDQAAIGCRIAQVVMQYCILANHYWFVVEAVYLYKLLIGAVFSEKNYYTLYLYLG
WGTPVLFVVPWVTLKYLKENSECWALNENMAYWWIIRIPILLASLINLVIFMRILKVILSKL
RANQKGYADYKLRLAKATLTLIPLFGIHEVVFIFATDEQTSGILRYIKVFFNLFLNSFQGFLV
AVLYCFANKEVGFPFQLSGLDEKLFAIL*
>jgi|Xentr4|311736|e_gw1.28.148.1
MEWNITVSANGSAGADSWGKHRFSVFSVLILTLLAMLVIATFLWNVLVLVTILRVRTFHRV
PHNLVASMAISDVMVAALVMPLSLVHELNDRRWRLGRVLCHIWISFDVLCCTASIWNVTAI
ALDRYWSITRHLEYTLKTRKRISNIMILLTWLLSAVISLSPLFGWGETYSEVNEECQVSQEP
SYTIFSTFGAFYLPLCVVLFVYWKIYKAAKFRIGSKKTNTITPMPEVIEVKEASHQPQMVF
TVRHATVTFQTDGDTWREQKEKKAALMVGILIGVFVLCWIPFFITELINPLCSCDIPPIWKS
IFLWLGYSNSFFNPLIYTAFNKNYNNAFRNLFSRQR*

>jgi|Xentr4|310307|e_gw1.24.102.1
MHKVNQTSGISFLLLGFQNSQIINEFLFVPFLWIYIVTLFGNLLIIILVITVSALRSPMYALLS
QLSLADVLLSTSITPNSLRLLLNGGGTISTTGCITQFYFYGVSTGSEFFLLTAMAYDRYLAIC
SPLHYASIMDSRLCLYMSLCSWGIALILCLIVNLLTFNLQFCGPFVIDHYFCDFTPIIKLSCTD
YKAVELTVIIFDIPFLLLPFCFIIFTYVSIGLAILRISSTEGRHKAFSTCSSHLIVVCMYYGTLII
VYMVPSKGHNFNIKKMLSLLSTVGTPCFNPIVYSLRNNEIKVALWKYMSNQISFFTLNFSS
FIHRDQKALLFG*
>jgi|Xentr4|310310|e_gw1.24.453.1
MHKVNQTSGISFLLLGFQNSQIINEFLFVLFLWIYILTLVGNVLIIILVVTVSALRSPMYVLLS
QLSLADILLSTSITPNLLRLLLNGGGTISATGCITQLFFCGISECSECFILTAMSYDRYLAICYP
LHYASIMGFIHRVYLSFFPWGLAIMLTLVLGLLISHLQFCEPFVIDHYFCDFAPLLELSCSDS
KVLELTDIILGIPVVLLPFCFIIYTYVSIGLAILRISSTEGRHKAFSTCSSHLIAVCMFYGTLIII
YMVPSTGHGFNMKKTLSLLYTAGTPFLNPIIYSLRNNEIKGALLKSMWNLLKKLFYVH*
>jgi|Xentr4|310372|e_gw1.24.349.1
MNQTTVTEILLLGFQNLHNFRIPFFSLILLIYILTFCANVLILVLVSVSRNLQSPMYFFLQQLS
ICDLLQTTTIVPIMLQTIIKGRATITIGWCITQWYLFGGSEIWECLLLTAMSFDRYSGFFTPLY
VAICNPLHYSSIIDHKVCVKLVILLWLTGFRVSLVMSSSIGTLVFCKQTAINHFFCDFAPLLEL
SCSDSNFVHIEAALLSIPTLITPFIIVIISYICIARAILKIVSSIGRQKAFSTCSSHLAVVSIFYGT
LIGIYVVPNQGQLVTKSKVISLLYTVVTPLLNPIIYTLRNKDIKDVCKKVLDIWATHIY*
>jgi|Xentr4|310522|e_gw1.24.609.1
MNNHSVVTEIFLLGFQHLNNFKILIFSLILLIHILTVSENALVIALVTVSRGLQSPMFFFLQQL
SFSDLLESMVVVPTILKTIINDGAKISLTGCIVQFYFFGATEDLQSFLLSVMSYDRYLAICNP
LRYSSLMSHRVCVKLIVMSWLLALSVTPVVVISAATQEFCNQNTINHFFCDFFPLLELSCSD
TSLARILIYIASVPAVLFPFVLIIGSYICIAHEILKIVSSIGRQKAFSTCSSHLAVVSLFYGSLIVT
FVVPTRIQSENTRKLLSLFYTVVTPFINPMIYSLKSADMKNALKNIKQ*
>jgi|Xentr4|310547|e_gw1.24.464.1
MRNQTVEEFLLVGFDVHSQLKCPLFLLFLVIYIITLAGNLVIIVLVYVHVHLHCPMFIFLGN
LSLSDILFTTNIVPKMLSIILGEGGTLSFHGCLSQFYFYASVGSTECFLLTVMSYDRYLAICH
PLHYNVIMNARLQKCLLVLSWLPVSIVTLVRIILIHQLDFCGPNVIDHFFCDLDPILQLSCSD
TSIIENEVLAASVHVILLPFLFIVTTYARIILAIVRIPSIHGRQKTFSTCSSHLIVVCTYFLTLIT
VYGIPSKAHSSAATKLRSLLYIVLTPMFNPIVYSLRNKEIRYALQKLKTTVKLIRFM*
>jgi|Xentr4|310569|e_gw1.24.561.1
MQVNNHTMVSEIFLLGFQHLNNFKILIFLLILLIHILTVVYENALVIALVTVSQHLHSPMFLLL
RQLSFSDLLQSVVIVPTLLRTVMDEGAKIPLTGCFEQFYFFGVTESLQCLLLSVMSYDRYL
AICNPLRYSSLMNHRVCVKLIVMSWLLALSFTLVTVMAAATQEFCNQNSINHFFCDYFPLL
ELSCSDTFLVRILAIALSVPVILFPFLFIIGSYICIAHEILKIVSNTGRQKAFSTCSSHLAVVSIF
YGTLIVTYVVPTRNQSQTISKLLSLLYTVVTPFINPMIYSLKSADMKNALKNIIK*
>jgi|Xentr4|310600|e_gw1.24.618.1
MFFFLQQLSFSDLLQSVVIVPTLLRTVMNEGAKIPLIGCIVQLYLFAITDILQCFLLTVMSYD
RYLAICNPLRYSSLMSHRVCITLISISWLLALSVTPYTVISAATQEFCNQNTINHFFCDYFPL
LELSCSDNTLAQILAVALTVPVILFPFMFIVGSYICIAHEILKIVSSIGRQKAFSTCSSHLAVVS
IFYGTLIGIYVVPTRNQSQTISKLLSLLYTVVTPFINPMIYSLKSADMKNALKNIIK*FLLPFC
VFFCSFHKGFSPSSGSTRSRQVIYRHYG*
>jgi|Xentr4|313085|e_gw1.31.218.1

MSISCENATRVHEDGHPAVSTLMFAGGVVGNLLALGILGVHRRERRARASPFWVLVTGLA
VTDLLGTCILSPMVFVSYAQQSSILALGAAPLCHLFAFAMTFFSLASMMLLFCMALERCLA
ISHPYVYAQHSWGHRLAHAAIPVSYVLGALLCALPLVGVGEHKQYCPGTWCFIRMTVPG
HRESGALAFSLLYASLTGLLIVAIVICNTSVTASLCRMRKGQKARRGSLRRCGARGWLLGA
GEEELEHLILLVLMTVIFMVCTVPLTVSTGDQRMHLYEGQLVRGLNSILNRR*
>jgi|Xentr4|313091|e_gw1.31.336.1
MLLALNLQNESFLLNLSHGETRDSLPPATGLSRTAHSVVAVCLGCILVLGSLYNSFVLLIFV
KFTAIRTPINMILLNISVSDLLVCIFGTPFSFVSSVSGGWLLGQQGCKWYGFCNSLFGLVSMI
SLSMLSYERYLTVLKCTKADMTDYKKSWLCIIVSWLYSLCWTLPPLIGWSSYGLESSGTT
CSVVWHSKSSNNISYIVCLFLFCLVLPLFIMIFCYGHIVRVIRGQVCRINMTTAQKREHRLL
FMVVCMVTCYLLCWMPYGLVSLMTAFGKPGMITPTVSIIPSILAKSSTFINPLIYIFMNKQV
RLTLYILHCIF*
>jgi|Xentr4|313788|e_gw1.34.66.1
MENRNNSKILEFIIVGFSDFPGMKIPLFFFFLSLYNLTLIGNVVLILVICWSPSLYTPMYFFLC
NLSSLDIVYTSVTSPKLIHIFVTSDRRISFRECIAQLYFFVAFGSTEYLLLTVMSYDRYVAVC
KPLHYPLIMNRRVCTLGATGAWVGGILASLALSIVTSNLNYCASNMVNHIFCDIIALVQLS
CGETIYIHNIILIQGVILVMTSFLLTLTSYICIISTILKIGSSTGRYKAFSTCASHLTVVSLFYLL
VLVLYMGPESTIISSQSKILVVLYAYFIPLLNPVVYSFRNKEVKGAIEKMLVQIRKASLR*
>jgi|Xentr4|313840|e_gw1.34.91.1
MEHIRNISVPREFHLLVFSNSEEHCYIIFIGLLLMYLLAVLGNLLIIVLVCLVPQLHTPMYFFL
CNLAAQDIISVSAVLPKLMAIIFIGVKSISFYGCMAQLFLYISGTDGDFLLLAIMAYDRYVAI
CIPLRYHLIMNPRFCIQLVTTSWIFCVTNAMCYALLVSHLSYCRLNNINHIFCEIISLVKLSCS
DTSLIKTLITVDAPLIGIFPFGLILTSYAYIIYTILKMRTSAAKLKTFSSCSSHLTVVLLYGGTCI
SLYMKPVSESSQEVDKLLSLIYLGFIPVLNPLVYSLRNRHVQSAAKIVFAKYLLNSVEN*
>jgi|Xentr4|313843|e_gw1.34.83.1
MSQDGNNITLPREFHFSTFSNSEEVNFWVFVVVLMMYLLIVLGNTMIATLICLIYQLHTPM
YFFLCNLAVQDILYASTTMPKLMAITATGNTDISFQGCITQIFVFDFCIITEFFLLVAMAFDRY
VAIGIPLHYLLIMRRIVCVVMASTCWVIGGFNSLLFTLLISKLSFCSSKEIDHFFCDPKALLK
LSCSDTTIIMSTVFAEGVFFGFLPCLIVMSYASIISTIVKIQTSEGRRKAFSRCSSHLTIVIIFFG
TSIGLYLKPQLDNSLECDKFLSLLYVALVPILNPLVYTLRNKQVLMSVTQIINKKSTAVKQK
SKMALNLRYPD*
>jgi|Xentr4|313847|e_gw1.34.75.1
MAENIQNNTVPTEFSLRAFPGFADVEMLLFILVLLMYLLSVLGNLVIIALICVAPQLHTPMY
FFLCNLAFQDIVHVSAIQPKLMAIIITGDHSISFPGCISQIFLFVFCLDAEFFLLPTMAYDRYV
AICVPLRYSVIMNKSVCTLLAVTVWLLAGLNALMCSSLISNLSFCKSHELNHFYCEYKTIL
SLSCSDVTTNLIIIFVEGFVIGLFPFVLIVTSYIFIISAVLKICSSAGRIKTFSSCSSHLIVVVLLY
GTCLSFYIKPESQHFQDKVLSMVYVSVVPMLNPLVYSLRNKDVLQALKKIIRIK*
>jgi|Xentr4|313849|e_gw1.34.67.1
MNFTTKKEFYLIAFSCSEEEQPFLFMGLLMIYLTAELANMMIIVLVCLVPQLHTPMYFFLC
NLAAQDIISVSAFLPKLMAITITGDTSISFPGCITQVFLFAFCIGIDIFLLATMAYDRYVAICIPL
RYYLIMNTRVCLLLVGIAWILYIPNAICLALLFSRLSFCKSRELNHFFCDLKMLLEISCSDTS
PIKQFMSAQKPFTGILPFVLIITSYVYVITTIIKLRSSAARLKAFSSCSSHLTVVLLFCGTSIGI
YIMPQSEKLQKQVKVLSLLYIGVVPMLNPLVYSLRNQQVRSAAKILLAKYVPGTLL*
>jgi|Xentr4|313851|e_gw1.34.59.1

MSILVNNNTLFEFYILAFRIYGPFQTLLFMGILVMYLLAILGNMMIIVLVCLVSHLQTPMYF
FLCNLAAQDIVFVSSTLPKLMAITATGDTSISFGGCITQMCIFTFCVTTEFFLLTSMAYDRYV
AICIPLRYFIIMSKRACTLFSTLSWLLGGFNSIFYFCIMYNLCYSQEINHFYCDLKAMIKISC
GDITHLQTLMSFESVFLGLLPFALILVSYTYIIYNILNIRTSAGRAKTFSRCSSHLTIVIIFCGT
LISTYLKPESETSEELEKLMSLLYTAVVPMLNPLIYSLRNKDVLKAMKNILQSLNTFSINIPT
RNLCSKNLKKK*
>jgi|Xentr4|313902|e_gw1.34.84.1
MQTPNQSLDIVFLFDGLTKDPRLDVVLFIIFLVIYIFTILGNGGLIVLVMKSPSLHTPMYFFL
KHLSFIDMCYTSVIIPRSLSDFLSNEKTITLLACAVQMYLYAAYFNAEVLLLAAMAYDRYA
AICKPLLYHVLMKRRVCHVLICACYSVGFFDSFIHTRNVFSQSYCNSLVISNFFCDVPPVLK
LSCSDTSMTELVLYAVIGINSFVCATTIVTSYAYIFSAILRIQTAQGRLKAFTTCSSHLISVGTL
FGTLMYMYMRPNSSYSNDQDKVVSVFYTMVIPMLNPIIYSFRNKDVRRAFLKLTF*
>jgi|Xentr4|313904|e_gw1.34.76.1
METNQSLDIFFLFDGLTDDPELETALFIIILLIYIFTIVGNCGLIVMIMTSPILHTPMYSFLKHL
AFIDMCYTSVVTPRTLSDLFSKKKDISFMACAVQMYFYAAFFGAEILLLAAMAYDRYAAIC
RPLVYHIIIKREVCRRMIIACYAISFLHSFIHTKNIFSHSYCNNYQISHFFCDAPPVLKLSCSD
TSLTQLLIFAIVGVYSSTCASLILTSYIHIFSAILRIKSTQGRQKAFTTCSSHLVSIGTLFGTMIY
MYLHPNASNADQDKVVSVFYTMVIPMLNPIIYSLRNKDVRRAFEKLM*
>jgi|Xentr4|313921|e_gw1.34.485.1
VSLKNQTMINEIFILGFQNLHNFKIPLLFLFLLLYIITVWENVLIIVLVSSSQNLQSPMYFFLQ
QLSLSDLLESTDIVPFLLVIVIHDGAIMSFAGCIMQHFIYSTTECFGCLLLAIMSYDRYVAIC
NPLRYPSIMTHKVCTKLIAWLWLSAFSITLIYEILLCTLDFCDQHSINLYFCDLFPLIEHSCSD
TFLLQIEVTLLSVPILLIPPILITISYMRISHTILKIVSSMGRQKAFSTCSSHLAVVSIFYGTIIA
MYVVPHKEESETISKVLSLLYTVGMPLLNPIIYSLRNKDIKEAFRKCKHLIHLLYL*
>jgi|Xentr4|313992|e_gw1.34.339.1
MDYGINITAPKVFHLLAFSNSEKNPHIIFIGLLLVYLLALVGNMMIIVLVCLVPQLHTPMYF
FLCNLAAQDIISVSAFLPKLMAITITGDTSILFSGCVTQLSFLIFSACGDFLLLAIMAYDRYV
AICIPLRYHLIMNPRFCILLLMATSWILCATNAMCYSLLISHLSFCRLFDINHIFCEIISVIKISC
SDTTHMRTLLTVEAPLVGIVPFGLILTSYAYIIYTILKMRTSATRLKTFSSCSSHLTVVLLYGG
TCISLYMKPDPENSREVDKLLSLMYLGFVPMLNPLVYSLRNRQVQSGAKIVFTKYILNANS
K*
>jgi|Xentr4|314017|e_gw1.34.305.1
MENQTISRGFHLYLLFNNGEKRLDFSIVILWIYLIGMLGNLVILTVICNDVHLHTPMYFFLC
TLAFVDICYPTVTLPKLMDILLSGNNSISFIQCFTQMYFFTALAVIETIVLSSMAYDRYVAIC
KPLRYHLIMNRRVCVLVIVGTWILGFVNASFITDLASKLLYVRSRGIKAFECYKKVLFFVL
WQTKIRGLICDDLCSVQKKNLFFCTLSTALAIVQTAINLWTTFSTCTSHLTVLIIFYGSIFWM
YMRPPSESENLNPGLSVLYIGVTPMLSPLIYSLRNKEVKNAVMRIMKRIS*
>jgi|Xentr4|314046|e_gw1.34.372.1
MNYTTQKDFHLLGFSSSEKDPSFLFLLVLLIYLLAVLGNMNITVLVCLTPQLHTPMYFFLC
NLAAQDIISVSATLPKLMAITITGDTSISFLGCLIQMFLFAFCTDTDFFLLATMAYDRYVAIC
VPLRYYLIMNAKLCIVLVVTSWILYVSNALCFSLLLSNLSFCKSHDLNHFFCDIKILMEISCS
DTSHIKQLLLLEIPFVGILPLLLILTSYVCIIGTIIKMGTSAARLKTFSNCSSHLTVVLLFCGTS
IGIYMKPKSKDSLEQEKLLSLFYVGIVPMLNPIVYSLRNRQVCSAAKRLLEKYRLWRLIPS
LH*

>jgi|Xentr4|314049|e_gw1.34.356.1
MNSTTQKDFHLLAFSLFVEDQPLLFMGLLIIYLTAVLGNMMIIVLVCLVPQLHTPMYFFLC
NLAAQDIIFVSAFLPKLMAITITGDTSISFPSCIIQMFLFALCIGTDIFLLTTMAYDRYVAICIPL
RYCLIMNARVCLLLVGIAWILYIPNAMCYSLLFSRLSFCKSRKLNHFFCEPKILLEISCSDTS
HIKQLMLVEIPFVGILPFVLIITSYVYIIATIIKLRSSTARLKAFSSCSSHLTVVLLLFGTSIGIYI
RPDSENYQEQEKFLSLLYTGVVPMVNPLVYSLRNQQVWSATKILLAKYVPGTPL*
>jgi|Xentr4|314119|e_gw1.34.593.1
MYLLAVLGNLIIIALICVAPQLHTPMYFFLCNLAFQDIVYISAIQPKLMAITITGDHSISFPGC
ISQIFLFIFCLDTEFFLLTAMAYDRYVAICVPLRYSVIMNKSVCTLLAVTVWLLAGFNALVH
SLLISNLFFCKSHELNHFFCELKTLLSLSCSDITANLIIIFVEGFFIGLFPFILIFTSYMFIISAVL
KIRSPAGQIKAFSRCSSHLIVVILLYGTSLSFYMKPESEHSQEQDKILSMVYVAVVPVLNPLV
YSLRNKEVLKALKKTGLNRKH*
>jgi|Xentr4|314135|e_gw1.34.315.1
ENWTVGNEFYIMAFPVQPQSRVVLFIGISGIYLIAMAGNIIITMLILLVAQLHTPMYFFLCNL
SLLDMTFVSVTLPKLLSITLKNIGTISFNDCIAQMYFFISTANIESFLLTSMAYDRYVAICNPL
RYTLILTKTSVLLLSSVSWFLGFLNSLVLTLLTSHLSFCRSRTIQNFFCDLKALLKLSCSDITT
IQRIILVDNFLIGVIPYTLTMTSYVYIIAAVLKIHSLQGKLKAFSNCTSHLTIVLIYYAIGICLYT
APDSEYSQVQDGILSLIYIALIPMINPIVYSLRNKEVLGALKKVMGWQIKKYT*
>jgi|Xentr4|314165|e_gw1.34.374.1
MSNNTSTYFYISWFSDSPDKQLFYFITFLLTYLIGLMGNLLLILVTISESHLHTPMYFFLGNL
AFVDMCYTSVTLPKQMDILLTRNNAVPYVLCFTQLHLFMMTATTEVFLLSSMAYDRYVAI
CNPLCYFVVMNKRINTLLVLGSWIFGFINAILVTVFASRLCFSGGRLIQQLFCDMKSLFSISC
SDMKDLQFIIYLEVFFAGFCPFLLSLISYAKIITCILKVQSTNGRRKTFSTCTAHLTVLLLFYG
TSISMYMKPLSENSDNQGQLFSVLYVAVAPMLNPMIYSLRNQDVKRALKKMFAVERR*
>jgi|Xentr4|314166|e_gw1.34.70.1
MTNHTIDVYFHISGFSVFTGRQILRFIAFLLIYLIGLVGNLILILLTLLESHLQTPMYFFLRNL
AFVDICYTSVTLPKLMDIFLTGDNAVAFTPCFAQLHFFTTTGCTEVVLLTSMAYDRYVAICN
PLRYFLVMNNSRCTQLVFGSWIFGFINSLLVTIFASRLSFCGSKVIHQLFCDIKSLLKISCSDI
GDFQVIIYLEVLFAGLCPFLMSLISYIKIISSVLKVQSDEGRKKTFSTCTSHLTVLLLFYGTIF
CMYIRPPSEHLENVDQFFSVLYVAVTPMLNPLIYSLRNREVKRALKCILGIKKINILSRRN*
>jgi|Xentr4|314168|e_gw1.34.62.1
MDEKCQNNTTSTQFILLAFTSSADVEMLLFIGVLLMYLLAVLGNLIIIALICVAPQLHTPMY
FFLCNLAFQDIVHVSAIQPKLMAITITGDHSISFPGCISQIFLFIFCLDTEFFLLTAMAYDRYV
AICVPLRYSVIMNKSVCTLLATIVWLLAGLNALMYVILISNLSFCKSHQLNHFFCEMKTMF
SVSCNNITADLILIFVEDIIIGIFLVLLIITSYIYIISAVVKIRSSAGQIKAFSNCSAHLIVVILLYG
TCLGFYMKPGLDQSQDQDKILSMVYVSMIPVLNPLVYSLRNTEVLKAFRKVTRKNRTLR
G*
>jgi|Xentr4|314228|e_gw1.34.63.1
MNEIEQNTTLPTQFSLLAFHSFAKMEMLLFIVVLLMYLLAVLSNVTIITLICAAPQLHTPMY
FFLCNLAFQDIVHVSAIQPKLLAITITGDHSISLPGCISQIFFFVFCINAQFFLLTAMAYDRYV
AICVPLRYFVIMNKRICALLVTAVWLLAGLNGLLYSILVSNLSFCKSHELNHFFCEYKTMLS
ASCNDITTNLIILFVEDIIIALFLFTLILTSYIFIISAVLKINSSTGRMKAFSNCSSHLTIVIILYGI
CLSFYMKPDFEHSQDQDKGLSMVVVLVVPMLNPLVYSLRNKDVLKALRKVTRTK*
>jgi|Xentr4|314229|e_gw1.34.359.1

MNENIRNRSLNEFYLRAFSASGGTQILLFVGVLVMYLLAAFGNIMINIVICLESNLHKPMY
FFLCNLGLLDLIYVSSTLPKLMAVTVTGGSSISFYGCITQMFIFATCVTAEFFLLTFMAYDRY
VAICIPLRYSVIMNKRSCRLFATLSWLGSSFNSLLYCFLIRDLPFCNIHEIQNFYCSPKTMIKL
SCSDITYIETLMSIETTFMALLPLLLILASYVFIICAIIKIPISTGRSKTFSSCSSHLTIVIIFCGTG
LSIYIKPESEYSPEQEMLLSLLYAGVVPMLNPLVYSFRNTDVLKAIRKIITIAGVKFNTTVHN
FFTCKLGNNNS*
>jgi|Xentr4|314251|e_gw1.34.341.1
MDNNTIFTYFYILAFSREGEKQPTLFIAFSFVYLVGVLGNLSIITVTCSDAHLHTPMYFLLA
NLSFIDICYTTTTLPKLLHILLTGNNIISFPECFTQMFFYSISAQNEGVLLAFMAYDRYVAVC
DPLKYHLIINIKKSSLILAGIWTSSILNCILITSITSQLRFCGSNTINQFFCDIKYMAEISCEAVS
INYYLYSETVIFGVLPLLLCVASYTKIILSILHIKSVTGRKKAFSTCSSHLTVLLLFYGTASW
MYLAPENSHNINLLFTVIFTAVTPMLNPLIYSLRNKEIKRSIFRITQKIIVCAS*
>jgi|Xentr4|314314|e_gw1.34.326.1
MWHLAWAEPSAPYNETVGRIFHIAAFPGSAEVQLIIFVGILFMYIITVSANLLITLLVCLVPQ
LHTPMYFFLCNLAILDIFYASVILPKLLAITITGDHTISFGGCMIQLYCFLWCVTQEVLLLAC
MAFDRYVAICIPLRYSQIIDKHLCIKMAAPSWLFSGINSLFHSMYTSHLSFDYFREIDHFFCD
LKALMMISSSDTRPMEIILFAEDIYAGLFPFLCILTSYIYIISTVLQIRSSAGRLKAFSSCTSHL
TVVLMSYGISISSYAQPLSEKSVEQDKLLAIFYAAVVPMLNPLVYSLRNKDVLKAVKRLTR
*
>jgi|Xentr4|314321|e_gw1.34.562.1
MDNHTQDQYFYICLFTDSPAKRPFCFVVFLLIYTIVLIGNLLLISATIFDSHLQTPMYYFLRN
LSFVDICFTSVTLPKLMDIFLTGINAVPFGLCFTQLLFFGSIVTTEILLLTSMAYDRYVAICNP
LCYFLVMSNRRNNVLVLSSWITGILHALPVVTLISQLPFCGSKEIHQLFCDVKSLLQNSCGD
LRKFQIILCFEVLSFGVVPFVLCLASYAKIISKVLKIKSPQGRMKTFSTCTAHLTVLLLFYGT
IICMYIKPLSDHSVIQDQLSTFLYLVVTPMLNPLIYSLRNQEVKNALKRMFNWLM*
>jgi|Xentr4|314342|e_gw1.34.377.1
MEHVRNITVSREFHLLVFSNSEEHRYIISIGLLLMYLLAVLGNLLIIVLVCLVPQLHTPMYFF
LCNLAAQDIVSASALLPKLMAIIFTGVKSISFYSCMAQLFLYVSSSDFPLLAIMAYDRYVAI
CIPLRYHLIMNPRFCILLMAMSWIFCVTNGLCYTSLASRLSYCRLNINHIFCEIVSLVKLSCS
DTSLINTLIIVDVPLIGMFPFGLILTSYAYIIYTILKMRTSAAKLKTFSSCSSHLTVVLLYSGTC
ISLYMKPVSENSQEVDKLLSLIYLGFIPMLNPLVYSLRNRQVLSAAKIVFTKYFLNSVEN*
>jgi|Xentr4|314345|e_gw1.34.369.1
MENQTVLEGFHLFPMFSKGEKRPIFFIVFLWIYLISVLGNLVILTVIYSSAQLHTPMYFFLCN
LSLVDIWYPTTALPKLMDMLISGNDSITFIQCFTQMYFFTALAEIETIVLSSMAFDRYVAICK
PLNYHLIMNRRVCVLLIAGTWSFGFVNASFITDLASKLSFSQTNKIHHLFCDVIALAKISTN
DITMFYNVVYVEVFLFGIMPFSLNLVTYINIIHSILQIKSKHGRQKAFSTCTSHLTVLIIFYGS
VFWMYMRPPSESENQNPVFSLLYIGVTPMLNPLIYSLRNKEVKNAVIRIMIKRLIIRR*
>jgi|Xentr4|314388|e_gw1.35.18.1
MNMANETSDAATYAAVYIGVETVIGISAVLGNILVIWAVRLNPSLQTTTFYFIVSLALADLA
VGFLVMPLAIVLSLGIHFHFHVCLFICCFIIVLTNASILSLLAIAIDRYLRIKIPTRYRTVTTRRI
GLSVCIVWIISFLVGMVPMFGWNNRFSLNEEHQHYLDCTFENVMSKEYIINFYVSGWVFL
PLIIMLILYIEIFRLLRKQLKQNPTNYAKKGVFYGKEYKTAKSLALVLLLFALSWLPLAILNC
VQFYNPNVKKLGLYQPTIFLFILLSHANSAMNPIIYAFKIKKFKEAYIHILRPNVNAEVYGS*
>jgi|Xentr4|314392|e_gw1.35.151.1

MGNQTTESLTTIYIVTESLIGATAVLGNTLVIWAVRLNPALQDATFYFVVSLAIADFAVGVLV
MPLAIILNMEIQMHFHVCLFVCCSIIILTNASILSLLAISIDRYLRIRIPIRYRSVVRKKRIFICIL
FSWILSALGALLPVFGWNHRSNLEENERDYLRCEFIKVMSLDFLVYFCFFGWVVIPLFLM
VGLYTQIFYLIQKNRRHNISIHQGKQNYYSKEHRTASSLALVMFLFALCWLPISIVNCISYFY
PSVAENSTFQPVVLLTILLSHANSAMNPIVYAFKIRKFKYTFLSIINKHILCKADVNESCSIDL
TLENVIPK*
>jgi|Xentr4|314680|e_gw1.36.139.1
MTNKSQFIERPDRSEQLAYVEISVLGVIFMVASAGNLTLILVLWNKRKKLSRMYVFMLHLS
LADLVVAFFQVLPQMIWDITDVFFGPDPMCRLIRYLQLVGMFASTYMIVVMTLDRFQAIC
YPMVTFQKKRALWNAPICASWCISLLFSIPQVFIFSKTEIYPGIFECWAKFMEPWGSKAYVT
WIFVAIFFIPINILIVCQIKICRTIKTNIYVKQHSDLELQEQHRIGASRASSINCISKAMIKTVK
MTVVTVVAFFLCWTPFFIVHLWVAWSSEDVTEGAAFTIIMLLGNLNSCANPWIYMYFSGH
IPRCVSHRETLSTREDSLNTASIDLGDRDCEDRTTAV*
>jgi|Xentr4|315368|e_gw1.38.27.1
MYANGAEESSDQAAVSYVTFGIFVITCVIGLLGNGIVIYITGFKMKQKSKIWFLNMAVADF
LFLLWLPLDATNTITGNWPFGSYACKLYHMCSTFNMYTSIFIMAALNVDRAISVAKPLWH
HKFITRSVCYRTCACIWILTALASLPAVFVSAVHTGGNQTCCANEETVAIWHDTIFKAYCFII
SVVVIGYFIPLCVILISNGVIVCQVWRSQATKTSKLYRITVSVVLVYFITWTPLIIAYIIFLIAIS
TMNILLVFKVSAFMVLLNSIAYTNSCLNPIMYVLIGKQARDVLAASIKSLTNTSTQGRMSK
HAENTDKLTLSSTRTNKGSHNTE*
>jgi|Xentr4|313113|e_gw1.31.58.1
TVEDTKQDWEKYRDECNERMRTEPPLSGVFCNRTFDYYACWGDTPANTTTGEPCPFYLP
WHAKVKEGFVFRRCDADGQWVRDDSNSTWRDHSQCETHEPEQSPVRHEPWILSKLRVM
YSVGYGISLAALIVAVFILTQLRRLRCTRNLIHCNLFVSFILRGVSLLTRDALLPLHHNMIQG
EGDPTNLLRNRTLVGCRVAQSITQYCVAANYYWLLVEGLYLHNLLVVLSFSEESVLPRYM
LLGWGAPVLFVVPWVVVRQLYENSVCWERNDNYSHWWIIRSPILLAVLINFFIFLRIIRILV
LKLRANQMRRSDRKYRLAKSTLTLIPLLGIHEAVFNLLPEESARGGVRYGKLGAELLLSSF
QGLLVAVLYCLCNKEVREAFTFLSHICSS*
>jgi|Xentr4|315523|e_gw1.38.448.1
MDFDDNCNTNLPTDFNKTSGIVQYTSFVLTVLTCAIGIVGNAIVIGVTGTIMKKHKCKIWF
LNLALADLAFTLSLPLHAVALWTGNWPFGSHLCKLHNYSSTCNLYASIFTIVALNIDRVLSI
AKPIWHLKFFSQRACFWTCAFIWTLTALLSLPLFNITLFFFPLPKPLGWCKIDLDDIVFITECI
LIPCLVMGYFIPLVVIVFSNVTIALQGSKSQGMRSPTLYRIITAVILFFFLTWTPVVSSEIILLA
ALRKRNIELVYKVYMIMPVLISIANTNCFLNPIIYVLAGTRVRGALSDFMSNTRLTFSRTSD
LHSN*
>jgi|Xentr4|315626|e_gw1.39.260.1
MGTLVHFQKFFIPLVYTLVFILGCLGNSLVLLILIKFRRSRSTTENFLLHLALADLLMLVTFP
FAITESVAGWVFGSFLCKFVGVINRINFFCSSLLLGCISVDRYIAIIHAIHTFRSRRLVAVHLP
CFGVWALCFLLSMPNLFVLGIQENGNVTTCTYHQSHFPSNGWWQTGRFLNHIVGFLLPLS
IMGFCYAHIVAALCRSPRLEKKKAVRLAIVVTGVFLLCWTPYNVTVFIDTLDQLGLVHNC
QVREELPIAITVTEFLGYVHCCLNPILYAFVGVKFRNDALRILRRAGCLSSLMPEVSLNFDR
KSSATDSENGTVMYSF*
>jgi|Xentr4|316065|e_gw1.40.139.1
MPVRMLLGIGAPRCLSLLLLLVCLVPWTLGCPVLIHSCKCVGERSKPSLSPANGPRKKVVC

TNEELSEVPDPALLPNKTVTLILSNNKISTIQSNSFYGLNILEKLDLKNNLISRLEPGAFLGLS
ELKRLDLSNNRIGCVTATAFQGLSSLNRLSLSGNIFSTLNPGVFDEVPSLKVVDFSTEHLTC
DCHLRWIASWTKNGTAQVSDKTMCAYPSALRNRPLRSLRENQLTCEGTPELHTHQLIPSL
RQVVFQGDRLPIQCTASYLGNSSQIIWYHNGKQVEEEKEMGIIFEETIIHDCTFITSELLLSNI
LLSANGEWECIVSTEQGNISKKVELVVLETSASYCPAERVSNNRGDFRWPRTLAGITSHQA
CYQYPFTSILPNGTGQEMKASRRCDRSGRWEEGDYSNCLYTNDITRVLYTFVLMPVNASN
VLSLAHQLRMYTAEAANFSDMMDVIYVAQMMEKFVGLVDHVRQLADVMLEMASNMM
QVDDHILWMAQVEDKACSSIVQTVEKIAGLTLSSNSQDLSLSTRNIALEAFLIKPESFIGLG
CTAFQRRDGHRGGHRREKDRREEEVESHADLRLKFRCTTGHINVSTHHFHAKNNHALAA
VHLPPSLFASTSSSTCKLQVLAFRNGKLFRSVGNSSRLAEDGKRRSISTPVIFVGTRGCGNT
NFTDPVTVTLRHLVMGSNPLPAQWNFKALEGYGGWSSEGCQLVSEKPNITIMKCHQLSNF
AILTELNRFPQEAQNGTEILHPVIYSCTAILLLCLFTTIITYIVNHSSIQISRKSWHMQLNLCF
HIAMTSAVFAGGITLTGYIVVCQAVSIILHYSSLSTLLWMGLKSRVIYKELTHKPQPQQEGE
ANQPPQRPMLRFYLIAGGIPLIICGITAAVNINNYRDNSPYCCLVWRPSLGAFYVPAGLILLV
TWIYLLCAGINLRRQPAEQKDVPETNDPQQQSGGISHLLSDSSSISLNINSAAPVAEVDNVY
SLEVQFWALLIVHILYIALWVFGAFAVSQGWYLNIIFSCLYGLTAVALGLFIFIHHCVRRQDV
QRSWFACCPSYTEALPIEAYVKTGSPVDDPPPVYNGCNPEMANSIKASSSPSNSSNSNTGP
CKITNLQVAQNQAESCPPKQASCEESEPANNKNVPVPSRYMNNLHTRRNHKSRSKQYRD
GKHHRLKVLRGPSSDQHSSESGSIHNSHSESYHSRNSPLNNGRTGGEAREPEGAITHSEGS
DSSGHHAQNFAKAQRKSASRDNLKQANSMERESKRRSYPLNTANQNGVLKGSKYDINFA
PPENTTVMKTGLWKSETTV*
>jgi|Xentr4|316076|e_gw1.40.44.1
MVNKNGSNASAPFTGVQLIQSYKPLFIPCYTLVVLIGIFGNYLLLYVICRTRKMHNVTNFFI
GNLAFSDMLMCATCVPFTLAYAFNPQGWVFGKFMCYFVFLMQPVTVYVSVFTLTAIAVD
RYYATVHPLKKRISVTTCIYVLMGIWLLSCALVGPAIAHTYHVEFQQEGFTICEEFWMGKE
TERLAYAYSTLIITYILPLSAVSLSYICITVKLKNRVVPGHPTQSQAEFDRLRKRKIFRLIVLV
VTAFGICWLPIHVFNIIRDIDINLINKHYFLHIQLFCHWFAMSSSCCNPFLYAWLHDRFRSEL
RKMFTFKRKIIPTNNCVAVSVML*
>jgi|Xentr4|316440|e_gw1.41.399.1
MRVSRVQRTSLLVIVGILLASLVTLIRSEKEHNKELEKFAAETSEPIRFRAKRGSQQEPKKD
QVALGYPRLIHPPPSGTPLLNQILNDTALTSTWHPQLQNPLYPLGESSLSAYGVLLLSLVVFA
VGIIGNLSVMCIVWHSLYLKSAWDSILAGLALWDFMLLFFCLPVVVFQEITRRRLLGVVSC
RVVPYMEVTSLGVSTFSLCALGIDRFQSITSPQMSPRPVERCQSILGKLAVIWLGSLILALPE
ILLWQFTQEQSPGSGMVIDTCVMLPAPNLPPVLHSLVLTYQHARMWWFLGCYFCLPLLFTI
TSLLVTLRIVGTSKDIKYGQCQRQLSWIVASLATVYGVCTLPENVTNVIITYTNLDVPKDLL
SLITQFFLFLKCAVTPVLLLCLSKQLGQAFLDCCCCCCEEAIRSDSKQQTVSERKETAEEPL
TLPYNTRESELHLGTPC*
>jgi|Xentr4|316453|e_gw1.41.403.1
MDTSLPSHTWNLSFNFSGASSPTNPQYGALFLMLLMDLLAVAGNVAVMGVIMKTPSLRKF
VLVFHLCVVDLLAALTLMPLAMLSGGGGTYLYEGQGLGQMACRAYLFLSVCLTSTGILSI
SAINIERYYYVVHPMRYQVKMTMGLVSWVLAGVWVKALLTSLVPVLGWNPPAPGHCSL
QGGGNSVFRAGFLLFYSSFYFLLPLTIIIVVYCSMFKVARVAALHHGPLPTWMDTSPQRRR
SESLSSRSTMVTGSGGTRGTPQQRLPGGGGGGKAAAVLAAVGGQFLLCWLPYFGFHLYA
ALRSPPPPPGSRAEWVVTWMGFLCFASNPIFYGCLNRQIRLELGRWVGCFFKRGGAGEDE

LRLPSREGSIEENFLQFLQGTGCPPEPRAPTHNIPKGGHPAVDFRIPGQIAEETSEFLEQPWD
VTAVNRDYISTGPSPKT*
>jgi|Xentr4|317125|e_gw1.44.40.1
MYDAILETNDGSSSDAGWYFPYPLGFQVSLTSFLMLEIVLGFSSNLTVLVLYCMQSNLVDS
VSNMVTMNLHILDIIICVICVPLTIVIILIPLEQNIALVCCFHEACVTFSSIATAINVLVISLDRY
DISVRPANRVLTPSRMVLLLSCVWFVSLMVFFIPFFEVEFFGDPDHMATWQNRTLLCVSVN
EYHTELGMYYHLVIQIPIFFAAVAVMLVTYSKILQALNIKIGNHFKRSQRRKTKKKKKRKSS
DQSTVGETKRLAPPAVSQNPPMRVQASVSVIIALRRAVKRHRDRRERQKRVFRMSLIIITTF
LLCWAPISIVNLLILCLGPSDLLVKLRICFITMAYGTTIFHPLLYAFTRQKFRNVLKNKMKKR
VVSVLQVDPAPGGTVIHNSWIEPKKGRKSKLECSDGTDRCLTDAVKE*
>jgi|Xentr4|318246|e_gw1.48.188.1
MEVPEEVCNYRPYSAGVLEAARYTSFALTALSCVLGLGGNAVVIAVTGFIMKRKNSKIWF
LHLAVADFTFCLLLGLYAHYVLTENWQFGSYLCKISNYVSTCNMYASVFIITALAEPHCMY
VLAAPIWHRKFFSVRICCWTCAAIWIVTALLSLPVLLLSDEIQYGDKMQCRIVIVKPSSATH
NIHKREANDSLEDTAFATGCIVIPCLVLGYLIPLAVILVSNLIIAQRGGKSQSVKSPRLYRIIIM
VILFFFLTWTPLITVQVILLAALYGEDAILMYKMYWVLPLVSSIAFSNSCINPIIYVLVGTQA
RKALTDFMSTILSFLSGTQSNG*
>jgi|Xentr4|318318|e_gw1.48.206.1
MPKAESLTADPCRDSIKLQANFTQISEPVRYASFVLSILTCALGLVGNALVIWVTGFHMKK
HRSKIWFLNLAVADFAFALCLPLYCVALFTDNWPIGRYSCKVYNYVSTCNMYASIFIITTLTI
DRVLATAKPIWHHKFASRRICYSTCAVIWVITALSSLPVFLLSETVIHGENVQCNSLETLSAP
KTSCDVDIAENYFLTKSILIPFLVVGYFIPLLVILFSNIIIAHQAGKSQSVKSPRLYRIIIVVILFF
FLTWTPVVIAQIILLVSLNNHNLILMSQIHRIMPLMTSIAYTNSCINPFIYALAGTQLREEISG
FISSTRKSLSKSSTSGRSYVEQ*
>jgi|Xentr4|318531|e_gw1.49.132.1
MENSTDDDPESNLTFCIVDHTLRQTLFPVVYVAVLGVGLPANCLSLYYGYLQIKAKNELGI
YLCNLTVADLLYIFSLPFWLQYVLQQDNWTYNETMCRICGILLYENIYISIGFLCCISVDRY
LALVHPFRFHKLRTMKAALVVSMIIWLKEIMTSYIFFIHGEVSEDPESHIVCFEHYPIKPWE
HNINYYRFFAGFLFPIILLLFSYCCIFREVGNSKGAQIKKKLQVKQLVLSTVIIFLVCFGPYHI
LVVIRSFFERNCFFAVKIFNVYHFSLLLTSLNCVADPVLYCFASENTYKDFIKLKSSCMTSLG
CLKDKQKEPQQLNSAETYTATQVPFEIEPVELHEKKFPSTKHSV*
>jgi|Xentr4|318784|e_gw1.50.6.1
MIIGGVFPVQRSRSTEDLSFTSNPDSTVCDGSFDFRSYRWIRGMIYAINEINRNEHLLPNFTL
GYAIHDTCDNVAESVEKTFTLVTGSVKYTPNYQCQITPLLTTVIGESASGISIAMARILGTY
HYPQVLYYSSLNVLSNKKEFPSFFRTIPSDTFQTTAFALLVAHLGWKWVGTLAEDNDYGH
LGVQLFTEEVQKLGTCIAFSETIPLAFSKTRYEEIAATIKRSSATVIIVFSGDTNLLPLFWEVA
KQNITGRTWLASEGWSTSAFVVAKDQAEYFSGALGLAIPVGHIAGLLEFLLQLNPMADPE
DLYIKPFWEHAFGCSWTETKLQDNITLCTGHENLLTLNNVYTDVSQLRITCNVYNAVYAI
ANALNNILSSGNKKPAIEEIKPWQIIQSLKKVRFTDQAGKILYFDENGDPVAKYDILNWYIG
EDGNLLYRKVGSYDASAAEDQRLTLDEQAIIWNDALQEPPRSQCSSSCKLGFRKSILQGQS
MCCFDCIPCSEGEISNGSDFTECIKCPEDYWSNKNKDSCIPKDMDFLSFEEPLGITFSVLAVI
GISQTLAVVVVFIKHIHTPIVRAHNIEMSFFLLFSLILCFLSSLTFIGKPSSGLCVLRQTVFGV
SFALCISCVLVKTLVVILAFTMSKPNQNMMKYFKPSHQRAFVGLATLIQIAICVGLISSANL
SIKKNRDAVITKIVLECNLELELVFLFAIGYIALLALPCFVLAFLARNLPDTFNETKFITFSLL

LFVMVWVSFIPGYISTKGKYVTAVEIFAILSSAAGLLGCIFFPKYYIILLKPERNTRKNVTGH
SSLVRM*
>jgi|Xentr4|319833|e_gw1.55.338.1
MFQLIILSLTLFFGTLFNSFAIWVFCCKMKKWTETRVFMMSLLFSDCCLLLTIPFRIYATQHR
WDLPRELCTTLAYAYFMNTYLSIAIITLISVDRYIAIKFPLRSRSLRSPKKAALACAIVFALLL
CTRIFILLTAEKSVPSPGFCFRKILTRPLKRNLYFTVTGFYIPLPIIIFCTTEVIRFLKMKDKTSV
YEHECIKKSIKIMSANLIIFLVCFLPLHILNIIRFAMESLKLDCTLIKLINDYVYAAQVIGDLN
CCLDSVCYYFVAKEFWESTSVLPRSNHLTTSPEQTEDTNL*
>jgi|Xentr4|319906|e_gw1.55.355.1
MNCSNVTQVTMSSLQKFSMTVIIPVFFFGIMCNSLALWVFCCKMKKWKVTVVYMVNLII
SDILILTTFPFRLYTYFYSSSLGNDLCKALVSLYLMNTYMSIFTIATISVDRYLAIRFPVKSKL
WRSPGKAAGKCCMLWLFLISISTVLALKIDYIIPVCFQKVSNTPNGLFVLFAVVGFIVPLIIIS
FCTIQVIRTLYGKETKDANEQRSVRKAINIILSNLVVFVICFLPVHMGYIIRFVAETVEATCH
TLEQIQVFSNVATLVANSNCVLDSLCYYFVAGEFWKASDLSPQIKYSHAKKRTLAAE*
>jgi|Xentr4|318799|e_gw1.50.1.1
MDMPKATFTFNALVFSLTLIITLTVFLGSIISLVSLLKMKSKSTLSLLVTSLSVDDLVSVVPAS
IFMLHQWTTHALPQPLCSAAAFLYLFQGISSNLKASSIICYNFYTVNKLGSYETTKISVKIFC
AVATIWVISILISAIPFFGWGRFHTTSWGCFTDCTSSYILFLFGLYSLCFCILVILSAPLTHQLL
CSDDHQHINYKDISKGYLTPTTPLGSNASSLSPDNPVGKTLKNSNEKYVSPEAVFKRSVAE
TSGDVQHSSNSVRGGRNITVLYAQKRFSLILALTKVILWLPIMIQMVVQHIVAFQSLSFETL
SFILSLLSAAATPLFVLSERWRHLPCGCIINCKRNTYETSAEENQIKKRGFDFNLSFNKGYGI
YKISQDNHATGENSISYHNLINYDCENMNETRCDRMLNSAMLDFSTISFQDSSLLRDANM
LKIPHTDAKLEQCKEKAFGCLLSEKIGAFKRDDVRNFDKSTFYEGQERRLSHEESRKPELT
DWEWCRSKSERTPRQRSGGALNIPLCAFQGTVSLHAPTGKTLSLSTYEVSSDGQKITPTSK
KIEVYRSKSVGHEPNKEDSPNQFPDTNVKIHLEVLEICDNEEAMDTVSIISNISQSSTQARSP
SLRYSRKENRFVSCDLGETASYSLVIPTNNPDSDINISIPDTVEAHRQNSKKQHMERDSYQE
EIQLLNKAYRKRESDGDN*
>jgi|Xentr4|320056|e_gw1.55.17.1
MENGNQTSEEFFFRAFLSSTRAPAMLFNVFLFIYLLILVWNGLMMLLIKSDSHLHIPMYFLI
FNLSFLDICYTSSVVPQTLASLLNPKHSITYSSCAAQMFCIAIFGIAQIVLITVMAYDRYVAI
CNPLCYRRVMTWTV*ILMVAAVSILSCVLATVIVLSVFSLPFCTSFEIDHFFCDIGQVLHLTC
PGVTMHAIVEILTFGIGLAVVSINFLLVLTSYMYIIAAILKINTSKGHLKAFSTCSSHISVVAV
EYAFLGFLYLRPKSTYSLDRDRILHVIFLFGTPILNPLIYSVRNKAVKRGFTKLFGCKKGHK
MT*
>jgi|Xentr4|320328|e_gw1.57.166.1
MWTTEGYDYDTLDYTDGSLISLCEKEDLQSFLSFYPIVFYILFVLSITGNMMILIILLKWEK
FNTVTNIFILNLVISDVLFSVTLPFTAFYISSSWIFGNVMCKLTTAFYFSGFQSFVIFLTLMTID
QYLMIVHSWSSTSRSRIQYAVNISVIAWCLSILFSLPEVILSSTKVMNTGETVCEMLTFQNE
QNKWWLVIGHYKHFSLFFIIPIIIIVICYIGIALKLTTCNIRRKAKVLKLIFVIAFLFFLCWIPY
NITMILMFQETIESFNNCKSVLHYVFYISQTLLYIHCCTNPLLYTFLGTKFKRYFRCSFSRLC
LPRVLSMEHDISLRTSMLNAV*
>jgi|Xentr4|320919|e_gw1.60.4.1
MGRIASEKNENRTEDIKTEMNTWFYAAGLTTPYNDLTKPELIDITKLLSVQVVLILAYSSIIL
LGVLGNSLVIYVVIKFKTMRTVTNFFIVNLAVSDLMVNTLCLPFTLVYTLLDDWKFGTVL

CHLVTYSQGLAVQVSTVTLMVIALDRHRCIVYHLESKISTKICFMIIGITWVCSALLASPLAI
FKEYSVIVISSDFQIQACSERWPVGDLNYGTIYSISMLLIQYIVPLAIISYAYIRIWTKLKNHV
SPGGGNDHYHQRRRKTTKMLVAVVVVFAVCWLPFHAFQLASDIDGKVLDLNEYKLIYTIF
HVIAMCSTFANPLLYGWMNNNYRTAFLTAFKCEQRMDSIHPEVSVAIQLKKKQLEVKEIN
GTSDNSHGTCTPIANV*
>jgi|Xentr4|321074|e_gw1.61.148.1
MDPWLILYVTGFISMDLIGIPGNLFILFAFLHIFHHHGKVTMGEIILAELALSNLLVILTWGIP
ITLQTTGLIKVYKDIFCQINLYFYCIGRAMSVSITSILGCFQCVSITPTAKLCLPLKRKFLDHL
YAILVLLWIFNLIVSSTRLLYSTVSLKNVTSPYVMSYGYCFVIFPSYLVYLGNGVIYVVVRDL
FFLSLMMLTSGYLLHVFYNHRKQVKCIQVYMNKHAEIQAAKAVLTLLIMYIISFGLDNVF
WIITLYAYKWSHRITEARIFFDSCYSAISPVVIILTNKKIQMGLRCSKKEKKPNSVKAISTVV
LGRGSR*
>jgi|Xentr4|321098|e_gw1.61.12.1
MQDLLKGLIFLLQMKFGVIANGIILVDYLGNIFSGIKKIDFIVCHLTLVNLVSLLSRGIPYSM
MRLGVPDVLTDVGCKAVIYTYRVTRALSITSTCSLSVFQLATIAPTVSPWAIFKSLVTKYILY
SFVGFWIFHMCANSTIPLTVGAPVNNTIPQRAVHLGFCYAIFLDYIPEIVSTMFTTGIDYLNV
TIMTLCSVTMLVLLKRHSQQVRYLQSTTQSVEVKASKIIISLVSLYVLFHGIDSLLLLYSISR
KELSSILAEVRVFLSSSFAAISPLILINFSKRMRAKWPLRKKKMLATKITNVSHVSC*
>jgi|Xentr4|321123|e_gw1.61.154.1
MDPNLLLKAVGFLLLVIIGIPGNVYILMQFTYLRITEKKLLPTNIILMALALVNMLVILSRIIP
QSLNALGVEELLDDTECKLVIFTFRVNRAMSICVTSFLSCYQCILIAPNTKIWIYLKHKVTQ
NVVAIIFIFWIINIAIYPYVFFTAHARKNETTSPYTLHLAYCDADFFNFMAYIVNGAVYSLRD
IIFVGPMALASSYIVFILLSHEKSVKGIRSSDKSQRRSVEYRASKAVILLVALYVMLYGLDN
CMWIYTLTLSNVSTNMSEIRIFLASSYASLSPIVIIITNPKLQQNLFTCSRKRDSRDDSKTEEH
VYAISK*
>jgi|Xentr4|321132|e_gw1.61.23.1
MYLLIKDLLKALVFIFLAVLGIPGNSFILLKFAYVRIAERKLLPTNIILMFLAFMNLLVVLYR
VILQILYAFGVKNVLDHDGCKFVFFNYRVGRAMSICVTCLLSCYQCILIAPNTKLWINLKL
KMTKQFLVFTIMVLIFINGCLYSSVLLYEAANGNFTTSPYSIHVIYCHADFLTYISYVGYGTI
YTLRDFLFVGLMTIASSHMVYVLLKHEKTLKNLKKSERQTKSAEYKASRSIIMLVALYVFL
FGLENIIWVYTLTIPSPDISINDVRIVLACSYATISPVIIIKTNPKLSLCIKNSNTESHIRIQKNY
NITCLSR*
>jgi|Xentr4|321168|e_gw1.61.18.1
MYILVKFAIIKVTEKKLLPTNSILMVLAFMNLLIVFSRINLQFLNAIGVENFLDDSKCKFFV
YTYRVGRAMSICTTSLLSCYQCIIIAPQTRLWAYLKQKAMQNVFFITTAILIANLILYPSSIIY
AIAKNNSTTSPYTLRLVYCDMDYLTYETYIINGAIFASRDFLFVGLMTVASIYIVYVLFCHE
KSIKGKRSTDRLPGKSVEYKASRAVVMLVILYVILFGFDNSMWIYTLTLSNVTPDMNDARI
VLACSYSALSPIVIIATNPKLQQMLTFLKRRKFVLCYGSAEEKDIRVNCIDK*
>jgi|Xentr4|321254|e_gw1.61.207.1
MIKGVSFFIQTSLGIFGNSIILISYANMLSRGPKLMPVDIVLSHLAFVNMMVLLTRGIPQTMS
VFEIEHILNDTGCKIVVYSYRIVRGLSVSVTCLLSVLQAVIIAPVKRWTIVKIKEVKYLSLSL
VAIWVINMAVCIAAPFFSKVPGTGKISKFTLNLGFCHVLFPDQVSYIINGFAVSFRDFTFVG
VMVYSSGYIIRILYRHRRQVQNIRRSTSAQNATAETKAAKDVLRLVLLYVIFFGLDNIIWIN
MLTVSDVPSVVTDLRVFFSSCYASFSPIFIIRSNKRIQKALKCSFKRQQTSQPEVYVCHI*

>jgi|Xentr4|321674|e_gw1.63.218.1
MFCFSAILTVLGNCAVLATAVKCSSHLKAPDLLSINLAVADLGMAISMYPLAIASAWNHAW
LGGDASCLYYALMGFFFGVSSMMTLTVMAIIRYRVTSSFKYSGCTIEKKAVCILIMCIWLYA
LLWAVLPLLGWGRYGPEPFGTSCTIAWGDFHHSSNGFSFIISMFILCTISPAVTIVVCYSGIA
WKLHKAYQEIKNQDKIPNSTKVEKKLTLLAILVSFGFLISWTPYAAVSFWSLFHSSKYIPPV
VSLLPCLFAKSSTAFNPMIYYAFSKTFRRKVKHLKCCCGWRVHFLQSENSVENPRVSVIWT
GKENVMVSSVPKLMKGVPGTPTGTQ*
>jgi|Xentr4|322001|e_gw1.64.184.1
MGLLSLPNETEPDNETLEMLENDYIAVILPVAYSIVALIGIPGNLFSLWILMFHTRPITSSIILM
INLSITDLVLAAFLPFQVVYHIYRSDWIFGKPLCTLVTVLYYANMYSSLLTIMLISIERYLGV
VHPMKSGMWRRKRYAIAAVIIIWILVMFILYPLLKTDLTYDVKHLKKRTCFDVLKWTMLP
NLMAWAAFIISLFLFYFLLPLIVTVICYVLIILKLVQTSNRYGRGQERRSIHLALMVLLVFITC
FAPSNFILLVHAINRLFFNKSYYHAYKLSLTTSSLSSCLDPFLYYFASKDFRKKAKEVWAKK
VKQNETCETRRSSIFSALSGRSLTISSGHGEVFENGHTHRNCKRQESDV*
>jgi|Xentr4|322040|e_gw1.64.134.1
MLQSKMLQTALPILYLLIFCISVPLNFSSLVMLWGNKPWTPTVVFLLNLAITDLIYGITLPFQ
VIYHLRGNDWPFGDTFCSVATILFYGNMHCSILTMSSISVERFIGIVLPLRSKNCVTVRRALL
TCLLIWPLVLLVDLPLMISKLTFHVQELHIVTCFDVLPKRLFSSQTYFSLYISCRLILFFFFPL
VIMGICYLSIIIALLRSDTIKAETKRQTIHIIVVLFTVFLVCYLPNNILLITHYHFYSKGKPLYI
EYKLSLALTSLNCCLDPVVYFFGSKQFRQKVQHKAGCCKSDNNAQENTRTLTYEYKAPV
SSY*
>jgi|Xentr4|322308|e_gw1.66.7.1
MNHFIKFVVYITVLILHCRFSLRYYSHPLVFKFAIEEINRSSWILPNVTLGYQVFDSCGAESKA
LSGALDIFSGRDYRIPNYACLKNMKLIGLLGDLSSTSSYPIAQLAGLFRYPQASIYFYGATD
PVYNDRIQFPSFYRTVPNELSEMNGIVQILKHFGWKWVGLIASSSESGEQALDRIRKGIESY
GGCVAFSIFLSEARFSSYFLFLIEAESIVNKINQKDVDVIVLFLTPMHVTVFKRLFSSRQITR
KIWLSSSFFPTVIHVTHGYSRTTLNGTLSLSAQGGEIPGFETFLYRMTPINYPNDDTVTEIW
ETLHECSFTGFLKTNTSVPVQKCSGNESLDDEVLSRFDFRVGYQVYTAVYALAHSLHNLFS
ARAPAHPQAPAGSLKHRFKPWQLNAFILNVTYKTPSGDTMFFKDNGDPPAQFDIVKWLFL
NDDSVVSRKVGRFDESNDKNHFYINSSADLWSPYFTEMPRSLCSEPCSPGYRKSKIEGEPP
CCYDCVQCGDGEMSNTTDAVTCVKCPEDQKSNRQKTDCVPKAVNYLSYMDTLGASLAS
AAIILFITASVVLGIFVKYWETPIVRANNLYLSCLLLISLMLCFLCTLLFIGRPTQICCLLRQV
TFGVVFTISVSSVLAKTLTVIIAFNATKPGSKLKKYVGTQLAIILVIVCFLGETLICVVWMAS
SPPFPEADTSSESDTIILLCNEGSVTFFFCIIGYIGTLALLSFIAAFLAKDFPDRFNEAKNITFS
MLGFCSVWGAFVPAYLSSKGSRMVAVEIFAILSSSAGLLGCIFAPKCYIIFLDLS*
>jgi|Xentr4|322315|e_gw1.66.153.1
MDTSFSKVIQFPSFYRSNELFEVDGIVRLLRHFGWRWVVLIVSDDDTGYRASKVLKEGIES
DGGCVAFIGVMHNPLEMRNGKLISNIPRTTANVIVLFLITDYIELYLHVFHCLEMPPKVWIL
PSLFAGKTMIKNSKCENIFNGAFSFSADLGEIQSFHQFFYDLRPYRYPDDVLMAVMRWVM
YSCSAELKQTPTDHLRDRCPRTEPGFGLQAQFEAYPAIYMVAQSLYEMYVFKDTRYHTKN
RPGVFNLFMQHVVKNTSTGDSYFFREKRDGLDRLDIVQWIFPPKKPFVKKTVGKCYVSES
QENRLYIINSSDLWTPYFSKFPQSLCNEPCPPGYRKSKIQGAQSCCYDCIRCVEGEMSNTTD
ASSCFKCPEFQRSNSQRDGCVPKYVKCLSYEEPLGTSLTSAALILSVTCAVIQGIFIKYRETP
IVRANNRYLSCLLLISLMLCFLCTLLFIGRPTQICCLLRQVTFGIVFTISVSSVLAKTLTVIIAF

NATKPGSKLKKYVGTQLAIVLVIVCSLGETVISIVWMASNSPFPDTDDVSNVDSVILLCNE
GSIIYFFSVISYMGMLALLSFIAAFLAKDFPDRFNEAKNITFSMLGFCSVWGAFVPAYLSSK
GSRMVAVEIFAILSSSAGLLACIFIPKCYIIFLRPELNTKANRII*
>jgi|Xentr4|322363|e_gw1.66.8.1
MIFEYILTLILLYRTSFKYCHHLLVFKFAIEEINKSLWILPNVTLGYQIFDSCASSPKALSGAL
DIISGANYKIPNNACLRNMKLIGVLGDLSSRSSYPIAQLAGLFRYPQASIYFYGATEPVYND
RIQFPSFYRTVPNELSEMDGIVQILKHFGWKWVGLILSENDSGERALDRIRKGIESYGGCV
AFSVFLSDTIIFHLFLFDGKSIINKINQKGVDVIVLFLTPMHQKIFLRLFSSAQITRKIWLSSSF
FPTVIHVAHGYSKTTLNGTLSLSAQSGEIPGFETFLYRMTPFNDPNIDENIVILELLHDCPVV
GFLKTNTSVPVQKSSGNESLVDEVLSRFGNFDFRVGYQVYTAVYALAHSLHNLFSARAPA
HPQAPAGSLKHRFKPWQLNAFFHNVTYKNPSGDIMFFKANGDPPARFDIVKWLFLNDGST
VSRKVGSFDESNEENQLYINSSADWWSPYFSEVRMPRSLCSESCSPGYRKSKIEGEPPCCY
DCVQCGDGEMSNTTGRNNAVTCVKCPEDQKSNRQKTDCVPKALNYLSYMDTLGASLAS
SAIILFITTSAVFRIFVKYWDTPIVRANNQHLSCLLLISLMLCFLCTLLFIGRPIQICCLLRQVT
FGIVFTVSVSSVLAKTLTVIIAFNATKPGSKLKKYVGTQLSIILVIVCSLVEFLISAVWLAYNP
PFPEADTVSDPDYIILLCNEGSELFFFCIIGYIGTLALLSFIAAFLAKDFPDRFNEAKNITFSM
LGFCSVWGAFVPAYLSSKGSRMVAVEIFAILSSSAGLLGCIFAPKCYIIFLRPELNIREIVVRK
S*
>jgi|Xentr4|322370|e_gw1.66.146.1
ISYGSTDPIFNDRTQFPSFYRTIPNEEAEMDGIVQILKHFGWKWVGLIISNDDTGYRARERIS
KGLSDIGGCLAFYILINDTFCLDDFYENTVIETIERTSANVIVLFISTKYTFAFFNLFSFNQMP
RKVWITSSFFPNTFYFRKKKIETTFNGSLSVLIQEGEIPGFEEFFSTFSPNENPKNELTQTTW
EGLFHCYFLDNPSLIRTVLGAKVIDCTGNETMSEADVSVYGDHNYRVTYRVYTAVYALAR
ALHNLYSAQPPANHWDKLQILSFQLNASVRNVSFITNGGTHSLSQYGDPPARFDIIKWIFLP
NKYMLKTKIGNFYASNGTHFYINSSDDLFGPHFKMIPQSLCNEPCAPGYRKAKIEGKPSCC
YDCAKCADGEMSNTTDALICLKCPAYQMPDRKRTACIPKATNYLSYIDTLGASLTAIAFIFF
ITASVVLGIFVKYWETPIVRANNQNLSCLLLISLMLCFLCTLLFIGRPTQICCLLRQVTFGIV
FTISVSSVLAKTLTVIIAFNATKPGSKLKKYVGTQLAIVLVIVCSFGEMLISTVWMASNPPFL
EADMFFEMDTIVLLCNEGSVTFFFCIIGYMGTLALLSFIAAFLAKDFPDRFNEAKNITFSML
GFCSVWGAFVPAYLSSKGSRMVAVEIFAILASSAGLLGCIFAPKCYIIYLRPELNSRKTTIRK
*
>jgi|Xentr4|322387|e_gw1.66.251.1
METENITIVKEFILLGLSTNPTIRILYFLLILPMYFMMLFGNIVFIYTITQTPKIHTPMYFFLCV
LSFLDVCVSSSTFPKMFVDTFLQEGRISVLGCMTQMGTIIVLASYECNLLTVMAFDRYIAIC
YPLHYMTIMTWRVCKTVTGILFVFCFLSVFLPTIIKPMTFCIENKLDHFACEILTVLQIMCG
DVSHYERSIFYQTLFATLLNLVFIVVSYICIIISLLKISSTVGRTKAFSLPPI*L**VCFMGQL*Q
CI*VWEITFHLVW*SMFLLFMVL*LPC*ILSYTV*EIMKLKKQ*RRISTPRH
>jgi|Xentr4|322554|e_gw1.66.10.1
LIYTIGEINKDPEILPNVTLGYRIYDSWASGMISFAGAFSILSGTEQPIPNYSCWNNRKVVGF
IGDLSSESSLSIAWLAGIYRYPQISYGSADPIFNNRLEFPSFYRMIPNDLSEIDAIMSLIRHFG
WKWVGLVVSDDFTGPRASERLQNQMNKDGGCLAFIIRIKNLSDTKVNKMDIYRSIEQTIY
ETTTKVIILFINLQYIDSFNLFFALYKIPKKIWIASSSFSRVIELRYLHVQLTFNGTLVLSLQQG
EIPGFRQFFYSLNPYKYRDDTLFSNVWEILFSCSFLDLVNDKTAKISLPKCTGNETFDDTVL
ESYGTFNYRIAYGVYTAVYTMAHTLHELYGTMTHSPKSAESLHMYFKQWQLNALMRHV

AFRTSSGDQIYFRDNGDPPANYDNLKWLFLRKGNVQSTKVGSFDTSRSDGDQLFINNSAN
LWGPYFSEFVHSRCSEPCKPGFSKAKVEGAPSCCYTCVLCADGEMSNITDAQSCTKCSKY
EKSNSGRTSCIPRDINYLSYDDHLGSSFSSISVTFSISCAVILGIFIKYRETPIVRANNRYLSCL
LLISLMLCFLCTLLFIGRPTQICCLLRQVTFGIVFTISVSSVLAKTLTVIIAFNATKPGSKLKK
YVGTQLAIILVIVCCLGEIIISIVWLVSNPPFPEDDTLSDADIIILLCNEGSVIFFFCIIGYIGTLA
LLSFIAAFLAKDFPDRFNEAKNITFSMLGFCSVWGAFVPAYLSSTGAKVVAVEIFAILSSAA
GLLGCIFFPKCYIIFLRPQLNTRDYIMHK*
>jgi|Xentr4|322644|e_gw1.67.33.1
MEQANQTTVSEFILLGLTNDPTTELFLFIFFLLIYIIILFGNISLISAVVFISDLHKPMYFFLSNL
SFSDLCFATTVTPKMLYNMLSGIKSIDFNSCALQLYFFAGFASTEAYMLSAMAYDRYVAIC
HPLLYTVIMNRKARIVLMAGVYMTGFLATSIHTFCTMRLSFCGPNTINHFYCDIPPLMELS
CSDTYISKTIIFVVVFCLGCFSVAVTLASYCYIFFTIMNIKSSGGRLKTFSTCSSHFLCVSLFY
GTVFFMYLRPASKYSVSQDKVVSVFYTMIIPMMNPIIYSLRNAEVKNAVSWYINKLLLSKI
LKFKTV*
>jgi|Xentr4|322724|e_gw1.67.6.1
MGDILGNQTVISELILLGVTDDRKAELFLFGLFLLIYIITLFGNSFIILSVIFISSLHSPMYFFL
GNLAFSDLCYSTVVTPKMLRDIVSERKSITFVGCALQLYFFAVFASTECYMLSAMAYDRYL
AICQPLLYAVIMNRKAIIHLAVGIYVSGFLTASIHTACTLTLSFCGPNTINHFYCDIPPLMELS
CSDTYIHKTVIFVVVFCLGLFNVAIILASYFYIFYTIIHMQSSRGRHKAFSTCSSHLLCVSLF
YGTVFFMYLRPASKYSVSQDKVVSVFYTMIIPMMNPIIYSLRNAEFYHAVSWYIKKLLLSN
ILEL*
>jgi|Xentr4|321090|e_gw1.61.135.1
MQDLLKGLSFLLQMNFGVIGNAVILAAYLGALFSGIKKIDFIVCHLSLVNLVSLLSRGIPYS
MMLLGVPDVLTDVGCKAVIYTYRVTRALSITSTCNLSVFQLATIAPTVSPWGKCKNLVTK
YILYSFVGFWIFHMCAYVFIPLTFGAPVNNTIPQKAINLGFCYAKFLDYIPETISTIFTTGIDY
LHVAIMTLCSVTMLVILKRHRQQVRYLQSTTQKVELKASKIIISLVSLYVFLYGIDSFLSLYFI
SRKDVSLIVTDMRVFLSSSYAAISPFILIHFCKKVFAKWLSRNKESISVKITNVSHVSC*
>jgi|Xentr4|321096|e_gw1.61.20.1
MNILLYIKAVGFFLLAAIGIPGNMYILFKFVKIKVTIKKLLATNSILMVLASMNLLIVFSRINL
QFLNAIGVENFLDDSKCKFFVYTYRVGRAMSICTTSLLSCYQCIIIAPQTRLWAYLRQKAM
QNVLLITTAVLIANLILYRNTIMYAIVKKNSTTSPYTLHLVYCDMDYLTYEAYILNGGIFAS
MDFLFVGLMTVASIYIVYVLLCHEKSIKGKRSTDRVPGKSVEYKASRAVIMLVILYVILFGF
DNSMWIYTLTLSNVTPDMNDARIALTCSYSALSPIVIIATNPKLQQLLTFLKRRKCFFCYRS
AKGKDIQVHNIDI*
>jgi|Xentr4|322329|e_gw1.66.140.1
MTFDYISSLILLYRTSFRYYRHLLVFKFAIEEINRSSWILPNVTLGYQIFDSCAAPPKALSGA
LDIISGANYKIPNYACLKNMKLIGVLGDLSPTSSYPIAQLAGLFRYPQASIYFYGATDPVYN
DRIQFPSFYRTVPNELSEMDGIVQILKHFGWKWVGLIVSDSDSGGKALDRIRKGIESYGGC
VAFSVFLTETHFLSFFFFWFDVKRIIDKINQMGVDVIVLFLTPMHQKIFIKLFSYRQITRKIW
LSSSFFPTVMHFAHGYSRTTLNGTLSLSAQGGEIPGFETFLYRMTPSNYPNDDTVTEIWETL
HECSFTGSLKTNTSVPVQKCSGNESLDDEALSRFGNFDFRVGYQVYTAVYALAHSLHNLF
SARAPAHPQAPAGSLKHRFKPWQLNAFIHNVTYKTPSGDIMFFKANGDPPARFDIVKWFF
LNNGSTVSRKVGSFDESNEENQLYINSSADWWSPYFPEMPRSLCSEPCSPGYRKSKIEGEP
PCCYDCVQCGDGEMSNTTDAVTCVKCPEDQKSNRQKTDCVPKAINYLSYMDTLGASLAS

AAIILFIKTSAVLGIFVKYWDTPIVRANNQNLSCLLLISLMLCFLCTLLFIGRPTKIFCLVRQV
TFGIVFTISVSSVLAKTLTVIIAFNATKPGSKAARYVGTQMSILVVFACSLGVTLICVVWMA
SSPPFPDVDTSSETDTIILLCNEGSVTFFCIIGYIGTLALLSFIAAFPSQGFP*PI**G*KHHFQY
VGVL*RVGGI
>jgi|Xentr4|322891|e_gw1.67.37.1
MLNSTKFSDFILLGLTDDPSLKILLFVLFSLFYVVTLFANTGIILIISASSRLHIPMYFFLKNLS
FLDLCYSSIITPKTLAGFLSGGKVITYIECALQMYLFGASVTTECFLLGIMAYDRYVAICNPL
LYLMLMNSRVCRQLVGSAYIAGYLNAAIHTTCTFQLTFCKSNTINHFYCDVPPLLKLSCTN
TMMNELVMFIFGGFAEMGSLITITVSYSYIIIILQHHCLYVLAPSL*LCYESRPHCLCLLHYN
YSYVEPTNIQLKKQRSRKGPDENLWKCSNNIQTPCWDNYL
>jgi|Xentr4|326770|e_gw1.83.131.1
MPQIIPNFYLALPIIYSIICAVGLTGNTAVIYVILKAPKMKSVTNIFILNLAIADELFTLVLPINI
VDYLLLQWPFGEFMCKLIISIDEYNTFSSLYFLTVMSIDRYLVVVATVESKKLSYRTYRAAK
IVSLCIWIFVTIIILPFVVFAKIHKEHGRLQCLFVFPNPESLWWQMSRIYTLIMGFAIPVSTICI
LYTMMLYKLRKMRLNTNAKALDKAKKKVTFMVIVILAVCLFCWTPYHLSTVVALTTDIP
QTPAVLGISLFITSLSYANSCLNPFLYAFLDDSFRKSFRKLLECRSS*
>jgi|Xentr4|327015|e_gw1.85.120.1
MTNITSPSSSHCQGHVGTKVLVTTVYSLFSVSGTLANVLVIYLVCSFKKLKTTSNAFIVNG
CVSDLLVCAFWMPQEVILLSTGRVENHAYRVFMEGVFFLWMTVSLLSHALIALNRFVMIT
KLPTVYHTVYQKRNTEWMIAMAWVLPLAFLLPWLFGQRPLSSTYTAILSAVTVLSQTAIV
LHCYFRIFRKVQISLKRVSVLNFQVVHNLPCSCPRKDKRLGLYVLIVCCVFALTTEPFAWT
VLYGLFQPLPRTLITGSWLLFCLLFVLNPFIYTWKNEEFRRSFRAIVGGELWKNTAIAADPA
VQTISQNEP*
>jgi|Xentr4|327450|e_gw1.87.104.1
MLRREEVALSYQIVTSLFLGTLILCAIFGNACVIAAIALERCLQTVANYLIGSLAVTDLMVS
VLVLPMAAHNQVLNKWTLGQVTCDIFISLDVLCCTSSILHLCAIALDRYWAITDPIDYVNK
RTPRRAAVLISITWIVGFSISIPPMLGWRTPEDRSDPNACKISEDPGYTIYSTFGAFYIPLILML
VLYGKIFKAARFRIRKTVKKAEKKKVADTCLSVSPAVAERKSNGEPSKNWKRTVEPKPSA
CVNGAIRHGEDGAVLEIIEVHHYVNSKSHLDLPNHAHDMAVAPCSEKRNDRATDAKRKV
ALARERKTVKTLGIIMGTFIFCWLPFFIVALVLPFCETCHMPHLLFDVINWLGYSNSLLNPII
YAYFNKDFQSAFKKIIKCKFCRQ*
>jgi|Xentr4|327701|e_gw1.88.112.1
MTPTFPTTLLESSYIEFNATLNTNISFNQNGTSPGLLIPLVYLVVCAVGLWGNTLVIYLAWRS
PAGQNSVTALYILNLALADDLFMLGLPFLAAQNALSYWPFGSPACRLVMTLDAVNQFTSIF
CLTVLSFDRYLAVVRPIQSAKWRKPKVAKCVNVTVWILSFLVVLPVVFFSGVPGDTGTCHI
AWPEPAQMWRTGFILYTAALGFFCPLLVICICHLLIVAQVRSSGKRVRVAPNRRQGPERKVT
KMVALTVTAFVLCWFPFYALNIINLLWPLPESPKLYGLYSFVVALSYANSCLNPIIYALLARP
FQRGLRRVLCRTSVRVADGTLKRGDDEVQEELSRVNGISQEGRSVRTDGGEGNNVGTSAQ
KSLPEDLGACEKESMLRISYL*
>jgi|Xentr4|327802|e_gw1.88.12.1
MSQAWNSSVSSELPPGGIIVPVVFSLIFALGTVGNGLVLAVLLRNGQTKYNTTNLFILNLGV
ADLCFILLCVPFQATIYTLDGWLFGAFLCKAVHFSIYLSMYASSFTLAAVSVDRYLAIRYPL
KSRDLRTSRNALAAIGVIWALSILFAGPYLSYYHIILYNQVPICIPNWEDRRRKIMDVSTFVF
GYLLPVLILGLSYARTICFLWTSVDPVKTASESRKAKHKVTKMIIIVAVLFCICWLPHHLVIL

CFWFGHFPFNKATYAFRLVSHCMSYANSCLNPIVYALISKHFRKRFKQVFTCLLTQKRARN
KVHAVQAANTIAGFYAGHTEVTQVQEDSGRGGRAAREEGADSGSPEDSVNQRMEGGSW
CTRLPKSVGFVTQTA*
>jgi|Xentr4|328141|e_gw1.91.201.1
MGNNHTCVYTFILLGLSSYGQYEIPMATLFFSISFLTLSGNLIIIETIRRNRNLHIPMYFFLSH
LSFLDMTSSFIIIPKMLANYLSSTQIITFYECLTQLHLFITFLATECFLLTAMAYDRYLATCNP
LHYSALMNYTNCVRMVAASWFLGILYATVHIILTSRLDFCYSLEIKHYFCDLPPLLYISCTNI
LPNIVAIFLGGILIGIGSFSFTMFSYIKIVSNILKIKSVKGRFKAFSTCASHITVFTLFISTLIAVY
FQSLSTNSLNSNRLVSLVYTVLTPLLNPLIYSLRNTDIKRAMSSMILQKRVQKTNKFHTRPL
NKKILCCVLQFG*
>jgi|Xentr4|328142|e_gw1.91.192.1
MQLSGLSAYRNTTSSWSHFTFLAFSSVSGTQNVLFVIFLSIYLVALLGNLTIIFVITSEARLQS
PMYFLLGHLSFLDLCYISVTVPEILSNFMGRRQIITYSGCVVQLLFFISMEGTEALILAVMA
YDRYVAICYPLRYSVIMKRGVCFLLVAASWLGGLLNSLVHTILTFSLSFCDTNLKHFFCDIP
PLLQASCTQTHVNELVLFVVGGIWVGFSPFIFIIISYTFIICTVIKIPSTEGRHKAFSTCASHLTI
VSLFYGTAIFTYIKPSATYSLQTGSLVSLFYSVATPMFNPIIYSLRNQEIKKVIGKILKTNFLG
GI*
>jgi|Xentr4|328144|e_gw1.91.184.1
MPWINWTNEVDFTLFGLSSDLSVQYFQFGIFLLLYLMSLLGNLTITIAIWVDQKLHTPMYF
FLGNLSFLDICYSSVTVPKMLVALIGGNNKISYRGCMSQLYFYVSCCSTECLLLSVMAFDR
YVAICNPMHYTVIMSKKTCFQLAAGLWLIGFTSSIVHTVFTARLPYCGSHSISHFFCDIPPM
LQLSCADTFMNNVLILTAGGFLGLSSFVLTLISYIKIISTILNIQTKVGRGRAFSTCASHLTVV
TIFFGSISFMYMRPTSSYSFETDKLISLLYAVLTPALNPIIYSLRNKEFKASLKKTLIKHIFSKV
IVKWTPAGPISSHDLF*
>jgi|Xentr4|328208|e_gw1.91.58.1
MLAAKRNGTEITTFTLLGLSSDPQPFLFAIFLLIYVCTVVGNTVIILTVTFESRLHSPMYIFLR
SLSLTEISYISVTVPRMLRDFLHKDKDISLLGCATQLYFFCFLGTTECFILAVMAYDRYVAIC
NPLHYMIIITKPKCLKLSLGSWLAGMLLSLGQISFVFSLPFCDSNIIDHFFCDILPVVRLACA
DTSANMITILIYSSFVIQLPFLLILVSYIHIVATVFRIPTAHGRNKAFSTCGSHLISVCLFYGTA
TITYLRTNGSHKSSKMWSLLYIVLIPMLNPLIYSFRNTEFKTALKRLIKKGKC*
>jgi|Xentr4|328213|e_gw1.91.202.1
MKNKSYVSEFILVGFSVNWIGTMSLFILFLLIYTLILSGNLLVILLIWKHKQLHAPMYLFLA
VFSFMEILMANVVFPESLKNFFSSKKNISFGRCFTQSVFYFLIGTSDFILLSVMSYDRYVAIC
NPLHYSTIMTWKRSICLVIASWLGGLLIIIVPALLKFQLPYCGPNIINHYFCDAVPLVKLACA
DTTHIQLIDFFLFSLVILGSLILVFYTYTCILIVVLRIPTTSGRKTAFSTCSSHFFIMSLYYVSSI
FIYVTPTQSKSLEINKMMSVVTTFITPVMSPFIWTLRNQQIKAILLESMKEL*
>jgi|Xentr4|328239|e_gw1.91.282.1
MNSGNCSAVSEFLILGFYSLQRFRLPLFLLLLLIYIVTFLENLLIIVLVTISANLQIPMYFILKC
ISLCELLYVTNLVPKILHDILSDQPAISVTACIIQLHMFGTTGNTMSFLYALMSYDRYVAIAN
PLRYTSLISKTLCLYTVLGFCVVSFTLACVIAGFLYKLEFCNQNIINHIYCDFSPVVNLSTSD
TRLLQMITSLFSIVIMLFPLVFTILAYVFIMQAILRMPSAIGRKKAFSTCSSHLFVVIIHIGILFS
VYVIPTKESNNNKGFSFFYIVVTPLINPIIYTLRNKEIHTSFVKAIKKVKY*
>jgi|Xentr4|328264|e_gw1.91.347.1
MNYCVNSFGNDTLLSSQASRISGTIFLALAASLGLPGNAFIIWSILWKMKGREKSVTCILIL

NLAVADGTVLLLSPFFITFLVLKTWIFGRAVCKLAYYLCCLNMYASIFIIALMSLDRFLAVFR
PYMAQSLRKKDMVTKILIAIWLLAGALALPAFAYREVIESQKLNAKICEPCHASRGESIFH
YTFETLVAFLLPFPMVLFSYILVLVKLKASRFGQRSRIEKLIAAILVTFAILWLPYHVVNAIQ
VTSNLTTGKVSENLKKAAKISRAGATALAFFSACVNPLLYAFAASDLFRIFGVGFVAKLLE
GTVAEIQKRVKSQRDLVKGLVRVGSRGESVDLEVRNGLKDTPLNGNQIPE*
>jgi|Xentr4|328286|e_gw1.91.203.1
MSSNLTEFILLGLPYSPRIQTCIYAVFQCVYILSLFGNTIIIATTCTYRQLQTPMYYLLTSLAL
LDIFFISCTVPKLLSILVGGNQVISLSGCLLQFYMYISLGGTEFFLLAVMSLDRYLAICHPLR
YSSIMTSNNCWRLAVGPWAFGFLEGIPFVFLISQLQFCSRTLAINHFFCDASALLHLSCSQT
QVARNIIFGFASLTILSSFIVTVSSYFFILLSIYGISSMSGRRKMFSTCSSHFVVVSISYGSCIFL
YVRSGESGHSNTEKAVSIFNSIFIPFLNPYIYTLRNQTIKNILKELWRMNIRLCVKDW*
>jgi|Xentr4|328290|e_gw1.91.62.1
MQNINNSQVTFFTLLQLWGSPVGQMSFSVSFSVIYVLTLSGNLIIIIVCRVEKSLCTPMYFFL
SHLAALEICYISVTVPKMIHYSVSGDVTISFAACLTQLYFFGSLGSTECFLLAVMAYDRYLA
LCKPLHYHSLMTSQFCSRSVGCCWSFGFIFTMSSVLLISSLKFCGSNHIQHFFCDISPILRLS
CTDIRHTETLIFLLASFTLVGSCLITLLSYLKILSTVLGISTQSGRGRASATFASHLTVVTIYYS
TMIFVYVRPTTANASSLNQILSMLYTILTPLLNPFIYALRNKEVQRALWKLKSRLKL*
>jgi|Xentr4|328302|e_gw1.91.41.1
MAILGVTTMMGIFTNSIIVVVNIVDKVKGKSFNPSDLILVTLGLSNITFQFTMTANDFLIILW
SDLYFSSAIYATFKVLLFSTIFASFWFTVCLCVYYCLQIVIFTHPILVRLKLALSRLVPYFLAA
SVFISVVISTPGIWSTNSDPPISNLTSNQSLEIEVPKLSLVYLFSSNIIGCSLPLVLVGISNCLIL
KSLIRKRAMLEKNKSDAHSPRTEARERAARTAGCLLLLYMTFYISEIFMFVDFFPPSSPGFC
TCLMIIYGYPPTQSVILIFGSPKLKKALLNLLRLPKKCNESKKTTKILFINF*
>jgi|Xentr4|328304|e_gw1.91.33.1
MLSKNLTEFILRGLPYSPLIQTCIYVVFQCVYILSLLGNTIIIATTCTYRQLQTPMYYLLTSLA
FLDIFFISSTVPKLLSILMGGSQVISLYGCLLQFYVYMSLGATEFFLLAAMSLDRYLAICHPL
RYSAIMTSDNCRRLTVGSWAFGFLDSIPLVILISQFQFCKRPAVINHFFCDASALLHLSCSDT
RVARNILFGLAAPTILSSFIVTVSSYFFILLSIYGISSPHGRRKLFSTCSSHFVVVSIIYGTCIFL
YVRSGESGPSSTGKVVSIFNSIFIPCLNPYIYTLRNEILMNILKGLWRKNVRL*
>jgi|Xentr4|328361|e_gw1.91.195.1
EKRVLLFMLLLSIYLLTFSSNMLIMVITKFDTILQNPMFFLLGNLSVLDICYISATVPNMLVI
LASQQNTISFVACVVQLFSVIFLESAECFLLAIMAYDRYAAICKPLHYILIMSREFCYSLLSM
VIIAAVLHSLLHTLLIVHLPFCDNKINHLFCDIPPLLKLSCTDTSLNEIILFAVSGVFVGLGPL
LFIFLSYIYIACTIFKITSKAGLRKAFSTGASHLIIVFIFYGSGSFTYIRPKASYSLERDKLLSLF
YNIVTPLANPIVYSLRNTEVQTALRKLFRTCQR*
>jgi|Xentr4|328378|e_gw1.91.174.1
MRNETERTEILLLGFETSTKLRFLLFIIFLSFYVLTITTNMFIIGIIRIDLNLQRKPMYIFLSHFS
FLEIWYTTVTLPKMLVDFIEDNHTLSYNGCLTQIYFFFALGLTELSFLAVMSYDRYFAVCHP
LRYNAIMTNNLCNKMAICSWCCGFLFSFILVIPSSRLLFCGPNTINHFFCDFIPLLHISCNKTL
TTDRLFYTLAWIIILFSFSVTIASYIFIIKTICSVLSRAGRRNAYSTCASHLTVVLTFYITVIFM
YIRPSAQYSFSVDKVVSLFYAVVVPLLNPLVYTLRNAEVQEALRKMLVKKIAFF*
>jgi|Xentr4|328395|e_gw1.91.21.1
MLFTNGTHANYFIILGLLNTTEMNMPLFIVFLCIFLVTLMGNIIIITVVCLDRALQNPMYFFL
TNLSFLEIWYTVTIVPKLLVNLLVKCIYISFVGCMTQLFFFVTFGACECYLLLVMAYDRYL

AICKPLYYSTLMNTKTCLYLVSGSWIISVFTGLITVTLISQLEFCGPREINHFFCDIPPLLQLS
CGEIYNTEISIFILSLVVLFFSLLLTLVSYLFIVVSVLSIPSSNGRSRTFSTCGAHLTVVLIYYGT
MIFMYVRPHSGHVSEMTKFVSVFYTVVTPGLNPIIYSLRNKEVKTSIKKITLRLFSPCRSCL
LTLKMQKCISLHMEPFHLF*
>jgi|Xentr4|328408|e_gw1.91.365.1
MAILGATTILGIATNLVIVVVNLVDRVKGKSLNPSDLILVTLGLSNMTFQFSMTANDFLSIL
WSDLYFSDAVYTTFITLLLFPIFSSFWFTVCLCVYYCLQIVIFTHPFLVQLKLKISQLVPFLLA
TSVFISVVISIPGIWSTYRDPPISNISNNQSLEMELPKLSFTYLFYSNIIGCSLPLVFVGISNCLI
LKSLISKSTMFEKNKSDVYSPRTEARERAARTVGCLLLLYMAFYISEIFMFVDFFPPGSPGF
CTCLMVIYSYPPTQSVILIFGSPKLKKALLNLLHFSKICGMEQIETPKILSVSF*
>jgi|Xentr4|328411|e_gw1.91.357.1
MENRNDTSWVTEFTLLGLTNDPMVELILFVFFLVVYIVTLLGNSLMIITITLSSTLQSPMYF
FLRSLSMVDISFTSSTVPKLLLDFLSEVKRISFTGCVAQLYLFISFGGIECVLLAAMAGDRYV
AICMPLRYKEVMSWKVCVVLTVACWIIGLLNSLVHTVFTFHLPFCKSEAINHFFCDIPALLS
LSCADTRANELVVYIAGGSVILGSFVITLLSYIFIVLTIIQIKTSSGRLKAFSTCASHLTVVTLF
FGTIVFTYIRPTSTYSLDQDRVVPVLYGIIIPMLNPIIYSFRNKEVQGAIRKAILRKLRLTILNN
NLAKSKNNT*
>jgi|Xentr4|328429|e_gw1.91.69.1
MTSNESTNEKAVAHFILLGFQSSPAIRSCLLLFFIVAYIFIILQNVAVIVIVQWDSKLHTPMYF
FLTKLACLESLYVSVTIPKILDGLVTSNNTISVGGCLLQLYFFLSLACTECFLLAAMAFDRY
LAICTPLRYHSIMTNKMCWSLTWASLFLGFLSCSFSVGLISKLNFCGPNVINHYVCDISPVI
NLSCEDISAVELVDFITALIVLLSSSVPITVSYISIISTVRKIPSGRGWKKAFSTCASHLAVVIIF
FGTTIFMYARPMAIQSFNVNKFLSVLYSVIIPVINPMIYTLRNNDIKRSMKNIFGTSLLLLTK*
>jgi|Xentr4|328437|e_gw1.91.196.1
SVGNQTSPMEFILTDFSEEPILQILSFVLFLFTYLVATVGNAMIILIIIHVPNLRTPMYFFILNL
GILDVCYISTTVPNMLGNSLKQWKRISFGGCVVQMYIFLSMAATESFLLVAMSYDRYVAIC
NPLRYTVIMNRRLCLQLAATSWMIGFTYSTIHTANTFTLKFCRSNVIDHFFCDIPPLLKISCS
NTKLNEILIFVVGAVLILPCFPLIVISYVHIVRTVMRIPSAKGRKKTFSTCVSHLMAVCLFYL
TGTSVYLRPASANSAFQQDKISAVFYTIFVPMINPIIYSLRNKELKGAIKTVVTKTFLYKMQ
*
>jgi|Xentr4|328448|e_gw1.91.191.1
LQAMQQNNYTAVYEFLILGFNRLHGFRIILFLLFLLIYIVICLENLLIIVLVTISARLQTPMYFI
LKCMSVCELCFATSVVPKMLHDILAERATISVMGCIIQLNIWGTMGMLIYLLYALMSYDRY
LAISNPLRYSVLIDNKLCLRLVIGFCIIFFLVAVLISVLLSRLEFCNRIIPVIDHFYCDFAPLMA
LSCSDTKLVQGVAALSSGIIMLLSLVFILFSYVFIIFKILAIPSATGRKKSFSTCSSHLFVVIINY
GILINVYVSPDTINSPQINKGLSFMYILVTPLVNPIIYTLKNHEFNIALQAKAREIKMHFFL*
>jgi|Xentr4|328455|e_gw1.91.35.1
MLSNNLTEFILLGIPCSPRIQTCIYAVFQCVYILSLFGNIIIIATTCTYRQLQTPMYFFLTSLAFL
DIIFISSTVPKLLNILVGGSQVISLPGCLLQFYVYMSLGATEFFLLAAMSLDRYLAICHPLRY
SAIMTSDNCRRLTVGSWAFGFLDSIPLMILISQFQFCKRPAVINHFFCDASALLHLSCSDTRV
ARNILFGLATPTILSSLIVTVSSYFFILLSIYGNSSPLGRKKMFSTCSSHFVVVLITYGSCIFLY
VRSSESGPSSAVKAVSIFNSIFIPFLNPYIYTLRNQMIVNILKRLWRKNVRLCAKD*
>jgi|Xentr4|328494|e_gw1.91.219.1
MDNRTEEDVFILNAFSDQGRLRILLFLIFLIIYLATVVGNILIILLSKLDCNLHTPMYFFLSNL

SFMDICYTNTTIPKMLLIILLKHSAISPAACAAQLFCFLSLAASESSLLAFMAYDRYVAICNP
LRYTSIMNKAVCLQMAAGTWIIGCIYGAIHTVTTFRLHFCGSKVLDHYFCDIPPLLKISCND
TFFVEATVFVVGGFLMLGCFILILGSYVRILFSVLKIPKANRRGRTFSICVSHLVVILLYYGS
ASIMYFRPKSKLVADKTWLLSIFYANITPLLNPVIYSLRNKDIRRALLKLIKSLQKHN*
>jgi|Xentr4|328158|e_gw1.91.163.1
MCPLVCNLLIIIAVFNDLTLHSPMYLFISVFSFISICYTSVIFPGLLHDLCSEDKRISLSFCVASF
CLYISHVCNIRHTFVCASILVTTRYRYLAICNPLRYTSIMNGQVCIMAASSCWLLAFFSVFFL
VFLISQLNFCQPTIINHFFCDIPPLLRLSCTETFLEDIMVFFFACSIILTSLFLTVVSYILIISTIYK
IPSNKGRKKAFSTCASHFSVVSILYGTVIFIYVRPNVSNSVDVNKVLGIFNTVVTPLLNPVIY
CLRNKEVKMALRKVMKKKSELGITKICNFR*
>jgi|Xentr4|330845|e_gw1.102.78.1
MFFFAGNLFVVSLSVADLVVAVYPYPVILIAIFQNGWTLGNIHCQISGFLMGLSVIGSVFNIT
AIAINRYCYICHSLRYDKLFNQKSTWCYLGLTWILTIIAIVPNFFVGSLQYDPRIFSCTFAQT
VSSSYTITVVVVHFIVPLSVVTFCYLRIWVLVIQVKHRVRHDFKQKLTPTDLRNFLTMFVV
FVLFAVCWAPLNFIGLAVAINPLHVAPKIPEWLFVVSYFMAYFNSCLNAVIYGLLNQNFRKE
YKRILMSLLTPRLLFLDTSRGGTEGMKSKPSPAVTNNNQADMYV*
>jgi|Xentr4|332501|e_gw1.110.119.1
MNLSSYFENQSVPNNISGNITFPMSEDCALPMPIVFTLALAYGAVIILGLSGNLALIIIILKQK
EMRNVTNILIVNLSFSDLLATIMCLPFTLIYTLMDHWIFGEVMCKLNEYIQCVSVTVSIFSL
VLIAIERHQLIINPRGWRPNNRHACFGITVIWGVAMACSSPLMMYSVLTDEPFRNISLDSYI
GKYVCLEDFPEDNFRLSYTTLLFILQYLGPLCFIFVCYTKIFLRLKRRNNMMDKMRDNKY
RSSETKRINIMLLSIVVGFALCWLPFFIFNLVFDWNHEAIATCNHNLLFLICHLTAMISTCVN
PIFYGFLNKNFQRDLQFFFNFCDFRSREDDYETIAMSTMHTDVSKTSLKQASPIA*
>jgi|Xentr4|332511|e_gw1.110.122.1
QDISCPLGYFPCGNITKCLPQFMHCNGVDECGNQADEDNCGDNNGWSQQLDKLFEKHY
EKIGLNVFDLEMKPSECTLGPVPTQCLCRGLELDCDGAKLRTVPSVSPNVTIMSLQNNML
RKLGPDEFRIFPDLRKLYLQHNNIRTVSVHAFKGLYNLTKLYLSHNEITTLKPGVFEDLHRL
EWLIIENNKITRIYPQTFQGLNSLILLVMMNNFLERLPDKSLCQHMPKLNWLDFEGNNVQS
LTNTTFISCATLTLIYINYSRLSEVNTQLLPVQGSSILYFTNNRIEAFSPSLMKGVKELSQLNI
SHNPIQKIQADQFDYVIKLKSLSLEGIEIPNIQRRMFMPLKNLTHIYFKKFQYCGFAPHVRN
CKPNTDGISSLENLLASIVQRVFVWVVSVITCFGNIFVICTRPYIRSENKLHAMSIISLCCAD
CLMGVYLFVIGYFDLKYRGEYNENAQAWMDSTQCRLVGSLAILSTEVSVLLLTYLTLEKY
ICIVYPFRCLKPGKCRTITTLILIWIIGFVIAFIPLSNQTFFHNYYGTNGVCFPLHSEQPESTAA
QIYSVIIFLGVNLAAFIIIVFSYSSMFYSIHRTAIMATEIHNHIKKEMTLAKRFFFIVFTDALC
WIPIFILKLLSLLQVEIPGSISSWVVIFILPINSALNPILYTITTRPFKEMIGQIWSNYKQRRSIG
NRNSHKACAPSFFWVEMWPLQEMSPEIMKPGLYTDSEMSVTTHSTRLSYT*
>jgi|Xentr4|332737|e_gw1.111.174.1
MPTNLTTGEVIGIFAPLGEGVLWPNSTQRNVSSPVAHKFVQPPWRIALWSLAYGSMVAVAV
FGNIIVIWIILAHKRMRTVTNYFLVNLAFSDASMAAFNTLVNFIYALHNEWYFGEAYCRFH
NFFPITSIFASIYSMSAIAVDRYMAIIDPLKPRLSATSTKVVIGSIWIFAILLAFPQCLYSKIRVT
RTRTLCMLVWPGKDERLTYQFILVLLVYVLPLIVMGVTYTIVGITLWGGEIPGDTSDKYHE
QLRAKRKVVVKMMIVVVVTFAICWLPYHIFFLADALDIKLDRWKYIQQIYLAIFWLAMSS
TMYNPIIYCCLNKRFRAGFKRAFRWCPFIEVSSYDELELKSTRFHPTRQSSLYTVSRMESS
MTVVFDPNDVENNKPSHSHKKRAATSESTFNGCSRRNSKSASTNSSFISSPYTSPDDYS*

>jgi|Xentr4|333086|e_gw1.113.38.1
GAHLKNMTIKTFHIFDSDSESEFEEIPWDELDQSGEGSGDEPLLSRSARKPSRRNITKEAEQ
YLSSQWLTKFVPSLYTVVFIVGLPLNLLAVIIFLFKMKVRKPAVVYMLNLAIADVFFVSVLP
FKIAYHLSGNDWLFGPGMCRIVTATFYCNMYCSVLLIASISADRFLAVVYPMHSLSWRTM
SRACMACAFIWVVSVASTMPLLLTEQTQKIPGLDITTCHDVLDFDLKKFNFYYFSSFCLLF
FFLPFIITTVCYIGIIRSLSSSSLENSCKKSRALFLAVVVLCVFIVCFGPTNVIFLTHYLLESNE
SLYFAYILSACIGSISCCLDPLIYYYASSQCQRYLYSLLCCRKVTEPGSSSGQLMSTAMKNH
TGSINAKSSIYRKLLV*
>jgi|Xentr4|334485|e_gw1.122.25.1
MDNLSLFSCSNDSTGITRLYQPSPAAGCNSSVYGSDQTPPLLVDAWLVPLFFALIMLVGLV
GNSLVIYVISKHRQMRTVTNFYIANLATTDITFLVCCVPFTATLYPLPSWVFGDFMCRCVN
YLQQVTAQATCITLTAMSVDRCYVTVYPLQSLRHRTPRMAMAVSLGIWIGSFVLSIPFAMY
QKLSTGYWYGPQTYCIEAFPTVLHQKAFILYTFLAVYLLPLLTICICYAFMLKRMGRPVVE
PIDNNYQVQLLAERSEAMRTKISRMVVVIVLLFTICWGPVQLFILFQAFDSSFEKSYETYK
LKIFAHCMSYSNSSINPIVYAFMGANFRKSFKKAFPFIFKQRVEGTAGVVANTEMHFVSSG
T*
>jgi|Xentr4|336213|e_gw1.130.180.1
MDLHDNQNFVNFTKSSLIEINHDRRFYRSDMLATGVNTSEILNWTMASENLTHLTTEGSK
FSLEKNWPALLTVTVILVTIAGNILVIMAVSLEKKLQNATNYFLMSLAIADMLLGFLVMPVS
MLNILNDYEWPLHRKLCPVWIYLDVLFSTASIMHLCAISLDRYIAIRNPIHHSRFNSRTKAL
AKIIAVWTISVGISMPVPVFGLQDDSKVFDQENCSLSDENFIIIGSFVAFFIPLIIMVITYFLTIK
SLQKEATLCINNLGAKSKLALFSFLPQSSISSEKLFQRSLQKELAPYGRKTMQSISNEQKAS
KVLGIVFFLFVIMWCPFFITNVMAVFCKESCNEHVIGELLKVFVWIGYLSSAVNPLVYTLF
NQTYRSAFSRYIQCRYREEKKPLQLILVNTIPALAYNSSQLQLAQMKSLKKEGKMKTNDY
SLVTIGLQPLDRASKTGVAPLNENVSCL*
>jgi|Xentr4|338721|e_gw1.145.5.1
MGSLDNTPEFIWGNNTLNGTSADSPGIAIFISFVYSLVCIVGLCGNSVVIYVILRYAKMKTA
TNIYILNLAIADELLMLSVPFLVTSTLLRHWPFGSLLCRLVLSLDAMNMFTSVYCLAVLSL
DRYVAVVHPISAARYRRPSVAKMVNLGVWLFSILIILPIVVFSSTAPNSDGTVACNVLMPEP
SQRWVVVFVLYTFLMGFLLPMAAICLCYILIITKMRVVALKAGWQQRRRSERKLTLMVTV
VVTVFVVCWMPFYVVQLVGVFARKGDTTVSQLSVALGYANSCANPFLYGFLSDNFKRSF
QKVLCLSWMENANEEPVDYYATALKSRAYSAEELQNGMLTTGGIYSNGTCTSRTH*
>jgi|Xentr4|338776|e_gw1.145.144.1
MEPNSTSLNNSNGTVPLKAHSLWEVITIATVSAIVSLITIVGNILVMVSFKVNSQLKTVNNY
YLLSLACADLIIGIFSMNLYTSYILIGHWSLGSLACDLWLALDYVASNASVMNLLVISFDRY
FSITRPLTYRAKRTPKRAGLMIGLAWFISFILWAPAILCWQYFVGGRTVPPEECQIQFLYEPII
TFGTAIAAFYLPVSVMTILYCRIYKETEKRTKDLAELQGSESVADFEMMRPQGTLFKSCFS
CKQQTRTKRERCQASWSSSSRSTTTTVKITHSPNTCNEWIKEDQLTTCSSYPSSDDEDKQT
KEAVFQGAYENQTEAQKEEETKQILTKEQPGLSNYESERFFLTPGKGHSQKSPKCVSYKFR
IVVNDDGSQEVNNGCRKVKITPCSSMSKGHSLRSMDPSISHQITKRKRMVLIKERKAAQT
LSAILLAFIITWTPYNIMVLVSTFCSDCIPSSLWHLGYWLCYVNSTVNPICYALCNKTFRKT
FKMLLLCRWRKKTVEEKLYWYGQHPPCHNKLP*
>jgi|Xentr4|338796|e_gw1.145.69.1
MEHNGSWVFANRSELGSFQQAVSRALGIRNVSYSQLLWSNRSAASDGLTYRHFTTSAQVL

IFVGSLLGNFMVLWSTCKTSVLKSVTNRLMKNLACSGICASLVCVPFDIVLSASPHCCWW
IYTLLFCKIIKFLHKVFCSVTILSFAAIALDRYYSVLYPLERKISDSKARDLLIYIWAHAVVAS
VPVFAVTNVTDIYAMSTCSPSWGHSLGHLVYVLVYNVTTVFIPVVVVFLFMLLIRRALSAS
QKKKVIIAALRTPQNTISIPYVSQKEAELHAMLLSMVVVFIICSVPYVTLVIYRTMLNSSHIS
DFLLLTAIWLPKVSLVTNPLLFLTVNKSVRKCVVGTIVQLHRRYSRRNVVNLGSVAEVNLE
PSVRSGSQLLEMFHIGQQQIFKSVEEDDDNEIKLEASRNYKIKEALPSTSVEVEHTLVQKFI
PQSTDSASQVAPVMPGESEVLNDKYSMQFGFGPFELPPQWLSDKRNSKKRLLPPLGNTPE
ELIQTKQPKCRPERRISRNNKVCIFPKVDS*
>jgi|Xentr4|337830|e_gw1.139.7.1
MDKAGGNFSTTNSSLAKPPLYAGGLQPMDSAEWSLVIPTFLVAICLTGIAGNLCVIAVLLHN
ARRAKPSLIHSLILNLCVSDLLLLTLSVPFKVAAYTRTSLSFGWLVCKTADWFTHACMSSK
SMTIASVAKACFMYASNPAKQVNIKQQTVCAVLLSTWLLSALLPLPEWLFTSSKQVDGSP
ACIMDIPPHAQEMMAIFVKFYPFIVYCVPFTLASFYFWRAYGQCRRRGTKTQNLRNQIRSR
RLTIMLMSVTITFAIMWLPEWVSWLWLWHQSPSGPSPPQAFKVLAQILMFSLSSINPLIFLV
MSDEFKESFKDVWKHLASRKSMVAHGIQDKAAGHCDIAPESPPSPQPNPSAVEEQSCSQQ
NFGSQESKDNQVLPDVEQFWHERETHLTDQDNDPVPWEHQEEQPVGSGNPSSAN*
>jgi|Xentr4|339043|e_gw1.147.27.1
RFMLTKITIIPNGAFAGFQDLEKIEISQNDVLTSIEANVFSNSPQLHEIRIEKANSLVYIDQEA
FQNLPKLKYLLISNTGIHFIPVVSKIQSLQMVLLEIQDNINIKKIERNSFAGLSIDNVILRLNK
NGIEEIQNYAFNGTNLNELILSGNERLARLPDLVFHGSTGPTVLDISNTRISSLPSFGLEKIKR
LKAKSAYNLKKLPALEKFEELIEADLTYPSHCCAFANWKREITDWDPICNKTYESQPEDLK
SLNKRSAQVDPILSNGETTYDLIDKEDDIAFDYFFCYGKRDFVCSPTPDEFNPCEDIMGYT
ALRVLIWCVNILAITGNSVVLIILLTSQYKFTVPRFLMCNLAFADLCMGVYLLLIAAVDIKT
KSQYYNYAIDWQTGAGCHAAGFFTVFASELSVFTLTVITLERWHTITYAMQLDRKVRFRH
ATMIMASGWVFSFTVALLPIVGVSSYMKVSICLPMDIENPLSQAYIMFLLVLNVLAFVVICT
CYIGIYLTVRNPDVISSNSDTKIAKRMAILIFTDFLCMAPISFFAISASLKIPLITVSKSKILLVL
FYPINSFANPFLYAIFTKTFRRDFYILMSKFGCCEARAQIFRTETSSSAHNLHARNGHFVPGP
KSSSGSVYTLVPLNH*
>jgi|Xentr4|339063|e_gw1.147.54.1
ISESNSLERIQSYAFSNLSSLTELTIQNTKNLIHIGEGAFGNLPTLKYLSIGNTGIRKFPDVTKI
FSSQLQFILEIYDNLHITTIPANAFQEMNNEPLILKLYRNGFEDIQSYAFNGTKLDQLFLNNN
NNLQYIHNDALKGASGPNRLDITSTALEYLPSYGLEFIQVLVAEYSYYLKQLPALDKLDSLI
DARLTYPSHCCAFRNMRLKEQNALLENLTKQCDSTLHKQFNGTKYNYPLLGAEQSYWAY
LMYLCQPKILKCSPEPDAFNPCEDIMGYDFLRVLIWFISILAIAGNAVVLFILLTSHCKLTVP
RFLMCNLSFADFCMGIYLLLIASVDSQTKSKYYNHAIDWQTGSGCSAAGFFTVFASELSV
YTLTVITLERWHTITYAMQLDRKLRLRHAILIMLGGWIFSLVIAMLPLVGVSNYIKVSICLP
MDIETPLSQAYIIFILVLNVIAFIIICACYIKIYIAVQNPELAPTNKDTKLAKKMAVLIFTDFTC
MAPISFFAISAAFKVPLITVTNSKILLVLFYPVNSCANPFLYAIFTKAFRRDFFLLMSRFGCC
KTQAELYRVNNFTAYTSNLKMDSLYTGPKKTSRAEFHVMAPDCQQLNDHEKPASTGS*
>jgi|Xentr4|339180|e_gw1.148.115.1
MNTTNVFSVQAVLANATLDPNETLFLSNLSRIGFCEQVLIKTEVFLTLGIISLLENILVILAIL
KNKNLHSPMYFFLCSLAVADMLVSVSNALETIVIAIQNKYLVIGDYLLQHLDDVFDSMICIS
LVASICNLLVIAIDRYITIFYALRYHSIMTVKKAIALIVVIWTSCIICGIVFIVFSESKTVIVCLIT
MFFTMLVLMATMYVHMFLFARLHVKRIAALPVDGVVQQRTCMKGAITITILLGVFVVCW

APFFLHLILIISCPSNSYCVCYTSYFNTYLILIMCNSIIDPLIYAFRSLEMRKTFKEIICCYGMN
FGKCG*
>jgi|Xentr4|339233|e_gw1.149.178.1
MNNHSMVTEIFLLGFQHLNNFKILIFTSILLTHILTIYGNALVIALVTISRDLQSPMFFFLRQL
SLSDLLGSIIIVPTLLRTVINEGAKISLIGCFAQLYLFAITESLQSFLLTVMSYDRYLAICNPLH
YSSIMNHAFSVKLIGVLWLLAFSATPVTVISAATQEFCNQNTINHFYCDYFPLLELACSDTF
MARVLTITLLVPVVLSPFMLIVISYICIAHEILKIVSKIGRQKAFSTCSSHLAVVSMFYGTIIGI
YLIPTRNQSQTISKLFSLLYTVVTPFANPIIYSWKSADMKNALKMLYNK*
>jgi|Xentr4|339362|e_gw1.149.145.1
MFPHQRYNSSLPSALSTLPPTSLSPSSPTPRPSSSVPLLPSLSMAFGVTSNALGLWILARAYT
YSRRQRRSRAQFLLLASGLLLTDLAGHLIAGSFVLWLYSHGGLPVAGCQFLGGCMVFFGL
SPLLIGLLMACERCLGLTRPLWHSQMVTQNRARLSLALAWAVALLVSILPFLGYGAYDLQP
SGTWCFLKGPTGFCLLFSGLGLGCLAAALVCNVLVGATLLRARLQRPPKEERRRRQSRSH
THDLEMVVQLLAITFVSCVSWTPLLVSNRCMGVFLNNQNIQGYCDTNIYSYALGFTEPDF
GPWVYILLRRSVLFRLYTIFVRATRLRNGKLSRWDAGEFQSSERSQVSHL*
>jgi|Xentr4|339481|e_gw1.150.252.1
MYIFLCVLSFLDICYASTTLPKLLVMLATQRKIISFVGCMTQLYFFLSFICTEFLLLATMAYD
RYAAICHPLHYTLLMSLKNCVKLLAASFAIGFLDPFVFALLIANLSFCASRLIDHFFCDVSP
VLKLTCSDTSILEILIYINGALVGSSAFILTSTSYIFIICTIMRIRSSHGQQKAFSTCASHLMCV
LIFYVTIICLDTRPIKAFNPKRDKFFSLLYMTLIPLLNPIIYTLKNNDFKESLKKLITKRCAYS
K*
>jgi|Xentr4|339482|e_gw1.150.244.1
MSSANVSGFIIQGFSDTPELQFSLFVLFLGIYLIILLGNLIIFLVISCNPHLHTPMYIFLLNLSLI
DISSTSNILPNLLHILLTQQNNISFLGCMTQMYVFVAFGASEYFLLTAMAYDRYVAICDPLH
YIARMSRKHCAGLITAAFTVGFGEAVSFIALIPKLSYCASHLINHFFL*CHTIAKTFLQQHF*
CGTFNLL*RDIAAF*FLTLTSYIFIISAILKIQSSEGRQKAFSTCASHLTCVITLYGTALSLYMR
PTTSYSLKRDKYFSLLYIALGPVLNPLIYTLKNKEFLSSLNKVKQKCLDFYKLKAVNKTSV
*
>jgi|Xentr4|339508|e_gw1.150.210.1
MASGSKTNASGFIIQGFSDTPELQISLFVLILVIYLIILLGNLIIFLVISCNPHLHTPMYIFLQNL
SLIDISSTSNILPNLLHILLTQQNNISFLGCMTQMYVFVALAASEFFLLTAMAYDRYVAICDP
LHYIARMSRKHCAGLITAAFTVGFGITFGFIILVSKLSYCASRLINHFFCDLSPLLKLSCSSTF
SVEVLIFVEGSMLIFNAFLLTLTSYIFIVSAILKIQSSEGRQKAFSTCASHLACVITLYGTVFC
LYMRPTTSYSLKRDKDKYFSLLYIALGPLLNPLIYTLKNREFQSSLIKLRQRDFFF*
>jgi|Xentr4|339575|e_gw1.150.211.1
MTLGNISGFIIQGFSDTPELQISLFLLLLGIYLIILLGNLIIFLVISCNPHLHTPMYIFLLNLSLID
ISFTSTVLPNLLHILLTQQNNISFLGCMTQMYVFASLAGNELFLLTAMAYDRYVAICDPLHY
IARMSRKHCAGLITAAFTVGIGNPVGIVVLISKLSYCASHLINHFFCDISPLLKLSCSSTFSVE
LLIYMEAILLTFNSFLLTLTSYIFIVSAILKIQSSEGRQKAFSTCASHLACVITLYGTVICLYMR
PTTSYNIKTDRFFSLLYTVLGPVLNPFIYTLKNREFLCSLSKVMQRFCQLMAVNKTSF*
>jgi|Xentr4|339614|e_gw1.150.238.1
MASENVSGFIIQGFSEIPELQISLFVLFLGIYLIILLGNLIIFLVISCNPHLHTPMYIFLQNLSLID
ISSTSNILPNLLHILLTQQNNISFLGCMTQVFVFVFLAASEFFLLTAMAYDRYVAICDPLHYI
ARMSRKHCAGLITAAFTGGFGGTVGFIVYIPKLSYCASHLINHFYCDIAALLQLSCSSTFSV

ELSIYIEGTLLNFSSFLLTLISYIFIISAILKIQSSEGRQKAFSTCASHLTCVITLYGTLLCLYMR
PKTSYSVKRDKYFSLLYIVLCPVLNPFIYTLKNREFLSFLNKVKQKCLYYKS*
>jgi|Xentr4|339638|e_gw1.150.220.1
MYIISGNQSKENPFTLQCFSDQQDLQIPLFVTFLLIFLIIMIGNSTIFTTITLIGQLHTPMYMFL
GSLSLLDISYTSTTLPKLLLMLCTQDKTISFAGCIIQLYFFIGFTCTEFLLLSVMAYDRYVAIC
RPLHYTLVMSLKHCVRLIIVVWAAGFLDTAPFALLIKNFTFCASHHIEHFFCDFTPVLKITCS
DTSTIELLTYMNGVFMAFSAFTLTSASYIVIICTVVTIQSSEGRKKAFSTCASHLICVTVFYG
TIICSYIRPVQSYTPKQDTYLALLYTVLVPMLNPFIYTMKNKEFKDGFGKLRARIALHLEQ*
>jgi|Xentr4|339677|e_gw1.150.247.1
MTSGNVSGFIIQGISDTPELHNFLIVLILGIYLIILLGNLIIFLVISCNPHLHTPMYIFLQNLSFI
DISFPSNVLLNLLHILHTQQNNISFLGCMTQMYVFVALADSEYFLLTAMAYDRYVAICDPL
HYIARMSRKRCAGLITAAFTGGFGGAVSFIVLISKLSYCASHLVNHFFCDVAALLKLSCSST
FSVELLIYIEGALLPFNSFLLTLTSYVFIISAILKIQSSEGRQKAFSTCASHLACVITLYGTALS
LYMRPTTSYSLKRDKYFSLLYIVLGPVLNPLIYTLKNREFQSSLNKVRQKCLYFFY*
>jgi|Xentr4|339678|e_gw1.150.239.1
MTMLALGNQSAVTRFTVQCFSDKQDLQTPLFTVFLLAFLIILIGNIIVIATITLSAQLHKPMY
MFLGSLSFLDISSTSTILPKLLAMLHTQDKTISFSGCMVQLYFFMAFACIEFILLSAMSYDRY
VAICYPLHYALRMSLKHCAKIILGVWAAGLLDPVIHTLLTANLSFCSSNHINHFFCDLTPLL
KLSCSDTSPIEMITYIYGVIVVISTFTITSVSYVFILYKILKIHSSQGQKKAFSTCTSHLTCVIIF
YGTIICLYMRPTKSISPNQDTFALLYVVLVPVLNPFIYTLKNKEFKDNLWKMKTRISSNLIS
VKCTMKSLSRLINCGCKA*
>jgi|Xentr4|339696|e_gw1.150.44.1
MTLENTSGFIIQGFSDTPELQISLFVLFLGIYLIILLGNLIIFLVISCNPHLHTPMYIFLQNLSLI
DISFPSTVLPNLLHILLTQQNNISFLGCMTQMYIFASLASNEYFLLTAMAYDRYVAICDPLH
YIARMSRKHCAGLITAAFTVGFGSTVSFVVLLSKLSYCASHLITHFFCDMAPLLQLSCSSTF
SVERLIYIEGTLLVFNAFPLTLISYIFIISAILKIQSSEGRQKAFSTCASHLACVITLYGTVICLY
MRPTTSYSLERDKYFSLLYIALGPVLNPPIYTLKNREFLSSLNKIKQKC*
>jgi|Xentr4|339758|e_gw1.150.219.1
MASENVSGFIIQLFSDTPEPQISLFVLFLGIYLIILLGNLIVFLVISCNPHLHTPMYIFLQNLSL
TDISFTSTVLPNLLHILLTQQNNISFLGCMTQMYVFVSLSGSEYFLLTAMAYDRYVAICDPL
HYIARMSRKHCAGLITAAFTGGFGGAVNFIVLIPKLSYCASHLINHFFCDITPLLKLSCSSTF
SVELSIYIEGILFTFSSFLLTLTSYIFIISAILKIQSSEGRQKAFSTCASHLACVITLYGTVICLY
MRPTTSYSTKRDKYFSLLYIVLGPVLNPLIYTLKNREFLSSLNKVKQKCLYYKS*
>jgi|Xentr4|339770|e_gw1.150.222.1
MASESPKNVSGFIIQGFSDTPELQISLFVLILGIYLIILLGNLIIFLVISCNPHLHTPMYIFLLNL
SLIDISSTSNILPNLLHILLTQQNNISFLGCMTQMYVFVAFAASEYFLLTAMAYDRYVAICDP
LHYIARMSRKHCAGLITAAFTGGFLDPVPYLVLISKLSYCASHLINHFFCDITPLLKLSCSST
FSVELLTYIIGTLLAFNAFLLTLTSYIFIISAILKIQSSEGRQKAFSTCASHLACVITLYGTGICL
YVRPTTSYSLERDKYFSLLYIALGPVLNPLIYTLKNREFQSSLIKLRQRGFFLLNHQCHLIK
H*
>jgi|Xentr4|339771|e_gw1.150.214.1
MASGSQKNASGFIIQGFSDTPELHVSLFVLFLGIYLIILLGNLIVFLVISCNPHLHTPMYIFLL
NLSLIDISSTSNILPNLLHILLTQQNNISFLGCMTQMYVFVALAGSEYFLLTAMAYDRYVAIC
DPLHYIARMSRKHCAGLITAVFTGGFGGAVSLIVLISKLSYCASRLINHFFCDLTPLLKLSCS

STFNMELLIYVEGSLLILNSFLLTLASYIFIISAILKIQSSEGRQKAFSTCASHLACVITLYGTV
FCLYMRPTTSYSLERDKYFSLLYIALGPVLNPLIYTLKNREFQSSLIKLRQRSFFILNRQCHL
MKH*
>jgi|Xentr4|339776|e_gw1.150.32.1
MKPQGIVGNQSEVTEFIVQCFSNAPELQPLLFVIFFLIYLLIVTGNTTICATVAADPHLQTPM
YWLLSNFSFLEMSYSSVILPKLLTMLVTQHKTISTTGCKFQLFFFTVFTCIEFFLLTVMAYD
RYVAVCHPLHYHSLMTLRHCAKYIAAVWTFGFLDPIPLNAIIAKFSFCSSNQIDHFYCDVTP
VLKLTCSDTFTLLLLVYMNGAFLFLSTITLTCISYILIIRTIQGIKSSEGRKKAFSTCASHLTCV
SIFYGTIFCLYMRPRAAYEPEKDKFFSLLNIVLVPSLNPFIYTLKNKEFKIAFAKQKKRIFSW
GQS*
>jgi|Xentr4|340244|e_gw1.153.5.1
MTFTDLTNDSVGNHTEPHIRGDNDTEDHANGTSLSLALDLPALSVGIVLAVFILFAIVGNIM
VILSVACNRQLQTVTNYFIVNLAIADLLLSTTVLPVAATLEVLGFWAFGRVFCDIWAAVDV
LCCTASIMSLCVISIDRYVGVKYSLKYPTIMTEKKAVVILILLWLSSMVISIGPLLGWKEPPP
PDDSKCSITEEPGYALFSSLFSFYLPLLVILVMYFRVYIVARRTTKSLEAGVKKERNKSMEV
VLRIHCRSVLEESSSSTRNHKGHTFRSSLSVRLLKFSREKKAAKTLAIVVGVFILCWLPFFL
ILPLGKSQVPPSC*
>jgi|Xentr4|341225|e_gw1.158.56.1
MAESWTFPGRDHALKIGIITALGCLIILGNSFVLLVIASSVSGWSSNSRYILISLTGTDVTLAL
VVVPLNLYGSLALEPGEEDDEESPISRYCHIVAFLNSSVFASSIYSLTTISLERYVAVFFPLHY
SRVMSRRRVKMLIAAAWLLPPIFLCPISIPGGRFIRVYFSRASLICNPDYASNVAYSLLLTAVI
FFPCSAIVTFANLRLWRVARRQSRRMSVGWAAKSKGLYAASRVLVPVVIVFYTCWAPCMI
TILYNGKNHCPSTITQDRVPEWVEFVALWLPCGNGFLNCFVYFWINKSFRHKFRQLGRKL
WPWGCARKNHQITPPTISATINCMNNNVVLQERSCSVSSSCMLLPQAGETVL*
>jgi|Xentr4|341373|e_gw1.159.121.1
MKVCALRLNSSQANVQEFILSSISSIYEVQLLLSISFLVIYIITLASNAVIISIIQLDAHLQTPM
YYFLTNLAFLDICYSSVTVPKLLDLQLTGRKTISFYGCIAQMYFFHVLGSSEMFLLVAMSY
DRYVAICNPLRYSVIMRRAVVMTLKVCSWAGGFIHSTFHTAFLLRLSFSQYNKVDNFFCDA
TPLIKLSCSDTTMDEIMLLATPGILGPICFILTLVSYSYIVRSILKINSAQGRYRTFSSCASHLT
VVTVFYGTGIFVYVQPMAVYWSANQFITVFYSIITPMLNPLIYTLRNKEIKKALGKILFKQS
PMHANIHLLH*
>jgi|Xentr4|341680|e_gw1.160.9.1
MDSAWANVSDSNGSSCEVHSNQRAAQTIFTVYYFIVFIIGSCGNILALHLTFKRGKKVNST
DIYLINLAVSDALFTLALPGRITYYILNFHWPFGDSLCRITAFIFYTNTYVGIYFMTCVSVDR
YIAVVHALRFSKLRKTCCVKYICLLGWCIVVLQTMPLLFRPMTKENKNTVTCMEYFNFED
VNHLPVLLLLACIFGYGTPFGIILFCYMCISMRLCKAAKNNPIADKNGQYNRAFNVIMIVLI
AIVLCFTPYHINIIQFMVRSLIRQQTCLDNKAFKMSLQITVAFMNMNCCVDPIIYFFAFKGY
KRRILGLFKMYVSGPSVTYSENQSTSQNQGGTVLVEL*
>jgi|Xentr4|341932|e_gw1.162.96.1
MFTEDTYHFLALIVATVGFLGLVNNLLVLILYCKFKRLQTPTNLLFFNTSLCHFVFSLLAITF
TFMSCVRGSWAFSVEMCVFHGFSKNLLGIVSFGTLTVVAYERYARVVYGKYVNSSWSKRS
ITFVWVYSLAWTGFPLIGWNLYTFETHKLDCSFEWTATDPKDTAFVLLFFLACITLPLSIMA
YCYGYILYEIQKLRSVKNIQNFQEITILDYEIKMAKMCLLMMLTFLIGWMPYTILSLLVTSG
YSKFITPTITVMPSLLAIASAAYNPVIHIFTIKKFRQCLVQLLFHNFWRLLKNLNGRLAMKK

VKPVLGKGRSHNRPEKKVFSSSDFFTRTTSDTGTHGITESTKGKRTNVRLIQVHPLYP*
>jgi|Xentr4|342326|e_gw1.165.64.1
MFQREYFHTVKILYTCGYAASLTALVTAISIFSLFRKLHCPRNFIHVNLFVSFILRGIAVFIKD
AVLFADEDVDHCTMSTVGCKAAVVFFQYSVLANFSWLLVEGMYLHTLLTLTFTYQRKYF
WWYIVIGWGAPALTISVWIKTRIQFDNTGCWDDYESIYWWIIKTPILLAIFINFIIFLNVIRILI
QKIRCPDISKNYKQQYMRLAKSTLLLIPLFGVHYVIFALFPEHIGIWARMYFELVLGSNQGF
IVALLYCFLNGEVQAEIQRHWGKWQSSLESNVFNLVTQDFTA*
>jgi|Xentr4|342715|e_gw1.167.123.1
MPGKAEYYSVKLFIYLPFLCMSLCISEINPSISACRLEMIEVEEKYEYIQDGDIMIGGVLTVS
KTETGPNNRTVYCDYPSPQKYRYLVEFIFHIKEINDNPARLPNMTLGYHIHDSCGNPKRAL
MSLFKILSGIGEPVPNYSCMGRKMVTAFIGDLDSEPTVAMAHILGLLGYSQISYGATDPALS
DRTTFPYFFRTVQSDEEEYIALSKLLKYFGWTWVGIIQFDDYSGYRDHQLLLRYLSREGIC
TEFTVKALFNNFFSLYQKTFLFLSKWLNHLDVTVHVKLLMSRSLTFTQHRLSGHHDAKFL
QFSDSFYPSNYPHDILLENMWIYSYQCLPKDKRNKRVNWQLVLYLYNCTEEGKLTDIPEY
LNAYHSASLIQAVDMMAMALQDMHNFHSKQTHGKGRWVGNYNYQLHRFLKNISYATDE
NSAVSSNDNGNISITYDIINPYYGEGKWSMNTVGYYKPLAPLDDQLVMIPQGIAWKTPNN
KVPRAQCSDSCFPGYRKVPKPGAQSCCYDCVPCPEGEISNTTDIENCIQCPEMEWADNKR
TKCIAKTEQFLSYTNDMISVIFSSITVLFFLITVLILRVFITYRDSPIVRANNRSLSFLLLVSIKL
SFLSVFLFLGRPVDITCMLRIITFGITFSIAVSSLLAKTIMVCVAFKATKPGSSWRKWLGVKL
SNSVVLFCSSIQIIICMTWLAISPPFQELDIHTSPGTIIIQCNEGSAIGFYSVIGYMGLLAAVSF
VLAFLARSLPDSFNEAKYITFSMLLFCSVWITMIPAYLSTKGKNTVCVEIFAILTSSAGLLAS
IFLPKCYVILFRPEINTKSNLLGNQKVAYKS*
>jgi|Xentr4|342911|e_gw1.167.125.1
MQPNSGPHVGQLLAKRSDLLLYISYICLSGGAVVEAMAFLELIYLTALYITTCSSKIQTKKP
ACHLEIIKTYEEYEYMKEGDIMIAGVFTVNTLIVPISYPMDFYNSMLCVGALAKNYRQFV
DFLFAIENINKDPVLLPNLTLGYHIYDSCGDPRKAVRSVFQILSGTREPVPNYSCVGKRNIA
GFIGDLTSETTVPIAQILNVYGYAQISYGATDPSLSDRHAFPYFFRMVQSDHSHYLALSKLL
KYFKWTWIGIIRFNDNSGEKELQLLSSYLSVEGICVEFTIKISNYIENIAVYKMYREIIQKSST
SVIILCGTPSVNFVGTFINMTDVFRTKTLVISSTVAANHNIIDYHLELFHGSLGFVQHAIYSL
DSPQMRSFIESLHPLRNPEDKLLEDIWMQYHLCLSEDPNKNDLYERVYAFKLHNCTRQDN
ITDILSIDSKLHFPPVHFAVHIMSGALHKMQNTSEKSKTGHYYKYKLHHYLKNIQYKTIKN
KTNIFNENGELIGEYLIYNYIMDPGAQVIVNYLGLFNPWASSNQKLEIASSKIRWKTNDSK
KFSKFDKCFPKIVEEIRSKVTLLCYYIFSDSEICTRCPDMEWPNENKNQCIIRNEEFLSYNN
YMISVFFSSISVLFFLTTLLILGVFITFRNSPIVRANNRSLSFLLLVSIKLSFLSVFLFLGRPVDI
TCMLRIITFGITFSIAVSSLLAKTIMVCVAFKATKPGSSWRKWLGVKLSNSVVLFCSSIQIIIC
MTWLAISPPFQELDIHTSPGTIIIQCNEGSAIGFYSVIGYMGLLAAVSFVLAFLARSLPDSFN
EAKYITFSMLLFCSVWIT
>jgi|Xentr4|342951|e_gw1.167.128.1
YTEIHTLLYGIDEINRNPDLLPNITLGYRVYDSCGDPRLAIGSALQILSGPGNVVPNYSCRG
KGEIAGFIGDRSTVTSLPIAQLLGVYGYSQISYGATDPVLNDRTLYPHYFSTGPNEHFQHIAI
AELVERLGWTWVIILATSNDGGQNLKNEINKHGACVDLIGTLTGDNDTDERTLERIQKSTA
EVVILCGGQSYDPYLVSVLEKMLNNKMVVVPVTWVFIPDYFLYNGCLQFQDTNMLFNDD
AEGKFTKHMFAPREDALLKDLLANCYFCLTHDKEKDNLFQRVYNHLYRNCSNITLPMSY
YQAYHRVTTAVNVLARAQHNLLSSSGKHNNTGLPTIIHRKQLHRYLRNVLLNEQRELPFH

EAYVIHSLYNDSEVKWDHIRVGEYTWSESGNSLQINTEEIVWMKDTKGQILKSQCSTNCP
PGYRKVPREGAPPCCYDCAPCSEGEISNLTDMDNCLKCGDYEWPNPEKTVCIEKQIQFLS
YDDSLTLVFIVHSLVFFIIAAVILGIFISFRDTPVVRANNHTLSFILLVSIKLSFLSVFLFLGRPV
DITCMLQQTSFGITFSIAVSCILAKTIMVCVAFKATKPGSPWRNWVGVKVAYCIVIFCSIIQIL
ISVIWLTISPPFLELNFLSEPGQIIIQCNEGSAIAFWFVLSYMGLLASVSFIVAFLARSLPDSFN
EAKYITFSMLLFCSVWITMIPAYLSTKGKYMVAVEIFAIISSSCGLLFCIFLPKCYIILFKPEM
NSKQYLLGNNK*
>jgi|Xentr4|343706|e_gw1.173.167.1
MDDLFTTTEFYYTDYTDFAEGKVCSLEDVRQFKKKFAPVVLSLIFAVGLVGNLLVVITFRY
YKRTKSMTDVYLLNMAVADILFVLTLPFWTVNYHKGEWIFKDFMCKFIRSIYAINFTCSML
LLACVGIDRYVAIVQVTKSFRFRTTTMAYKRVICFSVWIMSACLSGLTYYFSKCYKYNERF
VCEASYPEDATALKWKLAVIIVQISLGFCIPFFVMFFCYLCIIKTLLQAHNSQRHKAIRVIVA
VVAVFLVCQVPYNVVLVIKATQLGRTDGICSKNINYAYAFFITETVAFFHCCLNPVIYAFVG
VKFRNYFMKIMQDLWCISKQYMVGSRISRVPSETSRRTSEVYVTEGGSSFTM*
>jgi|Xentr4|344809|e_gw1.178.213.1
MSFATDMPFRTNLTTAVNASGDAPTSSMEDMIAMCSISAVLSIMCVVGVAGNVYTLVVMC
QSMRTAASMYIYIINLALADLLYLLTIPFIVGTYFVQEWYFGDVGCRILFSLDFLTMHASIFT
LTIMSTERYFAVLKPLDTVKRSKSYRKCIAVVVWVVSLLITLPMLIMIKLVHKDNKSICLPT
WSKVSYKVYLTVLFSTSIVGPGLIIGYLYIRLARTYWLSQTASFKQTKRLPNQKVLYLIFTIV
LVFWACFLPFWIWQLLTQYYESLPVSPKATKNINYLTTCLTYSNSCINPFLYTLLTKNYKEY
LRNRQRSWNNSSYFRNRFQRISGRSMSTSSQQCTESFVLAQCPGGNGTS*
>jgi|Xentr4|344875|e_gw1.179.105.1
MSLHFSWSIFICFSGPLPFNISSPGHGNTTQASRDNFTFSLYYQHSTSIAAMFILAYTFIFLMC
MIGNMLVCFIVLKNRQMRTVTNMFILNLAISDLLVGIFCMPTTLVDNLITGWPFDNIMCKM
SGLVQGMSVSASVFTLVAIAVERFRCIVYPFRQKLTLRKAIITIIIIWVLALIIMCPSAVTLTVT
KDDFHFMVDDYNNYYPLYSCWEEWPDTEMRKIYTTVLFSHIYLAPLTLIVIMYARIAFKLF
KSSAPIRGSVSEENEGRRVSKRKVRVIYMLIIVALFFSLSWLPLWTLMLLTDYGHLSDYQL
NLIAVYVFPFAHWLAFFNSSVNPIIYGYFNENFRRGFQEAFKVQVCSMKREHRETYSERKN
SGFLFGVRNRIFVEVQPSDSGQGSDSCNSTAKGGMFSLRSGRIAHHGLVVEDVDNKSSNN
TVSVPAWEI*
>jgi|Xentr4|345126|e_gw1.180.137.1
KDYSKMANHILNTSTLSNWTLVPQRNAGSVLLTSLHALASSVASNTQPLPNVTVQEEFLSL
QSQHISGDRDFTFYFNSSSQPVSGQILIEKAKARGLTDLSAVSLAYPTLGDILPTESENVSVN
GLVMSLAVSANISQIFLKFEKVNKSLSINTHCAAWDKGKETWSRDSCQKQEDSATHALCK
CKYSPNYLSFSILMSPKANSDPALHYITLIGLGISICSLVLCLMIEALVWRHVTRHSTAYMR
HVSIANIAVSLLLADIWFIIGDATFKAKSSNVCITSTFFTHFFYLSLFFWMLLMGLLILYRLFL
VYHDMSRTLMLATLFSVGYGCPFVISILTLAITLNQPGKPYTREDGCWLNWDPSKALLAFI
VPAFIILAVNAAVTCTVISVLLRPSIGQTPRTNERGYLVQIGRCVAILTPLLGLTWGIGIATIIE
DSSVVFDYLFTILNALQVSIYLILFWDVDKLPKGPHTRADQLTWQCRQASRSSPDIPTYGW
AIPGESRVI*
>jgi|Xentr4|345243|e_gw1.181.14.1
MFVNNSVHFSNKTTLVDFILTGLSTAYATELLLFVLFLVIYVFSFLSNLSMTLLIWAVPKLHT
PMYFFLSHLSLIDMCYSSTIIPKMLSDFLTKRKHISFSACATQMFFFAMFATSECFMVTAMA
YDRYVAICQPLVYHIIMNKNLCWLLVIGAYFSSLLASVTHTAAIFSFPLCGSQLINHFYCDIP

PLLKLVCMHSHARKSVVFSLSMVMGIISLLIILTSYVYIICAILGIQSTHGRSKAFSTCASHLT
VVILFYSSVFSIYFRPSLGLEFGNIDKFFSIFYTVASPLLNPLIYSFKNMEVKNALKHFLALKI
KTKLLNS*
>jgi|Xentr4|345687|e_gw1.184.106.1
SMVDSIVSQIEQMLTGEVQPNTATQLVGIMDSFLNLSMNLLAPVSKRLIGIVDKITLQLTFP
GQSINITSSALALAVEKINSSIFSGVAFGVKVSSDLQVSLGTNASQQNENSVVLPASLLNGL
SSTDQNIASRVQFNFFDKTSLFVDSSLQGKKQYLVSTVISASVSNLTLNYLQDNVTVLLQT
TSIKQTNVSMLCVFWNFKLNSGRGGWDSNGCSVVKTTVNQTICTCNHLTSFAILMDVSK
VNLSPEDTLILTFITYIGCGLSAIFLSVTLVTYIAFEKIRRDYPSKILMQLCAALILLNLTFLIN
PWIALYNNIPGLCISAAAFLHYFLLVSITWMGLEAFHMYFSLVKVFNTYVRKYILKFCIVG
WGVPAVVVAIILAVNKDLYGFQSKGKYPNGDSDDICWIADIIFYITVVGYYGIVFLMTISMF
IVVILQLCRIKKQKQLGFQKKITLQDMRSVAGITFLLGITWGLAFFSWGPGGVVIVYLFTIF
NTLQGFFIFIFYCVAKENVRKQWKRYLCCGKFRLAENSDWSKATTNKLKKQVSKQGASSS
SSSSIQSSGNSNSTTLLVSNEYSPNPTVNGNALKERNGLSFTLHNKELPLYEIPVKAPVKASI
SNERPQTSVRRTSKMGNVHFMDQK*
>jgi|Xentr4|345786|e_gw1.185.97.1
MTFNAIESYTKAYASFNTSSFPLENALIQSNRTCTLSESWEWIFSFQPIYMWIVFVLGFIEN
MFVLIVFLLHKKRCTVAEIYLGNLAAADFLLVCGLPFWAIYISNKFHWPFGSFLCVFINGLI
QLNLYGSIYFLMMVSIDRYLALVKTMSFGRMRTGRCAKLNCLLIWIFAVVSSMPKTIFRRIR
YFPEFNVTACVIDPPSPSWNVASGLLLTLVGFLIPVTVISYCSFHIINVLRNNSMQEFKEINK
EKKASVLVLVVLLVFIICWLPFHITTFIDTLHMMNLFSSCAMNNFIEIASQISSYLGYSNSFIN
PLLYVMVGNHFRKKVKEVYNQFQIRKQRGRNASIPTDFTADTVRTSISMEPQKNRILL*
>jgi|Xentr4|345966|e_gw1.187.97.1
MTARAEEGNTVSVAFPLSMMITGLVGNALAMLLVYKSYRNKESARKHSFLLCIGSLALTD
FIGQLLTSPIVISVYLSNRQWARVDPSGHLCPFFGLCMTMFGLCPLFIASAMAIERTLAIRTP
HWYSSHMKPKATKMVLLGIWIGVLGFALMPIIGVGQYTLQWPGTWCFISTGDGEAVGNIF
FASTYACLGLLSLFTTFACNLSTIVALVSRCKRRSSTANASRQWERITLETLIQLLGIMCVLF
ACWSPLLVIMLKMMSNHTSVEHCKPKASEQNTELQKDCNFFLTAIRLASLNQILDPWVYL
LLRHILLRKFCQVANAVSNCSNDEQKQQPMIPANDVRIAEG*
>jgi|Xentr4|346134|e_gw1.188.71.1
MENSSKFDSPTNISLANHDMVFSMIEYKAISVFLVLFICGIGIIGNIMVVLVVFTTRDMRTPT
NCYLVSLAVADLMVLVAAGLPNVSDSLAGTWIYGHAGCLGITYFQYLGINASSCSITAFTV
ERYIAICHPMRAQTVCTVSRAKRIIAIVWIFTSIYCMFWFFLVDIHVNKSQQVECGYKVSR
NLYLPIYLADFAIFYVTPLLVATILYGLIGRILFLSPIPQHPESTTERWREKSSKEKTESETEGN
KPSNRLKTKGALSSRKQVTKMLAVVVVLFALLWMPYRTLVLVNSFMDKPYLDPWFLLFC
RICVYANSAINPVIYNLMSQKFRTAFKRLCKCGHEAQRRSIYMTTTSYSMVKDGVNTGGE
KKEKQSLEKTNIIAKQTNKAGPEGDLYYSVV*
>jgi|Xentr4|346324|e_gw1.190.53.1
QVSYGAVDPAFSDRIRFPLFYRTVPSETAQYQVIIQLIKAFNWNWVGMISSDDETHQKASE
EMKDEIIKNGICVAFLVQIDEKNYRSFDFALQNIKKSKVYVVILISKLITLLMIGAVFPYTKV
VWLMLSSLASATGVGSHKFNGALAVLFHKGNIPGLKDFLYKADPSRFPKDPFTAAVWLDV
FDCHSEQAFPKPSSRSYRICNANHTLKQYEMNGYDTSNFRLTYSTYIAVYAVAHALHAMY
TDKTSGGTILHFHKTINPLKPTQLKNYLQKLNFKTYDGDDIFFNEKGEVLGHFDIINWNIF
QNETVITRHVGTFNSSLSPGLAIDKEAILWPTYFNGIPVSRCSASCLPGYRKIFVEGKQVCC

YQCVRCSESEISSLPDMGNCIKCPEDQWSNERRDKCIMRTIDFLSYKEPLGVALTTTALVLS
FCSAAVFFLFLKHKKSPIVKANNQELSYILLLSLMLSFLCSLLFIGRPTKVTCFLRQVVFGIIF
AICISSILGKTITVIIAFTATRPGSRLRNYVGTRVPKYILLLCTLPEVFICALWLIISPPFPDYDT
HSATGKIILQCNEGSPSAFYIMVGYIALLAFVSFFVAYLARKLPDIFNEAQYITFSMLLFCSV
WISFILAYVSTTGKYLAAVEIFAILVSSAGLLGLIFIPKCYFILIKQPVNTRHN*
>jgi|Xentr4|346328|e_gw1.190.37.1
SYSIHYYRHYLAAIFAIEEINKNPNILPNITLGFHVYETCSSEREALANVLSILTRRSQYVPN
YKCSGNKGTVGAFVGHLFSSISHAMYLILGMYRFSQVSYGTQDTSLRDRIRFPALYSTMA
SDNSQCKAVIHLLKTFNWTWVGILTSDDADRHRKNEELKAQIINGGAACVEFFIVVQKKQ
IGSLGAIVQIIKKATANVIIVDLDIFYFVNTVLSLENYSKRSIFWISLSTKASVVVSFPAQGLN
GSLLLVLAQEEIPGLPEFIYSANPSTFSRDPITASVWNQLFSPLIDKPDTNNTLKTFETSSYN
LNEYRLTYNIYTSVYALGHALHNMYSDSVSSRLGYTKSLATFMPWKLNSYLKNVHFKTT
NGHEIFFNEYGEAPGRLDILNFNIFPNGTMTTRKVGNFGSSALQGDSFIIYKDNIQWAPHFN
GVPYSTCSKNCPPGSRKVFIKLMPNCCYKCILCSEGEVANISDMENCVRCPKIQWSNEQRD
KCIMRALDFLSFEDPLGMFLAFLAISLSILSTAVLIIFIKYKHTTIVRANNRELSYYLLISLILS
FLCSLLFIGWPMKGTCLLRQVVFAVTFTFSISSVLGKTLTVVIAFNATRPGSKLRGWVGTRI
PKCLVLLCTLVEIFICSLWLIISPPFPGYDAESHTATMILQCNEGSALAFYSVIGYIALLAFICL
FVAYLSRKLPDIFNEAQYITFSMLVFCSVWVSFIPAYLSTKGKYLTSVEIFAILASGSGLLGLI
FVPTCYIMLLKPQQNTKMYLVQLKHK*
>jgi|Xentr4|346332|e_gw1.190.5.1
MLLCYYVCSSLQHYRHFLAAIFAIEEINSNLNILPNVTLGFHIHDSWASERKAISSTFSMLTG
SSDYVPNYKCTQNRIPSAFIGHLLSSVSNVMYQITSIYGFGQVSYGAVDPAFSDRIRFPLFY
RTVPSETAQYQVIIQLIKAFNWNWVGMISSDDETHQKASEGMKNEIKKNGICVAFLVQIGE
RPGTSFYFALQAIRQANASVVIVYSKLINFLMFSFILQSTHLSTNLVWLMLSPLASATERPY
LQFNGSLAILFRQGNIPGLKDFLYKADPSRFPKDPFTAAVWLEVFCCHSEQAFPKSPFRSYY
ICNANDTLRQYEMNGYDTSNFRLTYSTYIAVYAVAHALHAMYTDKTSGGTILHFDKTINPL
MPTQLKNYLQKFRFKTHSEDEIFFNEKGEVFVRFVIINWNIFHNGTVTTRHVGTFNSSQSP
GLAIDKEAILWLSDFNGIPVSRCSASCPPGYRKTFVEGKQVCCYQCVRCSEGEISSLPDME
SCMRCLEDQWSNEKRTKCIMRATDFLSYEDPLGVTLTATALFLSFCTAAVFCIFIKYKKSPI
VKANNQELSYLLLISLFLSFLCSLLFIGRPTKATCLLRQTLFGIIFAICISSILGKTITVIIAFTAT
RPGSRLRNYVGTRVPKYILLLCTLPEVFICALWLIISPPFPDYDTHSATGKIILQCNEGSPSAF
YIMVGYIALLAFVSFFVAYLARKLPDIFNEAQYITFSMLLFCSVWISFILAYVSTTGKYLAA
VEIFAILVSSAGLLGLIFIPKCYFILFKPPVSTRGHLVEQKL*
>jgi|Xentr4|346334|e_gw1.190.287.1
FIVTQVSYGAVDPAFSDRIRFPLFYRTVPSETAQYQVIIQLIKAFNWNWVGMISSDDETHQK
ASEEMKNEIINNGICVAFLVQIGDTDGRSFYFALQNIKQSNTSVVIVYSKLINFLMLSMILPS
THFSTNVVWLILSPLASATERQYREFNGSLAILFRQGNIPGLKDFLYKADSSRFPKDPFTAA
VWLEVFGCHSVQAFPKSPFRSYRICNANDTLKQYENYGYDTSNFRLTYFMYIAVYAVAHA
LHAMYTDKTSGGTILHFDTTINPLKPTQLNNFLKKIHFDMHFEEDIFFNEKGVVLGHFVIIN
WNIFHNGTVTTRHVGTFNSSQSPGLAIDKEAILWPSDFNGIPISRCSASCLPGFRKTFVEGK
QVCCYHCVRCSESEISPLPDMENCIKCHEDQWSNESRDKCIMRTIDFLSYKDSLGVALTTT
ALVLSFCSAAVFCIFLKYRKSPIVKANNQELSYILLLSLMLSFLCSLLFIGRPTKATCLLRQT
SFGINFGICISSILGKTITVIIAFTATRPGSRLRNYVGTRVPKYILLLCTLPEVFICALWLIISPPF
PDYDTHSATGKIILQCNEGSPSAFYIMVGYIALLAFVSFFVAYLARKLPDIFNEAQYITFSML

LFCSVWISFILAYVSTTGKYLAAVEIFAILVSSAGLLGLIFIPKCYIILIKQPVITRHN*
>jgi|Xentr4|346337|e_gw1.190.279.1
ISYGTQDSSFSDRIHFPTFYSTMTSENTQNKAVIHLLKTFNWTWVGILSSDDADRHIRNEKL
KAQIIDSGAACVEFFSVVKEKQIGKLYVLTKTIEKSTANVIIVDINMRYFIDILWSLADNSKR
KIVWIFSIVTSSVLVNYQMKALNGSLLLALPQGEIPGLQAFIYGVNPLTFSRDPITASLWDE
VFAQLNEIGTINNRNGTLKTLEPYIPSFNTYRLTYNTYISVYALGHALHNMYMDSISNRLD
SSQFLDTFKPWKLNSYLKKVNFKTISGDTIFFNEYGEAPRGLDILNLSIFPNGTMTTRKVG
NFDASALHGDNFIIYKENILWSPHFYGIPHSTCSKNCHPGSRKILIKEMPVCCYNCIQCSEG
EVSTVSDMENCVRCPKIQWSNEQRDKCIMKTLDFLSFEDPLGLSLAFMAIFLSILATAVLIIF
IKYKHTTIVKANNRELSYYLLISLILSFLCSLLFIGWPMKGTCLLRQVVFAITFTFSISSVLGK
TLTVVIAFNATRPGSKLRGWVGTRIPKCLVLLCTLVEIFICSLWLIISPPFSEYDTESHTATMI
LQCNEGSAFAFYTVVGYIALLAFICLFVAYLSRKLPDIFNEAQYITFSMLVFCSVWVSFIPAY
LSTKGKYLTSAEIFAILASGSGLLALIFVPKCYIILLKPEKNTRIYLIQLKNT*
>jgi|Xentr4|346345|e_gw1.190.11.1
MDLPLITITLCRLLLILLWYKDGGCETLLEGCRLVTEEVSGYSLPGDITLGGLFPVHVEVTY
PVITYRERPKPLLCSSFQIRFYRFLLAMEFAIMEINASNDLLPNITLGFHLYDACYNEIRSLIG
ATWILSGKKLGVPNFHCNKDLMPLAIVGDMPSKASEPLARILGLYRYPQISYGSGLPLLSN
KIHFPSFFRTIHKVEYEWFAISQLVKYFNWTWVGVISSNNDLGILGAQIVTSEIEKNGGCIA
FQETLPIISSMESVYRIIGLVKRSRATAIILFCTIENLVPLMEQASFHNITGKVWVGTSGWSIT
SDFPRTDILTTLNGSLGIAPQKGKIPGFKEFLYSIHPSRFPDDPYMKTFWETAFHCIWPGNDA
VNNTSPALLKEDIVWCTGEERLDSIDPNIYDVYNFIYSYRTHNAVFAVAHALHLMKNCVPG
KGPFKNGSCADIYNHRPWQLLHYIRKVDFNNTAGKRIYFDENGDVPQSVEILNWQLFPNG
SNQYVSIGSFDFGSLNGEGLSIQLNKILWNGGHGQVPSSVCSDPCPKGYRRAAIQGQKICC
FDCLPCSEGEILNPNDDSECLKCPEDKWPDSRKEECLPKSIKFLSYEETLGSILACISVLFCL
LTFSVFCLFIIKRKTPIVKANNRDLSYLLLISLMFGFMCSLAFIGRPNRIMCMIRQVMFAVIF
SLCVSTILAKTITVVMIFSATNPDSKLKKLVGLRIPIYIVPVCTMVQVILCIVWLTTESPFVEF
NMAAEIGIIVIECNEGSRVLFASVLGYMGLLASISLFVAFLARKLPDTFNETKFITFSMLVFA
SVWVTFIPAYLSTKGKQTVAVEIFAILSSSAGCLFCIFSPKCYTILLHPEMNSRQYITGRNTR
NRGKRF*
>jgi|Xentr4|346379|e_gw1.190.38.1
MFKTPTSLCTELCSSGHRRAHQNGRPPCCFDCVSCSEGEISNYTDLKTCVKCAEDQWPNP
ARDQCIKRTIDFLSYKDLLGYILSGCASVFIVLTAAVLFVFIKHRRTPIVRANNQNISYILLM
ALLMSFLCSFIFIGQPTGVTCMLRQALFLIAFSVAISSILGKTSMVLAAFKATKMKKGFREC
GRINISVGVVFLFSLGEFVICVIWLILYPPHVESDNKTLPEKTILQCNEGSIISFYLAVSYIGVL
SLISFAVAFIARKLPDRFNEAQHITFSMLVFCSVWASFIPTYLSTKGKHMVAVEIFAIQASAA
GLLLCIFTPKCYIILLKPELNVKGKPTAKIQSHTEKHMLHC*
>jgi|Xentr4|346395|e_gw1.190.20.1
YRHYLAAIFAIEEINKDPDILPNITLGFHIYNTCSIERLALTDVLSILTQRSQYVPNYRCNNK
GTVGAFVGHLFSSLSHAMYLLLGIYRYAQISYGTQDTSFNDRIRFPTFYRIMTSESTQNKAV
IHLLKTFKWTWVGILTSDDTGRQERNEKLKAQIIDSGAACVDFFIVIKEKHLSLIHGAIKTI
KRSTANVIIIDIHIMHFSNIAISLSDNSHRKIIWISLSSLSTAVVNYPLFALNGSLLLMLSQEEIP
GLQKFIYSASPSKFPRDPVIASLWNSVFRPLTAKRNTSNINDLLKICGMSPLNFNAYRLTYNI
YISVYALGRALHDMFLDKVVISRLGFPEFMPWKLNSYLKKVNIKTRSVDEMFFNEYGEAP
GRLDLLNWNIFPNKTIITRKVGEFNSSAFHKENFIIYKENILWAPHFYGIPHSTCSKNCPPGS

RKFLIKEMPTCCHNCVRCPEGEISDSGRNMENCVRCPKIQWSNEQRDKCIMKTLDFLSFE
DPLGLSLAFMAIFLSILTTAVLIIFIKYKYTTIVRANNRELSYYLLISLILSFLCSLLFIGWPMK
GTCLLRQVVFAVTFTFSISSVLGKTLTVVIAFSATRPGSKLRGWVGTRIPKCIVLLCTLVEIFI
CSLWLIISPPFPEYDTESHTATMILQCNEGSAFAFYTVVGYIALLAFICLFVAYLSRNLPDIFN
EAQYITFSMLVFCSVWVSFIPAYLSTKGKYVTSVEIFAILASGSGLLALIFVPKCYIILFKPEL
KPRLGLIQLKHM*
>jgi|Xentr4|346396|e_gw1.190.12.1
MLHALAFIYAIEEINSNENILPNKSLGYIMYDACSNEVIALDRIFRILSGTNEAMPNYICQKR
HNPVAVIGHSLSSTTYTIAQITQPYGYSQLSYGAMDPVFNDRTLFPSVYRTVPNEYLQFEVI
VQLLIHFKWKWVGIVSSDDISNHKASLELIKMMTNHGICVEYHIVIPVSTAVNMKRFLIFIQ
QSTATVIILYCTLLQIRNLTKLWDTDASQMTGKVFVASVTLNDIDDHLLKGFFKTLNGSLFI
SVHKGEIPGFQNYLSHRDWGRMPDKHFVENFKMFLKCLTVPSNKSQSRCLLFEHWENTY
YKATVIYNTVHILAQALHQMHLDRGLFDNGSKDLAAESKHKLNQYLKNVLLKTLSDEIFF
NEQGSVAGKFDILNLIIKPKNQIRKIPIGSFVPSASPKLIINESAIVWNPYFKQIPISLCTGICPT
GYRKSIQKGKLPCCYDCIPCSEGEIASSPDTENCLQCPEDHWSRPSRDICLEKTLDFLSYGN
CLGMVLATAALLFSVCTGAVLWLFIKNRSSQTVKANNRNLSYILLVSLFLSFCCCFLFIGRP
VPITCILRQTAFLFLYTVAISSLLGKTLTVIIAFHATKPGTRLRTFVGSRVSIYLVLLCSLGKLA
ICSTWLIWATPFVALDTKTTQQTMTLWCNEGSIAAFCVAVSYTAVLALLSFIVAFMARKLPD
RYNEAQHITFSMLVFFSVWVSFIPTYLSTKGKYMVAVEIFAILASTAGLLSCVFVPKCYIILL
KPKQMTTKHNIILL*
>jgi|Xentr4|346441|e_gw1.190.182.1
MLLCHKHGACNTSLEGCRLSSHDVIGYSLAGDITLGGLFSLHMEVTQPTVTYRESPQPFQ
CVKFHIRYYRFLLAMVYAIMEINASDDLLPNVTLGFILYDSCYNEVRSLTGTAWILSGTKN
GVPSFSCKEGNMPLAIIGDMPSKASIPIARILGLYRYPQVSYASATPLLSDKTQFPSFLRTIHN
SYYEGFIIADLLKYFNWTWVGIIVSDNDLGRTGLQIVTREYEKNGGCIAFQEILPIFSSMKS
VLRIVDVVKKSKATVIVLFCTTENLVSLMEQASFHNITDKVWVATPGWSITSDFPRRDILTT
LNGSLGLANQNGKIPGFKEFLYSIHPSRFPDDPYMKSFWENAFHCIWPGDDAVNNTSPALL
KEDIVWCTGEERLDSIDSNIYDVYNFKYTYRTHNAVFAVAHALHQMKNCVPGKGPFKNG
SCADIYNHRPWQLLHYLKRVDFKNTAGERIFFNENGDVPVSVDILNWQLFPNGSNRYVSI
GSIDTGTPKGKVFRIQLKEILWNTGNSQVPVSVCSDPCPKGYRRAALQGQKICCFDCLPCS
EGEILNPNDDSECLKCPEDKWPDSRKEECLPKLIQFLSYEETLGSALACISVLFCLLTFSVFC
LFIIKQKTPIVKANNRELSYLLLISLIFGFLCPLAFIGRPNWIMCMIRQVMFAVIFSLCVSTIL
AKTITVIMIFSATNPNSKMNKLVGFRIPVCIVPVCTVIQIILCIVWLAQAAPFAEFNMAAEIGI
IVIECNEGSRVLFACVLGYMGLLASVSLFVAFLARKLPDTFNETKFITFSMLVFASVWVTFI
PAYLSTKGKQTVAVEIFAILSSSAGLLVCVFFPKCYIILLHPEMNSREYITRRNAGSQET*
>jgi|Xentr4|346464|e_gw1.190.116.1
METSNKTSVNEFILVAFSDFQLNQILLFIIILLMYITCISGNITIISLVKYERSLHKPMYIFISVF
AFLEIMFVSATMPKLLANLLVSNKQISYVGCFAQMYIFNALGETECYLLAIMAFDRHLAIN
NPLRYTSIMNFKVCSELAFLPWIIGFSASFIPTMFTASLEFCGPNVIDHFFCDLSPVQNLACS
NPFISNIVTIALAFFMLIVPFIMIIAFYVGIIITISRIKTSEGKLKAFSTCSSHLIVASLFYGTCIIV
YVRPKGSRYDKFLALMYTIITPLLNPFVYTLRNKDVKDILKKRIRWLLQKSVM*
>jgi|Xentr4|346499|e_gw1.190.22.1
IFAIEEINQDQNILPNVTLGFHIHDSRANERKAISSTFSMLTGSSDYVPNYKCTQNRIPSAFIG
HLLSSVSNVMYQITSIYGFPQVSYGAIDASFGDRIRFPLFYRTVPSETAQYQVIIQLIKAFNW

NWVGMISSDDETHQKASEEMQNEIKKNGICVAFLLRIGGKDVRNYHRALEKIRQSNASAI
LFYSSLDIVMEFLFDVTLKNSSVHVVWIMLSPLSSGSGRLYIDAINGSLVLLFHHENIPGLK
DFLYKANPLGFPKDPFTAAVWLRLFDCYFEQASPKPSSNFYHICNANYTLKEYENFGYDV
NNFRVTYSMYIAVYAVAHSLHAMYTGKANGTNTFYSDKVTDGLTPTELNQHLKKIHFRTN
TGNEIFFNEKGEVLGNFDIMNYNVFQDVTGTATHVGAFISSQSPGLTIDKEAILWAPHFNGI
PTSNCSASCQPGYRKAFAEGKQICCYECVQCSEGEISPSPDMENCIRCPEDQWSNESRDKC
IIRLVEFLSYKEPLGVALTTTALVLSFCSAAVFCLFLKHKKSPIVKANNQELSYILLLSLMLSF
LCSLLFIGRPTKVTCLLRQAVFGIIFAICISSILGKTITVIIAFTATRPGSRLRNYVGTRVPKYIL
LLCTLPEVFICALWLIISPPFPDYDTHSATGKIILQCNEGSPSAFYIMVGYIALLAFVSFFVAY
LARKLPDIFNEAQYITFSMLLFCSVWISFILAYVSTTGKYLAAVEIFAILVSSAGLLGLIFIPK
CYIIFFKTPVSTRAHLI*
>jgi|Xentr4|346529|e_gw1.190.57.1
MELLFFFKTPISMCTGMCSSGHRKAHQTVRPSCCFDCLPCSEGEISNSSDMETCVKCPEDQ
WSNPTRDQCIQKTIDYLSYEDLLGYILSSCASVFIVFTAVVFLIFIKHRRTPIVRANNQNISYI
LLMALLMSFLCTFMFIGQPTGVTCMLRQTTVMFVLSIAISSILGKTVTVLVAFKSTKTSSNI
RRWGKIYFSMNAVFLFSFGEFLICIIWLTLSPPYVEVDIKIIPGTIILQCNEGSTISFYLAVSYIG
VLSLISFAVAFIARKLPDRFNEAQHITFSMLVFCSVWASFIPTYLSTKGKHMVALEIFAIQAS
AAGLLMCIFTPKCYIILLKPELNVKGKFTFTIKPTKCIYVH*
>jgi|Xentr4|346540|e_gw1.190.434.1
MENNTISKVMEFFLVAFSDFHQFQIVLFIIVLLMYIICIVGNITIIALVRTEPSLQTPMYFYISIF
ATLEMIFVSVTIPRLLANLIADNKSISFFGCFAQLYAFNALGETECCLLAIMAFDRHLAINNP
LCYSAIMSHEFCKELALLPWIIGLVTSFIPTIFTAGLQFCGPNKVNHFFCDFAPLQNLACSDP
FISNVMTSFVATVTVVIPFIIIIGLYIHIIFIIVSSIKSSESKHKAFSTCSSHLIVSSLFYGTVITVYI
RPKGSQYDRFLALAYTVFVPLLNPFIYTLRNRNVKGALYKLLRHLFNQPFF*
>jgi|Xentr4|346544|e_gw1.190.184.1
MYLKPIYFRHLYLFLTLLWYKDGICNTSLEGCRLFAEDAIGYSQAGEITLGGLFSVHMGVF
APSVTYRESPKPLQCVRFHIRYYRFLLAMVYAVMEINASGDLLPNITLGFILYDSCYNEVRS
LIGTRWILSGKINAPNFDCNKHNIPLAIIGDMPSKASVPVARILGLYRYPQVSYASGTPLLSD
KIQFPSFLRTLNNGDYEGYAIGKLLQYFNWTWVGIIFSDNDLGRAGLQMITKGLESYGGC
TEFQEILPIVPSMESVFRIINVVKKSKATVILLICTIENLVSLMEQASFHNITDKVWVGTSGW
VITSDFPRTDILTTLNGSLGLAAQKGKIPGFKQFLYSIHPSRFPDDPYMKTFWENAFHCIWP
GNDAVNNTSPALLKEDIVWCTGEERLDSIDPNIYDVYNFKYTYKVHNAVFAVAHALHQM
KNCVPGKGPFKNGSCADIYNHRPWQLLHYIKIIDFNNTAGERIFFDQNGDVPIFVDIVNWQ
LFPNGSNQYVHIGSFDAGAPKGQEINIQLNKILWNVEKSQIPVSVCSDPCSKGYRRAAIQG
QKICCFDCLPCSEGEILNPKDDTECLKCPADKWPDSRKEECLPKLIQFLSYEETLGSALACI
SVLFCLLTFSVFCLFIIKRNTPIVKANNRDLSYLLLISLMFGFMCSLAFIGRPNRIMCMIRQV
MFAVIFSLCVSTILAKTITVIMIFSATNPNSKLKTLVGFRIPIYIVPVCAMVQIILCIVWLAQA
APFAESNMAAEIGIIVIECNEGSRVLFACVLGYMGLLASISLFVAFLARKLPDTFNETKFITF
SMLVFASVWVTFIPAYLSTMGKQTVAVEIFAILSSSAGLLVCIFFPKCYIILLHPEMNSREYIT
RRNGKNQEI*
>jgi|Xentr4|346551|e_gw1.190.15.1
FLSRFNIKYLESLLAFVYAINEINSNPDLLQNITLGFHIVEPCFQEQKAMIGMVELLAGSKA
PVPNYRCNHSHPLLTVVDGISSKVTLLFARTFGSYKIPQISYASMDSVLSDKVQFPFFYRTV
PNEASQFQALVLLIKQFGWSWVGILVSDNDSGLQASQTLKVELELIGACVEFLEFLPYRKS

LDDSRKMKIYKTLIASSSKVIIAYGDRDYMLVLHIILYMYPVPPKVWIISVQWNFSTGSEAS
FLNFIPFNGSLALTLQSKSIPGFKEFVYGIRPDLYLNYQFISDSWGEFFGCDWKEKHDEQYK
CTGNENILNVENGNLDKDLSFYTYSIYNAVYALAYAFQALLSEKMEEHTYAGDIRPWELH
KYLKNVRFTNSAGEEVHFDDKGEFNSDIDILNWIVYPNETMDGIRVGKYHQKSLLKKIIIN
ESMIRWSAHFNQTPQSACSGTCFPGFRNSIEPGYRSGPVSHYPSKLNAQTCIKCAEDQWPN
ANKDKCIYKVITFLSYEEPLGISLVLISILFFIFTCVVLTIFTLYRNTPIVKANNRDLSYILLFSL
MMCFLCSLLFIGHPLRVTCILRQTVFAIAFAISLSSTLAKTVTVIVAFNATRPGNKLRDWMG
PSVFNTLVLLGGLIQVFICAGWVGISPPFPYYNMKDDIVVILAECKEGSLLGFYCVLCYLG
LLASISFVIAFLARRLPDAFNEAKLITFSMLVFCSVWISFIPAYLSTKGKYTVAVEIFAILSSG
AGLLSCIFLPKCYIILRRPEQNTKVFITQKG*
>jgi|Xentr4|346603|e_gw1.190.397.1
AFMYAIEEINNSTELLPNITLGYRIYDACNSEEIALMSTFSLLSEEETPALNYICQPDQKLVA
FVGHALSSTTYSIAEITQLYGYPQISYGALDPVFNDRVHFPSVYRTVPNEYSQFRVIIKLLNH
FGWTWVGIITSDDDSNRQASEELRKEMGRKGICVDFLKVISSSPDRLDKSATEAIETIKRSS
VRVIILYCRANSLLVLLVRLRSNKQLSESVWISSVGLDIVDEYEFKKYLYSINGSLLISLPKG
DIPGFSTFLSQVIWTDMLKNKFVKYFWGLTIGCSSVFSNSVENFTCRLAHRAEEYLLQEET
SNACIKNTIYMAVYLLAHALDKMHLVGDFSELPSKERIGKIRSKLNYYLKNTHFKSASGEE
LCFTKDGNIPGKFDILNWVIYKNGTVKKIHIGRFFPGSDQLIINESAITWGPYFRKNPVSLC
TEMCFSGYRRAHQNGRPPCCFDCVLCSEGEISNFSDLETCVKCAEDQWPNPTRDQCIKRTI
DFLSWDDLLGYILSGSASVFIILTAAVFVVFIKHRRTPIVRANNQNISYILLMALLMSFLCTF
MFIGQPTEVTCMLRQITFVFAFSVAISSILGKTMTVLVAFKSTKTSGNFRWWGKMHFSISAV
FLFSFGEFVICVIWLILFPPFVESDNQTIPGNTILQCNEGSIISFYLAVSYIGVLSLISFAVAFIA
RKLPDRFNEAQHITFSMLVFCSVWASFIPTYLSTKGKHMVAVEIFAIQASAAGLLMCIFTPK
CYIILLKPELNVKGKLTAK*
>jgi|Xentr4|346613|e_gw1.190.401.1
LSYGAMDPVFNDRTLFPSVYRTVPNESLKFEVLVQLLIYFKWTWVGIISSDDIRNHKASLE
LIKEMKNQGICIEYHIVIPVSTNNININQDIKLIQQSTATVVILYCTLLLIIRLIVLWDIGISRMT
GKVFVASVALNAIDDSIYMQSFITLNGSLLLSVNKEEIPGFKNYLSHKDWGRMPDKHFVE
NFNMFLKCYTVQSNNSPSHCLLSHRWKNSNYTTRVIYNTVHILAQALHQMHLDRALFNH
GSKDLTEESKYKLNRYLKNVHLKSFSDEIFFNEQGSVAGKFDILNLISESKTQIRKVPVGSF
VPSASLKLFINESAIVWNPYFNKTPVSLCNDICPTGYRKALEKGKFPCCYDCIRCSEGEIAS
SPDAENCLQCPEDHWSNPSRDTCLRKAVDFLSYGNCLGMVLATAALLFSVCTGAVLWLFI
KNRSSQIVKANNRNLSYILLVSLFLSFCCCFLFIGRPVPITCILRQTAFLFLYTVAISSLLGKTL
TVIIAFHATKPGTRLRKFVGSRVSISLVLLCSLGKLAICSTWLIWATPFVALDTKTTQQTMTL
WCNEGSIAAFYVAVSYTALLALLSFIVAFMARKLPDRYNEAQHITFSMLVFFSVWVSFIPTY
LSTKGKYMVAVETFAILASTAGLLFCVFVPKCYSIFLKLFNINTEKG*
>jgi|Xentr4|346618|e_gw1.190.119.1
MTITSHIKFNFLFSFLDVETCVKCAEAQWPNPAKDQCILRIIDFLSYQDILGHILSGCALVFI
VLTAAVFLVFIKHRRTPIVRANNQNISYILLMALLMSFLCSFIFIGQPTGVTCMLRQTTVLLV
LSIAISSILGKTTMVLAAFKATKMERTFRKWGRINISVVVVFLCSFGELVICVIWLSLYPPHV
ESDKTIPGKLVLQCNEGSIISFYLAVSYIGVLSLISFAVAFIARKLPDRFNEAQHITFSMLVFCS
VWVSFIPTYLSTKGKHMVAVEIFAIQASAAGLLMCIFTPKCYIILLKPELNVKGKPTAKIQL
HIENHIPHF*
>jgi|Xentr4|346654|e_gw1.190.398.1

MFPGLSWSALGGAELLYIILISIDQVFPSPTDDYYRADQGHGCVLSSGSSPTKFSRDGDIVI
GGILKIFFITANRPTDFQNFPDQIKCVGPTFHNLKNLLAFIYAIEEINSNTDLLPNITLGYHIY
DVCASEELALKSTLNLLSNNENPSPNFICHTRNLVAFVGHVLSTITNTVAQISQLHKYPQIS
YGALDSVFTNRILYPSVYGTVPTVSSQSRTIAQLLTHFGWTWVGIVAANEDSYLQASEELI
KEIKRVEICVEFKAIVATSKLARDQTIINTIGLIERSSARVIILYLGANQFLPILFNAPSKLISER
LWIASVTLDIITDYKFKQALRSFNGSLLISIPEGDIPGLSDFIPQYIVTNIANNTFIQSFWAFNL
GCTEELQLSNNNCSKNEILKEFLSQNLPSSYHTSHAIYVAIYALANAIHNMILATELLQPWL
WGLNHYLKDLHFKKASGENVV*NADGNIPEKFDVLNFVIYSNGTIRQIRVGRYLPEAYQL
DIKRKSLFPFDLPSLAFSCSLTEIMIFPSDNLILTCSSCSPLPASSLGPGPENMKICVKCPEDL
WPNPNRDKCIQRITEFLSFEDPLGSALSCCASIFIIVETILFTFIKHRNTPIARANNLKISYILL
VSLKLSFLCTFLFIGRPTQWTCLLRQAMFVFIYSVSISSVLAKTITVLLAFKATKPENKFRS
WVGARLSFVLVFLCSLGELVICATWLLYALPFVDTDSKSLPGIIILQCNEGSLTCFYLAIFYI
GTLAFFCFIVAFIARKLPDRFNEAQHITFSMLVFCSVWASFIPTYLSTKGKHTVAVEIFAILAS
NAGLLTCIFAPKCYIILLKPQLNTKAKLFVKIKPSK*
>jgi|Xentr4|346703|e_gw1.190.34.1
VSYGAVDPAFSDRIRFPLFYRTVPSETVRFEVIIQLIKTFGWNWVGIVTSNDESYQKTSEEM
RNEIVKNGFCVAFLVKIAENDYRTLHEARETIIQSTANVVILHSRLVILFNMFFKMNDRGNH
VWLMLSPFPLSLSSLYLEMNLLLNGSIFIHFHHEAIPGLKDFLNMGNPSEFPNDPFTAAVWL
EVFACKVFANSSLYETCNANHTLKQYEMNGYDMSNFRLTYSMYIAVYAVAHALHAMYM
DKASGGTILHSDKTTYGLMPMQLNRYLKKIHFKTPSGDEIFFNDKGEVPRHFDIINWNIFH
NGTATSRPVGIFISSPSPELVMDEKAIFWAPQFYGIPISRCSTSCPPGYRKAVIEGKQICCYEC
VQCSEGEISPLPDMENCIKCPEDQWSNEKRSKCIMRTIDFLSYEDLLGIVLTTTALVLSSCTS
AVFYIFIKYKKSPIVKANNQELSYILLLSLLVSFLCSLLFIGRPTKVTCLLRQAVFGIDFAICV
SSILGKTVTVIIAFKATRPGSRLRNYVGVRVSKYILLLCTLPEVFICALWLIISPPFPDYDTHS
ATGKIILQCNEGSPSAFYIMVGYIALLAFVSFFVAYLARKLPDIFNEAQYITFSMLLFCSVWI
SFILAYVSTTGKYLAAVEIFAILVSSAGLLGLIFIPKCYIIIIKPAVSTRCQLLE*
>jgi|Xentr4|346705|e_gw1.190.18.1
HYRHFLAAIFAIEEINSNQNILPNVTLGFHIHDSRANERKAISSTFSMLTGSSDYVPNYKCT
QNRIPSAFIGHLLSSVSNVMYQITSIYGFPQVSMGSKDPKSVNRSQKCISRYCWWPSLVQA
GTSLVSAETPRPQPLPVPTQPLLGSAMDFIVDLPKSSDMTTVLVVVDRFSKMDHFISLKKI*
QEPHVELFRKPPFSFSYLQEGDIMLGILTDIFALINTWRETFMARPEKELAVLPVQYTKEFN
GSLAILFHQGNIPGLKDFLYKADPSRFPKDPFTAAVWLKVFGCHSEQAFPKSSFKSYRICNA
NDTLKQHEKHGYDVNCFRLTYSMYIAVYAGAHALHAMYMDKASGGTIMYSDKTTNGL
KPTQLNNHLKKIHFRTDSGDEIFFNEKGEVLGHFDIINWNKFHDGTVTTRHVGTFISSQSPG
LAIDKEAILWASPFNGIPVSRCSASCLPGYRKAFAEGKHVCCYECVQCSEGEISPLQDMEN
CMKCPEDQWSNESRDKCIMRAIDFLSYKDLLGVALTTTALVLSFCSAAVFCIFLKYKKSPIV
KANNQELSYILLLSLMLSFLCSLLFIGRPTKVTCLLRQAVFGIIFAICISSILGKTITVIIAFTAT
RPGSRLRNYVGTRVPKYILLLCTLPEVFICALWLIISPPFPDYDTHSATGKIILQCNEGSPSAF
YIMVGYIALLAFVSFFVAYLARKLPDIFNEAQYITFSMLLFCSVWISFILAYVSTTGKYLAA
VEIFAILVSSAGLLGLIFIPKCYIIFFKPSVSTRGHLM*
>jgi|Xentr4|346712|e_gw1.190.276.1
MDLPLITIRLCHLLLILLWYKDGGCETLLEGCRLVTEEVSGYSLPGDITLGGLFAVHVEVTY
PVITYRERPQPLLCRRFHIRYYRLLLAMEFAIMEINASNDLLRNITLGFHLYDSCYNEIRSLI
GATWILSGAKNGVPNFHCNKDLMPLAIVGDMPSKASEPLARILGLYRYPQISYASGLPLLS

NKMYFPSFFRTIHNGDNEYFAIAQLVKYFNWTWVGVIYSDNDVGILGAQIVTREIEKNGG
CIAFQETLPIINSMESVYRIIGLVKRSRATVIILFCTIENIVPLMEQASFHNITDKVWVATSGW
SITSTFSRKFFPNLLILYMNFLVINHEITLSILTFSDLPCGWDRWCISQKLSFSF*GTTPGEWV
WKGGLHMALTFFLILPP*LAFTLIPLKRNPRPLLKTSLNVCKSCRHTKSITLLHYLRKVDFN
NTAGKRIYFDKNGDVPQSVDILNWQLFPNGSNQYVSIGSFDSGSLNGEGLSIQLNKILWNG
GHSQVPSSVCSDPCPKGYRRAAIQGQKICCFDCLPCSEGEILNPYDGSECLKCPEDKWPDS
RKEECLPKLIQFLSYEETLGSALACISVLFCLLTFSVFCLFIIKRKTPIVKANNRELSYLLLISL
MFGFMCSLAFIGRPNRIMCMIRQVMFAVIFSLCVSTILAKTITVVMIFSATNPDSKLKKLVG
LRIPIYIVPVCTMVQIILCIVWLTTEAPFAEFNMAAEIGIIVIECNEGSRVLFASVLGYMGLLA
SVSLFVAFLARKLPDTFNETKFITFSMLVFASVWVTFIPAYLSTKGKQTVAVEIFAILSSSAG
CLFCIFSPKCYTILLHPEMNSKENITGRNTRNQGKLF*
>jgi|Xentr4|346744|e_gw1.190.438.1
MNQTNQTTVKEFIFLAFSNFHQFQIFLFIIIQLAYIVCLIENISVIILVRVKPSLHTPMYYFISTL
SALEICFASAIIPKLLANLIAADNTISFAGCFAQLFVSDSLGATECFLLAVMAFDRDFAINNPL
YYKVIMTQNTSFGLAALPWALGFITVLIPTIYTARLQFSGPNEINHFYCHLAPIQDLICSNQF
TSKIITNSAAIFAIFIPFIVILGFYTHIIDAILKIKGTQNKHKAFSTCSAHLIVVNLIYFSALFVY
LDPKDGHYRKFFALLYTVVTPILNPLIYTFRNKEVKAAFWKLSAVHMGF*
>jgi|Xentr4|346568|e_gw1.190.493.1
MLCRLLLILLWYKDGGCETLLEGCRLVTEEVSGYSLPGDITLGGLFSIHVEVTHFEKTYRE
YPQPLQCSSFQIRFYRFLLAMVFAIMEINESNNLLPNITLGFHLYDSCYNEVRSLIGATWILS
GKKNGVSNFHCNKDLMPLAIVGDLPSKASEPLARILGLYRYPQISYGSVLPLLSNKIQFPSF
FRTIYNADYEYFAIAHVVKYFNWTWVGIICSNIELRILGAQIVTREIEKNGGCIAFKEILPIIN
SMESVYRIIGLVKRSRATAIILFCSIEDLVPLME*ASFHNITDKVWLEMAGWSITSDFPRTDIL
TTLNGSLALSPQKGKIPGFKEFLYSIHPSRFPDDPYMKTFWENVFHCIWPGNDAVNNTSPA
LLKEDIVWCTGEERLDSIDPNIYDVYNFIYSYRTHNAVFAVAHALHQMKNCVPGKGPFKN
GSCADIYNHRPWQLLHYIRNIDFRNTAGKRIYFDENGDVPQSVDILNWQLFPNGSNQYVSI
GSFDSSSLNGEGLSIQLNKILWNGGHSQVPSSVCSDPCPKGYRRAAIQGQKICCFDCLPCSE
GEILNPNDDSECLKCPEDKWPDSRKEECLPKLIQFLSYEETLGSILACISVLFCLLTFSVFCL
FIIKRKTPIVKANNRDLSYLLLISLMFGFMCSLAFIGRPNRIMCMIRQVMFAVIFSLCVSTIL
AKTITVIMIFSATNPDSKLKKLVGLRIPIYIVPVCTMVQIILCIVWLTTDAPFAEFNMAAEIGII
VIECNEGSRVLFASVLGSLSFR*FICCLSG*EAS*HI**D*VHYIQYVGICQCLGDIYTCLSQY
QGETDSGSRNLCHSLFKCWVSFLHLFPKMLYNIIAPRNEQQTVHHREKY*KPRDTI
>jgi|Xentr4|347160|e_gw1.193.6.1
MEHLTECQEVTDQLNVSQTNYSFDGRSCITSLDSYGQEDVLSFSKIILTVILALITLATLLSN
AFVITTVYQTRKLHTPANYLIASLAFTDLLVSILVLPIAIVYTVTGKWTLGQIICDMWLSSDI
TCCTASILHLCVIALDRYWAITDAVEYTKKRTPKRAVVMIALVWVFSISISMPPLFWRQSKA
EEIAVCAVNTDHILYTVYSTVGAFYIPTLLLIALYGRIYVEARSRILKQSPKTTGKRLTKAHL
ITDSPGSSSVSSTNSRTLEMPSDAGSPVYLNQVKVKVSDALLEKKKIMAARERKATKTLG
VILGAYIVCWLPFFIISLVMPICKDACWFHPAIFDFFNWLGYLNSLINPIIYTMSNEDFKQAF
HKLINRFKCSS*
>jgi|Xentr4|347711|e_gw1.196.86.1
AGGVCIAQSLKIPYERTTSDFDKIIKQLLETSSARAVVLFAVDDDDIRRNCAAATDAGHCG
TLELSPTQENEDVAEGAITILPKRATIEGFDAYFTSRTLENNRRNVWFAEFWEENFNCKLTI
TGSKKEDTDRKCTGQERIGQDSLYEQDERVQFVVDAVYAMAHALHNMNKDLCPGSTGIC

PEMEHAGGKKLLKYIRNVSFNGDAGTPVMFNKNGDALGRYDLFQYQITNGSTQGYRLIG
QWTDELQLNIEDMQWAKGPREIPPSVCSLPCMMGERKKMVKGMPCCWNCELCDGYKY
LRDEFTCNLCDYNMRPNKNRTGCDSIPIIKLEWHSPWAVIPVFLAMLGIIATIFVMATFIRYN
DTPIVRASGRELSYVLLTGIFLCYIITFLMIAKPDVAVCSFRRIFLGLGMCISYAALLTKTNRI
YRIFEQGKKSVTAPRLISPTSQLAITSSLISVQLLGVLIWFGVTPPSIIIDYGQHTVTPEQARG
VLKCDITDLQIICSLGYSILLMVTCTVYAIKTRGVPENFNEAKPIGFTMYTTCIVWLAFIPIF
FGTSQSAEKLYIQTTTLTISMNLSASVALGMLYMPKVYIIIFHPELNVQKRKRSFKAVVTAA
TMSTRLSHKPSDRPNGEAKTELCENVNPNSEYE*
>jgi|Xentr4|348032|e_gw1.198.327.1
MLGGTTASHHIKVLIYLSVLCVGPCSSVALPINPACQLKSADTLEEYEYIQEGDILIGGVMT
LNLYGYNTVKMYNHIGCKMLTPKSYRHLVDFLFVIEETNKNRGHLSNLTLGFHISDSCGN
EMKAVRSVLQILSGTREPIPNYSCAGKSNIAGFIGDLNSGTTVPIAQILSVLGYSQISYGATD
PLLSDRVAFPFLFRMVQNNQHHFFALSKSLKYFSWTWVGILTSDDDNGEREHKLLTRYLSS
DGICVEFTIKFPKYNIVLSDRLMFGQTIDKSTAKVIVLCGTVDITMAMQLSSLLIELSEKTF
VLTSIWASYSDTLELTDDLFHGSLIFVPHFLDPGNMYKLQFKQFAADRHPSKYPEDVFLKK
IWTDACRKGSNKRHLPDWLNNCLGKQRLTDLEGFNDTFHPPGVYLAALTMAQGLLINRS
KEKHERGYSYKHHLRHYLKRVTLRDTEDQMYYFDENGEFLTQYGITNLFYNHYYSSSMS
QTQVGKYTPWAPSDHRLNINTELIRWKSPDNKMPRSQCSESCLPGYRKAPAPSIHTCCYN
CIQCSEGEISSKIDSENCFSCSSMEWPNKKNTRCIPKKEDFLSYTTDVISIVLSSISVLFLLITF
LILGVFIKYRDTPIVRANNRSLSFLLLVSIKLSFLSVFLFLGRPGDITCRLLNITYGIAFSIAVS
SLLAKTIMVYIAFKSTKPGSSWGKWMGVKLSKSVVLVFSSIQIIICITWLAISPPFQELDIHT
YPGTIIIQCNEGSALGFYSVIGYMGLLAAVSFVLAFLARSLPDSFNEAKYITFSMLLFCSVW
ITMIPAYLSTKGKNTVCVEIFAIVTSSAGLLGCIFVTKCYIILFRPKMNVRSQLLGNKTR*
>jgi|Xentr4|348194|e_gw1.198.329.1
MLGGTTASHHIKVLIYLSVLCVGPCSSVALPINPACQLKSAETDIEYEYMQEGDILIGGVMT
LNMYSLYTQNNSRIFSCQMVTAKSYRHLVDFLFVIEEINKNRGHLSNLTLGFHISDSCGNE
MKAVRSVLQILSGTREPVPNYSCAGKRNIAGFIGDLNSGTTVPIAQILSVLGYSQISYGATD
PLLSDRVAFPYFFRTVQSVHHHYFALSKLLKYFGWTWVGIVISDDDAGERDHRLLSRYFSS
DGICVAFTVVFNIKIFDTKSMDKLRQQIVNLPAKLIVLCGTVDQTMVIGLAKVLFMGHDIT
FVLTTLWPSYRDYFKLASYVFHGSLIFAPHFLDPANMYKLKFKQFAADRYPSKYPEDVFLE
KMWKSKCIINVHGFCSIKRLIDVNGFNDTFHPPGVYIAALTMTHGLRLLLLNQSNEKNGK
GHSYKHQLHHYLKRVTLTDTDNLSSYFDKNGEFVTHYGINNLIKLPRSLSQTQVGKYIPW
APPDQRLNITTEAIRWNTLDNKLPVSRCSKSCLPGYRKAPAPSFHTCCYSCIQCSEGEISSK
TDSENCFRCPDLEFPNKRRNQCIPKKEDFLSYTTDVISIVLSSISVLFLLITFLILGVFIKYRDS
PIVRANNRSLSFLLLVSIKLSFLSVFLFLGRPGDITCRLRNITYGIAFSIAVSSLLAKTIMVYIA
FKSTKPGSSWGKWMGVKLSKSVVLVFSSIQIIICITWLAISPPFQELDIHTYPGTIIIQCNEGS
ALGFYSVIGYMGLLAAVSFVLAFLARSLPDSFNEAKYITFSMLLFCSVWITMIPAYLSTKG
KNTVCVEIFAIVTSSAGLLGCIFLTKCYIILFRPKMNIRSQLFGNKTR*
>jgi|Xentr4|348263|e_gw1.198.228.1
MFHSYFKTLTSQCSANCSPGFRKVSRKSSPPCCYDCAPCSEGDISNITDMENCLTCQDNKW
PNHEKTMCIEKQVEFISFDTDYFAIFFTINSLILFTVGAVILGIFISFRDTPVVKANNRNLSFIL
IVSIKLSILSVFLFLGRPTDIICTLRQSSFGITFSIAVSCVLAKTIMVYIVFKATKPGRSWSKW
VGVKLAHCIVLVCSVIQILITSLWLSISPPFVEYNILSEPGKIIIQCNEGSVVAFYIVLSYMGLL
ASMSFIVAFLARSLPDSFNEAKYITFSMLLFCSVWITMIPAYLSTKGKPMVAVEIFAIITSSCG

LLFCIFLPKCYIILLKPEWNTKHCLLGNPNKSRFVYT*
>jgi|Xentr4|348569|e_gw1.200.82.1
MNDSDFFWNQQNTNYCFQDTNNSCPKSNKELSSFSALLLFMLGAIIFTTVGNLMVIISVSH
FKQLQTPTNVLVMSLAIADFLLGLLVMPYSMVRSLTSCWYFGEVFCKLHSCIDMMLSTTSI
FHLFFISVDRYYAVCQPLHYYKNITTSVIEVFVFISWCLPCIYSFGLFFSNVDTEGTQDIGIFC
TGSCFILLDKLWGTISSLISFYIPGNFIIGIYIYIFSVAKKQAKIVHHYPSTQDQKPNSRIKLSL
TIETKAAKTLSIVMGTFLLCWLPFSVVALFDPYFNFASANGIYDIVLWLGYINSTLNPMIYA
FFYPWFRKCFVLIMKGNIFNADSSSFHVLSNS*
>jgi|Xentr4|348755|e_gw1.202.55.1
MNLTQEHLFCLELMANRNINIILWHYNLTGRLSGRSSKGIGIFHLLGILISVLIILENLSVLLA
LLRDPRMRRWVHCCLGNIALSDLLAGISYLLNLFLSGPTTFRLSPALWFLREGLLFTTLAA
STFSLLVTAVERYCTMVILVTENNSVKSMRVQGVIVLCWVLAGVVGFLPLLGWNCLCKIE
SCSSLLPLYSRTYLFFSIILLGVTLLGIIAAYCTIYYLVCTSGRRVAETNSSRRSFHLLKTVLII
LSAFVICWSPLFFFLIVDFTCSPPSCKSPLGLEWVLAVAVLNSAINPLIYSFRSSEMRRAVLEL
FCCVCIRAGVKPPACCQLGTEITSGSSNEGSYKNRSSVRLSRALSVRSPLTSISSAPSQ*
>jgi|Xentr4|349818|e_gw1.210.38.1
VLYSVVFIVGTLGNGLVIFMITFKLKKSVNVIWLLNLAIADFLFTFFLPFTIIYTAMDHHWIF
GRTMCKLNSFVLVQNMFTSIFLLTVISLDRCISVIFPVWSQNHRSAKMAKGLSAAAWIVAF
FLSAISFIVRDTTYRQEKTICFTNFSLMGENNRAKLQTAYTLTRFAFGYLIPLIIILCSYIIIVY
RLKRNRMAKSRKPFKIIFSIITAFFLCWTPFHVLHIMEIEAIRFPAYIFKIGVPIASSLATANSCI
NPILYVILGQDFKKFKMSILSRLDNALSEDTTNSRLSHRTFSRVSTMNEKESVML*
>jgi|Xentr4|349898|e_gw1.211.137.1
MIKLYNELRRNETEEAINGTGESTGLANKNPSSLPLSVFSLLICFTGLVGNGALFILLRFKV
KRNQFILYVINLTVADFIYLVGLSIWMLYMFCSLNGLKSSKIVMKHLAQISDLFYNFGFNTG
IYLLTVIGLERCFAVLYPLWYQCHRPKNLALYISTALWLLSALVTGLELFICDGEKHYLLQG
SENCTNVYFFTSALYVIVVLIMLWSSVTLLLDIDKAPQHCHSPKLYIIIIASITIFLVSVVPSRI
LGLLLYFNILSEKKYLVSIFFITSISSAINCSANPYIYIVVSKWGGKKSLKRSLKYMLENIFKE
PTESQTMATTPM*
>jgi|Xentr4|349955|e_gw1.211.138.1
MASNDTNTTQFNDTNNFDKSHAPNSVIHYSITAAVAMGLCLIGIVGNIIVFWYLFFRIPRTK
YTVVYIINLAAADLLLLTFAAILMMVNINTLVGSNPDFEGIVVFYTFLQSIYDLSLYSGMYFL
TAISMERCISVLFPLWYRSHRPKTLSVTMCICLWILGCSESLTVNLACPDNDFRNQTEVCTG
VQIMRFTLSICICLPLMILSSVTLLIAIKKTFRNRYPPRLYIIIIAAVFIFILSVIPFNFLWFLMYF
RLLPSIQDTLGLYFVSILTAALSSTANPYIYFIVGRLWKQKSSQSIRDVLHRAFKAQEDEKEE
IKCEKENTSSSNNSNARNSTSSDSHRTDNSNYTNTENIL*
>jgi|Xentr4|349988|e_gw1.211.136.1
MGLCLFGLVGNIIVFWYLFFRIPRNKYTVVYIINLAAADLLLLTFAAILMMVNINTLVGSNPD
FEGSAGFYTFLEIIYDLSLYSGMYFLTAIIIERCISELFPIWCHNYRPKTLSVTMCICLWILGCS
ESLIENLLCTQEDFSNQTPVCTAVEIMTFTLSIFICLPLMILSSFTLLIAIMWITRNRYPPRLYII
VIAAVCIFILSVIPFNFLWFLMYLRLLSSIQDTLGLYFASILTAALSSTCKPFMYFFVGRLWK
QKSSNSIYDTLHRAFEAEDDEKEEIKCEK*
>jgi|Xentr4|348594|e_gw1.201.2.1
ICTMTTAFLHFFFLASFCWVLTEAWQSYMAVTGKIRTRVIRKRFLCLGWGLPALVVAISMG
FTKAKGYGTPHYCWLSLEGGLLYAFVGPAAVVLVNMVIGILVFNKLVSRDGILDKKLKH

RAGQMSEPHSGLTLKCAKCGVVSTTALSATTASNAMASLWSSCVVLPLLALTWMSAVLA
MTDKRSILFQILFAVFDSLQGFVIVMVHCILRREVQDAFRCRLRNCQDPINADSSSSFPNGH
AQIMTDFEKDVDIACRSVLHKDIGPCRAATITGTLSRISLNDEEDEKVVKPEGMSYSTLPG
NIISKVIIQQPSGLHMPMSMGDITDQCLKKENSDLRRTVYLCTDDTLRAGDTDMGHSQDR
MMESDYIVMPRGTGNMQMKDENKMNIAMDTLPHERLLHYKVSPEFSMNPAVMEQFGIN
LDQHLVSQEHMQSLSFEPRTAVKNFIASELDDSAGLSRSETGSTISMSSLERQSARVCFITEE
KKVMHTRKRHMELFQELNQKFQTLDRFRDIPNTGSLDNPSVNKNPWDSFKNTEYQHYTT
INVLDSEAKDTLELRPSEWEKCLNLPLDVQEGDFQTEV*
>jgi|Xentr4|350422|e_gw1.216.5.1
NLSLSFSTQFIRHFLTFDYAVNEINMNMAILPNISLGYDIFDTCSMDFGAVVTTLQGLSGVN
PLYPNYNCRQQGKAIAFIGDLYSSSSLIISNILQVYHYPQVSYGATDTIFNDKRRFPYFFRTI
HTHQDQNEAIAMLLIHFGWTWVGIITVAEDSYERSSEDLRDRIVASGNCVAYVLKLSLGK
KKDMLYYIKEMDMIVKSLAKVIIVNFPTEYTLYLSSFVAQVLGPMIREKVLILIDCLPAAAF
PDPEFKLPPISACLLLRPSKGAIPGLKEFLYRVTPDTYPNYILKNTWQRLFKCSVPDNQFED
NVTCSGYEYFRTLPAFHFDVDNFRLTYNVYIAVYALAHALHQLLLDHFQKQPFSASDLHE
YLKSVKFITSAGDEILFDKEGNRPAQVDLLNWVQHPNETVAVTEVGHYRMSASGTHQFII
NDSDITWETKINGVPQSTCSESCSPGYRKVIPDGNPSCCYDCIPCAEGEISNVTNMETCMS
CPEEEWANSKKDTCVRKNIDFLSSQDALGATLVAFALLLIFMILSVLVIFIIYRDTPIVRANN
RDLSYTLLICLMLSFLCTFSFIGHPTTAICLLRQVAFSLIFTVAISSIVAKTITVLIAFRSTKPGS
KLSKWTGKKLSAFIIILCTLVELGICTWSLVQFPPFPNRDTKTYTDRIILQCIATSSMGFYTT
HGYILTLSLFNFALAFLVRKLPDRFNEAHYITFSMLLFCSVWISFIPAYLSAKGKYMVAVEV
FAILASSAGLFCCIFIPKCCTILLKPHLNSRIKLSVYK*
>jgi|Xentr4|352604|e_gw1.232.86.1
QTYRYLVEFLFIVKEMNDNPAQLPNVTLGYHIHDSCGDTQGALISLLQILSGTREPVPNYS
CVGKRNIAGFIGDLQSEPTISMAHVLGVLGYSQISYGATDPVLSDRSIFPYFFRTVLSDEEE
YIVLCKVLKHLGWTWVGIVQINNDSGYRDYQLLTKHLPKEGICVEFVVKMMGDLKSFNI
VKKTSTGVIIICGEINFDHFGRFINSYSLFRKRTCIFLSKWLNHIDALQYSSGVFTGTVSLMH
TRLNGHNDGKFREFSDTFHPSKYPEDKLLENIWLEVFSCFSKDESKNSFQEAYNYNFLHN
CSGEERLTDIPNYLGAYHSGSLVQAVDMLALALEDMQDFLTKQTSEKHRWMYHYNHQL
HPYLRNVFHATNRGQESSSNEKVEFPSQYDIVNPRLGPGDNWTWNVVGHYTPWASVEQR
LTLKIEKIIWNTKNNEIPRAQCTASCPAGFRKAIQPGHLTCCYGCVLCPEGEISNRTDSENCI
RCPDMEWPNRKSTQCTARTEEFLSYTKIISVIFSSVSVFFFLITLLILGVFISYRDSPIVRANN
RSLSFLLLVSIKLSFLSVFLFLGRPVDITCMLRIITFGITFSIAVSSLLAKTIMVCVAFKATKPG
SSWRKWLGVKLSNSVVLFCSSIQIIICMTWLAISPPFQELDIHTSPGTIIIQCNEGSAIGFYSVI
GYMGLLAAVSFVLAFLARSLPDSFNEAKYITFSMLLFCSVWITMIPAYLSTKGKNTVCVEIF
AILTSSAGLLACIFLPKCYIIMFRPEINTKTHLFGNIS*
>jgi|Xentr4|352819|e_gw1.234.47.1
TVEYGTRRQDAAGQLDAPAQVLYTIGSFILIIGFVGIIGNLLVLYAFYSNKKLRTAPNYFIMN
LAISDFLMSATQAPVCFLSSLHREWILGEIGCNVYAFCGALFGITSMMTLLAISVNRYIVIT
KPLQSIQWSSKKRTLQVIVLVWMYSFMWSLAPLLGWSSYVPEGLRISCTWDYVTSTTSNK
SYTMMLCCCVFFIPLIIISHCYFFMFLAIRSTGRQRSVQKEQQVEARGFTCSSGGKDRACLC
LPMLRSSCARSSMSRMQRFTGVSFFRHGKSLTPYSKTVPAVIAKASAIYNPIIYGIIHPKYRF
TEHKSHIRKEPKKDCFESSVRGSIYSRQSLVARKKNSCISTVSTAETVSSNVWDDTSNGHC
RKSLSQTLSNLCSPLFQDPNSSHKLEQPLTWPDDPSSKEILLSSSMNTVTYPIGLGSIVKDGI

SNIPNVRNHRIDKSRGLDWIINATIPRIVIIPTSESKISEGQEEHDNNTVEKSKRTEEEDDFFN
FNVDTSLLNLEGLTSSTDLYEVVERFLS*
>jgi|Xentr4|353542|e_gw1.240.124.1
MPTNVSLLATPENSTVWNPFTGPLKTIEAWNFHLLAALMFVVTSLSIAENFIVILVTAKFKQ
LRQPLNYIIVNLSVADFLVSVIGGTISIATNSRGYFYLGSWACVLEGFAVTFFGIVALWSLSV
LAFERYIVICRPLGNLRLQGKHSALAIIFVWVFSFVWTIPPTMGWSSYTTSKIGTTCEPNW
YSGEMRDHTYIITFLTTCFVFPLLVIFMSYGKLMRKLRKVSDTQGRLGSTRKPEKEVTRMV
VIMILAFLICWTPYAAFSILITAHPTIDLDPRLAAIPAFFAKTASMYNPIIYVYMNKQFRRCLY
QMFNINDPEAKESNLNPTSERGVLTRNNNGGEMLAIATHITSSAVTNREEEKSSSNSFAHIP
VSDNKVCPM*
>jgi|Xentr4|354312|e_gw1.247.114.1
VSSSLLFAAGFLGNLIALFILWLHKLHAKKTSVFYVLVTVLTVTNLMGKCLLCPVVQVAYF
QNQSLVGMTGNLNLCKVFGFLMIFCGLAPTFILLAMAVDCWLALGYPFFYQENINKKLAL
LVSLILYVFCLGFCCLPFFGFGRYKQYCPGTWCFIQMTAEESSTSALAYSMLYGTIMGLLIL
AIVFCNLMIMKNLYQMYKRENEKGIPSANFPNQQEPAGMEELEHLILLAIMTVLFAACSFP
LTARVYVGAFKGEKNEYADLTVLRLLSLNSTLDPWIFIICTTSKFRGHLKALCSKIQLLSISN
PFPLLDATKCDL*
>jgi|Xentr4|354425|e_gw1.248.123.1
MVGTQYPISLIPSNKNTNSTSAPLENEKNVSMIVIQFIYAIVCLIGLIGNSMVIFVILRYAKM
KTATNIYILNLAIADELFMLSVPFLAASAALQHWPFGSGMCRTVLSVDGINMFTSVFCLTV
LSVDRYVAVVHPLRAARYRRPTVAKMINICVWIVSLLVISPILIFADTMPSKNGVVVCNLM
WPHQTWSAVFVIYTFLLGFFLPVVAICLCYILIIIKMRAVALKAGWQQRKKSEKKITRMVL
MVVTVFVICWMPFYIVQLLNLFLPHMDATINHISLILSYANSCANPILYGFFSDNFKRSFQRI
VCFRWLENGTDEPVDYYATALKSRVCNNNPLDFQQEPLQSDPCYKHGTITRTTTL*
>jgi|Xentr4|355010|e_gw1.254.56.1
DYLTGIISISVNIIWRFTGEFMAPEIVCKTVRYLQVVLLYASTYVLVSLSIDRYHAIVHPMKF
LQGEKQAKVLIAVSWTLSFLFSIPTFIIFGKLKLPNGEMQCWALWPDDSYWTPYMTIVALL
VYFIPLIIISVIYFIVIRTIWVKSKGHAVIISNYTDGNFCTSYSHRGLISKAKMKAIKYSIVVIL
AFILCWSPYFLFDILDNFEMLPETKERFYASVIIQHLPFLNSAINPIIYCVFSNRHCRLSRDRN
SGKLGGTIRDKTEGIEMQVVSRPEYL*
>jgi|Xentr4|355245|e_gw1.255.102.1
MRGGTWEGSMMDATCALTFPCLLSGSTGLVYRVCSEEGVWQTAENSTSIWRDHGECSGK
KSLQTEYYASLSALQIIYTIGYSISLGALLLALVILLLFRKLHCTRNYIHMNLFASFIMRALA
VLIKDIVYKNTYFKKNDEMGWMSHLTSEISTSCRVAQVFMHFFVGANYCWLFVEGLYLH
TILVTVILSEKGLLLKYLFIGWFFPLLFVVPWVIAKLYYENNGCWGVNESPGIWWIIRGPM
LLGILINFLIFIKVLKLLYSKLKAQQMRYTDCKYRLARATLALIPLLGMHHVVFTFITDELV
EGATRHFWLLIQLAFESFQGFVVAIFYCFTNGEVSISIIT*
>jgi|Xentr4|355502|e_gw1.256.18.1
MASGSLPSKKLEPRRCDEVINRHYNYTGKLKERELDHMDMTEIVFLIICSLIVLENLMVLI
AIWKNNRFHNRMYFFIGNLALCDMLAGIAYIVNICMSGSKTMDISLTAWFVREGSMFIAL
GASTFSLLAIAIERHLTMIKMRPYDANKKYRVFLLIGTCWLISFSLGALPILGWNCIEDLPN
CSTILPLYSKRYVGFCISIFIAILIAIVILYARIYILVKSSSRRVTNHSNSERSMALLRTVVIVVG
VFIACWSPLFILLLIDVACEVKTCSILYKAQWFIALAVLNSALNPVIYTLASKEMRRAFFRL
VCSCLVKTNNSRSLPIQPTPDQSRSKCSSSSNSPKHKSIIQTNGHSKDEKSESSYHNGNFTK*

>jgi|Xentr4|355592|e_gw1.257.139.1
MDKGNGTTVTEFILVGISEIPELQFYLFCIFFCIYIITVLGNASIILIYKLSPNLHTPMYYFLSN
FSFLEICYVSASAPKMLHNIVMGRKTITFHDCFVQMYSILFLAGTEFYVLAAMAYDRYNAI
CHPLLYRVIMNNRVCIQLLCCSWAIGAANALVHTLLAYRLPFCSNKIQHFFCDVPPLLKLSC
INTWINELGMYVIGGSVTGGSFVLTLISYIKIILTILNIQSSSGRKKAFSTCTSHLTVVAIFYGS
IFFMYLRPKSSYAMHHDRLVAVMYTIVAPLLNPFIYSLRNNDVKLAVTKLLQKRLTWKY*
>jgi|Xentr4|355619|e_gw1.257.15.1
MHARNETTVTEFILVGFSVIPRLEYLFFTLYLIIYVTTVLGNTAIIFAYSLCSDLHTPMYLYLT
NFSFFEICYVSITVPKLISDLLTERKTISFYGCAVQMYCFFLLCGLECCMLAIMAYDRYNAIC
HPLLYQIIMNRKVCVQLLAGTWIISVINSLIHTLLTFSLPFCDNVINHFFCDIPPLLKLACKV
NLMNEIALFAIAGFIIMGSCVFIVISYTQIIATILGIHSTSGRRKAFSTCSSHLTAVTIYYGSGA
FYVFKTQTKPIFGSGQTDCPYVHRYCTTAESFHIYTQK*GFQGSFYKDNTTYVFSKIL
>jgi|Xentr4|355676|e_gw1.257.16.1
MHAGNETTVTEFILVGFSVIPGLEYLFITVLLIIYVITVLGNASIIFTYSLCSELHTPMYFFLT
NFSFLEICYVSTTFPKLLSDLLREQKSISFFGCAVQMYTFFLIAGLECCMFAAMAYDRYNAI
CHPLLYQTIMNRKVCVQLLAGGLIISSTNSLIHTLLTFKLPFCDKIINHFFCDIPPLLKLACRV
NLTNIIALFIIGGFILVGSCVCIVLSYTRVIATILGIHSTSGRMKAFSTCSSHLTAVTIFYGSGIF
MYLRPKPSYEIDQDKQIALMYTIIAPLLNPFIYTLRNVDFNVAFRKIIQHVFSRKF*
>jgi|Xentr4|355698|e_gw1.257.19.1
MDSRNRSTVTEFIILGFPTLYEYGPFLFPCLLLVYLLTVSGNVLVFTIIRLDSRLQTPMYFFIS
VLAFLEIWYTAVTIPKMLINILYPRKSISFNGCLLQTYFFHSLGASECYLLTAMAYDRYLAIC
QPLHYSTIMSSKMYFRLTAICFTCGFLCPITEVALISQLPFCGSNEIQHIFCDFPPLLSLACTD
TSVNVLVDFVINSFIILVTFLFIMISYIRIIIAISRISTSVGRAKAFSTCVSHLSVVLVFFSCIVFM
YVRLTKSYSLYYDRVLAVIYSVLTPIFNPLIYSLRNRDIRIAIKYRIFKR*
>jgi|Xentr4|355724|e_gw1.258.81.1
MDSLFHIYVLVPVTILCISLFLLGITGNLLTIIVFKRYRDMRSTVNMYLSSMAVSDILIFLGLP
SDLYRIWKYKPYAFGNFVCKFLVYLSESCTYCTILHITMVSIERYIAICFPLKAKIIITKRRVK
IVIILLWIFALLTASPILFLFGVEHPPGFQPEETKECKYTEQSAQNGLLHVMTWVSTIYFFLP
MFFLTFLYGLICRKLWQTRHWARGPATANRGKYNKATVKMLAVVVVCFMLCWLPFHIGR
ILFALAGVGEYVFFEVTQYFNLLSMVLFYLSASINPMLYNIMSEKYRSAMRRMLYPKQGL
QSGRTRSYRFSLEGTELSSGM*
>jgi|Xentr4|355732|e_gw1.258.49.1
MLLEDFYNFLSIVFAVDEINRNPKILPNVTLGYNVYDSYVDLFRTVQGAVRIYSGYTKQIP
NYNCDKYSVLAAVVDGMASSFSIQYSNIFGMYKHPQISFVSQDPLQSDKLHFPNFYRAVPS
EKTLYAAILALLNHFDWTWIGIICPDDDSSINAIKEIKRMFEENGGCIEFIKVVPSINDYSSQ
RIMDITHTIKNSTAKVILVYGSKNYVYYLEQGVHITSIPGKVWIHTAESSFRMLNIVNGTYV
NGSLYLNMHKKEIPDYIKFIHEVNPSRFPSGRTFTTWWDELCENRCPFNSRRRNCTGVESG
RVIEYSHCNLRFTAMSYSAYNAVYIVAHALHDMYQQTLKERLNDTKKLRFQDLKPWMLH
HFLKKVNFTNTMGQQIYFNNDEISHLYDIYNVVYLPNRTILSENVGNFNSEAPPGKQMTIN
EKAIIWENYFSETPRSVCSEKCPPGNRKSVWEGKPSCCYDCLPCPEGEFSNKTDVDVCTK
CPENQWPNKQKTSCSPMDITFLSYSDPIGITLTVIAIICLFASFWVLGIFMKYQNTPIVKANN
RDLSYLLLISLMCSFLCCLIFIGPPEQVTCFLQQAIFGITFTISVSSLLAKTFIVVVAFNATKPG
NNLRKWVGAKIPKYIVITFSVIQVCICFLWFVISPPYSYYNKDSQPGIIIVMCNEGSVTAFYT
ILGYLCLLASVCFVVAFLARKLPDTFNDAKLITFSMLVFFSVWVFFIFTSRSAKGTSTVAVE

VFAILGSSSGLLACMFAPKCYIILIKPEQNMKKNLVRDSAAKCS*
>jgi|Xentr4|355789|e_gw1.258.87.1
MGNESNNSTCLIDDSFKYNLYGAVYSVVFIFGLITNCASLFVFCFKMRMQNETAIFMTNLA
VSDLLFVFTLPYKIFYNFNRHWPFGDSLCKISGTAFLTNIYGSMLFLTCISVDRFLAIVYPFR
SRTIRTRKNSAIVCACVWIIVLSGGISASLFSTTNVSNTSTTCFEGFSKSIWKTYLSKITIFIEV
VGFIIPLLLNLTCSSLVLRTLRKPATLCQIGTNKEKVLKMIVVHVAIFVVCFVPFNSILFLYAL
VRSQAISNCAVERFARTMYPITLCIATMNCCFDPFVYYFTSKSFQKSFNINPIIKMDTLFKID
SATKIALPVTQEELTEQMNINNGGELMSEANYKD*
>jgi|Xentr4|356357|e_gw1.264.89.1
MGRLKSGTFALLFVILLLDYFAPVRPASKAIVCQEITVPMCKGIGYNHTYMPNQFNHDTQ
DEAGMEVHQFWPLVVIQCSLDLKFFLCSMYTPICLPDYRKPLPPCRSVCERAKAGCSPLM
RKYGFAWPERMNCDRLPEHGDPDTLCMDYNWTETTTTPPPTHPPKVKTPSSDCDGVCKC
REPFLSITRESHPLYNRIKTGQVPNCAMPCFQPYFTQDEKTFATFWIGLWSILCFISTFTTVAT
FLIDMERFRYPERPIIFLSACYLFVSIGYIVRLIVGHENVACNKDHIHYETTGPALCTVVFLLI
YFFGMASSIWWVILTFTWFLAAGMKWGNEAIASYSQYFHMAAWLIPSVKSIAVLALSSVD
GDPVAGICFVGNQNLDNLRGFVLAPLVVYLFTGTMFLLAGFVSLFRIRSVIKQGGTKTDKL
EKLMIRIGIFSVLYTVPATIVVACYIYEQHYREHWEKAHNCSCPGDKKRYRPDYAVFMLKY
FMCLVVGITSGVWIWSGKTLESWKRFTGRCCRNSKPINASAYSEASRALTARTGLSSSTLH
HKQVPLSHV*
>jgi|Xentr4|356602|e_gw1.266.34.1
DLKTVLSLPQYPGEFLHPVVYACTAVMLLCLFASIITYIVHHSTIRISRKGWHMLLNFCFNT
ALTFAVFAGGINRTNYPIVCQSVGIVLHYSTLCTVLWIGVTARNIYKQVTKKPHPPPNADQP
PFPKQPMLRFYLISGGVPFIICGITAATNMKNYGNEDNTPYCWMAWEPSLGAFYGPVAFIV
LVTCIYFLCTYIQLRRHPERKYELKERTEEQQRLAGAETGHSHLAEPGSVSQATCSMISSSL
LANEHSFKAQLRAAAFTLFLFTATWTFGALAVSQEPFLDMIFSCLYGAFCVTLGLFTLIHH
CAKRDDVWHCWWSCCPSKKNAYPVPANARQKTNINGDAQVHTTCLQDSPCPNKSPMY
NRPPAGHCKLTNIQSAQNHVNCLSPVTPCCAKIHCEQLMDDESHIHVHNESAFRPNMHVH
RCLKSRTKPRYFSRHRSAAEREYAYHIPSSIDGSIHSSHTDSPHSTHEGHTGHRRGCCGASD
PYPTISQPESSDASTAIFSCGKMPDTDTVHHGAHFELHPRRQSFPFNTTNPNGILKGNMHE
AMIYSSDSTGNIKTGPWKNETTV*
>jgi|Xentr4|357311|e_gw1.273.9.1
MNIRNQTTPRPVFLLGFHVFYNLKIPLFLLILTLYCLTLIGNIIIIALVSLSPTLHHPMFFFLSH
LSLSDILLTTTIVPIMLCGILRGGVSISLAGCITQFNFFCTALASECYLLVVMSYDRYLAICNP
LRYMSIMNTKLRVQLVVLCWFLSFTISTTGALSVSSLNFCGPNIIDHFFCDFFPILQLSCSDT
SAVELEQILIAVPTVVMPVVFIVGTYICIFIAVLKIPSTSGRQKAFSTFSSHLSVVCIFFGSLICL
YVAPPSGNLLTTNKVVSVLYTVVTPLSNSIIYSLRNSELRSAFKKHVNIYNICKMSKLSGL*
>jgi|Xentr4|357347|e_gw1.273.98.1
MDSRNHTSITSVLLLGFEILHSFKIPFFLLIFLLNILTVTGNATIVALVSSSPSLRNPMFFFLSH
LSTLDFILTTNIVPNMLHGILQGEITMSFPACKIQFYFFSALLASECLLLAVMSYDRYLAICK
PLRYISMMHNKFCLWLVISCWILGFLTTLVVLILLNRLEFCGPNVIDHFFCDFSPLLQLSCSD
ISSVILGQMLLAVPMTSFPFIFIIVTYICIFIAVLRIPSTFGRQKAFSTCSSHLAVVGAFYGSLIS
LYVVPSSANSTLAKKFLSMLYTVFTPLFNPIIYCLRNKEIKVAFKKWLQAL*
>jgi|Xentr4|357348|e_gw1.273.100.1
MDGENQTTIQSILLLGFQMPSPFKIPIFILIFLLYSLTLTGNLLIIYLIYGSPTLQHPMFFFLTHL

SLSDICLTTDIVPNMLYVIKSGPISITMAACMTQFQFFGTVVTFECLLLAVMSYDRQLAICN
PLRYASIMGNQLPLWLVIVCWSVSFTVTLGTVVMLTRLDFCNLSAIDHFFCDFDPILQLSCS
DTTAVELEQMIIGSSVAVFPLGYITVTYIHIFICIFRISSKSGREKTFSTCSSHLAIVSTFFSTLIA
IYVVPSRGTSLNANKLISLFYVIITPLFNPIIYSLRNHEIRMALEKYISTIKN*
>jgi|Xentr4|357554|e_gw1.275.6.1
MSGTQPTFETPNITVGVEEDKRDPIVAQWNIAMLAVIFSFATFGNCLVLFTLLRRRKHNAL
MHTFMIHLCLADLVVSFFQVLPQLVWDITDRFRGPDVLCRGVRYLQVVGMYASSYMIVA
MTFDRHQAICRPMMTFKKGSARWNIPVSLAWLASAIFSLPQGFIFSRIEVHPGVFDCWATFI
EPWGLKAYVTWITLAVFILPALFIATCQVLIFREIHNSINMGPGHSPRPRRKAKLINTRNGA
RSQSDTGVSSAMAKTVRMTLVIVLVYVVCWAPFFIAQLWNVWNQESAAGSSAIKVLMIL
ASLNSCTNPWIYTIFSSSVSKDVKEILCFACRPRQRKNSLPEDSCFTGSTTVPKECLY*
>jgi|Xentr4|356448|e_gw1.264.121.1
GLAYRRCNINGTWDFVHRLNKTFANYTECLHFLKPERSQGKREFFERLYVMYTVGYSISF
CSLAVAIFIIGYFRRLHCTRNYIHMHLFVSFMLRAGSIFIKDKVVHTHIGVKELDAVLMGDL
MISPVLDKSQYVGCKIAVVMFIYFLATNYYWILVEGLYLHSLIFVAFFSDTKYLWGFTLIG
WGFPAVFVVIWAVARATLADARCWELSAGDIKWIYQAPILAAIGLNFILFLNTVRVLATKI
WESNAVGYDTRKQYRKLAKSTLVLILVFGVHYIVFVCLPHTFTGLAWEIRMHCELFFNSF
QGFFVSIIYCYCNGEVQTEIKKTWTRWNLAFDWKSTTHCSNFRYGSVLTNMTHSTSSQSQ
MVTSSRMVLISSKVSRSVSKQADGHINLPGYVRSNSDHDCLPQWIQEETNEDEEKQVDDI
SLKEKQRPVQMSAEVVVNRNVIEETL*
>jgi|Xentr4|357979|e_gw1.278.90.1
MMDYTKLVGVQVILIAAYSLIILLGLVGNSLVIYMIVKYKNMRTVTNFFIANLAIADLMVD
SFCLPFTLVYTLMDEWKFGSVLCHLFPYAQAMSVNVSTLTLIVIALDRYWCIVFHLNSRIS
KNLSFLIISITWITAAILAIPLAVFREFRYEDLPPFNLKIAVCAENWTNRDSTIYSLSMLILQYA
LPLAVICYAYLRIWFKLKNHISPTTRSESQQRRKNTTKMLVMMVVVFAVCWLPFHIFQLAI
DLEWTVAIHENKLLYTIFHVVAMCSTFVNPFLYGWMNKNYRHGFLTFFGCKDTLQNSQPD
GSLRGHSYTFRPTTFHGSIKHACENGNPPTHV*
>jgi|Xentr4|359057|e_gw1.284.68.1
MLSNVSTGEVKCARSGMHNYIFITIPIIYSTIFVVGVFGNSLVVIVIYSYMKMKTVASIFLM
NLALSDLCFVITLPLWAAYTAMHYHWPFGDFLCKLASTAITLNLYTTVFLLTCLSIDRYTAI
VHPMKSRIRRTVMVARLTCVGIWVVAFLASLPSIIYRQIYIFPGTNQTVCALVYQSGQIYFTI
GMSLVKNIVGFFIPFLIILTSYTLIGKTLKEVCRAQRARNDDIFKMIVAVVLLFFFCWIPHQV
FTFLDVLIQMDVIQNCKIYDIVDTGMPITICIAYFNSCLNPFLYGFFGKNFRKHFLQLIKYLP
PKMRSHASVSTKMSTASQRLSDTKYASNKISLQMTDKEEHCK*
>jgi|Xentr4|359179|e_gw1.285.82.1
MPTLNPSYSVGVPNTTASVAVPEKCWLALLTIMVIVPTIGGNILVILAISLEKKLQNATNYFL
MSLAVADLLVGIFVMPIALINILFQQVWQLPQCLCAIWLFLDVLFSTASIMHLCAISLDRYIA
IKKPIQASQYNSRGKTLIKIAVVWVISAGIAVPIPIQGLLYPNTTFSASYTCVVGLEPFKYFII
YGSMAAFFVPFGIMVVIYFLTIHLLRKKAYLIKNKPPQRLTWSTVSTVFQRDMTPGSSPEKI
VMLEGPRKDGTFNFTGEELPIRRLSSVGKKSMQTITNEQRASKVLGIVFFLFVFMWCPFFI
TNVTSVLCGKDQCDEDVIKMLMDIFVWVGYISSGVNPLVYTLFNKTFRDAFRRYIKCNFH
GMQSVKLLRNCSSRISFRNSMAENSKLIMKHGMRNGINPVMYQSPLRLCNGQLESSAILL
DTLLLTENEAGKTEEQVSYV*
>jgi|Xentr4|359655|e_gw1.289.67.1

MSDLEKYGDPPRPPQLVDAWLVPLFFAMLMLVGLTGNSLVIYVISKHKQMRTVTNFYIAN
LAATDIIFLVSCVPFTAALYPLPGWIFGEFMCKFVNYIQQVSVQATCVTLTAMSVDRWYVT
VYPLQSLRQRTPRVAAAVSVGIWIGSFIVSIPVPIYNQLIEGYWFGPQIYCSESFPSVSHEKAF
ILYNFLVVYLLPLITICICYSAMVYQMGRPSVEPVDNSYQVQLLAERSELMRTKISRMVAVI
VLLFTICWGPIQLYILFQAFSPSFKRDYYTYKVKIWANCMSYTNSSINPIVYAFMGANFRK
AFNKVFPFLFKQRVGSTGVANANANTEMHFVSSGT*
>jgi|Xentr4|359757|e_gw1.290.234.1
MDLENKTTDKAILLLGFQILYKYKIIFFLLIFILYSLTLIVNVMIVALVSSSPCLHHPMYFFLS
NLSVSDLILTTTIVPNMLYGLLRRGVTMSIAACITQFQFFGVAIMSECLLLAVMSYDRYLAI
CNPLQYVPLMDNRHRFQLVIFCWSVSISVSFVMRILISRLDFCGPNIVYHFFCDFAPILQISC
SETFSLELVEKFLAFPISVLPFTFISVTYVRIIMSILKIQSSSGRQKAFSTCSSHLAVVGTYFAS
LITLYVVPSTGNTLNANKILSLLYTVITPLFNPIIYSLRNLEMRAALGKCIGVRYSD*
>jgi|Xentr4|359768|e_gw1.290.205.1
MITGGENKTMNIEFLLLGFQILHSLRIQFFLAILILYIFTISGNAMVAALVLSSHSLQQPMFLF
LGHLSLCDITLTTTIVPVLLHGLLRGPVNLSVAACLTQFQIFFAGVASECFLLGVMSIDRYVA
ICNPLQYSAIMRKELSVELVTLCWFLGYSFAFNLTVLISRLTFCGPNVIDHFFCDFLPILQLS
CSDTFAVILAQLFLAILTGVFTVMSIIVTYFNILVTVLKIPSTTGRQKALSTCSSHLAVVATFL
GSLIGLYLLPSSGKTCTANKILSLLYTVVTPLFNPIIYSLRNWEIRAAFKKIVWKIRLKGFDFI
WL*
>jgi|Xentr4|359784|e_gw1.290.167.1
LMVFINVSYLLLFSWSILGNFLIIVFVLTSNFRHLPMYFFLTHLSFCDIIFITNIVPNMLHVILK
EGSWLSLPGCVSQYHIFGLSASTESFLLTVMSYDRYLAICQPLRYSAIMDMRLQLCLVLCS
WLLSLMVTVLSLILICQLDFCGPGVIDHFFCDFTPLIELSCSDTSYVQIKINVASLPIVLVPFT
FIIWTYVCIFITITGISSITGRQKTFSTCSSHESVVSIYYVTLFAVYVVPKRQSSMNINKILSLL
YTVVTPLFNPIIYSLRNQDIREALKKQVCKLHLSFQSKAAALAE*
>jgi|Xentr4|359805|e_gw1.290.3.1
YQHYLAAIFAIEEINQNANILPNLTLGYRIYDACTSESRAAANTMSILTGRAASVPNYSCND
KGTLAAFVGHLLSTLTHVVSDMTTIYRFPQISYGAQDPALGDRQQYPTLYRTIISEKYHFVT
ISQILKLFGWSWVGIICSDDESHQSAVNKIKFEITKSGGCVEFVAVYTTENVWIKTKFLEKI
KSSSANVIIVSSDSHMLLHILLSIHTDIELKKFLIFSSSLENMSNFGDSSLNGSLIISGNQGAIP
GFREFVYTAHPTKFPNDEITALLWFILFGCLKFADNSSGIYIPACTMNDTLGMTGTSYFAIT
NFYITYNLYIAVYGLAHSLHEVLYGEAYQKQLYEPKLHLNVCKLNNHLKSIHFQTASGDEI
FFNEHGEVQGNLDIENWISYPNETTAIRKVGTFNSSAPPGEQLLINKPDIIWGTPLNDIPQSV
CIGICQAGYRKAEKKGRPACCYECVQCSDGEIANVSDMENCIKCPEDEWSNENRNKCIKK
TIDFLSYAEPLGLSLAILAILLSITASLVLGIVIKNRHSQIVKANNQELSYSLLLTLILSFLCTL
LFLGKPMKVTCMFRQVTFAVIFTTSISSVLGKTVTVLIAFHAITPGSMLKIWVGKRVPRCLV
LLLSLGEVIICIMWFLHCPPFPDYDTRSQPSVMTLQCNEGSAVAFYIAVGYIGMLASVSFITA
YLARGLPDIFNEASHITFSMLVFCCVWIAFIPTYLSSKGKYMVAVEIFAILSSSSGLLGCIFIP
KCYIILLKPKKRVKEKATSKKK*
>jgi|Xentr4|359808|e_gw1.290.272.1
YQHYLAAIFAIEEINQNANILPNLTLGYRIYDACTSESRAAANTLSILTGRAASVPNYSCND
KGTLAAFVGHLLPTLTHVVSDMTTIYRFPQVSYGAQDPALDDRQQYPTFYRTIINEKYHFV
TINQILKLFGWTWVGIIYSDDESHQRAANKISSEITTSGGCVEFMALYTTENLLTISITIDKIR
RSSANVIIISSNSHMLLHIVLTIEMTTTLKKHLLFSSSLENMISFAYSSLNGSLIISGNQGAIPG

FKEFFYTAHPTKFPNDEITALLWLALFCCLKYGDNSSGIYIPACTMNDTLSFFGMSFYAVIN
LHITYNMYIAVYALAHALHKGRQSMAHRQYREGGYGTHRQHRLNNHLKSIHFQTSSGDE
IFFNEHGEVQGNLEIENWISYPNETTAIRKVGTFNSSAPPGEQLLINKPDIIWGTPLNDIPQS
VCIGTCQPGYRKAGIKGLPACCYECVQCSDGEIANVSDMENCIRCQEDEWSNEKKNKCIK
KTIDFLSYAEPLGLSLAMLAILLSITASIVLGIFIRNRQSQIVKANNQELSYSLLLTIILSFLCTL
LFIGKPKKVTCLLRQITFAVIFTISISSVLVKTMTVLIAFHAITPGSMLKILVRKSVSRCFILVF
SLGEVIICIMWFLHCPPFPDYDTRSSVMTLQCNEGSAVAFYIAVGYIGMLALVSFITAYLAR
GLPDIFNEASHITFSMLVFCCVWITFIPAYISSKGKYMVAVEIFAILSSSSGLLGFIFLPKCYIIL
LKPEMNVKGKGTSKKK*
>jgi|Xentr4|359816|e_gw1.290.33.1
MYAIEEINNSTELLPNITLGYHIYDACTSEAIALMSTLSLLSEEETPALNYICQPDQKLVAFV
GHLLSSTTYTIAEITQLYGYPQISYGALDPVFNDRAHFPSVYRTVPNEYSQFRVIIKLLNHFG
WTWVGIIASDDESNRQASEELRKEMGRNGICVDFLKAISSSSDILEKSAIEAIETIKRSSVRV
IILYCKTHYLMAFLFAQTSYQMSERVFICSVALNIVTELNFNKFLYSMNGSFLISLPKENIQG
LNNFFSHRLMTDMNKNIFVNRFFGFSTKCAYGQSDDCQWLLLRSGLCPNPGILPHITIPTV
TGLVCGVSAIPCGFIILHPVYGLDYLFGCKTAAFISLQLNYYLKNIHLKMASGEDFFFTKDG
NIPGKFDILNLIIHKNGAVNRIHVGRFLPDTDQLIINDTAIAWAPNFGKTPTSVCMEPCSSGH
RRAHQNGRPKCCFDCIPCSEGEISNYTDVENCVKCAEDQWPNPAKDQCIKRVIDFLSYGD
LLGYILSGCASVFTVLTAAVFLVFIKHRRTPIVRANNQNISYILLMALLMSFLCSFIFIGQPTG
VTCLLRQTTFVFVFSVAISSVLGKTIMVLIAFKATKIEKKFRMLGGINISLGLVFICSFGEFVL
CVIWLIINPPHVESDNKTVPGKTILQCNEGSIISFYLAVSYIGVLSLISFAVAFIARKLPDRFNE
AQHITFSMLVFCSVWASFIPTYLSTKGKHMVAVEIFAIQASAAGMLMCIFTPKCYIILLQPEL
NVRGKPTAKILSHMENHTPHC*
>jgi|Xentr4|359819|e_gw1.290.25.1
YQHYLAAIFAIEEINQNANILPNLTLGYRIYDACTSESRAAANTLSILSGRAASVPNYSCND
KGTLAAFVGHLLSTLTYVVLDITAIYKFPQISYGAQDPALGDRQQFPTFYRTIINEKYHFVTI
SQMLKMFGWTWVGIICSDDESHQRAVNKMSSEITKNGGCVAFVAVYTVENIWTLSNSIEK
IRRSSANVIIVSSNSHFLHRILISMQLNIELKKYFLFPSSLENMSSFLFSLLNGSLILSGHQRAI
PGFKEFVFNAHPTKFSDDDFTNLVWFILFGCSKSADNSSGIYIPACSRNDTLSYFDTSFYAIN
NYITYNMYVAVYALAHSLHKVLYGEPHEKQLYKSRFHLDLYKLNNHLKSIHFQTASGDEIF
FNEHGEVQGNLEIENWISYPNETTAIRKVGTFNSSAPPGEQLLINKSDIIWGTPLNDIPQSVC
IGTCQLGYRKMEKKGLPVCCYECVKCSDGEISNVSDMENCITCPEDEWSNENRNKCIKKT
ITFLSYAEPLGLILANLAVLLSITASLILGILIKNRHSQIVKANNEELSYSLLLTLILSFLCTLLF
IGKPMKVTCMFRQVTFAVIFTISISSVLGKTVTVLIAFHAITPGSMLKIWVGKRAPRCLVLL
LSLGEVIICFMWFLHCPPFPDYDTRSQPSVMTLQCNEGSAVAFYIAVGYIGMLASVSFITAY
LARRLPDIFNEASHITFSMLVFCCVWVTFIPAYISSKGKYMVAVEIFAILSSSSGLLGCIFFPK
CYIILLKPERNISWKATSTKKSINQYNSPFVKGIASCFVE*
>jgi|Xentr4|359838|e_gw1.290.340.1
MQHANQSKLMELYLQGITEISELQTPLFLVFLSIYLLTLAGNVLIIIIICLDPRLYKPMYYFLC
NLSFLDFSLASAIQPKCLVMLVTRIHSISYNGCITQLYFFMFLTAAEFFLLAAMAYDRYIAIC
KPLQYHLLMNYKVCTVMSAECWVAGMLEPLSHVRAFVQLPLQTAQINHFYCDYSALLKI
SSTDTRRIEVMTYLAGALVGMPAFSLTIASYVSIIAAIVKINSSQSRQKAFSTCVSHLTVVFL
FYSTVLITIMQPISNYSSTHGKLVSLLYTIFVPSINPFIYTLRNQDVKRSFYKAKSKSINGRIR
QLI*

>jgi|Xentr4|359852|e_gw1.290.176.1
MENRNQSKAIQFILEGVSEVPEMQIPLFFVFCLMYLTALTGNLFVITLIIQSPALHRPMYSFL
CNLSILDVCSSTVTQPKLLSMLLTGNSVTYFTSCILQLYFFICFTAIEMFSLTAMAYDRYAAI
CKPLNYSVLINTRTCVLLCVSSWLFGFVEPIPHISTISQLSFCGPLIINHFYCDVSVLLKLSCT
DTGFIDNITYFLGSVVGCFACICIVTSYIYIISTILKIQSSKGRQKCFSTCVSHLTVVILFYGSL
FLNYMRPSSQYSSSEEKPFAVVYTALIPMINPFIYTLRNKDVHMAIWKAGRTNR*
>jgi|Xentr4|359856|e_gw1.290.76.1
MENLNISTTITFLLQGISEDPDMQIPLSAAFWLIYLLTLAGNIVIITVICQTPALHKPMYFFLC
NLSFIDISYSTVTQPKLLFMLLTGDKAIEFAACLAQFYFFITFTCAEMLVLTVMAYDRYVAI
CHPLTYPFLMNKKTCIFLTVASWIISLLDPVTHTVSISQLTFCPPLLINHFYCDLSVVLRLSCK
DTFLIDILTYFGSMVGCCAFICMIISYSYIISTVLKIKSGEGRQKGFSTCGSHLIVVLLYYGTIL
TTYLRPSSQYSSSKGKPFSVIYTVLIPMVNPFIYTLRNRDVKKAFGKLLKIK*
>jgi|Xentr4|359869|e_gw1.290.55.1
MITGGENKTLNNEFLLLGFQILHSLRIPFFLSILILYIFTILGNATVAALVLSSHSLQQPMFFFL
GHLSLCDITLTTTIVPVLLQGVLRGPVNLSVAACITQFQILFASAAFECLLLGVMSIDRYVAI
CSPLQYSAIMRKELSFELVTLCWFLGYSFVFNLTILISRLTFCGPNVIDHFFCDFLPILQLSCS
DSFAVKLAQLFLAILTGVFTVMSIIVTYIYILATVFKIPSTTVRQKALSTCSSHLAVVATFLGS
LLSLYVLPSSGNTLTANKLLSLLYTVVTPLFNPIIYSLRNWEIRAALKKVIWRRKLKLF*
>jgi|Xentr4|359875|e_gw1.290.273.1
FAFAIDEINKREDLLPNITLGFEIYDSGINEITTIERTFRILSGNHKLIPNYSCRKQENILALIGH
FVPSCSQAMADLLSLYKYPQLIYGAKDPILDDKNHYPHIFSTIPSEHSLIEAIVALLQYFEW
KWVGIITYSDVNFERSSEEIKKEIIKIGYCVEFLMKVKDHIIKSTIQKYNANVIIIFCSIYKIYY
TLNMYSKSSDKVLITVTEGKFPPFLYLTPVQYFLHGSLWFSIHKEEIPGLKRFIQNASPTTLP
GNTFLDGIWRKNDSSVTKGGLLAIFYESPNIENSIRLVHGVHTAVYAAAHALHGLLYKPH
MRENRNEPNLQKYISWQLIYHLKTVSFTTSGGNTEKHFDERGNVPGNFDIWNLVVFPNW
TLKFVEVGTFNSSAPQGEKLELNVSKIQWNSHFNQVPKSVCSASCSPGHRKVLREGHQVC
CYDCVLCPEGEITNSSDMNNCIKCNEHQWTDERRDKCIQRETEYLSFHDYLGSILMGTSL
LLCMMSTVIYLIFYRHWTTCIVKANNLLLSYILLFSLTLSFLSSLLFIGHPKKGTCLIRQMTF
GIIFATALSTLIGKTMTVVIAFSATKPGSKLAKWMGSQITNISVLLLSLGQVTICLFWLICCP
PFPDTDTKSITGKMILLCNEGSVVAFYIMIGYIGILAIVSFLLAYYARRLPDSFNESQLITFSM
LVFCSVWVSFIPAYINTKGRSVVAVEVFAILTSNAGLLGFIFIPKCYIILLRPELNKKEHLMRK
T*
>jgi|Xentr4|359893|e_gw1.290.228.1
MDIGNHTFSRPVILLGFHIFSHLKIPVFMLIFILYSLTIIGNITLVALVSSSPTLHHPMFFFLSHL
SLSDILLTTNIVPLMLYGILRGEVAISLVECITQFHFYGTALALESLLLAVMSYDRYLAICNPL
RYMSIMERKLCHKLVALCWFFSFIIVIALSSVIGRLHFCGPNIIDHFFCDLYPILQLSCSDTSA
VELEQILIAAPTAVTPFVFTVGTYIRIFITVLSIPSNSGRQKAFSTFSSHLTVVCTFFGSLIGLY
VAPPSGNYLNANKIVSLLYTVVTPLSNPVIYSLRNSEIRKAFKKYVSTIKTGKS*
>jgi|Xentr4|359913|e_gw1.290.210.1
MDNNNKTLFQQVVLLGFQLPRFKIPFFILVLILYSVALSSNVTIVGLVSSSPSLHHPMFFFLS
HLSLSDIILTIDIVPMMLHGICNGGITIPMFACITQFQFFAIALTSECLLLAVMSYDRYLAICSP
FHYITIMGTKLQYQLVISCWLLSFTISFVTVLFISKLEFCGSNIINHFFCDFVPLLRLSCSDTSI
VEIENLALATPTTLFPVLFITATYICIFVTIFRIPSITGRQKAFSTCSSHLSVVGIFFGTLVTLYV
VPSNGHSLSANKVLSLLYTLVTPLFNPLIYCLRNQEIRAAFQRYWQLKCLNKFIGKL*

>jgi|Xentr4|359923|e_gw1.290.77.1
MPAENQSSSSGFILQGFSDYPDIQIPLFCLFLLIYLLTLQGNVLILIVIYQTSLLHIPMYFFLCN
LAFIDLCASSVSQPKLLLILLRRDNTISFGGCMTQLHCFIALACTEFVSLTVMAYDRYVAIC
NPLRYLIVMNRRVCVMLVIACWVFSFAEPVSHTVLISHLPFCRSQTIDHFFCDPSILLTLSCA
NTFPIEMLKYVLGSLVALPAFALTVASYTYIISTIIKIHSATGRKKAFSTCTSHLAVVSLFYGT
TMITYMRPTSQYSSTTSKPFSFLYTALIPLINPFIFTLRNKDIKQHISDQIEVIRSHLPSVGAPA
K*
>jgi|Xentr4|359925|e_gw1.290.69.1
MTDKNQSSTNGFILQGFSSYTQLRVPLLCLFLLIYLVTLLTNVLISAVIYRTSLWHTPMYFFL
CNLAFIDIFTTSIYQPKIIFILLTQDSTISFGACLFQLHCFISLTCAEFVSLAVMAYDRYVAICN
P*RYMTVLNRRVCVILVIACWIIAFIDPLPRTMLISQLPFCGSLVIDHFFCDLTILIKLSCRDTA
FIELFTYISCSLIGLPAFVLIISSYTCIILTFLKIHSATGRKKAFSTCTSHLIMVILFCCSTLITYM
IPSSQYSSTFSKPFSFLYTALTPLINPFIYTLRNKDIKHCLSCKKVIRSQ*
>jgi|Xentr4|359933|e_gw1.290.72.1
TEIHSTGISEDPDMQIPLFLIFCLTYLLTLAGNIIIITVIRRTPALHKPMYFFLCNLSFIDISYST
VTQPKLLSMLLTGDKTINFLACISQFYFFIAFTCAEILVLTAMAYDRYVAICHPLTYSLLLNK
KTCIFISAASWAVSFLDPVAHTVIFFYLTFCPPLLINHFYCDPSVVLRLSCKDTFLIDILTYVL
GSMVGCWAFICIIISYTFIIATILKFKSVEERQKGFSTCGSHLAVVILFYGTILIIYTRPSSQYSS
SKGLKPFSVVYTILIPMVNPLIYTLRNKEVKSALWKIIRNNFFMNKY*
>jgi|Xentr4|359939|e_gw1.290.5.1
MCFFNSPYFSVSTTEILDVLTFEFAIREINEREDLLPNITLGFEIYDSGVSEIMTIERTFRILSG
NHKLIPNYSCRKHEKVFAFIGHFVPSCSHAMSDLLSLYKYPQISYGAKDPTLENKDDYPDI
FHTMPSDRYLKEAIVALLQYFEWKWVGMITDINVNYKRPSEEIKKDIIKIGYCVEFFIVVGP
GNDNGLAETIRNYHANVIILYCPLNSDGLPFLMLLNANGKVWIIITEEGKLLSADLVDYLS
PLHGSLWFAIHKEEIPDLKVFRHSASPTALPRNAFLYKYLKDDGYYLSSYFKNEINVKLSH
NIYTAVYAAAHALHGLLFKPHMRENRNQQILQHVIPWQLNLKNVSFTIPGGKEIYFDETG
NVLSYFDIWNGVILPNQTLTYVHVGTFDPSAPQGKKIKLNVSKIQWHPDFTQVPKSVCSES
CSPGYRRAVRKELQVCCYECVPCLEGEISTLNDMDNCIKCKEHEWTDDRRDKCIQRETEY
LSFHDYLGVFLTATCLFLCAISTGIYSIFYRHRATCIVRANNLHLSYILLFSLTLSFLSSLLFIG
RPKQVTCLVRQVTFGIIFATGLSTLIGKTITVIIAFSATKPGSKLAKWMKTQTIYIIVLFLSIG
QVVICLIWLICSPPFPDTDTKSKTGMIIVLCNEGSVVAFYIMIGYIGILAIVSFLLAYYARRLP
DSFNESQLITFSMLVFCSVWVSFIPAYINTKGRSVVAVEVFAILTSNAGLLGFIFIPKCYIILFR
PELNTKEHLMMKI*
>jgi|Xentr4|359969|e_gw1.290.232.1
MNLENKTTDKAILLLGFQMHYQYKIIFFLLIFTLYSLTLVVNVMIVALVSSSPCLHHPMYFF
LSNLSISDLILTTTIVPNMLYGLLRRGVTISIAACITQVQFFGIAIVSQCLLLAVMSYDRYLAI
CNPLQYVLIMSNRHRFQLVIFCWSVSMGVSCVTILLISRLDFCGPNIIYHFFCDFAPILQISCS
ETFGLEFLQKFFAFTITVLPLVFISVTYVHIIISILKIQSSSGRQKAFSTCSSHLAVVGTFFASLI
SLYVVPSTGNTLNANKILSLLYTVITPLFNPIIYSLRNLEMRATLGKCLGTFRYSVNRQSFCD
TMQW*
>jgi|Xentr4|359985|e_gw1.290.178.1
MNQSSSSGFILQGFSDYPDIQIPLFCLFLLIYLLTLQGNVLIIIVIYQTSLFYIPMYFFLCNLAFI
DLCASSLSQPKLLSILLRRDNTISFGCCMTQLHCFMTLTCVEFISLTVMAFDRYVAICNPLR
YLVVMNRRVCVMLVITCWMFSFTEPISHTVLISHLPFCRSRTIDHFFCDPSILITLSCANTFPI

EILTFVLGSLVALPAFVLTVASYTYIISTIIKIHSATGRKKAFSTCTSHLTVVSVFYGTTIITYV
RPTSQYSSTSKPFSFLYTALIPLINPFIYTLRNKDIKQRISNKTEIKRSHL*
>jgi|Xentr4|360016|e_gw1.290.1.1
FYISVKLTNIWHVLTFAFAIDEINKREDLLPNITLGFEIYDSGFSGTATIERTFRILSGNRKLIP
NYNCRKQEKILALIGHLVPPCSNTMADFLSLYKYPQKDLGVFVDNKLSNSRQCHSVATKA
NKVLSCPVFKVVVALLQYFEWKWVGIITYSEEKFQSLSKRMNKEIIQIGYCVEFFAVLTYG
SQVYKTKKIKNDHANVIILYCYLAQVNAALASFFKSNGKVFIIITEEETYAFKYTLGSYHPL
NGSLWFSTHKEEIPGLEGFIQSASPTALPGNTFLDEIWENNNTFSDSSGLKHYQEETDMNL
LHNIHTAVYTAAHALHSLLLKSHRTENRNELTVQNFIPWQLIYHLKKVSFKTPGGNTIYFD
EEGNVPGYFDIWNGVILPNGTFVYVHVGTFDSSAPQGEKLKLNVSKIQWHPDFTQIPKSF
CSESCSPGYRRAVRQGQVCCYDCVLCPKGEITNLSDMNNCIKCNEDQWTDERRDTCIQRE
TEYLSFHDYLGSILMGISLCLCATATLIYLMFYRHRATCIVRANNLVLSYILLFSLTLSFLSSL
LFIGRPRNVTCLVRQVTFGVIFATALSTIIGKTITVIIAFSATKPGSKLAKWTKTQITYRIVLLL
TNGQVIICSIWLICSPPFPDTDTKSMAGKMILLCNEGSVVAFYIMIGYIGILAIVSFLLAYYA
RRLPDSFNESQLITFSMLVFCSVWVSFIPAYINTKGRSVVAVEVFAILTSNAGLLGFIFIPKCY
IILFRPELNNKKHLMRKI*
>jgi|Xentr4|360023|e_gw1.290.254.1
MNLGNKTTDKAILLLGFQMLYPYEILFFLLIFTLYSLTLIVNVMIVALVSSSPCLHHPMYFFL
SNLSISDLILTTTIVPNMLYGLLRRGVTMSIAACITQFQFFGIAIMSQCLLLAVMSYDRYLAI
CNPLQYVRLMDNRHRFQLVIFCWSVSLTVSFVMILLVSRLDFCGSNIINHFFCDFVPILQISC
SGTFGLELVQKFLAFPITVLPLIFISVTYVCIIISILKIPSSLGRQKAFSTCSSHLAVVGTFFGSL
ITLYMVPSAGNTLNANKILSLLYTVITPLFNPIIYSLRNLEMRAALGKMSRG*
>jgi|Xentr4|360030|e_gw1.290.23.1
FAFAIDEINKREDLLPNITLGFEIYDSGISGTGTIERTFRILSGNRKLIPNYNCRKQEKILALIG
HFAPSCSHAMDDVLSLYKYPQVLEVSFVTRFSRQFYKVIDMVRRYLPVLHVVVALLQYFE
WKWVGIITYSEEKFQSLSKRTNKEIIQIGYCVEFFTVLTYGKEVFETEKIQKYRANVIIIFCY
LAEVTKVISSFLKSNGKVFIIITEEETLSFEYTLRSYHPLNGSLWFSTHKEDIPVLEGFIQSAS
PTALPGNTFLDEIWKNNNFSDSSVLTYYQEEIDIDMNLLHNIHTAVYTAAHALHSLLFKSY
RTENRNEQTVQNFISWQLIYHLKKVSFKIPGGNTIYFDEEGNVPGYFDIWNGVILPNGTFV
YVHVGTFDSSAPQGKKLKLNGSKIQWHPDFTQIPKSFCSESCSPGYRRAVRQGQVCCYDC
VLCPKGEITNLSDMNNCLKCNEDQWTDERRDKCIQRETEYLSFHDYLGSILMGISLCLCAT
ATLIYLIFYRHRKTCIVRANNLVLSYILLFSLTVSFLSSLLFIGRPRNVTCLVRQVTFGVIFAT
ALSTIIGKTITVIIAFSATKPGSKLAKWMKTQITYRIVLLLTNGQVVICLFWLICSPPFPDTDT
KSKTGQIILLCNEGSVVAFYIMIGYIGILAIVSFLLAYYARRLPDSFNESQLITFSMLVFCSVW
VSFIPAYINTKGRSVVAVEVFAILTSNAGLLGFIFFPKCYIILIRPQLNNKENLMRKTKI*
>jgi|Xentr4|360044|e_gw1.290.319.1
MCENNETTMTELFLLGFNINTNLKTLLFIIVLLIYISLLTGNLLIIILVSKSPNLKNPMYSFLK
HLAIADILFTTNIIPNMLYVMLNDGGRITTVGCFTQYYFHSLSIIAQSLILTSMAFDRYLAIC
YPLRYLSIMNPKVCSSLIFWSWATGFILLPGEFASISQLKFCGSNAVDHFFCDLSPVLAIAST
DTYRVQWEDFVLAVLVIIVPFVFVLTSYFCIFLAILKISTTAGKKKAFSTCSTHLVTLCIYYG
TMITIYAIPSGQNTPDESKFRSLLYTVLAPFLNPILYSMRNQEIQKSLRKLFCKIR*
>jgi|Xentr4|360055|e_gw1.290.314.1
MCENNETVVTELFLLGFNNHTNLKILLFIVFLLIYISLLTGNLLIIILVSKSPNLKNPMYSFLK
HLAIADILFTTNIIPNMLYVMLNDGGRITAVGCFTQYYFHSFSVFAQSLILTSMAFDRYLAIC

HPLRYLSIMNPKVCSLLIFWSWATGFILLPSEFTSLSQLKFCGSNVIDHFFCDFAPVLAITST
DTYRVQLEDFVLSVLLIIVPFVFVIISYMCIFLAILKISTAAGKKKAFSTCSTHLVTLCTYYG
TIITIYIFPLGQDTPNESKFKSLLYTVLTPFLNPILYSMRNQEIRNSLWKLFCKIK*
>jgi|Xentr4|360063|e_gw1.290.174.1
MLKQNQTKMNEFILLGISDDAEQQIPLFFLFALIYIFTLLGNFVIITVIYLSPQLHNPMYFLL
CNLSFIDLSSASITQPKLLSMLLLRENTISYGGCIAQLYSFMLFTGTEFFSLTAMAYDRYVAI
CKPLRYHILMNNTICVRITSTCWVLGMVDPLPHTVLITQLSFCGSHVINHFFCDMSVVLKL
SCVDTSFIESMTYLFGLLVEFPVFLLLVISYCCILCAILKIHSAKDRSKAFSTCSSHLIVVILFY
GTVWIMYLRPLSQYSADHSKPFSLLYTAIIPLVNPFIYSLRNRDVRNSIFRTSIVQID*
>jgi|Xentr4|360064|e_gw1.290.82.1
MSFRNYDNNKSSTDGFILQGFSEYTELRIPLFCLFLLIYLLTLKANLLILIVICYKTSLWQTP
MYFFLCNLAFIDIFTSSVYQPKLILVLSSGDNTISFSGCLFQLHCFMSLACTEFVSLTAMAYD
RYVAICNPLRYLIVMNRRFCVILVITCWMVGFTEPLSHTLLISTLPFCGSRKVDHFFCDLTV
LLKLSCKDTFFIQVLTYVLGSVVGLPAFALIIASYTYIIATILKIHSATGRKKAFSTCTSHLTV
VILFYGSILITYMRPPSQYSAILSKPTAFLYTSLTPLINPFIYTLRTKEMKNYVSRKQHH*
>jgi|Xentr4|360066|e_gw1.290.74.1
MPDNNQSSADGFILQGFSEYTELRIPLFCLFLLIYILTLKANLFILTVIYKMSLWQTPMYFFL
CNLAFIDIFTSSVYHPKLLLGLLGGNNTISFIGCLFQLQCFMFLACTQFVSLTAMAYDRYVA
ICNPLRYLIVMNRRFCVILITACWIFGFTEPLSHTLLISTLPFCSSRIIDHFFCDLTVLLKLSCK
DTFYIELLTYISGALVGLPAFALIIASYTYIISTIIKIHSATGRKKAFSTCSSHLTVVIVFYGSILI
TYLRPPAQYSAILSKPSAFLYTALTPLSNPFIYALRTKEIKQYIFRKGIIKC*
>jgi|Xentr4|360077|e_gw1.290.153.1
FKIPTSFCTQKCSSGQRRAHQTGRPPCCFDCVPCSEGEISNSSELEICVKCPDDHWSNPTRN
RCIKRIIDFLSYEDLLGYILSACASLFIANTAMVLLVLLKHRRTPIVRANNQKISYILLMALL
MSFLCTFMFIGRPTGVTCLLRQALFVCVFSVAISSILGKTMTVLFAFKSTETANRFRKWGK
MNISMVIVFLCSFVEFVICVIWLTTSPPYVESDIKAIPGKIILQCNEGSVISFYLAVSYIGVLSL
ISFAVAFIARKLPDRFNEAQHITFSMLVFCSVWASFIPTYLSTKGKHMVAVEIFAIQASAAGM
LMCIFTPKCYIILLKPELNVKANLTFKRNPKRINK*
>jgi|Xentr4|360078|e_gw1.290.61.1
MPAENQSSSSGFILQGFSDYPDIQIPLFCLFLLIYLLTLQGNILILIVIYQTSLLSFPMYFFLCN
LAFLDLCASSVSQPKLLSMLLRGDNTISFGGCMTQLHFFLSLTCAEFVSLTVMAYDRYVAI
CNPLRYLIVMNRRVCVILVITCWMFSLAEPMSHTVLISHLPFCRSQAIDHFFCDPSILLNTV
LCKHFFYSVVDICFGLIGGTSCIRTYSSLLYLHHFHYYKDPFCYRKEESFLYLYLPPHCGQCI
LRRNTDYLYEAYISVFIHFI*TIFPALHCLDTINQPFYIHISEQGF*TTNFQQNKNQKESL
>jgi|Xentr4|360081|e_gw1.290.2.1
VLTFAFAIDEINKREDLLPNITLGFEIYDSGINEIMTIERTFRILSGNRKLIPNYSCRNQEKVIA
FIGHLVPSCSQAMADLLSLYKYPQIIYGAKGDNYQYPHIYSTIPSGQSWNEAIVALLQYFE
WKWVAIFTVPQENFKRSSEEMKTEIIKIGYCVESFIVDDEENDTIVAENILKCHAKVIIIYCY
LNQVNEVMLLFSKSNGKVLIIMNEGENLSTRSFQPTNGSLWTFKYKENIQSFQEFMQSASP
TALSDSPFLEFIQDNKLLGYFQKERMKLLYNVYTAVYAAAHSLHGLLYKPHIKMNKNELN
NLISWQLNYHLKNVSFTIPGGNAIYFDKGGNVPGYFEIWNGVILPNLTLVYGKVGTFDSSA
PQGEKLKLNVSKIQWLPYFTQVPKSVCSESCSPGHRKVLREGQPICCYDCVLCPEGEITNIS
DMDNCIKCKEHEWTDERRDTCIQRETEYLSFHDYLGIFLTATSLFLCVITTGIYSIFHRHRAT
CIVRANNLHLSYILLFSLTVSFLSSLLFIGRPNEVTCLFRQVTFGIIFATELSTLIGKTITVIIAF

SATKPGSKLANWMKTQITYSIVLLLSIGQVVICSVWLVCSPPFPDTDTKSKTGMIIVLCNEG
SVVAFYIMIGYIGILAIVSFLLAYYARRLPDSFNESQLITFSMLVFCSVWVSFIPAYINTKGRS
VVAVEVFSILTSNAGLLGFIFIPKCYIILLRPELNTKEHLMRKM*
>jgi|Xentr4|360085|e_gw1.290.271.1
MDLWQVLTFAFVIDEINKREDLLPNITLGFEIYDSGICEITTIERTFRILSGNHKLIPNYSCRK
QEKVLALIGHLVPSCSQAMADLLSLYKYTQVSYGAKDPMLDNKDQYPYIFSTIPSELSLNE
AVVALLQYFEWKWVGIIAYSDVKFERPSEEMKKEIIKIGYCVEFFIVVGYGEEVFLRGKIQ
KSHANVIIIYCSFHTFTSASSLFFNSNGKVLITITEGGKFLSDDSNFYHANGSIWFSRQKEDI
LGLKGFIQSESSTPLPGNTFLDTFLEDPLIHFQDGFSINQLHNIYTTVYVAAHALHGLAVQN
TIDYRCFFAVETFAAKSLAVKKCHQLLQKRHLNGHMKNVSFTIPDGKEIYFDERGNVPGY
FDIWNVVILPNRSLVNVAVGRFDSSAPRGEKLKLNVNKIKWHPDFIQVPKSVCVESCSPGY
RKAPREGQQACCYDCVLCPEGEISYIGDMDNCIKCKEHEWTDERRDTCIQRETEYLSFHD
YLGIFLTATSLFLCVITTGIYSIFYRHRATCIVRANNLHLSYILLFSLTLSFLSSLLFIGRPKQV
TCLVRQVTFGIIFATELSTLIGKTITVIIAFSATKPGSKLAKWMKTQITYRILLLLLIGQVIICS
VWLVCSPPFPDTDTKSKTGMIIVLCNEGSVVAFYIMIGYIGILAIVSFLLAYYARRLPDSFNE
SQLITFSMLVFCSVWVSFIPAYINTKGRSVVAVEVFAILTSNAGLLGFIFTPKCYIILFRPELN
NKEHLMRKI*
>jgi|Xentr4|359790|e_gw1.290.59.1
MPDVNQSSSSGFILQGFSDYPDIQIPLFCLFLLIYLLTLQGNVLILIVIYQTSLLHIPMYFFLC
NLAFIDMFSSSVSQPKLLSMLLGRDNTISFGGCMTQLHSFMSLTCVEFVSLTVMAYDRYVA
ICNPLRYLIVMNKRVCVVLVIACWMFSLAEPMLHTLFISHLPFCRSQAIDHSSVILPYY*PCL
VQTLFLFSC*HMFCAHWWHFLPSHLQ*PLIPTSFPLY*RSILLQEGRKLSLLVPPTSLWSVSS
MAL*SLLI*GLHLSIHPLYPNHFPFSTLP*YH*STLLYTLYETKILKHIFPI**NSKGVVYNWQ
AQHC
>jgi|Xentr4|360636|e_gw1.296.79.1
MASSTVTPLTSNATEEYDPLGGHAVWEVVLIVITTGILSLVTVIGNVLVLLAFKVNSDLKTV
NNYFLLSLACADIIIGALSMNLYTTYIVMGRWALGPVSCDLWLALDYVASNASVMNLLIIS
FDRYFSITRPLTYRAKRTPRRAAIMIGMAWFISFVLWAPAIIFWQYIVGERTVEATDCYIQFF
SQPIITFGTAIAAFYLPVTIMIILYWRVYRETENRSKELAGLQGSGTDHTSHPLALGSVRSGS
SSGAGESERLSRGQRTVPRAKQGCCFPRQLTSNPSLRSYPEHRSNGSCNTMEDAASADSLS
SSSEAEDRPYEMKNICSAVIRLPMVSSVVGTPKGSRGSSDTLGRGDIEAETNGGKSEVTRK
TSRAQRKSNSLIKEKKAAKTLSAILLAFILTWTPYNIMVLVSTFCPDCV
>jgi|Xentr4|360664|e_gw1.296.4.1
MVHNISNHTSHSAVDPHYNFLALVFGFILIVCIICGNVLVCLSVCTEKALKTTTNYFIVSLAV
ADLLLAVLVLPLYVYTEFVGGVWTLSLVLCDVLMTMDVMLCTASIFNLCAISVDRFIAVSI
PLNYNRRQVDNRQLFLISTTWIFAFAVASPVIFGLNNVADRDPTMCKLEDNNYIVYSSVCS
FFIPCPIMLVLYCAMFHGLRKWEETRKTKLRSHMSPGLKPPQCPALLLDRSPTDSKLDELN
LYDQGDCPFPKSYVDNGHGIQTVAYPHLKYSTNMEPERKQAKINGRERKAMRVLPVVVG
AFLFCWTPFFVVHITRALCESCYIPEQLISIVTWLGYVNSALNPVIYTIFNTEFRNYFRKVLR
VCC*
>jgi|Xentr4|361337|e_gw1.302.61.1
PTVDVPDHVHYVVGAVILAVGITGMLGNFLVIYAFCRSRSLRSPANMFIINLAITDFLMSVT
QAPVFFATSLHKRWIFGEKGCELYAFCGALFGITSMITLMVIALDRYFVITRPLTSIGVMSK
KRAVLILSGVWLYSLAWSLPPFFGWSAYVPEGLLTSCTWDYMTFTPSVRAYTMLLFCFVF

FIPLFIIIYCYIFIFKAIKNTNRAVQKIGTDDNKESHKQYQKMKNEWKMAKIALIVILLYVVS
WSPYSTVALVAFAGYASVLTPYMNSVPAVIAKASAIHNPIIYAITHPKYRMAIAKYIPCLGSL
LRVKRRDSRSYSSYPSSRRSTVTSHCSQSSDVGGHPKLKNHLPSVSDSESVGKQFAIISVCT
IK*
>jgi|Xentr4|361402|e_gw1.303.58.1
MLHSTVNSTNATINVGTELKPTNTSDTVMDVPEELFLFLCVFSLLENILVVIAIFRNHNLHS
PMYYFICCLAASDMLVSSSNLGETLIIFMLKQGIIKSEPLLVKKMDYIFDTMICCSLVTSLSF
LGAIAIDRYITIFYALRYHSIMTLRRVVIAIGVIWSVSLVCAAIFIVYHESRAVILCLIVFFLFM
LALMVALYIHMFALARQHARSISALQKGKSRRITPHQARANMKGAITLTLLLGVFFLCWG
PLFLHLTLFVSCPGHHICNSYFYYFNIYLLLVICNSVIDPLIYAFRSQELRKT
>jgi|Xentr4|361539|e_gw1.304.36.1
MASSTGPAWCFFTFIWLLSRFCQLAHAQTTAVPSGCGAGINSLYYYLCDLNAAWGIVLQAI
TSAGIVSTFILTIVLVASTPFIQNKKKKSLLGTQVFFLLGTLGIFGLVFDMVVKKDFATCASR
RFLFGLLFAICFACLWVHGVSLNYLIRRNTQPSGWWIFGLAVCLSLVEAIINTEWLIITIVKT
ANPPADPCLIGNADFVMALIYVMFLILAGFITAWPALCGRYGHWRKHAIFILLTLFLSISIWI
VWIVMYVYGNVQVGNPLYWDDPTLAIALVSNGWAFIFFYIIPEISQLTKPTLEQTFEEEPYP
IRGVGYETILKEQSTQRMYVENKAFSMDEPPSAKKPVSPYSGYSGQTGKSVYQPTEMAL
MNKTLPERSYDVVIPRATANPPPVASSNSTLRADDAYAVQNFHIANPTAHNMQSQSPYNR
W*
>jgi|Xentr4|361577|e_gw1.304.39.1
TDDYHSKESYISVPVLCNKKEIQNFTQIYQPIVYSLLFVTGMVGNGLVLLTYWFCRKIKSM
TDVYLISLALADLLLVLSFPFLGINAVQGWIFGNIMCKVVQGLYSVNFFSGLLFLTCISVDR
YIEIVQAVQAHKCRHKSIYYSKLTCIVVWVFSLLLTLPQFIYSHSESIGGFYHCKMIFPEEVT
ATVKGISNVAQIIFGFIIPSLVMVFCYSVIVKTLLSSKTLRRHKTLKVIISLVVVFVMFQLPHS
VVIFLETADILQSKQMPCEVSKKKDVALIVTSSLAFTRCCLNPILYAFVGVTFRRDILLLLKN
LGCISRASTTYNGSRRLHASSVHIDTSSFSL*
>jgi|Xentr4|361585|e_gw1.304.107.1
MDEGNVSDPSPCVLGIIPVIYYSILLCLGLPVNMLTAVALSRLASRTKKSSYSYLLALTISDIL
SQIFIIFVGFILQTAILHRKVPSTLIHVVSVLEFSSNHASIWVTVILTVDRYVALCYPLQYRSF
SYPERTRRVIVFVFLSSFVTGVPFYWWSDVWRDPRTPGLLDLILKWTHCFIIYFIPCTIFLIT
NSVIIYRLRKRTGSKKCQLRNGKTTAILLGITSVFAVLWAPRTIVIIIHMYVSSVNKDWKVH
LAMDIGNMLALLNTAVNFFLYCFVNRRFRDTVREILGIFRRCHMKEGQSIQVEGCSDSLKP
VGIPVVTAV*
>jgi|Xentr4|361597|e_gw1.305.218.1
MLFHAATLGDLVVFKPRTVLIYMIVLCVGPCRSGIQPLSPACHLEIIKVVEEYDYIQEGDIMI
GGVMTAHLYMINVTFPRVNSKRFLCTAPSQQQFRHFIDFRFAVEQTNKDPARLPNLTLGYH
ISDSCEDPLKAVRSVLQILSGTREPVPNYSCVGKRNIAGFIGDEASETTVPIAQILSIYGLSQI
SYGATAQSLRDRVAFPYFFRTVQSEETSYLALGKLLSHFGWTWVGIITSDDISGEREHQLL
AKYLSREGVCVEFTIRIKTNNDIIDTDTLQKSFTSVILLCGTVSLFLLMRLILIKSVFLEKTWI
LSTNWGTNDLVIRHGQELFDCSLGLVPGYHYDLDTPEMRNFLDNLHPSNYPEDKLLENIW
MIFHLCLLEDPNKNHLYEYIYLQPLHNCTGQERITDLSYFTADFSSPQVHLAVDLMSQALH
DMNIHPSEKHVGKKEMYHYVHQLHRYLKALQYNSPDGPNFYFGENGEYITSYRIYNYVIT
AGYRLTRNLIGDFSPWDPPDQQLHIAWTALSWRSNTIPRAQCTDSCPLGFRKAPMPGVQT
CCYDCVPCSEGEISNITDSISCFPCPDMEWPNERKDQCIASKDDFLSYTNDTVSVLCSSISIL

FFLITSWISGVFITYRDSPIVRANNRSLSFLLLVSIKLSFLSVFLFLGRPVDITCMLRIITFGITF
SIAVSSLLAKTIMVCVAFKATKPGSSWRKWLGVKLSNSVVLFCSSIQIIICMTWLAISPPFQE
LDIHTSPGTIIIQCNEGSDIGFYSVIGYMGLLAAVSFVLAFLARSLPDSFNEAKYITFSMLLF
CSVWITMIPAYLSTKGKNTVCVEIFAILTSSAGLLACIFLPKCYTILLRPEINLKTNLLGNK*
>jgi|Xentr4|361602|e_gw1.305.213.1
ATAEYYRQFLDFHYFIEQRNNNTAFFPNLTLGYHIYDSCGDPPKAVRSVLQILSGTRETVPN
YSCVGKRNIAGFIGDLTSETTVPIAQILTLYGYSQISYGATDPLLRDRAAFPYFFRTVQSDHH
HCYLLTELLKHFGWTWVGVIRLDDDAGDREFQLLTKYFSNNGICIEFSIKINIYNIQSREHII
NKHKEQFQKSTTSVIVLCGTVSITIAEEFRLLKDVLREKTLVLTTNWAANHMIYYAIEVFN
GSLGFMQRSLYELNSPEIKAFLASIHPSKYPKDKLLEDLWIQHHLCSSTNEYKNKIYEYLYP
YSLHNCTGEERIQDIWNIDNALHSPWVHLAVTLLSQAMYKMHMTLSKLSPKQKKRLYNY
RYQLHHYLKVLRYKVQGAPYTFDEAGEFKSQYWMYNYMIEAEENVKVQYFEFNPLAPS
EQKLIPADITWKTGQKPRAQCSDNCPTGFRKASKPGTHSCCYGCAQCSEGEISNVTEREN
CTRCPDMEWPNEKRNQCVARTEEFLSYNDDVISICLLSLSVVLYLCTVLITTCFIIYRDTPIV
RANNRSLSFLLLVSIKLSFLSVFLFLGRPVDITCMLRIITFGITFSIAVSSLLAKTIMVCVAFKA
TKPGSSWRKWLGVKLSNSVVLFCSSIQIIICMTWLAISPPFQELDIHTSPGTIIIQCNEGSAIG
FYSVIGYMGLLAAVSFVLAFLARSLPDSFNEAKYITFSMLLFCSVWITMIPAYLSTKGKNT
VCVEIFAILTSSAGLLFCIFLPKCYTILLRPEINMKSNLLGNKYK*
>jgi|Xentr4|361629|e_gw1.305.43.1
PKSICSESCPPGYRKAPQKGQPACCFDCILCSEDEFSNETGKHNGTYWTHCCMKCPEVMW
ANEMHDGCRDKTLEFLSSEGALGGTLAALSIMGSLLPLSILFIFLKNADTPVVKANNRNLS
YTLLVSLFLCYLCALMFIGRPITITCILRQFIFGISFVMCISCVLGKTMMVVIAFNLTQPRSSR
RMWLNSRVTNTLVLVCTAIQVIICAGWLAHSPPFQYTDNKSKMGTINVECNKGSPIAYSCT
MGYMGFLATLCFVVAYLARKLPGSFNEAKLITFSMLIFGAVWISFIPAYLSTTGKYMVAVE
VFAILSSSSGLVACIFLPKVYIIILRPEINKIGRKIIKE*
>jgi|Xentr4|361659|e_gw1.305.214.1
MGACKPEVQNKHSACHLEMEKSFEEYEYIQEGDIMIGGVLTVNSYATFLKHPEDNSIRILC
MEAVAEYYRQFIDFRYFIEQTNNNRAFFPNLTLGYHIYDSCGDPPKAVRSVLQILSGTREPV
PNYSCVGKRNIAGFIGDLTSETTVPIAQILTLYGYTQISYGATDPLLRDRAAFPYFFRTVQSD
HHHCYLLTELLKHFGWTWVGVIRFDDDAGDREFQLLTKYFSNNGI*YEFSIKINIYNIQSRE
HIINKHKEQFQKSTTSVIVLCGTVSITIAEEFRLLKDVLREKTLILATNWAANHMIYYAIEVF
NGSLGFMQRSLYELNSPEIKAFLASIHPSKYPRDKLLEDLWMQYHLCSSTNEYKNKIYEYL
YPYSLHNCTGEERIQDIWNIDNILHSPRVHLAVNVLSHAIHNMYMSLSKLSPKQNERLYNY
RYQLHHYFKFLEYKVQGVPYNFDENGEFKSLYWMYNYMTETEEKTNVQYFEFNPLAPSE
QKLIPADITWKTGQKPRAQCSANCPTGFRKASKPGTQSCCYGCAQCSEGEISNVTGLSGSI
MEIKTGRYMADSVNAYKRGLDEFLNKQNIQGYCDTNIYS*CGLCVLGLLGWVDLMLSFL
SVFLFLGRPVDITCMLRIITFGITFSIAVSSLLAKTIMVCVAFKATKPGSSWRKWLGVKLSNS
VVLFCSSIQIIICMTWLAISPPFQELDIHTSPGTIIIQCNEGSAIGFYSVIGYMGLLAAVSFVLA
FLARSLPDSFNEAKYITFSMLLFCSVWITMIPAYLSTKGKNTVCVEIFAILTSSTGLLACIFLP
KCYIILFRPEINAKSYLFQNKSM*
>jgi|Xentr4|361660|e_gw1.305.206.1
MAVCNSLIELIYLIMLCVGPCAPGALPAIPACHLEIIKTYEEYEYIKEGDIMIGGVMTFSTKF
PGPRDERIIYCDDPQKFRYLVEFLFLIKELNDNPSRLPNVTLGYHIHDSCGTSQRALINVFKI
LSGTREPVPNYSCVAMRNIVGFIGDLESQPTISMAYMLGVLGYSQISYGATDPSLSDRVTFP

YFYRTVQSDEEEYIALCKLLNYFGWNWVGILKSDDYSGYRDHQLLIKYLSREGICVAFTIS
VWEKPSIDELKKKISTFYSASVFIICGDVAISNLQSFASTILYSLRRKTFIFLSKWLNHVDVL
DSSVLELFHGSLLFVQKRRNDQFVSRLKEFSDSYKPSNYPEDILLEDIWLLKFFCLSKVKS
NNAHHRYMYYRRHNCSGKETLTNIPSYLNPYHSASLMNAVEIMFVALTHMQNSLPKQTS
QYRHMYNYQLHKYLRNVSYTSDGSPALSFTEKGDFNYEYEIVNPQLGSDETWTWNTVG
NYVPWAQRLTLTLEKIRWKTPNNKLPISRCSESCVPGYRKVTRPGIYMCCYDCVWCPEGEI
SNTTDSENCIQCPKMEWPNRKKTECIPKTVDFLSYTHILSAFFSATSVLFFLTSLLILGVFIA
HRGTPIVRANNRSLSFLLLVSIKLSFLSVFLFLGRPVDITCMLRIITFGITFSIAVSSLLAKTIM
VCVAFKATKPGSSWRKWLGVKLSNSVVLFCSSIQIIICMTWLAISPPFQELDIHTSPGTIIIQC
NEGSAIGFYSVIGYMGLLAAVSFVLAFLARSLPDSFNEAKYITFSMLLFCSVWITMIPAYLS
TKGKNTVCVEIFAILTSSAGLLACIFLPKCYIIQFRSEINTKSQLLGNRQNQY*
>jgi|Xentr4|361710|e_gw1.305.105.1
SRPSVQKYKYLVDFLFLIKEMNDDPFFLHNMTLGYHIHDSCGSPQLALRSVLQILSGTREP
VPNYSCVGKRNIAGFIGDLHSDPTIAMAHILSVLGYTQISYGATDPALSDRVTFPYLFRTVQ
SDEEEYIALAKLIKYFGWNWVGILQFDDYSGYRDHQLLIKALSREGICVEFTAKLNYRTV
RQTRYMIQQASTDVILICGEVSFANSEMLFLIYDLRKKTCIFLSKLLNDFEELIFHENLLSGG
LTFMQNRENDQSDPKFIEFSDSVNPSRFPGENLLENIWIWYFSCLSKKKRWTYDINSPYSR
VPYNCSWKEKLTDIPNYLGLYRSGSLIQAVDMMAYALEDMQNLLKKQTSKKHTWRDNY
NYQLHRFLKHVPYIIGRSPESCFNEKGEFPHHYEISNPQLSPGHKWAWNILGRYIPWAPLEQ
RLILNPDKIRWKTPNNKAPRAQYSDSCVPGYRKAPKSGIYTCYYDCVLCPDGEISNTTDSK
SCFRCPDLEWPNEKKNQCIARTEEFLSYSNDVISVFFSSFSLLFFLLTVLILGVFFTYRDTPIV
RANNRSLSFLLLVSIKLSFLSVFLFLGRPVDITCMLRNISFGITFSIAVSSLLAKTIMVCVAFK
ATKPGSSWRKWLGVKLSNSVVLFCSSIQIIICMTWLAISPPFQELDIHTSPGTIIIQCNEGSAI
GFYSVIGYMGLLAAVSFVLAFLARSLPDSFNEAKYITFSMLLFCSVWITMIPAYLSTKGKN
TVCVEIFAILTSSAGLLASIFLPKCYTFLFTPEINTKTHLFVNKL*
>jgi|Xentr4|361753|e_gw1.305.16.1
YEKDGDIIFGGLIPVHLEPSNIKADFTTKPSRGRCNTVSVMCYLHVLAMVFAINEINENPEF
LPNLTVGFRIYDSCFNEFQAVEGTLQILSGADRAIPNYICESKVVGFIGDLQSLSSVAVARIL
GLYRIPQISFGSALFDLSNKVNFPSFLRTILSDNLQPIYFSILMIHFNWTWIGIVAADNDFGLY
DSELLRKDIEASGICVAFFVRISAQHTRDQSLSVLHVIRKSSATVVLLYCSLPELIPFMQVAT
EETLTGKVWIASGSVMSSPIFTYKQFWRILNGTIGLNLPVTLIPGFEKFMYTINPSLYPKDIY
MKAFWEANFGCLWNEQNYSITENPGNGIMLYCTGQEDLKSLERQGYHEDITPFAVLAYNA
VYSLAHGINNMISCRSGSPCLSIRTLEPWKVLHYVKHVKAKTTAGQELSFDANGDQLYDT
VLYVNWQALPNDVTRALHFAIVGAYPERYISTFNNTFIWSGGYTEVPTSICSESCPPGYRK
APQKGQPVCCFDCILCSEDEFSNETDSTSCTKCPEDMWANEEHNACGMRTLELLTYNEAL
GGTLATLSVMGFLVPLTILTIFIKNSETPVVKANNRNLSYLLLLSLCFCYLCALMFIGRPITIT
CILRQFIFGISFVMCISCVLGKTMMVVIAFNLTQPRSSRRMWLNSRVTNTLVLVCTAIQVIIC
AGWLAHSPPFQYTDNKSKAGTIIVECNKGSPVAYSCTM*YMGFLASLCFAIAYLSRKLPGS
FNEAKLITFSMLIFGAVWISFIPAYLSTTGKYMVAVEVFAILSSSIGLVACIFLPKVYIIILRPE
MNKTGRKIIKE*
>jgi|Xentr4|361771|e_gw1.305.208.1
MAVCNSLIELIYLIMLCVGPCRSGALTANPACHLEIIKTDEEYEYIKEGDIMIGGVMTVSQS
LGPEHKKRIFFCERPSPQKFRYLVEFLFLVKELNDNPSRLPNVTLGYHIHDSCGASQKALM
NVLKILSGTREPVPNYSCVGKRNIVGFIGDLESQPTISMASILGVLGYSQISYGATDPSLSDR

VTFPYVYRTVQSEEGEYIALCKLLKYFGWNWIGILRSDDYSGYKDHQLLIKYLSREGICVA
FTKSVREKLPIQALKKNINTFFSASVIIICGDVGISNLQSSSAEMYYLRGKTFIFLSKWLNHV
DVLHYCVLDLLHGSLLFMQKRINNQFVPRFKEFSDSYKPSNYPEDKLLEDIWLWRFSCLS
KNKYKNAHHKFMFHLLYDCSGNETLADLPSYLIPYHSASLMNAVEIMSVALTHMQNSLP
KQTSQYRHMYNYQLHKYLRNVSYTSDGSPALSFTEKGDFIYEYEIVNPQLGSNKTWTWN
TVGNYVPWAQRLTLTQEKIRWKTPNNKLPISRCSESCVPGYRKVTRPGIYMCCYDCVWCP
EGEISNTTDSENCIQCPKMEWPNRKKNKCIPKTVDFLSYTHILSAFFSAASALFFLASLLIL
GVFITYRDTPIVRANNRSLSFLLLVSIKLSFLSVFLFLGRPVDITCMLRIITFGITFSIAVSSLL
AKTIMVCVAFKATKPGSSWRKWLGVKLSNSVVLFCSSIQIIICMTWLAISPPFQELDIHTSP
GTIIIQCNEGSAIGFYSVIGYMGLLAAVSFVLAFLARSLPDSFNEAKYITFSMLLFCSVWITM
IPAYLSTKGKNTVCVEIFAILTSSAGLLACIFLPKCYIIQFRSEINTKSQLLGNRQNQY*
>jgi|Xentr4|361808|e_gw1.305.17.1
YKHILAIMFAIEEINRNPLLLPNVTLGYEIYDSCDYVSKAVEATLKLFSGRQDHVPGYRCTT
KGPLAAFIGLPYSMAELTQIYRYPHISYGTQDPVLEDKRLFPSFFRIVPNDRAMYDGFVQL
LHYFGWTWVGLVASDEEHHLKISQELRAVLLKSGICIAYFAVFPTEGLFNFIPVFEIIEKSNA
NVVITLCSQFSFLDIIYTVAIRGELGKVWITPSSLTFFTHNLIGTYLGVVNGSLQFSVHKEEIP
EFKNFLYSTNPTTFHNFFTAAFWNELFHCLPPNKTTYWKIVNPLHNCTGEESLNNVDANL
DVKTLRVTYTVYRAVYSFASALHEMLSSTQYSGQKLNLMKNFQPSQLSQYLKNLDFQTK
DFHSANQGQIGHYDIINWVTSNRTITFSQVGSFLPFAPPGQQLIINKSDIKWNRKFNETPLSL
CTNHCPPGFRKAARKAEPIFFCSHYESCAKSRFFRSVNTCAKNMEECMACSEDHWSDEK
RTMCISRTIDFLSFSDVLGITLAFVAIFLCLISLGVLSIFLKYRNTAIVKANNRELSYILLLSLI
MCFLCCLLFIGRPEASTCFFRQAAFGIIFAVAVSAILAKTITVVIAFNATKPGSRARKWVGSN
ISAYLVSLGAFGEAVICLVWFLCSPPFPDFDTTEKSAKMTLKCNEGSEVAFYCVVGYVGLL
AALSFVVAFLARKLPDTFNEATHITFSMLVFCIVWVSFIPAHVSAKGKYTVAVEIFAILASTA
GLLGCIFIPKCHIIVIRPDLNTKEHLIGRKVKRCIVVN*
>jgi|Xentr4|361817|e_gw1.305.12.1
FYFRAFVRYFRHVLVIIFAVEEINRNPLLLPNVTLGYEIYDSCDYVSKAVEATLKLFSGRQD
HVPGYRCTTKGPLAAFIGLPYSMAELTQIYRYPHISYGDRNPVLDDKTLFPSFFRIVPNDHS
QYDGFVQLLHYFSWTWVGLVASDEEHHLRTSQELRAVLHKSGICIAYFEVVSTQYVFSYL
HVFNVIEKSRANVVIPFCSHFIFLNLIYSAAQNAQHRRVWIIPASFSLFTDTLLLSVLSVLDG
SLQFSMHREEIPGFQDFIYSINPTTFSSFLTADFWNDLFLCLPPNKNTHRRFVDPKNCTGNE
SLKSFDNISEIKTFRVTYAIYRAVYSFAIALHKMLSSMQLSGHQLNLRNDFQPTKLSQYFKN
VHLQTKPRKKLYSANEGPIGQYDIMNWVTSKGTISLNQIGSFFPFVPPGQQLIINKSDIKWN
HKFKQAPESICSESCPPGYRKAPQKGQPACCFDCILCSEDEFSNETDSTSCMKCPEDMWAK
EMHDGCRNKTLEFLSFEEILGGTLAALSITGSLLPLSILFIFLKNANTRIVKANNRNLSYTLL
VSLFFCFLCALMFIGRPITITCILRQFIFGISFVMCISCVLGKTMMVVIAFNLTQPRSSRRMW
LNSRVTNTLVLVCTAIQVIICAGWLAHSPPFQYTDNKSKMGTIIVECNKGSPIAYSCTMGY
MGFLASLCFVVAYLARKLPGSFNEAKLITFSMLIFGAVWISFIPAYLSTSGKYMVAVEVFAIL
SSSSGLVACIFLPKAYIIILRPEMNNIEMKRT*
>jgi|Xentr4|361823|e_gw1.305.217.1
MLFHAATLGDLVVLKPRTVLIYILVLCVGPCRSGIQPLNPACRLEIIKAAKEYDYIQEGDIM
VGGAMTAHLYMINVTFPRENSKRFLCTDPSQQDFRHFINFRFAVEQTNKDPARLPNLTLGY
DISDSCGDPKKAVRSVLQILSGTREPVPNYSCVGKRNIAGFIGDEASETTVPIAQILSIYGLS
QISYGATDQSLRDRVAFPYFFRTVQSEETSYLALGKLLRHFGWTWVGIITSDDISGEREHQI

LANYLSREGVCIEFTMRIKTNNDMIDTDTLQKSFTSVILLCGTVSFYLLVTLILIKSVFLKKT
WILSTNWGTNDVVVQYDQELFNCSLGLVPSYHYQLDTPEMRNFLDNLHPSNYPEDDLLK
NIWMIFHLCLSEDPNKNHLYEYIYLKTLHNCTGQERITDIPYFTADFSSLQVHLAVDLMSQ
ALHDMNIRLSEKTVKKKEMYHYSFFLPRAPFTQLRALLLPLPASAVMSQTQHYMCAFIKL
RPVLTDVTHTGRNPIKVHMGGPWGKLKDAYGALEEAGGFWGGSVKKQRRAGHCMGTL
GEDHNVLGGPGLASNVLVGPCPGTNAKCNPWPQICFPCPDMEWPNEKKDHCIASREDFL
SFTNDTISVLCSSISILFFLITSWILGVFITYRDSPIVRANNRSLSFLLLVSIKLSFLSVFLFLGR
PVDITCMLRIITFGITFSIAVSSLLAKTIMVCVAFKATKPGSSWRKWLGVKLSNSVVLFCSSI
QIIICMTWLAISPPFQELDIHTSPGTIIIQCNEGSAIGFYSVIGYMGLLAAVSFVLAFLARSLP
DSFNEAKYITFSMLLFCSVWITMIPAYLSTKGKNTVCVEIFAILTSSAGLLACIFLPKCYIILIR
PEMNTKTYLLGNKDN*
>jgi|Xentr4|361849|e_gw1.305.158.1
IPRAQCTDNCLPGYRKLPIPEAPPCCYHCVPCSEGEISNITDSVSCTQCPDMEWPNEMRNQ
CILREEVFLSYTNDVISIFFSAASVIYFLITQLIIVVFIKHMDSPIVRANNRSLSFLLLVSIKLSF
LSVFLFLGRPVDITCMLRIITFGITFSIAVSSLLAKTIMVCVAFKATKPGSSWRKWLGVKLSN
SVVLFCSSIQIIICMTWLAISPPFQELDIHTSPGTIIIQCNEGSAIGFYSVIGYMGLLAAVSFVL
AFLARSLPDSFNEAKYITFSMLLFCSVWITMIPAYLSTKGKNTVCVEIFAILTSSAGLLACIF
LPKCYTILLRPEMNMKTNLLGNK*
>jgi|Xentr4|362276|e_gw1.309.20.1
MGCPGWPLALFALLLASCSGGPSGVSSPAPCPAPCACDLDGGADCSGKGLVTVPDGLSVF
THSLDLSMNNITKLPEGAFKGFPYLEELRLAGNDLSIIHPMALSGLKELKVLTLQNNQLKT
VPSESLKGLVSLQSLRLDANHIVTVPEDSFEGLVQLRHLWLDDNSLTEVPIRPLSNLPSLQA
LTLALNKISHIPDYAFSNLSSLVVLHLHNNKIRTLGPHCFHGLDNLEALSKLQKDHLSIKPQ
VRSILDDRSHTCFHSNSITIIPDGAFVKNPLLRTIHLYDNPLSFVGNSAFQNLSDLHFLIIRGA
SNVQWFPNLTGTNNLESLTLTGTKIRSIPIKFCQEQKMLRTLDLSYNEISALVGFEGCSSLEE
VYLQNNQIQEVQNETFQGLAALRMLDLSRNRIHTIHKEAFVTLKALTNLDLSFNDLTAFPT
AGLHGLNQLKLTGNPNFKETLTAKDLIKLSSVSVPYAYQCCAFSACNSYMTTTVEEDRLR
AQRLLLDHDRAAMDPDYMGTEDDKEHVQALIQCNPATGPFKPCEYLLGSWMIRLTVWFI
FLLALIFNVIVIVTMFASCSQLTSSKLFIGLIAVSNLFMGVYTGTLTVLDTISWGQFAEFGIW
WETGNGCKVAGFLAIFSSESAIFFLMLAAIERSLSAKDIIKKEKHQHLRKFQVASLLAVLLA
AAAGCLPLFHIGEFSSSPLCLPFPTGETPSLGFTVTLVLLNSLAFLIMVITYTKLYCTIEKEDL
SENAESSMIKHVAWLIFTNCIFFCPVAFFSFAPLITAIYISPEIMKSVTLIFLPLPACLNPVLYVF
FNPKFKEDWKLLRWRLTKRSGSVAVATNSQRGCVTQDFYYDFGMYSHLQGGNFAVCDYC
ESVLLKNPPPCKHLIKSHSCPTLAVVPCQRPDNYWSEFGTQSAHSDCADEEDSFVSDSSDQ
VQVCGRACFYQSRGLPLVRYAYNIPRMKD*
>jgi|Xentr4|362572|e_gw1.311.158.1
MSNSCCVFEAPILDQVLPPVLLFEFVLGLVGNSIGLWMICRQVKSWKPYSVYLFSLTLADF
LVLFSVLFRADYYMRKKDWIYGDMPCRICLFTISACRSAGIIFLTIIAIDRYCKILFPFHRVNS
ITVKEAGIFCFLMWLGILVLYSYILTGSHSVKMDNSTQCESFQICPKNFSLADLHDGLYILM
SIGSLVIMSYSTVCIAQHLKNNTIDKDGKIRRAVRCVLSITLVYTVCYLPSTLVRISVWMLK
LQKHGDCAAYTDASLAFYATICFTYFYSMLNPIVYYFSSTSPSHFNQTLLAKICCRKCSYC
CEN*
>jgi|Xentr4|361825|e_gw1.305.209.1
MLEGAAASKSLKVLIYIITLCVGPCKSGPMNPACRLQIIKAAEEYEYIQEGDIMIGGVMTV

DMSMAILTPPWEKSEIKPQLICLDFNQQNIKHLVDLRFAVEQTNKDPTQFPNLTLGYHIYDS
CGNQLKAVRSVLQILSGTREPVPNYSCVGKRNIAGFIGDLTSETTVPIAQILSIYGYSQISYG
ATDPSLSDRITFPYFFRTVQSNKAEFHALGQLLRHFGWTWVGVITSDDMSGEEEYNLLAK
YLSREGICIEFTIRLHTIIGTAYSITDKYTKIISSSSTNVIIICGTVTLKLFIYLTNVNDYYRKKT
VILASNWGANDIVGYNPILLNNSLVLVPGYHYDLDTPQMRDFLQHFHPSNYPEDKLLEDI
WLIYFYCLSKDPNKNKICEYLYVRDKPLCNCTGQERITDLPYFRGKHNSPRVHLAVDMMS
RALHDMNMELEAKSVRNGDRLVYKSELHHYLKKVLLQSKHGEIFSFDENGEYMTKHQIY
SNLVTPDSKLIRNFVGEFSPWAPVDQQLNITSMAIPWKTTDNTVPKAQCTDNCLPGYRKL
PIPGAQSCCYHCVPCSEGEISNITDSVSCTQCPDMEWPNEMRNQCIQREEVFLSYTNDVISI
FFSAFSVLFFLITQLILQVFITYRDTPIVRANNRSLSFLLLVSIKLSSSCGSVCEWIGQCGSVC
EWIGQCGSVCEWIGQCGSVCEWIGQCGSVCEWIGQCGSELDIHTSPGTIIIQCNEGSAIGFY
SVIGYMGLLAAVSFVLAFLARSLPDSFNEAKYITFSMLLFCSVWITMIPAYLSTKGKNTVC
VEIFAILTSSAGLLASIFLPKCYIMMFRPEMNTKTHLHGNK*
>jgi|Xentr4|361643|e_gw1.305.22.1
MGQIIFI*RIIIIII*EFLFFRLSLRYYRHILAVFFAINEINENPKLLPNITLGYEIYDSCSYETKAL
EGTFKLISRRQGAIPSYQCSAKGIIAGVIGDISSSSTYNMAQLTGIYMYPQISYGAQDPVLD
AKSLFPSFFRMLPNDKTQYDAFVQLCLQFGWTWVGLVATDDERNVQSCEEIRTELLKNGI
CIAYFEIITLTLLGSYKTTVNNMMQSTANVVIVYCSSVNFFEFIYSAQILGMSGKVFIIPTVL
SVAADSIFSSFLSVLNGSLLFSIHKGEIPGLKDFLYSVSPYTLPNEYFTAATWNYALNCLPAN
ATEFRIKFGPMHNCTGKESLKVFDLAYDVNTFRITYAAYRAVYAFASALHDMISSMQNKY
EFILNMKKTFHPSVLNKYLRNIHFQTKSGEEFYFVDGTPAGQYDIINWVIAPNSSVINTQV
GSFHYSAPKGQQFVINKNEIMWNPTFNQTPRSVCSESCPPGYRKAARKSEPICCYDCVACS
EGEISNSTDMENCITCAEDQWSNENRTMCISKTIDFLSYEDALGIALSVTAIFLSIVPLGVLG
IFLKHSETAIVKANNRELSYILLLSLSMCSLCSLLFIGRPVALSCFFRQAAFGIIFTVAVSSILS
KTITVVIAFNATKPGSQARKWVGSRVSTYLVSLGAFGEAVICLVWFLCSPPFPDFDTSENSA
KMTLKCNEGSEIAFYCVTGYVGLLAALSFVVAFLARNLPDNFNEATYITFSMLVFFSVWV
SFIPAYLSTKGKYMVAVEIFAILASTAGLVSCIFAPKCYIIVIRPELNTKEHLIGKKVNRYIVEP
A*
>jgi|Xentr4|361830|e_gw1.305.212.1
MAVCNSLIELIYLIMLCVGPCRSGALPANPACHLEIVKTDEEYEYIKEGDIMIGGVMTVIFW
VLALGSGRALLCNVGSLGSNYRYLVEFLFLVKELNDNPSRLPNVTLGYHIHDSCGASERA
LMNVFKILSGTREPVPNYSCVAMRNIVGFIGDLESQPTIYMASILGVLGYSQISYGATDPSL
SDRVTFPYFYRTVQSDEEEYIALCKLLKYFGWNWIGILRSDDYSGYKDHQLLIKSLSREGI
CVAFTISVREKLSIDEFQKKIIALYSASVIIICGDVGISNLKAFSTEFYYCRGKTFIFLSKWLN
HVDVLNSYVLELFHGSLLFMQKRRNDQFVPRFKEFSDRYKPSNYPEDKLLENMWLWRFH
CLSKDKSKNAYHRFMFRFLYNCSGNEKLTDLPAYLVPYHSASLMNAVEIMSVALTHMQNS
LPKQTSQYKHMYNYQLHKYLRNVSYTSDGSPALSFTEKGDFNYEYQIVNPQLGSDKTWT
WNTVGNYVPWAQRLTLTQEKIRWKTPNNKLPISWCSESCVPGYRKVTRPGIYMCCYDCV
WCPEGEISNTTGKQYKGKVQPMVLQRCNRTGTAYNDGIFPSLYGTGIRHGDKGTDLFSAG
KLCLLLPTPIAGTENREPDLLTSAGILIGGFFCISMLPLALGSLKSFIVQYCFKNTSFPSLLPM
TLIAYLVPRAGPPVRRKLHQPGLSVPSASSFLFPLLANVWDNRMCSRVKSRLHSLSSAFSL
YCACAHERTGRGKRSQLPRAVIGYMGLLAAVSFVLAFLARSLPDSFNEAKYITFSMLLFCS
VWITMIPAYLSTKGKNTVCVEIFAILTSSAGLLACIFLPKCYTILLRPEMNTKNNLLGNKDN
*

>jgi|Xentr4|362888|e_gw1.315.157.1
MDRSNNTAVGEFILLAFSDLNELQIPLFFVTLPVYIMCVFGNVAIIFLVTVEPSLHTPMYFFIS
KLSVLEIMYVTSIVPNLLANLIADMKSISFGGCFIQLFANSILGTVECYLFAVMAFDRDLAI
NKPLHYYAIMTKELCVQLTVVVWIVGIIHVSISVVFTANLEFCGPNEIDHFFCDFPLLQSLA
CSHPFITEVILICGAFLGAVLPFIATVGLYTHIIIVICKIKSAESKQKAFSTLSSHLTVAGLYYIT
VIFVHSVPTGTQYNKFLALVYAVIIPLLNPFIYAFRNKDIKKALIKLRRLKLCQGY*
>jgi|Xentr4|362919|e_gw1.315.192.1
MDKANKTTVEEFILLAFSDLYQLQIPLFFIILPVYIMCVFGNCLIIIIVRAEPSLHTPMYFFISA
LSALDIMFVSAIIPNLLANLIADKKKISFSGCFIQLFVGSTVGSTESYLLTVMAFDRYLAINK
PLQYVSIMTQQVCVQLALLPFIAGIVVSLILTVFTAELEFCGPNEIHHFFCDFASLQALACSD
SFNSQVVTSSGASFAIVLPFITTVGFYIHIIIVISKINSDKSKWKAFSTCSSHLTVTGLFYITTI
VVYATPRGTQYDRFFAIIYNVITPLLNPFIYTFRNKDVKRALMISKRLKLCQGSFQY*
>jgi|Xentr4|362930|e_gw1.315.41.1
MAQTNTTADKDFILLAFTDLHQFQILLFLVFLLIYITCMVGNIAIILIIKMDTCLHTPMYIFIS
LFAASEYFFMSSTVPNLLSNLIGNTKSISFAGCFAQFYAVCTLGGTECYLLAVMAFDRDLAI
NFPLRYSAIMSQSLCIKLGIFPWILCLSIASLPTIFTLGMEFCGPKELNHFICDLGPLEHIACS
VPFINKMIVIFTALSEIVSPFIITIVFYIHIITTILNIRSTIGKKKAFSTCSSHLMVASLFYSTAIIV
YMTPQGSHEKYLALIYNVVTPLINPFIYTLRNKDVIKAFKKLKAKLS*
>jgi|Xentr4|362936|e_gw1.315.217.1
MDKANNTAVEEFILLAFSDLYQLQIPLFCVTLLVYIMFVFGNCLIVIIVRAEPSLHTPMYIFIS
TLSALELISVSLIIPNLLANLIADKKKISFNACFIQLFVTATFGTAENYLLAVMAFDRVLAINK
PLQYVSIMTQGVCVQLSVLPFILGTINALIPTVFTAVLVFCGPNEVHHFFCDFATLQALACSS
PYTSQMVTNSGAIFASGLPFTITVGLYIYIIVIILKINSAESKRKAFSTLSSHLTVAGLFYITTII
VYAVPKGTQYDRFFALIYTVITPLLNPFIYTFRNKDVKRALIKSRRLKLCQGSFRY*
>jgi|Xentr4|362945|e_gw1.315.139.1
MNRTQFNGFILLGLSDIEDIRILLFVFFLSMFLLTLTANMLIIMAVQCHARLHTPMYFYIKVL
SFLEIWYTSSTAPKHLSLLLTKDNRISYGWCFAQLYMFHSLGTTDCALLAVMAFDRCMAIC
NPLRYTTIMNDRMCRCLATLSWAFGFLTISIPLAMTIKVPLCRAHIIDHYFCDLAPLLALAC
TDISFTIAINSSVIGFAIMFNFIFILIMYINIIWAIMKLQTNAGRMKAFSTCSSHLIIVAIIYGSAF
SVYGSPKGPQTANYEKLFSLVYTVFTPFLNPIIFSLRNNEMKIALRGIFQGKWHILMLK*
>jgi|Xentr4|362948|e_gw1.315.220.1
MDKANKTTVEEFILLAFSDLYQLQILLFIVTLLVYIMCVFGNCLIVIIVRAEPSLHTPMYFFIS
TLSALDIIYVSLVIPNLLANLIAAKKSISFNGCFIQLFVGATLGTAEGYLLAVMAFDRVLAIN
KPLQYVSIMTRQVCVQLALLPFIVGIVIALIPTVFTAGLEFCGPNEVHHFLCDFGPLQALAC
SRPYMSQMVTNSGAIFAFGLPFVITVGLYIHIIIIISNIKSSESKRKAFSTCSSHLTVAGLFYITT
IIVYAVPKGTQYDKFFALIYTVITPLLNPFIYTLRNKDVKRALINSRRLKLCQGSF*
>jgi|Xentr4|362959|e_gw1.315.187.1
MDKANNTAVEEFILLAFSDLYQLQILLFIVTLLVYIMCVFGNCLIVIIVMVKPSLHTPMYFFII
ALSTLEIMLVSTIIPNLLANLIAAKKSISFNGCFIQLFVGATLGTAESYLLAVMAFDRVLAIN
KPLQYVSIMTQRVCVQLALLPFIVGIVIALIPTVLTAGLEFCGPNEVHHFFCDFATLQALACS
RPYMSQMVTNSGAIFAFGLPFTITVGLYIHIIVIISKIKSSESKQKAFSTLSSHLTVAGLYYITT
IIVYTAPKGTQYDRFFGLIYTVITPLLNPFIYTFRNKDVKRALIKSRRLNLCQGSF*
>jgi|Xentr4|362971|e_gw1.315.190.1
MDKANKTTVEEFILLAFSDLSQLQIPLFFIIFNAFTICLLGNIVIIIIVRVEPSLHTPMYFFISTL

SVFETMFASSIVPNLLANLIAAKRSISYNGCFIQLFVGATLGLAECYLLAVMAFDRVLAINK
PLQYVSIMTQRFCVQLALLPLIVGIVFASAFTILTAELQFCGPNEVHHFFCDFASLLSLACSN
IYMSQIVLNSISTFATGLPFTITVGLCIHIIIIISKINSAESKRKAFSTCSSHLTVAGLYYITTFIVY
AVPKGSYYDRFFALIYTVITPLLNPFIYTFRNKDVKRALIKPRLKLCQGLL*
>jgi|Xentr4|362974|e_gw1.315.28.1
MEEMNHTQFSSFILRGFSDIEDIRILLFVFFLSLFLLTLTGNMVVIMVVQSHARLHTPMYFFI
TVLSGLEMWYTMTTAPKLLSLLLTKDNSISYGWCFAQLYMFHSLGMTECAVLAVMAFDR
CMAICNPLRYTIIMNDRMCRALASLSWTFGFLAATIPLTMTIKVPLCRAHIIDHYFCDLAPL
LALACADISFTNTINRCVIGFATMFNFMFILVMYINIIWAIMKLQSNTGRMKAFSTCSSHLIV
VALIYGTAFSVYGSPNKSQIVNYDKLFSLVYTVFTPFLNPFIYSLRNNEVKIALRGIIKGKIDI
FRLK*
>jgi|Xentr4|363013|e_gw1.315.132.1
MLPQLVGNQIQPECALTFPSSDRYYQEGDYIFGGLITVMTIMAQMKATFECRHFKDSIYIHE
NINLQKQVNYQNLLAFVFAVTEINKNPRLLPNITLGFHIVDPCLSEEKAMTGMIDIFSGKM
VPIPNYRCGLSPNLVAVVDGISSKVTLLIARVFGIYQIPQISYASMDPVLSDKNQFPSFYRTV
PNEIAQYDALVKLIKHFSWTWVGVLVSDNESGLKMSQMLQKEFALNGICFAFLEFIPYHD
GLDYPKKIQISKSLSNSAADVIIAYGDRDYMLSLQLILYESPISEKVWIISCQWDVASGFDY
YFLRFDPLNGSLAFSLHTSSIPGFDDFLLTLTPNAFPNDIFINYAWWELYYCVWKTNKTDIR
CNGTEEMHKEYESVYKDVSVYSYSIYNAVYTLAYALHYLQEKEENHSVHNIQAWEMHK
YIKKVSITDSDGNELHFDENGDMPSDFDILNWIVYPNQTLNGIKVGTYAQRSASQELRINE
SLIRWSPSFNETPRSACSETCRTGYKKKPKVGFPVCCYDCVPCPEGEISNQTDRNFCLKCPI
NQWPNLNRTICTDKVIIYLSYHEPLGASLALLSVLFCIMTCLVLMIFTKYRNTAVVKANNR
DLSYILLVSLKICFLCNLMFIGHPRHVTCILRQTVFGVAFSIVISSVLAKTVTVVIAFNATRP
GSKLKNYLGPRVSNSIVIFCSLMQVIICTSGLINSPPFPYYNMEDIVGVIVAECHEQSVGFYC
ILGFMGLLAALSFIIAFFARTLPDIFNEAKFITFSMLLFCSVWVSFIPAYLSTKGKYVVAVEIF
AILSSSSGILGCIFIPKCYIILFRADKNKKNYITKDSSHLCQ*
>jgi|Xentr4|363029|e_gw1.315.218.1
MDKANKTAVEEFILLAFSDLQVPLFFVTLLVYIMCVFGNFLIIVIVIAGPSLHTPMYFFISKL
SVLEIMFVTCIVPNLLANLIAGKKSISFSGCFIQLLFSSTMGTAECYLLAVMAFDRDLAISKP
LHYFNIMTKELCVRLAVLPWIVGIITVFISTVCTASLEFCGPNEIDHFFCDFYALQSLACSST
FITQVVTNSCAIFAVVVPFFLTVGLYIHIIFIILKIKSSESKQKAFSTLSSHLTVAGLYYITVIIVY
AVPKDSHNNKFLALIYTVIVPLLNPFIYAFRNKDIKKTLIKSRRIKLCQGSF*
>jgi|Xentr4|363045|e_gw1.315.13.1
MSGKGLPIPNYRWGLSPNLVAVVDGISSKVTLLIARMFGIYRFPQTPRTPCSETCLIGFRKKP
KEGFPECCNDCAPCPEGEISNQTGEETCFKCPINQWPNLNRTICTDKVIIYLSYHEPLGASL
ALLSVLFCIMTCLVLMIFTKYRNTAVVKANNRDLSYILLVSLKICFLCNLMFIGHPRHVTCI
LRQTVFGVIFSIVISSVLAKTVTVVIAFNATRPGSKLKNYLGTRVSNSIVIFCSLMQVIICTS
WLINSPPFPYYNTDDIVGMIVAECHEHSSYGFYCILGFMGLLAALSFIIAFFARTLPDIFNEA
KFITFSMLLFCSVWVSFIPAYLSTKGKYVVAVEIFAILSSSSGILGCIFIPKCYIILFRTDKNKT
DLKKRTHIN*
>jgi|Xentr4|363060|e_gw1.315.2.1
SDRLNVWNYQNVLAFVFAITEINKNPYLLPNITLGFHIVDNCLNEEQAMFGIIDVLCGGNM
RIPNYRCLLPYNLVAVVDGISTRLTLLLARILGIYKIPQISYSSLDPKLRDKIQFPSFYQTVPS
DDFQYLAIVKLLKYFSWTWVGILVSADEFGLKISQLLLEELARNGICVTFLEFFTKGEMQN

ESRKKVAERVNSTNVIILYCDRDYVSELQSILYLFPVSGKVWITSSQWDVLIGNDHYFKGL
QPFNGSLAFTPHKGDVPGFKEFISGIKPDLYPSDIFIFSAWVELYQCAYNVTIGFGTDTCTGE
EINQYESFSEEIAYHSYSIYNAVYALAHALHVMQLEKRRQGSNGHVIKPWEINKYLRDISF
TNSGGDTLSFDKYGEIPSKFDVLNWVLSPNDNLHSIKVGSYDQLNGLTINESLIRWHPAFS
QTPRSSCSETCIYGYRKTSKAGYPACCYDCIPCPDGEITNQTGKGTDTICIKCHQTQWHNE
KKEKCVNKYIIYLSYGEALGTSMTSVSVFLFVLTCLVMGVFTKYQNTPIVKANNRNLSYV
LLFSLKMCFLCSLIFIGQPIKLTCMTRQTVFAITFSISLSSILAKTITVVIVFHATKPGSKLNNY
IGSKGSVSFIGFCSLVQVVICACWLGISPPFPQYNMEDEVGKIIAECNEGSLIGFYCVLGYL
GVLASVSFIIAFLARDLPDTFNEAKFITFSMLVFCSVWVSFIPAYMSAKGKYVVAVEIFAILA
SSLALLGCIFIPKCYIILVKPECNTRDFIKKKDKNIIIHHQ*
>jgi|Xentr4|363079|e_gw1.315.224.1
MESLNKTSVSEFILLGITNIEHLKIVLFITFFFFYMFILLGNLSILAAVIIDRGLHRPMYFFLAN
LSCLDLFFSSTTVPKMLAGLMMGDMRISFQSCMVQLYFFHLFGCTEALLLTLMSYDRYIAI
CNPLRYQVLMSNRVYVQMASYCWVIGFVYSFSHTILTSRLPYCNMNKITHFYCDIKPLLK
LACANTKLNERLLTIISGFVSTSTFILVMISYIIIGSHLLNMPVGSKQSYSKAFSTCTSHMVV
VLLYFGIAMCTYLGPSNKETLEQDRLTALLVTVITPALNPVIYTLRNKEMKTALKRTFFKR
KSLRKF*
>jgi|Xentr4|363093|e_gw1.315.72.1
MDKANRTSVEEFILLAFSDLYQLQIPLFFAMLLIYITCVSGNLAIIFLVRVEPSLHTPMYFFIS
TLAVTEMMFVSCIIPNLLANLIAAKNRISLIGCFTQLFATDTLGTAECYLLAVMAFDRNLAIS
NPLQYSAIMTQKLCVQLAVLPGIVGIVTLSIPFVLTARLEFCGPNEVNHFFCDFAPLQSLAC
SDPFITKVATSSGAFFAIVLPFIATVGLYIHIIVIIFQIHSTESKQKAFSTCSSHLTVAGLHYITAI
TVYVVPKGSQYDRFLALIYAIMTPLLNPFIYTFRNKDVKRALIKSRRLKLCQESF*
>jgi|Xentr4|363119|e_gw1.315.19.1
MDKANKTTVEEFILLAFSDLYQFQILLFIVVFIAFIISLLGNIVIIVIVRVEPSLHTPMYCFISTL
SIWEIISVVLIIPKLLANLIASNKSISFSGCFLQMFVAATVGTAECYLLAVMAFDRVLAINKPL
QYVSIMTQRLCVQLALLPFIVGIVIALTPTVLTAGLQFCGPNEVHHFFCDFAALLSLACSSIY
MSQMVLNSSATFAIGLPFLITVGFYIHIIIIILKINSAESKRKAFSTCSSHLTVAGLFYITTFIVY
AVPKNSRYDRFFALIYTVITPILNPFIYT
>jgi|Xentr4|363134|e_gw1.316.113.1
MLAALLSNVVVLICFLGSADIRRQVPGLFTLNLTLCNLLLSGINMPLTLVGLIHGSQPGGEG
LCRAAGFLETFLTSNSMLSMAALSIDRWVAVIFPLSYHSKMRYQDAAVILLYSWIHSLAFPT
VALCLSWIGFHPIYGSCTLYNKRPEDEASFLAFIVLFHLLSFLLSFLVLCVTYLQVLKVARS
HCRRIDVITMQTLVLLVDLHPSVRQRCLEEQKRRRQRATKKISTFIGTFLLCFSPYVITRLVE
ISSSTPISSHWGIVSRCLAYSKALSDPFVYCLLRNQYKNCCRDLVNKLLKRSSANSAAQRM
NSYVGSLVLTTEQ*
>jgi|Xentr4|364022|e_gw1.325.172.1
MFRVINITLYSIIFILGTAGNGLVIWIIGFKMEKTATLIWFLNLAIADFSFCLFVPLSITEWALW
LYWPFGQIMCKTWIFNLQLNLSASVLFLMIISVDRCICVLYPIWAKIHRTSRLALTISVVIWF
LSVGLSSPYIVFSDTENVKQFTECIFISAVGNNSTTFDYDTFMARYKAMLMTRFVSMFLIPF
PIMLVCYGLIAFQVRKNSRIPGSGRTLKMIFTIVICFFLCWFLYHSVPMIQIAHIYIRYPWDVI
LFNLAYCLAFFNSCLNPIIYIFIGRDFKKSLRKSIPFLLESTFRETNDPSKILNNHRKSETMTA
GFLLAQRKDCNAS*
>jgi|Xentr4|364037|e_gw1.325.95.1

MSITCFSLTFVLGILGNGLVIWITGFQMRKTMTTTWFLNLGISDFSFCLFLPLYITEAARWG
NWPFGQIMCKVTYFTTGLNQCASLLFLATISMDRCICVLYPIRSRSSRTATLAAIISVIIWLLS
VALSFPYIFFTDIQNSNNFSVCFLTNGSLENITTIHEEMLSFSFPAIVISDFVFTFLIPFPIILVCY
GLIAFTVRKSKRIPESFRTLKIILTTVLCFFFCWVLYHVLPVIDIAGHYMPWPRKFFLYSLAE
CLAFSNTCLNPIIYVFIARDFQQSLKKSIPFLLESTFKEKNDTPEVLEDNNVL*
>jgi|Xentr4|364047|e_gw1.325.119.1
MSVSSVSEIYQNTTAQGSDTTVPTANILCTLILLITFLFGLVVNVLYLWILGLKMSKSVNTA
MIFHLILVNLVFTMTMPLLSVYMLTFPQWILGDFMCKLVNSLLSLCIYTTVFFLTVISLDRY
TLVFHPVWYRGHMNHRYASVTCILLWGFAALCSSPYFAFRQIRLLKDNKTAICYNDYTLS
GKWDGELLKWFMFGFRLVLGFLIPLSVISVCYLKLALRMKKENLARSNKPYRIIFIFITSFI
MCWTPYYLWYGMSMEQEKFPKATLNVLMVMTICLICLYYCFTPVLYLFAVKNFKKEFRK
SIMSLFESALTEAFRS*
>jgi|Xentr4|364064|e_gw1.325.114.1
MNATTDSEDAELRHVMQVLVTVIFSFIFLLGMMGNGTVIWITSCRLRRTINTVWFFNLAL
ADFISTFLVLVTVLYLLLDLHWPFGPILCKLVNCIFGLSIYASILLLTAISIDRCVFVLFPVWC
QNYRNPRLAATTCLVIWVVSVALVPGSYTISEMSTEKNRSSCKNLDVFEDWEKREAGIFTV
CIFLYQFLVPLSIILISYTILLLTLHKKKLNRSSKPLLVVTGVVFSFFLCWLPYHALAMARVF
LGGLSLWVSLVGVPLSKCLAMFNSCINPLLYVFVGREFKDEVKRSLTQVFRAAFEELPQ*
>jgi|Xentr4|364072|e_gw1.325.61.1
MHISSMLVYSVAFLLGTTGNGLVIWIAGFKMKKTVNIIWFVNLAIADFIFTLFLPLSVAYIAL
DFHWPFGTFMCKLNSSIAFINLFASVFLLTVISVDRCISVVFPVWSQNHRTPRLASFVALAV
WFFALCFSLPYFIFRDTFRHRDFVSCYNNFGYDKDNNFTDLGIQRIKATVIIRFLVGFLIPFS
VIIFCYTVIALKLQRNRMATSSKPFKIIIAVLISFFVCWFPYHVFSFLEMTEYTNPNANYGTV
LLIGTPITSSLAFLNSCVNPFLYVFMGRDFKNKFMTSIQSVFEKAFSEDSLHTDTKQTKSTK
SVSESNLV*
>jgi|Xentr4|364088|e_gw1.325.178.1
MTSLFGLVVNSLYLWVLRFKMCKSVNTTWFSHLILGNSVFTLTVPFLAAYRLMSPHWIFG
GFLCKLINAAIVLCMYANIIFLTIISMDRYALVYHPVWYRGHMTHRFASAICISMWGFTILC
SAPYFAFRQIRLLDDNKTTICYTDYTLYGLWDNQPKSQIQMEWIVFFINLALSFLLPFCIIAV
CYLKIALRMKKGNLTRSSKPYKIIFITIVSFFIFFIPYHIWKGMSIEKGKFHKTTRDVLKVIM
TCSFCFHYCFTPMLYLFIVENFKKLLRKSVITLFETVFYEPVSS*
>jgi|Xentr4|364100|e_gw1.325.80.1
MENDTVYYVPGLSTFDTLAEEEIYSRIYPVFRIINITLYSIIFILGTAGNGLVIWIIGFKMEKTAT
LIWFLNLAIADFSFCLFLPLSITEWALWDNWPFGQIMCKTWAINQQLNLSASVLFLMIISID
RCICVLYPIWAKIHRSSKLALTISVIIWFLSVGLSSPYIVFSDTENVNQLTRCAFISAVGNNST
MFDYDTWNVRYKSMSMTEFVSMFLIPFPIMMVCYGLIAFRVRKNSRIPGSGRTLKIAFTIVI
CFFLCWFLYHSVPMIYTADIYIRYPWDEILYYLAYCLAYFNSCLNPIIYVFIGRDFKKSLRTSI
PFLLESTFRESNDPPEIKLIF*
>jgi|Xentr4|364138|e_gw1.325.168.1
MRIFSIICHSITCLVGVIGNGLVIWIAGFKMKSISAKWYLNLAITDFICSVSAVVRIAEWILVD
DYYLCPFSFTLLLINMLTSVYFLTAISIDRCITIMWPFWAKSHRTKRSATAVIVLVWMVSLLI
SVKSIFIYFEFHDLLECAPEQSGYYRVYNESKVEKFEALKHPFHVTRFVVMFLLPFTIILLC
YGLIICKVASVRRRNKSQRSLKIIIAIVTCFFGCWFPYNLWQFIALRTRNDHIGADLIISSVSV
CLVYTSSCLNPILYVFLGREFKSSLTRSIPAILKNVFSDPEDRLRRETVATNEVICEQIHM*

>jgi|Xentr4|364141|e_gw1.325.22.1
MRIFSIICHSITCLVGVIGNGLVIWIAGFKMKSISAKWYLNLAITDFICSVSTVVRIAEWILVD
NNVLCEYSFILLILNMLTSVYFLTAISIDRCITIMWPFWAKSHRTKKPAITVIVVIWVVSVLFS
VSNKYFVWGFRDLLMCAPEESRYYPVLKKSGIMEPYILEQTIHITRFVVMFLLPFTIILLCY
GLIICKVASVRRRNKYQRSLKIIIAIVTCFFGCWFPYNVWQFIALWRGDHIGVDLIISSVSVC
LVYTSSCLNPILYVFLGREFKSSLTRSIPAILKNVFSDPEEG*
>jgi|Xentr4|364144|e_gw1.325.14.1
MGYTSIPAANVLASVFLFVALLFGLVVNSLYLWVLRFRMRRSINTTWFLHLILSYFLFTFIIP
FIAVYMLMFPRWVFGDLLCKLTTSLINVCMYASVFLLTVISLDRYCLVFHPVWYRGHMNN
RYATAICICIWGLAILFSSPSLAFSYTRLLKDNKTATCYDNMLPGSIRQKLAIQLGWVMFSF
RLILSFLLPFAVMTVCYVRIALRMKKGNLARSTKPYKIMFITVAFFFLSWVPYHLWYRISG
RFHKSISDILMALAICLICMNYCFTPLLYLFIAENFKKTIQKSVLLLIESVFNEIFNSFNRSFEL
KPEAPPSSASKIGTREEPLEEIQ*
>jgi|Xentr4|364146|e_gw1.325.163.1
MEDEEDDFTDMIPSELETALHFLSIVIYSISFLLGTTGNGLVIWIAGFRMKRTVNTVWFVNL
AIADFIFTFFLPLSVVYTALDFHWPFGTLMCKLNSTIAFLNLFASVFLLTVISADRCVSVVRP
VWSQNHRTPRLASIVAFFIWLAALFLCSPYIAFRDTQRNTEDNVTHCYNNYAFSTDFVDEE
VIALRSMRHQVVIAVRFVFGFLLPFGLIVVFYSLMALKLRRSQMAWSSRPFRVMATVVVV
FFICWFPYHVLSVLEVVMHHTNNRTLKSAVLIGTPLATSLAFFNSCLNPFLYVFLGRDFKES
LRKSILSAFESAFSEEPGKTNESHVRSRSLSAQESHFT*
>jgi|Xentr4|364166|e_gw1.325.41.1
MTYTAIPTANILSTVILLVSNLFGLVVNSLYLWVLGFRMRRSINTTWFFHFILSNLLFTFIMP
FLAVYLRTDPSWVMGTFLCKSIHSLFSVCMYTSVYFLTVISLDRYCLVSHPVWYRGHRNN
RYATAICICIWVWAILICSPHFAFRQTRLLEDNKTICYNDYTLSGSIWPKSATQLGWWRLYH
LWVVVFHVILGFLLPFAVMTVCYVRIALRMKKGNLARSTKPYKIIFITVTSFFVSWFPYHL
WYGMSIEEGRFHKSTLKILMVLSVSLTCFNSCFTPVLYLFIAENFKKSLQKSVLLLIESVFN
ETFNSLNRSFEDKSDVPSSSAANDRTREEQ*
>jgi|Xentr4|364180|e_gw1.325.166.1
MQPLPFSSPSPDTYHEEEHENRIFFIRQILIITCYSIILILGTVGNGLVIWIAGFRMKKTVNTI
WFLNLGIADFSFCLCLPLHILDWAMDGRWHLGQLMCKFMFTSLFLNMSVSIFLLMTISVD
RCTSVLCPVWSKNHRSVKLATNISAIIWLLCLALSSPYLVFYDIEHYSNTTYCTVTYATWD
NATYFDHQTWFLRHKAMYVTRFISMFLIPFSIILVCYGLIVFWMRRSSKHLVSSRPFKIIVT
VVLCFFCCWFPYHLWPYLFEIIEVDMNWLFGLIMNDVANCLAYCNCCLNPMIYVFVGRDF
KRSLRKSIPFLLESTFRERGDPLETQDNTVGTELVAYHE*
>jgi|Xentr4|364184|e_gw1.325.20.1
MSSYNIANISADANVQEEGSPGGIYRSVEVMRIVWLSTTFILGTLGNGLVIWVFGFKMKK
TVNTIWFLNLAIVDFCFCLSLGFYIPVVILNHWPFGQIPCKLTTCILDLNMAVSVFFLAVISF
DRCLCVLYPIRSRSHRTLRLAVISSVMIWLLGLAVSSPSLVFTNVVHGGNISVCGSTYGNGA
DGSNVHSPTRQKVMILTRFVSVFLIPFTIILVCYGLISFRVRRSSRPPGSARTFKIIITVVLCFF
FCWFPFHLMFLKEITGFNIKWPYGYIISHMAICLAFFNSCLNPMVYVFVGQNFKNSLRKSI
PFLLENTFREACDPP*
>jgi|Xentr4|364411|e_gw1.328.133.1
MNNRTDRGTQLLNSVEQFARKLQIDNGSISIINNSNVQLAGIIANSSRSYNESFNFSQGNNL
TGSVLISKDSVSNMENNTNIITIAYATLKDILLNTSTKVVNGLVMTTVLSTQVPSNFKISMN

FKKSNQTLNVPGCVFWNLETNNWDSTGCQGVNNAENVSCDCDHLTSFSILMSYKAVDSI
VLDYITYIGLAISIISLLICITIEGLIWKSVTKNKTSYMRHVCLVNIAVSLLIADIWFIIGSAIST
LLKSDPLNTANSNACVSATFFTHFFYLSLFFWMLTMGLILFYRLVYVLHDMSKSRMMAIA
FTMGYGCPLLISIITVAVTQPGRTYLNGNACWLNFSDSKAFIAFVLPALTIILVNFIILAVVIIK
LLRPSVGDKPKKEDKSTLIHIGKSIAILTPLLGLTWGFGIATIIAPDALWVLGIFAALNSLQVS
SLPGNMTFTCLVENVFI*
>jgi|Xentr4|364447|e_gw1.328.123.1
MLHRSAQYNFFLFLSLCLFGRGLCNSEHISPSQNIFISSTGTQSTNSTCSIYTIQADSSQCPSQ
YSGSVTNYTCTFQGIYGATGSAAVQVTYLKPATVTITGPTSVSAQSSLSLRCQSDVSNIDRV
TWSINGLAIATDKQILNKDGSTLTVSPVTMDWNGTYLCTLYQQNLPSSGSCEVKVLNLPN
QQTIQVDPISIYQKCGNPQPLNCCIPLDSSSVYTVEFVPSAALQLGNQVTHGQLKCFSANY
TATVCPQTAVFTCVISNGITSVSSPQMQITYWSGKSFTPRFFTPALHNQKPSPNGAQIIIPCQ
NTNPSLTGNITYTCSNKQWTNQTNCTSVVVFNQYVAVQNLANGPAPAQQMPTFIQSLSNT
TQTNKETIASSPTNINTIVNILSIVSDNSKNVQPETMDNFISTVNTIVNNTQVWTTIRDKSSD
LLKSVENFAEQFTNVTSYTSNQSNTNIQLKGQTFSANTSYNQTFQLSGLSGGVTIGDGSLS
GNSCTVVSIAYSTLKDILPSDLNKTINGLVISTKVVQGTINEGHFNVSISFSQSNDSLTNPDC
VFWDFNISGWNNRSCSPSVKGNTTTVCTCNHLTSFAVLMGADDTNIPPEIIKFLEVITYVGL
GVSLACLFLTLVIEAIVWKSVIKNKTSYLRHVCLVNIAVSLLVADIFFLIGAHLERFPDTPAC
TAVGFLSFYFFLVLFFWMLTTGLILFYRLVYILHDTSRKTMMIIAFTLGYGCPAVISIITVAST
APRHGFTSGTYCWLNYTTTNSFLAFVVPVLTIVFVNFVILCVVIFKLLRPNVGERQSKDEK
KILVQITKSLAVLTPLLGLTWVFGLFVAAKPKNATYQILFTIFNSFQVCVINSIFIYSQCCQLH
WIKTSFSY*
>jgi|Xentr4|364472|e_gw1.328.134.1
MLATDNITVTSLVFNTIGDLLANSTTGPFNGTQLNSVIQSTSIKSAGSTNLSGEISMNFTTNV
SGSNYQQRCAFWDFSQPIAGGGWSDRGCTTNVEDSLTTCTCNHLTSFAVLMSINSEPLLFI
DEITYAGLGASILSLCVCIITECLVWKSVVRNNISYFRHISLVNIAVSLLFADTLFFAATFPSV
TNYKFICLSITFLNHFFYLALFFWTFCQSVMLLHQLLFVFHHLRKRIFLSLSFFVGYFIPALI
AAGTFLYFYPKNTYLHQSVCWLNPESGAIYTFAIPAGSIIMFNFMTLMVVISKLSRPSVSEA
NRPEDRETAKSIMKAVLVLTPVFGLTWSFGFALLKDLDSLTRQIFTYGFAGLNAFQVCLK*
>jgi|Xentr4|365159|e_gw1.333.197.1
SVTRECFENGTWASWMNYSQCVPILDNKRKYALHYKIALIINYLGHCISVLALVIAFLLFL
CLRSIRCLRNIIHWNLITTFILRNIMWFLLQMIDHRIHESNEVWCRCITTIYNYFVVTNFFW
MFVEGCYLHTAIVMTYSTDKLRKWVFLFIGWCIPSPIIVTWAVCKLFYENEQCWIGKEPGK
YIDYIYQGPVILVLLINFVFLFNIVRILMTKLRASTTSETIQYRKAVKATLVLLPLLGITYMLF
FVNPGEDDVSQIVFIYFNSFLQSFQGFFVSVFYCFLNGEVRSAARKRWHRWQDHHSLRVR
VARAMSIPTSPTRISFHSIKQTAAV*
>jgi|Xentr4|365578|e_gw1.338.6.1
MCSVIEDGNTDSVVTCWKSVLYNVLIGAVLVLIIIITICGNVVVCLAVGLDRKLRSMTNCFI
VSLAITDLLLGILVLPFSALNLLHEEWPFGATFCNIYTSLDVMLCTASILNLFMISLDRYYGV
TAPLRYSMHVTPFRVAIAMCVIWVVSLMVSFLPIHLGWNTKDKSIQNYRDSNDKECKLEL
NKEYVLVDGLLTFYLPLSIMCLMYYRIFKIAREQAKRINHANCCNALSPTLPTVREHKATV
TLAAVMGAFIICWFPYFTVFTYEGVNETDVDETAFLIVLWLGYANSALNPILYAALNRDFR
TAYQRLLHCRRVGPQNHETPLTLLHIRRSEDRTHQIMQNTQGEKLNMVHNMNGKEGRPFI
TNTVER*

>jgi|Xentr4|366231|e_gw1.345.62.1
MPNSTTEENCTLDHTIHQTLFPVVYITVLVVGLPANCLSLYYGYLQIKAKNELGIYLCNLTV
ADLLYIFSLPFWIQYVLQHDNWTYNEMMCKICGILLYENIYISIAFLCCISVDRYLALVHPF
RFYKLRTMKTALLISVVIWLKELMTSYFFFSHGEFTKDPESHIICFEHYPMKPWEHSINYYR
FFVGFLFPIFLLGFSYCCIFKVVRQSQGTQRKLKIQIKQLVLSTVIIFLVCFGPYHVLVVIRSV
F
>jgi|Xentr4|366352|e_gw1.347.12.1
MFKPLSLHNRISFRQYRHLLVLIYTIGEINKDPEILPNVTLGYRIYDSCASGIISFASAFGILSG
TEQPIPNYSCWNNRKVVGFIGDLSSESSLSIAWLAGIYRYPQVCIKVHCRRRKLYGRCHKC
QVMGHQVWNYKGSTVQKGFQGLLIAKKSECNPIYTKLCQESVTYISSGRVASVGWSQLL
LVPSHTPSVTAHSCFIFFSDNMWMLIDIFKVIWDHPVWPNPLNWNPKTALKTRSKQFPLQS
PFNGSFILTFHEGEIPGFKQFFYSLNPYTYQHDILFRTIWEMLFHCTFSKITSISFPQCTGNET
FDDTVLESYGTFNYRIAYGVYTAVYTMAHTLHELYGTMTRSPKSAESLHMYFKQWQLNA
LMPHVKFRTSSGDKIYFKDNGNPQARYDILKLYFLKTGNKKSIKVGSFDGSESDGKLFVN
NSTNLWGPYFTEFVHSRCSEPCNPGFSKAKVEGAPSCCYTCVLCADGEMSNITDAQSCMK
CSKYEKSNTGRTSCILRDINYLSYGDQLGATLSSISVTFSISCAVILGIFIKYRETPIVRANNR
YLSCLLLISLMLCFLCTLLFIGRPTQICCLLRQVTFGIVFTISVSSVLAKTLTVIIAFNATKPGS
KLKKYVGTQLATILVIVCSLVELVISAVWLASNPPFPEADTLSDPDYIILLCNERSGYFFFCII
GYIGTLALLSFIAAFLAKDFPDRFNEAKNITFSMLGFCSVWGAFVPAYLSSKGSRMVAVEIF
AILSSSAGLLGCIFIPKCYIIFLRPELNRKDNIVRKQ*
>jgi|Xentr4|366384|e_gw1.347.260.1
MEMGNRSEPTEFILLGLSSDSSVQTLLFCLFLTLCVSSLVANGLLIVAVKRDGHLHSSMYFF
LANLSIMDICYTSVIVPKALVNFLGGKKSISYNGCVLQIFFYLFMGESECILLAFMAYDRYV
AICSPLSYSTILSTAVCARMISASWVAGCLISALDVGFIYRLTFCGTNTIDHFFCEVPSLIQLA
CTNVTINVILMFAGGAILLLLPLLLILFSYIQIFIAAKRISCGRYKAFSTCAAHLIVVVLFYGT
ATFMYMRPRHSAQQTDKIVSVFYTVVTPMLNPLIYRLRNREVHGALRGLRRSSASPCHR*
>jgi|Xentr4|366389|e_gw1.347.236.1
QISYGSADPIFNNRLEFPSFYRMAPNELSEIDAIMSLIRHFGWKWVGLIVSNDDIGHRANK
RLQEAMSKDGVCLAFLIILKDWTQCNWAYVMEIRKTFYRSTAKVIILFLSSQSVSYISVLFN
DDKIPHTIWIATSSLSLISELKYLPALVTFNGTLVISLQQGEIPGFKQFFYSLNPYTYQRDTLF
PYIWGLLFDCTFSETDTSRKNCTGNETFDDTVLSFYETFNYRIAYGLGGGFGVSAYLCPGY
PWNYSRVTVTPMFLYICNLLNALMPHVTFRMSSGDKIYFKDNGNPQARYDIVKWYFLEIG
NKKSIKVGSFDGSESDGKLFVNDSANLWGPYFSEFVHSRCSEPCKPGFRKAKVEGAPSCC
YTCVLCADGEMSNITGTSDITGVKVSTKKFQLFLLMMLYHNLPPAFPAISTKMICCLLRQV
TFGIVFTISVSSVLAKTLTVIIAFNATKPGSKLKKYVGTQLAIILVIGCSLVEIVISNV*LASNP
PFPEADTLSDPDYIILLCNEGSGFFLFCIFGYIGTLALLSFIAAFLAKDFPDQFNEAKNITFSM
LGFCSVWGAFVPAYLSSKGSRMVAVEIFAILSSSAGLLGCIFIPKCYIIFLRPELNRKDNIVRK
Q*
>jgi|Xentr4|366410|e_gw1.347.26.1
SLHNRISFRHYRHLLVLIYTIGEINKDPEILPNVTLGYQIYDSCGLGIISFASAFGILSGTEQPI
PNYSCWNNRKVVGFIGDLSSVSSLSIAWLAGIYRYPQISYGSADPIFNNRLEFPSFYRMIPN
ELSEIDAIMNLIGHFGWKWVGLVVSDDDIGHRARERLQNQMNKDGGCLAFLIRLKNWSD
VNMSQVNITDINRSIAQTIYKTTANVIILFISSQYIESFNRLFALNKMPKKIWIVSTSFSRVIEL
RCSQILVTFNGTLALSFQQGEIPGFKQFFYSLNPYKYRDDTLFSKIWEIIFSCSFSDLAPGKT

TKISLPKCTGNETFNDTVLESYGTFNYRIAYGVYTAVYTMAHTLHELYGTMTRSPKSAESL
HMYFKQWQVRSFVHDFLKKSQILIETNKLRKALQLGTNYDILKWYFFGEGNIQSIKVGSF
DTSRSDGDQLFINNSANLWGPYFSEFVHSRCSEPCKPGFRKAKLKGQPSCCYTCVLCADG
EMSNITDAQSCTKCSKYEKSNSGRTSCIPRDINYLSYDDHLGSTFSSISVTFSISCAVILGIFIK
YRETPIVRANNRYLSCLLLISLMLCFLCTLLFIGCPTQICCLLRQVTFGIVFTISVSSVLAKTL
TVIIAFNATKPGSKLKKYVGTQLAIILVIVCSSGEIIISIVWLVSNPPFPEADTLSDPDYIILLC
NEGSGFFFFCIIGYIGTLALLSFIAAFLAKDFPDRFNEAKNITFSMLGFCSVWGAFVPAYLSS
KGSRMVAVEIFAILSSSAGLLGCIFIPKCYIIFLRPELNTKANRII*
>jgi|Xentr4|366422|e_gw1.347.21.1
FIYTIGEINKDPEILPNVTLGYRIYDSCGSGMKSFASAFGILSGTEQIIPNYSCWNNRKVVGF
IGDLSSESSLSIAWLVGIYRYPQISYGSADPIFNNRLEFPSFYRMAPNELSEIDAIMSLIRHFG
WKWVGLVVSDDDTGNRASERLQNQMNKDGGCLAFLIRLKKLSDLTRSQVNITDIYRSIA
QTMYKTTAKVIILFISSQYIDNFNRLFGGYKMPKKIWIASSSFSRVIELRYSQTPVTLNGTLS
LSLQQGEIPGFKQFFYSVNPFKHRDDTLFSKIWEMFFSCSFSDLAPGKTTEVSLPKCTGNK
TFDETVLESYGTFNYRIAYGVYTAVYTMAHTLHELYGTMTRSPKSAESLHMYFKQWQLN
ALMRHVAFRTSSGDQIYFKDNRDPPANYDILKWYFFGKGNIQTIKVGSFDTSRSDGDQLFI
NNSADLWGPYFSECVQSQCSKPCKPGFRKAKIKGAPSCCYTCVLCADGEMSNITDAQSCT
KCSKYEKSNRGRTSCIPRDINYLSYDDHLGSTLSSISVTFSISCAVILGIFIKYRETPIVRANN
RYLSCLLLISLMLCFLCTLLFIGRPTQICCLLRQVTFGIVFTISVSSVLAKTLTVIIAFNATKPG
SKLKKYVGTQLATILVIVCSLGESIISIVWLASNPPFSETDFFSDPYNIILQCNEGSGSFFFCII
GYIGTLALLSFIAAFLAKDFPDRFNEAKNITFSMLGFCSVWGAFVPAYLSSKGSSMVAVEIF
AILSSSAGLLACIFIPKCYIIFLRPELNTKANRII*
>jgi|Xentr4|366470|e_gw1.347.347.1
MADGEWVAIGWALLVLLGTSFLAGAAINIFIVAVNLADWLKRRQLSDLDKVLTCIGVSRL
GLQITTTMTVYGVIFFQMDLLYEAKTVSEALRSLELFFNYSSTWVTTLLSFFYLVKIANFH
HPAFVLLKERISQLLTALVISCLVFALINTLLLTLWPPHINGLLGNSTHDLHENVTASQSDRR
VFVYLFVAGKLLPFAISAVSLTLLLVSLSLHIRQMRDSSHSSPNLDKYYTAIKSMIFCFLIYS
MHIGIGFAGLPYSFSMNIIWIQIMKNLFPTLHSLFLIVGTVKLRRQFWSITQRAANCFSRGSS
MELTETIPP*
>jgi|Xentr4|366471|e_gw1.347.339.1
MSYLIDDVLSTVFSVTAVLLGLAVNGFIPLVNIKDWVTVRKMKPSDRILTVLCSTRFLLQFT
FFLELTGISFGLIPLSAYAAYCITYVVELLLDLFSHWLSMWLCCLYYVKITTSKHPLILHLKS
RIPGITKYALLLFGFLSFLTGLVYYVSGDDISCLHGISKNLTANRTFESLQERLMIAYFFGQA
FPFMMEMMSSMYLLSLLVSHVKHTMSNFSSFKAPSMDAHWSLIRYILLLYFLSACNLVGN
LLLWQVTSHSIGRSVGYFIIFSYPSFHSITLVLCNPKLKREMVKTFIWTRNVLCCFRGDGGF
RTETVAQ*
>jgi|Xentr4|366478|e_gw1.347.43.1
SLCIARLAGIYRYPQISYGSRDPVFNDRVQFPSFYRTVPDELSEIDGIVELIKHFGWKWVGLI
VSKDETGLWAGKNLEREMKKDGIFLAFFIRIELDAMVSKISINIREILIESTANVIVLLVSLM
YVEFVMFFFATFDIPAKIWIVSSTFLRVLDTAYPQNRIIFNGLLALSIQQGEIPGFREFLHGLN
PSDYRDYILFTNISERLSDCKMPNAIPSRTGISLPKCTESESFDEYGLSLNNTKNYRISYGVY
TAVYTMALALHKLYMKETRANHPDRRESQEMHVEQWQVRSLLNAIIQKMTSGNKMNFK
YHDSSNYYEIVKCFFLEEGGARTVTVGSFDTSKPAGNQLYINSSADLWVFFAPQCPQSQCN
EPCMPGYRRSKIEGKPSCCYNCVPCVEGEISNTTGSCIKCSEYEKSNTEQDGCIPKNINFLS

YKDILGATLSSITILCAVTCAVILGIFIKYRETPIVRANNRYLSCLLLISLMLCFLCTLLFIGRP
TQICCLLRQVTFGIVFTISVSSVLAKTLTVIIAFNATKPGSKLKKYVGTQLAIILVIVCSLGEII
ISIVWMVSYPPFSETDTLSDPDYIIVQCNEGSGCFFFCIIGYIGSLALLSFIAAFLAKDFPDRF
NEAKNITFSMLGFCSVWGAFVPAYLSSKGSRMVAVEIFAILSSSAGLLGCIFIPKCYIIFIRPE
LNTKDTIVRKR*
>jgi|Xentr4|366480|e_gw1.347.35.1
MVY*FFSRLKV*RSILQKRPLIRNTLGPKHSG*WVLYLYIVYIVNKKNICWHQIFTFFKNYR
SSFRQYRYLLVFIHTIEEINKDPEILPNVTLGYHIFDSCDDAQKSLEGTFSILSGTQQLIPNYS
CWNNRKVVGFIGDLSSVSSLSMASLTGIYRYPQISYGSRDPVFNDRVQFPSFYRTIPDELSEI
NGIVELIKHFGWKWVGLIVSDDEIGLRAGKNLEGEMKKHGICLAFNIRIDNGAMTSTTSIK
IREKLLSSTANVIVLLVSLMYVEFFMFFFAVFDMPEKIWIVSSTFLKVLDTAYPQNRIIFNGL
LALSVQQGEIPGFREYLYGLNPSLYKYNALFPNVSQRLSDCELFKTNQTIPSRTGISLPKCT
ESESFDEYGLSLNDTMNYRISYGVYTAVYTMALALHKLYMEQTLTSHPERNARLQKNFM
QWKLNEIIQNKGFEMTSGDKMHFKLKGDPSTQYDILKCFFLEEGGARTVKVGSFDLSKPA
GNRLSINTSADLWGPYFAECPISQCNEPCVPGYRKSKIEGKPLCCYKCVSCAEGEISNTTGR
SYSGHCIRCSKDEMSNKEKNGCIPKNINFLSYEDMLGATLSFISVICSIACAVILGIFIKYRET
PIVRANNRYLSCLLLISLMLCFLCTLLFIGRPTQICCLLRHVTFGVVFTISVSSVLAKTLTVII
AFNATKPGSKLKKYVGTQLSIILVIVCSLGESIISIVWLASNPPFSETDFFSDPYYIILQCNEG
SGSFFFCIIGYIGTLALLSFIAAFLAKDFPDRFNEAKNITFSMLGFCSVWGAFVPAYLSSKGS
RMVAVEIFAILSSSAGLLGCIFIPKCYIIFLRPDIYNSKHTIVKK*
>jgi|Xentr4|366487|e_gw1.347.4.1
MFKPLSLHNRISFRHYRHLLVLIYTIGEINKDPEILPNVTLGYRIYDSCASGIMSFAGALSILS
GTEQPIPNYSCWNNRKVVGFIGDLSSVSSLSIAWLAGIYRYPQISYGSADPIFNNRLEFPSFY
RMGPNELSEIDAIMSLIGHFGWKWVGLIVSDDDTGHRANKRLQEAMSKYGVCLAFLIIFK
EMSEVHQAYPTEIRETIYRSTARVVILFLSSQRINCISLLFHPNKIPPKIWIASSSASRIAELEY
LPALVTFNGTLVISLQQGEIPGFKQFFYSLNPYKYQRDDLFPQIWEMLFHCTFSETDISLRK
CTGNETFDDTVLESYGTFNYRIAYGVYTAVYTMAHTLHELYGTMTRSPKSAESLHMYFK
QWQLNALMPHVRFRTSSGDKIYFKDNGDAQARYDIVKWYFLNSRNKKSIKVGSFDGSKS
DGKLFVNDSANLWGPYFSEFAHSRCSEPCKPGFRKAKVEGAPSCCYTCVLCADGEMSNV
TDAQSCMKCSKYEKSNTGRNSCILRDINYLSYEDHLGFTLSSISVILSVTCAVILGIFIKYRE
TPIVRANNRYLSCLLLISLMLCFLCTLLFIGRPTQICCLFQQVTFGIVFTISVSSLLAKTLTVII
AFNATKPGSKLKKYVGTQLAIILVIVCSLVDIVISAVWLASNPPFPEADTLSDPDYIILLCNE
GSVTFFFCIIGYIGTLALLSFTAAFLAKDFPDRFNEAKNITFSMLGFCSVWGAFVPAYLSSK
GSRMVAVEIFAILSSSAGLLACIFIPKCYIIFLRPELNTKDPIVRKH*
>jgi|Xentr4|366493|e_gw1.347.30.1
YRYLLVFIYTIGEINKDPEILPNVTLGYRIYDSCDDAQRSLEETFSILSGTQEIIPNYSCWNNR
KVVGFIGDLSSAPSLSMASLTGIYRYPQISYGSRDPMFNDRVQFPSFYRTVPDELSEIDGIVE
LIKHFGWKWVGLIVSDDETGLRAGENLEREMKKDGICLAFNIRIDLRSISSTNPIEIKKIIVR
STANVIVLLVSLMFVDFILFFFAVFDMPEKLWIVSSAFLRVLDSTYPQNRIIFNGLLALSIQQ
GEIPGFREFVHSLNPSELSGGVLFPNVTERLSDCGLSSTTQITPNRTGVSLPKCTEDEFFFVN
DLSWNDTLNFRISYGVYTAVYTMALALHKLYMNQKMASHPDRKTRLQAQFKPWQLNG
MIENRDFEMTFGDKVHFTIKGDPSTHYEIVKCFFSEEDSVQTMKVGSFDTSKPAGSQLYIN
RSADLWGPYFTEQPQSQCNKPCGPGNRKSKLDGKPPCCYNCVPCTEGEISNTTGSCIRCTE
YEKSNKEKNSCIPKNIDFLSHKDTLGVTLTSITVICSIICAVILGIFIKYRETPIVRANNRYLSC

LLLISLMLCFLCTLLFIGRPTQICCLFRQVTFGIVFTISVSSVLAKTLTVIIAFNATKPGSKLK
KYVGTQLAIVLVIVCSLGESIISIVWLASNPPFSETDFFSDPYNIILQCNEGSGSFFFCIIGYIG
TLALLSFIAAFLAKDFPDRFNEAKNITFSMLGFCSVWGAFVPAYLSSKGSRMVAVEIFAILS
SSAGLLGCIFIPKCYIIFLRPELNTKDNLVRKQYK*
>jgi|Xentr4|366497|e_gw1.347.14.1
LIYTIEEINKDPEILPNVTLGYRIYDSCASGMISFASAFGILSGTEQPIPNYSCWNNRKVVGFI
GDLSSESSLSIAWLAGIYRYPQISYGSADPIFNNRLEFPSLY*MIPNVLSEIDAIMSLIRHFGW
KWVGLIVSDDVIGHRARERLQNQMNKDGGCLAFLTRFKNWSDINRSKVNITIYETTANMI
ILFNS*QYIDIFYLFFALDKMPKKIWIASSSFSRVIELQYSHVLVTFNGTLALSLQQGEIPGFK
QYFYSLNPYTYRDDTFFSKIWEILFCCRFSDLAPGKITKIPLPKCTGNETFDDTVLSFYETF
NYRIAYGVYTAVYTMAHTLHELYGTMTRSPKSAESLHMYFKQWQLNALMRHVAFRMSS
GDQIFFRDNGDPPANYDILKWYFFGEGNIQSIKVGSFDTSRSDGDQLFINNSANLWGPYFS
EFAHSRCSEPSKPGFRKAKVEGAPSCCYTCVLCADGEMSNVTDVQSCTKCSKYERSNSGR
TSCIPRDINYLSYDDHLGSTFSSISVTFSISCAVILGIFIKYRETPIVRANNRYLSCLLLISLML
CFLCTLLFIGRPTQICCLLRQITFGIVFTISVSSVLAKTLTVIIAFNATKPGSKLKKYVGTQLA
IILVIVCSLGEMIISIVWMVSYPPFLEDDTLSDADTIILLCNDGSGYFFFCIIGYIGTLALLSFI
AAFLAKDFPDRFNEAKNITFSMLGFCSVWGAFVPAYLSSKGSRMVAVEIFAILSSSAGLLG
CIFIPKCYIIFLRPELNTKANRII*
>jgi|Xentr4|366517|e_gw1.347.86.1
PKSLCNEPCLPGYRKYKIKGEQPCCYKCVPCAEGEISNSSDAQSCIKCSEYEKSNEKRIVCI
PKNINFLSYSDPLGATLSSISIIFSIGCSAILGIFIKYRETPIVRANNRYLSCLLLISLMLCFLCT
LLFIGRPTQICCLLRQVTFGIVFTISVSSVLAKTLTVIIAFNATKPGSKLKKYVGTQLAIILVF
VCSLGEIIISIVWLASNPPFREADTLSDPNYIILQCNEGSGCFFFCIIGYIGTLALLSFMAAFL
AKDFPDRFNEAKNITFSMLGFCSVWGAFVPAYLSSKGSRMVAVEIFAILSSSAGLLGCIFIPK
CYIIFLRPELNRKDNIVRKQ*
>jgi|Xentr4|366539|e_gw1.347.123.1
MSFPIPVAVCSLVPTVTGLVVNAFITLVNIRNYLREQGLKPCDKILMALCLTRFLLQWTFLL
DIIGILLQLIPFSTFAIYCIFYVVEQFLDYFSRWLAMWLSILYYVNITIFKNVLVLYLKSRIPH
VTSYMIFVSAFLSFGPGLIYSLTSSEVSCLRVSNQSLSKNATYETKEFLSISFVFGQCLPSVIE
MVSSIYLLWTLFAHVNYTKANVSSFTAPNMDAHWSAIRCILLLYFMSACNFIGNLILYFFS
NDSFGSSLSYIITFAYPTLHSVVFVLNHSKLKKELAKILYCEKSVVGFRAETVVRSSLGQA*
>jgi|Xentr4|366544|e_gw1.347.60.1
FQLNAMMQSKDFESITGDKPHLKANRDSSNFYEILKCLFLKEGGVQVVKVGSFDTTKPAG
NQLHIDESDDLWGPYFTKHPPSQCNEPCGPGYRKSKIEGKPSCCYQCVLCSEGEITNTTDA
QNCIKCSKYEKSNKERNVCIPKNINFLSYEDILGSALSSIAVIYFIICAVILGIFIKYRETPIVRA
NNRYLSCLLLISLMLCFLCTLLFIGRPNQICCLLRQVTFGIVFTISVSSVLAKTLTVIIAFNAT
KPGSKLKKYVGTQLAIILVIVCSLGEIVISAVWLASNPPFPEVDTFSDPNYIIICNEGSGFFFF
CIIRYIGTLALLSFIAAFLSKDFPDRFNEAKNITFSMLGFCSVWGAFVPAYLSSKG
>jgi|Xentr4|366552|e_gw1.347.28.1
LIYTIGEINKDPEILPNVTLGYQIYDSCASGIKSIASAFGILSGTEQPIPNYSCWNNRKVVGFI
GDLSLESSLSIAWLSGIYRYPQISYGSADPIFNNRLEFPSFYRMGPNELSEIDAIMSLIGHFG
WKWVGLIVMDDDIGHRANKRLQEAMSKDGVCLAFLIIIKVWSRVDLQYTKKIRETIYRST
AKVVILYLSSQYIHYITLLFDPNQIPHKIWIASSSVSRIAELQYFPLLVTFNGTLVPSIQQGEIP
GFKQFFYSLNPYKYQRDTLFTEMWEMLFHCTFSETDISFPKCTGNETFNDTVLESYGTFN

YRIAYGVYTAVYTMAHTLHELYGTMTRSPKSAESLHMYFKQWQLNALMPHVRFRTSSGD
KIYFKDNGDPQARYDIVKWYFLEIGNKKSIKVGSFDGSESDGKLFVNDSANLWGPYFSEF
VHSRCSEPCKPGFRKAKVEGAPSCCYTCVLCADGEMSNVTDAQSCIKCSKKEKSNSVKN
SCIPRNINYLSYEDQLGAAFSSISVIFFVTCAVILGIFIKYSETPIVRANNRYLSCLLLISLMLC
FLCTLLFIGRPTQICCLLRQVTFGIVFTISVSSVLAKTLTVIIAFNATKPGSKLKKYVGTQLAI
VLIIVCSLGEIVVSAVWLASNPPFPEADTLSDPDYIILLCNEGSGCFFFCIIGYMGTLALLSFI
AAFLAKDFPDRFNEAKNITFSMLGFCSMWGAFVPAYLSSKGSRMVAVEIFAILSSSAGLLG
CIFIPKCYIIFLRPELNTKEIIIRKQ*
>jgi|Xentr4|366563|e_gw1.347.31.1
LIYTIGEINKDPEILPNVTLGYRIYDSCASGMISFASAFGILSGTEQPIPNYSCWNNRKVVGFI
GDLSLESSLSIARLAGIYRYPQISYGSADPIFNNRLEFPSFYRMIPNELSEIDAIMSLIRHFGW
KWVGLIVSDDFTGHRASGRLQNQMNKDGGCLAFLIRLKLWSDINRSEVNITDIYRSIAQTI
YKTTANVIILCISSQYINSFNLFFAFNKMPKKIWIASSSFSRVIELRCSQTPVTFNGTLVLSFQ
QGEIPGFKQFFYSLNPYTYRDDTLFSKIWEMLFSCRFSDLVRRKTAKIPLPKCTGNETFDDT
VLESYGTFNYRIAYGVYTAVYTMAHTLHELYGTMTRSPKSAESLHMYFKQWQLNALMR
HVAFRTSSGDQIFFRDNGDPPANYDILKWYFFGEGNIQSIKVGSFDTSRSDGDQLFINNSAN
LWGPYFSEVTSQI*VAALGRLRTTVVEQRIGCWDIVPQNLTSYFPFLNVITDAQSCTKCSKY
EKSNSRRTSCILREMNYISYDDHLGSTFSSISVILSVTCAVILGIFIKYRETPIVRANNRYLSC
LLLISLMLCFLCTLLFIGRPTQICCLLRQVTFGIVFTISVSSVLAKTLTVIIAFNATKPGSKLK
KYVGTQLAIILVIVCSLGEIIISIVWLASNPPFPEDEILSDANTIILLCNEGSGCFFFCIIGYIGT
LALLSFIAAFLAKDFPDRFNEAKNITFSMLGFCSVWGAFVPAYLSSKGSRMVAVEIFAILSSS
AGLLACIFIPKCYIIFLRPELNTKANRII*
>jgi|Xentr4|366565|e_gw1.347.23.1
FRYLLVLMYTIEEINKDPEILPNVTLGYRIYDSCASGMKSFGSAFTILSGTEQPIPNYSCWN
NRKVVGFIGDLSSESSLSIAWLAGIYRYPQISYGSTDPIFNNRLEFPSFYRMIPNFLSEIDAIM
SLIRHFGWKWVGLIVSDDDFGHRARERLENIMSKDGGCLAFLIIFNKWSVRKITYIEDYIM
QNNRQTIFRTKANVIISISSRDVDSLHPVFHFNKIPKKIWIGSLSFARLTELRYPLVQGTFNGT
LVLSLQQGEIPGFKPFFYSLNPFKRQHDYLFTKTLTNLFPCRILWKKCTGNETFNDTVLESY
GTFNYRIAYGVYTAVYTMAHTLHELYGTMTRSPKSAESLHMYFKQWQLNALMRHVKFR
TSSGDQIYFEDNGDPPARYDILKLFFFRDKTVQSIKVGSFDSSRSDGDQLFINNSANLLGPH
FSESCFYSTPQFAHSRCSEPCKPGFRKAKVEGAPSCCYTCALCADGEMSNITGTLHAQSCT
KCSKYEKPNSGRTSCIPRDINYLSYDDHLGSTFSSISVTFSISCAVILGIFIKYRKTPIVRANN
RYLSCLLLISLMLCFLCTLLFIGRPTQICCLLRQVTFGIVFTISVSSVLAKTLTVIIAFNATKPG
SKLKKYVGTQLAIILVIVCSLGEMIISIVWLVSNPPFPEDDTLSDADTIILLCNEGSGYFFFCII
VYMGTLALLSFIAAFLAKDFPDRFNEAKNITFSMLGFCSVWGAFVPAYLSSKGSRMVAVEI
FAILSSSAGLLGCIFIPKCYIIFLRPELNTKANRII*
>jgi|Xentr4|366628|e_gw1.347.336.1
MYWAVCCGVIMAAGLAVNGFITLVNITEWLREGRLKLCDKVLTILCLTRFFLLWIFFLEMI
GVLLQLIPFSAFGIYCIFYVFELSLDYFSRWLFMDFFSRWLSAWLAMLYYVKITIFKNPFFL
QLQSLIPRITGYVIFISVFISFVPGLIYSLSAKQGFCEDAKGVNVTTNTDELLAFRIIAFFFGH
SFPFMLEMLSSIYLLCTLFAHIRHTESYISNFTAPNMEAHWTVIRYIFLMNLLSMCNFFGNF
FLWSFISSFIGRAVGYFLAFSYPTFHSVVLILSNPKMKREVVNMFHCATKLWSFSKQEPGT
VTQ*
>jgi|Xentr4|366636|e_gw1.347.24.1

SFRHYRHLLVFIYTIGEINKDPEILPNVTLGYRIYDSCASGMRSFASALSILSGTEQPIPNYSC
WNNRKVVGFIGDLSSESSLSIAWLAGIYRYPQISYGSADPIFNNRLEFPSFYRTIPNELSEID
AIMSLIRHFGWKWVGLIISDDFTGHRANERLQNLMNKDGGCLAFLIRLKNWSEIKSSKVH
FTVIYRDIEQTIYKTTASVIILFFNLQYMASLNLFFAHNKMPKKIWIVSSWIVKELRYSQMQ
VTFNGTLALSFQQGEIPGFKQFFYSLNPYKYQRDALFSEIWEMLFSCRFSDLVPGKTAKSP
LPKCTGNETFDDTVLESYGTFNYRIAYGVYTAVYTMAHTLHELYGTMTRSPKSAESLHMY
FKQWQLNALMRHVAFRTSSGHKIYFRDNGDPPANYDILKWYFFGEGNIQSIKVGSFDTSR
SDGDQLFINNSANLWGPYFSEVTSQIKSGSFVGPWAKAHFTHLLIQPCSWGHNPCQECPPL
ILCTKCSKYEKSNSRRTSCILREMNYISYDDHLGSTFSSISVILSVTCAVILGIFIKYRETPIVR
ANNRYLSCLLLISLMLCFLCTLLFIGRPTQICCLLRQVTFGIVFTISVSSVLAKTLTVIIAFNA
TKPGSKLKKYVGTQLAIILVIVCSLGEIIISIVWLASNPPFPEDDILSDANTIILLCNEGSGCFF
FCIIGYIGTLALLSFIAAFLAKDFPDRFNEAKNITFSMLGFCSVWGAFVPAYLSSKGSRMVA
VEIFAILSSSAGLLGCIFIPKCYIIFLRPELNTKANRII*
>jgi|Xentr4|366638|e_gw1.347.16.1
RRYRYLLAFIFTIGEINKDPRILPNHTLGYHILESCNEEDRTIKSTFSILSGRKQIIPNYSCWN
NRKVVGFIGDLSTGSSLCIAQLAGVYRYPQISYGARDTMFSDRVQFPSFYRTLPDELSEING
IAKLIKHFGWKWVGLITSDDEDGELAGNRMERAINKDGGCLAFLSRINHNSFFDESVITSP
LRESTANVIVLLVTLKYINSAMLFFSFYPIPKKIWIVSSSFLRILDTRAGNRIPFNGLLVISFQ
KGEIPGFREFLYGLNLYQYENSLLFQNVTQRLSDCTQLNVNPNRPGISLPKYTESEFFSATD
LQLNNTLNYRITYGVYTAVYTMALALHKLFIKQKMETEESLQTHFKQWRLNSLIQNEGFE
MPSGDIIHFKENGGPPARYDILKGVFSEEGDIQITKVASFDASKPEGSQLDVNNIADLWGPY
FKEFPQSRCSEPCRAGQRIFKLEGKPSCCYKCVPCVEGDISNTTDAQSCIKCSKYEKPNNE
KTECIPRNINFLSYEDTLGATLSFITLILFGICAVILGIFIKYRKTPIVRANNQYLSCLLLISLM
LCFLCTLLFIGRPTQICCLLRQVTFGIVFTISVSSVLAKTLTVIIAFNATKPGSKLKKYVGTQL
AIILVIVCSLVEVVISAVWLASNPPFPEADTLSDPDYIILLCNEGSELFFFCIIGYIGTLALLSFI
VAFLAKDFPDRFNEAKNITFSMLGFCSVWGAFVPAYLSSKGSRMVAVEIFAILSSSAGLLGC
IFIPKCYIIFLRPELNTKDTVVRKL*
>jgi|Xentr4|366647|e_gw1.347.11.1
MFKPVSLHNRVSFRHYRHLLVFIYTIGEINKDPEILPNVTLGYRIYDSCGSGIISFASAFGILS
GTEQPIPNYSCWNNRKVVGFIGDLSLESSLSIARLAGIYRYPQISYGSADPIFNNRLEFPSFY
RMGPNELSEIDAIMSLIGHFGWKWVGLIVSNDDFGHRANKRLQEAMSKDGVCLAFLIILG
DVLKYDHTYSMKSSETIYRSSAEVVILFLSPQSVHLLHFTSVFSASGKIPHKIWIASSSLSYI
AEMQYVSVLVTFNGSFILSFQGEIPGFKQFFYSLNPYTYQRDILFTQIWKRLLFCTISETDKS
LPKCTGNETFNKSVLPYYETLNHRISYHVYTAVYTMAHTLHELYGTMTRSPKSAESLHMY
FKQWQLNALMPHVKFRTSSGHKIYFKDNGDAQARYDILKLYILNTTNIKSIKVGSFDGSES
DGKLFVNDSANLGGPYFSEFVHSRCSEPCKPGFRKAKVEGAPSCCYTCVLCANGEMSNIT
GTSDAQNCLKCSKYKKSNTGKTSCIPRDINYLSYEDQLGTTLSSISVTFSISCAVILEIFIKYH
ETPIVRANNRYLSCLLLISLMLCFLCTLLFIGRPTQICCLLRQVTFGIVFTISVSSVLAKTLTV
IIAFNATKPGSKLKKYVGTQLATILVIVCSLGEIVISAVWLASNPPFPEADTLSDPDYIILLCN
EGSGFFFFCIIGYIGTLALLSFIAAFLAKDFPDRFNEAKNITFSMLGFCSVWGAFVPAYLSSK
GSRMVAVEIFAILSSSAGLLGCIFIPKCYIIFLRPELNRKDNIVRKQ*
>jgi|Xentr4|366687|e_gw1.347.235.1
MDNRSHTHNNSLLTYLLFILVWKKLIFFLYLQISYGSADPIFNNRLEFPSFYRMAPNELNEI
DAIMSLIGHFGWKWVGLIVSNDDTGHRANKRLQEAMSKDGVCLAFLIILRDIFDYDPAYS

MKSIETIYRSTAKVVILFLSPQSVQLFPTLTHSGKIPPKIWIASSHFSHLSLRKFLISSLNGTLV
ISLQQGEIPGFKQFFYSLNPYTYQRDHLFIEMWEFLFHCTFSKITSISFPKLASNCSVGPWHS
YLDFKQSEFSFRAAKLFGSVPASVGGVYLTIGNKQVVLSRRMRVHVWSKLNALMPHVTF
RTFSGDKIYFKDNGDPQARYDILKWYFLGIGNKKSIKVGSFDGSKSDGKLFVNDSANLEG
PIRKPQTLFFAHSRCSEPCKPGFRKAKVEGAPSCCYTCVLCADGEMSNVTDAQRCMKCSK
YEKSNTGRTSCIPRNINYLSYGDQLGATLSSISVTFSISCAVILGIFIKYRETPIVRANNRYLSC
LLLISLMLCFLCTLLFIGRPTQICCLLRQVTFGIVFTISVSSVLAKTLTVIIAFNATKPGSKLK
KYVGTQLAIILVIVCSLVEIVISAVWLASNPPFPEADTLSDPDYIILLCNECSGFFFYCIIGYIG
TLALLSFIAAFLAKDFPDRFNEAKNITFSMLGFCSVWGAFVPAYLSSKGSRMVAVEIFAILS
SSAGLLACIFIPKCYIIFLRPELNTKDPIVRK*
>jgi|Xentr4|366698|e_gw1.347.7.1
ISYGSADPIFNNRLEFPSFYRMGPNELSEIDAIMSLIRHFGWKWVGLIISDDDTGHRANKRL
QEAMSKDGVCLAFLIIITDVLKYNPTYCKESSETIYRSSAKVVILFLSSQSVRLINFINDYLT
LLTKSGKIPHKIWIASSSVSYIFKMHYVPVFATFNNSFILSFQAEIPGFKQFFYSLNPYTYQR
DILFTQLWEMVIHCTISETDKSLPKCTGNETFNNTVMEFYEAFNYRIAYGVYTAVYTMAH
TLHELYGTMTRSPKSAESLHMDFKQWQLNALMPHVKFRTSSGDKIYFKDNGDPQARYDI
LKVYILNTTNIKSIKVGSFDGSESDGKQLANLGGPYFSEVTAPIQSFAHSRCSEPCKPGFRK
AKVEGAPSCCYTCVLCANGEMSNITGMSGLKGKGSLINLEAGGMNCMKCSKYKKSNTG
RTSCIPRDINYLSYEDQLGATLSSISIMLFISCAVILGIFIKYRKTPIVRANNRYLSSLLLISLML
CFLCTLLFIGRPTQICCLIRQVTFGIVFTISVSSVLAKTLTVIIAFNATKPGSKLKKYVGTQLA
TILVIVCCLVEIVISAVWLASNPPFPEADTLSDPDYIILLCNEGSGSFFFCIIGYIGTLALLSFIA
AFLAKDFPDQFNEAKNITFSMLGFCSVWGAFVPAYLSSKGSRMVAVEIFAILSSSAGLLACI
FIPKCYIIFLRPELNTKDPIVRK*
>jgi|Xentr4|366704|e_gw1.347.41.1
YRHLLVLIYTIEEVNKDPEILPNVTLGYRIYDSCGSGMKSIVSAFGILSGTEQPIPNYSCWN
NRKVVGFIGDLSSESSLSIAWLTGIYRYPQISYGSADPIFNNRLEFPSFYRMIPNELSEIDAIM
SLIGHFGWKWVGLVVSDDDIGHRASERLQNQMNKDGGCLAFLIRLKNWSDINRSKVNST
EIYRSIEQTIYKTTANVIILFVSPQYIDSFNLFFALYKMPKKIWIVSSSFSRVIELRHSQILVTF
NGTLSLSFQQGEIPGFKQFFYSVNPYKYQRHEFFTEIWEIFINCTFSETDISLRKCTGNETFD
DTVLESY*TFNYRIAYGVYTAVYTMAHTLHELYGTMTRSPKSAESLHMYFKQWQLNALM
RHVAFRTSSGDQIFFRDNGDPPANYDILKWYFFGEENLQSIKVGSFDTSRSDGDQLFINNSA
NLWGPYFSEIAKDTIPKYRSYSMSFWLHLNCLNLDINYLSYDEHLGSTLSSISVTFSISCAVI
LGIFIKYRETPIVRANNRYLSCLLLISLMLCFLCTLLFIGRPTQICCLLRQVTFGIVFTISVSSV
LAKTLTVIIVFNATKPGSKLKKYVGTQLAIILVIVCSLGEIVISIVWLVSNPPFPEADTLSDPD
YIILLCNEGSGFFFCIIGYIGTLALLSFIAAFLAKDFPDRFNEAKNITFSMLGFCSVWGAFVP
AYLSSKGSRMVAVEIFAILSSSAGLLACIFIPKCYIIFLWPELNTRVTVIRTY*
>jgi|Xentr4|366708|e_gw1.347.25.1
RFSFRHYRYLLVLIYTIGEINKDPEILPNVTLGYRIYDSCASGLKSIASAFTILSGTEQPIPNYS
CWNNRKVVGFIGDLSFESSLSIARLAGIYRYPQISYGSADPIFNNRLEFPSFYRMGPNELSEI
DAIMSLIRHFGWKWVGFVVSDDDTGNRARERLEKAMSKDGGCLAFLIRLKDGVQAGLT
DTKIIRETIYRSTAKVIILFLGSQYINYINFIFDPDTVHKKVWIASSSVSHIDELQYLYVFKTF
NGTLALSFQQGEIPGFRQFLYSLNPYTYRDDHLFTEMWRKIFQCTISETDIPFPKCTGNETF
DDTVLPYYGTFNYRIAYGVYTAVYTMAHTLHELYGTMTRSPKSAESLHMYFKQWQVRQ
FLPNIKLTGTQFINKQFIKLVQNMSPFWDFHIPGLLPHPGTFSGFLTIPRDLYTSTVTLILFVH

SRCSEPCKPGFRKAKVEGAPSCCYTCVLCADGEMSNITDAQSCMKCSKYEKSNSGRNSCI
PRDINYLSYEDQLGSILASISITFSITCAVILRNFIKYRETPIVRANNQNLSCLLLISLMLCFLC
TLLFIGRPTQICCLLRQVTFGIVFTISVSSVLAKTLTVIIAFNATKPGSKLKKYVGTQLAIILVI
VCSLVEIVISAVWLASNPPFPEADTLSDPNYIILLCNEGSGFFFFCIIGYIGTLALLSFIAAFLA
KDFPDRFNEAKNITFSMLGFCSVWGAFVPAYLSSKGSRMVAVEIFAILSSSAGLLACIFIPKC
YIIFLRPELNTKDPLVRKL*
>jgi|Xentr4|366911|e_gw1.351.108.1
MEYFDGAPWLFLIVYIITFVAGLPLNLLAFVTLIRKFRQQLVSIDILLFNLTVSDLLLLAFLPF
RIVEAASGMDWFMPYVFCPLSFFMYFSSIYITSLFLMAISVERYLAVAYPIKYKLLKNPVYS
VLGSVVIWILGTMHCSVVYIVEHFIPANMNKTNVTRCYDQFSPEQLKILLPVRLEMFVVLF
CIPLLVTVFCYFNFVKILVAQPRIQKEKKKRAIGLVVATLINFILCFMPYNVSHVVGYMEGE
SPLWRTYVLLLSTFNSTVDPVIFYFSSASFKKMFLEGLLQVMNKIQLGSCWGSCMERCKK
AKEDTYESHGS*
>jgi|Xentr4|366968|e_gw1.351.15.1
MAQVQGCLQLAVYIITFVFGLPLNILAFVTFLRKFKQKVISVDILLFNLTLSDLVLLAFLPFR
IVEAASEMEWRMPYFICPFSLCVHFSSNYINSFLLTAISVERYLAVGFPLRYKRLVKPLYIIV
GIVCIWILSTTHCSIFYIVDHFAPEKANKTNVSICYSQFSPVQLQVLLPIRLEVFFLLFCIPFM
VTTFCSISFVHIMVTQPFISRKRKLRAIGLVTVTMINFILCFMPYNVSHVVGFIQGDNPPWR
MHALLFSTFNATLDPIMFYFCSVSYQKMHLEDLLYIMNKTRLGAHRDNCLELFKKDHEEP
HGLVQDCNGEKA*
>jgi|Xentr4|366971|e_gw1.351.63.1
MLVDNLLLAVYVLTIFLGLPSNIMVLYLFFKEARSRLTPNLIYMINLCVSDLVFIMVLPIKIIE
IFSSTWTLPQILCPLYNLIHFSTIYASVCFLSAVSVGRYLSTAFPIKYNIYKKPRYSCLVCVIL
WVIVIFHITFVFLVETSQNGSIALFLTNHDNTWLCYENFTSEQLALVVPVRFEISIVLYFFPL
AITLFCYVSCIRILMRSRMHVNLKRKAMRVAVTTLVVFIVCFAPYNISHVVGFVIHESVWW
RKWALLPSTCNAFLDPLIFYFISSSMDQGFYHMWKSLQLKYSVSRRKFSSMFSREQQGSSR
TLGVAAISATV*
>jgi|Xentr4|367003|e_gw1.351.13.1
MSPSPAQRNTHLAIYFITILTGFPTNLLALHALIQKLRNKATPNAILLFNLTISDLSFLTFLPFK
VAEILQGQWKMPSFLCPLSGLFYFSTIYSSTLFLTAVSVERFLGVAYPLKYKLYRKPSYAAAI
SGFLWVCSFAHCSIVYVTEYKNDANASMRIVCYDNFTEKQMEVLGPFRLELGIVLFCFPFL
ITCFCYCSFIRILVSSPHIHREKKQRAIGLVITTLSVFAICFAPYNASHLVGFVQRKNVSWRD
EALLLSTLNTCLDPIIFYFSSTAVQHSCRRCLVKLGLCKTHSALKKIIGEPHIKTNPPDISDSQ
FHSSKL*
>jgi|Xentr4|367023|e_gw1.351.134.1
MDTDTHSKLVLAVYIFTFLTGLPSNLLAFYTFLIKVRQRATPVDILLLNLTVSDLILLMFLPF
KMDEAASNMMWRLPQFLCPLTGFCYYSSIYISTLFLTAVSVERYLGVAHPIKYKLNRKPLY
AVVASIIIWIVACANCSIVYIVQYRLPSNETLSTNFTCYEQFSNEQLQILLPVRLELGLFLFFV
PFIVTVYCYVNFIRILMSLPNIPAKRKQRAIGLAVATLTNFVICFSPYNISHIVGFIQDDSPPW
RVDALLLSTFNASLDPIVFYFSSSAVQAMACECIVGITRKLRTICPCNCLCFSRCDGSVKEN
SVDKSSG*
>jgi|Xentr4|367593|e_gw1.356.15.1
MEILHESYLEHHSRNVSNDSIPGTVSDVSVDNLVATSAIGILLSLMCIGGVIGNFYTLVVMC
LSMTTFTSMYIHIVNLALADLLYLSTIPFIVHNSFVKDWYFGEIGCRVLLSLDLLTMHASIFI

LTVMSTERYIAVAKPFDTVRRSRGYRKSLACAIWLLSFLLTLPMMLMIHQEERTLESGNIR
KLCTPIWSDDQYKVYLTVLFTTSILAPGVIIGYLYTKLARAYWISQTKSLLNKEVQRSPKQR
VILMIFVIVLAFWACFLPFWLWQLLPLYSHDVLRISVQTEIYINHLVTCLTYGNSCINPFLYT
LLTRNYKEYLRNRQRGALGSLSLKSGIASPGKQAKRTASGGSQQCTETITMSNAKGACDS
LKL*
>jgi|Xentr4|367779|e_gw1.358.81.1
MAVYGLAIILGTLLSGISCCLTPDDLIGAKSHGDIIIGGLFSVHGKMMNSPTGYPNIPAIQNC
SGFEMQGFLQMLAMSHAIEMINNSPLIPGIRLGYEIYDTCSEVTMALSATMRLLSEFNSSE
DNLKIKCNYTTYTPKVKAIIGDSYSEVSIAVARLLNTQLIPLVSHSSSAEILSDKFRFPTFLRT
IPNDFHQTRAMAKLIHFSGWNWIGLIAMDDEYGRSALESFGAQSMKINVCIAFKHIIPAHL
SDSTVQSKINKTIMTILRETRVNIIVVFLKPSLVIRLFKKVIQLNIQKTWIASDMWSAASTISS
IPNIQRVGRVIGFTFKSGNMSPFLNYLKNLNSQHFEMNHLLDQYAWLLYHCPKVKYNELS
SCISNYSKETPYDIKNRKGIFLKEDFLLESMQPGFVYSTQLAVTAIAYAIRNICSNRNCKDPD
AFAPWELLQGLKTVNFTFDGRNIYFDSRGDANTGYDVLMWMVGDDGRIDITPVAEYDSQ
KDSFLFKNTEKENEFIRLKKIESTCSDQCKPGQMKKTSASLHTCCYECVACPENHHTNKSD
MGYCLQCNNKTEWSPVNSSVCYNKDIKYLRWNDGFAIILLIVSFLGKGLIIAIALLFTKNF
NTPVVKASGGILCYVILLSIFFSFVSAVFFIGKPEDFKCKVRQTLFGISFTVVVACIFLKSVKI
LLAFSFEPRVQRILRLLHKPFTLVFICTGIQVLICATWLVFWPPHMKENFSLPQTIIYECDEG
STIAFSVMLVYIAVLAFICFIFAFKGRKLPENYNEAKFITFGMLIYSIAWITFIPIYATTFGIYLP
AVEMIVILISSYGILTCTFFPKCYIILYKQDSNTKTAFLKMIYKYSSKSASNLTISQASSSSVV
LESSSSASDVYVSTTLSCISNTFSFHERLVTTDIPSSHTGLPTRKRLSSI*
>jgi|Xentr4|367803|e_gw1.358.80.1
MLWNLAFLGFSSLWSFSFPSDKIKHAVAWQRGDIMIGGIFPIHDGIFKQFENTFSEDFQCTG
LRLRHMIDAMSMIYSIEKINNSTLLPGITLGYEIYDSCSSALKAVQAVTYLIPETVAVNNSTN
CNSHTDIIPTIKAVIGEEYSEMSIAISRILNIHLIPQISPSSSAVVLSDKVKFPSFLRTIPNDNHQ
TLALAKLVGTFGWNWVGIISADDDYGHSAVEQLNILLKQEGVCIAFTKIIPTSVDLPNSLN
NIINEFTNCSTEVVIVMAKAPIVSKLFNECIKMNITKTWIASDIWATSNEVSSAEEIKKVGTV
LGLNFKGGNVPGFADYLKDLKASKNGEINKFIREYKNLRFGCTEDYIKYLECVNSSSEYC
VLTDSIEKKSPLACKVKDVLGANDDYLLQIAEWRAEYSTALAVNAIAQALKNILCKKGKC
DIDWNLPPNQLLNELKTGHYHFNEEKFNFDSYGDVLIDYDIIYWKPTDDTIEFLTIGAYNTT
AGKVNIERNNITWHTKDNQVPFSNCSKTCKPGFSKKHSDIACCYKCIECAEGYYTSEADM
TECSKCPIYQWSDNGSSYCTNRTTQYLEWKNPYAVSLMIFVAIGIVAVLLIVILFIKHRDTPA
VKAAGGNYTYLLVSSLLLSLMSTIFFIGQPSNIFCQIRQPFYGISFTLCVSGILIKSLRILLAFE
SGKRGHKVVVVYQPIIIICTSTFLQICICAIWQAVKSPFLSESNSVPKLHILQCNEGSYVAFSV
MLGYIGLLAFICFILAYKGRKLPKKYNEARCITFSMLTYMFVWIAFIPIYMNTSDMYVSAV
QVFAILASVYGVIFCHLLPVSYIVLFKGNTNNTERYMNSIRAFWIKKPEISLPQNKVFYTTH
SNSILRKRRKSF*
>jgi|Xentr4|367831|e_gw1.358.47.1
MLLKVLFYILCSCSFWGLTSSCDLHKYAAAWQPGDIMIGGIFPIHAGVSNLLQRTPTDDFIC
TGLQLRYIVKALSMIYAIERINNSTLLPGIKLGYEIYDSCADVSKAVQSTIKLFPELDSWISLP
FCNNTQLTPTVKVVIGESFSELSVAISRILSLYSIPQISTASSAPSLSDKLRFPSFLRTIPSDKHQ
TQAITKLISTFGWNWVGIIASDDDYGRSAAELLKSYFEKEMICTAFSKTVPSYVDHPSMQE
YIDNVISELNISTANAVIVIAKGTIVIKLFQEAIRLNISRTWIATDIWSTSREVLNMKDIDKVG
AVFGLMFQAKLVEGFTAYLQNLKPPPNGATNNFLEEYKNIRFGCTKEYRKHLECINSLSKN

CSVSDSVKMKSPLACKVENVSLANDDYLVENIEWSTPYRVSLAVAAIGQSLHNILCRNGT
CIKNMDLSPAEILKEIRKVQFFYNSDPYTFDENGDFFSGYDIINWHTSLQPPVYRAVGLYN
MVTSEIRIDETLLFWNTKGNVIPFSDCAKPCPPGYFKRHSLISCCYQCIPCPEDQFSSETDM
SECTKCPVHQWSNNGSSHCENRTVEYFQRSNPLAITLMSFAAFGFLLVLMIGVLFIKYRDT
SVIKEAGHNYIYLLFASLLLSLGSTGFFIGQPSNIICKVRQPLYGIGFTICLSCIIIKSLNIFVAIH
STKGGNSVNLIYQPAALLSGLTGFQFCLCLLWNILKSPFVREIYTKPEILILQCDEGSYVAFG
MMLGYTGILALTSFFLVFHGKKLQSRYNEAWCIKFNILIFMFVWTIFIPIYINTSDVYQSAV
QVIAMLASIYAVIACQILPTSYIILFKRRKIRSTGNTSNLLKISELSLQIKNEAFSPDISQQYSR
HNSSLPQSKINFRSTKQTVRRRHKSY*
>jgi|Xentr4|368544|e_gw1.365.1.1
MPCLPHCLLHLILLLPYTDASCPVGCECTDGTNHKTTCRNVHVLPEVPLYTETLRLEETHL
QTIPVDAFLNLPNISNIYISLDTALQKLEAGSFRRLHRVAHIEIRNVKNLMYIDPDAFQDLPS
LKYLPRCFMYGTRESQFCTPLFSRPRCFVYGTRESQFCTPLNNSVNIVCVTSRKLYNNGFT
HLQGHIFNGTKLDEVYLHKNNLLGGLHEELFLGAISGPNLLDMSQTAITSLPTKGLENLKH
LLAQSTWTLKKLPPIKTFAHLRKAELSYPSHCCAFQNWTKKKSYLESVICNQSTLHSLIKK
RSLGLFVGPYYQDYIEGDSDQTENSKVIDFHSNNHYSVFFEEQGEEEVGFGQEIKNAQGE
NTPAFENHYEIIICGGSEELECTPEPDEFNPCEDIMGYNFLRIVVWFVNLLAILGNVFVLFILI
TSHYKLTVPRFLMCNLAFADFCMGIYLLLIASVDVHTRSEYYNYAINWQTGPGCNAAGFF
TVFASELSVYTLTMITLERWYAITFAMRLDRKIRLRHASFIMLGGWTLCFFLALLPLVGISS
YIKVSICLPMDTEAALSQAYIVFVLMLNIIAFIIICACYIKIYITVRNPQYKSGDKDTKIAKR
MAILIFTDFICMAPISFYALSAIMNKPLITVSNSKILLVLFYPLNSCANPFLYAIFTKAFRRDV
FILLSKFGICEHQAQVYRGQTVSAKNSSGSCCQRESGGTGQTLANMADFIKQEQPERAVE
NSLLLEDSSLTVF*
>jgi|Xentr4|369966|e_gw1.378.43.1
MAMKNQTFLGDFILFGFSEDHVLLSFLMAAVYTMILMGNVAIFSIIHIDGNLQAPMYFFLT
YLSILDICYSTVTLPSMLFNSITGNRRISFHRCFIQLYFFVSFGGAEGLLLAAMAYDRYVAIC
NPLHYPIIMNRKLCFHLVAGSWVCGFLNSVLHTVMTSQLYFCEIRYVSHFFCDVPPLLKAS
CTDTHTSKILLYVVNVFLGMTTFVFVIISYIHIISTIIKIRSAAGRRKAFSTCSSHLIVVTMFY
VSGNYNYIGPTPGDSFDTERLASLLYSILTPLLNPFIYCLRNKEVKRALYKKLLPYQFSIKI*
>jgi|Xentr4|369984|e_gw1.378.12.1
MKNTTGATEFLMLGLSDVPDRQMLLFVSFLIMYLLNILGNMTIILLVKLDSHFHTPMYFFL
GNLSFVDICYTSSIVPKMLINIMSKEKSISLTECILQLYFFLLFACTECFLLAAMAYDRYVAIC
NPLHYTMIISKKLCILLVIASWLASALHSLLHSLMVSRLTFCGTNKMNHFFCDMAPLLKLS
CSDTSINKLLIYTEGGLPVLIPFGIVLVSYVRIISSIMKIHSADGRYKAFSTCSSHLTVVILFYG
TIAFMYLRPSSSYSLNYDRVVSVVYTVVAPMLNPFIYSLRNNEVKGALKKLFIKKKIL*
>jgi|Xentr4|369987|e_gw1.378.9.1
MAMKNQTFLGDFILFGFSEHGVLLSFLMAVVYAMILMGNAAIFSIIHNLQAPMYFFLSYLS
ILDICYSTVTLPSMLFNSITGNRRISFHRCFIQLYFFVSFGGAEGLLLAAMAYDRYVAICNPL
HYPIIMNRKLCFHLVAGSWVCGFLNSVLHTVMTSHLYFCEIRYVSHFFCDVPPLLKASCTD
THTSQVLLYVVNVFLGMTPFVFIIISYIHIIYIIIKIRSAAGRRKAFSTCCYHVLCDR*L*LYW
PYTRRLL*HRTIVLFII*HSNSFI*PYNILL*KQGSNNGFEKKIDTQD*HHQGACLPANQV
>jgi|Xentr4|369996|e_gw1.378.42.1
MSVSEEFKYENRTTVEYFRLLDFSEGWLLVAILFLCIYLTILIGNCCIFGIIRFSSSLHTPMYF
FLSNLSLLDICYSSVSLPFMLINCISGNTRVSFIKCIVQLYFFVSLGGAECILLAVMAYDRFVA

ICNPLHYAKVMSGTLCAGLAAASWASGFLNAILHTVMTSILSFCQAEQSIDHFICDIPPLIK
AACNSTNTSKTLLYVVSVFLGFTPFLFIVISYVHIISTIVKIKSSEGRQKAFSTCSSHLIVVTM
FYGTANLNYVGPTSGYPLHLERMFSLLYSISTPLLNPVIYCLRNKDVKGALKKTFRKALYY
*
>jgi|Xentr4|371266|e_gw1.394.52.1
MALSHSQVLIYLIILCVGPCKSVRPACRMEIIKAVEEYEYSKDGDVIIGGIMTGHISSIVIHPE
QWSQRLLCTDPHQQNSKLFVDFRFAIEQINKNPELLPNLSLGYHIYDSCGDVQKAVRSILQI
LSGTREPVPNYSCVGKRNIAGFIGDAASDTTIPIAQILNLYGLSQISYGATDPELSDRITFPYF
FRTVQSDEGYYFALGKLLRHFGWTWVGIVISDGISASQEQLLKKSLSSEGICVEFTAKISKN
TIKEADSQQELNIIRKSATSVVILCGTLTSVFLGKFSAYFYMVREKTFILPPHWGTDDYIINY
AEESFNGSLVFVPVYSYNLNTPEMRNFLEDFHPTKYPKDKLVEDIWMIFHLCLSKDPNKN
QLYEYIYLQTLHNCTGKETITSHLYSGNNSYSPRVHLAVHMMAHAIHNLHNLLQKQSPGK
NTELHVYKHQLYHLLRNLHYEIPEGRETSFDDSGGYITKYGIQNYIIIPPHQVVINFLGTFSP
WESHDQLGIDTTSIVWKTKNRNIPRAQCSDNCLPGFRKVSKLGAQICCHDCIPCSEGEISA
GTDSEYCMRCPDMEWPNKKKDQCVSKMEDYLSYTKELISVLFSLISVTFALIAIVILGVFIF
YMDSPIVRANNRSLSFLLLVSIKLSFLSVFLFLGRPVDITCMLRIITFGITFSIAVSSLLAKTIM
VCVAFKATKPGSSWRKWLGVKLSNSVVLFCSSIQIIICMTWLAISPPFQELDIHTSPGTIIIQC
NEGSAIGFYSVIGYMGLLAAVSFVLAFLARSLPDSFNEAKYITFSMLLFCSVWITMIPAYLS
TKGKNTVCVEIFAILTSSAG
>jgi|Xentr4|371500|e_gw1.396.81.1
MEPSNWTRGCFTLLGLSEKPYHWLPLFSFFLCSYCLCIAGNCLISLLILTKPQLHTPMYILL
GNLSVVDVCLASVTVPRAMCGLLSGDTSISFRGCFLQLFLFHAVGNMDSFLLAIMALDRC
AAVCRPLHYYSIMSRRTCACLITLSWVIVCLHSTLFTLMTFYLPFCSWVVHHFFCDVPAILL
LSCRDTSAQQMVVFVEGSVIVMGPMLFILGSYILIIRAVLRLRTPSGRNRTFSTCSSHLTVV
VIFYSSIIFMYFRPSSLYSPVYDRGVSVVYSVLTPVINPLIYSLRNKEVKAAVKGILHCGEKK
KGIKAGKSLGVSDIFPSILYVWLILHSSTSIHSKNSNLVKLYGISNYL*
>jgi|Xentr4|372149|e_gw1.403.4.1
MSECGQGSSENATKCGTQVMDIQCFLILNSTSQKIFIAVLCIGAGALCILENTVILCMIWTSS
HLRRKPSYIFLMSLALADFLASMVFSYSFVDFHVFHGAGTHAVFLFKLGGVTASFTASLGS
LLLMAFDRYVSIHKPSMYKSKVTRKRALLALAGMWLITMFIAYLPLMGWNCCQLDTSCS
ELFPLISNNYLASWMILVVVLLTLIIYSYAHILWKAHKHAVYMEQHNMQSGRGQARLRLD
IMLAKTLALVLLVLVICWSPVLILMMHSLLFSLDRSIKTIFAFCSTLCLVNSMVNPMIYAWR
SRELHRKLIKGFQNIKQLLRVTGTDPEEEGAQKNSGLDTVGEETLCDTEISQ*
>jgi|Xentr4|372317|e_gw1.405.5.1
MKKSIHPGETVCFCNHTTNFAVLLQLYDRNPDEEWTLRTLTFIGCGVSLCALLVTFILFLAV
GVPKSERTTVHKNLIFALAAAESLLMFSELAKNNEVVCITVTASLHLFFMAAFAWMLVEG
LLLWSKVVAVNMSEDRRMRFYYITGWGLPIIIVSVTLATSFNKYVADSHCWLNVQTDIIWA
FVGPVLFILTVNTFVLFRVVMVTISSARRRSKMLTPNCSLEKQIGIQVWATAKPIVVLLPVL
GLTWLCGVLVHLSVVWAYVFIALNSFQVRNPLLNFIQTYTNKSK*
>jgi|Xentr4|372358|e_gw1.406.100.1
MSVNKDLNVTTDNRFILLGLTDVPYLQVLCVLLLLVLYIITLLANGILLIAVRLNTQLQTPM
YFFLSNLSVVDIGFSSNIVPKLLVNTVTLDKSVSLLECALQMYFQIALGSTESMLLTVMAY
DRFAAICNPLHYNTVMNKRFCVCMAVGCWTVGFTNAIIHVVLTFKLPFCKSHNLDHYFCE
EHPFFKLSCQDTWLNEVAMFVATFIIGICCLSFTLVSYAQIIRTIINIPSTHGRRKAFSTCASHL

AVISIFYGTILFIYLGPGVAYFTEANKFVSMIYTVVTPMLNPIIYSIRNKEIKDTIRKQLAKLN
FKKANVIS*
>jgi|Xentr4|372362|e_gw1.406.129.1
MDNLNETSFNGFFLLSLADTPSLQALSSITFLIMYILTLSINALILIAVRINLRLHTPMYFFLS
NLSIIDIGISTSVVPKLIIITITQYKSISLLDCAVQIFFHSALVVTECVLLAVMAYDRYTAICKPL
HYNTLMNKRFCISMVAVCWAVGCINSSFHVTYTFQLPFCRSHHINHFFCEIPVLFSVSCQDT
WFHEISMYIAACILGLIAFISILFSYIYIISTVLNIRSTEGRHKAFSTCASHLTVVSLYYGPLMF
MYFRPHSRNSPSIERTVSIIYL*LQC*IPSSIA*GIKISKLPYKELAAF
>jgi|Xentr4|372365|e_gw1.406.92.1
MDYRNQTLTNRFVLLGLTDIQYLQVFYVLIFSVIYIITVLGNSLLIIAVLINVHLQTPMYFFL
TNLAVIDICCASAVVPKILVNTLSLDKSVSLAECALQMFLSLVLGTTECLILAVMAYDRFVA
ICRPLHYNAVTNKRVCILLTAFCWSIGFINSTIHMALTFQLPFCRSHHIDHYFCEIPPILQLSC
SDTWYNEVSVYISACLIVISSFCLTLASYIYIISTIFRIHSTQGRQKTFSTCASHLTVVCMYYG
TIMAMYLGPHSAYSPERAKITSIIYTSVTPMLNPIIYSVRNKDIKHTITVKMIKKCVVWF*
>jgi|Xentr4|372368|e_gw1.406.132.1
MENSNKTFFNGFFLLSLADTPYLKHLCLLTFLIMYILTLSVNSLLIIVVRNNFQLHTPMYFF
LSNLSIIDIGISSSVVPKLITITITQDKSISYYGCALQMFFHSALVVTESILLAVMAYDRYMAI
CKPLHYNTLMNMGFCISMAAVCWAVGCISAGIHVPYTFQLPFCRSRHVNHFFCEIPVILRL
SCQDTWLHEVSMYISACTLGLSAFILTLLSYAYIISTILNIRSTEGRHKAFSTCASHLTVVSLY
YGPLMFMYFRPHSRNSPSIEKTVAVIYTVVTPMLNPLIYSIRNKDIKVSIQGTLNKH*
>jgi|Xentr4|372375|e_gw1.406.116.1
MNINVTSGNRFILLGLTDTLDLQAVCVLLFMIVYLITWLANSLLIMVVSINAHLQTPMYFF
LLNLSIIDISMSSSTVPKILQITLAQDKSVSLLECAAQMFIVLVLGVTECTILAVMAYDRYAA
ICRPLHYNTIMNKRLCICLAAKCWTVGLINAVIQVFLTFQLPFCRSRHINHYFCEVPPILQLS
CRDTLFNVIAMYVAACIIVICSFFLILVSYVYIISTILKIRSTEGRYKAFSTCASHLTVVSLYYC
TIMSMYLRPRSAYSPETDKTVSLVYTSVTPMLNPIIYSIRNKDIKLTIRKYLTKQKILFSSSIH
Q*
>jgi|Xentr4|372376|e_gw1.406.23.1
MEDFNQTSSDRFFLLGLTNIPYLQALYVSLFFIIYITALSGNSLLIIIVRINEQLQTPMYFFLSN
LSILDICLSSTIVPRIIINTLSQDRSVSLLDCALQMFFHLAVGGTECLILAVMAYDRYAAICQP
LHYNTVMNKTFCICMATGSWILSFISAFLHVYFTFQLPYCRSHNLDHFFCEMPPFFRLSCR
DTWPNELANYITASIIAMCSFLLILISYIHIISTILNIRSSDGRKKSFSTCTSHLTVVSLFYGTIL
SMYMSPHTAYSSIGNTLSIIYSTVIPMLNPIIYSMRNKDVKSTIRKQMKNKNYF*
>jgi|Xentr4|372401|e_gw1.406.34.1
MSFTKNSNLTTENRFILLGLTDVPYQKALCVLILLIIYIITLWGNFLLLIVVRMNTQLQTPM
YFFLRNLSVVDIGFSSTIVPKLLIITVMLDRSVSLLECALQMYLHLALGGTESMLLAVMAY
DRFAAICNPLQYNMVMNKRFCICMAVGCWTVGFTNSIIHVVLTFKLPFCRSHHLDHYFCE
EPPFFKLSCQDTWLNEVLMYIAAFILGICCLFLTFVSYAQIIRTIISIHCTHGRRKAFSTCASH
LAVVFLFYGTILFIYLGPGIAYFTETNKSVSMIYTVVTPMLNPIIYSIRNKEIKETIRKKLKHL
FHKTPEEFPYHPR*
>jgi|Xentr4|372448|e_gw1.406.109.1
MSDNKNSNLTTENRFILLGLTEIPYQQAVWVLLLLIIYITTLSANLILLIVVRLNTQLQTPMY
FFLRNLSVVDIGFSSTIVPKLLVITVTLDKSVSLLECALQMYFHSALGGTDSMLLAVMAYD
RFAAICKPLQYNTVMNKRFCVCMAVGCWTVGFTNAIIHVVLTFKLPFCRSHHLNHYFCEE

PPFFKLSCQDTWLNKMAMYIATVIIGICCLFLTLVSYAQIIRTIINIPSTHGRRKAFSTCASHL
AVISIFYGPILFIYLGPDISYFTEANKSVSIIYTVITPMLNPIIYSIRNKQIKDTIRKKLASCCLK
TPEEFP*
>jgi|Xentr4|372472|e_gw1.406.128.1
MENINETSFNGFFLLSLADTPSLKVLSSLTFLIMYIFTLSINSLLIIMVRINLRLHTPMYFFLS
NLSIIDIGISTSVVPKLIIITMTQDTSISLLDCAVQMFFHSALVVTECVLLAVMACDRYTAICK
PLNYNSLMNKRFCISVAAACWAVGCINSSIHVPYTLQLPFCRSHNINHFLCEIPIFFHLSCQD
TWSHELSMYITACTLGLIAFILILFSYIYIISTILNIRSTEGRHKAFSTCASHLTVVSLYYGPLM
FMYFRPHSRNSPSTERTVSVIYTVVTPMLNPIIYSIRNKDIKVTIQRTLNMQV*
>jgi|Xentr4|372496|e_gw1.406.99.1
MENSTQTSTKRFILLGLTNVPYLQALCGLLFFIIYITTLSGNSLLIIIVRINEQLQTPMYFFLS
NLSMIDICLSSTVVPRIIINTLSWDKSVSLLDCALQMFFHLSLEGTECLILAVMAYDRYAAIC
QPLHYNTVMNKKLCICIATGSWTLAFISAFIHVFFTFQLPFCTSVANHYFCEMPPFFRLSCR
DTWPNLVAMYSTASIIAMCSFLLTLFSYVKIISTILKIRSTEGRKKSLSTCTSHLTVVSLFFGT
ILFMYMSPHSTYSDLDKSVSIIYSAVIPMLNPIIYSIRNKDVKGTIRKEMNKLRDSEKS*
>jgi|Xentr4|372499|e_gw1.406.102.1
PDRFILLGLTSTPYLQELCVLLILIMYIVTIAGNLLLIIVVGTSVQLQTPMYFFLCNLSVIDIC
FSSTIVPKLLINTLAHDKSISLFGCASQMFFHLALGCTECMILAAMAYDRYAAICKPLHYY
QVMNKRVCICLASGSWAVSLINSAIHVVFTFQLPFCRSNHLNHFFCEIPPFLYISCRDTWFN
EVAMYISASIIGIGSLSLTLISYVYIVLTILNMCSAEGKMKAFSTCASHLTVVLLYFFTLFFM
YLHPHSDYYPETSKMVSLIYTVVTPMLNPIIYSIRNVEVKSSIKNKLTIETFKKF*
>jgi|Xentr4|372510|e_gw1.406.134.1
MITGGENKTLNNEFLLLGFQILQSLRIPFFLLILILYILTILGNATVAALVLSTHSLQQPMFFFL
GHLSLCDIKLSTTIVPVLLQGVLRGPVNLSVAACITQFQIFVAGVASECFFLGVMSIDRYVAI
CNPLQYSAIMRKELSVELVTLCWILGYFFAFNVTILISRLTFCGPNVIDHFFCDLLPILQLSCS
DTSAVKLAQLFLASVTGVFTVLSIIVTFIYILATVFRIPSTTGRQKALSTCSSHLAVVATFLGS
LLGLYVLPSSGNTLTANKLLSLLYSVVTRDVANCSPEN*
>jgi|Xentr4|372514|e_gw1.406.118.1
MEDLNQTSSNRFILIGLTNIPYLQAIYVSLFFIIYITALAGNSLLIIVVRINEQLQTPMYLLLSN
LSILDICLSSNIAPRIMINTLSQDRSISLFDCALQMFFHLAVGGTECLILAVMAYDRYAAICQP
LHYNTVMNKTFCICMATGSWILSFISAFFHVYFTFQLPYCRSHNVNHYFCEMPSFFRLSCR
DTWPNDLANFITAGIIAMCSFLLTLISYIHIILTILKIRSNEGRQKSFSTCTSHLTVVSLFYGTI
LFIYMIPNSANSSIGKTVSIINSSVITMLNPIIYSVRNKDVKGTIRKQMKKTEIF*
>jgi|Xentr4|372720|e_gw1.409.77.1
MACNQSCQYYGNYETTLFPVIYSLVFLFGLVGNLAAIGIIVQHVKRGKILGVYLANLCASD
LMYVFTLPVWIAYTAKDDWVFGALSCKIVGFFFNANLYTTIAFLSCIAMDRFVATVFPFQA
RSLRSMKTALIICAGVWLIILGTHCVFLGHDELFLSSQNVQLCYEKYPMDEWMAHINYFRI
FVVFLIPLVLLIVSYCSVGRVVHQSTSLEKEQKRKIIGLLMTMMAIFIICYLPYHVVLFIRSYI
SDKDMCTCPLEVKVRPAYRISFCLTSLSSALDPFINIFVSDSIKQDLLKEIRALWAGFRILRHP
RDSLRRRSSKITGYNTVTHNIENDNLLIEHKKEETVL*
>jgi|Xentr4|372865|e_gw1.411.62.1
MTHYNTSFDQDIHIISESLNSSDIMIGQEESTLLGLKITLAVLLSIITLATLLSNVFVIITIVMT
RKLHTPANYLIGSLAFTDLLVSILVMPISIAYTVTHTWTFGQIVCDIWLLSDITCCTASILHLC
VIALDRYWAITDALEYTKHRTARRAAVMILVVWIISICISIPPLFWRQAKAHEELTVCTINTD

QISYTIYSTCGAFYIPTVLLILLYGKIYIAARSRILKPPSIYGKRFTTAQLIPGSTGSSFCSINAN
VHMEHPHTSETPVCINHIKIKLADSIIERKRLSAAREKKATKTLGIILGAFIVCWLPFFVVSL
VLPICRDACWFDPLLFDFFTWLGYLNSLINPVIYTVFNEDFKKAFQKLIRLNNAS*
>jgi|Xentr4|373542|e_gw1.418.18.1
MSYEQNFSVPNATSIQNLVAPRGSTPPIIPAVMFLLGVVGNLIAIIVLCKSRKEQKETTFYTL
VCGLALTDLLGTCLISPVTIATYIQGEWPGGHALCEYGSFILLFFGLAGLSIICAMSIERYLAI
NHAYFYNHYVDKKLAGLTLFAIYVSNVLFCALPSMGLGKTTFQYPKTWCFIDWRTNVST
YAAYSYMYAGFSSFLILATVLCNVLVCVALIRMHRQFVRRTSLGTDNRLSDFRRRRSFRRM
AGAEIQMVIVLIGTSVVVVICSTPLVVRIFINQLYQPAVVEDISKNPDLQAIRMASVNPILDP
WIYILLRKTVLSKLIEKIKCLFCRIGARRRPHSAGSFNCTEGRRASSALSSHSPSFISRELREV
SSTSQTLLYPLELSDGSLKGSAVLPGTTIAINLPGSTDRTLRGSDQSVSSQGPESEQVSLVNE
TKPTRVTQNGSLSKGNPLRVTFPNETLHLSGKSI*
>jgi|Xentr4|373758|e_gw1.421.46.1
MDNTTFSELNGTVLQKREWHGLEYQVVTVFLVVLICGLGIVGNIMVVLVVLQTKHMRTP
TNCYLVSLAIADLIVLVAAGLPNITESVYGSWVYGYIGCLCITYLQYLGINASSCSITAFTVE
RYLAICHPIKAQFLCTISRAKKIIVFVWAFTSLYCLMWFFLLDLNTTIYKDATVVNCGYRVP
RSFYSPIYLIDFGIFYAVPMTLATVLYGLIARILFLNPIPSDPKENSKTWKNDVTHQNKAFNS
KMSSRCSNNTIASRRQVTKMLAVVVILFAFLWMPYRTLVVVNSFLSRPYLQTWFVLFCRIC
IYLNSAINPVIYNLMSQKFRAAFRKLCKCKKKRSEKPANYGVALNYSVIKESSNGGSPDHF
STELEDITVTDTYLSTSKMSFDDTCLPT*
>jgi|Xentr4|371557|e_gw1.396.13.1
MNSTPASEFILLRLSESPPLRILLFLLFLIVYMVTVFGNIMILSIILQNPMLHYPMYKLLWSFS
LMDLSFSSNTVPGMLKTILFQNNRITFSSCMTQVFFFHAFGNSENLLLSGMAYDRYVAICH
PLRYSVLMGGRTCLSLCSGCCVCASLHALLHEMMTSLLHFHRPAYLHHYFCDIPPLLEIST
SDTSINQLLIYTEGSFVALIPTLLVLISYMRIGVCILQIQSYTGRQKAFSTCASHLTVFLLFSA
SAVSMYFNPTSVSSLYKGRAVTLMYTAITPMFNPYIYCLRNNEVKAALKKVFAKKELQ*
>jgi|Xentr4|372777|e_gw1.410.10.1
MENKNITKVEEFIFSGLSDNPQLKPYLFILFFIVYTLTLVENSALILVISNNAQLYTPMYEFLR
QLSIIDICYSSSVTIKMLSDFVSEKRSISVAGCGLQMLTYAGFGGSECFLLAAMSFDRYVAIC
HPLTYLKTMNKQTLVILILFCYLGGLLNGIVQTWFSFFHLNFCGLKQYINHFYCDVMALIEI
SCGNTRANEVVLLTMVGFIELSSLFVILSTYLCIIYTVLGIRSSEGRKKAFSTCASHMTVVS
LFYGTIIFMYLRPLSAYRPEEDKIIAVFYTVIIPLLNPIVYSLRNRDVKKSAKKTVCQVKLKN
VSTCCH*
>jgi|Xentr4|375423|e_gw1.441.53.1
MYQPFQHLDSGQVACCESPEMLMNSTVSHESQQRKELVAGQIVTGSLLLLLIFWTLFGNIL
VCTAVMRFRHLRNRVTNIFIVSLAVSDLLVALLVMPWKAVAEVAGHWPFGAFCDIWVAFDI
MCSTASILNLCVISVDRYWAISSPFRYERKMTQRVALLMISAAWALSVLISFIPVQLSWHKS
ETDDHLLSNQSTGNCDSSLNRTYAISSSLISFYIPVAIMIVTYTRIYRIAQIQIRRISTLERAAE
HAQSCRSNRVDCRHHQSSLKTSIKKETKVLKTLSIIMGVFVCCWLPFFILNCMVPFCDRSS
GHPQAGLPCVSETTFDIFVWFGWANSSLNPIIYAFNADFRKVFSSLLGCGHWCSTTPVETV
NISNELISYNQDTIFHKDIVTAYVNMIPNVVDCIDDNEDAFDHMSQISQTSANNELATDSM
CELDSEVDISLHKITPSMPNGIH*
>jgi|Xentr4|375511|e_gw1.442.52.1
MKNQTSSKYLYFFAFSNHGGELPLLSIVFFLIYVIGVLGNLIIMTVIYLDSHLHTPMYFFLFS

LAFVDICYPTVTLPKLMDILLLGNNSIAFIQCFTQMYFFVALVTVEVTVLSSMAYDRYVAIC
KPLRYHLIMNRRVCLLVIVGTWISGFANAAFFTSHVSKLLVCGSNKIKQFFCDIKAVANISC
DRTAFYNAIYVEVFSFGPMTFSLNLISYVNIIRNILNIKSKHGRQKTFSTCTSHFTVLIIFYGSI
FWMYMRPPSESENLDPMFSVLYVGVTPMLNPLIYSLRNKEVKNALIRTVRKNCR*
>jgi|Xentr4|375527|e_gw1.442.23.1
MENQTNSKYLYFLAFSNHGEKQPLLSIVFFLIYIIGVIGNLIIITVIYLDSHLHTPMYFFLCTL
AFVDICYPTVTLPKLMDILLSGNNSITFIQCFTQMFFCVAMAGVEITLLSSMAYDRYVAICK
PLRYHLIMNRRSCVLVIVGTWVSGFVNSAFLTSLASKLSICNSDEIKGFFCDIKAVADISCDA
ISFYNAIYWEASSFGVMTFCLNVLSYINIIRNILHIKSKHGRQKTFSTCTSHFTVLIIFYGTVF
WTYFSPPSRNLDPVFSVFYIGVTPMLNPLIYSLRNKEVMNALIRTVQKKFKVSLPLTTKHG
QPS*
>jgi|Xentr4|375532|e_gw1.442.128.1
MENQTNSKWLYFLAFSSCGDRRPLLSIVFFLIYVTGIFGNFVIITVIYLDSHLHTPMYFFLCS
LAFVDICYPTVTLPKLMDILLSGNDSITFIQCFTQMYFFFALGRVEVMLLSSMAYDRYVAIC
KPLRYHLIMNRTVCVLVIVGTWIAGFSNSAFLISSVSKLSVCRSNKIKQFFCDIKAVADISCN
KTSFYNYIYAESFSLGLTTCSLNIISYINIIRNILNIKSKHGRQKAFSTCTSHFTVLIIFYGSAF
WMYMRPPSESVNLDPMFSVLYVGVTPMLNPLIYSLRNKEVTNALKRITRKMLV*
>jgi|Xentr4|375554|e_gw1.442.102.1
MGNNTIFTYFYLLAFSREGEKQPTLFIAFFFMYMFGVLGNLSIITVTCSDAHLHTPMYFLL
ANLSFIDICYTTTTLPKLLHILLTGNNIISFPECFTQMFFYSISAQSEIILLAFMAYDRYAAICD
PLKYHLIINIRKCAQVLAAIWTSSLPNALLGSSIILELHFCGSNTIDQFFCDGKYMTKISCEA
VSINYYLFFGTMIYGVLPFLLCVASYTKIIQSILYIKSSSGRKKAFSTCSSHLTALLLFYGAAS
WMYMAPENSQQLDRIITVIFTAMTPMLNPLIYSLRNKEIKRAIKNIFS*
>jgi|Xentr4|375555|e_gw1.442.147.1
MENQTISNSIHILAFSHDGEKQPLLSIVFFFMYVSGVLGNLVILTVTYNDVHLHTPMYFFLS
NLAFVDICYPTTTLPKLMDILLSGNNSITFIQCLTQMYFFLAFAAVEVTLLSSMAYDRYVAI
CKPLIYHCIMNRKVCMFLVAGTWISGFVNSLFFTYMASKLSFCHSNKINQLFCDVKALAKI
SCDIHIFYNIIYAEVFLFGLLPFCLNLLSYINIIHSILQIKSKHGRQKAFSTCTSHFTVLIIFYGS
VLWMYMRPPSELPNLDPVFSVLYIGVTPMLNPLIYSLRNKEVKNALIRIVRVKSRQLQVDN
*
>jgi|Xentr4|375559|e_gw1.442.5.1
MENQTNLKYLYFLAFYNHGEKQPLLFIVFFWIYMIGVLGNLIIIIVTYLDSHLHTPMYFFLC
TLAFVDICYPTVTLPKLMDILLSGNNSITFIQCFAQMYFFFALAGIEVTLLSSMAYDRYVAIC
KPLRYHLIMNSRVCVLVIVGTWVFGFTNSTLLISLVSKLSVCHSNRIKHFFCEIKAVADISCN
KTSFYNYIYAESFSLGLTTCSLNIISYINIIRNILNIKSKHGRQKTFSTCTSHFTVLIIFYGSAF
WMYMRPPSESENLDPVFSVLHVGVTPMLNPLIYSLRNKEVKNALLRLARISRIKSV*
>jgi|Xentr4|375566|e_gw1.442.142.1
MTENEVRENTMQKEFHLLAFLIHENLQLLIFIWLLLMYILAVCANLIITALVCLVPRLHTPM
YFFLCNLSVQDIVYVTAILPKFLAITITGDNSISFLGCMTQLFLYGSCVTVEFLLLTSMAYDR
YVAICIPMHYSLIMKTSVCVLLVSVPWLMGATNGFMYCWFISNLFFCDHQNIDHLFCELK
AILELSCSSFETIKIAMIVTCVIMGLLPFGLILISYGNIISSALKIRTSAGKLKTFSSCCSHLTV
VLLFCGTCMSLYMRPDSGNSQEMDKLLSLLCVALTPLLNPLVYSLRNKDIIHVIKNIINRHF
*
>jgi|Xentr4|375571|e_gw1.442.98.1

NGTLPIEFHLLAFSKYESAYLAFFLVVLLIYLISLCGNIIITAVVFLISHLHTPMYFFLCNLAIQ
DLLCVSAILPKLLAITVTKDTTISFAGCIVQGFVFGSCILAEFFLLTSMAYDRYVAICNPLQY
PLIVTKSICVLLASTSWLIAIFNSFIFAWLVSNLLFYSSQEVNHFFCEMKSVLVHSRSDTETIR
VAIFWESLILGFIPFILILISYLYIISAILNIHTSAGRLKIFSSCSSHLTIVVLFCGTTISFYVSPET
ENSQEQKKFLSLLYTALVPMLNPLVYSLRNQDVGRAIRTFFQM*
>jgi|Xentr4|375590|e_gw1.442.145.1
MENQTHLKYLYFLAFYNHGEKQPLLSIIFFLIYVIGILGNLIIIIVTYLDSHLHTPMYFFLCSL
AFVDICYPTVTLHKLMDILLSGNNSITFIQCFTQMYFFFALAGIEVTLLSSMAYDRYVAICK
PLRYHLIMNRRVCVLVIVGNWIAGFTNSTFLISLVSKLPVCHSNRIKHFFCEIKAVADMFCD
RNTFYNIIYVEIFSLGLIPFSLNVISYINIIRNILHIKSKHGRQKTFSTCTSHFTVLIIFYGSAFW
MYMRPPSKSEDLDPVFSVLYVGVTPMLNPLIYSLRNKEVKNALLRIARIKSV*
>jgi|Xentr4|375597|e_gw1.442.64.1
MKNLTNSKCLYFLAFHNNGQKQRLLSIVFFFIYGIGVFGNLIIITVTYLDSHLHTPMYFFLFS
LAFVDICYPTVTLPKLMDILLSGNNSITFMQCFTQMYFFVALAGVELMLLSSMAYDRYVAI
CKPLRYHLIMTRRVCVLGIVGTWIFGFINSALLTCLASKLSICGSNKIKQFFCDVKAVADISC
DRTTFYNSIYVEIFTLGLTTFSINIISYINIIRNILRIKSKHGRQKTFSTCTSHFTVLIIFYGSIFW
MYMRPPSESEDLDPVFSVLYVGVTPMLNPLIYSLRNKDVKNALVRTARKTLV*
>jgi|Xentr4|375600|e_gw1.442.48.1
MKNQTTSKCLYFFAFSNHGEKQPPLFIIFFLIYVTVVLLNLVIIVVIYLDSHLHTPMYFFLCS
LAFVDICYPTVTLPKLMDILLSGNNSISFVQCFTQMYFFVAFSGLEVTLLSSMAYDRYVAIC
KPLRYHVIMNRKFCALVIAGTWISGFANAAFFTSQVSKLLICGSNKIRQFFCDIKAVADISC
DRTAFYNAIYVEVFSFGLMTFSLNVISYINIIRNILHIKSKHGRQKTFSTCTSHFTVLIVFYGS
IFWMYMRPPSESENLDPVFSVLYLGVTPMLNPLIYSLRNKEVKNALIRTVMKNRL*
>jgi|Xentr4|375607|e_gw1.442.51.1
MKNQTVFTGFQILGFSSNGDKEPLLSIVFFLIYLIGVFGNLVILTVIYRNVQLHTPMYFFLCN
LAFVDICYLNVTFPKLIDILLSGNNTITFSQCFIQMFFFVTLAGVEVLLLTSMAYDRYVAICK
PLRYHLIMNRRVCELLIVGTWMSGLVNSLICTSLASKLPYCRSNRIKQLFCEIKALAEISCD
VTVFFNVIYIDAFLFGLMPFSLNITSYVNIIRSILHVKSSHGRKKAFSTCTSHLTVLIIFYGTV
LWLYMRPPSQSKNLDPAFSVLYIGVTPMLNPLIYSLRNKEVKNALVRTVKIKTR*
>jgi|Xentr4|375615|e_gw1.442.148.1
MGNNTIFTYFHILAFSREGEKQPTLFIAFFFVYMFGVLGNLSIITVTCSDAHLHTPMHFLLA
NLSFIDICYTTTTLPKLLHILLTGNNIISFPECFTQMFFFSLTAKSEILVLSFMAYDRYAAICDP
LKYHLIINIKKCALILIGIWTSAVPNALLACSVISQLHFCGSNTINQFFCDGKYVAKISCEVG
DIYVCLYFGAVIYGVLPFLLCVASYTKIIYCILQIKSSSGRKKAFSTCSSHLTALLLFYGTAS
WMYLAPENSQQLDHIITVIFTAMTPMLNPLIYSLRNKEIKRAIKTLLLGYHGKS*
>jgi|Xentr4|375624|e_gw1.442.59.1
MNNQSYSNCLYFLAFSSYGENQRFLSIAFFLIYVTGVIGNLIIITVIYLDSHLHTPMYFFLFSL
AFVDICYPTVTLPKLMDILLSGNNSITFIQCFTQMYFFFAFAGIEVTLLSSMAYDRYIAICKP
LRYHHIISRRLCVLVIVGTWIFGLINSALFTSLVSNLSICRSDKIKQFFCDVKAVADISCDRTT
FYFYIYVETLTVGLITFSLNVISYINIIKSILHIKSKHGRQKTFSTCTSHFTVLIIFYGSAFWMY
MRPPSESENLDPMFSVLYVGVTPMLNPLIYSLRNKEVKNALKRYARIDMTKPPLSV*
>jgi|Xentr4|375626|e_gw1.442.151.1
MPENELTNKTLQREFHLLAFLSHEKLQLLIFLLVLLMYSLTVCANLIITALVCLVPRLHTPM
YFFLCNLSVQDIVYVTAILPKFLAITITGDNSISFLGCMTQMYLYALCVMVEFLLLTSMAYD

RYVAICIPMRYSLIMKKSVCVLLASDSWLIGAIISFTYCWLVVNLQFCDHQEIDHFFCDLKT
VLELSCGDTTNLKSVILAVCVLIGFLPFGLILISYGNIISSALKIRTSAGKLKTFSSCCSHLTV
VLLFCGTSLSLYMKPDSGNSQEMDKLLSLLYVAVTPLLNPLVYSLRNKDIINAIKHIINLI*
>jgi|Xentr4|375630|e_gw1.442.119.1
MTGNEVRNVTLQREFHLLAFLSHEKLQLLIFVWVLLMYILTVCANLIITALVCLVPRLHTP
MYFFLCNLSVQDIVYVTAILPKLLAITITGDNGISFLGCITQLYLHVLCVTVDYLLLTSMAY
DRYVAICIPMRYSLIMQKYICLLLAFVPWIIGATSASMFSWLVSNLSFCDHQEISHFFCELKA
ILELSCSGTGNIKIALIVVCIAFGLIPFGLILISYGNIIYSVSKIRTSAGKLKTFSSCCSHLTVVV
LFCGTCLCLYMKPDSGNPQEMDKLLSLLYVAVTPLLNPLVYSLRNKDIINAIKNIIKLYF*
>jgi|Xentr4|375645|e_gw1.442.41.1
LSNHGVKQPLLSIIFFLIYVTGILGNLVIIIVIYLDSHLHTPMYFFLCSLAFVDICYPTVTLPKL
MDILLSGNDSITCIQCFTQMYFFLALVTVEAIILSSMAYDHYVAICKPLRYHLIMNRRIFVLV
IVGTCLARFTNSALFICQVSKLLACGSNKIKQFFCDIKAVADISCDRTAFYGAIYAEAFSFGL
MTFCLNVISYLNIIRNILHIKSKHGRKKTFSTCTSHFTVLIIFYGSTFWMYMRIREP*RTVFC
TLSWGDSHVKPPHI*STE*RSKECPDKNCEEELSI
>jgi|Xentr4|375646|e_gw1.442.9.1
MKNQSLSDYLYFLAFSNYGGKQPLLFIVIFLIYVIGVLGNLIIMTVIYLDIHLHIPMYFFLCS
LAFVDICYPTVTLPKLMDILLSGNNSISFMQCFTQMYFFLALVVVEVTLLSSMAYDRYVAI
CKPLRYHLIMNRRVCTLVTLGNWILGLTNSAWFSSLVSKLAFCGSNKIKQFFCDVKAMADI
FCDKNTFYSSIYAETLSIGLMTLSLNVISYINIIRNILHIKSKHGRKKAFSTCTSHFTVLIIFYG
SVFWMYMRPPSKSEDLDPVFSVLYLGVTPMLNPLIYSLRNKEVKNALKRFARKKSI*
>jgi|Xentr4|375648|e_gw1.442.33.1
MTGNKGTNSTLQREFHLLAFLSHETLQLLIFLLVLLMYSLTVCANLIITALVCLVPRLHTPM
YFFLCNLSVQDIVYVTAILPKFLAITITGDNSISFLGCMTQLFLYGSCVTVEFLLLTSMAYDR
YVAICIPMHYSLIMKTSVCVLLVSVPWLMGATNGFMYCWFISNLFFCDFQNIDHLFCELKA
ILKLSCSSFESIKIVMIVTCVIIGFIPFGLILISYGNIISSALKIRTSAGKLKTFSSCCSHLTVVVL
FCGTSLSLYMKPDSGNSQEMDKLLSLLYVAVTPLLNPLVYSLRNKDIINAIKNIKKSIFLM*
>jgi|Xentr4|375814|e_gw1.445.19.1
MIPADMHHTVRILLYSLIFLLSVLGNSLVIAVLIRNKRMRTVTNIFLLSLAVSDLMLCLFCMP
FTLIPNLLRDFIFGSAVCKAATYFMGISVSVSTFNLVAISLERYSAICKPLQSRVWQTKSHAL
KVIAATWCLSFAIMSPYPIYSTLVPLNKIQNRTANMCRLLWPSDLTQQS*SITLSGDSDNRKF
AHISFINVLCCFSTISCSLVKKYRYVFLLLILFLVPGIVMTIAYGLISMELYRGIKFEMSQRKE
RSSSISSNQFKNGDGCYLQSIKKRKKLEMHQLSSHHSAKIERVRSNSSAANLMAKKRVIR
MLIVIVILFFICWTPVFSANAWRAFDTDSADISLSGVPISFIHLLSYTSACVNPIIYCFMNKRF
RMGFLATFTCCSSRGAQGRRMVPGVADEEGRTTGASLSRYTYTNISLCATASAPT*
>jgi|Xentr4|376414|e_gw1.449.135.1
MEHNIPFIFLEIILALSIVSMNVLVCATVYLHKELRTVTNFLIVSLAAADIGVGAVAIPFSIVL
SMEYTLCFYTCIFITCFPLVTTQFSILLLLLIAINAHLKIRLPNSYSLHVTNVRVFLAVVLCLL
LSCLIALSPMMGWNQFQQYADNNSGIQGEHLGCCSLTAVFSPEYLVYFVFFGCTLLPLFIM
LAIYGNIFRVARSHLRSAKRWEAHMACTLLLLVGMFCLCWIPLNVINSLRLLCWSCSIPVN
LIHVAVIFSHMNSFANPLVYAMRKKDFGGALRSVCLDLLTCWSPVKACCRKTKVHPQNG*
>jgi|Xentr4|376790|e_gw1.453.71.1
MEFSNGSSVEFYILLGFSHLPQYRQPLISIFTLLYIFTFIENSLIVLLIIGDHRLHTPMYFLLAN
FSVLDLLSPSVSVPKMISDLISTEGIISFHGCITQMTFFIAITITECYFLSVMAFDRYVAICFPL

HYKTIMSFKICFRLTCLAWLLGCCHSIIHTILTINVDFCGPNHVNHFYCDILSLLQLACSPTN
LNIALVCVSAFFNGICNCTIIFSSYVGIVHAILKINSRDGRRKAFCTCASHLIVVLLYYGTLII
AYFRPINSYTYSKDRTVSVIYTTVTPMLNPIIYTLRNNDVKKAFRKLWKRANY*
>jgi|Xentr4|378832|e_gw1.478.2.1
MDLELELSTSRLYSDFLFYNATQNFSSSILPNTSQSAPGPSISRNHASIIMAVAITALYSVVCV
VGLLGNVLVMYGIVRYTKMKTATNIYIFNLALADALATSTLPFQSAKYLMETWPFGEALC
KVVLSIDYYNMFTSIFTLTMMSVDRYIAVCHPVRALDFRTPSKAKAINVCIWILSSVIGVPI
MVMAVTKTTKAGTITCTLQFPRPDWYWDTVTKICVFIFAFVVPVLVITMCYGLMILRLRS
VRLLSGSREKDRNLRRITRMVLVVVAAFIICWTPIHIFVIVWTMVDIDKRNPYVVASWHFC
IALGYTNSSLNPVLYAFLDENFKRCFREFCLPFRSHSEQSSFSRARNTTRDQVSTCAQSHAP
DKPV*
>jgi|Xentr4|378988|e_gw1.480.22.1
MLEEEDFYLFKNVSSVSPWDGPQYHIAPKWAFTLQALFMGAVFLFGAPLNLIVLVVTMK
YKKLRQPLNYILVNIAVGGFLMCIFSIFPVFISSSKGYFFLGKVACNIDAFIGTVSGLVTGWS
LAFLAFERYIVICKPMGNFTFSSSHAMAVVICTWIIGIGVSVPPFFGWSRYMPEGLQCSCGP
DWYTVGTKYRSEYYTWFIFIFCFVMPLTMICFSYGRLLGALRAVAAQQQESASTQKAERE
VSRMVVVMVGSFCLCYVPYAAMAMYMVTNRDHGLDLRLVTIPAFFSKSACVYNPIIYSF
MNKQFRGCIMETVCGRPMTDDSSVSSTSQKTEVSTVSSSQVSPA*
>jgi|Xentr4|379343|e_gw1.484.73.1
MGAQNKTNQTTCFIDEDVCFNVLTAIYTVIFIGGLAGSVMMLFLICRTNTRTLTMTAVINLL
IGHSIFLMTVPFRIDYYVRRDWQFGPDFCKLVSAMIHIHMYVSFAFYVTLLIIRYLSFFKQK
DKIEFYRNLHSVVASASVWAVILIIFLPPFLYYYGNKKPKGSKRECFHFHTEITNIVRILNYV
VIAVILIVVCSLLGVQIFIIVKVVKNLQSKFSNYQVFWVQLKSLFFILVMIICFLPYHMFRIFY
LDHVDDCFMFNEICLSITALSCLDLLSFALQSGIRKGCQCTVFSGQ*
>jgi|Xentr4|380417|e_gw1.498.74.1
MNFNQSFIDYKTVTSTIVSFEVLIGSLANGFMVTASLIDCASRRKLGSCESILICLGLSRFAF
LWTLFFMYLMSVYLTSLNGKYTDAVMYIFLCFSNSSLWYATWLCMFYCVRIVNISNCVFV
VFKKNFDRCLPVLLLGSLAISVAFSLLLLSNMIFSLPPVYVTFHNMGNWSISGTSSAFLFVR
TNSVAFVSVSFLGSVLPFLVFCRAVIVLVVFLWKHVLKMKRQERTDYKEPSMQAYYRAAK
ALGSFFLFYSFYIVAFNLYISGIATLNNLIGCFCTMLIGSYPSVHSVFLILQNTKLQQALASFQ
QKIRCCSSPGNQTVTDPT*
>jgi|Xentr4|379762|e_gw1.490.1.1
TDQERIGVGSAYEQEGKVQFVIDAVYSMAYALHTMHKTVCPGSVGLCSRMDPVDGVELL
KYIRSVNFTGIAGTPVTFNENGDAPGRYEIYQYQINNGSTEYRVIGQWADHLHLKARAEL
QWPFHLPKIPPSICSQPCRPGERKKVVKGIPCCWHCERCDGYQYQLDTYTCKRCPLHMRP
NVNHTSCIPIPIIKLEWSSPWAVVPLLLAVLGIIATLVVVGTFMRYNDTPIVKASGRELSYVL
LSGIFLCYVNTFIMVAQPGFGTCSLRRVFLGLGMSISYAALLTKTNRIYRIFEQGKRSVSAPR
YISPSSQLVITASLSSIQLLGVLVWFLVDPSRPIVDYENQRTPDPQLTRGVLKCDISDLSLICL
LGYSMLLMVTCTVYAIKTRGVPETFNEAKPIGFTMYTTCIIWLAFIPIFFGTAQSAEKVKLC
YLLNFYIHI*
>jgi|Xentr4|376774|e_gw1.453.84.1
LHTPMYFLLANFSVLDLLSPSVSVPKMISDLISTEGIISFHGCITQMTFFIAITITECYFLSVM
AFDRYVAICFLHYKTIMSFKICFRLTCLAWLLGCCHSIIHTILTINLDFCGPNHVNHFYCDIL
SLLQLACSPTNLNIALVCVSAFFNGICNCTIIFSSYVGIVHAILKINSRDGRRKAFCTCASHL

TVALLYYSTLIIAYFRPINSYTYNRFCYLHHIDPDVKSDHIYSAE**CKEGICETMEKSNLL
>jgi|Xentr4|380898|e_gw1.504.100.1
MSDRNETQVIYLLLQGFKNLHSFNIFFFIIFLVIFISTLIGNLLIILLVSTSRPLHAPMYFFLCH
LSVSDIILSWNIVPILLCTMLGGAQRVSLPGCIVQFHVFSAITIIECILLLVMSYDRYLAICYP
LRYASVMDFRTCIHLAAGSWVLGYSAHLCGFIPITRLRFCHNNLIIDHFYCDLSPLRRLACS
DTSIVKLIVFVFAIPVFVLPCCLIVMSYIYIFITILRIPSTRGKQKAFSTCSSHLIVVATFYGTLI
TKYMLPSQGHSLLIEKVISLWHTVLTPLFNPVIYTLRNKDIKTAARTLCRKYCT*
>jgi|Xentr4|382640|e_gw1.523.79.1
MIYSTWIPTRELLPDQLRVILCLAAIWVTLGSTKLSGSDSPCRIHITKPEYEYKYFKDGDIII
GGVFTVHNYVEYNFTGPERYTSLCTLPVFEFFSQIHSFRFAIDEINRNPDLLPNITLGYHIYD
SCGDPCLAIGSILQILSGPGNVVPNYSCRGKGEIAGFIGDQSSLTSLPIAQLLGVYGYTQISY
GATDPTLNDRNMFPYYFSTGLSDDIQHIAVAELVEQLGWTWVIIVAPDNDSGEKQSQNLQ
KEINKRGACVEMIILLTDDFYTNMKLFEQIMTAEVIVLCGTPSDLMLLSLSFLQKMAPQKT
LVITLTWGLIGYEFKLLYNCSLTFLYPQKRIKGLNDQFNDYLLSVEEDMLLKDILEKYPLCD
APEPFNLTDFSNCTKRTALKDMNIYFTINDEVYRSVYTLVHALRTELSVSVTHRDKYVPIN
THRNQLHRYMKNLHFKDPWGEESYYEEFRDILSVYWIINSFYMSDMVITSSLVGTVTFPQ
SKPKIEIYVERIMWKKDTKNQILKSQCSANCPPGSRKIPGKSAVPCCYACVPCSHGEISNRT
DMENCLKCEDNKWPNQEKTLCTEKQIEFLSYSDDPLTVIFIIISMMLFIIAAVILGIFISFRDT
PVVKANNHTLSIVLLVSIKLSFLSVFLFLGCPVDITCMLRQISFGITFSIAVSCVLAKTLMVSI
AFKATKPGSPWQKWVGVKLADWLVFICSLIQFLISVIWLVISPPYVEHNTHSEPEKIIIQCNE
GSVVAFCIVLSYMGLLASVSFIVAFLARSLPDSFNEAKYITFSMLLFCSVWITMIPAYLSTKG
KYMVAVEIFAIISSSCGLLFCIFLPKCYIILFKPEMNTKQYLLGKCYR*
>jgi|Xentr4|382735|e_gw1.523.87.1
MVPQLMWLFLLCLSSGLKRSESLHSNASLKGSFQIPNITGFFSWNNYSLADWQSFVGKKR
YGAESQNPMVKALLIVAYSFIIVFSLFGNVLVCHVVIKNKRMHSATSLFIVNLAVADILITLL
NTPFTLARFVNSTWMFGKGMCHVSRFAQYCSLHVSALTLTAIAVDRHQVIMHPLKPRIST
VKGVAYIAIIWIMATCFSLPHAIYQKLFTFEYSEENIRSLCLPDFPEPSDLFWKYLDLATFILL
YVLPLLIISVAYTTVAKKLWLRNAIGDVTTEQYFALRRKKKKTIKMLMLVVVLFAVCWFPL
NCYVVLLSSQTIRTNNALYFAFHWFAMSSTCYNPFIYCWLNDNFRSELKALLNMCKKPTG
PSEHMLPSSVPSYRVAWPENRSFKRSLVSHTLPSSSNIQSGKTDISAVEPIVSVG*
>jgi|Xentr4|383275|e_gw1.530.50.1
SNEGYGVAARIVLISFISAVILMTILGNLLVMVAVCRDRQLRKIKTNYFIVSLAFADLLVSVL
VMPFGAIELVQEKWIYGEMFCLVRTSLDVLLTTASILHLCCISLDRYYAICCQPLVYRNKMT
PLRITLMLSGCWIIPTFISFLPIMQGWNSIGILDLIETRKYNKSSNSTNCIFMVNKPYAITCSV
VAFYIPFFLMVLAYYRIYITAREHARQIGVLQRAGAPADHRHQHPDQHTTHRMKTETKAA
KTLCIIMGCFCLCWAPFFITNVVDPFINYSVPVELWTAFLWLGYINSGLNPFLYAFLNKSFR
RAFLIILCCGDEKYRRPSILGQTVPCSTTTINGSTHVLSGCSSVSKFLLLFCNRPVPV*
>jgi|Xentr4|383428|e_gw1.532.199.1
MENQTKEYILVFTGLTDLPELQLPLFCMFLLIYLVTLTGNLLILLLIFTDSHLHTPMYFFLGT
LACLDMSYSSVTVPRMLFDLLTGRRVISVPACITQIYLFVFCAVSEMSVLAVMSYDRYLAIC
RPLHYMQIMSWKVCVQLISGMLVFGSVYALLHTFSLTNLMFCSSHDLQSFFCDLPQLLEA
SCSDTFINVLLIFLSGICYGVVILGVTFYPYITIISTILKISSKHTRSKAFYTCSSHLTAVFIFYS
TSFFNYFHLNTNEQHAQDKIASVFYAILIPFLNPLIYSLRNKELKIALRRALQRL*
>jgi|Xentr4|383443|e_gw1.532.31.1

MENQTIVYVLVLTGFSDLPSLQLLLFLVFLLIYLMTLTVNLLILLLIFTDSHLHTPMYFFLGT
LACLDMGYSSVTAPRILFDLLTDRRVISVPACITQIYFFIFFVSSEISVLAVMSYDRYIAICRPL
HYMLIMNWKVCVQLILILMGFGALYSMVHISFLSQITFCSSNPLKSFFCDLPQLLQISCSDT
FINVLLIFVCGILYGVVVLGFTFYPYITIITTVLKMTSKHTRSKAFSTCSSHLTVVFIFYTTAF
FNYFRPNAKYHFTEDKVASVFYATLTPFLNPLIYSLRNQELKTSLRKALHRQGWSEIDKSL*
>jgi|Xentr4|383445|e_gw1.532.133.1
XIFTDSHLHTPMYFFLGTLACLDMSYSSVTVPRMLFDLLTGLRIISVPACITQFYFFLFFAVS
EASVLAVMSYDRYIAICRPLHYIQMMRWKVCVQLVSSMLGFCAVHALVHTLLLTKLTFCK
SNTLQSFFCDLPQLLQVSCSDTLVNVVLIFIFAILFGGVILGVTFYPYITIIRTVLKIPSKQMRS
KAFSTCFSHLTVVFIFYSTSFFNYLRSKVSDQHTEDKVASVFYSIFIPFLNPLIYSLRNQELKT
SLRKALQRK*
>jgi|Xentr4|383447|e_gw1.532.23.1
MENKSDVSAFILTDLSDLTDSHVPLLVLLVYITTLVGNLVIILLVNTDAELQTPMFFFLKNLS
ILDISMSSIPAPHILYSFLTQSISVSYASCIAQVFFFVLFATTEVCLLTVMSYDRYVAICYPLHY
IIIVNKKLCAALTSCAWVVGFCCSLVHTLGTLRLTFCYTATIRGLFCELYQLIQSSCSDSFPNI
LLVYVLASAVGIFALSTISISYSRVFSIILHNRFNKGRTKGLSTCISHLTVIIIFYITGIFNYFQPK
TNNVAGGRLLSLTYSMFTPCLNPIIYSLRNNELKRALRRTLRRTKLMD*
>jgi|Xentr4|383469|e_gw1.532.50.1
MENQTIVYVLVLSGLSDLQHLKLLLFLVFLLIYLMTLTGNLLILLLIFTDSHLHTPMYFFLG
TLACLDMSYSSVTAPRMLFDLLTDRRIISVRGCLTQIFFFLFCVVSELSLLAVMSYDRYIAIC
RPLHYMQIMSWKVCVQFVLILFVLSIVYSLLHTLCLTKLTFCRSDVLQSFFCDLPQFLQLSC
SDTFINVLLIILLGMLLGIGLLGATFYPYIPIITTVLKIPSNNMRSKAFSTCSSHLTVVFIFYST
LFFNYLRPNANEYYTEDKVASVFYAIFTPFLNPLIYSLRNQELKSSIRRALHVNLQSLRR*
>jgi|Xentr4|383479|e_gw1.532.26.1
MMNKSEISGFLLSDLPNITDYQFPFVLFILLIYLMTLTGNLLIILLINTDAELQTPMFFFLENL
SILDIGMSSIPAPHLFYSFLRESRSITYITCIAQIFFFIFFATAEVCLLTVMSYDRYVAICYPLHY
TSIVNKKLCVVLTSCVWIFGLFYSLVHTLCALRLTFCYTATIRGFFCELYQLIQTSCSDSSINI
LLVYVLASLVSFLAFFTISISYVRVFRIILHNKSNKGRTKGFSTCTSHLTVIIIFYTTFIFNYFQP
KTNNATAGGLVSLTYSMFTPCLNPIIYSLRNNELKRALRRTLRKTKLLFCK*
>jgi|Xentr4|383511|e_gw1.532.29.1
MENQTIVYVLVLSGLSDLPSLQLPLFLVFLLIYLITLTGNLLILLLIFTDSHLHTPMYFFLGTL
ACLDMSYSSVTVPRMLYDLLTVKRIISVPACITQNFFFLFCAESEMSVLAVMSYDRYIAICC
PLHYMQIMNWKACAQLVSIMLVFCLVSSSTHTFFLTKLIFCSSRDLQSFFCDLPQLLEASCS
DTFINVLIIYLTGMYFASVILVVTFYPYITIIGTILKIPSKHTRAKAFSTCSSHLTVVFIFYTTSF
VNYLRSNTNDQHAQDKIATVVYAILIPFLNPLIYSLRNQELKSSLRKALQRM*
>jgi|Xentr4|383524|e_gw1.532.24.1
LEMENQTTVYMLVLTGLSDLPSLQLPLFLVFLLIYLMTLIGNLLVLLLIFTDSHLHTPMYFF
LGTLACLDMGYSSVTVPKMLFDLFTQKRVISMKHCLTQVFFFMFLGTSEFFVLAVMSYDR
YIAICRPLHYMQIMNWNVCVQLVSSMLGFCAVHALVHTLLLTKLTFCRSEALQSFFCDLPQ
LLKVSCSDTFINVLIIFIFVILFGDVILGVTFYPYITIIRTVLKIPSKHMRSKAFSTCFSHLTVVF
LFYSTSLFNYLRSNVSDQHSEDKLASVFYSIFTPFLNPLIYSLRNQELKISLRKALQRK*
>jgi|Xentr4|383535|e_gw1.532.166.1
MKNQTTEYVLVLSGLSDLPSLPLFLAFFLIYLITLTGNLLILLLISTDSHLHTPMYFFLGTLA
CLDMGYSSVTVPRMLFDLLAGRRIISVPACITQFYFFLFFAVSEVIVLAVMSCDRYIAICRPL

HYMQMMSWNVCVQLVSSMLVFGAVNALLHTLLLTKLTFCRPDALQSFFCDLPQLLRVSC
SDTLVNVVLIFIFAILFGGVILGVTFYPYITIIRTVLKIQSKHMRSKAFSTCFSHLTVVFIFYST
SLFNYLRLNVSDQHTENKVASVFYSIFTPFLNPLIYSLRNQELKISLRKALQRK*
>jgi|Xentr4|383555|e_gw1.532.27.1
LFLLFLLIYLITLTGNLLVFFLIFTDSHLHTPMYFFLGTLACLDMGYSSVTVPRMLFDLLTGS
KIISVQACITQVYFFFFFSVSEVFLLAVMSYDRYVAICRPLNYMQIMSWKVCAQFVSFVLVF
SAVYGFVHIFFLAKLTFCSPNVLQSFFCDMPQLLQISCSDTFINVLLILLFGMLFGGGFLVAT
FSPYIIILGTVLKIPSKNMRSKAFSTCSSHLTVVFIFYSSLFFNYLRPNAKYRFTEDKVASVFY
AILTPFLNPLMYSLRNQELKTSLRRTLRKL*
>jgi|Xentr4|383556|e_gw1.532.129.1
MISDTKCYSNFTEFLLLGFSDFHYKHQLFIFVLFCSTYIFTLLGNGLIIITVTQNSRLHTPMY
FFLRNLSILELCYVTATVPKAMHLFMSGMKSISLIGCAFQMQAFVSIGGIQCLFLGVMAFD
RYMAICNPLRYSSVMSHKMCFNLTAGSWVVGCLLSFGLTSSIFSVPCCNSNLIGHFFCDIPP
VLNLACVNTFNNELSVLIACILVVVMPFLAILCSYINILYSVGLIHSIEGRQKALSTCISHLVS
VTLFYGTAMFFHLRLGTQGSTGNDRIMALVYCIIIPAINPLIYSLRNKDMKQSVRKLVQVGI
RW*
>jgi|Xentr4|383561|e_gw1.532.140.1
MPSDNKCLSNFTEFLLLGFSDFHFKHQVSIFMFFCSAYVFTLLGNVLVIVTVTSDSRLHTP
MYFFLRNLSFIELCFITVTVPKAMHIFMSEMKSISLIGCAFQMLAFVSLGGTECLFLAVMAF
DRYMAICHPLRYMTVLSHKMCFHLTTGSWVVGIFVAFGLTSSVFSVPCCNSNRILHFFCDI
PPVLNLACVNTFKSEMFVLIACILVGMIPFLAILCSYINILYSVGLIHSKEALHKAVSTCGSH
LVSLILFYGATMFVYLRLGTQGFTGNDRIIAPVYCIIIPAINPLIYSLRNKDMKLSVSKLLHK
GIR*
>jgi|Xentr4|383585|e_gw1.532.156.1
MENKSEIGFLLFDLSDFTHSQFPFVLLILLIYLMTLTGNLLIILLVNTDAELQTPMFFFLENLS
ILDIGTSSIPAPHLFYNFLTRTTSITHASCMAQIFFFVLFAIAEVCLLALMSYDRYVAICYPLH
YSSIVNRKLCVALTSCVWTFGLCYSLVHTLGTLRLTFCYTATIRGFFCEIYQLIQISCSDYFIN
ILLVYVMASTVGLFALSTISFSYFRVFRIILHNKSNKGRTKGFSTCTSHLTVIIIFYVACLFNY
FQPKSNNATAGRLLLSLTYSMFTPCLNPIIYSLRNNELKRALRRTLRRTKLIIVK*
>jgi|Xentr4|383610|e_gw1.532.130.1
MISDKKCYSNFTEFLLIGFSDFHYKHQLSIFMFFCSAYIFTLLGNGLIIITVTQNSRLHTPMY
FFLRNLSFLEVCSITVTVPKAMHIFLSETKSISLLGCAFQMQTFLLMGVSECIFLAVMAFDR
YVAICNPLRYTSVMSHKTCFYLTSGSWVVGCFVSFGLTSSTFSVACCNSNHILHFFCDITPV
LNLACVNTFNNELFVFIVCTLVVVIPFLAILCSYMNILYSIGLIHSAQGRHKAFSTCGSHLVS
VILFYGTAMIIHFRMRSQGSAGNDRMIAPVFCILIPAINPLIYSLRNKDVKQSVRKLLHDIN
ADSNTR*
>jgi|Xentr4|384568|e_gw1.539.207.1
MENSTKITEFVFVAFAFPQRFQYCMFFIFLILYVLLLSGNILIMMTIRADPHLHCPMYFFLAK
LSFIDLCYSSVTVPKMLADLLSDRKTISFNGCITQLYFFHFFACAECFLFTAMGYDRYVAIC
KPLHYSTIMNRKLCLWMVAATWTIGFVHSNIQTSLTMGLPFCGPNEIDSFFCDIPPLIRLAC
SDTKMIDAMIVANSGFVSLGCFLAVLFSYIGIGSTILKIRSAEGRRKAFSTCVSHLTVVTFFF
GPCVFIYMRPSTALKVDRVVTVFYTVIAPMLNPVIYTLRNEEVKTSIKKVWRRGILYK*
>jgi|Xentr4|384578|e_gw1.539.65.1
LLLLFFIISLTGNLLIIIIICIDRHLHSPMYFFIVNLSFMEICGISSVMCNLLAILLTNDTHISKAG

CFLQSYLYYFFFTADFLILGIMSFDRYVAVCNPLKYNSIMRNSVCVKLVIGCFVTSFLCLLY
QTITILKLPFCGHVLEHFFCESAALMGLICADVSLIKLTTVILSVFILIGSLTLTATSYIIIVSTIL
RLSSDTGRQKTFSTCLSHLTMVGILFGSAIFIMIRPHRHYSTQTDQVINLVSTMIGPLLNPFV
YTLRNQMVKDSIRGAIHYIRMHHL*
>jgi|Xentr4|384668|e_gw1.539.9.1
MASKNQSTVTEFTLSDFSSSLESQVTFFSLFLALYLMTLFGNILIMASVHVDSRLHSPMYY
FLSKLSFLDMCYSTVTIPKMLTNTLLGSKTITFNQCMSQLFFLHLFGGTECFLLTVMAYDR
YVAICHPLRYHIVMTQRFCHWLMASTWIGGFLHSFTQAFLTYQLPFCGPNNLNYFFCDVH
PLSALSCSDTLLIDSFIVANSGMIGLSCFVVLLFSYLIIIKTIFKMHSAEGRSKAFSTCASHLM
VVIIFFGPSVFIYLRPPVNYSVDKLLSFLYTVLTPLLNPIIYTLRNQEMKNALKKLTKGFETK
FKT*
>jgi|Xentr4|384735|e_gw1.539.209.1
MDFGNQSTVKMFFLSGFSQSLPLQVSFFVLFLVLYLITLSGNTLIMASVYMDSRLQSPMYF
FLGNLSVLDICYSTVTIPKMLSNVITNSKTITFNQCMSQLFFLHMFGGTECFLLTLMAYDRY
VAICNPLRYHTIINHTFCLGMVASTWLAGFLHSFTQAFLTYQLHFCGPNTINHFFCDVHPLA
VLACSDTSFIDMFIIANSGMISLVCFVILLMSYIGIISTVLKMQSTERRFKAFSTCASHLMVV
TLFFGPCIYIYLRPPINYSADKLISVLYTVLTPLLNPIIYTFRNHEMKSALKKILWETVLQGPR
N*
>jgi|Xentr4|387114|e_gw1.571.45.1
MPAPTPTPLSTTSPGLPSTSFPSAVSSLAVSAPADTASSSPESTSSLPFSPSTSSTSFSVTTLAPS
PDVDKADEEGSKAALNFLHSGDHLLLSEANCSKSFELTDLQGPPPETLLSHIRGPQDSLLH
ATNFLNMIFQASDMRESSIKEDMEWYHALVRSLAGGQPNIRRALLSLTAHPMSSKPLLLLR
ATKKGHEILLQDLSSDFYHGRHKGNWGSWDNFHASSSLTKAILLNDLRSLDTPKWSRGDS
YIVDLSHVRWSKPFLECEGGHFLQGWMISLSTAFYGLKPDLSPEFKGTLRVDVQLLNLGID
QCAKGPGWFANSHSCDENSTQCISDEKDGSILGRYRCVCQPGYYRTRHEGNVTLYTYRY
MYVYMYIDLSYRRTCSELSNVACRPCSEGCATCVDGSPCLVDEDWALRVTVLSVQATGM
LAVFLSMLVSYSFRDSKRIRASGLILLETILFGSLLLYFPVFILYFKPSVFRCVALRWVRLLGF
CIVYGTIVLKLYRVMKVFLSRTAQRVPYLTSMRLLRMLFVFLLFCIWFLVGWTVGALENL
QRGVPVVIRTQTREGLVFYTCDHDRWDYMMSIAEVLFLCWGSFLCYGARSIPSAFHEPRY
MGIAIHNEMIVSAAFHVLRFLIVPSLHPDWTLLLFFIHTHGTVTMTLTLLFIPKFLHAGVPP
REEIATEVYEDELDMRRSRSNLNSSITSAWSEHSLDPDDIRDELKKLYAQLEVHKTKKMTI
NNPHLQKKRSSRRGLGRSIMRRITETPDSANRQSTREDKEGSPGSSGGQRRKQQDSGSVK
QREESLRQRVLSLRKSHSTYDHMQECKDAGPPSPRESSTRDPSLRDSVMRRNLARNVSMR
SRGDSLCHAPLVCKSLSAHNLLADKKPLSVRPGPLQKSHSVMGSGKGPLVGAA
>jgi|Xentr4|387116|e_gw1.571.4.1
MPPVSKPPEAEVRLSIYAKSSISLTYMLLLFAGILGNAMVIRVLWGLRRRRVQASLSHHMC
SLASCDLLQLVLGIPAELYGSIWSPFPWPLGNIGCCGFYYLWEVLCYAAIFNVLSLSCERHL
ATCRPLSLHLRQSSVRLRLCFIWLMSLLAGLPVLFTMGLEDVYAADVDDQHTELWVCTPL
SSRKGLFVATIWASFLTYLGVLSVVGITCWRMRRALQGSASQDVEVAGPGGSVQLLGRFC
SGQTVTARKQNARMLGCIVGALAVCWLPFQARRLMTVLRSKDQWTENYYRSYITLQPIT
NCFYYLSSCLTPLLYNLTSQSFRRAFVHGITHCAWGTTSTACPYRPQPRDCVRMSAKGQN
ESYV*
>jgi|Xentr4|387457|e_gw1.575.9.1
RLAGNDLTYIPKGAFAGLGSLKVLMLQNNLLRQVPSEALHNLRSLQSLRLDANHISYVPP

SSFNGLFSLRHLWLDDNSLTEIPVRALESLSALQAMTLALNKIHHIPDYAFRNLSSLVVLHL
HNNRIYSLGKKCFDGLHSLETLDLNYNNLDEFPAAIKTLKNLKELGFHSNNIKSIPEQAFIG
NPSLITIHFYDNPIQHVGRSAFQHLPELRTLILNGASQITEFPDLTGTTSLESLTLTGAQLVYL
PSAVCTQLPNLQVLDLSYNHIKDLPSFSGCQRLQKIDLRHNEVYEIRSTTFQQLVGLRSLDL
AWNKIAVIHPNSFSSLPSLIKLDLSSNHLTSFPVTGLHGLTHLKLTGNSALQDLIPSEHFPKLR
VMEMPYAYQCCAFAVCDNLKHSGQMNKDENSSADDFYRKDIGLLHLQDDRDFEDFLLDF
EEDVKVLHSVQCTPSAGPFKPCDHLFGSWLTRTGVWLIVLLSFVCNALVIATVFRPLSYVP
SIKLLIGLIAIMNTLMGLSSGVLATVDALTFGNFAQYGAWWESGVGCQITGFLSVFAAETSI
FLLTVAALERGFSIKCTTKFETKSSFINVKLSIVFCFLLSIVIAVSPLLSGSTYGTSPLCFPLLF
GDPSSMGFMVALVLLNSLCFLVMTIAYTKLYCSLEKGELENIWDCSMVKHIALLLFTNCIL
YCPVAFLSFSSLLNLTFISPEVNKSILLLIIPLPACLNPLLYILFNPHFKEDIGSLKNGDILWSRS
RQTSLASVSSEDAEKQSCDSTQALVTFANSSISYDLPATSSSSSYQMTNNYKLSAVAFVPCH
*
>jgi|Xentr4|388526|e_gw1.592.4.1
MQIIHLLAQLPNAHHYYSSLNSCFHSTPQFAHSRCSEPCKPGFRKAKVEGAPSCCYTCVLC
ADGEMSNITDAPSCMKCSKYEKSNSVRNSCFPRDINYLSYGDQLGATLSSISVTFSISCAVI
LGIFIKYRETPIVRANNRYLSCLLLISLMLCFLCTLLFIGRPTQICCLLRQVTFGIVFTISVSSV
LAKTLTVIIAFNATKPGSKLKKYVGTQLAIILVIVCSLVEIVISAMWMASNPPFPEADTLSDP
DYIILLCNEGSGFFLFCIFGYIGTLALLSFIAAFLAKDFPDRFNEAKNITFSMLGFCSVWGAF
VPAYLSSKGSRMVAVEIFAILSSSAGLLGCIFIPKCYIIFLRPELNRKDNIARKQ*
>jgi|Xentr4|388554|e_gw1.592.112.1
LYLQISYGSADPIFNNRLEFPSFYRMIPNELPEIDAIMSLIRHFGWKWVGLIVMDDDTGHR
ANKRLQKAMSKDGVCLAFHLILRDWSKFDFKYATKIRKTIYRSSAKVVILFLSSQSIDYITV
LFDPEKSPHKIWIATSSLSRITELQYVAALVTFNGTLVISLQQGEIPGFKQFFYSLNPFKYKH
DILFTEIWEILFNCTISETDISKKKCTGNETFDDTVLSFYETFNYRISYRVYTAVYTMAHTLH
ELYGTMTRSPKSAESLHMYFKQWQLNALMPHVKFSTSSGDKIYFKDNGDPQARYDIVKW
YFLKTGNKKSIKVGSFDGSKSDGNLFINDSANLWGPFFSEFVHSRCSEPCKPGFRKAKVEG
APSCCYTCVLCADGEMSNITDAQSCMKCSKYEKSNTGRTSCIPRNINYLSYGDQLGTTLSS
ISIMFSITCAVILGIFIKYRETPIVRANNRYLSCLLLISLMLCFLCTLLFIGRPTQICCLLRQVTF
GIVFTISVSSVLAKTLTVIIAFNATKPGSKLKKYVGTQLAIILVIVCSLVEIVISAVWLASNPPF
PEADTLSDPDYIILLCNEGSGFFFFCIIGNIGTLALLSFIAAFLAKDFPDRFNEAKNITFSMLG
FCSVWGAFVPAYLSSKGSRMVAVEIFAILSSSAGLLGCIFIPKCYIIFLRPELNRKDNIVRKQ*
>jgi|Xentr4|388565|e_gw1.592.2.1
SLHNRISFRQFRHLLVLIYTIGEINKDPEILPNVTLGYRIYDSWGSGMKSFASTLSILSGTEQP
IPNYSCWNNRKVVGFIGDLSLESSLSIAWLAGIYRYPQISYGSADPIFNNRLEFPSFYRMGP
NELSEIDAIMSLIRHFGWNWVGLVVMDDDTGNRASERLQKAMSKDGVCLAFLIIIRDRYIF
DQEYAAEIRETMYRSTAKVVILFLSSHSIYRIFTLFDPDKIPHKIWIATSSASSIAELQYLPLL
VTFNGTLVISLQQGEIPGFKQFFYSLNPYTYQRDDLFPQIWGLLFNCTFLDMDISRKNCTG
NETFDDTVLEFYETFNYRIAYGVYTAVYTMAHTLHELYGTMTRSPKSAESLHMDFKQWQ
LNALMPHVKFTTSSGDEIYFKDNGNPQARYDIVKWYFLEIGNKKGIKVGSFDGSKSDGKL
LVNDSANLWGPYFSEFVHSRCSEPCKPGFRKAKVEGAPSCSYTCVLCADGEMSNITDAQS
CMKCSKYEKSNSVRNSCFPRDIDYLSYDDHLGFTLSSISVTFSISCAVILGIFIKYRETPIVRA
NNRYLSCLLLISLMLCFLCTLLFIGRPTQICCLLRQVTFGIVFTISVSSVLAKTLTVIIAFNAT
KPGSKLKKYVGTQLSIILVIVCSLVEIVISAVWLASNPPFPEADTLSDPDYIILLCNEGSGFFF

FCIIGYMGTLALLSFIAAFLAKDFPDRFNEAKNITFSMLGFCSVWGAFVPAYLSSKGSRMV
AVEIFAILSSSAGLLGCIFIPKCYIIFLKPELNTKETIIRNNRILLRIVVNHGEHEEEKYLYKIRL
STLLLFCILFMTLGASFYIVT*
>jgi|Xentr4|388614|e_gw1.592.8.1
SLHNRYSFRRYRHLLVLIYTIGEINKDPEILPNVTLGYRIYDSCGSGVISFASTLSILSGTEQII
PNYSCWNNRKVVGFIGDLSFESSLSIARLTGIYRYPQISYGSADPIFNNRLEFPSFYQMIPNE
LNEIDAIMSLIRHFGWKWVGLIVMDDDTGHRASERLQNQMNKDGGCLAFLIILKYLSTPG
RAYSREIKNTIYRSTAKVVIFFLSSHSIYHIADLFDPEKIPQKIWIASSSVSRIAELEYLEALVT
FNGTLVISLHQGEIPGFKQFFYSLNPYTYQRHTLLPQIWGLLLNCTFSKRDISLKKCTGNET
FDDTVLSFYETFNYRIAYGVYTAVYTMAHTLHELYGTMTRSPKSAESLHMYFKQWQLNA
LMPHVRFRTSSGDKIYFKDNGNPQARYDIVKWYFLGIGNKKSIKVGSFDGSESDGKLFVN
DSANLWGPYFSECVHSRCSEPCKPGFSKAKVEGAPSCCYTCVLCADGEMSNITDAQSCM
KCSKYEKSNTGRHGCIPRDINYLSYGDQLGATLSSISVILSVTCAVILGIFIKYRETPIVRAN
NRYLSCLLLISLMLCFLCTLLFIGRPTQICCLLRQVTFGIVFTISVSSVLAKTLTVIIAFNATKP
GSKMKKYVGTQLAIILVIVCSLVEVVISAVWMASNPPFPEADTLSDPDYIILLCNEGSGFFL
FCIIGYIGTLALLSFIAAFLAKDFPDRFNEAKNITFSMLGFCSVWGAFVPAYLSSKGSRMVA
VEIFAILSSSAGLLGCIFIPKCYILFIRSELNTKETITRK*
>jgi|Xentr4|388617|e_gw1.592.113.1
CMKCSKYAKSNVGRNSCIPRDINYLSYGDQLGASLSSMSLILSISCAVILGIFIKYRETPIVR
ANNRYLSCLLLISLMLCFLCTLLFIGRPTQICCLLRQVTFGIVFTISVSSVLAKTLTVIIAFNA
TKPGSKLKKYVGTQLAIILVIVCSLVEIVISAVWLASNPPFPEADTLSDPDYIILLCNEGSGY
FFFCIIGYIGTLALLSFIAAFLAKDFPDRFNEAKNITFSMLGIFCQRFSYQFGLTPYFPFLNFIT
DAQSCMKCSKYAKISSFRHYRHLLVFIYTIGEINKDPEILPNVTLGYRIYDSCASGMKSFAS
AFGILSGTEQPIPNYSCWNNRKVVGFIGDLSSESSLSIAWLAGIYRHFGWKWVGLIVSDDF
TGHRASERLQNQMNKDGGCLAFLIRFKDLSDKKVNITDIYRSIEQTIYKTMANVIILFISSQ
YIDSFNIFFALNKMPKKIWIASSSFSPYGVYTAVYTMAHTLHELYGTMTRSPKSAESLHMY
FKQWQLNALMRHVAFRTSYGGKIYFRDNGDPPARYDILKWYFFGEGNKKSIKVGSFDTSR
SDGDQLFINNSADLWGPYFSEFVQSQCSEPCKPGFSKAKIQGQPSCCYTCVLCADGEMSNI
TGTSHTNGNVGHRQIPPTKSIYGSKIMTERQRVATLKYM*
>jgi|Xentr4|388636|e_gw1.592.20.1
MSYFNRFSFRHYRYLLVLIYTIEEINKDPVILPNVTLGYRIYDSCGSGMISFASAFGILSGTE
QPIPNYSCWNNRKVVGFIGDLSSVSSLSIARLAGIYRYPQISHGSADPIFNNRLEFPSFYRM
GPNVLSEIDAIMSLIGHFGWKWVGLIVSNDDTGNRARERLEKAMNKDGVCLDFLIRLKD
RGQSDRTDNKKIRETIYRSTAKVIILFLGLQYIFHINDIFDPDTVHKKIWIASSSVSHIDELQY
LHVFKTFNGTLALSFQQGEIPGFKQFLYSLNPYTYQHDKLFTEMWRQIFHCTVSQINNIPFP
QCTGKERFDDTVLESYGTFNYRIAYGVYTAVYTMAHTLHELYGTMTRSPKSAESLHMYF
KQWQVSYPNLFLKIGITSAFLQSIGTIPDETSVFLFNFINFFEFCSESESGEKIHSSLTKTVGSF
PAPSNDLSIHSTACNCIVLTKELFVHSRCSEPCKPGFRKAKVEGVPSCCYTCALCANVEMS
NITGTSDAQSCTKCSKYEKSNSGRTSCIPRNINYLSYGDQLGSTLSSISIMFSITCAVILRNFI
KYHETPIVRANNQYLSCLLLISLMLCFLCTLLFIGRPSQICCLLRQVTFGIVFTISVSSVLAK
TLTVIIAFNATKPGSKLKKYVGTQLAIVLVIVCSLVEVVISAVWLASNPPFPEVDTVSDPDYI
ILLCNEGSELFFFCIIGYIGTLALLSFIAAFLAKDFPDRFNEAKNITFSMLGFCSMWGVFVPA
YLSSKGSRMVAVEIFAILSSSAGLLGCIFIPKCYIIFLRPELNTKDPFVRKS*
>jgi|Xentr4|388801|e_gw1.595.76.1

MLKIGVHWAVWGYNSGSHCYYFIKVLIYLAIPCIILYRSGVQPLNPACHLQIIKAVKEYEY
MKEGDIVIGGVMASHFHMANVILPPYKSKRLLCNSVNQQGYRYLVDFIFAIEQTNKDPAR
FPNLTLGYDIYDSCGDPRKAVRSVLQILSGTREPVPNYSCVGKRHIAGFIGDLTSETTVPIA
QILSLLGYSQISYGATDPSLRDRAAFPYVFRTVQSDEANYFAISKLLSHFGWNWVGIITSDD
ISGEREHHSLAKYLSREGVCIEFTLRICTNNEVEMKHNQYDITIQESSTSVIILSGTVSTMFV
SELPVLYKVLQEKTWILSSNWGNNDILVGYAVEIFNYSLLFLPRYQYDLGTPEMSRFLEGL
HPSKFPDDKFIEDIFMMFHSCLSKDQNKNDFYQYIYINTLRNCTGQERITDLPYFRGETNSP
RVHLAVDIMTQALHEMLFIKESIQYTYKYQGLHRYLKKHQYSPQTGPTSSFDEHGEYVSG
LWIYNYIISKHDLNRKIVGEFSPWAPPDQQLNITSSVIQWKTNNNKIPRSQCSHNCLPGFRK
APKPGAQSCCYLCVPCSEGEISNTTESGRNSERAVRSPSFFTPFLFPIFKNTKISKMAKNLHP
LHDFFDMNTTFLHNCDLFDATKTFFDVTVTMRANNRSLSFLLLVSIKLSFLSVFLFLGRPV
DITCMLRIITFGITFSIAVSSLLAKTIMVCVAFKATKPGSSWRKWLGVKLSNSVVLFCSSIQII
ICMTWLAISPPFQELDIHTSPGTIIIQCNEGSAIGFYSVIGYMGLLAAVSFVLAFLARSLPDSF
NEAKYITFSMLLFCSVWITMIPAYLSTKGKNTVCVEIFAILTSSAGLLACIFLPKCYTILLRPD
VNMKSNLLRNN*
>jgi|Xentr4|388837|e_gw1.595.79.1
MLGVTALCYLIYLIFLCVGPCRSGVQPLNPACRLQIIKPVKEYEYMQEGDIMIGGVMASHF
YMINMTFPWDNTSRFACVSPIEQAYRYLVDFRYAVEQTNKDPAQFPNLTLGYHIYDSCGDP
RKAVRSVLQILSGTREPVPNYSCVGKRNIAGFIGDLTSETTVPIAQILTLYGYSQISYGATDP
SLRDRVAFPYVFRTVQSDEANYFAISKLLSHFGWNWVGIITSDDISGEREHQILAKYLSREG
VCIEFTITINISKGDHWSISQYESKFQGYSTNVIIISGSASIMFALSLPYVLREKTLILSSNWG
NNDFIVGYARHIYNYSLVFVPRYHYDLGTPEMSRFLEDLHPSKLPNDELIEDIFLMFHLCLS
KDQNKNDLYQYIYGKLHNCTGQERITDLRYFRGETSSPRVHLAIDIMSQALHEMLFIKESIE
YPYKYQLYHYLKNHQYSPQTDPTSSFDEHGEYISGLRIYNYIISSKHDLNRKIVGEFSPWAP
PDQQLSITSSLIQWKTNNNDIPRSQCSDNCPPGFRKAPKPGAQSCCYDCVQCSEGEISNTT
DSESCIQCPDMEWPNEKKIQCIAKTEEFLSYRDGIISVIFLFFSVLFFLITEVILGVFIKHQDT
PIVRANNRSLSFLLLVSIKLSFLSVFLFLGRPVDITCMLRIITFGITFSIAVSSLLAKTIMVCVA
FKATKPGSSWRKWLGVKLSNSVVLFCSSIQIIICMTWLAISPPFQELDIHTSPGTIIIQCNEGS
AIGFYSVIGYMGLLAAVSFVLAFLARSLPDSFNEAKYITFSMLLFCSVWITMIPAYLSTKGK
NTVCVEIFAILTSSAGLLACIFLPKCYTILLRPDVRIKTNLLRKK*
>jgi|Xentr4|389101|e_gw1.600.7.1
MQSLNITPEQFARLLQENNVTREQFIELYQLQPLVYIPELPFRTKIAFVTICVLIFVLALFGNS
LVLYVVTRSKAMRTVTNIFICSLALSDLLIAFFCIPFTMLQNISSNWLGGAFACKMVPFVQS
TAIVTEILTMTCIAVERHQGIVHPLKMKWQYTNRRAFTMLGIVWLIAAVVGIPMWHAQRL
EVKYDFLYEKQYVCCLEAWNSQVHQKIYTTFILVILFLLPLTVMLLLYSKIGYELWIKKRV
GDASVLQTIHGSEMSKIARKKKRAIIMMITVVVLFAVCWAPFHVVHMMIEYSNFENEYDD
VTIKIIFAIVQIIGFFNSICNPIVYAFMNENFKKNFLSALCFCFLRDPSSPSRRPGNSGITLIQQ
KSSSSRRENTCEDTRREAFSEGNIEVKFFDQPVSKKRHLHLFTSELTVHS*
>jgi|Xentr4|389498|e_gw1.605.26.1
MTPRNISTTQSLFPAMASNSSQQFMGLQLIHSYKALIIPCYSLVVFTGIFGNYLLIYVICRTK
KMHNVTNFLVGNLAFSDMLMCATCVPLTLAYVFEPRGWVFGHFLCYFVFLMQPVTVYV
SVFTLTVIAVDRYHATVYPLRRRLTIPTCAYILAGIWLLSCLMAAPALAHTYHVEFPQLDFSI
CEEFWFGMEKKRLAYAYSTLILTYALPFIIIALSYLRISVKLKNRVVPGNITNVQAEWDRAR
RKKTFRLLVLVVGAFGMCWLPLHIFNIMKDIDIHLIDKQYFNLVQLLCHWLAMMSACTNS

LLYAWLHDSFRGELKKMFLWKRKRIGPTAPCVLSVVL*
>jgi|Xentr4|389596|e_gw1.606.29.1
MMDEKPDSNLSVIWYRKSLNKTLNYEEPNITYVDFYLHRPSVAAVFIASYLLIFILCMLGN
GVVCFIVLSSKHMRTVTNLFILNLAISDLLVGIFCMPTTLLDNIIAGWPFGSTVCKMSGMV
QGISVSASVFTLVAIAVDRFRCIVYPFKQKLTISTAVVIIVVIWVLAIAIMCPSAVMSHVKED
KNFRVVIGNSNQTNPIYWCREDWPNPEMRKIYTTVLFSNIYLAPLSLIVIMYARIGITLFKSE
LNVENKNHQQPRHAVSRRKQKVIKMLIIIALLFILSWLPLWTLMMLTDYANLTQNQYQMI
NIYIYPFAHWLAFFNSSINPIIYGFFNENFRRGFQAAFKLQLCSEELERRTMYSHRAQGNAI
LPAASHNTARQNSAFEKQSSDDKESDKRKPLTSDQDLIMEDLEKFSNNNGIQKDMV*
>jgi|Xentr4|388655|e_gw1.592.111.1
LGYRIYDSCASGMKSIASAFSILSGTEQPIPNYSCWNNRKVVGFIGDLSLIPSLSIARLAGIY
RYPQISYGSADPIFNNRLEFPSFYRMSPNELSEIDAIMSLIGHFGWKWVGLVVSNDDTGNR
ARERLEKAMSKDGVCLDFLIRLKDRVQSDLTDTKKIRETIYRSTAKVIILFIGSQYINYINVI
FDPNTVHKKIWIASSSVSHIDELQYLHVFETFNGTLALSFQQGEIPGFKQFLYSLNPYTYQD
DHLFTEMWRKIFNCTISGINNIPFPKCTGNETFDDTVLKYYGTFNYRIAYGVYTAVYTMAH
TLHELYGTMTRSPKSAESLHMYFKQWQLNALMRHVTFRTSSGDQIYFRDNGDPPANYDIL
KCYFLGIGKIKRIKVGSFDASKSDGNQLFFNISADLWGPYFSEFVNSRCSEPCKPGFRKAKI
EGQPSCCYTCVLCADGEMSNITDAQSCMKCSKYEKSNSGRTSCIPRNINYLSYGDQLGSIL
SSISVMFFITCAVILRNFIKYHETPIVRANNRYLSCLLLISLMLCFLCTLLFIGRPTQICCLLRQ
VTFGIVFTISDLNFSSSV*LDI*ERWPY*VSLLHF*PRISLTDLMRLKTSLSVCWGSVACGGH
LSLHT*AVRAVEWWQLRYLPSYPPVLGYWPVYSFPSAILFF*GLN*IQKTLWLEHY
>jgi|Xentr4|388778|e_gw1.595.77.1
VLIYIITLCVGPCRSEPINPACRLQIIKAVEEYEYIQEGDIMIGGVMAAHVSMFLRHLPGEIF
KKLLCYDFSEQNIKYLLDFRFAVEQTNKDPARLPNLTLGYHIYNSCGDPRKAVKSVLQILS
GTREPVPNYSCVGKRNIAGFIGDLTSETTVPIAQILTLYGYSQISYGATDPLLSDRIAFPYFFR
TVQSNKAEFHALGQLLRHFGWTWVGVITSDDMSGEEEYKLLAKYLSSAGICIEFTLRLQS
KEIRYTRLQLNKAIASSSSSVIIFCGRVILSIFLHLSDDINMDTEKTAILSSNWGANDEFIWYR
KELFNCSLVLVPGYHYDLDTPEMRSFLENSHPSNYPGDKLLEDIGMIIFFCLSGDPDKNKL
YEYSFKIYLHNCTGQERTTDLRYLRGKYNSPRVHLAVDLMTRALHDMNINEKYVTNRER
FLSKPQSHNFHLKIGNVAFEFIRISFLPRLVTCHLRYYDVDIDIMRHFATGPLSFLMVFSIWY
FSSYPVARVLFRVLIPRSQCTDSCPPGYRKISIPEAPPCCYNCVPCSEGEISNRTGKEKICGFS
FPLVMIPNTELCNKYKLQRLETEAEAVTKVSVTLLLQTAPLGINCPIVTVSLAGKLCLLLPT
PIAGTENREPDLLTSAGILIGGFFCILMQPLALCCCFDNLLSWGCHTQPVYNSETQTLSSVS
VTLQISMPGKEHSVFLFCSSIQIIICMTWLAISPPFQELDIHTSPGTIIIQCNEGSAIGFYSVIGY
MGLLAAVSFVLAFLARSLPDSFNEAKYITFSMLLFCSVWITMIPAYLSTKGKNTVCVEIFAI
LTSSAGLLACIFLPKCYTILLRPDVNIKTNLLRNN*
>jgi|Xentr4|388849|e_gw1.595.82.1
MLGITAVCYLIYLIFLGVGPCRSGVQPLNPACRLQIIKPVKEYEYVQEGNIMIGGVMASHFY
MINMTFPWDNTSRFACVSPIEQGYRYLVDFRYAIEQTNKDQTQFPNLTLGYHIYDSCGDPR
KAVRSVLQILSGTREPVPNYSCVGKRHIAGFIGDLTSETTVPIAQILTLYGYSQISYGATDPS
LRDRVAFPYVFRTVQSDETNYFAISKLLSHFGWTWVGIITSDDISGEREHQILAKYLSREGV
CIEFTISINISKGDHWSISRSLVNKFQGYSTNVIIISGSASIMFALSLPYVLREKTLILSSNWGN
NDFIVGYARHIYNYSLVFVPRYHYDLGTPEMSRFLEDLHPSKLPNDELIEDIFLMFHLCLSK
DQNKNDLYQYIYGKLHNCTGQERITDLYYFRGETNSPRVHLAVDIMSQALHEMLKGRAES

GCKSTEAEIKASFSFLKAVRASTDQILGSTPFLVRAIETRSTWGFYYYIFLYIFDWLRPNAS
WAVAMGCIRFICSNMYIPRSQCSDNCLPGFRKAPKPGAQSCCYDCVPCSEGEISNTTGTNA
WFLKMYQTCETIGVRQSSDIVQRCHKPLLCFQLVVSVLQKIPPLLLLQTICLLCPSNSNRPV
ILNQWLGGNIFLSVFLFLGRPVDITCMLRIITFGITFSIAVSSLLAKTIMVCVAFKATKPGSSW
RKWLGVKLSNSVVLFCSSIQIIICMTWLAISPPFQELDIHTSPGTIIIQCNEGSAIGFYSVIGY
MGLLAAVSFVLAFLARSLPDSFNEAKYITFSMLLFCSVWITMIPAYLSTKGKNTVCVEIFAI
LTSSAGLLASIFLPKCYTILLRPDVNMKTNFLRKK*
>jgi|Xentr4|390296|e_gw1.616.167.1
MLADSPIISIIILSLNVMCVGPCRSETPTTNPACRLQIIKSAEEYEYIQEGDIMIGGVLTVSMF
RPVDYFRGLTCIFPSAPNYKYLVDFLYVIENINRNPDIFPNTTLGYHIYDSCGDPRKAVRSV
LQILSGTREPVPNYSCVGKRNIAGFIGDLISETTIPIAQILSVFGYTQISYGATDPALSNRFSFP
YFFRTTGSDDSYYLVISKIAKYFQWNWVGIITFDDDRGERDHQLLKDYLSRENICIEFTLKI
TDIIHGNIRYREIIQKSSTNVVIFCGAVNLQILIEFHFLYRLVSEKTFIFTSNWLYYNHVLDFG
HKVFRGSLLLMQNKEEDYSNTSKYTQFSKQFHPSRYPDDKLLENIWMCYHFCLSKNKKK
NEIFKKEFKMLLYNCSGQESLSKIGMYNNGFHSLNMMYAVDMLASALHYRHDTLRMETS
GRNRDMYNFRYKVHQYLKKINFRYKANHVYYLNEKGEFVSRYMIINIYGKSAKQSAWK
QCGTYAPWVAEHLELNITSGVIQWKTKDNKVPRAQCSDTCPTGFRKTQKPRAQSCCYDC
VLCSEGEISNISDSENCIRCPYNEWPNEKKDKCIARIEEFLSYSTDVISVFFSSLSVLLFLITQ
LILQVFISYRDSPIVRANNRSLSFLLLVSIKLSFLSVFLFLGRPVDITCMLRIITFGITFSIAVSS
LLAKTIMVCVAFKATKPGSSWRKWLGVKLSNSVVLFCSSIQIIICMTWLAISPPFQELDIHT
SPGTIIIQCNEGSAIGFYSVIGYMGLLAAVSFVLAFLARSLPDSFNEAKYITFSMLLFCSVWI
TMIPAYLSTKGKNTVCVEIFAILTSSAGLLACIFLPKCYIILFRTENNRKSHLLKNIKT*
>jgi|Xentr4|390307|e_gw1.616.170.1
MALTDPFPVLIYLVLCARYCYSGDQPLSSACNLKIIKALEDYEYIKEGDIMIGGVVTVNAL
VSRFRHKEDYDKIILCLEPSPQNYKYVLDFLYYIKKFNNDPLLAQNLTLGYHISDSCGDVY
KAERSVFQILSGLRDPVPNYSCVGKRNIVGFIGDLTSETTIPIAHILSVLGYSQISYGATDPFL
SDRDTFPFFFRTVQSEEGQYFAITQLVKYFGWTWVGIITSDDINGDRAYQLLTKYLSSEDICI
DFIMKIHHEDIHQQFPLYNKIIQQSSTSVVIFCGTIRLETAEHLHELSDILSKKTFIFPSNWLD
YSLALGYALALFNGSLVIMQNMAEYDKNDHSFSEFLDNLHPSKYPEDKLLADIWMRYHY
CESENKSMNFLHEFIYSRRLHKCTGEERLMDIEDFKEELHTFNMFAAVHTLSMVITRLYCM
DILVNKQSFKKTRKEINYNYWIHRLLKDMQSPYQEKSLRSFNEKGEIVSAYQITHFYVQPN
GQVTETRVGQYIPWAPRDQRLNITLGAIKWKTTNNQIPRAQCSDNCPPGFRKAPRPGAQS
CCYDCVQCSEGEISNITDSDICIQCQDKEWPNEKKNQCMTKSEDFLSYTNDIISMFFSSISV
FFFVITLMILGTFITYRDSPIVRANNRSLSFLLLVSIKLSFLSVFLFLGRPVDITCMLRIITFGIT
FSIAVSSLLAKTIMVCVAFKATKPGSSWRKWLGVKLSNSVVLFCSSIQIIICMTWLAISPPFQ
ELDIHTSPGTIIIQCNEGSAIGFYSVIGYMGLLAAVSFVLAFLARSLPDSFNEAKYITFSMLLF
CSVWITMIPAYLSTKGKNTVCVEIFAILTSSAGLLACIFLPKCYIIMMRPEMNTKSYVLGNN
T*
>jgi|Xentr4|390311|e_gw1.616.162.1
MAASSPLALLLYLTAVCLVTNTSWAWVRTSACNLEITGTYEDYEYVQEGDIMIGGVLTVNS
FIVPFKYPWDSYSRMLCVWPLPEYYRQFVDFHFAIEKSNNDPLLLPNLTLGYHISDSCGDP
RKAVRSVLQILSGTREPVPNYSCVGKRNIAGFIGDLSSETTVPIAQILTLYGYSQISYGATDT
SLSDRVTFPYFFRTLQSDHGHYFSLSKLLKAFGGNWVGIITSDDISGEKELQILTNYLSIDGI
CVEFTIKISDNMDDISAVGNIRHHLPYKNIIQKSTTNVVILCGKISLGVIGKLIIYTDVLSEKI

LVVSPTLTSKNHIIDYSLEIFSNILGFVQHSAYSVDSREITHYLESFRPSTYPEDKLLEDIWLL
YHLCLSKQQHKNYLYANLYKYELHNCTGQKRITDIRHFDSKLYSPSVALAVDMMSHAIHA
MHISLAERPPENRKQMYKYQVNIYLNKLHYKTTGGTTVSFEKNGELVTWYMIYNCILEP
NNQISVNYLGTFQPRDPLDDKSMIRSHQIIWISNKSQIPSAQCSASCLPGLRKVPRIRAQPC
CYDCAQCSEGDISNATDSENCYKCPDMEWPNEKRDQCILKSEDFLSYSNHEISMLFGSVS
LFLSVLTVLIMGIFLKYRDTPIVRANNRSLSFLLLVSIKLSFLSVFLFLGRPVDITCMLRIITFG
ITFSIAVSSLLAKTIMVCVAFKATKPGSSWRKWLGVKLSNSVVLFCSSIQIIICMTWLAISPP
FQELDIHTSPGTIIIQCNEGSAIGFYSVIGYMGLLAAVSFVLAFLARSLPDSFNEAKYITFSM
LLFCSVWITMIPAYLSTKGKNTVCVEIFAILTSSAGLLACIFLPKCYIILITPETNKKFYLTGK
TCGYVK*
>jgi|Xentr4|390322|e_gw1.616.21.1
QNYKYFLDLFYYINEFNKKKNLTLGYHIYDSCGDVYKAERSVLQILSGLKEPVPNYSCVG
KRNIVGFIGDLTSETTIPIAHILSVLGYSQISYGATDPFLSDRDTFPFFFRTVQSEEEQYFAITQ
LLKYFGWTWVGIITSDDINGDRAYQFMTKYLSSEGICIDFIMKIHYEDFQQQFPLYNQIIQQ
SSTSVVIFCGTIRLETAEHLHELSDILSKKTFIFPSNWLDYSFALGYALALFHGSLVFVQNTA
DYNKNNNHFRQFLESVHPSEYPEDKLLEDIWMLYHSCLSKDKRRNILHEYLYSQRLHTCT
GEERLTDIKVFDDEFHTINMVVAVQALSEVSSSLNDIHSLNQQSSGDKKGINYKYQPHRLL
KEMQLQGQSLKYFNEKGEFVSAYQVTNFYLQPNGQVTETRVGQYIPWAPRDQRLTVMLD
AIKWKTTSNQIPRAQCSDNCLPGFRKATKSEAQSCCYDCIQCSEGEISTIIGINPCKRKLLK
NIIIDARNFKKLPTIPAIPQPHLSFLLLVSIKLSFLSVCLFLGRPVDITCMLRIITFGITFSIAVSS
LLAKTIMVCVAFKATKPGSSWRKWLGVKLSNSVVLFCSSIQIIICMTWLAISPPFQELDIHT
SPGTIIIQCNEGSAIGFYSVIGYMGLLAAVSFVLAFLARSLPDSFNEAKYITFSMLLFCSVWI
TMIPAYLSTKGKNTVCVEIFAILTSSAGLLASIFLPKCYTILFMSAMNKKFNLLRNKSL*
>jgi|Xentr4|390335|e_gw1.616.98.1
QHTATLKSQCSANCPPGYRKVPQKSAPPCCYDCAPCSEGEISNVTDMENCLQCPDNEWPN
QGKNMCIEKQTDFLSYDGSLTLSFAFISVICFLMNKIIFGIFISYRDTPVVKANNRDLSFILLI
AIKLSFLSVFLFLGRPVDITCMLRIITFGITFSIAVSSLLAKTIMVCVAFKATKPGSSWRKWL
GVKLSNSVVLFCSSIQIIICMTWLAISPPFQELDIHTSPGTIIIQCNEGSAIGFYSVIGYMGLLA
AVSFVLAFLARSLPDSFNEAKYITFSMLLFCSVWITMIPAYLSTKGKYMVAVEIFAIISSSCG
LLFCIFLPKCYIIMLKPEMNTKQYLLGRLNT*
>jgi|Xentr4|390357|e_gw1.616.160.1
MVRVTDITNAINLIIYIIVLCLVPCTSTVPTINPACELKIIPTYEDYEYIQEGDIMIGGVLTVNS
YTIPFRYAQDGYLRMACVDVFPEYYRQLVDLLIDIKQINQSPNILLNLTLGYHIYDSCGDPR
KAVRSVLQILSGTREPVPNYSCVGKRNIAGFIGDLSSENTVAIAQILNLYGYSQISYGATDTE
LSDRVSFPYFFRTTQSDRGHYFALSKLLKYFGWTWVGIIRLDDYGGEKEHQLLKSILSNDG
MCIEFTLKITSYILRNEIETNIPYYMVYKRIIEESTASVIVFCGTVPTSSVGKLIPLSDLFIKKT
LIVSNNLASSLHFMDYAIEIFNGSLGITQMLLSSRYSTENTQVLKEFHPSKYPKDKLLEDIW
MQYHSCLSKDPAKNKAYEQLYSGFLYNCSGRELITNVRNFDNKFFSPRVPLAVDIMARAL
HIMQISLANRTTGKAKKEQNYRNQMHHYLKKVQYNFSDPTMNRTLSFEEDGEFITQYKIR
YVTFRSYKQITMQLFGVFTPWAPPDKYLKFISKIGWKTKKNEVPKSQCTDNCLPGFRKVP
KPGAHSCCYNCVPCSNGEIANTSDSENCVHCPDMEWPNENRTQCIAKLEEFLSYTNDIISII
FSAISILFFLITMLILILFIKNWDSPIVRANNRSLSFLLLVSIKLSFLSVFLFLGRPVDITCMLRII
TFGITFSIAVSSLLAKTIMVCVAFKATKPGSSWRKWLGVKLSNSVVLFCSSIQIIICMTWLAI
SPPFQELDIHTSPGTIIIQCNEGSAIGFYSVIGYMGLLAAVTIGFYSVIGYMGLLAAVSFVLA

FLARSLPDSFNEAKYITFSMLLFCSVWITMIPAYLSTKGKNTVCVEIFAILTSSAGLLACIFLP
KCYIILIKSEMNTKTHLLGNKM*
>jgi|Xentr4|390387|e_gw1.616.171.1
MLGGTSAIKILLYLTVMCVGPYRSETPTIKPACCLEIIKSAEEYEYIQEGDIMIGGVLTVSMF
QPADYFTGLTCVYPSAQNYKYLVDFLYVTKKVNKNPDYLPNITLGYHIYDSCGDPRKAIK
STLQILSGYREPVPNYSCVRKRQIAGFIGDFTSETTIPIAQILSGFGYTQISYGATDPVLSDRL
TFPYFFRTTESDDSYYFVISKIAKYFQWNWVGIITFDDDRGERDHQLLKYYLSSENICVEFT
LKITHIIHGNIRYREIIQKSSTNIIIFCGAVNIQIVLEFNYLYEMLSEKTLIFTSNWLYYHHVLD
FTREIFHGSLILMQSKEDHPSNSPYAQFSKQFHPTRYPDDKLLENIWMYYYSCLSKNQKKN
EIFEKELGFRLHNCSGQKSLSEIDMYDRGFHSFNMIYAVNMFATTLSYMHKPLGMETSGM
GRKMYNYRYKVHHILKAFYYRVKKNHVLYFNENGEFVTQYLIITLYRMLTNGTGFKPCG
TYTPWAPVHLKLNITPGKIQWKTKDKKVPRSQCSENCPTGFRKAPKPGAQSCCYDCVSCS
EGEISNQTDSETCIRCPYNEWPNQRKNQCISKIEEFLSYTNSVIPVFISAVSAILLLFTVIILGV
FIAHWDTPIVRANNRSLSFLLLVSIKLSFLSVFLFLGRPVDITCMLRIITFGITFSIAVSSLLAK
TIMVCVAFKATKPGSSWRKWLGVKLSNSVVLFCSSIQIIICMTWLAISPPFQELDIHTSPGTII
IQCNEGSAIGFYSVIGYMGLLAAVSFVLAFLARSLPDSFNEAKYITFSMLLFCSVWITMIPA
YLSTKGKNTVCVEIFAILTSSAGLLACIFLPKCYIILFLTEINKKSNLLVIKT*
>jgi|Xentr4|390389|e_gw1.616.19.1
MSGFSRPLPEYYRQLLDFRFVIEQTNKDPAWLPNLTLGYHIYDSCGDPRKAVRSVLQILSG
TQEPVPNYSCVGKRNIAGFIGDLTSETTVPIAQILSLLGYSQISYGATDTLLSDKISFPYFFRT
LQSDHGHYFSLSKLLKAFGGNWVGIITSDDISGEKELQILTNYLSIDGICVEFTIKISETMGG
VKDYARKDRPYKEILQKSSTNVVVLCGKVSIGFIGKLDIYTDAFIEKTLIVSSTLTAKSHIM
AYTLEIFQNIVGFVQRSPYSAESPDIKPFIESIHPSRYPQDTLLEDIWMKYHSCLSKQQHRN
DVYESLYMSKLHNCTGQERITDIRHFDSKLYSPSVALAVDMMSHAIHEMQISLAERPPENQ
IYRYQLHHYLKRLLYKTSTGTTASFEENGELITQYMICNCVLEPDEQLSINSLGVFKPLDPS
NDRLTINSYQIIWMSNKSKFPSAKCSESCLPGFRKVPRIGAQSCCYDCAECSKGEVSGVIDS
VNCLRCPDMEWPNVKKDQCIPKTEEFLPYSHDGISVFFSAVSLLFALITLLILRVFIVYQDSP
IVRANNRSLSFLLLVSIKLSFLSVFLFLGRPVDITCMLRIITFGITFSIAVSSLLAKTIMVCVAF
KATKPGSSWRKWLGVKLSNSVVLFCSSIQIIICMTWLAISPPFQELDIHTSPGTIIIQCNEGSA
IGFYSVIGYMGLLAAVSFVLAFLARSLPDSFNEAKYITFSMLLFCSVWITMIPAYLSTKGKN
TVCVEIFAILTSSAGLLASIFLPKCYTILIRPETNKKTHFHGNKSL*
>jgi|Xentr4|390434|e_gw1.616.33.1
PQARCSHSCTPGFRKAPKAGTQPCCYDCVSCSEGEISLITDSEICIRCSGMEWPNEKRNQC
VAKMEDFLSYTNDVISAVFISVSICLYLVALLILGVFIKYRDTPIVRANNRSLSFLLLVSIKLS
FLSVFLFLGRPVDITCMLRIITFGITFSIAVSSLLAKTIMVCVAFKATKPGSSWRKWLGVKLS
NSVVLFCSSIQIIICMTWLAISPPFQELDIHTSPGTIIIQCNEGSAIGFYSVIGYMGLLAAVSFV
LAFLARSLPDSFNEAKYITFSMLLFCSVWITMIPAYLSTKGKNTVCVEIFAILTSSAGLLFCIF
LPKFYLICFKPKLNIKSNLLGNKS*
>jgi|Xentr4|390535|e_gw1.617.42.1
MLTGNGTECNVDATFVNNIYAITYTIILIPGLIGNVLALWIFYAYIKETKRAVIFMINLSIADL
MQVLSLPLRIFYYLNQSWPFSHFVCMFCFYLKYVNMYASIFFLVCISVRRFLYVIYPFKWN
DRKRVCDVYISVAGWITVCVSCLPFPLLRVSNDTTTNDRCFVDLPLVDIGMNNSVLLVTLA
ELFGFVTPLLIVLYCSWRTVMSLREPDSISQDLGEKKKALKMILTCAVVFLICFAPYHFSFPL
DFLVKANKITQCKQRKAILTLHPVALCLASLNSCLDPVIYYFTTDEFKRRLSRQDIQDSNEL

QMYCNKVFIKRNSRPEL*
>jgi|Xentr4|391165|e_gw1.627.39.1
MWDQKLNESLENSTNVFSGLDLLFDLKPLFIPIYALLVTVACLGNCALILLIAFTKKLHNTT
NFLIGNLALADLVMCIFCVPLTASYAFENRGWLFGEFMCHFVTLMQAATVCVSVLSLTAIA
VDRYVVVVYPIRRRIGCRSCIYIVSLVWLVSIGISMPTFFHTHYLDLKSAGHDMIICEEFWR
NQEKQRLLYSCSLLLLYYMLPLSAITISYCAIAYHLRKRNVPGATSHNQDKWSKKKQKTF
RMLSISVMAFGICWLPLQILNLIRDIDEEFAILDKNYINVIQVTCHLIAMSSACYNPFIYASL
HDKFRFHLRNYFFHHKRRANTMSTKNSRHNTCSTLADIPVVLSEKITFHGRL*
>jgi|Xentr4|391219|e_gw1.628.70.1
METRRPLSSSASSEALDLWNHSSEYNYFIYEGEGPETGGKVLFMARLFIGVTLACVMLICG
FGNLIFILTLAMKKKLRSVTNILIANLAVSDLLVAVVCCPFEMDYYVVREQSWTFGHVICSS
VSYLRMVSLYVSTNALLAIAVDRYLVIIHPLQPRVTLRTACGVLLTIWLTSIFVAAPTAYFAT
ETDFDAPREPGGKVFCGQIWPADRALLYKSYSLFLLAVEFLAPVIIMSLCYIRICHELWFKN
LPGVQTKQLRERLQSRRKTVLVLVAVLLAYVFCWSPFYGYAIVRDFYPGLLLKERHAIALY
YIVECIAISNSIINTLFFVTVKNWPWFCLKGKCERYIIFQGCTTAVQPTAV*
>jgi|Xentr4|391450|e_gw1.631.27.1
SQCSPNCPPGYRKVPREGAPPCCYDCARCSEGEISNITDTEICLKCNDFDWPDQKKIKCIK
KKTEFLSYGEDPLTLAIIVLSVVFILKADIILVILISFRDTPLVKANNRILSFILLVSIKMSILSV
FLFLGQPINMTCILQKASFGITFSIAMSSVLAKTVMVCIAFKASKPDSPWRNWMGAKVPY
CMVLVCSLIQILITVTWLASSPPFVEHDIHSEPGKRIIKCNEGSAIAFYIVLSYMGLLASVSFI
VAFLARSLPDSFNEAKYITFSMLLFCSVWITMIPAYLSTKGKYMVAVEIFAIISSSCGLLFCIF
LPKCYIILFKPEMNSKQYLLGNNHKIINGKY*
>jgi|Xentr4|391494|e_gw1.631.137.1
MLGGSAVPQYLRLLIYLTALPMAPCISVDQSNNSACHLNMKVKAHEEYEYVLEGDIMMG
GVLTVGVRGPFHEQTLLICDKQSAQKYRYLVDFAFRVKEINENPALLPNITLGYHIDDSCG
DTRRALMSLLKIISGTREPVPNYSCRRTGKMAGFIGDLYSVPTESIAHILSALRYSQISYGAT
DPALSDRTTFPYFFWTLQSDEEEYIALCKVMKFFGWNWVGIIYMNNKSGERDYQLLTKYL
SSEGICIEFAWEFSEPFELRQFIRKTTTRVVIICGDPSYEYRVQSYHLHDIIIEKTCIFLSKWLN
HYESLDLASLLTGTVLFMQNRLDNQYDARFREFSDTFHPSNYPHDDLLHNIWLWTQNCL
VKDDDINYFTIFHDIPDKCTGKEKLTDIPEYLNAYHSASLIQAVDMMAMALQDMHNFHSK
QTHGKGRWVGNYNNQLHRYLRNAVYAIDGSPVSSFNEKGEFVHQYDINNPFFDSEGKFS
WKTVGRYVPWAPMEQRLILNPDKLIWNTPDHEVPRAQCTDNCLPGLRKVIEPEKLICCYS
CAPCSEGEISNKTGITACLISITCPRASWTLRARVTCNPLSQEWASAIWELCQGLYGVAELQ
TTLVLCNIIILAVAQDGCPLLEQIPSSQLCFLLLVSIKLSFLSVFLFLGRPVDITCMLRIITFGIT
FSIAVSSLLAKTIMVCVAFKATKPGSSWRKWLGVKLSNSVVLFCSSIQIIICMTWLAISPPFQ
ELDIHTSPGTIIIQCNEGSAIGFYSVIGYMGLLAAVSFVLAFLARSLPDSFNEAKYITFSMLLF
CSVWITMIPAYLSTKGKNTVCVEIFAILTSSAGLLASIFLPKCYTILYRPEINTKANLLRNKSL
*
>jgi|Xentr4|391529|e_gw1.631.15.1
YIEILSFLFIIDEINRNPDLLPNITLGYHVLDSCADPSLAIGSALQILSGPREMVPNYSCRGKG
EISGLVGDRSAITSLPIAQLLSIYGYSQVSYGATDPLLNDRTLYPYYFSTGLNEHIQHIAIAEL
VERLGWTWVIILIDDDDHGQSKNLRNEITKHNACIDFIATLTGDINTNRRTLERIKQSSAEV
VILSGGIFRTHSLVSYLETMIQEKTLVVPAMWVPIYTLRLFNGSLLFGEARDHFEDNTEFEQ
YSLAITEDMLLYDLMTIEGFCLTHDEEKDGLFQQAYQKVYQNCSGLKWSAGYYPSLQVY

RAVNGLVHAEHMMLSSNGKYHSKDSYNNIHKHELHQYLRNVRFPETWGTEIDFNQLLNL
PVKFLIFSRCIYLSFELRQVKVGQYVWSESKSSLEIDLQKIFWKKNTNNQTLKSQCSASCPP
GYRKVPRKRAPPCCYDCIRCSEGEISNATDMENCIKCQDNEWPNQEKTMCIEKQTEFLSY
RNDSLTLVFIVLSMVLILITTLILGIFISFRDTPVVRAHNHTLSFILLVSIKLSFLSVFLFLGRPV
DITCMLRQTSFGITFSIAVSCVLAKTLMVSIAFKATKPGSPWRKWVGVKLANGLVFICSLIQ
FLISVIWLVIAPPYVEKNFHFEPGKIIIQCNEGSVVAFYIVLSYMGLLASVSFIVAFLARSLPD
SFNEAKYITFSMLLFCSVWITMIPAYLSTKGKYMVAVEIFAIISSSCGLLFCIFLPKCYIILFKP
EMNSKQYLLGNSKQRTITQQTGSI*
>jgi|Xentr4|391531|e_gw1.631.135.1
MLGGSAVPQYLKLLIYLTALPMAPCISVDQSNNPACHLNMKVKAHVEYEYLQDGDIMMG
GVLTVGVRGTFTEQTVLVCDKQSAQKYRYLVDFAFRLKEINENPALLPNITLGYHIHDSCG
DTRRALMSLLKILSGTREPVPNYSCRRTGKMAGFIGDLFSVPTESIAHILSALGYSQISYGA
TDPALSDRTTFPYFFRTLQSDEEEYIAVCKVMKFFGWNWVGIIYMNNKSGERDYQLLTKY
LSREGICIEFAWEFTEFKLSQFIKKTTTRVVIICGDPSYEYNIQSYDLHDIIIEKTCIFLSKWLN
HYESLDLASLLTGTVLFMQNRLDNQYDARFREFSDTFHPSKYPHDDLLHNIWLWKQKCL
VRDDDDINYYSLLDIPPGKCTGKEKLTDIPEYLNAYHSASLIQAVDMMAMALQDMHNFH
SKQTHGKGRWVGNYNYQVSGFVCRCSYWDGTTCCTCLGSVSGCGTEQSLPNHSLVVISR
HVVGFWTRYYKRGHSS*QNMAELPTSREKGEDRQAQGSPASEPKFAKEPHTAQKLLIYCN
PCWAVKGKCPANSENCIRCPDLEWPNKNRTICILKLEEFLSYNNDAIAVVLLSISVLFFLITL
LVLVVFISNHDTPIVRANNRSLSFLLLVSIKLSFLSQIFGRALNFFAADFFMPVSSLLAKTIM
VCVAFKATKPGSSWRKWLGVKLSNSVVLFCSSIQIIICMTWLAISPPFQELDIHTSPGTIIIQC
NEGSAIGFYSVIGYMGLLAAVSFVLAFLARSLPDSFNEAKYITFSMLLFCSVWITMIPAYLS
TKGKNTVCVEIFAILTSSAGLLACIFLPKCYTILYRPEINTKSNLLGNKSL*
>jgi|Xentr4|391551|e_gw1.631.79.1
MEVILCLAVIWVRPCSTELSGSDSPCRIPLTKPKYEYKYFKDGDIIIGGILSVHAGVYHIPNN
TGKYIPLCINPNSNYYRDIISLLLVTEEINRNPDLLPNVTLGYHVYDSCLDPSLAIGNVLQIL
SGPGEMVPNYSCGDYGKVAGFIGDRSGVTSLPIAQLLSPYGYSQISYGTTDPALNDRTLYP
YYFSTGLNEHIQHIAIAELVERLGWTWVTILAPGDDRGERESNSLKDEITKHGACVDFIGR
LTEDKNTNIRTLERIQKSTAEVVVLCGGLFRTESLISLVETMIKDKTLVVPHTWLHLYSLML
FNGSLLFYHEYNGCYKISEYKPNKEDNLNKDLFFYTHRCLSQDKEKDELFQRVYGRVFQN
CSRLKNSHWPHYPTDQVYRAATGLAQAEHIMLSSSGKSHNKDIPKYIHREQLHRYLRNVR
FPEPCGTEIDFSKLNKSPVKYRILTWPIYLNKKIKQIPVGEYYWSDSGSSLKIDIQKIFWKKN
TNNQILKSQCSTNCPPGYRKTPRERAPPCCYDCARCSDGEISNSTDMENCIKCHIYEWPNE
EKTMCIEKQTEFLSYGDDSLTLVFIVLSLVSVLLVTVILGIFISFRDTPVVRANNHTLSFLLLV
SIKLSFLSVFLFLGRPVDITCMLRIITFGITFSIAVSSLLAKTIMVCVAFKATKPGSSWRKWLG
VKLSNSVVLFCSSIQIIICMTWLAISPPFQELDIHTSPGTIIIQCNEGSAIGFYSVIGYMGLLAA
VSFVLAFLARSLPDSFNEAKYITFSMLLFCSVWITMIPAYLSTKGKYMVAVEIFAIISSSCGL
LFCIFLPKCYIIILKPEMNTKQYLLGNNK*
>jgi|Xentr4|391559|e_gw1.631.138.1
QNYKYLLDFLYLIKTFNDNQLLAQNLTLGYHIYDSCGDVHKAEKSIFQILSGLRNPVPNYS
CVGKRNIVGFIGDLTSETTIPIAHILSVLGYSQISYGATDPFLSDRDTFPYFFRTVQSKKGQF
YVITQLLKYFGWTWVGVITTNDINGEGAYQLLKHYLSSEGICIDFTLNIQVEEIDAEENILH
KGIIQKFSSSVIIFCGTINLKTAMTLNELSDTLSEKTFIYTSDWLYYSHVFNYGFQLFNGSLV
FMQNRKAYKKSNPQFTMFLESLHPSRYPEDKLLETIWLHHHSCISENMSLSPFHKIHFSHL

KKCKGEERLTDIEAFRGHFHTSNMILAVDALSQISIKYHSMQALLSKESPKNYRNELNYKF
RIRSLLKIWQFHYKDTLIKAFNEHGELVSPFWITNFYSRSKEYISETQVGWYIPWAPLDQKL
NISLEKIKWKTKHNKIPRAQCSDKCLPGFRKAPRPRAQSCCYDCVQCSEGEISTITDSENCI
RCSDMEWPNEKRNKCTKKMEDFLSYTNDVMTVIFSSISVLLFVITLMILGVFITNWDTPIV
RANNRSLSFLLLVSIKLSFLSVFLFLGRPVDITCMLRIITFGITFSIAVSSLLAKTIMVCVAFKA
TKPGSSWRKWLGVKLSNSVVLFCSSIQIIICMTWLAISPPFQELDIHTSPGTIIIQCNEGSAIG
FYSVIGYMGLLAAVSFVLAFLARSLPDSFNEAKYITFSMLLFCSVWITMIPAYLSTKGKNT
VCVEIFAILTSSAGLLASIFLPKCYVIMFRPEMNHKSGILGGNN*
>jgi|Xentr4|391562|e_gw1.631.74.1
MLSASWSLTKDLSLAQLRLLFCLAVIWVRPCSTELSGSDSPCRIPLTKPKYEYKYFKDGDIII
GGVLSVHTGVYHIPNNTGKYIPFCINLNYNYYHDIISLLVVTEEINRNPDLLPNVTLGYHV
YDSCLDPSLAIGNVLQILSGPGEMVPNYSCGDYGKVAGFIGDRSGVTSLPIAQLLSPYGYS
QISYGATDPALNDRTLYPYYFSTGPSEHIQHIAIAELVERLGWTWVTILAPGDDREERESNS
LRDEITKHGACVDFIGRLTEDKNTNIRILERIQKSTAEVVVLCGGLFYTESLISLVETMIKDK
TLVVPHTWLHLYSYMLFNGSLLFSNEYNRCYETYEYKPNKEDNLNKDILFYSHRCLTQDK
EKDELFQRVYGRIFHNCSHLDISLWPHYSNDQVYRAATGLAQAEHIMLSSSGKSHNKDIP
KYIHREQLHRYLRNVRFPEPCGTEIDFSKLNKSPVMYEILTWPIYLNKSSEQITVGEYYWS
DSGSSLEIDIQKIFWKKNTNNQILKSQCSTNCPPGYRKTPRERAPPCCYDCARCSDGEISNS
TDMENCIKCHIYEWPNEEKTMCIEKQTEFLSYGDDSLTLVFIVLSLVSVLLVTVILGIFISFR
DTPVVRANNRNLSFILLIAIKLSFLSVFLFLGRPVDITCMLRQTCFGITFSIAVSCVLAKTMI
VCIAFKASKPGSPWRKLLKIHLAHGIVVICSFIQTLINVIWLALSPPFMELNILSDPGKIIIQC
NEGSVVAFYIVLSYMGLLASVSFIVAFLARSLPDSFNEAKYITFSMLLFCSVWITMIPAYLST
KGKYMVAVEIFAIISSSCGLLFCIFLPKFYIIMLKPEMNTKQYLLGNNK*
>jgi|Xentr4|391569|e_gw1.631.133.1
MALIILFRFLIYIMSVNSVGYCLSAVPPVSSACKLQIIKTLEDYEYIQEGDIMIGGVITVNAL
AHHYDSKSGKFVLCYKPNLQNYKYLLDFLYLISEFNKDQYLSQNLTLGYHIYDSCGDVYK
AESSVLQILSGLRDPVPNYSCAGKRNIVGFIGDLTSETTIPIAHILSVLGYSQISYGATDPFLS
DRVTFPYFFRTVQSKDGQYFAISQLLKYFGWTWVGIITTDDMNGDEAYQLLSNYLSSEGIC
IEFSIKLNNQNVYKELMQHKSSSVVIFCGTINLKTAVKLQELSDMFSDKTFIFTSDLIYYSYL
IYHALTLVNGSLIITQNRADYIRSNTRLTQFIASVHPSRHPEDKLLEAIWMLHHSCMAENM
SLTFTHKLIYSPHLPNCTGEERLSDIEAFNEEFHTRNMIIAVHALILECSKMHYRQNFITEQK
SGKNRKVIHFKHQLYHLLNKMLYPYEGQFMKSFNENGELVSPYHINNLFITNDYVSDKKI
GLYIPWAPPNQKLNITHNAINWKTKYNKVPRAQCSENCLPGFRKAPRPRAQSCCYDCVPC
SSGEISNTTDSENCIRCPDTEWPNANKSACVVKGAEFLSYTNDVISVFFSSISVLLFVITLLI
LGIFIIYRDTPIVRANNRSLSFLLLVSIKLSFLSVFLFLGRPVDITCMLRIVTFGITFSIAVSSLL
AKTIMVCVAFKATKPGISWRTWVGVKLSNSVVLFCSSIQIIICMTWLAISPPFQELDIHTSH
GTIIIQCNEGSAIGFYSVIGYMGLLAAVSFVLAFLARSLPDSFNEAKYITFSMLLFCSVWITM
IPAYLSTKGKNTVCVEIFAILTSSAGLLACIFLPKCYTILFRPEINTKSILRGNKFC*
>jgi|Xentr4|393174|e_gw1.657.173.1
MCNLIISSLSDRNQTKVKNVILQGIVGPESLTVPLYFIFLMIYILTLCGNLLIIGLFLQGRHLTS
PMYFFLSHLSLCDILLSTSVVPNLLSALLKKGETMTFGGCITQFFASGLATAAECHLLTAMS
YDRYLAICTPLHYIRIMTSRLCVRLVTLSWFLTLIYCIIDITEILDLDFCGCNVLDYVYCDVA
PLLEISCKNTSNVQMTTTILSIPGALLPFLFILSTYIKISLTILRISSNIGKQKAFSTCSSHLTIVC
TYFGILIAKYIVPSKGQSLNMNKAISLLYTAVTPLLNPMIYSFRKKEIRAAVTKWTSMKTGK

FT*
>jgi|Xentr4|393176|e_gw1.657.120.1
MKMNNHSMVTEIFLLGFQHLSNFKILVFSVILLIHILTVYENVLVIALVTVSRGLQSPMFFFL
RQLSFSDLLESMVIVPTLLSTVMNEGAKIPLIGCIVQLYLFGVTATLQFFLLTVMSYDRYLAI
CNPLRYSSIMSHRVCVKLIAISWLLALSVTSVVVIPAVTQEFCNQNTINHFFCDYFPLLELSC
SDTSLAQILAITLSTPVVVFLFILIIGSYICIAHEILKIVSSIGRQKAFSTCSSHLAVASIFYGTLI
GIYVVPTRNQSQTISKLLSILYTVVTPFINPMIYSLKSADMKNAIKNIVQ*
>jgi|Xentr4|393200|e_gw1.657.8.1
MNTTSSCNQSKITEFLLVGFSAPRPLRVLLFSICLVIYIMALGANLMIIALYLGSHHLRSPMY
FFLSNLSATDILLSTTVGPNLLCTFLNNGNPMSVPACVTQFFAYGLFTGVECLLLTVMAYD
RYLAICKPLHYVTIMTNKHCLHLVIWSWFEILILSLSSTIFTSILGFCGCNTLDHVYCDFAPL
LKASCSDAFVMDSMTTILIPTGTILVLLSPLFFIITSYVSISLSIFKISSKTGRQKAFSTCGSHL
TVVCTYYGILFSKYSVPSKGQSLNVNKFTSLLYTLVTPLFNPIVYCFRNKEIQKTLSTLISVR
LRQKREHIRIK*
>jgi|Xentr4|393206|e_gw1.657.86.1
MNTTSSCNHSKITEFLLVGFSAPRPLRVLLFSICLVIYIMALGANLMIIALYLGSHHLRSPMY
FFLSNLSATDISLSTTVGPNLLYTLLKDGNPMSVSACVTQFFAYSLFTGVECLLLTVMAYDR
YLAICKPLHYVTIMTNKHCLHLVIWSWLEILIFSFSGTIMILHSGFCGCNTLDYVYCDFSPL
AKLSCADVFVMDTMATILLMIATLLLCLSPLLFITTTYVSISLSIFRISSKTGRQKAFSTCGS
HLTVVCIYYGILSSKYTVPSTGQLVDVNKLSSLLYTLVTPLFNPIIYSLRNQEIQKALIKLVL
KIQDGTKRTGLKKSNL*
>jgi|Xentr4|393214|e_gw1.657.25.1
MNTTSSCNHSKITEFLLVGFSASHPFRELLFSICLVIYIMALGANLMIITLYLGSHHLRSPMY
FFLSNLSATDILLSTTVGPNLFCTLLQDGNPMSVSACVTQFYAYCFFTGVECLLLTVMAYD
RYLAICKPLHYVTIMTNKHCLHLVIGSWLGILLLSLPITIIISHSGFCGCNTLDYIYCDLTPLL
KVSCSDVFLVDTIATIILTIISLLVLQLPVLFVITTYVSISLTILKMSTKTGRQKAFSTCGSHLT
VVCTYYGILISKYTFSSKGQSISVNKLISLLYTLVTPLFNPIVYSFKNQELQKALSKLISLRLP
TPMH*
>jgi|Xentr4|393215|e_gw1.657.107.1
MNSMSSCNQTKITEFLLLGFHGSETLRRFFFCICLLIYMLVLGGNLLIVVLYLGSHQLRSPM
YFFLSNLSITDILLCTNVVPNLLSALLSGGNPMSVSACVTQFFAAGIFTGAECLVLTVMAYD
RYLAICKPLHYVTIMTNKHCLHLVIWSWVEIVFTGVSITITTSHSGFCGCNILDYIYCDYLP
LLKVTCSDNSALESMIAVISIPVLVLPFFFLVTSYVCIFLSILRISSRTGRQKAFSTCSSHLTVV
CTYYGILFAKYIVPTKGQSLNVNKTISLLYIMVTPLFNPIIYSFRNQEIWAALNKWISVKVSP
HKMVFV*
>jgi|Xentr4|393217|e_gw1.657.3.1
MGSNKTAVTDFFLLGLQQYNNQKFLVFFVLLIMYVTTLTGDLMIILLVSTSQRLQSPMYYF
LKHLSMSEIMFTTNIIPNMLYIVLKEGRFMSIVCCFIQFYIFIASGSVESLLLTIMSYDRYLAI
CNPLRYSYLMNLSICRTLVSCSWLLCFMLVLVTVILLCQLQFCFSNIIDHYFCDLEPILELSC
SDTSLVRTEVLVLSIPTVIVAFTFIIITYISIFYTVLRITSSAGRHKAFSTCSAHLTSVCSYYGPL
IIIYVVPYRGSLLNANKLLSLLYTVVTPLINPIIYSLRNQEIREVLATFWNTKRKH*
>jgi|Xentr4|393226|e_gw1.657.102.1
MDSCNQTKIAEFLLLGFHGPQATRRSIFCICLSIYMMALGGNLLIVALYLGSHHLRSPMYFF
LSNLSVTDILLCTSVVPNLLSSLLRDGNPMSVSACVTQFFANGVFTGVECLLLTVMAYDRY

LAICKPLHYVTIMTNKHCLHLVIWSWLEISFINVPATVIISHSGFCGCNTLDYIYCDFLSLLE
VSCSRMFIMEKVIAVVSIPVVVLPFFFIVTTYVCIFHSILRISSNTGRQKAFSTCSSHLTVVCT
YYGILFAIYTVPSKGQSLNVNKIISLLYIMVTPLFNPIIYSFRNQEIWRALSNWLSIDVLQHK
LISG*
>jgi|Xentr4|393239|e_gw1.657.142.1
MEGNKTTATDFFLLGFQQYSGHKILVFISLLLVYLLTLTGDLMIILLVSTSQQLQSPMYYFL
KHLSMSEILATTNIVPNMLYIILREGSSMPIICCFIQFYIYIASGGVESLLLTIMSYDRYLAICN
PLRYSYLMNLQTCRTLVICSWVLCFLLMLVTVSFMCQLQFCVSNIIDHYFCDYEPLLELSC
SDTSPVRTEVLVISIPTVIAAFTFIIITYICIFMTILRISSSVGRKKAFSTCSAHLTSVCSYYCPLI
IIYVVVPYRGSSLDANKSLSLLYTVITPLLNPLIYTLRNREIMNTLHNFLNKKRSNIKRKQHK
LK*
>jgi|Xentr4|393251|e_gw1.657.182.1
MIGPNGSNKTTITYFILHGINGPQILRMFFFITFLILYMMTLFGNLLIAGLFLQSQHLGSPMY
FFLSHLSLCDIFLSSSVVPKLLSTLLRTGERMSIIGCFVQVFASGSFTGAECNLLTVMSYDRY
LAICNPLHYINIMNTRFCFQLLVWSWCLSLLYCLIIIAEMSDLEFCRCNIVDYIYCDVAPLLE
ISCKDSMILQITTIVISIPAVISPFLIIIATYVSISITILRISSNSGRQKAFSTCSSHLTIVCTYYGILI
AKYTIPSKGRSLNMNKFISLLYTVVTPLLNPIIYSLRNKEIRETVAKNIYMK*
>jgi|Xentr4|393262|e_gw1.657.105.1
MNTMSSCNHSKITEFLLVGFSAPRPLRVLLFSICLVIYIMALGANLMIIALYLGSHRLRSPM
YFFLSNLSATDILLSTTVGPNFLYTLLKHGNPMSVSACVTQYFASGVLTGVECLLLTVMAY
DRYLAICKPLHYVTIMTNKHCLHLVIWSWLEIVLFMLPVTITVSHSGFCGCNTLDYIYCDT
APLLKMSCSDVFVLERAITVINIPVTVIPLLFIFTTYGTILLSIFRISSNTGRQKAFSTCSSHLT
VVSMFYGILITKYTVPSKGPSLNMNKIISLLYTLVTPLFNSIVYSFRNQEIQNALSKVNSFRL
EKRRVSAGE*
>jgi|Xentr4|393265|e_gw1.657.161.1
RNDSLIQDFVFEGFKDLNHFRIPVFVLVLLVYVATIAGNNLIITLVTTCRQLQSPMYFFLSHL
SLCDILISTTITPNTLRVIFDRTSPISLNCCLTQWYFFGASAIIECCLLTVMSYDRYLAICNPLL
YSSIMSHHLPRYLALWPWLVGFLLAIVTNVLVSEVQFCGSIIDHFFCDLAPLLKISCSDTSA
LQIYVSVLAFVIGISQMLFVIATYIAISISILKISTTKGRQKTFSTCSSHLAVVSVYYGTLISVY
MAPSKGYSLTLNKILSLFNTVVTPLFNPIVYSLKNKEIRTATAKFLLKAPYPK*
>jgi|Xentr4|393269|e_gw1.657.153.1
RNDSLIQEFVLEGLKELKCFQIPVFVLVITVYVATILGNLLIITLVTTCRQFQSPMYFFLCHLS
LCDILISTNVTPNTLRVILNRTSPISVNCCLTQLYFFAASAIIECCLLTVMSYDRYLAICNPLL
YSSIMSHNLPRYLVLWPWLAGFLLAVITNVLVYKLQFCGHNIINHFFCDLAPLLDLACSDT
SAMESYASIVTFVLGISQFSFVIATYIAISISILRIPTTMGRQKAFSTCSSHLAVVSTYYGTLIT
LYLNPSKGHSMNVNKILSLVNTVVTPLFNPIIYSLKNKEIRAATAKLLMKATYRK*
>jgi|Xentr4|393270|e_gw1.657.100.1
MNSMSNCNQSKITEFLLFGFHGPQTTRRSIFCICLSIYMVALGGNLLIVALYLGSHHLRSPM
YFFLSNLSITDISLSTNVVPNLLSALINDGKLMSVSACVAQYFASGVFTGGECLLLTVMAY
DRYLAICKPLHYGTIMTNKHCLHLVIWAWVEIVVICAYTSTATSHSGFCGCNTLDYIYCDY
LPLLEVSCADVSILEKMIPVVTMPVLVLPFWFIITTYVCISLSILRISSNTGRQKAFSTCSSHL
TVVCTYYGILVAKYLVPSKGWSLNVNKIISLLYIMVTPLFNPIIYSFRNQEIWKALSKWISV*
>jgi|Xentr4|393302|e_gw1.657.164.1
MEIINQTKTTGFILLGLEGSYTQLVFLFVLFLLLYIATLCANFVTIALVLGSRRLHTPMYFFL

SQLSSSDILLSTNIVPNLLCALLKEGKDMSVPSCISQFFACSSFTAAECFILAVMSLDRYVAIC
NPLRYVSIMSIKLSVHLVLWSWLLSFLLCVIVAVFVSLLEFCGLNAIDYIYCDFAPLLEVSCS
DTSTVEIIAIAFTFPIIIFPFLVIILTYTSISFSIMKISTNSGRQKSFSTCSSHLAVVCTFYSILIAK
YTVPSKGQSLNISKVISLLYIVVTPLLNPIIYSLRNNEIRKALIKWIS*
>jgi|Xentr4|393304|e_gw1.657.156.1
MQMNNHTMVTEIFLLGFQHLSNFKILIFSLILLIHILTVYENALVIALVTVSRGLQSPMFFFL
QQLSFSDLLLSVVIVPTLLSTVMNEGAKISLIGCIVQLYFFGTTEALQCLLLTVMSYDRYLA
ICNPLRYSSLMSHKFCDKLIAISWLLSLSVTSVTVITLATQEFCNQNTINHFFCDYFPLLELS
CSDTSLAQILVITVSVPAIVFPLMFIIGSYICIAHEILKIVSSIGRQKAFSTCSSHLAVVSIFYGT
LIGIYVVPTRNQSQTISKLLSLLYTVVTPCINPMIYSLKSADMKNALKNIMQ*
>jgi|Xentr4|393316|e_gw1.657.98.1
MNTTSSCNQSKITEFLLVGFSAPRPLRVLLFSICLVIYIMVLGANLMIIALYLGSHRLRSPMY
FFLSNLSATDILLSTSVGPNLLCTLLKDGNPMSVSACVTQYFASGFFTGFECLLLTVMAFDR
YLAICKPLRYVTIMTNKHCLHLVIWSSLEIVLFLLLVIILISHSGFCGCNTLDYIYCDIAQLLK
KSCSDVFVLETSITMITISVAVIPLLFIVTTYVIISISIIRISSNTGRQKAFSTCSSHLTVVSMFY
GIIIALYTVSSKGPSLNMKKIISLLYTSFIPLFNPIIYSCRNQEIWKALGLL*
>jgi|Xentr4|393330|e_gw1.657.93.1
MNTMRTCNQSKITEFLLVGFSAPRPLRVLLFSICLVIYIMALGANLMIIALYLGSHHLRSPM
YFFLSNLSATDILLSTSVVPNLLSALLSDGKLMSVSACVTQFFAYIFFTGVECLLLTVMAYD
RYLAICKPLHYVTIMTNKHCLHLVILCWFEILILSLSGIIFTSILGFCGCNTLDYVYCDFAPLL
KVSCSDAFVMASMTTLLIPIGVVLVFLSPLLFITTTYVSISLSIFRISSKTGRQKAFSTCGSHL
TVVCTYYGILISKYAVPSKGQSGNLNKLTSLLYTLVTPLFNPIVYSLRNQDIQNTMTALISVR
PRGYDIKENI*
>jgi|Xentr4|393331|e_gw1.657.175.1
MSSINSCNQTKITQFLLLGFHGPQILRISLFYIFLLIYIMALGGNLLIVALYLGSHHLRSPMYF
FLSNLSITDILLSTNVGPNLLSALINDGKLMSVPACVTQFFTSGVFTGVECLLLNVMAYDR
YLAICKPLHYVTIMTNKHCLHLVIWSWVEVAVISLFAATATSHSGFCGCNTLDYIYCDYLPL
LEVSCDDVSILERVIPVVTIPVVVLPFLFILTTYVCIFNSIFRISSNTGRQKAFSTCSSHLTVVC
TYYGILCAKYIVPSKGPSLNMNKIISLLYIMVTPLFNPIIYSFRNQEIWVALSKWVTSQDNL
QLIQPPGPRARDKIISMH*
>jgi|Xentr4|393332|e_gw1.657.85.1
MCSLIIGSPSDRNLTEITHFILGGIGGPQSLKVPLYFIFLMLYIMTLCGNLLIIGLFLQGQQLRS
PMYFFLSHLSLCDILLSTSVVPNLLSALLTEGESITFIGCITQFLATGATTAAECYLLTAMSYD
RYLAICNPLRYIRMMNSRLCAQLVTWSWFLSLLYCTIDITEIYDLDFCGCNVLDYIYCDIEP
LLDISCKNTSKLQKTTMIISVPAALFPFVFILSTYIKISLTIFRISSNTGKQKAFSTCSSHLTVV
CTYFGILIAKYTLPSNVQSLNVNKAISLLYTAVTPVLNPIIYSLRNKEIRDAVTKWTFMRMS
KFR*
>jgi|Xentr4|393342|e_gw1.657.24.1
MNTTSSCNQSKITEFLLVGFSAPRPLRVLLFSICLVIYIMALGANLLIIAVYLGSHHLRSPMY
FFLSNLSVTDISLSTSVGPNLLYTLLKGGNPMSVSACVTQFYAFGLFTGLECLLLTVMAYD
RYLAICKPLHYATIMTNKHCLHLVIWSWLEIFICCSSSTIIISHSGFCGCNTIDYVYCDFSPLA
ELSCSDDFVMNTVTTIVLTIATLLLILSPLLFVITTYVFISISIFRISSKTGRQKAFSTCGSHLTV
VCTYYGILISQYTVPSKGQSGNVNKLTSLLYTLVTPLFNPIIYSIRNKEIQKALSNLKAHRFF
RE*

>jgi|Xentr4|393343|e_gw1.657.106.1
MNSMSSCNQTKITQFLLLGFQGSQVLRSSLFYLCLLIYIMALGGNLMIIALYLGSHHLRSP
MYFFLTYLSITDILLCTNVVPNLLSALLTNGKPMSVSACIIQYFISGVFTGGECLLLTVMAY
DRYLAICKPLHYVTIMTNKHCLHLVIWSWLQIVFLSGSLAIWTLHKGFCGCNTLDFIFCDF
LPLIKVACSDAFVIERWMTMLCIPVVVLPFLFIVTTYVCIFHSILRISSNTGRQKAFSTCSSHL
TVVSTSYGILLAKYAVPTKGQSLNVNKIISLLYVMVTPLFNPIIYSFRNQEIWKALDKWISA
KVLIQYPGIRAH*
>jgi|Xentr4|393361|e_gw1.657.96.1
MNSMSSCNQTKITQFLLLGFHGPQVLRSSLFYICLVIYIMALGANLLIIALYLGSHHLRSPM
YFFLSNLSVTDISLSTNVVPNLLSALLTNGKPMSVSACIIQYFISGFITGAECLLLTVMAYDR
YLAICKPLHYVTIMTNKHCLHLVIGSWVEILLLSVLATIMTTNSGFCGCNTLDYIFCDFLPL
LEVSCSDAFIIEKGVAVLTIPVLVLPLLFIVTTYVCIFLSILRISSNTGRQKAFSTCSSHLTVVS
TCYGILLAKYAVPSKGQSVNVNKIISLLYIMVAPLFNPIIYSFRNQNIWKALSKWMYVMFP
HAK*
>jgi|Xentr4|394406|e_gw1.672.81.1
NVLKMSLTMEVIKITIIIVTWLCGSILNSSIVAVYLRDWKNGMSLGECDRTILSMGCNNLLL
QGCLSVSEFINIFQLYRLFFKEFTVLGCILFLFLSYLSMWLTVCLSICYCVKLANISHGLFVR
LKRGISTASTLFLFGSVVASCLINVPLIWTMDTEFLENTTADNVIYKLDIKFMSFNIVFGCCV
PILVTSLCIGLSVMSLLRHVQRMKNNTSPSWNPQLKSHERACRTMSLVLILNLTFSVTIITL
AMGLMNHKDVLVNDILYWSVVLTNPSAAAIVLIFGNTKLKTALSKICC*
>jgi|Xentr4|394438|e_gw1.672.84.1
MLSEFDLIFAVALVISWTCGTVLNSSIVAVYLSDWKKGLNLGACDQIILTMGCNNLLLQWF
LTLNLIFVVYGLYSLLAKVLLVAVVSLVLNFGIAFSFWLTAWLSSYYCVKLVNFSNRFFIRL
KREASGVVTYCLLGTAVILFSTRLPIIWSVQITTDQNLTRISNVLYDNIVHLSFNTIFIYFLPTI
ITSFCIGLSLMSLLKHVRKMKQNTSQFWNPQLKSHIKACRTMFLLLTVNVIFFLSISIFSMLF
SKTEGTGQYVTRFIMLLNPSSQAIILLFGTSRLATAWSKVLFSK*
>jgi|Xentr4|394463|e_gw1.672.22.1
MVNETLLSMLVLSGLSDLQGLQLPLLLFFLLVYLMTIISNFIILLSICKDSHLKTPMYFFLGC
LAWLDMCCSSVTAPRMLFDIHTKKRVISVEACITQLFFFIFFVISEVFLLAGMSYDRYIAICY
PLHYMQLMHSRLCVQMLSGILFLALVYSLVHTLCVLRLHFCRSNIVESFFCDLPQLFQLSC
TDTTINILLIFLLGGVMGLGSLAMTFLPYISIFTTVLNIRVQNTRQKAFSTCTSHLAVVFIFY
GTLFFTYFRPTTNYHVTEGRLVSVFYTVFTPLLNPLVYSLRNQELKSAFRRVLRSL*
>jgi|Xentr4|394464|e_gw1.672.59.1
MDLHNFGLVFWFFLLILEVIIGTLTNAFIVVVLLLGYFKKQTMNESDKILIALSITNICSSLVS
SAAIMILFMWPWIYSHSNATFCIFSLTIFGTISMVWLTACLCVFYFVKILNFSSGILLWAKMK
ISNFVPWLIFFSELVSLCWTFFTMLPLVTKEQSSGNISSLHSVNATSDTNTIIFELICTAVSLPL
MIIIITTFSTTGSLYLHRRRMEKNLGASSSLKAHQSVVWMMIRLLLLYTLVFVVQILHFSGT
VAPLSFEYCLNYFILFSIPVAQSVLLIQGNPKLKDKLKQISLVCTTADGTK*
>jgi|Xentr4|394468|e_gw1.672.87.1
MLSEFHLIFVIALVLSWTCGTVLNSSIVAVYLSDWKKGLNLGACNGIILAMGCTNLLLQWF
LTFHLMSLTYQLFIIFAKPLLLSVVSFIVNFSVSLSFWLTAWLSGYYCVRLVNSSNRFFIRLK
RGMSMVVTYCLLGTVVTLFIIQVPVIWKVHTKLNQNLTNIYSAFDNYTELASVNATFACF
LPTIITSFCIGLSLISLLKHIWRLKQNASQFWNPQLKNHFKACRTMLLLLTVNLIFFLAISISF
RYKLDDPRQYVAWFIMSSNPSSQAVILLFGNSRLATAWSKVLFSH*

>jgi|Xentr4|394485|e_gw1.672.21.1
MVNTTILSTLVLSGLSDLQWLQLPLFLFFLLVYLLTMISNFIILLSICKDSHLKTPMYFFLGC
LACLDMCCSSVTAPRMLFDIHTKRRVISTAACITQLFFFIYFAICEMFLLAVMSYDRYIAICY
PLHYMQIMHSKLCVQILLGILFLALVCTLAHTLSVLRLNFCRSNIVENFFCDLPQLFQISCT
DTTVNILVILVLGVFMGMGALAMTFIPYIRIFVTVLNIKEKNMRQKAFSTCISHLAVVFIFY
GTLFFNYFRPATNYHFTEGRLISVFYTVFTPLLNPFVYSLRNQEFQSALRRVLRGFRIFNKNI
FSSSL*
>jgi|Xentr4|394489|e_gw1.672.9.1
MVNETILLMLVLSGLSDLQGLQLPLFLFFLLVYLMTMISNFVIILSICMDSRLKTPMYFFLG
CLACLDMCCSSVTAPRMLFDIHTKKRVISVEACITQVFFFMFFVISEVFLLAGMSYDRYIAI
CYPLHYIQMMHSKLCVQMLSGILFLALVCTLVHTLSVLRLRFCRSNIVESFFCDLPQLFQLS
CTDTTINILLIFLLGGLLGLSALAITFIPYIRIFATVLNIKEKNMKQKAFSTCTSHLTAVIIFYG
TLLFNYFRPTTDSHFKVGRLVSVFYTVFTPLLNPLVYSLRNQELKTALRRMLNRL*
>jgi|Xentr4|394686|e_gw1.674.7.1
MSNSVEIHLSNSTMGFSTTTYEDETIVYMCSNDESIKFGAAAVPFFYYTVFTLSLLGNGLIL
FLLLKYEKIKTVTNLFILNLVISDLLFTITLPFWAFYHSNEWVFGNGMCKVVSSVFFIGFFSC
ILFLTVMTMDRYLAVVHAVSAARTRKLIYVYVASIAIWVISFVSTVPKFVLYGTRKHDSAGI
LCEETGFSADKIDTWRRLGYYQQLTMFFLFPLIVILYCYTLIVVKLFNTKMHNKDKAVKLI
SVIVLAFFICWTPYNVVIFLRLSPGDPCNDYLNNAFYICRNIAYFHCCINPFFYTFVGTKFRR
HLSALLGTNCLSMFRRSSSSSRTSEYSPQTIYE*
>jgi|Xentr4|394697|e_gw1.674.51.1
EEYEDQYTLSTLDYIIFDPGVFCEKNSVREFASYVLPPIYWCVFLFGLVGNSLVLAVYVYN
RKLKTMTDTFLINLAIADILFLITLPFWAIAASHDWVFKTALCKAVNSMYSVNVYSGMLLL
ACISIDRYIAIVQATKAQKYQTKKLLISKLTCFIVWALSTGLSLPEILFSVVKEEFNSTTCTM
SYPAELSKTFKVSVLSLKVTVAFCLPFLVMVFCYAMIIPILVQARGFQRHKALKVIFAVLSV
FILSQLPYNSILVLRVLNAANINDFECATTQNIDIAYKITQSVAFLHCCLNPFIYVFVGVKFRS
DLLKILQKCIGEQQWAKGLWGDNNKKPLSETRESKMGTLSL*
>jgi|Xentr4|394765|e_gw1.675.79.1
MCKGIGWFILFTVWVLCTFATEACAAPGSNTNCRLPKEAVTGYLSRPGDIIMGGTFLIHLD
RVYSDIKFTSKPPELQCQMFAIEYYQGMQALIFAVEEINSNPELLPNITLGYDVLDTCYTLR
RAAQGTLSMLSGGTQSTPNYNCHQGTRLAGVVGDSGSTRTILMAQILGLYQYPQISYFATS
PVLSDRNLFPSFFRTVPSDEFQMRGLAQLVSHFGWSWVGLLATDNDYGQYGLQIAMQEII
NSGACVAFNEYILTGRPDRNAPRLAQVIKESTSKVVIAITGDSDLVIVLEELLRQNVTGKIW
IATEAWATANLLSNERFQGILLGAIGFAIYSGQMPSFSDYLNSLHPSNDLYDPFIKEFWEQAF
SCKWPSQKNVTLVDNAREQTCTGNERLESVLTKVDRRVSGNVYTAVYAIAWALHNLLNC
TAGVGPFHHGACANISSFHPWHLLQYIKKVNFKTPAGNRVFFDERGNPPAIYDIVNWHLTS
QGSLGQITVGKYDLSAPNGKSLYIDNGLINWASKTQVPVSKCSPSCSSGFRKVIVPRKPPC
CYDCAQCPQGQISNQTDAVECHPCSWDTWPNLQQDRCLPRPTEFLSYGDPLGYSLAAISIF
SSLIPLGVLGVFIHQKKTPIVRANNYSLSCLLLLSLFLCFLCSLGFIGYPQPEKCLLRQVAFG
MVFALCISCVLAKTITVVIAFNATKPGSRLRKWTGVKVSYCVIGFCIFVQIIDCALWLIFSPP
FHELDTDTQPGVIIANCNEGSPTAFWCMLGYLGLLASISFIVAFLARRLPDSFNEAKLITFS
MLAFLSVWVSFIPAYLSARGMYTVAMEVFAILSSSWAVVGCIFVPKCYIVLFRPNLNSREN
LIGKRTT*
>jgi|Xentr4|394771|e_gw1.675.26.1

MLSRGTESTPNYNCHQGTRLAGVVGDSGSTRSILMAQILGLYQYPQIPPSKCSPSCPSGFR
KVIVPGKPPCCYECARCPQGQISNQTDALDCHPCSWDMWPNLQQDRCLPRITEFLSYENP
LGYSLAAISMLSSMIPLVILGIFIHYKNTPIVKANNYSLSYLLLLSLFLCFLCSLGFIGYPQPE
KCLLRQVAFGMVFALCISCVLAKTITVVIAFNATKPGSRLRKWTGVKVSYCVIMLCALIQII
DCALWLIFSPPFHELDTDTKPGVIIVYCNEGSPTAFWCMLGYLGLLASISFIVAFLARRLPD
SFNEAKLITFSMLAFLSVWVSFIPAYLSARGMYTVAMEVFAILSSSWAVVGCIFVPKCFIILF
RPNLNSRENLIGKRT*
>jgi|Xentr4|394777|e_gw1.675.90.1
MSQPGDIVIGGTFMVHLERSFNDPDFRTKPPELQCKLFATDYYQNMQALIFAVEEINADPE
LLPNVTLGYQVFDTCFTLRKAAQGTLSALSGGEEMTPNYHCLKGAPLAGVIGDSWTTTSI
LMAQILGLYRYPQASISYFATNPILSDRNLFPSFFRTIPSDEFQMRGLAQLISYFGWTWVGL
LANDNDYGQYGLQILMQEIINGGGCVAFAENILTGQPNRNAPYLAHVIKMSNAKVVVMIT
SDTYFVALMEEMLKQNVTGITWIVSEALSTSNLLSKESIKSIVLGTVGFAIHRGKIPKFTEH
LRSLSPSKNHNDLFISEYWEQIFSCKWPAKETGMENETMGACTGSEKLGNLLTEEYHRLS
LNVYTAVYALAWALHHLQNCIPGTGPFQHGNCANISSFHPWQLLHYVNNVRFKATDGSQ
VFFDARGNPPPIYDIVSWRPSTKGILEPAMVGFYDLNAPDGDTLTIDTTGITWNSNTQVPV
SKCSQSCSPGSRKVIVPGKPLCCYECARCPQGQISNQTDAVECHPCSWDTWPNLQRTKCIP
RMTEFLSYESPLGTSLAAIAILSSMGPLLILGVFKSYKQTPIVRANNYSLSCLLLLSLFLCFL
CSLGFIGYPQPEKCLLRQVAFGMVFALCISCVLSKTIIVVIAFNATKPGSRLRKWTGVKVPY
FLILCCVLIQLCICLIWLIFFPPFHELDTDTKPGLIIVNCNEGSPTAFWCMLGYLGLLVTISFIV
AFLARRLPDSFNEAKLITFSMLAFLSVWVSFIPAYLSAQGMYTVAMEVFAILSSSWALVVCI
FIPKCFIILLRPNVNSRENIMGKRRGCKLRTPELAKNRDFYSPNNFS*
>jgi|Xentr4|394779|e_gw1.675.82.1
MAGCSLPSGNTGESINSPGDIIIGGTFHVHLERVDTNLYFTSKPTEPQCQLIATEFYQTMRA
LIFAVEEINADTELLPNVTLGFHVLDSCDTLRRAAQGTLSMLSGGKEAIPNYHCHGGTPFA
GLVGDSGSTRSTLLAQILGLYRYPQISYFATSPSLTNRNLFPSFFRTIPSDEFQMKGLAQLIDH
FGWSWLGLLANDYDYGQFGLQTVRQEIINSGACVAFAENLLTSQPDMNAPHIINVLKKST
AKVVVVIASDSEFVVVLEELLIQNVSGHIWVASEAWATSDLLSEERFKGILLGTIGFAIFHG
KMPRFGKYFNSLNPSKDVSDPFITEFWEQTFACTWPSEKNQVIVMDNATISPCTGNEMLY
VQPSEEDQRLSLNVYTAVYAIAWALHNLLYCKPGTGPFYLGGCANISSFQPWQLLHYIKN
VNFKTRDGRQVFFDASGNPPAVYDIVNWQVGARGMLEQVTVGSYELNGSRGKTFNIDRA
KILWTNNNTQIPNSQCSPSCPSGYRKVIVRGKPLCCYECARCPQGQISNQTDAAECHPCSW
DTWPNLQQDRCLPRPTEFLSYGDPLGYSLAAISIFSTLAPLVILRIFICNNKTPIVRANNYSL
SCLLLLSLFLCFLCSLGFIGYPQPEKCLLRQVAFGMVFALCISCVLAKTITVVIAFNATQPGS
RLMKWTGVKVSYCVIGFCIFVQLCVCGIWLIFSPPFSEFDTETKPGIIIVNCNEGSPTDFWC
MLGYLGLLATISLIVAFLARRLPDSFNEAKLITFSMLAFLSVWVSFIPAYLSARGMYTVAME
VFAILSSSWAVVGCIFVPKCYIVLFRPNMNSREHLLGKSRT*
>jgi|Xentr4|394784|e_gw1.675.66.1
ISYFASSPILSNRYLFPSFFRTIPSDEFQMKGLAELVFYFGWTWVGLLANDNDYGQYGLQI
AKQEIINGGACVAFTENILIGRPNRNAPNLVQVIKESTAKVVVVISSDSHFVIVAEELLRQN
VTGNIWVASEAWATSDLLFKERFRQILLGTIGFAIHRGKIPAFSKYLKSLRPSNDQYDSLIKE
FWEQTFSCKWPSQENPIVPTDNATMRVCTGEEKLESLLTAEYHRLSLNVYNAVYAMAWAL
HSLLNCTSGTGPFPFGGCTNISSFHPWHLLHYIKNVNFKTKDGSQMFFDAKGNPPAVYDIV
NWHGSANGALEPVTVGSYGLSAANGKALNIDSSGIIWTNSETQYRYKDIPSVTFLGGSMS

QFELQPPEEGNGSCRTYAAECHPCSWDTWPNLQQDRCLPRPTEFLSYGDPLGYSLAAISIF
SSLIPLGVLGVFIHQKKTPIVRANNYSLSCLLLLSLFLCFLCSLGFIGYPQPEKCLLRQVAFG
MVFALCISCVLAKTITVVIAFNATKPGSRLRKWTGVKVSYCVIGFCAFLQLVVCVLWLSLS
SPFPELDTDTKPGVIIVNCNEGSPTAFWCMLGYLGLLASISFIVAFLARRLPDSFNEAKLITF
SMLAFLSVWVSFIPAYLSARGMYTVAMEVFAILSSSWAVVGCIFVPKCYIVLFRPNMNSRE
NLMGKGRSQK*
>jgi|Xentr4|394806|e_gw1.675.5.1
MPRGHGIPPVPLSAVLFCTLAVTVASKISCNLPSEDIGSYISRPGDIIVGGIIPVHFNIYNKAN
FTSQPRELQCRTVVDEYYLTLQALIFTVERINADPELLPNITLGFHIYDTCRTLQRAAQGTL
LMLSGGKDITPNYQCHIVAPYAGVIGDSTSRRSILMAQILGLYRYPQYVSYYATSPILSDRN
QFPSFLRTIPSDEFQMKGLAQLVSYFGWSWLGILATDDEYGQFGLQIIKQEIAKTGACVAFA
EYILTSKPDRNAPHLIQVIQMSTARVVVVISTSTDFAIVLEEMLKQNVTGYTWIGSEGWSTS
DLLSNKRFQGILTGTAGFAIHQRQMPQFPQHLSSLSPLNDPYDTFMREYWEQTFSCRWPDS
DTSAANGSLKACTGNENLASLIAGIDYRTGHTLYVAVYAIAWGLHSMINCSPGYGPFHAGS
CANISSFYPWQLLHYVRNVNFMTSDGARVFFDEKGNPPAIYDIVNWRLSAKGSLEKVTVG
SYDSSSPDGKTLKLNVSNIKWITQTQVLKGLMVIVPGKPLCCYECARCPQGQISNQTDAA
ECHPCSWDTWPNLQQDRCLPRPTEFLTYTEPLGYSLAAITSFSTLTPITILGVFIYYRKTPIV
RANNYSLSCLLLLSLFLCFLCSLGFIGYPQPEKCLLRQVAFGMVFALCISCVLAKTITVVIAF
NATKPGSRLRKWTGAKVPYCVIVFCALIQLFLCVMWLILSPPFHEYDTDTKPGVLIVNCN
EGSTTAFWCMLGYLGLLASISFIVAFLARRLPDSFNEAKLITFSMLAFLSVWVSFIPAYLSA
RGMYTVAMEVFAILSSSWAVVGCIFVPKCFIVLFRPNLNSREHLLGKK*
>jgi|Xentr4|394815|e_gw1.675.85.1
MQWVPITLYTIAIAVSPCSESERGCSLSSLSTDGHARRPGDIIIGATFLIHVNRIQSKALFTSQ
PPELQCQAFGLDYYQSMRALLFAVEEINANPRFLPNITLGFQIFDTCTAVRRAAQGTLWML
SGGQEITSNYNCFPESHLAGIIGDSASVRAIIMAQILGLSRYPQISYLATSPILSNRDLFPSFFR
TIPSDELQAIGLAQLVSHFGWTWVGLLAVDTDYGQFGIQLVKQEIVKAGACVAFSEDIVTG
KPNRNAPHIAQVIKASTAKVVIVISADYDLLIVLEELLTQNVTGRIWIASEAWATSTLLSDK
KLQAILVGTIGFAIHGGLISGFPEYFKSLSPFDHLYDAFIREFWEQAFSCKWLDDLADNTTF
RGCTGNEKMESLKMKIDIRITLNVYSAVYAFAWALKNLIDCKPGTGPFKSGSCANISSFHP
WHLLHYIKNVHFETKDKNVIFFDAKGNPPAIYDIVNWRLSATGAMEQVVIGSYSSVAGGE
KTLTINNSAITWIYGETQVPLSKCSRSCPVGFMKVALPGKPSCCYDCARCHQGEISNQTDA
VVCHQCSPETWPNLQQDQCIPRVIDFLAYEDLLGLSMATMSVSSSAVPLGILSIFIVYQTTPI
VRANNYSLSCLLLLSLFLCFLCSLGFIGYPQPEKCLLRQVAFGMVFALCISCVLAKTITVVI
AFNATKPGSRLRKWTGVKVSKSVVMFCTCIQFSICVLWVSFSPPFPEQDTKTQPGVIIYSC
NEGSPFAFWIMLGYLGLLASISFIVAFLARRLPDSFNEAKLITFSMLAFLSVWVSFIPAYLSA
RGMYTVAMEVFAILSSSWAVVGCIFVPKCFIVLFRPNMNSREHLMGKGQR*
>jgi|Xentr4|394823|e_gw1.675.69.1
MILEAAHKRYYYVIVLLCTIAIEVSPYREPIVGCSLPNEDIAGYMSRPGDIVIGGTFPVHFNI
IYRDLHFTSKPGGPQCQMISSEFYLSLRALIFAIEEINADPELLPNVTLGFHIFDTCKTLRRA
AQGTLLMLSGGKEITPNYHCYKGPPLAGVIGDPASRRSILMAQILGLYRYPQVSYLATSPIL
SDRNLFPSFFRTVPSDEFQMRGLAQLISHFGWSWFGLLANDDDYGQFGLQIVKQEIMKAG
ACVAFAENILTSKPDRNAPYLARVIKTSTAKVVVVITSDSDFLIMVEELLRQNVTGNTWIAS
DAWATSTLLSNKRFNGILSGTVGFAIYHGQMPQFNEYLNSLSPSQDLHDSFLKELWEQSFS
CRWLDQENVHALAFNGTLRVCTGEEKLESLMMEAEHRVSLHVYTAVYAIAEALHNVLYF

RFPLDCNMDPSCHTYLLHYVKHVNFQTKDGRRVFFDAKGNPPAMYDIVNWHVSANGLL
KKVTVGSFDGAIFKVYSAEIIWNNHTQVPLSKCSPSCPSGFRKVIVPGKPLCCYECARCPQ
GQFSNETDAAECHPCSWDTWPNLQQDRCLPRPIEFLSYGDPLGYSLAVISSFSSLTPITILGV
FIRYNKTPIVRANNYSLSCLLLLSLFLCFLCSLGFIGYPQPEKCLLRQVAFGMVFALCISCVL
AKTITVVIAFNATKPSSRLRKWTGVKVSYCVIGFCIFVQITDCALWLIFSPPFHELDTDTQP
GVIIANCNEGSPTAFWCMLGYLGLLATISFIVAFLARRLPDSFNEAKLITFSMLAFLSVWVS
FIPAYLSARGMYTVAMEVFAILSSSWALVVCIFMPKCYIVLFRPNLNSREHLMGKGRGQK*
>jgi|Xentr4|394827|e_gw1.675.132.1
FSFRSYRWVLAMMFAIVELNGTPQSLPNVTLGVGVLDTCSSASRALKGAAWLLSGAPGG
TLTHHCQRSSSQLAAVLADSGADSVLSVANVLGLYRYPQVSYFIPPPELSNQVLFPTSLSVA
PALTAQAKGLIRLLQFFDWSWVGVLVQEGSVSTVAQTFLRELEGSGICVAFKENVPSVSAD
VSRIVSVVRESTATVVVVFSLEAYLNPVLLDLALKGDSRPRIWLTTEGWSTSPGLVAPWLS
QFLRGSLGLVLRNGAAPGFKEFVFGLQPSTLHKDPFLIEFWEEAFGCRWDASADTSSFTPN
LTVSSISPSTTPTFSSTTSSSPHQVLCTGKEKPGSLQLFSDIGDLRVTYNVYKAVRMVSGAL
RDMSKCRDGEGPLPGGKCVNMSDFKPWQFHHYLRRVHTKGNIGDDLYFDVLGNAPAVY
DIINWQSAPSGSTGWVTVGSYESGAPPGQDMLINDNFIHWAEGIKKWPSSVCSEHCTPGF
RKAIRQGQPNCCFDCVPCSSGEVSNQTGKC*CYLCSDDEWPNSARNHCLSRAIELLSLSEP
FGLSLGTTAVIGSLLPAAVLVIFIRNRDTPLVRANNRGLSFLLLAALLLSFLCPLLLLFPPGTF
LCFIRQAAFGILFALCISCLLAKTVIVVLAFRANQLGKGLMLIMGPRSPILIALSCTVFQLTL
CLSWIIQSPPFPEQDIKSQVGTITIKCNEGLGFWFMLGYLGLLSTICFVAAFLARKLPGAFNE
ATHITFSMVVFLCVWVSFVPAYLSTHGKLAVATEIFAILSSSAGLLFCIFSPKCYIILLKPQLN
TRPLVSGHHRRRPGSH*
>jgi|Xentr4|394851|e_gw1.675.221.1
MSRPGDIIIGGTFPVHLDRVYVDIDFTNKPPELQCQMYAIEYYHSMQALIFAVDEINSDPDL
LPNITLGYHIFDTCITVRRSAQGALWMLSGGQEITLNYHCYQEAPLAGIIGDCGSTRSILMA
EMLGLYRYPQISYFATSPVLNNRDQFPSFFRTIPSDEFQMRGLAELVSYFDWTWVGLLAND
DDYGQFGMQMVKQEVLISGGCFAFIENILTGLPNRNAPHLAQVIRESSAKVVLVISSDSDF
VIVLEELIRQNATGTIWVGSEGWANSALLSDERFQQVLVGAVGFSIHGGQLPKFTKYLKSL
HPFKDSHDAFVREFWEEAFLCKWLTQKNATEFSNNGTIHACTGKEITESLVVEEVHRGSL
NVYTAVYAMAQALHNLHQCTPGTGPFLNGSCANISSLRPWQLLHYVKNVNFKTKDGGQ
VSFDATGNPPAVYDVVNWRASVGGKMDQIVVGSYDLNSANGKTLNVERDSIIWSNRDTQ
APISKCSPSCLSGFRKVILPGKPLCCYECARCPQGQISNITDAAECNPCSWDTWPNLQQDR
CLPRPTEFLSYGDPLGYSLAAISIFSSLIPLIILAVFIHQKKTPIVRANNYSLSCLLLLSLFLCFL
CSLGFIGYPQPEKCLLRQVAFGMVFALCISCVLAKTITVVIAFNATKPGSRLRKWTGIRVSY
CLILLCALNQLTICGIWLIFFPPFYELDTGTKPGIIILNCNEGSPFAFWCMLGYLGLLASISFIV
AFLARRLPDSFNEAKLITFSMLAFLSVWVSFIPAYLSARGMYTVAMEVFAILSSSWAVVGCI
FVPKCYIVLFRPNMNSREHLMVKGRGHK*
>jgi|Xentr4|394853|e_gw1.675.213.1
MGCSLPSEGITGHLSRPGDIVIGGTFMVHLERVYSDLSFTYKPPELQCQMFALQYYQSMR
ALIFAVEEINANPKLLPNITLGFQVIDSCNNLRPTAQGTLSMLSGGKEVIPNFHCRKEATLA
GIIGDTGSTRSILMAHILGLYRYPQISYFATNPKLSDRNLFPSFFRTIPSDEYQMRGLAQLVR
YFDWSWVGLLANDNDYGQYGLQMVKQEIMNGGACVAFTENILTARSNRNAPHLVRVIK
ESTAKVVVVITTDSDFLIVAEELLRQNVTDNIWVASEAWSTTDLLSSQRFKPILLGTIGFAIH
RGQMPKFSRYLSSLRPTDYLYDWLMRELWELVFSCKWSSHENVTVDKACTGNEKLESILT

EEDYRVSLNVYTAMYAIAWALQSLHNCTTGYGPFYGGGCANITSFHPWQLLHYIKNVNF
KTEDGSPIFFDAKGNPPAVYDIVNWRQSRQGTLEQVTIGRYDLSAPNGKILNVDSADVIWI
NGNTQVPASKCSPSCSSGFRKVIVPGKPLCCYECARCPQGQISNQADAAECQPCSWDTWP
NPQHTRCLPRPTEFLSYEEPLGYSLVVIAILSSLIPLVILGVFIRYNKTPIVRANNYSLSCLLL
LSLFLCFLCSLGFIGYPQPEKCLLRQVAFGMVFALCISCVLAKTITVVIAFNATKPGSRLRK
WTGVKVSYCLILFCVFIQLFLCIMWLIFSPPFHELHTDTKPEVIVVCCNEGSPTAFWCMLG
YLGLLASISFIVAFLARRLPDSFNEAKLITFSMLAFLSVWVSFIPAYLSARGMYTVAMEVFAI
LSSSWAVVGCIFVPKCYIILFKPNRNSRELLMGKGRNQK*
>jgi|Xentr4|394855|e_gw1.675.88.1
MGVCRAPKSDITGLYREGNIVIGGVFPVHVYRVYQQLSYRSPPPTVTCSTFVPLTFQWLQT
LLLAVQEISASPYLLPNIPLGVHLFDSCASPGRALEGISWLLSGGASTPIPNYHWRSSMSSL
VGVVGDSSSASSLAMARLLGLYRYPQISYFSTSSLLSDRTQFPSFFRTVPSDIFQSKGLAQL
VSYFGWTWIGMMGEDTDYGQEGIRTTTTEILRSGACVAFTGYILTSRPDRNAPYLSRVIIES
NVSVIVVFSSGSNFVPVVEELLKHNVTGKTWVASESWSTSALVSKEKYWRVLKGTIGFAL
HSGHISGLKQFMNSLDPKKTPDDIFLKEFWEVNFRCKWQIQEVGWTNSTNVCTGQESNG
PLFTDGIQRVTYSVYIAVYALAWALHGILMSRQRACLQIHKTCNESLSFQPWQVMDSMRT
VHFEAEDGRNVYFDPNGNLPAVYDIVNWQMDPAGNIHHVKVGNYDGSTEDENTFSVNTS
SIQWNTEGSQVPLSFCSRSCPIGFRKAAILGKPICCFQCVPCPSGEISNQTDSVDCFRCPWD
QWPNNQKDQCILKPVEFLSCDDPVGVTLITSSVISSIIPVALLGLFINFKTTPIVRANNYLLS
CVLLVSLSLCFLCSLGFIGYPQPEKCLLRQVAFGMVFALCISCVLAKTITVVIAFKATKPGS
KLRKWTGTKVTYLVTGLGVLLQLGICIIWLSTFPSFPENDIQTQPGVIIAHCNEGFPTAFWC
MLGYLGLLATISFVLAFLVRRLPDSFNEAKYITFSMLAFLSVWVSFIPAYLSARGKYTDAM
EVFAILSSTWALVICIFLPKCFIIIFQPELNNKDSLMGRGKK*
>jgi|Xentr4|394872|e_gw1.675.83.1
MGVSKSFPFSCNLDSESITGYLSRPGDIIIGGTFMIHLERVFSDLSFTSKPTDLQCQMFSTEY
YQTMQALIFAVEEINSDPELLPQYTLGFQVFDSCFTLRKAAHGTLSLLSGGRKITPNYHCH
KGAPVAGIIGDSGSSQSILMAQILGLYRYPQISYFATSPTLSDRNLFPSFFRTIPSDEFQMRGL
AQLVSHFNWSWVGLLASDTDYGQFGLQMIKQEIVKGGACVAFAENIRTNHPDRNAPHLV
QVIRKSTAKVVIVISSDFDFVIVADELLEQNVSGITWIASEAWATSELLSKEPYKGLLSGTVG
VAIRRGQMTRFTKYINSLSPSENVNDLFIKEFWEEIFNCKWLNRDNVTIPLGNETRKACTG
SEKLEDLLNEEYHRVSLNVYTAVYAFAWALHSWLNCTSGIGPFYLGNCANLLHYIKKVYF
KTQDGSQIFFDAKGNPPAVYDIVNWCVNAKGALGQVIVGRYDMNAPETFNVDSDGVIWA
NGSTKVPLSKCSPSCAAGFRKVMVPSKALCCYECARCPQGHISNQTDAVECNPCSWDTW
PNQHRAKCIARTTEFLSYEDDLGLILAAVSILSSLVPLVFWGVFVHYKKTPIVRANNYSLSC
LLLLSLFLCFLCSLGFIGYPQPEKCLLRQVAFGMVFALCISCVLAKTITVVIAFNATKPGSRL
RKWTGVRLPYFVIILCTFIQFSLCMIWLIFYPPVPELNTETKPGIIIVSCNEGSPTAFWCMLG
YLGLLASISFIVAFLARRLPDSFNEAKLITFSMLAFLSVWVSFIPAYLSARGMYTVAMEVFAI
LSSSWAVVGCIFVPKCLIVLFRPNMNSREHLMGKRNIYNFS*
>jgi|Xentr4|394910|e_gw1.675.94.1
YKSMRALVFAVEEVNANPEILPNITLGYHIFDTCNTLRRAAQGTLWILSGGQEIIPNYYCAG
GDPLAGVIGECGSTPSIIMAQILGLYRYPQISYFATNPILSDRSQFPSFFRTISSDDIQMRGLA
QLISYFGWSWVGLLANDDDYGQSGLQMAKQEIINAGACVAFAENILIGKEDKNAPYLAR
VIKKSNAKVVLVIASDSDFLIVVTELLRQNVTGNIWVATEGWATSTLLSEEKFQRVLEGTIG
FAIHGAQMQKFTEYLSNLLPSQSVNDPFIWEYWEKTFLCKWSYQGNLEWAGNATTRVCT

GNETLDGALTTEDHRVSFNVYTSFYAIAWALHSLLYCRPGIDPFHQYNCANIRSFHPWHLL
HYVQNVNFKTKDGSNMFFNAKGNPPAVYDIVNWRLGAKGRLEQVVVGRYDLTSPDQET
LNVDKKAITWANEETQVPLSKCSPSCPSGFRKVIVPGKPTCCYECARCPHGHISSQTDAVE
CHPCSWDTWPNLQQDRCLPRTREFLSYEEPLGYSLAAISILSSLIALVILGLFIQYKRTPIVR
ANNYSLSCLLLLSLFLCFLCSLGFIGYPQPEKCLLRQVAFGLVFALCISCVLAKTIIVVIAFKA
TKPGSRLRKWTRVRVSYWIIGFGVFIQSFLCVLWLLFSPPFPETDTDTKPGVIILNCNEGSPI
SFWCMLGYLGLLAAISFIVAFLARRLPDSFNEAKFIIFSMLAFLSVWVSFIPAYLSARGMYT
VAMEVFAILSSTWAVLVCIFMPKCYIVLFRPNLNSRKYLMSNGRSKK*
>jgi|Xentr4|394912|e_gw1.675.86.1
MVGDSISRPGHILIGGTFRVHYDRVYTAIDFTSSPGEFQCQMVSIEFYQTMQALLYAVDEIN
TDAELLPNITLGFQVLDTCNTLRRTAQGVLLMLSGGLGMTPNFHCYKGRLLAGVIGDSAS
TRSILMAQILGLYRYPQISYFSTNPILSNRDLFPSFFRTIPSDDFQMRGLAELISYFGWSWLGI
LANDDDYGQSGLQMVKREIMKGGACVAFVENILTTTADKNAPHIVKVLRESTAKVVVVIS
SDSHFVFVVEELLRQNVGGNVWIASEAWATSDLLTKERFSRVLLGTIGFAIYHGQMPDFTK
YFNSLNPSKDLHDPFIREIWEQTFSCKWPSQESLATWMENATTKVCTMKETLENILPEEQR
LSLNVYTAAYALAWALHNMLHCKPGTGPFHNGTCADISSFQPWQLFHYLKNVTFKTQDG
RQIFFDAKGNPPAIYDIVNWRVSARGTLEQVAVGSYDLNRPDRTTFQVDTGEMIWIDNRTQ
VPLSNCSPSCSLGFRKVIVPGKPVCCHECARCPQGHVSNQTDAVECQPCSWDTWPNLQQ
DRCLPRTIEFLSYEEPLGYSLAAITIFSSIIPLVILGLFIQYKETPIVRANNYSLSCLLLLSLFLC
FLCSLGFIGYPQPEKCLLRQVAFGMVFALCISCVLAKTITVVIAFNATKPGSRLRKWTGVK
VPYCVIICCVFIQLCVCVLWLTLSSPFPEQDTRTKPGLIIVNCNEGSPPAFWCMLGYLGLLA
SISFIVAFLARRLPDSFNEAKLITFSMLAFLSVWVSFIPAYLSARGMYTVAMEVFAILSSSWA
LVGCIFVPKCYIVLLRPDMNSREHLMGKRKDQK*
>jgi|Xentr4|394920|e_gw1.675.25.1
IPISKCNPSCYPGFRKVIIPGKPPCCYECARCPQGQISNQTDTVECHPCSWDTWPNLQQDR
CLPRPTEFLSYGDPLGFSLACTAIFSSLIPLVILRVFVCYKETPIVRANNYSLSCLLLLSLFLC
FLCSLGFIGYPQPEKCLLHQVAFGMVFALCISCVLAKTITVVIAFNATKPGSRLRQWTGVK
VSYSVILTCVSIQLFVCAIWLVILPPFLELDVDTKPGVMIVNCNEGSPTAFWCMLGYLGLL
ASISFIVAFLARRLPDSFNEAKLITFSMLAFLSVWVSFIPAYLSARGMYTVAMEVFAILSSSW
AVVGCIFVPKCFIILLRPNMNSREHLMGKGRGQK*
>jgi|Xentr4|394928|e_gw1.675.81.1
MENSSGYLSQPGDIIIGGTFPVHLDRVYHNNNFTETPPEIQCQMFSLEHFQSMEALIFAVEEI
NVDPELLPHITLGFQIFDTCIALRQAVYGALWMLSGGQGISPNYQCHKEAPLAGIIGDSGST
QSILLAQLLGLYRYPQISYLATSPILSDRHLFPSFYRTIPSDEFQMRGLAQLVSHFGWTWFG
LLASDDDYGQLGTQLVKQEITKAGACVAFTENIITGLHHRNAPRIAEAIKKSTAKVIVVIAT
DHNFVIVVEELLRQNMTGIIWVASEGWATAVVLSKENFRLVLEGTIGFATHGRQISKFTKYL
QSLHPSKELHNVFLRQFWEQAFDCKWPELAKGATNATDQTCTGNETLEGLLMEKDYRVA
HKVYTAVYAIAWALQNLLYCKLGTGPFSNGSCSNISSFHPWHLLHYIKHIHFKTKDGSQVF
FDGKGNPPAIYDIVNWRVSANGALEQVVVGSYDLNAVDGKTMKVNSTAITWNSNGTQIP
VSKCSPSCSSGFRKVIIPGKPPCCYDCNPCPQGHISNQTDAVECHPCSWDTWPNLQQDRCL
PRPTEFLSYGDPLGYSLTAFSVFSSLIPLAILGVFICYKKTPIVRANNYSLSCLLLLSLFLCFL
CSLGFIGYPQPEKCLLRQVAFGMVFALCISCVLAKTITVVIAFNATKPGSRLRKWTGVRVS
YWITVFGTLIQLFVCVMWLIFSPPFPELDTITSPGVIIVTCNEGSPTAFWCMLGYLGLLASIS
FIVAFLARRLPDSFNEAKLITFSMLAFLSVWVSFIPAYLSARGMYTVAMEVFAILSSSWAVV

GCIFVPKCLIVMFRPNMNSRELLMGKGQNLKN*
>jgi|Xentr4|394932|e_gw1.675.65.1
MAICAGSSSGISCTQPAENITGLLSRPGDIVIGGTFMVHFDRVSSGLDFTSQPHELQCGMFII
EYFQNMHALIFAVEEINSDPELLPNITLGYQVFDTCFTSREAARGTLLMLSGGNEITPNYHC
HKGAPLAGIIGDSWSTGSIIMAQILGLSRYPQISYFATSPVLNDRDLFPSFFRTISNDEYQMR
GLAQLIFYFGWTWVGLLADDDDYGQHGLQMVKEELNNGGACVAFTEYILTGQADRDAP
RLVQVLQKSTAKIVIVISSNFVVVVEELLRQNVTGKIFMATEAWATLDLLSKPRYQGILTGT
IGFAVHNGLMSNFSKYLRSLRPWKYLHDQFVLEFWEQAFACKWPTDETLAWTGHVTIKA
CTGNENPESVEAEDYQRASLNVYTAVYAIAWALQNMVNCRPGTGPFSGGSCANISSFQAW
QLLHYIKNVNFKTKDGSQIFFDAKGNPPAIYDVVNWHLGAEGAAEHITVGTYDLRAPDG
NTLKVDSKRIVWINGDTQVPVSKCSPSCSPGFRKVIVSGKPLCCYECARCPQGQISNQTDA
AECHPCSWDTWPNLQQDRCLPRPTEFLSFGDPLGFSLTGTAIFSSLIPLLILRVFTWFKKTPI
VKANNYSLSCLLLLSLFLCFLCSLGFIGYPQLQQCLLRQVAFGLVFALCISCVLAKTITVVIA
FNATKPGSRLRKWTGVKVSYPFILICTLIQLCVCVLWLFVIPPFPELDTDTKPGVIIVNCNEG
SPTAFWCMLGYLGLLASISFIVAFLARRLPDSFNEAKLITFSMLAFLSVWVSFIPAYLSARG
MYTVAMEVFAILSSSWAVLGCIFVPKCYIVLFRPNMNTKGHLLGKSKSQK*
>jgi|Xentr4|394954|e_gw1.675.89.1
MEVFYIFVSFILSKVWILCTLVIEVCASFHSKISCSLPGESIRGYLSRPGDIIIGGTFPIHLDRL
YYGFNFTSKPLELQCQTSQFSPEYYQTMQAFVFAVDQINADLELLPNITLGFQILDTCISIRR
AMLGVLWMLSERQETTPNFRCQEGAPLGGIVGESGSTQSILMAQILGLYRYPQISYFATSSI
LSNRNLFPSFFRTIPSDELQMKGLAQLVSHFGWSWVGVVVSNDDYGQYGIQVVKQELSSS
GVCIAFIEKILTGETNKNAPDIVQVIKRSTAKVVVAIASDPDFVMVVEELVMQNLTGIIWVA
SEGWATSTLLSDKRFQKVLVGTIGFAIHGGKVPGFTTYLSGLHPLKDLNDSFLREFWEKTF
SCKWNSGQRIGNSSIQGCTGDEQLEADAFGENHRVSLNVYTAVYAIAWALQHLLYDKLGT
DLFHHGNCPSMSSFQPWQLFYYIKNVNFKTKDGRRLFFNSKGNPPAIYDIVNWQVNAKG
ALEQVVVGSYDLNTTDGNPLKVDSATITWANKEDGKKIPLSKCSPSCPSGYRMVAVPGKP
PCCYECAPCPQGQISNQTDAVECLPCSWDTWPNLHQDRCLPRPTEFLSYRDPLGYSLAATA
VFSSLITLVILGVFYFFKKTPIVRANNYSLSCLLLLSLFLCFLCSLGFIGYPKPAQCLLRQVAF
GMVFALCISCVLAKTITVVIAFNATKPGSRLRKWSGIKVSYCVIVFCVIIQIGICTVWLLFYP
PFSELDNDNQQGVIIINCNEGSPTAFWCMLGYLGLLASISFIVAFLARRLPDSFNEAKLITFS
MLAFLSVWVSFIPAYLSARGMYTVAMEVFAILSSSWAMVVCIFVPKCFIVIFRPNKNAREH
LMSKGRSRK*
>jgi|Xentr4|394968|e_gw1.675.84.1
MLLCNRRAYHRLSWLLFSTSWIFSILAIEVCECLLSSCSLPSRSITGYMSQPGDILIGGTFMI
HLERVFFNDPDFTVKPSDLQCQLFATEYYQSLQALIFAVEEINVDPELLPNVTLGYQVYDT
CFTLQKAAEVTLSVLSGGKEIAPNYHCLKRAPLAGIIGDSWTTTSILMAQILGLYRYPQISY
FATNPILSDRNLFPSFFRTIPSDEFQMRGLAQLITHFGWTWVGLLANDNDYGQFGLQMVK
QEIINGGGCVAFAENILTGQPNRNAPHLANIVKRSTAKVVVMITSDFDFVAVMEEMLRQNV
TGITWIASEAMSTSNFLSHKSLKSIVLGTIGFAIHRGKMPKFTGYLNSLSPSKNLHDSFISEY
WEQIFSCKWPNQEDLNTGMKNLTVKACTGSEKLGSRQTEEYHRVSLNVYTAVYAFAWAL
HHLQNCTPGTGPFQHGNCANISSFHPWQLLHYLKNVNFKTKDGSQVFFDAKGNPPPIYDI
VNWCASDNGILEPATVGSYDLNAPDGKTLTIDTVGITWNSNETQVPVSKCSPSCAPGSRK
VIEPGKPTCCYECARCPQGKISNQTDAVECHPCSWDTWPNLRRTECIPRMAEFLSYETPLG
FSLAAIASFSSLVPLIIWGVFMYYKETPIVRANNYSVSCLLLLSLFLCFLCSLGFIGYPQPEK

CLLRQVAFGMVFALCISCVLAKTITVVIAFNATKPGSRLRKWTGVKVPYFLILCCALIQLCI
CVIWLIFFQPFHELDIDTKPGIIIVNCNEGSPTAFWCMLGYLGLLATMSFIIAFLARRLPDSF
NEAKLITFSMLAFLSVWVSFIPAYLSARGMYTVAMEVFAILSSSWALMVCIFLPKCFIILFRP
NMNSREHLMGKGQQCK*
>jgi|Xentr4|394970|e_gw1.675.76.1
MLKRIHWSVYFSSWVLCLVAVRVCNALDSNLGCNLPIKNSSGYLSQPGDIIIGGTFPVHLD
RVYHNNNFTETPPEIQCQMFGVGQYQNMQALIFAVEEINADPDLLPNITLGFHIFDTCTVL
RRAAEGTLWMLSGGKGITPNYRCHKTAPLAGIVGDSGSSQSILMAQILGLSQCPQLSYLAT
SPLLGSRDLFPSFFRTIPSDEFQIRGLVKLIIQMGWSWLGILASDDDYGQFGVQIAKQEISTT
RACVAFTENILTGQPNRNAPHLVQVIKESSAKVVLVIATGYNFVIVVEELLRQNMAGITWI
ASQGWATSLLLSENRFRGVLTGAVGFAIHSGKMVQFTNYLNSLRPSMQHNDSFIAELWEK
TFSCKWLSRPLEGTGPTPTKPCTGNEKPESLLPSGKDYRESLNVYTAVYAIAWALHNLLQ
WTPGNGPFPHVSPPTISSFQPWQLLHYVKYVDFKTQDGSHVVFDTKGNPPAIYDIVNWQV
GATGVLGQVTVGSYDLTNLDLNLLNIDSAKVVWSNGSNQVPVSKCSPNCPAGFRKVIIQG
KPPCCYECGRCPPGQISNQTDAVECHPCSWDTWPNLQQDRCLPRPTEFLSYGDPLGYSLA
AISIFSSLIPLGVLGVFIHQKKTPIVRANNYSLSCLLLLSLFLCFLCSLGFIGYPQPEKCLLRQ
VAFGMVFALCISCVLAKTITVVIAFNATKPGSRLRKWTGVKVSYCVIMLCALIQITDCALW
LIFSPPFHELDVDTKPGVIIVNCNEGSPTAFWCMLGYLGLLASISFIVAFLARRLPDSFNEAK
LITFSMLAFLSVWVSFIPAYLSARGMYTVAMEVFAILSSSWAVVGCIFVPKCYIILFRPNMN
SREHLMGKDKNKK*
>jgi|Xentr4|394977|e_gw1.675.15.1
MVNDTTQVCTGEEQLDSLPFEEYHRVSLNVYTAVYAIAWALHNLLYCAPGSKPFHNGNCF
NISSFHPWQVPISKCSPSCFPGFRKVIVPGKPPCCHDCAPCPQGHISNQSDVVECQPCSWDS
WPNLQHTKCLPRPTEFLSYEEPLGYSLAAIAIVSSLIPLNIFGVFIHYRKTPIVRANNYIVSC
LILLSLFLCFLCSLGFIGYPQPEKCLLRQVAFGMVFALCISCVLAKTITVVIAFNATKPGSRL
RKWTGVKVPYCLITLCVSIQFFLCVMWLIYACPFPELDINTKPGIILVNCNEHSPIAFWCML
GYLGLLASISFIVAFLARRLPDSFNEAKLITFSMLAFLSVWVSFIPAYLSARGMYTVAMEVF
AILSSSWAVVGCIFVPKCFIILLRPNMNSREYLMRKDKGHNN*
>jgi|Xentr4|390423|e_gw1.616.174.1
MLADSPIISIIILSLNVMCVGPCRSETPTNKPACRLQIIKSAEEYEYIQEGDIMIGGVLTVSMF
RPVDYFRGLTCIFPSAPNYKYLVDFLYVIEIINRNPDIFPNTTLGYHIYDSCGDPRKAVKSVL
QILSGTREPVPNYSCVGKRNIAGFIGDLTSETTIPIAQILSVFGYTQISYGATDPVLSDRFSFP
YFFRTTGSDDSYYFVISQIAKYFQWNWVGIITVDDDRGERDHQLLKYYLSSENICIEFTLKI
THIIHGNIRYREIIQKSSTNIIIFCGAVTLQILAEYNFLYRLVSEKTFIFTSNWLYYNHVLDFSL
KVFHGSLLLMQNKEEDYSSTSQYTQFSNQFHPSRYPDDKLLENIWMCYHFCLSKNKKKN
EIFKKEFKMLLHNCSGQESLSKIGMYNNGFHSLNMMYAVDMLASALHYRHDALRMETS
GRNRDLYNFRYKVHPYLKKINFRYKANHVYYLNEKGEFVSRYMIINIYGKSAKQSAWKQ
CGTYTPWVPEHLKLNITSGEIQWKTKDNKVPRAQCSDTCPTGFRKTQKPRAQSCCYDCV
LCSEGEISNTSGINDHKWRREVKMRTITKFGHTRTDIIVRNVVFDDFWTVYGLRIIVPIIFIPS
IRKSQNFCIPAIGNGGKTFLTLILLGMTLEASHRAQWHFAAPTRPKETVSSLLAKTIMVCVA
FKATKPGSSWRKWLGVKLSNSVVLFCSSIQIIICMTWLAISPPFQELDIHTSPGTIIIQCNEGS
AIGFYSVIGYMGLLAAVSFVLAFLARSLPDSFNEAKYITFSMLLFCSVWITMIPAYLSTKGK
NTVCVEIFAILTSSAGLLASIFLPKCYIIMLSPELNTKSHLLGNQA*
>jgi|Xentr4|391468|e_gw1.631.134.1

MLGGSAVPQYLKLLIYLTALPMAPCRYVDQSNNSACHLNLKVKAHEEYEYVQEGDILIGG
VLTVGVRGTFIEQTLLACDKPSAQKYRYLVDFAFRVKEINENPALLPNITLGYHIHDSCGDP
RRALMSLLKIISGTRKPVPNYSCRRTGKMAGFIGDLLSVPTESIAHILSALGYSQISYGATDP
ALSDRTTFPYFFRTLQSDEEEYIAVCKLMKFFGWNWVGIIYMNNKSGERDYQLLTKYLSR
EGICIEFARNISKLSEHSQFIKKTTTRVVIICGDPSYEYNIQSYHLHDIIIEKTCIFLSKWLNHY
ESLDLASLLTGTVLFMQNRLDNQYDARFREFSDTFHPSKYPHDDLLHDMWLWKQECLVK
DDDDINYYSLLDVPPAKCTGKEKLTDIPEYLNAYHSASLIQAVDMMAMALQDMHNFHSK
QTHGKGRWVGNYNYQLHRYLRNAVYAIDGSPVSSFNEKGEFVHQYDINNPFFDSEGKFS
WKTVGRYVPWAPMEQRLILNPDKIIWNTPDHEVPRAQCTDNCLPGLRKVIEPGKLICCYS
CAPCPEGEISNKTGITACLISVTLRANNRSLSFLLLVSIKLSFLSVFLFLGRPVDITCMLRIITF
GITFSIAVSSLLAKTIMVCVAFKATKPGSSWRKWLGVKLSNSVVLFCSSIQIIICMTWLAISP
PFQELDIHTSPGTIIIQCNEGSAIGFYSVIGYMGLLAAVSFVLAFLARSLPDSFNEAKYITFS
MLLFCSVWITMIPAYLSTKGKNTVCVEIFAILTSSAGLLASIFLPKCYTILYRPEMNTKCHFL
GNKT*
>jgi|Xentr4|391505|e_gw1.631.132.1
MLGVTAVCFSVKVLIYLILMCVGPCRSGDQPLSPACHLQIIKAPKEYEYIKEGDIMLGGVM
ASHFYMINSIVPWDNSSSFVCICPNEQAFRYLVDFRFAIEQINKDLAWSANLTLGYHLSDSC
GDARKAVRSVLQILSGTREPVPNYSCVGKRNIAGFIGDLTSETTVPIAQILSRLGYSQISYGA
TDPSLRDRHTFPYFFRTVQSDEANYFALSKLLRHFGWTWVGIITSNDMSGEEEHHNLAKY
LSREGVCIEFSIKIQIYKVKTYSFNQHESTIKGSSTSVIILSGTASVMFVAVLPYVLQEKTLILS
SNWGNNDIVVGSAIEIFNYSLVFVPRYHYDLGTPEMSRFLEDLHPSKFPDDELIEDIFLMFH
LCLSKDQNKNNLYQFTSQLTPHNCTGLERIKDIYYFSGKANSPRVHLAVDIMSRALHDMN
MLFIKKSIKPPYKYQLHHFLRKHQYRTQTGPTFSFDEHGEYISGLWIYNYIISANGDINKQL
FGEFSPWAPPDQQLKITSSLIQWKTSNNGIPRAQCSASCIPGFRKALRPGARSCCYDCVPCS
EGEISNITDSESCIRCPDMEWPNEEKIQCFAKIKEFLSYTDDVISIIFSSISCNEGSAIGFYSVIG
YMGLLAAVSFVLAFLARSLPDSFNEAKYITFSMLLFCSVWITMIPAYLSTKGKNTVCVEIFA
ILTSSAGLLASIFLPKCYTILLRPDVNMKTSLLRNK*
>jgi|Xentr4|393175|e_gw1.657.165.1
MYSKNQTLVSEIVLLGFQDLHNFKFPLFSLFLLIYIMSLWENLLIIALVASSRNLQSPMYFFL
RQLSQSDILESSNIVPTLLQTVIHDRATLSFGGCLTQFYFFSVTEAFECLLLTIMSYDRYVAIC
NPLHYNSIMTHRVCKKLAFLSWALVFAVELIPMNLIRTLQFCDQNTIDHFFCDFFPLIELSCS
DAFLLQIVGFFLSVPILFMPFILTTVSYICIAHAIRKIVSNIGRQKAFSTCSSHLIVVSIFYGTLI
SIYVVPPRRGSQTISKIFSLLYTVLIPLVNPVIYSLRNKHIKDAFKTCRLHSRL*
>jgi|Xentr4|393209|e_gw1.657.168.1
MSSCNQTKITQFLLLGFQGSQVLRSSLFYICLLIYIMALSGNFLIIALYLGSHHLRSPMYFFL
SNVSATDILLCTNVVPNLLSFLSTNGKPMSVSACIIQYFISGVFTGGECFLLTVMAYDRYLA
ICKPLHYVTIMTNRHCLHLVIWCWVEIVFLCVLATIMTSYSVGAIPWTTFTVIFPLFLKFHV
LMLLL*KKEWLY*LYLCLCYLYCLSLQHMSVSPSPSSGSPPTLGGRKPSPPVAPTSLWSPHF
MGYYLLNMQFHPKGSQ*M*IKSSPFCT*WLHHYSTPSYTVLGIKTSGKL*VNGCMLCSPM
QN
>jgi|Xentr4|393264|e_gw1.657.1.1
MDSIRHHLETDNYTICYTKYINQTKITNFILLGLNVPQSMKTLLFVMLLVLYAMTVSGNSLI
IALYLASQSLRSPMYFFLSHLAASDILLTTSVVPNLLCTVVDEGKVMSVPSCVTQFFASGFF
TVADSFLLTAMSFDRYLAICNALRYGSIMNIKLCLYLVTWSWLLSLLVCTTEVVLMSRSGF

CGCNVIDFIYCDFSPLLELSCEDTFIVEKVTMMLSILCVLAPFFFITSTYVSIVLSILRISSNTG
RQKAFSTCSSHLTVVCTYYGTLFSKYTVPPKGQSLSMNKAISLLYTVVTPLINPIVYSFRNQ
DIMLCLQKWISIVVTN*
>jgi|Xentr4|393278|e_gw1.657.9.1
MNTTSSCNQSKITEFLLVGFSAPRPLRVLLFSICLVIYIMALGANLMIIALYLGSHRLRSPMY
FFLSNLSATDILLSTTVGPNLLYTLLKRWDPCLSLLVLLSTLLLVSSQELSASYSQLWLMTD
TWPSANHCTMSP**PISIVSIWLFGVGWKLYCLCSLLLLQSHTQGSVGAIPWTTFIVILLLFF
RCHVQMCLFWKEQSQ*LLYQWL*YLYCSFSLHMAPSYSPSSGSPPTLGGRKPSPPVAPTSL
WSPCFMGY*LLIIQFHPRGLH*K*IK*SHFYTLWLHHYSTLLYTVLGTRKSRKL*VK*TLSD
*RREEFL*EN
>jgi|Xentr4|394433|e_gw1.672.56.1
MENQTVSEFTLLGLSYLPELKHQVFVMFLLIYFMTLCGNLLIVVLIFTDNHLHTPMHLFLG
HLAGVDFCYSQVTCPRMLLDFYSEKKTISYSSCLTQAFFFMCFARCECYLLAVMSYDRYV
AVCQPLHYLHIMSWKRCTQLITVVWALGSSYSLVEILLTLRLTFCGPSTIHNFFCDLPQLLT
LSCTDTSLNMLLLFVLGGIISFCAFFTTFLPYVYIFYTVHRIQTKNIKLKAFSTCTSHLTVVCI
FYGTLCFAYLHPHSRYFDADIVVAVVYAAILPLLNPIIYSLRNGEIQSALGRIKKKYV*
>jgi|Xentr4|395955|e_gw1.678.13.1
GNMFVVSLAVADLVVAIYPYPLVLTSIFHKGWNLGYLHCQISGFLMGISVIGSIFNIAGVAV
NRYCYICHSLKYDKLYSDKNSLFYVILIWVLTFIAIVPNLFVGSLQYDARIYSCTFTQSVSSA
YTIAVVFFHFILPITIVTFCYLRIWILVIQVRRRVKPDNKPKLKPHDFRNFVTMFVVFVLFAV
CWAPLNFIGLAVAVNPDTILPRIPEWLFVGSYYMAYFNSCLNAIIYGLLNQNFRREYKRIIISI
CTAKVFFQESSNDGVEKMKSKPSPMLTNNNLVKVDSV*
>jgi|Xentr4|396032|e_gw1.680.60.1
MDLNGVLVPGDILLGAVIPIHIDRNSSLATYQERPSQDICIMFRIELYQHFQALSFAVEEINRN
PTILPNRTLGFYVVYNTCADLQREMRGTFSILTGQSQAIPNYCCRKYPPLAAIIGLSKSSYSIL
MAHVLGLYKLPQISYYSTSSLLSDRTLFPSFFRTVPSDAFQFRGLAKLALRFNWTWVGLIA
INDDYGNEAIKTIKEEIIKGEACVAYTLYITYNPVSQTITKIVNIIKGSSANVVVAISVDVYLV
PLMEEMLKQNVTGKNFVASEAWSISTLLSLAKYSPIVSGTIGFAFQSSEIPRFQKYLNSINPL
NSPGLTWTQMLWEQTFGCTFSDPTNQTFKMNNSKNTCTGEESLEGIQNGYNDVSRLRAS
YNIYTAVYVIATALHGLRQCRFLGGPLFGDECSKIENFKPYQLLYFLKNVLVKLSNNMEVF
FDKDGNPPAVYDIVNWHPELQDTIKQVKVGSYDTRNFSGDLFTLNTSSLWWGRANQEIPL
SVCSQSCPLGFKKSIKREEPVCCFLCVPCAQGDISNQTDAIECWSCPWDMWPNSARDRCF
PKLIEFLSYEDPLGITLASTSVLSSMVPISIFYLFIQYKSTPIVRANNYSVSCILLVSLSFCFLC
ALAFIGYPQPEKCLLRQVEFGLVFTLCVSCILAKTVIVVFAFMATKPGSSLKKWTTPRVPY
MIITICTFIQLTLCVFWLTIFPPFLQFNIEAKPGIIVVECNENSQFAFWCMLGYLGILASVSFT
VAFLARRLPDNFNEAKLITFSMLAFLSVWVYFIPASLSAQGKYTVAMEIFAILTSSWALVFC
MFLPKCFIILFRPDMNLRENLMVKTGV*
>jgi|Xentr4|396034|e_gw1.680.97.1
MPALYSRDQACRLQVPELEGIFQAGEIMLGALLPLHIDKEYHPVTFKERPPNINCTTFNVE
NFQQLQALIFAVEEINNNPNILPNITLGFQAYDSCNVLQKDLEGTLQVLTGYNGIIPNYRCL
ENIPIAAIIGPYTSTHSILLAHILGLYRYPQVSHFSTSPILSDKRMFPSFFRTVPSDMFQSQGL
AKLVLHFGWTWIGLLALDNDYGQQGIQLVKQELIKAGACIAFSENILMSQPDRNARHIVN
VIKKSAATVIIVFSLPVDLVPILDEMLTQNITKKVFVASEAWSTSTLFSDGKFSDILTGTIGLA
LHSEMIPGFGDFLNKSLHSMTWLKLLWEEFFVCKFAGERNLTVTLDAPIKMCTGMEALQI

NDVSSLRATYNVYTAVRVVAKAFENMKNIEAPKVFSCGKCEYFKPWKVISHKLKVKVVL
SSGREIFFDENGDPPAVYDIVNWKLSPEGTIKQVKVGSYDIAALSGRVFSVNTSSIFWATGN
EQVPVSVCSQSCPPGFRKVAINGKPACCFQCIPCPQGEISNHTGKKPLCSLADAVDCVTCT
WDEWPNLQKTKCLQKNIEYLSYEDPLGASLAVTGIISSLVPVVILRLFIKYKSTPIVKANNY
SLSCLLLLCLPLCFLCSLTFIGYPQPEKCLLRQAAFGLVFTLCISCILAKTIMVVFAFMAHQP
GSKLRILVRPFVSYLIISICFLLQLILCLVSLVKLPSFTEYDTQTKPGIIIVQCNNGFSLAFWSM
LAYLGLLATISFIVAFLARRLPDSFNEAKFITFSMLAFLSVWVSFILASLNAQGKYTEAVEVF
AILSSSWALVACMFLPKCFIMLFRPALNSKKRLIKDNSHL*
>jgi|Xentr4|396035|e_gw1.680.52.1
MKVCPATICFVLSTTWTFNTFAIGSDSKFSCNLPNENNTGFLYRPGDIVIGGTFMVHVESVY
HNIDFTRKPQELQCLKFAPEYYQTMRALIFAVEEINSDIELLPNITLGFQIFDTCVTLRRAAQ
GALSMLSGGEEITPNYKCYKGGPLAGIVGDSGSSRSILMAQILGLYRYPQISYFATSPILSDR
KLFPSFFRTIPSDEFQMRGLALLVSHFGWTWVGLLASDNDYGQFGLQLVKEEITNAGACL
AFSEIILTGQYNRNAPHLAQIIKRSSANIVVVIAYDFVVVLEELLRQNVTGKIWLASESWST
SGLLSTKRYQGILLGTLGFAIHSGHMPMFNKYVNSLHPSTDLYDPFIKEFWEEAFTCKWPS
QENVVLGTENISKVCTGNEKLGSQLKEEYQRESLSVYTAVYAIAWALHHLANCTPGTGPF
HHGHCANISSFHPWQLLHYVKNVNFETKKGTQVSFDAKGNPPAVYDIVNWKMSTKGTLE
HVVVGNYDFNAPIGKSFNIDNSGITWINNNTQVPISKCSPSCHLGFRKVIVPGKPTCCYEC
ARCPQGQISNQTDAVKCLPCSWDTWPNLQQDRCLPRPTEFLSYGDPLGYSLAAASVFSSL
TPLFILGVFYFFKKTPIVRANNYSLSCLLLLSLFLCFLCSLGFIGYPQPEKCLLRQVAFGMVF
ALCISCVLAKTITVVIAFNATKPGSRLRKWTGVKVSSSIIIVCTSIDFCLCVIWVIYFPPFSEL
DTYTKPGFIIVSCNEGSPTAFWCMLGYLGFLASISFIVAFLARRLPDSFNEAKLITFSMLAFL
SVWVSFIPAYLSARGMYTVAMEVFAILSSSWAVVGCIFVSKCFIVLFRPNLNSREHLMGRG
RRKHIEG*
>jgi|Xentr4|396049|e_gw1.680.92.1
MIIFNIRPCNPFVIIQKVPLSKCSPSCSSGFRKVIVPGKPPCCYECARCPQGQISNQTDAAEC
HPCSWDTWPNLQQDRCLPRPTEFLAYEEPLGYSLAAIAIFSSLFPLIILGVFIYYKKTPVVR
ANNYSLSCLLLLSLFFCFLCSLGFIGYPQPEKCLLRQVAFGMVFALCISCVLAKTITVVIAFN
ATKPGSRLRKWTGVKVPYCIIGFCSSSQSCMSITRLIFSHPFAKLDTSTKPGVIIIYCTEGSPI
AFWCMLGYLGLLASISFIVAFLARRLPDSFNEAKLITFSMLAFLSVWVSFIPAYLSARGMYT
VAMEVFAILSSSWAVVGCIFVPKCYIVLFRPNMNTREQFKIKMKFPV*
>jgi|Xentr4|396052|e_gw1.680.84.1
MILFDLQLPYSFIWLSFFLAWILGTRVEDVFAYSDSKISCRLPSENITGYLSQEGDIIMGGTF
TVHSDRVHRDIDFTSKPQDIRCQRFAVEYYQTMRALIFAVEEINADSELLPNITLGFQIFDTC
NTLRPAAQGTFSMLSGKTEITPNYHCRKGGPLAGIIGDSGSTRSILMAQILGLYRYPQISYFA
TNPTLSNRVHFPSFFRTIPSDEFQMRGLAQLVFHFGWSWVGLLANDDDYGQYGLQMVKQ
EIINAGACVAFTENILTGQPNRNAPHIVQVIKESTAKVVVVITFDSDFIIVVEEMLRQNVTG
NIWVASESWATSVLLCNKRFQIVLGGSIGFAIHGGKMPKFTKYLNSLHPMDYLYDSLILEF
WEQIFSCKWYSQNDVDIQRNNDSIKICTGNEKVESLPPEEDRRLSLNVYIAVYAMAWALQ
SLLHCTQGTGPFHKGACANISSFYPWQLFHYIKNVNFKTKDGRQVFFDARGNPPAIYDIV
NWRLSDEGTLEQVTVGSYDFSAADGNTLSIEDAGIMWANSKTQVPVSKCSPSCPPGFQKV
ILPGKPLCCYECGQCPLGHISNQTDSVECHPCSWDTWPNLQQDRCLPRPTEFLSYGDPLG
YTLAAIAILSSLIPAAILGVFICFKKTPIVRANNYSLSCLLLLSLFLCFLCSLGFIGYPQPEKCL
LRQVAFGMVFALCISCVLAKTITVVIAFNATKPGSRLRKWTGVKLSCSIIILSSFIQLCICGM

WLIFSPSFPELDTDTKPGIIILNCNEGSPTAFWCMLGYLGLLASISFIVAFLARRLPDSFNEA
KLITFSMLAFLSVWVSFIPAYLSARGMYTVAMEVFAILSSSWAVVGCIFVPKCFIVLFRPNM
NSREHLTIKGHK*
>jgi|Xentr4|396053|e_gw1.680.76.1
GDILVGAVIPIHIDTLYRKVTFQEKPTSDICTKFLLQFYQQLQALRYAVEEINRSSDLLPNATL
GFYAYDSCAAMRNELQGTLWMLTGLSQEIPNYCCRESPPLSAIIGHSKSTYSMLMAHILGL
YRFPQISCYSTSSLLSDRTQFPSFFRTVPSDDFQSRGLAQMVLHFKWTWVGLVASNDDYGF
EGLKIIKDEITKGGACVAYTVYIATNQDNKNMPDIVQMIKESTANVIIVFSVDVFLFPFLDE
MVNQNVTGKQFVASEAWSVSNFLTNFSKILSGTIGFALQSSNIPGFQEYLNSVNPYNTPGT
TWSKMFWEEAFNCSFSQPQNQTLNLRKAKKTCTGDENLEHVQNGYNDVSTLRSSYNIYT
SVYVTAKALDDLSRCQSLDGALPETKCSDLTNFGPWQLTHYIKNVRVKLRNGREVFFHKD
GNPPAVYDIINWQPASNGTLKQVKVGSYDTLNFSGEIFSLDTSSVWWATSNAEIPLSVCSQS
CPVGFKKVAKKGEPVCCFQCVPCSQGEISNQTDAIECWSCPWDMWPNSARDRCIVKPIEF
LSYEDPLGIILATISALSSMVPIFIFRLFIHYKSTPIVRANNYSVSCILLVSLSFCFLCALAFIGY
PQPEKCLLRQAAFGLVFTLCVSCILAKTVIVVFAFMATKPGSSLKKWTTPRVPYMIITICTFI
QLTLCIFWLSISPPFPQYNIEAKPGIIVVECNENSQFAFWCMLGYLGLLASVSFTVAFLARRL
PDNFNEAKLITFSMLAFLSVWVYFIPASLSAQGKYTVAMEIFAILTSSWALVFCMFLPKCFII
LFRPDMNLRENLMVKTGV*
>jgi|Xentr4|396062|e_gw1.680.197.1
MGCNLIIERTRDYFSQPGDIIIGGTFPVYWNRVYKDLNFTTKPPELHCERYMPLRFGIPTMQ
ALIFAVEEINAQPKLLSNITLGFQVFDTCFNLRKAAHGTLSMLSGGKETTPNYHCNKLSPL
ASIIGDSGSSVSIIMAQILGLNRYPQNISWVKRSKIKKRGDIYKSLIKYTPAPHHPITPEAKN
VDWFFQDRKYLKTNAYLRKNVLPQIDNSMLGDLSLPFQLQEVLEAIENSTAKVVVALEAE
SSFVIVLEELLRQNVSGIIWVASESWATSAVHSESRFQRVAVGTLGFAIHGAQLPMFSKYLN
SLSPSKTLYDPFIKEYWEQSFSCKWLSQENLGNGTIQACTGKENLENTSPESYHRATLNVY
NAVYAIAWALQNLILCVPGNGPFDHGSCTNLSSFQPWHLLRYIKNVTFQTTYDSQVFFDA
KGNPPAIYDIVNWHVSNKGNLEQVTVGRYDLNAPDGKVFKIDSTGIIWPNNNTQAPLSNC
SPSCPPGFRKVMVTGKPPCCYECALCPLGRISNQTDAAECHSCSWDTWPNLQRDKCLQR
TTEFLSYKEPLGYGLSAISIFSSLIPLVILFVFIYYKTTPVVRANNYSLSCLLLLSLFLCFLCSL
GFIGYPQPAQCLLRQVAFGIAFSLCISCVLAKTITVVIAFNATKPSSRLRQWAGVRVSYCVIV
LCTFTQLVICVIWLFFFPPFPELDNDTQPGIIIANCNEGSPTAFWCMLGYLGFLASISFIVAFM
ARRLPDSFNEAKFITFSMLAFLSVWVSFIPAYLSARGMYTVAMEVFAILSSSWTVMLCIFVP
KCFIILFRPNMNSREHLMGKVRGQK*
>jgi|Xentr4|396067|e_gw1.680.71.1
MIAVNPCTCLNSRYGCSLPGESVAGSLSRPGDIVIGGTFMIHLDKVYTRLDFRTKPPQLQCK
RFAAEYYQSMYGLIFAIDEINSDPELLPNITLGFQVFDTCFTLRRAAQGTLSMLSGGKEITP
NYNCHGGGTLAGIIGDSGSTASILMAHILGLYRYAQISYFATNPVLSDRNLFPSFFRTIPSDEF
QMRGLAQLVSHFGWSWVGLLANDNDYGQYGLQMVKQEIMKAGACVAFSENILISQANR
NAPHIVQVIKESTAKVVVVITSDSDFVILAEELLRQNVSGIIWVASESWATSVLLSEERFQNI
LIGAMGFAIHGGQMPEFTNYLGRLHPSNNVNDSFMRDFWEQVFSCKWPDPGNTFTDNAT
VKPCTGNEKMESLAIESYHRLSLIIYTAVYAIAWALHNLLQCTPGTGPFQDGNCANLLHYI
KNVHFKTKEDNQVFFDAKGNPPAVYDIVNWRPTGKGTLEQVTVGRYDWSAQDGKVFNV
DSSIIIWPDNYTQVPVSKCSPSCPSGFRKVIVPGKPLCCYECARCPQGQFSNQTDAVECHPC
SWDTWPNLQQDRCLPRPTEFLSYGDPLGYSLAAISIFSSLIPLIILAVFIHQKKTPIVRANNY

SLSCLLLLSLFLCFLCSLGFIGYPQPEKCLLRQVAFGMVFALCISCVLAKTITVVIAFNATKP
GSRLRRWTGVKVSYSLIALCAFIQSILCLLWLVLSPPFPESDISTKPGIIIVNCNEGSPTAFWF
MLGYLGLLASISFIVAFLARRLPDSFNEAKLITFSMLAFLSVWVSFIPAYLSARGMYTVAME
VFAILSSSWAVVVCIFMPKCFIILFKPEKNSREHLRAKGKGHT*
>jgi|Xentr4|396069|e_gw1.680.63.1
MPLNWVQQVHKICYFLLSTSWVLCTLAIEVCAFSGSKAACNLPSYNVHGYLSRPGDVIIG
GTFPIHVDRLHYNLDFTRKPQELQCETFGIEFYQSMQALIFAIEEINAYPELLPNITLGFQIFD
SCITLRRAAQGALWILSGGPAITPNYNCHKGAPLAGIIGDCGSTQSILIAEILGLYQYPQISY
LSSHPILSDQNLFPSFFRTIPSDDFQMRGLAQLISYFGWSWLGILANDDDYGQYGTQMAK
QEIMNVGACVAFAEKIIIGQPNRNAPHIVQVIKESTAKVVVVITTDPDFMIIIQELLRQNVTG
NIWVASEGWSTSVLLSEELFRDMLLGTIGFAIHGRKMPEFTKYLNSLHPSKDAQDSFIRQF
WEKNFSCKWLNQSNLFETVGNTTLRPCTGNEKLETLMTEHRISLNIYTAAYTFAWALQSL
LECTPGNGPFYQGTCANITSFRPWHLLHYIQNVKFQARDGSQVSFDAKGNPPVIYDIVNW
HMSTKGTQKQILVGHYQFNAPNERNLTVDTTSIIWPNNNKQVPISKCTPSCSSGFRKMTVP
GKPPCCYECARCPQGQISNETDAVECHPCSWDTWPNLQQDRCLPRPTEFLSYGDPLGYSL
AVISIFSSWVPVIILGVFICYKKTPVVRANNYSLSCLLLLSLFLCFLCSLGFIGYPQPEKCLLR
QVAFGMVFALCISCVLAKTITVVIAFNATKPGTILRKWTGLKVSYSVIVLSAFIQLCVCLLW
LIFSPPFSELNSEDQPGIIIINCNEGSPAAFWCMLGYLGLLATISFIVAFLARRLPDSFNEATFI
TFSMLAFLSVWVSFIPAYLSARGMYTVAMEVFAILSSTWAVVVCIFVPKCFVVLFRPTMNS
KELLRRKDIGQH*
>jgi|Xentr4|396085|e_gw1.680.107.1
MILCKRPICHKLTQCTLYSTLVWCTITIDGCATSNPKGCTLPSESVNSYLSRPGDIVIGGIFRI
YLYAAYSDFHFSSKPLDLKCREVSIQHYQTMQALIFAVEEINADSDLLPNITLGFQMFETCIT
IRRAAQGTLSLLSGGNGITPNYHCYGGAPLAGVIGDSASPRSIIMAQILGLYRYPQISYYSTS
PILSKRDLFPSFFRTIPSDEFQMRGLAQMVTHFGWSWVGILANDDDYGQFGLQVAKQELM
NVGVCVAFSETILTGQSNTNAPHIVQVIRESSAKVVIVIATDHKFIVVVEELFKQNVTGTIW
VASEGWATSTLLSDKRFQIILKGTIGFAIHSGHLPKFADYLNSLRPSTYPGDSYMMEFWEK
VFTCKWLGQENIPIRVDNATFEVCTGNEKLESLLTQQDPRLSLNIYTAVYALAWALHSLIYC
TPGTGPFHDGTCANITSLHPWQLLHYVKNVNFKTKDGTHIFFDANGNPPAIYDIVNWRLN
AKGGLEQVTVGSYTLEHTDSKTLQIVSNGIIWPNNNTEVPISRCSPSCPSGFRKMIVPGKPP
CCYECAQCPQGQISNQTDAVECQPCSWDTWPNLQQDRCLPRPTEFLSYGDPLGYSLAAISI
FSSLIPLSIFGIFIRFKKTPIVRANNYSLSCLLLLSLFLCFLCSLGFIGYPQPEKCLLRQVAFGM
VFALCISCVLAKTITVVIAFNATKPGSRLRKWTGVKVSYCFIAFCLFFQLFLCIMWLIFSPPF
TELDTITYPGVIIVNCNEASLIAFWCMLGYLGLLASISFIVAFLARRLPDSFNEAKLITFSML
AFLSVWVSFIPAFLSARGMYTVAMEVFAILSSSWAVVGCIFVPKCYIVLFRPNMNSREHLT
VKSKVQK*
>jgi|Xentr4|396090|e_gw1.680.1.1
MDGIIQLGDIMLGAVLPLHIDKVYYPVSFRQRPPQINCTQFHLENFQQFQALVFAVDEINRN
PDILPNITLGFQVFDSCNVLRKDLEGTLQILTGYNKAMPNYLCLPSTPLIAVIGSYVSTPSIM
LAHILGLYRYPQVSHSSTSPLLSNRKTFPSFFRTVPTDAFQSKGMAKLVLHFGWTWVGLL
ALDNDYGQIGIQLVKQEIIKAGACVAFSETIIMSQPDRNAKHIVNVIKESTATTIIVFSLPIDL
VPILEEMLRENITDKMLVACQAWSTSSLFSDGKYSKIVSGTIGLALHSGSIPGFREFLNKAH
PFKGIAKHWLKLLWENMFSCQFLDKTNSTGPQEVPTKECTGEETLESINNNYNDVSSLRR
TYNIYTATHIVAKALEDLKSCKNGDDMFSDQTCTDIRHFNPWQLLRYLKKVKIMLNSGRE

LFFDENGDIPAIYDIVNWKVGPGGTIKQVKVGSYDTTAFFGQVFTINTSAILWATEDQQIPV
SVCSQSCPPGFRKVPISEKPACCFQCIPCPQGEISNHTGKSNSVDCAACSWDEWPNLQRSS
CIPKTIEYLSYEDPLGAALAYTGSLSSLVPVLILRLFITYRTTPIVKANNYSLSCLLLLCLSLC
FLCSLAFIGYPQPVKCLLRQVAFVLVFTLCVACILAKTILVVLAFMATKPGSKLKKWTSPY
MPYVIISFGFFVQCILCMVWLLMSPPFKEYNIQLKPGHIIVDCNNGSPTAFWSVLAYLGLLA
TISFIVAFLARRLPGNFNEAKFITFSMLAFLSVWVSFIPASLSAQGKYTEAMEIFAILSSSWAL
LGCMFIPKCFILLFRPNMNSKTQLIKKDSSKMKANHFT*
>jgi|Xentr4|396098|e_gw1.680.110.1
MFPKNNWFIFSTACVLSIIYNAACYSCTLPSESITIFQSRPGDVVLGGTFRIYLDGVFDDLHF
TKKMPEVQCKRFGIEPYQSLQALTFAVDKINADPELLPNITLGFQVFDTCRSLRRAAQGTF
AMLSGGEEIIPNYNCHEGAPLAGIIGDSGSTRSILMAQILGLNRYPQISYFSTSPILSERDLFP
SFFRTIPSDEFQMKGLAQLVSHFGWSWVGLLASDNDYGQYGLQLVRQEIINHGACVAFTE
NILTTEPNRNAPRIVQVIKQSTANVVIVIASDSDFVIVVEELLKQNVTGKVWVASESWSTSG
LLSAKRFQVVLMGSVGFAIHAGQIPNFTNYLSSLHPSKNPQDSHLWEYWEEVFSCKWLSP
KDLVANVTYKACTGNEKLESQLSEENVRLSFNVYTAVYAIAWALHNLQQCVSGTGPFYHS
DCANIFSFHPWHLFHYIKNVNFKNQDGSYMFFDEKGNPPAIYDIVNWRPRESTTLEKVVV
GSYDLNVPDGKTLKVDSSRITWSNTFDQVPLSNCSPRCPLGFRKVIIPGKPLCCHECSSCPQ
GQISSQIDAVECHPCSWDTWPNLQQDRCLPRPTEFLSYGDPLGYSLAAISIFSSLIPLIILAVF
IHQKKTPIVRANNYSLSCLLLLSLLLCFLCSLGFIGYPQPEKCLLRQVAFGMVFALCISCVL
AKTITVVIAFNATKPGSRLRKWTGVKVSYCLIMFCVFIQLFVCIMWLILSPPFHELETDTKP
GVIIVNCNEGSPTAFWCMLGYLGFLASISFIVAFLARRLPDSFNEAKLITFSMLAFLSVWVS
FIPAYLSARGMYTVAMEVFAILSSSWAVVGCIFVPKCVIILFKPNLNTRDHIMFKGSNK*
>jgi|Xentr4|396108|e_gw1.680.58.1
RFRLRFYQPFQAIRFAVEEINRNPDLLPNSTLGFYVVYDSCVVLQRELVGTLWMITGLDRVIP
NYCCREHPPLTAVIGHSKSSHSILMAHILGLCKFPQISHYSTSSLLSDKRQFPSFFRTVPSDDF
QSRGLAQMVLHFKWKWVGLIASNDDYGTKAIEVIRQEVVKEGACVAYTVYISDNLLNQN
LPNIVKTIKNSSANVLVVFSVDFYLFPLLVEMLKQNVTGKTLVASEGWSISEILAVEKYSSL
LFGTIGFAFYSSNIPGFQEFLNSINTHNTPGILWTQKLWEEAFGCTFINQSNLTDALKNVKK
QCTGEENLNSVQNSYNDVSRLTASYNIYTAVYVIATALHDLSQRQALDGPLVGDNFSDIGH
FKPWQLLGYFKNVRVKLSNNREVFFDKDGNPPAVYDIVNWQLGLDGTIKQMKVGSYDTL
NPSGDIFSINKKLIWWPTGNQEIPLSECSQSCPVGFKKAVIEGAPVCCFHCVPCAQGEVSNQ
TDAIECWKCPWDMWPNSKRDRCFRKPIEFLSYEDYLGITLATISTFSSMVPIFIFRLFIHYKS
TPIVRANNYSVSCILLVSLSFCFLCALAFIGYPQPEKCLLRQAAFGLVFTLCVSCILAKTVIV
VFAFMATKPGSSLKKWTTPRVPYMIITICTFIQLTLCIFWLSISPPFPQYNIEAKPGIIVVECNE
NSQFAFWCMLGYLGLLASISFIVAFLARRLPDSFNEAKLITFSMMAFLSVWVYFIPASLSAQ
GKYTVAMEIFAILTSSWALMFCIFLPKCFIILFRPNMNSRENILAGTK*
>jgi|Xentr4|396110|e_gw1.680.212.1
LNCLHYRFLAEFYQHFQALRFALEEINGNPTLLPNKTLGFYVVYDSCAALRREIEGTLWILT
GKSQAIPNYSCREKPPLTAIIGHSKSTYSILMAHILGLYKYPQISYYSTSSLLSDRAFFPSFFR
TVPSDAFQFRGLAKLVLHFKWTWVGLIATNDDYGNEAIKTINEEIIKGEACVAYTLYITSNL
VSRTTTNIVNIIKESSANVVIAISVGAYLAPILEEMLKQNVTGKNFVASEAWSISNFLLLSKY
SPLLSGTIGFAFQSSEIPGFQEYLNSINPITTPGKQWVQMLWEETFGCTFSDPTNQTFPMNN
SKKPCTGEESLKSIHNGYNDVSKLRSSYNIYTAVHVIATALHDLSQCHLSNGTLFSDECSDI
GYFKPHQLLHYFKNVRVKLSNNREIFFDKDGNPPAVYDIVNWQPGPDGSIKHVKVGSYDT

LNSSGDVFSLNINSVWWGTGNQETPRSVCSQSCPVGFKKLVKRGEPVCCFQCVPCAQGE
VSNQTDSIECWKCPWDMWPNSKRDKCFLKLTEFLSYEDPLGLILAAVSAFSSMVPMSIFR
LFFQYKSTPIVRANNYSVSCILLVSLSFCFLCALAFIGYPQPEKCLLRQAAFGLVFTLCVSCI
LAKTVIVVFAFMATKPGSSLKKWTTPRVPYMIITICTFIQLTLCVFWLTIFPPFLQFNIEAKPG
IIIVECNENSQFAFWCMLGYLGILATVSFTVAFLARRLPDNFNEAKLITFSMLAFLSVWVYF
IPASLSAQGKYTVAMEIFAILTSSWALVFCMFLPKCFIILFRPDMNSREKLMGK*
>jgi|Xentr4|396137|e_gw1.680.85.1
MDGNPSILGSHGILVPGDLLIGAVIPIHTDRIFPLATYKEKPTPDICKRFRLDFYQQFQTFRFV
VEEINRNPELLPNSTLGFYVYDSCAVLQRELGGTIWMITGLDRVIPNYCCQVHPPLTAVIGH
STSTISILMAHIMGLYRFPQISYFSTSSLLSDKRQFPSFFRTVPSDDFQSRGLAQMVLHFKW
KWVGLIASNDDYGSEAIKVIRQEVMKGGACVAYTVYISDNVQQQNLPNIVKMIKESSAN
VIVAISLPFFLTPLLDEMLKQNVTGKTFVASEAWSIANRLAVNKYVSLLSGTIGFAFFSSEIK
GLQKFLNSMTIYNTPGIYWAKELWEEVFGCTFVDQKNLTVTLNIKKQCTGEEHLSSVQNI
YNDVSILRASYNIYTAVYVIATALHDLRQCRSLGGALVGNNCLDIGHFKPWQVKEQIRMK
VRVTLNNARDVFFDKDGNPPAVYDIVNWQLGSDGTIKQMKVGSYDTMNSSGDVFSINTT
LIWWATGNQGVPNSVCSQSCPEGFKQSLKEGEPVCCFQCSPCAQGEISNQTDAIECWKCP
WDMWPNSARDRCLTKPIEFLSYEDYLGITLATISTFSSMVPIFIFRLFVHYKSTPIVRANNYS
VSCILLVSLSFCFLCALAFIGYPQPEKCLLRQAAFGLVFTLCVSCILAKTVIVVFAFMATKPG
SSLKKWTTPRVPYMIITICTFIQLTLCVFWLTISPPFLQFNIEAKPGIIVVECNENSQFAFWCM
LGYLGMLATVSFTVAFLARRLPDNFNEAKLITFSMLAFLSVWVYFIPASLSAQGKYTVAM
EIFAILTSSWALVFCMFLPKCFIILFRPDMNSRENLMRKEGGENKEHN*
>jgi|Xentr4|396161|e_gw1.680.202.1
MACKLPSQRINGFQTSPGDIVIRGTFPIHLDRLHHNFDFTSKPQEVQCQRFAPEYYQSMQA
LIFAVQEINQDPELLPNITLGFQIFDTCIALRRAAEGALWMLSGEQDITPNYCCDKGAPLAG
IVGDSGSTRSILMAQILGLRQYPLISYFASNPLLGDRNLFPSFFRTIPSDEFQVRGLAHLVSFF
GWSWVGLLASDDDYGQYGILMAKQEITNSGACIAFIQNILTGQPNRNAPHIAQVIKESTAK
VVIVIASDSDFLIVMEELLRKNVSGNIWVGSEAWVTSSLLSDKQFLAVLAGTIGFAIHGGQ
MPKFGNYLNSLRPSEDLHDSFIKELWEKTFSCKWLSQDSRDILMVNTTIQACTGNETHKD
LLTEELYRVSLNVYTAVYAIAWALQGFLHCIPGKGPFQHGACANISSFYPWQLLGYVKNVN
FQTKDGRHIFFDANGNPPARYDIVNWRVNAKGGLEQVVVGSYDLNAADGKILKVNSSAI
TWANSNTQVPVSKCSPNCTPGFRKVIVPGKPHCCYECAPCPQGQMSNQTDSVECHPCSW
NRWPNLQQDKCLPRPTEFLSYGDPLGYSLAVTSILSSLIPLIMFGIFLHYKKTPVVRANNYS
LSCLLLLSLFLCFLCSLGFIGYPQPEKCLLRQVAFGMVFALCISCVLAKTIIVILAFNATKPGS
SLKKWIGVKLSLCVIGFCVSIQFFVCLMWLIVSSPFSELDTETKPGILIVNCNEGSSIAFWC
MLGYLGLLASISFIVAFLARRLPDSFNEAKLITFSMLAFLSVWVSFIPAYLSARGMYTVAME
VFAILSSSWAVVACIFVPKCFIVLFRPTMKSRELLSKGRGQK*
>jgi|Xentr4|396181|e_gw1.680.218.1
MGPQGILVPGDLLIGGVVPIHVAKLYPTVTFHEMPPADICTMFRFENYQQLQAMRYAVEEI
NQRSDLLPNITLGFYAYDSCAALQSEIKGTLWMLTGKTEEVPNYCCRKSPPLAAIIGHSKST
YSMLMAHILGLYKVPQISYFSTSSLLSDRTQFSSFFRTVPSDVFQSRGLAHLVLHFNWTWV
GLIASNDDYGYKGLEVIKQEITKGGACVAYIIYMSRNPMDQNIHNIVQVIKESSARVLVAFC
TDVYLIPVLDEMLKQNVTGKSFIASEAWSISNVLSVPKYSSLLSGTIGFAFHSSTIPGFQRFL
NSINPLNTPGTMWSKFFWEEAFGCTFSNQTNSSDIVNALENPCTGDEDLENLQNSYNDVS
NLRASYNIYTAVYVIAKALDDLNHCQTLAGPLFNKYCATLENFEPWQLLHYLKKVRVKLS

NGREVYFDEDGNPPAVYDIVNWHPGSDGSLRQVKVGRYDTMDTSRNVFSINISAIWWPS
GNEEVTSTTFFLQQIQLTFSNIPLSVCGQSCPEGFRKAARRGEPVCCFECVPCAQGEISNQT
DSIECWKCPWDMWPNSKRDRCFPKPIEFLSYDDSLGITLAAISVFSCVVPLAIFRLFIHYKS
TPIVRANNHLVSCVLLVSLSFCFLCALAFIGYPQPEKCLLRQAAFGLVFTLCVSCILAKTIIV
VFAFMATKPGSSLKKWTTPRVPYMIITICTFIQLTLCVIWLTISPPFPQYNSEAKPGIIFVECD
ENSPYAFWCMLGYLGFLASVSFTVAFLARRLPDNFNEAKLITFSMLAFLSVWVYFIPASLS
AQGKYTVAMEIFAILTSTWALVICMFLPKCFVILFRPNMNSKENLMGKDRRKEIKKKIIF*FI
KWYSFVFLHYFAKINFLTKQYIGLADFHSHLIISTLKLYINTLVGEEDWSLGKGRSR*
>jgi|Xentr4|396189|e_gw1.680.83.1
MAWILCTLALGVSTSLDFTSGCSLTSDSIRGYLSRTGDIIIGGNFMIHLSRTYNYLDFTRKPL
ELQCEMFATEYYQCMRALIFAVEEINADPDLLPNVTLGYQILDKCITLQLTTQTALWMLSG
GQEMTPNYHCGQGPPLAGIIGDSASTHSMLMAQILGLYRYPQISYLSTNPILSDRNLFPSFF
RTIPSDEYQMIGLGQLISHFGWTWVGLLANDDDYGQFGMQMAKQEIVSLGGCVAFSENIL
MGQPNKNAPHIARVIKESTAKVVVVIATDPNFVIVVEELLRQNVTGIVWVASEGWANSAV
LSEKRFRAILKGAIGFAIHGGQMPSFTKYLNSLHPLKDIHDPFIKELWEKTFTCKWPDQNY
LYQWADNATIQACTGNERLDTVLTEDFHRIPLHVYAAVYAMAWALHNLIYCKPGDGPFHH
GACANISTFQPWHLLHYVKNVNFKTKEGSQVFFDAKGNPPTIYDIVNWQESAKGALEQV
GIGTYDLSTPDGKAMKVNSGDIIWTNNETQVPISKCSPNCQTGYRKLIVPGKPICCYECAR
CPQGHFSNQTDAAECHPCSWDTWPNLQQDRCLPRPTEFLSFQEPLGYSLAVAAIFSSLSPL
TVLGVFIHYKNTAIIRANNYSLSCLLLLSLFLCFLCSLGFIGYPQPEKCLLRQVAFGMVFAL
CISCVLAKTITVVIAFNATKPGSRLRKWTGIKVSYCVIIFCVFIQLWICILWLLFFPPFHELDL
DTKPGFIIVTCNEGSPTAFWCMLGYLGLLASISFIVAFLARRLPDSFNEAKLITFSMLAFLSV
WVSFIPAYLSARGMYTVAMEVFAILSSSWAVVGCIFVPKCFIVLFRPHMNSRDYLMGKGR
NLK*
>jgi|Xentr4|396192|e_gw1.680.67.1
MQALIFAIKEINADPGLLPNVTLGFQVFDTCLALRKAIQATLQMMSGRGEITPNYHCRKES
PLAGIIGDSESTGSILMAQILGLNRFPQISYFATNPMLSDRGLFPSFLRTIPSDEFQMKGLAQL
VLHFGWSWVGLLASDNDYGQYGLQMVKQEIINGGACVAFSENIITGQPNRNAQYLAQVI
TKSTANVVVVVVTSDVDFVIVLEELLRQNVTGITWVASEAWATSDLLSGERFRKILSGTIGL
AIHSGTMPEFSKYLNSLHPASHLHDLFIREFWEKTFSCTWPSQETLVTRLNSSTLPACTGNE
KLEGLATEEGHRVSLNIYSAVYAIALGLQNLLDCKPGTGPFADGSCADISTFQPWQLLHYI
KNINFKNKDQNPIFFDAKGNPPAVYDFVNWRLGTKGTLKRTTVASYDLSGPDWKTFNVD
TSGIIWANRNTQVPLSKCSPSCSPGFRQMIVPEKPPCCYECARCPQGQISNQTDAVECHPCS
WDTWPDPNRIKCVPKTTEFLSYEGPLGYGLTSTAMISSPVPLFILGIFICYKKTPIVRANNYS
LSCLLLLSLFLCFLCSLGFIGYPQPEKCLLRQVAFGMVFALCISCVLAKTITVVIAFNATKPG
SRLRKWTGVKVSYCVIGTCVLIQLFLCVIWLILSPPFPEYDTGTKPGIKIVVCNEGSPTAFW
CMLGYLGFLAMISFIVAFLARRLPDSFNEAKLITFSMLAFLSVWVSFIPAYLSARGMYTVA
MEVFAILSSSWAVVGCIFVPKCYIVLFKPNVNSRKHILGKRGPM*
>jgi|Xentr4|396195|e_gw1.680.59.1
MICCKEQRGHRLKWVIFLKTLALCNISIDKCSCSDLKIGCTLPSESVNGNLSRPGDIVIGGIF
RIYMKATYSDFRFTRKPLELKCEEVSIQHYQSMQALIFAIEEINASPEVLPNITLGFQIFDTCI
TIRRAAQGTLSLLSGGEGITPNYHCHKGAPLAGVVGDSGSTRSILMAHILGLYKYPQISYFS
TSPILSKRDLFPSFFRTIPSDEFQMRGLAQMVAHFGWTWVGILANDDEYGQFGLQVAKQE
LMSGGACVAFTENILTGKPNRNAPHIAKVINDSTAKVVVVITSNSDFYIVAEELLKQNVTG

KIWVASEAWSTSAYLSIDRFRALLVGTIGFAIHSGQLPKFNKYLNSLHPSKYPHDSYMAEF
WEKVFSCKIIQQENVDNTTIKFCTGNEKLENFFIQDDHRSSLNVYTAVYAYAWALHHFLYC
TQRSGPFQRNTCANITMFHPWHLLHYVKNVNFLAKDGSHVFFDAKGNPPAIYDIVNWRV
NPRGTLEQATVGSYNLNAPDGNILNIVSAEIIWFNNDTQVPVSKCSPSCGSGFRKVIVPGKP
PCCYECARCPQGQISNQTDAVECHPCSWDTWPNLQQDRCLPRPTEFLSYEQPLGYSLATIS
MFSSLTPIAILGIFIHYKTTPIVRANNYSLSCLLLLSLFLCFLCSLGFIGYPQPEKCLLRQVAF
GMVFALCISCVLAKTITVVIAFNATKPGSRLRKWTGVKVSYCVIMLCALIQIIDCALWLIFS
PPFHELDTDTQPGVIIVNCNEGSPTAFWCMLGYLGLLASISFIVAFLARRLPDSFNEAKLITF
SMLAFLSVWVSFIPAYLSARGMYTVAMEVFAILSSSWAVVGCIFVPKCFIVLFRPNMNSRIH
LSFKGKGA*
>jgi|Xentr4|396211|e_gw1.680.200.1
MDTSYCLILHLLLPCLIVTLQPNQHSCRLHVSATEGIFQPGDVMLGGLFPVRIDKEYQPVSF
RERPPGINCTTFHLENFQQLQAMIFAVNEINTNPNILPNITLGFQVYDSCDVLQQDLEGTLQ
VLTGHTGAISNYRCLKNTPLAAVIGASSSTHSILMAHVLGLYRYPQVSYFSTSPLLSNHKM
FPSFFRTVPSDAFQSQGLAKLVLHFGWTWVGLLAADNDYGQQGIQLVKQEIIKAGACVAF
TESILTSQPDRNAQHIVKVMKESTATAIVVFSPTIYLVPILEQMFIQNVTKKILVASEAWSTST
LFSNGKFSEVLSGTIGLALHSGGIPGFGDFLNKIHPSVSLGQYWVRLLWEKSFNCKFQNES
HLTSASADAMQKCTGEEHLGNIANSFNDVSSLRVTYNVYTAVHAVANALEDLTNCISERS
KFSNQKCPSISNFTSWQLLQYMRKVKIPLSSGRMLIFDENGDPPAVYDIVNWKLRPDGSM
KQVNVGSYDITAAFGHVFSVNMSAISWATGHQQVPLSVCSQSCPPGFRKATVREKLACCF
QCIPCPQGEISNHTGNSRCSWCSWDEWPNLQKSKCLQKNIEYLSYEDPLGAGLAATGIASF
WAPVLVLNIFIKYKSTPIVKANNYCLSCLLLLCLPLCFLCSLAFIGYPQPKKCLLRQAAFGL
VFTLCISCILAKTFMVVFAFMATKPGSRIKKWTSPSVSYMIIFICFGFQFILCISWLLEAPPFP
QYDIQTKPGIIIIECNNGSATAFWCMLGYLGLLATASFFVAFLSRRLPDNFNEAKFITFSMLA
FLSVWVSFIPASLSAQGKYTEAMEIFAILSSTWALVVCLFLPKCFIIVFRPNMNSKKELMKK
TSAHLQISMY*
>jgi|Xentr4|396222|e_gw1.680.94.1
MFVSLLSFRFRRELYQHFQALRYVVEEINRSPDLLPNVTLGFYAYDTCSVIKSELQGTLWM
LTGLTQEIPNYRCRDSPLAAIIGHSKSTFSILMAHILGLYKFPQISYYSTSSLLSDRTQFSAFFR
TVPSDVFQSKGLGQLVLHFKWTWVGLIASDDDYGYEALQVIKEEIIKSGACIAYTIYISTN
QKNQDMPNIVKLINKSTANVIIAFSVDVFLIPLLDEMLEQKVTGKTFVASDAWSISTPLTTV
KYSTILLGAIGFAFHSSKIQGFQEYLNSLNPINPPGTDWSKMLWEETFNCTFSDHTNLSDIL
NNEESMCTGKENLEHIHNSYNDVSSLRASYNIYTAVYLIATALDDLSHCQGLTGPFSGGKC
SDIRNIKPWEVLYYIQKVRVKMSNQREVYFDKDGNPPAVYDIVNWQPGSDGTLKQVKLG
SYDTLNSTGNVFSLNTSLIWWGTVNQETPISVCSQSCPVGFKKVMKKEEPVCCFQCVPCS
QGEISNQTDSIDCWKCPWDMWPSGARDRCISKPVEFLSYVEPLGITLAATSVFSSLVPISIF
YLFIHYKSTPIVRANNYSVSCILLVSLSFCFLCALAFIGYPQPEKCLLRQAAFGLVFTLCVSC
ILAKTVIVVFAFMATKPGSSLKKWTTPRVPYMIITICTFIQLTLCIFWLSISPPFPQYNIEAKP
GIIVVECKENSPFAFWCMLGYLGFLASVSFTVAFLARRLPDNFNEAKLITFSMLAFLSVWV
YFIPASLSAQGKYTVAMEIFAILTSSWALVFCMFLPKCFIILFRPNMNSRDNLMGKDRSSR*
>jgi|Xentr4|396231|e_gw1.680.216.1
GDILIGVLLPFHIDKTFLKISFTETPPTVSCTRFLLETYQQFQAMRFALDEINKSPTLLPNVTL
GFYVVYDSCANLRKEQEGTLWMLTGMNKTVPNYCCQEWPRLAAVLGHSVSTYSILMAHIL
GLYRYPQISHYSTSSSLSDRSQFPSFFRTVPSDAFQSRGLAHLLLYFGWTWVGLVGMGSDY

GEQGVYLITQELIKAGACVAFTGFILSNREDKNVPYLTRIIKESTARVIVVFAPDIYFAALLD
ELLKQNLTGKIFVASEAWSISSMLSAEKYSSVLTGTIGFAFSSSPLPGLRGFLNKVRPSNTPG
QTITKIFWENIFKCKFMDQGNLTGTWIDSTKLCTGKEDLERVESSYNDVSNLRSSYSIYTSI
QILARSLHNLFSCWNGKETLSGGTCVDAKNFKPWQVRNYNAHVKLSNGREVSFDISGDL
PAVYDIVNWQLRADGTMKQVKVGSYDTADTNGNIFSINITALMWAQGDHQVPSSVCSKS
CPPGFRRAVIRGQPICCFQCVPCPYGEISNKSGSNECSKCPWNMWPNVDKDRCVPKATDF
LSFEEPLGTTLTAATITSSLVPLSVLGLYYCYRSTPIVRANNYSLSCTLLVSLTLCFLCSLSFIG
CPQQLKCFLRQVAFGMVFTLCVSCILAKTIMVVLAFMATKPGSSLKKWTGPRVSYIIVSIC
NILQLCLCICWLSLFPPFPEYNTDTQPDIIIIVCNEGSTIAFWCMLGYLGLLATISFIVAFLAR
HLPESFNEARFITFSMLSFLTVWISFIPASLSARGQYIVAMEVFAILSSSWALLVCMFLPKCFI
IVFRPNLNSREHLIRRARN*
>jgi|Xentr4|396233|e_gw1.680.208.1
MILSTAWILYCIGEGFGSGCSLPSEPITGYVSQPGDIVIGGTFPVHLSRIDNNLDFTRKPPEM
HCQTIATEYYQSMQALIFAVEEINGDHELLPNITLGFQIFDKCTTLRRSAQGALCMLSGGN
KITPNYRCHNGAPLAGVIGDSGSTHSILMAEILGLYRYPQISYLSTNPILSDRNLFPSFFRTIP
SDEFQMRGLAQLVSYFDWSWVGFIADDDDYGQFGLQMAKQEIINAGACVAFAENILIGQP
NRNAPRLVQVVRESSVKVVVVIASDHNFVIVVEELLRENVTDKIWVASEGWATSALLSQK
KFQVVLVGTIGFAIHGAKMPEFTKYFQRLHPFQDFYDPFIEEFWGQTFSCILLNQGNPVQL
SSNSTIQTCTGNETLESLLTGNYHRISFNVYTAVYTIAWALQALLDCPPRTEPFHYVGCSSIF
SFRPWQLLYYIKNLNFQTKFGNQVVFDAKGNLPAVYDIVNWHGGTNCTLEQVVVGSYDL
STSNGNTLKINIPAIIWTNNNTQVWAAQLVSVPLSKCSPSCTSGFRQMMVPRKPSCCYECA
RCPQGQISNQTDAVECHPCSWDTWPNLQQDRCLPRPTEFLSYEKPLGYSLAAISIFSSLIPLI
MLAVFIYYKKTPIVRANNYSLSCLLLLSLFLCFLCSLGFIGYPQPEKCLLRQVAFGMVFALC
ISCVLAKTITVIIAFNATKPGSRLKQWIGLKVSYCVTVLCVFVEFCMCMIWLIYFPPFPELD
TNTKPGIIIVNCNEGSAAAFWCMLGYLGLLATISFIVAFLARRLPDSFNEAKLITFSMLAFLS
VWVSFIPAYLSARGMYTVAMEVFAILSSSWAVVGCIFVPKMFILLFKPNLNSREHLIRNE*
>jgi|Xentr4|396242|e_gw1.680.65.1
MVKVSIYSYIEYIEIWIFIANILLILPLTRLRQTLQPCDLLLLCLCRLNAPSHWCFWHLVIFCF
IPDPHCSDVRCSLGKSEVSMILEPGDVLIGVLLPFHVGRIPLKTTFTETPPQLPCARFLLETY
QQFQAMRFALDEINKSPTLLPNVTLGFYVYDSCAHLRKEQEGTLWMLTGMNKTVPNYCC
QEWPRLAAVLGHSVSTYSILMAHVLGLYRYPQISHYSTSSSLSDRSQFPSFFRTVPSDAFQS
RGLAHLLLYFGWTWVGLVGMGSDYGQQGVYLITQELIKAGACVAFTGFILSDHDDKNVP
YLTRIIKESTARVILVFAPDIYVAALLDELLKQNLSGKTFVASEAWSISSMLSAEKYSPVLTG
VIGFTFSSSPLPGLRGFLNKVHPSNTPGETITKIFWENIFKCKFMDQGNLTGTWINSTKLCT
GKEDLETVESSYNDVSNLRSSYSIYTSIQILARSLHNLFSCWNGKETLSDGTCVDAKYFKP
WQLTHYIQNAHVKLSNGREVSFDLNGDLPAVYDIVNWQLGVDGIMKQVKVGSYDTAAA
DGNIFNVNISAVIWAPGDHQIPSSICSKSCLPGFRRAVIRGQPLCCFQCIPCPQGEISNKTGSD
DCTKCSWNMWSNADKDRCIPKAIEFLSSEEPLGATLTITSIISSLVPLLVLGLFYHYKSTPIV
RANNYSLSCTLLGSLSLCFLCSLPFIGCPQQLKCLLRQVAFGIVFSLCISCILAKTIMVVLAF
MVTKPGSSLKKWTGPRVSYIIVSICNILQLCLCICWLSLFPPFPEYNTDTQPEIIIFICNEGSAI
AFWCMLGYLGLLASISFIVAFLARRLPDSFNEAKYITFSMLAFLSVWVSFIPAYLSARGMY
TVAMEVFAILSSSWAVVGCIFVPKCFIIVFRSSMNSREHLMRRARN*
>jgi|Xentr4|396247|e_gw1.680.49.1
MDGIFQPGEIIVGALLPLHIERVYYPISFRERPPRINCTTFHSENFQQLQALIFAVEEINKNPSI

LPNITLGFQVFDSCNVLQKDLEGTLQALTGYSWPIPNYRCLDSTLAAVIGPYVSTPSIMLA
HILGLLRYPQVSHSSTTSLLSNRKTFPSFFRTVPSDAFQSKGIGKLVLLFGWTWVGLLALD
NDYGQIGIQLVKQEIIKAGACVAFSETIIMSQPDRNAKHIVKVIKESTATTIIIFSLPADLAPIL
EEMLRQNIYNKMLVGCHGWSASSLFSDGKYSKIVSGTIGLAFSSGTIPGFREFLNKAHPFR
GIAKPWLKLLWEDTFSCQFLDKTNSTGPKEVPTKECTGEESLESINNNYNDVSSLRRTYNI
YTATHIVAKALEDLKNCNNGYDMIPDKTCLDILHFQPWQLLKYIKKVRIMLNSGRELFFD
KNGDIPAIYDVVNRKLSPDGTTKQVKVGSYDSTAFFGQVLTINTSAISWGTEDQQIPVSVC
SQSCPPGFRKAPLSGKPACCFQCIPCPHGEISNTTGKWWVHLLLTYSVDCMKCSWDEWPN
LWKSKCIEKNIEYLSYDDPLGAALTTTGIVSTLVPVLILRLFITYKETPIMKANNYSLSCLLL
VCLSLCFLCSLAFIGYPQHEKCLLRQAAFGLVFTLCISCILAKTILVVLAFMATKPGSKLRK
WTSRSVSYMFISICFFVQFLLCLVWLLRAHPFTEYNIQLRPDLIIVECNNGSPTAFWSVLGY
LGLLATISFIVAFFARRLPGNFNEAKFITFSMLAFLSVWVSFIPAYLSAHGKYTEAMEIFAILS
SSWALIGCMFIPKCYILLFRPNMNSKIQLIKRP*
>jgi|Xentr4|396248|e_gw1.680.203.1
MEGIFQAGDIMLGALLPLHIDKEYHPVSFRERPPNINCTTFNLENFQQLQALIFAVEEINAN
PNILPNITLGFQAYDSCNVLQKDLEGMFQILTGYSRAIPNYRCLKNIPVAAVIGPYVSTHAIL
LANILGVYRFPQISHFATSRLLSNYMMFPSFFRTVPSDAFQSQGLAKLVLFFGWTWVGLLA
VDNDYGQQGIQVVKQEIEKAGACVAFSETILMSLPDRNAKHIVKVIKDSTATAIVVFSLPA
DFLPVLEEMLVQNITKKMLVASEGWSTSTLFIDHKFTDILYGTIGLGLQSGIIPGFRGFLNKA
HPSTGLGKYWLKLLWEEIFTCKFLEEKNLTAPTEKPAHICTGMESLESISNNYNDVSSLRTT
YNVYTAVHVVAKALEDLKNCCDAGDLCPHRRCPDIWNFTPWQLLYFMRKVRIKLSSGRD
LFFDQNGDPPAVYDIVNWKLSPEGTIKQVKVGSYDTAASFGQVFTVNTSAIVWATGDQVP
VSVCSQSCPPGFRKAPISGKPACCFKCIPCPQGEISNHTGKAYIAVIIFSSDSIDCFKCSWEEW
PNLQNSKCIQKGIEYLSYEDPLGATLAAISILSSLVPVFILRLFIKHRTTPIVKANNYSLSCLL
LLCLPLCFLCSLAFVGFPQPEKCLLCQAAFGLVFALCVSCILAKTIMVVLAFMATKPVSKL
RRWASPLVSYMIISLCFLLQFLLCTVWLLKAPPYTEYDTQTKPGLIIVQCNNGSTAAFWSM
LGYLGLLATISFIVAFLSRGLPDTFNEAKYITFSMLAFLSVWASYTLASFSAQGKYTVATEIF
AILSSSWALVVCMFASKCYIILFRPLLNSKKSLMKNDSRNIKSKYKT*
>jgi|Xentr4|396295|e_gw1.682.1.1
MMRYLNSTEEYLAYLCGPKRSRLSLPMTLVYATIFLIGVIGNTLVCLVILKHHNMRTPTNY
YLFSLAVSDLLVLLLGMPLEVYEMWSNYPFLFGAWGCYFKTVLFETVCFASILSVTTVSVE
RYVAIIHPFQAKLKSTRSRALKILVTLWIFSILFSIPNTSTHGILLQYFPNGSLIPDSATCTVVQ
PLWIYNCIIQVTSLLFYILPMGVISVLYCLMGIKLRGDHSLEADKMSVNVQRPSRKSITKML
FVLVMVFGICWAPFHVDRLFFSFVLDWTEPLANAFNLTHVVSGVFFYLSSAVNPLIYNLLS
RRFRSAFQNVLPPFFKSLKPKIPVQSLAPPKSTLILTVRNRMDSGEEGSPSFPHRTSVCSSHL
STVL*
>jgi|Xentr4|396611|e_gw1.687.55.1
MEEALNRSNHTRMTYFILAGISDFPEMQALVFVLVLLIYLITIWGNLIILLLIFLDRALHRPM
YFFLSNLSCLDLMYTTVTLHRILFMLISGNNVISFSECIAQLYFFMSLVGIELLILTAMSYDR
YVAICNPLRYHVVMNYKICILIASSCWVLGFCDTGPFTYIVYGFSCYKTDIIDHFFCDLLAL
MKLTCNDTSRLEHVILLEAAFSGLTPFLLTVISYIAIICTILRMQTKSGRHKAFYTCSSHLTV
VLLFYATLSCLYLRPLSMFSDSDKLFALVYTAVVPMINPLIYSFKNKDVKLALKRVLKH*
>jgi|Xentr4|396614|e_gw1.687.7.1
YLFAVGGNIGILFLVGMDSKLHNPMYFFLGNLSVLDISSITVTLHKTLAIFLTGNKTLSFPGC

ITQVYFFMALECTELLILAAMSLDRYAAICNPLHYPMIMNPRTCAFLASVCWVLGFIEVIP
HAVITNNLSCYTSNEINHFFCDVLPLMKISCSDTSLLKLWIVTEGVFVSGVVPLVTTLVPYIF
IIRAILKLRSTSGRQKAFYTCSSHVTLVIVLYLTLYCLYLTPPSENTLDSHKLFSLFNTAAVPIL
NPLIYSLKNKDVKSAVKRQLHYLYCKMIYPLTLNFKKCEKHIES*
>jgi|Xentr4|396621|e_gw1.687.49.1
MYTIAANKTVVGYFIIKGISDVPELQLLIFLLVLLLYLIILVGNLSILLLVCSDSQLKTPMYFF
LGNLSILDMSSATVTLHKIFLSYMSGDRTVSFIGCMSQVYVFASFTSQELLLLTAMSYDRYV
AICKPLHYHIVMTPRVCMLLAMFCWLWGFLQVLPPVYILANFSCYLSNELNHFFCDITSL
MKLSCSDTGVLVLLNLTEGLLVSTLTPFILTFISYIFIINSILKIQTSTGRHKAFYTCSSHLTVV
VLLYIILTCQYLVPNSTSSIDFNKHFSLFNTMVVPIMNPLIYSLKNEGVKSAIRRRLRLWRNS
V*
>jgi|Xentr4|396677|e_gw1.689.7.1
FIVGILGNMIIVIGLTCLLQSKSIANIYIVNLAIADLSFVATLPLWAVSMAAKYQWTFGSFMC
KFCATMSSVNMYSSIFLLTCLSIDRYFGIVHPMKSLNWRTQAKAKIATFIVWISACATSFPT
MYFRQTYYSKKHQIITCAMKYPKHGVFWPNFVDLMKNIFGFVIPFIVQGVCYCLIYKNML
ASRKNRVKKSRSDKVLKVVLAMVFAFLVCWLPFQIASFLKVLIRMHWIRDCKTAEIINAV
MPVTVCIAFSNSCVNPILYFFASKRFRNQLAIALRKALSQSYSTTRTR*
>jgi|Xentr4|397613|e_gw1.702.9.1
MSEPDDFAGLAGPWNSSDLSLFSPASIVVPVIFSLIFLLGTVGNSLVLAVLLRSGQRTHNTT
NLFILNLSVADVSFIIFCVPFQATIYSLEDWVFGAFMCKAVHFFIYLTMYASSFTLAAVSVDR
YLAIRYPLRSRELRTPCNAIATMAIIWGLSVVFAGPYLSYYDLMDYESSHICMPGWKERTR
KIMDTGTFIVGYVIPVLIVSLSYTRTIKYLWTAVDPLEDMSESKKAKRKVTKMIIIVTVLFC
LCWLPHHVVVMCYLYGDFPFNQATYAFRLLSHCMAYANSCLNPIVYALVSKHFRKGFQK
VFSCLLRKKGRNKVHVVNAAHAEPGFDAASSEMSHINDDNGKMHGHHAGANHSDSSRP
LGTP*
>jgi|Xentr4|398387|e_gw1.713.23.1
MYHFKNSTENTSHCVFNSVITSRVLPVFYSLVFIVSIILNGLNFWIFFYVPSNRSFIVYLKNI
VFADLLMTLTLPLKIVSDVELGSALLNIIVCRYTAVIFYLNMYIGIIFLGILGLDRYQKVVRP
MHTSSVQNVGYSKALSAVVWMFMAVVSVPNMILTNQPFHAANYTNCARLKSHLGIQWH
QASNYICVSVFVVVFFLLVIFYVSISRTIYKSNQRFKNGSNMKAKSSRNIYSILFVFFVCFVP
YHALRIPYTLSQVGADYSCSSKTILYSMKEVTLLLAASNVCLDPIIYFFMCQPFRNMLFKK
LHLTCGEMEQDKTCRVSSTMPGISM*
>jgi|Xentr4|398403|e_gw1.713.20.1
MNVTSPRANSTAFSELATIIFPVLYLVIFLGSIMLNGLAVWIFFHIRNKTSFIVYLKNIMIADL
LMTVSFPFKIIQTSGIGPWNFNVYHCRYSSILFYTSMYISIVFLGLISTDRYLKVVKPFGSSK
MYNIKFTKVVSLMVWLAMSFMAMPNVIFTNVQPTRDNIYDCIYLKSSFGAKWHEAVAYI
DTCIFFIVMVVLIVCYISISRHIQKSSKPFVSCSSRTRRHNQSIRVVVAVFFVCFLPYHLCGLP
FMFSRLDKILDKRIYTILLYCKESTLFLSACNVCLDPIIYFFMCRSFSQRLFNRSGMRSRSESI
RSLQSVRKSEVRIYCEYTEV*
>jgi|Xentr4|398412|e_gw1.713.24.1
ESFLKKYYLSAAYTTEFVVGFLGNCVVLFGYIFCLKNWSSGSVYLFNLCISDFAFLCTLPM
LVFSYIHEEWTFGDFLCKCNRYLLHENLYTSILFLAFISIDRYMLIKYPFREHILQRKSTAIMI
SLGIWILVSLKILPILFLVKQVEVNNVTICLDFASSGEASWSLVYSLCLTLTGFVIPLCVIWIF
YLKMMCFLKNRNKELTNGASFEKPVTIVVLAATMFSIFSLHTMSLEM*ELHLGQTHGHSW

TPMIIRSAFIIARPIAFLNSMINPVFYFLMGDHFREMLLAKIQSLYKSLKCTINSRWA*
>jgi|Xentr4|398416|e_gw1.713.26.1
MVITYINKLINK*N*NPSSFFSQDPIEQNNCTDTESFLKKYYLSAAYTMEFVIGFIGNCVVLF
GYIFCLKNWSSGSVYLFNLCISDFAFLCTLPMLVFSYIHEEWTFGDFLCKCNRYLLHENLY
TSILFLAFISIDRYMLIKYPFREHILQRKSTAIMISLGIWILVSLEISPILSFIKQVEINNVTICLD
YASSGEATGSLIFSLCLTFIGFVIPLCVIWIFYLKMMCFLKNRNQQLPNGASFEKPVTIVVLA
ATMFSIFFTPYHVMRNVRIASRTDSWTVSECSSMIISSAYTVTRPIAFLNSMINPVFYFLMG
DNFREMLLAKIQRLYKSLKCTFNST*
>jgi|Xentr4|399214|e_gw1.725.76.1
MEAVTYNYDEYSDYDNDTEPDCQDENLPHGHWILPTLYLIFFFFGLTGNAIVIAIVSRRSSR
RADIFILHLAISDLLFVLTLPFWASSLALGGQWSFGVHMCRASGFIIAVTRCASSLLMAIMS
VDRYLAVIGGHRIHPFRTRTCSLGACCAIWLLSLVTGIPPLVFRNLVENVCVESSESRLSVGI
KLATLFLTFVLPLTVVAFCYSCMAKHLWNYFGGPQNVAGGKAKPRRRHSWLRIVSCVVAA
YSLSWLPYNTLSTVRLVAELGEGFPCHTTVAINQALSATAAIAFANSCTNPLIYSILDAGFR
HRTKQTLPSIFPICQAVFHLPFRGGSQQSTVASTESSSSYTRSY*
>jgi|Xentr4|399458|e_gw1.729.82.1
MLGFRPTYSSFKILIYLFAQCIKPSGAEPIKPACYLKPATVLEDFEYIQDGDIIIGGLLSVNTN
AMSLLYEDEQTGDKVTVCIDIEYQYYRQLVEFRLAIEEINKNPSLLPNVTLGYHIYDSCGH
EMKAVRSTLQILSGTKEPVPNYSCGRKRNIAGFIGDLTSETTMISAQILSLFGFSQVSFGATD
PSLSDRVAFPYFFRTKQSFHGSTFAISKLMKHFRWTWVGIIGLNDSDMDDEPQVLTDYLSR
DGICVEFTIKMSRYLDEFLDGGIDRIGKIIKKSTTNIIIVCGKAYKVIVVVSYIRSMLMLIKKT
LVFSPSASVIYNNVDFITAIFDGSFIFEPYPVYPRETHEIIEFINRTHPSKDPKDTLFENIMLRQ
CNCLSKDPHKNKVYEHLYKAHYTECDGKKATECLLQIISCLLAALSPNVHLAVNIMSQAI
HEMHTSLREQSPERDREAHRYQYQLHHYLKKSHYQTKYGGEVSFDGRGEMDTGYIIYFF
PNGTDATYLGTLGRFIPSAPSEYKLIIYPDAIPWKMKKKMIPRSQCTDSCQPGYRKAVNPG
AQPCCYGCLPCSEGEISNRTDSENCIRCPDLEWPNNKRNQCIVKTEEFLSFTDGTISVSLLSI
TVLFSFITLLMLRIFILYRDTPIVRANNRSLSFLLLVSIKLSFLSVFLFLGRPVDITCMLRIITFG
ITFSIAVSSLLAKTIMVCVAFKATKPGSSWRKWLGVKLSNSVVLFCSSIQIIICMTWLAISPP
FQELDIHTSPGTIIIQCNEGSAIGFYSVIGYMGLLAAVSFVLAFLARSLPDSFNEAKYITFSM
LLFCSVWITMIPAYLSTKGKNTVCVEIFAILTSSAGLLACIFLPKCYIIMFRPEMNQKSCLLG
SKT*
>jgi|Xentr4|399468|e_gw1.729.86.1
MLMYLLVLCIGPCRSRLQPINPACCLKTAEIFEDFEYFQDGDLIIGGLLTVNTYATYEDLPW
ETNHKVMCSNLQPRYYRQLVEFRLAIEEINKNPSLLPNVTLGYHIYDSCGHALKTVRSTLQ
MLSGTKDFVPNYSCGRKRNIAGFIGDLTSETTIISAQILSLFGFSQISYGASDPFLSDRVAFPY
FFRTLKGFHGSSFALSKLLKHFGWTWVGIIRLDDNSGETELQVLTDYLSRGGICVEFTMKL
IYYRYRISYDQVYINWITATIQKSTTNIIVICGKLSLTVLKLLVQLRAELAEKTLILSPSASVM
GTISNQIVAIFDGSLMFEPYPVYPGDTHEIIEFINSIHPSKDPEDKLLEDFLLVRFMCSTKDQ
YKNQFYGNVYGTYNTECSDRDITEALGRLYKTLLAALSPNVHLAVNIMSQAIHEMHTSLR
EQSPERDREAHRYQYQLHQYLKKNRYQTKYGGEVSFDGRGEMDTGYIIYFYLSSYETAYL
GTFGRFIPLATSDYELEIHSVVIPWKMKNKTIPRAQCAESCQPGYRKALNPGIQPCCYGCL
PCSEGEISNRTDSENCLRCPDIEWPNENKNQCLAKTEEFLSFYNDTISVFLLSMPILFFFITLL
ILGIFVIYRDSPIVRANNRSLSFLLLVSIKLSFLSVFLFLGRPVDITCMLRIITFGITFSIAVSSLL
AKTIMVCVAFKATKPGSSWRKWLGVKLSNSVVLFCSSIQIIICMTWLAISPPFQELDIHTSP

GTIIIQCNEGSAIGFYSVIGYMGLLAAVTIGFYSVIGYMGLLAAVSFVLAFLARSLPDSFNEA
KYITFSMLLFCSVWITMIPAYLSTKGKNTVCVEIFAILTSSAGLLACIFLPKCYIIMFRPEMN
HKSCLLGSKT*
>jgi|Xentr4|399483|e_gw1.729.81.1
MLMYLLVLCIGPCRSRIQPINPACCLKTAEIYEDFEYFQDRDLIIGGLLTVNTYATYEDLPW
EKSHKVLCVNLQPRYYRQLVEFRLAIEEINKNPSLLPNVTLGYHIYDSCGHALKAAMSILQ
ILSGTKEPVPNYSCGRKRNIAGFIGDLTSETTMISAQILSLFGFSQISYGASDPFLSDRVAFPY
FFRTLKGFHGSSFALSKLLKHFGWTWVGIIRLDDNSGETELQVLTDYLSRGGICVEFTIKLI
YSRYKMSYDQVYINRIKATVQKSTTNIIVICGKLSLKIIKLLVQLGAELVEKTLILSPSASVM
GTTSNQIIAIFDGSLIFEQYPVYPGDTHEIIEFINSIHPSKDPEDKLLEDFLLVRFKCSTKDQY
KNQLYGNMYGTYNTECSDRDMTKALGRLNQTLLVALSPNVHLAVNIMSQAIHEMHTSLR
EQSPERDREAHRYQYQLHHYLKKNHYQTKYGGEVSFDGRGEMDTGYIIYFFLYSYGECY
WDTFGRFIPLAPSGYELEVHPALIPWKMKNKTTPRAQCTESCQPGYRKALNPGVQPCCYV
CVRCSEGEISNQTDAEKIAKNTKKSQNVRFQSEFFQFGFEIVSSVLFSKSEIFFSILCILDLAE
EALSSREEILSSISNSLSFLSVFLFLGRPVDITCMLRIITFGITFSIAVSSLLAKTIMVCVAFKAT
KPGSSWRKWLGVKLSNSVVLFCSSIQIIICMTWLAISPPFQELDIHTSPGTIIIQCNEGSAIGF
YSVIGYMGLLAAVSFVLAFLARSLPDSFNEAKYITFSMLLFCSVWITMIPAYLSTKGKNTV
CVEIFAILTSSAGLLACIFLPKCYIIMFRPEMNHKSCLLGIKT*
>jgi|Xentr4|399511|e_gw1.729.80.1
MLMYLLVLCIGPCRSRLQPINPACCLKTAELYEDFEYFQDRDLIIGGLLTVNTYATYEDLPW
EKNHKVMCSKLQPRYYRQLVEFRLAIEEINKNPSLLPNVTLGYHIYDSCGHALKTVRSTL
QMLSGTKDFVPNYSCGRKRNIAGFIGDLTSETTIISAQILSLFGFSQISYGASDPFLSDRVAFP
YFFRTLKGFHGSSFALSKLLKHFGWTWVGIIRLDDNSGETELQVLTDYLSRGGICVEFTMK
LIYYRYKISHDQVYINRIKDTIQKSTTNIIVICGKLSLNVLELLVQLRAELVEKTLILSPPASI
MGTTVNQIVATFDGSLMFEQYPVYPRDTHEIIEFINSIHPSKDPEDKLLEDFLLVRFKCSTK
DQYKNQLYGYFYSTYNTECSHRDITKALGLLNQTLLAALSPNVHLAVNIMSQAIHEMHTS
LREQSPERDREAHRYQYQLHHYLKKNLYQTKYGGEVSFDGRGEMDTGYIIYFYLYLSSYE
TAYLGKFASFTPMTTLNYKLEIHPVIIPWKMKNKMIPRAQCAESCQPGYRKALNSGAQPC
CYDCVPCSEGEISNRTEKLCGFSTERISKCREVGLFILAEAGIIGEQSRFVAADSDRDLIFWH
LLFLFSCSSTPSPLHSFHVVKGHPCVLKSNVTDALFNLAGHSLFLSVFLFLGRPVDITCMLR
IITFGITFSIAVSSLLAKTIMVCVAFKATKPGSSWRKWLGVKMSNSVVLFCSSIQIIICMTWL
AISPPFQELDIHTSPGTIIIQCNEGSAIGFYSVIGYMGLLAAVSFVLAFLARSLPDSFNEAKYI
TFSMLLFCSVWITMIPAYLSTKGKNTVCVEIFAILTSSAGLLACIFLPKCYIIMFRPEMNQKS
CLLGSKT*
>jgi|Xentr4|399586|e_gw1.730.176.1
SYPHYLALKFAIEEINRRADILPNITLGYMVYNTRAIERRAMRGVMSVLSGGKEFIPNYNC
ERRGILAGFIGDLTSASSYLISLLTGLYHYPQISYGATDAIFTDRWRFPYVYRMVPNEGYLY
KALIQLLKHFGWSWVGIFFTDIRGDYKKRDLTELITSNGICVEFAYELDWKELKRESCWAC
VIQRTTARVIIVSVPREQITLDLASIHANSRFRRIWILIVTSYKDLVFHYDFTEIFNGSLAFLIS
GDEIPGFEDYFLRANPARYPEDPYIYSMWLSCFICITPQMKKEALTMSDAPRLQDYMLKD
CLGNETLASIPHYRQLDLRVPYSVYKAVYLLAQALHVLLSEKTSHGAPTEKARLERELNP
WKLNHLLRNIHFTSSSSQEEIYINEDGEAPGQYDLTNWVVPPDPREGQVNVIKVGSFSTLT
PDQLIVNDSAILWHPSFTEIPISVCSDSCIPGYRKNSREVEFSCCYDCVPCAEGHISNTTDME
TCIQCPEDQWPNDKRTICIQKITEFLSYEDPLGQSLAAVSALSSLTVILVFLIFLKYHKTPVV

KANNQTLSYSLLLSLTLSFLCCFLFIGRPQKVTCLLRQVTFGINFTLSVSCVLAKTVTVAIAF
NATKPGSKIKKWVGTRVSLCLVLLCSLLQVGICLVWLISSPPFPDYDTHTYTGKMILQCNE
GSVTAFYTVIGYLGFLSGLSFIVAFLVRKLPASFNEAQLITFSMLVFCSVWVSFIPAYLSTKG
KYMVAVEIFAILASSAGLLGCIFIPKCYIILFRPEQNTRRGLTGKHLQ*
>jgi|Xentr4|399958|e_gw1.735.3.1
MEHYSPESMVTLIVMALTFVVGVPGNILVIWVTGIKMRRTVNTVWFCNLAVADLICCLSL
PFSMAHEILHNAWPNDSISCKVLPSIIILNMFASVFTLVAISVDRYILVVKPVWAQNHRSVPL
AYLLCLVIWLVSLTLCLPVFMYRTITYYNNHTYCGYTYKQVEGDFEYSGLEEDYDFGNYT
YNNFAHDNSDVEPSGHETSVAITITRVIFGFFLPLLVILFCYIRLSWKVQSGRFANVGRKTR
KVVIAIVLAFCMLWTPYHVIGIALEYISSSTLTRLDHLSQALAYSNSCINPIIYVFMGKDFKS
KMSKSIRGLLQSALTEEITRTTGGSKSRESARYSSML*
>jgi|Xentr4|400770|e_gw1.745.2.1
MLRDYIFFCYIFCNLFFRISLQYYRHYLAFIFAVEEINRSSWILPNITLGYQIFDSCGSTTKSL
SGALSAISGKQQNVPNFSCWGNSKVVGFVGDLSADTTYTIAQLVGVLSYPQISYGAKDPV
FNDRTQFPSFYRTIPNEEAEMDGIVQILKHFGWKWVGLIVSSDGTGYRARERISKELASSG
GCLAFTAFIEFPYDFPKEITKTTVIVMLISLETNLLYLSSLYDLPPKLWITSSFIYNIIMLNQQK
TKFTFNGSLSLLIQEGEIPGFKQFFYTFSPNNYPNNALIAYTWQSLFVCAFIDIPMSVTKETC
PGNATFSEADVLVYGNHHYRVTYRVYTAVYALARALHNLYSAQPPANHWGKLESLKRNI
KPWQINQFVRNVTFTTFPNDTHSFNKYGDPPAQYNIIKLLFLPGDRSVITKVGYFNVSDHG
TQFYINNSADLWGPHFNMVRTPQSLCNEPCAPGYRKSKIEGAPSCCHDCVPCIDGEMSNT
SDSSTCFRCPGYEMSNKQRTACVPKMINYLSYEETLGASLASIALVLFLTTSAVLGIFVKY
WETPIVRANNRYLSCLLLISLMLCFLCTLLFIGRPTQICCLLRQVTFGIVFTISVSSVLAKTLT
VIIAFNATKPGSKLKKCIGSQVPIVFVTICSLGKIGICIVWMAFDPPFLKADMFSERNTIILQC
NEGSVTFFFCIIGYIGTLALLSFIAAFLAKDFPDRFNEAKNITFSMLGFCSVWGAFVPAYLSS
KGSRMVAVEIFAILSSSAGLLGCIFSPKCYIIFLRPELNTKVTACQIH*
>jgi|Xentr4|400787|e_gw1.745.14.1
MLLLAVLLYIFPAASDAHLPPGSACALRPPRHTTNVKPGDLVIAAFLQLNDVFIINKVDFVG
ESFIISCTRATFRYYRHYLAFIFAVEEINRSSWILPNITLGYQIFDSCAAVPKSLSGALGAISGK
QQHVPNFSCWGKSKMVGFVGDLSTDTTYAIAQLVGVLSYPQISYGAMDPVFNDRTQFPSF
YRTIPNEEAEMDGIVQILKHFGWKWVGLIISDDDTGYRGRERISKGLFSNGGCLAFTVVLQ
DTNKIGVFHENMIVGEIEKTTANVIVLFISTKFTYSLAHLFRFHKMPPKLWITSSFITNIMTF
KQEEMETTLTGSLSLMIQEGEIPGFEQFFYTFSPNNYPNDALIVSTWQKLFDCYFIDSPSFI
WGIPGTVVKTCTGNETMSAADVSVYGNQNYRVTYRVYTAVYALARALHNLYSAQPPAN
HWGKLESLQKEFKPWQLNKFVRNVTFTTFSGNTHSFNKYGDPPVRFDIIKWFFFPRHHITT
KVGTFNKSDNKTQLYINISADLWGPYFIMTPQSLCNEPCAPGYRKAKREGAPSCCYDCVW
CVDGEISNTSDAPRCFKCSEYQMSNIRRTGCISKTVNFLSYKDTLGASLASIALVLFLTTSA
VQGIFVKYWETPIVRANNQNLSCLLLISLMLCFLCTLLFIGRPTQICCLLRQVTFGIVFTISV
SSVLAKTLTVIIAFNATKPGSKLKKYVGTQLAILLVLMCTLGKIGISVVWMASNPPFLEAD
TFSEMDTVILLCNEGSVTFFFCIIGYIGTLALLSFIAAFLAKDFPDRFNEAKNITFSMLGFCS
VWGAFVPAYLSSKGSRMVAVEIFAILASSAGLLACIFVPKCFIIFFRPKLNKRANVLK*
>jgi|Xentr4|400791|e_gw1.745.99.1
METANQTIIQEFIFIGLSRNPTTKTLLFALFFPMYIFTIFGNGILIFIIVKSSNIHTPMYFFLCNL
AILDTVFSTCTVPKVLVDLLLAEGRISYTGCMIQMCVGLFLGQTECLLLAVMACDRFVAIC
NPLRYAVIMSWRSCKYIMAVTWLLSFFSSILPILSKPVSFCRENKLNHVACEILAVVKLACG

DMSYYENGIAFQSLFTILVPFSFIVVSYICILTSVLQIRSVEGRTKAFSTCASHLTVVIMFYGL
SMTMYLKPSSNFSSKQKYISIFYGFLTPMLNPLIYSFRNDEVKKAVRRILFSSSQIK*
>jgi|Xentr4|400805|e_gw1.745.25.1
CLYCPFSYLMTTTKCFPDASSCFKCPAFQRSNKKRDGCVPKYVKFLSHEEPLGTSLASAAL
ILSLMCVVILGIFIKYRETPIVRANNRYLSCLLLISLMLSFLCTLLFIGCPTQTCCLLRQVTLG
IVFTTSVSTVLAKTLTVIIAFNATKPGSKLKKYVGSQLAIILVIVCSLGETVISAVWMASNPP
FIDADDVSNMDSVILLCNEGSIIYFFSIVGYMGASALLSFIAAFLAKDFPDRFNEAKNITFS
MLIFCSVWGAFVAAYLSSTGTKVVAVEIFAILSSAAGLLGCVFFPKR*
>jgi|Xentr4|400858|e_gw1.745.117.1
YRHYLAFIFAVEEINRSSWILPNITLGYQIFDSCAAGHKSLSGAMGAISGKQQNVPNFSCW
GNSKMVGFVGDLSTSSTYTIAQLVGVLSYPQACISYGATDPDFNDRTHFPSFYRTIPNEEAE
MDGIVLKHFGWKWVGLIISNDETGYRARERISTELTNMGSCVAFTSVITQNSDIVHENENR
IAVEIEKTTANVIVLFISTKFTYGFAHLFSLHKIPPKLWITSSFIPSILTFKQEKMETTLNGSLS
LMIQEGEIPGFEQFFYTFSPNNYPNDALIVSTWQNLFKCYFIDNPSSIRKIPETLQNHIFSEST
SSDKTKRGKYIFLLQTTKLQSYAKHNGFYEISENLQKACILPHYLNQFVRNVTFTTFPNDT
HSFNKYGDPPARFDIIKWLFSPGHHIVTRKIGGFNVSDHEAQFYINHSADLWGPLFNMIPQ
SLCNAPCAPGHRKSKREGAPSCCYDCVPCVDGEMSNTSDSPKCFRCPGYEMSNKQRTAC
VPKMINYLSYGETLGASLASIALVLFLTTSAVQGVFVKYWETPIVRANNQNLSCLLLISLM
LCFLCTLLFIGRPTQICCLLRQVTFGIVFTISVSSVLAKTLTVIIAFNATKPGSKLKKYVGTQL
AIVLVIICSLGTIGISTVWMASNPPFLEADMFSEMDTIILKCNEGSVTFFFCIIGYIGTLALLSF
IAAFLAKDFPDRFNEAKNITFSMLGFCSVWGGICPCIPEQ*GQ*NGGS*DICHLILQCWVIG
LYICPQVLHYISQA*VEHKSSCCSKLL
>jgi|Xentr4|400873|e_gw1.745.98.1
LMYILTLFGNGILIYIIARSSNIHTPMYYFLCNLAFLYTVFSSSTVPKMLVDLLLAEGRISYT
GCMIQMCVGLFLGQTECLLLAVMACDRFVAICNQLRYAVIMSWERCKRITAGTWLLSFLS
SILPVLSKPMLFCGENQLNHYACEILALVKLACGDMSYYENIIAIQSSFTILVTFSFIVVSYIC
ILTSILHIPSVEGRTKAFSTCAAHLTVVSMFYGPSMIMYLGPSSNFSSNQEKYLAVFYSILTP
VLNPLIYSLRNDEVKKAVRRILISSSQIP*
>jgi|Xentr4|400875|e_gw1.745.4.1
FAVEEINRSSWILPNITLGYQIFDSCGSAKKSLSGALGAISGKQQNVPNFSCWGNSKVVGF
VGDLSTGTSYSIAQLVGVFNYPQISYGAMDPVFNDRTQFPSFYRTIPNEEAEMDGIVQILK
HFGWKWVGLIISDDATGYRARERISKELVSRGGCLAFSALITFPFDFPKEIKKTTVIVLFISR
ETNLVYLSSLYDLPPKLWITTSFIYNVIMLNEYTWHLLFVCPFIDIPMSVTKETCPGNATFSE
ADVSVYGDHNYRVTYRVYTAVYALARALHNLYSAQPPANHWGKLESLKHKINQFVRNVT
FTTFPNDTHSFDKYGDPPAQFNIIKLLFLPGDRSVITKVGNFNVSDHGTQFYINNSADLWG
PHFNMTPELLCNAPCAPGYRKSKIEGAPSCCYDCVPCVDGEISNTSDSPSCFRCPGYQMSN
KQRTACVPKMINYLSYEETLGASLASIALMLFLTTSAVQGVFVRYWETPIVRANNQNLSCL
LLISLMLCFLCTLLFIGRPTQICCLLRQVTFGIVFTISVSSVLAKTLTVIIAFNATKPGSKLKK
YVGTQLAIVFVTICSLGEIGICIVWITFDPPFLETDIFSERDTIILQCNEGSVTFFFCIIGYIGTL
ALLSFIAAFLAKDFPDRFNEAKNITFSMLGFCSVWGAFVPAYLSSKGSRMVAVEIFAILASS
AGLLGCIFAPKCYIIFLRPQLNTKVTAYKIH*
>jgi|Xentr4|400892|e_gw1.745.80.1
MSLYITIQTPELLCNAPCSPGHRKSKREGAPSCCYDCVPCVDGEMSNTSDSSTCLKCPGYE
MSNKQRTACVPKMINYLSYEETLGASLASIALVLFLTTSAVLGIFVKYWETPIVRANNQNL

SCLLLISLMLCFLCTLLFIGRPTQICCLLRQVTFGIVFTISVSSVLAKTLTVIIAFNATKPGSKL
KKCIGSQVPIVFVTICSLGKIGICLIWMMFHPPFLEVDILSERDTIILQCNEGSVTFFFCIIGYI
GTLALLSFIAAFLAKDFPDRFNEAKNITFSMLGFCSVWGAFVPAYLSSKGSRMVAVEIFAIL
FSSAGLLGCIFIPKCYIIFLRPELNTRVAVNQNH*
>jgi|Xentr4|400900|e_gw1.745.7.1
ISYGAMDPILNDRIQFPSFYRTIPDEESEIDGIVEILKHFGWKWVGLIISDDDTGYRAQERIS
KELSRIGGCLAFSAVLKHNSYIRISHENKIVQELEKTTANVIVLFVSTKYTFGFSHFFSNYKL
PTKLWIASSFFPNIMRFIESGNGEMETTLNGSLLLLVKEGEIPGFDKFFHRFSPNNYPNNVF
VTSLWEELFDCSFSESPVYKREGETINLCIGNENFSDVDVSVYGNHNYRVTYRVYTAVYAL
ARALHNLYSAQPPANHWGRLETLKVKPWQLNKFVYDVTTLLGDADSFNEHGDPLGNFDI
IKWFFLPNHYVARPKVGNIHASSNKTSIFINSSSDIWGPYFQTTPQSLCNEPCPPGYRKSKR
QGAPSCCYDCVHCADGEMSNTTDAPSCFKCSEYQMSNKQRTVCIPKSVSYLSFEDTLGA
CLASIALVLFLTASVVQGTFIKYWETPIVRANNQNLSCLLLISLMLCFLCTLLFIGRPTQVCC
LLRQVTFGIVFTISVSSVLAKTLTVIIAFNATKPGSKLKKYVGTQLAIVLVTICSLGKIGISVV
WMVSNPPFLEADLFSEMDTVILLCNEGSVTFFFCIIGYIGTLALLSFIAAFLAKDFPDRFNE
AKNITFSMLGFCSVWGAFVPAYLSSKGSRMVAVEIFAILSSSAGLLGCIFLPKCFIIFFRPEIN
TRETVVLKW*
>jgi|Xentr4|401314|e_gw1.754.105.1
MIHFTCKWSLTRGLSSDQLRVILCLAAIWVTPCSTELSGSDSPCRIPLTKPKYEYKYIQDGD
IIIGGVFSVNTGVVHISDDTGKHIPLCINPYHEYYTEIQTFLYGIDEINRNLDLLPNITLGYRV
YDSCGDPRLAIGSALQILSGPGNVVPNYSCRGKGEIAGFIGDQSSLTSLPIAQLLGVYGYSQ
ISYGATDPVLNDRTLYPHYLSTGPNEYIQHIAIAELVERLGWTWVIILATSNDGGQKESQNL
KNEINKHGACVDLIGTLTGNNDTDKRTLERIQKSTAEVVILCGGRSYNPYFVFILKEIINNK
MVVVPVTCVFIPNDFLYNGCLQFQDTNMMSDESLEVKFTEHIYAPREDELLKDLLANDHL
CLTHDKEKDDLFQRVYKLLYRNCSNITSPMLYYYPSHRVSTAVSVLARAQHNLLSSSGKHS
NSGLPTIIHRKQLHRYLRNVLLNEQRELDYGEAYLIHSLYKDSELKGQEIHVGEYTWSESG
SSLRINTEEIVWKKDTKGQILKSQCSTNCPPGYRKVPREGAPPCCYDCAPCSEGEISNLTD
MDNCLKCGDYKWPNPEKTVCIEKQLQFLSFDDPLTLIFIISSVILFVIAAVILGIFISFRDTPV
VRANNHTLSFILLVSIKLSFLSVFLFLGRPVDITCMLRQTSFGITFSIAVSCVLAKTLMVCFA
FKATKPGSPWRKWVGVKVAYCIVLSCSIIQILISVIWLTISPPFLELNFLFEPGQIIIQCNEGSV
VAFYIVLSYMGLLASVSFIVAFLARSLPDSFNEAKYITFSMLLFCSVWITMIPAYLSTKGKY
MVAVEIFAIISSSCGLLFCIFLPKCYIILFKPEMNSKQYLLGNNR*
>jgi|Xentr4|401372|e_gw1.754.103.1
MLGHLVLPNPITIQIYMIVLCVGPCRSGIQPLSPACHLEIIKAVEEYEYIQEGDIMIGGVMTA
HLNIINVTLPMENIDKLLCITGSTISNCQQNFRHFIDFRFAVEQTNKDPARLPNLTLGYHISD
SCGDPRKAVRSVLQILSGTREPVPNYSCVGKRNIAGFIGDLTSETTVPIAQILSIYGLSQISY
GATDQSLRDRVAFPYFFRTVQSDEGSYFALGMLLRHFGWTWVGIITSDDISGEKEHQHLIQ
YLHSEGICVEFIIRMNFFDFTDSSNHRNFNLIDKSLTSIILLCGTVSINFLVKLLHRESLIEKTL
ILSTNWGTNDISLSYDQNIFNCSLGFVPGYHYHLDTPEFRNFLENLHPSNFPEDKLLENIW
MIFHLCLSEDSKKNSLYKYIYLQNLHNCTGQERITDNHYFSADLSSPRVHLAVDLMSQAIH
DMNIRLSEKPLKNREKYHYVNQLHHHLKALMYNSSHDTTFFLDENGEYITSYWIYNYILT
PDELTRNFIGEFSPWTPTDQQLTISLNSIAWKGNTIPRAQCSDSCLPGFRKAPMPGAQSCCY
GCVPCSEGEISNITDSIRCFKCPDMEWPNEKNIQCIASKEDFLSYTSDKISLFISPIVIFFLFITL
LILRVFVMHWDSPIVRANNRSLSFLLLVSIKLSFLSVFLFLGRPVDITCMLRIITFGITFSIAVS

SLLAKTIMVCVAFKATKPGSSWRKWLGVKLSNSVVLFCSSIQIIICMTWLAISPPFQELDIH
TSPGTIIIQCNEGSAIGFYSVIGYMGLLAAVSFVLAFLARSLPDSFNEAKYITFSMLLFCSVW
ITMIPAYLSTKGKNTVCVEIFAILTSSAGLLASIFLPKCYTI
>jgi|Xentr4|401415|e_gw1.754.106.1
MLGDIPAIKILIYLIALCLRPHNSEISVPKPDCHLEMIKTEEEYEYIGEGDIIIGGLVTVSMPYS
SDYFTGLMCIHASPQSYRDLVDMLYEIEENHPSYPPNITFGYHIYDTCGDPQRAIKSTLQIL
SGSRVPVPNYSFREMENIAGFIGDFASETTIPIAHILSVLGYTQISYGATDPALSDTFTFPYFF
RTTESDDRNFFIISKIVTYFGWNWVGIIRFDDDRGDRDQQLLEYYLSRESICIEFTLKITSKS
NGNIQYKEIIKKSSANVIIFCGPVNVKLATEFNYLWDLLREKTSIFPPNWLYYNHIFNFLHE
KFHGSLLLVQEQLKNRNDGKYKQFSERFHPSKHPDDKLLENMWIEHLGCLSDNQRKNQF
YERELRLNLRNCSGEESLSRIHIYERGGFHNANMLRAVHYLFFMLDNMHYFLNVQGSGTT
RKVYNYRYKLHQYMEINHYGSRINSKMLNENLSYYLLSVHSKFVKQSYYMKFGFSYAW
DLEIYPDSIKWNNKDNKVPRSQCSDNCPPGFRKAPRPGAQSCCYDCVPCSEGEISNITDSE
FCIRCPYSEWPNERRNQCIAKAEAFLSYIDVISMFFSAVSVLSFFISLLLILGVFISYRDSPIVR
ANNRSLSFLLLVSIKLSFLSVFLFLGRPVDITCMLRIITFGITFSIAVSSLLAKTIMVCVAFKAT
KPGSSWRKWLGVKLSNSVVLFCSSIQIIICMTWLAISPPFQELDIHTSPGTIIIQCNEGSAIGF
YSVIGYMGLLAAVSFVLAFLARSLPDSFNEAKYITFSMLLFCSVWITMIPAYLSTKGKNTV
CVEIFAILTSSAGLLFCIFLPKCYVIMFRPEINSKSKLLGNRSF*
>jgi|Xentr4|401426|e_gw1.754.4.1
FHYVIEQMNNSTTQFPNLTLGYHIYDPCGDARMAVKSVFQILSGTREPVPNYSCVGKRNIA
GFIGDLSSKTTLPIAQILSIYGYSQISYGATDTSLSDRLTFPYFFRTVQSDQKNYLMLVRLIQ
YFGWTWVGFITLDDISGFYEHQTFSQYLSSQGICMEFAIYLNFDTSNYDYIPRYRKIIADSS
SSVIIVGGTVSTTILSTITAVGDVLANKNLIFSPQWETYLLLPFDSAASINNSLIFSSLYPYNL
DTPEINYYYEHLHPLKYPEVEFLEYIWFLIFYCFSRDLNKTITFGKYYLDNFTFPNCTGEER
MTNLQTFKLYRNSPRVHFPVTTMVRAIEALHVAHNQEPTEKPISPIIYRHQLQHYMKKMPL
FMEYSQFYEFDEYGNLEIDYGIFNYIIAASIETGELEVTSRPVGKIRPRAPSDQQLEINKHLI
MWNSDGLEIPRSQCSDNCLPGFRTATKATIQSCCFRCVSCAEGEISNTTDSESCFPCKNTEW
PNKKRTQCIAKAEAFLSYRDVISMFFSAVSVLSFFITLLLILGVFISYRDSPIVRANNRSLSFLL
LVSIKLSFLSVFLFLGRPVDITCMLRIITFGITFSIAVSSLLAKTIMVCVAFKATKPGSSWRKW
LGVKLSNSVVLFCSSIQIIICMTWLAISPPFQELDIHTSPGTIIIQCNEGSAIGFYSVIGYMGLL
AAVSFVLAFLARSLPDSFNEAKYITFSMLLFCSVWITMIPAYLSTKGKNTVCVEIFAILTSSA
GLLACIFLPKCYIIMFKPEMNTKKNLF*
>jgi|Xentr4|403043|e_gw1.778.62.1
MAVSNSLKELIFLIMLCVGPCRSGVLTANPACHLEIIKTYEEYEYIKEGDIMIGGMMTAGM
AIEPDHKNRFFYCNEPSPQKYRYLVEFLFLVKEINENPARLPNKTLGYHIHDSCGSPQRAL
MSLFKILSGTREPVPNYSCVQKRNIVGFIGDLYSEPTVSVALILGVLGYPQISYGATDPSLSD
RVAFPYVYRTVQSDEEEYIALCKLLRYFGWNWVGIIQFDDYVGYQNHQLLVKYLSREGIC
VAFTIKLAINLKENVIKSITIEKSSTDIIIICGNVGEFHFKPLYDIHHSVSKKTFIFLSKWLNHL
DALKGVFLQYFGGSLLFAQKGIRDQFDPRFKEFSDSFHPSKYPEDNILEDIWLWQFSCLSK
HKSKNEYHEFMFHYLYNCSSGKEKLTDLPSYLNLYHSPSLMYAVEMMSLALTHMHNSLP
KQSFQNNKMYKFQLHHHLKSISYTRNGSPALSFNEKGELVNQYEIVNLQFDPSGNWSWNI
VGNYVPWALPDQRLMVTPEKIIWKTPRNKVPRAQCSSSCPPGFWKAIMAGILTCCYECVQ
CPEGEISNRTDSEICIPCPKMEWSNKKRIQCIAKMEDFLVYTNVFSVFFSASSVLFFLTTLLIL
EVFIAHRDTPIVRANNRSLSFLLLVSIKLSFLSVFLFLGRPVDITCMLRIITFGITFSIAVSSLL

AKTIMVCVAFKATKPGSSWRKWLGVKLSNSVVLFCSSIQIIICMTWLAISPPFQELDIHTSP
GTIIIQCNEGSAIGFYSVIGYMGLLAAVSFVLAFLARSLPDSFNEAKYITFSMLLFCSVWITM
IPAYLSTKGKNTVCVEIFAILTSSAGLLACIFLPKCYVILFRTEMNTKSLLGNRQYQY*
>jgi|Xentr4|403056|e_gw1.778.58.1
MLGVTAVCYSVKVLIYLIFLCGGPYRFGVQPLNLACNLQIIKPVKEYEYIQEGDIVIAGVM
ASHFYMINVTFPWDNSSRFLCTLPNEQAYRYLVDFRFAIEQINKDPTRLPNLTLGYHIYDSC
GDPRKAVRSVLQILSGTREPVPNYSCVGKRHIAGFIGDLTSETTVPIAQILTLYGYSQISYGA
TDISLRDRAAFPYFFRTVQSDEANYFALSTFLRHFGWTWVGIITSDDISGEKEHRSLSKYLN
MEGICIAYTIKMNSNNKYAIKSKQYKSTVEGSSTSVIILIGTVSTLFVTELPIENPLFQEKTLI
LSPNWGNNDFVIAYGMEIFNLSLVFVPHYHYDLGTPEMRRFLENLHPSKLPHDKLTEDIFM
IFHLCLSKDQNKNDLYQYIYLNTLRNCTGQERITDLYYFSGRTNSPPVHLAVDIMSQALHE
MLLINKSMNYSYKYQLHHFLKKHQYSTQIGPVFLFNEYGEYISGLWIYNYILAADRHQDK
QLFGEFSPWRPSHQQLNVTLSLIKWKSNDNEIPRAQCSDNCLPGFRKATKRGAQSCCYDC
ILCSEGEISNTSDSESCIQCPDMEWPNEQKDQCIAKMEEFLSFGDDVISAIFLFISVLLFLITG
GIMRIFIIYRDSPIVRANNRSLSFLLLVSIKLSFLSVFLFLGRPVDITCMLRIITFGITFSIAVSSL
LAKTIMVCVAFKATKPGSSWRKWLGVKLSNSVVLFCSSIQIIICMTWLAISPPFQELDIHTS
PGTIIIQCNEGSAIGFYSVIGYMGLLAAVKFVLAF*LGAYRTVLMKASTSLSACLSSAVFGS
Q*SRLSEHQRQNTVCVEIFAILTSSAGLLACIFLPKCYIILLRPEINLKTSLLRNQ*
>jgi|Xentr4|403068|e_gw1.778.61.1
MCVGPCKPACHLEIIKSAEEYEYTKEGDIIIGGVLTVSMFQPEDYFTGLTCIYPSAPNYKYL
VDFLYVIDAHNKNPHILPNATLGYRIYDSCGDPRKAIKHLLQILSGSREPVPNYSCTGVGQT
AGVIGDLISETTIPIAQILTVFGYTQISYGATDPVLSNRINFPYFFRPTGNDYSYYLVISKIAK
YFHWDWVGIITFDDDRGESDHQLLKQYLSSENICIEFTLKITNIMHRDMIARKTLKKSTTD
VVILCGAVNLQIALHFDFLSDMLSEKTFIFTSNWLHYNHILAFSHRLFHCSLLLMQNKEQY
PKNSPYGPFSKQFHPSRYPNDKLLENIWMHYHSCLSKNRQKNEAFEKALNRRLHNCSGQ
ESLSTIDMFNNGFHTLNMVLAVDMLVTAVHYVYNSLGNGISWGNRKMFNYRNKVHHYL
KNMFIRYGPDQVLSLNEHGEFVSIYLIMNLYRKSAEQTDWKIFGTYTPWKPEHLKLEIAQE
LIQWKNKDNQVPRAQCSDNCPHGFRKTPKPRAQSCCYDCILCSEGEISNTTDSEKCIRCPY
NEWPNEKRNQCIAKTEEFLSYTTDVISVCFSVISVLFLLITVMILGVFIAHRDTPIVRANNRS
LSFLLLVSIKLSFLSVFLFLGRPVDITCMLRIITFGITFSIAVSSLLAKTIMVCVAFKATKPGSS
WRKWLGVKLSNSVVLFCSSIQIIICMTWLAISPPFQELDIHTSPGTIIIQCNEGSAIGFYSVIG
YMGLLAAVSFVLAFLARSLPDSFNEAKYITFSMLLFCSVWITMIPAYLSTKGKNTVCVEIFA
ILTSSAGLLACIFLPKCYIIMFRTEMNKKSHLLVMKS*
>jgi|Xentr4|403080|e_gw1.779.92.1
MLGVTAVCYSVKVLIYLTFMYVGPYGCWNQPINPACHLQTINVVKEYEYMQEGDIIIGGV
MASHFYMFNFTFPWDKSTGFSCICPNQQAYRYLVDFRFAIEQINKDPARLPNLTLGYHISDS
CGDPRKAVRSVLQILSGTREPVPNYSCVGKRNIAGFIGDLTSETTVPIAQILTLYGYSQISYG
ATDPSLRDRAAFPYFFRTVQSDEANYFAISKLLSHFGWTWVGIITSDDMSGEEEHHSLAKY
LSREGVCIEFTIKINKVNDYYIKQYESTIEKTSTSVIIISGTASIMFVVALPLVVSKKTLIVSSN
WGITDIVLGYDRKSFNNSLVFLPRYHYDLGTPEMSRFLEDLHPSKFPEDKLIDDIFLLFHLC
LSKDQNKNNLYKYTSLCTLRNCTGQERITDLYYFSGGTNSPLVHLAVDIMSQALHDMNM
LFIMKSNQHPYKYQLCHFLKKHQYSPQTGPTSSFDEHGEYMSGLWIYYYVFMPNLKRKL
VGKFSPWAPPDQQLNITSSEIKWKTNNNKIPRAQCSDNCPPGYRKAPNPRAQSCCYVCVP
CSEGEISNTTDSVSCIPCPDMEWPNENKIQCFARMEEFLSYSVDFISLFFSFFSLLFFLVTEVI

LGIFLKYRDTPIVRANNRSLSFLLLVSIKLSFLSVFLFLGRPVDITCMLRIITFGITFSIAVSSLL
AKTIMVCVAFKATKPGSSWRKWLGVKLSNSVVLFCSSIQIIICMTWLAISPPFQELDIHTSP
GTIIIQCNEGSAIGFYSVIGYMGLLAAVSFVLAFLARSLPDSFNEAKYITFSMLLFCSVWITM
IPAYLSTKGKNTVCVEIFAILTSSAGLLACIFLPKCYIILFRPEINTKSHLFENKY*
>jgi|Xentr4|403099|e_gw1.779.102.1
MLGVTAVCYSLKVLIYLTFMYVGPCGCMNQPPNPACNLQIFKAVKEYEYMQEGDIIIGGV
MASHFYMFNFTFPWDNSTGFACLTPNQQAYRYLVNFRFAIEQINKDSSRLPNLTLGYHISD
SCGDPRKAVRSVLQILSGTREPVPNYSCVGKRNIAGFIGDLISKTTVPIAQILSVLRYTQISY
GATDPSLRDRVAFPYFFRTIQSDEANYFALSKLLRHFGWNWVGIITSDDMSGEEEIHSLAK
YLSREGVCIEFTAKAKISTENDFSFSQYESIIGRSSTSVFIISGTASIMFVNALPYALRQKTLII
SPNWGNNDDVLGYNIMIFNNSLVFLPRYHYDLGTPEMSRFLEDLHPSKFPDDKLIEDIFLL
FHLCLSKDQNKNNLYQYTSPHTLHNCTGQERITDLPYFRGETNSPLAHLAVDIMSQALHE
MNMLFIRKSNQYPDKYQLHYFLKNHQYSPQTGPTFSFNEHGEYMSGLWIYNYIISPNYYL
ERKLVGEFSPWAPPDQQLNITSSVIQWKTDNNDIPRAQCSDNCLPGYRKAPNPGAQSCCY
NCVLCSEGEISNTTDSESCIQCPDMEWPNEQKDQCIPKIQEFLSYTSSSLSPLKLSFLSVFLF
LGRPVDITCMLRIITFGITFSIAVSSLLAKTIMVCVAFKATKPGSSWRKWLGVKLSNSVVLF
CSSIQIIICMTWLAISPPFQELDIHTSPGTIIIQCNEGSAIGFYSVIGYMGLLAAVSFVLAFLAR
SLPDSFNEAKYITFSMLLFCSVWITMIPAYLSTKGKNTVCVEIFAILTSSAGLLACIFLPKCYI
ILFTPEINTKSHLLENKY*
>jgi|Xentr4|403123|e_gw1.779.98.1
MCVGPCRSEPTNPACGLQIIKAVEEYEYIQEGDIMIGGVVTVSMLLPKNYFTGLTCIFASAL
NYKYLVDFFHVMEEINKWNAFSNKTIGYRIYDSCGDPPRAVRSVLQILSGTTEPVPNYSCV
GKRNIAGFIGDLTSETTVPIAQILTVFGYSQISYGATDPALRDRSTFPYFFRTTASDDSYYLLI
SKIVRYFQWNWVGIIRSNDERGERDHQLLKYYLSSENVCIEFTLKITDLYNEIQKYREIIKN
SSTNVIIFCGTVNVLIVTNFRNLLKGDTFTEKTLILTTNWIYYQNEIGFSHEAFHGSLLLTQN
LFHFISDSPYTRFTEQFHPSRYPDDKLLENIWIEALSCLSKNPQKNEFYLSRNAQLHNCSGQ
ESLSQIPEYNHGFHTANMMFALNAFSNALQIHDSYYRMIKRKMDHYRYKIHHYLKKLYSL
SSLKEESELGFPYVIVNIYRNSEQQTAWKLCGTYMPNAPLHEQLNIILEQAQWKTKDNKV
PRAQCSDNCLPGFRKAPKPGAQSCCYDCVPCSEGEISNTTVKLSCFTIKFMKWQENSQNA
FVVWFFAACVFFVVHSYFFVALIYSFVAAAISWLPMHFSTTAPVFDVAVVHGSSNHFLVLY
KILALSLSSSLFTFFL*HFRICAKSLLKFHEFLMVFVKFSRKNRTFSWRKWLGVKLSNSVVL
FCSSIQIIICMTWLAISPPFQELDIHTSPGTIIIQCNEGSAIGFYSVIGYMGLLAAVSFVLAFLA
RSLPDSFNEAKYITFSMLLFCSVWITMIPAYLSTKGKNTVCVEIFAILTSSAGLLACIFLPKCF
IILFRTEMNRKSNLLVIKTG*
>jgi|Xentr4|403129|e_gw1.779.100.1
KYLLDFRYAIEQINNRTTRLPNLTLGYHIYDSCGNEKKAVRSVLQILSGTREPVPNYSCVGK
RNIAGFIGDAASDTTIPIAQILSVYGYSQISYGATEPSLSDRASFPYFFRTVQSDETNYSMLV
QLIKYFGWTRVGFITSGDISGYYEHQIFTQYLSNEGICIEFTIFLQEKKNYHDFVDQMKSFK
NSSSSVIIVGGTVSMSFPIEILNMYQEVAIAKINENVMRKNFIFSPHWETYLLLPFDLMGTV
NSSLLFVSLYPYNFNTPETHHYYENLQPLKYPDDKSLEEIWPLYFFCLSRDQTWNNLLNVS
RNCTGGESLVNLAIWKHYRHSPLVHFPVTIMIGAIEDMHAAHNKESPEKPKGPIIYRHRLH
HYLKKMTIDTQNGQFYKFDAYGNLGLDYEILNCITEFSAEDPEMTYRPVGKIRPKAPSDQ
QLEIYRDLITWNSNSSEIPRSQCSDNCLPGSRKATNSTVKTCCYKCVPCSEGEISNITDSEKC
FQCKETEWPNGKRTRCIAKVKEFLSYTDDVISMVFSAVSVLFFFITLLILGVFISYRDSPIVR

ANNRSLSFLLLVSIKLSFLSVFLFLGRPVDITCMLRIITFGITFSIAVSSLLAKTIMVCVAFKAT
KPGSSWRKWLGVKLSNSVVLFCSSIQIIICMTWLAISPPFQELDIHTSPGTIIIQCNEGSAIGF
YSVIGYMGLLAAVSFVLAFLARSLPDSFNEAKYITFSMLLFCSVWITMIPAYLSTKGKNTV
CVEIFAILTSSAGLLACIFLPKCYTIFFKPEMNTKKYLL*
>jgi|Xentr4|403131|e_gw1.779.93.1
MDKLKVLFCLAALWVTPCSTESDSPCRIPLTKPKYEYKYIQDGDIIIGGIFTVNTAVDYIPDT
TEKYIPLCVSAVITEILLIYENSFVVSTTYYSWFSFLGKFLLLLAKMLEDSCSAPRFAVGSAL
QILSGPGKAVPNYSCRGRGEIAGFIGNLYSASSIPLAQLLGNYGYTMISSAATDPALSDPELY
PYFFSTVPSDHIKHIAIAMLIERLGWTWVIILVADDDSGELVGQNLQSEIIKHGACVDFMAT
LRESEFKKTRIFERIKNSKAEVIVLCGTPSSFFLALLYDKSRLIAEKTLVVPMPWLSAHYIT
MPLSHGSLVFRYPTEHNKGQWRQFPEYLLSITEDELLTDLLELQFMCRRHDEERNRLLDK
AYGKFYGNCTGKESLTKIKKSYEPSIEVYRSVYVMALALHNMVSLHRLNGSKDIPARIYR
KKLHHFLRNVLYDDAWGREKYINDKLPQGYLINNWYNTGWKQNEKNVGRFTWSESKG
NLDINIQEIIWKHGNKNKTLKSQCCANCRPGYRKVLQKSLTPCCYNCAPCSEGEISNGTD
MENCLQCPDNEWPNRGKNMCIEKQLDFLSYDGNFLAISFACISLICFVVTQIILGIFILYRST
PVVKANNRSLSFLLLVSIKLSFLSVFLFLGRPVDITCMLRIITFGITFSIAVSSLLAKTIMVCV
AFKATKPGSSWRKWLGVKLSNSVVLFCSSIQIIICMTWLAISPPFQELDIHTSPGTSSFSAM
RAQLLAFTQLLGIWGFWQLLVLF*HF*LGAYRTVLMRPSTSLSACCSSAVFGSQ*SRPI*AP
KAKTLCVWRYLP*SLQAVVFFSVYFYLSVTLYYLSQK*TEKNI**ADQII
>jgi|Xentr4|403162|e_gw1.779.91.1
KYLLDFLYAIEQINNNTTRLPNLTVGYHIYDSCGNEKKAVRSVLQILSGTREPVPNYSCVG
KRNIAGFIGDLFSRTTVPIAQILSVYGYSQISYGATDPSLSDRASFPYFFRTVQSDETNYSML
VQLIKYFGWTRVGFLTTNDISGYYEHQIFSRYLSNKGICIEFTLYLNQKYNFDDIMVNHMK
ILEKSSSSVVIVGGTASMIFADKIGGMYEDIVSKNFIFLPQWETYLLLPFDVMGRINSSLMF
VSLYPYNFNTPETHHYYENLHPSKYPDDKSLEDIWMLYFFCLSRNQNRNDLFEASIDVSLY
NCTGGERVANFNILKLFRHVPLVHFPVTIMIGAIEDMHAAHNKESPGKPKGPIIYRHRLHH
YLKKMTIDTQNGQFYKFDEYGNLGLDYDILNCVTEISTEDLKVTYRPVGKIRPKAPSDQQ
LEIYRDLITWRSNSSEIPRSQCSDNCLPGSRKATNSTIKSCCYKCVLCSEGEISNITGINLYINI
IKMTTFYSDKLMFNSISERNFYLSTISVSEVLAAIMVAQCRFMPIPAIVPWGRRWIFLLLVSI
KLSFLSVFLFLGRPVDITCMLRIITFGITFSIAVSSLLAKTIMVCVAFKATKPGSSWRKWLGV
KLSNSVVLFCSSIQIIICMTWLAISPPFQELDIHTSPRTIIIQCNEGSAIGFYSVIGYMGLLAAV
SFVLAFLARSLPDSFNEAKYITFSMLLFCSVWITMIPAYLSTKGKNTVCVEIFAILTSSAGLL
ACIFLPKCYIILFNSEMNTKKYLLGNKDV*
>jgi|Xentr4|403178|e_gw1.779.99.1
MSPTGGKTLRHNPAIQIFLYLIVMCVGPCRSETPITKAACRLQIIKVVEEYEYIQEGDIMIGG
VLTVSMFQPEDYFTGLTCVSPSAQNYKYLVDFLYVVEYFNKKPDILPNKTLGYLIYDSCGD
LRKAVRSVLQILSDIHEPAYHITGYCVDTIKTFIWLISTSSLLKCLVNNHPKGGKISYGATDP
ALSDRSTFPYFFRTTESDAGYYVLISKIAKYFQWNWVGIIVSNDERGERDHQLLKYYLSSE
NVCIEFTLKITNTNNGNIFYREILKKASANVIIFCGAVNIHIVLQFKYLYDMLSDKTFILTSN
WLYYNHLIDFTHKIFHGSLLLMQNKENYPSNSPYAQFSEGFNPSRYPDDKLLKYIWLQNH
FCLHKKQYIWQFYNCSEQESISEIPMYNSGFHTFNMIYAADMFVKALHYMHHSPGLGTSG
MSKKVYNYRYKIHQYLRQIQYLNFTNHVFSFNENGEFVTLYLIINLYPDLRRQSAWKICGN
YFSLVPTHRKLNFTIAKIQWKTKNNEVPISQCSDSCPTGFRKTLKLKVQPCCYDCVLCSEG
EISNRTDSENCIRCPYNEWPNEKRNQCIAKIEEYLSYTDNAIPVFFSVISALLLLKTVIISGVF

IFYRDTPIVRANNRSLSFLLLVSIKLSFLSVFLFLGRPVDITCMLRIITFGITFSIAVSSLLAKTI
MVCVAFKATKPGSSWRKWLGVKMSNSVVLFCSSIQIIICMTWLAISPPFQELDIHTSPGTIII
QCNEGSAIGFYSVIGYMGLLAAVSFVLAFLARSLPDSFNEAKYITFSMLLFCSVWITMIPAY
LSTKGKNTVCVEIFAIISSSCGLLFCIFLPKCYIILFKPEMNRKEYLTGRLNN*
>jgi|Xentr4|403198|e_gw1.779.20.1
KVPRAQCSDNCLPGFRKAPKPGAQSCCYDCVPCSEGEISNTTGIHYSKDCFTCPYNEWSN
ENKNQCIAKIEVFLSYTNDTIAVVFFAILAFLLFITMMILAIFIYYLDTPIVRANNRGLSFLLL
VSIKLSFLSVFLFLGRPVDITCMLRNVTFGITFSIAVSSLLAKTIMVCVAFKATKPGSSWRK
WLGVKLSNSVVLFCSSIQIIICMTWLAISPPFQELDIHTSPGTIIIQCNEGSAIGFYSVIGYMG
LLAAVSFVLAFLARSLPDSFNEAKYITFSMLLFCSVWITMIPAYLSTKGKNTVCVEIFAILTS
SAGLLACIFLPKCYNILFRTEMNRKSNLLVIKTG*
>jgi|Xentr4|403211|e_gw1.779.36.1
YEDIKSLLFTVDEINKNPDLLPNITLGYHVYDTCGDPKLAIGSVLQILSGPGEPVPNYSCRG
KGEIAGFIGDQSAVTSLPIAQLLGIYGYSQISYGATDPVLNDRTLYPHYFSTGPNEHIQHVAI
AELVERLGWTWVIILALGDDRGERESKNLRDEINKHGGCVDFIGTLTDDETTSKRTLTRIQ
QSTAEVVVLGGGLFKSVSLIMLVESKIKDKTLVIPVTWVPIMADKLFNGSLQFRELFHLFR
NILTEYDEYALAAKEDLLHKDILSYVYLCFTHDEEKDALFYHVYGSFYQNHSCSEQLVGF
SNLNHRVYRAVNGLAQAEHNMLSSTGKSHHKDIRKNIHRTQLHRHLTNLRLTEAGGTEID
FNNLKNSPSKYEIISWSVFANSSPETFQAKVGEYFWSESESSLEIDIRNIFWKKNTNNQTLK
SQCSANCPPGYRKVPRNGAPPCCYDCTRCSEGEMSNSTDMENCLKCHIYEWPNQEKTLCI
KKQTEFLSYGEDCLSLAFIVLSVVFIFIAAVILGIFILFQDTPVVKANNRNLSFILLVSIKLSFL
CVFLFLGRPVDITCMLRQTSFGITFSLAMSSVLGKTIMVCVAFKATKPGSSWRKWLGVKL
SYFTVVICSFIQFLISIVWLVISPPFVELSSLSDPGKIFIQCNEGSVIPFYIVLSYMGFLASVSFI
VAFLARSLPDSFNEAKYITFSMLLFCSVWITMIPAYLSTKGKNTVCVEIFAILTSSAGLLACI
FLPKCYIILLKPEMNTKQYLMGTNKQATIIGS*
>jgi|Xentr4|403228|e_gw1.780.109.1
MLFSQLALGVAINGFIVGTYLLEWRKKRSLRMTDVVLVCLAVNRFLWQLFHTLGSVFSFV
QVVVTITYQVVYSVCAFLNWSGLWLASVLSVIHCVKISNYNNWLFINLKRRICWLVQWL
LLANLLTSLAFTLALVWFTFPENPANSTDVSSQTNATVNLLQNIPSPLLVLCLGSLLPFIIFCV
AVSLLINSLRIHVQQMRSRATGFQTPHLEAHIRVIKSMAFFLVLFLLYLVVTILGAGKKNVG
NWLYFYLGMAFYPSLHSAVLIYSTRQLRMACLAIYYGTKKLICVHHGKVPPDLQ*
>jgi|Xentr4|403255|e_gw1.780.88.1
TMDLGFQVILLVQFFLGISVNGFILGVLFTEWITGGSLNAIDMILISVGLIRFLWHWVISLTC
LFPDAGLLVSAICITLSTLLNWSSFALVSVLCVTFCIKISNYNNAIFIYVKLRISDILKRLILGS
LGISLAYSVISVPWDMVVPRQNNSDILLERSAFFLMGTSTDYSFALFCFAFSVSFIMRFASIS
ALIHSLRRHMRQIQCSGEALQNPHLAAHIRVIKVMLIFLALYIMYFTSLVMGSLERYRTKQ
LPNYYVIVLCLYPSVHSGVLIYSNRRLRKACVAMCLGSIHWAKARKPIAQTQNPV*
>jgi|Xentr4|403791|e_gw1.787.24.1
MGNHSYFEAFHILAFSNTSVNHILLFFIFFLIYCSAVVGNVILIILVLADSRLHTPMYILLSNL
SSLDVGFTTVTIPKLMDILLTGYHSISYRSCLTQLYFFVFFGSTEALILSSMAYDRYVAICHP
LQYQAIMSGKKYSLIVGGPWIMGSVNSLFVTLLASKLSFCGTHNIDAFFCNVKALSKISCN
SSGFQTIIYIDTFIIGLFQLMLIFISYIKIVIVTLGIRSSGARMKLFSTCSSHLTTLIIFYGSVLCM
YARPPSQHRDNLDNVFSVVYSAITPMLNPLVYSLRNNDVTSALESSLKKIKKKVYEN*
>jgi|Xentr4|403797|e_gw1.787.5.1

MGNHTYCEAFHILAFSDTPVNHLLLFCIFFFIYWTAILGNLTLITLVSVDSRLHTLMYILLSN
LSFLDICFITVTIPKATAILLSGYNDISFTHCFIQLCFYIFFGTTETIILSSMAYDRYVVICHPLH
YKIIINGGTYALIVVGPWIMGCVNSLCLALLVSNLSFCGVHDIEAFFCNIKSLYKISCNSSGF
QTVIYVDALIIGLFQFILTLMSYTKIIMVTLRIKSSSGRMKSFSTCSSHLTSLVIFYGTMLGM
YIRPPSEHADDLDNVFSVLNSAIIPMLNPLVYSLRNQEVKSAMVKMMPFKNK*
>jgi|Xentr4|403812|e_gw1.787.4.1
MINQTYPEAFHIVAFSDTSVNHLFLFFTFLLNYCTAILGNVLIMTLVFADPCLHKPMYILLT
NLSFLDICFITVSISQAMEILLTGYRYISYARCFTQLYFFVFFGSTEALILSSMAYDRYVAICH
PLQYLMIMNGKKYALIVGGPWIMGSVNSLFLTSVASKLSFCGELDIDTFFCNVKSLCKISC
NSSGFQTIIYVDALIIGLFQSILSLMSYTKIITVTLRIKSSSGRRKSFSTCSSHLSSLVIFYGTAL
CMYVRPPSEHKDDWDNVFSVLHSAVTPMLNPLVYSLRNKEVKSATLKILAFLRKSS*
>jgi|Xentr4|403816|e_gw1.787.6.1
MGNHSYFEAFHILAFSDTPVNPLLMFFVFFVIYSTTVVGNVIIISLVLADSRLHTPMYILLSN
LSSLDVGFTTVTIPKLMDILLTGYHSISYRSCLTQLYFSVFFGSTEALILSSMAYDRYVAICH
PLQYLMIMNGKKYALIVGGPWIMGSVNSLFLTSVASNLSFCGTHDIDAFFCNVKSLSKISC
NSSGFQTIIYIDTFILILFQLMLIFISYIKIIIVTLGIRSSGARMKLFSTCSSHLTTLIIFYGSVLC
MYARPPSENADDLDNVFSVLHTAITPMLNPLVYSLRNNEVKSALEKIKEKFKKVL*
>jgi|Xentr4|404365|e_gw1.795.57.1
MNEDLQVNISRPPPENVTAAASPLPPVLDSEPELVVNPWDIVLCTSGTLISCENAIVVLIIFH
NPSLRAPMFLLIGSLALADLLAGVGLIVNFIFAYLLQSEAAKLVTVGLIAASFCASVCSLLAI
TVDRYLSLYYALTYNSERTVTFTYVMLIFLWGASTCVGLLPIMGWNCLEDESTCSVIRPLT
KNNAAALSVSFLLMFALMLQLYIQICKIVMRHAHQIALQHHFLATSHYVTTRKGVSTLAII
LGTFAACWMPFTLYSLIADYTYPSIYTYATLLPATYNSIINPVIYAFRNQEIQKALWLICCGC
VPSSVAQRVRSPSDV*
>jgi|Xentr4|404476|e_gw1.799.95.1
MANQSEVLEFVLIGFPGLPKNFHILVSLMMFLIYITALVANGTVIGLIVMKEHLHQPMYIVI
GNLSLSDLLFDTLTAPKIIAKYWFGDGNISFPGCFFQLFFVHCLGSVDSFILMLMAADRSVA
IFQPLRYFAIVTKKLAVALCCFFWALSAAVVSVITYMTLTLPFCGPNKIIGCFCSSSVVFPLA
CTDVTFVRKVTLILALSVHLVPLTLIILSYLLIVLTIHSMLHSDNWQKLFYTCTTHLLVISLY
FIPRLFVYLANYVRLLFNADINVLILYLYTFLPHLANPIIYCLRNKEIKIILENLMKVKIGIKV
*
>jgi|Xentr4|404482|e_gw1.799.79.1
MTISMPNQTAVTEFILLGFPGLQPNFFLPVSLTLFLAYIVSLIANSTVIILIILREQLHQPMYIII
ANLALSDLLFDTITLPKIIAKYWFGAGSISFFDCFFQLFCVHSLGYLDSLIIMLMAIDRYVAI
CQPLRYHSIISNKVLTLLCCMFGFCAALYGLYITLIAVQVPYCGPNHVNNCFCSNQAVIVLA
CVDVSLEKRERFIIGMSVHLFPLAVIILSYILIIRVVHLSANNGNWQKAFYTCTTHLIVIGMH
FIPRLFVYSTSQTSLIFDDDINVLIVCLYTFIPHLASPIFFCLRTIEIRNILGQTFNIFLGQTLHKI
CEKASY*
>jgi|Xentr4|404485|e_gw1.799.70.1
MTISMPNQTAVTEFILLGFPGLQPNFFLPVSLTLFLAYIVSLIANSTVIILIILREQLHQPMYIII
ANLALSDLLFDTITLPKIIAKYWFGAGSISFNWCFFQLFCVHSLGSLDSLIIMLMAIDRYVAI
CQPLRYHSIISNKLVALICYFLWIFAALMGLALTLIAVQVPYCGPNRIKNCFCASQSVIVLAC
VDVSLERKKGVIIGMSVHLIPLAVIILSYILIIRVVHLSANNGNWQKAFYTCTTHLLVIGLYFI
PRLFVYSTSQIPLILDADINILILCLYTFIPHLASPIIFCLRTKEIRNILGQTFHNTIHMEPKHRC

RTLR*
>jgi|Xentr4|404489|e_gw1.799.54.1
MQNSSDPHPAFLTLGYGQLTSIRYFYCLLVSVGFLLNVVSNSLIVVAVIIHKTLHEPVYIFISA
LCLNGIFESMYFYPSIFAALLYKVQTISYSFCLLQAFLIHLYGCFEMTTLSAMAFDRYVCIC
NPLRYNTIMSHSTVYKILAGSWTYSIVAFGTHILLTYRLPLCGAEILKIYCDNWSIVRLSCID
TTINNVFGLFIVTTFVCMLLMLIMYSYFEILRVCARSSQAVIAKALQTCTPQLITTFNFVAGS
LFDIFLYRYIPTIVPYEFRLFMSLEFLVVSPILNALAYGLKMSELRAKIFQPFNLKRFAGNTLS
V*
>jgi|Xentr4|404493|e_gw1.799.90.1
MANGSGATDFVLMGFPGVPQGYHIPISLAFFVIYMMSLGANGTVIALILPKRNLHQPMYIL
VANLALSDLLFDTVTLPKIIAKYWFGDGRMSFSGCYFQMSLVHLLGSLDSYLIMLMAGDR
LIAIGQPLRYAALITPKVTLIICFFSLVLATGVSSVITFLILDVPFCGKYKINSCFCSRTFVVPLI
CSDLSTFRVVNLSLSLIVLLVPLAFIILSYVIIIGVINSSLRSENLGKMFYTCTNHLLVTGMYY
IPRLFLYVASYVRLIDSADCDVLILCLYTFLPHVANPVIYCLRNKEIQVTLRDLYRRKVGSL
M*
>jgi|Xentr4|404496|e_gw1.799.74.1
MTISMPNQTAVTEFILLGFPGLQPNFFLPVSLTLFLAYIVSLIANSTVIILIILREQLHQPMYIII
ANLALSDLLFDTITLPKIIAKYWFGAGSISFNWCFFQIFCVHNLGSLDSLIIMLMAIDRYVAI
CQPLRYHSIISNKLATLLCYLCWFCAALIGLAISINAGQLPYCGPNRVKNCFCSNAVLIVLA
CVDVPLERRKGFIIAICVHLFPLAVIILSYILIIRVVHLSANNGNWQKAFYTCTTHLIVIGLYF
IPRLFLYIISQAPLTLDADTNVLIISVFTFIPHFASPITFCLRTKEIRNILGQMFNNFGPRFEHGC
RTPLKKHCTSAIFQAQ*
>jgi|Xentr4|404498|e_gw1.799.21.1
MLNSTYSHPSELTLTFVEMAGIKYAYSTLIFLCFAVIAASNCAVVGTIVAHRSLHEPMYILIA
ALSVNGLYGSAAFFPNLFINLLSKSHTIPYVACIIQMFGLHTYVGCEMAILAVMAYDRYVCI
CIPLRYNSLMSLYTVLRLIAAAWVYAVVQFTVQLVLTVRLPLCGSVVQKFYCDNWSVVKL
SCVDTTANNIYGLFITAVILGLIPGTVLISYLQILRVCVRSSSTHRSKALQTCTPHMMSLTYF
VLDLISEALLNRSPANVLPIELRVLISVQVFVIAPFLNPLIYGLKLKEIRVKAKHMFCKVKIF
GVDGKIGAQ*
>jgi|Xentr4|404509|e_gw1.799.17.1
MQNSTPHPAVLLLGFGEMTSVKYLYCSLILIGYSVIILLNSAVIAVTVLHETLQRPMYIFISAF
CINGLYGSTAFFPSVFINLFKEAQTISYGACILQDFAVNTYGGSEMTLLAVMAFDRYVSICH
PLRYNSIMSLPTVFRLIGAAWLYPFILVSILVLLTVRLPLCDWVILKIYCDNWSVVRLSCVD
TSTNNAYGILCTVAILVVMPILIVISYIAILRVCAKSSKDFQAKALQTCAPHLLAIATYLVDV
LFEIFLYRYSPTSLPPALRVFMSIHFLVVPPLLNPLIYGLKIRELRVKINKLLQKQSIPLREPKH
P*
>jgi|Xentr4|404530|e_gw1.799.77.1
MVAMPVPNQTAVSEFILLGFPGLQQIFHTPVSVIMFLVYSVSLIANSIVIILIVLKGNLHQPM
YIVIGNLSLSDLLFDTITLPKIIAKYWFGAGSMTYYGCFFQLFCVHYLGSLDSLIIMLMAID
RYVAICKPLRYHSMMTIRLVVLICYCFWALDAIQVAILVVYNARLSYCGPNKIKNCFCSITF
LSALACDDRTFVITLSYSFAMCALLIPLAVIILSYLLIIRVVHMSAGSGNWQKAFYTCTTHL
FVIGLYYIPRVFLYTTSKIPLILDADVNVLLLCLYTYIPHLASPLIYCLRTAEIRTIVGQIFYKT
LNIKCEGTL*
>jgi|Xentr4|404532|e_gw1.799.69.1

MTISMPNQTAVTEFILLGFPGLQPNFFLPVSLTLFLAYIVSLIANSTVIILIILREQLHQPMYIII
ANLALSDLLFDTITLPKIIAKYWFGAGSISFNWCFFQLFCVHSLGSLDSLIIMLMAIDRYIAI
CQPLRYHSIISNKVATLLCYLFWIFAALIGLAMTLIAVQVPYCGQNRVKNCFCASQSVIVLA
CVDVTLERRKGYSIAMCVHLFPLAVIILSYILIIRVVHLSANHENWQKAFYTCTTHLLVIGL
YFIPRLFVYSTSQAPLILDADINILILCLYTFIPHLASPIIFCLRTKEIRNILGQIINTIFHIDSEHG
SRTLQ*
>jgi|Xentr4|404537|e_gw1.799.5.1
MQNSSDPHPAFFTLGYGQLTSIRYFYCLLVSVGFLLNVVPNSLIIVAVIIHKTLHEPVYIFISA
LCLNGILESVYFYPGIFAALFYKVQTISYSFCLLQAFLVHLYGCFEMTTLTAMAFDRYVCIC
NPLRYNNIMLLSNVYKILAGSWTYSIVAFGTHVLLTYRLPLCGSEILKIYCDNWSIVRLSCI
DTTINNIYGLFIVSTFVCLLLMLIMYSYIEILRVCARSSQAVIAKALQTCTPQLITTFNLITAS
GFDIFLYRYIPTIVPYEFRLFMSLEFLVVSPILNAFVYGLKMSELRAKIFQPFKFQGTP*
>jgi|Xentr4|404547|e_gw1.799.9.1
MTSIKYLYCALALLGFLMILLASGSVIAAVLLNKSLQEPMYIFVCVLCANGIYGSVGFFPSL
FVNLIGETQTITYIGCLIQMFCIHTYTAAEMSILALMAFDRYLCICNPLRYNGIMTLAVAYK
LIVAAWLFVLIPFTIHFILTILLPLCGSSIVKIYCDNYSLVKLSCVDTTVNNIYGLFLTVAMVV
MVPAFIVASYIQILRVCARSSMDFRKKSLQTCTPHLISLTNYVADILFEVLLHRFTLKNLPYE
LRIIMSVQAFVVPPLLNPVIYGLKMREIKLRIAHMFHINKIRVQEE*
>jgi|Xentr4|404548|e_gw1.799.56.1
MTQARYLYSVLTLIGFTVIVLANGAVIIVIVLHQTLHKPMYIFISLLCINSLYGCFAFFPSLFV
NLLSTTQTISYIGCITQVFCLHTYGGCELSLLTIMALDRYVCICNPFWYNQIMSLPTVCKLA
MAAWLIGIIPVSTFVLMTIRLPLCGNLILKIYCDNWSVVRLSCIDTTINNIFGLCIIVGFLTVM
PAFILFSYAEILKVCMKSSKVFQAKAWQTCAPHIISVINFMVIRLFEFLNNRLSLSSWSNNLS
IIMVVQAFVLQPVLNPLIYGLKLKKIKTKMIQFFFQKTYGSER*
>jgi|Xentr4|404561|e_gw1.799.4.1
MINVTAAHPEFLTLGFGELTSIKYLYSLLVFLGFALTLLLNSLVVAAVALHRSLQEPVYMFIC
ALSINGIYGSVVFYPGIFVTLLYQVQKISYGSCLAQVFFIHTYGSFELATLAGMAIDRYICIC
NPLRYTSLMSHSTVLKIIAVGWVNSIVAIGTNLVLTYRLPLCDSVILKIYCDNWSVVRLSCV
DTTINNIFGNLVSTSVIYVPILIIVFSYVEILRVCVRSSKEVISKALQTCSPQLIISFNFVTGALF
DIFLYRYMPTSVPYQIRLFMSLEFLVISPILNSFIYGLKMKEMRSRILKLLHLETSKVHEKHR
RSLWYG*
>jgi|Xentr4|404566|e_gw1.799.27.1
MLNVTYSTPAVLKLGFGELRSMRYLYSILVLGGFVIVVTSNVAIIAAVLLNRGLQESMYIFIC
MLCLNGLYGSLAFFPSLFINLLCETQTISYTGCLIQAFCLNTYTGCEFTILTIMALDRYICICY
PLRYIAIMSLPTVLRLIIAAWLTAILPFTVHYILTLRLPLCDSTIVKIYCDNWSVVRLSCNDTS
WNQISGLILTVGFLVVMPALIFFSYAMILRACTKSSKQIRTKALQTCAPHLITITNFIADRLFE
VILHRITLNNIPYSLMIFISVYVYVVPPLLNPLIYGLKLKDIRVKIVQLICNQ*
>jgi|Xentr4|404698|e_gw1.803.23.1
MDVSCNLSYCSIAASMQEAEQSDLLGMFPLFLEERLLNRSNESLQDFIKGFNLDKSDIIGD
SSAIVRIIISIVYSVVCALGLVGNVLVLYLMKTKQGWKKSSINLFVTSLAITDFQFVLTLPFW
AVENALDFSWLFGKAMCKIVSYVTAMNMYASVFFLTAMSVARYYSVASALKVKRRPAGC
SAKWVSVLIWVSAIVASLPNAIFSTTATVSSEVLCLVKFPVNNGNAQFWLGLYHAQKVLL
GFLIPLFVITICYLLLVRFITDRNISSSSTKRRSKVTKSVTIVVLSFFLCWLPNQALTTWGILIK
LNIVQFSYEYFTTQAYIFPITVCLAHSNSCLNPILYCLMRREFRKALKNLFWKIASPSLTNM

RPFTATTKPEQDDHAHGMVPLNPAEPDVICYPPGAVLYNGRYDLLPNSSTEQRY*
>jgi|Xentr4|405352|e_gw1.819.5.1
MGAQWILSGKEKVVPNFDCNKDIMPLAIIGDLPTKASIPIARIMGLNRYPQIPVSVCSDPCP
RGHRRATIQGQKICCFDCLPCSEGEIPNPKGLKCLKCPEDQWPDSMKEKCLPKPIQFLSYD
EPLGSSLACISVLFCLITFSAFCLFIAKRKTPIVKANNRELSYLLLISLMFGFLCSLAFIGRPN
LKTCMIRQVLFAVIFSFCISVILAKTVTVIMIFSATNPDSKLKRLVAMRIPVYIVPVCTMVQII
LCIVWLARAAPFAEFNMAAEIGIIVIECNEGSRVLFACVLGYMGLLAAVSLLVACLARKLP
DTFNETKFITFSMLVFASVWLTFIPAYLSTKGKQTVAVEIFAILSSMAGLLVCIFIPKCYIILLH
PEMNSKQYITGRNTRNRGT*
>jgi|Xentr4|405358|e_gw1.819.20.1
MHFIFIVVVHLILLLIGYKVDEGEALLEGCRLFTEDVIGYSQAGDITLGGLIPVHMDTNHPV
INFRENPQPLQCGRFHLRYYRFFLAMVYTIMEINASDDLLPNITLGFKHYDSCYNEVKSLM
GAQWILSGKEKVVPNFNCNSDIMPLAIIGDLISKASIPIARILGLTRYPQVSYRSVHPLLSDK
KQFPSFLRTSHNADYEVFALAQLLKYFNWTWVGIIFSDNDLGRSGAQLVSREIENNGGCIA
FNEVLPIVNFMESVYRIIDVIQKSRATVIIAYCTIESFIPLIEEASIHNITDKVWVVTTSWSISS
DFPRKDFLTTLNGSLGIATRHGKIPGFKEFLYSLQPSVSLDDLLVKTFWENAFQCVWPENG
AYNNSPHREDIVWCTGKERVDSIDPNIFDVYNLRVTHKVYTAVLSVAHALHQMNNCVPG
KGPFKNGSCADIYNHQPWQLLHYIKNINFNNTEGEQIYFDVNGDVPLYLDILNWQMFPNG
SNQYVDIGSFDARALNGQELKILDNKILWNTGHSQIPVSVCSDPCPRGHRRATIQGQKICC
FDCLPCSEGEILNPNDDSKCLKCPEDQWPDRMKERCLPKIIQFLSYDEPLGSSLACISVLFC
LITFSAFCLFIAKRKTPIVKANNRELSYLLLISLMFGFLCSLAFIGRPNLKTCMIRQVLFAVIF
SFCISVILAKTVTVIMIFSATNPDSKLKRLVAMRIPVYIVPVCTMVQIILCIVWLARAAPFAE
FNMAAEIGIILIECNEGSRVLFACVLGYMGLLAAISLLVACLARKLPDTFNETKFITFSMLVF
ASVWLTFIPAYLSTKGKQTVAVEIFAILSSMAGLLVCIFIPKCYIILLHPEMNSKQYITGRNTR
NQRT*
>jgi|Xentr4|405360|e_gw1.819.49.1
MYFIFIFVCHLSLLLVLSWYQGGVGEALLEGCRLLTEDVIGYSLAGDITLGGLFSVHMNVS
YPVVNFRERPQPIQCSRFHLRYYRFFLAMVYTIMEINASDDLLPNITLGYKLYDSCYNEVR
SLMGAQWILSGKEKVVPNFDCNKDIMPLAIIGDLPSKASIPLARIMGLNRYPQVSYGSLHP
SLSDKIQFPSFLRTIHNANYEVFALAQLLKYFNWTWVGIISSDNDLGRSGAQLVTREIENN
GGCIAFNEILPIFNFMESVFRIIDVIQKSRAKVIIVFCTVVSFIPFIEEASLHNITDKVWLGTAS
WSISSDFPRKDFLTTLNGSFGIAAQHGNIPGFREFLYSLHPSVSLDNLFVKTFWENAFRCV
WPENGTYNNSSPAPHREDIVWCTGKERVDSIDPNIFDVSNFRFTHKVYTAVLSVAHALHQ
MNNCVPGKGPFKNGSCADIYNHQPWQLLHYIKHIRFNNLSGDEIYFDENGDVPLYLDILN
WQMFPNGSNQYVSIGSFNARAPKGQQLKIQEDKILWNVDKIPVSVCSDPCPKGHRRATIQ
GQKICCFDCLPCSEGEILNPNDNSECLKCPEDQWPDRMKERCLPKTIQFLSYDEPLGSSLA
CISVLFCLITFSAFCLFIAKRKTPIVKANNRELSYLLLISLMFGFLCSLAFIGRPNLKTCMIRQ
VLFAVIFSFCISVILAKTVTVIMIFSATNPDSKLKRLVAMRIPVYIVPVCTMGQIILCIVWLAR
AAPFPEFNMAAEIGKIVTECNEGSRVLFACVLGYMGLLAAISLLVACLARKLPDTFNETKFI
TFSMLVFASVWLTFIPAY
>jgi|Xentr4|405387|e_gw1.819.21.1
MLLPYKHGKCEALLEGCRLLTEDVIGYSLAGDITLGGIFSVHVDTNHPVINFRESPQPLQC
NRFHLRYYRFFLAMVYTIMEINASDDLLPNITLGYKLYDSCYNEVRSLMGAQWILSGKEK
VVPNFNCNKDIMPLAIIGDLPSKASIPLARILGLYRYPQVSYGSVHPSLSEKTQFPSFLRTIH

NANYEVFVLAQLLKYFNWTWVGIISSDNDLGRSGAQLVSREIENNGGCIAFNEILPIVNFM
GSVYRIVDVIQKSRATVIIAYCTIESFIPLIEEASIHNITDKVWLGTTLWTISSDFSKNEIRTTL
NGSLGIATQHGNIPGFKEFLYNIHPSVSLDDLLIKTFWENAFQCVWPENGTYNNSSPAPHR
EDIVWCTGKERVDSIDPNIFDVYNFRVTHNVYTAVLSVAHALHQMNNCVPGKGPFKNGSC
ADIYNHQPWQLLHYIKHIRFNNTAGEEIYFDANGDVPLYLDILNWQMFPNGSNQYVSIGG
FDAQAPKGQELKIQINKILWNVGHGQSMANVLVSSSIKSVSSIKVCSFLMHCSANLKGICA
GDSKCLKCPEDQWPDSMKERCLPKTIQFLSYDEPLGSSLACISVLFCLITFSAFCLFIAKRK
TPIVKANNRELSYLLLISLMFGFLCSLAFIGRPNLKTCMIRQVLFAVIFSFCISVILAKTVTVI
MIFSATNPDSKLKRLVAMRIPVYIVPVCTMVQIILCIVWLARAAPFAEFNMAAEIGIIVIECN
EGSRVLFACVLGYMGLLAAVSLLVACLARKLPDTFNETKFITFSMLVFASVWLTFIPAYLST
KGKQTVAVEIFAILSSMAGLLVCIFIPKCYIILLHPEMNSKQYITGRNTRNRGT*
>jgi|Xentr4|405848|e_gw1.828.77.1
MENQTTVKTFILAGLSDLPSLQLPLFLVFLLIYLITLTGNLLILLLIFTDSHLHTPMYFFLGTL
ACLDMSCSSVTAPRMLFDLLRDRKIISMRVCITQFYFLIFFMTLEMSMLAVMSYDRYIAICR
PLHYMQIMSWNVCVQFVSGVLLFSTIHSLAHTLSLTKLSFCRQNVLQSFFCDLPQLLEASC
SNTFINVLLLFLFGIVGGIGILGGTFYPYIPIITTVLKMTSKYTRSKAFSTCSSHLTVVFSCYT
TIFFNYFRSKANDHLVEGKVASVFFAILTPSLNPVIYSLRNQELKLSLRKTLQRLK*
>jgi|Xentr4|405867|e_gw1.828.9.1
EMENRTTVNNFILAGLSDLPSLQLPLFLVFLLIYLITLTGNLLVIFLIFTNSHLHTPMYFFLGT
LACLDMSYSSVTAPRMLFDLLREKRTISIPACITQIYFFFCFAVLEMLVLAAMSYDRYIAICR
PLHYMQIMSWKVCVLIASGVLVFSALYSLVYILFLNKLTFCLPNVLQSFFCDLPQLLEASCS
DTFINVMLTLVLGPLFGVGILGGTFYPYIPIITTVLKMTSKHARSKAFSTCASHLTVVFIFYT
TIFFNYFRTNGNDNLVEGKVASVFFAILTPFINPLIYSLRNQELKLSLRKAVQRLFHRLRNVL
MNDHLHF*
>jgi|Xentr4|405878|e_gw1.828.19.1
MENQTTVKTFILAGLSDLPSLQLPLFLVFLLIYLITLTGNLLILLLIFTDSHLHTPMYFFIGTL
ACLDMGYSSVTAPRMLFDLLRERKIISVRACITQIYFFIFFIALEISVLAVMSYDRYIAICRPL
HYMQIMSWNVCVLLLFGVLLFSTICSLVHTVFLTKLTFCRSKVLQSFFCDLPQLLEASCSD
TFINVLLIFLLGTLFGGAVLGGTFYPYIPIITTVLKMTSKHTRAKAFSTCSSHLTVVFIFYTTC
FFDFFRSNTNDNLVEDKVASVFYAILTPSLNPVIYSLRNQELKLSLRRTLQRLQ*
>jgi|Xentr4|406263|e_gw1.834.63.1
YRHYVDFLFAIENINKDRILLPNLTLGYHISDSCGDARKAVRSVLQILSGTREPVPNYSCVG
KRHIAGFIGDLTSETTVPIAQILSLYGYTQISYGATDPMLSDRFSFPYFFRLLHSDAHYEALA
EFLYYFGWTWVGIIRTEEDNEDREHQLLTKHLSRLGICVAFDIKMRKERDMLTKNNFNYY
EEWKRIIKQSSVNVTIIFGTFSVTFQQELIKLIDVLDKNTVVFTSNWALNGDSLNYIMGIVN
GSLIIMQKPINFITTRTEHFLENIHPYNYPEDKVLEDIWMQYHFCLSEDQDKNQLYETIYSG
TLHNCTGQEHITDIGNFNNVLHSALVYSAVQVMYRALYMMHFSFSSKSPPNSKRISSYRN
QLHQFLKLIKIKGKDHFLDKNGNFQIEYLLYNCYIELFGKLYVNWVGHFCPWNKSDRALR
VSPDEITWKTSKGQIPRAQCSASCPPGFRKAPRPGAQSCCYDCVLCSEGEISNITDSISCIRC
SDMEWPNEKRNRCMTKTEEFLSYTNDLISVFISSISVLFFLITLLVLVVFISNHDTPIVRANN
RSLSFLLLVSIKLSFLSVFLFLGRPVDITCMLRIITFGITFSIAVSSLLAKTIMVCVAFKATKPG
SSWRKWLGVKLSNSVVLFCSSIQIIICMTCLAISPPFQELDIHTSPGTIIIQCNEGSAIGFYSVI
GYMGLLAAVTVSFVLAFLARSLPDSFNEAKYITFSMLLFCSVWITMIPAYLSTKGKNTVCV
EIFAILTSSAGLLASIFLPKCYVILFKPEINTKSHVFANKS*

>jgi|Xentr4|406281|e_gw1.834.79.1
MIHCAWIPTRELLSDQLRVIFCLAAIWVTPCSTELSGSDSPCRGHITKPEYEYKYIQDGDIII
GGVFTVHSNIKYVYNGPVTSLPLCTVPFLARYQQIRTFLFAIDEINRNPDLLPNITLGYRVY
DSCGDPRLAIGSALQILSGPGNVVPNYSCRGKGEIAGFIGDQSSLTSLPIAQLLGVYGYSQIS
YGATDPTLSDREMFPYYFSAGLSDDIQHIAVAELVEQLGWTWVIIVATDNESGEKQSQNLQ
NEISKHGACVDIIIFITEDDDTNKRHFERIRISTAEVIVLCGTPSDSVLLSLCTRESLIHEKTLV
ITLSWGDYGYSCNFLYNASLMYFYPYNLIEELDTQFHDYVLSVTEDSKLLKELLSQYPSCL
EQENINNTYFRNCTEKKTIKEVGLYFRHNAEVYRSVYTMVHALHTKLSVSVTHRDKYLP
HYIGNLHFRDPWGRETHYKEFRDILREYWILNWCFFPGSGVCENYVGKIVLSESQPKIEINI
QEIIWKKNQTVKSQCSANCPPGSRKIPGKSAVPCCYACVPCSHGEISNRTDMENCFKCEDT
EWPNQEKTLCIERQTEFLSYAGDPLTLISIISSVILFIIAAVILGMFISFRDTPVVRANNHTLSFI
LLVSIKLSFLSVFLFLGRPVDITCMLRQTSFGITFSIAVSCVLAKTLMVSIAFKATKPGSPWR
KWVGVKLANGFVFIFSLIQSLISVIWLVIAPPFVEQNTHSEPGKIIIQCNEGSVVAFYIVLSY
MGLLASVSFIVAFLARSLPDSFNEAKYITFSMLLFCSVWITMIPAYLSTKGKYMVAVEIFAII
SSSCGLLFCIFLPKCYIILFKPEMNTKQYLLGKCNT*
>jgi|Xentr4|406303|e_gw1.834.78.1
MLRATSVFKAIELMLFVTVLCVGPCRSGVQPINPACHLETTKSAIEHEYIQEGDIMIGGVMT
AHFVVEELNGTNDRLVKHLCTAANEEYLKYLLDFHYVIEQMNNSTTQFPNLTLGYHIYDS
CGDPGKAVRSVLQILSGTREPVPNYSCVGKRNIAGFIGDLSSKTTLPIAQILSIYGYSQISYG
ATDTSLSDRLTFPYFFRTVQSDQTNYLMLVRLIQYFGWTWVGFITLDDISGFYEHQTFSQY
LSSQGICMEFAIYLNFNVFNDDFIPRYRKTIADSSSSVIIFGGTVSIAILSKITAVPDVLANKN
LIFSPQWETYLLLHFDSTALINNSLIFSSLYPYNLDTPEINYYYEHLHPLKYPEVTFLEDIWF
VVFYCFSGDLNKKNTFEFWYDCTLPKCTGEERITNHQTFKFYRNSPRVHFPVTMMVRAIE
ALHVAHNQEPPEKPIRPIIYRHQLQHYLKKMPLYMEYGQFYEFDEYGNLEIDYGIFNSIIAP
RTETNEVEVTLRPVGKIRPRAPSDQQLEMNKDLIMWNSDGLEIPRSQCSDNCLPGFRKAT
KETIQSCCFRCVPCAEGEISNTTDSELCFPCNDTEWPNKKRTQCIAKAEAFLSYRDVISMFF
SAFSVLSFFITLLILGVFISYRDSPIVRANNRSLSFLLLVSIKLSFLSVFLFLGRPVDITCMLRII
TFGITFSIAVSSLLAKTIMVCVAFKATKPGSSWRKWLGVKLSNSVVLFCSSIQIIICMTWVIIS
QLGSSTGTVLLLQRKGNNLTLYLATTCLAHCLAVKDSLCRDGMYPFCREFLAMTAPWATS
QGGCVGWCLDHNGVWITMILAYLSTKGKNTVCVEIFAILTSSAGLLASIFLPKCYIIMLRPE
MNIRSHIPYILEYKPSF*
>jgi|Xentr4|406331|e_gw1.834.52.1
YQQIRTFLFAIDEINRNPDLLPNITLGYRVYDSCGDPRLAIGSALQILSGPGNVVPNYSCRG
KGEIAGFIGDQSSLTSLPIAQLLGVYGYSQISYGATDPTLSEREMFPYYFSTGLSDDIQHIAV
AELLEQLGWTWVIIVAPDNDSGEKQSRNLQNEISKHGACVDIIIFITEDITVNMRKFERIRIS
TAEVIVLCGTPSDSVHLSLCALESVTHEKTLVGTLSWGDYEYDCDLLFNASLMYFYPSNPI
EGLDTQFHDYILSVTEDSKLLKELLSHYPSCWKQENINKTYFVNCTEMKTIKEVGVYFRH
NAEVYRSVYTMVHALHTKLSVSVTHRDKYIPINTQQVRSPNSLPLQLSNPPRGYLPPTDT
WGSNYSFTNHTVSPKASLVIYCRSGNIYKVTAAEIPNSASRISADTPPSCIFNSSLQTVKSQC
SANCPPGSRKMAGKSAVPCCYACVPCSHGEISNRTDMENCYKCEDTEWPNQEKTLCIEKQ
IEFLSYAGDPLTLIFIISSVILFIIAAVILGIFISFRDTPVVRANNHTLSFILLVSIKLSFLSVFLFL
GRPVDITCMLRQTSFGITFSIAVSCVLAKTIMVSIAFKATKPGSPWRKWVGVKLANGLVFI
CSLIQFLIIVFWLVIAPPFVEKNFHSEPGKIIIQCNEGSAIGFYLVLSYMGLLASVSFIVAFLAR
SLPDSFNEAKYITFSMLLFCSVWITMIPAYLSTKGKYMVAVEIFAIISSSCGLLFCIFLPKCYII

LFKPEMNSKQYLLGKCNT*
>jgi|Xentr4|406549|e_gw1.839.5.1
YRHYVAFLFAVEEINRSSRILPNISLGYIIYDTCENELKSIYDALAVMSGMIYQAPNYDFWE
KAKLMGYVGDVTSSTTFSLAQLISMFRYPQASIISYNTMDPVFNDRTRFPSFYRTIPNEEAE
MDGIVQILKHFGWKWVGLIISDDATGYRGRERISKELASRGGCLAFTAVLREIYFNYRHSE
EIVQQIRKSSAHVIVLYISTRFAFFFTNIFAIYKIPTKFWITSSFFPRVMIFRQQNIKITLNGSVS
LLIQEGEIPGFEKFFYRFSPYNNSDDLTVNTWSWLFGCNFPQRVYVRLQNSKIAKDCTRNE
TMSEADVSVYGNHNYRVTYRVYTAVYALARALHNLYSAQPPANHWGKLQSLRKIKPWQ
LNKYIRNLTFSTSTGDTVFFNDKGEPPPAFDIVKWFFLPDGRVKRQKVGQFNQLNSKKRFY
INGRDDVWGPYFKKMPLSQCNEPCAPGYRKAKIEGAPSCCYGCVRCADGEMSNTTDAA
NCERCSEYQKSNTERTECLLKAINFLSYTDTMGASLTVIAFILFITASVVLGIFVKYWETPIV
RANNQNLSCLLLISLMLCFLCTLLFIGRPTQICCLLRQVTFGTVFTISVSSVLAKTLTVIIAFN
ATKPGSKLKKYVGTQLAIVLVIICSLGSTGISAVWMASYPPFLEADTVSEMETVILMCNEG
SVSLFFCSIGYMGTLALLSFIAAFLAKDFPDRFNEAKNITFSMLGFCSVWGAFVPAYLSSKG
SRMVAVEIFAILASSAGLLGCIFAPKCYIIFLGPDLNKRTF*
>jgi|Xentr4|406558|e_gw1.839.9.1
IQTPQSLCNAPCAPGYRKSKIEGAPSCCYDCVPCVDGEISNKSGKRQICCFKCSEYLMSNI
NRTACNPKTMNYLSYEDALGASLASIALVLFLTTSVVQGIFVKYWETPIVRANKQNLSCLL
LISLMLCFLCTLLFIGRPTQICCLLRQVTFGIVFTISVSSVLAKTLTNSPIGFISWLNDSLFSVII
KQYGDPNYPEYPWSQAFWITGPIPAIHFFLMIKYSENQRGIQESTK*SVAFFFCIIGYIGTLA
LLSFIAAFLAKDFPDRFNEAKNITFSMLGFCSVWGAFVPAYLSSKGSRMVAVEIFAILSSSA
RLLGCIFLPKCFIIFLRPEMNTKKAVVLK*
>jgi|Xentr4|406570|e_gw1.839.26.1
IQIPQSLCNEPCAPGSRKSKREGELSCCYDCIKCVDGEMSNITDAPGCSKCPEDRKSNLQR
TDCIPKTITYISYMDSMGFGLTTITLILFFAVYAVFTIFLKYWHTPIVRANNQHLSCLILISLM
LCSLCTLLFIGRPTQICCLLRQITFGIVFTISVSSVLAKTLTVIIAFNATKPGSKLKKYVGTQL
AIILVIVCCLAEMGISVGWVASYPPYVEADMISEKDIIILQCNEGSVAFFFCIIGYIGTLALLS
FIAAFLAKDFPDRFNEAKNITFSMLGFCSVWGAFVPAYLSTKGSRMVAVEIFAILSSSAGLL
GCIFLPKCFIIFLRPEMNTKKLLS*
>jgi|Xentr4|406580|e_gw1.839.8.1
ISHRASLKYYRHYLAFLFAVEEINRSSRILPNISLGYRIFDSYEHEPKSLYDALAIIYNTKDPA
LSDRTQFPSFYRTIPNEEAEMDGIVQILKHFGWKWVGLIISSDATGYRARERISKELSNNGG
CVAFTAVLRDAAYISDYNAEEIFQQIRESSAYVIVLYIGTRYVPAIAGLITNPYGLPEIPTKFW
ITSSFFTKVTLYMQENIKRTLNGSVSLLIQEGEIPGFEQFFYSFSPYNYRDDLTKDVWEGIFG
CHFPAGSYAETLSTVSKVAYNCTGNETIVDVNILVYGDHNYRVTYRVYTAVYALARALHN
LYSAQPPANHWGRLETLKRRLNKYIRNLTFTTSTGDADSFNDKGDPSAFDIVKWFFLPDG
RVKRQKVGEFNQLNNSGKQLYINGSADIWGPYSKQMPLSQCNEPCAPGYRKAKIEGAPSC
CYGCVRCADGEMSNTTGKSQSLNQKPMILPINMFISDAANCERCSEYQKSNTERTECLLK
AINFLSYTDTMGASLTAIAFILFIAASVVLGIFVKYWETPIVRANNQNLSCLLLISLMLCFLC
TLLFIGRPTQICCLLRQVIFGIVFTISVSSVLAKTLTVIIAFNATKPGSKLKKYVGTQLAIVLVI
ICSLGSTVISAVWMASHPPFLEADTFSEMDTVILTCNEGSVFFFFCIIGYIGTLALLSFIAAFL
AKDFPDRFNEAKNITFSMLGFCSVWVAFVPAYLSSRGSRMVAVEIFAILSSSAGLLGCIFAP
KCYIIFLQPELNTKTF*
>jgi|Xentr4|406581|e_gw1.839.66.1

SLCNAPCAPGFWKSKIEGRPSCCYDCAQCADGEMSNTTDAVDCMKCPEDQKPNRQRTDC
VPKAINYLSYMDTLGASLASIALVFSITASVVLGISVKYWETPIVRANNQNLSCLLLISLML
CFLCTLLFIGRPTQICCLLRQVTFGIVFTISVSSVLAKTLTVIIAFNATKPGSKLKKYVGTQLS
VLVVFTCSSGETAISVAWIVSSPPFPDTDTSFETDIFLQCNEGSVTFFFCIIGYIGTLALLSFIA
AFLAKDFPDRFNEAKNITFSMLGFCSVWGAFVPAYLSSKGSRMVAVEIFAILSSSAGLLICIF
APKCYIIFIRPELNKKENCCASKRSLSSKELSHVKRIVDQHFR*
>jgi|Xentr4|406597|e_gw1.839.29.1
MSAADVSVYGNHHYRVTYRVYTAVYALARALHNLYSAQPPANHWGKLQSLRKIKPWQM
PLSQCNEPCAPGYRKAKIEGKPSCCYGCVRCADREMSNTTDAANCERCSEYQKSNTERTE
CLLKAINFLSYTDTMGASLTAIAFILFITASVVLGIFVKYWETPIVRANNQHLSCLLLISLML
CFLCTLLFIGRPTQICCLLRQVTFGIVFTISVSSVLAKTLTVIIAFNATKPGSKLKKYVGTQL
AIVLVIICSLGSTGISAVWMASHPPFLEADTVSEMDTVILLCNEGSVTFFFCIIGYIGTLALLS
FIAAFLAKDFPDRFNEAKNITFSMLGFCSVWGAFVPAYLSSKGSRMVAVEIFAILSSSAGLL
ACIFAPKCYIIFLQPELNKRTL*
>jgi|Xentr4|407367|e_gw1.855.15.1
MISEKIKKWQKYFVFFFHLFILLHFPQVSIQQNNCTDTESFLKKYYLPAAYAIEFVIGLIGNC
IALFGYFFCLKNWSSGSVYLFNLCISDFAFLCTLPLLVFSYTNEEWTFGDFLCKCNRYLLHE
NLYTSILFLAFISIDRYMLIKYPFREHILQRKSTAIMISLGIWILVSLEISPVIIFLKQDIINNVTI
CPDYSSSAHATGSLVYSLCLTFTGFIIPLCVIWIFYLKMMCFLKNRNQQLPNGASFKKPVIIV
VLAATMFSFFFTPYHVMRNLALASQIESWTLSGCSSMIISSAFIISKPIAFLNSMINPVFYFL
MGDHFKEMLLAKIQRIYKSLKSIFNSRWA*
>jgi|Xentr4|407530|e_gw1.861.14.1
MNRTSFNFTDSRIGEWERFAELAKYESDAQKPTVQALLIVAYSVIIVMSLFGNMLVCHVV
MKNKRMHSATSLFIVNLAVSDIMITLLNTPFTLVRFVNSTWVFGKEMCHISRFVQYCSLHV
STLTLTAIALDRHQVILNPLKPRMSLSKGVLCISFIWVMATCFSLPHAIYQKLFRYNYRENK
VRSLCIPDFPEPSDLFWKYLDLSTFLLLYLLPLLIITIAYSRLAKKLWMRNAIGDITTEQYIT
HRKNKKKSIKMLVLVVVVFAVCWFPLNCYVVLISSLGIKTKNALYFALHWFAMSSTCYNP
FIYCWLNESFRLELKSLLSMCQRIPAAQDHALPPVLLPYREAWVEQASFKQGPSSQSIRSTT
NVQTVNTDL*
>jgi|Xentr4|407828|e_gw1.869.2.1
AGCKASWDNITCWRSASVGEVVVMPCPDYFGFFSNVQGNVSRNCTEHGWTDMFPAPYA
IACGFDTNNTTNGQTEFFSMIKTGYTIGHSVSLISLTAAMIILFFFRKLHCTRNYIHMHLFMS
FIMRAIAVFVKDIVLFESGEFDHCFVGSVGCKAAMVFFQYCIMANFFWLLVEGLYLHTLLV
ISFFSEKKYFWWYILIGWGSPTVFITAWTVTRIHFDDSGCWDTIESPFWWIIRAPILISISVNF
IFFICIIRILVQKLHSPDVGRNENSQYTRLAKSTLLLIPLFGVHYIMFAFFPDNFKVEIKLIVEL
VLGSFQGFVVAILYCFLNGEVQGELKRKWRRWHLERFLGSDMKYHHPSLGSNGTNFSTQI
SMLTKCSPKTRRCSSFQAEFSLV*
>jgi|Xentr4|408149|e_gw1.877.86.1
MIVPNGSSFTEFLILGFSPSEKQKVPLFLLFLLIYLFTVISNTLIIALISLNSNLHKPMYFFLCN
MSFLDIFFTSVTAPTLLHMQLSEDKRISFRACMTQLYFYDSFGAMEYMMLTAMAYDRYQ
AICNPLTYHQNMSRNVCYGLAGLSWAGGFATNLLLNILISRFRYCISKQINHFFCDLAALL
KLVCDDTSAVEFLIYAMGGLIVLNCFLLTVTSYVFIVIAIFKIATREGRFKAFSTCTSHLTVVI
LFYGMIALLYLKPLSSNSLNNGKVLFPPEFIHHSYAKPSYIHVKE*RCYCSTKKIYM
>jgi|Xentr4|408153|e_gw1.877.25.1

MYLSLSSSSSSLLWQGDSTILIQNKSGAAGFLIVGFSDSPELQGPLFLFFLLIYLVTVWGNLL
LISLISTDSHLQTPMYFFLCNLSCLDISYSSVTAPYLLHIFSTGKNYIPFTMCMVQLYFFSSLA
TMEYMLLTTMAYDRYVAICRPLFYPQVMNQRTCILLAAAAWIGGLLAGAPLVILISALTYC
KSNIINHFFCDITPLLKLSCDDTSTVETVMFLQGASLLFSCFILTMMSYIYIISAILKITSAEGR
YKTFSTCGSHLTSVTLFYVLLEKSSQFFM*MSYPC*TQSYTA*GTEMRKKLLLNFQGDGST
>jgi|Xentr4|408154|e_gw1.877.17.1
MPEVVPNGSSFTEFLILGFSPSEKQKVPIFLLFLLIYLFIVISNTLIIALISLDSNLHKPMYFFL
CNMSFLDIFFTSVTAPTLLHMQLSEAKRISFRACMAQLYLFNSFGAMEYMVLTSMAYDRY
QAICNPLVYHQTMSRNVCCILAALPWGGGFISAIPINILISALCYCSSNRINHFFCDLTALLM
LACNDTSTVEFVIFAEGVLIVLNCFLLTVTSYGFIGFAIIRIGSSKGRFKAFSTCASHLTVVIL
FYTMIACLYMKPSSSYSLSDGKFLSVFTVNVTPMLNPVIYTLRNKDIREAVKNIYHFFYVK
WLSL*
>jgi|Xentr4|408164|e_gw1.877.20.1
MENKSGAAGFLIVGFSDSPELQGPLFLFFLLIYLVTVWGNLLLISLISTDSHLQTPMYFFLS
NLSCLDISYSSVTVPYLLHIFSTGDNNISFTKCMTQLYFFTSFTVTENMLLTTMAYDRYVAI
CRPLFYPQVMNQRTCILLAAAAWIAGAPLSVLISALTYCK*NIINHFFCDLTPLLKLSCDDT
STVETVMFLQGASPLFSCFALTMMSYIYIISAILKITSAEGRYKTFSTCGSHLTSVTLFYVLL
FCVYMKPVCL*SLDEGKVVSVLYVNVIPMLNPIIYSLRNKDVKKALQELPRRWFHCMPRA
ILGTL*
>jgi|Xentr4|408180|e_gw1.877.36.1
LLLVGYSLLMPVGVLLNGVSLYVFIRFMRPLSVVGIFLCNLALSDLLFSLSLPLRIYYYANH
YWPFGTFLCSFNGSIFHINMYGSCLFLLCINVDRYLAIVHPLRFRHLRRRKVARLACVAVW
VLIVGGSVPAAIVHSSSDCLLHGEKVSRCFEAFSTWDKGVLPLLVVAEVLGFLLPLAAVLY
GSCHIFVELYRAQEIGQGRHGKTIKLLCLNLVIFLFCFVPYNTTLVGYGLLRANLVEDTWNI
KPTLKKIISVTVLFASTNCALDPLIYYFSTEGFRRTLSRMSGHKTQKFISNQETKSTSATDSS
MKPSSRTISRATEAPKLKTNETNGKEGKNLIEESEI*
>jgi|Xentr4|408183|e_gw1.877.72.1
MSSPSPFLRQIESPRLTENHSRADGFLILGFSDSPELQGPLFFIFLLIYLMTVWGNLLIISLVFT
DVYLQKPMYFFLCNMSFLDISYSSVTAPYLLHIFSTGDNNIPFKMCMVQLYFFNSFASMEY
LLLTTMAYDRYMAICRPLFYPQVMNQRTCRILAAAAWIGGFLAGTPVTILISALTFCASNII
NHFFCDVTPLLKLSCDDTSTVEFVMFAQGVFLLFSCFVLTVMSYIYIISAILKITSKEGRSKT
FSTCGSHLTVVTLFYVLVVCVYMKPTSSYSLDEGKFLSVLYVNFIPMLNPVIYSLRNEDVK
KVLQKKFRGNIKKRI*
>jgi|Xentr4|408184|e_gw1.877.64.1
MHSSSSSSTSSSRFLLHLESAPLVHNSSADGFLIMGFSDSPELRGPLFLIFLLIYLVTLLGNLII
ITLISTNAHLQKPMYFFLCNLSCLDISYSSVTAPYLLHIFSTGDNNISFTMCMVQLYFFSSLA
TMEYMLLTTMAYDRYMAICKPLFYPQVMNQRTCILLAAAAWIGGFVSATPVTILISTLNY
CSSNIINHFFCDLTPLLKLSCDDTSTIELIIFIEGFFLLFGCSVLTMMSYLFIISAILKITSTQGR
YKTFSTCGSHLTSVTVFYVLMISVYLRPASVYSLAESKFISVLYMNVIPMLNPIIYSLRNKD
VIKALKAISGNAQQMNY*
>jgi|Xentr4|408201|e_gw1.877.19.1
MYFSSSSRFWQGDSAILIQNKSGATGFLIVGFSDSPELQGPLFLFFLLIYLVTVWGNLLLISL
ISTDSHLQKPMYFFLCNLSCLDISYSSVTAPYLLHIFSTGDNSISFTMCMVQLYFFSSLATME
YLLLTTMAYDRYMAICKPLFYPQVMNQRTCILLAAAAWIGGLLAGAPLSVLISALTYCKS

NIINHFFCDITTLLKLSCDDTSTVETVIFLQGATFLFSCFALTMMSYIYIISAILKITSAEGRYK
TFSTCGSHLTSVTFFYVLLACVYMKPASSYSLDEGKVLSVLYVNVIPMLNPIIYSLRNRDV
KKALNALPRRWFY*
>jgi|Xentr4|408222|e_gw1.877.18.1
MENKSGAAGFLIVGFSDSPELQGPLFLFFLLIYLVTVWGNLLLISLISTDSHLQKPMYFFLC
NLSCLDISYSSVTAPYLLHIFSTGDNYIPLTMCMVQLYFFSSFTSMEYLLLTTMAYDRYMAI
CRPLFYPQVMNQRTCILLAAAAWIGGFLAAAPISILISALTFCKSNIINHFFCDVTPLLKLSC
DDTSTVEYVMFAQGVFLLFSCFALTMMSYIYIISAILKITSAEGRSKTFSTCGSHLTAVTLFY
VLLVCVYMKPASSYSLDEGKVVSILYVNFIPMLNPMIYSLRNKDVKKALKQLPRRWFYC
MPRAVF*
>jgi|Xentr4|408226|e_gw1.877.6.1
MFSSSSSLWQGDSAILIQNKSGAAGFHIVGFSDSPELQGPLFLFFLLIYLVTVWGNLLLISLI
STDSHLQKPMYFFLCNLSCLDISYSSVTAPYLLHIFSTGDNNISFTMCMVQLYFFGSFATME
YLLLTTMAYDRYVAICRPLFYPRVMNQRTCILLATAAWIGGFLSGAPVTVLISALTFCNSNII
NHFFCDVTPLLKLSCDDTSTVETVVFLQGVFLLFSCFILTIISYIYIISAILKMTSTEGRHKTF
STCGSHLTALTLFYVLLACVYMKPASSYSLDEGKVLSVLYVNVIPMLNPIIYSLRNKDVKK
AFTELRRRWFC*
>jgi|Xentr4|408386|e_gw1.881.45.1
MFPFFVLLNECNSKTVNVKLFLSSLSDHQSNQLILFLTFPFLYIITLIGNLLILFIIITDTYLHSP
VYFFLGNLVLLDLCCSSITTPRMLSDLNTRNHMVSLSACITQIYFFISFATSEITLLAVMSYS
RHVAVCYPLHYMQRMTWKACVQLAMGVWLLGFLYSLLLTLFTMSLTFCDSNTVHSFFCD
LTDFLH*HLHQHCNHFYFRGIP*IRFFHKNLFFSTSVYLPLC*AISTEEAK*KHFPHVHPTSQ
WLSSSMELFILTTKWFLFFTLLQYIKPYHLQS*ERGAQESCKKTTSM
>jgi|Xentr4|408412|e_gw1.882.108.1
MLRENQTLINELYLVGFGDLHNFNIILFAVVLIIFIMALMSNDLVIILVASCKSLHSPMYFFLT
QLSLSEVLFTSNIVPNMLRLILAGGGTMSVNGCITQFYLLCAPTIAQCLLLAAMSFDRYVAI
CKPLHYTSVLTFRLQLHIVMFCWTSGFTLALPIDIFLHKLQFCHSNVINHFYCDIAPVLELS
CSDTSSVKLLTSLVSMPVVLLPFTFIIATYISIIFTILRIPSNHGRQKAFSTCSSHLIVVSMYYG
TLTTIYIVPTGEHSMNANKVLSLLYSLVTPLFNPIIYSLRNQDIRRAISNCNIVKRKTIFSHVY
*
>jgi|Xentr4|408421|e_gw1.882.103.1
ENQTSITEFFLLGFGDLHNFRMFFFIMFLLIYIVAFAGNLLVILLVATHHALHSPMYFFLSQLS
LSEILFTTNIVPNLLHLILQGGANMSVTNCMVQFYLLCVPTIAQSLLLAAMSFDRYAAICNP
LHYMSVMTFKLQLYIAAYCWASGVLTALLAFAFLHPLVFCLANVINHFYCDIAPIIELSCSD
TSIVELVVSLDSMFVFFSPFVFIIGTYVSILRTILRIPSGCGRQKAFSTCSSHLSVVFVYYGTL
TAIYLYSPGKHSLNANKFLSLLYIVVTPLFNPIIYSLRNQDIKRAIRKCVLGKVTGIT*
>jgi|Xentr4|408446|e_gw1.882.61.1
MNTTSSCNQSKITEFLLVGFSAPRPLRELLFSICLVIYIMALGANLMIIALYLGSHHLRSPMY
FFLSNLSATDISLSTTVGPNLLCTLLKHGKLMSVSTCVTQFFAYGFFTGVECLLLTVMAYD
RYLAICKPLHYVTIMTNKHCLHLVIWCWVEMLLISLSSTILTSILGFCGCNTLDHVYCDFAP
LLKVSCSDAFVMASMTTIFIPTGIVFLFLSPLLFIITTYVFISLSIFRISSKTGRQKVFSTCGSH
LTVVGTYYGVLISKYAVPSTGQSENLNKLTSLLYTLVTPLFNPIVYSLRNQDIQKTMTALIS
VRLRQKRENM*
>jgi|Xentr4|408457|e_gw1.882.77.1

MNQTSVLDIVIIGFEGPQDFRIALSAILFLIFLMILGGNSLIVVLVTISRRLHSPMYFFLCHLAI
TDIIVPSNIVPNLLYVTLLDRGTISLSNCLAQFFFFGMAANAECLILTVMSYDRFLAICRPLH
YRSIMTLKFQILLTSNCWLLAFTLALLVVAHMLTFQFCGSNVINHLFCDFHPLLKLSCSDTF
VVDMEVLVTGILIVVLACVFIVVTYIWIFRTIFGSSMTTGRQKAFSTCTSHLTVVCTYYATL
VTNYLVPLTGRTTYAAKYISLLYTVITPLMNPIIYSLRNQEIKSVLNSYIC*
>jgi|Xentr4|408459|e_gw1.882.69.1
MNTTSSCNHSKITEFLLVGFSAPRPLRVLLFSICLVIYIMALGANLMIIALYLGSHHLRSPMY
FFLSNLSATDILLSTTVGPNLLCTLLKDGNPMSVSACVTQFFAYGFFTGVECFLLTVMAYD
RYLAICKPLHYVTIMTNKHCLHLVIWSWLEIILFLLPITVMISHPGFCGCNTLDYIYCDTAPL
LKLSCSDVFVVERAITVVIIPVAVIPLLFILTTYVIILLSIFRISSNTGRQKAFSTCSSHLTVVSM
FYGILIAKYTVPSKGLSLNIKKIISLLYTLVTPLFNPIIYSFRNQEIWRALSRWVSVKVSSQRP
ILP*
>jgi|Xentr4|408465|e_gw1.882.109.1
MKDMNQTSVLDIVIVGFEGPQDFRIVLSAILLLLFLITVGGNSLIVVLVAISRRLHSPMYFFL
CHLAITDIIVPSNIVPNLLYVTLIDTISLSNCLTQFFFFGMAANAECLILTVMSYDRFLAICRP
LHYKSIMTLKFQILLASNCWLLAFTLSLLVLSHMLTFQFCGSDVINHFFCDLHPLLKLSCSD
TFVVDMEVLVTGILVIVLACVFIVVTYIWIFRTILGTSMTTGRQKAFSTCSSHLTVVCTYYA
TLVTNYLIPLTGRSTNLSKYISLLYIVITPLMNPIIYTLRNQEIWFALNSIISTRPI*
>jgi|Xentr4|408468|e_gw1.882.64.1
MQEWNQTIITELFLVGFGDLHRSKYFFFCMFFLLYLVALKGNLLVITLVIINEALHLPMYFF
LSQLSLSEIIFTSNMVPTILRLILSGGGNMSVSGCKIQFFALCVPTLTQCLLLAAMSFDRYVA
ICNPLHYTSIMTFKLQLWVAIFCWVCGFSACVLVIVFIYQLQFCRSNVINHFACDIGPVMEIS
CSDTSRMELVTSLVSTVSIAFPFLFIIGSYISIILTILRIPSSFGRQKAFSTCSSHLAVVCMYYGT
LVALYIYPFGKHSANAKFIFILYTSVTPLFNPIIYSLRNQDIKRAIRKLVSICKKKIDV*
>jgi|Xentr4|408477|e_gw1.882.2.1
MSQTNQTSITEIVLLGFEGPVNIRVIMFILLFVIYMGSLGGNSLIILLVSLSQRLHSPMYFFLC
HLSVTDIIISSSVVPNMMYFSLQGRGIISFSNCLTQLFFFGMAIGVECLILTVMSYDRYLAIC
RPLHYNSIMGLRLQSLLVTFSWLVGFSAPFIIACSIPTLDFCGPNIINRFFCDTGCFLELSCSD
TFLINSELLLTGYPMTVIAFMFIIVTYVNIFRMIFGSSITIGRQRAFSTCSSHLTVVCAYYGSLI
INYMIPIKGDTIIINKYLSLLYTVMTPLFNPIIYTLRNKEIRLAFNYYLQRSTVT*
>jgi|Xentr4|408488|e_gw1.882.75.1
MQEGNHTLIMEFFLLGFGDLCGLKYLCFTFFLLFYLVSLMENLLVIILVIVTEALHCPMYFF
LSQLSLSEILFTTNIVPNLLRLVLFGGGNVSVLGCKIQFFVLCVPTVTQCILLAAMSFDRHA
AISNPLHYTSIVTFKVQLHVVIFCWLSGFMVPLVVFVFFNQLQFCRSNIIDHFYCDISPVME
LSCSDTSTLELVTSLASTLAILSPCFFIIGTYISIIVTILRIPSSCGRQKAFSTCSSHLAVVCMYY
GTLISMYIYLPGNHSVNANKYLSLLYTFVTPLFNPIIYCLRNQNIRQVIRKYFIFVGMGGAL
KL*
>jgi|Xentr4|408520|e_gw1.882.20.1
METNATSVTYFFLVGFGELDGFRVVFFLVFLALYLMAFNANILVIILVVACQTLHFPMYFFL
GQLSLSEILFTNNIVPNMLRLILIGGGTMSVRGCITQFCLLCVPTTAQCLLLATMSFDRYVAI
CNPLHYTSIMTFKLQLNIFTFCWLFGFMLALVICIFLNRLGFCHSNTINHFYCDIAPVLALSC
TDTSHVEMLMTLSSVFVVFSPFIFIIVTYVFIVVTILRIPSTFGRQKAFSTCSSHLIVVCMYY
GTLTLIYIYPPGNHSLNANKFLSLLYTLVTPLFNPIIYSLKNQDIRRVIYKYLSKCREQLV*
>jgi|Xentr4|408522|e_gw1.882.8.1

MSSCNQSKITEFLLVGFSAPRPLRELLFSICLVIYIMALGANLMIIALYLGSHHLRSPMYFFL
SNLSATDILLSTTVGPNLLCTFFTYGKLMSVSACVTQFFASGFFTGFECLLLTVMAYDRYL
AICKPLHYVTIMTNKHCLHLVIWCWVEILLLSLPATILTSVFGFCGCNTLDHVFCDFAPLLK
ASCSDAFVMASMATLLIPAGVVLLFLSPLFIIITTYVSISLSIFRISSKTGRQKAFSTCGSHLTV
VCIYYGILISKYTVPSKGQSGNVNKLTSLLYTLVTPLFNPIVYSLRNQDIQKTMTALISVRLR
QKRENI*
>jgi|Xentr4|408544|e_gw1.882.15.1
MQEQNQTSVTHFFLVGFGELHSMKMMFFSMILIIYIMVFNANFLVIILVAACPTLHFPMYFF
LSQLSLSEILFTTNIVPNMLRLILIGGGTMSVRGCIAQLCLLCVPTITQCLLLATMSFDRYVA
ICNPLHYTSIMTFKLQLNIVTFCWISGFMLSFAIYIVSSRLIFCHSNKINHFYCDIAPLIALSCS
DTTYVELVVALISAFLAFSPFLFIIITYIFILANILKIPSSFGRQKAFSTCSSHLAVVCMYYGTL
TVIYLYPPGNHSLSINKFLSLLYTLVTPLSNPIIYSLRNHDIRRVVHNFLSKCRNNACGVGFP
RL*
>jgi|Xentr4|409214|e_gw1.894.58.1
MLLFRPTYSPFKILIYLFAQCITPSGAEPIKAACYLKPATFLEDFEYIQDGDIIIGGLLSVNTN
AISLPYENEQNSNNVTVCTDIECQYYRQLVEFRLAIEEINKNPSLLPNVTLGYHIFNSCGNEI
KAAMSILRILSGTKEPVPNFFCGRKRKIAGFIGDLTSETTMISAQILSLFGFSQISFGASDPSL
SDRVAFPYFFRTKQSFHGSALAMSKLMKHFGWTWVGIIGVNDSDMDDEPQVLTDYLSRD
GICVEFTIKMSRYLDEFLDGGIDRIGKIIKKSTTNIIIVCGKAYKVIVVVVYLRRMLMPINKT
FVFSPSASVIYNNVDFITTVFDGSFIFEPYPVYPRETHEIIDFINRTHPSKDPNDKLFENIMLT
VCQCLSKDKIKNKVYKDLYNAHYVECNSKRTIKCVSYITSSLLAALSPNVHLAVNIMSQAI
HEMHTSLREQSPERDREAHRYQYQLHHYLNKSHYQTKYGGEVSFDGRGEMDTGYIIYFF
PNGTDATYLGTLGRFIPSAPSEYKLIIYPDATPWKMKNKTIPRSQCTDSCQPGYRKAVNPG
AQSCCYGCLPCSEGEISNRTDSENCIRCPDLEWPNDKRNQCIAKMVEFLSFTDGAISASLLS
TTVLFFLITLLILGIFISYRDSPIVRANNRSLSFLLLVSIKLSFLSVFLFLGRPVDITCMLRIITF
GITFSIAVSSLLAKTIMVCVAFKATKPGSSWRKWLGVKLSNSVVLFCSSIQIIICMTWLAISP
PF
>jgi|Xentr4|409230|e_gw1.894.57.1
MLGFRPTHNPFQILIHLLVLCIRQSETEPIKPACCLKPARYWEDFEYIQDGDIIIGGLLTVNTK
TMRHQQEFKRKRKRACVELQPQYYRQLVEFRLTIEEINKNPSLLPNVTLGYHIYESCGNG
MKAVRSILQILSGTKEPVPNYSCGRKRNIAGFIGDFTSETTMISAQILSLFGFSQVSFGTSDP
SLSDRVAFPYFFRTLQSFHGSALAISKLLKHFGWTWVGIIGLNEGDMADEPQVLTDYLSRD
GICVEFTLNANRYLTKLTGREGNRMYDIIRNSTTHIIIVCGKAVNAFLFLRCLRSVHIVDNK
TLILSPSALVMGNYMKFTDTTFDDILIFEPYPVYPRETHEIIEFIYSIHPYKDPKDKLFENIML
TVCECLVKDPHKNKVYESFSVMKNAECDGKEATECLSNIISSLLAALSPNVHLAVNIMSQ
AIHEMHTSLREQSPERDREAHRYQYQLHHYLNKSHYQTKYGGEVSFDGRGEMDTGYIIY
FFPNGTDTTYLGTLGRFIPSAPSEYKLIIYPDAIPWKMMNKTVRNPFLLLINNKMFPELIVA
AHPPTYYCIIGRCRCPPLVLALSQNSENCIRCPDLEWPNDKKNQCIAKTEEFLSFTDGAISA
SLLSITVLFSLITLLMLRIFILNRDSPIVRANNRSLSFLLLVSIKLSFLSVFLFLGRPVDITCML
RIITFGITFSIAVSSLLAKTIMVCVAFKATKPGSSWRKWLGVKLSNSVVLFCSSIQIIICMTWL
AISPPFQELDIHTSPGTIIIQCNEGSAIGFYSVIGYMGLLAAVSFVLAFLARSLPDSFNEAKYI
TFSMLLFCSVWITMIPAYLSTKGKNTVCVEIFAILTSSAGLLACIFLPKCYIIMFRPEMNQKS
CLLGSKT*
>jgi|Xentr4|409380|e_gw1.899.100.1

MASESQINVSGFIIQGFSDTPELQISLFVLFLGIYLIILLGNLIIFLVISCNPHLHTPMYIFLLNL
SLIDISFSSNVLPNLLHILLTQQNNISFLGCMTQMYVFVSLAGSEYFLLTAMAYDRYVAICD
PLHYIARMSRKHCAGLITAAFTVGFVEPVGHLVLIPKLSYCASHLISHFFCDITPLVKLSCSS
TFSVELLIYIEGTLLIISSFLLTLISYIFIISAILKIQSSEGRQKAFSTCASHLACVITLYGTALSL
YMRPTTSYSPKRDKYFSLLYIALGPVLNPLIYTLKNREFQASFTKVRQRLLAFLFWIRLAK
KVKIST*
>jgi|Xentr4|409381|e_gw1.899.75.1
MVSESQENVSGFIIQGFSDTPELHISLFVLFLGIYLIILLGNLIVFLVISCNPHLHTPMYIFLQN
LSLIDISSTSNILPNLLHILLTQQNNISFLGCMTQMYVFASLAASEYFLLTAMAYDRYVAICD
PLHYIARMSRKHCAWLITAAFTVGFVDNVTYLFHISKLSYCASHLINHFFCDVTPLLTLSCS
STFSVELSIYIEGTFLIFGSFLLTLTSYIFIISAILKIQSSEGRQKAFSTCASHLACVITLYGAVFC
LYMRPTTSYSLERDKCFSLLYIVLGPVLNPLIYTMKNREFQSTFNKVRQRCLAFLF*
>jgi|Xentr4|409399|e_gw1.899.62.1
MVSGSKENVSGFIIQGFSDTPELHISLFVLFLVIYLIILLGNLIIFLVISCNPHLHTPMYIFLLNL
SLIDISSTSNILPNLLHILLTQQNNISFLGCMAQMYVFGFLADSEYFLLTAMAYDRYVAICDP
LHYIARMSRKHCAGLITATFTVGFFDPVPYLVLISKLSYCSSHLINHFFCDVAPLLKLSCSST
FSV*LLNYLVGTLLVFNSFLLTLTSYIFIISAILKIQSSEGRQKAFSTCASHLACVITLYGTGIC
LYMRPTTSYSLKRDKYFSLLYIALGPVLNPLIYTLKNRDFQSSFKKVRQRFLAFFYFR*
>jgi|Xentr4|409401|e_gw1.899.99.1
MASESKENVSGFIIQGFSDTPELQTSLFVLILGIYLIILLGNLIIFLVISCNPHLHTPMYIFLQN
LSLIDISSTSNILPNLLHILLTQQNNISFLGCMTQVYFFASLAGSEYFLLTAMAYDRYVAICDP
LHYIARMSRKHCAGLITAAFTGGFVDTIGHVTLISKLSYCASRLINHFFCDITPLLKLSCSST
FSVELLIFIEGTLVVFNSFLPTLASYIFIISAILKIQSSEGRQKAFSTCASHLACVITLYVTVFFL
YMRPTTSYSLKRDKYFSLLYIALGPVLNPFIYTLKNREFQSSFNKLRQRCFAFYFY*
>jgi|Xentr4|409405|e_gw1.899.38.1
EESKENVSGFIIQGFSDTPELHISLFVLFLGIYLIILLGNLIIFLVISCNPHLHTPMYIFLQNLSLI
DISFPSNILPNLLHILLTQQNNISFLGCMTQMYLFMALASSEFFLLTAMAYDRYVAICDPLH
YIARMSRKHCAGLITAAYTISFVEPFGPVVLISKLSYCASHLINHFFCDLSPLLKLSCSSTFIA
EFFIYIEGTFLIFSSFLLTLTSYIFIISAILKIQSSEGRQKAFSTCASHLACVITLYGTVLCLYMR
PTTSYSLKRDKYFSLLYIALGPVLNPLIYTLKNREFKSSFNRIHFIYFR*
>jgi|Xentr4|409416|e_gw1.899.111.1
MSSEFKENVSGFIIQGFSDTPELHISLFVLFLGIYLIILLGNLIIFLVISCNPHLHTPMYIFLLNL
SLIDISFTSNILPNLLLILLTQQNNISFLGCMTQVYVFVALASSEYFLLTAMAYDRYVAICDPL
HYIARMSRKHCAGLITAAFTVGFGEAVGFIVLIPKLSYCASRLINHFFCDVPPLLKLSCSSTF
SVELLTYIDGTLLAFNSFLLTLISYIFIISAILKIQSSEGRQKAFSTCASHLTCVITLYGTGFCLY
VRPTTSYSLERDKYFSLLYIALGPVLNPLIYTLKNREFQSSFNKVKQRCLAFYFR*
>jgi|Xentr4|409418|e_gw1.899.103.1
MASGTNGNVSGFIIQGFSDTPELQIAFFVLFLGIYLIILLGNLIIFLVISCNPHLHTPMYIFLLN
LSLIDISFSSNILPNLLHILLTQQNNISFLGCMTQIYVFVALASSEYFLLTAMAYDRYVAICDP
LHYIARMSRKHCAGLITAAFTVGFGGTVGIVVLVSKLSYCASRLINHFFCDVTALLKLSCS
STFSVELYIFILGALLIVNSFLLTLASYIFIISAILKIQSSEGRQKAFSTCASHLACVITLYGTAL
SLYVRPTTSYSLQRDKYFSLLYIVLGPVLNPLIYTLKNREFQSSLNKTRQRCLYFFHF*
>jgi|Xentr4|409448|e_gw1.899.16.1
MVSGSQQNVSGFIIQGFSDTPELQTSLFVLILGIYLIILLGNLIIFLVISCNPHLHTPMYIFLLN

LSLIDISFPSNIFPKLLHILLTQQNNISFLGCMTQLYVFGFLAASEFFLLTAMAYDRYVAICDP
LHYIARMSRKHCAGLITAAFTVGFVDPVAYLVLIPKLSYCASRLINHYFCDVTPLLTLSCSS
TFSVELLNYLVGTLLGFSSFLLILISYIFIISAILKIQSSEGRQKAFSTCASHLICVITLYGTVFC
LYMRPTTSYSLERDKYFSLLYIALGPVLNPLIYTLKNREFKFSFNKVRQRFLALFILDKC*
>jgi|Xentr4|409467|e_gw1.899.31.1
MDSESKENVSGFIIQGFSDAPELKISLFVLFLGIYLIILLGNLIIFSVISCNPHLHTPMYIFLLN
LSLIDISFTSNILPNLLHILLTQQNNISFLGCMTQMFVFVSLAGNEYLLLTAMAYDRYVAICD
PLHYIARMSRKHCAGLITAAFTVGFGESVGLIVLISKLSYCASHLINHFFCDITPLLKLSCSS
TFIVELFIYIDGTLLPFNSFLLTLTSYIFIISAILKIQSSEGRQKAFSTCASHLACVINFYGTLM
GMYMRPTTSYSVKRDKYFSLLYIVLGPLLNPFIYTLKNREFLSSINKVRQRYLAFLILEKC*
>jgi|Xentr4|409470|e_gw1.899.23.1
MVSESKENVSGFIIQGFSDTPELQTSLFVLFLGIYLIILLGNLIIFLVISCNPHLHTPMYIFLLN
LSLIDISFASNILPNLLHILLTQQNNISFLGCMTQLYVFGSMVGSEYFLLTAMAYDRYVAICD
PLHYIARMSRKHCAGLITAAFTGGFGSTVSFVVLMSKLSYCASRLINNFFCDVAALQKLSC
SSTFSVELSIYIEAILLVFNSFLLTLTSYIFIISAILKIQSSEGRQKAFSTCASHLACVITLYGTLL
CLYMRPTTSYSLERDKYFSLLYIALGPVLNPLIYTLKNREFQSSLNKIRQRLFIL*
>jgi|Xentr4|409517|e_gw1.900.87.1
MSNLSGSNQMSSPFILVGIPGMEEMHPWISVPLCCMYIVTISANVSVLLIIRADRRLHQPM
YLLLSMLLLTDLVQSNAALPKMLLIFWFKLREITFEECLVQMFFIHSFPLMGSSVLLTMAFD
RYVAICHPLRYSTILTNSMIVKMGLFVIIRGILVEFPLPFLVRMLPFCGSRVIQHTYCEHMAV
AKLSCGDIRINPLYGLVSLVMAALDLICVAGSYTMIIMAVLRLPNREARYKVGNTCSAHVG
LILIAYIPALFSFISQRIEVSSALSVQILVSSLYLIIPPMCNPIIYGIKTQEIRKKVSNLISLPAKGS
VKDMRFPSV*
>jgi|Xentr4|410080|e_gw1.913.82.1
MTSESKGNISGFIIQGFSDTPELQFSLFVLFLGIYLIILLGNLIIFLVISCNPHLHTPMYIFLRNL
SLIDISSTSNVLPNFLHILLTQQNNISFLGCMTEMYVFVFFGASEYFLLTAMAYDRYVAICDP
LHYIARMSRKHCAGLITAAFTVGFVDPVGIVVLTSKLSYCASRLINHFFCDVPPLLKLSCSS
TFSVELLIYIEGTLFTFSSFLPTLISYIFIVSAILKIQSSEGRQKAFSTCASHLACVITLYGTVFC
LYVRPTTSYSLERDKYFSLLYIALGPVLNPLIYTLKNREIQSSLNKMKQKWYSNLIFVLAN
VKQ*
>jgi|Xentr4|410081|e_gw1.913.74.1
MASESKENISEFIIQGFSDTPELHISLFVLFLGIYLIILLGNLIIFLVISCNPHLHTPMYIFLLNLS
LIDISSTTNILPNLLHILLTQQNNISFLGCMTQMYVFASLTGSEYFLLTAMAYDRYVAICDPL
HYIARMSRKHCAGLITAAFTVGFGESFGLVVLIPKLSYCASRLINHFFCDVTPLLKLSCSST
FSVELVIYIVGPFLVFNSFLLTLASYIFIISAILKIQSSEGRQKAFSTCASHLACVITLYGTALSL
YMRPTTSYSLERDKYFSLLYTVLGPVLNPLIYTLKNGEFQSSLHKIKQRCFNFFILC*
>jgi|Xentr4|410087|e_gw1.913.41.1
MASGTKENVSGFIIQGFSDTPELHISLFLLILGIYLIILLGNLIVFLVISCNPHLHTPMYIFLLN
LSLIDISSTSNILPNLLHILLTQQNNISFLGCMTQTYVFASLVDSEYFLLTAMAYDRYVAICDP
LHYIARMSRKHCAGLITAAFTVGFINTVGLVELISKLSYCASHLINHFFCDVTPLLKLSCSS
TFSVELFIYIIGILLVFNSFLLTLISYIFIISAILKIQSSEGRQKAFSTCASHLTCVITLYGTVFSLY
MRPTTSYSLERDKYFSLLYIVLAPALNPLIYTLKNREFQSSLKLYVNRKKKFLPNKRNHNS
KQI*
>jgi|Xentr4|410090|e_gw1.913.25.1

MTSESKENVSGFTIQGFSDTPELQISLFVLFLGIYLIILLGNLIVFLVISCNPHLHTPMYIFLLN
LSLIDISFSSNIFPNLLHILRYQQNNISFLGCMTQMYLFASLASSEYLLLTAMAYDRYVAICD
PLHYIARMSRKHCAGLITAVFTGGFGATASFVVLISKLSYCASRLINHFFCDILPVLKLSCSS
TFSVELFIFIEGTLLLFNAFLLTLASYIFIISAILKIQSSEGRQKAFSTCASHLACVITLYGTVIC
LYMRPTTSYSLKRDKYFSLLYIVLGPVLNPLIYTLKNREILSSLNKMKQKMV*
>jgi|Xentr4|410103|e_gw1.913.57.1
MTLECKENVSGFIIQGFSETPELHISLFVLFLGIYLIILLGNLIIFLVISCNPHLHTPMYIFLQNL
SLIDISFSSNILPNLLHILLTQQNNISFLGCMSQMYVFVALAASEYFLLTAMAYDRYVAICDP
LHYIARMSRKHCAGLITAAFAGGFVDTVAHLVLISKLSYCASHLINHFFCDMAPLLQLSCS
STFSVELIFYIDGTLIFFNAFLLTLISYIFIISAILKIQSSEGRQKAFSTCGSHLACVITLYGTAF
CLYMRPPNSYSLKRDKYFSLLYIALGPVLNPLIYTLKNREFQSSLNKMRQRCLSLFYF*
>jgi|Xentr4|410110|e_gw1.913.24.1
MASESKENVSGFIIQGFSDTPELQTSLFVLFLGIYLIILLGNLIVFLVISCNPHLHTPMYIFLLN
LSLIDISSTSNIFPNLLHILLTQQNNISFLGCMTQMFVYGFMAASEYFLLTAMAYDRYVAIC
DPLHYIARMSRKHCAGLITAAFAIGFLDPICIVVLVSKLSYCASRLINHFFCDVTPLLKLSCS
STFSVELLMYIEGAFLIFSSFLLTLTSYIFIISAILKIQSSEGRQKAFSTCASHLACVITLYGTVF
CLYMRPTTTYSLKRSKFFFLLYIVQGPVLNPLIYTLKNREFLSSLNKMKQKCFRLFYFYVS*
>jgi|Xentr4|410112|e_gw1.913.60.1
MTSESKQNVSGFIIQGFSDTPELHISLFVLILGIYLIILLGNLIVFLVISCNPHLHTPMYIFLLN
LSLIDISFPSTVLPNLLHILLTQQNHISFLGCMSQLYVFGSLAGSENFLLTAMAYDRYVAICD
PLHYIARMSRKHCAGLITAAFTVGFLEPVGLVVLISKLSYCASHLINHFFCDFTPLLKLSCN
STFSVEFLIYIEGTFLLFNAFLLILTSYIFIISAILKIQSSEGRQKAFSTCASHLACVITLYGTVI
CLYMRPTTSYSLERDKYFSLLYIALGPVLNPLIYTLKNREFESSLNKLKRRCLSFLFFVS*
>jgi|Xentr4|410127|e_gw1.913.68.1
MTSESKENVSGFIIQGFSDTPELHISLFLLFLGIYLIILLGNLIIFLVISCNPHLHTPMYIFLQNL
SLIDISFSSNILPNLLHILLTQQNNISFLGCMTQMFVFALLAGSEYLLMTAMAYDRYVAICDP
LHYIARMSRKHCAGLITAAFTVGFLEPVGLVVLMSKLSYCASHVINHFFCDITPLLKLSCSS
TFSVELVIYIEGTLLLFNSFLPTLISYIFIISAILKIQSSEGRQKAFSTCASHLTSVLTLYVTAICL
YMRPTTSYSLKRDKYFSLLYTVLGPVLNPFIYTLKNREFLSSLNKIKQKWFSILIFVLANTK
Q*
>jgi|Xentr4|410151|e_gw1.913.50.1
MASESQENVSGFNIQGFSDTPELHISLFVLFLGIYLIILLGNLIIFLVISCNPHLHTPMYIFLLN
LSLIDISFPSNILPNLLHILLTQQNNISFLGCMTQMYVFVALAATEYFLLTAMAYDRYVAICD
PLHYIARMSRKHCAGLITASYTVGFVHSVAYLVLISKLSYCASHLINHFFCDMAPLLKLSCS
STFSVELFIYIEGTLLGFSSFLLTLTSYIFIISAILKIQSSEGRQKAFSTCASHLTCVITLYGTLFC
LYLRPTKNYSLQRDKYFSLLYIALGPVLNPLIYTLKNREFQSSFNKMRQKSLFISRKY*
>jgi|Xentr4|410228|e_gw1.915.37.1
MHLQNESLVFLTSIQKCGQHPDMTPFLGTAYSLVTILGLLGNISLMYVICRQKERGNVTHIL
IANLAFSDVLVCAFCLPFTAVYTVMDYWVFGLSLCKITNFLQCASVTVSVLILVLIAFERYQ
LILHPTGWKPSVLQAYAAVLLVWVIASLLAFPLASSMVLTDTPNKNISKVIGFLADKSVCV
ESWSSNQQLVAYTISLQLLQYIVPLCFILGCYLRISVHLKHRGAMFGRNDNHMRRVNLMF
VIMVGAFAVCWLPFHIFNIIVDWHHQLIPVCYHNLIFSLCHLLAMISTCVNPVIYGLLNSNV
KREVKALVQNCTGRKLDTRSAVEEPERYPLSLKQSATLPGSPSICLEGGTFLGQTLSTEEH*
>jgi|Xentr4|410495|e_gw1.918.43.1

MLIYIIILSMAFCRSEPTNPACRLQIIKAVEEYEYIQEGDIMIGGVMTAHLNVFNITFPWDNS
TRFLCIDPVQEYVKFLMDFRYAIEQTNKDPARLPNLTLGYHIYDSCGDPRKAVRSVLQILSG
TRELVPNYSCVGKRNIAGFIGDLTSETTIPIAQILTLYGYSQISYGATDPELSDRITFLYLFRTV
PSDESYYSVMSKLLSHFGWNWVGIITSDDISGEREHQILAKYLSREGVCIKFTLRINKVRS
NRFHWEKDSAIIQENSTNVIILCGTVSVNLILQEKQFNNERAFQDKTLILSPSWGSTEDILY
YEAELFNCSLVLVPGYHYDLDIPEMRSFLENSHPSNYAGDKLLEDIWIIYFYCLSEDQNKN
DLYKYIYNYIRTLYNCTGQERITDRDYFRGKYNSP*VHLAVDMMSRALHDMNMKLNKGS
KGNSEVDSYKDQLHHYLKKVQYRSKHGETFSFDENGEFVTNYLIYSNFMPNSLTVNKSFL
GEFSPWAPLDQQLNIPSALIQWKTKDNKVTPAGNTLSQFNPGKILSPVVVHGTYLIEIGYM
FFVSISTDSVSCTKCPDMEWPNEMNQCIGRKEEFLSYTKDLISICISMISVLLFLMTQLILGV
FIKYRDSPIVRANNRSLSFLLLVSIKLSFLSVFLFLGRPVDITCMLRIITFGITFSIAVSSLLAKT
IMVCVAFKATKPGSSWRKWLGVKLSNSVVLFCSSIQIIICMTWLAISPPFQELDIHTSPGTIII
QCNEGSAIGFYSVIGYMGLLAAVSFVLAFLARSLPDSFNEAKYITFSMLLFCSVWITMIPAY
LSTKGKNTVCVEIFAILTSSAGLLACIFLPKCYIILLKPEMNTKSNLLYKV*
>jgi|Xentr4|410508|e_gw1.918.47.1
MLGITAPSSPLKVLIYLIILCVGPCSSTDQPPNPACHLRIIQSPMEYEYIQTGDIIIGGVMAAH
LSTQDIIYPNDRMMRLVCTCFSREHSKYFLDFRYAIEQTNKDPTRLPNLTLGYHIYDSCGDP
RKAVRSVLQILSGTREPVPNYSCVGKRNIAGFIGDLTSETTVPIAQILTLYGYSQISYGATDP
SLCDRRTFPYFFRTVQSDETNYLALGKLLKYFGWTWVGFITSDDISGEREDETLRKYLSKE
TICVEFTVKINRDLSDQVHLSRDSKIIQNSTTGVVIIGGTVSLIFVRKLIELTVALTKKTFILSS
VWGANHYVLIFTKQTFNGSLSIQPLYPYYLTAPEDFRYLVNLHPFKYPNDTMLEDIWLMC
FGCLAQTQAKNDYYKQVIVMSNCTGAERYTNIGYFQYFTFSPRVHLAVGMMSLAIDWME
VSVSEKLPKNQRLGYSYRNQLHHYLKDIIYYTRDGKATAFDEYGDYVTNYGIYNNIITSAL
VLRYNFIGKFTPWGPPEQLMNINPDRIIWKTNRTKIPRSQCSDNCLPGFRKAAKTGTKACC
YDCVPCSDGEISHGTDSEICIRCPDMEWPNEQKEQCIPKIEEFLSYTNDVISVVLSSISVLFF
LITLLVLGLFISYWDTPIVRANNRSLSFLLLVSIKLSFLSVFLFLGRPVDITCMLRIITFGITFSI
AVSSLLAKTIMVCVAFKATKPGSSWRKWLGVKLSNSVILFCSSIQIIICMTWLAISPPFQELD
IHTSPGTIIIQCNEGSAIGFYSVIGYMGLLAAVSFVLACLARSLPDSFNEAKYITFSMLLFCS
VWITMIPAYLSTKGKNTVCVEIFAILTSSAGLLACIFLPKCYVIFLLPERNTKSHMLGNKRQ
SFAHSSKFRRKKTLPTQHLKN*
>jgi|Xentr4|410931|e_gw1.925.31.1
MSNNSSCQSLSPTVYLSGYSIILTLGLFLNITALWFFIVRLPSLRSPTKVYMKNLAFADFLLV
CMLPLMIYQHAAPLGTDFENIRTLCIVAGTFLLLNMYGSIFLLACISLDRCLALCFPLRSRSF
RRFAPWFCAGVWMLNLGACATLFFRHTSNSTDSTNECFSSHPPVVTMRAPTIASLLIGFLV
PLGIMVVSSCSLLRAVSKSQSAQDGTVNRRKLIHMLSANLAIFLFCFLPYHAVLLSYQIWE
RNCILQEAYKIALLMACSNTVLDPIAYYFATETMQKKIVEEKIRLAGGSGGETEKNSNVNL
LARHKGTQA*
>jgi|Xentr4|410973|e_gw1.926.12.1
MVSESKDNISGFIIQGFSDTPELHISLFVLFLGIYLTILLGNLIIFLVISCNPHLHTPMYIFLLNL
SLIDISSTSNILPNLLHILLTQQNNISFLGCMTQMYVFASLACSEYFLLTAMAYDRYVAICDP
LHYIARMSRKHCAGLITAAFTVGFVDPVGHFFLISKLSYCASRLIKHFFCDVTPLLKLSCSS
TFSMELLITLKGHC*L*MPSSLL*PHTYLSSLLS*KYNPQRGDKKPFLPVLLTWPV**PFMG
QFFACT*NPAQVIPCKETSISHCCTLPWALC*ILLFTH*KIENFNLP*IK*GK*FN*FLINIWLIA
V

>jgi|Xentr4|410987|e_gw1.926.89.1
MAPGSQENVSGFIIQGFSDTPELQISLFVLFLGIYLIILLGNLIIFLVISCNPHLHTPMYIFLLN
LSLIDISFPSNVLPNLLHILLTQQNNISFLGCMTQMYVFVSLASSEYFLLTAMAYDRYVAICD
PLHYIARMSRKHCAGLITAAFTVGFVDTVSMVVLIPKLSYCASRLINHFFCDITPLLKLSCS
STFSVVLFMYIEGTLLSFNAFLFTLISYIFIISAILKIQSSEGRQKAFSTCASHLACVITLYGTA
FCLYMRPTSSYSLKRDKYFSLLYIVLGPVLNPLIYTLKNREFQSSSKKMRQRCLAFSF*
>jgi|Xentr4|411029|e_gw1.926.95.1
MEIKENVSGFIIQGFSDTPELQISLFVLFLGIYLIILLGNLIIFLVISCNPHLHTPMYIFLLNLSLI
DISFPSTVLPNLLHILLTQQNNISFLGCMTQMFVFVAFVSNEYFLLTAMAYDRYVAICDPLH
YIARMSRKHCAGLITAAFTVGFGEAVSFVVLIPKLSYCASHLINHFFCDVTPLLKLSCSSTF
SVELLIYIEGTLLGFNSFLLTLTSYIFIISAILKIQSSEGRQKAFSTCASHLACVITLYVTAFCLY
MRPTTSYSIKRDKYFSLLYIALGPVLNPLIYTLKNREFQSSFNKVRQRNRALLDINT*
>jgi|Xentr4|411030|e_gw1.926.50.1
MASENVSGFIIQGFSDTPELHISLFVLFLGIYLIILLGNLIIFLVISCNPHLHTPMYIFLLNLSLI
DISSTSTVLPNLLHILLLTAMAYDRYVAICDPLHYIARMSRKHCAGLITAAFTGGFAEPVGLI
VLIPKLSYCASHLINHFFCDVTPLLKLSCSSTFSVELFIYTEGSFLSFSSFLLTLTSYIFIISAILK
IQSSEGRQKAFSTCASHLACVITLYGTVICLYMRPTTSYSLERDKYFSLLYIALGPVLNPLIY
TLKNREFQSSFIKVRQRLSFLI*
>jgi|Xentr4|411042|e_gw1.926.38.1
MASESKDNVSGFIIQGFSDTPELQISLFVLFLGIYLIILLGNLIIFLVISCNPHLHTPMYIFLLN
LSLIDISFSSNVFPNLLHILLTQQNNISFLGCMTQMFVFVALAVSEYFLLTAMAYDRYVAICD
PLHYIARMSRKHCAGLITAAFTGGFGGTVGLIVLIPKLSYCASHLINHFFCDVTPLLKLSCS
STFSAELVIYVEAILLSFNAFLLTLISYIFIISAILKIQSSEGRQKAFSTCASHLTSVLTLYVTAF
CLYVRPTTSFSLERDKYFSLLYIALGPVLNPLIYTLKNREFQSSFNKVRHRG*
>jgi|Xentr4|411043|e_gw1.926.90.1
MASENVSGFIIQGFSDTPELQISLFVLFLGIYLIILLGNLIIFLVISCNPHLHTPMYIFLQNLSLI
DIFSTLDIFPKMLHILLTQQNNISFLGCMTQMYVFLFFAASEYFLLTAMAYDRYVAICDPLH
YIARMSRKHCAGLITAAFTVGFVDPLAIVVLLSKLSYCASHLIDHFFCDISPLLQLSCSSTFS
VELLIYIEGILLPFNSFLLTLTSYIFIISAILKIQSSEGRQKAFSTCASHLACVITLYGTLICLYM
RPTTNYSPKRDKYFSLLYIVLGPVLNPLIYTLKNKKIQSSFIKVRQGLFNFR*
>jgi|Xentr4|411050|e_gw1.926.13.1
MKTTSETKENISGFIIQGFSDTPELQISLFALILGIYLIILLGNLIIFLVISCNPHLHTPMYIFLLN
LSLIDISFSSNILPNLLHILLTQQNNISFLGCMAQMYVFVSLACNEYFLLTAMAYDRYVAICD
PLHYIARMSRKHCAGLITAAFTVGFLDPVGHVVLISKLSYCASHLINHFFCDITPLLTLSCSS
TFSVELLNYIEGPLLTFNSFLLTLTSYIFIISAILKIQSSEGRQKAFSTCASHLACVITLYGTVIC
LYMRPTKNYSLQRDKYFSLLYIALGPVLNPLIYTLKNKEFQSSFNKMWQMYFIYFR*
>jgi|Xentr4|411052|e_gw1.926.86.1
MVLENVSGFIIQGFSDTPELQASLFVLILGIYLIILLGNLIIFLVISCNPHLHTPMYIFLQNLSLI
DISFTSTVLPNLLHILLTQQNNISFLGCMTQTYVYVALAASEYLLLTAMAYDRYVAICDPLH
YIARMSRKHCAGLITAAFTVGFGESISLIVLISKLSYCASHLINHFFCDVPPLLKLSCSSTFSV
ELSIYIEAILLTFNAFLFTLASYIFIISAILKIQTSEGRQKAFSTCASHLICVITLYGTIFCLYMRP
TTSYSIKRDKYFSLLYIALGPVLNPLIYTLKNREFQSSFKKVRQRCFELLYFR*
>jgi|Xentr4|411053|e_gw1.926.41.1
MASESRGNFSEFIIQGFSEIPEHQTSLFVLFLGIYLIILLGNLIIFLVISCNPHLHTPMYIFLQNL

SLIDISFSSNILPNLLHILLTQQNNISFLGCMTQMYLFASLASSEYLLLAAMAYDRYVAICDP
LHYIARMSRKHCAGLITSSYTGAFVDSVGHFVLISKLSYCASHHINHIFCDVAPLLQLSCSS
TFSVELFIYIVGTFLVFNSFLLTLISYIFIISAILKIQSSEGRQKAFSTCASHLACVITLYGTVICL
YMRPTTSYSLERDKYFSLLYIVLVPVLNPLIYTLKNKEFQYSFSRIKQRMLF*
>jgi|Xentr4|411402|e_gw1.936.19.1
MSAAIEGKKPITSLWFSTVFSIIGLCSNLFAFCVLVNASRKNQSRSRSSFLILLCGLVVTDFM
GLLVTGIIVVTYHVTQFDWQVFDQNCYLCNFFGLSMVFYGQCPLILGAAMALERFFGINK
PFSRSATVSKKRACGIVLMVWCFAFFLGLLPIMGVGKYSLQYPKSWCFLTLLQDSKNKSF
GLIFSFIGIFSVSLSFILNTISVVTLCRVYHDRECVQRRRDSEVEMMVQLVGIMIIASLCWLP
LLIFIAQAVLQHPNGELLEDEIPRNTEKLLLIYLRIATWNQILDPWVYILFRRAVLKRVYPSL
KSRPSIVSLYPTLTASLRRKYTQESVIH*
>jgi|Xentr4|411627|e_gw1.942.79.1
MYPNNWTSVNEIVLLGFQNLHNFKVPLFSLFLLIYILTVWENVLIIVLVAFSRNLHSPMYFF
LQQLSLTDLLGSTNIVPTLLQTVINEGKTIPLVNCITQLYIWGVSEAFECLLLAVMAYDRYV
AICIPLRYTSIVSHRLCVTFILVSWAVGFGIVLIIVHMAATLTFCKRNTINHFFCDLIPLVELSC
SDTVFVQIDLILNTVPLVFFPFILISVSYMCIAHAILKIVSHTGRQKAFSTCSSHLAVVSMFFG
VTVAIYLVPPRKQSLAISKVLSLLYTVVIPMINPLIYSLRSKEIKHSLRKCF*
>jgi|Xentr4|411632|e_gw1.942.70.1
MNEKNQTWVSEIVLLGFQNLHNFKVPLFSLFLLIYILTVWENVLIIVLVAFSRNLHSPMYFF
LQQLALTDLLEASNIVPTLLQTVIHDGATVSLVGCLTQMYFLGVLEVFECIFLAVMAYDRY
VAICIPLRYTSIMSHRVCVYLTVLSWLLSLSSEIIFIHMIGSLQLCHQNTINHFFCDFFPFLELS
CSDTFFIKMNAVLLCVFVILLPLILISVSYMCIAHAILKIVSHTGRQKAFSTCSSHLAVVSMF
YGTLCSVYVVPPGNQSQTISKVLSLLYTVVTPLINPLIYCWRSTDMTESLKKHKDRITDYK
NMGMKPFLIYLLHIRS*
>jgi|Xentr4|411637|e_gw1.942.90.1
MNEKNQTWVSEIVLLGFQNLHNFKVPLFSLFLLIYILTVWENVLIIVLVAFSRNLHSPMYFF
LQQLSLTDLLGSSDIGPTLLQTVINNGATMSFYGCLTQLYFWGCSGIFECLLLAVMAYDRY
VAICIPLRYTSIMTHRVCTKLVVIPWALGFGISVITVKLTGTLEVCDQNTINHFFCDVFPLVE
LSCSDTFSLQIEAFFLFVPIIVFPVVVIIVSYMCIAHAILKIVSHTGRQKAFSTCSSHLAVVSM
FYGTIIAIYVVPPRKDLQTISKVLSLLYTVGTPLVNPLIYSLRNKDLNIALKNLKVN*
>jgi|Xentr4|411640|e_gw1.942.82.1
MNEKNQTLVSEIVLLGFQNLHNFKVPLFSLFLLIYILTVWENILIIVLVAFSRNLHSPMYLLI
QQLSVSDLMQSTNIVPTLLLTIMNEGAAVQLVGCITQLCCFGGLETLQCLLLAVMAYDRYV
AICIPLRYTSIMSHKVCVTLILISWLLGLIITFNTGNAVHSLQFGNKNSIDHFFCDFFPFVELS
CSNTFFFHIEVILLSVPAIFFPFILISVSYTCIAHAILKIVSHTGRQKAFSTCSSHLAVVSMFYG
TLIAIYLAPSKTRSQTISKVLSLLYTVVIPLVNPVIYSFRSKDIKDALKKLLEGYCSQLDTYG
VVKQI*
>jgi|Xentr4|411652|e_gw1.942.41.1
MNEKNQTWVSEIVLLGFQNLHNFKVPLFSLFLLIYILTVWENVLIIVLVAFSRNLHSPMYFF
LQQLALCDLLNSTVIVPTLLQTVINGGTTVSFIGCITQFSFFSVPEAFECLLLAVMAYDRYV
AICIPLRYTSMMAKRTCVEFIVMAWTSGITCALLLSSIISTLQFCNRNTINHFFCDFFPVVEL
SCSSTFLAQTGAALLSVPMIIFPFILIIVSYMCIAHAILKIVSHTGRQKAFSTCSSHLAVVSIFY
GTIIALYMVPPRKESQNICKALSLLYTVVIPSVNPVIYSLRSTDIKEALIRRPTIRLLASARPRI
IHKTV*

>jgi|Xentr4|411666|e_gw1.942.101.1
MNEKNQTWVSEIVLLGFQNLLNFKVPLFSLFLLIYILTVWENILIIVLVAFSRNLHSPMYFFL
QQLALSDLQGSTIIVPTLLQTVMKGGTRVSLVGCISQWYFFSSSDAFQCLLLAVMAYDRYV
AICIPLRYTSIMSHRVCVTFILMSWAVGFGLAVITVNLIGILEFCDQNIIDHFFCDFFPLIELSC
SDTFLLQLEAFIVSVPVIFIPFLVISVSYMCIAHAILKIVSHTGRQKAFSTCSSHLAVVSMFYG
TIISIYVVPPRKESQTISKVLSLLYTIVIPLSNPLIYSLRNKDIHAALILIKPINVNK*
>jgi|Xentr4|411678|e_gw1.942.40.1
MNEKNQTWVTEIVLLGFQNLHNFKVPLFSLFLLIYILTVWENVLIIVLVAFSRNLHSPMYFF
LQQLSLTDLLESSNIVPILLQTIIDDGTTLSFVGCITQMYFLGSFEVFECLLLAVMAYDRYVA
ICIPLRYTSIMSHRVCAQVISISWLVTLTSEAIFANMIGSLQLCNRNTVDHFFCDLFPFLELSC
SDTFSVQMDTVLLCVFVVLSPFILISVSYMCIAHAILKIVSHTGRQKAFSTCSSHLAVVSMF
YGTLISVYVVPPRKQSHTISKVLSLLYTVGTPLINPLIYCWRSTDLKEMLKKIKTNKLCNID
*
>jgi|Xentr4|411689|e_gw1.942.109.1
MSMKNQTWVSEIVLLGFQNLHNFKVPLFSLFLLIYILTVWENILIIVLVAFSRNLHSPMYFF
LQQLSLSDLLESSNTAPTLLLTILSDGTTMPLVGCITQLYFVAYLVTFQSLLLAVMSYDRYV
AICIPLRYTSIMSHKFCIKMVLVSWIITICSTLILVNLIGTLKFCEQKNINHFFCDLDPLLQISC
SDTFFVKIEATLMAIPVVVCPFILICGSYMRIAHAILKIVSHTGRQKAFSTCSSHLAVVSMY
YGALIAIYLAPSKKETETISKVLSLFYTVVIPLVNPVIYSLRSKDIKEAFKRFKMF*
>jgi|Xentr4|411690|e_gw1.942.36.1
MNEKNQTWVSEIVLLGFQNLHNFKVPLFSLFLLIYILTVWENVLIIVLVAFSRNLHSPMYFF
LQQLALSDLLEASVTAPTLLQTVINNGATVSLVVCRIQFYFFVCTEAFECLLLAVMAYDRY
VAICIPLRYTSIMNQKVYVRCILVPWLLGLTATINTANMIDRLQFCDQNTINHFFCDFFPLLE
LSCSDTSVVQIDIILQSVLVIICPFILISVSYMCIAHAILKIVSHTGRQKAFSTCSSHLAVVSMF
YGALAIYVFPPRNQSLNMNKILSLLYTVVTPLVNPLIYSWRSRDIKKAVEGALRADVMVG
RAVRG*
>jgi|Xentr4|411691|e_gw1.942.100.1
MNEKNQTWVSEIVLLGFQNLHNFKVPLFSLFLLIYIVTVWENVLIIVLVAFSRNLHSPMYFF
LQQLALSDLLQITNIVPILLQTIIYGGTTLSLPGCITQLYFFGGSEGCEFLLLSVMAYDRYVAI
CIPLRYTSILNPRVCFILILMSWLLGFGIAVITANLMGALEFCDQNTIDHFFCDFYPLLELSCS
DKFPLQIEAFFLFVPMVFMPFILLIVSYMCIAHAILKIVSHTGRQKAFSTCSSHLAVVSMFY
GALIAIYLFPSKKQSSAVSKVLSLLYTVVIPMVNPLIYTLRNKEIQGALKIKLN*
>jgi|Xentr4|411701|e_gw1.942.112.1
XTWVSEIVLLGFQNLHNFKVPLFSLFLLIYILTVWENVLIIVLVAFSRNLHSPMYFFLQQLS
VCDFMESTNIVPTLLQTIIYDKIMLPFVSCMIQFAFFSATEAFECLLLAVMAYDRYVAICIPL
RYTSIMSHVVCIKIILMSWIFSLCSTLATANLIGTLKFCEQKKINHFFCDLDPLLKISCSDTFF
VKIEATLMSIPVVVCPFILISVSYMCIAHAILKIVSHTGRQKAFSTCSSHLAVVSMFYGTIIAI
YIVPPRKESQTISKVLSLFYTVVIPLVNPVIYSLRSKDIKEAFTKILIFN*
>jgi|Xentr4|411713|e_gw1.942.108.1
MDEQNQTWVSEIVLLGFQNLHNFKVPLFSLFLLIYILTVWENVLIIVLVAFSRNLHSPMYFF
LQQLALTDLLGSTNIVPTLLLTVIHDGTTLSLVGCFVEFYFFCAPEAFECLLLAVMAYDRYV
AICIPLRYTSIMSHRACVTFILMFWALGFSNAMITVNIIGSLQFCYQNTINNFFCDFLPLLEL
SCSDLSFLEIDVIIQSLPLIICPFVLIIVSYMCIAHAILKIVSHTGRQKAFSTCSSHLAVVSMFY
GTLIAIYMVPPSNQSQTVSKVLSLLYTVVIPLVNPVIYSLRNKDIKEAFKMILK*

>jgi|Xentr4|411723|e_gw1.942.47.1
MFPKNQTWVSEIVLLGFQNLHNFKVPLFSLFLLIYILTVWENVLIIVLVAFSRNLHSPMYFF
LQQLSLTDLLESSNIVPTLLLTVIHNGATVSLVGCFMEFYLFCVLEAFECLLLAVMAYDRYV
AICIPLRYTSIMSHRVCVTFILISWTVSLSSALVTVNLLRTLQFCDNKNINHFFCDLDPVLQL
SCSDTFLVQIEVISLSIPVIVFPVVLITVSYMCIAHAILKIVSHTGRQKAFSTCSSHLAVVSMF
YGTLIAIYLAPSRRETKTTSKVLSLFYTVVIPMGNPLIYSLRNKDIKEAFKKLETLKHQRRY
L*
>jgi|Xentr4|411724|e_gw1.942.39.1
MNEKNQTWVSEIVLLGFQNLHNFKVPLFSLFLLIYILTVWENVLIIVLVAFSRNLHSPMYFF
LQQLALSDLLTSSNIAPTLLETILNEGATVSLVGCITQFNIFGGLETFVGFLLAVMAYDRYVA
ICIPLRYTSIMSHRVCVTLMFVSWLIGLGAILITANLIATLHFCDRNTIHHFFCDFFPLLELSC
SDTFIVQLEVIILSVPVIICPFILISVSYMCIAHAILKIVSHTGRQKAFSTCSSHLAVVSMFYGT
IIALYIVPPRKESQTISKVLSLLYTILIPLFNPIIYSLRNKDAQEALKYIIKYHTVCHHIIIPCCY
SNI*
>jgi|Xentr4|411754|e_gw1.944.65.1
MALNILFAVLLYVISVKCVAYCHSVVEPNHSACKLEVVKMSGEYEYIKEGDIMIGGVVTV
NAFIAADKDEKSYRRTIWCANPTPQNYKYLLDFFHLINEFNNNPLKPQNLTLGYHISDSCG
DVYKAERSVLQILSGTREPVPNYSCAGKRNIVGFIGDLTSETTIPIAHILSVLGYSQVSYGAT
DPSLSDRDTFPYFFRTVQRMDGQYFAITQLLKHLGWTWVGIITTNDIKGDSDYGLLTNYLS
SEGICIDFTIKIQNQISYDHKMLYRNIILKSSTSVVIFCGTVTWRIAIKLGPVLDIFSKKTFIFT
SDWLCYSNIINYARGLFNGSLVFMANIIDYKNDDHFSVESIHPSNYPEDELLGTIWIRYFSC
KPENMSMGFSALLKLLPNCTGKERLTDIKDFTKTLHTRNMLLAVEAFSVASCKLHFIHNSC
NNQSSEKQRKVMNFKYQLHRLLKEAQFPSQGQLLKYFNENGEFVSAYEISNFYGTSKESI
AETRVGQYVPWAPSDQKLNITLDAIKWKTANNMIPRAQCSDNCPPGYRKAPKPGAQSCC
YVCVPCSEGEISNITDSENCIRCPDMEWPNDKRTKCIARTEVFLSFTNDVISLFFSSVSLLFF
LLTLLILGVFIIYRDSPIVRANNRSLSFLLLVSIKLSFLSVFLFLGRPVDITCMLRIITFGITFSIA
VSSLLAKTIMVCVAFKATKPGSSWRKWLGVKLSNSVVLFCSSIQIIICMTWLAISPPFQELD
IHTSPGTIIIQCNEGSAIGFYSVIGYMGLLAAVSFVLAFLARSLPDSFNEAKYITFSMLLFCSV
WITMIPAYLSTKGKNTVCVEIFAILTSSAGLLACIFLPKCYIILFRSEINTKSHLLGNKY*
>jgi|Xentr4|411774|e_gw1.944.64.1
MYILLSQGAVLGDPVVPNLITALIYMIVLCVGPCRSVFQPLNPACHLEIIKAVKEHEYIQEG
DIMIGGVMTAHLNMINLSFPLYNFTKFLCTEPSQQDFRRLIDFRFAVEQTNKDPARLPNLTL
GYHIYDSCGDPRKAVRSVLQILSGTREPVPNYSCVGKRNIAGFIGDLISETTVPIAQILTLYG
YSQISYGATDSFLRDRVTFPYIFRTVQSEESSYFALGKLLSHFGWTWVGIITSDDISGDKEH
QILAKYLFTEGVCIEFTIRIKMNALSNIHSDVSIIKYSSTRIILLCGTVSLYFLTILLFFMKSVL
LEKTLILSTNWGTNDMSVRYAHDIFNCSLSFVPGFHYHLDTPEMRSFLETLHPSKYPEDKL
LENIWMIFHFCLSEEPIKNHLYEFIYINTLHNCTGQERITDLPYFTADYGSPQVHLAVDMMS
QALHEMSIQLREKAVKTREMYHYVHQLHHHLETLKYNSPNGPIFCFDENREYITRYWIYN
YITFACNNITRNLIGDFSPWAPTDQLLRITSLTWKRNTVRILYFIHTQCATFCRPHFFCAHFA
MTTAILTRSRLYSIRCSKCPDMEWPNKRKNQCIPRREDFLSYASDKISVCLSVIAILFFLITL
WISAVFVTHRETVIVRANNRSLSFLLLVSIKLSFLSVFLFLGRPVDITCMLRIITFGITFSIAVS
SLLAKTIMVCVAFKATKPGSSWRKWLGVKLSNSVVLFCSSIQIIICMTWLAISPPFQELDIH
TSPGTIIIQCNEGSAIGFYSVIGYMGLLAAVSFVLAFLARSLPDSFNEAKYITFSMLLFCSVW
ITMIPAYLSTKGKNTVCVEIFAILTSSAGLLASIFLPKCYIILFKPEINVKSHLFGNKSR*

>jgi|Xentr4|411776|e_gw1.944.56.1
MVVNILSRLLIYIISVKCVGYYDSDVEPINSACKLEIVKVAEDYEYIKKGDIMIGGVITVNA
LVSFFGLKGRYDKNIVCINPTPQNYKYLLDIMYMINKFNKNPLMSQNLTLGYHIFDSCGYR
QKAVRSVLQILSGTREPVPNYSCARKRNIIGFVGDLTSDTTIPIAHILNVLGYSQISYGATDP
SLSDRDTFPFFFRTVQSKELQYFAIAQFLKYFGWTWVGILTTDDINGDRAHQLLANYLSSE
GICIDFTIKIRRDKSAKDKFLFNKIIQQSSTSVVIFCGTVNWGNAVHLGSTLDIFNEKTLIFTS
DWLDYSDIINEARGLFNGSLVFTQNMVDYTMYDDRFSHFLETIHPSNHPEDKLLEVIWLR
HLSCKTENMTLFYLHVLYDLHPCTGEERLTDIKAFMETFHTRNMLLAVEALSVASSRLHFI
HNSLNKPSSEIKTEVTHYKYQLHRLLKEAQFSSQGQLMKFFNENGEFVSPYQISNFYGTSN
EPVEETRVGQYIPWAPSDQKMNITLDAIKWKTKNKMVPRAQCSDNCLPGFRKALKPGAQ
SCCYDCVPCSEGEISNITDSETCIRCSDMEWPNEKKNWCIERMKDFLSYTNDVISVVFVSI
CLLLFVLTLLILIVFVIYRDTPIVRANNRSLSFLLLVSIKLSFLSVFLFLGRPVDITCMLRIITF
GITFSIAVSSLLAKTIMVCVAFKATKPGSSWRKWLGVKLSNSVVLFCSSIQIIICMTWLAISP
PFQELDIHTSPGTIIIQCNEGSAIGFYSVIGYIGLLAAVSFVLAFLARSLPDSFNEAKYITFSM
LLFCSVWITMIPAYLSTKGKNTVCVEIFAILTSSAGLLACIFLPKCYII
>jgi|Xentr4|411794|e_gw1.944.55.1
MALHILFRVLIYVMSINCVGYCYSDVHPINSACKLEIFKVLEDFAYVKHGDIMIGGVVTVN
TLANHFETEYGNFLMCYKPNPKYYKYLLDFLYYITAFSENLVLKQNLTLGFHIYDSCGDV
YKAERSVLQTLSGLREPVPNYSCVGKRNIVGFIGDFTSETTIPIAHILSVLGYTQISYGASDP
SLSDRATFPFFFRTVQSDVRRYFIITQLLKYFGWTWVGIITTNDINGERDYQLLTKYLSSEGI
CIDFIIKMNYKKENTLYEESLRKSSTSVVIFCGKINMKTVDQFIGLSQIFREKTFVFASEMFN
YMHLVNYALGLFNGSLVLMQNGASYSKNNQHFREFLENVHPLNYPQDKLLEDIWMLYHS
CITEYMTRVFIHKRLYSNPLHNCTGEELLTDIKEFQEELHTRNMFIAAHALSFASSMLHFKQ
RSLNELSTDTDNKEIHYKYQLHHFVRKWRFQEEFLGTFDENGEYVSPYRIINFYGSSYQSV
AQTTVGLYVPWASLDQKLNITLGLIKWKTTNNTIPRAQCSDNCLPGFRKAPKPGAQSCCY
DCVPCSEGEISNTTDSENCIRCPDMEWPNEKRNRCIAKMENFLSYTNYGISVCFSSISVLLF
VITLLILAVFISYRDTPIVRANNRSLSFLLLVSIKLSFLSVFLFLGRPVDITCMLRIITFGITFSIA
VSSLLAKTIMVCVAFKATKPGSSWRKWLGVKLSNSVVLFCSSIQIIICMTWLAISPPFQELD
IHTSPGTIIIQCNEGSAIGFYSVIGYMGLLAAVTIGFYSVIGYMGLLAAVSFVLAFLARSLPD
SFNEAKYITFSMLLFCSVWITMIPAYLSTKGKNTVCVEIFAILTSSAGLLACIFLPKCYIILYR
SDMNTKSHLLGNK*
>jgi|Xentr4|411804|e_gw1.944.59.1
MKVLIYIIMMCEGPLRSGNATINPACRLEMIKALEEYEYIQEGDIMIGGVMSLSLYGYRQW
DTNLLACYLTDLRNYRSFLDFLFVIEEINNNPALLSNLTLGYHIYDSCGNEQKAVRSVLQIL
SGTREPVPNYSCVGKRNIAGFIGDLKSRTTIPVAQILNLYGYSQISYGATDPSLSDRVAFPYF
FRTVQSDHHHYFALSKLLRYFGWNWVGIITSDDDSGEKERELLTRYFSAHGICVEFTIKIM
DMSEYYVNRTELYEQIIDESSANIIILCGTANIAFSINLQSLTDRLSQKTLILTSKWESTSHVL
EYYYKIFHGSLISSQHFIYPVSMYRFWQFAASRHPSKYPEDTLLKDIFMFYLCMPSNYKLH
SFHSNLYQDQLHNCSGEERLTDIEDFNTTYHSPSVYLAVFTMFRGLQSLLSKQTAERKGIS
YRHQLHRYLKNVTFTDTENKTLYFDKNGEFVTQYEIQNVFLDDNKPLVSKPVGMYTPWA
QPDQKIHITSELIRWKTLDNKIPRSQCSENCSPGYRKAPAPSVHSCCYSCVQCSEGEISNVT
GKDATHFLMY*GNCGCGIAGIVGRDGAYSSSGGIIASGKGLGLWDSRYSRERCFLSVFLFL
GRPVDITCMLRIITFGITFSIAVSSLLAKTIMVCVAFKATKPGSSWRKWLGVKLSNSVVLFC
SSIQIIICMTWLAISPPFQELDIHTSPGTIIIQCNEGSAIGFYSVIGYMGLLAAVSFVLAFLARS

LPDSFNEAKYITFSMLLFCSVWITMIPAYLSTKGKNTVCVEIFAILTSSAGLLACIFLPKCYVI
LFRPEMNTKSNLLYNV*
>jgi|Xentr4|411823|e_gw1.944.58.1
MPGGTATYNPIKVLIYIIMTCEGPLRSGNATTNPACHLEIIKALEEYEYIQEGDIMIGGVMSL
SLYGYREQKTNLLVCFLTDMRNYRYFLDFLFVIEEINNNPPLLSNLTLGYHIYDSCGFEQKA
VRSVLQILSGTREPVPNYSCVGKRNIAGFIGDLKSRTTIPVAQILNLYGYLQISYGATDPSLS
DRVAFPYFFRTVQSDHHHFFALSKLLRNFGWNWVGIITSDDDSGEKEHELLTRYFSTHGIC
VEFTIKIIEINEHYVNRTELYEQIIDGSSANIIILCGTANVAVSEYLLSLTDRLSQKTLILTSKW
ESFTHVLEYCHKTFNGSLIFSQHFIYPVSMYRFWQFAASRHPSKYPEDILLKDIFMFFFCMP
SNYTLHSFHFNIYPVQFHNCSGKERLTDIKGFNTTYHSPSVYLAVFTMFRGLQSLLSKQTA
ERTGISYRHQLHRYLKNISFTDTENKTSYFDKNGELVTQYEVQNVFLNDNKPLATKPVGM
YTPWAQPDQNLHITSELIRWKTSDNKIPRSQCSENCSPGYRKAPAPSIHSCCYSCVQCSEGE
FSNVTDSEKCLRCPAIEWTNEWRNQCIAKTEDFLLYNTDAIAVVLSSLAVSLFLLTLFILGV
FITYQDTPIVRANNRSLSFLLLVSIKLSFLSVFLFLGRPVDITCMLRIITFGITFSIAVSSLLAKT
IMVCVAFKATKPGSSWRKWLGVKLSNSVVLFCSSIQIIICMTWLAISPPFQELDILTSPGTIII
QCNEGSAIGFYSVIGYMGLLAAVSFVLAFLARSLPDSFNEAKYITFSMLLFCSVWITMIPAY
LSTKGKNTVCVEIFAILTSSAGLLACIFLPKCYIILFRPEINVKSHLFGKNM*
>jgi|Xentr4|411830|e_gw1.944.61.1
MKVLIYIIMMCEGPLRSGNATVNPACRLEIIKALEEYEYIQEGDIMIGGVMSLSLYGIRQLE
TNLLVCYLTDLRHYRYFLDFLFVIEEINNNPALLSNLTLGYHIYDSCGIEQKAVRSVLQILSG
TREPVPNYSCVGKRHIAGFIGDLKSRTTIPVAQILNLYGYSQISYGATDPSLSDRVAFPYFFR
TVQSDHHHYFALSKLLKYFGWNWVGIITSDDDSGEKERELLTRYFSAHGICVEFTIKIMD
MNEKFVNRTELYEQIIDESSANIIILCGTANIAFSINLLSLTDRLSQKTLILTSKWESFTHVLE
YCYKIFHGSLISSQHFIYPVSMYRFWQFAASRHPSNYPEDTLLKDIFMLYFCMPSNHKLHS
FHSHFYPVQLHNCSGEERLTDIEGFNTTYHSPSVYLAVFTMFRGLQSLLSKQTAERKGISY
RHQLHRYLKNISITDTENKTSYFDKNGEFVTQYEIQNVFLNDNKPLVLNPVGMYTPWAQP
DQKIHITSELIRWKTLENKIPRSQCSENCSPGYRKAPAPSVHSCCYSCVQCSEGEISNVTGK
DATHFLMSPFYTKVPTISCIQRQITQNMNARGLLNSLMPNMHRFTIVCGIQGTQIKIDTPIV
RANNRSLSFLLLVSIKLSFLSVFLFLGRPVDITCMLRIITFGITFSIAVSSLLAKTIMVCVAFKA
TKPGSSWRKWLGVKLSNSVVLFCSSIQIIICMTWLAISPPFQELDIHTSPGTIIIQCNEGSAIG
FYSVIGYMGLLAAVSFVLAFLARSLPDSFNEAKYITFSMLLFCSVWITMIPAYLSTKGKNT
VCVEIFAILASSAGLLACIFLPKCYIILFRPEINVKSHVFGKNM*
>jgi|Xentr4|412224|e_gw1.955.1.1
RHYVAFIYAVEEINKNSQILPNITLGYQIVDSCGCDFKAISGVLDTMSGAEQTIVNYDCRTN
SKLVGYLGDFSSGTTYAMAQFLAVFRYPQACIHLSISYGAMDPVFNDRTQFPSFYRTIPNEE
AEMDGIVQILKHFGWKWVGLIVSDDDTGYRARERISTELARMGGCLAFSLVIEYGAAIPY
LKRKIMIETIEKTAVNVIIMFVTAKYISASLVILRYCTIPQRICITSSLFTDFITSPYNVKLTVFF
NAALSLLIREGEIPDFHYFLNCFSLSNYPQSEFMEYIWQSVFHCTFPYLPWSVKPTYYRICT
GNESMGGFSQTGDYRVTYRVYTAVYALARALHNLYSAQAPTNHWDKLEYMRGNLKPW
QINTFVRNVTFVTPSGDTIFFNDKGDPPAQFDVMTFLLLPNRTFARQKVGSFHTRASYIFTP
NILGTSQYFIHLFTLQIQMPQSLCNEPCAPGYRKAKIEGKPSCCYDCAKCADGEMSNTTD
ALDCFRCSEYEKSNKQRTGCVPKEINYLSYTDTLGASLTSIALVLFIAASVVLGIFVRYWET
PIVRANNQHLSFLLLISLMLCFLCTLLFIGRPTQICCLLRQVTFGIVFTISVSSVLAKTLTVIIA
FNATKPGSKLKKYVGTQLATILVTVCCLALRTTLCGLIQTLIGTKGNHLSNRNQRDQKLHL

MNINITRSVLFFFSVIGYMTALALFSFIAAFLAKDFPDRFNEAKNITFSMLGFCSVWGAFVP
AYLSSKGSRMVAAEIFAILSSSAGLLACIFVPKCYIIFFKPELNKRKM*
>jgi|Xentr4|412246|e_gw1.955.9.1
LFVYFKISYGAMDPIFHDRTRFPSFYRTIPNEEAEMDGIVQILKHFGWKWVGLIVSDDDTG
YRARERISTELARMGGCLAFSFEFEYQGYLSYSDEKAIAKTIENTAVQVIVLFITTKYIMAF
LNFLSYCTIPQRICITSSLYTNDITSPYNMRKTIVFNGTLSLLIQAGEIPDFHHFLNSFSLHKY
QNAFTERIYHDIFLCLYPNSSWVETTAPGYGICKGKESMSKSDFSQTGDYRVTYRVYTAVY
ALARALHNLYSAQAPTNHWDKLEYMRGNLKPWQINTFVRNVMFVSPSGDTIFFNDKGDP
PAQFDVMTFLLLPNRTFARQKIGSFHLLSDGTKLLHINSSADLWGPYYKEMPQSLCNEPCA
PGYRKAKIEGKPSCCYDCAKCADGEMSNTTDVLNCFKCSDYQRSNKQRTGCVPKEINYL
SYTDTLGASLTSIALVLFIAASVVLGIFVRYWETPIVRANNQNLSILLLISLMLCFLCTLLFIG
RPTQICCLLRQVTFGIVFTISVSSVLAKTLTVIIAFNATKPGSKLKKYVGTQLATILVTVCCL
GEMMISAVWMASNPPFLDADTLTDINTVFLMCNEGSVLFFFSVIGYMTALALFSFIAAFLA
KDFPDRFNEAKNITFSMLGFCSVWGAFVPAYLSSKGSRMVAVEIF
>jgi|Xentr4|412654|e_gw1.962.2.1
MENNSYLLDYALKLPSALNYTDPRNEDLAKAEVALLGAILVITTGSNLIVLFAIYQRRKKM
TRMHLFIVHLTFTDLAVALFQILPQMIWDITFRFIGSDILCRAVKYTQVMSMFASTYMLMM
MTIDRYIAVCHPLKTLQQPSKQAYLMIGGTWILSCILSLPQIFIFSMKEISQGAGVIDCWADF
RYPWGAKAYITWITVSIFFVPVGILLLCYSLICCEICKNLKGKMQTSGVGQRESNGQVVPS
RVSSIRTISRAKIRTVKMTFVIVLSYILCWTPFFSVQMWSVWDENSPDEDSTDFAFTITMLL
ASLSSCSNPWIYMCYNSPQLCRGTSRPHGHRHNSTGSVSSRRDTLLTQLRNRSLLRGGGS
NGTCNSIRDLYPALDDTVIESGIL*
>jgi|Xentr4|412689|e_gw1.964.35.1
MEIEFNTSKSFVLLGIEEMERFKYLYCSLFFLIYFFILLFSCTIISVVLLDESLHEPMYTLIASL
LLNGIFGSSCFFPKLITDLLLSSKEISRVGCLAQTFSVTLFAHFQISTFTIMAHDTYLAVGHPL
RYPTLMTNSVALKLILGSLIFNFLLILSSLLLSARLPLCGSRISNIFCDNMSILVLSCVDTSFN
KLYGTIIFVGYLVFMVLLIAHSYLRILLICLKISKDACKKAIHTLVTHLLNFSIFMIGVLFVFV
RYRLGATNLLLTYHIILSILGFVFPPLLTPFIYGMRTQTLKVKIIHDLQNLKQRVCTS*
>jgi|Xentr4|412742|e_gw1.964.36.1
MGIEFNISKNFVLLGIEEMERFKYLYCLLFFLIYFFILLFSCTIISVVLLDESLHGPMYTLIAIL
LLNGIFGSSSIFPKLITDLLLSSKEISHVGCVAQAYTIILFAFCEISTFTIMAHDTYIAVGHPLR
YPTLMTNSVALKLIMGSLIFNIILMLPIPILSARLPICGSRISGIFCDNPSILVLSCVDVSLNKL
YGNVTLVGYISVMALLIAHSYLRICLICLKISKDACRKAIHTLVTHLLNFSIFMIGMLFIFVR
YRLENTNLPLTYHIVLSFICFICTPLLTPLIYGIRMQTLKIRIIHHLRQRVFAR*
>jgi|Xentr4|412747|e_gw1.964.11.1
LDESLHGPMYTLIASLLLNGIFGSSCVFPKLITDLLLSSKEISRVGCFTQASTMILSGYFEIST
FTIMAHDTYLAVGNPLRYPTLMTNSVALKLILGSLIFNIILTLPAPLLSARLPICGSQISNVFC
DNASILILSCVDTSINKLYGNVSFVSYLTVMALLIAHSYLRILLICLKISKDACRKAIHTLVT
HLLNFSIFMAGGLFLFVRYRLGNTNLPLTYYIILSIMGFIFTPLFSPLIYGIRMQTLKVRVIHH
LRQRVFAR*
>jgi|Xentr4|412751|e_gw1.964.15.1
MEIEFNTSKNFVLLGIEEMERFKYLYCSLFFLLYFFILLFSCTIISVVLLDESLHEPMYTLIAS
LLLNGIFGSSCVFPKLITDLLLSSKEISHVGCLAQTVSVTLFAYSEISTFTIMAHDTYLAVGH
PLRYPTLMTNSVALKLIMGSVIFNFILMLPSLLLSARLPLCGSHISNVFCDNMSILILSCVDT

SVSKLYGTISFLGYLVFLALLISHSYLRILLICLKVSKDACRKAIHTLVTHLLNFSIFMIGVLF
VFVRYRLGATNLPLTYHIILSIPGFVFPPLLTPFIYGIRMQTLNIKIIQHVQTLKQRVCTS*
>jgi|Xentr4|413184|e_gw1.976.55.1
MEPGISNQSFIFSYTDFTLLGFPGISRWRPLLAIPFFSVCLVILSGNSLIIHLIYTEKTLHSPMY
LLISVVFAINMSVSTAILPKFLLDLLFQLNQVSLTGCLLQMFVIYFMSLCESGVLLLMSLDR
YIAICRPLRYHNIMTKRFLAWLTVIIIIRNCFLICPLVILTSMVQFCRSNIILNFTCENMALLSL
GCGDTTKPQIAGLIVRITVTVLDVGLLLLSYSTILYTAMKTTTGKSQHKALNTCGTHLLVV
AVFYLCALASSIVQRMETTISTDVKILFTALYLLIPALLNPFIYGFRMSEIRKSLLQ*
>jgi|Xentr4|413185|e_gw1.976.10.1
IVPNMLMGLAFGLDHISLGSCLFQMFFIYTSVILETTVLMIMALDRYLAICRPLRYHNIMN
NPLVGQLFLTGLVQSSLFSSSIIIVASQVQFCRSNIIWSFVCENMVLLNLGCGDISKIQVVGL
MVRVLVTAMDISFLLVSYLYIFHSTMKIARGKALHKTLHTCSTHLIVVVLNYSCGLLSSILY
RMPVSVNVQNLFSAIYYLFPATIHPIIYGYRMKESRTCLVKSWDNY*LFI*VTDALYILMFV
LKE*
>jgi|Xentr4|413195|e_gw1.976.14.1
MEPGISNQSFIFSYTDFTLLGFPGISRWRPLLAIPFFSVYLVILSGNSLLICLICIKKTLHSPMY
LLISVLFAINITSCTAILPKFLLDLLFQLNQVSLTGCLLQMFVIYFMAVCESNVMLLMSLDR
YVAICRPLRYHNIMTKNLLAWLTVTVIVRSFILVCPLVILTSMVQFCRLNIILNFTCENMALL
SLGCGDTTKPQIAGLIVRVLVTVLDVSLLMISYSTILYTAMKSATGKSQHKALNTCGTHLLV
AMVVYLCGLASSIVYRMETTISPDVKNLFSAIYLMVPATLNPFIYGLRVSEIRKSLMKYGM
KKNLFPS*
>jgi|Xentr4|413204|e_gw1.976.62.1
MEPGISNQSFIFSYTDFTLLGFPGISRWRPLLAIPFFSVYLVILSGNSLIIHLIYTEKTLHSPMY
LLISVLFAINITVSTAILPKFLLDLLFRLNQVSLTGCLLQMFSIYFMSVCESGVMLLMSLDRY
VAICRPLRYHNIMTKRFLLSLTFIVIIRSFILVCPFVLFTSMVQFCRSNIILNFTCENMALLSLG
CGDTTKPQIAGLIVRIIVTALDGSLFFLSYSTILYTAMKTVDNKSRHKALNTCGTHLLVTMQ
VYVSGLVSSLVYRMEATVSMDVKNLFSALYLIIQGALNPLIYGLRVTKIRKGLVANWRT*
>jgi|Xentr4|413213|e_gw1.976.58.1
MEPGISNQSFIFSYTDFTLLGFPGISRWRPLLAIPFFSVYLVILSGNSLLICLICIKKTLHSPMY
LLISVLFAINITSITITLPKFLLDLLFHLNQISLTGCLLQMFFIYFMMTCESAVTVLMSLDRYV
AICRPLRYHNIMTKSLLAWLIVIIIIRNCILICWVLLLISVVQFCRSNIILNFTCENMALLSLGC
GDTTKPQIAGLIVRIIVTVTDGSLLLISYSTILYTAMKTATGKSRHKALNTCGTHLLVAGVV
YVCGVPSSVVWNMETTLLADLKNLFTALYLIIPAAIDPLIYGLRVSEIRKSLVEYWREKKN
NLFSS*
>jgi|Xentr4|413228|e_gw1.976.73.1
MEPGISNQSFIFSYTDFTLLGFPGISRWRPLLAIPFFSIYLVILSGNSLLICLICIKKTLHSPMYF
LISLLFAINITSITTTLPKFLLDLLFHLNQVSLTGCLLQMFVIYFVTACESTVLLLMSLDRYIA
ICRPLRYHNIMTTSFLAWLTVIVLIRNCLLICPLIVLISMVQFCRSNVILNFACENMALLSLG
CGDTTKPQIAGLIVRIIATVLDGSLLLISYSTILYTAMKTATGKSRHKALNTCGTHLLVAMV
VYLCALASTIVQRMETSISTDIKNLFIALYLIIPALLNPFIYGFRVSEIRKSLLGYWKERKNVF
SSS*
>jgi|Xentr4|413229|e_gw1.976.65.1
MEPGISNQSFIFSYTDFTLLGFPGISRWRPLLAIPFFSVYLVILIGNSLIIHLIYNEKTLHSPMY
LLISVFFASNMGVTTAILPKFLLDLLFHLNQVSLTGCLLQMFSIYFMSLCESGVMLLMFLD

RNIAICRPLHYHNIMTKNLLVSLTLIVIARSFALVCPFVIFTSMTQFCKSNMILHFACEPLAIL
SLVCGDTTKLETVGLIIRILVPVLDGILLMISFISILYTAMKSATGKSRYKALNTCGTHFIVAIV
ASLCSLAYSSVWKMETVSVDVKHLFTALYIMLPASLNPFIYGVRVSEIRKSLVKYWGKKN
KLFSS*
>jgi|Xentr4|413233|e_gw1.976.49.1
MEPGISNQSFIFSYTDFTLLGFPGISRWRPLLAIPFFSVYLVILSGNSLIIHLIYTEKTLHSPMY
LLISVLLAINMSVTTAILPKFLLDLLFQLNQVSLTGCLLQMFVIYFMSVCESSVMVLMSLD
RYVAICRPLRYHNIMTTSFLACLTVIVITRGLVLVCPFIILASMVQFCRSNTILHFACETMAL
LSLASGDTTKLEIAGLILRILVAVLDGILLMVSYSTILYTAMKTVTGKSRHKALNTCGTHLL
VAMVVYLCALVSSSVWKKETILSIDVKHLFTALYIMIPAALNPLIYGLRISIIRKSLVKYWRK
KDNLFLS*
>jgi|Xentr4|413242|e_gw1.976.52.1
MEPGITNQSFIFSYTDFTLLGFPGISRWRPLLAIPFFTMYLVILSGNSLLICLICIKKTLHSPM
YLLISVLFAIHITSCTAILPKFLLDLLFQLNQVSLTGCLLQMFVIYFMAVCESNVMVLMSLD
RYVAICRPLHYHNIMTKRFLVSLTFVIIIQSFALVCPFVIFPSMTQFCKSNTILHFACEHLALL
SLGCGDTTKLEKAGLIIRILVPVRDGILLMISYSTILYTAMKTATGKSRHKALNTCGTHLLVA
VLAYVCALASSSVWKVEATVSEDVKHLFTALYIVIPAASNPFIYGLRISKIRKTLVKYWRER
KILFSS*
>jgi|Xentr4|413265|e_gw1.976.19.1
MNSSDSFSHTEFILFGFPGISQSRHWLFIPFFFIYLEILMGNFIILILIWVEKSLHPPMYLLICL
LFAVNISCTTAIVPNMLMGLAFGLDHISLGSCLFQMFFVYTTLILETTVLLIMALDRYLAIC
RPLRYHNIMNNRLVGQLFLIGLVQSSLFPSPIIIVASQVQFCRSNIICNFVCENMVLLNLGCG
DISKIQVLGLMVRVLVTVMDISLLLVSYLYIFHSAMKIARGKALHKALHTCSTHLIVVVLN
SSCGLIAAILYRMPISVDVQNLFSAIYYLFPATIHPIIYGYRMKEIRRCLVKSWKIKK*
>jgi|Xentr4|413616|e_gw1.983.1.1
YLEIQALILTVDELNKAPDLLPNVTLGYHVYDSCGDPNLAIGSVLQILSGPGEPVPNYSCR
GEGEIAGFIGDRSSLTSLPIAQLLGTYGYPQISYGATDPVLNDRVQYPYYFTTGPKDYIQHV
AIAELVERLGWTWVIILAASGDYGERETKSLRKEITKHGAYVDFIGTLTQDRNTDRETLERI
QMSSAEVVILYGEQFQTNALSVLVEEMIKDKTLLVPVTWFPSYLESLFNGSLRFEEKIVEFG
YTTKFTAHVLAIEDDVLLKDLLATVSYCLTQDEEKDKFFQRVNKFIYSNCSKLQLIPRIFYP
SHRVHRAVMGLAHAAHSLLSTHQKCAKLQKICETHLSHNFLMQPCPFCSMTNGRGIPFDE
LMESRENYIIFNLYTDPDGGFSETEVGTYYWSESGSSLEINTKKIVWKKDTHNQTLKSQCS
ANCPPGYRKLLRQRAPPCCYDCARCSEGEISNATDAENCLKCGDYEWPNQEKTQCIKKQ
NEFLSYRDDSLTVAFILLSLMFFLIAAVILGIFISYRDTPIVRANNRSLSFLLLVSIKLSFLSVF
LFLGRPVDITCMLRIITFGITFSIAVSSLLAKTIMVCVAFKATKPGSSWRKWLGVKLSNSVV
LFCSSIQIIICMTWLAISPPFQELDIHTSPGTIIIQCNEGSAIGFYSVIGYMGLLAAVSFVLAFL
ARSLPDSFNEAKYITFSMLLFCSVWITMIPAYLSTKGKNTVCVEIFAILTSSAGLLACIFLPK
CYIILLKPEMNTKQYLLGNNKEANKD*
>jgi|Xentr4|413617|e_gw1.983.50.1
MLGFRPTHNPFQILIHLLVLCIRQSEAEPIKPACCLKTAKFLEDFEYIQDGDIIIGGLLSVNTN
VGWHQPEYKQNGNRVCLALQPQHYRQLVEFRLAIEEINKNPSLLPNVTLGYHIYDSCGNE
MKAVRSILQILSGTKEPVPNYSCGRKRNIAGFIGDFTSETTMISAQILSLFGFSQVSFGASDP
SLSDRVTFPYFFRTLQSFHGSALAIRKLLKHFGWTWVGIIGLNDGDKEDEPQVLTDYLSRD
GICVEFTIKANHYLTKLSDDEGNGIDKIIQTSTTHIIIVCGKLFKTFAVLYCIHYLHMLDNKT

FILSPSASVIGNNEMLTAATFDDMLIFEPYPVYPRETHEIIEFINRTHPSKDPEDTLFENIMLT
VCECLSKDPHKNKVYESFSVMNHTECDGKETTECLSTIISSLLAALSPNVHLAVNIMSQAI
HEMHTSLREQSPERDREAHRYQYQVNIPYLAHILKNTDIYLGKKITSFTSSFDINQLPATAS
KLFVNHAIVFFSARKLAHAIWRLNACDNTIIPRSQCTDSCQPGYRKAVNPGAQPCCYGCLP
CSEGEISNRTDSENCIRCPDLEWPNDKRNQCIAKIEEFLSFTNGTISVSLLSITVLFSLITLLM
LRIFISYRDSPIVRANNRSLSFLLPVSIKLSFLRCVLFLGRPVDINCMLRIITFGITFSIAVSSLL
AKTIMVCVAFKATKPGSSWRKWLGVKLSNSVVLFCSSIQIIICMTWLAISPPFQELDIHTSP
GTIIIQCNEGSAIGFYSVIGYMGLLAAVSFVLAFLAFLARSLPDSFNEAKYITFSMLLFCSV
WITMIPAYLSTKGKNTVCVEIFAIFTSSTGLLACIFLPKFYIIMFRPEMNCKSGILGGKN*
>jgi|Xentr4|413675|e_gw1.983.47.1
MLGFRPTYNPFQILIHLLVLCIRQSEAEPIKPACYLKTAKFFEDFEYIQDGDIIIGGLLTVNTN
AGRHPKEYYQNGNKLKVCFTLQPQYYRQLVEFRLAIEEINKNPSLLPNVTLGYHIYDSCG
LEIKTVRSTLQILSGTKEPVPNYSCGRKRNIAGFIGDLTSETTVISAQILSLFGFSQVSFGASD
PSLSDRVAFPYFFRTLQSFHGSALAISKLLKHFGWTWVGIIGLNDGDMADEPQVLTDYLSR
DGICVEFTFNYNHYLTHVPSDEGYRMHGIIPKSTTHIIIVCGKSLKTVVFVYYMRYSMLEF
NKTLILSPSASFIGITEKFTGATYDDILIFEPYPVYPRETHEIIQFINRTHPSKDPEDTLLENIM
LSVCRCLSKDPHKNKVYETLFHIRYTECDGKEATECLLRIISSLLAALSPNVHLAVNIMSQA
IHETHTSLREQSPEKDREAHRYHYQLHHYLNKSHYQTKYGGEVSFDGRGEMDTGYIIYFF
PNETDGTYLGTLGRFIPSAPSEYKLIIYPDAIPWKMKNKTIPRSQCTDSCQPGYRKAVNPGA
QPCCYGCLPCSEGEISNRTDSENCIRCPALEWPNDKRNQCIAKTEEFLSFTDGAISVSLLSIT
VLFSLITLLMLRMFISYRDSPIVRANNRSLSFLLLVSIKLSFLSVFLFLGRPVDITCMLRIITFG
ITFSIAVSSLLAKTIMVCVAFKATKPGSSWRKWLGVKLSNSVVLFCSSIQIIICMTWLAISPP
FQELDIHTSPGTIIIQCNEGSAIGFYSVIGYMGLLAAVSFVLAFLARSLPDSFNEAKYITFSM
LLFCSVWITMIPAYLSTKGKNTVCVEIFAILTSSAGLLACIFLPKCYIIMFRPEMNHKSSLLG
SKT*
>jgi|Xentr4|413963|e_gw1.993.34.1
MFLCAADGCGGRPVVFCIILGYIVLCSVFPCRCGAQAENPACQLNIIETYEDYEYIQEGDIM
IGGILAVNSEMIEYRKAGEGYKRKMCVVVLPQYYRQLVDFRLLIKIINHKRSLFPNLTLGY
HIYDSCGDAQKAVRSVLQILSGTREPVPNYSCVGKRNIAGFIGDVYSATTVPIAQILSLFGY
SQISSGATDPLLSDRVTYPYFFRTVQSDHHYYLAFSQLLKHFGWTWIGIIRLDDYEGGKEH
QLLMKYLSSHGICVEFSVKISSFIDQTSSGWNEYNKDMQSKQVIQKSSARVILLCGMVSGT
SVESLQKFTEEFIERTLILSYTIYVNYHMMDYAIDIFNGSLGLIQYLEYSLQGPILKPYLESIR
PSNYPEDKLLEDIWMRYHLCLSKDNSKNNVYKQVYKDELRNCTGKELITNVRHFDNPLH
SPHVAFAAYILTHAIHIMQMSIDQEGPKKSKNMYNYKHQLHHYFKNIPFKTNTTLNFEENG
EFLTHYRIYYYTMKSYSEIHMEILGWFLSRGLSDQQLYIGPYDVRWKTNNSQIPKSQCSDN
CLPGFRKAPDHGTHSCCYHCVPCSEGEMSNLTDSENCIRCPDMEWPNEKRNQCIAKTEQF
LSYTNDVISDAFISVSILFSLITVIVLGIFVLYRDSPIVRANNRSLSFLLLVSIKLSFLSVFLFLG
RPVDITCMLRIITFGITFSIAVSSLLAKTIMVCVAFKATKPGSSWRKWLGVKLSNSVVLFCSS
IQIIICMTWLAISPPFQELDIHTSPGTIIIQCNEGSAIGFYSVIGYMGLLAAVSFVLAFLARSLP
DSFNEAKYITFSMLLFCSVWITMIPAYLSTKGK
>jgi|Xentr4|414099|e_gw1.999.55.1
MASGSKENVSGFIIQGFSDTPELHISLFVLFLGIYLIILLGNLIIFLVISCNPHLHTPMYIFLLIL
SLIDISSTSNIFPNLLHILLTQQNNISFLGCMTQLYVFGSVASSEFFLLTAMAYDRYVAICDPL
HYIARMSRKHCAGLITAAFTVGFVEPFGIVVLISKLSYCASHLINHFFCDVTPLLQLSCSSTF

SVELFIYIEVTLLVFSSFLLTLTSYIFIISAILKIQSSEGRQKAFSTCASHLACVITLYGTVFCLY
MRPTTIVCLYLRPKIFYFSLLYIALAPVVNAPYLHTEKQRVSIFP*
>jgi|Xentr4|414588|e_gw1.1012.12.1
MASRRQANISGFIIQGFSDTHEHYISLFVLILGIYLIILLGNLIIFLVISCNPHLHTPMYIFLLNL
SLIDISLPSDILPNLLHILLTQQNNISFLGCMTQMYVFLSLAATEYFLLTAMAYDRYVAICDP
LHYIARMSRKHCAGLITAAFTVGFVDTVGHVTLIPKLSYCASRLINHFFCDITPLLKLSCSS
TFSVELVIYIVGTFFFVNSFLLILISYIFIISAILKIQSSEGRQKAFSTCASHLACVITLYVTVFC
LYLRPQNNYSLQRDKFFSLLYIALGPVLNPLIYTLKNREIKSSFNKMRKRMVFILNK*
>jgi|Xentr4|414591|e_gw1.1012.40.1
MTSASKENVSGFIIQGFSDTPELHISLFVLFLGIYLIILLGNLIIFLVISCNPHLHTPMYIFLLNL
SLIDISFMSTVLPNLLHILLTQQNNISFLGCMAQMYLFGSMVGSEYFLLTAMAYDRYVAIC
DPLHYIARMSRKHCAGLITAAFTVGFGESVGLIVLIPKLSYCASRLINHFFCDVAALLKLSC
SSTFSVELVMYIEGTFLFFNSFLLTLISYIFIISAILKIQSSEGRQKAFSTCASHLACVITLYGTA
FCLYMRPTTSYSLKRDKYFSLLYIALGPVLNPLIYTLKNREFQSSLNKMRQKSSGFCFFLI*
>jgi|Xentr4|414613|e_gw1.1012.68.1
MASGSQGNVSGFIIQGFSDTPELHISLFGLFLGIYLIILLGNLIIFLVISCNPHLHTPMYIFLLN
LSLIDISFSSTVLPNLLHILLTQQNNISFLGCMTQIFVFGSLAGSEYFILTAMAYDRYVAICDP
LHYIARMSRKPCAGLITASYTFGFSESISIVTLISKLSYCASHLINHYFCDVTPLLKLSCSSTF
SVELLIYIEGTLLIFSSFLPILISYIFIVSAILKIQSSEGRQKAFSTCASHLACVITLYGTVICLYV
RPTTSYSIKRDKYFSLLYIVLGPVLNPLIYTLKNKEFQCSLNKMKQRFVSFFVGNASQ*
>jgi|Xentr4|414627|e_gw1.1012.47.1
MTSGSHENVSGFIIQGFSDTPELQIPLFVLILGIYLIILLENLIIFLVISCNPHLHTPMYIFLLNL
SVIDISFSTNILPNLLHILLTQQNNISLLGCMTQIYVFGFFTCSEYFLLTAMAYDRYVAICDPL
HYIARMSRKHCAGLITAAFTGGFVEPFGIVALISKLSYCASHLINHFFCDVAPLIKLSCSSTFI
VELLIYIEGTLLIFNSFLLTLASYIFIISAILKIQSSEGRQKAFSTCASHLACVITLYVTVICLYM
RPTTSYSLERDKYFSLLYIALGPVLNPPIYILKNREFQSSFNKVRNLVMPTNKISIKPE*
>jgi|Xentr4|414656|e_gw1.1012.41.1
MAIENQGNLSGFLIQGFSDTPRFHLSLFVLFLGIYLIILLGNLIIFLVISCNPHLHTPMYIFLQN
LSLIDISFSSNILPNLLHILLKQQNNISFLGCMTQMYLFMALACSEYFLLTAMAYDRYVAIC
DPLHYIARMSRKHCAGLITAAFTVGFVDPIGLVVLISKLSYCASHLINHFFCDISPLLKLSCT
KIFSVELLIYIGGTLFTFSSFLLTLTSYIFIISAILKIQSSEGRQKAFSTCASHLACVITLYGTVL
CLYMRPTTSYSLERDKYFSLLYIVLGPVLNPLIYTLKNREFKSCLHKVRQRFSIKPFR*
>jgi|Xentr4|415359|e_gw1.1033.6.1
MLILAGSLLIILATSDVYLEPRAACSIQKYHFPTNFKAGDVVIASFVQLYSDFYLERNDFTQ
YPEEIHCSSFLSLRHYLAFIYAVEEINKNSQILPNITLGYQIFDSCGCDFKAISGVLDTMSGA
EQTIVNYDCRRNSKLVGYLGDFSSGTTYAMAQFLAMFRYPQACIHLCYINYGSMDPVFHD
RTQFPSFYRTIPNEEAEMDGIVQILKHFGWKWVGLIVSDDDTGYRARERISTELARMGGTT
YADDFRYEVFDLRIPKGFFIASSSLADIVTDHYMVQGAMLNGTLSLLIQAGEIPGFRHFFKS
FSPNNYPRNNVLEDIWHDVFRCYFPSSPWGNDTLCTGNKSLTDEVEYKLGDYRVTYRVY
TAVYALARALHNLYSAQVPTNHWDKLEYMRGNLKPWQLNTYVRNVTFTTPSGDTIFFND
KGDPPAQFDVMKLSLLPDGRVAKEKVGSFHVLSDGTKLLHINSSADLWGPYYKEMPQSL
CNEPCAPGYRKAKIEGKPSCCYDCAKCADGEMSNTTDALDCLHCSDYEKSNKQRTGCVP
KEINYLSYTDTLGASLTSIALVLFIAASVVLGIFVRYWETPIVRANNQHLSFLLLISLMLCFL
CTLLFIGRPTQICCLLRQVTFGIVFTISVSSVLAKTLTVIIAFNATKPGSKLKKYVGTQLATIL

VTVCCLGEMMISAVWMASNPPFLDADTLTDINTVFLMCNEGSVLFFFSVIGYMTALALFS
FIAAFLAKDFPDRFNEAKNITFSMLGFCSVWGAFVPAYLSSKGSRMVAVEIFAILSSSAGLL
ACIFVPKCYIIFFKPEQNRRRM*
>jgi|Xentr4|415669|e_gw1.1042.29.1
MTPYNLKLLISLMLLWTKTYRAEFQHKNLACQLEMLKLYEEYEYIQEGDIMIGGVLTVNS
YMKLLELPEDNSMRMLCMEVVPEHYRQFIDFRYFIEQTNNNTALFPNLTLGYHIYDSCGD
PRKAVRSVLQILSGTREPVPNYSCVGKRHIAGFIGDLTSETTVPIAQILTLYGYSQISYGATD
PLLRDRAAFPYFFRTVQSDHHHCYLLTELLKYFGWTWVGVIRFDDDAGDREFQLLTKYFS
NNGICIEFSTKINIDNFKSHEHITNKHKELVRKSTTSVIVLCGTVSAAVIVGLRILKDVLKEK
TFVLTTNWAANHMMNFATEVFNGSLGFMQCSLYSLNSPELKAFIASIHPSKYPKDKLLEDL
WMQYHFCSSSNEYKNKVFKYVYPQGSLYYCTGEQRIQDIWNIANALHSPRVHLAVTLLS
QAMYKMHIKLSPKLDNIIYDYRYQLHHYLNVLRYEVQGAPYTFDENGEWKSRYWIYNY
MIEDKKKKIHTKYFEFNPLAPSEQKLFPTNIRWKKSQKPRAQCSANCPTGFRKASKPGTHS
CCYGCAQCSEGEISNVTDSESCIRCPDMEWPNEQRNQCIARTEEFLSYTNDVISIFLPSLSV
VLYLCTVLILAIFIKYRDSPIVRANNRSLSFLLLVSIKLSFLSVFLFLGRPVDITCMLRIITFGIT
FSIAVSSLLAKTIMVCVAFKATKPGSSWRKWLGVKLSNSVVLFCSSIQIIICMTWLAISPPFQ
ELDIHTSPGTIIIQCNEGSAIGFYSVIGYMGLLAAVSFVLAFLARSLPDSFNEAKYITFSMLLF
CSVWITMIPAYLSTKGKNTVCVEIFAILTSSAGLLASIFLPKCYIILYRPEMNTRTFVFANKI*
>jgi|Xentr4|415682|e_gw1.1042.34.1
MKTKCVPKAVEYLLYSDGISLFFSSTSGFFFLINLLILGTFTLYQSSPIVRANNRSLSFLLLVSI
KLSFLSVFLFLGRPVDITCMLRIITFGITFSIAVSSLLAKTIMVCVAFKATKPGSSWRKWLGV
KLSNSVVLFCSSIQIIICMTWLAISPPFQELDIHTSPGTIIIQCNEGSAIGFYSVIGYMGLLAAV
SFVLAFLARSLPDSFNEAKYITFSMLLFCSVWITMIPAYLSTKGKNTVCVEIFAILTSSAGLL
ACIFLPKCYIILFRPEINAKCHLLTKQTRSF*
>jgi|Xentr4|416238|e_gw1.1063.24.1
VETCVKCAEDQWPNPTRDQCIIRVIDYLSYEDLLGYILSGCASVFTVLNSAVLLVFIKHRRT
PIVRANNQNISYILLMALLMSFLCTFMFIGQPTGVTCMLRQTTVMFVLSIAISSMLGKTLM
VLAAFQATKMEKMFRKLGRINISVGVVFLCSFVELIICVIWLSLYPPHVESDNKTVPGKIIL
QCNEGSIISFYLAVSYIGVLSLISFAVAFIARKLPDRFNEAQHITFSMLVFCSVWVSFIPTYLS
TKGKHMVAVEIFAIQASAAGLLMCIFTPKCYIILLKPELNVKGKPRVTH*
>jgi|Xentr4|416251|e_gw1.1063.54.1
MFKTPTSVCMEPCSSGHRRAHQNGRPPCCFDCIPCSEGEISNYTDVESCVKCAEDQWPNP
ARDQCIKRVIDFLSYGDLLGYILSGCASVFIVLTAAVCLVFIKHRRTPIVRANNQNISYILLM
ALLMSFLCSFIFIGQPTGVTCMLRQTTFVFVFSVAISSVLGKTIMVLIAFKATKIEKMFRML
GRMNISVGVVFICSFGEFVLCVIWLILNPPHVESDNKTVPGKTILQCNEGSIISFYLAVSYIG
VLSLISFAVAFIARKLPDRFNEAQHITFSMLVFCSVWASFIPTYLSTKGKHMVAVEIFAIQAS
AAGLLLCIFTPKCYIILLKPDLNVKGKPTAKIQSEGKGN*DGKGMESLSYEERLAKLGLFT
LGKRRLRGDMITMYKYIRASYNNLSNV*
>jgi|Xentr4|416262|e_gw1.1063.1.1
AFMYAIEEINNSTELLPNITLGYHIYDACTSEEMGLMSTISLLSEEENPAFNYICQPKQKLVA
FVGHLLSSTTYTIAEITQLYGYPQISYGALDPVFNDRIHFPSVYRTVPNEYSQFRVIIKLLKH
FGWTWVGIITSDDESNHQASEELRKEMGRNDICVDFVKTMSNSPASAHISAIKVVEVIKHS
SVRVIVLYCRTNSLLALLMSKTFKKISERVFICSVALDIYIEPTHERLFHSINPLSASRIRHFKE
IPGFNSFFSHRLWNDMLKNIFVKSFFEFHTECLNAHDDSVKNLTCLKNNKIKEYLLQEETI

NHRIKHTIFMAVYALAHALDSVQLHREFSQMSSKKIGKMIFKLNFYLKKLHMKTASGEDF
FFTKDGNTPGKFDILNWIIYENRTVHKIHVGRFLPNTDQLIIYDTAIAWGPYIQKTPTSLCTE
TCSSGQRRAHQTGRPPCCFDCVSCSEGEISNSTDMETCMKCAEDEWPNPTRDQCIIRVIDF
LSYEDLLGYILSGSASVFIVLTAAVCLVFIKHRRTPIVRANNQNISYILLMALLMSFLCSFIFI
GQPTGVTCMLRQALFIFSFSVAISSVLGKTITVLIAFKATKMERSFRKWGRINISVVVVFICS
FGEFVLCVIWLSLYPPHVESDKTIPGKLVLQCNEGSIISFYLAVSYIGVLSLISFAVAFIARKLP
DRFNEAQHITFSMLVFCSVWASFIPTYLSTKGKHMVAVEIFAIQASAAGLLMCIFTPKCYIIL
LKPELNVKGKPTAKIQSHTANHMPLC*
>jgi|Xentr4|416267|e_gw1.1063.3.1
QISYGALDPMFNDRIHFPSVYRTVPNEYSQFRVIIKLLKHFGWTWVGIITSDDDSNRQASEE
LRKEMEKNGICVDFLKAISSSPDLREKRAIEAVETIKNSSVRVIILYCRASSLFVFLGFQTSH
QISERVFICSVALNIVTELGFNKLLYSMNGSFLISLPKQDIQGLNNFFSHRLMTDMNKNIFV
NRFLMLNIGYSYGQSGGVINSSRLNYDMIKEHLLQEETITHRIKHTIYMAVYALAHALDK
MQLSREFLEMSSKEKISKMRLKLNYYLKNIHLKMASGEEFFFTEDGNIPGKFDVLNWVID
KNGAVNRIHVGRFLPNTDQLIINETAIAWAPYFGKTPTSVCMEPCSSGHRRAHQNGRPPCC
FDCIPCSEGEISNYTDVERCEGCAEDQWPNPAKDQCIKRVIDFLSYGDLLGYILSGCASVFI
VLTAVVFLVFIKHRRTPIVRANNQNISYILLMALLMSFLCSFIFIGQPTGVTCMLRQTTFVFV
FSVAISSILGKTIMVLIAFKATKIEKIFRMLGGINISVGVVFICSFGEFVLCVIWLILNPPHVES
DNKTVPGRTILQCNEGSIISFYLAVSYIGVLSLISFAVAFIARKLPDRFNEAQHITFSMLVFCS
VWVSFIPTYLSTKGKHMVAVEIFAIQASAAGLLMCIFTPKCYIILLKPELNVKGKPTAKILSH
MENHTPHC*
>jgi|Xentr4|416293|e_gw1.1063.12.1
MVSADKLDYTYIKFFFFFFQTPFSMCTLPCTYGFRTAHQRGKAPCCFDCIPCPDGEIANGT
NMENCLRCPEDKWSNFLKDDCIERTKDFLSYGDILGVTSSCIAFIFVVITAAVLFVFVKFRR
SPIVRANDRNLSYILLMSIMLSFLCSFLFIGYPVELTCMLRQAAFGFIFTVAVSAVLGKTVTV
IIAFNATKPNSTFRKWIGTRISIGLVLFFSLGELFICIIWLICSPPFVDTDTKTIPGTIIIQCNEGS
FAAFYVVISYIGLLALFSFIVAFLVRKLPDRFNDAQYITFSMLVFCSVWISFIPIYLSSKGKYV
VAVEIFTILSSTGGLLLCIFAPKCYIILVKPELNSRKHILKPKL*
>jgi|Xentr4|416337|e_gw1.1066.37.1
MALILLFRMLIYIVSIKNAGYCHSTLQSMNSACKLQILKTFEDYEYVKEGDLMIGGVVTV
NAVAKRYSDDTYGKLLMCFDPSPQNYKYLLDFLYIIKTFNENPNMSQNLTLGYHISDSCGN
VYKAERSVLQILSGLRDPVPNYSCAGKRNIVGFIGDLTSETTIPIAHILNVLGYSQISYGATD
PSLSNRDTFPFFFRTVQSKKGQFLVITLLLKYFAWTWVGIITSNDINGDGAYQLLKNDLSGE
GICIEFVISVESGENVYTESSLHKGIIQRSSTSVVIFCGTINIQTALALRELSDVLIEKTFIFTSD
WLYYSHVFNHVFRLFNGSLVFMQNRETYKEKNPQLIQFLESIHPSKYPEDKLLEAIWVQR
HSCISENMTLSGFHKILFPQNQLKKCRGKKRLMDIEAFREDLHTNNMILAINALCNVGSRL
HFMHILLSEQSPEKYRNVPHYKYQMHPFLKKLNFKYKDQILMAFNENGECVSPYQITNFY
SRSKEYITETQVGRYVPWAPFDQKLNISLDKIKWKTKYKKVPRAQCSDNCLPGFRKALKP
GAQSCCYDCVPCSEGEISNTTDSENCIKCSDMEWPNEKKNQCTEKMEDFLSYTDDVISVF
FSSISVLFFVITVLILRVLIIYRDTPIVRANNRSLSFLLLVSIKLSFLSVFLFLGRPVDITCMLRII
TFGITFSIAVSSLLAKTIMVCVAFKATKPGSSWRKWLGVKLSNSVVLFCSSIQIIICMTWLAI
SPPFQELDIHTSPGTIIIQCNEGSAIGFYSVIGYMGLLAAVSFVLAFLARSLPDSFNEAKYITF
SMLLFC
>jgi|Xentr4|416340|e_gw1.1066.39.1

MIGGTTTSYPIKVLIYLAAMCVRPCRSGDQPNNPACHLDIMKEHEEYEYIQEGDIMIGGVL
TVSRSKGDINMNIFDCACPSPERYRYLVEFLFLIKEINENPVRLPNVTLGYHIYDSCGNTRR
ALISLLKILSGTRVPVPNYSCVGKRKFAGFIGDLDAEPTVSMAHILGVLGYSQISYGATDPA
LSDRVAFPYFFRTVQSDEEEYIALCKVLKYFGWTWVGIIYFDNYSGYRDHQLLTKYFSREG
ICAEFMLKIRMNVDEYYLHIDTIEKSSSGVIIICGDFDYASESALLYLRGSVRKKTCIFLSKW
LHHVNGLETTVALVRGSLSFMQNRLNDRFDPKFTEFSKTFHPLTYPHDKFLEKLWMFFLF
CLSKDDKKNRYFMWDYHIPLRNCSGKEKLTDIPEYLNAYHSASLIQAVDIMVIALQDMYN
SHSKQTHGKRTWMDNHNQLYLYLKNISYENKDNLVMSFNERGEMANKYFIVNPYIDTKN
YSMEAVGYYVPWAPFNQRLSLTPQKIIWKTPNNKTPRAQCSDYCFPGYRKVPKPGAQSCC
YDCVPCPEGEISNTTDNKNCIQCQDMERPNSRRTKCTAKTEEFLSYTNDVISIILSLISVSSF
FISVLILGVFITYRDTPIVRANNRSLSFLLLVSIKLSFLSVFLFLGRPVDITCMLRIITFGITFSIA
VSSLLAKTIMVCVAFKATKPGSSWRKWLGVKLSNSVVLFCSSIQIIICMTWLAISPPFQELD
IHTSPGTIIIQCNEGSAIGFYSVIGYMGLLAAVSFVLAFLARSLPDSFNEAKYITFSMLLFCSV
WITMIPAYLSTKGKNTVCVEIFAVLTSSAGLLACIFLPKCYTIFFRPEMNTKSHLYGNKS*
>jgi|Xentr4|417095|e_gw1.1085.53.1
MDIANQTQVSEFIIVGFPDVKGLQTLLFPLLLIIYLFTISGNIIVISLIWTHRHLHVPLYIFVAIL
SFLEIWYTAVTIPKMLANLLNNKTISYHGCLLQIYFLHCLGITETYLLTAMAYDRYLAICNP
LRYPSIMTPSCCFQLAGFCWFIGLIGPLAQIILLSRLYFCGLNKVEHIFCDFAPLINLSCSDTS
LNFVVDFTINAVIICVAFTCIICSYAKILSAVLKISTKEGRKKAFSTCGAHLTVVTLFFGSVAF
MYIRLTKSYPANYDRSMAVIYSVLTPMCNPIIYSLRNQEIREVMRKKLSKLM*
>jgi|Xentr4|417101|e_gw1.1085.10.1
MGNQTVVSEFFLVGFSDIPQWNMLLVLAFTFSYIWTLSGNTFILSLIIFDSGLHTPMYFFLS
NLCVLDICCISVTAPQFLYMFYHLHYSVSFSNCITQVYFYLAFTNVQFFLLPVMAYDRYRA
ICNPLRYHVFMSKRACVSLAVGTWAFGFINTLSYRISHLIYCISPVINHFFCDLTAVMKLSCS
DTSTIEHFTFIEGVLCGFLPLFLTVASYIDIIRSIMKIHSIKGRRKAFSTCSSHLTVVTLFYGTII
FMYVRPTSKYSPAQDKLYAAFYTTVIPMMNPLIYSMRNRDVKVAFRKMIRDVTWFSKKM
*
>jgi|Xentr4|417107|e_gw1.1085.12.1
MDVTNQSVIREFIIVRFPERNGIQLLLFPFFFIIYLFIICGNVTIITLICSHQSLQVPLYFFVAVLS
FLEIWYTTVTIPKMLASLLDQKNISYNSCLAQVYFLHCLGITETYLLTAMAYDRYLAICNP
LRYSSIMTNRCCLQLSACCWLTGLLGPITQITLLSRLHFCNANKIEHIFCDFNPLISLACSDT
TLNVRVDFYINSFLLFLAFACIILSYVKIISAVLRISTEKGRKRTFSTCSAHLTVVILFFGSVTF
TYIRLTKNYPLNYNYSMAVIYSVLTPMCNPVVYSLRNREIKELLKKKIRTFWETT*
>jgi|Xentr4|417118|e_gw1.1085.16.1
NQTSVTEFILLSLTDILEFRITLFGVFLAFFLLNLTGNISILVITILEKALHTPMYYFLGNIAFF
DIFVSYVTVPKMLTDFFFLQKTISFNSCISQLHFFHFLAGSEVVLLTVMSYDRLIAIGNPLRY
SSIISPTFCVILIFGSWVFGFLHSLLHTVLTAMLPYCGPNLVEHFFCDIKPLLKLACADTTLN
QKILNILTGYLTCMAFLLTVISYVLIGRIILKMKTVEGRKRAISTCSGHLTVVILLYGTALFT
YIRPATQDILDQDRGAAVLFTVVTPALNPIIYTLRNKEMKKAIRRVIKKLTL*
>jgi|Xentr4|417137|e_gw1.1085.13.1
MDSKNQTSIGDFTLIGLTDLVEIQCIVYGIFLLFYCLTLLGNLSIMITTIRDSNLHTPMYFFLW
NLSFIDIGFSSVSVPKMLMDFFATRKLISFGGCMSQICFFHFFGTAEVMLLAVMSYDRFVAI
GNPLRYILIMNSKVCLSLALSCWISGFFHSLLHTVMTAKLPYCQANLVNHFFCDVKPVLKL
ACTDTSFILQLLTRVAGSVSTITFAFTFLSYFFIIKFVIQIRTKKGRRNAFSTCSAHLTVVFML

YGTAIFTYMRPLTQQSLDHDRVVAVLFTVVTPALNPLIYTLRNKEMRVALKRIYKNVFYL*
>jgi|Xentr4|417234|e_gw1.1088.8.1
MVFVPVLFLSSLFFLLPIVQALPCTLHSPQETGFIKDGDLMISGLFPVHTAWIDTKYLFTER
NPPLTCSSFSTVLYSYVQAMVYAIEEINANDSLLPNISLGYKIFDSCMHLFQSLRGTLWALT
GQDHPILNYECHPNVPIAIIGDATSGTSVAMATLLGVYRYPQVSYSASMSSFSNHFLYPSFF
RTIPNDAMQSLALAHLISYMGWTWVGLISLDDDYGIDGSHVLKQELYKLAVCIAYHESFGI
ESSSKRIQSVANIIDQSSARVVIIYSRDPFVSMLMEYFVERQDTKRIWLATTGWSNSQMLPT
KLFSHTVMGTLGLSLHQAEMPWLKKFLLNIHPSTSLPYDIFIKTFWEIVHNCQWPEEYSVV
TNGTVWCTGEEKLLRFKSSVYEFDEFDFRVQYLVYNAVYAVAYALHNLIYCLTDEEPFQW
KLCKNKTDVTPWQVYHHLRKVSFVNRMGEQMYFDQHGDPPAYFDIVNWQRSQDGTIQF
LKVGWYDARAPLGKEIVINTTAIQWITGDTEVPPSVCSESCAPGYRKAAEKGQPICCFDCI
RCSEGEISNQTDSSNCFPCTDETWPNEDRTECLPKPVDFLSFEEPLGITITCTTIFSSFSTLTIL
CVFIRYKDTAIVKANNRDLTYVLLGSLALSFLCPLLFIGQPHRFTCLFRQVTFGVIFVLSVS
CVLAKTIMVVIAFRATKPGSNMRRWLGPIVPGLIVSACTNLQLFICIFWMLYCPPFPERNSR
IKIGVIVFQCNECSDTLLWCMVGCMAFLSCVCFLVAFLARKLPDTFNEAKWITFSMLIFLS
VWIAFAPAYLSTQGKYMAAVEVFGIICSSAGILVCIFLPKCYIILLRPDLNTRGNILNNRSTY
NT*
>jgi|Xentr4|418131|e_gw1.1101.4.1
YRHYVAFLFAVEEINRSSRLLPNISLGYRIYDTCDHEPKSLYDALAVMSGAEYQVPNYDCW
EKAKLMGYVGDVPSSTKFSLAQLISMFRFPQAIHYNTMDPSLNDRTQFPSFYRTIPNEEAE
MDGIVQILKHFGWKWVGLIISNDATGYRGRERISKELAGIGGCLAFTVKLNSTAYINFPHAI
KVLKQIKNSSAHVIVLYIGTKYANGFGNMFALYQIPTKFWITSSFFLNVVNGRLKEIGTTLN
GTLTILVQEGEIPGFEQFFYRFSPNSYNDDLTKSTWESLFLCVFPKFAKSQFVKGSLVENCT
GNEFLNVDLSMYGYRIYVHTYRVYTAVYALARALHNLYSAQPPANHWGRLENLIRRVKP
WQVNKYLHNLNFTTSSGDTVFFNDKGEPPAAFDIVKWFFQPNGRAMRQKVGQFNLLNN
SEKQLYINGSADIWGPYFKQVTDEDISKCESCSEYQKSNTERTECLLKAINFLSYTDTMGA
SLTAIAFILFITASVVLGIFVKYWETPIVRANNQHLSCLLLISLMLCFLCTLLFIGRPTQICCLL
RQVTFGVVFTISVSSVLAKTLTVIIAFNATKPGSKLKKYVGTQLAIVLVIICSLGSTVISAVW
MASHPPFLEADTVSEMDTVILMCNEGSVFFFFSAIGYIGTLALLSFIAAFLAKDFPDRFNEA
KNITFSMLGFCSVWGAFVPAYLSSKGSRMVAVEIFAILASSAGLLGCIFTPKCYIIFLRPELN
TRTF*
>jgi|Xentr4|418140|e_gw1.1101.22.1
YYRHYLAFLFAVEEINRSSRILPNISLGYRIFDSCEHEPKSLYDALAVMSGAEYQVLNYDC
WEKAKLMGYVGDVPSSTKFSLAQLISMFRFPQATYVLFFFKISYNTKDPALSDRTQFPSFY
RTIPNEEAEMDGIVQILKHFGWKWVGLIISGDDTGYRGRERISKELSNNGGCLAFTAVLRD
AAYIYNYNAEEIIQQIRESSAYVIVLYIGTRYVPAIAALFANIFGLPEIPRKFWITSSFFAKLILY
RRDNIERTLNGSVSLLIQEGEIPGFDQFFYSFSPYNYRDDLTKDVWQWIFGCHFPAGSYAET
LSTYGEVAYNCTGNETIADVNLFKYGDHNYRVTYRVYTAVYALAHALHNLYSAQPPANH
WGRLETLKRRVKPWQLNKYIRNLTFTTSTGDADSFNDKGDPSAFDIVKWFFLPDHRVMR
QKVGEFNQLNNSGKQLYINGSTDIWGPYSKQMPLSQCNEPCAPGYRKAKIEGKPSCCYGC
VRCADGEMSNTTDAANCERCSEYQKSNTERTECLLKAINFLSYTDTMGASLTAIAFILFITA
SVVLGIFVQYWETPIVRANNQNLSCLLLISLMLCFLCTLLFIGRPTQICCLLRQVTFGIVFTI
SVSSVLAKTLTVIIAFNATKPGSKLKKYVGTQLAIVLVIICSLGSTVISAVWMASHPPFLEAD
TVSEMDTVILMCNEGSVFFFFCAIGYMGAPAVLSFIAAFLAKDFPDRFNEAKNITFSMLVF

CSVWVAFVPAYLSSKGSIMVAVEIFAILASSAGLLGCIFAPKCYIIFLQPELNTRTF*
>jgi|Xentr4|418143|e_gw1.1101.1.1
VNISPRPSFQYYRHYLAFLFAVELINRSSRILPNISLGYRIFDSCEHERKSFYDALAVMSGAE
YQVPNYDCWEKAKLMGYVGDVPSSTKFSLAQLISMFRFPQATLVNPIDPALSDRTQFPSFY
RTIPNEEAEMDGIVQILKHFGWKWVGLIISNDDTGYRAREKISKGLSSVGGCLAFTAVLREI
AHISYHHSEEILQQIRKTSAYVIVLYIGTRYASAFTSIFAISRIPTKFWITSSFFPKVTIFRHQKI
KTTLNGSLSLLIQEGEIPGFEQFFYRFSPNKYNDDLTINTWAWLFGCSFPPHVYVRLQTGGT
AKNCTGKETMSAADVSVYGDHNYRVTYRVYTAVYALARALHNLYSAQPLANHWGQLRT
LKRKLNKYIRNLTFTTSTGDTVAFNDKGDPSAFDIVKWFFLPDGRVKRQKVGQFNLLNKN
EKQLYINGGADIWGPYSKQMPLSQCNEPCAPGYRKAKIEGKPSCCYGCVRCADGEMSNT
TGKSQSLDQKPMRCSEYQKSNTERTECLLKAINFLSYTDTMGASLTVIAFILFITASVVLGIF
VKYWETPIVRANNQYLSCLLLISLMLCFLCTLLFIGRPTQICCLLRQVIFGVVFTISVSSVLA
KTLTVIIAFNATKPGSKLKKYVGTQLAIVLVIICSLGSTVISAVWMASHPPFLEADTVSEMD
TVILMCNEGSVFFFFCAIGYMGAPAVLSFIAAFLAKDFPDRFNEAKNITFSMLVFCSVWVA
FVPAYLSNKGSRMVAVEIFAILSSSAGLLGCIFAPKCYIIFLQPELNTRTF*
>jgi|Xentr4|418148|e_gw1.1101.26.1
MIVPANQTTDMGVILLGFPQDPTTNVTTFLLFFLAYLATCIGNCLIICIILLTPSLRIPMYYFIC
NLSFLDLCYSSSVVPKLLADVFSTQRTISHKACIAQLYITMLLGGAEGLLLAMMAYDRYV
AICYPLYYGRLMSSKMCYLMSVCVWIGSFLLSVVPSLAMRVTYCFNKEINHFMCEILEMI
KLVCEDIYVKEVTLHAISSICLIIPFAFIIGSYVCIIATVLKIPSLGRWKTFSTCTSHIIVVFFCY
GTAMIMYFGPASNYSANQLKFASIFYVVITPMLNPIIYSLKNKDVKDAFKKLLSKMTL*
>jgi|Xentr4|418597|e_gw1.1115.71.1
MENQTTVYTLVLAGLSDLPSLQLPLFLVFLLIYLITLTGNLLILLLIFTDSHLHTPMYFFLAT
LACLDIGYSSVTVPRMLFDLLTGRRVISVRDCITQIYFLIYFALLEVSVLAVMSYDRYVAIC
HPLHYMQIMNWKVCVQLVSGILGFAAIYTMVHTLFLTKLTFCRPDALQSFFCDLPQLLQV
SCSDTFINVLLIFLFGIFYGGVVLSVIIYPYITIIRTVLKISSKAMRSKAFSTCSSHLTVFFIFYS
TIIFNYFRPNAKYHLTEDKVASIFYAILTPFLNPLIYSLRNQELKISLRRMLQNVDCRHKIAQ
TR*
>jgi|Xentr4|418608|e_gw1.1115.22.1
MENQTIVYMLVLAGLSDLPSLQLPLFLVFLFIYLLTLTGNLLILLLIFTDSHLHTPMYFFLGT
LACLDMSYTSVTAPRMLYDFLRRRRAISIPACITQIYFFICFGVSETSVLAVMSYDRYIAICR
PLHYMQIMSWKSCVQIVSGMLVFSVLYSLMHTLFLNKLTFCRSDTLQSFFCDLPQLLEASC
SDTFINVLLIFLSGILFQLGIVVVTFFPYITIITTVLKIPSKTMRSKAFSTCSSHLTVVFIFYSTIF
FNYFFLNTNNSTEDKVASVFYAILDPFLNPLIYSLRNQELKMALTRALQKL*
>jgi|Xentr4|418612|e_gw1.1115.3.1
MENQTIVYILLLAGLSDLPSLQLPLFLLFLLIYLMTLTGNLLILLLIFTDSHLHTPMYFFLGT
LACMDLCSSSVTAPKLLFDLLIQKRVISVRACITQIFFFLCLCGSEISVLAVMSYDRYIAICRP
LHYMQIMSWKVCVHLVLVVFVLSAVHSLVHTLFLTKLTFCRPDVLQSFFCDLPQLLQVSCS
DTFINVLLIFVVGIIFGLGILALTFFPYIAILRTVLKIQSKTTRSKAFSTCSSHLTVVFIFYTTIFF
NYFRSNANDHFAEDKVSSVFYTILIPFFNPLIYSLRNQELKISLRKTLQTLQIKNYSD*
>jgi|Xentr4|418624|e_gw1.1115.66.1
MYFFLGTLACLDMSYSSVTVPRMLFDLLTGRRVISVQHCITQIYFFLFFAVSEMSVLAVMS
YDRYIAICRPLHYMQIMSWEVCVQLVSGILSFASIYTLLHTLFLAKLTFCRPDALQSFFCDL
PQLLQISCSDTFINVLLIFLSGILYGGVALSVIIFPYITIIRTVLKIPSKAMRSKAFSTCSSHLTV

VFIFYSTIIFNYFRPNAKYHLTEDKVASIFYSTLTPFLNPLIYSLRNQELKTSLRRMLQTVNFS
GGKVAQTM*
>jgi|Xentr4|418625|e_gw1.1115.21.1
MLLLVGLSDLPSLQLPLFLVFLLIYLITLTGNLLILLLIFTDSHLHTPMYFFLGTLACLDMSY
SSVTVPRMLFDLLRERRIISLPACITQFYFFLFFALSEMALLAVMSYDRYNAICHPLHYMQI
MSWKVCVQLILIVFVFSAVCSLVHTLFLAKLTFCRSEALQNFFCDLPQLLQISCSDTFINVL
LIFVLGIIFGLGILTVTFFPYIVILTTILKISSKTTRSKAFSTCSSHLTVVSIFYTTICFNYFRSNT
NDHFAEDKVSSVFYTILTPFLNPLIYSLRNQELKISLRKTLQTLQIKNYSD*
>jgi|Xentr4|418627|e_gw1.1115.13.1
MENQTRVYILVLAGLSDLPSLQLPLFLVFLLIYLITLTGNLLILLLIFTDSHLHTPMYFFLGTL
ACLDMGYSSVTVPRMLFDLLR*RNIIIVRDCITQVFFFSFFCTSEVLLLAVMSCDRYIAICRP
LHYMQIMSWNVCIWLVSSICFCGAVHSLLHTVFLTKLTFCRPDALQSFFCDLPQLLEASCS
DTFINVLLIFFLGILLGVGSLGVTFYPYIPIITTVLNIPSKHTRSKAFSTCSSHLTVVFIFYSSLF
FNYFRSYTNDHYTEDKVASVFYAILTPFLNPLIYSLRNQELKSSLTRALQRLKIQTKQKTTI
AEEQ*
>jgi|Xentr4|418656|e_gw1.1115.28.1
MLLLAGLSDLPSLQLPLFLVFLLIYLMTLTGNLLILLLIFTDSHLHTPMYFFLGTLACMDLC
SSSVTAPRMLFDLLRERRIISVPACITQVYFFIFFALSEVSVLAVMSYDRYIAICRPLYYMQI
MSWRVCVDLVLIVFVFSAAYSSVHTLFLTKLTFCRSEALQSFFCDLPQLLQISCSDTFINVLI
ILVLGVMFGLGILALTFFPYIVILTTILKISSKTARSKAFSTCSSHLTVVFIFFTTIFFNYFRSXA
NDHSAEDKVSSVFYTIL
>jgi|Xentr4|418664|e_gw1.1115.68.1
MENQTTVDILVLAGLSDLPSLQLPLFVVFLLIYILTLIGNLLILLLIFTDSHLHTPMYFFLGTL
ACLDTGYSSVTAPRLLYDLFTEQRTISVRACIAQIYFFFLFATSEVSLLAVMSYDRYIAICHP
LNYMQIMRWEVCIKLVSVVLVFAATYSLLQTLLLNKLTFCKSDALQSFFCELPQLLKVSCS
DTLTNTLVINILGILFGLIILGVIFSPYISILHAVLKIQSKNMRSKAFSTCSSHLTAVFIFYSTTFF
NYFRSNGNDSTDHFTADKVASVCYAILTPFLNPLIYSLRNQELKTSLKKTLQRHQMFG*
>jgi|Xentr4|418668|e_gw1.1115.4.1
MENQTTVYVLVLAGLSDLPSLQLPLFLVFLLIYLITLTGNLLILLLIFTDSHLHTPMYFFLGT
LACLDMSYSSVTVPRMLFDLLTRKKVILVRDCITQVFFFFFFCTSEVFLLAVMSCDRYIAIC
RPLHYMQIMTWNVCVQLVSIILFFGLVCSSLNIGFLTKLTFCNSDALQSFFCDLPQLFQVSC
SDTIINVLLMFLLLIVFGMGPFGVTVCPYITIITTVLKIPSKQMKSKAFSTCSSHLTVVFIFYS
TAFFNYFRSFANDQNAEDKVSSVFYAVLTPFLNPLIYSLRNQELKSSLRKAWQTR*
>jgi|Xentr4|418969|e_gw1.1125.15.1
MNESLNDNESSFPLPWIQANDVNASLELSAFFSVFTINPWDIMLCISGTIIACENAIVVAIIFY
TPTLRTPMFVLIGSLATADLLAGFGLILNFVFQYVIQSETISLITVGFLVASFTASVSSLLAITV
DRYLSLYNALTYSSEKTILWIHMMLVVTWGVSLCLGLLPVLGWNCLDDDSSCSIVKPLTK
SNVTLLSTSFFFIFILMLHLYIKICKIVCRHAHQIALQQHFLTASHYVATKKGVSTLAIILGTF
GASWLPFAIYCVVGDHDYPSVYTYATLLPATYNSMINPIIYAYRNQEIQRSMWVLFCGCYQ
SKVSFRSRSPSDV*
>jgi|Xentr4|420259|e_gw1.1162.35.1
MREKNQCIARTEQFLLCTNDGISVFFSSISDLSFLTTLLILGAFITYRDSPIVRANNRSLSFLL
LVSIKLSFLSVFLFLGRPVDITCMLRIITFGITFSIAVSSLLAKTIMVCVAFKATKPGSSWRKW
LGVKLSNSVVLFCSSIQIIICMTWLAISPPFQELDIHTSPGTIIIQCNEGSAIGFYSVIGYMGLL

AAVSFVLAFLAWSLPDSFNEAKYITFSMLLFCSVWITMIPAYLSTKGKNTVCVWRYLPYSP
QARGF*
>jgi|Xentr4|420456|e_gw1.1169.28.1
MQVSEGFVILITALTSCLISLTVLGNIFVILAFIVDKRLRNQTDFVLLNLAVCDFVIGAFICPI
DVTYLLTGKWMFGATVCKLWMTVDVTVYTASAFNVVLISYDRYLSVTKVVLYRSLQNK
RSHTFICIASVWIFSFLLCGPAILSWKNDVIDSSDAESTCIAGFKDVWYINFGSSCVGFALPL
ISISFFNLSIYCSIKKRSRKKRQKSINISQVNLSRDEKIARSLSILVGVFVICWAPYTFLSSIRA
ACSGYCIQSYWYDITIQMLYIHSAINPILYPLCHKSFRKAFIMVLKKSKSCLKIYISD*
>jgi|Xentr4|420465|e_gw1.1169.3.1
MQFSEGFVILTTALTSCLISLTVLGNTFVILAFIIDKRLRNQTDFVLLNLAICDFVIGAFIGPID
LTYLLTGKWMFGATVCKLWITVDYTVYTASVFNIVLISYDRYLSVTKVVLYQSLQNKRSH
TFICIASVWIFSFLLYGPAILSWKNDVIDSSDAKSTCIVGFNDIWYINLGSSCVGFALPLISISF
FNLSIYCSIKKRGRKKRQKSVLQNSKGKENDGNLHIIATNRVLVSAQLHVMENLGTEKRLT
VSLRHCFPYRKPSSFTHNDATLQHRNISQVNLSQDEKIARSLSILVGVFVICWAPYTFLSSIR
AACSGYCIQSYWYDITAWMLYMNSAINPILYPLCHKSFRKAFILVVKKSKSCLKIYILD*
>jgi|Xentr4|420904|e_gw1.1189.25.1
MYTFILAGLSDLPSLQLPLFLVFLLIYLITLTGNLLILLLIFTDSHLHTPMYFFLGTLACLDMS
CSSVTVPRMLFDLLRERRIISVPACITQFYFFLFFFTAEISVLAVMSFDRYIAICRPLHYMQIM
SWNACVHFVSGVLVISAIYSLVHTLFLAKLTFCHSYVLQSFFCDLPQFIESSCSDTFINVLLI
FLLAILFGGGVLGLTFYPYIPIFTTVLKMTSKHTRAKAFSTCSSHLSSLYFTLLLSSTSFAQM
QMII*LKTKWLL*FLQY*LPL*TL*STV*GTRNSNYP*EEHYKDCN
>jgi|Xentr4|420934|e_gw1.1190.34.1
MLIYIIILCLRPSSSEPINAACHLQIIKAAEEYEYIQEGDIMIGGVMASHLSMLNVTFPWDNS
TRFLCTDPNQENIKLMVDFQFAVEQTNKDPARLPNLTLGYHIYDSCGSQLKAVRSVLQILS
GTREPVPNYSCVGKRNIAGFIGDLTSETTVPIAQILTLYGYSQISYGATDPELSDRVTFPYLF
RTIQSDEFNYFVMSKLLSHFGWTWVGIITSDDISGEREHQILAKYLSREGVCIEFTLRIDMY
NCKLIQLQKDKRIIRESSTSVIILCGTVSILLMVRTDHDPLYRAFYEKTLILSPSWGSMEAIL
YYEPELFNCSLVLVPRYHYDLDTPEMRSFLENSHPSNHPEDKLLIDIWMIYFYCLSNDQNK
NDLYKFIYFYNPTLHNCTGQERITDLDYFRGKYNSPRVHLAVDMMSRALHDMNMKLNEK
SKEKRDMNINRHKLRWYLKEVQHRSELGQTFSFDDNREYVTKYLIHSNFMASREKVIKY
SLGEFSPWAPLDQQLNVTPTSVPWKTKDNKIPRAQCSDSCLPGQRKMPIPGAQSCCYVCA
PCANGEISNTTDSVSCIRCSDMEWPNEMKNQCIGRKEEFLSYTEDLISVFISLVSVLFGLITQ
LILGIFIKYRDSPIVRANNRSLSFLLLVSIKLSFLSVFLFLGRPVDITCMLRIITFGITFSIAVSSL
LAKTIMVCVAFKATKPGSSWRKWLGVKLSNSVVLFCSSIQIIICMTWLAISPPFQELDIHTS
PGTIIIQCNEGSAIGFYSVIGYMGLLAAVSFVLAFLARSLPDSFNEAKYITFSMLLFCSVWIT
MIPAYLSTKGKNTVCVEIFAILTSSAGLLACIFLLKCYIILLKPERNTKTNLFYKA*
>jgi|Xentr4|420942|e_gw1.1190.11.1
IPRAQCSDSCLPGQRKMLIPGAQSCCYGCAPCVEGEISNITGREFQRQAESSSDRQRDRQR
VPERCREFQRQAENLISAFISLVTILFFLVTQLILGVFIKYQVTPIVRANNRSLSFLLLVSIKLS
FLSVFLFLGRPVDITCMLRIITFGITFSIAVSSLLAKTIMVCVAFKATKPGSSWRKWLGVKLS
NSVVLFCSSIQIIICMTWLAISPPFQELDIHTSPGTIIIQCNEGSAIGFYSVIGYMGLLAAVSFV
LAFLARSLPDSFNEAKYITFSMLLFCSVWITMIPAYLSTKGKNTVCVEIFAILTSSAGLLACI
FLPKCYIILFRPKRNTKMNLFYKA*
>jgi|Xentr4|421107|e_gw1.1195.35.1

LFLQVSYGATDPVYNDRIQFPSFYRTVPNELSEMDGIVQILKHFGWKWVGLIVSSNNNGG
RALDRITKGIESYGGCVAFSIFLSETSFFGWFYLWLKVKHIVDTINQKDVDVIVLFLTPMHL
NVFKVVFSSRQITRKIWLSSSFFPTVVPHIHGYSRTTLNGTLSLSAQGGEIPGFETFLYRMTP
INYPNDDVITVIWEKLHRCSFTDFLKTNESVPVQKCSGNESLDDEVLSRFGNFDFRVGYQV
YTAVYALAHSLHNLFSARAPAHPQAPAGSLKHRFKPWQLNAFIHNVTYKTPSDDIMSFKA
NGDPPARFDIVKWLFLNDDSTVSRKVGSFDESNEENQLYINSSADLWSPNFPEMPRSLCSE
PCSPGYRKSKIEGEPPCCYDCVQCGDGEMSNTTDAVTCVKCPEDQKSNRQKTDCVPKAL
NYLSYMDTLGASLASAAIILFITASVVLGIFVKYWDTPIVRASNRNLSCLLLISLMLCFLCT
LLFIGRPTKIFCLVRQAAFGIIFIISVSTVLAKTLTVIIAFNATKPGSKLKKYVGTQLAIFIVSA
CSLCVTLICVVVWMASSPPFPEADTYSETDTIILLCNEGSVTFFFCIIGYIGTLALLSFIAAFLA
KDFPDRFNEAKNITFSMLGFCSVWGAFVPAYLSSKGSRMVAVEIFAILSSSAGLLICIFAPKC
YIIFLRPELNTREIVVKRS*
>jgi|Xentr4|421952|e_gw1.1224.2.1
MQVPLGHHSLLAPGYDMLLDNDNSSDSNQTFANKTIPSVNLTRAMLLGFTLGAFISVAIIG
NIMVIISVVTNKQLRIPTNYLIVNLAIADLLLSSTVLPFSATKEIVGYWVFGRILCDVWAAM
DVLCCTASIFSLCTISIDRYIGVRHSLRYPTIVTRKRTILALLGVWMFSTVISIGPLLGWKEPA
PPDTYVCEITTEIFYAIFSSLASFYIPLIVILVMYCRVYVVAKRITKNLEAGVMKERMDSKEL
TLRIHCRNKQDDSPNNSKVQNNQPRSALSLKLLKFSREKKAAKTLGIVVGMFILCWLPFF
TVLPLSKSKCLVNRNSTQQFTACECSEWYQDAGYPQMQLGGDFGLFLFVFKNEHKVGLM
C*
>jgi|Xentr4|421995|e_gw1.1225.7.1
MTSANVSGFIIQGFSDTPELQISLFVLILGIYLIILLGNLIVFLVISCNPHLHTPMYIFLLNLSLL
DISSTSNILPNLLHILLTQQNNISFLGCMTQMHVFVALASSEYFLLTAMAYDRYVAICDPLH
YIARMSRKHCAGLITAAFTGGFGEAVSFIVLIPKLSYCASHLINHIFCDVIALLKLSCSSTFS
AELLIYIEGTLLGFNSFLLTLASYIFIISAILKIQSSEGRQKAFSTCASHLACVITLYGTGICLY
MRPTTSYSLERDKYFSLLYIALGPVLNPLIYTLKNKEFLSSLNKVKQKCLYFYKLKAVNKT
SV*
>jgi|Xentr4|422171|e_gw1.1233.3.1
FSFRPSLQYLRHLIAFIYAIEEINNSAEILPNITLGFQVYDACTSEVIALLSTFSILSERPEPVL
NFICQKRLKTVAFIGHMSSSASLTIAGITQLYRFPQVSYGALDPLFNDRIRFPAMYRTVPNE
YSQFTAIIQLLTHFEWTWVGIITSDDDSNLQASEELSKQMRQNGICVPFLEIIPTVVGSDME
DILRVTRVIKTTSARVIILYCSTSGFMSLLLNLKHFTRITWISSVTLNIATDYPVDRFLLAFSG
FNGSLAISVPSQELPGFQGFFSEATLGKMPGNTFIEDYWFYNSGCPNVSIDNRGNQCQTEK
YFKDILMLENKYRITYTTYMAVYALAQALHEMHSSTSLLTHLPEVSSGKIRMKLNHYLKN
MHSRTSSGTPFHFDENGNIPGMFDIINWNIYPNGTITRTQVGSFLISRVPQLLMDDSNITWN
PHFNQTPFSRCTIPCSFGFRIAHQTGKAPCCFDCIPCSDGEIANVTNMEYCWSCPEDRWSNI
NKDKCIQKTIDFLSYGDILGVTSSCIAFIFVVITAAVLFVFVKFRRSPIVRANDRNLSFILLVSI
MLSFLCSFLFIGYPVELTCMLRQAAFGFIFTVAVSAVLGKTVTVIIAFNATKPNSTFRKWIGT
RVSIGLVLLFSFGELFICIIWLICSPPFVDTDTKTVVGTMIIQCNEGSFTAFYVVISYIGLLALF
SFILAFLVRKLPDRFNDAQYITFSMLVFCSVWISFIPIYLSSKGKYVVAVEIFTILSSTGGLLL
CIFAPKCYIILVKPELNSRKHLLKPKL*
>jgi|Xentr4|422204|e_gw1.1233.4.1
MFNSNIPFLLKVSYSSLDVILSDKLQFPAFYRTVPSDDLQCEAIVQLLKYFYWTWVGLFVS
DDESGLKISQTLQKDFARNGICLAFLEFLPLKDLLDETRGPRIAKSINSTNVIIVYGDRNYM

FSLQLILYLFPVPEKVWVISSQWDVSTGSDLYLLKFQPFNGSLAFTLYKTPIPNFEEFTRGIK
PELYPKDIFIDNVWFGFFQCKWKKNASDPEKCTGKEKLHYADLSYYSYSVYNAVYALAN
ALHSMDIYTIFWHKSFIQAVWHFNLFDLIFFQLHKYLKNLHFINNAGSEVFFDENGNMPIT
FEILNWIVYPNDTLDGIKVGSFNPQHGFHNLTISESLIRWSSAFNQTPRSTCSETCLPGYRKT
PRVGRPTCCYDCISCLEGEISNQTGEK*CVKCPENQWSNTNRDTCVYKVIIYLSYEEPLGTS
LAFLSIIFFLLTCFVSLIFTKYRKTPIVRSNNRDLSYILLFSLKTCFLCNLLFLGHPIRVTCVLR
QTVFGVTFSISLSSILAKTITVIIAFSVTKPASKCRNWVGSRVPNSVIFICSILQVTICACWLG
TSPPFPYYNMEDEVGMIIAQCNEGSVFGFYCVLGFTGILACVCFIIAFLARNLPNSFNEAKF
ITFSMLVFCSVWVSFIPAYMSTKGKYVVAVEIFAILASSMALLGCIFIPKCYIILVKPECNTKV
CLKKV*
>jgi|Xentr4|422210|e_gw1.1233.6.1
ISYSTLDPVLSDKVRFPSFYRTVPSDIMQYLAIVQLVKYFSWSWVGILVSDDESGLKMSQV
LQEQLADYGICVAFLEFIPQSNFIDDDRGPQIARSINSTNVLIVYGDRSYMFSLQLILYMFPV
SDKVWIISYQCDISAGSNLYFLSFAPFNGSLAFVLHKDTIPGFKEFFLGIRPDLYPQDIFISHV
WMYFFDCKWAVNAEDPKKCTGKEKMNDSVPLVLSSYSYNIYNAVYALAHSLHLMYLKE
PKEQMNRHHVKAWKLNKYMKQVRFTNTAGAKVYFDEHGDMPLEFDILNWIVYPNETL
NGIIVGSFAYQNGFFNLKMNESLIRWSTAFNQVKTPQSACSETCTTGYRKTPKAGYPACCY
DCIPCPEGAISNQTDMELCFQCQENQWPNTKGNACVFKEIIYLSYGEPLGMSLALISILFFL
LTSLTLLIFTIYRSTAIVKANNRDLSYLLLLSLKTCFLCNFIFMGHPIHMTCILRQTVFGVTFS
ISLSTILAKTVTVIIAFHATKPRTRLRTFLGPYVAYFIVIFCSLIQVLISASWLGTYPPFPQYNM
VDEVGKIIAECNEGSKLGFYSVLGFMGLLACISFIIAFFARNLPDNFNEAKLITFSMLVFCSV
WVSFIPAYMSTKGKYVVATEIFAIIASSMALLVCIFIPKCYIILLRPERNTKVCIRN*
>jgi|Xentr4|422648|e_gw1.1245.27.1
MEVNNHSMVTEIFLLGFQHLSNFKILVFSVILLIHILTVYENALVIALVTVSRGLQSPMFFFL
QQLSFSDLLLSVLIVPTLLSTVMNEGAKIPLIGCIVQLYFFGATDILQCLLLTVMSYDRYLAI
CNPLRYSSLMSHRVCITLIAMSWLLALSFIPVTMIPLATQEFCNHNSINHFFCDLFPLLELSC
SDTSLAQILAMALTVPVILFPFMFIIGSYICIAHEILKIVSSIGRQKAFSTCSSHLAVVSIFYGT
LIGIYVVPTRNQLQTISKLLSLLYTVVTPFINPMIYSLKSADMKNALKNIIK*
>jgi|Xentr4|422851|e_gw1.1254.34.1
MEWPNEKKIQCIAKLEEFLSYSDDVISIIFSFISVFFLLITGEILGVFIKHCNTPIVRANNRGLS
FLLLVSIKLSFLSVFLFLGRPVDITCMLRIITFGITFSIAVSSLLAKTIMVCVAFKATKPGSSWR
KWLGVKLSNSVVLFCSSIQIIICMTXLAISPPFQELDIHTSPWNHHHSVQ*GLSYWLLLSYW
VYGGFWQLVVLF*HF*LGTYRTVLMRPSTSLSACCSSAVFGSQ*SRPI*APKAKTLCVWRY
LPYSPQARAF*PVYFCPSATQYYSGQV*I*KVICLEANVS
>jgi|Xentr4|423301|e_gw1.1273.11.1
MFRYFCHRLAFIFAIEEINRSRWILPNVTLGYQIFDSCASEVRSVSGTLNIMSGAEHKIPNYN
CQTNSKLVGYLGDLSTRTTYIIAQLAGVFGYPQAGFVFLTLFLYLKKIVLLEQHLEKVIAE
MDGIVQILKHFGWKWVGLIISDDETGYRFRERISKALAGIGGCLAFSSVIRQKTITLDYEMI
TGDITKTTANVIVVFVTTKFASSFVEYFAVKKMPSKVWITTSFFSSLMIYRECELGRTFNGS
LSLSIQEGVIPGFEDFINTFSLNSYPGDYYIEVAWKTFFSCSIPNISNTSKLINCMGNESLHNL
FVYVTTNYRLTYRVYTAVYALALALHNLNSAQPPANHWEGAKQLKRKVKPWQINNYLRS
ATFTMSSGDTIYFDEYGDPPARFDITKCFFLPNHLVDIIKVGYFKASKNEKHLYINNSADLW
SPYFKMAPESLCNAPCAPGYRKSKIEGKPSCCYGCVRCADGEMSNSTGNLIYKYISMSPC
HSLFGRPNKVQKRSIIITTK*SYLPYQSVSSCTGMESIIRKPAIQKAPNYGKPVSHRLNFNQII

QIFKIDFLFLCNKKTVPVLDPN*DIITPYWGRTALLGLFNVTFGIVFTISVSSVLAKTLTVIIA
FNATKPGSKLTLYVGTQLAMLVVSACSLGETVISVVWMASCPPFPDADTSSKMDTIILLCN
EGSELFFFCIIGYIGTLALLSFIAAFLAKDFPDRFNEAKNITFSMLGFCSVWGAFVPAYLSSK
GSRMVAVEIFAILSSSAGLLICIFVPKCYIIFKRPELNKKRKICST*
>jgi|Xentr4|423307|e_gw1.1273.13.1
PESLCNAPCAPGFRKSKIEGRPLCCYDCAQCSDGEMSNTTDAVDCTKCPEDQKSNRQRTD
CVPKAINYLSYMDTLGASLASTAIILFITASVVLGIFVKYWETPIVRANNQHLSCLLLISLM
LCFLCTLLFIGRPTQICCLLRQVTFGIVFTISVSSVLAKTLTVIIAFNATKPGSKLKKYVGTQL
AIILVIVCSLVEIVISAVWLAFNPPFPEADTLSDPDYIILLCNEGSEFFFFCIIGYIGTLALLNLN
YFCSHLFLNFFGSWHDAKNITFSMLGFCSVWGAFVPAYLSTKGSRMVAVEIFAILSSSAGL
LGCIFLPKCFIIFLRPEMNTKKLLS*
>jgi|Xentr4|423309|e_gw1.1273.14.1
PESLCNAPCAPGYRKSKTEGKPSCCYTCVQCANGEMSNSTDAVTCVKCPKDQMSNWQK
TDCVPKALNYLSYTDTLGASLTAIAFILFITASAVQGIFVKYWETPIVRANNQNLSCLLLISL
MLCFLCTLLFIGRPTQICCLLRQVTFGVVFTISVSSVLAKTLTVIIAFNATKPGSKLTMYVGT
QLSITLVLVCSLGETGISAAWMASNPPFLEADTSSETDTIILQCNEGSVTFFFCIIGYIGTLAL
LSFIAAFLAKDFPDRFNEAKNITFSMLGFCSVWGAFVPAYLSSKGSRMVAVEIFAILSSSAG
LLGCIFAPKCYIIFIRPELNKKRKCCST*
>jgi|Xentr4|423315|e_gw1.1274.41.1
MVLTNRTLVTEFVLRGFAGGPVLHIILFVVLLLVFILILAGNIFMIVIIIKDYHLHLPMYMFL
ASLSFTETLVNCMVIPKMLSIFIARQAFISRVGCFTQCFVFYFLTSSCFLFLGLMSIDRYAAIC
HPLRYPRIMTNSVCLRSVCACFIMSFLLIFYPLITVPTLPICNQVLDHFFCEGEAIMRLFCVD
TSIFRLLAVVSSVFILLGSLIITIISYILILSSILKISSVTGRSKTFSTCLSHLTMVSIVFGMSIFNQ
LRPHRDDFIELEKALNVGNIMVAPLLNPFVYTLRNQMVKDSIRNAIRSRKIL*
>jgi|Xentr4|423568|e_gw1.1283.17.1
LSGALDIFSGRNYRISNYACLKNMKLIGVLGDLSSTSSYPIAQLAGLFRYPQASIYFYGATD
PVYNDRIQFPSFYRTVPNELSEMDGIVQILKHFGWKWVGLIVSHSDSGERALDRIREGIES
YGGCVAFSIFLSEARFSSIFIFLVEAESIVNKINEKDVDVIVLFSTPMHVTVFISLFSSRQITRR
IWLSSSFFPTLAPHIHGYSRTTLNGTLSLSAQGGEIPGFETFLYRMTPSNYPNDDTVTEIWE
ALHECSFTGSLETNTSVPVQKCSGNESLDDEALSRFDFRVGYQVYTAVYALAHSLHNLFS
ARAPAHPQAPAGSLKHRFKPWQLNAFINNVTYKTPSGDTMFFKANGDPPAQFDIVKWLFL
NNGSTVSRKVGHFDESNDKNHFYINSSADLLMRWAEEREQTENAVTCVKCPEDQKSNRQ
KTDCVPKALNFLSYMDTLGASLASAAIILFITTSVVMGIFVKYWETPIVRANNQNLSCLLLI
SLMLCFLCTLLFIGRPTQICCLLRQVTFGIVFTISVSSVLAKTLTVIIAFNATKPGSKLKKYV
GTQLAIVLVIICSLGSTGISAVWMASYPPFLEVDMFSEMDTIILLCNEGSVTFFFCIIGYIGTL
ALLSFIAAFLAKDFPDRFNEAKNITFSMLGFCSVWVAFVPAYLSSKGSRMVAVEIFAILSSSA
GLLGCIFAPKCYIIFLRPELNIREIVARKA*
>jgi|Xentr4|423799|e_gw1.1293.12.1
MALRSQENVSGFIIQGFSDTPELHISLFVLFLGIYLIILLGNLIIFLVISCNPHLHTPMYIFLLN
LSLIDISFSSTVLPNLLHILLTQQNNISFLGCMTQMYVFVSLNCSEYFLLTAMAYDRYVAICD
PLHYIARMSRKHCAGLITAAFTGGFVEPIGLVVLMSKLSYCASRLINHFFCDMAPLLKLSC
SSTFSVELLIYIEGTLFTFSSFLLTLTSYIFIISAILKIQSSEGRQKAFSTCASHLACVLTLYGTA
FCLYMRPSTSYSLERDKYFSLLYIVLGPVLNPLIYTLKNREFQSSIKKMRQNFFLF*
>jgi|Xentr4|423807|e_gw1.1293.6.1

MTSESKETVSGFIIQGFSDTPELQISLFVLFLGIYLIILLGNLIIFLVISCNPPLHTPMYIFLLNL
SLIDISSTSNILPNLLHILLTQQNNISFLGCMTQMYAFMSVVGNEFFLLTAMAYDRYVAICDP
LHYIARMSRKHCAGLITAAFTGGFVDPVGIVVLISKLSYCASHLINHLFCDVTPLLKLSCSS
TSSVELLIYIVGTFLIFGSFLPTLTSYIFIISAILKIQSSEGRQKAFSTCASHLACVITLYGTVFC
MYMRPTTSYSLKRDKYFSLLYIVLGPLLNPFIYTLKNREFQSSLNKIKQR*
>jgi|Xentr4|423814|e_gw1.1293.28.1
MTSESKENVSGFIIQGFSDTPELHISLFVLFLGIYLIILLGNLIIFLVISCNPHLHTPMYKFLQT
LSLIDISVTTNIFPNFLHVLLTQQNNISFLGCMTQIYIFMSLTCSEFFLLTAMAYDRYVAICDP
LHYIARMSRKHCAGLITAAFTAGFLEPVSIVVLTSKLSYCDSHLINHFFCDVAPLLKLSCSST
FSVELSIFIEGMLLGFNSFLLILISYIFIISAILKIQSSEGRQKAFSTCASHMTCVITLYGTVLSL
YMRPNTSYSLERDKYFSLLYIVLGPVLNPLIYTLKNREFQSSLNKLKQRYFSFLCLC*
>jgi|Xentr4|423820|e_gw1.1293.31.1
MTSESKINISGFIIQGFSDTPELQISLFVLFLVIYLIILLGNLIIFLVISCNPHLHTPMYIFLLNLS
LIDISFSSTVLPNLLHTQQNNISFLGCMSQLYLFVSFGSSEYLLLTAMAYDRYVAICDPLHYI
ARMSRKHCAGLITAAFTVGFVDPVGIVVLISKLSYCASHLINYFFCDISPLLKLSCSSTFSVE
LFIYIEGTLLGFSSFLLTLISYIFIISAILKIQSSEGRQKAFSTCASHLACVITLYGTALCLYMRP
TTSYSLERDKYFSLLYIALGPVLNPLIYTLKNREFLSSLNKMKQRCFSFLFLC*
>jgi|Xentr4|424116|e_gw1.1310.30.1
MGAAGYGETLELGNQTVVMEFTLLGLSYLFSFGGLAFIAYLVIFLLTLLGNCLVIVAIAGD
AHLHTPMYFFLANLSLLEMFYVSITVPGTLVGIALQSSTISFAGCMAQAHLFTLCATVECT
LLAAMALDRYVAICQPLHYTLIISQRVCVLLGAFAWLCGVLNATVHTSLILGLRFCGAKSI
NHRYCEVRPLIRLSCSDTRTSDICATLSALVFGFGCLGLILTSYFFIGMAIVRTPCPTGRLKAF
STCTSHVMVVVLYYGALIFMYLLPSNAPPQALNMAASMVYSTGIPLLNPIIYSLRNQQVK
GALRKRMSWKYPLWSGRKS*
>jgi|Xentr4|424131|e_gw1.1310.6.1
LGFSAFTDIRLTFFLLFLCVYVAILAGNGAVVSIISASSSLHTPMYLFIWNLSALEMVYSSVT
VPKILANLLSEDKRVSFLGCAVQMYLFLALGTAECFLLAFMAYDRYSAICRPLHYTMVMS
RRRCLQFSASAWVSGLLLSLEQTTFIFTRPFCGPNVIDHFFCDIPPVLKLACTDTSHNELSIA
GVCVAILLVPFLLILLSYTRILSTLLRMHSGESRGKAYSTCVCHLTSVTLFYGSASIMYLRPR
SLYSVQMDRFLALFYSVGTPMLNPLIYSLRNQEVQTSAMKLMKAWASPNNR*
>jgi|Xentr4|424219|e_gw1.1315.6.1
MTFYYDFISTLILLYSKSFRYYRHLLVFKFAIEEINRSSWILPNVTLGYQIFDSCAAPPKALS
GALDIISGANYKIPNYACLKNMKLIGVLGDLSSTSSYPIAQLAGLFRFPQASIYFYGATDPV
YNDRIQFPSFYRTVPNELSEMDGIVQILKHFGWKWVGLIVSNTNGRRVLDRIRKGIESYGG
CVASSIFLSETIFQGFLLLLFNINRIVDTINQKGVDVIVLFLTPMHLKAFIQVFSSRQVTRKIW
LSSSFFPTVAPHIYGYSRTTLNGTLSLSAQGGEIPGFETFLYRMTPRNYPNDVTVTEIWETL
HECSFTGSLKTNTSVPVQKCSGNESLDDEALSRFGNFDFRVGYQVYTAVYALAHSLHNLF
SARAPAHPQAPAGSLKHRFKPWQLNAFFHNVTYKTPSGDIMFFKANGDPPAHFDIVKWLF
LNNGSTVSRKVGSFDESNEENQLYINSSADWWSPYFPEMPRSLCSEPCSPGYRKSKIEGEP
PCCYDCVQCGDGEMSNTTDAVTCVKCPEDQKSNRQKTDCVPKALNYLSYMDTLGASLA
STAIILFITTSVVMGIFVKYWDTPIVRASNQNLSCLLLISLMLCFLCTLLFIGRPTKIICLVRQ
ATFGIIFMISVSTVLAKTLTVIIAFNATKPGSKLKKYVGTQLSILVISASFLGVTLICIVWMAS
SPPFPEADNYSETDTIILLCNEGSVIFFFCIIGYIGTLALLSFIAAFLAKDFPDQFNEAKNITFS
MLGFCSVWGAFVPAYLSSKGSRMVAVEIFAILSSSAGLLACIFAPKCYIIFFQPEMNIREIVV

RKTSPCNISH*
>jgi|Xentr4|424234|e_gw1.1315.5.1
MARKNESYVISILFSLYIVLFCSIISHSPLFRYFRHRLAFIFAIEEINRSRWILPNVTLGYQIFDS
CASEVRSVSGTLNIMSGAEHKIPNYNCQTNSKLVGYLGDLSTRTTYTIAQLAGVFGYPQA
VGSALPLSDTGIESIGYGSMDPALNDRTQFPSFYRTIPNEEAEMDGIVQILKHFGWKWVGL
IISNDDTGYRFRERISKALAGIGGCLAFSSVIRQKTITLDYERITGDITKTTANVIVIFVTTKF
ASSFVEYFAVKKMPSKV*FTTSFFASLMTYRECELGRTFNGSLSLSIQEGEIPGFEDFINTFS
LNSYPGDYYIEIAWETFFSCSIPNTSNTSKLENSMGNESLRNLFVYVTTNYRLTYRVYTAVY
ALARALHNLYSAQPPANHWGRLETLKRRVKPWQINNYLRSAAFTMSSGDTLYFDEYGDP
PARFDITKCFFLPNHLVDIIKVGYFEASKNEKYLYINNSADLWSPYFKMAPESLCNAPCAPG
YRKSKIEGKPFCCYCCVRCADGEMSNSTDAVTCVKCPEDQKSNRQKTDCVPKALNYLSY
MDTLGASLASAAIILFITTSAVMGIFVKYWETPIVRANNQNLSCLLLISLMLCFLCTLLFIGR
PTQICCLLRQVTFGIVFTISVSSVLAKTLTVIIAFNATKPGSKLKKYVGTQLSILVVSACSLG
ETVISVVWMASSPPFPDADTSSKMDTIVLMCNEGSDTFFFCIIGYIGTLALLSFIAAFLAKD
FPDRFNEAKNITFSMLGFCSVWGAFVPAYLSSKGSRMVAVEIFAILSSSAGLLICIFVPKCYII
FKRPELNKMRKICST*
>jgi|Xentr4|424755|e_gw1.1343.3.1
MENQTIVYAFILAGLTDLPSLQLPLFLVFLLIYLITLTGNLLILLLIFTDSHLHTPMYFFLGTL
ACLDMSYSSVTVPRMLFDLLRELRMISVPACITQFYFFSFFMASEISVLAVMSYDRYIAICR
PLHYMQIMSWNVCVQFVSGVLFFSTIYSLVHTLFLTKLTFCLPNVLQSFFCDLPQLLEASCS
DTFINVLLIFVIGMPHGAGIFGLTFYPYIPIITTVLKMTSKHSRSKAFSTCSSHLTVISICYTTL
LFFNFFRSNANDNLVEDKVASVFFAILTPFLNPVIYSLRNQELKLSLRRTLQDYSSFRKIRVT
GFKKMCFNSSLILVRVLMCYPSSPL*
>jgi|Xentr4|424769|e_gw1.1343.13.1
MENQTTVYNFILAGLSDLPSLQLPLFLVFLLIYLITLTGNLLILLLIFTDSHLHTPMYFFLGTL
ACLDMSCSSVTAPRMLFDLLRELRLISVPACITQTSFFIFFIASETSLLAVMSYDRYVAICRPL
HYMQIMSWNVCVQLVSCVILFSTVDSMVHTIFLTKLTFCHPSVLQSFFCDLPQLLEASCSD
TFINVLIIFVFGILFGVGILVLTFYPYIPIITTVLKMTSKHTRSKAFSTCSSHLTVVSICYTTFYL
NYFRSNTNDHLVEGKVASVFFAILTPSLNPVIYSLRNQELKLSLRRTLQRLQ*
>jgi|Xentr4|424772|e_gw1.1343.23.1
MENQTTVYTLVLAGLSDLPSLQLPLFLVFLLIYLITLTGNLLILLLIFTDSHLHTPMYFFLGT
LACLDMSYSSVTVPRMLFDLLRERKIISVPACITQMSFFILFIASETSVLAVMSYDRYIAICR
PLHYMQIMTWNVCVQFVSGVLVCSALYSLLHTFFLTKLTFCVPNILQSFFCDLPKLLEASC
SDTFINVLLIFLLGLLLGVIILGGTFYPYIPIITTVLKMSSKHTRAKAFSTCSSHLTVVFIFYTT
SFFNYFRSNANVHLVGDKVASVVFAILTPSLNPLIYSLRNQELKLSLRRALQRLQ*
>jgi|Xentr4|425054|e_gw1.1355.5.1
MLVLIGLSDLPSLQLPLFLVFLLIYLITLTGNLLILLLIFTDSHLHTPMYFFLGTLACLDMGY
SSVTVPRMLFDLLTGRRVISVPACITQIYFFLFFAVSEMSVLAVMSYDRFVAICRPLHYMQI
MSWNVCVQFVLGVLIFGAVHSLVHTLNLTKLTFCRADALQSFFCDLPQLLQVSCSDTFINV
LLIFVFGTSFGVAILLVTIYPYITIITTVLKIPSKTMRSKAFSTCSSHLTVVFLFYITFMFNHFR
PNAKYHFTEDKVASVFYATLIPFLNPLIYSLRNQELKTSLRRSLHRL*
>jgi|Xentr4|425057|e_gw1.1355.17.1
MENQTIVYMLVLAGLSDLPSLQIPLFLVFLLIYILTLTGNLLILLLIFTDSHLHTPMYFFLGTL
ACLDMSYSSVTAPRMLFDLLIVRRDITVPACITQIYFFFFFAISEAFVLAVMSYDRYVAICRP

LHYMLLMSWNVCVQLVSGVLVFGAVHSLVHTLFLTKLTFCRPDALQSFFCDLPQLLQISCS
DTFINKLLLFFSGIMFGFVILVVTFYPYVTIINTVLKIPSRNMRAKAFSTCSSHLTVVFLFYG
SFFFNYLRPNAKYHFTEDKVASVFYAILTPFLNPLIYSLRNQELKASLRRSSHRCIGG*
>jgi|Xentr4|425059|e_gw1.1355.18.1
MENQTTVLFLTRLSDLPNLQVPLFLVFLLIYLFTLTINLLILLLIFTDSHLHTPMYFFLGTLAC
LDMSYSSVTVPRMLYDLLTGRRDISLPACITQIYFLMFFAVSEMSVLAVMSYDRYIAICRPL
HYMQIMNWNVCVQLVSIMLVLSAIYTSVHTLFLTKLSFCRPDALQSFFCDLPQLLQVSCSD
TFINVLIVLLLGILFGGVLLGVTFYPYISIIMTVLKIPSINMRSKAFSTCSSHLTVVFIFYSTIFF
NYFSPNSKYHVIEDKVASVVYAILTPLLNPVIYSLRNQELKTSLRRSLHRL*
>jgi|Xentr4|425062|e_gw1.1355.9.1
MLVLAGLSDLPSLQLPLFLVFLLIYLLTLTGNLLILLLIFTDSHLHTPMYFFLGTLACLDMG
YSSVTVPRMLFDLLTRKRIISVPACITQIYFFIYFATSEIIVLAVMSYDRYIAICRPLHYMQIMS
WKTCVQVVLGVLVLDAVYSLVHTLFLNNLIFCKSEALQSFFCELPQLLQISCSDILINVLLIF
LLGLLFAVGPLGVTLYPYITIIRTVLKIPSKNMRSKAFSTCSSHLTVVFILYSTITFNYFLPNA
KDHYTKDKVTSVFYAILTPFLNPLIYSLKNQEIKTSLRRTLYKLQILRG*
>jgi|Xentr4|425066|e_gw1.1355.1.1
MENQTIKDTLVLTGLSDLPSLQVPLFLLFLFIYLFTLTVNLLIFLLIFTDSHLHTPMYFFLGTL
ACLDMSYSSVTVPRIDLLTQKRVISVRDCITQFYFFLFFAISEMSVLAAMSYDRYIAICRPLH
YMQIMNQKAFCVQFVSGMLVLSAIYPLVHTVFLTKLTFCRPDVLQSFFCDLPQLLQVSCSD
TFINVLIIYLLGTLFGAVLLGVIFYPYISIIMTVLKIPSKQMRSKAFSTCSSHLTVVFIFYTAIIF
NYFSPNAKYHVIEDKVSSVFYAIITPCLNPVIYSLRNQELKTSLRRALHRL*
>jgi|Xentr4|425145|e_gw1.1357.26.1
MLLCLTALCVGLCTPGVQPLNPACRLQIIEAIEEHEYIQEGDIMLGGVLTAHLLVITKKIPGL
HILICTDPTQQNSKHFLDFRFSIEQLNTNPSLLANLTLGYHIYDSCGDPRKAVRSVLQILSGT
REPVPNYSCVGKRNIAGFIGDLTSETTVPIAQILSIYGYSQISYGATDPSLSDRTTFPYFFRTV
QGDSGQYYALSKLLRHFSWTWIGIIRSDDDSGEKEVELLRRFLHGESICIEFTVKIGSNIGD
DPQWLWGQNIIQKSSSRVIVLCGTVSLTLVGKLAKFARWIREKTFVLPSKWGANDQILSYA
QHIFTGSLVLTQIYPYKLDTAEMRDFLNSLTPSNYPDDKLIEDIWTIHLLCLSNNPNKNSLYE
HIYLRTLHNCTGQERITDLGNFNDHGNSGRVHLAVNMMSLALHDMNVSLGDQSPESSKV
AVKYQHQLHRHLQKVQYRTPKGRAVSFNENGEIITRFGIYNSVTEADGELYLYLVGIFIPWA
TAGKQLILHSNIIKWKGTDSDIPRAQCSDNCLPGFRKAQRVQTQLCCYDCVRCSEGEISNT
TDSANCARCPDLQWPNENKTQCITSTETFLSYTSDGISVVFFSISLLFFLKTVLIFGVFIIYRE
TPIVRANNRSLSFLLLVSIKLSFLSVFLFLGRPVDITCMLRIITFGITFSIAVSSLLAKTIMVCV
AFKATKPGSSWRKWLGVKLSNSVVLFCSSIQIIICTTWLAISPPFQELDIHTSPGTIIIQCNEG
SAIGFYSVIGYMGLLAAVTIGFYSVIGYMGLLAAVSFVLAFLARSLPDSFNEAKYITFSMLL
FCSVWITMIPAYLSTKGKNTVCVEIFAILTSSAGLLACIFLPKCYIILLIPENNTKSNLLGGKV
LIIS*
>jgi|Xentr4|425192|e_gw1.1358.37.1
MGTPGAPQPSGSTCSLNSRRKFFEPRYFRDGDYIIGGIVTVRHYYFEAVLLQKKSSLITCFM
PLMEYYRHVLAFIFAVDEINRNPAILPNVTLGYHIFDSCRNVNNAMQDVFSILSGPGETVPN
YSCREQHRVAGFIGDLFTETSLPIAQLLRPYGYSQISYGATDHLLSDKRFYPAFFRTLPNDQ
TQYQAIAKLLRHLGWNWVGIIRSDDDIGEQLSRDLSKVLGEYGICIEYTLSIPEEQNPRRDE
LYARLQGKLRQTTSRGAVMCSRVSLFYMNSLTREEGSLLEKILIVPVNWNYRPYSTARSLR
TLYNGTLFFKRPSKNLPRLHRLLENLNRTKGDKMLEDIFATYFNCLTSVPLRNTMIQTIYPL

VLGNCSGKEKITQLKRKGYNTVTFRTTYHVYKAVYAMAYALHHMLLPRGPPHLHTGAHI
HRERFRHFLTKVHFKDPTGEEIYFKENGEMSSVYHLLQQINWVNGTTELRHVGTFNSSAP
EEEQLVIAHRGFMWHLDGSGIPVSRCTPRCPPGQRKLPRRGQQHACCYDCVQCSEGMISN
DTDREACQPCPEDKWPNQKKDQCLPRSSEFLSYQSDWKTLLFSIVSILLSGVTAIIVGIFVA
FRHSPIVKANNQRLSFLLLGSIMMSFLCVFLFLGRPLHVTCMLRQTCMGIIFSVVVSSVLA
KTIMVCIVFKASKPGNYWKKCVGAKIPNGVVVACSLLQVSISIAWLSISPPFMELNRSFPG
KILIQCNEGSPVAFYAVLGYLGLLAAVSFIVAFLARKLPDSFNEAKYITFSMLVFCSVWVSFI
PAHLSVTGKNTVVVEIFAIMASSFGVLASIFFPKCYIILIKPEQNTKRNLLGRSPNRR*
>jgi|Xentr4|426023|e_gw1.1400.39.1
MENLSWFGPPNSSCHPAGNFCHANTSHHGSSEMSPDFYIVLPIIYAIICVVGLTGNTAVIYVI
LKAPKMKTVTNLFILNLAIADDLFTLVLPINIAEILLHYWPFGVVLCKVILSIDLYNIFSSIYF
LTVMSIDRYLVVLATVRSKRMPYRTYRAAKVISLLVWLLVIIIVLPFTIFAGVYMDDMDFK
SCGLNFPKPEKLWFKASRIYTLLLGFAIPVSTICVLYMVMLYKLRNMRLNSNAKALDKAK
KRVTVMVFVVVAVCLFCWTPFHLATIVSLTTDLQETSLVIGISYFITSLSYANSCLNPFLYAF
LDDSFRKSFRKLLECKPA*
>jgi|Xentr4|426284|e_gw1.1412.7.1
MVLTGLSDLPSLQLPLFLVFLLIYLITLTGDLLILLLIFTDSHLHTPMYFFLGTLACLDMSCS
SVTVPRMLFDLLNQKRVISIRACLMQVFFFMFLGASEVSVLAVMSCDRYIAICRPLHYMQI
MSWRVCVQLVSIILGFGLVCSVLNISFLTKLTFCRSDVLQSFFCDLPQLLEASCSDTFINVLL
MFLLTILFGVGILGGTFYPYIPIITTVLKMTSKHTRAKAFSTCSSHLTVVFIFYSTGLFNYFHS
YSNDHYSGDKVASVFYTILTPFVNPVIYSLRNQELKSSLKKSFARSGNPNGTKNRFID*
>jgi|Xentr4|427000|e_gw1.1457.1.1
AFMYAIEEINNSTELLPNITLGYCIYDACTSEKIALMSTFSLLSDDENPLNYNCQHNQKLVA
FVGHLLSSTTYTIAEITQLYGYPQISYGALDPVFNDRINFPSVYRTVPNEYSQFRVIIKLLKH
FGWTWVGIIASDDKSNYQASEELRNEMEENGICVDFLKSIANSPPFSDASAVDAIKVIKHS
SVRVIILYFRISSLTNLLSYRTSELIPERVFICSVALDIVIENDFTEFYYLMNGSLLIALPRGDIP
GLNDFLSYKLWTDLSENIFLQMVFDLIAECSDVVLDGVKNVTCLKKNRIKEYLLQEETITH
RIKHTIYMAVYALAHALDNMQLPGELWSSKEMSKIRFKVCMNLNYYLKNLHLKTPSGEE
FFFSKEGNIPGKFDILNWIINENGTINKIHVGKFLPNTLVLSHFSESTPFYLMFKTSQCSEMC
SSGQRRAHQNGRPPCCFDCVSCSEGEISNSAEGQRDPSEDFFWGDLSHCNWKTNILRLFRI
FCNFFVVATTCANFRNFFIAITNFVNSRNFFIAIMTFVICRNFFIAITTFSTCVFCSALRRLYLI
CIDYIMTVEFALIISLDITSTRIAASKMTHSGLILWATILSCLFICCIIILYPPHVESDNKTIPGK
NILQCNDGSIISFYLAVSYIGVLSLISFAVAFIARKLPDRFNEAQHITFSMLVFCSVWASFIPTY
LSTKGKHMVAVEIFAIQASAAGLLLCIFTPKCYIILLKPELNVKGKPTAKIQSHTTNQTLNN
N*
>jgi|Xentr4|427508|e_gw1.1491.16.1
EFVLICFPEIHSWHISGLLMFFLSVALVSNLTLLLVIGTEPRLHHPMYYFLAMLSFVDVLLCT
VATPKVLANLWNEDKTISSAACFTQMFFINVWSAMESSIFLVMAYDRYVAICHPLHYPTIIT
NKLVAKASAFILIRNLVLSLPLPLLAARLDYCSLREIPHCFCENMSVEKLSCSNYSASSIYGL
VVFFVVGGADLLFIIFSYLVVLRVVVASRSFSAAFKAFRTCSSHLILICFFYITIGITMVSNRA
DKQIPRHVHVLLALLHHLLPPALNPLVYGAMTEEIRHAIRRLLERIKVHPYLGA*
>jgi|Xentr4|427624|e_gw1.1499.8.1
MALGSEENVSGFIIQGFSDTPELHISLFVLILGIYLIILLGNLIIFLVISCNPHLHTPMYIFLLNL
SLIDISFPSNVLPNLLHILLTQQNNISFLGCMTQLYVFASLADSEYLLLTAMAYDRYVAICDP

LHYIARMSRKHCAGLITAAFTVGVGESTIIVTLIFKLTYCASHLIKHFFCDAPPLLKLSCSST
FSAELVIYIEGTLLVFSSFLLTLTSYIFIISAILKIQSSEGRQKAFSTCASHLACVITLYGTVICL
YMRPTTSYSVKRDKYFSLLYIVLGPVLNPLIYTLKNKEFQCSLNKMRERFESCIFR*
>jgi|Xentr4|427631|e_gw1.1499.2.1
MALGSEENVSGFIIQGFSDTPELHISLFVLFLGIYLIILLGNLIIFLVISCNPHLHTPMYIFLQN
LSLIDISFPSTVLPNLLLILLTQQNNISFLGCMTQMYVFGSLAGSENFLLTAMAYDRYVAICD
PLHYIARMSRKHCAGLITAAFTVGFGETVGLIVLISKLSYCASHLINHFFCDVTALLKLSCS
STFSVELFIYFEGTLLNFNSFLLTLTSYIFIISAILKIQSSEGRQKAFSTCASHLACVITLYGTII
CLYMRPTTSYSIKRDKYFSLLYIVLGPVLNPLIYTLKNKEFQCSLNKTRQRFVSCFFR*
>jgi|Xentr4|427977|e_gw1.1525.2.1
MQIYRLGNDSGINLTLDTAGSTTQFSDAVNILIIALISLLILLTVGGNTLVILAFFVEKRLRNQ
SNFFLLNLSIADFILGTFAIPLYVPYLLTGKWLLGKFLCKLWLIVDYTMCTASAFNVALISW
DRFLSVTQAVLYRSQQNRPCRTVIKMAAVWILSFLLYGPAIIFWDFVPSTEEIPENICVAGFY
YTWYYLLTASAFDFVLPLISISFFNLSIYCNIKKRSRRKMQNSISLPPQKSRKEAKLCTIATNI
TLQSPQIDIQKKSFRRRINISCNQCFGIKSSSHNNKRSDRENNQVSDLSRDKKVAKSLSVLV
CIFAICWAPYSFLMSIRAACHGYCIHIYWYDITFWLLWTNSAINPIIYPLCHKGFQKAFLNIV
KYVCMKKTNDSA*
>jgi|Xentr4|427980|e_gw1.1525.6.1
MQFSEGIVILITAVASCLISLTVLGNTFVILAFIVDKRLRNQTDFVLLNLAICDFVIGALTYPM
YVQYYLTGKWMFGRFLCRLWLTVDYTVCTASAFNVVLISYDRFLAVTKAVLYRSLQNKRI
NIVVSIASVWIFPFLLYGPAILSWKNDVIDSSDAESTCVPGFIGIWYFNFGTSCVDFALPLISI
SFFNLSIYCSIKKRGRKKRQKSVLQNSKENVLVSAQLHVMENLGTEKRLAVSLMHCFPYR
KPSSFTHNEATLQHRNISQVKLSRDEKIARSLSILVGAFVICWAPYTFLASIRAACSGYCFDS
YWYDITTWMLYMNSAINPILYPLCHRSFRKAFILIVKKIKKFFNI*
>jgi|Xentr4|428257|e_gw1.1551.17.1
MENSSYSQPSMLTLNFGQLMEIKYFYSTLVLLCFMMIVVSNSAVISAISMHRNLQEPMFIFI
AFLCINGLYGSVIFFPFLFVNLLSKTQVVSYVGCLIQVFCNHTYIGCEMTILAVMALDRYVC
ICNPLRYSSIMSLATVFKLIGAAWLYVIVLIAILVLLTIRLPLCGSVIQKIYCDNISVVKLSCTD
TTASNIFGLLITAAVVGLMPVLTVMSYAQILRVCMKASKAFRAKALQTCTPHLVTLTYFVA
DVLFEILLPRFPSTTLPYELRVLMSVQAFIIAPILHPLIYGWKLREIRLRVLQMFGAKPIADL
HNNL*
>jgi|Xentr4|428275|e_gw1.1551.13.1
MANATYSQPTVLALSFGELTAVKYLYGAIVFLIFLMIVASSSAVIGTIILHRSLHEPMYMFIA
ALCMNGLYGGICFFPALFVNLLSQVQTVSYIGCLIQVFGIHSYYGCEITVLAVMAFDRYMC
ICNPLRYHSLMTSGTVLKLIGAAWLYIIVLVTIHVILTIRLPLCGSVIQKIYCDNWSVVRLSCI
DTTLNNVFGLLMSSITGLLPGGVLISYVKILRVCMKSSKDVRAKALQTCTPHIVSLLYFVV
DILCEVVLLRFPFNSIPYELRIIISVQAFVFAPLLNPLMYGLKLRKIRVKIGQIFCPKNKTEGH
GKM*
>jgi|Xentr4|428280|e_gw1.1551.3.1
MEHACNTSNSFVLLGILEIEGSRFFYCVLCSLTYILTVFLSLTIAFVIWTEQSLHEPMYILIGS
LVLNGIFGSSAFVPKVMIDLLTRSNCISRVGCFAQAFCIGVFPISEISIFTMMAYDTYLAVCH
PLRYSTLMTKETALYLLVGFWIMNFFSVLAAILLSARLPLCGTQISGLLCDNTGLVFLSCVD
DSINYFYGTVLFSAYLSVCMLLISYSYLQIALVCHRLTSETYKRSVHTLVTHILNFSVFLVG
VLFVFIRYRVGRKNSPLLSVLLALTTLVLPPVLNPLIYGVRTKKLNAKVLNRLKKLNKGTF

TKF*
>jgi|Xentr4|428443|e_gw1.1566.2.1
IPRAQCSDSCLPGYRKMPIPGAQSCCYVCAPCAEGEISNKTENPLCLSDIVAIFALSLVEKY
QRTGIKVVGQNPSLTPLGDLVACFTLCVLWVVSKVVEKKSLQMVQAPNTGKMYYILEKL
VSFFLSVFLFLGRPVDITCMLRIITFGITFSIAVSSLLAKTIMVCVAFKATKPGSSWRKWLGV
KLSNSVVLFCSSIQIIICMTWLAISPPFQELDIHTSPGTIIIQCNEGSAIGFYSVIGYMGLLAAV
SFVLAFLARSLPDSFNEAKYITFSMLLFCSVWITMIPAYLSTKGKNTVCVEIFAILTSSAGLL
ACIFLPKCYIILLIPENNTKSNLLGGKC*
>jgi|Xentr4|429309|e_gw1.1637.10.1
MNNQSVVTEIFLFGFQHLNNFKIPFFSLILLIHILTVYENALVIALVTVSRGLQSPMFFFLQQ
LSFSDLLESLVIVPTLLSTVMNEGAKIPLIGCFVQLYFLGVSEALQCLLLSVMSYDRYLAIC
NPLRYSSLMSHRVCVKLIAISWLLALSFPPFTVIAAATQKFCNRNTINHFFCDFFPLLELSCS
DTSLARILAITISTPVIVFPFILIIISYICIAHEILKIVSSIGRQKAFSTCSSHLAVVSLFYGTLIGI
YVVPTRNQSQTIRKLVSLLYTVVTPFINPMIYSLKSADMKNAIKNIIQ*
>jgi|Xentr4|429519|e_gw1.1663.11.1
MEVLIFLCVGPCRSGVQPLNPACNLQIIKAVKEYEYVQEGDIMIGGVMASHFYMANVIFPP
YKSNRFLCNSPNQQAYRYLVDFRYAIEQTNKDPARLPNLTLGYHIYDSCGDPRKAVRSVLQ
ILSGTREPVPNYSCVGKRNIAGFIGDLTSETTVPIAQILTLYGYSQISYGATDPSLRDRVAFPY
FFRTVQSDEANYFAISKLLRHFGWTWVGIITSDDISGEREHHSLAKYLSREGICIEFTIKICT
NEEHTKKLNQYEIIIQESSTSVIILSGTVSTMFVIELPVTLNNVLQEKTLILSSNWGNNDIVV
GYAVEIFNYSLVFVPRYHYDLGTPEMSRFLEDLHPSKFLDDELIEDIFMMFHLCLSKDQNK
NELYKYIYINTLRNCTGQERITDIYHFRGETNSPRVHLAVNIMSQALHEMLFIKESIQYPYK
YQLHCYLKKHQYSPQTGPTFLFDEHGEYISGLRIYNYIISAEGYLNKMLFGEFSPWAPPDQ
QLNVTSSLIQWKTNNNEIPRAQCSDNCPPGYRKAPKPGAQSCCYDCVPCSEGEISNTTDSE
SCIRCPDMEWPNEKMIQCIARNEEFLSYSDNVVSAIFSFISVLFFLITEVILGFFITYRDRPIV
RANNRSLSFLLLVSIKLSFLSVFLFLGRPVDITCMLRIITFGITFSIAVSSLLAKTIMVCVAFKA
TKPGSSWRKWLGVKLSNSVVLFCSSIQIIICMTWLAISPPFQELDIHTSPGTIIIQCNEGSAIG
FYSVIGYMGLLAAVSFVLAFLARSLPDSFNEAKYITFSMLLFCSVWITMIPAYLSTKGKNT
VCVEIFAILTSSAGLLASIFLPKCYIILLRP
>jgi|Xentr4|429724|e_gw1.1684.2.1
MGGTNQTDIQQILLSGFQVPHSFKIPFFLLVLILYNATLTANITIVGLVSSSPSLHHPMFFFLS
HLSLSDIMLTTDIAPLMLHGILKGGVTTWTSACITQLLFHGIAVTSECLILTVMSYDRYLAIC
NPFHYVSIMCSNLQLSLVSLCWFVSFAMSLIPVVLISKMEFCGSHTINHFFCDFAPLLELSCS
DTSLVELIDMVLAIPLSVFPVLFITITYMCIFNAILKIPTTTGRHKAFSTCSSHLTTVTIFFGILV
AVYIVPSNDISMSKIIISLIYTVVIPLFNPLIYSLRNQEVRSALQKCLQRMVSYRGVRMRRLV
LLRFFC*
>jgi|Xentr4|429822|e_gw1.1701.10.1
MLMYLLFTCIGPCQSKVNPISPACHLNIPEMLEDFEYFQDGDIIIGGLLTVNEYAARAINLQS
RYYRQLVEFRLAIEEINKNPSLLPNVTLGYHIYDSCGHEQKVMKSILQILSNTKEPVPNYSC
GRKRNIAGFIGDLKSETTVISAQILSLFGFSQISYGASDPSLSDRVAFPYFFRTKQSFRGTSV
VLIKLLKHFGWTWVGIIRLDDNSGETELQVLTDYLSRAGICVEFTIKMSHIDDTYNRQMDI
DGMKKIITNSTTNIIVFCGRLSKISASKLTHLNAELNNKTLILSPSVALLEHLQMYIMPSFDG
SLMFEQYPVYPRDTHEIIEFINSIHPSKDPEDKLLEDILLIRFKCSSKNEYKNLLYEYVYITFN
AECTDKDVAEALVILNYTLLNVHSPNVHLAVNIMSQAIHEMHTSLREQSPERDREAHRYQ

YQLHRYLKETKHQTQSEGTVSFDGNGEMNTGYIIYDFMFSREGLNLHQFGKFVPLSPSDS
QLIVYLQTKPWKTKNSTIPRAQCADSCQPGYRKALKPGLQPCCYECIHCSEGEISNRTDSE
NCFRCPDLEWPNEKRNQCIAKTEEFLSFTDGTISTSLLSITVLFSLTTLLMLRIFISYRDSPIV
RANNRSLSFLLLVSIKLSFLSVFLFLGRPVDITCMLRIITFGIPFSIAVSSLLAKTIMVCVAFKA
TKPGSSWRKWLGVKLSNSVVLFCSSIQIIICVTWLAISPPFQELDIHTSPGTIIIQCNEGSAIG
FYSVIGYMGLLAAVSFVLAFLARSLPDSFNEAKYITFSMLLFCSVWITMILAYLSTKGKNT
VCVEIFAILTSSAGLLACIFLPKCYIIMFRPEINQKSCLLGNKAF*
>jgi|Xentr4|430803|e_gw1.1852.1.1
MQINTYVRNVTFTTPSGDTIFFNDKGDPPAQFDVMKLSLLPDGRVAKEKVGSFHTRASYIF
TPNILGTSQYFIHLFTLQIQMPQSLCNEPCAPGYRKAKIEGKPSCCYDCAKCADGEMSNTT
ATDIQTQLSYCPLSNKQRTGCVPKEINYLSYTDTLGASLTSIALVLFIAASVVLGIFVRYWE
TPIVRANNQHLSFLLLISLMLCFLCTLLFIGRPTQICCLLRQVTFGIVFTISVSSVLAKTLTVII
AFNATKPGSKLKKYVGTQLATILVIVCCLGEMMISAVWMASNPPFLDADTVTDINTVFLM
CNEGSVLFFFSVIGYMTALALFSFIAAFLAKDFPDRFNEAKNITFSMLGFCSVWGAFVPAY
LSSKGSRMVAVELFAILSSSAGLLACIFVPKCYIIFFKPEQNRRKM*
>jgi|Xentr4|430916|e_gw1.1879.6.1
ESKENVSGFTIQGFSDTPELQISLFVLFLGIYLIILLGNLIIFLVISCNPHLHTPMYIFLLNLSLI
DISSTSNILPNLLHILLTQQNNISFLGCMTQMHVYGFMAASEYFLLTAMAYDRYVAICDPL
HYIARMSRKHCAGLITAAFTGGFVVPVGFIVLVSKLSYCASHLINHFFCDISPLLKLSCSSTF
SVELSIYFEGTLLTFNSFLLTLTSYIFIISAILKIQSSEGRQKAFSTCASHLACVITLYGTVICLY
MRPTTSYSLERDKYFSLLYIALGPVLNPLIYTLKNREFQSSLNKMWQRCSAFSILEK*
>jgi|Xentr4|431129|e_gw1.1930.3.1
MSLLCLNRTGSYSIYPIYILGVIIQGFSDTPELQIPLFVLFLVIYLIILLGNLIIFLVISFNPHLHT
PMYIFLQNLSLIDISFPSNILPNLLHILLTQQNNISFLGCMTQMYVFGFLAASEYFLLTAMAY
DRYVAICDPLHYIARMSRKHCAGLITAAFTVGFLDPVAHLVLIPKLSYCASHLINHFFCDITP
LLKLSCSSTFSVELLNYLVGTLLGFNSFLLTLISYIFIISAILKIQSSEGRQKAFSTCASHLACV
ITLYGTVICLYMRPTTNYSLERDKYFSLLYIALGPVLNPLIYTLKNREFQSSFNKARQRLLYF
R*
>jgi|Xentr4|431629|e_gw1.2024.1.1
MDEKTIGHSPLVNSKHLYDPPRRTSSLIHNIPFYLSFIRFHLENFQQLQAMMFAVKEINTNP
NILPNISLGFQAYDSCDVLQQDLEGTLQVLSGRNGAIPNYRCIENMPLSGVIGASISTHSILL
AHILGLYRYPQVSHYSTSPILSNQKMFPSFFRTVPSDAFQSQGLAKLVLHFGWTWVGLLAV
DNDYGQQGIQLVKQEIIKAGACVAFTESILTSQPDRNARNIVKVMKESTATAIVVFSPAIDL
VHVLAEMLAQNVTEKILVASEAWSTSTLFSDGKFSEILSGTIGLALHSGKIPGFREFLNKVH
PSTSLGKYWVKLLWEESFQCKFLGEKNPTATSEDLRRECTGEETLASIENNYNDVSSLRAT
YNVYTAVHVMAKALEDLTNCNKGRSRLSNQTMGFFIQLLHYVKKVRVTLSSGRELFFDK
NGDPPAVYDIVNWKGTPNGTIKQVNVGSYDTAASFGQVFTLNKSAISWPAGILQVPLSVC
SQSCPLGFRKTSERGKPACCFQCIPCPQGEISNITGKWWHLAQCSLHDNICLWDEWPNGH
KTRCMQKNIEFLSYEDPLGAGLAATGITSSLVPVVILKLFMKYKTTPIVKANNYSLSCILLS
CLPLCFLCSLAFIGYPQQEKCLLRQAAFGLVFALCVSCILAKTVMVVFAFMATKPGSRLRK
WTSPIVSYMIIAICFALQCILCISWLLQAPPFPQYDIQTKPGVIIIECNNGSATAFWCMLGYL
GFLAAISFSVAFLARRLPDNFNEAKFITFSMLAFLSVWVSFIPASLSAQGKYTEAMEIFAILS
SSWALVVCMFFPKCCIIKFRPNMNSKKQLMKKAGSQI*
>jgi|Xentr4|431709|e_gw1.2051.3.1

MENQTFILAGLSDIPSLQLPLFLVFLLIYLITLTGNLLILLLIFTDSHLHTPMYFFLGTLACLD
MSYSSVTVPRMLFDLFRELRTISVPACITQVFFFLFFAVSEMSVLAVMSYDRYVAICRPLHY
MQIMTWNICVQFVSGVLFISTLYSLVHTLFLMKLTFCLPNVLQSFFCDLPQLHGASCSDTLT
NRLLIFLLGLLLGVGILGSTFYPYIPIITTVLKMNSKLTRSKAFSTCSSHLTVVFIFYTAGFFN
YLRSNANDHLVEEKVASVFFAILTPSINPVIYSLRNQELKLSLRRTLQKLK*
>jgi|Xentr4|431711|e_gw1.2051.11.1
MKNQTTVYTLVLAGLSELPSLQLPLFLVFLQVYLTALTGNLLILLLIFTDSHLHTPMYFFLG
TLACLDMSCSSVIVPRMLFDLLRETRIISVPACITQFYFFSLFIASEMSVLAVMSYDRYIAIC
HPLHYMQIMSWDVCVQFVSGVLLFTAIYTLVHTLFLTTLTFCLPNVLQSFFCDLPQLLQISC
SDTFINVLLIFLLGIPYGAGILGLTFYPYIPIITTVLKMTLKHTRSKAFSTCSSHLTVVSVFYT
TIFFNYFRSNANGHLFEGKVVSVFYAILTPSLNPVIYSLRNQELKLSLRKTLQRLK*
>jgi|Xentr4|431713|e_gw1.2051.12.1
MENQTTVYTLVLAGLSDLPSLQLPLFLVFLLIYLITLTGNLLILLLIFTDSHLHTPMYFFLGT
LACLDMSSSSVTAPRMLFDLLRERKIISVPACITQVFFFFLFVLSELSVLAVMSYDRYNAICR
PLHYMQIMTWNICVQFVSGVLGFSITCSMVHTVFLTKLTFCRQKVLQSFFCDLPQLLEASC
SDTFINVLLIFLLGILYGVIIIGLTFYPYIPIITTVLKMTSKHTRSKAFSTCSSHLTVVSIFYTTIF
FNYFRLNANDHLVEDKVASVFFAILTPSLNPVIYSLRNQELKLSLRRTLQDFNSFRTIIKAM
GL*
>jgi|Xentr4|431945|e_gw1.2098.5.1
MTSDNETCFHPTTFILLGIPGLEPFHTWISIPFCSIFLIAVIGNLVVLQIIISEVSLHQPMYIFVT
VLSIIDLVLANSTMPKLLSIFWSSSNEIPYHTCLFQMFLLHAFSAIESGIFVAMAFDRYVAIC
NPLRYKVILSNGTIIRTSALAVIRGVICILPLFLLAERLPWYRSNIILHSYCEHMAVVGLACQ
DVSLNDHIGMVVGFLVLAMDSLFIVLSYIKILRVLQRLPSTAGLKAFGTCVSHVCAILTFYV
PILVSSLVHRFGRNVPHPTHILLANFYLLIPPMLNPLVYGMKTKKIQQKVRKYLYLF*
>jgi|Xentr4|431946|e_gw1.2098.6.1
MSWTFTLSGIPELGGNQQWFAPPLCALYVTAFLGNCTILFLIRTERGLHQPMFLLLAMLAV
TDVGVSLTTLPTMLGIFCFNHHEIYSELCLTQMYFLHTFAAMESGVLVAMAVDRLVAICAP
LRYASVLTNSAVGRMGLVIVARGVCVVLPVPLLTRRFPFCKTRVLSHSYCLHQDVIRLACA
DTTVNSVYGLVAVLLTKGVDSMFILISYGLILRAVINMRANDARLKAFSTCVAHMCAVLLF
YIPLIGLSVLHRVGTHASPLLAIVMADVYLLLPPVVNPVIYSVNTKQIRHKMTRLFRRRRV
GVPANGIAVVKV*
>jgi|Xentr4|432654|e_gw1.2264.3.1
MFKTSLCSKMCSSGQRRAHQNGRPPCCFDCVPCSEGEISNSTDLETCVKCAEDHWPNPTR
DQCIIRVIDFLSQEDHLGNILSGSASVFTVSTAAVCLVFIKHRRTPIVRANNQNISYILLMALL
MSFLCTFIFIGQPTGVTCMLRQTTVMFVLSIAISSILGKTLMVLAAFKATKVHKKFRKWIRI
NISVGVVFLCSFGEFVLCVIWLSLYPPHVESDKTIPGKLVLQCNEGSIISFYLAVSYIGVLSLI
SFAVTFIARKLPDRFNEAQHITFSMLVFCSVWASFIPTYLSTKGKHMVAVEIFAIQASAAGLL
LCIFTPKCYIILLKPELNVKGKPTAKIQSHTTNQTLNNY*
>jgi|Xentr4|432862|e_gw1.2326.5.1
MGPLPSPRILFFLLVQRSVGVQASTAQHSIKIDGDITLGGLFPVHAKGPAGTPCGEVKKEA
GVHRLEAMLYALDQINGDPELLPNLTLGARILDTCSRDTYALEQSLTFVQALIQKDTSDIRC
SNGDPPILSRPERVVGVIGASASSVSIMVANVLRLFSIPQISYASTAPELSDNNRYDFFSRVVP
PDSYQAQAMVDIVKALGWNYVSTLASEGNYGESGVEAFMQISREAGGVCIAQSIKIPREP
RPGEFDKVIRRLLETPNARGIIIFANEDDIKRVLEASQRANQTGHFLWVGSDSWGSKTSPVL

GLEEVAEGAVTILPKRASIEGFDQYFTSRTLENNRRNIWFAEFWEDDFKCRLTRSAARPEDP
RRKCTGTRLGAPRIAKDSAYEQEGKVQFVIDAVYAMAHALHNMHVDLCPGHSGVCDKM
DPIEGRLLLRYIHRVNFNGSAGTPVMFNENGDAPGRYDIFQYQMANDSAPSYRVIGQWTE
YLRLSIEEMQWSGGQSDIPSSVCSLPCQPGERKKMVKGVPCCWHCELCDGYQFQLDDFT
CQLCPFDMRPNENRTACRATPIIKLEWTSAWAVLPLFLAIVGILSTLFVVGTLVRFNDTPIVR
ASGRELSYVLLTGIFLMYAITFLMMGEPGVAVCALRRLFLGLGMCISYAALLTKTNRIYRIF
EQGKRSVTPPRFISPASQLVITFSLIGVQLVGTSVWLAVLPPHSFIDYEEQRTPNPENARGVL
KCDMSDLSLVSCLMYSILLMVTCTVYAVKARGVPENFNEAKPIGFTMYTTCIVWLAFVPIF
FGTAQSAEKIYIQTTTLTVSMSLSASVSLGMLYVPKVYVIIFHPEQNVQKRKRSFKAVATAA
TMSTRLSQKNNNEQQNGESKAPAGKSLPESK*
>jgi|Xentr4|434333|e_gw1.2898.1.1
MYITCFTITFILGTVGNGLVIWITGFKMKKTMTTIWFLNLAITDFSFCLILPLFITEKAMWG
NWPFGQIMCKVTYFTRYLNLCTSVLLLTAISIDRCVCVMCPIWSRNHRTSRSAAIISAIIWFF
SMAAGSPLICFSNILYYRNFSECSLTYEAWENIIDYETYSVIMIAFGITTFVLYFLIPFLFITIC
YGLIAFKVFKSKRIPGSARTLKMIITTAFCFFFCWFLLYLLCIIEIAGPNIMRHHLEMTLYTLA
QCLAFFNSCLNPIIYVFTGRDYKQILKKSIPFLLESAFIEKREPAHI*
>jgi|Xentr4|434796|e_gw1.3130.1.1
MHKVNQTSGISFLLLGFQNSQIINEFLFVPFLWIYILTLFGNLLIIILVITVSALRSPMYALLSQ
LSLADVLLSTSITPNFLWLLLNGGGTISATGCITQFYFYGVSTTSEFYLLTAMAYDRYLAICS
PLHYVSIMGFSLCSWGVALILSLFVNLLTFNLQFCGPLVIDHYFCDFTPLIKLSCTDYKPVEL
TLIILTIPSMLLPFCFIIFSYVSIGLAILRISSTEGRHKAFSTCSSHLIVVCTYYGTLIIVYMVPS
KGHNFNTKKMLSLLYTVGTPCFNPIVYSLRNSEIKVALYKCMSNQISFLHSI*
>jgi|Xentr4|435511|e_gw1.3406.4.1
FSFQTRYYRDVLGLIFAIDEVNRSPDLLPNITLGFRLFDSCMSELRATGGALTVMSEMRNPA
PGYDCHEHSHVVGIVGELFSALSLPIARVLGVLHVPQISHGSTLSALSNKINFPSFLRTVPSN
MFQNTALTRLVGLFGWTYVGMLVVDNDAGEQGGQVIQAGIEKAGSCVAFLEKIHLSYSM
AQIQRVVDIIRKSSVNVIVLHSPEVHVKVLLDSLYDEEVTHKVFISSASFGLTPGVFSRKSW
KVLNGTIGLIPSTGAMPGFEEFLGSLHPSRYNKYPLIRALWEKAFSCRWPGGEIQGNKTDP
AVFLPLCTGEEDLGGLVPSLFEMKDLSYTYHAYLAVFAYAQALHSLLLCQAPTEKSHYGT
CGDARDTQPWQVLNYVKRTPFRPNTGEPIAFNADGDIPAAYEIVNVQILNGSFNLVKVGR
FDPEAGDSILLDISSIRWNERFSQVPPSMCSTNCRPGSWKAPRRGQPICCYDCIPCPLGDITN
TTDAAECFTCPRDQWPNEEQNMCVPKVIEFLSYQEPLGIFLAITVIIFFLITLAILFIFMKYR
GTPIIKATNRELSFILLVSLTLCFLCCLIFIGSPSPMTCPLRQTLFMVVFSISISSVLAKTIMVIV
AFKATKMHSPLRKWLGPKIPRTVVALCTTAQVGICGAWLLLSPPFPQVNAEIEKHKLIREC
HEGQSLFFYVTLGFMGFLAMVSFFAAFLARNLPGSYNEAKLITFSMLVFCSVWVSFIPAHL
STKGKYTVSVQIFAILASSAGLLACIFLPKCYIVLLWPDRNSRGRLTSGASRQHIGHMVQEP
PITSNR*
>jgi|Xentr4|435823|e_gw1.3527.2.1
LRKVHFNNTAGQELYFDINGEQPSIADFVNWQALPDGTTKYVRLINGMQGSQWDEASKN
ITIFWSGGFTEVPLSICSERCPPGYRKAPRKGQPVCCFDCISCSDNEFSNETDSIVCTNCPEW
MWPTEQHDGCRPRSLEYLAYEDPLGGTLASLSVVGSLIPLSILGIFLRNSKTPVVKANNRN
LSYLLLIALFLCFLCSLMFIGAPVNLICVLRQITFGVSFVLCVSCVLGKTIMVVIAFNATQPK
SSRRMWLNSRIPNTLIIVCMTIQLIICSVWVMHSPSFKNNDITSKLGVTILECVEGSPVAFW
CMMGYMGFLATLCFVVAYFSRKLPGSFNEAKMITFSMLIFGAVWISFIPTYLSTRGKESVA

VEIFAILCSCSGLLALLFFPKCYIIILRPEMNNKEFLTGKRGFKHSKVK*
>jgi|Xentr4|438798|e_gw1.6057.1.1
SQCSPNCPPGSRKLLRERAPPCCYDCARCSEGEISNVTDAENCLKCGDYEWPNQEKTHCI
QKQMEFLSYTDDSLTVAFILLSLVFFIIAAVILGILISFQDTPVVRANNHTLSFLLLVSIKLSFL
SVFLFLGRPVDITCMLRQTSFGITFSIAVSCVLAKTLMVSIAFKATKPGSPWRKWVGVKLA
NGLVFICSLIQFLINVIWLVIAPPYVEHNTHSEPGKIIIQCNEGSAIAFYIVLSYMGLLASVSFI
VAFLARSLPDSFNEAKYITFSMLLFCSVWITMIPAYLSTKGKYMVAVEIFAIITSSCGLLFCIF
LPKCYIIILKPEMNSKQYLLRNNK*
>jgi|Xentr4|438858|e_gw1.6134.3.1
MTISMPNQTAVTEFILLGFPGLQPNFFLPVSLTLFLAYIVSLIANSTVIILIILREQLHQPMYIII
ANLALSDLLFDTITLPKIIAKYWFGAGSISFFGCFFQLFCVHSLGSLDSLIIMLMAIDRYVAI
CQPLRYHSIISNKLATLICYFLWPFAALIGLAMTLIALKVPYCGPNRVKNCFCASQFLIVLTC
VDVTLEKKERFIIGMCVHIFSLAVIILSYILIIRV*HLSANNGNWQKAFYTCTTHLLVIGLYFI
PRLFVYSTSQIPLILNADINVLIVCLYTFIPHLASPIIFCLGTKEIRNILGQTFNSILPRAEHRPR
TSMKRHCTSITLKRH*
>jgi|Xentr4|439625|e_gw1.7181.1.1
MHKVNQTSGISFLLLGFQSSQIINEFLFVLFLWIYIVTLLSNLLIITLAITASSLQCPMYALLS
QLSLADVLLSTSITPNFLRLLLNGGGTISATGCTTQFYFYGISAASEFYLLTAMAYDRYLAIC
SPLHYASIMGFRLCLYMSLCSWGLALILNLVMTLLTFNLQFCGPFVIDHYLCDFDPLLKLS
CSDYKAVEITDYILAIPFMLLPFCFIIYTYVSIGLAILRISSTEGRHKAFSTCSSHLIVVCMYY
GTLIIVYMV
>jgi|Xentr4|440199|e_gw1.7949.17.1
MKVLILSLCEMIFTTNIVPKMLQVILEGGCLISFFGCALQLYAFAATGIAECLLLTSMSYDRY
LAICKPLHYTKIMNFRCYLWLVVLSWAAAIILPTISISMIFQLNFCGSSVVDHFFCDLAPVLE
LSCSDTSPAEFEVFAQTIPVFVFTFIYIIATYISIFIAILKIKSTMARKKAFSTCSSHLTVFCTYY
GTMISLYVTPSGCQSVKVNKTLSLLYSVVTPLLNPIIYSFRSAEIRKALASIIFRKTGNKKVY
NN*
>jgi|Xentr4|440390|e_gw1.8039.2.1
MENQTGSTCLFYLAFSNHGEKQPLLSIVFFLIYVIGVLGNLIIIIVIYLDSHLHTPMYFFLFSL
AFVDICYPTVTLPKLMDILLSGNNSITFVQCFTQMYFFLALAAVEATLLSSMAYDRYVAIC
KPLRYHHIMNRRVCVLVIVGTWVSGFANSAFLTFLASKLLICGSNKIKQFFCDIKAVADISC
DRTAFYNAIYVEAFFFGLITFSLNLISYINIIRNIIHIKSKHGRQKAFSTCTSHFTVLIIFYGSGL
WTYLRPPSESVKLDPVFTVLLVGITPMLNPLIYSLRNTEVKNALKRILREKICMPIAEWDLV
TMLGKIIYAGKQHEN*
>jgi|Xentr4|440516|e_gw1.8130.1.1
VNISPRPSFQYYRHYLAFLFAVELINRSSRILPNISLGYRIFDSCEHERKSFYDALAVMSGAE
YQVPNYDCWEKAKLMGYVGDVPSSTKFSLAQLISMFRFPQASRSTIFFLYGALEKLTLND
RTQFPSFYRTIPNEEAEMDGIVQILKHFGWKWVGLIISNDDTGYRAREKISKGLSSVGGCL
AFTAVLREIAHISYHHSEEILQQIRESSAYVIVLYIGTRYASAFTSIFAISRIPTKFWITSSFFPK
VTIFRHQKIKTTLNGSLSLLIQEGEIPGFEQFFYRFSPNKYNDDLTINTWAWLFGCSFPPHVY
VRLQTGGTAKNCTGKETMSEADVSVYGDHNYRVTYRVYTAVYALAQALHNLYSAQPPA
NHWGQLRTLKRKVKAWQFILATYFLKIALFSLHLQMEMTPKVSHGIFQSMPLSQCNEPCA
PGYRKAKIEGKPSCCYGCVRCADGEMSNTTGKSQSLDQKQMRCSEYQKSNTERTECLLK
AINFLSYTDTMGASLTAIAFILFITASVVLGIFVKYWETPIVRANNRYLSCLLLISLMLCFLC

TLLFIGRPTQICCLLRQVIFGVVFTISVSSVLAKTLTVIIAFNATKPGSKLKKYVGTQLAIVLV
IICSLSSTVISAVWMASHPPFLEADTVSEMDTVILMCNEGSVFFFFCAIGYMGAPAVLSFIA
AFLAKDFPDRFNEAKNITFSMLGFCSVWVAFVPAYLSNKGSRMVAVEIFAILASSAGLLGCI
FAPKCYIIFLQPELNTRTF*
>jgi|Xentr4|442474|e_gw1.9575.1.1
MLPALQVIWTVMLIVTWPCGTILNSSIIAVYLSDWKKGVKLGECDQITLSMGCTNLLLQCF
ITLGVAFNSYGLHLPFAVSLAIGAVLLFSIYFSFWLTAGLSVCYYLRLANPLPKFFLQLKRRL
SRIVTPLLLWSVAISFTVTFSNIILYTGTNQNLTILYHNNKSNNITTNDHYIISSVIFMILDGAF
GIGFPTLITSICILLSLISLLRHIRRMKQNPQFGNTQQKTLIGACRTMILLMALNSILFLVILRS
MLPPYNVETIWQTVMFSCVMLNPSGQAVVLIFGNSKLLSAWTKTLVPQG*
>jgi|Xentr4|442627|e_gw1.9700.3.1
MENDTVYAPGLSTVAILAEQEVSRRIYSLFPLFRIINITFFSIIFILGTAGNGLVIWIIGFKMEK
TATLIWFLNLAIADFSFCLFVPLSITEWALWLYWPFGQIMCKTWIFNLQLNLSASVLFLMIIS
VDRCICVLYPIWAKIHRTSRLASTVSVIIWFLSVGLSSPYIVFYDYEEYDGQYSYCFPTFAV
WDNSTTFAEETWNLREKAMIMTRFVSMFLIPFSIILVCYGLIAFRVRKGRRMHGSGRTLKM
IFTIVICFFLCWFLFHILPLIENADIYIGFPCDVILNNLAYCLAFLNSCLNPIIYVFMVQDFQK
NLRKSIPFLLERTFRESSDPPETLNN*
>jgi|Xentr4|442714|e_gw1.9769.1.1
MDGLKCFQILVATIYSLVCALGLMGNFFVMYLIRAKRATGLTAIDVFIFCLALTDFQFALTM
PFWAVDALLDFSWPFGHPMCKIVLTMTVLNVYVTVFLLTAMAITRYWAVASALSLRGRVS
TSAAKGISLALWLVALVATIPTTIFATTFPVLGEELCLLKFPENKWLATYHLQRVIVGFVIPFL
LISTSYIMLLSFLRQHKVNANNRDRQSRINNSVQLVIMVFFICWFPNHAATFWGILIKYRAV
QWSDAFYFFHTYVFPVTLCLAHSNSCLNPIIYCLMRKEFRKALKASFWQLYSFAASFWPS
WSPKRTTSDQETMVPLYRETSLPNVGSRDYAVTSVCTITTIQEFTTKDQSHQNTGQRRQDT
ATV*
>jgi|Xentr4|443278|e_gw1.10510.2.1
MDITNQTDIPGFVLRVFGGSSKFHPILCLILFLCFFLTLAINLLVIIIICMDRHLHSPMYFFIAS
LSFMEICGISSVIFNLLAILLTNKTYISKAGCFLQSYIYYFFSTSDYLILGIMSFDRYVAVCNP
LKYNSIMRNSVCVKLVIGCFVTSFLCLLYPTLMITNLPFCGNVLDHFFCESEAIMRLFCVDT
SIFRLLAVVSSVFILLGSLIITIISYILILSSILKISSVTGRSKTFSTCLSHLTMVSIVFGMSIFNQL
RPHRDDFIELEKALNVGNIMVAPLLNPFVYTLRNQMVKDSIRNAIRSRKIL*
>jgi|Xentr4|444103|e_gw1.11769.3.1
MEIFTPSLYFPNVSTAATLTDEEFDDRSFVSFQIITITIHSIICILGIAGNGLVIWIIGFKMEKTA
TLIWFLNLGISDFSFCLFFSIYMIQGALPHNWLFDWIMCKTXAFILHLNLFTSVLFLMIISID
RCICVLYPLWSKIHRTSRLASVTSVIIWIISVALNFPYIIMNDYREYGNWSSCSPAFDAETWI
LSYKAMSMTKLVSMFLIPFSVMLVCYGLIAFRVKRSRIPGSGRTLKIIFTIVICFFFCWIPFHII
YMIDYADIDIGYRCRAILYILAESLAFFNSCLNPIIYVFIGRDFKKSLRKSILFLLESTFRESND
PPEMLNKKVSET*
>jgi|Xentr4|444591|e_gw1.12684.2.1
KMENQTTVYVLVLAGLSDLPSLQLPLFLVFLLIYLITLTGNLLILLLIFTDSHLHTPMYFFLG
TLACLDMSCSSVTVPRMLFDLLRERRIISVPACITQVFFFTFFIVSDLLVLAVMSYDRYIAIC
RPLHYMQIMSWNVCVQFVSGVLVSSALYSLVHTLTLTKLTFCLPNVLQSFFCDLPQLLEVS
CSDTFINVLLIFLLGLLSGVGIVGVTFYPYIPIIRTVLKMTSKHTRAKAFSTCSSHLTVVSIFY
TTVFFNYFRSNANDHSVEDKMASVFYAILTPSLNPVIYSLRNQELKLSLRKTLQRLQ*

>jgi|Xentr4|445661|e_gw1.14842.1.1
MGYTSIPAANVLASVVLFVALLVGLVVNSLYLWVLRFRMPRSINTIWFFHLILCCFLFTFIM
PFIAVYVLMFPYWLFGSLLCKLTSSLINVCMYASVFLLTVISLDRYCLVFHPVWYRGHMNN
RYATAICICIWGLTILFSSPYFAFSQTRLLKDNKTAICIDYILSRSIWPKSTMQLEWVIISFRLIL
GFLLPFAVMTVCYVRIALRMKKGNLARSTKPYKIMFISVASFFVSWFPYHLWYGMNGRIH
RSTSNILLALAICMACMNYCFTPLLYLFITENFKKSIQKSVLLLIESVFNEIFNSLNRSFELKP
EAPPSSASKTGTGEEQIEEIQ*
>jgi|Xentr4|446244|e_gw1.15666.3.1
MYPSSSSPQWQLQSTIWTQNKSGAAGFLIVGFSDSPELQGPLFLFFLLIYLVTVWGNLLLIS
LISTDSHLQKPMYFFLCNLSCLDISYGSLTSPYLLHIFSTGKNNISFMMCMVQLYFFSSFTVI
EYLLLTTMAYDRYVAICRPLFYPQVMNQRTCILLAAAAWIGGFLSGAPVTILISALTFCASN
IINHFFCDILPLLKLSCDDTSTVEFVMFAQGSLFLFSCFILTMMSYIYIISAILKITSVEGRHKT
FSTCGSHLTSVTLFYVLLFCVYMKPVSLYSLDEGKVVSILYVNIIPMLNPIIYSLRNKDVKK
ALPKAWFHCMPRAILGDSLI*
>jgi|Xentr4|446549|e_gw1.16175.1.1
MLSELHLIFSIGLVLSWICGTVLNSSIVAVYLSDWKKGLNLGACNQIILTMGCTNLLLQSFL
TFHLMFITYQFSVLLDKELLLAAVSFGLHFSISLSFWLTAWLSGYYCVKLANSSNRFFIRLK
REVSTVVAYCLLGTVLTLFIAEMPVIWTTHIITDQNQTSIYCIMDDNIALVSFNFTISCLLPTI
MTSFCIGLSLISLLKHVHKMKQNTSQFWNPQLKSHVKACRTMLLLLAVNLIFSLAVFISSQ
PTDKAEVIGVYVPWFILMSYPSAKAIILLFGQSSCFLSKRVI*
>jgi|Xentr4|449615|estExt_fgenesh1_pg....
MVAPLSYSIQATAVIAAIITFLVLFTIFGNVLVIIAVLTSRSLKAPQNLFLVSLAAADILVATLII
PFSLANELMGYWYFGKTWCEMYLALDVLFCTSSIVHLCAISLDRYWSVSQAIEYNSKRTP
KRIKCIILIVWTLAALISLPPLIYKGKSKDQNAKPECKLNEEPWYILSSSICSFFAPCLIMILV
YLRIYLIAKRRNRKNSTDKKLKKMAPGLFSRLALDDLHCGVPVLSSTREERNGHSESFGL
QPQGENLKHTNGCEPAVMDTVATVKGVMLMPKGAKDSDLSSAKKKSYINREKRFTFVLA
VVIGVFVLCWFPFFFTYSLGAICTELCYIPDSVFQFFFWIGYCNSSLNPVIYTIFNQDFRKAF
RRILCSHWSQTTW*
>jgi|Xentr4|450188|estExt_fgenesh1_pg....
MVILYENNTVESIQLCLNCTHPGATINISKAIVVGVIFGVFITFGVLGNILVILSVACHRHLQT
VTHYFIANLAVADLLLMSMVLPFSATFEIQGYWLFGRIFCNIWAAVDVLCCTASIMSLCAIS
IDRYIGVSYPLRYPSIMTEKRGVLALLCIWVLSLVISIGPLFGWKQPAPEDDKICEITKEPGY
ALFSAFGSFYLPLSIILVMYFRVYIVAKRESKVLTSGMKLEKSDSEEVTLRIHRKSTPESIGS
TSSSKQKTHFSVRLLKFSREKKAAKTLGIVVGCFVLCWLPFFLVMPIGSIYPPANSSDTIFKI
TFWLGYFNSCINPVIYPCSSPEFRKAFQNILHARCSHRRQAASKYSSGFLLNSPVHRSSEEN
KNVVRIPVGSGEMFYRISKSDEGCEWKYFTPLPSPATPYGTPRDFANCSSARVSSKSFLRTC
CCVGTQQTRSCHDVPTIKIHTISVSENGEAV*
>jgi|Xentr4|450589|estExt_fgenesh1_pg....
MTEVFLSALLNVTQSTLLASGSSAGNVTKCSLTKTGFQFYYLPAVYIVVCITGFIGNSVAIW
MFIFHMKPWSSISVYMFNLALADFLYVLSLPALIFYYFNKTDWIFGDALCKLQRFIFHVNL
YGSILFLTCISVHRYTGVVHPLKSLGRLKKKNSIYISALVWFIVIAGISPILFFSGTGIRKNKTI
TCFDTSSDEYLRSYFIYSMCTTVFGFCIPFILILGCYGLIVRALIYKDMNNAPLRKKSIYLVII
VLTVFAVSYLPFHVMKNLNLRARLDFQSPEMCNFNDRVYATYQVTRGLASLNSCVDPILY
FLAGDTFRRKLSRATRKASRRSEANVQSKSEEMTLNILSEYKQNGDTSL*

>jgi|Xentr4|450905|estExt_fgenesh1_pg....
MIENKKEKEPLQIKLMDINLLSKAAFFLFLVIIGIPANSYIVLKFIFIKMVEKKFLPTNIILTAL
ALMNLLIVFSRVILQYLNAIGVENLLDDTQCKLSVYTYRVSRSMSISTTTFLSCYQCILIAPS
SGIWSYLKQKVTPNVLAITICLLVINIILDPSSGLYAQAKKNSTASPYTLYLVYCNMAFGTY
NNYIANGSLFATRDFLLVGLMALASTYIVYILIKHKRSVKDIRSSSRAQGKSAEDKASRAVI
MLVILYVLLFGFDNCMWIYTLTLTRVTNDMNDARIALACSYSALSPIVIIANNPKLQPRFK
WLEQRKTLNENHKKVVDGNLKVASE*
>jgi|Xentr4|451024|estExt_fgenesh1_pg....
MELKSSRMAIWILCSIILVLRDTRAACPKDKGRSLKPNNCSSSILDDVIKDYLQSSVMTLIIP
AVYTLVLVLGLPANFIALWVLGFKTKKVPSTFLLINLAAADLLFLLALPFKITYHYFGNNWI
FGEKPCGAVTAIFYGNMYASVLFLMAISIDRYIALVHPFSNKRLRGWGSSIGVSVGIWIVVA
AGMSAFLIVPQTKTFKTPNITTCHDIWAFCCEFEWYKYYFLGLFTIGFAIPLAVILFCNVPVL
VVIAKSREPPRHVIKIITLVLFTFILFFTPSNILLLLHYQKDQWESHNQLYFWYVIALSLSSM
NSCTDPLIYYYMSGDFRTLVKATFCSSKGKDISSASTKSSKLYFSSDNKHSKRMDP*
>jgi|Xentr4|452640|estExt_fgenesh1_pg....
MVFSVMLTPAHSAAYYSTHVIFENGFIKVTSPQPSLSGNLDSLLRYSPVAMTTTETTILPLNI
STTPSTKDLVETLSLPLQIILSAVMIIILLLSFLGNFVVCLMVYQKAAMRSAINILLASLAFA
DLLLSILNMPFALITIITTEWIFGDVFCRVSAMFFWLFVMEGVAILLIISIDRFLIIVQKQDKL
NPYRAKILIIISWATSFCVAFPLAVGKPHLQVPSRAPQCVFGYTTNSGYKAYVILIVLIFFFIPF
MVMLYSFMGILNTVRHNAVRIHSHPDSICLSQASKLGLMSLQRPFQMNIDISFKTRAFTTIL
VLFIVFILCWAPFTTYSLVATFNSDFYNKHNFFEISTWLLWLCYLKSALNPIIYYWRIKKFRD
ACLDLMPKYFKFLPQLPGHTRRRIRPSAIYVCGEHRSVV*
>jgi|Xentr4|453013|estExt_fgenesh1_pg....
MECLYLLLLAVSWLQGSQCAAAKELSCQEITVPLCKDIGYNYTYMPNQFNHDTQDEAGM
EVHQFWPLVVIHCSPDLKFFLCSMYTPICLEDYKKPLPPCRSVCERARAGCAPLMRQYGF
AWPDRMRCDRLPEQGNPDTLCMDYYNRTEQTTAAPSHPEPPKPPARSAPKGRTRVEPPRP
RPRAPGCEPGCQCRAPMVPVTNERHPLYNRVRTGQIPNCAMPCHNPYFSPEERTFTEFWIG
LWSVLCFASTFATVSTFLIDMERFKYPERPIIFLSACYLLVSAGYLVRLIAGHEKVACSRGEL
DLEHIIHYETTGPALCTLVFLLIYFFGMASSIWWVILSLTWFLAAGMKWGNEAIAGYSQYF
HLAAWLVPSIKSIAVLALSSVDGDPVAGICFVGNQNLDNLRGFVLAPLVIYLFIGSMFLLAG
FVSLFRIRSVIKQGGTKTDKLEKLMIRIGIFSVLYTVPATIVVACFFYEQHNRQGWEVAHNC
NSCQPEVAQPRRPDYAVFMLKYFMCLVVGITSGVWIWSGKTLESWRAFCTRCCWGSKAT
GGSMYSDVSTGLTWRSGTGSSVSYPKQMPLSQV*
>jgi|Xentr4|453819|estExt_fgenesh1_pg....
MNSSSDGANEGAGAAADNGPTKVAESIAIIIDILICLGNLVIVVTLYKKSYLLSLSNKFVFS
LTFSNLLLSMLVLPFVVVSSILREWIFGVVWCNFSALLYMLISSASMLTLGIIAIDRYYAVLY
PMVYPMKITGNRAVLALVYVWLHSLIGCLPPLFGWSTLEFDHFKWMCVAAWHKEAGYT
AFWQVWCALLPFIVMMICYGFIFRVARIKARKIHCGTVIIVQEASQKNGRKNSSTSTSSSGS
RKNGFSSIVYSANQCKALITILVVIGAFVLTWGPYMIVISTEALKGKNSVSPVLETLATWLS
FTSAICHPLIYGLWNKTVRKELLGMCFGNRYRDPFHQQHRTSRMFSISNRITDLGLSPHLTA
LMARGTESEHQSTTTNTGFSCTDVMLLDDTSSDATQLHRVIYSRRKSSVTFEDEVEQKND
ARTMPTQPTAPSESLESYAFNLAKAIEMDAKISLFGEDVFPSNVQALPGNSGINRNRINFIQ
KQRVQLQSIEEGNIETSKCDV*
>jgi|Xentr4|454665|estExt_fgenesh1_pg....

MGVRALALCLPLLGCLVGHIAGFGDEEERRCDPIRISMCQNLGYNVTKMPNLVGHELQA
DAELQLTTFTPLIQYGCSSQLQFFLCSVYVPMCTEKINIPIGPCGGMCLSVKRRCEPVLKEF
GFAWPESLNCSKFPPQNDHNHMCMEGPGDDEVPVHSKTPVLPGEDCNSFGPNSDQYTWV
KRSMNCVLKCGYDSGLYSRLSKEFTDIWMAVWASLCFISTAFTVLTFLIDSSRFCYPERPIIF
LSMCYNIYSIAYIVRLTVGRERISCDFEEAAEPVLIQEGLKNTGCAIIFLLMYFFGMASSIW
WVILTLTWFLAAGLKWGHEAIEMHSSYFHIAAWAIPAVKTIVILIMRLVDADELTGLCYVG
NQNIDALTGFVVAPLFTYLVIGTLFIAAGLVALFKIRSNLQKDGTKTDKLERLMVKIGVFSV
LYTVPATCVIACYFYEISNWNVFRYTADDSNMAVEMLNIFMSLLVGITSGMWIWSAKTLH
TWQKCTNRLVNSGKVKRKKRVDGWVKPGKGNETVV*
>jgi|Xentr4|455539|estExt_fgenesh1_pg....
MENLSIYNVTVNVLHADLDVGSSDLSLRALTGLLLSLLILSTLLGNTLVCLAVIKFRHLRSK
VTNFFVISLAVSDLFVALLVMPWKAVTEVAGYWLFGDFCDTWVAFDIMCSTASILNLCIISL
DRYWAIASPFRYERKMTQRVAFIMIGVAWTLSILISFIPVQLSWHKSQEVDEELNAINHTEN
CDSSLNRTYAISSSLISFYIPVVIMIGTYTRIYRIAQTQIRRISSLERAVEHAQSGHPDCSNENS
LKTSFRKETKVLKTLSIIMGVFVFCWLPFFVLNCMIPFCHMSLPGQNEPEPPCVSETTFNIF
VWFGWANSSLNPVIYAFNADFRKAFTTILGCNRFCSSNNVEAVNFSNELVSYHHDTTFQK
DIPVTFNNSHLPNVVDQDQEVLEGTCFDKVSVLSTSHGTRSHKNLHLPASVQFECEAEITL
ETITPFTSTGPLECLPQLVADEDRHYTTKLY*
>jgi|Xentr4|455626|estExt_fgenesh1_pg....
MNAFGGRENSTYKCTFNEDFKKVLLPVSYGIVFCVGLILNILALYIFLFRIKPWNASTTYM
FNLAISDMMYVISLPLLVYYYSQGDNWPFGVALCKIVKFLFYTNMYCSISFLLCISIHRFLG
ICYPMKSLGWLKVRNARIISVVVWVIVSACQSPILYFVTTSSNGDSTTCHDTSSKDLFDNF
VVYSTVDLALLFCVPFITIIICYCLMARTLMKPSAATAQTSASKKKSIKMIIIVLVVFIICFLPF
HINRTLYYYFRKMELDCDILNAINLAYKVTRPLASANSCLDPILYFLAGQTVRRSIIPRNGL
SKMKNKFTSYVLENNWSGSATGVSNSEARQSPAVISKV*
>jgi|Xentr4|455743|estExt_fgenesh1_pg....
MTWKLILSALLVLLMLCQAPSAQIMDEAYENWRKYETECRHNMSQEPPAAVQHGLVYK
HCEPDGQWAERDAQECENDDKQIEDKYFKIYDNFKIMYTVGYSVSLGTLILAFAILVGFSK
LHCMRNYIHINLFASFILRAVSVLVLDTMLKTRYNENEKFEDTHLWLSSEALVGCRVAAVL
MQYGIIANYYWLMVEGIYLYNLLVLAVFSERSYFALYLCIGWGAPALFIIPWVAVRYTYEN
TLCWSTNNNMGFWWIIRSSVLLAIVINFVIFVRIIQILVSKMRAHQMRYTDYKFRLAKSTLT
LIPLLGIHEVAFAFLPEETVHGTLRLVKLFFDLFISSFQGMLVAVLYCFVNKEVQSELLKKW
KRWKLGKDIKGEYKHTYTQTPRVGASTVYENHKLVGCWHKGTGKPRHNGSMYYDDKN
STTENITLSERQLSFNYPDSGENTF*
>jgi|Xentr4|456291|estExt_fgenesh1_pg....
MDTHNLSMYNDDINNGTNGTAVDQKPHYNYYAMLLTLLIFVIVFGNVLVCIAVSREKALQ
TTTNYLIVSLAVADLLVATLVMPWVVYMEVVGEWRFSRIHCDIFVTLDVMMCTASILNLC
AISIDRYTAVAMPMLYNTRYSSKRRVTVMISVVWVLSFAISCPLLFGLNNTASKVCIIDNPAF
VIYSSIVSFYVPFIVTLLVYVQIYIVLRKRRKRVNTKRNSHGVEVNAHKDKCTHPEDVKLC
AVFVKSNGSFPTDKKKVILVQEAGNHPEDMDVEMMSSTSPPEKTKHKSASPNHHQLAVPA
TSNQCNNANLPSPVDSPYKAEKNGHPKDSTKPAKVFEIQTMPNGKTRTSIKTMSKRKLSQ
HKEKKATQMLAIVLGVFIICWLPFFIIHILNMHCNCNIPQALYSAFTWLGYVNSAVNPIIYTT
FNVEFRKAFIKILHC*
>jgi|Xentr4|456895|estExt_fgenesh1_pg....

MGDTGGTGENIIIYQNFLVNRHYLIGLVLSCLYTILLFPIGFIGNIMILVVNISFREKMTIPDLY
FINLAAADLILVADSLIEVFNLNEKYYDITILCTFMSLFLQINMYSSIFFLTWMSFDRYIALA
KVMKSNIFRTKMHARLSCGLIWMASISATLLPFTAVQVQHTGDMHFCFADVKEIQWLEIT
LGFIIPFAIIGLCYSLIFRVLIRAHKHRCMRPRRQKALRMIVIVVLVFFICWLPENVFISISLLQ
SEDEEQSYNRSFRHNHPLTGHIVNLAAFSNSCLNPLIYSFLGETFRDKLRLYIKGKKSMAAF
NRFCQTSVKSVIPESSDHSEAKYITGV*
>jgi|Xentr4|457013|estExt_fgenesh1_pg....
MAENQDDYYSSDDIFTGLPIIPPGASPCNYEVTSRFNKWFIPATFLLVFLLGLVGNGLVLYV
LKSRRCSWHLSDHYLFHLTLSDLFLGLTLPFWATQYAYGWVFGSVPCKLVGALFSINMYSS
IFFLACIGLNRYFAIVHAVELHRKQRPIHTFLICAVVWATSCLLSLQEFYFRDVDFIKQLKSH
SCHYKFDPETADTWRTTIRLINLSLGFLLPLFLMFFFYCRIFCTLRKSRHGHSYRSQVVIVV
LLFVFVLCWGPYNTLLLIDSLQRLDVIAPSCPLFQKLDIGLTVTETLGLSHVCLNPFIYAFV
GVKFKSELSRLSKRVSGKVISSGVTGSKEETIVIETNNSYTKVF*
>jgi|Xentr4|457534|estExt_fgenesh1_pg....
MGGDILRFTVLVVLAGIAVSSSFGEHYQNRTDANTDFTMLNRSYTPPVRRMNRSELCPEK
TKINHVFKYINTILSCAIFIVGMVGNATLLRIIYQNKCMRNGPNALIASLALGDLIYIVIDIPII
VFKLLAQRWPFDQSPVGSFLCKLVPFIQKASVGITVLNLCALSVDRYRAVASWSRVQGSGI
PLITAIEIISIWVLSFVLAVPEAIGFVMVPFEYRGEEFRTCMFHATSPFMMFYKVAKDWWLF
GCYFCVPLACTGVFYTMMTCEMLHQRKGSLRIALSEHLKQRREVAKTVFCLVVIFALCWF
PLHLSRIIKKTIYNELDPSRCELLSFLLVMDFISINLAALNSCINPIALYFVSKKFKNCFQSCL
CCCCQSKTHINTAPMNVTSIQWKNHEQNYYGADRSIHKDSIN*
>jgi|Xentr4|458479|estExt_fgenesh1_pg....
MDLLQPPNRLLRLYREYQNNEVISLHYNFTGKLNTSRYKGGLKAEAVFFLVVCVFIVLEN
LIVLLALWRNKKFHSPMFYLLGNLTLSDLLAGVAYIVNILMSGANTLRLTPAMWFSAGYR
HRTAHYYGSDETLQRGQKGRMALLIGAAWLVSILLGVLPILGWNCISNLQSCSTILPLYSK
NYLLVCISIFLAILIAIVVLYLRIYRIVKVNSQRLGTLRKGTLKKSQKYMALLKTVTIVVGTF
IMCWLPLFVLFLFDVSCEVNACPILLKADYFLGLAMINSLLNPIIYTLTSRDIRRAILKLVFC
LCLSSDEGEGRRRLWFLPVIEGSTSKSEKSSHKHEALETTVSSGNGTPTPVKCLMPKNVDY
*
>jgi|Xentr4|458936|estExt_fgenesh1_pg....
MGSQNESSSHPEYFLLVGIPGMEDAHFFFSIPFCSMYILALAGNLLLIYVIATNTSLHQPMY
QFLVMLAMSDILLCTTTVPKSLAIFWFQSSRIPFDGCIAQVFFIHFLFVTESSVLLTMAYDRY
VAICYPLAYTTKMTNSFIWRVVIVALTRGFCTTGPFTLLLKRLPYQGSNVIAHSYCEHMAM
AKLATADILVNVVYGLIIAFGITGIDMILIAVSYVVIIRAVIRLPSSEARYKAFNTCVSHLCVI
TLFYVPAFFSFITHRVGHNVIPLYAHILLANLYCLVPPMMNPIIYGVKTKEIRRKVFGLFCRK
AFLM*
>jgi|Xentr4|459908|estExt_fgenesh1_pg....
MGCNNTALDSCMLPNLSIATAPPDSRFAFSTPLRILLAIIMIVMIAIAFLGNAIVCLIVYQKPA
MRSAINLLLATLAFSDIMLSLFCMPFTAVTIITGSWLFGTQFCQISAMLYWFFVLEGVAILLII
SVDRFLIIVQRQDKLNPHRAKIMIATSWVLSFCISFPSVVGWTLVEVPTRAPQCVLGYTEFS
ADRVYAVMLIVAVFFIPFSVMLYSYLCILNTVRRNAVRIHTHADSLCLSQVSKLGLMGLQR
PHQMNVDMSFKTRAFTTILILFVGFSLCWLPHSVFSLLSVFSRTFYYSPSFYVISTFILWLSY
LKSVFNPVIYCWRIKKFREACLEFMPKTFKILPKVRGRTRRRIRPSTIYVCGEHQSAV*
>jgi|Xentr4|460257|estExt_fgenesh1_pg....

MQGVTRSSNLIVCHLFALCLLSFAQLHGEKGISVPEHGFCQPISIPLCTDIAYNQTIMPNLLG
HTNQEDAGLEVHQFYPLVKVQCSSELRFFLCSMYAPVCTVLEQAIPPCRSICERARHGCEA
LMNKFGFQWPERLRCENFPRHGAEQICVGQNHSEDGGPTLLTTSPPHHGTPGPPIYATLDH
PFHCPRVLKVPSYLNYRFLGEKDCAAPCEPSKNDGFMFFSQDEIRFARVWILIWSVLCCAS
TFFTVTTYLVDMQRFRYPERPIIFLSGCYTMVSVAYIAGFVLGDKVVCNENFSEDGYKTVV
QGTKKEGCTILFMMLYFFSMASSIWWVILSLTWFLAAGMKWGHEAIEANSQYFHLAAWA
VPAVKTITILAMGQIDGDLLSGVCFVGLNNIDPLRGFVLAPLFVYLFIGTSFLLAGFVSLFRI
RTIMKHDGTKTEKLERLMVRIGVFSVLYTVPATIVIACYFYEQAFREHWERSWVSQNCKS
LAIPCPLQYTPRMTPDFTVYMIKYLMTLIVGITSGFWIWSGKTLHSWRKFYTRLTNNKHG
ETTV*
>jgi|Xentr4|460715|estExt_fgenesh1_pg....
MVLAHGMDSSRSQHSNPTILYPAHNTSLTTCPMSTGELWVPGLYKDVSTGKTNGTVPAQL
LFPGEIAAAGLIFGVIWILSLFGNALVCLVIHRSRRTQSTTNYFVVSLACADLLLSVGSAPFT
LLQVTSGHWALGSAMCKLMRYFHYLTPGVQIYVLLSICLDRFYTILYPLSFKVSREKAKR
MIAASWLFDAAFISPIFFFYDGVDGHCNFFPPPSWDGAAYGVVHLIVGFVVPSGLIILFYQK
VVKYIWRIGTDGLTVRRTMNIVPRTKVKTIKMFLMLNTIFMVSWLPFYVVQLWHPDEND
RRQSCLVFLAVSWLSFGSSAAKPTLYSVYNANFRRGMKETFCMSSMKCYRSNAYTITTSS
RMAKRNYVGICEMPVPAKTAVKESVYDSFDREAKEKKLAWPIDSNPPNTFV*
>jgi|Xentr4|460929|estExt_fgenesh1_pg....
MPVIKIPENDENFTLILTNVFGGAEINSSRSKVQIMIRKNDSPVRFSQSSYMVPETAHVIVIP
VIRGKDLTDTAVGSDDVDVSIKYKIVTKNTTASAQLGKDFSDLQPNNTIIFPPKAFEYSLKF
QIIDDTIPEIAESFQILLMGDTLQGDAVLLFPDVVTVTIEPNDKPYGVLSINSALYAQPVIIDE
DQISRYEGISIVRNGGTHGHVSVNWTIQRNSSDPSLVTAGILPSSGVLQFTQDQMMVSLPLT
IISDDFPEEAEAYILRLLTNTVQGGAEVGSPTELIFFIQDSDDVYGLIQFHAIEDQKIEISHEG
RFLTLKFARQHGTRGVVKLIYSALYIPAGPVDPWRAKDGVLNASAQNSVTFAEGVSLITV
NLPIRNDAFLQNGANFLVQLKTCELLSPFPIVPPVSPRLGTLQNVSLSVTADIANGEIGFTSN
LSIIVSEPENASEVVSIALHRDGTGGEAVVFWSLKPTGLNANAVTLNDLSPFNGSVKFLHG
QSDTTINITIKPDDIPEVNETVIISLNRVSVENHILKSGFTSCELTILQNDDPGGVFEFSTSSRG
PYYVKEGDSVELRISRSKGILVQQFLRYTVEPKDSNEFYGNTGILEFKTGEKEIVITLLTRID
WIPELDELYSVVLSSHGEFPTTLGDARAVNITILKNDDPHGVIEFISDGIVMSLNESKGDEIC
NATFTVIRKQGNFGNINVSWFIIPNSTNDVYPTQGILSFQDKEISKMITISSLPDEIPEPKETF
TLNLLNATGGARMGSIITAKLEINKNDDAVFFKEPVVIWVEEGQSANLTVVRNGSADFVSS
VMYTTINGLARAEDKDFIYSSGNLLVFDVGQRVLNITINTSEDDIPETDEPFYILLLNATGD
TVVSGNGNATVIIKANDDPNGIFSIEPVNKSVEEGTTTNFKVVRNRGHFGNVYVQWAIFQ
NNSALQPGEEFYNHSGSIWFRNGEQLNYIILHAIPDLIPEFNEFYTLMLVNISGGSPGPGGK
LAQKDLSVTVMIPFNDDPFGVFVIGPESLNREVAEDVNSEDDMSYITNFSIWRHQGTYGN
VRVGWEVLSSTYRNGLPPMMDFLLHALFPPSVLLQPHKRRHYSGTDALYFNGAEDSFGLI
DEHDISDMNNTLANFTFSAWAIPQVNTNGFIISKENHNGILYYGVQVETNGSYIFLTLCYTT
FGSNITYIARAGMPKLIDETIWIHLLITLDDGFIEFYIDGSPIPGGVKSLKGEAIINGPGTFRV
GAGLNGNTSYTGMLQDVRLYFQRLSRAEISELHATPAKSDLHPISGYLEYRQGETHKSFIIS
ARDDSEEEGEELFILKLVSVYGGARIPEENTTAVIRIQKSDNANGLFGFTGPCIPQSVEEGST
ISCVVERTRGVLDTVHVFYSISQTDVPSTNQTLADFASSTGNITFLPFQRSRVLNLNAVDDE
IPELAEVFRVTLVSALSADGKIGSTPTSGASIDPNKRVEDIKIVASDHPYGLLQFSLGPPPKT
EDKMTLPATSVLTITVKEEIGQVKLLVVRAQGLLGRVRVDYRTISLTAFSPKDYKETSDLLD

FQPGERYKYITVNITDNSIPELSKSFRVELLNADGGVAELFRNDGSGSGDGDMEFFLPAVH
QRASLGIASHIIVTIEDSDDAHGVFEFSQESLQVLRDRGALSQVLLFWTIDVDPEKDFAFTS
GNVTFDIGQRKANITIYVSPDEIPELDKIFSVSLTNVSRGRLGNFTNASVIIFANDDPYGLFV
FSEKNRPIKVEEANRNITLTVQRLKGLMGTVMVTYRTILDTEKIGFLPSTIARATLGKDYLP
VSGQLTFKVNISEANILLPILDDNDPERAEWLFVELFNVTLIVKEQSQPVLESPRLGSKSNV
TAQIVINASDDAFGVLQLSATYVRVAESYVGPIVNVTRIGGMFADVSVKFKAVPITAIAGED
YTVASSDVVLLEGETSKAVPIYIINDINPEVEESFRVVLINETTGGALVGEITETVIVIEASDD
PYGSFVFQATSVTVQEPDTNTVSVKLPIIRNAGTLGNVLVQWAAFINGQLATTDLKVASGN
VTFMAGETLKILSLEIVADDIPEDNEIIFVQLTGASNGGSIGLDGVAKITIPANDNPYGTISFY
RQEYRVQEPLEQSSLANITVRRSAGRFGRLQILYSTSEIDVIAVAAGQGQDMLSYYEHPLPG
IPSLPSRQTFNVSVAGDPLQSCAINCLKNQVCAAFSFSNTSGIPQCFWLTTLSNLLNNGTRF
LTYKKNTTSVSVLFSTQATAGSDYESVTGQWFTMLEGQEFANLTVSILTDTFPELDERFIVS
LLEVKLLNISTSLKNQPTIGQPNSSVVVIKMNGDAFGVFVIYSLNPNALEQGHYLEVEEQP
KTTLQLVIERREGSLGQVAVEWGIVGGTATKNEDFIVEDNVLIFSEGETRKLVTLTIKDDLV
PEESETIIIQLTQTDGGSRILPSSNTVKIVILANDNVAGVISFHSNSRSVIGHEGEQLEFHVLR
SFPGSGNVTVNWKIVGAHLELNFENSSGLLFFSEGSLNASFLVHLLDDQIPEERELYQLILY
NVTTQGITGNGVAVLDKQGYEAVLTVEASDEPYGVINFAPSSRNVLTQEGNKTIQLFINRE
YGSLGDINITYATTTDILNQSNIHPAEPGRDYISAFGSLIMKDGATTAAINISILEDDIPELQEY
FFVSLTSVELIVKLMTSSPPHLDIKGLTAQIIIDANDGAQGVIEWQSTSYEINETQGILTLVAY
RNKGTYGNVTLFFYAQNLEAQLGIDYNATSMVLSFDDGERYKFIDIMIYDDNIPEGDESFQ
LILANPSRGLELGTNTTATITILANDDGHGILSFNNSEHFYLREPTASNMFESVALLYIVRDP
PQGIFGRVTVQFIVKGENGSDAEGDLSPTQGYVTLEDGERFKTLEITAILDEEPEMDEHFTV
ILLSPTGGSKLGTRVQTLITVLQNQAPQGLFSIFPANNRSNSMTVEEGNVTVYLKVSRNNG
LNMSVSVEWETLSGTALGIRGPYSVLSVLQSFGNVTGSSWCFFNHADIQYAVLLRKSRMS
TSTVPFSTVYEWRGVFVPIQDFTIETPFGCVGFAINGSQFLAITFGNGRPTNTSIFTFSPKQG
LSLKQTIMVAETTGVKHFSASKQDYLVIASGMGSTDALQDFTIETPFGCVGFAINGSQFLAI
TFGNGRPTNTSIFTFSPKQGLSLKQTIMVAETTGVKHFSASKQDYLVIASGMGSTDALQVY
KWNNGLFALHQKLPVLWTSGLTVFTRGESIYLVVSLASPVQNSLIYTWSMNQFGTPQEVP
VKGATCVESLASGADVYIILSGNTSVDIFLWESGQTFFKKIQTIPYEGGTNMIHAFMPPSGL
IHFLVSGENASALYSLNSGWNQFSLVLLAPASKHLTSAIVRSMNATKTIIAQAGDLSSQIFEL
TSISNQSDFIPSSGELKFEPGEREAVIAISILDDLIPEEDESFTIRLKNPKGGAEIGANSYVRIII
PTNDDAHGVIGFAQSSLIKQAEEMEQDNLVSFTIERLYGNYGRVAVEWFANGSISDISPSSG
VDDLPELYEGFLLNITSVAILNASTSREQPTVKRPGTEIAEIIILENDEPKGVFQFNVSKDING
AVNAYEVPPPRNVLILPIIRRAGKFGPVTVYWEAKPMTASQEDFYPSSGNLTFSDGQGSGV
IQIFIVDDTFVEFSEAFTVVLKNLTNGAKFGNETTVLVNIPPNDSPIGVFGFEEKTVRVSEAK
YTGDLNGEVTLTVARSPGGRGAVKIIWVLEEAAQYDLRPLNGTLIFNESDTRKSFTLQAIQ
DNLLEGEETYTIQLVSGDYSVISPVDGTAFVVITGDTGASGVVGIAPSSRNIIIGEPLGNYNG
TAYISLVRGPGIFGEITVYWNITPAYQNEFLEVTGSLIMRDRQSAAVVIIQAMDDNSPEEKS
YYRFQLNKVSEGATINESSRSANITMASSDLPYGNFQFLQTLLQISEDENWANVTVVRSGG
IFGTVLLKYQTENGSALSDLDYAHTMGQLVFNPNETIKHVSIKIHNDILPEGPEDFFVVIQE
VKLLGRDYDHTIRENGLQLDQPPIIGNNSLARIIIQKNDNAEGVIQFDPDFVDVQVEEDVG
TLSIPVMRLYGTYGYVTAEFVSRGISALANGIDYTVHNTSVTFQHGQQLSLINISITDDEES
ELAEQFEIRLVAASGGAILGRHLVSTVTIAKSDSLSGAVRFLNESQITLPNPNVSLSLLLALE
RTGGTLGASQILWKILGPNSKETIKANNDDFSEPLNGSVHFNDGEGGIKTISLTILPHGEIEV

EEKFIIILSVGSGDAVIDRKAGNVTLTIKKFGDPNGIIQFAPKSLLLKNYTEPGAAEGPLNITF
PLNRVQGTMGNLTIHWELRSTSVIQGDFAALMGWVSIPEKKNTAEIIIQLLPDDEPELDEIC
MVHLTYVEGGADLDNDKRVAQFTVLGNDDPHGVFVVFPAGQSIIVTNDSSRYIQINVSRL
AGTFGNVSVAYQISAGGGEQQFFTEKIVGNVLIKDGATYGLTTVPISSQVFLSPGFNFTVEL
TNVTLMGQSSYGRPQIGKGQKPTSVSVPKDAANSEVGFEAIAFRLSNITLGEGKAVISRTG
LYGSVKVGWSVGYPMGFKPINIHLGKITPESGTVTFMQGEDRKTISVLLEANSSLPEAFAL
QLTQLSSNVSGGVRLKPGFTVSEFEPMGVFQFSSNSKHVTVAEDVKTVRVHVQRLYGFQG
NITVLKFQTIPGSAKAIDDYVPIYNGELVFQKYQTSAVIEVSIVNDDIYEADEIFYLNLTSVE
NIGVNHSKSLLNVDSHLATITILANDLASGFVSIGPVTTYVEEDTNNSALNTVYLHVKRTR
GFSGVVHFTITTFGKINATKGLKGLPFENSHKSTNLSWATEVMDFEEKSMLITMLDGEME
HNISVKIHDDEEPEGSEVFYIFIKDPRGGVQIIDGRDESGYASYATIIIRGSDMQNGILGFAME
SQQGLLLDEDSVKHTVQLLVLRQINRAFEDVKVFWRATFNKTAVELYKDEVNLVNELQV
VSGHTVCSAGQTQCIITVEAKPDKVPEFETYFYVELHQVSAGASINSSSRFAKIILLESDAP
YGLIYFAVGSREAVAHKKTTLISLQISRESSTAQSISVGFTMQELTVSEVIGHTTISPAVAGSD
FVKSEGILTFEVGQKTVVLDIILTPETGSINPFPKRFQVLLVNPSGSAKVDPLYGIANITILSD
PTSQSVWGLIDQLHQPYEETVFIRVLQTLNNKASSEVSEEQLGAMLNIMEKIANEGEIQVL
TNDSRALFYDTLCSLASPERLDTRGYSPLIDVAEKYAFSLLSGVECGSSGERGKTILDTCPH
ISISSYHWYPQEINGKTFQGKDGDSIQVPETFLTVSPISAAEEGCAFIHFTEYSSQQWFLTSG
TETILNNKVLSVSLKGRPSYRIADSNEVVYRIHAAGDRIIPKKSRCLLWNQGLERWMSDRE
LCRVVSDTTDYVECACSHMSSYAVLAHTDRLSSYNEAFFFAGFICISGFTLAILSQLFCTRS
TMFAAKLLTHFRVSCLGTQVSERERVILGAFPGVLIIADFACAFELETNHREEDAAMSGAV
SGASQFEPAAAATGTEENRGGMEISFLAAAYISQELSDESCSALASVTHYFYLSQFFWMLI
QAVNFWYVLVMNDENAERRHLIFFIVGWGLPAVVVILQLVILRSIYHKSLPEIYGLVHGDIL
DVSSAKPELLANHSYFGRMTSRQSVNNIECGRCFTPNMYAALSTAVLLPLVCLVVVLVVFI
HAYQVAQQWKSYDDVFRGRPNASEIPLILYLFALTVLTWLWGGLHLAYRDLWMLVLFIIF
NSLQGLYVFVVYFILHNQLCCPVKASYSIEGDGHRSQASAFFTPSSGMPPAGGEISKSTQN
LISAMEEISDWERSGSQEIKQDPQNGNVFTTSGGYGNGSLVADEELQEFDDLIFVLKAGSG
LNISDTESGHGSQDGGSMANSQIVELRRIPIADTHL*
>jgi|Xentr4|461178|estExt_fgenesh1_pg....
MSQPGDIVIGAVVPVHVDKMYPATSFTETPSSAICKTFRFENYQRIQALRFAVEEINKDPDL
LPNITIGYQILDSCTVLQRALSGILQLLTGHKEPVPNYRCQQAMPLAAIIGHSISTYSMMLA
HILGLYRYPQISHFSTSSLLSDRTQFHSFFRTVPSDAFQCQGLAQLVLHFGWTWVGLVATD
NDYGQQGIQMIKRDLLNAGACVAFTENIILNQQNRNAPRIARVIKESAAKVVVFFSTDIDL
LFVLDEMLRQNISEKILVASEAWATSAFLAVDKYSEILSGTIGFALHSGTIPGFKDFLNSIHFS
LSLDDELLKIFWEQAFGCQFQDQANTTVTSEYSVKICTGSEDLGNIQNSYNDVSSLRVTYN
VYTAVRVVAKALHDLSRCNEGEGPFSKGTCAYIGRFEPWQLLHYMKQVRLKTGSGTEFAF
HENGDPTAVYDIINWQFSPLGKIQQVKVGSYDTGATAGDIFTVNISALHWSTGQKEVPRSI
CSQSCPPGYSKAVLPGKPVCCFECFPCPHGEISNKTDSPYCFKCPWDMWPNIKRDSCLPKP
REFLSYEDSLGETLATTSISSSLVPFAILVLFVHYKMTPIVRANNYLLSCLLLVSLSLCFLCSL
FFIGFPHPEMCRLRQVTFGMVFALCISCILAKTFMVVVAFKATKPNSSLKKWTRPRISYLIV
VLCILIQTCLCTMWLLLSPPFFEENIRTKVGVVIVECNDGSSVAFWCMLGYLGLLASVSFF
VAFLARRLPDSFNEAKFITFSMGTFLSVWISFIPATMSARGKYTVAMEIFAILSSTWALVVC
MFVPKCFIILFRPNMNSREHLMGKEKRGSEKQACKQNNTSGIH*
>jgi|Xentr4|462280|C_scaffold_5000008

MEKQRSLCNNAGCKDHPNSTLFNSSSSQIDPTADPYVRNEALAKIEIAILAIIFVAAVLGNC
SVLIGLYKSKKKTSRMHLFIKHLSLADLAVAFFQVLPQLCWEVTYRFRGPDILCRIVKHLQ
VFGMFASAYMLVVMTADRYIAICHPLKTLQQPTKRSYVMIISAWIISFLLSIPQYFIFSFSPV
NGSEVYDCWAHFIQPWGARAYITWMTASIFVVPVAILTTCYGFICYNIWRNIQCKTKREEN
EGRKSHGLLSTSVSSVKTISRAKIRTVKMTLVIVTAYILCWAPFFIVQMRSVWDKNFEWTD
SEDIATTVTALLGSLNSCCNPWIYMFFSGHLLQDFIHSFPCFQKIKQTFSKEDSDSSTRRQTS
FTRIQTRSPTNSTHTLKESPKSSKSIKFLPIQT*
>jgi|Xentr4|462318|C_scaffold_6000003
MVTPGSRMQTHWLFLVITILQCTEAFSRAALPFGLVRRELACEGYPIDLRCPGSDVIMIESA
NYGRTDDKICDADPFQMENTDCHLPDAYKIMSQRCNNRTQCVVVTGSDVFPDPCPGTYK
YLEVQYECVPYIFVCPGILKAVVDSPYIFEAEQKAGAWCKDPLQAADKIYFMPWTPYRTD
TLIEYASLQDFQNGRQTTTYKLPNRVDGTGFVVYDGAVFFNKERTRNIVKFDLRTRIKSGE
AIINYANYHDTSPYRWGGKTDIDLAVDENGLWVIYATEQNNGMIVISQLNPYTLRFEGTW
ETTYDKRAASNAFMICGVLYVVRSVYQDNESETGKNAIDYIFNTKLKRGESVDIPFPNQY
QYIAAVDYNPRDNQLYVWNNNFILRYSLEFNLQDLAQVPTTAVAETPPVDLIETTTSTTTTT
TTSQKALISTTLATGTKEGSRGPRPPPAVSTTKNPPLTSVYPLPEKYCEAKEARGISWPQTH
RGTIVERPCPKGTRGIASYLCLISTGTWSLRGPDLSNCTSHWVNQLAQKIRSGENAASLAN
ELAKHTKGPIFAGDVSSSVRLMEQLVDILDAQLQELKPNEKDSVGRSYNKLQKREKTCRA
YLKAIVDTVDNLLRFDALQSWRDMNSSEQAHAATMLLDTLEEGAFVLADNLAEPTRVSM
PTENIILEVAVLSTEGQVQELKFPQGSKGGNLIQLSANTVKQNSRNGLAKLVFILYKSLGQF
LSTENATIKLGADLAGRNTTIAVNSHVIAASINKESSRVYLTDPVFFTLEHIDPNNYFNANC
SFWNYSERTMMGYWSTQGCRLVDTNKTHTTCACSHLTNFAILMAHREIVYPNRVQELLL
TVITWVGIVISLVCLAICIFTFCFFRGLQSDRNTIHKNLCINLFVAEFLFLIGIDKTDYQVRLL
TLFRTIISNYLSFSSIVQGYMIHFHFSINIKGKYTGYLHSVKIDKPLHEVQYDRCWLRIDNNF
IWSFIGPVTFIILLNLVFLVITLCKMVKHSNTLKPDSSRLENINNYRVCDGYYNTDLPGYED
NKPFIKSWVLGAFALLCLLGLTWSFGLLFINEETVVMTYLFTVFNAFQGMFIFIFHCALQK
KVRKEYSKCFRHSYCCGGLPTESPHSAVKASTARTSARYSSGTQSRIRRMWNDTVRKQSE
SSFISGDINSTSTLNQGMTGNYLLTNPLLRPHGTNNPYNTLLAETVVCNTPSAPVFNSPGHS
LNNARDSSAMDTLPLNGNFNNSYSLRNGDYSDGVQVVDCGLSLNDTAFEKMIISELVHN
NLRSSSKIHNLERTLPVKTVIGGSSSEDDAIVADASSLMHGDNPGLELHHKELEAPLIPQRT
HSLLYHPQKKVKTEGTDSYVSQLTAEAEDNLQSPNRDSLYTSMPNLRDSPYPESSPDIEED
LSPSRKSENEDIYYKSMPNLGAGHHLQMYYQISRGNSDGYIIPINKEGCIPEGDVREGQMQ
LVTSL*
>jgi|Xentr4|462448|C_scaffold_9000010
MDIENLTDYNCTSEHLQKGITPKILISLTLSILTLMTTAINSLVIAAIIVTRKLHHPANYLICSL
AVTDFLVAVLVMPFSIMYIMKETWIMGQAICDIWLSVDITCCTCSILHLSAIALDRYRAITDA
VEYARKRTPKHAAFMIAVVWIISIFISMPPLFWRHQALNKEDECIIKHDHIVFTIYSTFGAFY
IPLALILILYYKIYKAAKTLYHKRSVSRVEREQNGQVLLDATTTLCIAEKSFSDHSTDFDKIH
ITVRNARVEMKQEKAYRKQKISSTRERKAATTLGLILGAFVICWLPFFVKEVIVNICESCYI
SDDMSNFLTWLGYLNSLINPLIYTIFNEDFKKAFQKLMRCRHYL*
>jgi|Xentr4|462494|C_scaffold_11000007
MSSMVPWICWIGGFAFLLVICTSAGVQINETFYMDKCKKTTTCEPLKYNVCLGSVLPYAL
TSTVLAEDSSTQEEIHDKLLLWSGLRNAPRCWDAIRPLLCAVYMPKCEGGKVELPSQSLC
QTTRVPCAIVARERGWPDFLKCTTDHFPDGCSNEIQTVKFNSSGQCEAPLVRTDNPKSWY

EDVEGCGIQCQNPLFTKKEHREMHVYIAIFSSVTIFCTFFTLATFLSDWKNSNRYPAVILFY
VNACFFVGSIGWLAQFMDGARDEIVCRGDGTMRLGEPTDKHLLSLKKSTVSTRNVGFFQ
MFSLIVVLVRAALCPYISSFCQHPRHLSLYKRVCSTLPCIYVDGDSVSGICFVGYKNYHYR
AGFVLAPIGLVLIVGGYFLIRGVMTLFSIKSNHPGLLSEKAASKINETMLRLGIFGFLAFGF
VLITFGCHFYDFFNQAEWERSFREYVLCEANVTIAEQTNKPIPECEIKNRPSLLVEKINLFA
MFGTGISMSTWVWTKATIIIWKRAWCRILGRSDDEPKRIKKSKMIAKAFSKRKELLNNPE
KELSFSLHTVSHEGPVAGLNFDINEPSADMSSAWAQHVTKMVARRGAILPQDVSVTPVATP
VPPEEREQWLIEGDLPQGMVKKMSKKKRRRKKKEIQPLEENTGMDHFHSRANTAVPRLP
KLPLKTGLVTRSKDPQEIDETLPGSYREFKRLPNRQEKLYPVESVNNNLGYTRYPQSNMM
SHPFAADKMPHTDQSRVTYFPVPKHDGVFQYSNSNSYSNHMDLTSYSGRTQARRAGIPPM
HSRANLMDAELMDTDSDF*
>jgi|Xentr4|462936|C_scaffold_24000014
MQNRSMVTEIFLLGFQHLNNFKTLTFALILFVHILTVSENALVIALVTVSRGLQSPMFFFLQ
QLSFSDLLLSVLIVPTLLRTVMNEGAKIPLIGCIVQLYFFSITEALQCFLLNVMSYDRYLAIC
NPLRYSSLMNHTFCFRLIAISWLLALCVTPVAQIPAATQEFCNQNTINHFFCDFFPLLELSCS
DTSLVRILTITVSIPAIVFPFLLVVISYICIAHEILKIVSSIGRQKAFSTCSSHLAVVSIFYGTLIGI
YVIPTRNQSQTISKLLSLLYTVVTPFINPMIYSLKSADMKNALKNIIN*
>jgi|Xentr4|462941|C_scaffold_24000019
MCSDNETEVTDILLLGFHDINNYKSPLFILFLLIYSVILCGNILIFSLVTLMENLQMPMFFFL
KHLGLADVLLTTSVVPMMLNIILNDELIITLVGCIGQLYIFGLSGFVQCFLLAVMSYDRYLA
ICYPLHYISIMSPNVCLLLVAGCWLLVFILITTDIIMVWQLQFCGFNQIDHFFCDFGPVVALS
TSDTSVLTLFDFIISIPMMFVPFVFIIGTYVCIFITIFRMHSTVGRQKMFSTCSSHLTVVCTYY
GTLIIVYMGPSAEYSVNMKKFLSLLYIVIAPFMNPIIYSLRNKEIRETLKKYIKMV*
>jgi|Xentr4|462950|C_scaffold_24000028
MRMQNRSMVSEIFLLGFQHLDNFKILIFSLILLIHILTVCENFLVMALAQISQNLQSPMFFFL
QQLSVSDLLQSVVIVPTLLSTVMNEGAKIPLIGCIVQLYFFGVTEALQCFLLTVMSYDRYL
AICNPLRYSSLMSHRVCVKLIAISWLLALSVTSVTQIPVATQEFCNQNTINHFFCDFFPLLEL
SCSDTSLARILTIALSTPVILFPFMLIIGSYICIAHEILKIVSSIGRQKAFSTCSSHLAVVSIFYGT
LIVTYMVPRRNQSQTVSKLLSLLYTVVTPFINPMIYSLKSADMKNALKNVLQ*
>jgi|Xentr4|462958|C_scaffold_24000036
MYKVNQTSDISFLLLGFQNSQIINEFLFVLFLWIYIVTLFGNLLIIILVITVSALRSPMYALLS
QLSLADVLLSTSITPNFLRLLLNGGGTISATGCITQFYFYCVSGSSEYYLLTAMAYDRYLAI
CSPLHYASIMGFRLCLYMSLCSWGLALILSVIMTLLILNLQFCGPFFIDHYFCDLSPLLELSC
TDYKLVVKLTQMILSIPFILLPFCFIIYTYVSIGLAILRISSTEGRHKAFSTCSSHLIVVCMYY
GTLIIVYMVPSKGHHFNIDKMLSLLYTVGTPFFNPVVYSLRNKDIKVVLLKHLRNRVSLGD
LYALRNVIKLLC*
>jgi|Xentr4|462961|C_scaffold_24000039
MHKVNQTSGISFLLLGFQNSQIINEFLFVLFLWIYIVTLFGNLLIIILVITVSALRSPMYALLS
QLSLADVLLSTSITPNFLWLLLNGGGTISATGCITQFYFYCVSTVSEFYLLTAMAYDRYLAI
CSPLHYVSIMDFRLCLYMSLSSWGLALIISLVMNLLTFNLQFCGPFVIDHYFCDFTPIIKLSC
TDYKALELTDIILGIFITLLPFCFIIYSYVAIGLAILRISSTEGRHKAFSTCSSHLIVVCMYYGT
VIIIYMVPSKAHNFNINKILSLLYTVGTPFSNPIVYSLRNNEIKNALWKYILQSL*
>jgi|Xentr4|462962|C_scaffold_24000040
MALENQTAVQEFLLVAFQNIDKLKFLLFFSVFTVYLLALFGNILIIILVSTDPKLHSPMYFFL

CHLSTSEILFITSIVPNMLYVILSKVGTISFGSCVTQYYLFSSTTSVECLLLAVMSYDRYLAIC
VPLHYSAIMNKRLCLYLVALCWVTGFGLSLIALAFMSHMDFCGSGIIDHFFCDLAPILNLS
CSDTSFLEMEDFVLCFVFLIFPFVIIIVTYIRIILTILRIKSTTGKNKAFSTCSSHLTVVCTYYG
TLIVIYMMPSTEYSSIGNKTSAILYTVVTPMLNPIIYSLRNNDIRISLRNISKRLQL*
>jgi|Xentr4|462973|C_scaffold_24000051
MQTNNGSMVSEIFLLGFQHLNNFKILFFSLILLIHILTVNENVLVIALVTVSRGLQSPMFFFL
QQLSFSDLLQSVVIVPTLLRTVMNEGAKIPLIGCIVQLYLFGVTEALQCFLLTVMSYDRYLA
ICNPLRYSSLMSHRVCVKLIAMSWLLALSVTPVLVISAATQEFCNQNTINHFFCDYFPLLEL
SCSDTSLLRILAIALTVPVVLSPFMLIIGSYICIAHEILKIVSSIGRQKAFSTCSSHLAVVSIFYG
TLIGIYVVPTRNQSQTISKLLSLLYTVVTPCINPMIYSLKSADMKNALKIIMK*
>jgi|Xentr4|463262|C_scaffold_31000048
MWENQSTLTYFFIKGISDLPHLQAAIFLVVLSIYLFTLVGNMLILTLVCLDTQLHTPMYFFL
AQLSILDMSCSTIALHKELAIYITGDHTVSILTCFAQLYIFSCLECVELLIITAMSYDRFVAICR
PLHYPTIMRHSTCANFVILCWLLGFLEVIPYVVLLSNYTCYRSNVINHFFCDLVPLMKLSC
NDTTDLEHLMLIEGSFLLTGFTPFLLTFISYIYIIATILQITSNIGRRKAFYTCSSHLTVVIILYL
SLFCQYLRPSSTDTLDSNKVFSLFNTAAVPVLNPFIYSLKNKDVKFAFRRQLKSLR*
>jgi|Xentr4|463305|C_scaffold_34000003
MPMNNSTYVGIFQILAFSDSSEKHPFLFSIFCLIYLTGVIWNLLMIIVISKNRCLHTPMYLFL
AHLSFVDICYPSVTLPKLMDILLSGDHSISFVQCFTQMFFFIFMGGTEIVLLSSMAYDRYVAI
CQPLHYHFIMDRKKCLLLLGVTWISGCGNALFLLILASKLLFCRSNKIYQFFCDVKALLSIS
CSNEVLQVMIFIEALLYGLCPFLLSLSSYMKIINIILQIKSTDGRKKAFSTCTSHLLVLILYYA
TILCMYMRPPTEESAVLDHIFSVLYSTVTPMLNPLIYSLRNKDVKTALMKLVTIKNLKN*
>jgi|Xentr4|463345|C_scaffold_34000043
MKENIENKSLTDFHLLAFSSSAGTQILFLVSVIIMYLLAIFGNLITLIICLVSKLHRPMYFFLC
YLALLDIIYVSTTLPKLMAITITGDNSISLNGCIIQMFMFATCVMGEFFLLAFMAYDRYVAIC
IPLRYSVIMNKRTCKVFANLSWLCSSLNAVIYCIIILNLPFCQSHDINNIYCGLKTIVKLSCG
DITQIETFMSIESIFLGLLPFMLIITSYIFIINAILKIPTSTGRAKTFSSCSSHITTVIIFYVIALMM
YMKPESEYSQEQEKVLTLLYATVIPILNPLVYSLRNTEILQALNKIMHNILLNCKVNQRQSH
*
>jgi|Xentr4|463348|C_scaffold_34000046
MHFPSSNQSLNIIFLLEGLTKDPTLEAVLFIMFLVIYIFTIFGNGGLIFLIIKIPALHTPMYFFLQ
HLSFIDICYTSVIIPRTLSNFLAKEKTITLLACAVQIQMFMFIICVTTEFFLLTFMAYDRYVAIC
IPLRYLIIMNKRKCIQFAFLSWFFGLVNSAFYSIVMCTLPFCTMREINHFYCDLKAMAKLSC
SDITLLETLMSLESVFLGLFPFALILVSYVCIICTIVKMRTSTGRAKTFSSCSSHLTVVIIFCGT
VTSTYMKQESENSQELEKLMSLLYSTIVPVLNPLIYSLRNKDVLKAVMNIIKLKSNV*
>jgi|Xentr4|463354|C_scaffold_34000052
MNSTSQKDFHLLAFSLFVEDQPLLFMGLLIIYLLALFGNLLIIVLVCLVPQLHTPMYFFLCN
LAAQDIISVSAFLPKLMAITITGDTSISFPSCITQIFLFAFCTDTDFFLLATMAYDRYVAICIPL
RYYLIMNARVCLLLVAIAWILYIPNSLCYSLLFSYLSFCKSRELNHFFCEPKILLEISCSDTSHI
KQLMLVEIPFVGILPFVLILTSYVYIIATIIKLRSSAARLKAFSSCSSHLTVVLLFCGTSIGIYIK
PDSENSQEQEKFLSLLYIGFVPLLNPLVYSLRNQQVRSAAKILLSKYVPGTQL*
>jgi|Xentr4|463356|C_scaffold_34000054
MDNAINITIPRYFHLIAFSNSEEKHYIILIGLLPMYLLAVLGNMLVVVLVCLVPQLHTPMYFF
LCNLAAQDIISVSAFLPKLMAITITGDTSISFPGCITQLFLFITCTDGDFLLLAIMAYDRYVAI

CIPLRYHLIMNPRFCILLVAMSWIVCAANAMCFSFFISHLSFCRLLDINHIFCETISMLKLSCS
DTTHIQTLIAVDAPLIGILPVGLILISYVYIIYTILKMRTSAARPKTFSSCSSHLTVVLLFSGTC
LSLYMKPESGNSQEVDKLLSLMYLGFIPMLNPLVYSLRNRKVQSAAKIVFSKYIIKDPYKG
SSSGRQ*
>jgi|Xentr4|463358|C_scaffold_34000056
MYFFLCNLSCVDIIFTTITLPKLLDILLFGNNSMSAMQCYSQMYFYMCVACTEDTLLSFMA
YDRYVAICRPLHYHQMMNKKKYVLLIVTTWIAGCFNSLFLTLWVSQLTLCNPGKLYHFFC
DVKAVLKISCSTITFRIIIYADTLLFGFCPFLLSVISYIKVIRIILSIRCSNGRRKAFSTCSSHLIV
LAMFYGSGMCTVMSPYSAHSEGLDQGFSVLYAAVTPMLNPIIYSLRNKEVKTALFRIAGV
TK*
>jgi|Xentr4|463397|C_scaffold_35000023
MANETSDTATYSAIYIGVETVIGISAVLGNILVIWAVRLNPSLQNTTFYFIVSLALADLAVGF
LVMPLAIVLSLGMHFHFHACLFICCLIIILTNASILSLLAIAVDRYLRIKIPTRYRIVITSRRICF
SICIVWIISFLVGMVPMFGWNNRSSLKEEHQHYLNCTFENVMSMEYMVYFNIFGWVYLPL
MIMLILYIEIFYLIKKQLNQSNSNYLRRGVFYGKEYKTAKSLALVLLLFALSWLPLAILNCV
QFYNPNVKKLGLYQPTIFLFILLSHANSAMNPIIYAFKIKKFKEAYIHILRTILMQKSVVPDA
ANAEHTMEEISKD*
>jgi|Xentr4|463915|C_scaffold_55000017
MNTNFSCNFSHVDSIMNNVELGIYIPTFILGCILNIFALWIFFFSIKKWTEASIYLINLAILDLF
LLLSLPFKMYSSIQIEQIVDRHLCTFAEILYFANMYGSIYIITFISLDRYIAIKHIFWAKRLRSP
KKTTSICFFIWVFVWIVGGSIFKKEDNNRCFHKMSNKIWSPSIIICLELFGFLMPMIVIMGCS
IQIVRKLGDHRGISEQDHGQKTTVRRIIISNLVVFLLSFLPSHLGIFLQFLVKQDIIANCSHKQ
SIILFLRLSLCLANVNTCLDAACYYFAVKEFRKMSVNSHSIIRRTMSLQQSRSE*
>jgi|Xentr4|464115|C_scaffold_64000004
MFRLSSLFTSAFVWQLLAVTLALEMGLYDVERGREAKCEPIQIPMCQGIGYNMTRMPNY
VGHESQEEAAAKLQEFAPLVEYGCHIHLRFFLCSLYAPMCTEQVSTSIPACKPMCEAARQK
CAPIMESFQYVWPESLDCDRLPSKNDPHALCMEAPENATNGDPPTNGHGMLPVAPRPPRP
SGSGIGISSRCANPDKFMYVERSGVCAPRCIPGVDVYWSSGDKDFALVWMAAWSGLCFIS
TAFTVFTFLLHPQRFQYPEKPIIFLSMCYNVYSTAFLIRAAAGAPSIACDREGGAPYLIREGL
ESSGCTLVFLILYYFGMASSLWWVVLTLTWFLAASKKWGHEAIESHGSYFHLAAWGIPAIK
TIIILTMRKVGGDELTGLCYVGGSDPSALTGFVLVPLSCYLVTGTSFLLTGFVALFHIRRVM
KTGGTNTEKLEKLMVKIGVFSILYTVPATCIIVCCFYERLNLAHWDSRAREESCRTVPGSA
RPDCNLPHSIPSVAVFMLKIFMSLAVGITSGVWVWSSKTLQAWQGILCQRRLVEVGARTRG
KPQGGLGVPCSLGSCPYKPPPVTLQVAKTDPFMDSPTHV*
>jgi|Xentr4|464149|C_scaffold_66000004
MISHRPFFRYYRHYLAFMFAIDEINKNPQILPNISLGYQIYDSCGAEVRAGSGTLGILSGTQ
RTVPNYNCWNNGKLLGFLGDFSTSTTYVIAQIVRLFKYPQISYSSTDPVFNDRTQFPSFYRT
IPNEEAEMDGIVQILKHFGWKWVGLIISDDDTGYRARERISKGLSDIGGCLAFHILINDTFC
LDDFYESTVVETIERTSANVIVLFISTKYTFAFANLFSLNQMPRKVWITSSFFPNTFYFRKK
KIETTFNGSLSVLIQEGEIPGFEEFFSTFSPNKNPKNELTQTTWEGLFHCYFLDTPSLIRTVPG
AKTCTGNATFSEADVSVYGNHNYRVTYRVYTAVYALARALHNLYSAQPPANHWGKLESL
KRNIKPWQINQFVRNVTFTTFPNDTHSFNEHGDPPARYDIIKWLFSPGHHIVSRKVGSFNV
SDHETQFYINSSADLWGPHFNMTPQSLCNAPCAPGHRKSKRKGAPSCCYDCVPCVDGEM
SNTSDVPSCFRCPGYEMSNKQRTACVPKMINYLSYEETLGASLASIALMLFLTTSAVQGVF

VKYWETPIVRANNQNLSCLLLISLMLCFLCTLLFIGRPTQICCLLRQVTFGIVFTISVSSVLA
KTLTVIIAFNATKPGSKLKKYVGTQLAIVLVTICSLGSTVISAVWMASHPPFLEADTVSEMD
TIILKCNEGSVTFFFCIIGYIGTLALLSFIAAFLAKDFPDRFNEAKNITFSMLGFCSVWGAFV
PAYLSSKGSRMVAVEIFAILASSAGLLGCIFAPKCYIIFHRPELNTRVAISCSN*
>jgi|Xentr4|464368|C_scaffold_74000008
MDGTPNSSLRSADSLLNSTLSGLGDNGSQIPMICPVGGMGNWSRLEGNQSYLCNQSETFP
EDSDSIIIAIIITALYSMICVMGLFGNVLVMYVIVRYTKMKTATNIYIFNLALADALATSTLPF
QSVNYLMGTWPFGNIVCKIVISIDYYNMFTSIFTLTTMSVDRYIAVCHPVKALDFRTPRNA
KIVNVCNWILSSAIGLPVMFMATTKSERGSTDCALLFPHPSWYWDNLLKICVFIFAFIMPV
LIITVCYGMMILRLKSVRMLSGSKEKDRNLRRITRMVLVVVAVFIVCWTPIHIYVIIKALINI
PPSLFQTVTWHVCIALGYTNSCLNPVLYAFLDENFKRCFREFCIPTSSTIEQQNSTRMRHNT
RDRASTANTVDRTNHQV*
>jgi|Xentr4|464410|C_scaffold_75000018
MVTSYPLPEGFTETEVFAIGTTLLVEALLGLLLNGLTLLSFYKIRELRTPSNLFIISLAVADTG
LCLNAFVAAFSSFLRYWPYGSEGCQIHGFQGFVAALSSIGSCAAIAWDRYHQYCTRSKLH
WSTAVSVVFFIWGFSAFWSAMPLFGWGEYDYEPLRTCCTLDYSKGDRNYISYLFTMAFFE
FLVPLFILMTAYQSIYQKMKKSGQIRFNTSMPVKSLVFCWGPYCLLCFYAVIQDATILSPKL
RMVSVRTLCDCLYLDKEST*
>jgi|Xentr4|464511|C_scaffold_80000015
MFIESQNSITDPECRMGFFPCGNVTICLPRTFHCDGVKDCENGADEEYCGDNSGWANIFYE
VHGKTNDVIEPQECCLSRFPESCECKDTELECVDVNLEAVPYVSSNVTLLSLKRNKIHALP
DEVFIGYHDLTKLFLQHNCLRNISQKAFFGLYHLQRLYLSNNCISYLQQGIFSHLRELKWLI
LDENPIIRISQDIFAGLTSLFFLDFEGNRIKTLESSSFVTCNELTVLFLRGNQISLVKENIFSSLR
SLAEMQFESLQHLQSLDLEKIEIPNISTRMFQPMKNLSHIYFKKFRYCSYAPHVRVCTPLTD
GISSFENLLANTILRVFVWVIACITCFGNIFVIGMRSCIQSENKTHTMSIKVLCCADCLMGIY
LFFIGVFDVKYRGQYKKYALLWMESLQCRSLGFLAMLSTEVSVLLLTFLTLEKYLAIVFPF
SNIRPGKRQTLIILISLWAVGFIIAIVPFWNEDFFGNYYGKNGVCFPLYPDQTEEAGGQGYSL
GVFLGVNLLAFIIIVFSYISMFCSIQKTALRTSEVNSHIHTDVAVANRFFFIVFSDAVCWIPVF
LLKILSLFRVEIPGTVTSWIVIFILPINSALNPILYTLTTSFFKEKLKQLLHRQRRRSVFRNERK
SLSTSLVWTEETLVQHPSLKLGFLHKKSLDENIIKTT*
>jgi|Xentr4|464595|C_scaffold_83000013
MESPVQIFREEEAIIPERAPVGSTCSSSCAPACPSSCFPNTSWLLGWDDHDNVSAYPDLPLN
EGNHTSISPTISVIITAVYSMVFVVGLVGNALVMFVIIRYTKMKTATNIYIFNLALADALVTT
TMPFQSTSFLMNSWPFGDVLCKIVVSIDYYNMFTSIFTLTMMSVDRYIAVCHPVKALDFRT
PLKAKCINICIWMLSSSVGISAIVLGGTKISDGSTECALQFPTHYWYWDTVMKMCVFIFAFI
IPVFIITICYTLMILRLKSVRLLSGSREKDRNLRRITRLVLVVVAVFIVCWTPIHIFVLVEALV
DVPQSIAVVSIYYFCIALGYTNSSLNPILYAFLDENFKRCFKDFCFPSKHRLDRQPNSRVGN
TVQDPACNRHGSQKPV*
>jgi|Xentr4|464801|C_scaffold_91000027
MEFILTDFSEEPILQILSFVLFLFTYLVATVGNAMIILIIIHVPNLRTPMYFFILNLGILDVCYIS
TTVPNMLGNSLKQWKRISFGGCVVQMYIFLSMAATESFLLVAMSYDRYVAICNPLRYTVI
MNRRLCLQLAATSWMIGKHFCNTNKISYFYCDLPPLLSISCQDTSVNELLLLSIGIFIGWTP
FLCIIVSYVYILFTIMKITSTESRQKAFSTCISHITVVVLYFGSVNFSYVRPISTYSLEKDRLIS
VLYSVISPMLNPLIYTLKNQDVKKAIGKQFIPHYRR*

>jgi|Xentr4|464808|C_scaffold_91000034
MNHTNNTVVNEFILLGFPSLQKFYPVLFLVFLLIYLFTVTGNILIFLTIILNCNLQIPMFLFIN
HLSAMEILYTSVIIPKMLSTFVTSSNRIPFNSCMIQLYLFSSLGASECYLLTVMAYDRFLAIC
KPLHYSALMTNLKSFQLASGSWVGGFLSPVLPVTLISKLVFCGPNSINYFYCDAQPLLRLS
CSRTYLTEATITILASGLIFSSFLLTVISYIFIILTILRIPTSSGRKKTFSTCASHLIVVIMYYGTIT
CIYMQPMSSFPLEINKVLSLLYTVVTPMLNPIIYSLRNKEIKKALWRTLQRVKCNTS*
>jgi|Xentr4|464809|C_scaffold_91000035
MEETGNMTRGTFILLGFPSCRPLQILLFFIFFLAYILTIMEHLFVIVILWTNSRLHKPMYFFLG
HLSFLELWYTTVTIPKLLSVLLVESGQISLPACMSQLFFFISLVCTECVLLAVMAFDRFVAIC
KPMHYITIMRWRLCFILVSGSWLTGFLISFIKVYYIARLKFCHSGVINHFFCDISPLLNMSCT
DIKVTELVDFILALIILLLPLLLTVISYVCILITIIQMPTSSGRYKAFSTCASHLTVVIIFYTATLF
MYARPSRAQALNYTKFVSVIYTVITPFLNPIIYCLRNKDVKHAIFKLFQPKNMAHGQFFPG
S*
>jgi|Xentr4|464953|C_scaffold_99000013
MANSTYPYADVPSVGHPESPDDILYTRFYRLSLALMVINILIFLVGIVLNSLAIYVFCFRTKT
KTTSVIYTINLVVTDLLVGLSLPTRIVMYYSAGNCKYCYFVHSFTYFVNMYCSILFLTCICV
DRFMAIVQVEASRRWRNPNYAKLICICIWIFAIVVTFTILTTTINHPSCCLFQLFSLTAFEYFV
PLIIITFYTLRIMWALSRSTLMNQSRERRMKAVQLLITVLIIFTVCFTPFHVSQVILCINSSISL
DVNIVVYHVTVTLSSLNSCMDPIVYCFVTNNFQSTMRSIFRKYQPEPISVDIVNLHKTPKG
SGTITTISNAIVSLPLQSSTLI*
>jgi|Xentr4|465823|C_scaffold_149000017
MAGAWLVLWGLCSAAILLTSSTQGLSRAALPFGLMRRELACEGYPIELRCPGSDVIMVEN
ANYGRTDDKICDADPFQMENVQCYLPDAFKIMSQRCNNRTQCVVVAGSDSFPDPCPGTY
KYLEVQYDCVPYKVEQKVFVCPGTLQEILEPTSTHESEHQSGAWCRDPLQAGERLYVMP
WIPYRTDTLTEYASWEEYAAGRHTTTYRLPSRVDGTGFVVYDGAAFYNKERTRNIVKFDL
RTRIKSGEAIISTANYHDTSPYRWGGKTDIDLAVDENGLWVIYATEGNSGRLVVSQLNPYT
LRFEGTWETTYDKRSASNAFMACGVLYVLRSVYVDDDSESAGNRVEYAFNTHRNREEPL
GGNGGTGLDFPNPYQFVSSVDYNPRDGQLYVWNNYFLVRYALKFGPPDPAEGPVTPTPPL
VTATLPQPPLTVTTPPSITTTARHPPLTTHPVGVVNPIGPELPTPGTRRPPPSNQHSYSEVNCP
PREERRVQWPAAQQGMLVERPCPKGTRGTASFLCLPTLGVWNPRGPDLSNCTSPWVNQV
AQKIKSGENAANIAGELARHTRGQIYAGDVTSSVRLLDQLLDILDAQLQALRPVERESAG
KNYNRMHKRERTCKDYIKAVVETVDNLLRPEALPSWRDMNVTEQEHAATMLLDILEEGA
FLLADNVKEPARFQTARPNVVLEVSVVNTEGSVTELIFPQDNLSLSSIQLSANSIKQNSRNG
VVKVVFVLYNNLGLFLSTENSTVRNGGESSSTGHSLVVNSQIIAASINKESSRVFLMDPVIF
TLPHLQTKNHFNANCTFWNYSERSMLGYWSSQGCRLVQTNKTHTTCACSHLTNFAVLMA
HRDMYQGRINELLLSVISWVGIVISLVCLGICISTFCFLRGLQTDRNTIHKNLCISLFLAELL
FLTGIDKTQYQVVCPILAGLLHFFSLSAFSWLCLEGVQLYLMLVEVFETEHSRRKYYYLCG
YIFPALVVGISAAVDYRSYGTDKACWLRVDNYFIWSFIGPVSLVIVVNLLILLVTLHKMLRS
SSVLKPDSSRLENIKSWALGAVTLLFLLGLTWAFGFLFINKESLVLAYLFTTFNALQGLFIFIF
HCALQKKVHKEYSKCLRHSYCCVRGPGAEHGTLKSTVNSRYYTQSRIRRMWNDTVRKQ
TESSFIAGDLNSTPTLNRGTMGNHLLTNPVLQSRVGTSPYNTLITESVGFSPSSPGYNATEH
TLSRDPCGKDSLPPLPLNGNFNNSYSLRAGDFPPDPSKLAEGTGGVGGRRNLTDAAAFEK
MIISELVHSNLRGKSHGGTTETTEEEVTSGVVPESGEVPTLEMERMYKALEEPLLLQRAQS
ILYQSDLEESEAEAAPRDSPNRDSLYTSMTNLRDSPYPDSSPETGLPPSGEMLPCHQAVPPP

TGPDAMYFPGRLGSVPRGQLQAFYQMPQGFLGLEGSVPEGDGQMQLITSL*
>jgi|Xentr4|465998|C_scaffold_159000002
MTKASYASSPCQYSPSALPAYIHEGEVRIGALIPVHEKTVKPTITFKEKPPERICEKFYTEKY
QYTLALLFAIKEINRNPLLSPNVTLGYEVYDSCYSDAAALDSIMSYLSGKQTKVPNYSCNT
APPKLSAVVGDSPSSGSVAIARILGLTLFPQISYASALPTLADKTQFPSFVRTVGTVDSQPVA
VVQMINYFNWTWVGILSSNNDYGDQGAQKLKTEMAKHGICVAFAKTLLTPPTKENLDTI
VSVIQKSTANVIVLYAYATELIALLQAVTDQKISGKIWIAVGSWLPSAVFSQKELWGTLNGTI
GLAKYSSDIPGFRDFLYSIHPSQYPTDIFIKEFWGHVFSCKWVDNRTENDTLYYGNSTTFCT
GKEKLITLDSTIYDTTNFRLAVSVYNAVYAVANAIHNMLSCKPGTGPFYNGTCATIKDFQP
WQLYYYIKNVNFLNTAGERVAFDEKGELKGLYDVLNWQVSHDMKSNMAKIGIFDDWGP
NGEKLVLNEKSILWGEKYSQVPSSVCCQSCLPGYRKAPREGEPFCCYDCIPCSDGEISNAT
DTTDCMRCPEDQWSDETRIHCIPKVIEFLSYEESLGMALSSVSTVFSLLTVFVLCIFIRYRDT
PLVKANNRSLSYLLLVALLLCFLCSFLFIGHPVLITCLIRQAIFGIIFSLCVSCIFAKTITVVIAF
SATKPNSPLRKWVGSSIPNYMILGGSSIQTVICALWVIIYPSSPELNTKASKGKITVECKEAS
PAFFYVMLGFLGFLALLTLTVAFLARKLPDGFNETKLIAFSMLVFTSVWITFIPAYVSTVGK
YTVAVEIFAILSSSFGLLVCIFAPKCHIIILRPHQNTRDFLTGKTGKT*
>jgi|Xentr4|466007|C_scaffold_159000011
MATLLWALLLLPVLETGSGGGPPRPEIRVDGDFVIGGLFPVHEKGAPGAECGRMNEHRGL
QRLEAMLFALDAINNDPHLLPGLQLGAHILDTCSKDTYALEQALELVRGSLTRTDGPQHLC
PDGSYAIHGESPTAISGVIGGSYSDVSIQVANLLRLFQIAQISYASTSAKLSDKSRYDFFARTV
PPDFYQAKAMAEILRYFNWTYVSTVASEGDYGETGIEAFEQEARARNICIATSEKVGRSM
NKKTFAGVIKALQQKPSARVVVLFTKIENAREFDGWGALESVVLGSEQVAEGAITIELASY
PLMEFSEYFQSLHPDNNTRNPWFREFWENKFQCSLTSPGCSEHSLRQVKFEQESKIMFVV
NAVYAMAHALHNMQHAVCLNSTYLCPNMNPPNGKRFYRDYILNVKFNAPFPPPDTKSTV
RFDKYGDGIGRYNIFNFQNTGGRYHYQKVGYWAEELTLNTSLIPWAKSAVPVSQCSDPCK
KNEVKSMQPGDVCCWICISCQPYEFLVDEFTCKDCGLGYWPNLDLTGCYELPQEYIRWG
DAWAVGPVCLSCLGLLSTLFVIGVFVQNNNTPIVKASGRELCYILLCGVLLCYAMTFIFISK
PSTSVCTLRRLGLGTSFAICYSALLTKTNRIARIFSGARDGVQRPRFISPASQVGICLVLISCQ
LLVVLIWLLFEPPGTRKDTAPDKRYIVTLKCNSGDGSMLISLSYNVLLVLLCTLYAFKTRKC
PENFNEAKFIGFTMYTTCIIWLAFLPIYYVTSSDYRVQTTTLCVSVSLSGSVVLGCLFTPKL
HIIIFQPQKNVTSHRIATNRFSATGPGGTHGSAVQYVPTVCNGREVVDSTTSSL*
>jgi|Xentr4|466121|C_scaffold_167000027
MIGGVLTVGVRAAFMDETLMTCDQPSAQKYRYLVDFAFRVKEINENPALLPNITLGYHIH
DSCGDTRRALMSLLKIISGTREPVPNYSCRRTGKMAGFIGDLFSVPTESIAHILSALGYSQIS
YGATDPALSDRTTFPYFFRTLQSDEEEYIAVCKLLKFFGWNWVGIIYMNNKSGERDYQLLT
KYLSSEGICIEFAREFNESIELSEFIKKTTTRVVIICGDPSYEYKVESYDLHDIIIEKTCIFLSK
WLNHYESLELASLLTGTVLFMQNRLDNQYDARFREFSDTFHPSKYPHDDLLHDIWLFHRL
CLLKDDDKYYYSMFDYFPDKCTGKEKLTDIPGYLNAYHSASLIQAVDMMAMALQDMHN
FHSKQTHGKGRWQVLKSLAVLKLHRYLRNAVYAIDGSPESSFNEKGEFVHQYDINNPFFD
SEGKFSWKTVGRYVPWAPMEQRLILNPDKIIWNTPDYKVPRAQCTDNCLPGLRKVIEPGK
LICCYSCAPCPEGEISNKTDSENCIRCPDLEWPNKNRTICIVKTEDFLSYNNDAIAVVLSSIS
VLFFFFSLLILGVFIANRDSPIVRANNRSLSFLLLVSIKLSFLSVFLFLGRPVDITCMLRIITFGI
TFSIAVSSLLAKTIMVCVAFKATKPGSSWRKWLGVKLSNSVVLFCSSIQIIICMTWLAISPPF
QELDIHTSPGTIIIQCNEGSAIGFYSVIGYMGLLAAVFDSFSLLSPHRHGFVINISFIFLLFSIDI

GSFYCKPGLPHSESQQPEPELPPPCLHQAELPHVWITMIPAYLSTKGKNTVCVEIFAILTSSA
GLLACIFLPKCYTILYRPEINTKSNLLGNKSL*
>jgi|Xentr4|466122|C_scaffold_167000028
MLFCLIVLCVGPCTSGVQPPNPACNLRIIQSDMEYEYILAGDIMIGGVMAAHLNLQTIRDP
GKGMYKLRCVFPSQEQSMYLVDFHFAIEQMNHNPAVLPNLTLGYHIYDSCGDPRKAVRSV
FQILSGTREPVPNYSCVGKRNIAGFIGDLSSETTVPIAQVINVYGYAQICYGATYYSLSDRV
NFPYIFQTLPNPDNAAITLVRLLKDFSWNWVGIITSDDLSGSYEQQILAEAFSFHGICVEFTI
KMNKDKPETSQRDKTTLKESSTEVIIVCGTVSLNHIVIYEKLTNEVSEKTFVLSPSWGDNH
YLFIYTRKTFNCSLFIRSLYSYYMNTPEYLNFIAKYYPLNYPEDKILEDIWIMHFDCLVKDQ
TKNQYYRKDKKLHNCTGAERLTSLPLFYYISHSPRVHLATVLMSLAINHLNNLVHEESPQS
CKEISNYRHQIPRAQCSDNCLPGYRKAPKPGVHSCCYDCVLCSEGEISNRIDSENCIRCADI
EWPNEKRNQCIAKMEDFLSYTSDGISVFFYSISVLFFLLNLLIFGVFIRHLDTPIVRANNRSL
SFLLLVSIKLSFLSVFLFLGRPVDITCMLRIITFGITFSIAVSSLLAKTIMVCVAFKATKPGSSW
RKWLGVKLSNSVVLFCSSIQIIICMTWLAISPPFQELDIHTSPGTIIIQCNEGSAIGFYSVIGY
MGLLAAVSFVLAFLARSLPDSFNEAKYITFSMLLFCSVWITMIPAYLSTKGKNTVCVEIFAI
LTSSAGLLASIFLPKCYIILLKCQMNTKSHLLGNKH*
>jgi|Xentr4|466492|C_scaffold_190000027
MNIFLTTICFCHLYLFLILLWYKDAGCESSLEGCRLSTEDVFGYSLAGDITLGGLFPVHIEAS
RPIITYKECPQPLLCSRFHIRYYRFLLAMVFAILEINASNELLPNITLGFKLFDSCYSEVRSLR
GAKWILSGETNGVPNFHCNKDLMPLAIVGEMLSKASEPLARILGLYRYPQISYGSGLPLLS
NKIQFPSFFRTIHNGVYEIFAIAQLVKYFNWTWVGVISSNNDVGILGAQIVTREIEKNGGCI
AFQETLPIISSMESVYRIIGLVKRSSATVIILICSIGNLVPLMELASFHNITDKVWVATSGWTIT
SDFPRTDILTTLNGSLALAAQKGKIPGFKEFLYSIHPSRFPDDPYMKTFWENVFHCIWPGND
TVNNTSPALLKEDIVWCTGEERLDSIDPNIYDVYNFIYSYRTHNAVFAVAHALHQMKNCVP
GKGPFKNGSCADIYNHRPWQLLHYLRKVDFNNTAGERIYFDENGDVPQSVEILNWQLFP
NGSNQYVSIGSFDSGSLNGEGLSIQLNKILWNGGHSQVPSSVCSDPCPKGYRRAAIQGQKI
CCFDCLPCSEGEILNPNDVSECLKCPEDKWPDSRKEECQPKLIQFLSYEETLGSALACISVL
FCLLTFSVFCLFIIKRETPIVKANNRDLSYLLLISLMFGFMCSLAFIGRPNRIMCMIRQVMFA
VIFSLCVSTILAKTITVIMIFSATNPDSKLKKLVVLRIPIYIVPVCTMVQIILCIVWLTTDAPFA
EYNMAAEIGIIVIECNEGSRVLFACVLGYMGLLASVSLFVAFLARKLPDTFNETKFITFSML
VFATVWVTFIPAYLSTKGKQTVAVEIFAILSSSTGCLFCIFSPKCYTILLHPEMNNRQYITGR
NARNQGIQ*
>jgi|Xentr4|466559|C_scaffold_196000003
MENRTGPTNTTRDPLKRNEDVAKVEVAVLALILFLALAGNICVLIAIHINRHKHSRMYFFM
KHLSIADLVVALFQVLPQLIWDITFRFYAPDFVCRIVKYLQVVGMFASTYMLLLMSLDRCL
AICQPLRSLHRRSDCVYVVLTWILSFLLSVPQIHIFSLKEVGNKVYDCSASFIEPWGLKTYI
TWITITVYILPVMILSVCYGLISYKIWQNIRLKTMCESSVRLSSNKRATLSRVSSVRLISKAK
IRTVKMTFIIVLAYIVCWTPFFFVQMWSVWDPDAPKEDSLFIIAMLLGSLNSCCNPWIYML
FTGHLFHDLLQRFLCCSARYLKSSQQGSDMSTSRKSNSSTFVLSRKSSSQKSITQPSIA*
>jgi|Xentr4|466604|C_scaffold_198000021
MLGGTTASHRIKVLIYLSVLCVGPCSSVALPINPACQLKSAETLEEYEYMQEGDILIGGVM
TLNMYSHIIKDDSRIFFCQMVTPKSYRHLVDFLFVIEEINKNRGHLSNLTLGFHISDSCGNE
MQAVKSVLQILSGTREPVPNYSCAGKSNIAGFIGDLNSGTTVPIAQILSVLGYSQISYGATD
PLLRDRVAFPYFFRTVQSVQHHYFALSKLLKYFGWTWVGIVTSDDDAGEREHRLLSRYFS

SDGICVAFTVALNSNKIAFNANLLHELEQKIAKSYSSVIVLCGTVDQIMVSELFKIILDHNDI
TLVLTALWASDSVFMEFTYYNILHGSLVFAPHFLDPANMYKLQFKQFAADRHPSKYPEDV
FLKKILNYECKAIRLPCPGERRLTDLDGFNDNLNDTFHPPGVYLAALTMAHGLRLLLLNQ
SNEKNGKGHSYKHQLHHYLKRVTLTDTDNLSSYFDENGEFVTHYGIYNLFLNDFPYALSW
AQVGKYTPWAPPDQRLNITTEAITWNTLDNKIPKSKCSKSCLPGYRMAPGPSIHACCYFC
VQCSEGEISSKTDSENCFRCPDLEFPNKRRNQCIPKKEDFLSYTTDVISIVLSSISVLFLLITF
LILGVFIKYRDTPIVRANNRSLSFLLLVSIKLSFLSVFLFLGRPGDITCRLRNITYGIAFSIAVS
SLLAKTIMVYIAFKSTKPGSSWEKWMGVKLSRSVVLVFSSIQIIICITWLAISPPFQELDIHT
YPGTIIIQCNEGSALGFYSVIGVWITMIPAYLSTKGKNTVCVEIFAIVTSSAGLLGCIFLTKCY
IILFRPEMNVRSQLLGNKTR*

>jgi|Xentr4|466905|C_scaffold_224000015

MSVMGAELALPVNATSLVWVDTCVRETSLENALLSLCYFLGLLVGGLGNILALSLFIQDR
QQRSPSDIFLLHLAISDLFLLLSLPTRLFYHLSSNSWPFGALPCRLSAFVFYLNMYASLYFL
AGISIDRYLAIVHPLNSVKIRRPLHAHVTCGFLWVIVAFATAPLLIGNGHATDDRACRLLYR
ETPSLRALSSLSAAFAIPFLGTVTCYGLILKRLRNGGDRKPKERAVKMVLLVLTIFLICFVPY
HLSRALYHVLMPGGEAQAPMSSCSLRQGLALANRFTSCLSTLNAALDPLVYFFAVKKFRE
ALPCRNKGPDAARSREGKTEDSSLSAKTEV*

>jgi|Xentr4|466999|C_scaffold_232000001

MLGGTATYKLLKVLMYLMVLFVGPCTSPVQTNKPACRLEMIKVEEEYEYIGEGDIMIGGV
LTVSSSLGVNDRDITLRCIQPSVQKYRYLVEFLFLVNDINDNPNLLPNLTLGYHIHDSCGDP
QRAVRSVLQILSGTREPVPNYSCVGKRHIAGFIGDLTSDSSMTMAYILGVLHYSQISYGATD
PSLRDRNTFPYLFRTVQSDEEEYIALCKLLKYFGWNWVGIIQFDDYSGYRDHQLLTEHLSR
EGVCVEFSVTLNPESNYRAIVKKASTNVIIICGQLSVTNVFLQLLELYSLGQKTYIYLSKVL
NDFEEFIFNTKLLSDSLTIMQNSINYVFDSTFMDFSNTVRPSRYPEEKLLENIWIRYLCCLSK
NTSKPNVRDSLDRHVPYVCSGKEQLTDIPNYLGLYHSDSLVQAMDMMAFALEDMQDFLS
KQTSEKHGRRNNYNDQLHHFLTKNPYTGDKSHISHFNEKGEFPTVMKIGNPHLHTDGHT
WVWKTVGQYVPWAPLEQRLKLNAEKIIWKTPTNKVPRAWCSERCVPGHRRALVPGFHS
CCYNCIPCPEGEISNITDSEMCFRCPDLEWPNEKKNQCIARTEEFLPYTNDVISLFVSSFSVL
FFLLTVLILGVFISYRDSPIVRANNRSLSFLLLVSIKLSFLSVFLFLDYHGLCCFQSHQAREL
MEKWLGVKLSNSVVLFCSSIQIIICMTWLAISPPFQELDIHTSPGTIIIQCNEGSAIGFYSVIG
YMGLLAATIMVCVAFKATKPGSSWRKWLGVKLSNSVVLFCSSIQIIICMTCVWITMIPAYL
STKGKNTVCVEIFAILTSSAGLLACIFLPKCYIILFRSKMNTKTHLLKNKS*

>jgi|Xentr4|467311|C_scaffold_258000004

MYSIVCGLSLLSNLLALLVFWSNSQRCTSMIVYMRNLAVADLLLALCLPFRVAYQNHNGL
LILCKIVGAFFYLNMYASIMFLSLISLDRYLKIIRPLQKYKIHSVSWSTRATWIIWLINLVCIIP
FLFENRTGPCSQKCFHFQRKGLTGAVINLTAVISFFILLLLFVYFYAKISSKLHKASLGRTQP
QTKRNSNRAMKKTFIVLIIFIVCFVPYHIVRVPYILAQIDIIDTVPWKQTLHIANELVLCLSTL
NSCLDPVIYFFLSDSFKQAVICTFQGKLKLIMNNQDKVGNSNKSITDM*

>jgi|Xentr4|467315|C_scaffold_258000008

MRENNISSVTEFHLLGFPVSNEVAQLLFFIISVVYILTISVNTIIFVIVINDKHLHKPMYVFIA
GLSFLEIWYPSVTVPRLLWDLQTKQKSIPLSGCLTQFYFHLSCGATENFCLAVMAYDRFVAI
CNPLRYLTIMNPSACKKLLMGSWVCGSLVVVPPCLQISNLSFCGHNEIDHYYCDFAPLVKL
SCSDISSVEKTVFISACFVILGCFIVILVSYAFIILTIMKFPLHSERQKAFSTCGTQLIVIFIFYGT
TIFLFLRSNTGDFLHVNKILSVFPSIVVPFLNPIIYTLRNKEVKVSLKKTFRSIIISPKE*

>jgi|Xentr4|467317|C_scaffold_258000010
MLNSTHILSAYDDNNSCSIDDFRNKVYSTAYSMFTLFGLFGNSFALFVLLKTYRQRTAFHI
YMINLAVSDLLFICTLPFRIVYYVSKGRWYFGNFLCRISSYVFYVNLYCSIFILTAMSITRFL
AIVFPVRNLKLVSIKRAKWACAGIWIFVTVTSSPFLMTGSYTVDNMTKCFEPPPSNEAKIM
VIVLNYISLVFGFIIPFAIILVCYTMIIKTLLKNSMKKQQESRKKAIRMIIIVMAVFFLSFMPY
HIQRTIHLHFLKGNKNCKESLHMQKTVVITLALAASNCCFDPLLYFFSGENFRRRLSTFRK
QSVTSMAHEGKRKKSASSPEKSEMIPELEKQDSNES*
>jgi|Xentr4|467493|C_scaffold_273000011
MDGGNHTRITSVLLLGFEMLHKFKIPFFLLILILHCLTVTGNTIIVALVSSSPSLHHPMFFFLS
HLSLSDFILTTNIVPNMLHGILQGEIAMSFPACIIQFHFFSTVIASECLLLAVMSYDRYLAICK
PLRYFSIMHNKLRLQLVIFCWILGFITTLAVVIMLSRLEFCGSNVIDHFFCDFAPILQLSCSDI
SALTLGQMLLSAPMTSFPFILIIVTYICIFIAILRIPSLSGRQKAFSTCSSHLAVVGAFYGSLISL
YVVPSSGNSIFTKKVISLLYTVCTPLFNPIIYCLRNKELKEAFKKWLNTL*
>jgi|Xentr4|467694|C_scaffold_290000010
MDIWQVLTFAFVIDEINKREDLLPNITLGFEIYDSGICEITTIERTFRILSGNHKLIPNYNCRK
TEKILALIGHFVPSCSHAMADLLSLYKYPQISYGAKDPMLDNKDQYPYIFSTIPSEHSLNEA
VVALLQYFEWKWVGIISCSDVKFERSSEEMKKEIIKIGYCVEFFIVVDDRREIFLTGKIQKS
HANGGKFLSDDSNLYYANGSIWFSTQKEDILGLKGFLQSESPTALPGNMFLDTLWEDPLIH
LQNEFSINLLYNIHTAVYAAAHALHGLAYKPHMKENRKQQILQKFIPWQLNGHMKNVSFT
TPDGKKIYFDERGNVPGYFDIWNVVILPNGTLVNVEVGRFDSSAPQGKKLKLNVSKIKWH
PDFIQVPKSVCVESCSPGYRKALREGQQACCYDCVLCPEGEISYIGDMDNCIKCKEQEWT
DERRDKCIQREEEYLSFHDYIGIFWMATSLLLANNLHLSYILLFSLTLSFLSSLLFIGRPKQV
TCLVRQVTFGIIFATELSTLIGKTITVIIAFSATKPGSKLAKWLKTQITYRIVLLLSFGQVIICS
VWLVCSPPFPDTDTKSKTGMIILLCNEGSVVAFYIMIGYIGILAIVSFLLAYYARRLPDSFNE
SQLITFSMLVFCSVWVSFIPAYINTKGRSVVAVEVFAILTSNAGLLGFIFIPKCYIILFRPELNN
KDHLMRKM*
>jgi|Xentr4|467702|C_scaffold_290000018
MYVLLSQLSVSDVLLITNITPNYLCLLLNGGGTISATSCITQFYFYCASVASECFLLTVISYD
RYLPICYPLHYASIMDFSLCLYMSICSWGLGFILSLLLNLLTFDLKFCGPLVIDHYFCDFTPL
LKLSCTDYKIVARTHIILAIPFVLLPFCFIIYTYIFIVLAILGISSTNGRHKAFSTCSSHLIVVCM
YYGNLIIVYMVLSEGHQFNIYKMLSLLYTVGTPFFNPIVYSLRNNEIKVTILKLMSN*
>jgi|Xentr4|467708|C_scaffold_290000024
MPAENQSSSSGFILQGFSDYPDIQIPLFCLFLLIYLLTLQGNVLILTVICQTSLLHIPMYFFLC
NLAFIDMFSSSVSLPKFLSMLLGGDNTISFVGCMTQLYCFMTLTCAEFISLTVMAYDRYVAI
CNPLRYLIVMNKRVCVILVIAGWMFSFAEPMLHTLLISHLPFCRSQAINHFFCDPSILLTLSC
ANTFSVQLLTYVLGSLVGLPAFALTVASYTYIISTIIKIHSATGRKKAFSTCTSHLTVVSLFYG
TILITYMSPTSQYSSTLSKPISFLYTALIPLVNPFIYTLRNKDIKTHIFNIIEFKRSHLQLAGPAL
*
>jgi|Xentr4|467731|C_scaffold_293000004
MLTVSQISWAVILIITWPCGFILNSSIVAVYLRTRKNEMKRGECDKILLAMACSNVLLLSMI
AFDMTFVTYGLYIMFAKEFSLAISVILFFSIHFSFWLTACLSLFYCLRLVNFSHQVLTHLQRR
MSIVVPLFLLASLLISWLINVPLIWMVQIDTNQNSTSIYQDYIFHYDRLYMIFNIVFGSTLPF
VVTSLCIGLCLISLLKHVQSMRQHISQYWSPQLKSHVKAFRTMLLLLILNLIFFTTFGSLYL
VQNNLGAVFQAVLWSATMFIPSGQATILIFGNSKFASTWSKALPMFGSCDRNV*

>jgi|Xentr4|467876|C_scaffold_305000010
MLSWNIYISKGDSGCKLPTENLPVYEKDGDIIFGGLIPVHLEPSNIKTDFTTKPSQRNCSKL
SIFYYLHVLAMVFAINEINENPELLPNITLGFRIYDSCFNEMQAVTGALQLLSGRENPIPNYT
CDMKSKVAGLVGDIQSSNSVAVARILGLFRFPQISYGSALAYLSDKISYPSFLRTVINDKIHA
FYFNMLMIHFNWTWIGILAADNDFDLYDSEFLRRDIEAAGICVAFFMRISAQHTRDQTLSI
LQVIKRSSATVVVLYCSLPELIPFMKVATEENITGKVWFASGSAITSPIFSYKEYWRTLNGTI
GQKLPLTIIPGFGEFLYSIHPSLYPNDIFMKSFWEASFGCLWHNETPVNGTTYCTGQENLKT
LEHQGYNEAASVFAILVYNAVYTLAHGIHNMISCRPQSPCLTISRLDPWKILQYVKQVRVR
NTAGQELFFDENGDVNSYHEYVNWQALPNGETQYVNFATLKEFPESHIVLSNNTIFWSGG
YTEAPESICSESCPPGYRKAPQKGQPACCFDCILCSEDEFSNQTDSTSCMKCPEDMWANDK
HNGCWMKSLEFLSFRETLGGTLTTISVLGSLFPLSILAVFIKNAETPVVKANNRNLSYILLL
SLCFCYLCALMFIGRPITINCILRQFIFGISFVMCISCVLGKTMMVVIAFNLTQPRSSRRMWL
NSRVTNTLVLVCTAIQVIICAGWLAHSPPFQYTDNKSKMGTIIVECNKGSPIAYSCTMGYM
GVLASLCFAIAYLSRKLPGSFNEAKLITFSMLIFGAVWISFIPAYLSTTGKYMVAVEIFAMLS
SSSGLVACIFLPKVYVIILKPEMNKKDRKMTRNSYIHTEITH*
>jgi|Xentr4|468004|C_scaffold_315000013
MDNSNNTAVEEFILLAFSDLYQLQIPLFFVTLPVYIMCVFGNFAIIFLVIAEPFLHTPMYFFIS
KLSVLEIMYVTCIVPNLLANLIAGKKSISFSGCFIQLFANSVLGTAECYLLAVMAFDRDLAI
NKPLHYSTIMTQELCVRVAVLPWIVANITVFISTVCTASLEFCGPNEIDHFFCDFYAVQSLAC
SSTFITQVVTNSCAIFAVVVPFFLTVGLYIHIIFIILKIKSAESKRKSFSTLSSHLTVAGLYYITAI
IVYAVPKDSHNNKFLALIYTVIVPLFNPFIYAFRNKDIKKTLIKSRRLMLCQGSFQY*
>jgi|Xentr4|468097|C_scaffold_324000001
MTVSTLGIFGNSLVIWFLSFKIKRNYSTVYILNLAVADFCFLLVYATLDIIGIALKERLPVVG
KENLELISTIYPLVLPCLFAYNTSLCLLTAISVERCLSVLFPIWYHCNRPRHLSSVVCISIWAIS
CLLTVLEFCYCYQPEYISIYTKVNTTVKECYIVFAIICCLSYIAFIPLMTVSSLVLLIKVWTSS
QQKQPPKLYIVITVTVIFFLVFGMPMRILLLVWYKHHIFPPLPVLNIVSLFSSMNCSINPFVY
FLVGRQGRSSGKLNLVIILQRVFRDDGIQHRRQQKKETTKIQTMMI*
>jgi|Xentr4|468328|C_scaffold_347000009
MNYFSDGDLIIGGILQIHDMFINFLPYIQVKERTKCLAISFRHYRHLLVLIYTIGEINKDPEIL
PNVTLGYRIYDSCGSGMISFASASQILSGTEQPIPNYSCWNNRKVVGFIGDLSSVSSLSIAW
LAGIYRYPQISYGSADPIFNNRLEFPSFYRMIPNVLSEIDAIMSLIRHFGWKWVGLIVSNDD
TGNRARERLEKAMSKDGVCLAFLIRLIDREKTDLTNMEKIRETIYRSTAKVIILFLGLQYIIH
INDIFEPNTVHKKIWIASSSVSHIDELQYLHVFKTFNGTLALSFQQGEIPGFKQFFYSLNPYK
YQRDDLFTEMWEMLFKCTISETDIPFPKCTGNETFDDTVLESYGTFNYRIAYGVYTAVYT
MAHTLHELYGTMTRSPKSAESLHMYFKQWQLNALMPHVKFRTSSGDEMSFKDNGDPPA
RYDILKCYFLKIGNIKRIKVGSFDASRSEENQLFINNSTNLWGPYFSEFVHSRCSEPCKPGFR
KAKVEGKPSCCYTCVLCADGEMSNVTDAQSCTKCSKYEKSNSGRTSCIPRNINYLSYEDQ
LGSTFSSIAVILSITCAVILGIFIKYHETPIVRANNRYLSCLLLISLMLCFLCTLLFIGRPTQICC
LLRQVTFGVVFTISVSSVLAKTLTVIIAFNATKPGSKLKKYVGTQLAIILVIVCSLGEIVISAV
WLASNPPFPEADTLSGPDYIILLCNEGSGFFFFCIIGYIGTLALLSFIAAFLAKDFPDRFNEAK
NITFSMLGFCSVWGAFVPAYLSSKGSRMMAVEIFAILSSSAGLLGCIFIPKCYIIFLRPELNTK
DPFVRK*
>jgi|Xentr4|468329|C_scaffold_347000010
MPEGHTLAALSLDVSPSAQEPALSEMPSPSSGSWLRRNVRMNYFSDGDLIIGGILQINNLF

GNHFIQRIDDLKCIIISFRHYRHLLVLIYTIEEINKDPEILPNVTLGYRIYDSCGSGLKSIASAF
AILSGTEQPIPNYSCWNNRKVVGFIGDLSLESSLSAAQLTGIYRYPQISYGSADPIFNNRLEF
PSFYRMAPNELSEIDAIMSLIRHFGWKWVGLIVMDDDIGHRANKRLQEAMSKDGVCLAF
LIILKYWTQFDWSYAKKIRETIYSSTAKVVILFLSSQSIYHTAVLFDPLKVSHKIWIATSSASR
IAELQYLPALVTFNGTLVISLQQGEIPGFKQFFYSLNPYTYQRDTLFPKIWEMLFNCTFSKK
DISLRKCTGNETFDDTVLSFYEAFNYRIAYGVYTAVYTMAHTLHELYGTMTRSPKSAESL
HMYFKQWQLNALMPHVTFRTSSGDKIYFKENRDPQARYDILKWYFLEIGNKKSIKVGSF
DGSESDGKLFVNDSANLWGPYFSEFVHSRCSEPCKPGFRKAKVEGQPSCCYTCVLCADGE
MSNITDAQSCMKCFKYEKSNSVRNGCIPRDIDYLSYEDHLGCTLSSISVIFSIICAVILGIFIK
YRETPIVRANNQYLSCLLLISLMLCFLCTLLFIGRPTQICCLLRQVTFGVVFTISVSSVLAKT
LTVIIAFNATKPGSKLKKYVGTQLAIILVIVCSLGEIVISAVWLASNPPFPEADTLSDPDYIILL
CNEGSVLFFFSVIGYMTALALFSFITAFLAKDFPDRFNEAKNITFSMLGFCSVWGAFVPAYL
SSKGSRMVAVEIFAILSSSAGLLGCIFIPKCYILFLRPELNTKETIIRKQ*
>jgi|Xentr4|468330|C_scaffold_347000011
MNYFSDGDLIIGGILQIHDIYISSHPIFYGMKEADCRSYLWTKYDYPLIFIYSIGEINRNPEIL
PNVTLGYRIYDSCGSGMKSFASAFGILSGTEQPIPNYSCWNNRKVVGFIGDLSSVSSLSIAR
LAGIYRYPQISYGSADPIFNNRQEFPSFYRMIPNELNEIDAIMSLIRHFGWKWVGLVVSDDD
TGHMVINRLEKVMSKDGVCLAFCFTMKRRCLSNKADIKNIREQIYRSTAKVVILFSSSKYF
TCIGIIYESKKLPKIWIATSSFFHSVELQHLRVLFPFNGTLVLSFQQGEAPSFKQFFRSINPFK
YQRDVLFTKIWETLFFCTFSETEPREGTSINKCTGNESFDDSNLPSYETFNFRTSLQIYTAVY
TMAHTLHELYGTMTRSPKSAESLHMYFKQWQLNALIHRVKFQTFFGAEIHLTNDGVPPAR
YDIHKWYFLKKGPHYNTQSIKVGSFDPSRSDGNPLYINNSANLWGPYFSEFVHSRCSEPCK
PGFSKAKVEGAPSCCYTCVLCADGEMSNITDAQSCMKCSKYAKSNVGRNGCIPRNINYLS
YGDQLGSTLSSISVILSITCAVILGIFIKYRETPIVRANNRYLSCLLLISLMLCFLCTLLFIGRP
TQICCLLRQVTFGIVFTISVSSVLAKTLTVIIAFNATKPGSKLKKYVGTQLAIILVIVCSLVEI
VISSVWLASNPPFPEADTLSDPDYIILMCNEGSDFFFFCIIGYIGTLALLSFIAAFLAKDFPDR
FNEAKNITFSMLGFCSVWGAFVPAYLSSKGSRMVAVEIFAILSSSAGLLGCIFIPKCYILFLRP
ELNTKANVIRK*
>jgi|Xentr4|468338|C_scaffold_347000019
MKILCLAEFVMGILLNAFIVVANAVSWMERKPLDSIDLILTSLGLSRLALLITWLLYVLSED
RYEHIEILNVMSSFFGFCSLWFGTVLCTFYCVKIPNYNHRFFLYVKLRISKMIPWLLLVSVT
SSFISCLPIGWSMDSSMFHYNSTNGTNMETTLYLQFFIYVAGNSVPFFMFCVAITLLIRSLW
NHTRQMAAGEVGFGTPQLQAHYSAIRCMMSFMVLYIIFSSSVFLALPMVLMNDTLLWLY
LFIAGLYPSLHSHILILSNRKLRRALCSLLSYTSTFIPQERNISPCQ*
>jgi|Xentr4|468339|C_scaffold_347000020
MASPLEIFLLTLIWIVTAVGILLNAFIVAMPVIWWVRYNKVEMIEFLLASVGMSRVVLLILW
DVVYLWFPSNSVLFAVVSMFLSFWSLWVATILCVFYSVKISSCHHPFFMFLKLNVSKMLLG
LFLVSLASSLLFSLPFKWLVYSTSINNATNSTNTTNSTGQGGTILKVNNINQFFLILTGSSVP
LLIFCVAVAILIRSLWSHTRNMAGGNVDFGNPQIQAHLSAVKSMVSFLILFTIYFALFVVSCL
PPLLDDTVLQLVFNIISNAYPLLHSLILVMYSRKLREALYWCLHCTCRVPSMARGSA*
>jgi|Xentr4|468379|C_scaffold_354000004
MNPEGCLLLDLEADFALTSSCLNQSSNHSSDDWFYPGVTYAIVPVYGIIILIGLIGNITLIKIF
CTVKSMRNVPNLFISSLALGDLLLLVTCAPVDASRFLADKWLFGRVGCKLIPFIQLTSVGV
SVFTLTALAADRYTAIVRPMDIQANNALTKICIKAALIWILSMTLAIPEAVFSDMHPFYDKEI

NVTFVSCAPYPHSNGLHPRIHSTTSFLIFYVIPLSIISVYYYFIAKNLMRSASNMPVEGNVH
VRRQIESRKRLAKTVLVFVGLFAFCWLPIHIIYLYRSYHYSEVDTSVLHYISSICARVLAFTN
SCVNPFALYLLSKSFRKQFNNQLFCCRPRLIVRTQSMGRSTTRMTSLKSTNHSMASFSLIN
GKHICHEGCV*
>jgi|Xentr4|468863|C_scaffold_406000008
MESLNETSADRFILLGLTNTPYLQELCVFVFLIMYLVTIAGNLFFIIVVGTSVQLQTPMYFFL
CNLSVIDIGFSSTIVPTLLINTVAHDKSISLLGCGVQMYFHLALGSTECFILSVMAYDRYAAI
CQPLHYYKVMSKTVCICLASGSWTVSLINSAIHVVFTFQLPFCRSNHINHFFCEMPPFFYIS
CRDTWFNEVAMYISASIIGLCSFFLTLISYVSIVLTILKICSTKERMKAFSTCGSHLTVVSLYY
GTLTFMYFHPRSDCFPETAKTTSLIYTVVTPMLNPLIYSIRNEEVQNSIKNKLTIILY*
>jgi|Xentr4|468877|C_scaffold_408000002
MYSVVLVIGFPANMLTLWLTILQIRRKNVLAVYLFSLSLSELMYLGTLPLWILYVKNDHKW
QWGALACKITGYIFFNNIYISILLLCCISLDRFVAVQYSLEFRGIRRQRIAAIITLVLCFTVALI
HSTAFMINEGDTEKQSTCFETLPMPQMVANFYFARFIIGFLLPLTVLMFTNCSIKRRIQTSDS
FTHHQKSKVKYLSIAVITIFMICFAPYHLVLLIRAIAFVLNPDNSCPFEENIYTSNAVLLCLVT
VNSVADPFIYVLVSENVRKDICRGLRTWRRQLSTSVKSDNSIYPHIQRSRELPEENSSAQPV
QLL*
>jgi|Xentr4|468902|C_scaffold_410000002
MDHKNKTVVKEFIFAGLSENPKVKPFLFVLFLIIYSLTWLENSALISVIASNEKLHTPMYIF
VSQLSLIDICYSSSISINTLVNLICRKRSISVSGCALQLFTYAGLGGTECYLLAAMAYDRYVA
ICHPLAYLKIMNKETIRILLLVSYLSGFLNSFIHTVMSFFHLDFCEPEQHINHFYCDIMALVK
ISCGDTKINEILIFYLAGFIELSSLVVILVSYIFIIIAVLRISSSTGRKKAFSTFTCHLTVVSLFYG
IVIFMYLRPSSAYIPDQDKAISVFYTIIIPFLNPLIYSLRNKDVKNCAKKSLSRVSCKQ*
>jgi|Xentr4|468999|C_scaffold_423000002
MTASPAYIALEVIIAVLSIAGNVLVCWAVAINSTLKNATNYFLVSLAVADIAVGLLAIPFAITIS
IGLETDFHSCLFFACFVLVLTQSSIFSLLAVAIDRYLAIKIPLRYKSLVSGKRARAIIAVFWILS
FVIGLIPLMGWNRLDKNCSHEPGMSHNCTVQCLFEKVVTMSYMVYFNFFGFVLLPLLIM
LGIYIKIFMVARKQLRQIELKCVGSDSSRSILQKEVNAAKSLAIIVGLFAFCWLPIHILNCITL
FMPDFDKTKPEWVMYTAIILSHANSVVNPIIYAYKIRDFRYTFRKILSKYIVCRKDNFSKCP
NGTVSHNRQLHISSVSAGNAIM*
>jgi|Xentr4|469061|C_scaffold_432000001
MLGGTTASHHIKVLIYLSVLCVGPCSSVALPINPACQLKSAETVGEYEYIQEGDILIGGVMT
LNMYSPIIKYDSLIFFCQMVTPRSYRHLADFLFVIEEMNKNRGHLSNLTLGFHISDSCGNE
MKAVRSVLQILSGTREPVPNYSCAGKRNIAGFIGDLNSGTTVPIAQILSVLGYSQISYGATD
PLLRDRVAFPYFFRTVQSVQHHYFALSKLLKYFGWTWVGIVTSDDDAGEREHRLLSRYFS
SDDICVAFTIALNSNKIASKNYHVKLLHELEQKIANSYSSVIVLCGTVNQIMVIGLFKLIFN
HNDITLVLTALWASDSVFMQVTYHNILHGSLIFAPHFLDPANMYKLKFKQFAAERHPSKYP
EDVFLEEILIYGCKEIRPPCPGEQRLTDLDGFNDNLTDTFHPPGVYLAALTMTHGLRLLLIN
QSNEKNGKGHSYRHQLHHYLKRVTLTDTDNLSFYFDENGEFITHYGINNLFFKNRFPFALS
WAQVGKYTPWAPPDQRLNITTEAITWNTLDNKMPRSQCSESCLPGYRKAPAPSIHACCYF
CIQCSEGEISSKTDSENCFRCPDLEFPNKRRNQCIPKKEDFLSYTTDVISIVLSSISVLFLLITF
LILGVFIKYRDSPIVRANNRSLSFLLLVSIKLSFLSVFLFLGRPGDITCRLRNITYGIAFSIAVS
SLLAKTIMVYIAFKSTKPGSSWEKWMGVKLSRSVVLVFSSIQIIICITWLAISPPFQELDIHT
YPGTIIIQCNEGSALGFYSVIGYMGLLAVVSFVLAFLARSLPDSFNEAKYITFSMLLFCSVW

ITMIPAYLSTKGKNTVCVEIFAIVTSSAGLLGCIFLTKCYIIFFRPEFHVKSHLFRNKTK*
>jgi|Xentr4|469137|C_scaffold_442000006
MTQNEARNITSDTDFHLLAFLIYEKLQLLIFILVLLMYSLTVCANLIITALVCLVPRLHTPMY
FFLCNLSVQDIVYVTAILPKFLAITITGDNSISFLGCMTQIYTYGSCALAEFLLLTSMAYDRY
VAICIPMRYSIIMKRSVCVLLASVSWFVSAINGFLFSWLISNLLFWDHQKINHFFCELKTVV
GLSCSDTDTINIVLIVICIFFGFLPFGLILISYVNIMSVVSKIQTSAGKLKTFSSCCSHLTVVLL
FCGTSLSLYMKPDSGNSQEMDKLLSLLYVAVTPLLNPLVYSLRNKDIINAIKHKIVSMSLFR
*
>jgi|Xentr4|469138|C_scaffold_442000007
MTKNEGTNSTLQREFHLLAFLSHEKLQLLIFLLVLLMYSLTVCANLIITALVCLVPRLHTPM
YFFLCNLSVQDIVYVTAILPKLLAITITGDNSISFLGCMTQFYLHISCVTGEFLLLTSMAYDR
YVAICIPMRYSLIMKRSVCVLLASVPWFIDINNFFCELKTILELSCSGTENIKMVILVKSLVL
GFLPFSLILISYGNIISSALKIRTSAGKLKTFSSCCSHLTVVLLFCGTCISLYMKPDSGNSQEM
DKLLSLLYVAVTPLLNPLVYSLRNKDIINAIKNIMKHNSFFNSNINIASNISR*
>jgi|Xentr4|469144|C_scaffold_442000013
MENQTHSKCLFFLAFFNHGEKLPLLSIVFFLIYVIGILGNLIITMVIYLDSHLHTPMYFFLCS
LAFVDICYPTVTLPKLMDILLSGNDSITCIQCFTQMYFFLTLAAVEAILLSSMAYDRYVAIC
KPLRYHLIMNRRVCVLVTIGTWVSGFVNSAFLTFLASKFLICGSNKIKQFFCDIKAVADISC
DRTAFYGAIYVEAFFFGFTTFSLNVISYINIIQNIIHIKSKHSRQKAFSTCTSHFTVLIIFYGSG
LWTYLRPPSESNQKLDSVFTVLLVGVTPMLNPLIYSLRNKEVKNALIRIVRKKSSCP*
>jgi|Xentr4|469149|C_scaffold_442000018
MENLTNSNCLYFLAFSSYGDKRPLLSIVFFLIYVIGILGNLIIITVIYFDSHLHTPMYFFLFSLA
FVDICYPTVTLPKLMDILLSGNNSITFVQCFTQMYFFFALAAVEVMLISSMAYDRYVAICKP
LRYHVIMNRRVCVLVIVGTWVSGFTNSALLISLVSKLSVCGSNKIKQFFCDIKAVAAISCER
TTFYNSIYVEIFSVGLMTFSLNVISYINIIRNILRIKSKHGRQKTFSTCTSHFTVLIIFYGSAFW
MYMRPPSESENLDPVFSVFYLGVTPMLNPLIYSLRNKEVKKALIRILRVKIR*
>jgi|Xentr4|469806|C_scaffold_523000004
MFCSTYNPFPIFIYLFALRIRQPAAHPTNPACNLEIAKLIEDFEYIQDGDVMIGGLLTVNAYT
MHLLRRNNNYNNKLTLCGGLQPRHYRQLVELRLAIEEINKNPSLLPNLTLGYHIYDSCGDP
GVAVRSVLQILSGTKQPVPNYSCGRKRKIVGFIGDLTSETTIISAQILSLFGFSQISYGASDPL
LSDRVRFPYFFRTLQGFGGSALAISKILKHFGWTWVGIIRIHDSDRENEVHVLTDYLSRDGI
CVEFTHTLSAYLDAPSRDKEINRINEIVNTFTTDIIILCGKLSNDILLALYKLNVQMLINKTFI
LSPSASVIGHNVKSIPATFDGSLILTQYTVVYPRETHELIELINAIHPSKDPTDKLLEDILLLICQ
CSSKDPYKNKLYGLLYKQNYTECEDKNITQCVLFVKSLVLAALSPHVHLAVTIMSRAVHE
MHRSLSEKGRKAQRYQYQLHHYLKKYPYQTKYGGEVSFNGRGEMDTGYTIYFFPSVND
ASYLGTLGRLIPSAPSDYKLIIHPDEIPWEMKNKTMPRAQCTDACQPGYRKALEPGAQPC
CYHCVRCSEGEISNQTDSDNCLKCPDLEWPNEQRNQCIARTEEFLSFTDCTIAEFLSSVSIL
FYIITLLILGIFITFRGTPIVRANNRSLSFLLLVSIKLSFLSVFLFLGRPVDITCMLRIITFGITFSI
AVSSLLAKTIMVCVAFKATKPGSSWRKWLGVKLSNSVVLFCSSIQIIICMTWLAISPPFQEL
DIHTSPGTIIIQCNEGSAIGFYSVIGYMGLLAACLDHNDPGLSEHQRQNTVCVEIFAILTSSA
GLLASIFLPKCYIIMCKPERNQKSCLLQSKT*
>jgi|Xentr4|469875|C_scaffold_532000006
MENQTIVYMLVLTGLSDLPSLQLPLFVLFLLIYLLTLAVNLLILLLIFTDSHLHTPMYFFLGT
LACLDMSYSSTTAPRMLFDLLIGIRIISVQMCITQFYFFLFFAVSEMSVLAVMSYDRYIAICR

PLHYMQIMSWNVCVQFVSIMLVFGAIHALVHTLLLAKLTFCSSNALQSFFCDLPQLLQVSC
SDTFINVLIIFLSGILFGGIIIGVTFYPYITIIKTVLKMPSKQMRSKAFSTCFSHLTVVFLFYSTS
LFNYFRPNASQHTEDKVASVFYAVLTPFLNPLIYSLRNQELKRSLRKTLQRM*
>jgi|Xentr4|469877|C_scaffold_532000008
MLQNQTLFNEFILLSFTNSQIIHCLVFPVFSFLYLLTISINFLIMVLIYHDFHLHTPMYFFLMN
LAGLDAFSSSVVIPRMLIDLLSTKRTISRVACVGQIFFFLMFATAESFLLVGMSYDRYVAICH
PLHYVKTMSWKVCGVLALVAWSLGFSNALIHALCTRKLVFCAQNTIHSFFCDLPLLLQLS
CTDIFLNILAIFFATFTLGFLAVGLTFAPYVPILQAIWRIPTKEGKLKAFSTCTSHISVVFTFYG
SLFFTFLRPASNHHPVADLLVPIVYTAITPMLNPIIYSLRNKDLKEALHTILHRSQVLRCPYT
GQF*
>jgi|Xentr4|470007|C_scaffold_550000004
MHSNIRSYVIEELKDHSTSIMISGSFDPRLPKILQDATTASPISTSTFSSENDTQCGELMILNY
GHIEKLVIGAILSLITLLTIAGNCLVVISVCFVKKLRQPSNYLIVSLALADLSVAIAVMPFVSV
TDLIGGEWIFGHVFCNVFIAMDVMCCTASIMTLCVISIDRYLGITRPLTYPVRQNGRCMAK
MILCVWLLSASITLPPLFGWAQNVNDDKVCLISQDFGYTIYSTAVAFYIPMSVMLFMYYRI
FKAAKRSAAKHKFTGLPRPEESEGMISANGNVKTHKESEECTNLSKLLRNDRKNISIFKRE
QKAATTLGIIVGAFTICWLPFFLLSTARPFICGTECSCIPLWLERTFLWLGYANSLINPFIYAFF
NRDLRTTYRNLLQCRYRNINRKLSAAGMHEALKLAERPDRAL*
>jgi|Xentr4|470205|C_scaffold_583000001
MNPAEVLLAIFLIAVLIVSLLANLLVVICFLYSTEIRKQVSGIFLVNLSLCNLLLTILNMPSTFL
AIVKHQQPFGESLCQAVGFLETFLTSNTMLSMAALSIDKWIAVVFPLSYTSKMRYKDAAL
MMGYSWLHSLTFPLVSYFFSWLDYSSMYASCTLHAQEEADTRRFMVFTIVFHAATFMLSL
LILCFTYLKVLKVARFHCRRIDIITMQTLVLLVDLHPSVKQRCLSEQKRRRQRATKKISVFI
GSFVVCFAPYVVTRLIELLPFVKINRYWGIVSKCLAYSKAASDPFVYSLLRQQYKNVLLNI
VNRVLKRELYPSSGYNSSLDTENDYCLHRPS*
>jgi|Xentr4|470261|C_scaffold_592000001
MSNPSSTCQLSSIRKTKYFREGDLMVGGIFHIKLISQYTVNERFNRKRRRYACTQKEFRRY
RYLLAFIFTIGEINKDPRILPNHTLGYHILESCNEEDRTIKSTFSILSGRKQIIPNYSCWNNRK
VVGFIGDLSKGSSLCITQLAGVYRYPQISYGARDTMFSDRVQFPSFYRTLPDELSEINGIAK
LIKHFGWKWVGLITSDDEDGELAGNRMKRAINTDGGCLAFLSRINHNSFFDESVITSPLRE
STANVIVLLVTLKYINSAMLFFSFYPIPKKIWIVSSSFLRILDTRARNRIPFNGLLVISFKKGEI
PGFREFLYGISLPKCTESEFFSATDLQLNNTLNYRITYGVYTAVYTMALALHKLFLKQKME
TEESLQTHFKKWRLNSLIQNEGFEMPSGDIIHFKENGGPPARYDILKGVFSEEGDIQITKVA
SFDASKPEGSQLEVNNIADLWGPYFKEFPQSRCSEPCRAGQRIFKLEGKPSCCYECVPCVE
GDISNTTDAQSCIKCSKYEKPNNEKTECIPMNINFLSYEDTLGATLSFITLILFGICAVILGIFI
KYRKTPIVRANNRYLSCLLLISLMLCFLCTLLFIGRPTQICCLLRQVTFGIVFTISVSSVLAK
TLTVIIAFNATKPGSKLKKYVGTQLAIILVIVCSLVEIVISAVWLASNPPFPEADTFSDPDYIIL
LCNEGSELFFFCIIGYIGTLALLSFIAAFLAKDFPDRFNEAKNITFSMLGFCSVWGAFVPAYL
SSKGSKMVAVEIFAILSSSAGLLGCIFIPKCYIIFLRPELNTKENIVKK*
>jgi|Xentr4|470262|C_scaffold_592000002
MNYFSDGDLIIGGILQIHDRFIHFLPYIQVKERTKCLAFSFRQYRHLLVLIYTIGEINKDPEIL
PNVTLGYRIYDSCGSGIISFASAFGILSGTEQPIPNYSCWNNRKVVGFIGDLSSVSSLSIARL
AGIYRYPQISYSSADPIFNNRLEFPFFYRMGPNVLSEIDAIMSLIRHFGWKWVGLIVSNDDT
GNRARERLEKAMSKDGVCLDFLIRLKDRVLSHLTNIGKIRETIYRSTAKVIILFLGLQYIIHI

NDIFEPNTIHKKIWIASSSVSHIDELQYLHVFETFNGTLALSFQQGEIPGFKQFLYSLNPYTN
QHDHLFTEMWEMLVHCTISETDISFPQCTGNETFDDTVLESYGTFNYRIAYGVYTAVYTM
AHTLHELYGTMTHSPKSAESLHMYFKQWQLNALMPHVKFRTSSGDEISFKDNGDPPARY
DILKCYFLKIGNIKRIKVGSFDTSRSEVNQLFINNSANLWGPYFSEFVHSRCSEPCKPGFRK
AKIEGKPSCCYTCVLCADGEMSNVTDAQSCTKCSKYEKSNSKRNSCIPRNINYLSYEDQL
GSTFSSISVTFSVTCAVILGIFIKYRETPIVRANNRYLSCLLLISLMLCFLCTLLFIGRPTQICC
LLRQVTFGIVFTISVSSVLAKTLTVIIAFNATKPGSKLKKYVGTQLAIILVIVCSLVEIVISAV
WLASNPPFPEADTLSDPENIILLCNEGSGFFFYCIIGYIGTLALLSFIAAFLAKDFPDRFNEA
KNITFSMLGFCSFVHSRCSEPCKPGFRKAKIVISAVWLASNPPFPEDDTLSDPENMILLCNE
GSDFFFFCIIGYMGTVWGAFVPAYLSSKGSRMVAVEIFAILSSSAGLLGCIFIPKCYIIFLRPE
LNTKDPFVRK*
>jgi|Xentr4|470411|C_scaffold_616000004
MVRVTDITNAFKLTIYIIVLFLVPCTSTVQTINPACELKIIPTYEDYEYIQEGDIMIGGALTVN
SYTVPFRYPHDGYLRMLCVDIYPEYYRQLVDLLIAIKQINQSPNILLNLTLGYHIYDSCGDP
RKAVRSVLQILSGTREPVPNYSCVGKRNIAGFIGDLSSENTVAIAQILNLYGYSQISYGATDT
ELSDRVSFPYFFRTTQSDRGHYFVLSKLLKYFGWTWVGIIRLDDYGGEKEHQLLKSILSND
GICIDFTIKITSFISRTELEANIPNLMVYKRIIEESTASVIVLCGSVPSGAVEEFIHLSDLFIKKTI
IVSYNLASSMHFMDYAIEIFNGSLGITQMLLSSPYSPENTQVLKEFHPSKYPKDKLLEDIW
MQYHSCLSKDPAKNKVYEQLYSGSLYNCSGRELITNMHHYLKKVQYNFFDPKTMNRTLS
FEEDGELITQYKIRYVTFQSYKQIYMQIFGVFTPWAPPDKYLQFISKIGWKTKNNEVPKSQ
CTDNCLPGFRKVPKPGTHSCCYNCVPCSNGEISNTSDSENCVYCPDMEWPNENRTQCIAK
VEEFLSYTNDIISIIFSAFSILFFLITMLILILFITYRDSPIVRANNRSLSFLLLVSIKLSFLSVFLF
LGRPVDITCMLRIITFGITFSIAVSSLLAKTIMVCVAFKATKPGSSWRKWLGVKLSNSVVLF
CSSIQIIICMTWLAISPPFQELDIHTSPGTIIIQCNEGSAIGFYSVIGYMGLLAAVSFVLAFLAR
SLPDIVWITMIPAYLSTKGKNTVCVEIFAILTSSAGLLACIFLPKCYIILFKTENNRKSNLLVI
KS*
>jgi|Xentr4|470503|C_scaffold_632000001
MALESQENVSGFIIQGFSDAPKLQISLFVLFLVIYLIILLGNLIIFLVISCNPHLHTPMYVISCN
PHLHTPMYIFLQNLSLIDISSTSNVLPNLLHILLTQQNNISFLGCMTQMYVFVALASSEYFLL
TAMAYDRYVAICDPLHYIARMSRKHCAWLITASFTVGFVDSLADVVLIPKLSFCASRLINH
FFCDVTPLLKLSCSSTFSVELSIYIVGTLLIFSSFLPTLTSYIFIISAILKIQSSEGRQKAFSTCAS
HLACVITLYGTVIFLYMRPTKSYSLEKDKFFSLLYIVLVPLLNPLIYTIKNREFNLLLIE*
>jgi|Xentr4|470640|C_scaffold_657000009
MYPTNWTSVGEIVLLGFRNLQNFKIPLFSLFLLIYIMTLSENLLIIALVGSSRNLQAPMYFFL
QQLSLSDLLGSTNILPILLQTVINEGAVVSLVGCITQMYIFGGTETFECLLLAVMSYDRYVAI
CNPLRYTSIMSHQVCVGLILLSWLLGFGIILIIVYMVGKLQFCKHNTINHFFCDFTPLLELSC
SDTFFLLQIDIIFSIPLVICPFIFIIGSYICIAHQILKIVSSIGRQKAFSTCSSHLAVVSIFFGTLTAI
YVVPPRKDSQTISKVLSLLYAVAIPLVNPVIYSLRSKEMKETLEKSLRK*
>jgi|Xentr4|470647|C_scaffold_657000016
MSSINRCNETKITQFLLLGFHGSLILRSFLFYLCLLIYIMALGGNLLIVALYLGSHHLRSPMY
FFLSNLSVTDILLSSNVVPNLLGTLISDGKLMSVSACVTQYFAAGVFTGGECLLLTVMAYD
RYLAICKPLHYVTIMTNKHCLHLLIWAWVEIVAISVPAITATSYSGFCGCNKLDYIYCDFLP
LLEVSCSDVSTLEMLIPVMTMPVVVLPFLFILTTYVCIFFSIFRISSNTGRQKAFSTCSSHLTV
VCTYYGILCAKYIVPTKGQSLNVNKIISLMYMMVTPLFNPIIYSFRNQEIWRALNKWVSV

KVPT*
>jgi|Xentr4|470706|C_scaffold_669000004
MANRTRVSEFILLGFPGLDESYDVPVSIGLFLAYIISLFANATVILLIAFSRHLHQPMYKIMV
NLAVSDLLFDTITLPKIIAKYWFGAGSIPYCVCLLQLFCVHFLGSFDAYIIMLMALDRYIAIC
HPLRYACIMTNRTTAILCCFFWIFAAAISAVITILDSTVPICNKNKIKSCFCTNTGLTALSCAD
VTFVKRLAFGLAMFVLLLPFTFIIFSYIAIIKAICSRDHFENWRKTFYTCATHLLVIGLYFIPRI
FVYISNQVQLILEEDLNVLLLCLYTFVPHMANPIIYCLRTKEIRKTIARFFQKIVIKVKNPISV
SVIVN*
>jgi|Xentr4|470724|C_scaffold_672000003
MLGNHTTPFEFIFLGLSNVPSAGLPLFVLFLLIYLMTLVGNLLILLMICTDSHLHTPMYMFL
SNLAGLDIFYSTVTSPRMLSDFFSKTKTITLPVCITQFFFFFSFICIELYLLAAMSYDRYVAIC
HPLHYIQIMHPKFCAMMVSVAWAGGFLTSLIHTLCTLRLTFCGPNLIQGFFCDLPQLFALSC
TNTFVNVLVMFLVGIFMGSGALGITFVPYIHIFRTIKKIKTWRGKLKAFSTCASHLTVVFVF
YGTLIFTYLRPTPRNGFSEDRLVSVVYTVVTPILNPIIYSLRNKDLKEAFRRTAHKIGFLRCN
TS*
>jgi|Xentr4|470727|C_scaffold_672000006
MLFSIKVIKMTIVVVTWLCGSILNSSIVAVYLREWKNGMSLGECDRTILTIGCNNFFLQCFL
TSYEIMAIFELYGLFLKEFKVAGLIFFFFFNYITMWLTACLSICYCVKLVSFSHELLIRLKRG
MSSAITLFLLGSVVLSGLINVPFIWTMDTEFLGNTTLTADNVIYKPDLKFLCFNIVIGSCVPI
LVTSLSIGLSVMSLRRHVQQMKNNTSQSWTPQLKSHVRACRTMSLLLILNLIFFITFITVAL
GLTQFKGILIGDILYWSVIMSGPSIEAVLLILGNTKLKTAFSKICF*
>jgi|Xentr4|470730|C_scaffold_672000009
MLSALQVIRAVMLIVTWPCGTILNSSIIAVYLSDWKKGVKLGECDQISLSMGCTNLLLQCF
VTLGVAFLSYGLHLPFAVQVSFTVGHIYLFFVFLSFWLTASLSTCYCLRLVNPLPKCFIQLK
RRLSRIVTPLLVWSVAISSITTVPMNWTLAIATDQNITTMSYNISVNVVHLIFETAFGIGLPSI
ITSICILLSLISLLRHIRRMKQNPQFGSPQLKNLIRACRTMFLLMALNLLFFLVILSSALPPYS
GNTLWQTVIYSCVMLTPSGQATVLIFGNSKVLSAWSKTLVPQG*
>jgi|Xentr4|470731|C_scaffold_672000010
MLAAYTVIVTVILIVTWLCGTILNSSIIAVYLSDWKKGVKCGECDQITLSMGCNYLLMQCF
IIFFWAFRFYGLDLPFAEKLSFAISTVFWFSVFLSFWLTASLSICYCLRLVNLSSAVFNQIKGR
LSRIFIPLLLWSVAISFIFPVTRIVDIKIDQNGTFIYHENISNVGILISAVAFNVCLPFIITSICIFLS
LISLLRHIWRMKQNTQFGSPQLKNLIKACRTMFLLMALNLLFFLIICSSMLISDRMGTVWG
KVVLLGNMLNPSCQAIVLIFGNSKLLGAWIKTLFPQ*
>jgi|Xentr4|470809|C_scaffold_680000003
MGPKGILVPGDLLIGAVIPIHIDSFFPLVSFLKKPLPDICKMFRLEFYQQFQALRYVVEEINRS
PDLLPNITLGFYVYDSCSVLKSELQGTLWMLTGLTQEIPNYRCRDSPLAAIIGHSKSTFSIL
MAHILGLYKFPQVSYFSTSSLLSDRKQFSSFFRTVPSDIFQSKGIAQLVKHFKWTWVGLIAA
DDDYGHEGIEVLRQEIIKEGICVAYTEYISSNFIFKNIWNIANVIKESSAKVVVAFSTDIYLIP
VLDEMLKLNVTGKIFISSEGWSTSNVISAKKYSSLLYGSLGIAFYSSNIQGFHDFLNSINPFN
TSGITWSKLFWQEAFRCRLNSYNDVSNLRASYNIYTAVYVIAKAMDDLSHCQYLDGPLPK
SKCSDLDNFKPWQLLYYIKNVRVNLSNGREVSFDKDGNPPAVYDIVNWQPGADGTIKQV
KVGSYDSRNSSGDVFGIKTTSISWGTKNKEIPLSVCSQSCPEGFRKATRQGEPVCCFECVP
CGQGEISNQTNSIECWKCPWDMWPNSARDRCLSKPIEFLSYEEPLAIILASTGVISSLIPISIF
RLFIRYKSTPIVRANNYSVSCILLVSLSFCFLCALAFIGYPQPEKCLLRQAAFGLVFTLCVSCI

LAKTVIVVFAFMATKPGSRLKKWTTPRVPYMIITICTFIQLTLCIFWLSISPPFPQYNIEAKPG
IIVVECNENSQFAFWCMLGYLGFLASVSFTVAFLARRLPDSYNEAKFITFSMLAFLSVWVS
FIPASLSAQGKYTVAMEIFAILTSTWALVFCMFLPKCFVILFRPNMNTKEKLMGKDRSRM*
>jgi|Xentr4|471030|C_scaffold_729000001
MIQFIFFLVALWVTPCSTQLSGSDSQCRIHITKPKYEYKYIQDGDIIIGGVFSVNYGVKYILD
SNGKYIPICITPVQDRYIEIQTLLFTINEINKDPDLLPNVTLGYHVYDSCGDPNLAMKSVLQI
LSGPGIIVPNYSCGDQREIAVFIGDRSTVTALPMAQLLGTYGHTQISYSAIDPVLNDRALYP
YYFSTGPNDYVEHVAIAELVEYLGWTWVIILAAGDERGERESKNLMKELNKHGACVDLI
GTLTGDINTDTRTLERIQKSTAEVVVLCGEKFRIKTYFVENMIKEKTLVVPATWIPGYTFPL
FNGSLAFKEEFFIFDNSEFEDYVMKIKEDVLLKDLLTVASSCLTHDKEKDTLLQTVYRVRY
RNCSGSKLLPFLGRSYRVYTAVYGFAHAEHDMRSSSGKYCNKRVHNNINRKQLHHFLRK
THFKDPWGKNRNYVGTYTWSEPKGFLEIDIQKIIWKKNTSNQTLKSQCSSKCPPGYRKVP
QKGAPPCCYDCTSCSEGEISNLTDMENCVTCLDYEWPNNGKTRCIEKPTDFLSYDRDSLTL
VFNVITLILFVIALSILGTFISYRDTPVVKANNRSLSFILLVSIKLSFLSVFLFLGRPVDITCML
RQTSFGITFSIAISCVLAKTIMVSVAFKATKPDSSWRKLAGAKLANTIVLVSSMIQVVISVIW
LAISPPFAEQNIHSEPGKIIIQCNEGSVVAFYIVLSYMGLLASVSFIVAFLARSLPDSFNEAKY
ITFSMLLFCSVWITMIPAYLSTKGKYTVAVEIFAIISSSCGLLFCIFLPKCYIILLKPEMNTKLN
LLGNKKKQCKSFKHSIHI*
>jgi|Xentr4|471039|C_scaffold_730000006
MHRNPFPLACHGGLFLFIIILLVSTVLEGKFNPKCALMSSFSSQHLYQPGDIVLGGIMQLSF
DKDLIHSGSMIMKRSTDLQLFVNAKFYYHHYMALKFAIEEINRRADILPNITLGYMVYNT
RGIERGSMAGVMSVLSGVKELVPNYNCERRGILAGFIGDLTSASSNIISLLTGLYHYPQISY
GATDVTFTDRRRFPYIYRTVPNEGYFYQALIQLLKHFGWTWVGILIADNEAEYIKRQLIELI
TSNGMCVEFAYGFLWDGRFDDRIVHRILRTTSKVVIISDSTNLFWPILRQVKIASSFKRTWI
FSVSSSFTDYILEYEIAEFFAGSLAFPVAAREMDGFQDFLFSANPDRYPEDIFIQDAWLGFVS
DLSTEMTDSETYVSNITGSEDFLNASSLGNETLASIPHYRQLDLRVPYSVYKAVYLLAQAL
HVLLSEKTSHGAPTEKAQLERELNPWKLNHLLRKVHVTLSSSQEEIYINKEGEAPGQYDL
TNWVMFPDRRTNELNVITVGRYNTLNPNQLICNDRAIVWYPNDTEIPISMCSDSCIPGYRK
NSREVEFSCCYDCVPCAEGHISNTTDMETCIQCPEDQWPNDNRTICIQKITEFLSYEDPLGQ
SLAAVSVLSSLTVISVLLIFIKYHKTPVVKANNQTLSYSLLLSLTLSFLCCFLFIGRPQKVTCL
LRQVTFGINFTLSVSCVLAKTVTVAIAFNATKPGSKIKKWVGTRVSLCLVLLCSLLQVGICL
VWLISSPPFPDYDTHTYTGKMILQCNEGSVTAFYTVIGYLGFLSGLSFIVAFLVRKLPASFN
EAQLITFSMLVFCSVWVSFIPAYLSTKGKYMVAVEIFAILASSAGLLGCIFIPKCYIILFRPEQ
NTRRGLTGKHLQ*
>jgi|Xentr4|471110|C_scaffold_745000002
MAFIFAVEEINRSPWILPNITLGYQIFDSCGATPISLSSALGVISGKEFNVPNFSCWGNSKMV
GLVGDLSTDTTYTIAQLVGVLSYPQISYGAKDPVFNDRTQFPSFYRTIPNEEAEMDGIVQIL
KHFGWKWVGLIISDDDTGYRARERISKELASRGGCLAFTALIKFYYKLLKEIEKTTANVIV
LFISPETNLIYLSSLYDLPPKLWITSSFIYNVIMLHEQKMETTLNGSLSLMIQEGEIPGFKQFF
YTFSLNNYPNNALIASTWHLLFVCPFIDIPMSVTKEACPGNATFSEADVSVYGNHHYRVTY
RVYTAVYALARALHNLYSAQPPANHWGKLESLKRNIKPWQINQFVRNVTFTTFPNDTHSF
NKYGDPPARFDIIKLLFLPGDRSVITKVGNFNVSEHGIQFYINNSADLWGPHFNMTPQSLC
NEPCAPGYRKSKIEGAPSCCYDCVPCVDGEMSNSSDVPSCFRCPGYEMSNKQRTACVPK
MINYLSYEETLGASLASIALVLFLTTSAVQGVFVKYWETPIVRANNRYLNCLLLISLMLCFL

CTLLFIGRPTQICCLLRQVTFGVVFTISVSSVLAKTLTVIIAFNATKPGSKLKKYVGTQLAIIL
VIICSLGEIGICLIWMTFDPPFLEVDILSERDTIILQCNEGSVTFFFCIIGYIGTLALLSFIAAFL
AKDFPDRFNEAKNITFSMLGFCSVWGAFVPAYLSSKGSRMVAVEIFAILSSSAGLLGCIFAP
KCYIIFLRPQLNIKVTAQNK*
>jgi|Xentr4|471237|C_scaffold_778000002
MQDGDIIIGGVFSINSALYYIPEHNGKHKPLCINPTESHYVDILSFLFIIEEINKDPDLLPNVT
LGYHVYDSCGDPSLAIGSVLQILSGPGEPVPNYSCGDQGEIAGFIGDVSPVTSLPIAQLLSV
YGYSQISYGATNPALSDRTLYPYYVSTGLNDHVQHVAIAELVEHLGWTWVIILVDDDDYE
QSKNLRTEITKHNACVDFIAALTGDINTNRRTLEHIKQSTAEVIIFCGRVSRGRSLVSYLETII
QDKTLVVPAIWVSMLTLRLFNGSLLFAEARDFFEDNTEFNQFSLAITEDVLRNDLFSVQSS
CLTHDKEKDRLFQKVYDKVYRNCPGLKWSDEGYPTLQVQRAVNGLARAEHIMLSSFGK
YHKDIYKNIHKNKLHQYLRKVRFTEASGREIDFSNLINSPVKFQIFSWYCYLTFEIRQVYV
GEYVWSGSERSLEIQIEKIFWKKNTNNQTLKSQCSANCPPGSRKVPRKTAPPCCYDCISCS
EGEISNLTDMENCLKCQDYEWPNQEKTMCIEKQTEFLSYQDDPLTLAFIVLSVVFILITTVI
LGIFISFRDTPVVKANNRNLSFILLVSIKLSVLSVFLFLGRPMDITCMLRQTSFGITFSIAMSS
VLSKTIMVCMAFKASKPDSPWRKYVSAKVAYWIVFVCSVIQILISVIWLASSPPFVEHNIHS
EPGKILILCNEGSVVAFYIVLSYMGLLASVSFIVAFLARSLPDSFNEAKYITFSMLLFCSVWI
TMIPAYLSTKGKYMVAVEIFAIISSSCGLLFCIFLPKCYIILFKPEMNTKQYLLGSSK*
>jgi|Xentr4|471249|C_scaffold_779000005
MCVGPCRSEILITKAACRLQIIKAVEEYEYIQEGDIMIGGVLTVIQHFNKKPDILPNKTLGY
HISDSCGDLRKAVKSILLILSGTREPVPNYSCVGKRNIAGFIGDLTSETTIPIAQILSVFGYTQI
SYGATDPALSDRSTFPYFFRTTESDAGYYVLISKIAKYFQWNWVGIIRSNDERGERDHQLL
KYYLSSENICIEFILKIADLYGNIFYREILRKASANVIIFCGAVNIHIILQFRYLYDMLSDKTFI
LTSNWLYYNHLIDFSHKIFHGSLLLMQNKENYPSNSPYAQFSKGFKPSRYPDDKLLEYIWL
QNNFCLHKNQFIWPFYNCSEQESIAEIPMYNSGFHTYNMIYAADMFVKALHYMHHSPGL
GTSGMSKKVYNYRYKIHRYLRQIQYLNNTNHVFSFNENGEFVTLYLIINLYPNLRRQSAW
KICGNYFSLAPTHQKLNITPAEIQWKTKNNKVPRSQCSDSCPTGFRKTLKLKAQPCCYDC
VLCSEGEISNRTDSENCIRCPYNEWPNEKRNQCIAKIEEFLSYTNNAIPVFFSVISALLLLKT
VMILGVFIFYRDSPIVRANNRSLSFLLLVSIKLSFLSVFLFLGRPVDITCMLRIITFGITFSIAVS
SLLAKTIMVCVAFKATKPGSSWRKWLGVKLSNSVVLFCSSIQIIICMTWLAISPPFQELDIH
TSPGTIIIQCNEGSAIGFYSVIGYMGLLAAVSFVLAFLARSLPDSFNEAKYITFSMLLFCSVW
ITMIPAYLSTKGKNTVCVEIFAIITSSAGLLACIFLPKCYIILLRPERNRKEYLIGRSNN*
>jgi|Xentr4|471252|C_scaffold_780000002
MFLILQNIHLQFHATGKSSIVYLRQYMAFLYAIEEINNSSQILPNITLGYNIFDSCGCDKKAIS
GVLDIISGTKNTVPNYDCWSKIKMVGFLGDLTSSTSHTMAQFLAIYGAMDPVFHDRTQFP
SFYRTIPNEEAEMDGIVQILKHFGWKWVGLIVSDDDTGYRARERISTELARMGGCLAFSIV
IEYTSDMTATKNPVYLKFANTAVNVTVLVMSMKYVHGFEFMLDQIRIPMRFIITSSLFTNF
MTLRYLRKGATALLDGALALTAPAEEIPHFDHFFNSFSLYKYPQSKLINNLWLSFYNCVSKS
DSRFKGMDPNTWKFCKGNESVNDERLSQRESYRDSYRVYTAVYALARALHNLYSAQAPT
NHWDKLEYMRGNLKPWQINTYVRNVTFTTPSGDTIFFNDKGDPPAQFDVIKYKRYRGYF
GSQKIGSFHVLSDGTKLLHINSSADLWGPYYKEMPQSLCNEPCAPGYRKAKIEGKPSCCY
DCAKCADGEMSNTTDALDCLHCSEYEKSNKQRTGCVPKEINYLSYTDTLGASLTSIALVL
FIAASVVLGIFVRYWETPIVRANNQHLSFLLLISLMLCFLCTLLFIGRPTQICCLLRQVTFGI
VFTISVSSVLAKTLTVIIAFNATKPGSKLKKYVGTQLATILVIVCCLGEMMISAVWMASNPP

FLDADTLTDINTVFLMCNEGSVLFFFSVIGYMTALALFSFIAAFLAKDFPDRFNEAKNITFK
SPTYFSLYGMELFSSWLVSAGLLACIFVPKCYIIFFKPEQNKRKM*
>jgi|Xentr4|471258|C_scaffold_780000008
MDFTTVDIVELVVSFIQFLFGITINGFIMGTFSMQWRRNKSLQASDTVLMFLSTTRFFWQW
VLSLMILYNYITFSLDFLQLLTYIIFLGLSVFFHTSSLWFASFLCAIYCVTVANYSNSLFVYV
KRNISRLGSWMIVAILLTALTFSLLDWYIAYGFGLSPSNSTGIIQQRNSTGADGNAATTLGV
QFVIYFMAVALPFTLYCLAALLLLNSLWRHVRQMRISGTSFRSRSVDAHVGVLMKLAVSL
LLYAVYYVTDTLGLYGQLETPWLLFCFVICCFYPTAHSLVLIYSNSRLRKACVATIRGAMS
WEKRETQDNQTQSAD*
>jgi|Xentr4|471320|C_scaffold_793000001
MAKFIEDFEYIQDGDVIIGGLLTVNAYSMHLLRRNNNYNNKLTMCGGLQGRHYRQLVEL
RLAVEEINKNPSLLPNVTLGYHIYDSCGDPRKAVRSVLQILSGTREPVPNYSWMISYGASD
PLLSDRVRFPYFFRTLQGFGGSALAISKILKHFGWTWVGIIRIHDSDRENEVHVLTDYLSRD
GICVEFTHTLSPYLDAPSRDKEINRINDIVKKFTTDIIILCGKLSNDIMLALYKLNVQMLINK
TFILSPSASVIGHNVKSIAATFDGSLMLTQYTVYPRETHELIELINAIHPSKDPTDKLLEDILL
LICQCSSKDPYKNKLYGHLYKQNYSECEDKNITQCVLFVKSLVLGALSPHVHLAVTIMSRA
VDEMHRSLSEKGRKAQRYQYQLHHYLKKYPYQTKYGGEVSFNGRGEMDTGYTIYFFPS
VNDASYLGTLGRLIPSAPSGYKLIIHPDEIPWEMKNKTMPRAQCTDACQPGYRKALEPGA
QPCCYHCVRCSEGEISNQTDSDNCLKCPDVEWPNEKSNQCIARTEEFLSFTDCTIAAFLSS
VTIVFYIITLLILGIFITFRDSPIVRANNRSLSFLLLVSIKLSFLSVFLFLGRPVDITCMLRIITFG
ITFSIAVSSLLAKTIMVCVAFKATKPGSSWRKWLGVKLSNSVVLFCSSIQIIICMTWLAISPP
FQELDIHTSPGTIIIQCNEGSAIGFYSVIGVWITMIPAYLSTKGKNTVCVEIFAILTSSAGLLAC
IFLPKCYVIMFEPETNQKLCLLRSKTL*
>jgi|Xentr4|471325|C_scaffold_793000006
MMGGVLTVGVRGTFNETTVLGCDRPSAQKYRYLVDFAFRIKEINENPALLPNITLGYHIDD
SCGDTRRALMSLLKIISGTREPVPNYSCRRTGKMAGFIGDLLSVPTESIAHILSALGYSQISY
GATDPALSDRTTFPYFFRTLQSDEEEYIAVCKVMKFFGWNWIGIIYMNNKSGERDYQLLTK
YLSSEGICIEFAWEFSELSEHSQFLTKTTTRVVIICGDPSYEYRVQSYDLDYIIIQKTCIFLSK
WLNHYESLDLASLLTGTVLFMQNRLDNQFDARFREFSDTFHPSKYPHDDLLHNMWLCTH
KCLLSYDDIKYYNIFDDIPDKCTGKEKLTDIPEYLNAYHSASLIQAVDMMAMALQDMHNF
HSKQTHGKGRWLHRYLRNAVYAIDGSPESSFNEKGEFVHQYDINNPFFDSEGKFSWKTVG
RYVPLAPMEQRLILNPDKIIWNTPDHKVPRAQCTDNCLPGLRKVIEPGKLICCYSCAPCPE
GEISNKTDSENCIRCPDLEWPNKNRTICIVKTEDFLSYNNDAITVVLSSISALFFFLTLLILGV
FIANRDSPIVRANNRSLSFLLLVSIKLSFLSVFLFLGRPVDITCMLRIITFGITFSIAVSSLLAKT
IMVCVAFKATKPGSSWRKWLGVKLSNSVVLFCSSIQIIICMTWLAISPPFQELDIHTSPGTIII
QCNEGSAIGFYSVIGYMGLLAAVSFVLAFLARSLPDSFNEAKYITFSMLLFCSVWITMIPAY
LSTKGKNTVCVEIFAILTSSAGLLACIFLPKCYIILLRPEMNSKSNLLGNKSL*
>jgi|Xentr4|471350|C_scaffold_799000007
MSNQSTISEFILMGFPGLQQKYFIPVSITMFLVYCVSLVANSSVIILVLQKEQLHQPMYIIIRN
LALSDLLFDTITLPKIIAKYWFGAGSITFYGCFFQLFCVNSLGSLYSFIIMLMAIDRYVAICQP
LRYHSIISNKLVTLLCYFLWVFAALIGSIVAVIVGQLPYCGPNRVKNCFCVNSAVAVLACVD
VTLARRTVFILAMCVLLLPLAVIILSYVLIIRVIHSTNTENSWKAFYTCSTHLMVIGLYFIPRV
FVYSTSQIPLTLDADINVLLLCLYTFIPHLANPVIYCLRTKDIRNIFAQSFNNIFHAKI*
>jgi|Xentr4|471466|C_scaffold_828000002

MENQTTVYNFVLAGLSDLPSLQLPLFLVFLLIYLITLTGNILILLLIFTDSHLHTPMYFFLGTL
ACLDMSSSSVTVPRMLFDLLRERRIISVPACITQIYFFVFFIASEMSVLAVMSYDRYIAICRP
LHYMQIMSWNVCVQFVSCVLVCSTVWSLVHTLPLTKLTFCRPNVLQSFFCDLPQLLEASC
SDTFINVLLIFLIGILSGVGILGLTFYPYIPIITTVLKMTSKHTRSKAFSTCSSHLTVVSICYTTI
FFNYFRSNANDHLVGDKMASVFFAILTPSLNPVIYSLRNQELKLSLRRTLQQLQ*
>jgi|Xentr4|471468|C_scaffold_828000004
MENQTTVNNFILAGLSDLPSLQLPLFLVFLLIYLITLTGNLLILLLIFTDSHLHTPMYFFLGTL
ACLDMSSSSVTAPRMLFDLLRDRKIISVPACITQVFFFTFFIASDLLVLAVMSYDRYIAICCP
LHYMQIMSWNICIQLVSCVLVCGAVCSLVHTLSLTKLVFCRANVLQSFFCDLPQLLEASCS
DTFINRLLIFLLGSVFGAGILGLTFYPYIPIITTVLKMTSKHTRAKAFSTCSSHLTVVSICYST
GLFNYFRSNANDHLVEDKVASVFFAILTPSLNPVIYSLRNQELKLSLRRTLQRLQ*
>jgi|Xentr4|471496|C_scaffold_834000002
MIGGVMTAHFILKELTDTNDRLVKHSCTGPNEEYLKYFLDFHYVIEQMNNSTTQFPNLTL
GYHIYDSCGNEQKAVRSVLQILSGTREPVPNYSCVGKRNIAGFIGDLSSKTTLPIAQILSIYG
YSQISYGATDTSLSDRLTFPYLFRTVQSDQTNYLMLVRLIQYFGWTWVGFITLKDFSGFYE
HQTFSQYLSSQGICMEFAINLNLNIFNDEFIPSYRKTIADSSSNVIIVGGTVSIAIISKIAALAD
VLANKNVIFSPQWETYLLLHFHSYTSVNNSLIFSSLYPYNLDTPEINYYYEHLHPLKYPEVT
FLEDIWFFIFYCVSRDLNKKSPFGKYEWNDLTLHNCTGEERITNLLGFKLFGNSPRVHFPV
TMMVRAIEALHVAHNQEPPEKPIRPIIYRHQKSPFGKYEWNDLTLHNCTGEERITNLLGFK
LFGNSPRVHFPVTMMVRAIEALHVAHNQEPPEKPIRPIIYRHQLQHYLKKMPLYMEYGQF
YEFDEYGNLEIDYGIFNCIIEPSIETGEVEVTLRPVGKIRPRAPSDQQLEMNKDLIMWNSDG
LEIPRSQCSDNCLPGFRKATKETIQSCCYRCVPCAEGEISNTTDSELCFPCNDTEWPNKKRT
QCIAKGEAFLSYTNDVISMFFSAFSVLSFFITLLILRVFISYRDTPIVRANNRSLSFLLLVSIKL
SFLSVFLFLGRPVDITCMLRIITFGITFSIAVSSLLAKTIMVCVAFKATKPGSSWRKWLGVKL
SNSVVLFCSSIQIIICMTWLAISPPFQELDIHTSPGTIIIQCNEGSAIGFYSVIGYMGLLAAVSF
VLAFLARSLPDSFNEAKYITFSMLLFCSVWITMIPAYLSTKGKNTVCVEIFAILTSSAGLLAC
IFLPKCYIILFKPAMNTKKNLL*
>jgi|Xentr4|471626|C_scaffold_877000005
MPEVVPNGSSFTEFLILGFSHSEKQKVPLFLLFLLIYLFTVISNTLIIALISLDSNLHKPMYFF
LCNMSCLDIFFTSVTAPTLLHMQLSEAKRISFRACMAQLYLFNSLAGVEYMMLTAMAYDR
YQAICNPLVYHQNMNRNVCCILAAVAWGGGFIAALPINILISALCYCSSNRINHFFCDLTAL
LTLACNDTSAVELMIFVAGVLIVLNCFLLTVTSYGFIGFAIIRISSSKGRFKAFSTCASHLTVV
ILFYTMIACLYMKPSSSYSLSDGKVLSVLNVHVIPMLNPVIYTLRNKDVKKAMERIYQASL
PC*
>jgi|Xentr4|471653|C_scaffold_882000008
MNTTSSCNQSKITEFLLVGFSAPRPLRVLLFSICLVIYIMALGANLMIIALYLGSHHLRSPMY
FFLSNLSATDILLSTSVGPNLLCSFLKDGNPMSVSACVTQYFASSVFTGGECFLLTVMAYD
RYLAICKPLHYVTIMTNKHCLHLVIWCWLLIFLFVLPIIVTISQSGFCGCKTLNYIYCDAAP
LLKMTCSDVFVLERAITVLIVPVAVIPLSFIITTYVCISLSIFRISSNTGRQKAFSTCSSHLAVV
SMFYEIIIAKYAVASKGPSINVKKMFSLLYTLVTPLFNPIIYSLRNKEIWKALRRWSTVKVLT
QRSFLS*
>jgi|Xentr4|471655|C_scaffold_882000010
MNDSFVFEVVITGFEGPQNVKIIISAILCILYLMTVGGNSLIVVLVIICRRLHSPMYFFLCHL
AITDIIATTNIVPNLLYVTLLDRGTISLSNCLAQLFFLAIATAAECLILTIMSYDRYLAICRPLH

YMSIMNPKVQILLATSCWFGSFTVALLFVCSTSTLKFCGSNVINHLFCDLHPLLNLSCSDTF
IVDLEILVFGNLNVVVVCVFIVTTYIWIFRTILGTSMTTGRQKAFSTCTSHLTVVCTYYATLV
INYLVPLTGRSTDLSKYISLLFTVITPLINPFIYTLRNREIRSAFHYYIQKTMKLS*
>jgi|Xentr4|471658|C_scaffold_882000013
MKGINQTSVIDIVIIGFEGPLRLRILMTVILFLLYLMTLGMNSLIIVLVTISRRLHSPMYFFLC
HLAITDIIIPSIIVPNLLYVTLLDRGTISLSNCLAQFFFFGMAIDFECLILTLMSYDRFLAICRPL
HYMSIMSLKFQILLATSCWLLAFTLALLVLSHMLTFRFCDSNVINHLFCDLHPLLKLSCLDT
FVVDMEVLVAGILLTVLAFVFIVVTYVWIFYTIFGSSMTTEKQKAFSTCSSHLTVVCTYYAT
MITNYLIPVTGRTTDLAKYISLLYTVITPLMNPIIYTLRNREIRSALNYYTH*
>jgi|Xentr4|471698|C_scaffold_894000002
MSVKCVGYCLSAVPPINSACKLQIIQTHEDYEYIQEGDIIIGGVITVNALARHDDSEEPGKF
VYCYKPSLQNYKYLLDFLYLIGEFNKDEYVSQNLTLGYHISDSCGDVYKAEKSVLQILSGT
REPVPNYSCAGKRNIVGFIGDLTSETTIPIAHILSVLGYSQISYGATDPFLSDRATFPYFFRTV
QSKKGQYFAISQLLKYFGWTWVGIITTDDINGDESYQLLSNYLSSEGICIEFSLKLYNQNIP
YEELQHKSTSVVIFCGTINLKTAVTLWELPDIISDKTFIFTSDLIYYSYLIYYALTLFNGSLIIIQ
NRADYIRTNARLRQFIASVHPSMHPEDKLLEAIWLLYHSCMAENMSLTSIHKRIYPPYLPD
CTGEERLTDLQAFNDDFHTRNMIIAVQAMILESSKMHYRQNLITEQESAKNRKEKKRYNY
WGLYHLLKKMIFPYEGQFLKSFNENGELVSPYHINNLFITNDYLSDKKIGLYIPWAPPNQK
LNITHNEIIWKTESNKVPRAQCSDNCLPGFRKALKPGAQSCCYDCVLCSEGEISNRTDSEN
CFRCPDTEWPNANKNVCIVKGAEFLSYTNDVISVFFSSISVLLFVITLLILGIFIIYRDTPIVR
ANNRSLSFLLLVSIKLSFLSVFLFLGRPVDITCMLRIITFGITFSIAVSSLLAKTIMVCVAFKAT
KPGSSWRKWLGVKLSNSVVLFCSSIQIIICMTWLAISPPFQELDIHTSPGTIIIQCNEGSAIGF
YSVIGYMGLLAAVSFVLAFLARSLPDSFNEAKYITFSMLLFCSVWITMIPAYLSTKGKNTV
CVEIFAILTSSAGLQGCIFLPKCYTILFRPEINSKSFLRVNKFC*
>jgi|Xentr4|471710|C_scaffold_899000002
MVSENKENVSGFIIQGFSDTPELHISLFVLFLGIYLIILLGNLIIFLVISCNPHLHTPMYIFLQN
LSLIDISSTSNIFPNLLHILLTQQNSISFLGCMTQMYVFASLTCSEYFLLTAMAYDRYVAICDP
LHYIARMSRKHCAGLITAAFTVGFCNPVGIAVCVSKLSYCASHHINHFFCDVTPLLKLSCS
STFSVEFFIYIETILLGFSSFLLTLASYIFIISAILKIQSSEGRQKAFSTCASHLACVITLYGTAFC
LYMRPPTSYSLERDKYFSLLYIALGPVLNPLIYTLKNREFQSSINKMLSFFVLDRC*
>jgi|Xentr4|471714|C_scaffold_899000006
MVSESKENVSGFIIQGFSDTPELHISLFVLFLGIYLIILLGNLIIFLVISCNPHLHTPMYIFLQN
LSLIDISFSSTVLPNLLHILLTQQNNISFLGCMTQMFVFLSLNCSEYFLLTAMAYDRYVAICD
PLHYIARMSRKHCAGLITAAFTVGFGEIVSPVVLISKLSYCASHLINHFFCDATPLLKLSCSS
TFSVELLIFIEGILLVFISFLPTLISYIFIISAILKIQSSEGRQKAFSTCASHLACVITLYGTVLCL
YMRPTSSYSVKRDKYFSLLYIALGPVLNPLIYTLKNREFQSSFNKAKQGLLAFFLF*
>jgi|Xentr4|471770|C_scaffold_913000001
MTTERKENVSGFIIQGFSDTPELQTTLFVLFLGIYLIVLLGNLIIFLVISCNPHLHTPMYIFLL
NLSLIDISFTSTVLPNLLHILLTQQNNISFLGCMTQMYLFVAFAASEYFLLTAMAYDRYVAIC
DPLHYIARMSRKHCAGLITASFTSGFVGTVGHLVLISKLSYCASHFINHFFCDMAPLLRLSC
SSTLSVELLFYIEGTLLFFNAFLLTLTSYIFIISAILKIQSSEGRQKAFSTCASHLACVITLYGA
AFCLYMRPPKSYSLKSNKYFSLLYIVLGPVLNPLIYTLKNKEYQSSLNKMRQRCLPFDF*
>jgi|Xentr4|471822|C_scaffold_926000006
MDTPNDGNTSYFIIQAFSDSNRLQIPIFVVLLLIYIIILMSNMTVLLVILVDSHLHTPMYIFLIN

LSFLDISFTSNIIPHLLHSLITQRRVISFMGCMIQMYFLLSFFCTEFILLASMAYDRYVAICHP
LLYVFRMSQKHCVYFVSAAWIIGFLDPTSYVVLISKFSFCSSNIIDHFFCDLAPLLKLSCSDT
FPIEILNYVESALVTLNSFVLTVTSYIFIISAILNIKSAEGRHKAFSTCTSHLTCVIIFYFAIISLYI
RPVSTYAPKQDQYFALLYTVLIPLLNPFIYTLKNKEFRAALGKLRRSRLSFYS*
>jgi|Xentr4|471823|C_scaffold_926000007
MKMASENVSGFIIQGFSDTPELHISLFVLFLGIYLIILLGNLIIFLVISCNPHLHTPMYIFLQNL
SVIDIFSTSNIFPNLLHILLTQQNNISFLGCMTQMYVFLSFGASEYLLLTAMAYDRYVAICDP
LHYIARMSRKHCAGLITAAFTGGFGESVGLIVLISKLSYCASHLINHFFCDISPLLKLSCSST
FSLELLMYIEGTLLAFNSFLLTLISYIFIISAILKIQSSEGRQKAFSTCASHLACVITLYGTLLC
LYMRPTTNYSLTRDKYFSLLYIVLGPVLNPLIYTLKNREFQSSLNKMRQRCLAFLF*
>jgi|Xentr4|471827|C_scaffold_926000011
MVLEIKENVSGFIIQGFSDTPELHISLFVLFLGIYLIILLGNLIIFLVISCNPHLHTPMYIFLQNL
SLIDISFTSTILPNLLHILLTQQNNISFLGCMTQTYVYVALAASEYFLLTAMAYDRYVAICDP
LHYIARMSRKHCAGLITAAFTGGFVESISLIVPISKLSYCASRLINHFFCDVTPLLKLSCSSTF
SVELSIYIIAILLTFNCFLLTLTSYIFIVSAILKIQSSEGRQKAFSTCASHLACVITLYGTVFCLY
MRPTTSYSLERDKYFSLLYIALGPVLNPLIYTLKNREFQSFFKKVNARKL*
>jgi|Xentr4|471866|C_scaffold_942000003
MNEKNQTWVSEIVLLGFQNLHNFKVPLFSLFLLIYILTVWENVLIIVLVAFSRNLHSPMYFF
LQQLSLSDLLQTATVVPILLQTVINNGFAMSLIGCISQLYFFAYSETFQSFLLAVMAYDRYVA
ICIPLRYTSIMSHRVCVQVILTSWGISLCSTLVTVNLIGSLQFCENKTIDHYFCDVDPLLQMS
CSDTFFVQIDIILLSIPVIICPFILISVSYMCIAHAILKIVSHTGRQKAFSTCSSHLAVVSMFYG
TLIAIYLAAPSKQSLAIIKALSLIYTVVIPWLNPLIYSLRSTDIKEAFKNYLNKYPLCL*
>jgi|Xentr4|471867|C_scaffold_942000004
MFMKNQTWVSEIVLLGFENLHNFKVPLFSLFLLIYILTVWENVLIIVLVSTSRNLHSPMYFF
LQQLSICDFLESTNIVPTLLQTIIYDKIMLPFVGCMIQFAFFSATEAFECLLLAVMAYDRYVA
ICIPLRYTSIMSHRVCVTFILASWILGLICPLLLANIMATLQFCDQNTINHFFCDFFPLLELSC
SDTFVMQITITLQSIPVVFLPIILITVSYMCIAHAILKIVSHTGRQKAFSTCSSHLAVVSMFYG
TLIAFYLVPPQKESQTTSKVLSLLYTVVTPFVNPLIYSLRSKDIRESLYHLRGRFTKIL*
>jgi|Xentr4|471868|C_scaffold_942000005
MSMKNQTWVSEIVLLGFQNLHNFKVPLFSLFLLIYILTVWENVLIIVLVAFSRNLHSPMYFF
LQQLALTDLLGSTIIVPTLLQTGIYDKVMLPFVGCIIQFSFFTATEAFECLLLAVMAYDRYVA
ICIPLRYTSIMSHRVCVTFSLMSWLLGLISVLLLGNIMVTLQFCDQNTINHFFCDFFPLLELS
CSDTSLVRLTNILLSFPVFFFPFILISVSYMCIAHAILKIVSHTGRQKAFSTCSSHLAVVSMFY
GTLIAIYVVPPRKESQTTSKVLSLSYTAVTPLINPLIYSLRNKDIRQALDHLTNNQKALGVT*
>jgi|Xentr4|471874|C_scaffold_942000011
MGEQNQTWVSEIVLLGFQNLHNFKVPLFSLFLLIYILTVLENVLIIGLVAFSRNLHSPMYFF
LQQLALSDLLEATDIVPTLLQTVMHDGTTLSLIGCIMQYYFFSTAEAFECLLLAVMAYDRY
VAICIPLRYTSIMSHRVCTKLVVIPWTLGFGISVITVKLIGTLQFCDQNTINHFFCDVFPLVEL
SCSDTFPLQIEAFFLSVPIIVFPIVVIIVSYMCIAHAILKIVSHTGRQKAFSTCSSHLAVVSMFY
GALTAIYEVPSQRQSQTISKVLSLLYTVVIPLVNPLIYSLRNKDLNIALKNLKVN*
>jgi|Xentr4|471877|C_scaffold_942000014
MDEKNQTWVSEIVLLGFQNLHNFKVLLFSLFLLIYILTVWENFLIIVLVAFSRNLHSPMYFF
LQQLSLSDLLQTATVVPKMIQTIIKEGATTTFGVCIIQFYFFAGWEAFQCLLLAVMAYDRYV
AICIPLRYTSIMSHRVCVGLILFSGFLGFGTVLITVNLIVNLPFCGLNIINHFFCDLSPLLELSC

SDTFLVRIEITLLSIPVIICPFILITVSYMCIAHAILKIVSHTGRQKAFSTCSSHLAVVSMFYGT
LIAIYVVPPRKESQTISKVLSLLYTVGIPLGNPVIYSLRCQDMKEALKKSLINVACCAPTH*
>jgi|Xentr4|471958|C_scaffold_964000005
MENEFNTSKSFVLLGIEEMERFKYLYCSLLLLIYFFILLFSCTIISVVLLDESLHEPMYTLIAS
LLLSGIFGSSCVFPKLIADLLLSSKEISRVACFTQTFSVTLFGYFEVSTFTIMAHDTYIAVGNP
LRYPTLMTNSVVFKLMMGSLIFTVILTLPVPLLSARLPICGSHIRNVFCDNPSILILSCVDTS
MNKLYGNVSFVSYVMVMTLLIAYAYLRIYLICLKISKDASKKAIHTLVTHLLNFSIFMAGG
LFLFVRYRLGNTNLPLTYFTILSIMGFIFTPLFSPLIYGIRMQTLKVRIIHHLRQRVFAR*
>jgi|Xentr4|471960|C_scaffold_964000007
MERFNYLYCALSLLAYLFILLCSTIIILVVLQEESLHEPMYTLIANLVLNGIFGSSTFFPKLTV
DLLLSYKEISRAGCLIQSFCVLFFGYCEISLFAIMAYDTYWAVCHPLHYAAIMTNRKILRLV
LASLFMNCLLVGTAVLLSARLPLCGVHIKSVFCDNTALFVLSCVDTSVNKIFGTINFTCFLS
VHLLLIAYCYVRIFLTCLKVSENAGRKAAHTLVTHLLNFSIFLVATLFIFIRYRLGDINIPVIV
HVILSASGLIFPPLLNPLIYGVRTKALKVKVLCYLQRTNILVYN*
>jgi|Xentr4|471962|C_scaffold_964000009
MMENSSNVSTSFVLLGLVEMEGFWYLYCLVCLVTYTFIMVLSAIIVLVVVTEESLHEPMYL
LICTLVLNGILGSSTFFPKLIIDLLSSSKTISRAPCLGQAFCVLFFAFFEICTFTLMAYDRYVAV
CYPLQYVLIMTIEKTLRFIVASFTFTFTANLVAIVLSARLPLCGSQIRNIFCDNMSFFILSCVN
TSINNIYGATVFMMFLVFTLLVIAYSYIRIFLICMKISQHAYEKAIHTLVTHLVNFSIFLIGVLF
IFIRYRLGNVDLPLGVQILLSATCLVVPPLLNPFIYGIRTKALTMKISRFLNK*
>jgi|Xentr4|471963|C_scaffold_964000010
MENVSSVSRHFVLLGLVEMEDLRYLYCILSLFLYIFILLLSLGIVLVVLTEESLHEPMYIFICN
LTFNGMLGSSSFFPKLIIDLLASSHQISHVGCFLQVLALMIYVFFEIFSFTLMAYDRYLAVCD
PLRYATKMTKAKAVRLILGFFAFSFFSVLVGVILSARLSYCGTQIKNIFCENLSLIVLSCGDS
SVNSLYGISVTVILLVFTLLIIAYSYINIFMVCLEISKEACEKAIHTVVTHFLGFSIFLVGGLFIF
VRFRLGNNNLPLFAQILLSVTFIVFPPLFNPLIYGIRTKALRVKIFHHLHKMRILGGN*
>jgi|Xentr4|472006|C_scaffold_976000006
MMDTTSELNTSFSHTDFLLMGFPGIAVSRPILVIPFLFIYIGILMGNSLLMYRIWVEPSLQYP
MYWLISLLFAVNLSCTTSIMPKFLLGLAFGLNQISLSGCLIQMYLIYSAIVFESALVLIMALD
RFVAICRPLHYHAIMTKHLLMWLNVINVARVLLLVSPIVVSFMKVPFCRSNTILSFACENM
GLLSLGCGDISKLQIIGLVVRILVSVVDGGILFISYIKILYTAMKLVSGKAHNKALSTCGTHL
MVAALIYSCGLLSSIVYRLGTSVSINVQNTISAIYYLFPAAVNPIIYGLRVSEIRICLEKAYGR
RIKNNDGEGHGN*
>jgi|Xentr4|472078|C_scaffold_999000008
MALESQQNFSGFIIQGFSDTPEPQISLFVLFLGIYLIILLGNLIIFLVISCNPHLHTPMYIFLLNL
SLIDISFPSDILPNLLHILLTQQNNISFLGCMTQMYVFGSLASSEYLLLTAMAYDRYVAICDP
LHYIARMSRKHCAGLITAAFTGGFGGTVGFIVLISKLSYCASHLINHFFCDVTPLLKLSCSS
TFSVELLMYIEGTLFTFSSFLLTLASYIFIISAILKIQSSEGRQKAFSTCASHLACVITLYGTVI
CLYMRPTTSYNIKTDKYLSLLYIVLGPVLNPFIYTLKNREFQTSLNKDGRL*
>jgi|Xentr4|472124|C_scaffold_1018000001
MTLNITSMDEDVLLAERDSSFRVLTGCFLSVLILSTLLGNTLVCAAVIRFRHLRSKVTNFFV
ISLAVSDLLVAVLVMPWKAVAEIAGFWPFGTFCNIWVAFDIMCSTASILNLCVISVDRYWAI
SSPFRYERKMTPKVAFIMISVAWTLSILISFIPVQLNWHKAKTTSFFDLNITLHGRTMDNCD
SSLNRTYAISSSLISFYIPVAIMIVTYTRIYRIAAKQIRRISALERAAVHAKNCQNSTGNRNSL

DCQQPESSLKTSFKRETKVLKTLSVIMGVFVCCWLPFFILNCIVPFCDPSLTTSGTEPFCISS
TTFDVFVWFGWANSSLNPIIYAFNADFRKAFSNLLGCYRLCPTSNNVIETVSINNNGAVVY
SCQQEPKGSIPNECNLLYLIPHAIICPEDEVLKKEDESGLSKSLEKMSPAFSGILDYDADVSL
EKINPITQNGQPKT*
>jgi|Xentr4|472208|C_scaffold_1063000003
MKSDYLQTILCHLYLWSFMTIALTLTSAKGEVNRQGCGLSTSISPYSYTRDGDINFGGILQI
FIHAAWVIQDFSEEPYPVKCFLPSLQYLHHVIAFIYAIEEINNSAEILPNITLGFQIYDSCTSE
VIALLSTFSILSERPEPVLNFICQKRQKTVAFIGHMLSSASHTIAGITQLYRYPQVSYGALDPI
FNDRVRFPAMYRTVPNEYSQFKAIIQLLTHFKWTWVGIIASDDDSNLQASEELSKQLKQSG
ICVTFLEKTPKPINSNEKDILRVISMIERTSARVIILYCSTESFLSLLTNLNQVPRITWITSVTL
NIITDLPSETNLHEFSAFNGSLAISVSSEEMPGFQIFFSEATLGKMPGNTFIEKYWLFNHGCV
NISVDSNGNQCLKEGYIKDTLMLDNKYRITYTIYMAVYALAQALHEMHSSASLLTHLPEV
SSGKIRMKLNHYLKNVHLRTSSGMAFHFDENGNIPGMFDIINWNIYPNETITRTQVGSFLIS
RVPQLLINDSRITWNPHFNQTPFSSCTVPCTYGFRTAHQTGKAPCCFDCIPCPDGKIANGTN
MENCWSCPEDKWSNPNKDKCIQRTKDFLSYRCILGATLASIASIFLVITAAVLFVFVKFRRS
PIVRANDRNLSFILLVSIMLSFLCSFLFIGYPVELTCMLRQAAFGFIFTVAVSAVLGKTVTVII
AFNATKPNSTFRKWVGTRVSISLVLLFSFGELFICIIWLICSPPFVDTDSKTIPGTMIIQCNEG
SFTAFYVVISYIGLLALFSFIVAFLVRKLPDRFNDAQYITFSMLVFCSVWISFIPIYLSSKGKY
VVAVELFAILSSTGGLLLCIFAPKCYIILVKPELNSRKHLLKPKP*
>jgi|Xentr4|472211|C_scaffold_1066000002
MYLTALPMVPCRSVDQPNNPVCHLNLVVKTSEKYEYLEEGDIMIGGVLTVGTRGTPSAQK
YRYLVDFAFCVKEINEDPARLPNMTLGYHIHDSCGDTRRALRSLMQIVSGTREPVPNYSCR
RKGRMAGFIGDLLSEPTVSIAHILSVLGYSQISYGATDPALTDRATFPYFFRTLQSDEEEFIA
LCQLLKHFGWNWVGIIYTDNESGERDYQLLTKYLSREGICTEFGMKLNLKLEKKKSLKKT
KTRVVIICGDPNFEFEPFSDTLPKLIRQKTCIFLSKWLNQYESLELASLLTGTVFFMQNRLD
EQYGARFREFSDTFHPSKYPHDDLLQHMWLWHHQCVLKDDGKSMYYIYNFIATKNCTG
KEKLTDIPEYLSSYHSASLIQAVDMMALALQDMHNFHSKQTHGKGRRMDNYNYQLHRY
LRHVPYAIDGSPVSSFNEKGEFVHQYDIINPFFDPGGKMSWKPVGSYVPWAPVEQRLILNS
DKIIWNTPNHEVPRAQCTDNCLPGLRKLIVPGTLTCCYQCAPCPEGEISNKTDSENCISCPD
MEWPNKKRTFCIAKTEAFLSYTNDVISVIFSSISVFFFVITVMILGVFIINQDSPIVRANNRSL
SFLLLVSIKLSFLSVFLFLGRPVDITCMLRIITFGITFSIAVSSLLAKTIMVCVAFKATKPGSSW
RKWLGVKLSNSVVLFCSSIQIIICMTWLAISPPFQELDIHTSPGTIIIQCNEGSAIGFYSVIGY
MGLLAAVSFVLAFLARSLPDSFNEAKYITFSMLLFCSVWITMIPAYLSTKGKNTVCVEIFAI
LTSSAGLLACIFLPKCYTIFFKPEMNMKSQLFGNKQH*
>jgi|Xentr4|472353|C_scaffold_1115000012
MENQTIEYTLVLTGLSDLPSLQVPLFLVFLLVYLFTLTVNLLILLLIFTDSHLHTPMYFFLGT
LACLDMSYSSVTAPRMLFDLLTGRGDISVPACITQIYFLIFFVASEVSVLAVMSYDRYIAICR
PLHYMQIMSWKFCVQLVSGVLVLSAIYPLVHTLFLTKLTFCRPDALQSFFCDLPQLLEASCS
DTFINVLLIFLLGILFGGVFFGVIFYPYISIIMTVLKIPSINMRAKAFSTCSSHLTVVFIFYTTIF
FNYFSSNAKYNVTEDKVVSVFYAILTPFLNPMIYSLRNQELKTSLRRALHRL*
>jgi|Xentr4|472475|C_scaffold_1190000002
MLGNALPGPNQQGYRYLADFLFAIKQTNKDPARLPNLTLGYHISDSCGDPRKAVRSVLQIL
SGTREPVPNYSCVGKRHIAGFIGDLTSETTVPIAQILTLYGYSQISYGATDPSLRDRAAFPYF
FRTVQSDEANYFAISKLLSHFGWTWVGIITSDDISGEREHHSLAKYLSREGVCIEFTIRINTN

NEHTMKQNQYGITIQGSSTSVIILSGTASTILVVMLSLLNNVLPEKTLILSSNWGNNDIVVG
YALQIFNNSLVFVPRYHYDLGTPEMSRFLEDLHPSKFPDDDLIEDIFLMFLLCLSKDQNKN
NLHQYIYGNILLNCTGQERVTELYYFSGETNSPRVHLAVDIMSQALHEMLFIKDSIQYPYK
YQALHRYLKKHQYSTQTGPTFLFDEHGEYVSGLRIYNYIISADYYLKRKLFGEISPWAPPD
QQLNIKSSLIQWKTKNNKIPRAQCSDNCPPGFRKALKPGAQSCCYDCVPCSEGEISNTTDS
VSCTRCPDMEWPNEMKNQCIRRNEDFLSYTNDLISIFISVVSVLFFLLTQLILVVFIQYRDSP
IVRANNRSLSFLLLVSIKLSFLSVFLFLGRPVDITCMLRIITFGITFSIAVSSLLAKTIMVCVAF
KATKPGSSWRKWLGVKLSNSVVLFCSSIQIIICMTWLAISPPFQELDIHTSPGTIIIQCNEGSA
IGFYSVIGYMGLLAAVSFVLAFLARSLPDSFNEAKYITFSMLLFCSVWITMIPAYLSTKGKN
TVCVEIFAIVTSSAGIIACIFLPKCYVILLRPERNTKTNLLHFC*
>jgi|Xentr4|472535|C_scaffold_1233000003
MSGDKFTVPNYHCGLSQDLLAVVDGISTRLSLLFARLFGIYMVPQISYNSLDPILRDKIHFP
TVYLTIPSDNFQYLAIVKFLKHFSWTWVGILVSDDEFGLKISQLLLEELARNGICVAFLDFL
NQRSLSSTDKIAKNINLTNVIILYCDKDYIFALQKALYLYPVSEKVWIISSQWDVLTGNDFY
FTGIGPFNGSLAFTPYKGAMPGFKEFISSIKPELYPKDIFIVDAWFELFQCTYNKTLSLCTGD
EKTQYERFNEEVAHNSYSIYNAVYVLAHALHLMQLEKMKENRNKNDNKPWQLNKYLK
NIHFTNGGGETMLLNENGEIHSKFDILNWVIYANQTLRSIKVGSYDHQNTVQGLIVNESLI
RWNPAFNQTPRSACSETCINGFRRTSKQGFPACCYDCIPCPEGWITNTTDMKFCIKCPQTQ
WPNEKKDTCLDTVIIYLSFADELGTSIAFLSILFFFFTCLVMGVFTKYRTTPIVKANNQNLS
YVLLFSLKMCFLCPLIFIGQPIKLSCMTRQTVFAITFSISLSSILAKTITVVIVFHATKPGSKLK
NYIGSKGSVSFTVFCSLVQVVICACWLGISPPFPQYNMEDEVGKIIAECNEGSLIGFYCVLG
YLGVLASVSFIIAFLARDLPDTFNEAKFITFSMLVFCSVWVSFIPAYMSAKGKYVVAVEIFAI
LASSLALLGCIFIPKCYIILVKPECNTRDFVKKGNCIILK*
>jgi|Xentr4|472537|C_scaffold_1235000001
MSQCFYNESIKFFYNNSGKELYDTWRTKDMLVVGLGLTVCVFVLLFNLLVIAAIIINRRFH
YPIYYLLGNLAAADLFAGVAYTYLMFHTGPNTSKLTIKTWLVRQSLLDTSLTASVANLLAI
AVERHQTIFTMQLHSKMSNQRVCILIACIWIIALLLGLIPSFGWSCICNISTCSRMAPLYSRS
YLIFWAVSNLVAFLIMMGVYMHIFIYVKRKMTRMSQHTSSQPKYRETMINLMKTVFIILSA
FVICWTPGQVVLLLDGLNCKSCNVLAVEKYFLLLAEINSVINPIVYSYRDNEMRNTFKQIL
CFFCNRGSKYSPTSLKSTNTERRILSDNGHLMRECSL*
>jgi|Xentr4|472553|C_scaffold_1244000001
MFKKYETNWCFGRLVIKIELPHSPACRAFQVPTGKKVSYGATEPVYNDRIQFPSFYRTVPN
ELSEMDGIVQILKHFGWKWVGLIVSDTSNGERALHRIRNGIESYGGCVAFSVSLPETSFLS
WLFLYFDVERILDTINQKNVNVIVLFLTPMHINVFIAVFSSRQITRKIWLSSSFFPTVAPHIHG
YRRTTLNGTLSLSAHGGEIPGFETFLYRMTPSNYPNDDTVTEIWETLHECSFTGSLKTNTS
VPVQKCSGNESLDDEALSRFGNFDFRTGYQVYTAVYALAHSLHNLFSARAPAHPQAPAGS
LKHRFKPWQLNAFIHNVTYKTPSGDIMFFKANGDPPARFDIVKWLFLNDGSTVSRKVGSF
DESNEENQLYINSSADWWSPYFPEMPRSLCSEPCSPGYRKSKIEGEPPCCYDCVQCGDGE
MSNTTDAGTCVQCPKDQKSNRQKTDCVPKALNYLSYMDTLGASLASAAIILFITASVVM
GIFVKYWETPIVRANNQNLSCLLLTSLMLCFLCTLLFIGRPTQICCLLRQVTFGIVFTISVSS
VLAKTLTVIIAFNATKPGSKATRYVGTQLSIFIVFACSLGVTLICIVWMASSPPFPEADTSSET
DTIILLCNEGSVTFFFCIIGYIGTLALLSFIAAFLAKDFPDRFNEAKNITFSMLGFCSVWGAF
VPAYLSSKGSRMVAVEIFAILSSSAGLLICIFAPKCYIIFLRPELNIRETVVKRS*
>jgi|Xentr4|472568|C_scaffold_1254000001

MQEGDIIIGGVMTSHFYMINVTFPWDNSNGFLCISPNQQGYRYLVDFRYAVEQTNKDPARL
PNLTLGYHIYDSCGDPRKAVRSILQILSGTREPVPNYSCVGKRNIAGFIGDLTSETTVPIAQIL
TLYGYSQISYGATDPLLSDRVTFPYFFRTVQSDEANYFALGKLLSHFGWTWVGIITSDDISG
EREHHSLAKYLSREGVCIEFTIKINTNKEHTMKPNQYGIIIQGSSTGVIIISGTASTTFVIQLPI
LNNVLQEKTLILSSNWGNNNIVVGYAVDIFNYSLVFVPRYHYDLGTPEMSRFLEDLHPSKF
PEDELIEDIFLMFHSCLSKDKNKNELYKYIYGNTLCNCTGQERITELYYFIGTNSPRVHLAV
DIMSQALHEMLFIKDFIQYPYKYQELHRYLKKHQYSTQTGPTFLFDEHGEYISGLRIYNYII
SAEGYLNQEPIGEFLPWESPDQQLNIISFIEWKTNTNEIPRAQCSDNCLPGFRKAPKPGAQS
CCYDCVPCSEGEISNTTDSESCIRCPDVEWPNEEKSQCIAKMEEFLSYSDDVISVICLFISVL
FFLITEVILGVFIKYQDTPIVRANNRSLSFLLLVSIKLSFLSVFLFLGRPVDITCMLRIITFGITF
SIAVSSLLAKTIMVCVAFKATKPGSSWRKWLGVKLSNSVVLFCSSIQIIICMTWLAISPPFQE
LDIHTSPGTIIIQCNEGSAIGFYSVIGYMGLLAAYPYKYQDSVWITMIPAYLSTKGKNTVCV
EIFAILTSSAGLLACIFLPKCYTILLRPELNIKTSLLRN*
>jgi|Xentr4|472598|C_scaffold_1274000001
MDITNQTDIPGFVLRVFGGSSKFHPILCLILFLCFFLTLAINLLVIIIICMDRHLHSPMYFFIAS
LSFMEICGISSVIFNLLAILLTNKTHISKAGCILQSYIYYFFSTSDFFILGIMSFDRYVAVCNPL
KYNSIMRNSVCVRLVIGCFVTSFLCLLYPTLMITNLPFCGHMLDHFFCESAALMNLICGDIT
LIKLTSLITSVFILIGSLTLTATSYIIIVSTILRLSSDTGRQKTFSTCLSHLTMVGIVFGSAIFIMIR
PHRRYSTQTDQVVNLVSTVMGPLLNPFVYTLRNQKVKDSIRGAMNCNRMPQK*
>jgi|Xentr4|472654|C_scaffold_1310000002
MVCPLLLLLIPLPFVHVLGQSGCLLSPPTLTAYTEEGDVTLGLIVPINTISSRKVLSFKDRPAP
PQCDLFRRDQYKSILALLFAIEEVNKDPHLLPNVTLGFHMLDSCFAEDFSLRATLQAVTGN
NKNAPNFKCAPRTKVPAILGDPLSTSSVPMARVLGLWRVPQVSYAASFPSLSNKVEFPSFL
RTIVSTEGQPYALVELCKTFGWNWIGVLISSTDYAVQGGVVFKREAAKNGICIAYYETIALE
TISQRIPSVMKNVRGITATAIVLFCSPAEVSAILTEALQQNVTGKIWIGVEAWFTSPVFFKPE
FWNLLNGTIGISRSRRILPYFPQFLQDFYPSRYPRMLNIQQFWERTFNCKWTSANGTLGPNI
KACTGSERLITDDITEYNDPVSQYVYMAHSALFALANALHDLMACRPGEGPFSGGSCAN
KQDFQQWQLLYYLKKVQFVNTAGELVAFDVNGDSYGYFDILNWRLDGNLTSRYVNVGT
FNNYMNSEQKLSINASFILWADGLAKVPSSVCTESCPPGYRKAPRTGQPLCCFDCVLCPDG
MISTQINSIDCTSCPLDQWPNSERNQCLPKVIEYLSYQEPLGLVLALVSMTFSLMTVAVLCV
FLRFRDTVIVKANNRSLSYVLLLSLTFCFLSSLTFIGSPTAVTCSLRQSIFGVIFSLCVSCILAK
TIIVVMAFKATRPDSRLRQWVSSKTTVLVVSLSTMVQVILIALWLIISPPFLEHTVQSITGKV
LLECNEGSSPMFYSVLGYLGFLAFLSFVVAFLARSLPDNFNEAKYITFSMLIFLSVWVSFIP
AHLSTKGKYIVATEVFAIIVSGSGLLGCIFCPKCFIILLRPSMNTKEFLVSSRSNFD*
>jgi|Xentr4|472699|C_scaffold_1343000004
MENQTTVYNFVLAGLSDLPSLQLPLFLVFLLIYLITLTGNLLILLLIFTDSHLHTAMYFFLGT
LACLDMGYSSVTVPRMLFDLLRERRIISVPTCITQVFFFLFCAASELCVLAVMSYDRYIAIC
RPLHYMQIMTWKVCVQFVSGVFLFSAVDSLVHTLSLTKLTFCLPNVLQSFFCDLPQLLEAS
CSDTFINVLLIFLLGIPYGVGILGLTFYPYIPIITTVLKMSSTNTRSKAFSTCSSHLTVVFICYT
TFLYNYFRSNANDHLVEGKVASVFYAILTPSLNPVIYSLRNQELKLSMRRTLKKTAIVLKK*
>jgi|Xentr4|472857|C_scaffold_1444000001
MLGGAAGNNSLKVLIYIIIQCVKPCSSEPINPACHLQIIKAAEEHEYIQEGDIMIGGVMAVLP
EYVKLLIDFRFAVEQTNKDPARLPNLTLGYHLYDSCGSQLKAVRSVLQILSGTREPVPNYS
CVGKRNIAGFIGDLISETTVPVAQILTLYGYSQISYGATDPELSDRITFPYLFRTVQSIDSHYF

VMSKLLRHFGWTWVGIITSDDISGEREHQILAKYLSREGVCIEFTLTINANGLHFFNYKDIT
MIQRSSTNVIILSGTVSLILILKAGIFNNERAFYEKTLILSPSWGSTEAILYYAPKLFNCSLVL
VPRYHYDLDTHEMRSFLENSHPSNYPEDKLLKDIWLIYFYCLSEDQNKNDLYKYVKYYN
PTPHNCTGQERITDLPYFRGKYNSPRVHLAVDMMSRALHDMKSKGNNEMDIYKHQLHH
YLKKVQYRSKHGGTFSFDENGEYNTKYLIYSNFMADSITVIKSFLGEFFPWAPLDQQLNIT
FMLVQWKTKDNKIPRAQCSDSCLPGQRKMPIPGAQSCCYGCAPCANGEISNTTDCVSCIK
CPDMEWPNDKKNQCIRRNEEFLSYAKDLISAFISLVSVLFILTTQLILGVFMKYRDSPIVRA
NNRSLSFLLLVSIKLSFLSVFLFLGRPVDITCMLRIITFGITFSIAVSSLLAKTIMVCVAFKATK
PGSSWRKWLGVKLSNSVVLFCSSIQIIICMTWLAISPPFQELDIHTSPGTIIIQCNEGSAIGFY
SVIGYMGLLAAVREPVPNYSCVGKRNIAGFIGDISSETTVPIAQILTLYGYSQVREKNISDK
NIKA*
>jgi|Xentr4|472874|C_scaffold_1466000001
MLTNTKNTIILSPTYFYNHQASWRFRRHLTAFLFAVNEINENPDLLPNITLGFQIHDTCYSES
LSIESALRILSGTKYMVPNYNCHKRGGLAAIVGHLLSSLSLSISTIISNYRIPQISYGAMDPSF
SDREQFPSFFRTVPNQLSHHQAIIELLKHFGWVWVGIVASADESNAKSSSLLREQLISHGIC
VEFHEIFSVDKEETVNEQETKRTNVLIKKSTATVIILYCNIEYFDQLMIGEHWEEIKGKVFV
TSVTLTLILKNAFHVKTLYPLNGSLLFVVRRGNIHGMKDLMDYVPRRAIRHIEEMLFSLPK
DEEGNPLNKSNSEPFRLTYSIYTAVYAVAHAFHDMMIPHLNKVQFQTQSDGDFSFSDEGEM
PGEFDILNCIVSPDGEVRMVEVGSYRYHPAQFQIKDSAIVWGSPYTEAPRSVCSSSCLPGSR
KAKREGQPLCCYDCVPCEEGEVSNQTDMDYCMKCPEDQWPNKRRDRCLQRLVEFLSYQ
DPLGAGLGGASIGLSACAITILGVFIKFRATPVVRANNTNISYTLLVSLCLSFLSCLLFIGQP
KPLACMVRQAAFGLAFSIAESSILAKAVTVAVAFRATSPDSQLRRWVGPRLPIYIVIGCSVG
QAIICLAWMVLSPPFPDYDIQSEKGKMILICNEGSAVLLYVEISYLGVLALLSFTVAFLVRNL
PDRFNEAKYITFSMLVFLSVWVSFIPSYLSTKGKYMVAVEIFAILGSSTGLLACIFIPKMCLIL
LKRGLSSKVHVAGMDNK*
>jgi|Xentr4|472876|C_scaffold_1466000003
MSFLYNYRASWRFRRHLTAFLFAVNEINKNPDLLPNITLGFQIHDTCYSESLSIESALRILSG
TKYMVPNYNCHKRGGLAAIVGHLLSSPSLSISTIISNYRIPQISYGAMDPSFSDREQFPSFFH
TVPNQLSHHQAIIELLKHFGWVWVGIVASADESNAKSSSLLREQLISHGICVEFHEIFPVDK
EETKRTNVLIKKSTATVIILYCNMGYFLQLMIGEHWEEIRGKVFVTSVTLTLVLDNRYFLKD
VYPLNGSLLFVTRRGNIHGMKDLMDYVPRRAIRDIRAMLFSLPSDRKGNPLNKSNSEPFR
LTYSVYTAVYAVAHAFHDMVSKARTQPGFLSTGQWLRHFHPWQMIPHLNKVQFPTQSDG
DFSFSDKGEMPGEFDILNCIVSPDGEVRMVEVGSYRSHPAQFQIKDSAIGWGSPYTEAPHS
VCSSSCLPGSRKAKREGQPLCCYDCVPCEEGEVSNQTDMDYCMKCPEDQWPNKRRDRC
LQRLVEFLSYQDPLGAGLGGASIGLSACAVTILGVFIKFRATPVVRANNTNISYTLLVSLCL
SFLSCLLFIGQPKPLACMVRQAAFGLAFSIAESSILAKAVTVAVAFRATSPDSQLRRWVGPR
LPIYIVIGCSVGQAVICLAWMVLSPPFPDYDIQSEKGKMILICNEGSAILLYVEISYLGVLAL
LSFTVAFLVRNLPDRFNEAKYITFSMLVFLSVWVSFIPSYLSTKGKYMVAVEIFAILGSSTGL
LACIFIPKMCLILLKRGLSSKVHVTGMDNK*
>jgi|Xentr4|473025|C_scaffold_1583000001
MPLGIMTSWCIMGIFYSHYDTQLGCGHFRHHLVVIYAVEEINSNEKLLPNVTLGYHIYDSL
AYESQAQKHIFRILSGGRNMIPNYLCSSGGVLAAFIGDKSSPTSLSMATLIRIYHYTQISYGA
MDPIFTDRLRFPPFYRTVPDGYAQHGAIVQILQHFGWNWVGILASDAESDQMGSWELKG
QIMRSGLCVEYLVTVSGKFSRKEGEQKRVESVLRRATAKVIILYNSSDNVKWFLLMNKRM

ELPDAVWINLGLDFYPTDHGFEVIFFNGSLGLSIKRGNIPGFTDFLYSLSPQKYPDNMRLKW
AWSWTFRCHYNGTNPEELPWETLPCSGSESLLDLPQNKYDAYNFRTTYSIYTAVYALAQA
LHAMISEAPSKAKGTVGLKKGFQSWQLRHYAQNVGFATASEEFFFDEYGGRPARYDIVNW
AVLPDLSVTQETVGHFDSTAPESQRLLINDSAIRWHHRFTQLPQSLCTEPCEPGYRKSPQEG
KPPCCYACVQCPEGEISSTMDTDSCLACPEDHWSNQGRDQCIPRIRDFLSYEDPLSAALVC
VALVLTLITVLVLGVFILYRDTPIVRANNRNLSYMLLLFLIMSFLCSLLFMGPPDNLTCQLR
QVSFGIVFTGSVSSVLAKTITVILAFKATKPGGKLRKWLGNRMSFSLVLICSLVQALLCTM
WLIRSPPFPDYDTWSVKGQIILQCNEGSVPAFYMVVGYMGFLAALSFLVAFLVRKVPDRY
NEAQLITFSMLGFCSVWVSFIPAHLSTKGKYTVAVEIFAILASGAGLLACIFVPKCYIILIRPE
LNAREKPMIIRGDISAER*
>jgi|Xentr4|473058|C_scaffold_1623000001
MHEKNQTWVSEIVLLGFQNLHNFKVPLFSLFLLIYIVTFCANVLIIVLVPTSRSLHSPMYFF
LWQLALTDLLGSSSRCPNPPPYSVMAYDRYVAICIPLRYTSIMSHRVCLKLILVSWAINLVSS
ISTMYMITALWFCKVDTINHFFCDLYPLLDLSCSDTFFVKLDVSLQSVLVFSSPVILIIVSYIC
IAHEILKIVSHTVRRKAFSTCSSHLAVVSLYYGTLIATYMAPPVKKQTQTISKVLSLLYIVVI
PLLNPLIYSLRSKDLKEALTKILVIGTSISTN*
>jgi|Xentr4|473246|C_scaffold_1836000001
MVGFFGSCFALWAFTRSDSSQKCTSIYLINLLIADFLLTLALPFKIVVDLGIATWKLKIFHCQ
VTAVIIYINMYLSIIFLGFVSMDRSLQTINSSRLYQVQKRGFAKMISAVVWTLKGILLAALTF
RTKLEGTGHVLSNFISLAIFFNCSCIILISNFLTIKRLYRHKDCDEFLNIKQALVQILLVTAGY
VICFVPYHIVRIPYTLSQSNIIMDCSLKQVLFFAKESTLLLSVSNLCFDPILYFYFSKTFRTKIT
KTFSMKKEVTAV*
>jgi|Xentr4|473325|C_scaffold_2010000001
MQANQSRYFIFVFDGLTNNPTIERVLFIIFLLIYIFTILGNGGLIVLINKSPILHTPMYFFLKHL
SFIDMCYTSVIIPRTLSDLLSKKKTITLLACVVQMYFYASLFISEVYLLSAMAYDRYVAICQP
LLYHVMMQKEKCIILIAICFAIGFSKSVIHAVNTFSQVYCNNHHIAHFFCDMPPVLKLSCSD
TSVTELLVFAVVGFNSFVCISVIVISYSYIFSTILTIQSAQGRLKAFTTCSSHLISVGTLFGTLM
YMYLRPNSSYSNDQDKVVSVFYTMVIPMLNPVIYSLRNKDVHNSFVKLMRLKTGGQL*
>jgi|Xentr4|473364|C_scaffold_2098000002
MCVFNASNYSNSFILTGIPHLQDQFWVGFPLLLMYLVAIVGNCIIIYIIKVEEDLCEPIMLGIF
WFDSTEIFFDSCLLQMFFIHFLSALESGILMAMAVDRYVAICHPLRYSSVLTESTLIKISLTIL
LRGALFMIPIPLLIKRLPFCQKNVLTHSYCLHQEIMNMTSADNKVNVVYGLFIILFIMGMD
SIFIALSYLLIIRAVVDLVEEASLKAFSTCAAHICAVLMYYIPLIGLSVVHRFGINEEFPNLHIF
FGNVYLLVPPVINPIVYGIKTKEIRSRLRKIFCSKRHWIKTSGDL*
>jgi|Xentr4|473494|C_scaffold_2456000001
MKFYNDISSSRGCDSLSCCGARIMGTVSGSSVWHKVLQAADTQDPQRAFPAEFQLIQVGS
PSISYFRHFLAFIFAIEEINRRTDILPNVTLGFMIYDSCQWEKKAVYSALSILSGVDSMVPNY
KCQIYGDVVAFIGHLLAYSSFSLAQLNEIYKYPQISYGTMESYFDDKIQFPYFYRTVPNDQ
AQFHAIVQLLQHFGWNWVGLITSYEDQGDQTSVELEKFLLQNHICLDFKYTVSNLLLDG
DPVEFKSEVIILYISSSSFTKLMFELSFNSTFGKIFILPASLQIHYQVSRDTFLNGSLMFRLHN
RNIPGLRDFLLRDGPREFPENPFLGAIRKEKRLCLPESFEPDPFIREATCNDTDSLNIMDPLI
YDVENFRLTYSIYAAVYAVAYALHHLYSHRSHQEKWELRPWEINRYLRNLHFSTPGGEEVT
FDDKGNVVATYDILNIIQFPNKRVEYVLVGHVYPAEGGTRLVINDSAIRWDSRYARTPNSLC
NPSCPPGHRKVHKERAKCCYDCARCPEGEISNGTGHAPMISGRMKAETLVRMEFLSYEDP

LGFSLAILAAIVSSLAAGMLGIFIKHKDTPVVKANNQTLSFILLLSLMFCSLCPLLFIGRPTEI
SCLLCYISFGTFFTVAISTLLAKTFIVTLAFRVVKPDRKVRGWLWKNMFMIVVLLCFCGEG
LIGTTWLAHSPPFPDYNTKLEADKIILRCNEGSVIAFYVQIGYMGLLASFSFIVAFMARKLP
DAFNEAQYITFSMLGFCSVWVSFIPAHLSTKGKYMVAVECFAILASSLGLLSCIFIPKCYIILI
RPELNIRGSLIVKKQDKSIKI*
>jgi|Xentr4|473563|C_scaffold_2706000001
MTLENVSGFIIQGFSDTPELHISLFVLFLGIYLIILLGNLIVFLVISCNPHLHTPMYIFLQNLSLI
DISFTSNILPNLLNILLTQQNNISFLGCMTQLYVFVSLSGNEFFLLTAMAYDRYVAICDPLHY
IARMSRKHCAGLITAAFTVGIGVAVSFIVLISNLSYCASHLINHFFCDVTALLQLSCSSTFSV
ELVMYIEGTLVVFSSFLPTLTSYIFIISAILKIQTSEGRQKAFSTCASHLACVLTLYVTALSLY
VRPTTSYSLKRDKYFSLLYTVLGPVLNPFIYTLKNREFQSSLNKVRQNCLYFLLVRGSQ*
>jgi|Xentr4|473664|C_scaffold_3394000001
MENEFNTSTSFILLGIEEMEHNKYLYCAFLLITYLLILLFSCLIISVVLLDESLHCPMYTLIAN
LMLNGIFGASCFLPKLILDLFFSSKVISRAACFIQSFCVTLFAFCEISIFTTMAYDTYLAVGHP
LRYPTLMTNKTALRLIAGFLFFNIISMLINLLLSARLPLCGSHINNFFCDNLSIFILSCVDNSP
NKLYSAAVFATYIPLTVLTIAYSYVRILLICWKVSQSASRKAIHTLVTHVSNFAIFMGAVLFIF
IRYRLKSINLPLVVHILPSIIGSTFPPLLNPLIYGIRTKALKIKVISHLQKMMVKI*
>jgi|Xentr4|473746|C_scaffold_3749000001
MHKMNQTSVSLCSVSLDLYCDIIGNLLIIILVITVSALRSPMYALLSQLSLADVLLSTSITPNF
LRLLLNGGGTISATGCITQFYFYCVSTASEFFLLTAMAYDRYLAICSPLHYASIMGFRLCLY
MSLCSWGLALILSMTMTLLILNSQFCGPFVIDHYFCDFTPLIKLSCTDYKAVELTDIILLIPF
MLLPFCFIIYTYVAIGLAILRISSTEGRHKAFSTCSSHLIVVCMYYGTMIIVYMVPSKGHKL
NINKILSLLYTVGTPFFNPTVYSLRNNDIKSVLLKYL*
>jgi|Xentr4|473766|C_scaffold_3972000001
MSYQEIMDPCALNLSCFHINEQEKNSGPNITLLNDKAFVLPIFSTAAKIRVAITCILFVFSTCF
NVAALWTITYKYRKKSHIRILIINLVAADLFITLVVMPLDAVWNVTLQWYAGDLACRVLMF
LKLAAMYSSAFVTVVISLDRQAAVLNPLGIRDAKKKNKIMLCVAWSLSYLLAIPQLFVFHT
VSRSEPVHFVQCATVGSFQAHWQETIYNMFTFFCLFLLPLLIMVFCYARILMEITHKMKAA
CVSSKEIDLRRSSNNIPRARMRTLKMSLVIVLTFIVCWTPYYLLGIWYWFSPEMLTKEKVP
PSLSHILFLFGLLNTCLDPIIYGLFTIHFRREIRRVCRCAAQGKDHDTASLGTGSFRISTTTVP
MKRAVLGGSGKLELEVTGYGLHSGKCEQCRGKIMESFM*
>jgi|Xentr4|473800|C_scaffold_4501000001
MENQTTVLTGLSNLPSLQLPLFLVFLPIYLITLTGNLLILLLIFTDSHLHTPMYFFLGTLACL
DMSCSSVTVPRMLFDLLRERKIISMGVCITQFYFFFFFCGSEISVLAVMSYDRYIAICRPLHY
MQIMSWMCWLVAQYVPCFFCDLPQLLEASCSDTFINVLLIFVFGIMYGIGILGSTFYPYIPII
TTVLKMTSKHTRSKAFSTCSSHLTVVSICYTTLFFNYFRSNANDHLVEGKVASVCFAILTPS
INPMIYSLRNQELKLSLRRSLRRLQ*
>jgi|Xentr4|473882|C_scaffold_6102000001
MSVQNGSHLPPSFILLGAPGLEELYGFISIPYFAMFATSIAGNGLVIVVILQDYLLHQPMYLF
LSMLSFADLLFSVSTCPTVMNIFWFNSRQIQSFMCLVQMFFVQALSVVSSGLLLAMAFDR
FVAICHPLRYSSILSNSMVVKIGVASALRSIVIHIPLVYSLELLPYCKGNLLSHSYCLHQDVM
KLACDGTNTFNIAYGLVIILSTVTLDAILIFISYVLIMRAVFGLTSGVDRYKVFDTCISHLCAV
FLFYVPVVALSFIHRFGTQLSPSFKIFMASVYILVPPMFHPIIYSIKSKQIRGSFDRLFRRHRV
RATCT*

>jgi|Xentr4|473892|C_scaffold_6196000001
MLSAIQIIKTIILIITGSCGLILNSWIVAVHLSHWKKGVSLGDCDQIILIKGVTNVLLQCLVTF
NGILINFQLNDYFDKEFLYVTNIVFFFLTSLWNWLTAWLAICYCFRLGNISHRVFIGLKKRIS
SGITQLLLGTVVVLGMISIPYFWTTHIKAKQNTTSTSVFEQDIKYLYFMTAFCCCLPTLITSL
CMGLSLKSLLKHVHRMKQNHSQSWSGKMKTHARACMTIFLLMALNLFFFLMIFISVISTN
ILSLWDIFFWSIIMASPSGQALILLFGNSKLRSDLLKTCF*
>jgi|Xentr4|473919|C_scaffold_6944000001
MLSAIQIIRTIILIITGPCGIVLNSCIVAVHLSHWKKGVSLGDCDQIILIMGVTSVLLQCSLTFN
GIADNFELYGHFDKEIVFVNDMFFLFLNFFWIWLTAWLAICYCLRLVNISHRFFIGLKKRISS
GVSLLLLGTAVILGVINIPIFWTLNIKAKQNITSTLPVDFLISDSDIKYLSFTAAFGCCLPTLIT
SLCMGLSLMSLLKHVQKMKQNHSQSWSGKMKTHARACMTIFLLMALNLFFFLTIFSIILS
KFEIENNWNTLIFCIIMASPSGQALILLFGNSKLRSDLLKTCF*
>jgi|Xentr4|474281|C_scaffold_9462000001
MTSGNVSGFIIQGISDTPELQNFLFVLFLGIYLIILLGNLIIFLVISCNPHLHTPMYIFLLNLSLI
DISSTSNILPNLLHILLTQQNNISFLGCMTQLYLFVSLADSEYFLLTAMAYDRFVAICDPLHY
IARISRKHCAGLITAAFTVGFGGTVGFIVLISKLSYCASHLINHFFCDITPLLKLSCSSTFSVE
LFIYIKAILLGFNAFLLTLTSYIFIISAILKIQSSEGRQKAFSTCASHLACVITLYGTVFCLYLRP
PKSHSLKRDKYFSLLYIALGPVLNPLIYTLKNREFLSSLNKVKQKCLYLFTS*
>jgi|Xentr4|474401|C_scaffold_10271000001
MVSESKENVSGFIIQGFSDTPELQISLFVLFLVIYLIILLGNLIIFLVISCNPHLHTPMYIFLLNL
SLIDISFPSTIFPNLLHILLTQQNNISFLGCMTQMYVFASLADSEYFLLTAMAYDRYVAICDP
LHYIARMSRKHCAGLITAAFTVGFLDPVSFIVLVSKLSYCASHLINHFFCDISPLLKLSCSST
FSVELSIYIDGILVVFSSFLPTLISYIFIISAILKIQSSEGRQKAFSTCASHLACVITLYGTAICLY
MRPTKSYSLKKDKYFSLLYIALGPVLNPLIYTLKNREFQASFNKVRERLLAFLF*
>jgi|Xentr4|474405|C_scaffold_10316000001
MEPGISNQSFILSYTDFTLLGFPGISRWRPLLAIPFFSVYLVILSGNSLIICLICIKKTLHSPMY
LLISVLFAINITISTAILPKFLLDLLFHLNQVSLTGCLLQMFVIYFMSVCESGAMVLMSLDRY
VAICRPLHYHNIMTKRFLAWLTVIIIIRNCFLICPLIILISMVQFCRSNIILNFTCENMALLSLG
CGDTTKPQIAGLIARTIAIVLDISLLLISYSKILYTAMKTATGKSRQKALSTCGTHLLVATLAY
MSALSSSAVYRMETTLSIDVKNLFTALYLILPASLNPFIYGLWVSEIRKSLVKYWRKKLVSS
*
>jgi|Xentr4|474500|C_scaffold_11921000001
MHKVNQTSGISFLLLGFQNSQIINEFLFVLFLWIYIVTLFGNLLIIILVITVSALRSPMYALLS
QLSLADVLLSTSITPNVLRLLLNGGGTISATGCITQFYSYCVSAGSEFYLLTAMAYDRYLAI
CSPLHYVSIMGFRLCLYMSLCSWGVALMLGLFFNLLTFNLQFCGPFVIDHYFCDFAPIIKLS
CTDYKALEITDIILIIPFTILPFCFIIFSYVSIGLAILRISSTEGRHKAFSTCSSHLIVVCMYYGT
LIIVYMVPSKQYMFNMKKILSLLYTVGTPCFNPIVYSLRNNDIKVALRKYMSNQISFFTLNL
SIRKGD*
>jgi|Xentr4|474544|C_scaffold_12910000001
MALESQENVSGFIIQGFSDTPELQISLFVLFLVIYLIILLGNLIIFLVISCNPHLHTPMYIFLLNL
SLIDISFPSNILPNLLYILLTKQSNISFLGCMTQMYLFVALASAEYLLLTAMAYDRYVAICDP
LHYIARMSRKHCAGLITAAFTGGFVDSVGHVVLISKLSYCASHLINHFFCDVTPLLKLSCS
STFSVELLFYFVGTLLVFNSFLLTLASYIFIISAILKIQTSEGRQKAFSTCASHLACVITLYVTA
ICLYMRPTKSYSLERDKYFSLLYIALXPVLNPLIYTLKNREFKSYFNKMRERISLRLKSCSC

SAM*
>jgi|Xentr4|474575|C_scaffold_14109000001
MLAAYTVIVTVILIVTWPCGTILNSSIIAVYLSDWKKGVKCGECDQITLSMGCNYLLIQCFII
IFWAFRFYGLDLPFAEKLTFAINTGFWFSVPLSFWLTADLSICYCLRLVNLSSALFNQIKRRL
SRIVTPLLLWSVGISFIFPVTRIFAIQIDQNETFINLENTSNVNLGIVIPAVAFNVCLPFIITSICI
VLSLISLLRHIRRMKQNTQFGSPQLKNLIRACRTMFLLMALNFIFSLIICSSMLISDRMGTV
WGKGALLGNMLNPSCQAIVLIFGNSKLLGAWIKTLFPQ*
>jgi|Xentr4|474614|C_scaffold_14796000001
MSNTRTCNQSKITEFLLVGFSTTRSLKVLLFSICLVIYIMALGANLMIIALYLGSHHLRSPMY
FFLSNLSATDILLSTTVGPNLLCTFLKDGNPMSVSACVTQFFAYGTFIAAECFLLTVMAYDR
YLAICKPLHYATIMTNKHCLHLVIWPWLGILLLSLPITVIISHSGFCGCNTLDYIYCDLTPLL
KVSCSDVFLVDTIATIIGTIVTLLVLQLPLLFVITTYVSIFLTILKISTKTGRQKAFSTCGSHLT
VVCTYYGILISKYTFSSKGHSVSVNKLISLLYTLVTPLFNPIVYSFRNQELQKALSKLISVRL
*
>jgi|Xentr4|474665|C_scaffold_15055000001
MLSALQLIRAVMLIVTWPCGTILNSSIIAVYLSDWKKGVKLGECDQISVSMGCTNLLLQCF
VTLGVAFISYGLHLPFASKVSLAVGAVLWFSVFLSFWLTAGLSVCYCLRLVNPLPKCFIHLK
RRLSRIVTPLLLWSVAISFIITVPINAIVGMATDQNITTNDHYIISSVIFMILDGAFGIGFPTLIT
SICILLSLISLLRHIRRMKQNPQFGNTQQKTLIGACRTMILLMALNSILFLVILRSMLPPYNV
ETIWQTVMFSCVMLNPSGQAVVLIFGNSKLLSAWTKTLVPQG*
>jgi|Xentr4|474666|C_scaffold_15055000002
MLSALQLIRTVMLIVTWPCGTILNSSIIAVYLSDWKKGVKLGECDQISLSMGCTNLLLQCF
VTLGVAFLSYGLHLPFASKVSLAVGAVLWFIVFLSFWLTAGLSVCYCLRLVNPLPKYFIQLK
RRLSSIITPLLLWSVAFLFILTVPINCMEGIATDHNTTTNYNISNTVAVYMIFDGILGIGFPTLI
TSICIVLSLISLLRHIRRMKQNTQFGSPQLKNLIGACRTMFLLMALNFIFFLVILRMILSPYNP
ETIWQTVVFSCVMLTPSGQAIVLIFGNSKLLSAWSKPVAPQG*
>jgi|Xentr4|474678|C_scaffold_15276000001
MGQIRSSLLILYFASTGRKENGFRNLQISLFVLFLGIYLIILLGNLIIFLVISCNPHLHTPMYIF
LLNLSLIDISSTSNILPNLLHILLTQQNNISFLGCMTQMYVFASLAGSEIFLLTAMAYDRYVAI
CDPLHYIARMSRKHCAGLITAAFTGGFVDPVGHFFLISKLSYCASRLIKHFFCDVTPLLKLS
CSSTFSVELLNYIEGTLLTLNAFLLTLASYIFIISAILKIQSSEGRQKAFSTCASHLACVITLYG
AIFCLYIKPTSSYSLKRDKYFSLLYIALGPVLNPLIYTLKNREFQSSFNKMRHIF*
>jgi|Xentr4|474704|C_scaffold_15549000001
MSGNYSVFEEFLILAVSDKPDLKVLLFCILLCIYVLTVTGNAIIIMVSQMDKRLHKPMYFFL
SNLSFLDICNTSTTIPKMLQVLIIKRKSISFIGCVTQMYFFLAFVGTECILLGIMAYDRFIAICS
PLKYYTIMNHKLCFYLAGFSWVSGLGNSVVHTVLTFRLDFCNSNKINYFYCDIPPLLSLSC
DDTSINEVLLLLIGVFIGWTPFFCITVSYVYIIVTVMKIRSIESRKKAFSTCASHLTVVVLYY
GSSIFNYVRPISSYSLGKDRIISVMYSVVTPMLNPLIYTFKNQDFEKAIGRQFIHRHYLQH*
>jgi|Xentr4|474753|C_scaffold_16280000001
MENCLKCEDTEWPNQEKTLCIEKQIEFLSYAGDPLTLIFIISSVILFIIAAVILGIFISFRDTPVV
RANNHTLSFILLVSIKLSFLSVFLFLGRPVDITCMLRQTSFGITFSITVSCVLAKTLMVSIAFK
ATKPGSPWRKWVGVKLANGLVFICSLIQFLISVIWLVIAPPYVEKNFRSEPGKIIIQCNEGSA
IGFYIVLSYMGLMLLFCSVWITMIPAYLSTKGKYMVAVEIFAIISSSCGLLFCIFLPKCYIILFK
PEMNSKQYLLGKCNT*

>jgi|Xentr4|474755|C_scaffold_16389000001
MDNNTIFTYFSILAFSRAGEKPPTLFIAFSFVYMFGVLGNLSIITVTCSDAHLHTPMYFLLSC
LSFIDICYTTTTLPKLLHILLTGNNIISFPECFTQMVFYSLTAQSEIILLAFMAYDRYVAICDPL
KYHLIINNKKCAQILAAIWTSSLPNALLGSSIILELNFCGSNTIDQFFCDAKYMIKISCEAVSI
NYYLFFGAMIYGVLPFLLCLASYTKIIHSIFQIKSSSGRKNAFSTCSSHLTVLLLFFGAASW
MYMAPENSHQLDRIITVIFTAVTPMLNPLIYSLRNKEIKRAIKTLFS*
>jgi|Xentr4|474842|C_scaffold_19611000001
MYLLTVLGNLLIIVLVCLVPQLHTPMYFFLCNLAAQDIISVSAFLPKLMAITITGDTRASFPG
CITQIFLFAFCACGDFLLLAIMAYDRYVAICIPLRYHLIMNPRLCILLMTTSWILCATSAMCY
SLFISQLSFCRLFDINHIFCEPVSMLKLSCSDTTHIQILIIIELPLIAIFPFGLILTSYVYIIYTILK
MRTTAARLKIFSSCSSHLTAVLLFGGTGIGLYTKPESNDSQDIDKMLFLIYLGFVPMLNPLV
YSLRNRQVQSAAKNIFTKYISYWQCKLRVIHYT*
>jgi|Xentr4|474891|fgenesh1_Sanger_cdn...
MLLIIETFEMGLLLRSALFKYIIIVLIMLNLRGYVLAEQEQGSQIPLEEIIQVGVTRNKIMTA
QYECYQKIMQEPANGKEGHFCNRTWDGWLCWGDVSAGVISEQRCPDYFQDFDPSEKVT
KECGKNGHWFRHPDSNRTWTNYTRCNTFTHEKVKTALNLYYLTIIGHGLSIASLLISLGIFF
YFKNLSCQRITLHKNLFFSFVCNSIITIISLSAVANNQALVATNPVSCKISQFIHLYLMGCNYF
WMLCEGIYLHTLIVVAVFAEKQHLMWYYLLGWGFPLIPACIHAVARSLYYNDNCWISSET
HLLYIIHGPICAALLVNLFFLLNIVRVLITKLKVTHQAESNLYMKAVRATLILVPLLGIEFVLF
PWKPEGRIAEEIYDYVMHILMHYQGLLVATIFCFFNGEVQAVLKRHWNQYKIQFGSSFAHS
EGLRSASYTVSSISEIQGTTYTHDYSEQSNGKNCHDMENVFFKTEKQYM*
>jgi|Xentr4|477554|fgenesh1_Sanger_cdn...
MFGVIRPLCFLCCFILDSAIPINSPNDLYARLQGDIMIGGLFPIHKEISDLAICTEPSELICTGF
DLRGFLRSLGMVHAIERINSFNLLPGIKLGYEIYDTCGDASRGLQETIRFTGYEELAYEILH
GMCNVTDLLPLVKAVVGAGYSEVSVAVARLLGFQLVPQISYGSTAAILSDKRRFPSFLRTV
PSDVHMTKALAKLIRIFKWSYVGIISSDDDYGRSVLESLSMQFDSKYVCTAFKEKLPADIS
KPGVHVAIKTVTDTIKQSPAEVVILALKVPVVMELFNEIIKKNITRLWIATDYWSTSREVAA
MPDIDSVGNILGLSFKNIEVPGFRIYLRNLTVGPNATNVFIEEYKRLRFECTDEYKEYKHCL
KASPRNCTKSQSLKFKSPLACSTENIALASDDYLDKNIELDGTYSAYLSVTAIAKALNNILC
SNGICNHNMTFAPWQLLKELKKIEFYDYDEKIFFTDDGNANIGYDVVSWYTVNGSMEFH
VVGNYELSNGSIYLNKSLIIWNTEDKKAPSSLCTKPCIPGQYKIHSDVHCCYNCSDCAEGY
YSESYDMTECKKCPYDQWSNKGSAYCENRTIEYLEWTNPFAVVLCGFALIGFVIVVIVGIG
FLQHVASPAVKAAGGIYICVMNFSLLISFANSILFIGEPRNVSCKIRQPLFGVSFTLCVSCILI
KSFRIVLAFEMGNKFQHSIRITYQPAIVVIVLTAFQVCICTLWLLLRGPCIKNIVLIPQTLIVQ
CDEGSTIGYAIMLGYIGFLAFVCFVLAYRGRNLPDKYNESRCITFSMLIYLFVWFAFIPVYV
TTNGMYLPAVEMVAILASNYGVLGSHLIPTCYIIFFKKDNNKREKYLESIQSFSKVKCAVD
YHVPEISRSQYQAKQLCFVHRMRKRCKSV*
>jgi|Xentr4|457721|estExt_fgenesh1_pg.C_4470002
MEQRVTASPYPNSTLEQLVSNVSNTSNASDKPGDAWMVGMGIIMSCIVLVIVLGNVLVITA
IAKFQRLQTVTNYFITSLACADLLMGLIVVPFGASSIILDTWVFNNFWCEFWTSVDILCVT
ASIETLCVIAVDRYFAITSPFRYQSLLTKCKARIVILLVWVVSTLTSFLPIYMHWYRIEEESAL
HCYDDPSCCFFFTNPAYAISSSIISFYLPLVVMIFVYARVFQEAKKQLKKIDKSEGRFHNQNN
QQDTNGKQGNKRTSKFWLKEHKALKTLGIIMGTFTLCWLPFFIVNIVQVMHKDFIPVKVY
NFLNWVGYVNSAFNPLIYCRSPDFRYTFQELLCLRKPGSKAYANGYPQSNGNSAYNEDTE

PCQYSKDRTCEVLFKEEKTPNPSLLNCEGTLLDFSLEPLGKNHNTHDCLL*
>jgi|Xentr4|51578|gw1.84.33.1
GLCSVLGNSTLLYISYKRRHLLKPAEYFIVNLALSDLAMTVTLYPLAITSSFSHRWLYGRH
VCLFYAFCGVLFGICSLSTVTLLSTICCMKVCFPVYGNRFGHKQGCFLVACAWLYAAIFAFS
PLLHWGEYGAEPYGTACCIDWYSSNKSRVAMSYTTTLFVLCFVIPCGIIITSYTLILVTVKD
SRKAVEQHGVAGPSSMNNVQIIIVKLSIAVCIGFFTAWSPYAVIAMWAAFGSIDIIPPLVFAVP
AVFAKSSTIYNPIIYLFLKPNFRNILAK
>jgi|Xentr4|61494|gw1.48.71.1
LQMGNKSDASAFYSSISETDDIVLGVLYSVFGLLSLSGNSMLLLVAYRKRSILKPAEFFIVN
LSISDLGMTGTLFPLAIPSLFAHRWLFDKVTCNYYAFCGMLFGLCSLTNLTVLSSVCCLKV
CYPAYGNKFSTAHSRILLLGIWAYAGLFATAPLADWGKYGPEPYGTACCLDWEASYRERK
ALSYTISLFVFCYLIPSSLIFISYTLIFVTVKGARRAVQQHLSPQAKGSSIHSLIIKLSIAVCIGF
LIAWTPYAIVAMMAAFGDPTKIPSLVFALAAAFAKSSTIYNPVVYLLLKPNFLNVVTKDL
>jgi|Xentr4|135162|gw1.328.138.1
SLSGNCTVVSIAYSTLKDILPSDPNTIISGLVISTKVVQGTINQDHFNVSISFSQSNDSPTYSQ
CVFWDFNINNWNQENCSSTVTDSSVFCNCNHLTSFSVLTGDIQSGVMYSSRTLFYIGKGFS
LACMVITLVIEAIVWKSVTKSKTSYLRHMCLVNIAVLFLVEDIAYILPLPTYFLIFFSALAQF
FWMLNMGILLFYHIVFILHDCRGKTMVAIAFCVGYGCPALISIVPLTSLLVTSDSTSDRFCW
IGFYSLRDLLAFILPAFTIVFVNLIILLVVIFKLLRPNVGDNQSKDKKIVTIQIFKSLAVLTPLL
G
>jgi|Xentr4|159594|fgenesh1_pg.C_scaffold_47000036
MACSVSLSAWLLCVGLVAASRRDSLEGRTAAHPQQHFPGRYPIYSSPSNPQHSALWIQTTE
SAPLAQTLAQDLPKHVASFLYKGAAGELKAALCSQSYELSSLPGASPGHPTLLRAVDILLH
GTNFLNMMLQSNKSREHNWERDVEWYYALIKSILEGEPAIFRSAVAFNVEPSSHVPQVFLR
ATREEGKIHLQDLSASAHGPGNATWEHDWLHTFKHRSRPHLHKRLLHGAKTFDDGWKK
GNSYVTDRSHVKWSSPYLECENGKYRPHWLLSLSAAFYGLKPNLVREFRGVLKVDVNLQ
NIDIDQCSNEGWFSGTHRCQHNSSKVLDLLSSFYQITIMRTLRRLECGQYLCENFNKKVVF
FTRLRIGRPRDSATLGWCGFLLIGAPTWGISVYQRKVLDLFSEPTNALARLVSIIPMACFQR
TAFKSVRASGLVLLEVILFGSLLLYFPVVILYFEPSTFRCILLRWVRLLGYATVYGTVTLKLY
SELALVGGVHFTACLTLDHMLEDAGSCSAETSEFLFLLWGVYLCYAVRTVPSAFHEPRYM
AFAVHNELIFSALFHTIRFVLASKLQPDWMLMLFFVHTHLTVTVTVGLLLIPKFSRSHNNP
RDDIAAEAYEDELDLGRSGSYLNSSITSAWSEHSLDPEDIREELKKLYTQLEIYKRKKMIAN
NPHLQKKRCSKKGLGRSIMRRITEIPDSVSRQCSKEEKDTTDHGNPKNNVGSSKKHPQES
VTHNAKSKEEPSKHKLYSMKKSHSSYDHLRSESDTPNGISTEKVDVCKNSIYSPVNGKKP
NKNGSELEAVSADNVPLVCKSASAHNLSVDKKPLHPSLSVLQKSLSAVANSKDNSLGLRD
KTQSVEDTDKCHRTDSAYHEHQPSVATFDKEERQQHTSSPIEEIQKLHKSGIMKQQAMSPH
LPESEKFITAMGYKDKFDIEEVCPWEMYDLPPSVPSENKVQKHVSIAPLESEKNHTSRSKS
KSHNRSKSGEHGHQQSKQKGHTKTEPNIRGTQEQLSKEEQFKQLPTNDANTMDQQAATK
QTTKSHEKEGKKNIALVEGVLTSPEPHHNSNNNLPQPLVLRAEVCPWDFENPDVLSVEKS
KTSPTTTVLSPTSPSRNMATSPLKKKVLGMPIKVTSANKDAGTKTKDLDPLGSMLKIDKS
KSAEVCASAGDMTLGVETNKVKSSSNEHGIDRCRLEEICPWESLSPSQSEQDTLASDAKH
NTTMQGSNMVEICPWDFEDDSKGKEG*
>jgi|Xentr4|173417|fgenesh1_pg.C_scaffold_277000029
MENVTLGSAASNSCTFHEEFKQILLPVVYSVVLLFGLPLNFIVILQICLSRKALTRTAIYTLN

LAVADLLYVCSLPLLIYNYIQQDYWPFGDFTCRFVRFQFYNQPARQHHVPQLYEFPEVHG
HLPTLCPFGTQRGARSSLGCCVASCWLLVGIQCGPTFIFASTGTQRNRTVCYDLSPPALSDQ
YFPYGITLTVTGFLIPFIAILACYCRMTKILCQKDDLNGAAVRQKKDKAIRMIIIVVMVFAV
SFFPFHVTKTIYLVVRSHYDVPCLVLQTFAIVYKCTRPFASMNSVLDPILFYFTQQKFRQST
KVLVQRVTSKWKSTSCEKTSG*
>jgi|Xentr4|264948|estExt_Genewise1.C_40124
MFLVCCVPFTATLYPLPSWVFGDFMCKFVAYLQQVTVQATCITLTAMSADRCYATLYPLRS
LRHRTPKVAMIVSICIWIGSLLLSTPIIPYQKIQKGYWYGPRTYCIEQFPSDVMKKVCILYQF
LAVYLLPLLTICLCYSLMLKRVGRPVVEPTDNNYQVQLLSERTIAMRSKISKMVIVIVLLFT
ICWGPIQLFSLFQGFYPGFQANYATYKIKTWANCMSYANSSINPLVYAFMGASFRKSFKKA
FPFMFRNKVRDGSITSGTVNNEMKFVAMESTNNEIK*
>jgi|Xentr4|325308|e_gw1.78.18.1
FACKMVPFVQSTAIVTEILTMTCIAVKRHQGIVYPLKMKWQFTNRRAFTMLGIVWLIAAV
GGIPMWHAQRLEVIISSVFTKQRQICWHRGLHFRIENKFSITDFYYIPPYMKKKRAIIMMIT
VVVLFAVC*AAFHVVHMMIEYSNFENEYDDVTIKFILTHFNESTPIFAIVQIIAFFNSICNPIV
YAFMNENFKKNFLSALCFCFLRDPSSPARRPGNSGITLIQQKSSSFRRENTCEDARREVFSE
GNIEVKFFDQPV*
>jgi|Xentr4|326427|e_gw1.82.390.1
LFWQFIVGERTVASGECYIQFLSNPVVTFGTAIAAFYLPVVIMTILYIHISLASRSRVRRHCT
ETRQEKKKPISSMKSPLIKQTKNLPKQDPGDEAVKKENGVRNGKIEKSMTNLQTAEEKET
SNESSSASLSHNPPEKQPVSDASSGVVLAPTQNMPPLPSKVNVASKWSKIKIVTKQTGNEC
VTAIEIVPECAIPLPQQANNRPGNVARKFASIARNQVRKKRQMAAREKKVTRTIFAILLAFII
TWTPYNVMVLINTFCQTCIPETVWYIGYWLCYVNSTINPACYALCNATFKKTFKHLLMCQ
YKSIGMAR*
>jgi|Xentr4|329880|e_gw1.99.296.1
GPAAAVVLVNMVIGILVFNKLVSKDGIADKKLKERAGQMTMPLYGMTLKCSKCGIVSSAE
VSATATSNAMASLWSSCVVLPLLALTWMSAVLAITDRRSALFQILFAVFDSLEGFVIVMVH
CILRREVQDAVKCRVVDQQEEGNGDSGGSFQNGHAQLMSDFEKDVDLACRSGECPTKDP
VLNKDMSTLRTSTITGTLKRPSLQDEEKMKMNHQKGSNFNSLPSNVSKIHHQGSPHYLGG
INLNEYNNHTLTLKKDKSQSSKPMYMCEGDIFKKLDSELTRAQEQSMDPSYVILPSNTSTL
RAKPKEENKYSMNIDQMPQTRLIHLNMADPSYMLKSPPRERMGMKCPEQGSPMQKHQI
HPSESQIPLGLCDKGDSGTLGIVTKGETISTLSMSSLERRKSRYAELDFEKIMHTRKRHQD
MFQDLNRKLQHAEKEKESPTAESKLEKQQTPNKRPWEGMRKIQSPPSWVKKDLEPVSPSP
LELKTVEWEKTGATIPLVGQDIIDLQTEV*
>jgi|Xentr4|166711|fgenesh1_pg.C_scaffold_140000009
MAPLLELPIDPLASLSVCEMKSGRVLTVPARSTVEGGEPITALQAEKHRQASVPYQGQVVF
RNGERMGTIKFTQFQDAGDNLQLPHSENPEKTLSLKNLFGLPIQMFSLFLNLTLNESATFH
FVSEFTNQGQNSPYYIGINGKSCNSQQKYRQDGKEVKVGEYNAVADSLELINDTIRFQGV
EPPKDQTFIREERRQIYLPLYSILSTLTIIGMIMASTFLFFNIKNRNQKLIKMSSPYMNNLIILG
GMLSYASIFLFGLDGSFVSANTFETLCTVRTWILTVGYTTAFGAMFAKTWRVHAIFKNVK
MKKKIIKDQKLLVIVGGMLLIDLCILICWQIVDPLKRTVEEYNLENQYSKLYIARTRHFLGN
IRSKEAEKQQLYFESSNFQ*
>jgi|Xentr4|434449|e_gw1.2963.1.1
MDTADPCHLLINHTHVGEFEVRLAVKISLTVLYGCILLAGLVGNSVTIRTTKVLRDKGYLQ

KGVTDHMISLACSDLLVLVLGMPVELYSLIWFPFSSGYGDAACKAYSFLFEACSYATIFHVA
TLSLERFVAICYPFRFKAVSGSRAAKLMIGCAWAASLCVALPLLFAMGAEYPLNPTQGHRR
QRNCNRTGPYGPRPNVTLCTSLSNRWAVFQSSIFSGFIAYILVLGAVAFMCRRMMLAIMAT
KQPTVLVRGQNRTGVATEMAKSTSPQAKAARKQTIVFL
>jgi|Xentr4|6175|gw1.14076.2.1
LITDMEECVKCPENQWSNTNRDTCVYKVIIYLSYEEPLGTSLAFLSIIFFLLTCFVSLIFTKY
RKTPIVRSNNRDLSYILLFSLKTCFLCNLLFLGHPIRVTCVLRQTVFGVTFSISLSSILAKTIT
VIIAFSVTKPASKCRNWVGSRVPNSVIFICSILQVTICACWLGTSPPFPYYNMEDEVGMIIA
QCNEGSVFGFYCVLGFTGILACVCFIIAFLARNLPNSFNEAKFITFSMLVFCSVWVSFIPAY
MSTKGKYVVAVEIFAILASSMALLGCIFIPKCYIILVKPECNTKVC
