# Supplementary figures and images for: The repertoire of G-protein-coupled receptors in Xenopus tropicalis
Source: BMC Genomics. 2009 Jun 9;10:263. doi: 10.1186/1471-2164-10-263 (PMC2709155; doi:10.1186/1471-2164-10-263)

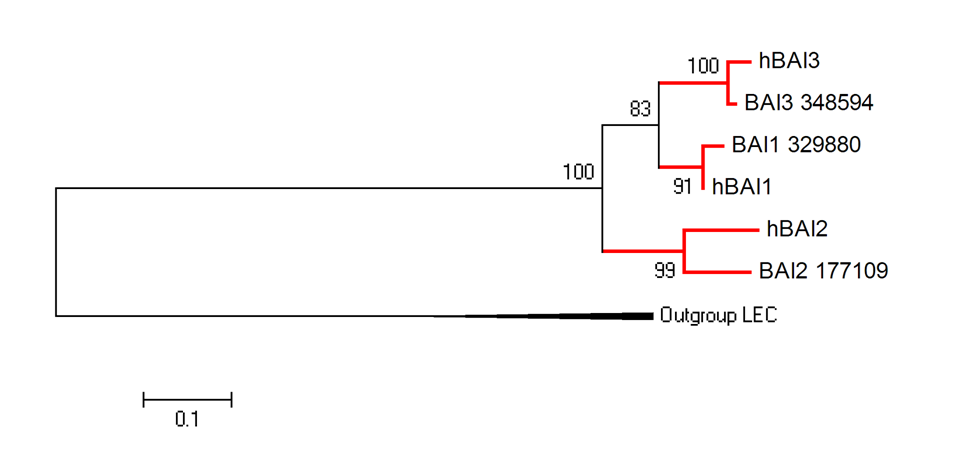

Supplement: Additional file 3 — One-to-one orthologous relationships between human and X. tropicalis brain-specific angiogenesis-inhibitory receptors. [file 1471-2164-10-263-S3.bmp]

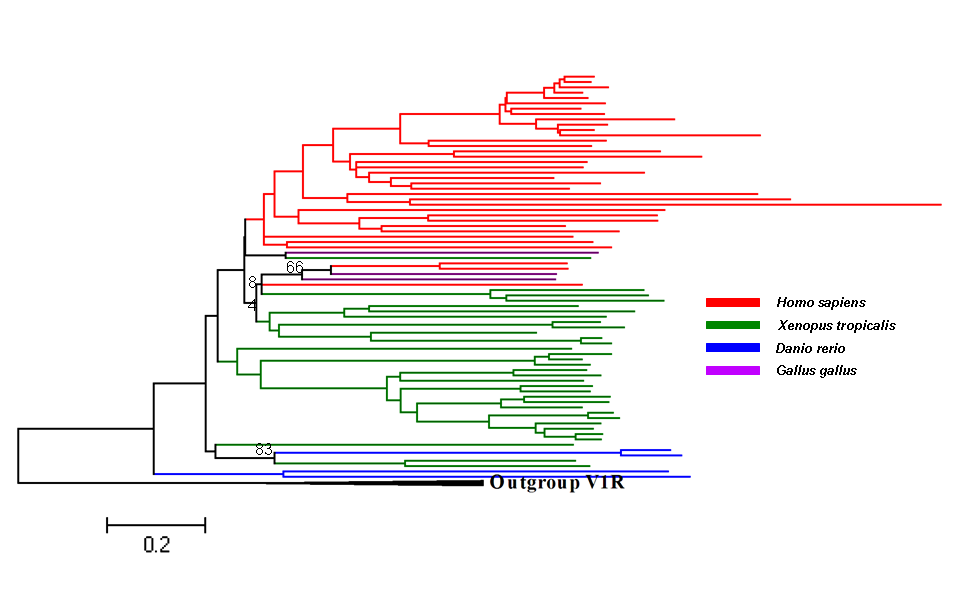

Supplement: Additional file 4 — Phylogenetic tree of Taste 2 receptor family. [file 1471-2164-10-263-S4.bmp]

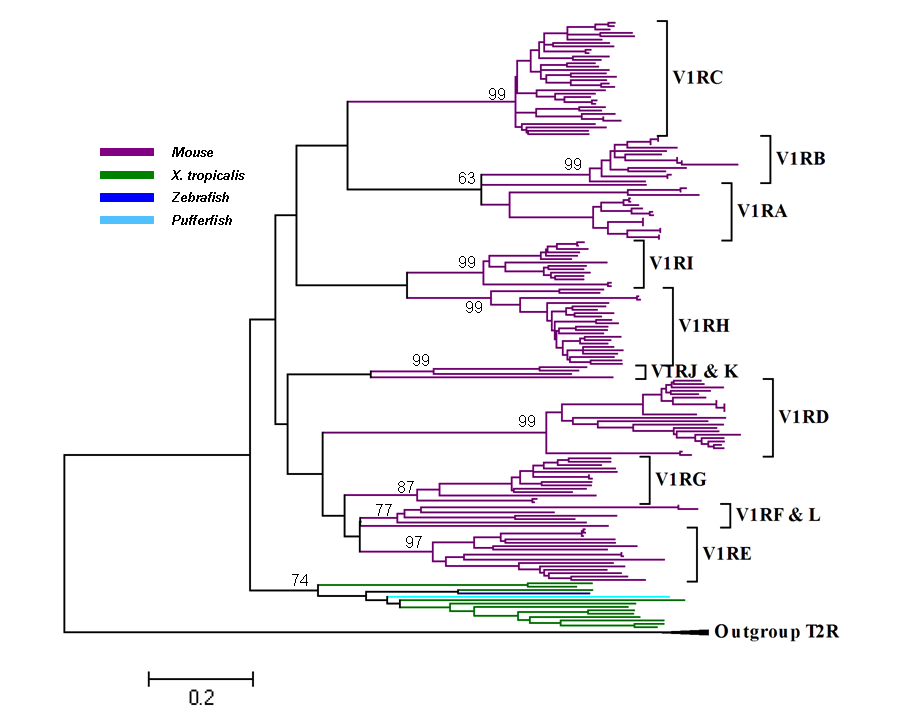

Supplement: Additional file 5 — Phylogenetic tree of Vomeronasal 1 receptors family. [file 1471-2164-10-263-S5.bmp]

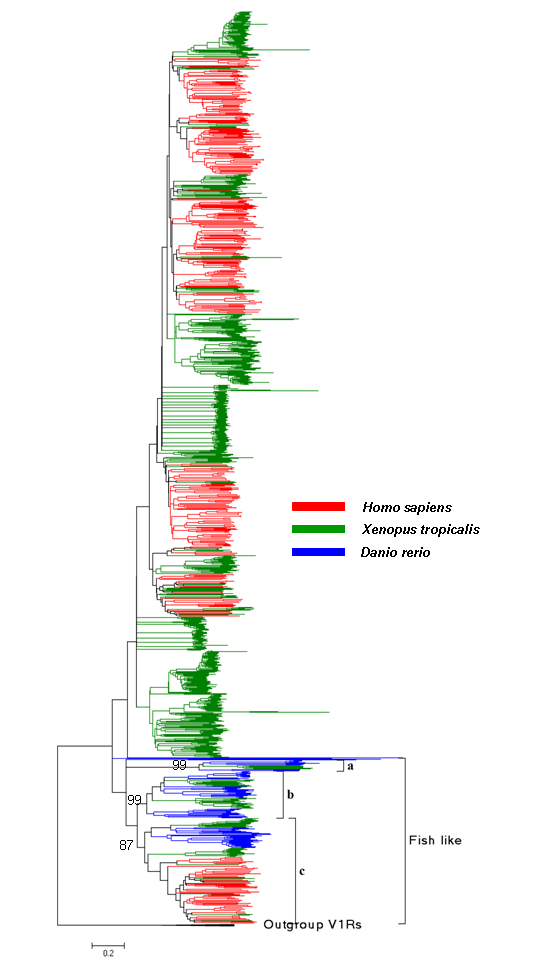

Supplement: Additional file 6 — Phylogenetic tree of olfactory receptor subfamily. [file 1471-2164-10-263-S6.bmp]

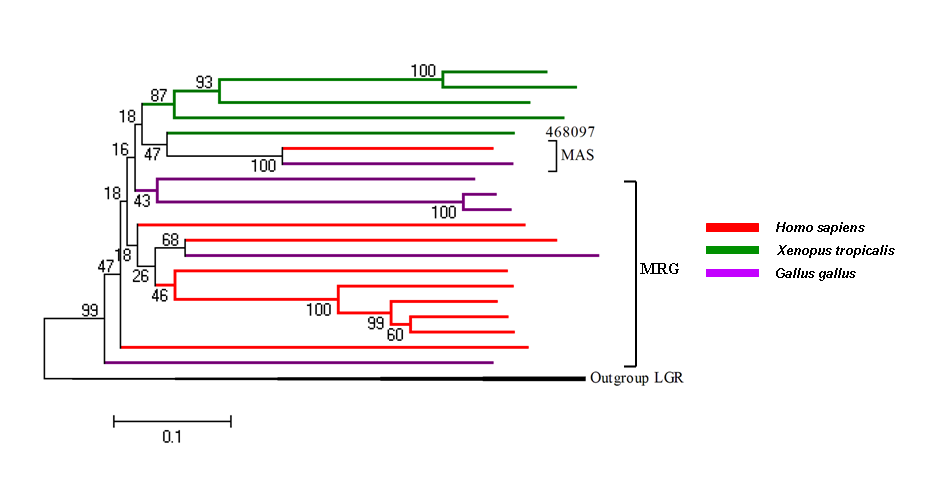

Supplement: Additional file 7 — Phylogenetic tree of MAS-related receptor subfamily. [file 1471-2164-10-263-S7.bmp]
